# Supplementary material for: SPORTS1.0: A Tool for Annotating and Profiling Non-coding RNAs Optimized for rRNA- and tRNA-derived Small RNAs
Source: Genomics Proteomics Bioinformatics. 2018 May 3;16(2):144–51. doi: 10.1016/j.gpb.2018.04.004 (PMC6112344; doi:10.1016/j.gpb.2018.04.004)
Supplement: Supplementary Figure S1 — The mouse sperm tsRNA mapping results against tRNA loci revealed by SPORTS1.0Mapping result for each annotated tsRNA was provided. [file mmc1.pdf]

AA Mus_musculus_tRNA−Ala−AGC−1

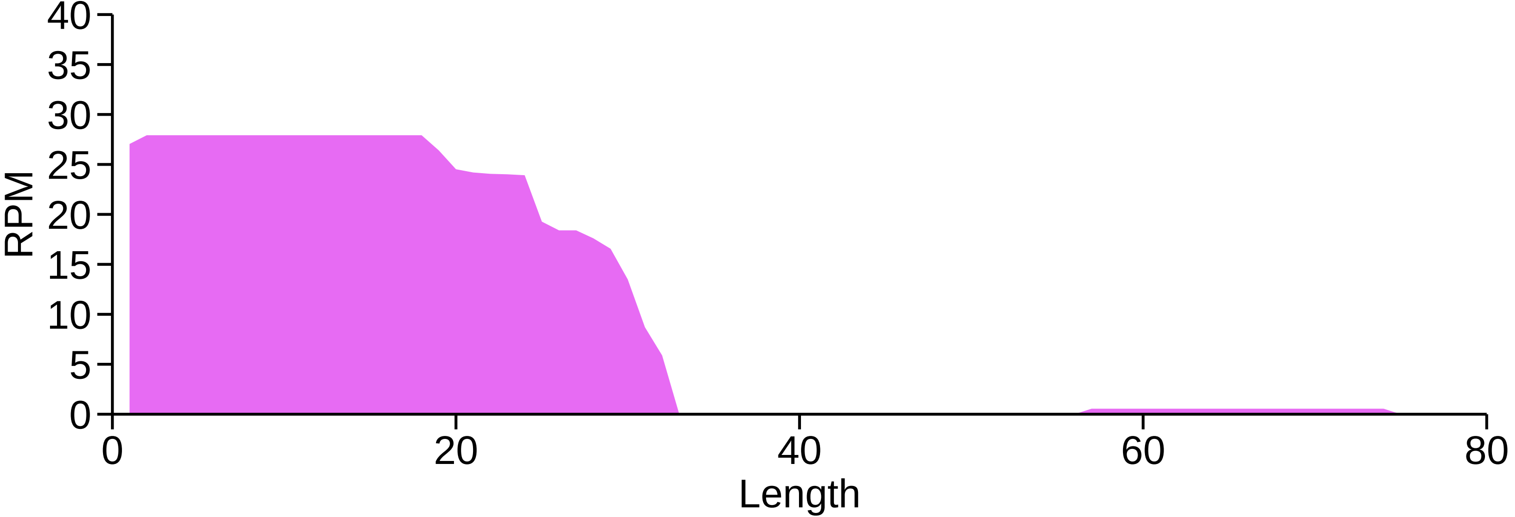

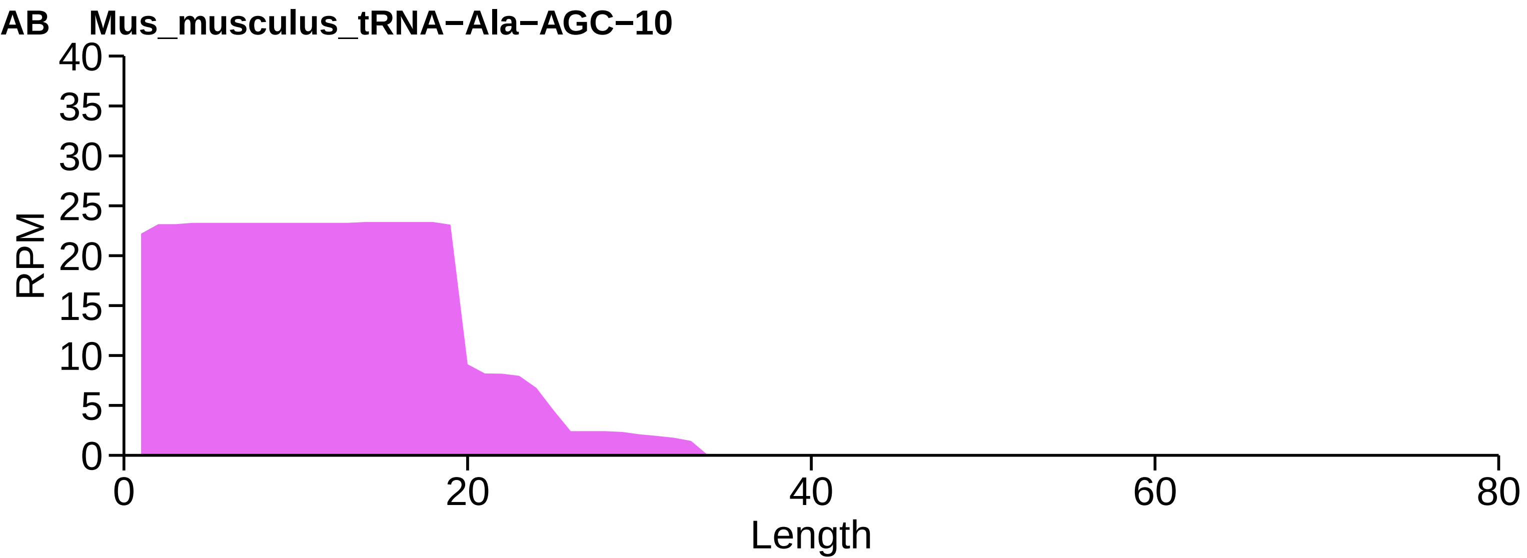

AB Mus_musculus_tRNA−Ala−AGC−10
RPM
40
35
30
25
20
15
10
5
0
0
20
40
60
80
Length

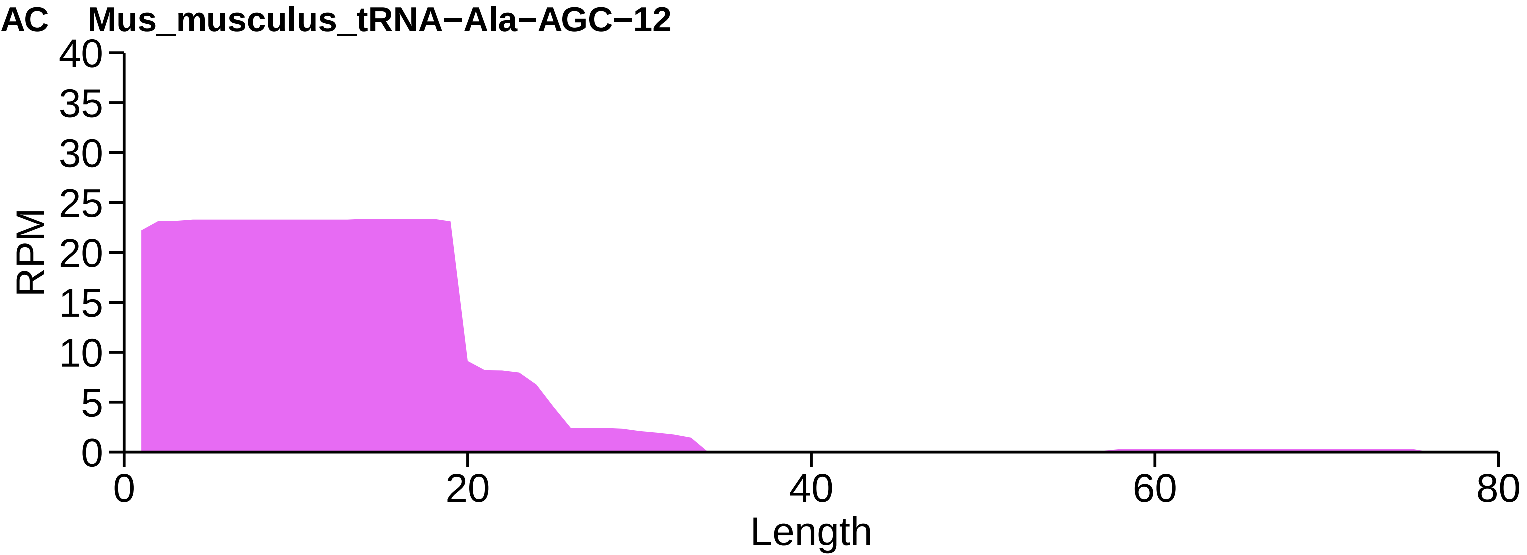

AC Mus_musculus_tRNA−Ala−AGC−12
RPM
40
35
30
25
20
15
10
5
0
0
20
40
60
80
Length

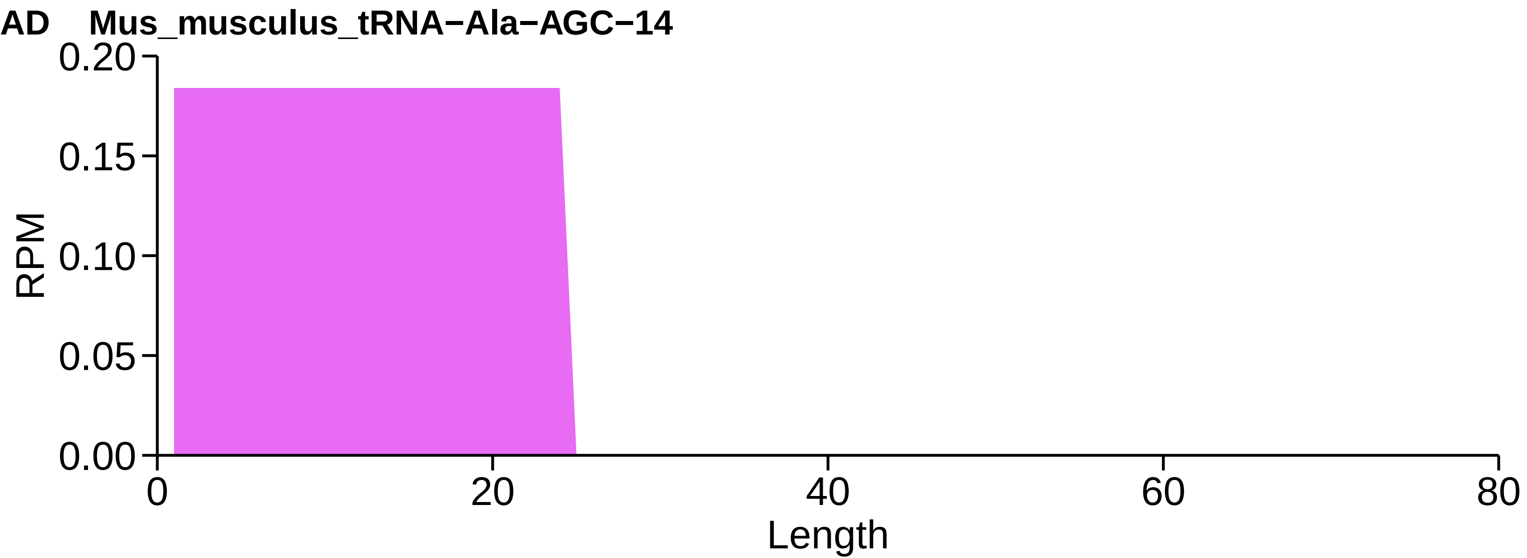
AD
Mus_musculus_tRNA−Ala−AGC−14
0.20
0.15
0.10
0.05
0.00
RPM
0
20
40
60
80
Length

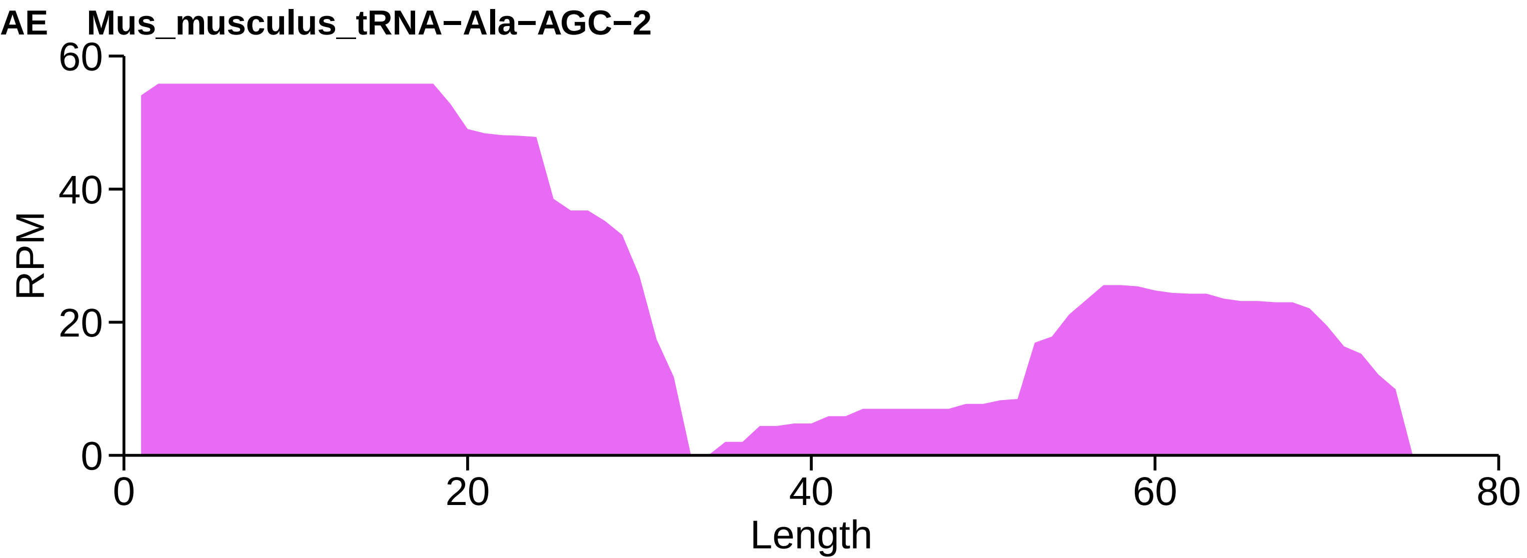
AE Mus_musculus_tRNA−Ala−AGC−2
RPM
0
20
40
60
0
20
40
60
80
Length

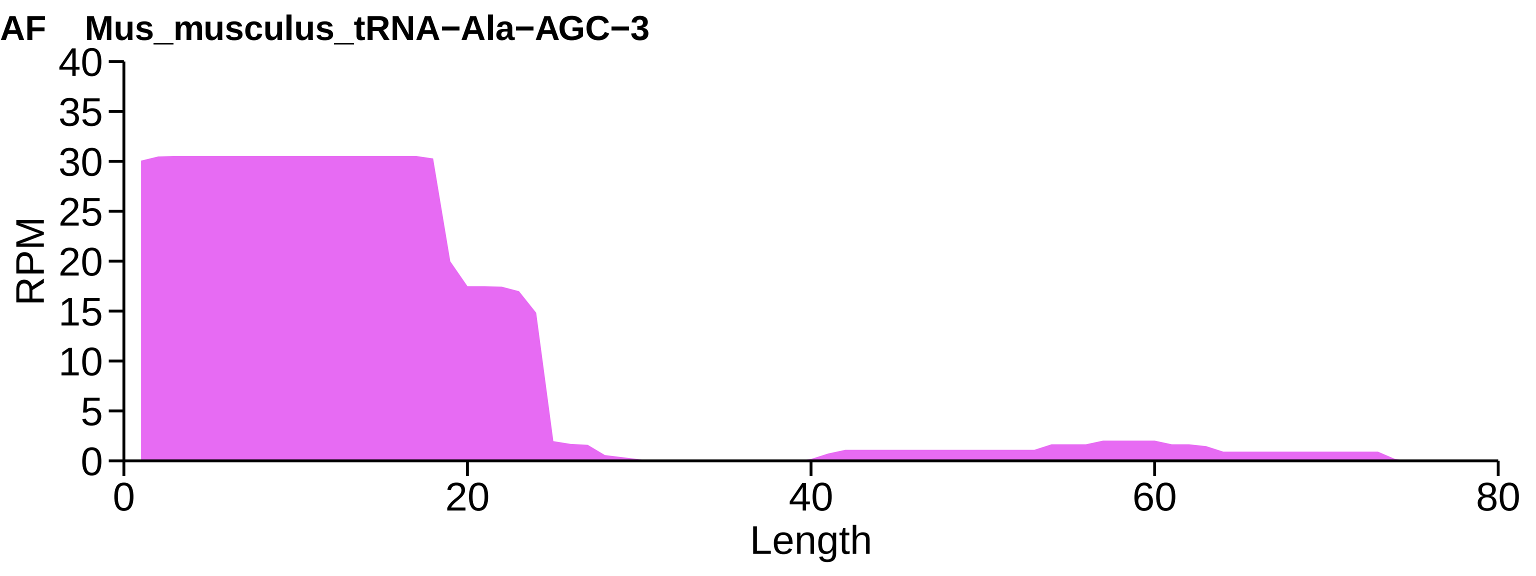

AF Mus_musculus_tRNA−Ala−AGC−3
RPM
0
5
10
15
20
25
30
35
40
0
20
40
60
80
Length

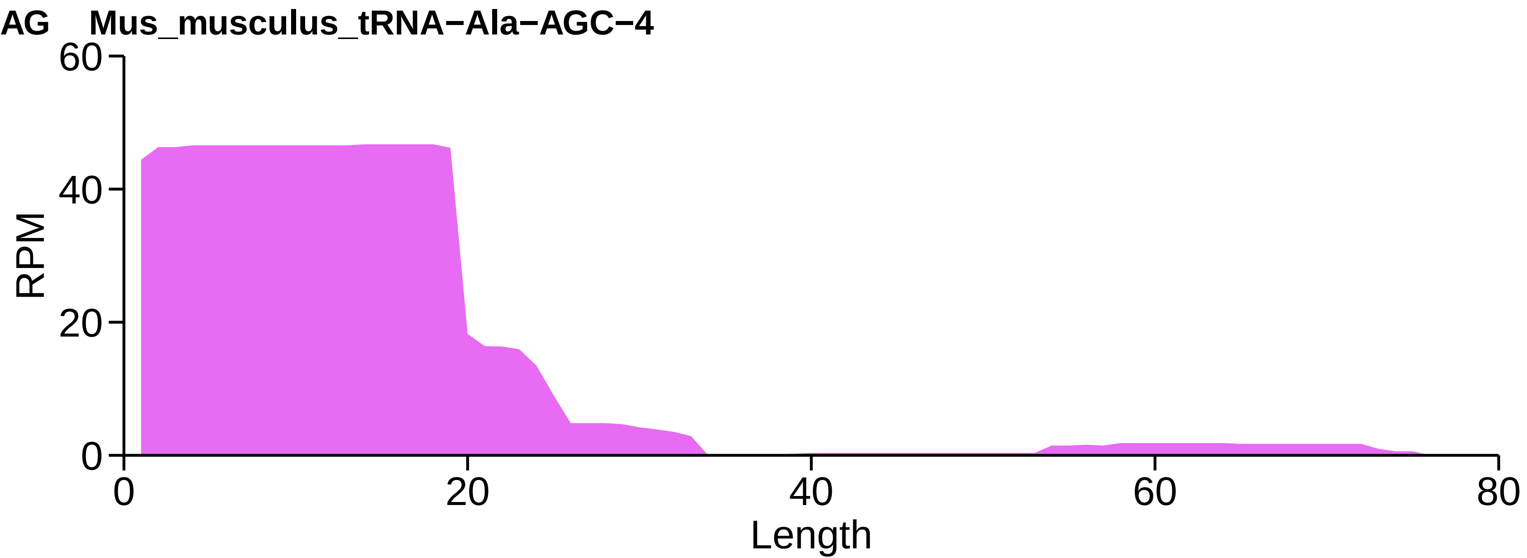
AG Mus_musculus_tRNA−Ala−AGC−4
60
40
20
0
RPM
0
20
40
60
80
Length

AH Mus_musculus_tRNA−Ala−AGC−5

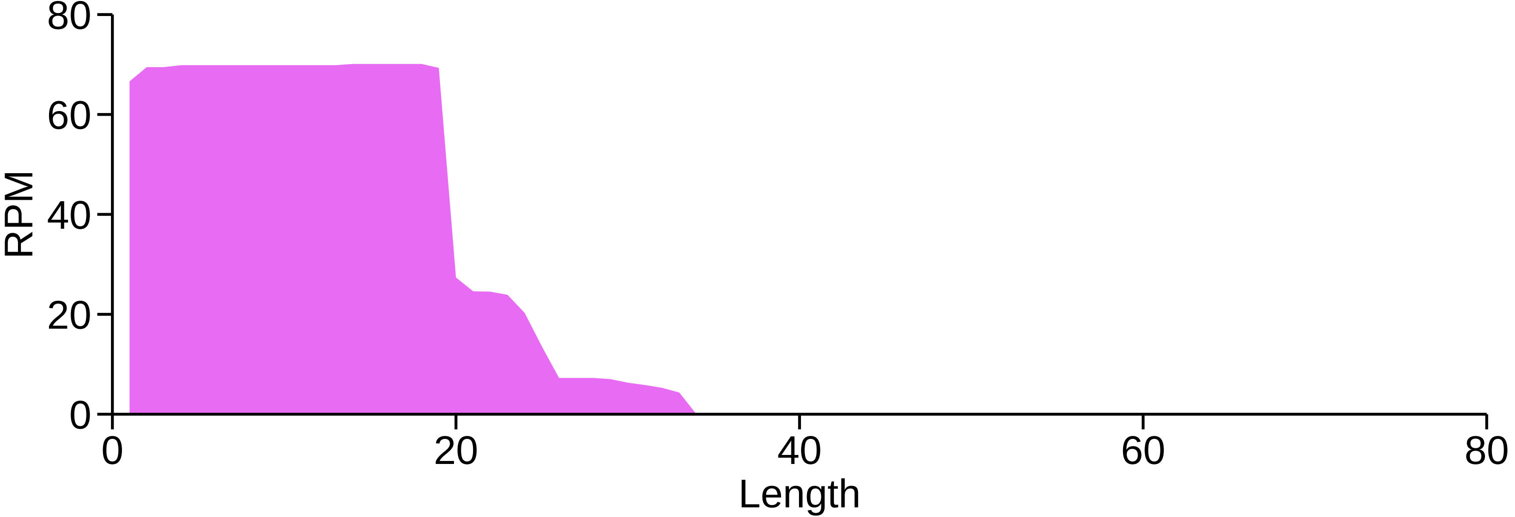

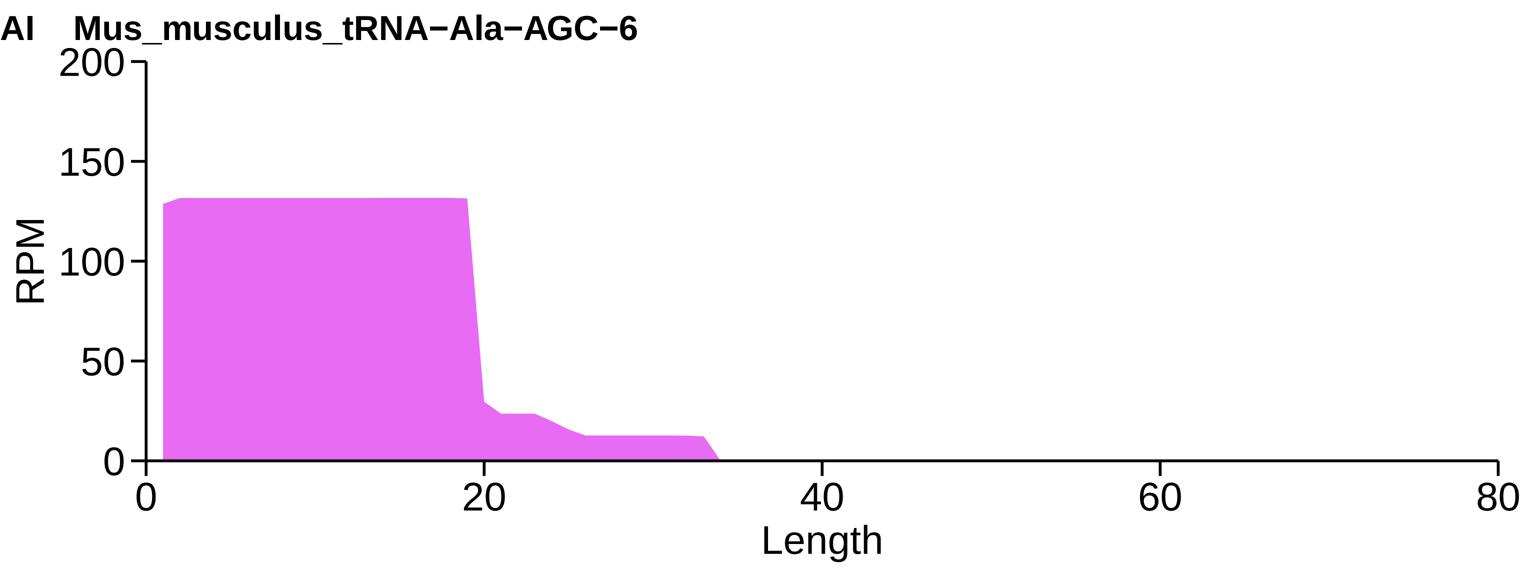
AI Mus_musculus_tRNA−Ala−AGC−6
200
150
100
50
0
RPM
0
20
40
60
80
Length

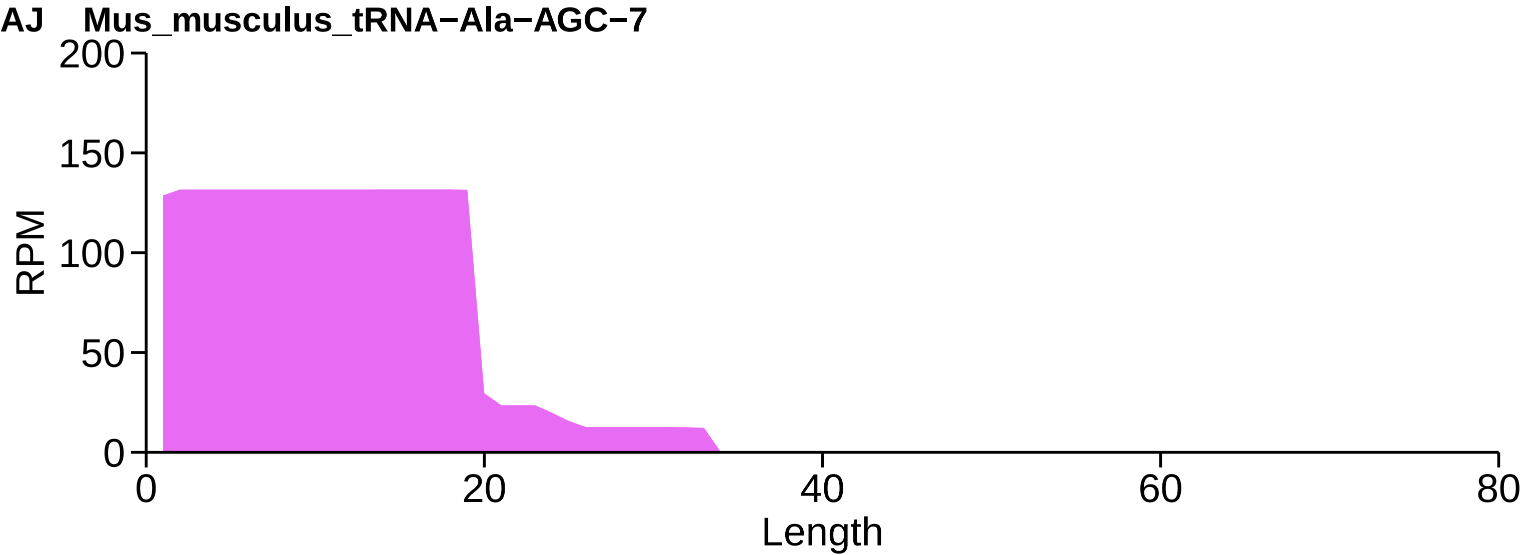
AJ Mus_musculus_tRNA−Ala−AGC−7
200
150
100
50
0
RPM
0
20
40
60
80
Length

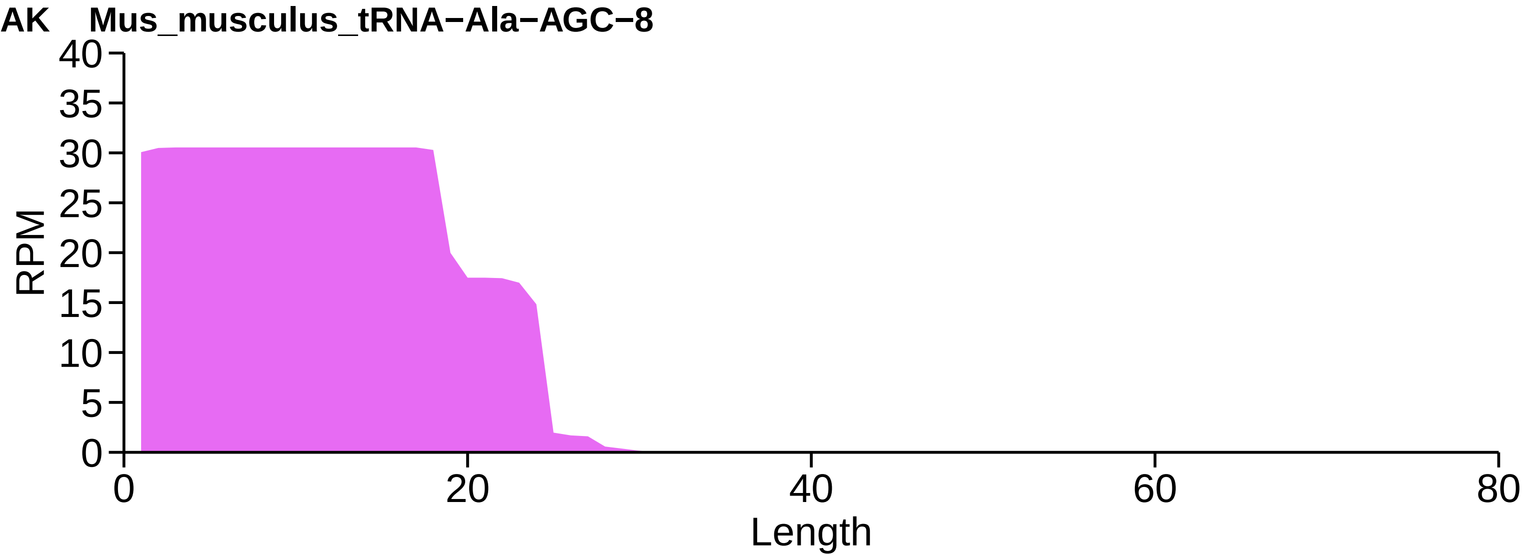
AK Mus_musculus_tRNA−Ala−AGC−8
RPM
Length
0
5
10
15
20
25
30
35
40
0
20
40
60
80

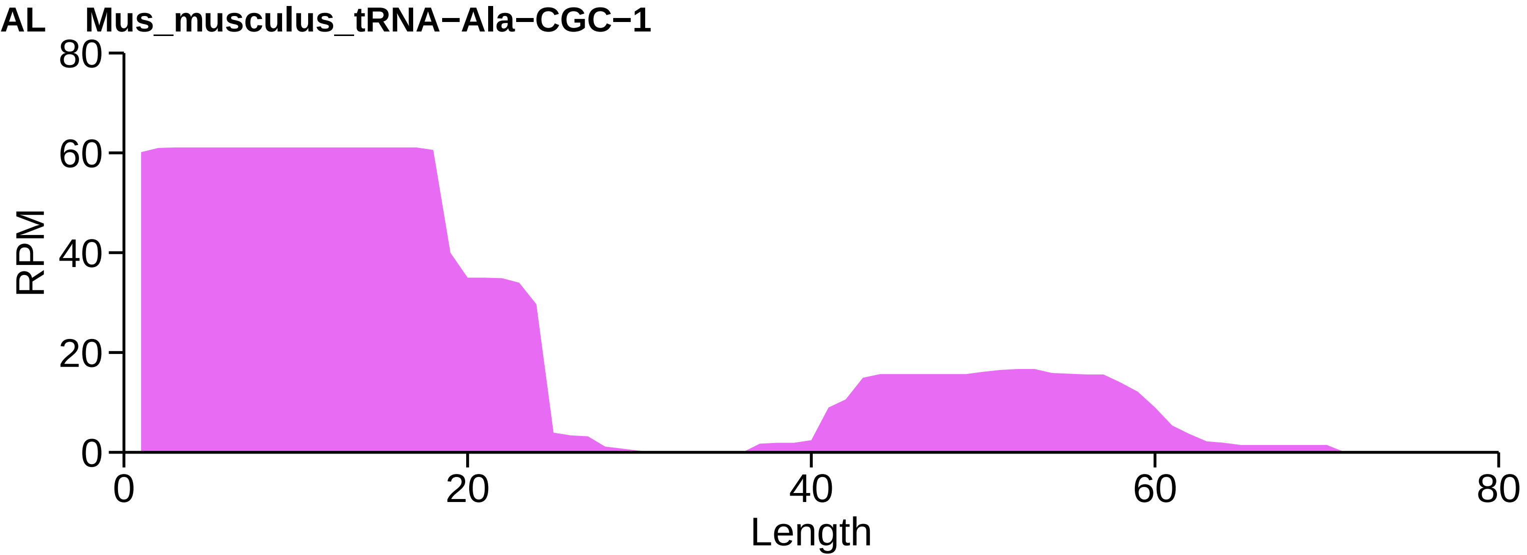

AL Mus_musculus_tRNA−Ala−CGC−1
80
60
40
20
0
RPM
0
20
40
60
80
Length

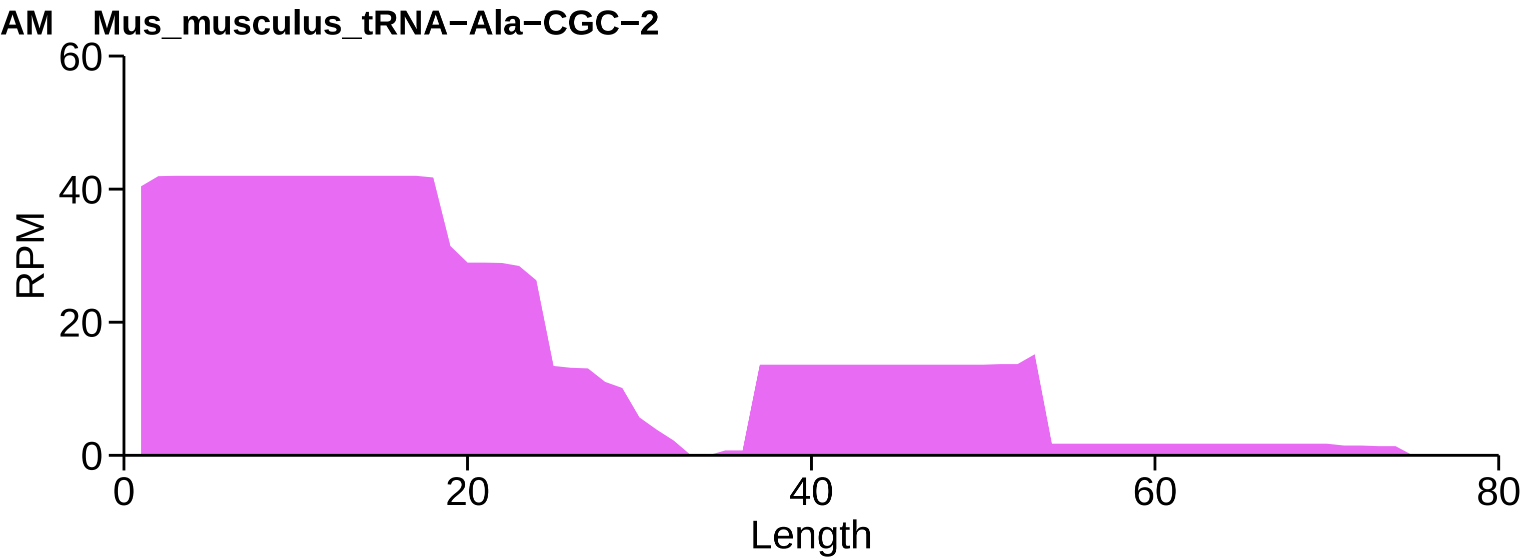

AM Mus_musculus_tRNA−Ala−CGC−2
RPM
Length
0
20
40
60
0
20
40
60
80

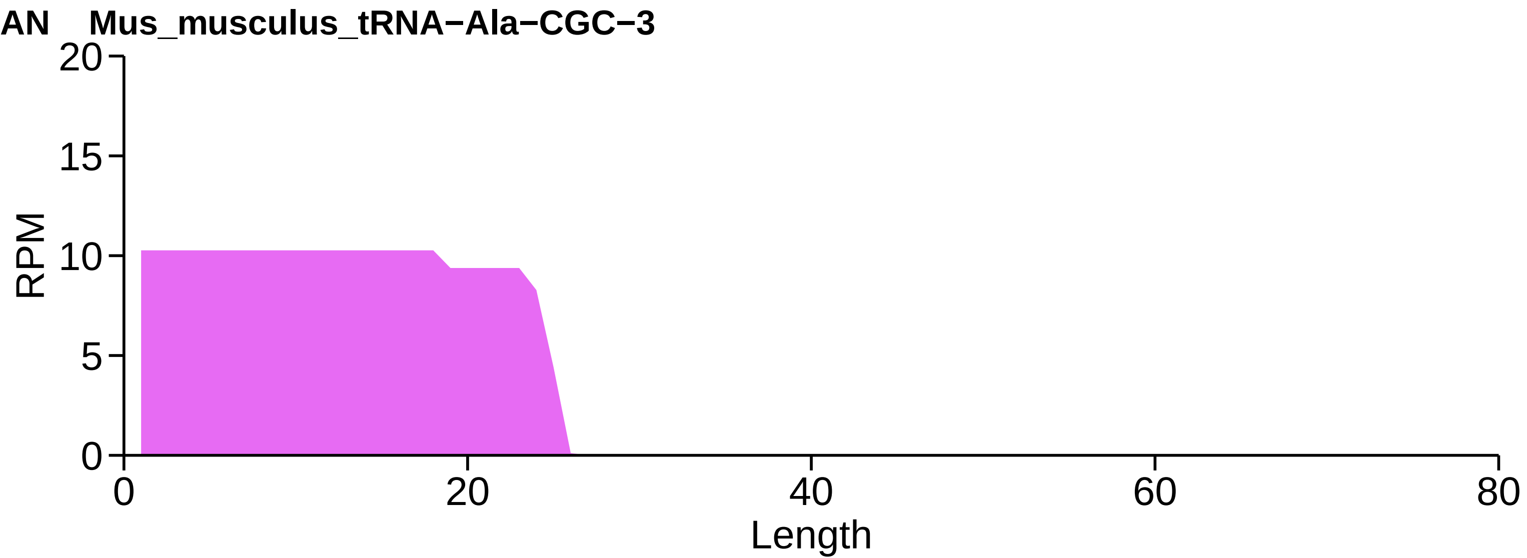

AN Mus_musculus_tRNA−Ala−CGC−3
RPM
20
15
10
5
0
0
20
40
60
80
Length

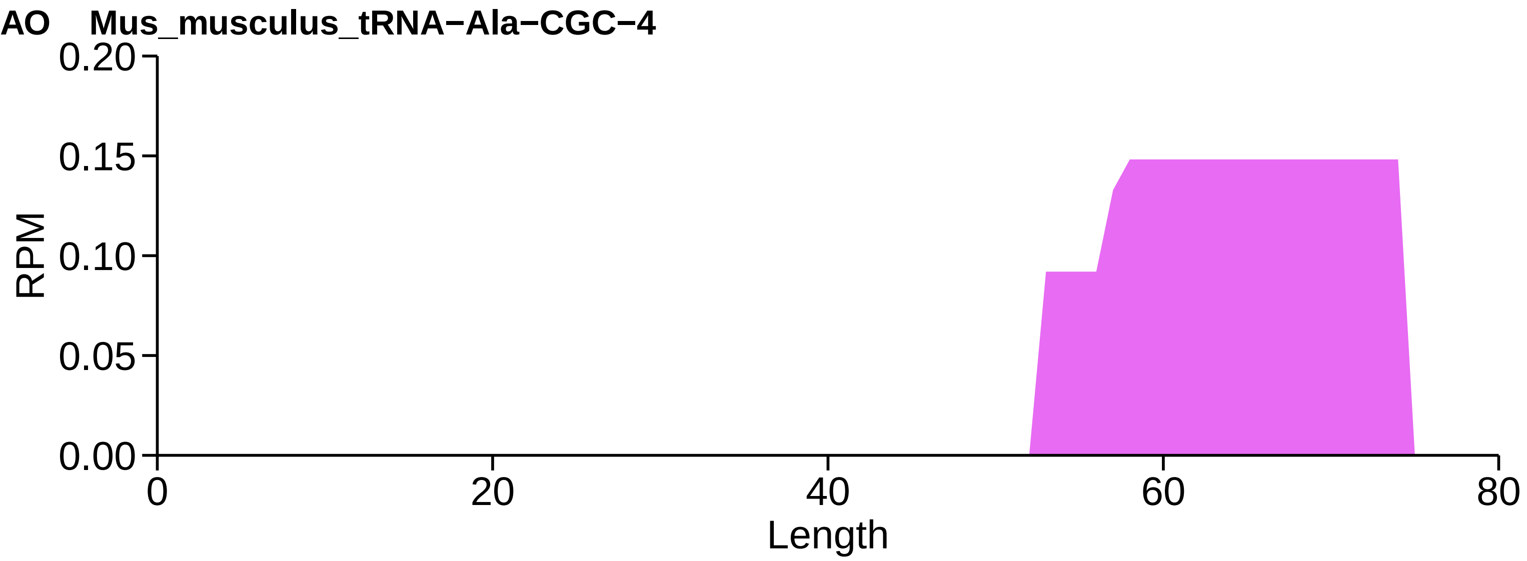

AO Mus_musculus_tRNA−Ala−CGC−4
0.20
0.15
0.10
0.05
0.00
RPM
0
20
40
60
80
Length

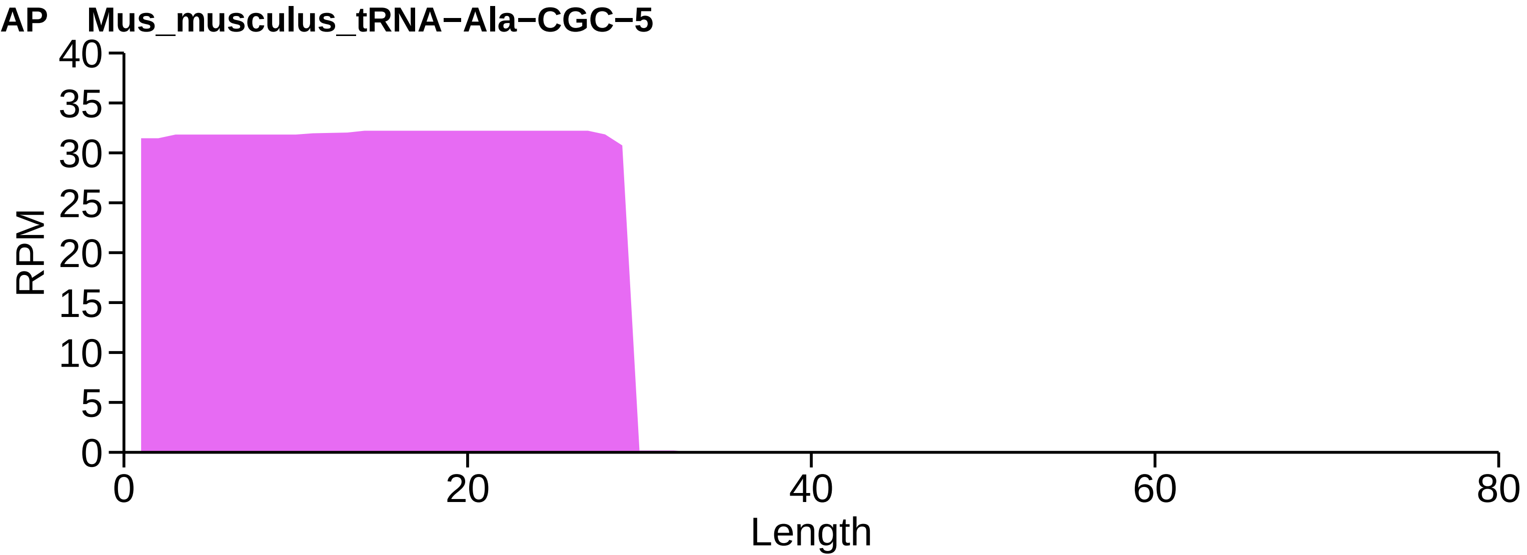
AP Mus_musculus_tRNA−Ala−CGC−5
RPM
Length
0
5
10
15
20
25
30
35
40
0
20
40
60
80

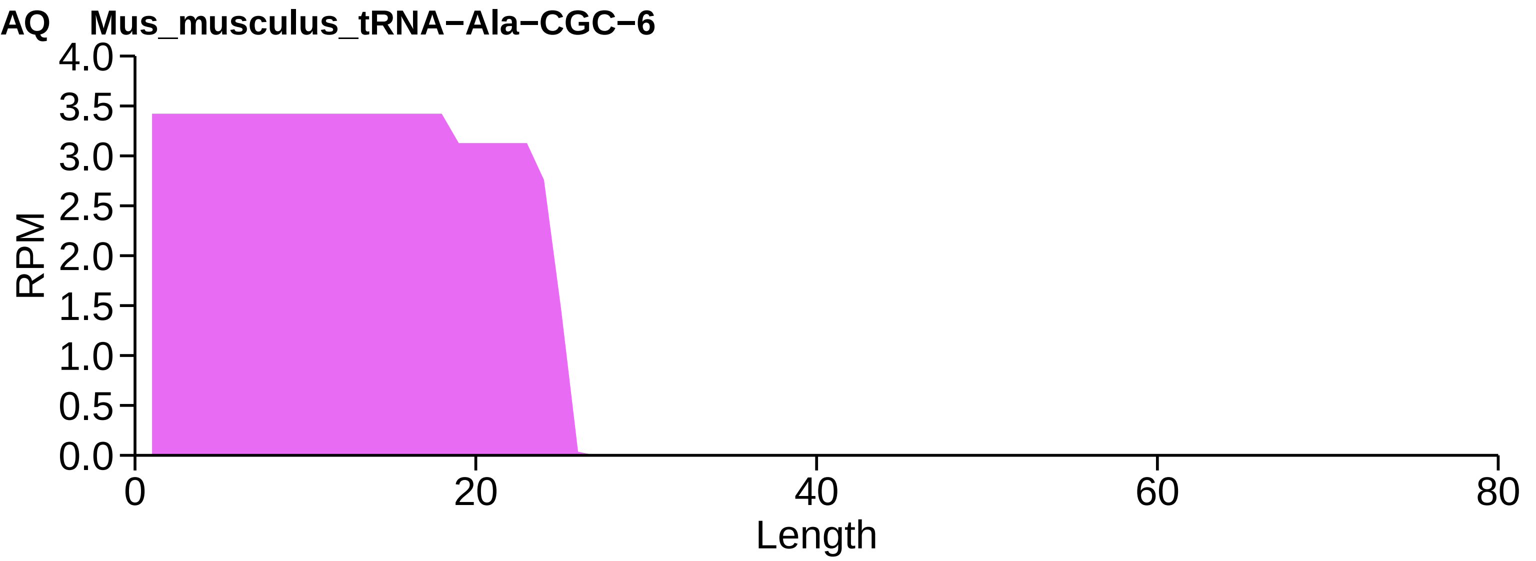
AQ Mus_musculus_tRNA−Ala−CGC−6
RPM
4.0
3.5
3.0
2.5
2.0
1.5
1.0
0.5
0.0
0
20
40
60
80
Length

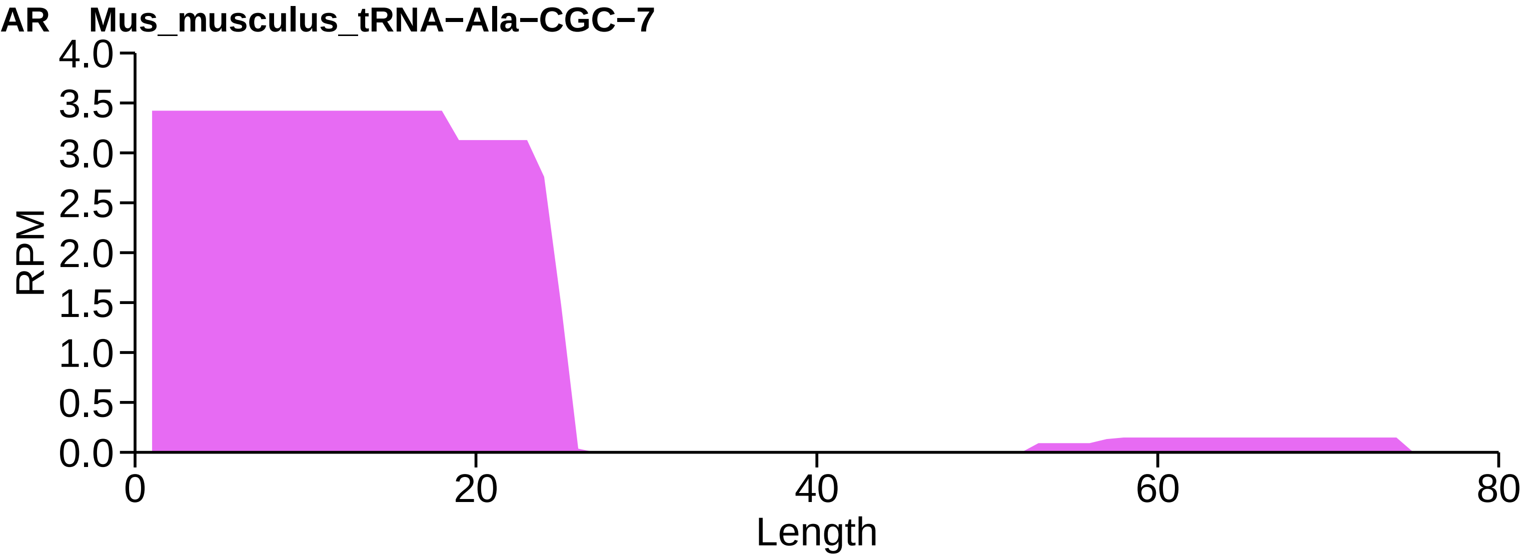
AR Mus_musculus_tRNA−Ala−CGC−7
RPM
4.0
3.5
3.0
2.5
2.0
1.5
1.0
0.5
0.0
0
20
40
60
80
Length

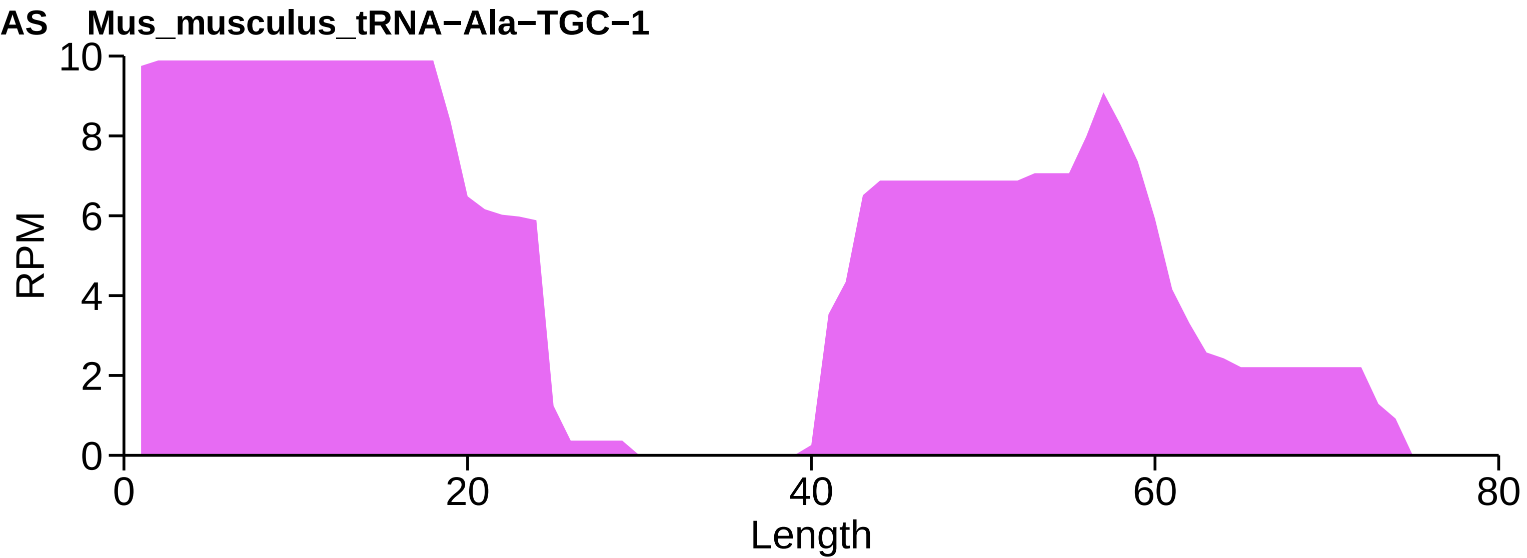

AS Mus_musculus_tRNA−Ala−TGC−1
RPM
0
2
4
6
8
10
0
20
40
60
80
Length

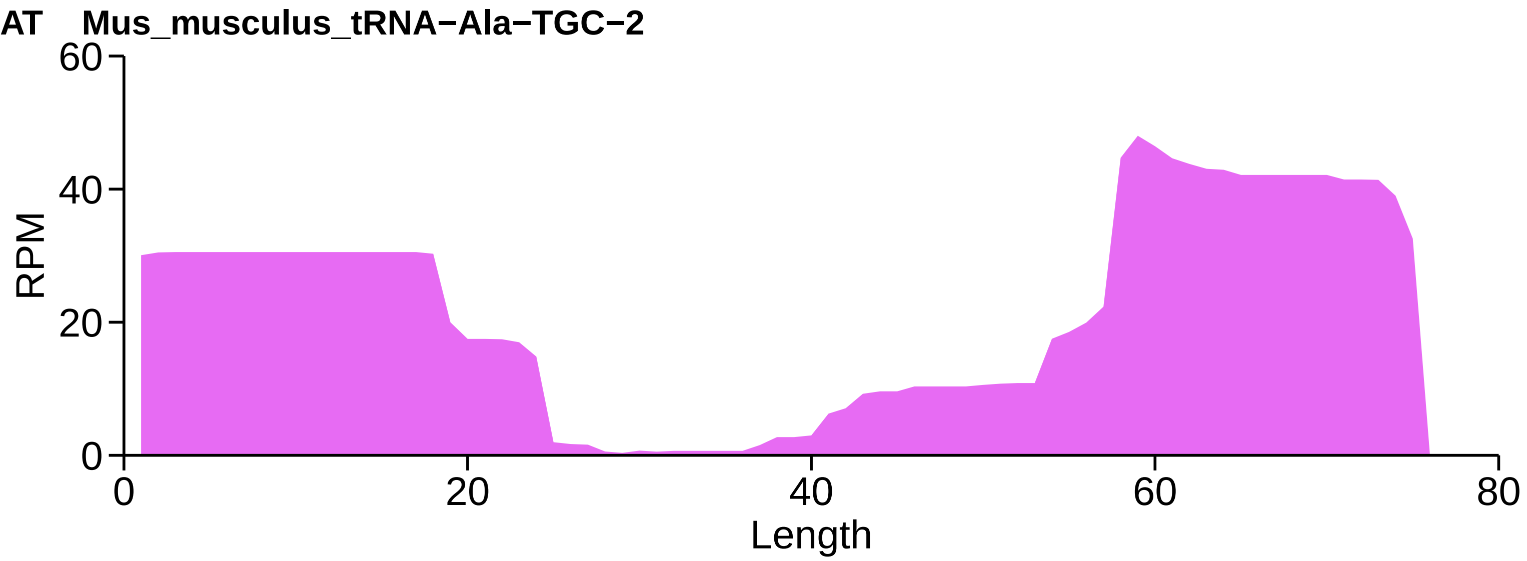
AT Mus_musculus_tRNA−Ala−TGC−2
60
40
20
0
RPM
0
20
40
60
80
Length

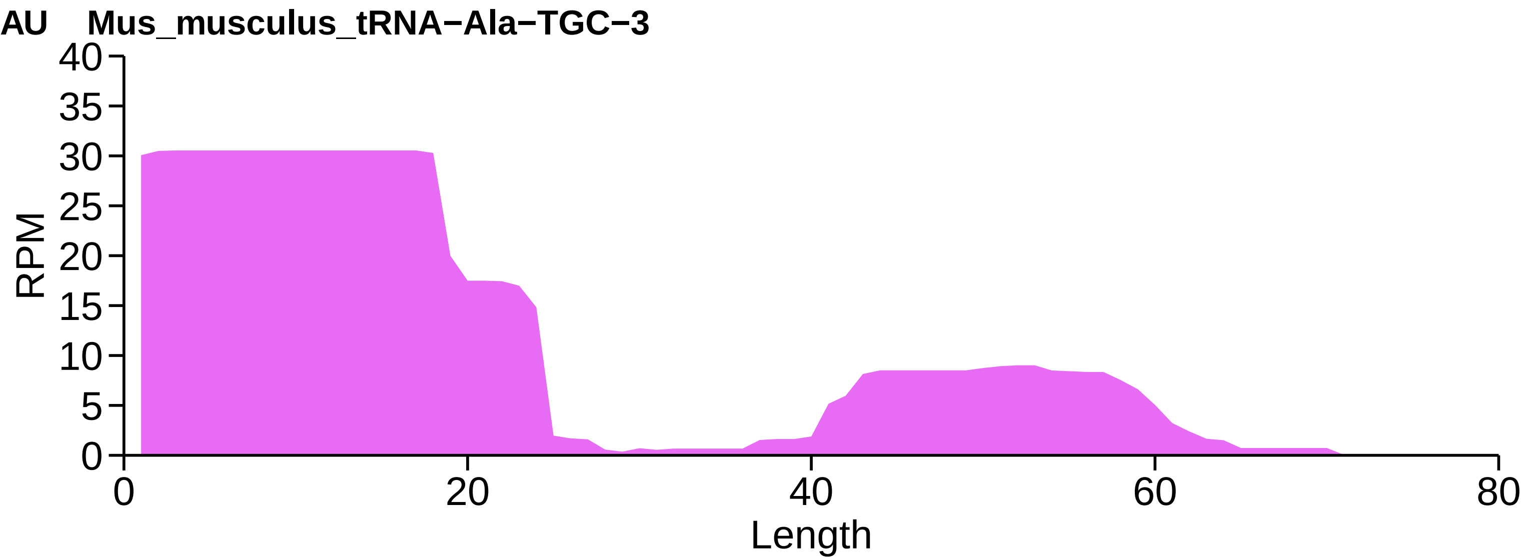
AU Mus_musculus_tRNA−Ala−TGC−3
RPM
0
5
10
15
20
25
30
35
40
0
20
40
60
80
Length

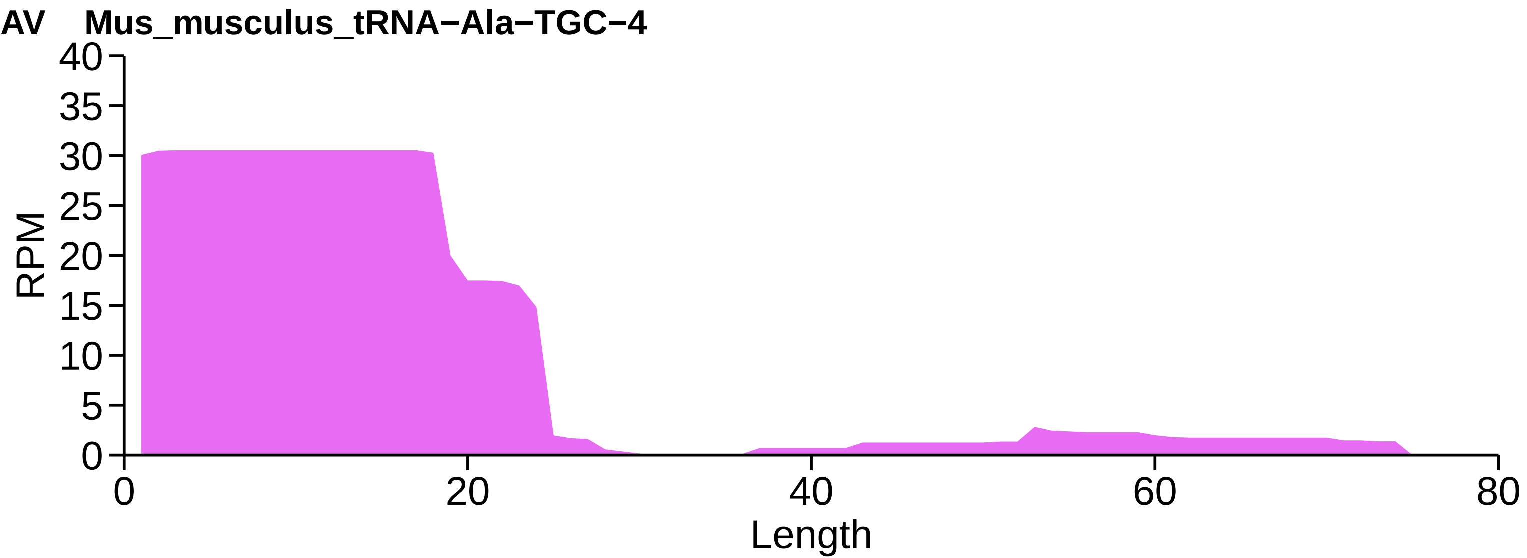

AV Mus_musculus_tRNA−Ala−TGC−4
RPM
0
5
10
15
20
25
30
35
40
0
20
40
60
80
Length

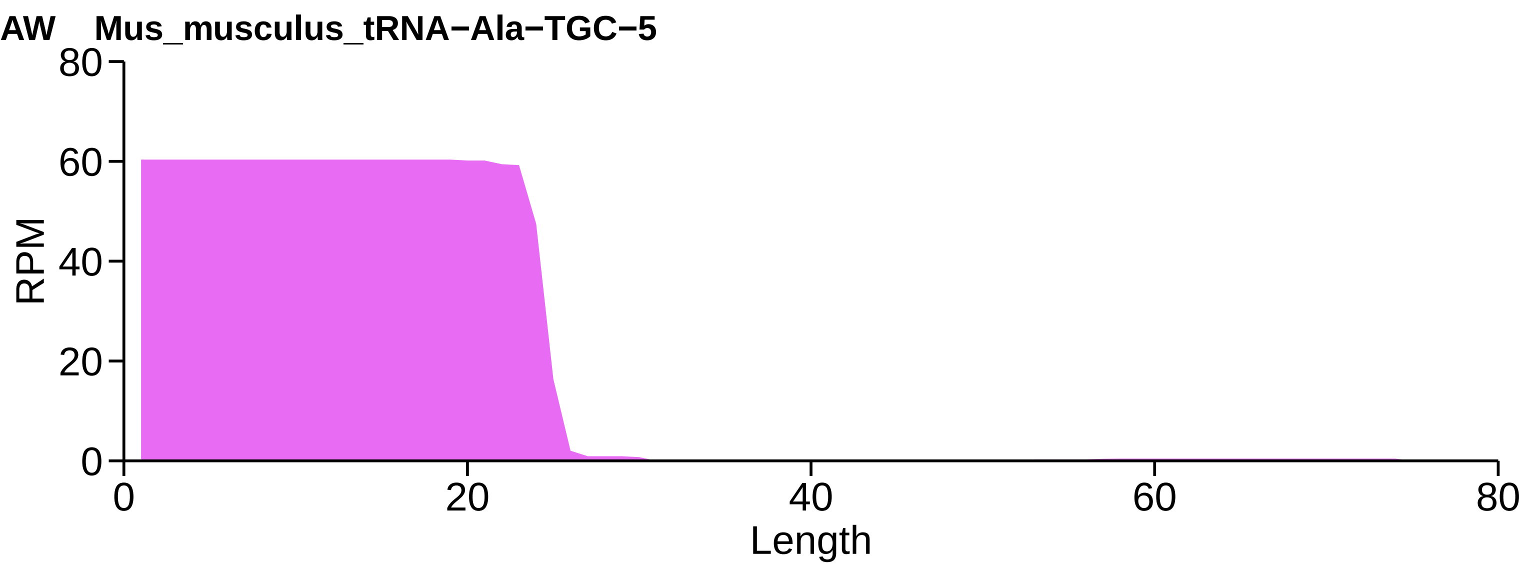
AW Mus_musculus_tRNA−Ala−TGC−5
RPM
0
20
40
60
80
0
20
40
60
80
Length

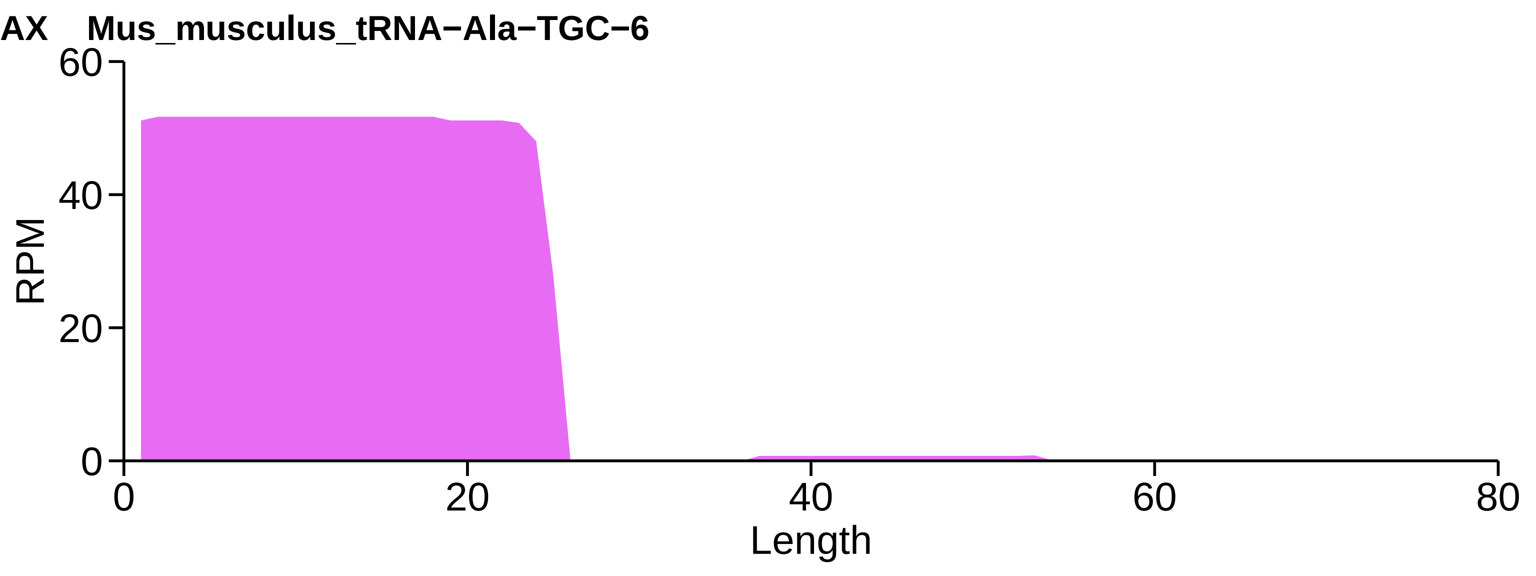
AX Mus_musculus_tRNA−Ala−TGC−6
60
40
20
0
RPM
0
20
40
60
80
Length

AY Mus_musculus_tRNA−Ala−TGC−7

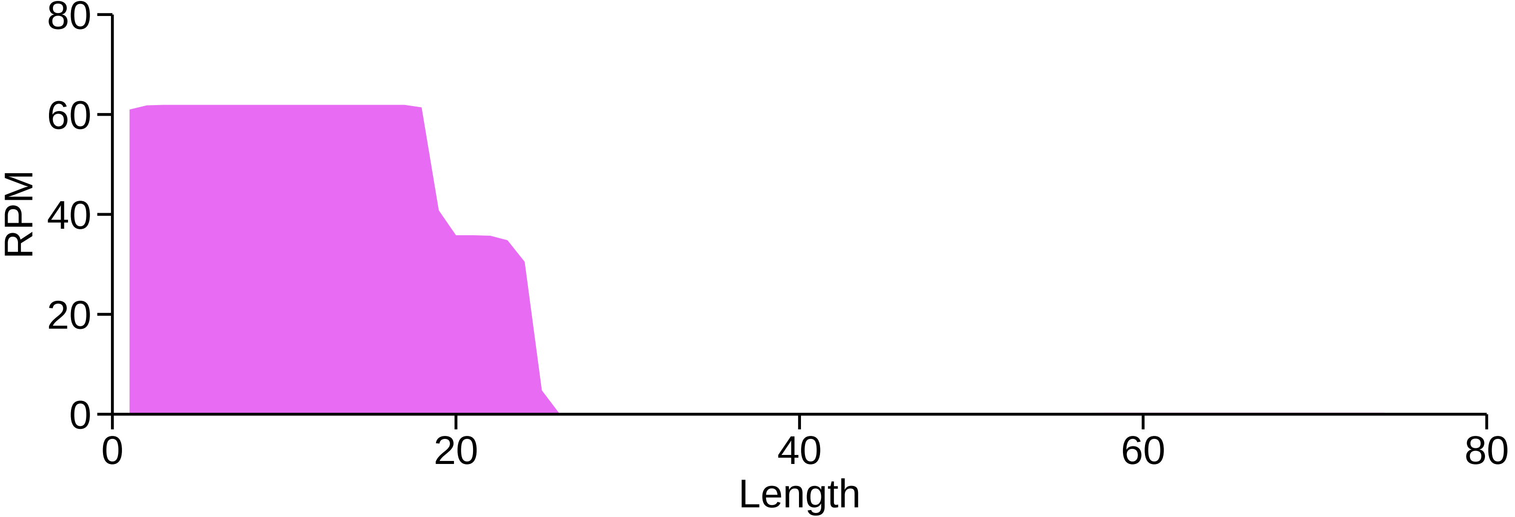

AZ Mus_musculus_tRNA−Ala−TGC−8

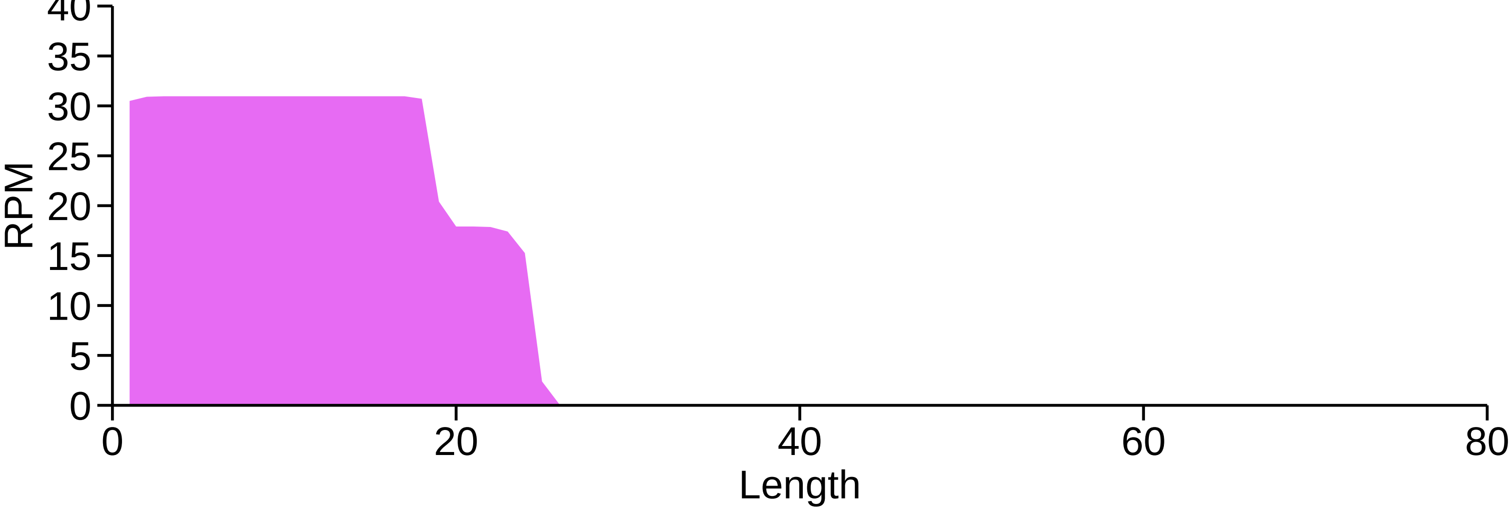

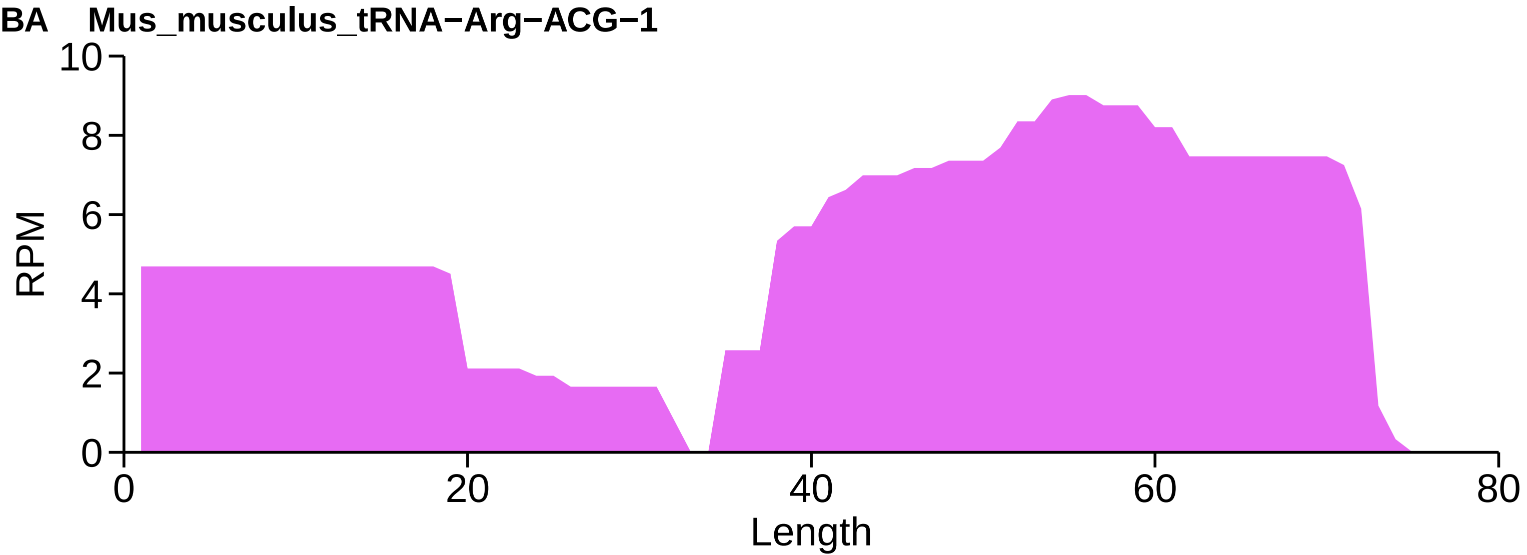

BA Mus_musculus_tRNA−Arg−ACG−1
RPM
Length
0
2
4
6
8
10
0
20
40
60
80

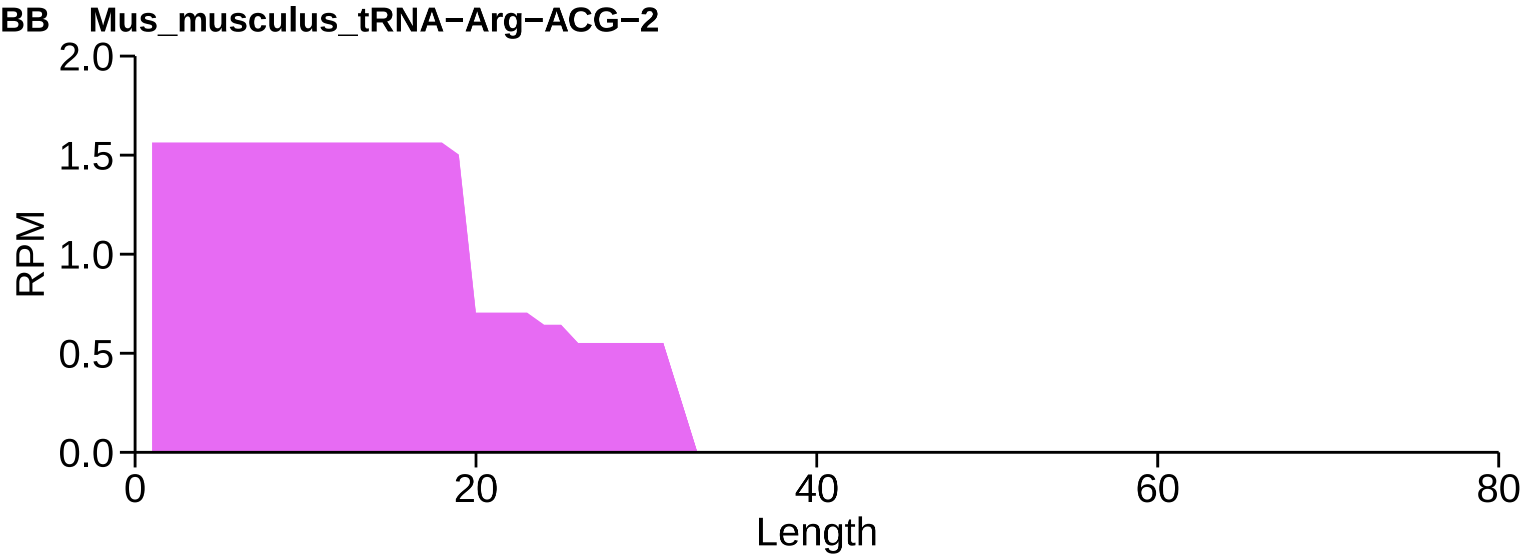
BB Mus_musculus_tRNA−Arg−ACG−2
2.0
1.5
1.0
0.5
0.0
RPM
0
20
40
60
80
Length

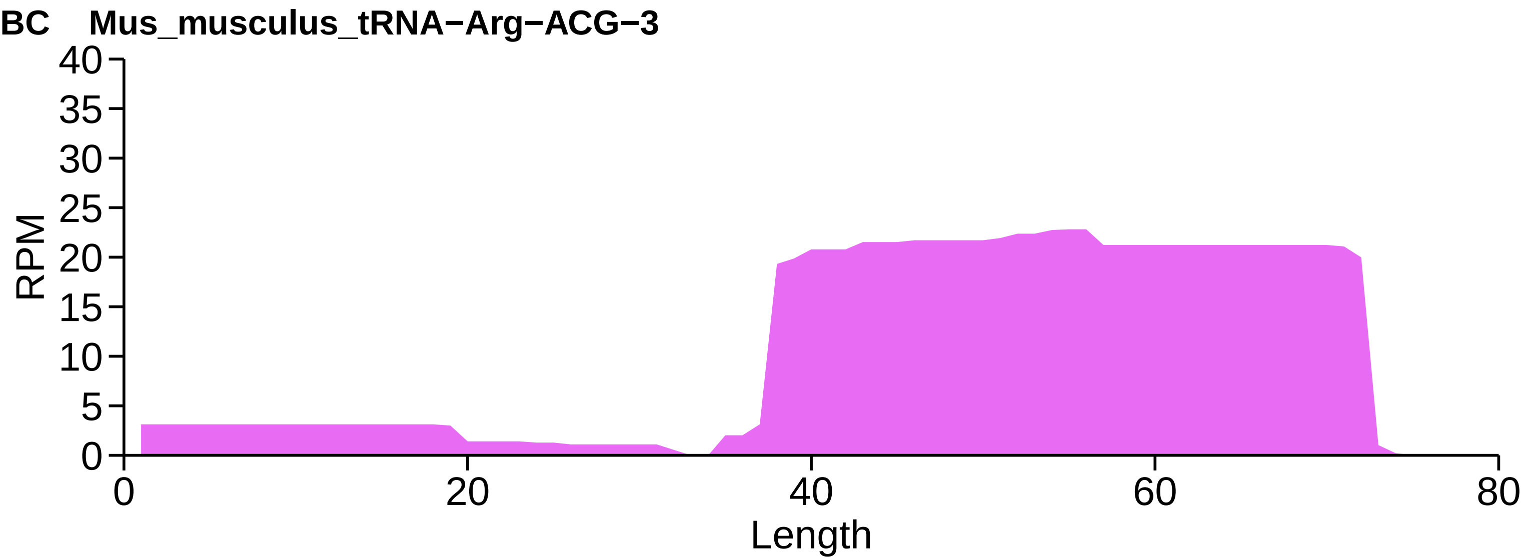

BC Mus_musculus_tRNA−Arg−ACG−3
RPM
0
5
10
15
20
25
30
35
40
0
20
40
60
80
Length

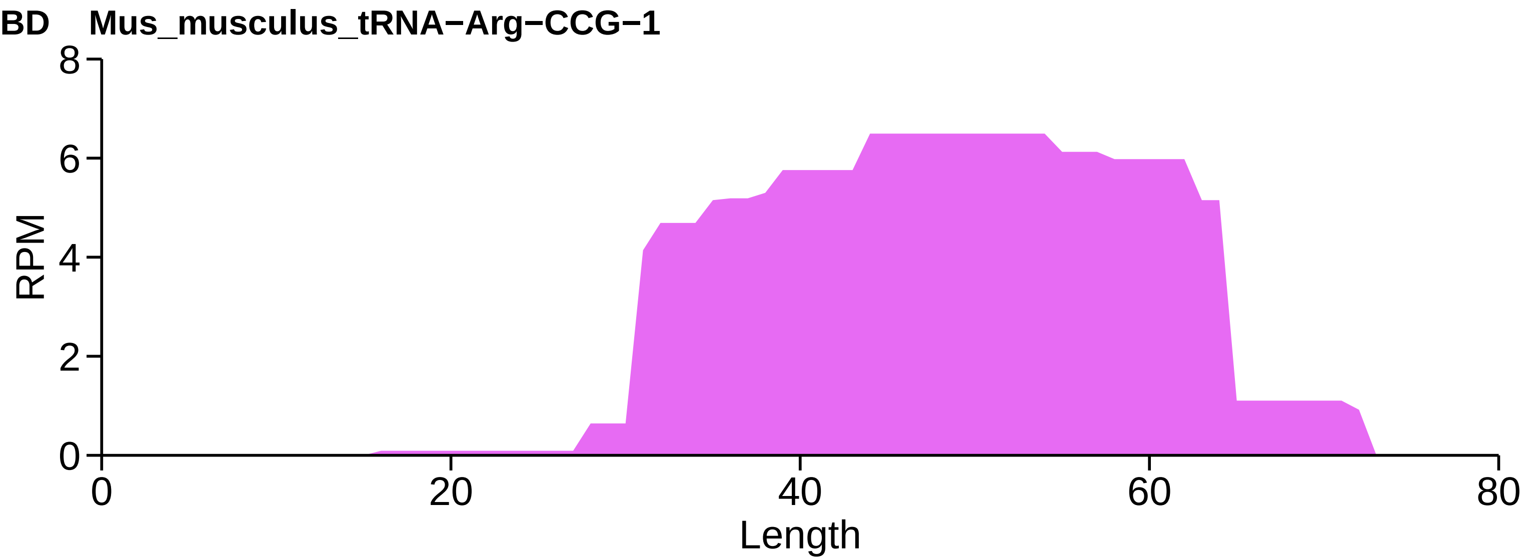
BD Mus_musculus_tRNA−Arg−CCG−1
RPM
Length
0
2
4
6
8
0
20
40
60
80

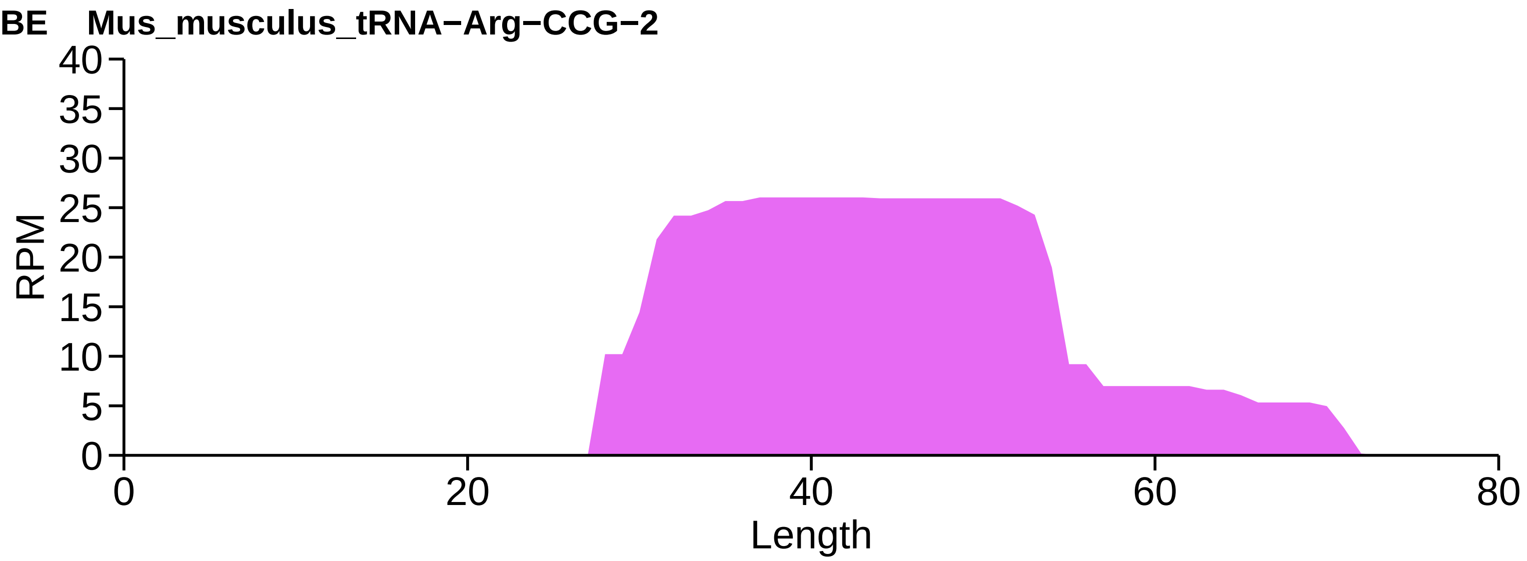
BE Mus_musculus_tRNA−Arg−CCG−2
RPM
0
5
10
15
20
25
30
35
40
Length
0
20
40
60
80

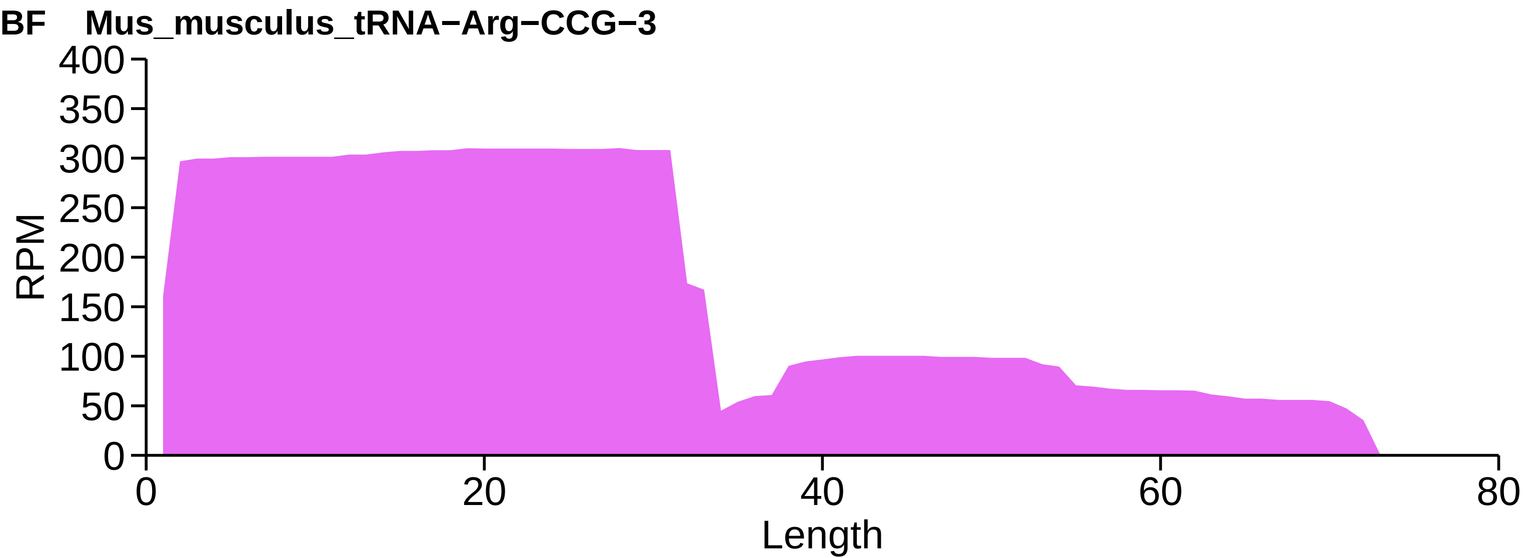
BF Mus_musculus_tRNA−Arg−CCG−3
RPM
0
50
100
150
200
250
300
350
400
0
20
40
60
80
Length

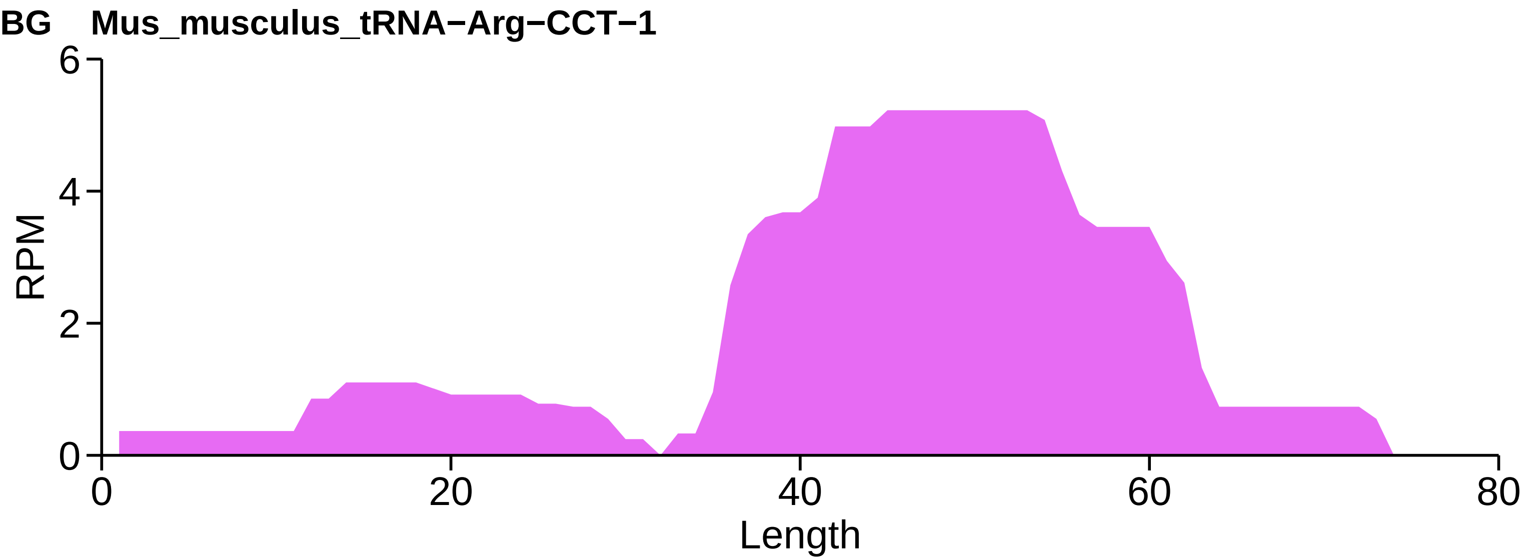
BG Mus_musculus_tRNA−Arg−CCT−1
RPM
Length
0
2
4
6
0
20
40
60
80

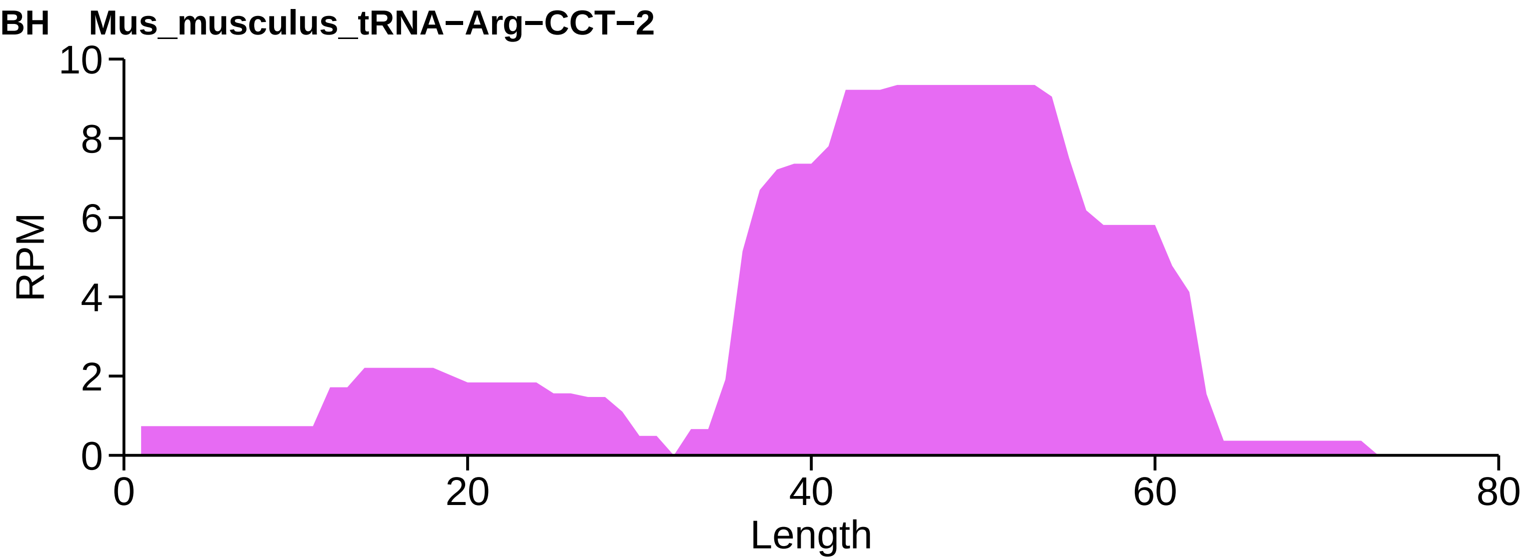
BH Mus_musculus_tRNA−Arg−CCT−2
RPM
Length
0
2
4
6
8
10
0
20
40
60
80

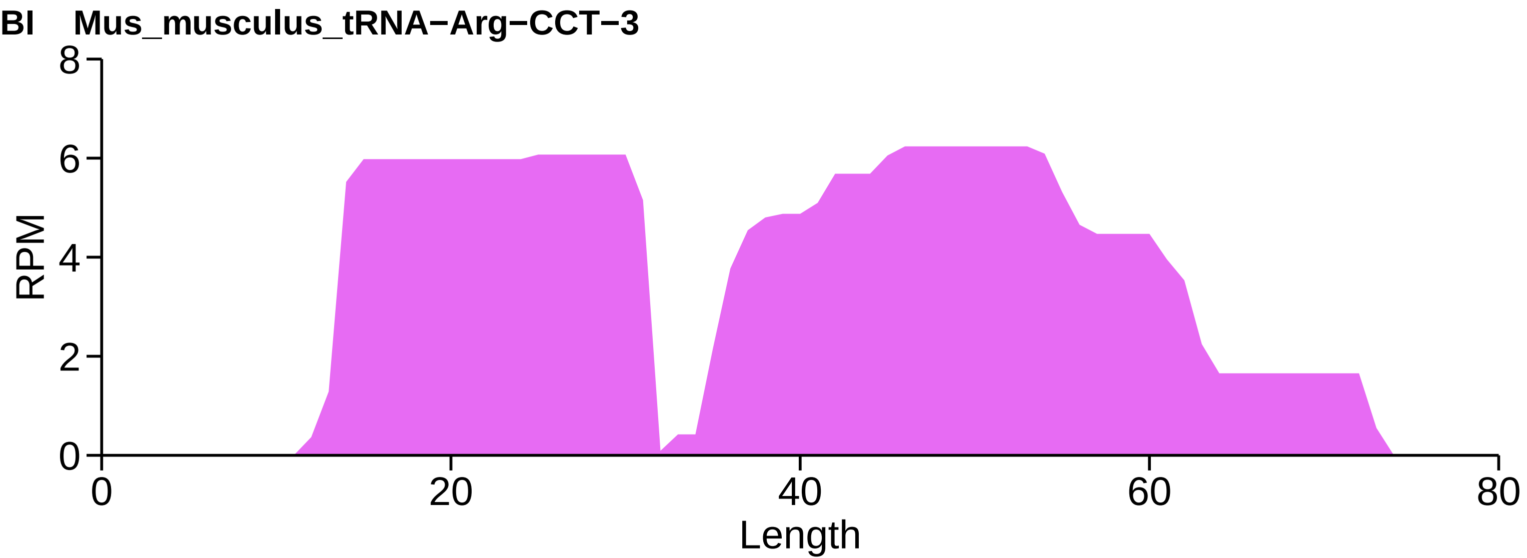

BI Mus_musculus_tRNA−Arg−CCT−3
RPM
8
6
4
2
0
0
20
40
60
80
Length

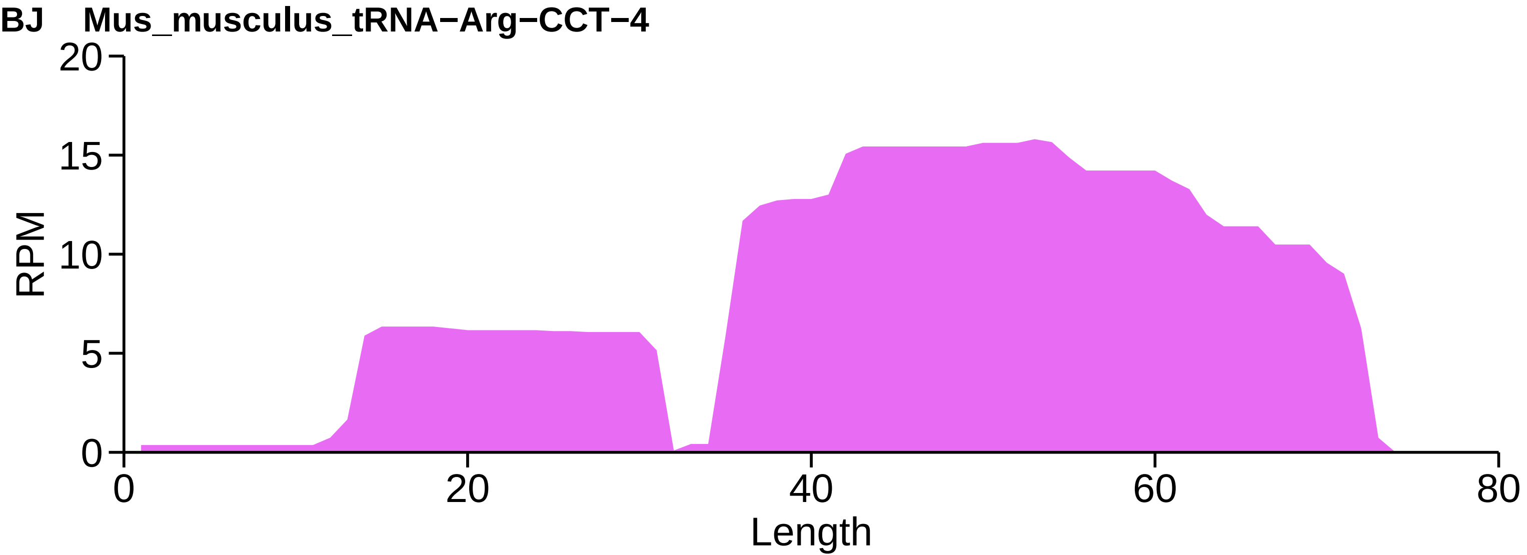

BJ Mus_musculus_tRNA−Arg−CCT−4
RPM
20
15
10
5
0
0
20
40
60
80
Length

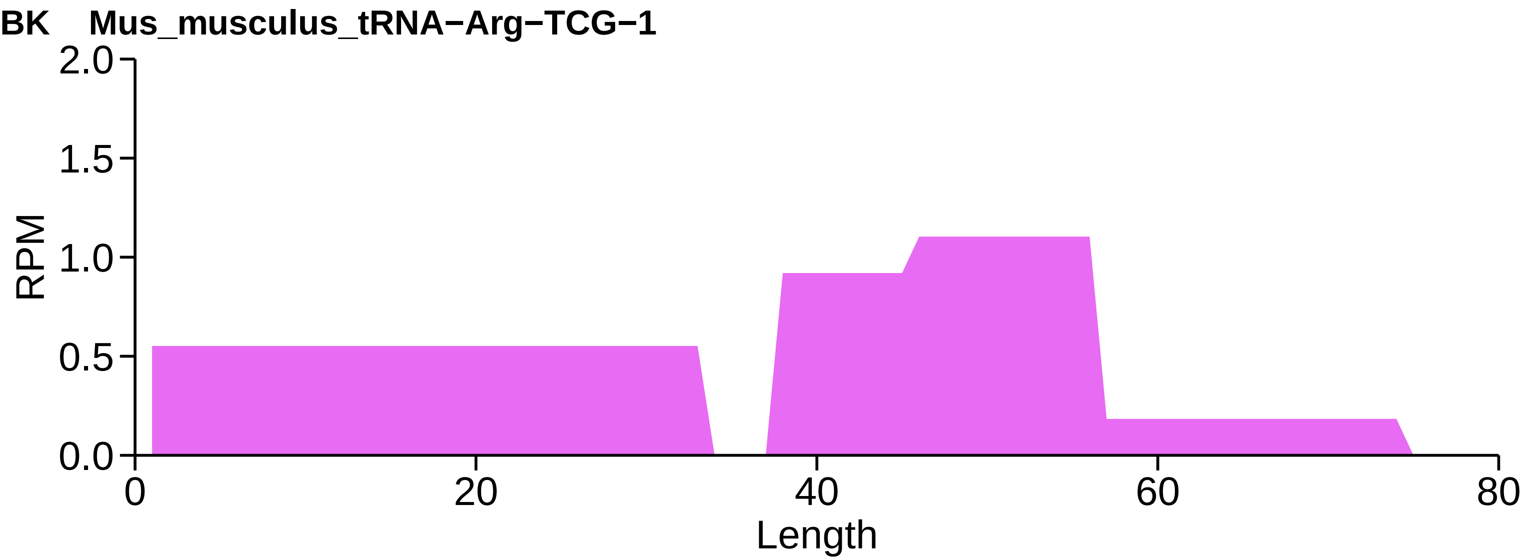
BK Mus_musculus_tRNA−Arg−TCG−1
RPM
2.0
1.5
1.0
0.5
0.0
0
20
40
60
80
Length

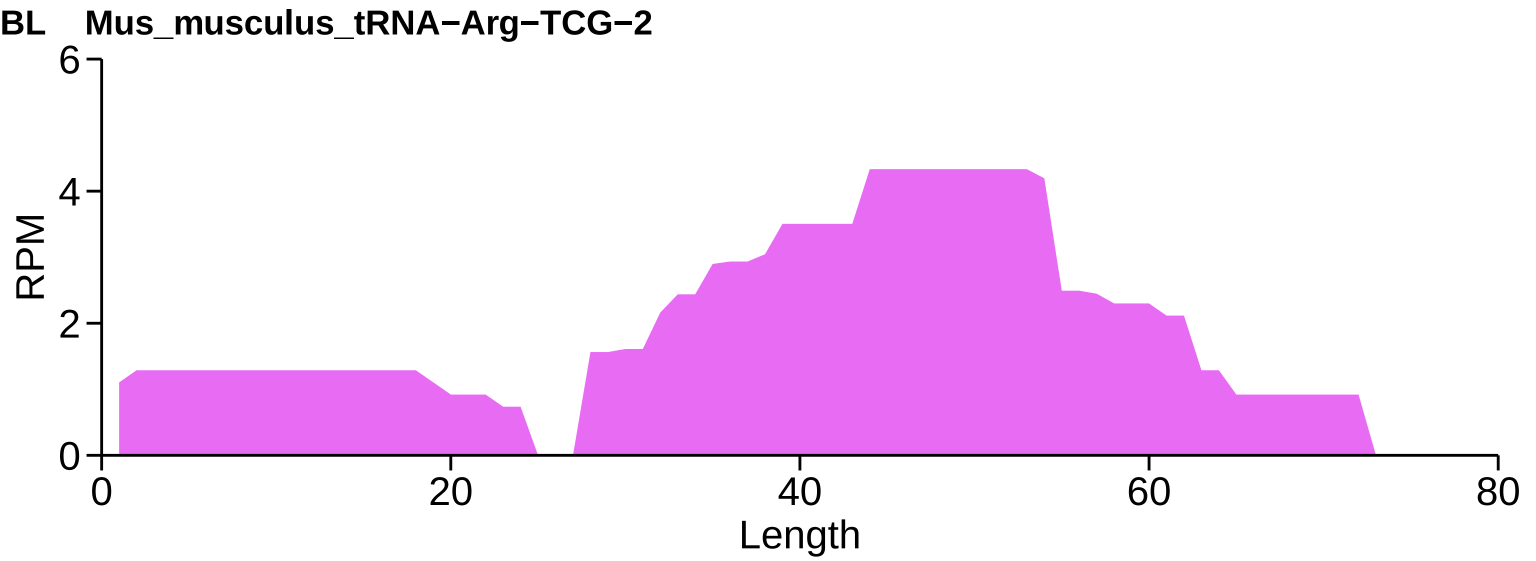

BL
Mus_musculus_tRNA−Arg−TCG−2
RPM
0
2
4
6
0
20
40
60
80
Length

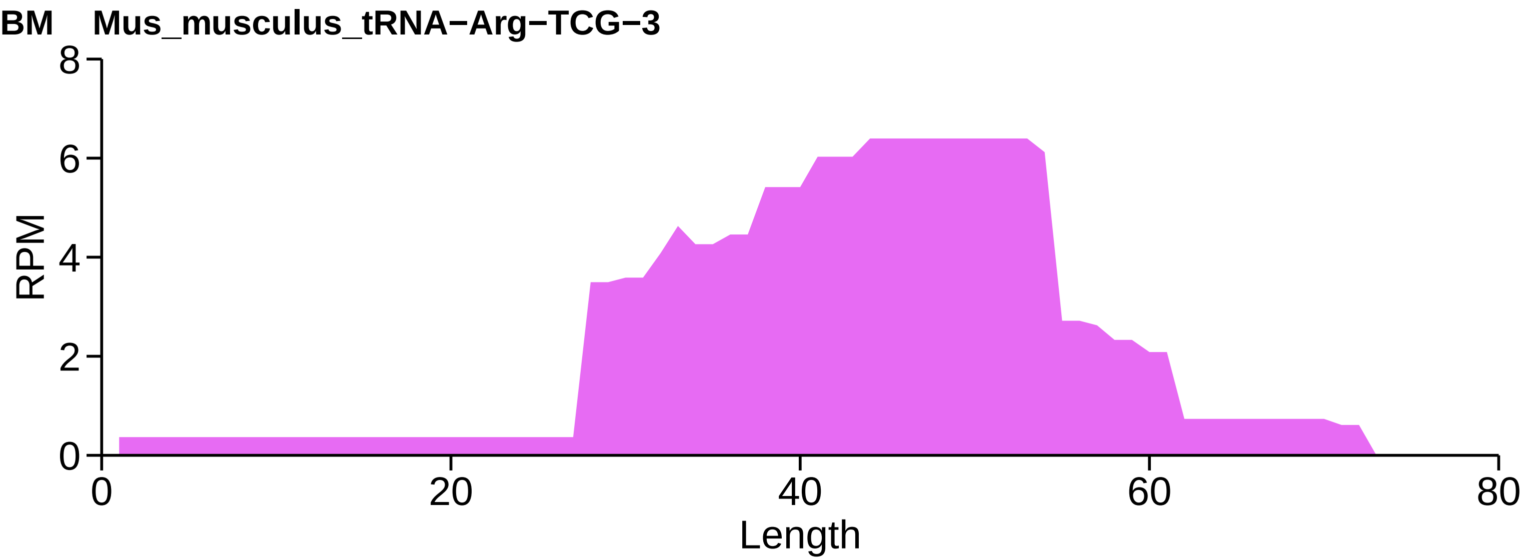
BM Mus_musculus_tRNA−Arg−TCG−3
RPM
Length
0
2
4
6
8
0
20
40
60
80

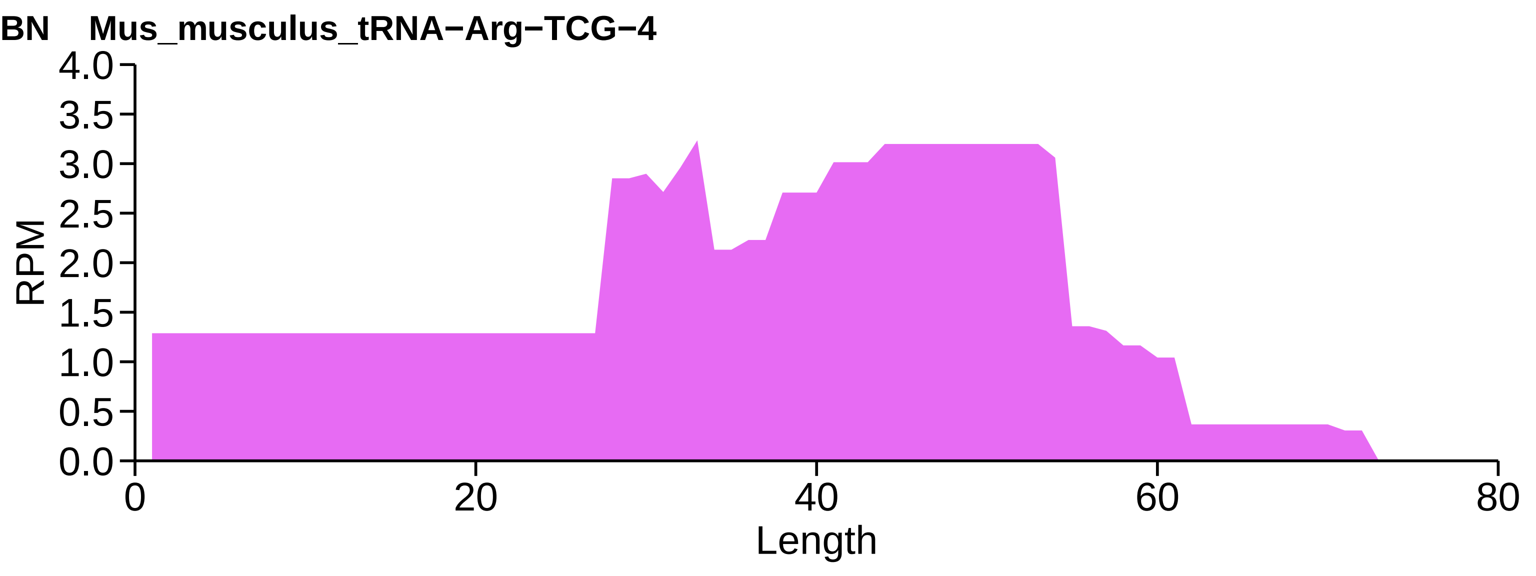

BN Mus_musculus_tRNA−Arg−TCG−4
4.0
3.5
3.0
2.5
2.0
1.5
1.0
0.5
0.0
RPM
0
20
40
60
80
Length

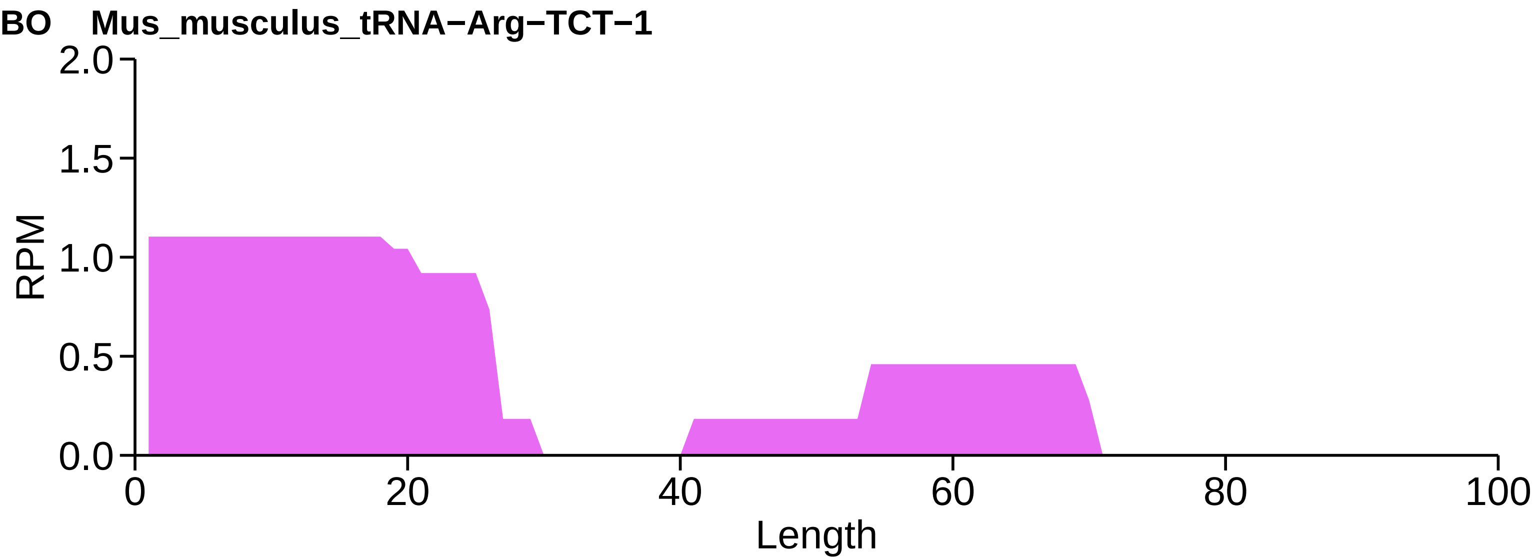
BO Mus_musculus_tRNA−Arg−TCT−1
2.0
1.5
1.0
0.5
0.0
RPM
0
20
40
60
80
100
Length

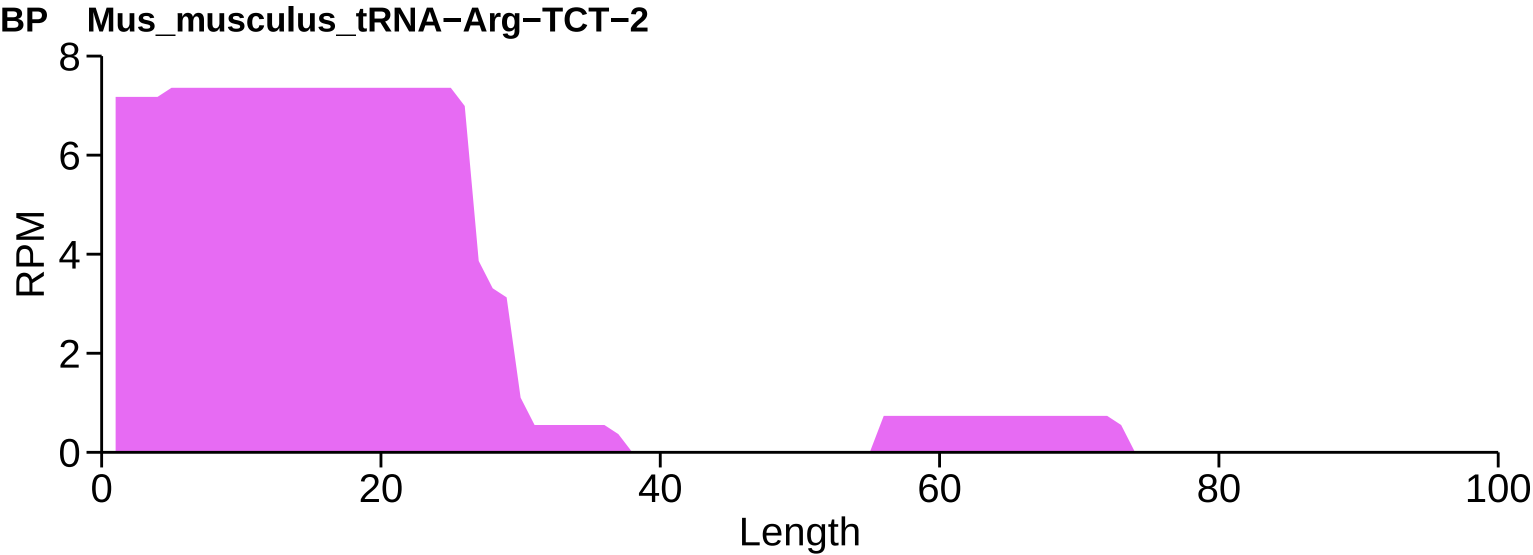
BP Mus_musculus_tRNA−Arg−TCT−2
8
6
4
2
0
RPM
0
20
40
60
80
100
Length

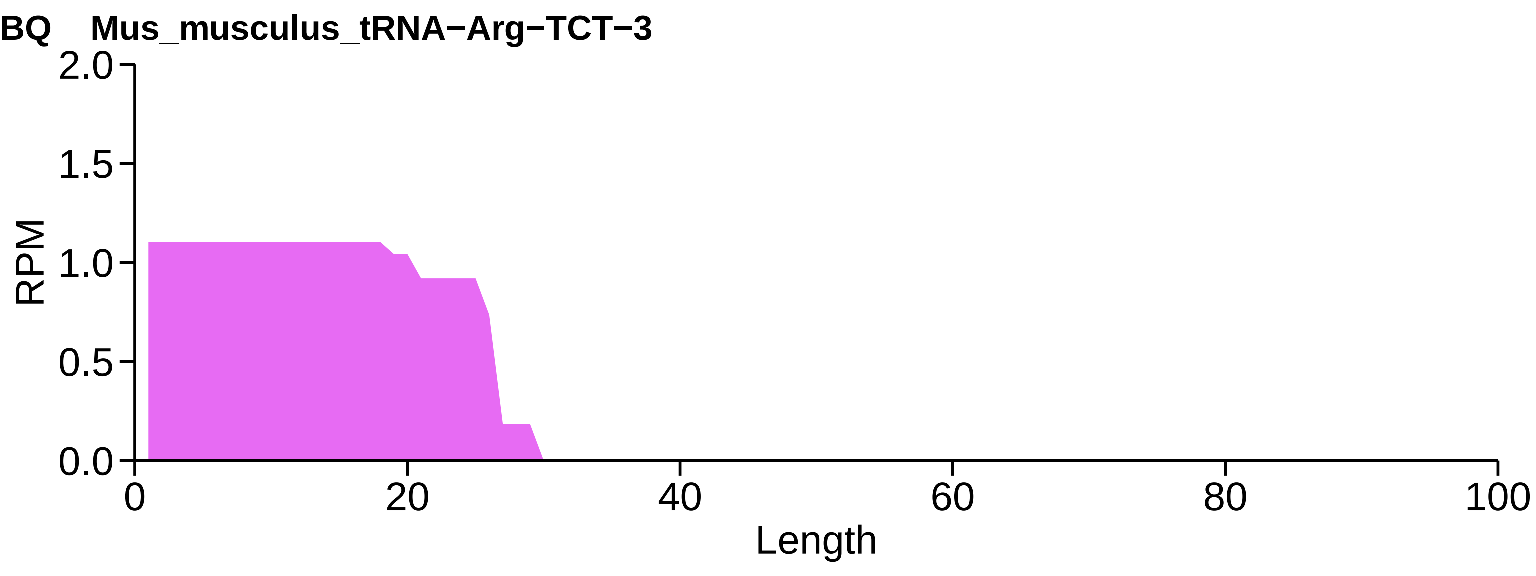

BQ Mus_musculus_tRNA−Arg−TCT−3
2.0
1.5
1.0
0.5
0.0
RPM
0
20
40
60
80
100
Length

BR Mus_musculus_tRNA−Arg−TCT−4

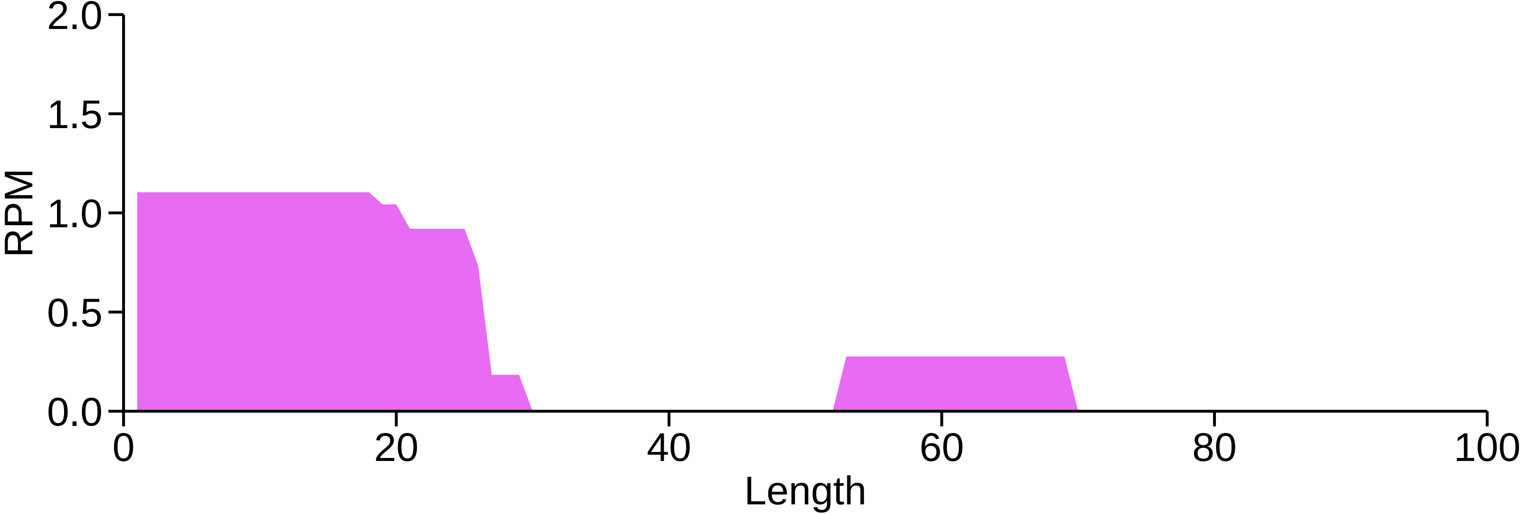

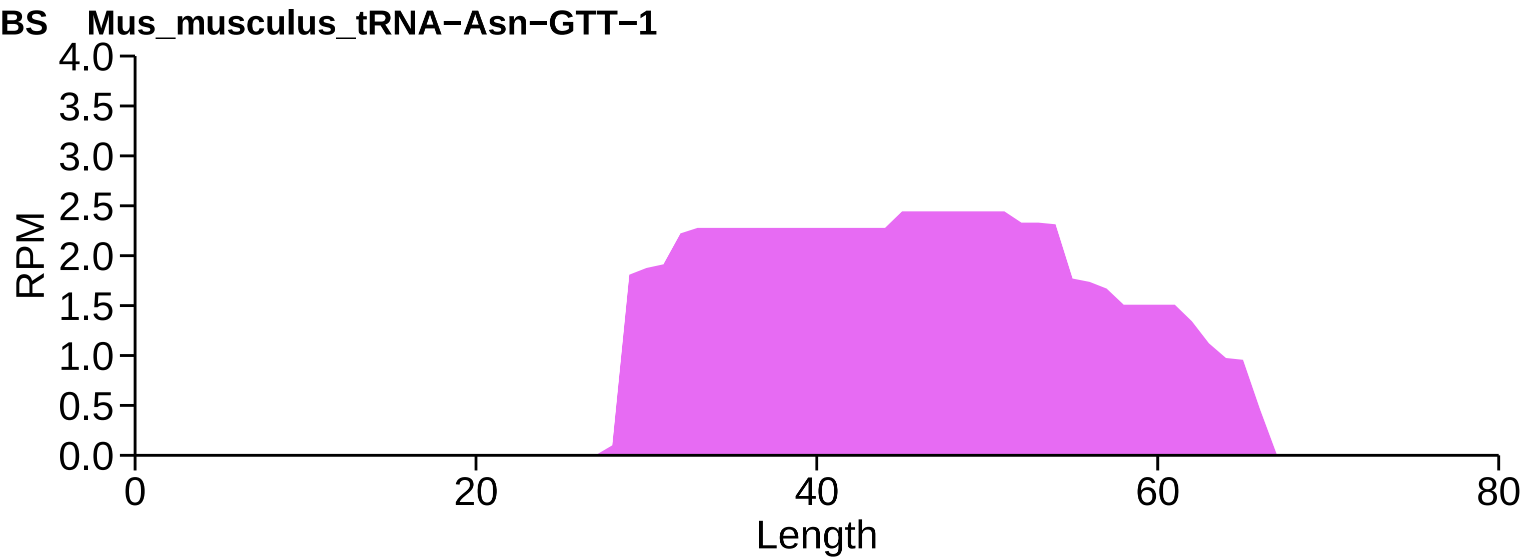
BS Mus_musculus_tRNA−Asn−GTT−1
RPM
4.0
3.5
3.0
2.5
2.0
1.5
1.0
0.5
0.0
0
20
40
60
80
Length

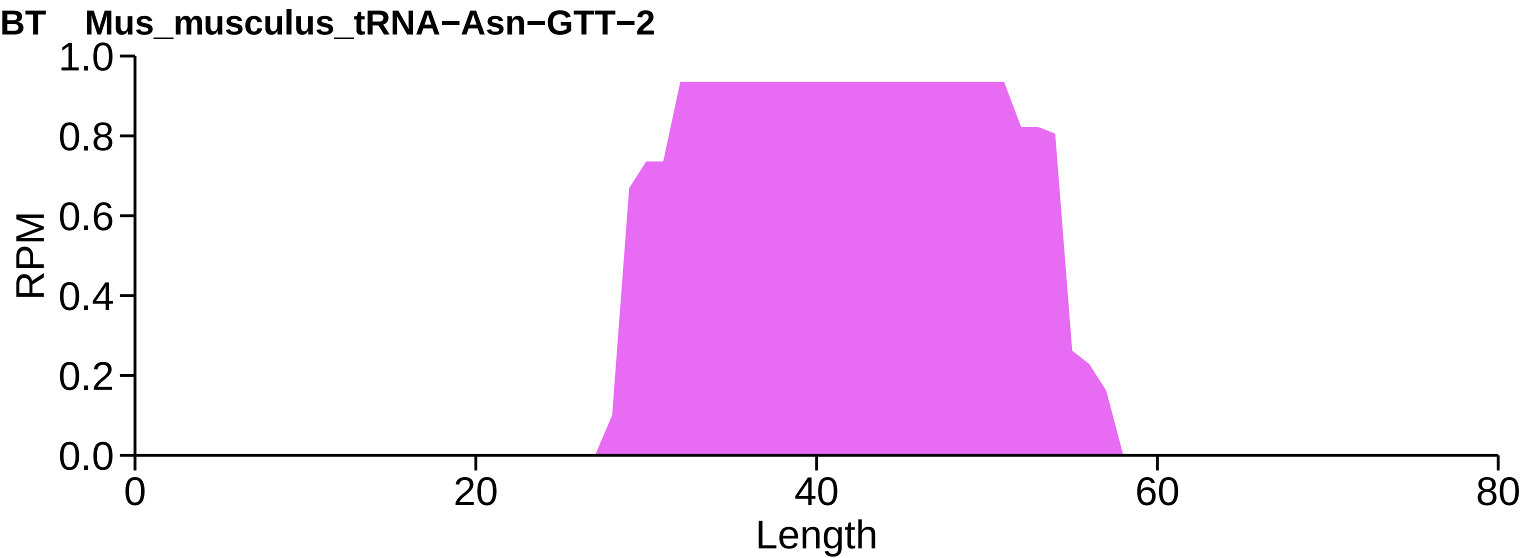
BT Mus_musculus_tRNA−Asn−GTT−2
1.0
0.8
0.6
0.4
0.2
0.0
RPM
0
20
40
60
80
Length

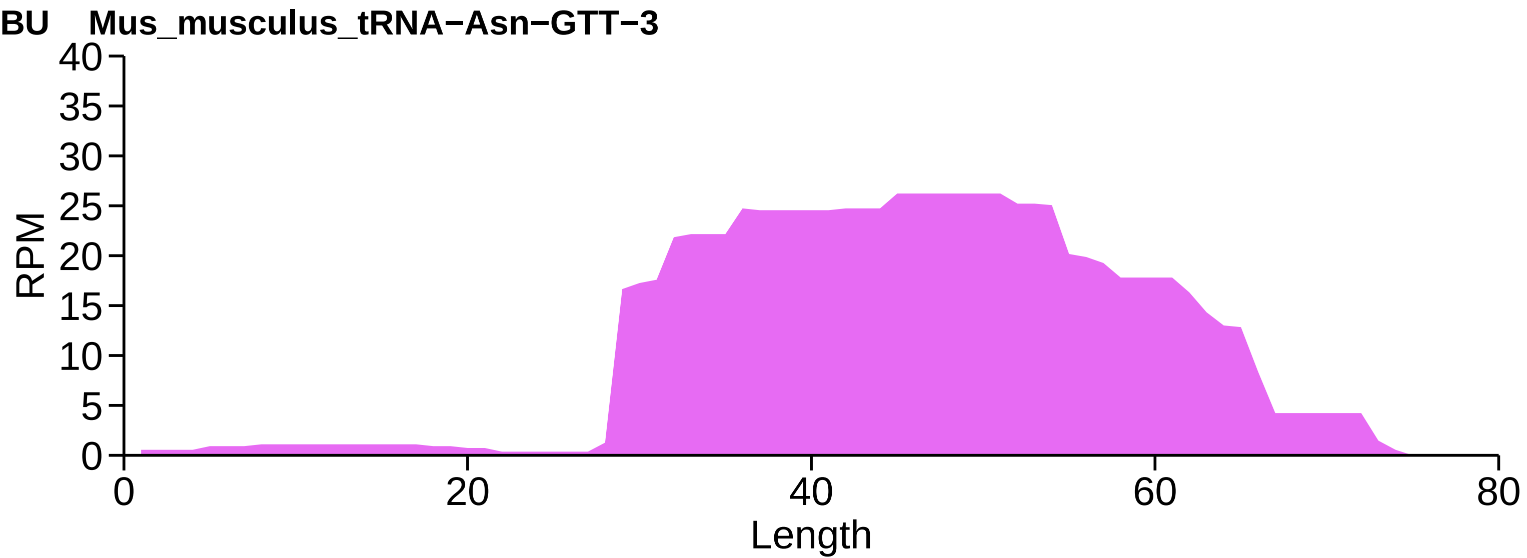

BU Mus_musculus_tRNA−Asn−GTT−3
RPM
0
5
10
15
20
25
30
35
40
0
20
40
60
80
Length

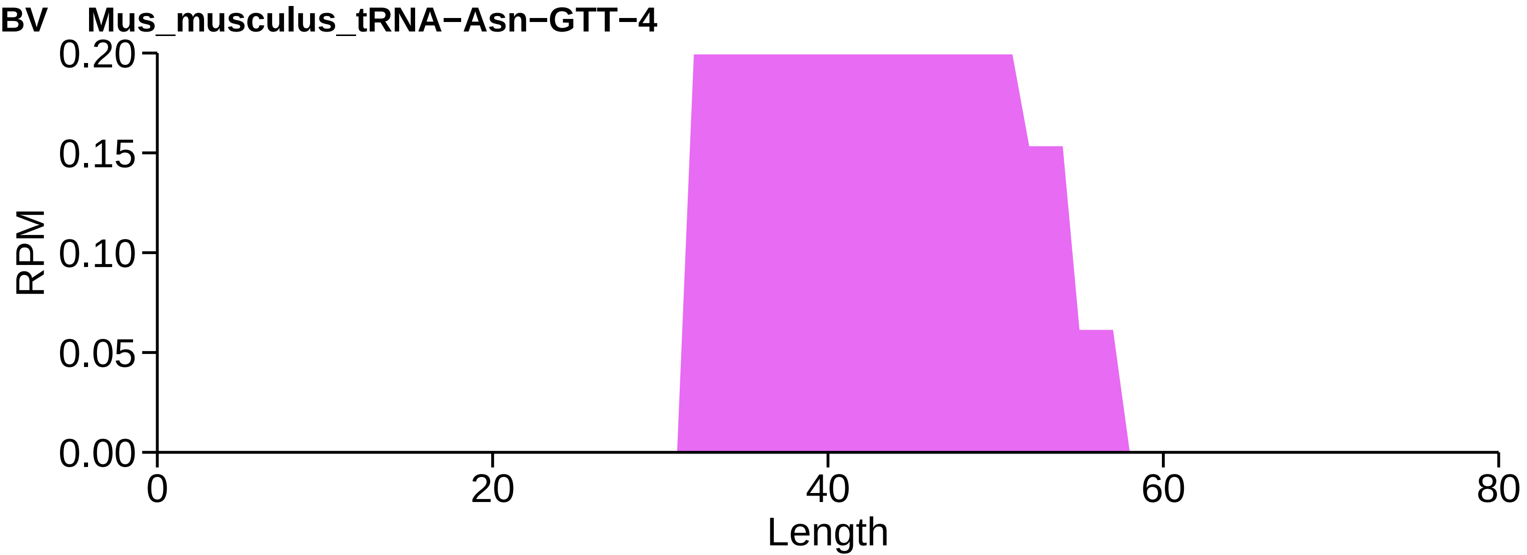

BV Mus_musculus_tRNA−Asn−GTT−4
0.20
0.15
0.10
0.05
0.00
RPM
0
20
40
60
80
Length

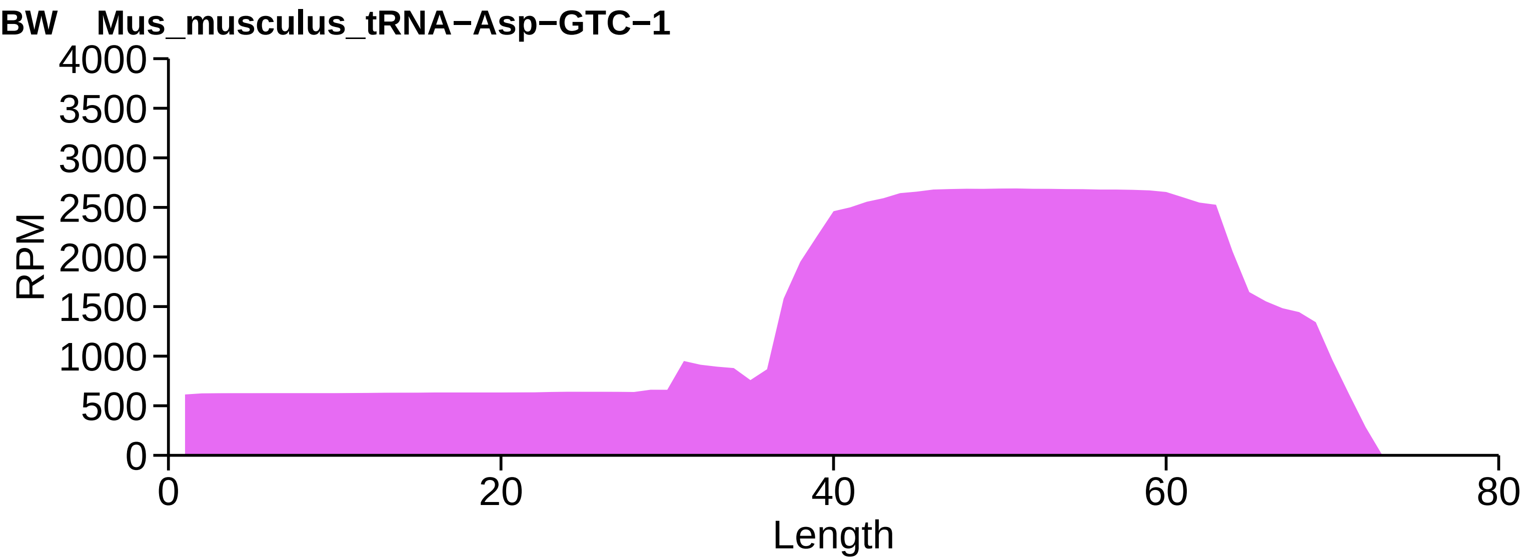
BW Mus_musculus_tRNA−Asp−GTC−1
RPM
4000
3500
3000
2500
2000
1500
1000
500
0
0
20
40
60
80
Length

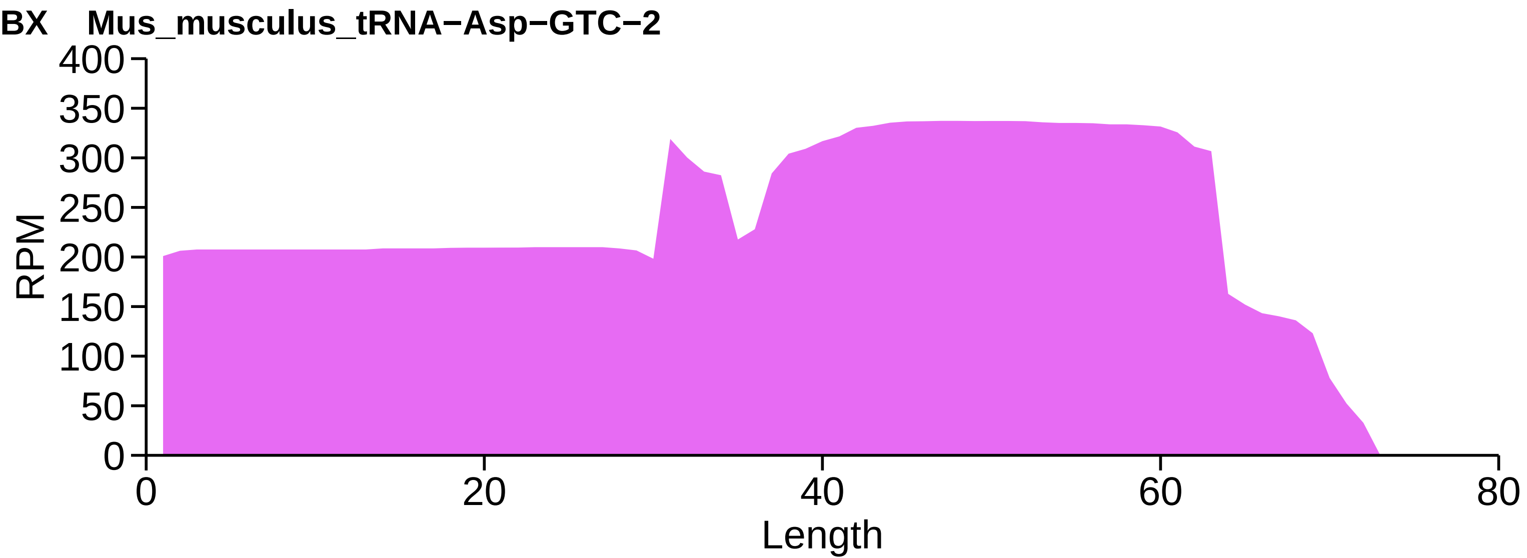
BX Mus_musculus_tRNA−Asp−GTC−2
RPM
400
350
300
250
200
150
100
50
0
0
20
40
60
80
Length

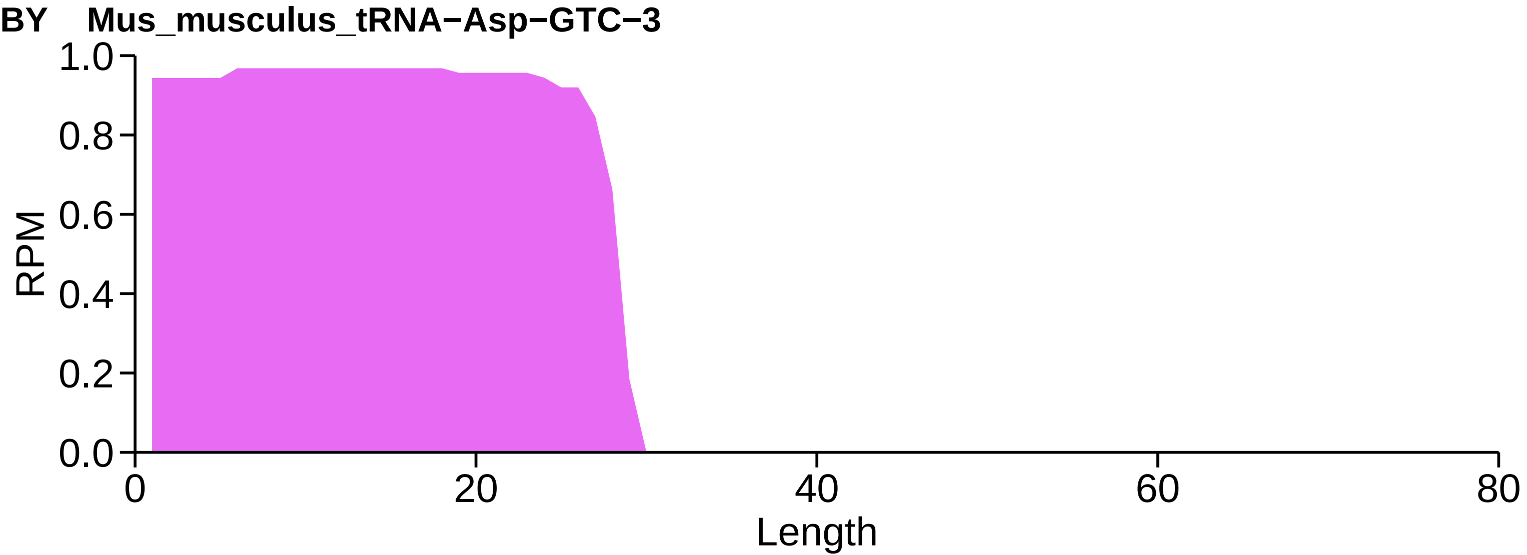

BY Mus_musculus_tRNA−Asp−GTC−3
RPM
Length
1.0
0.8
0.6
0.4
0.2
0.0
0
20
40
60
80

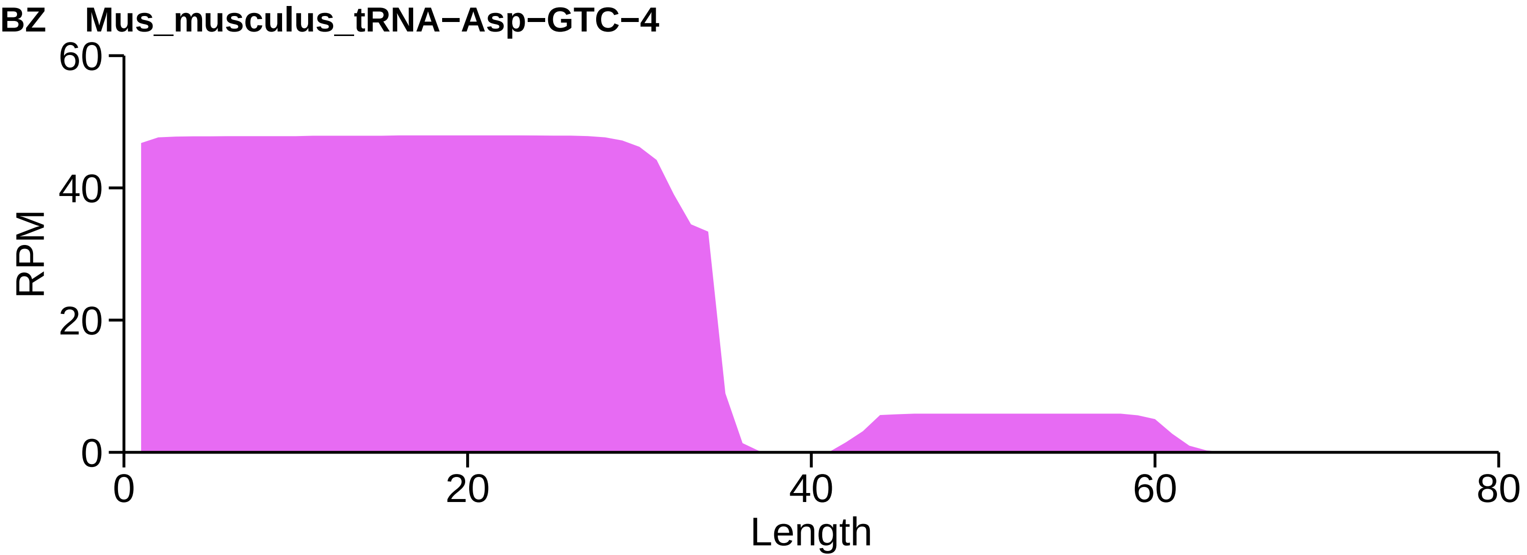
BZ Mus_musculus_tRNA−Asp−GTC−4
60
40
20
0
RPM
0
20
40
60
80
Length

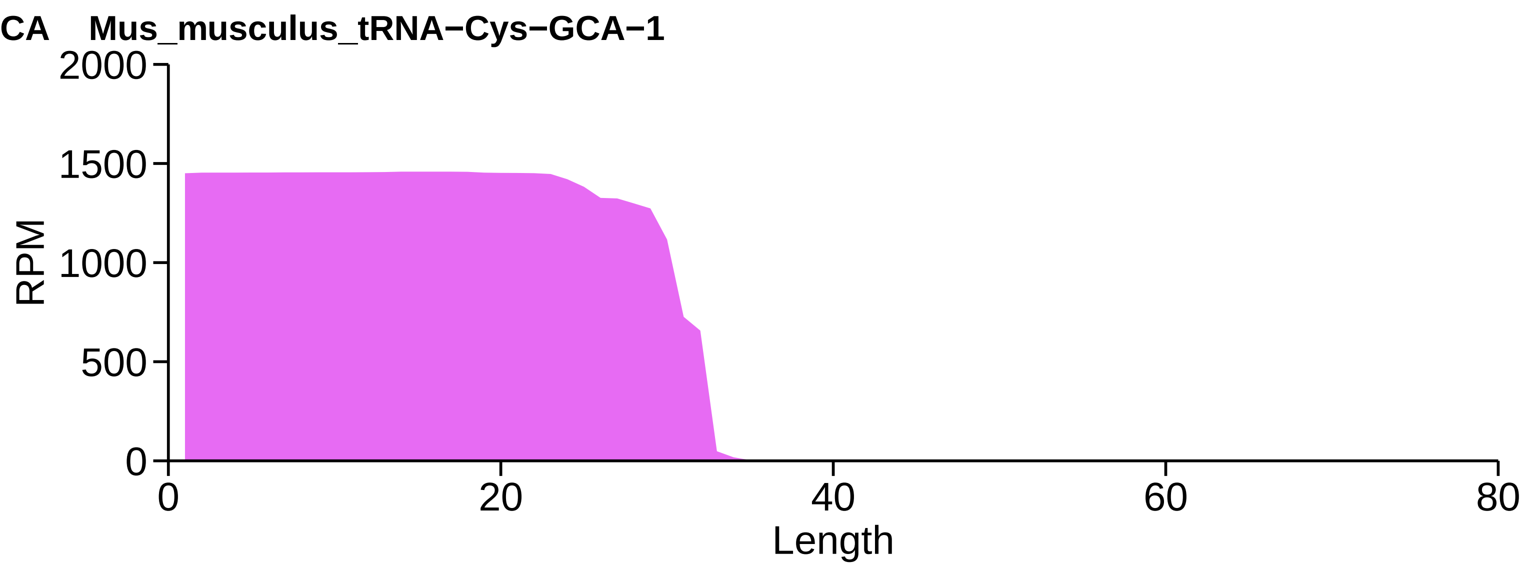
CA Mus_musculus_tRNA−Cys−GCA−1
2000
1500
1000
500
0
RPM
0
20
40
60
80
Length

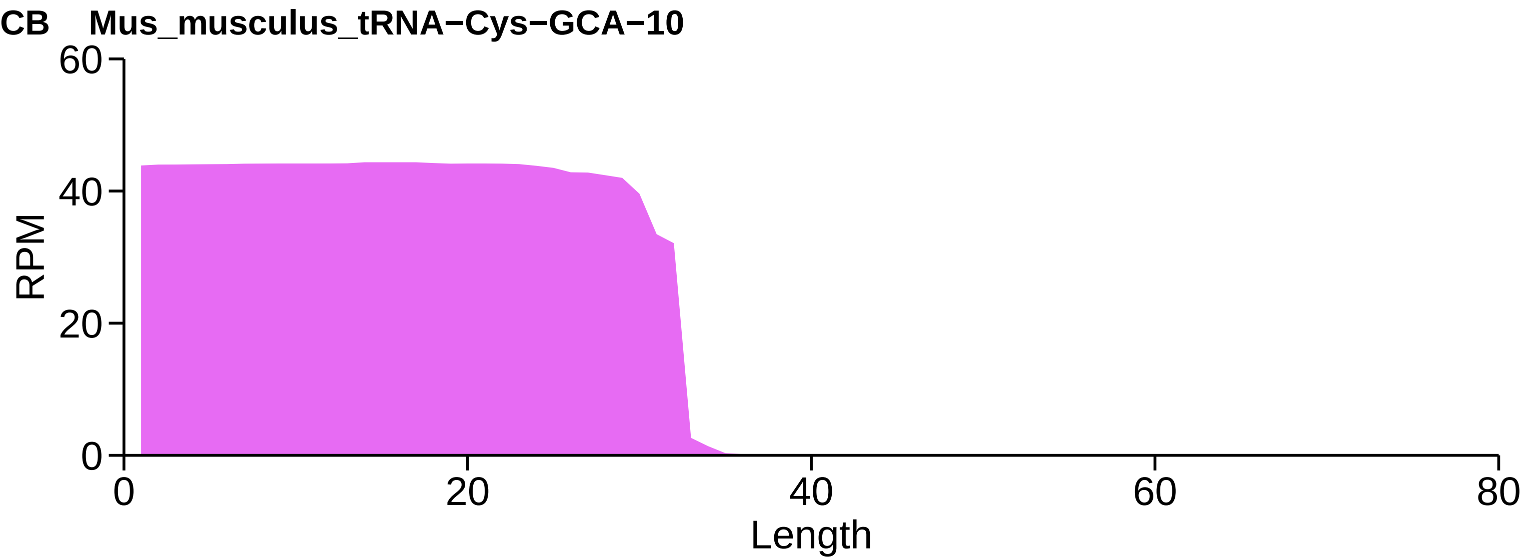

CB Mus_musculus_tRNA−Cys−GCA−10
60
40
20
0
RPM
0
20
40
60
80
Length

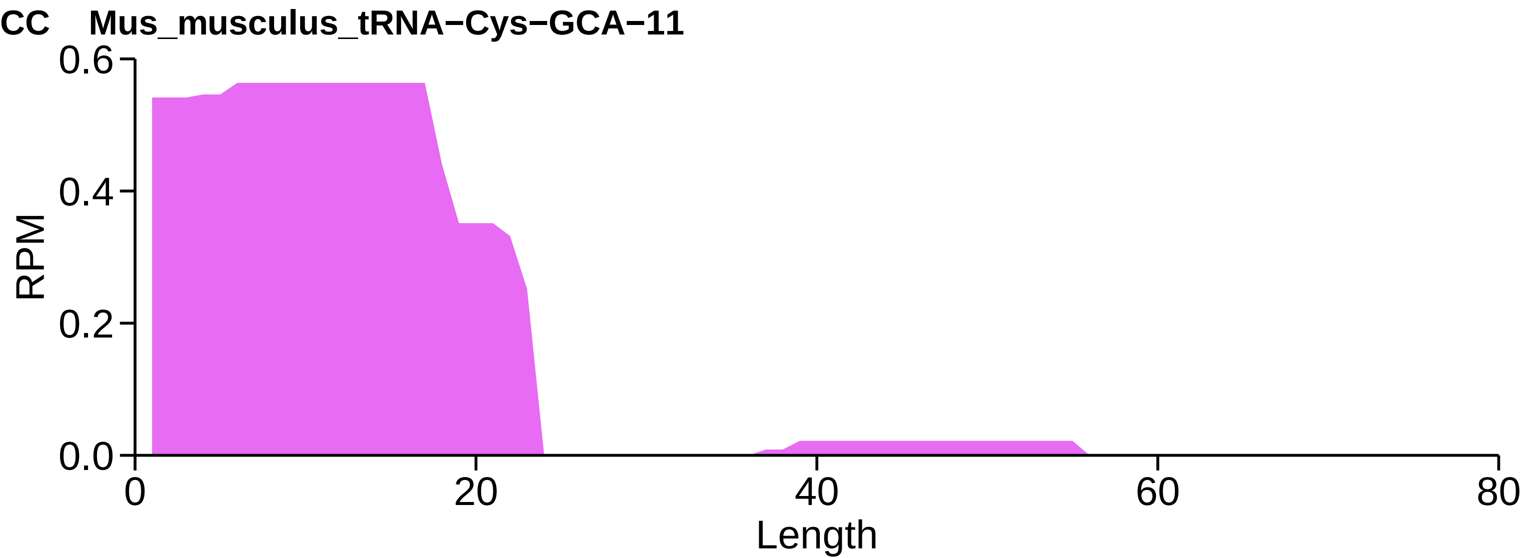
CC Mus_musculus_tRNA−Cys−GCA−11
0.6
0.4
0.2
0.0
RPM
0
20
40
60
80
Length

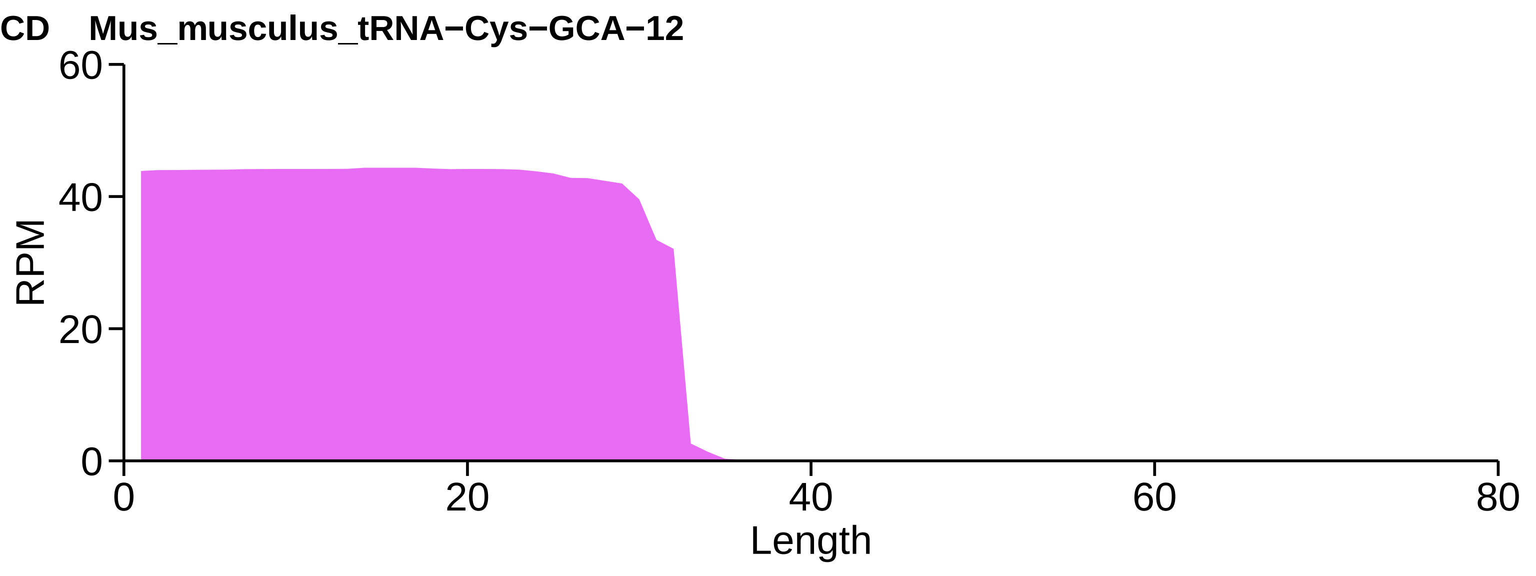
CD Mus_musculus_tRNA−Cys−GCA−12
RPM
0
20
40
60
0
20
40
60
80
Length

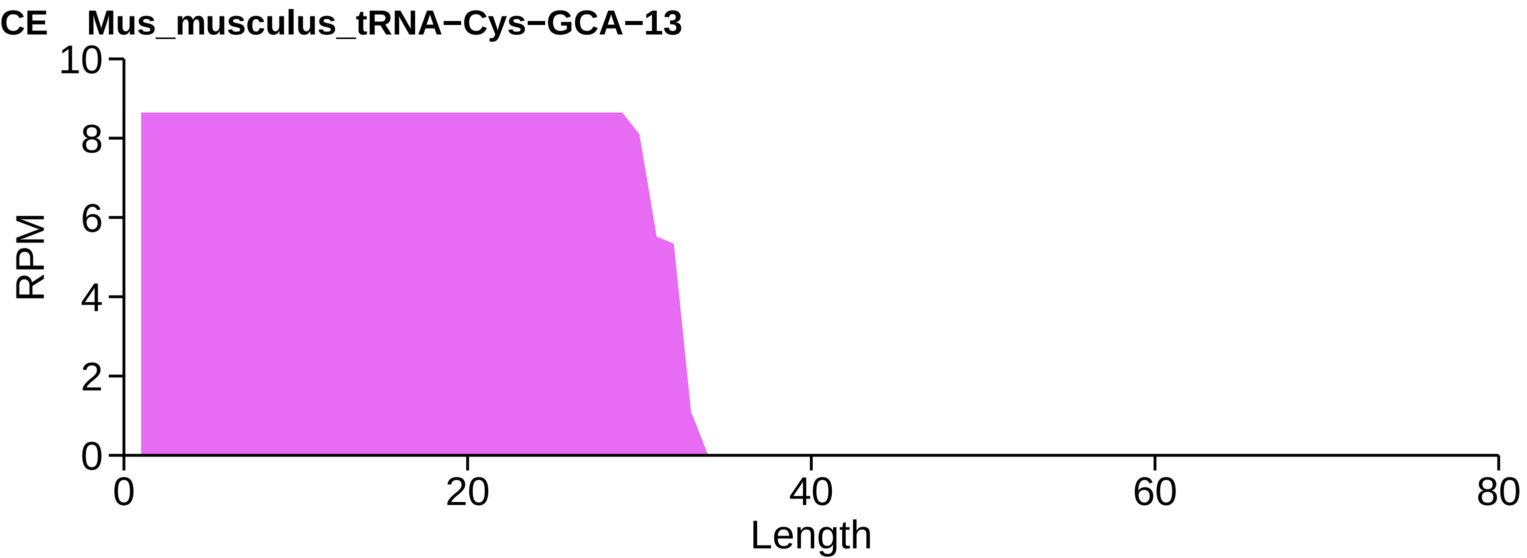
CE Mus_musculus_tRNA−Cys−GCA−13
RPM
Length
0
2
4
6
8
10
0
20
40
60
80

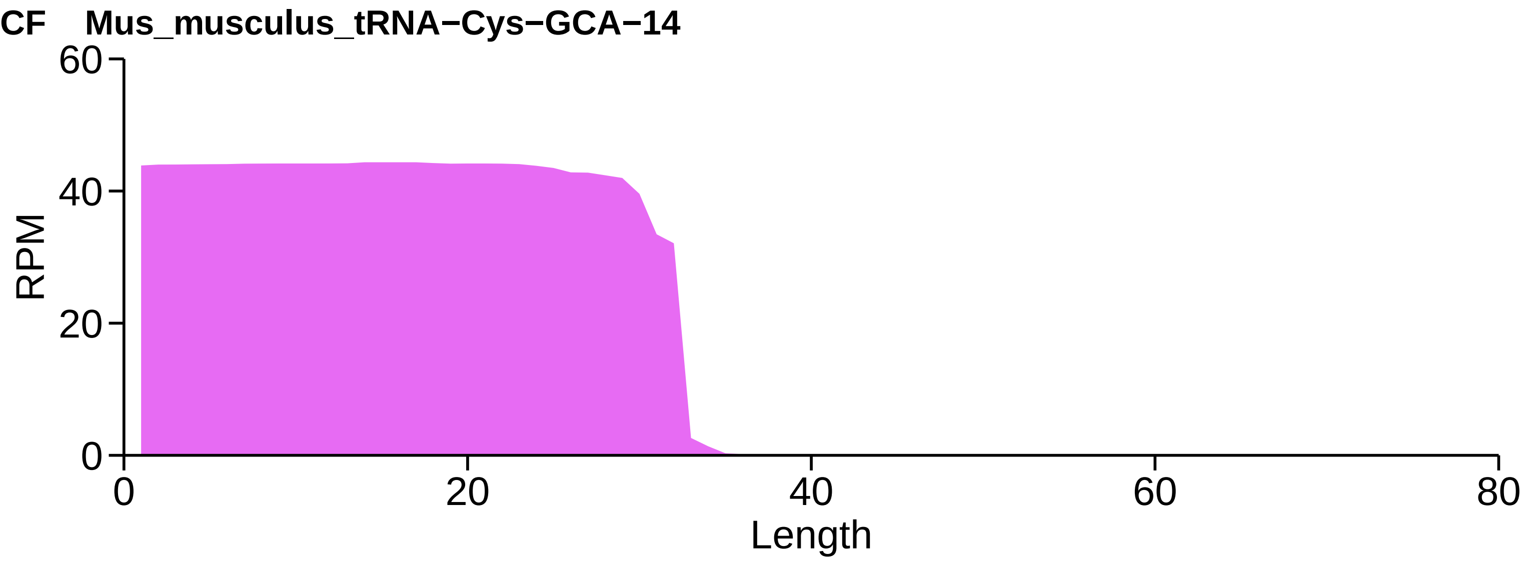
CF Mus_musculus_tRNA−Cys−GCA−14
RPM
Length
0
20
40
60
0
20
40
60
80

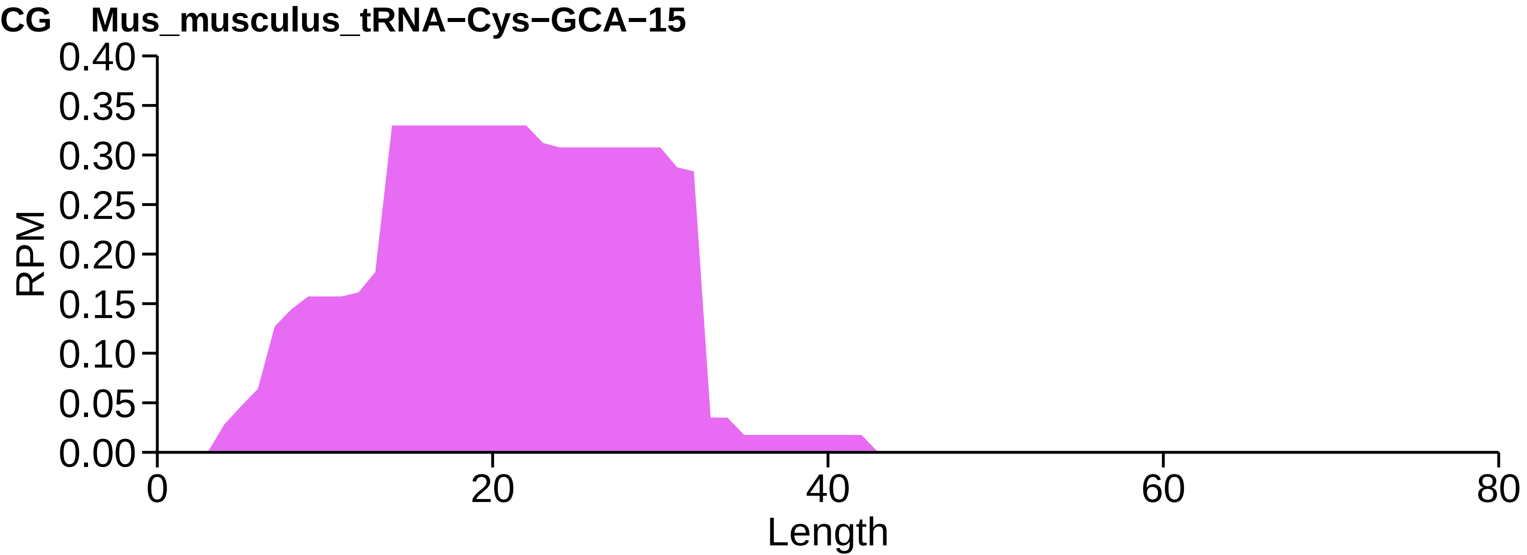
CG Mus_musculus_tRNA−Cys−GCA−15
RPM
0.40
0.35
0.30
0.25
0.20
0.15
0.10
0.05
0.00
0
20
40
60
80
Length

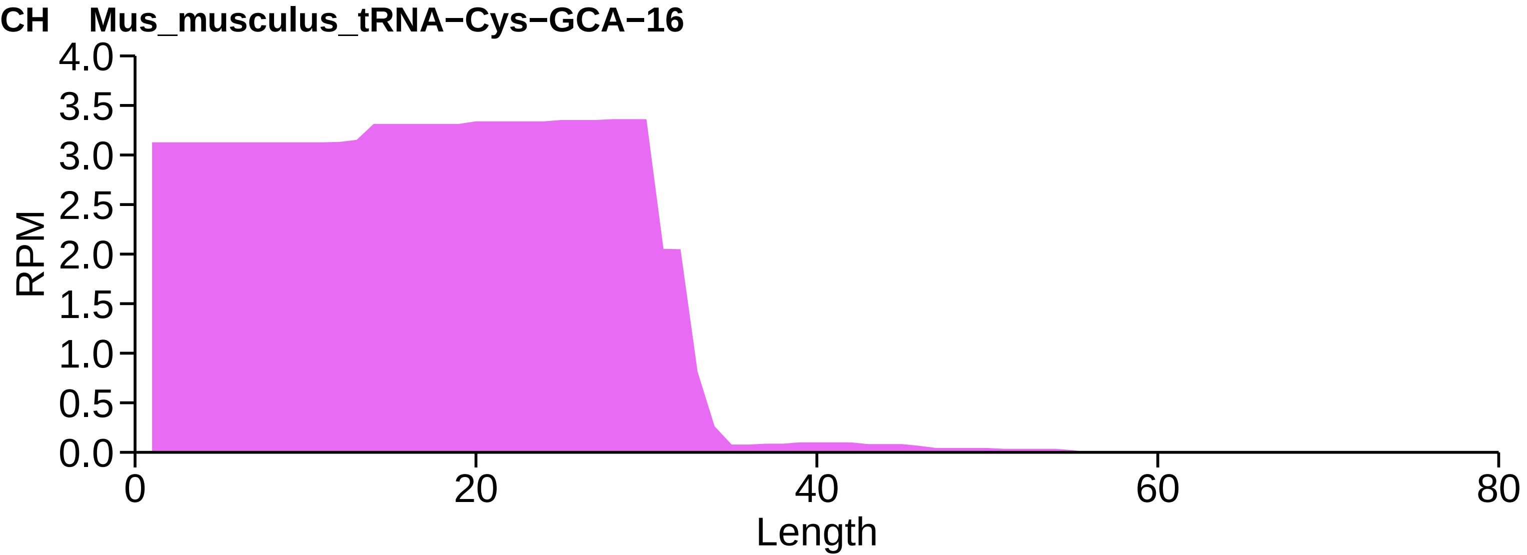

CH Mus_musculus_tRNA−Cys−GCA−16
RPM
4.0
3.5
3.0
2.5
2.0
1.5
1.0
0.5
0.0
0
20
40
60
80
Length

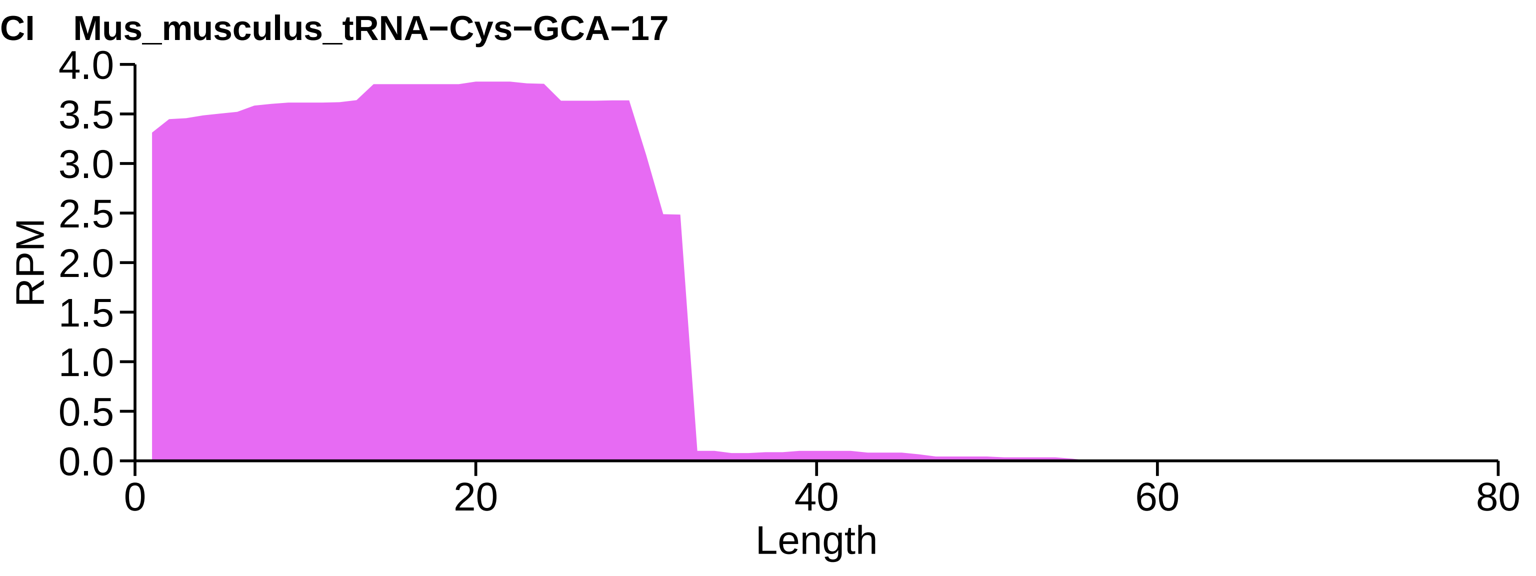

CI Mus_musculus_tRNA−Cys−GCA−17
RPM
4.0
3.5
3.0
2.5
2.0
1.5
1.0
0.5
0.0
0
20
40
60
80
Length

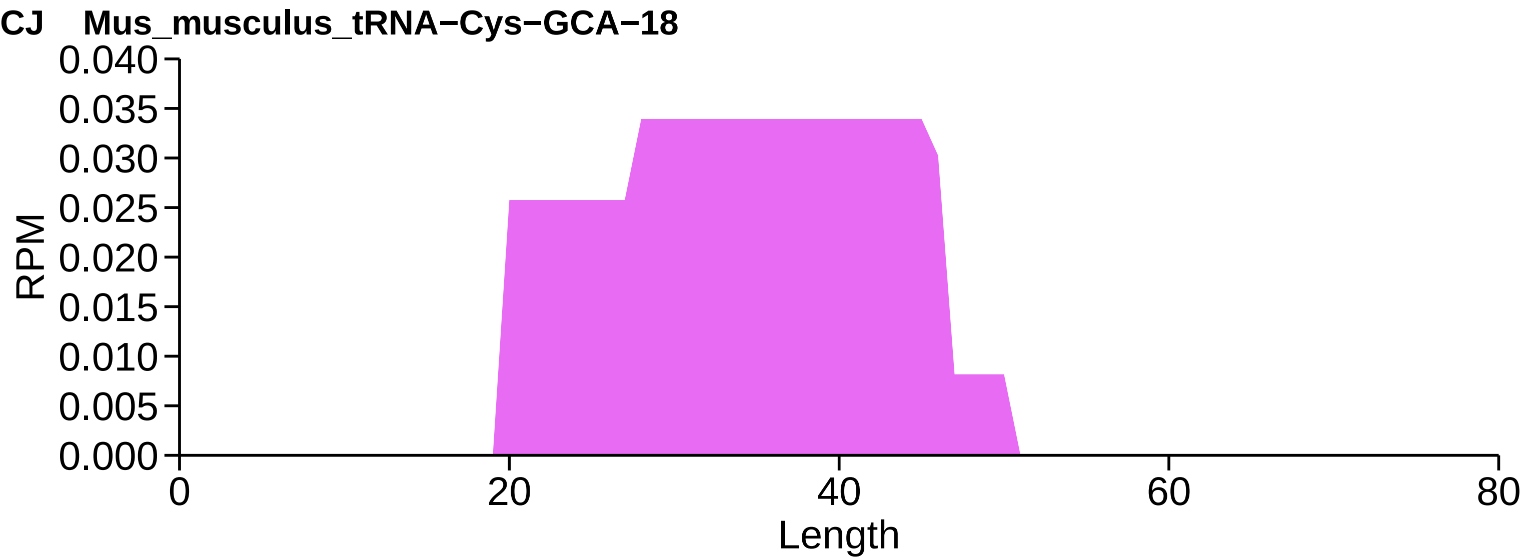

CJ Mus_musculus_tRNA−Cys−GCA−18
0.040
0.035
0.030
0.025
0.020
0.015
0.010
0.005
0.000
RPM
0
20
40
60
80
Length

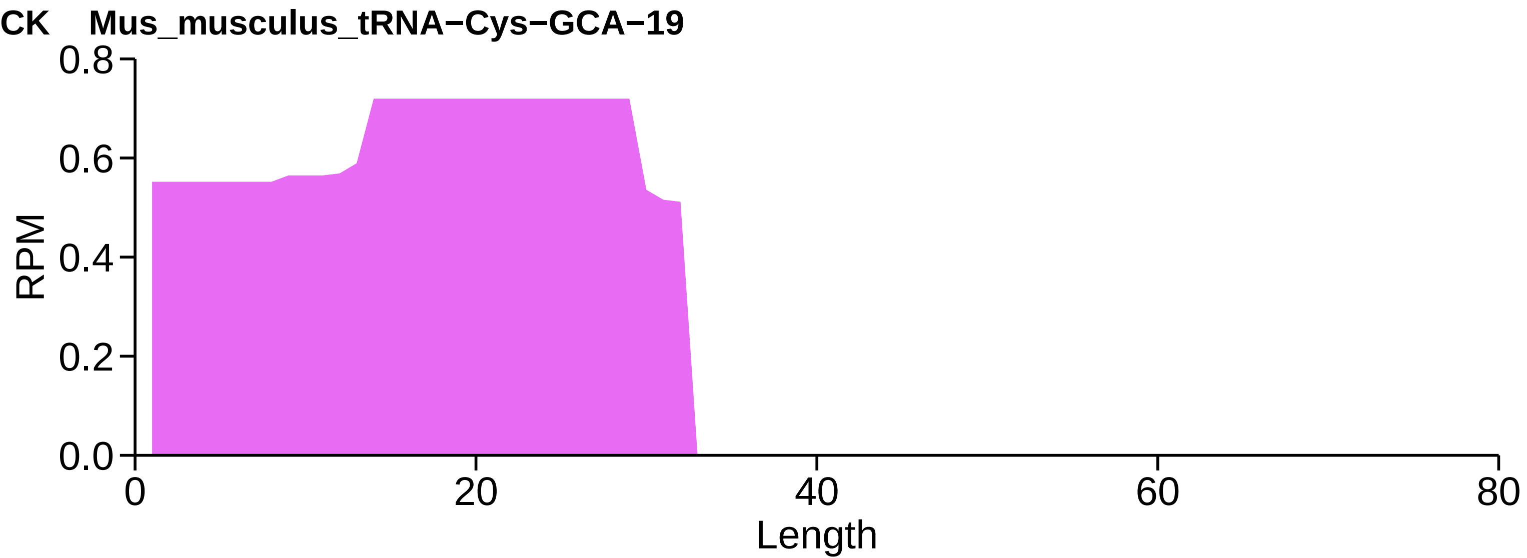

CK Mus_musculus_tRNA−Cys−GCA−19
0.8
0.6
0.4
0.2
0.0
RPM
0
20
40
60
80
Length

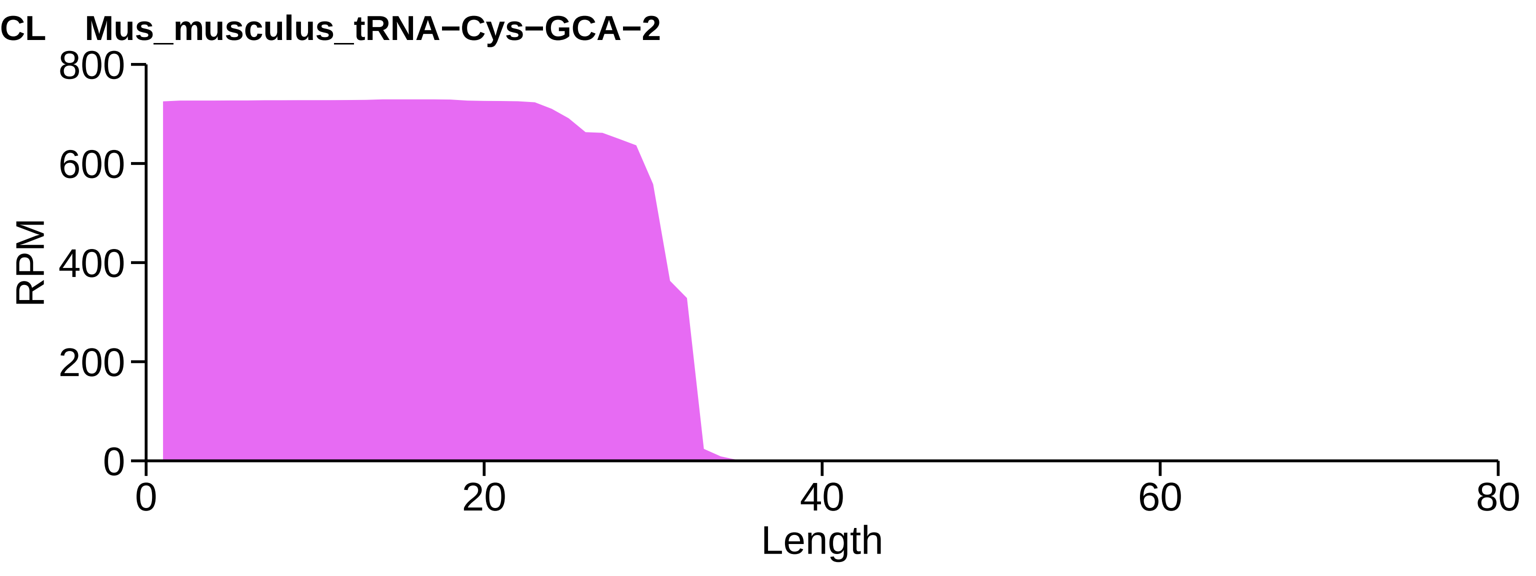
CL Mus_musculus_tRNA−Cys−GCA−2
RPM
800
600
400
200
0
0
20
40
60
80
Length

CM Mus_musculus_tRNA−Cys−GCA−20

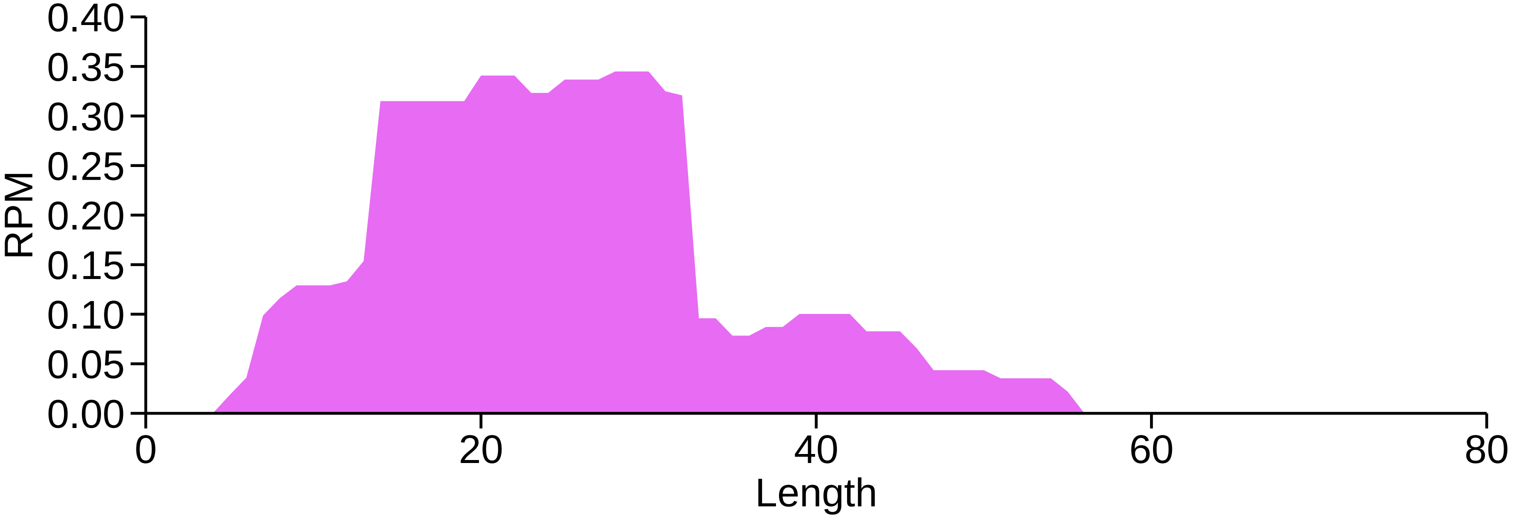

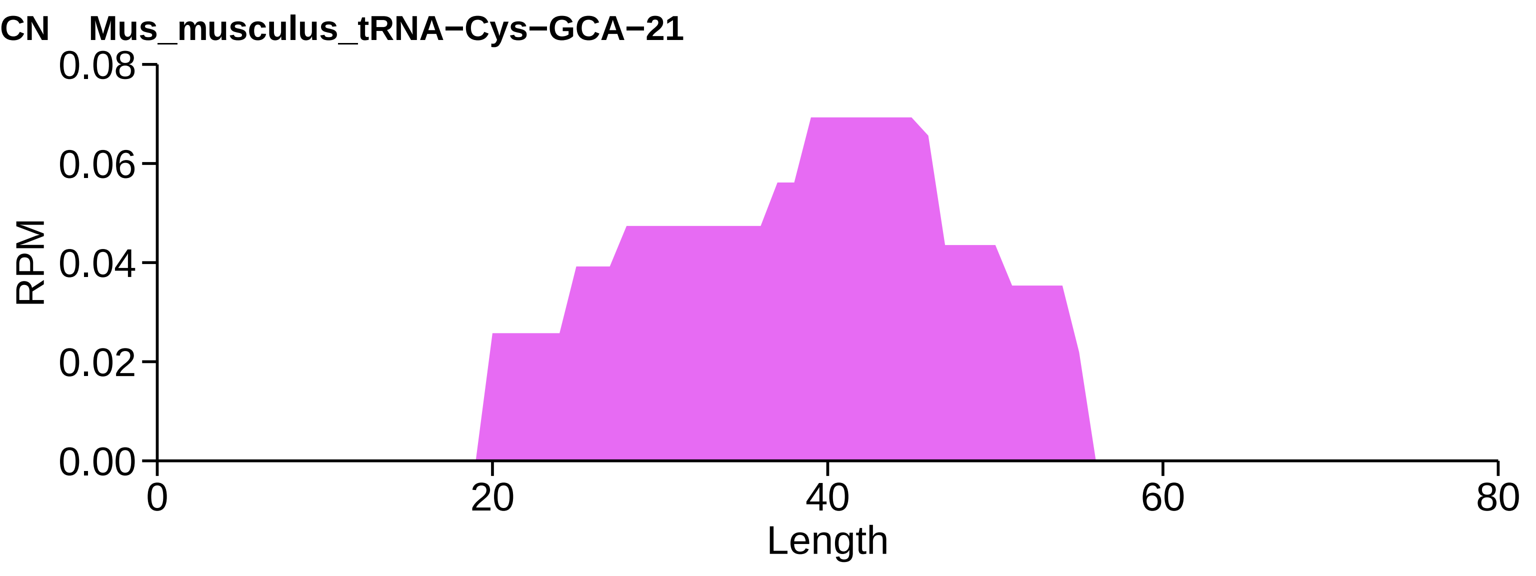
CN Mus_musculus_tRNA−Cys−GCA−21
RPM
0.08
0.06
0.04
0.02
0.00
0
20
40
60
80
Length

CO Mus_musculus_tRNA−Cys−GCA−22

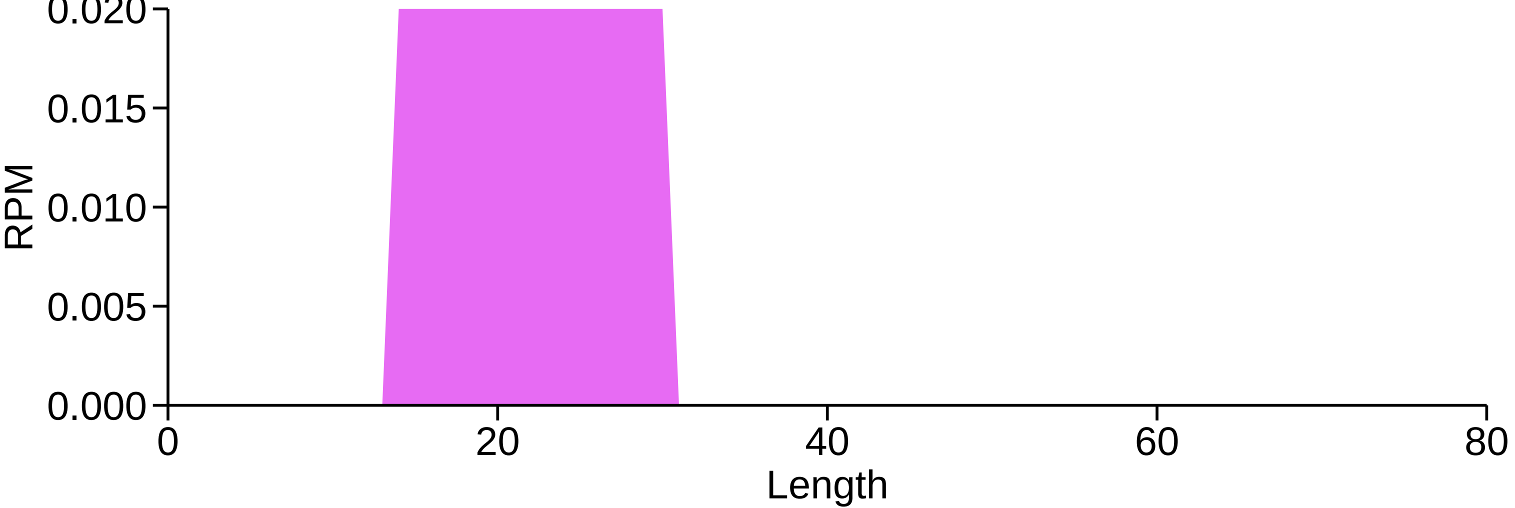

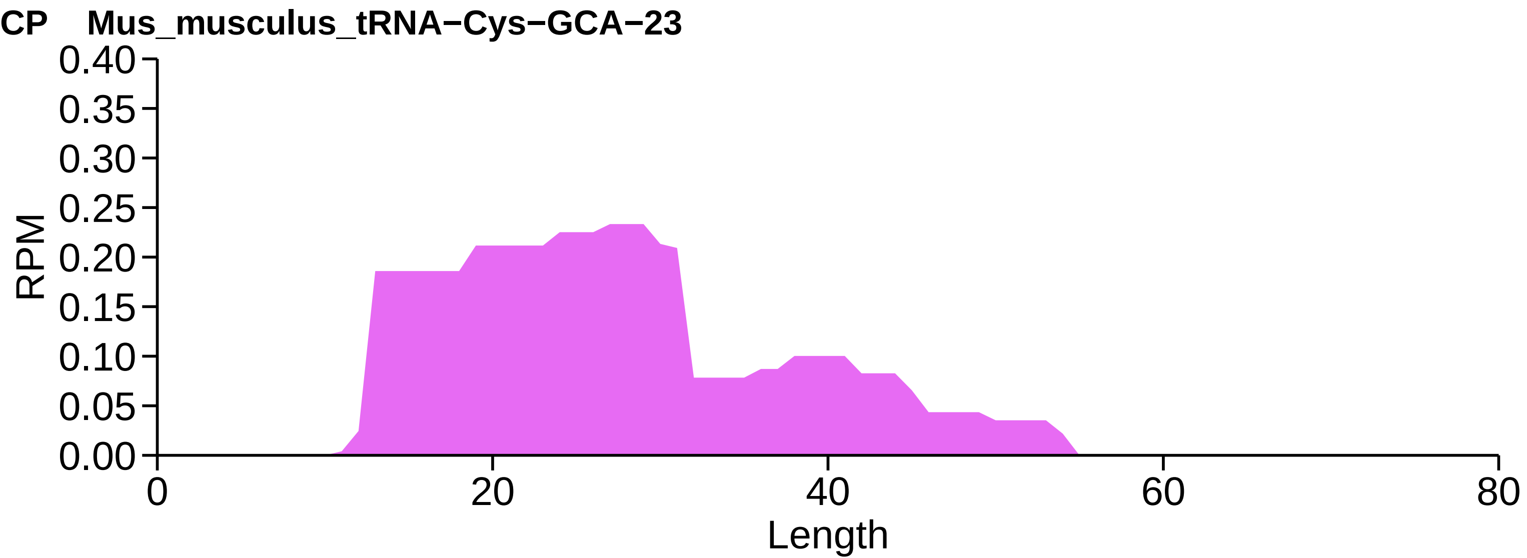
CP Mus_musculus_tRNA−Cys−GCA−23
0.40
0.35
0.30
0.25
0.20
0.15
0.10
0.05
0.00
RPM
0
20
40
60
80
Length

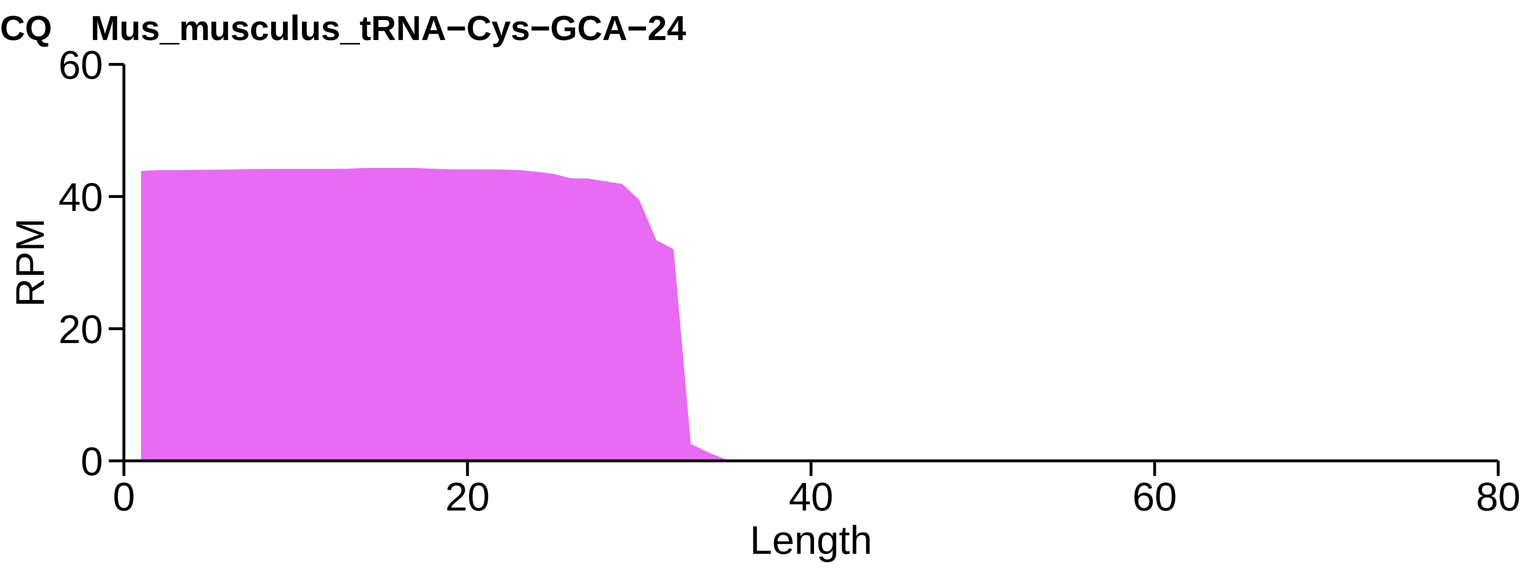
CQ Mus_musculus_tRNA−Cys−GCA−24
60
40
20
0
RPM
0
20
40
60
80
Length

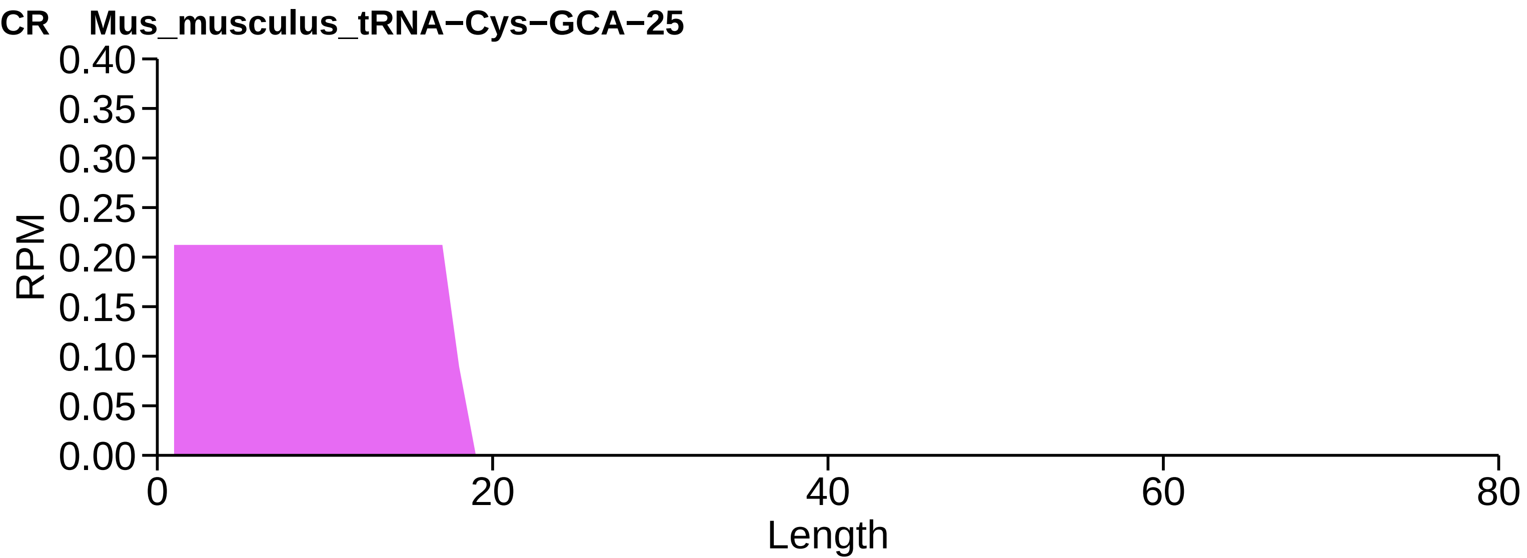
CR Mus_musculus_tRNA−Cys−GCA−25
RPM
0.40
0.35
0.30
0.25
0.20
0.15
0.10
0.05
0.00
0
20
40
60
80
Length

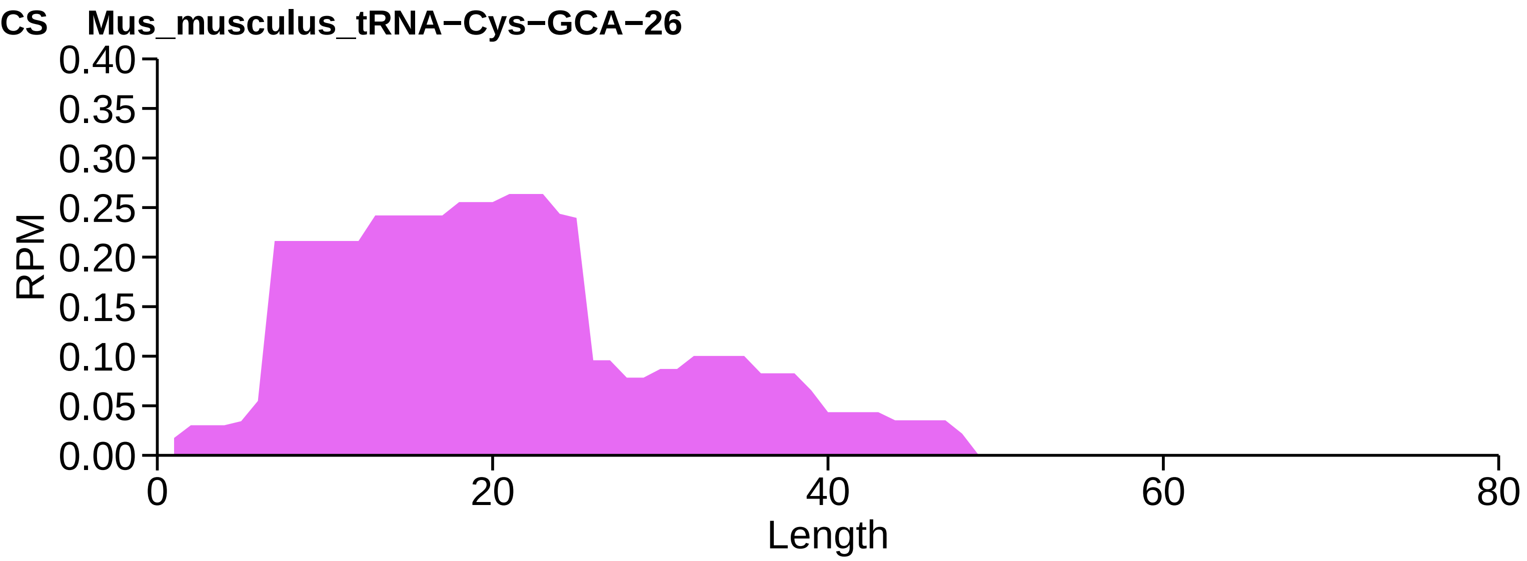
CS Mus_musculus_tRNA−Cys−GCA−26
RPM
0.00
0.05
0.10
0.15
0.20
0.25
0.30
0.35
0.40
0
20
40
60
80
Length

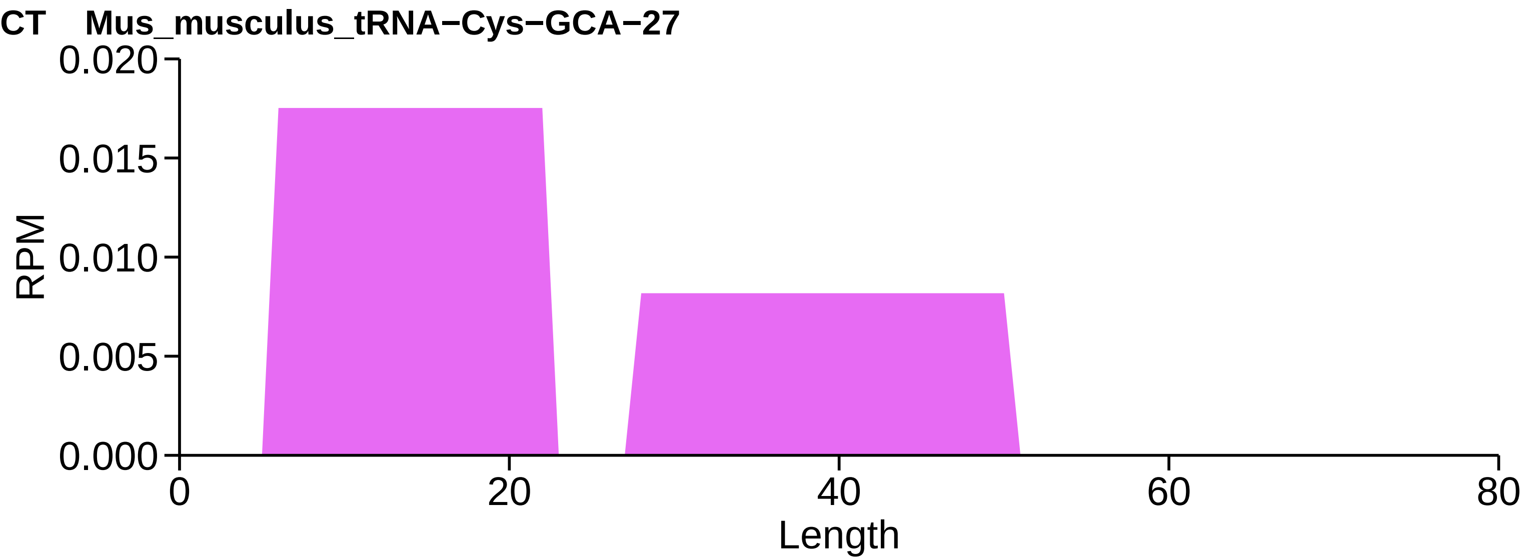
CT Mus_musculus_tRNA−Cys−GCA−27
0.020
0.015
0.010
0.005
0.000
RPM
0
20
40
60
80
Length

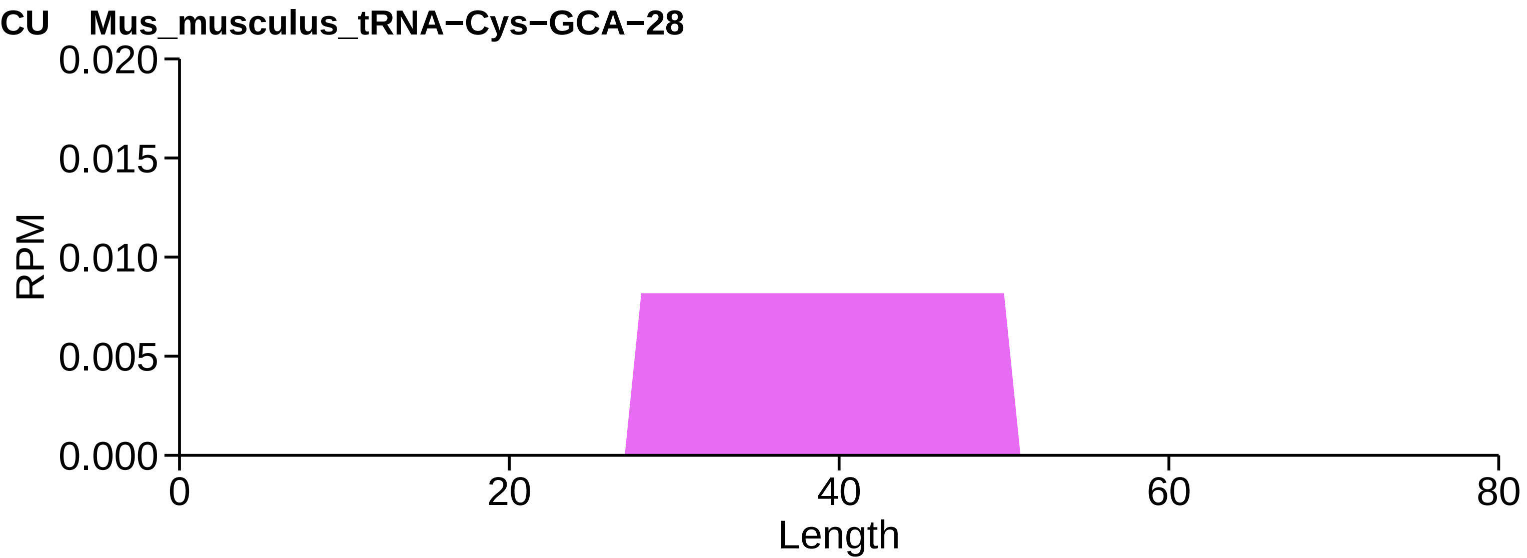
CU Mus_musculus_tRNA−Cys−GCA−28
0.020
0.015
0.010
0.005
0.000
RPM
0
20
40
60
80
Length

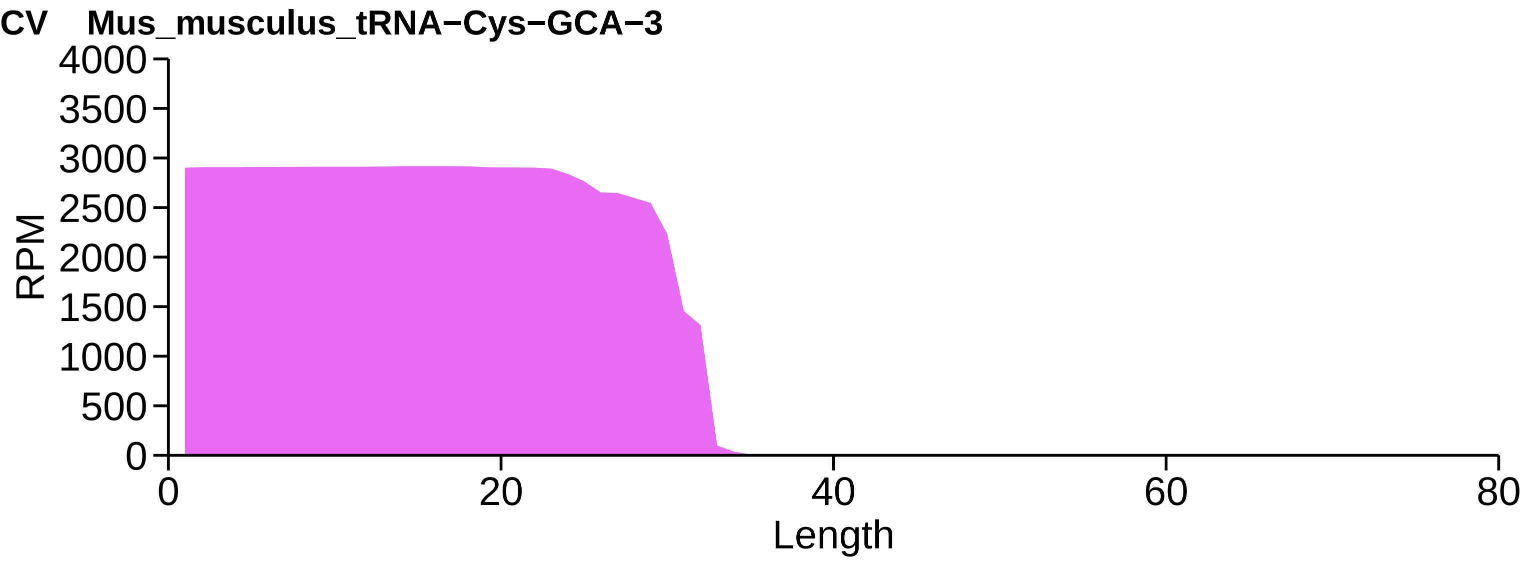
CV Mus_musculus_tRNA−Cys−GCA−3
4000
3500
3000
2500
2000
1500
1000
500
0
RPM
0
20
40
60
80
Length

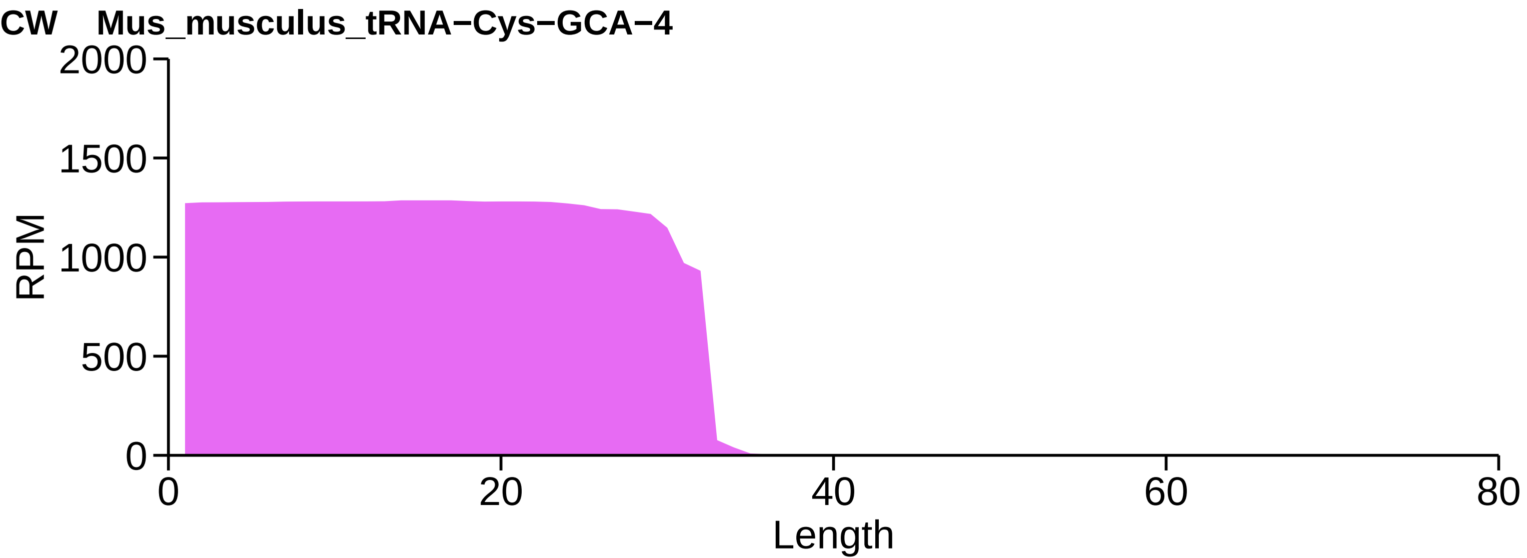

CW Mus_musculus_tRNA−Cys−GCA−4
2000
1500
1000
500
0
RPM
0
20
40
60
80
Length

CX Mus_musculus_tRNA−Cys−GCA−5

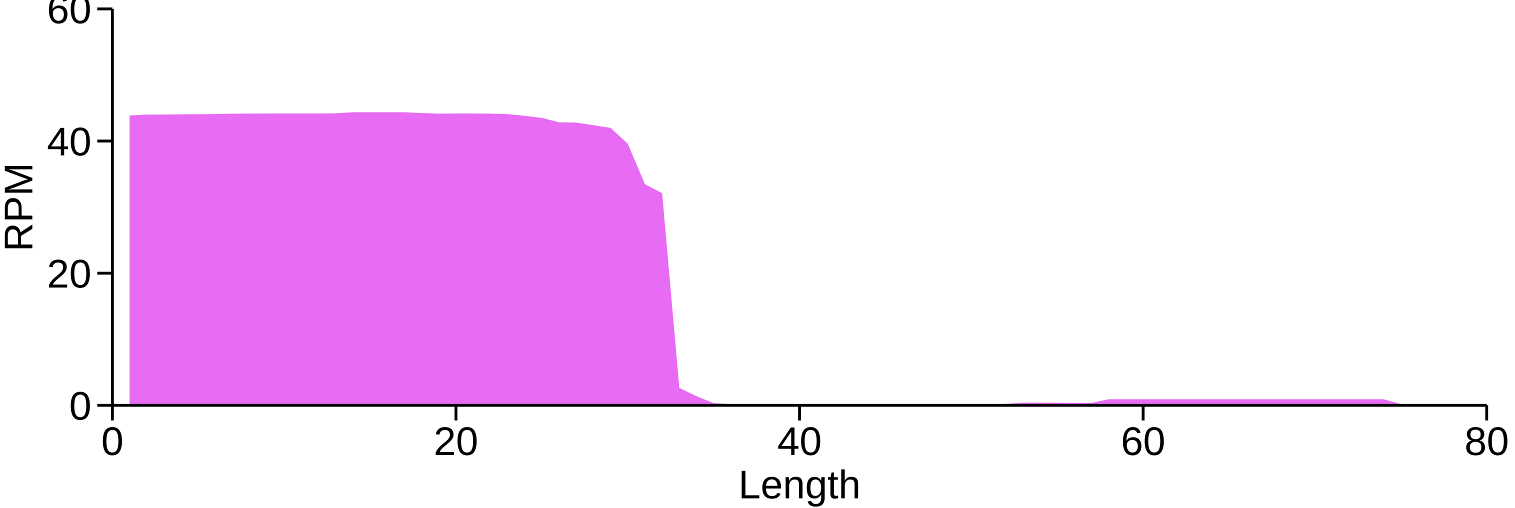

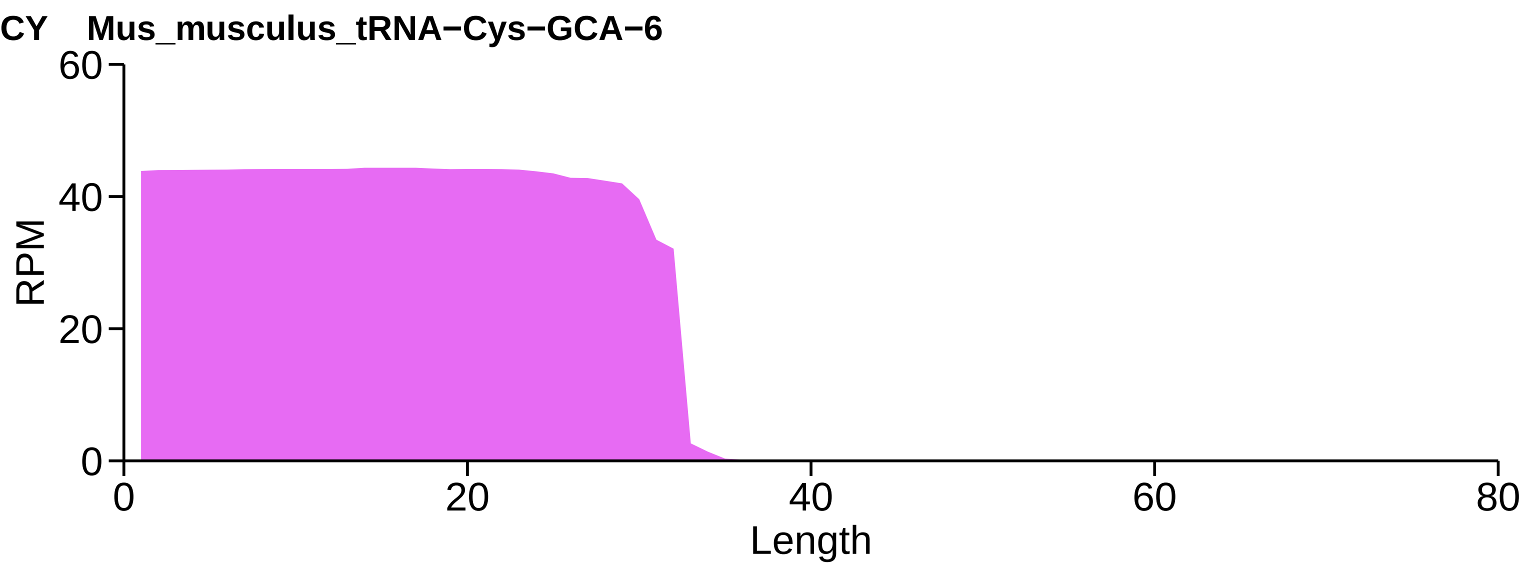
CY Mus_musculus_tRNA−Cys−GCA−6
RPM
Length
0
20
40
60
80

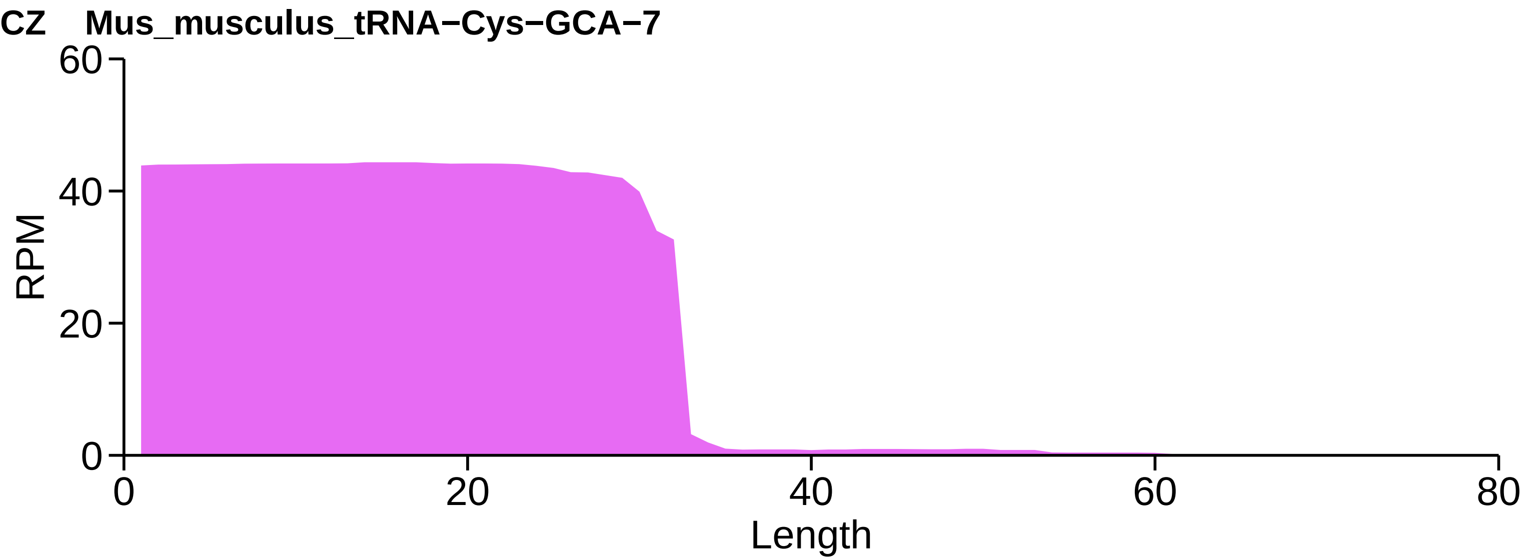

CZ Mus_musculus_tRNA−Cys−GCA−7
RPM
Length
0
20
40
60
0
20
40
60
80

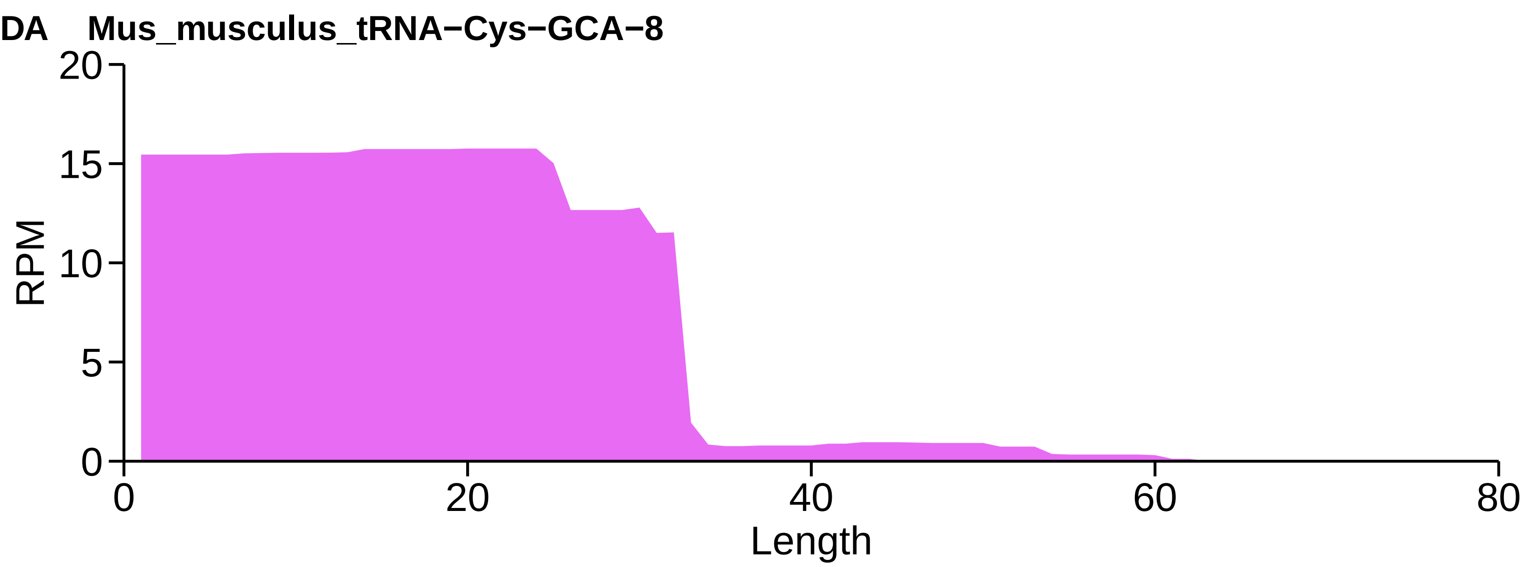
DA Mus_musculus_tRNA−Cys−GCA−8
RPM
20
15
10
5
0
0
20
40
60
80
Length

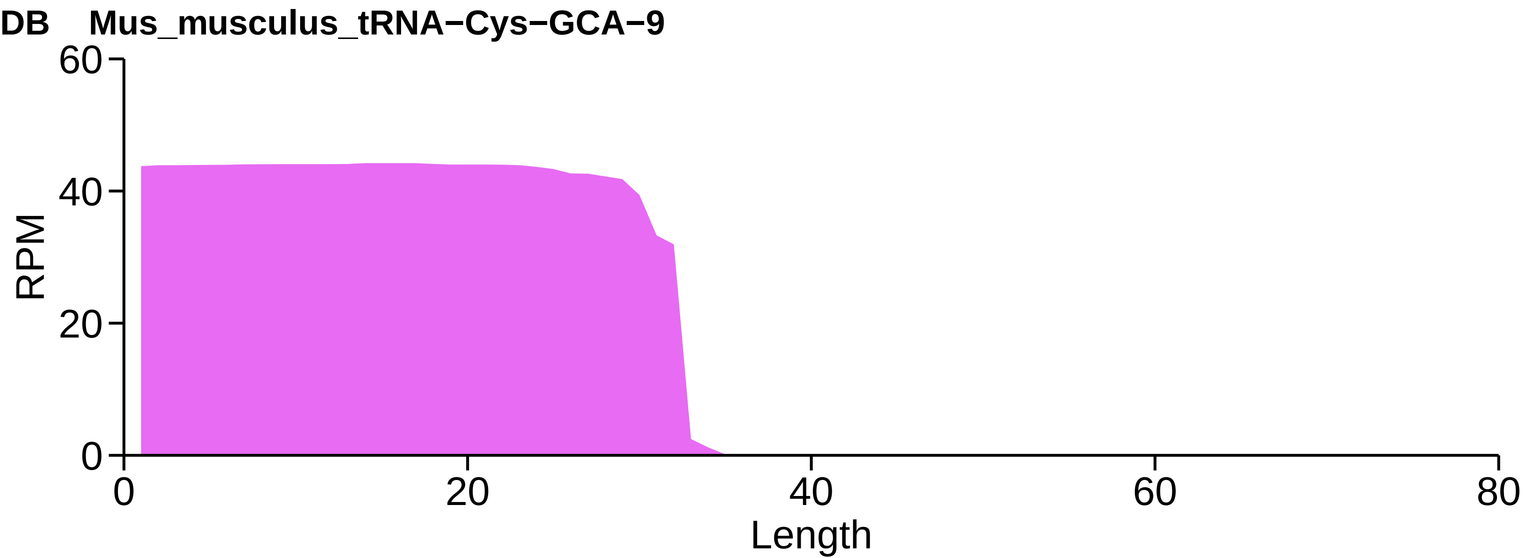
DB Mus_musculus_tRNA−Cys−GCA−9
60
40
20
0
RPM
0
20
40
60
80
Length

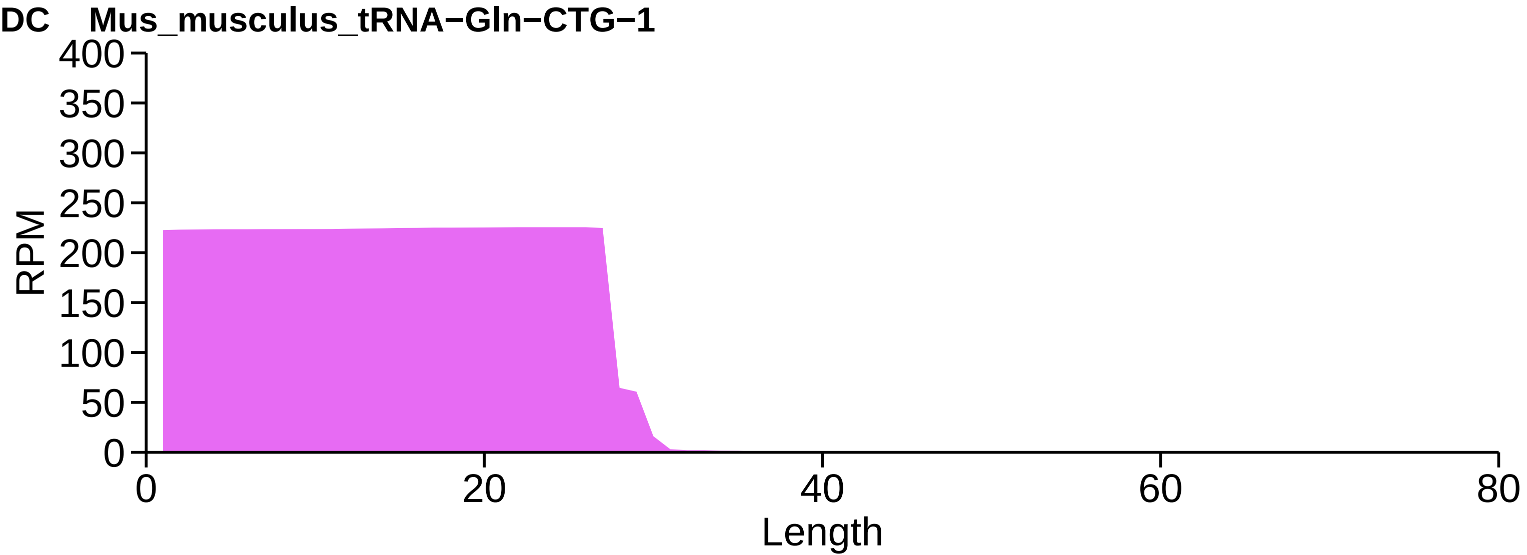
DC Mus_musculus_tRNA−Gln−CTG−1
RPM
400
350
300
250
200
150
100
50
0
0
20
40
60
80
Length

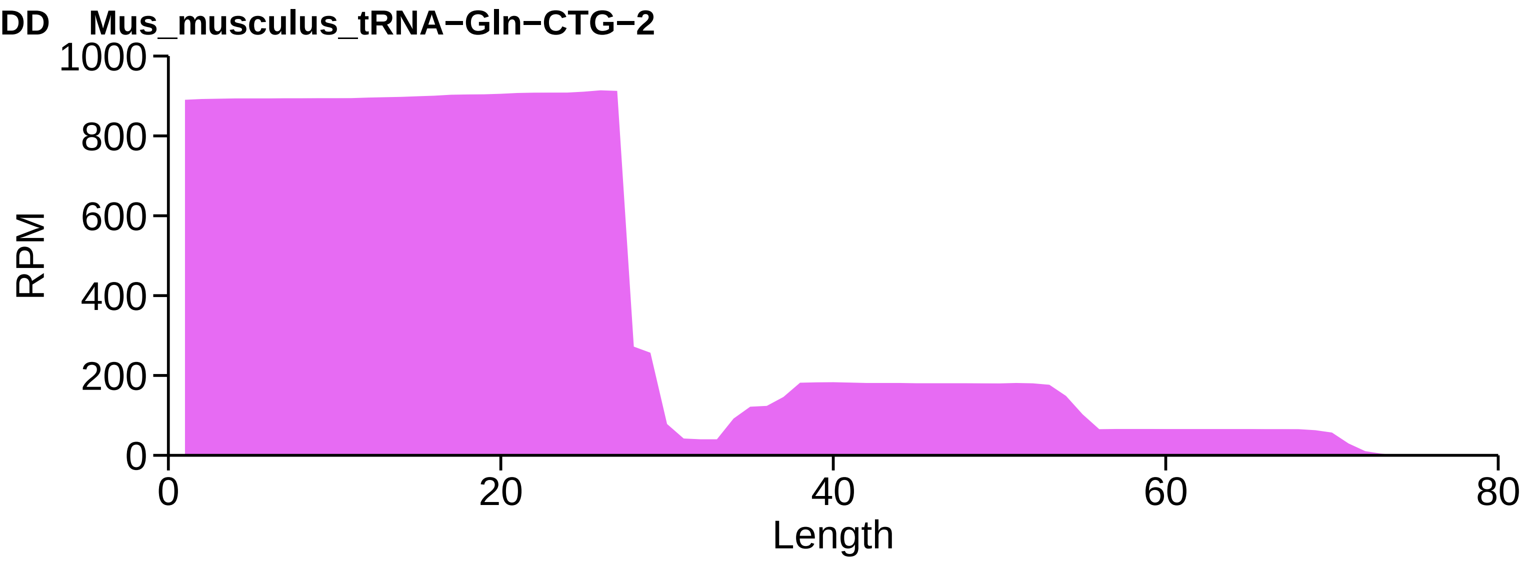
DD Mus_musculus_tRNA−Gln−CTG−2
RPM
1000
800
600
400
200
0
0
20
40
60
80
Length

DE Mus_musculus_tRNA−Gln−CTG−3

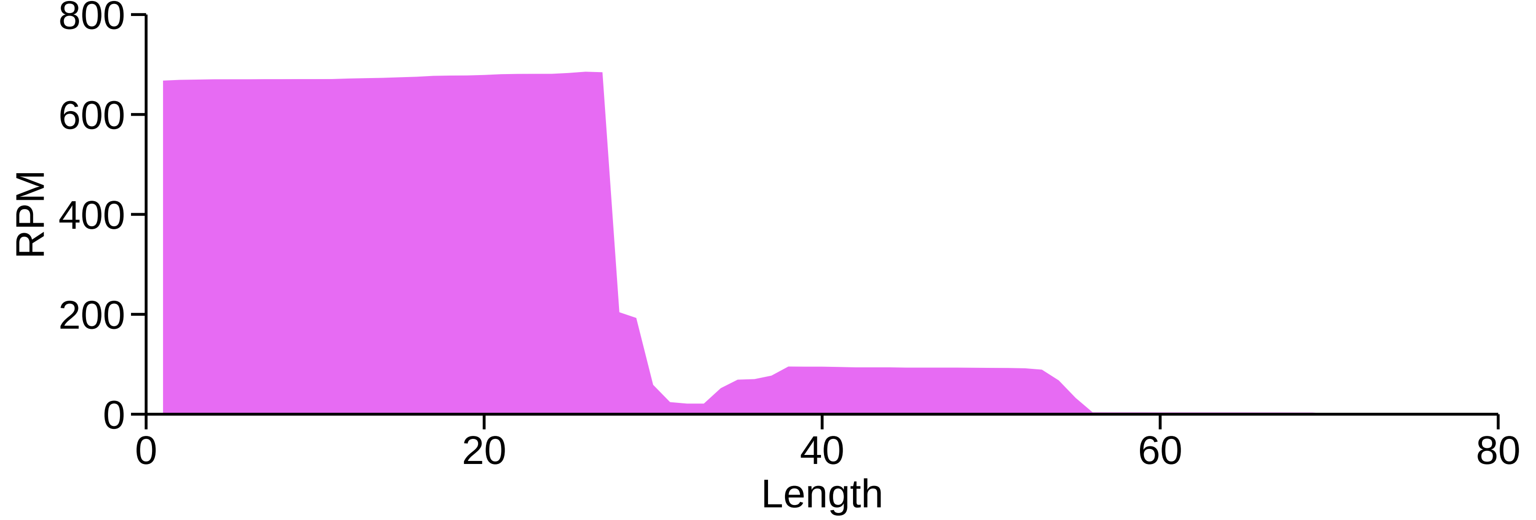

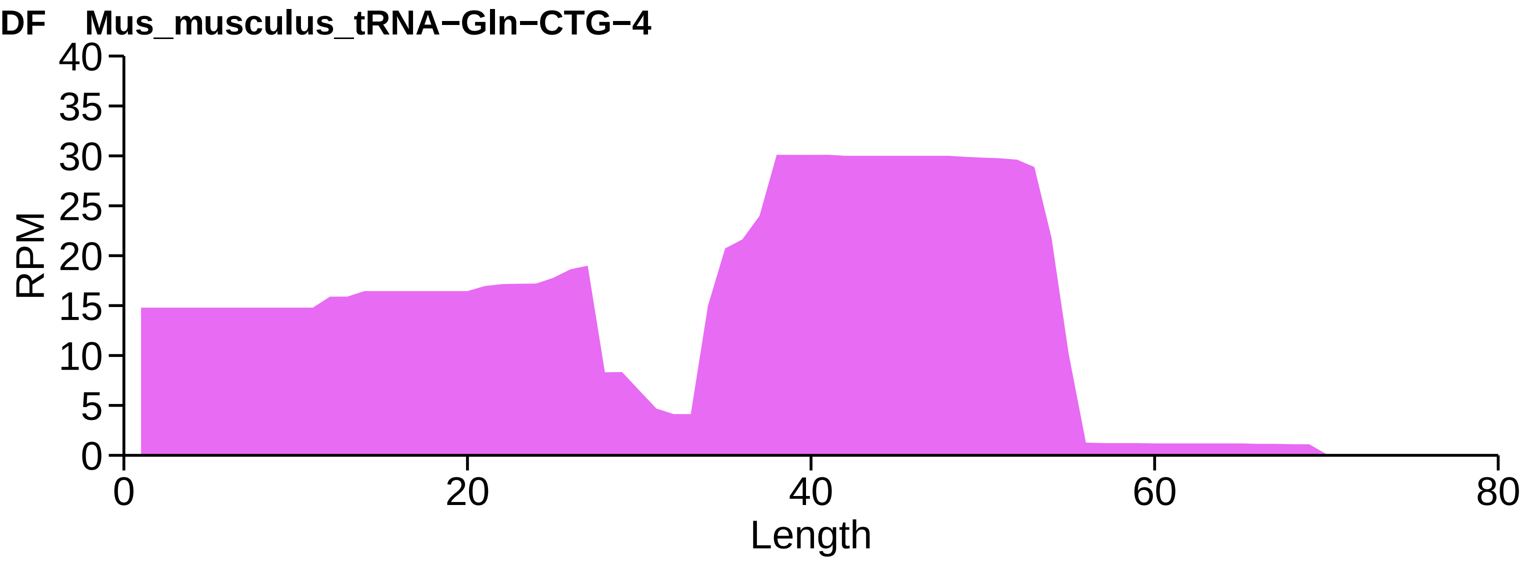
DF Mus_musculus_tRNA−Gln−CTG−4
RPM
0
5
10
15
20
25
30
35
40
0
20
40
60
80
Length

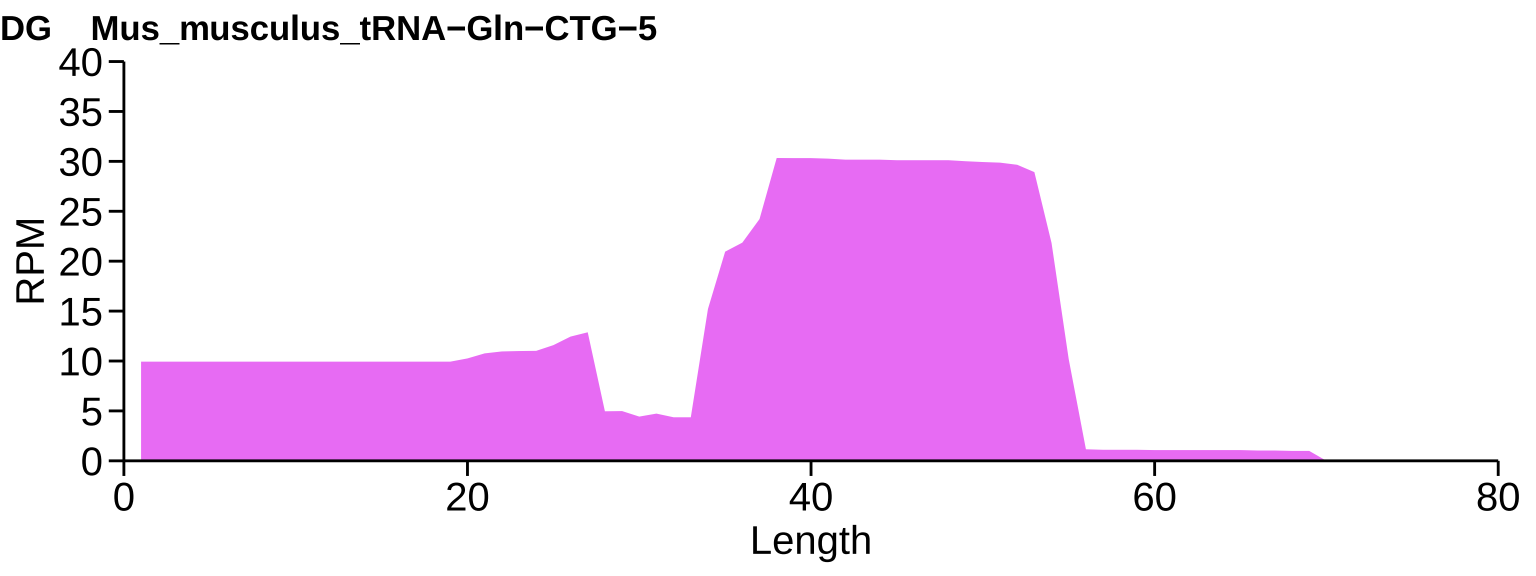
DG Mus_musculus_tRNA−Gln−CTG−5
RPM
Length
0
5
10
15
20
25
30
35
40
0
20
40
60
80

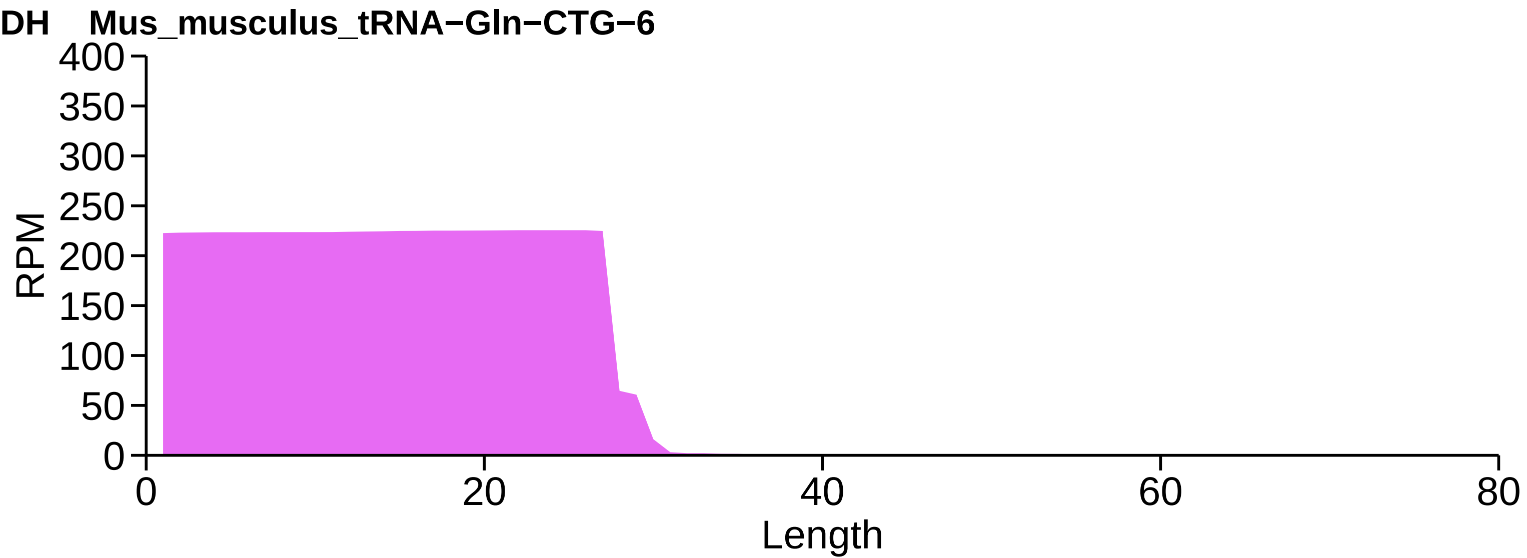
DH Mus_musculus_tRNA−Gln−CTG−6
RPM
400
350
300
250
200
150
100
50
0
0
20
40
60
80
Length

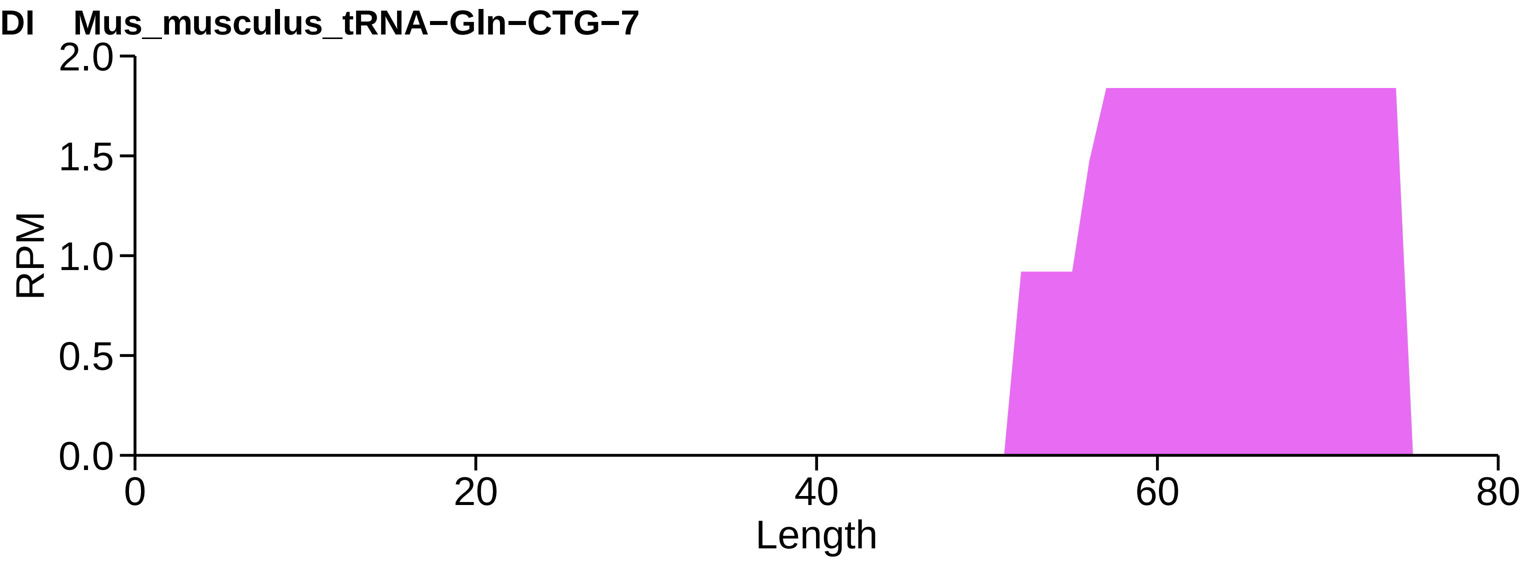
DI Mus_musculus_tRNA−Gln−CTG−7
2.0
1.5
1.0
0.5
0.0
RPM
0
20
40
60
80
Length

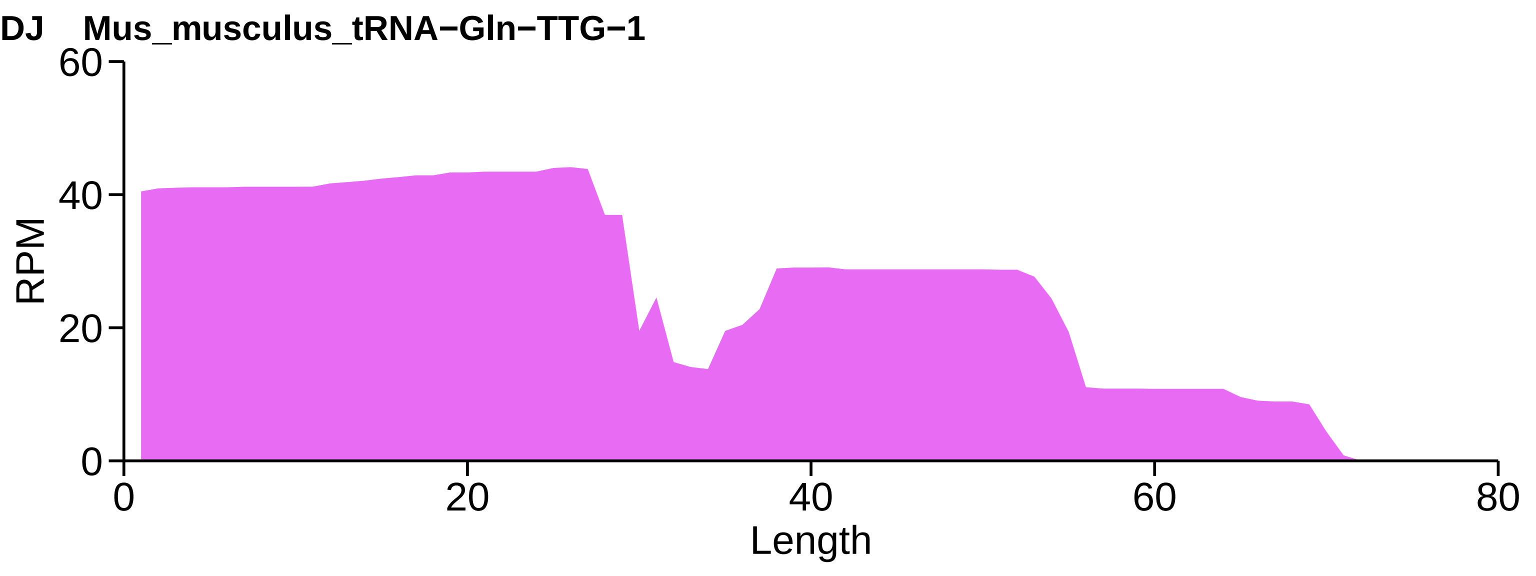

DJ Mus_musculus_tRNA−Gln−TTG−1
RPM
Length
0
20
40
60
0
20
40
60
80

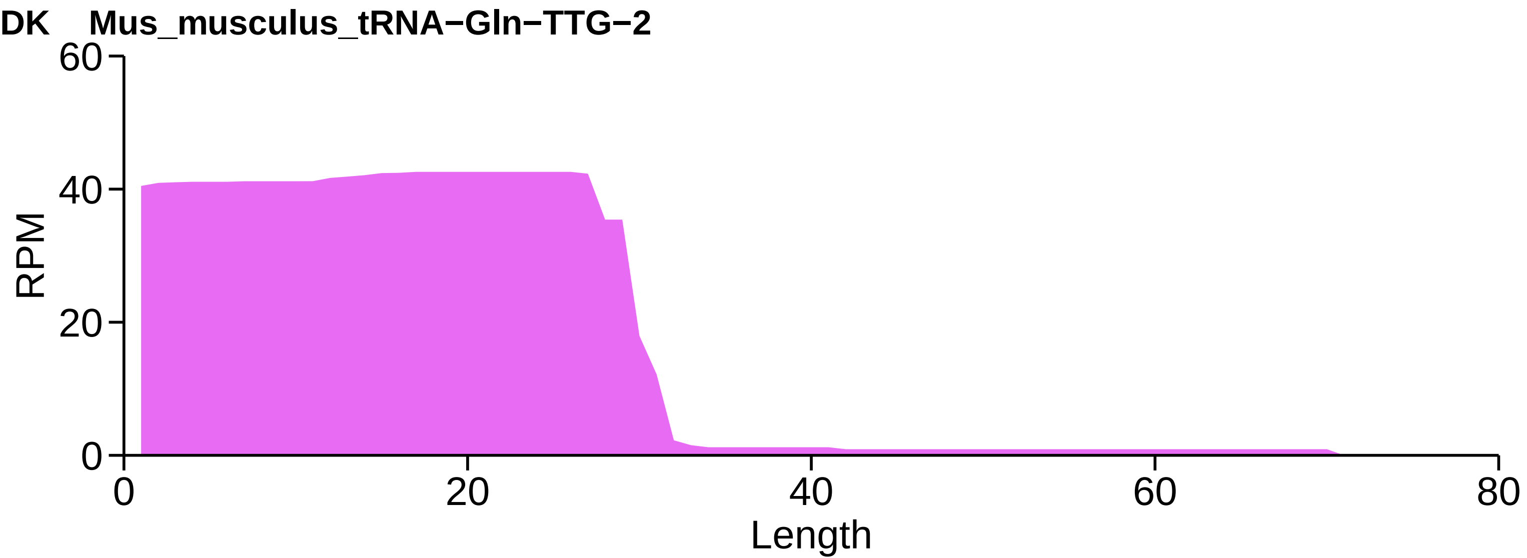
DK Mus_musculus_tRNA−Gln−TTG−2
RPM
60
40
20
0
0
20
40
60
80
Length

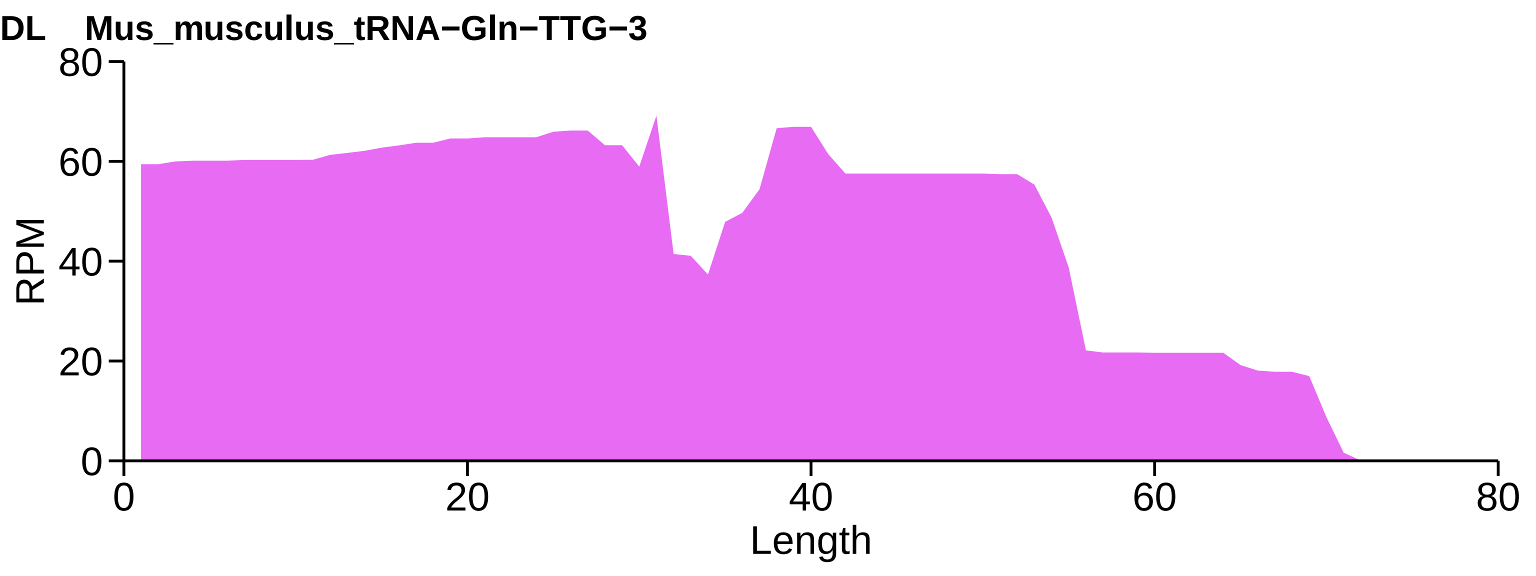
DL Mus_musculus_tRNA−Gln−TTG−3
RPM
80
60
40
20
0
0
20
40
60
80
Length

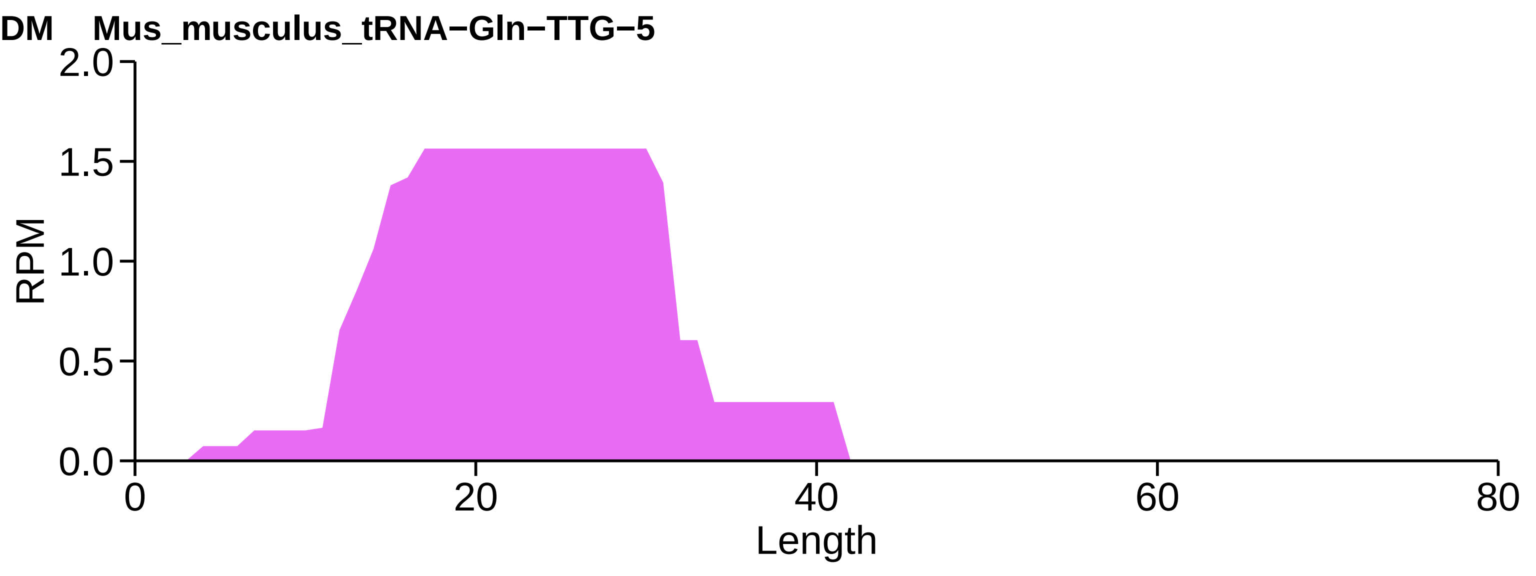
DM Mus_musculus_tRNA−Gln−TTG−5
RPM
2.0
1.5
1.0
0.5
0.0
0
20
40
60
80
Length

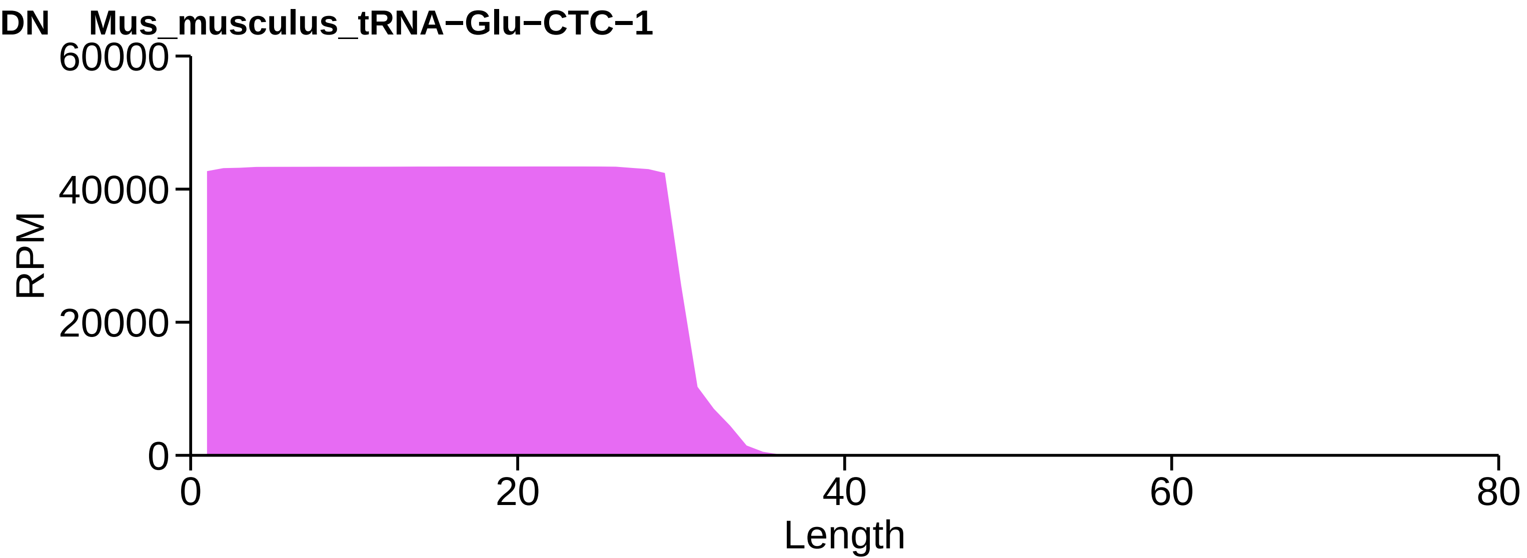

DN Mus_musculus_tRNA−Glu−CTC−1
RPM
60000
40000
20000
0
0
20
40
60
80
Length

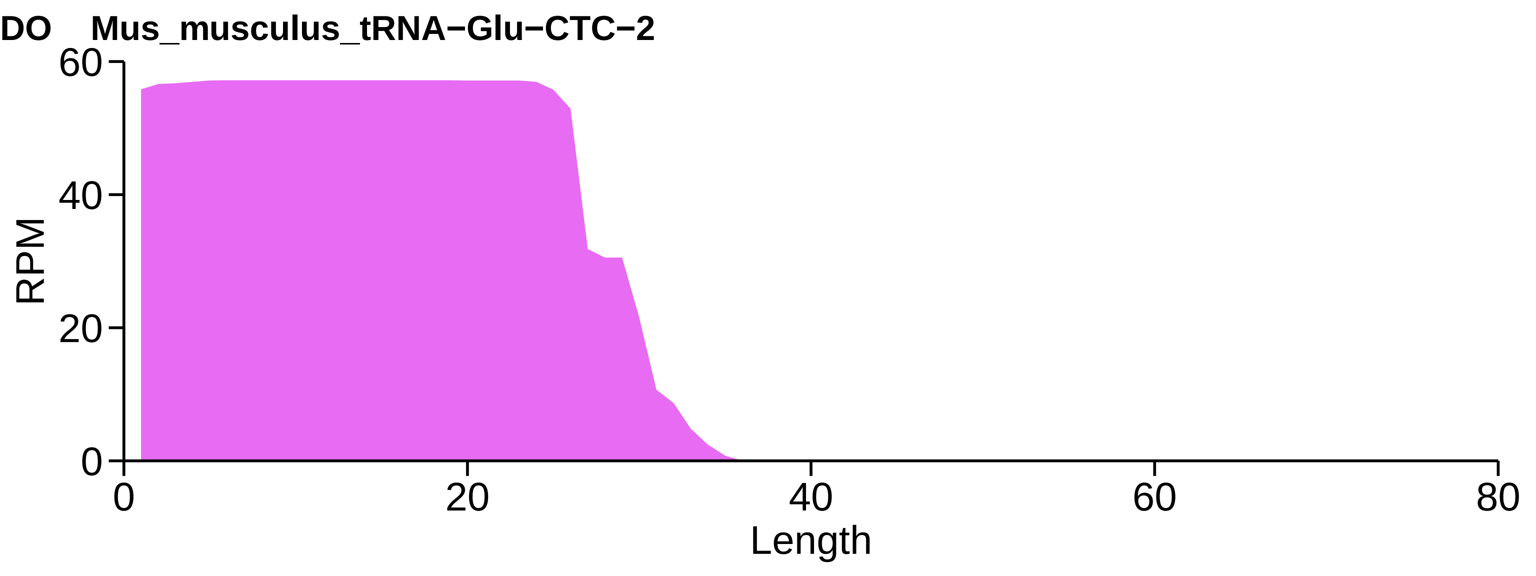

DO Mus_musculus_tRNA−Glu−CTC−2
RPM
Length
0
20
40
60
0
20
40
60
80

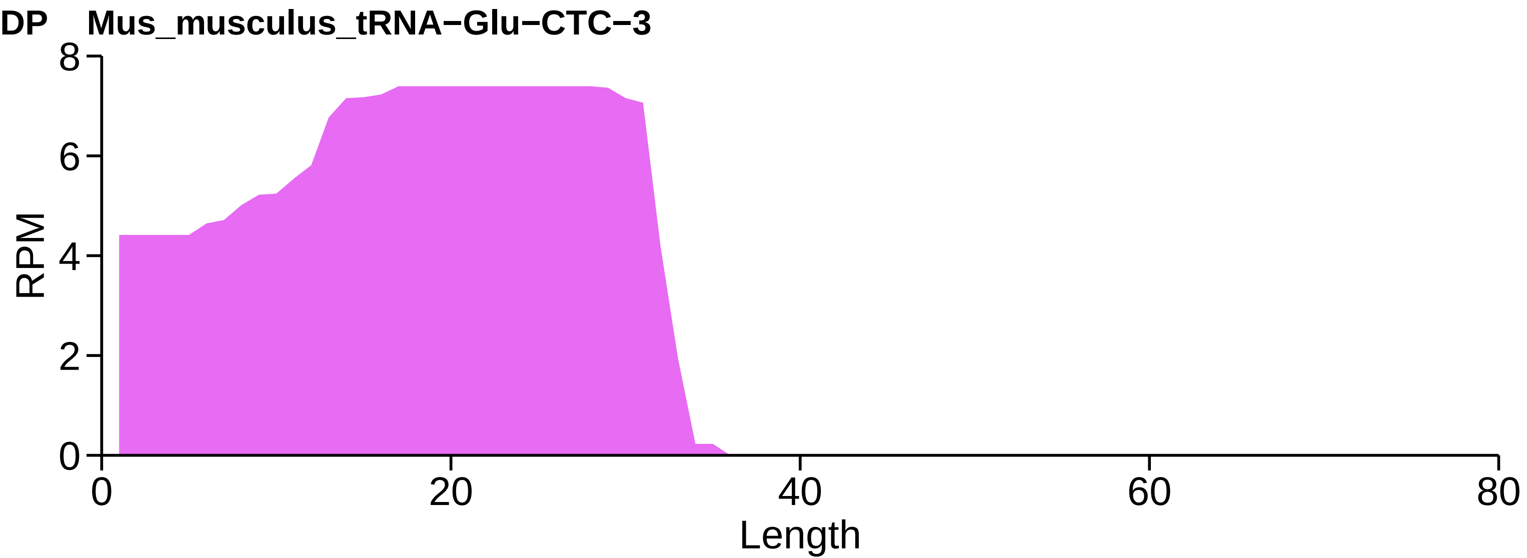
DP Mus_musculus_tRNA−Glu−CTC−3
RPM
0
2
4
6
8
0
20
40
60
80
Length

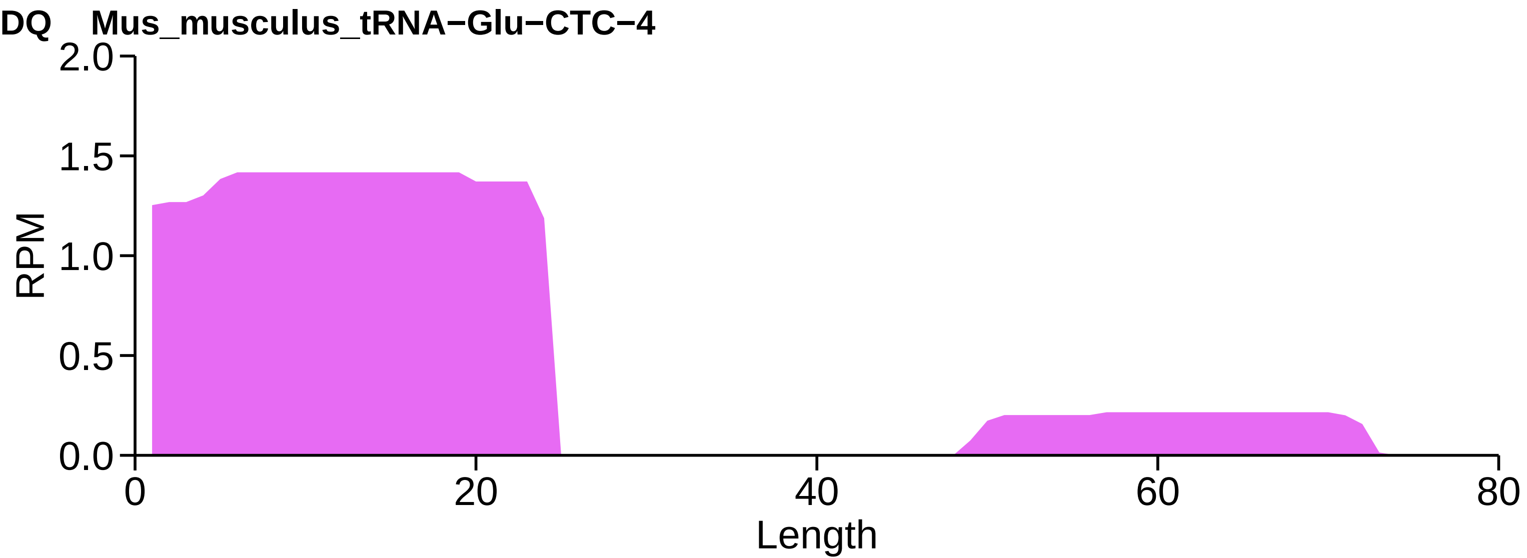
DQ Mus_musculus_tRNA−Glu−CTC−4
RPM
2.0
1.5
1.0
0.5
0.0
0
20
40
60
80
Length

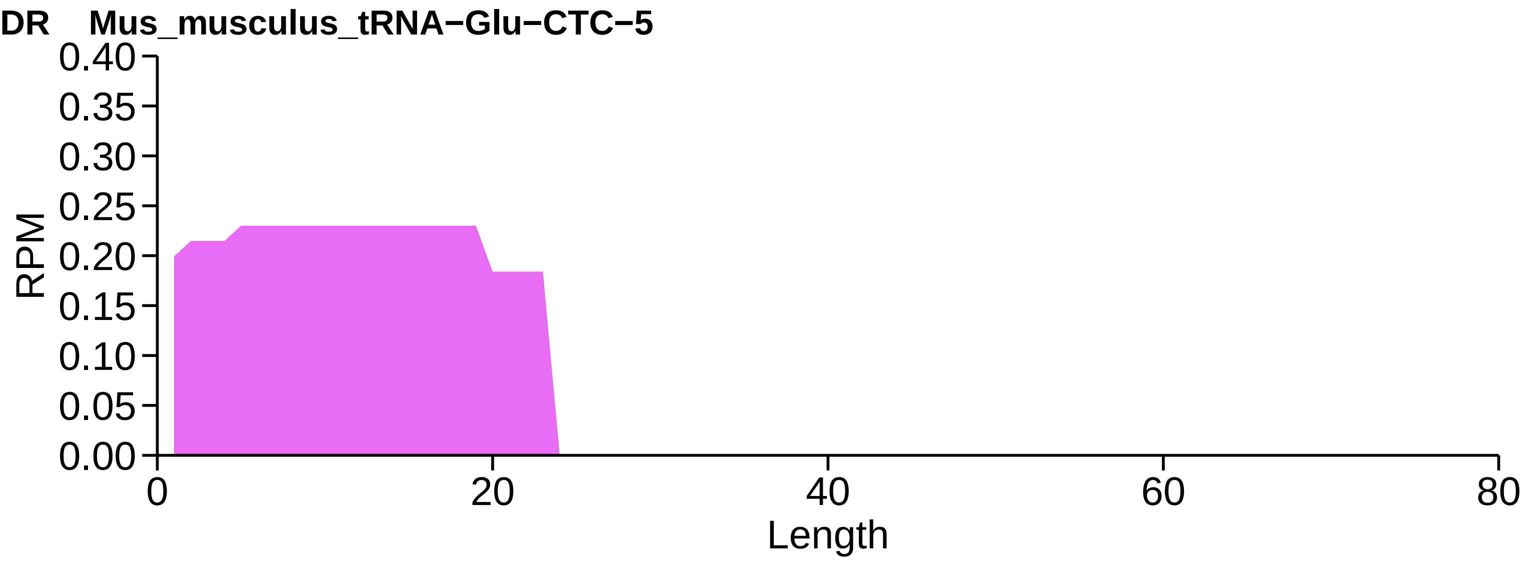
DR Mus_musculus_tRNA−Glu−CTC−5
RPM
0.40
0.35
0.30
0.25
0.20
0.15
0.10
0.05
0.00
0
20
40
60
80
Length

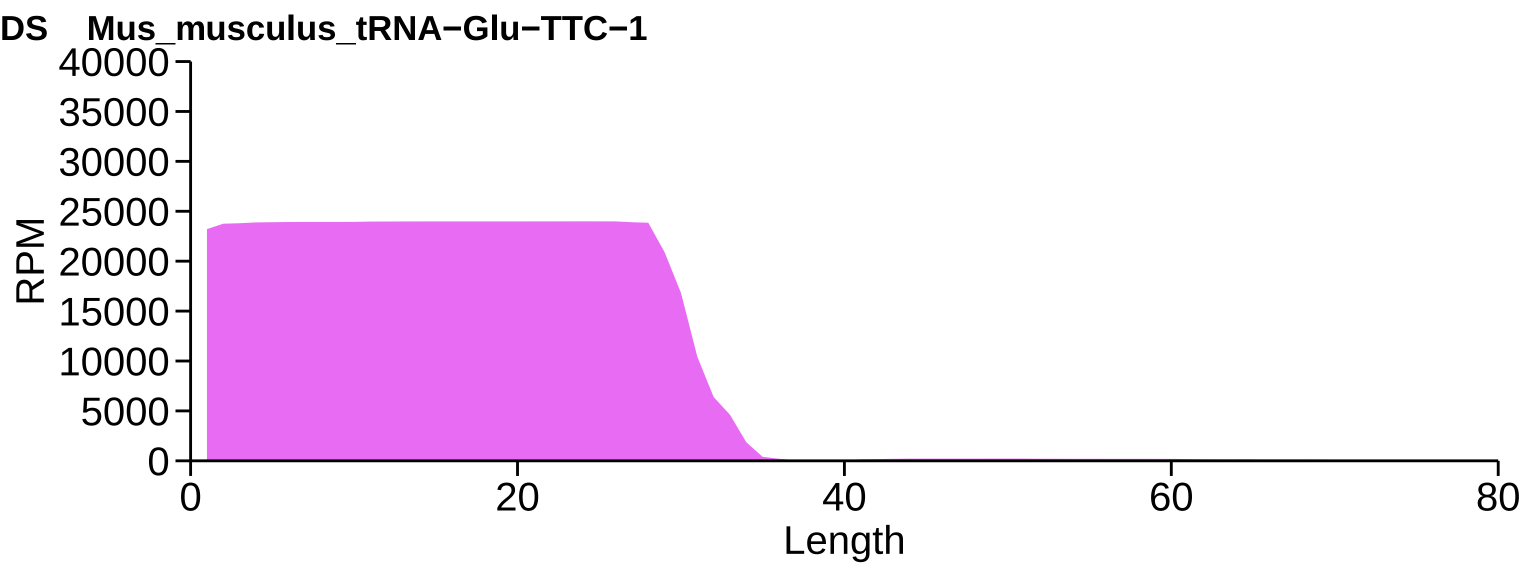
DS Mus_musculus_tRNA−Glu−TTC−1
RPM
40000
35000
30000
25000
20000
15000
10000
5000
0
0
20
40
60
80
Length

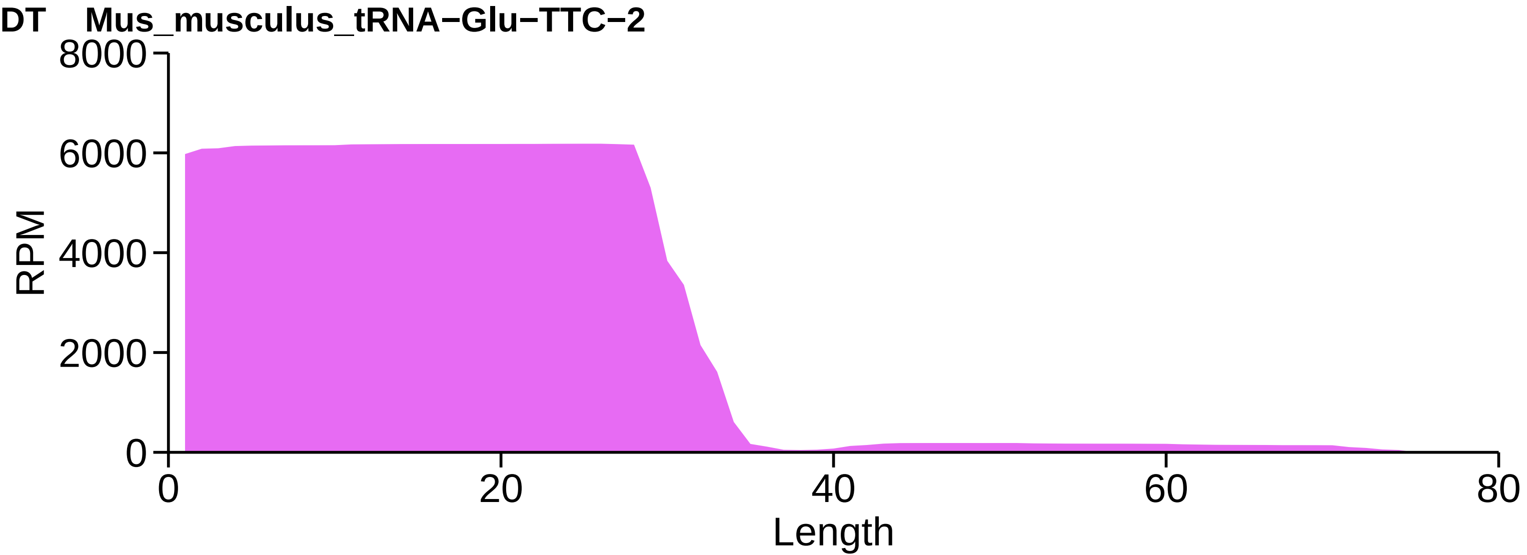
DT Mus_musculus_tRNA−Glu−TTC−2
8000
6000
4000
2000
0
RPM
0
20
40
60
80
Length

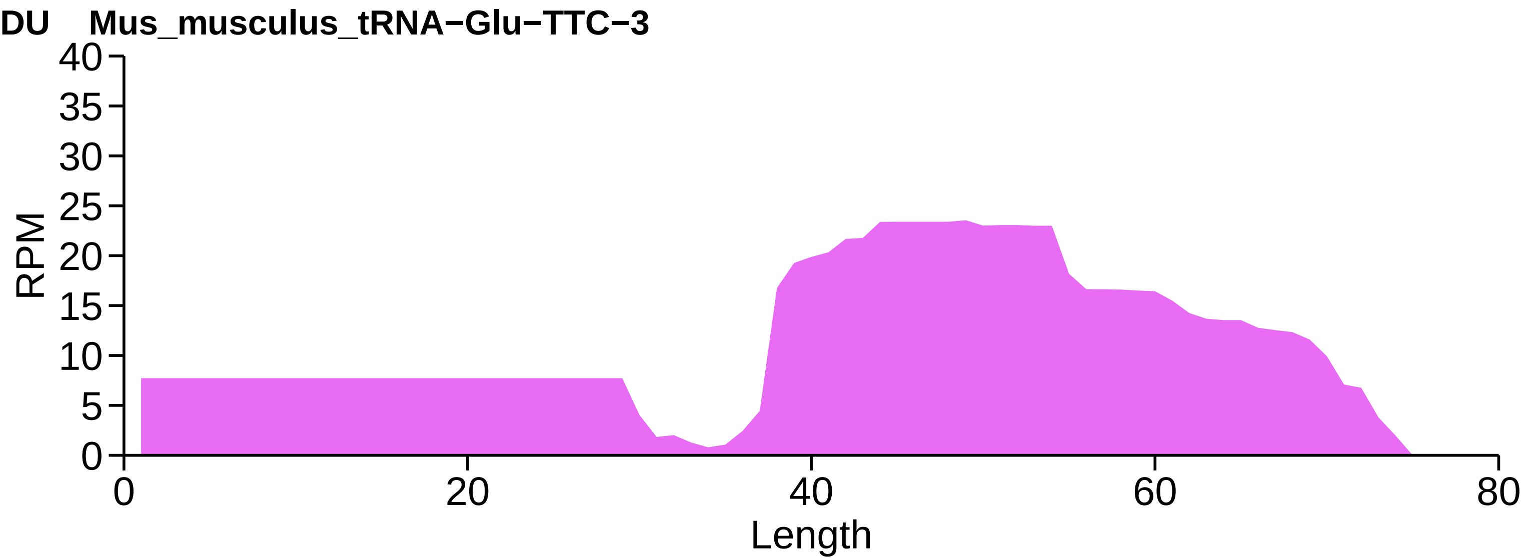

DU Mus_musculus_tRNA−Glu−TTC−3
RPM
0
5
10
15
20
25
30
35
40
0
20
40
60
80
Length

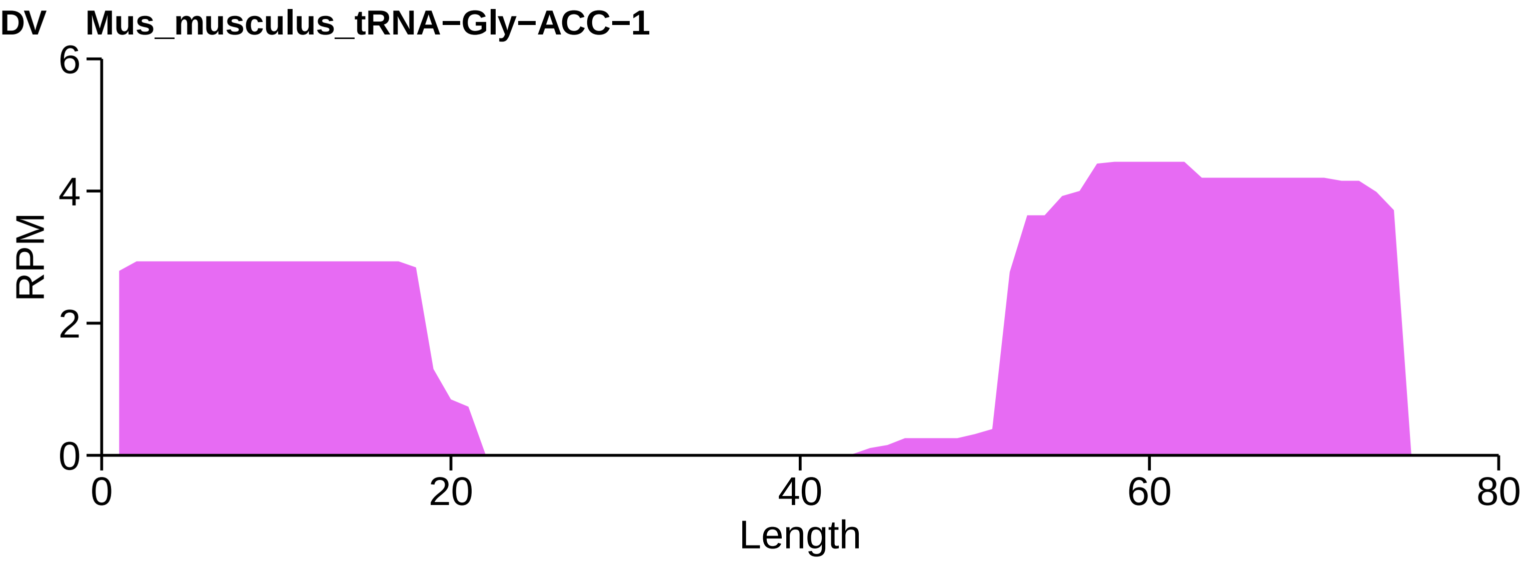
DV Mus_musculus_tRNA−Gly−ACC−1
RPM
0
2
4
6
0
20
40
60
80
Length

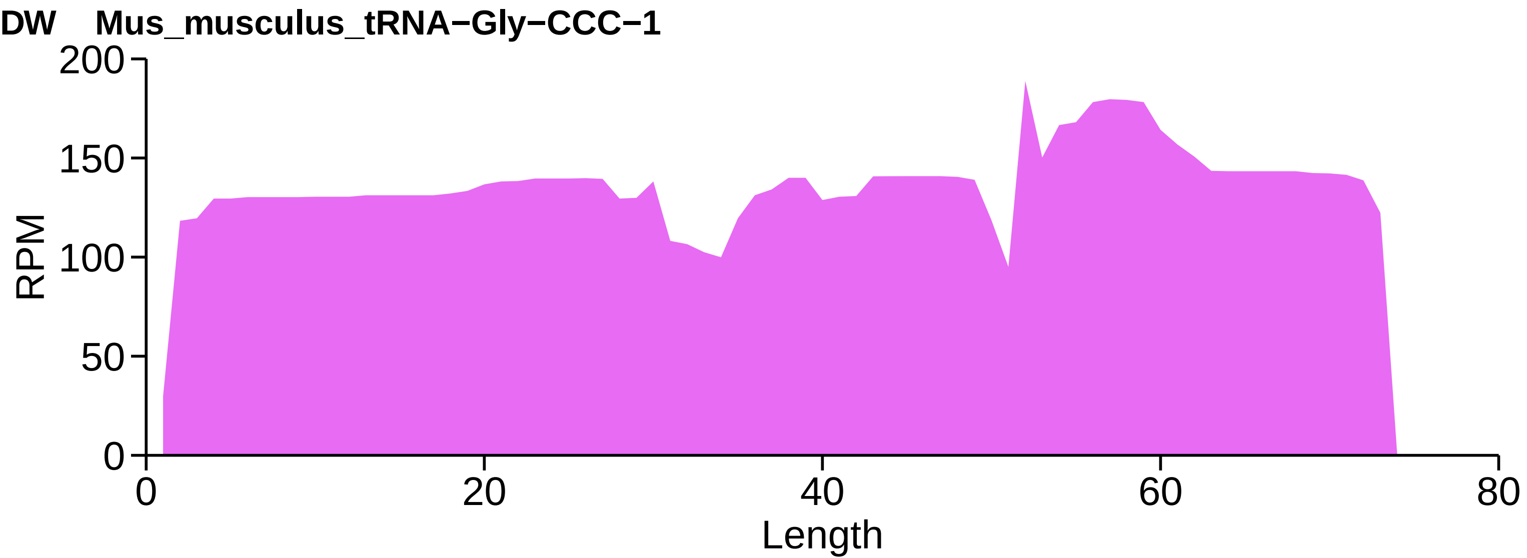
DW Mus_musculus_tRNA−Gly−CCC−1
RPM
0
50
100
150
200
0
20
40
60
80
Length

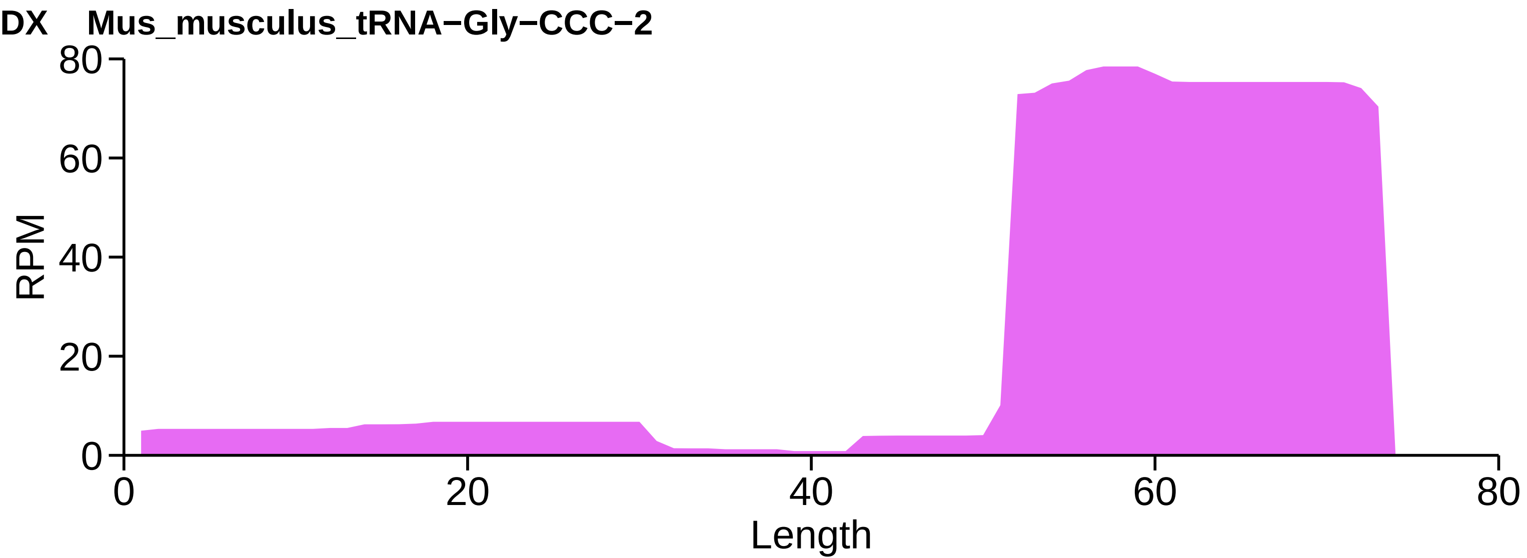
DX
Mus_musculus_tRNA−Gly−CCC−2
RPM
0
20
40
60
80
Length

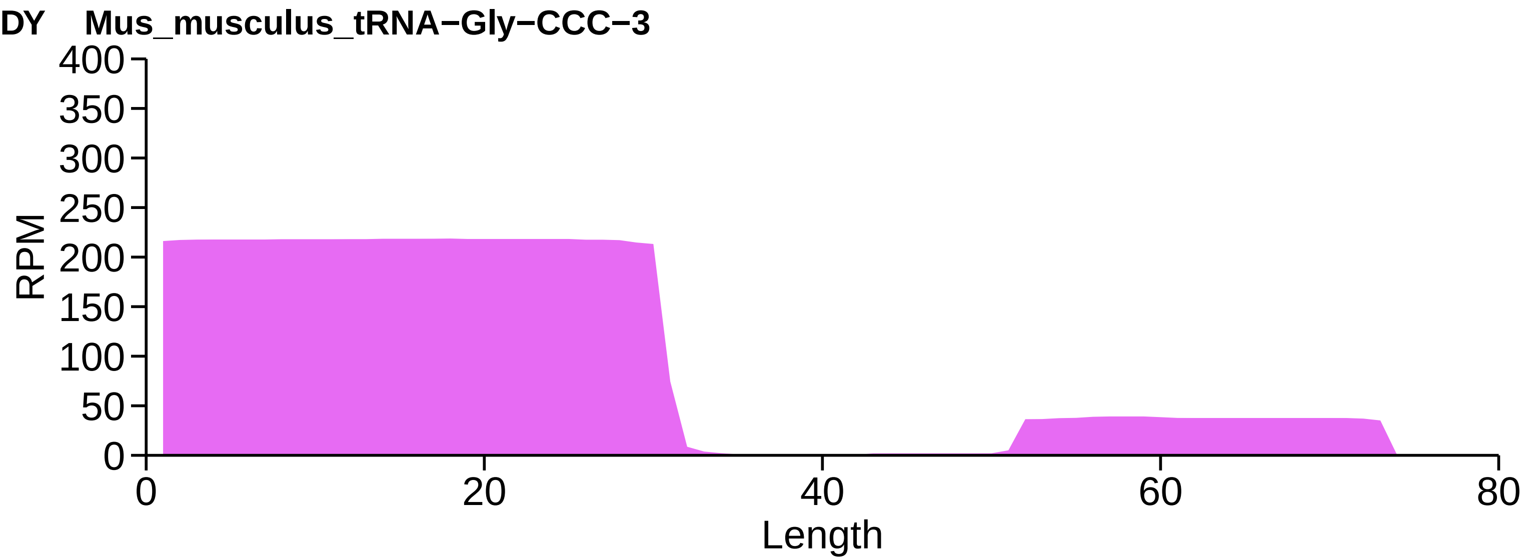
DY Mus_musculus_tRNA−Gly−CCC−3
RPM
400
350
300
250
200
150
100
50
0
0
20
40
60
80
Length

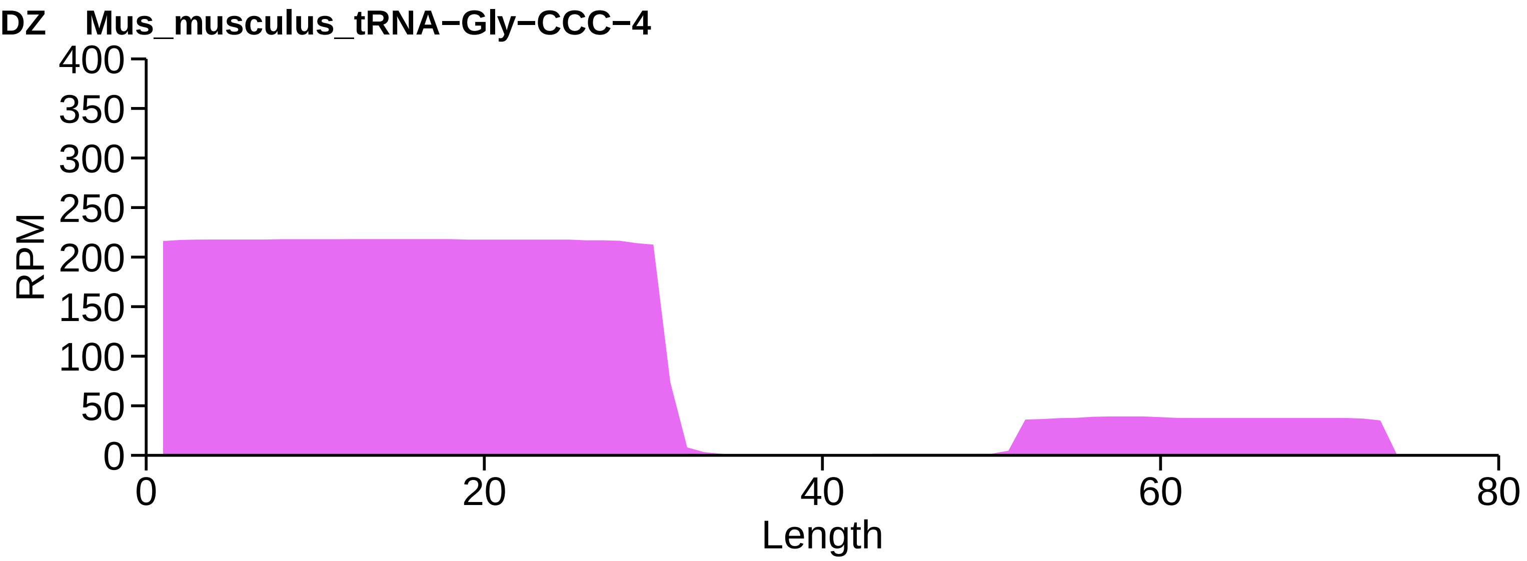

DZ Mus_musculus_tRNA−Gly−CCC−4
RPM
0
50
100
150
200
250
300
350
400
0
20
40
60
80
Length

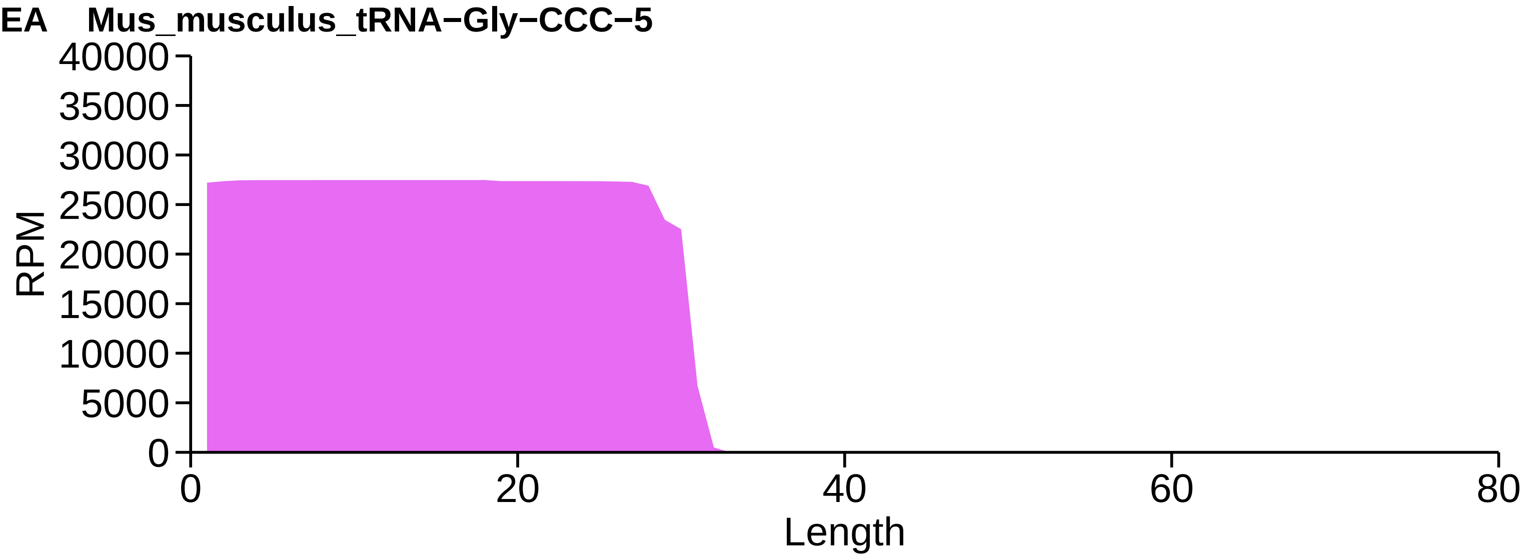

EA Mus_musculus_tRNA−Gly−CCC−5
40000
35000
30000
25000
20000
15000
10000
5000
0
RPM
0
20
40
60
80
Length

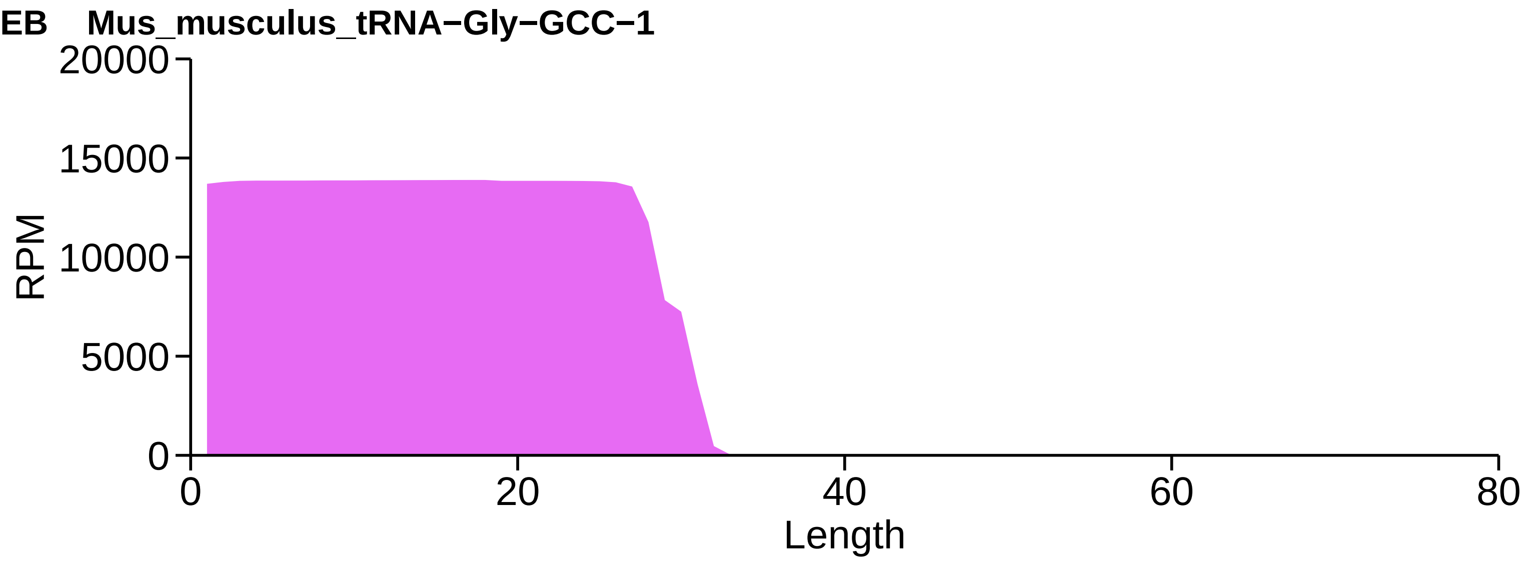
EB Mus_musculus_tRNA−Gly−GCC−1
RPM
20000
15000
10000
5000
0
0
20
40
60
80
Length

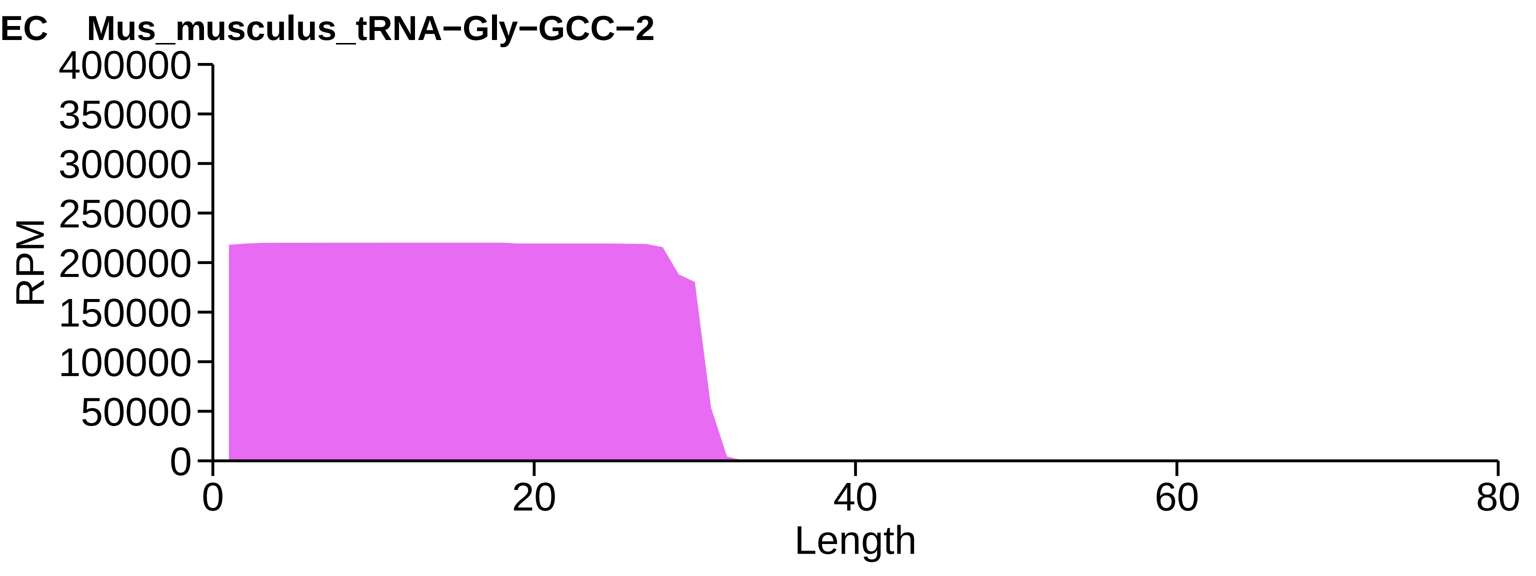
EC Mus_musculus_tRNA−Gly−GCC−2
400000
350000
300000
250000
200000
150000
100000
50000
0
RPM
0
20
40
60
80
Length

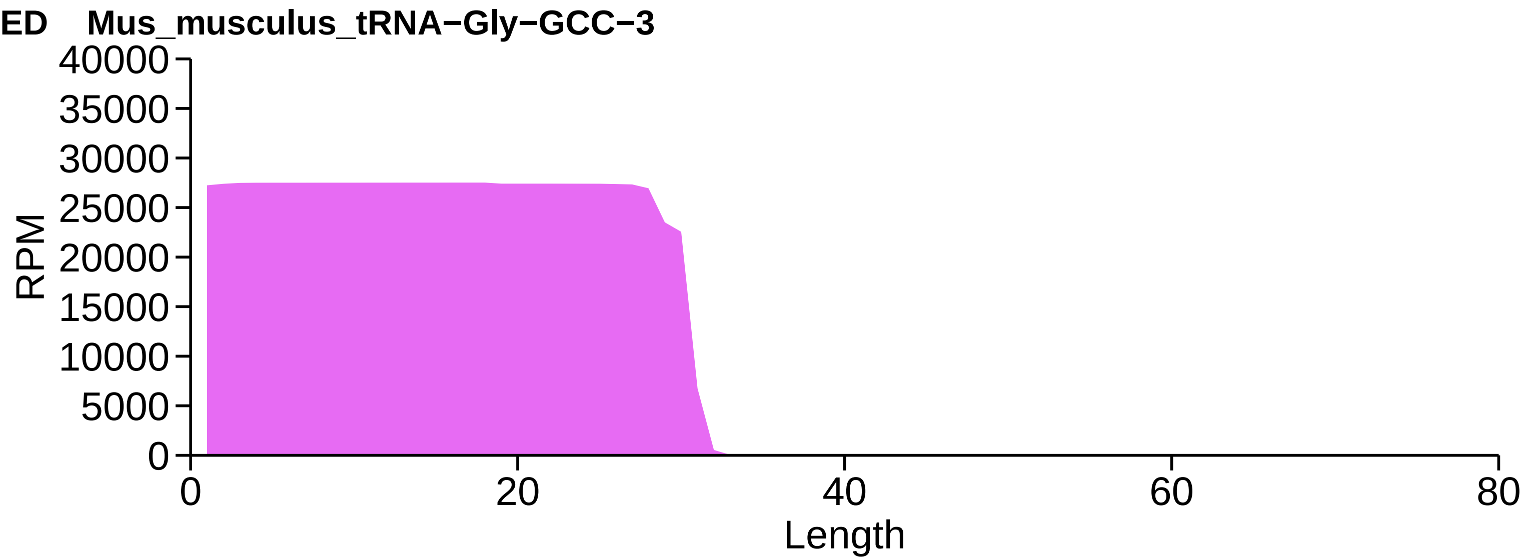
ED Mus_musculus_tRNA−Gly−GCC−3
RPM
Length
0
5000
10000
15000
20000
25000
30000
35000
40000
0
20
40
60
80

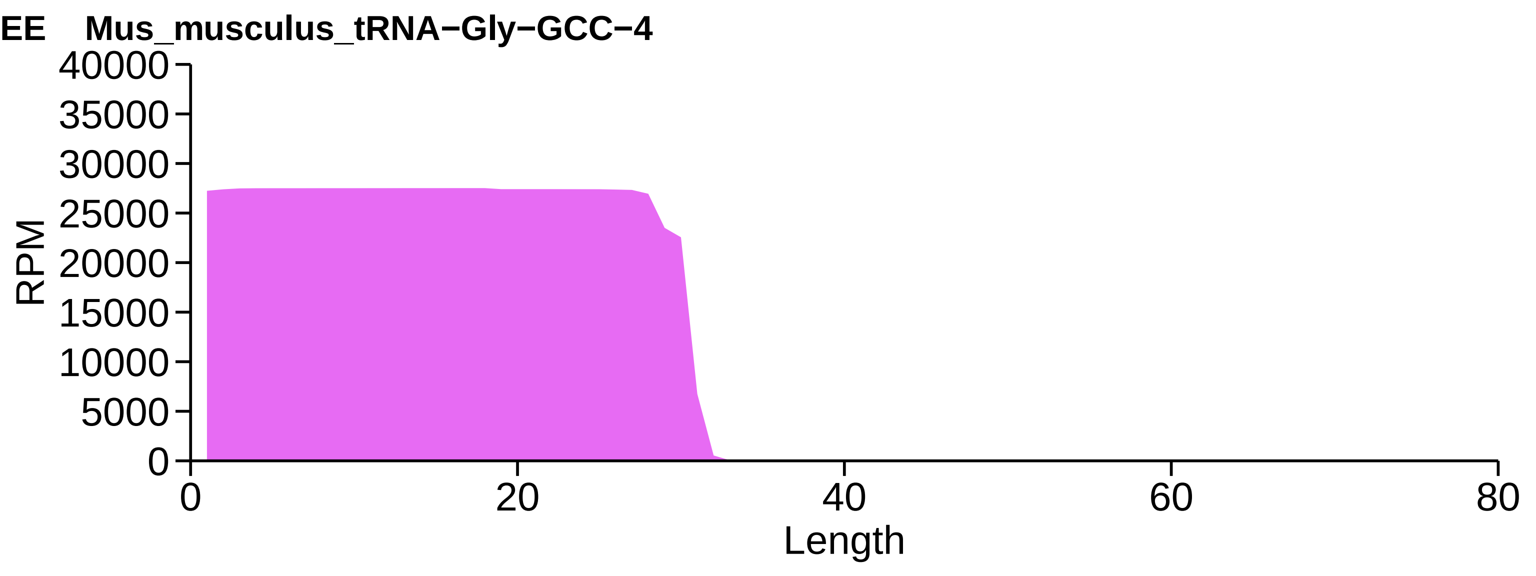
EE Mus_musculus_tRNA−Gly−GCC−4
RPM
40000
35000
30000
25000
20000
15000
10000
5000
0
0
20
40
60
80
Length

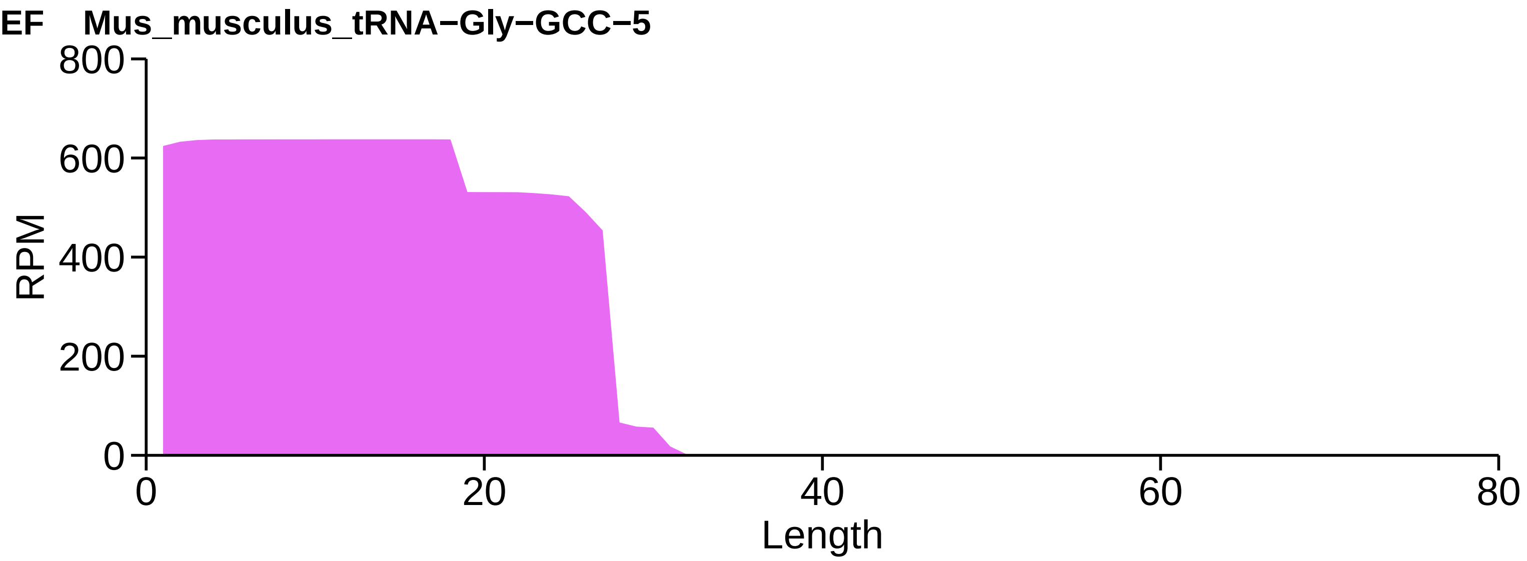
EF Mus_musculus_tRNA−Gly−GCC−5
RPM
800
600
400
200
0
0
20
40
60
80
Length

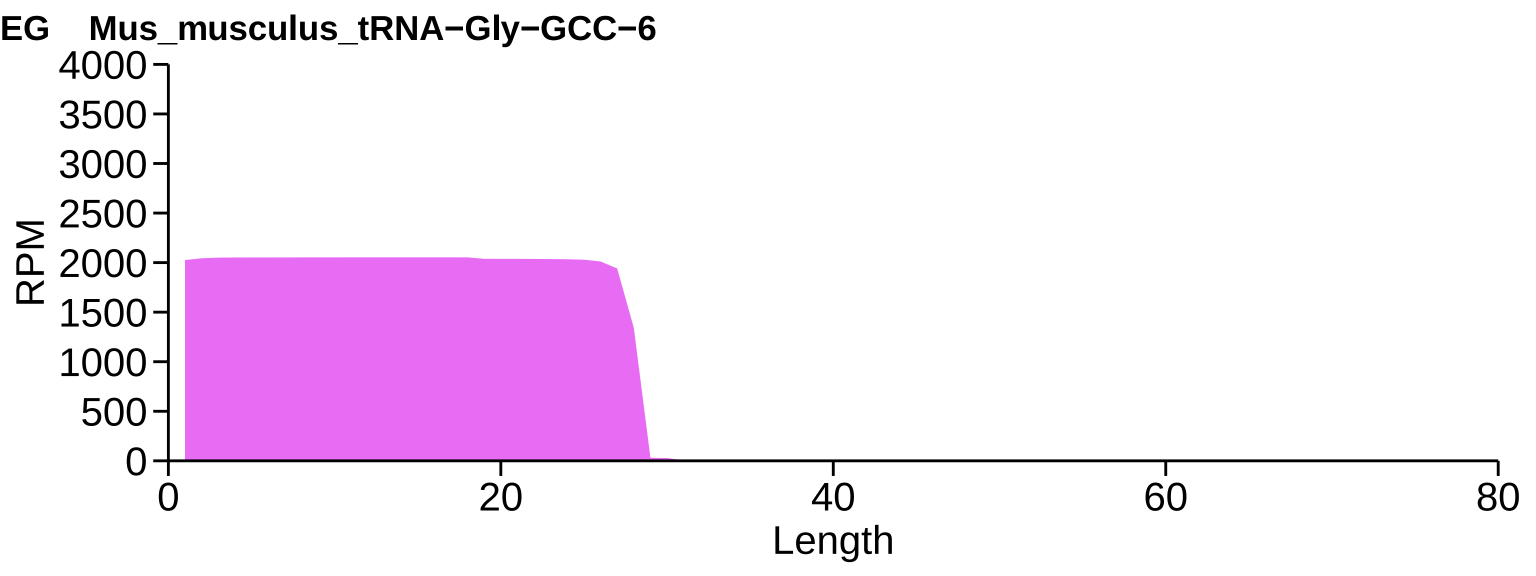
EG Mus_musculus_tRNA−Gly−GCC−6
RPM
4000
3500
3000
2500
2000
1500
1000
500
0
20
40
60
80
Length

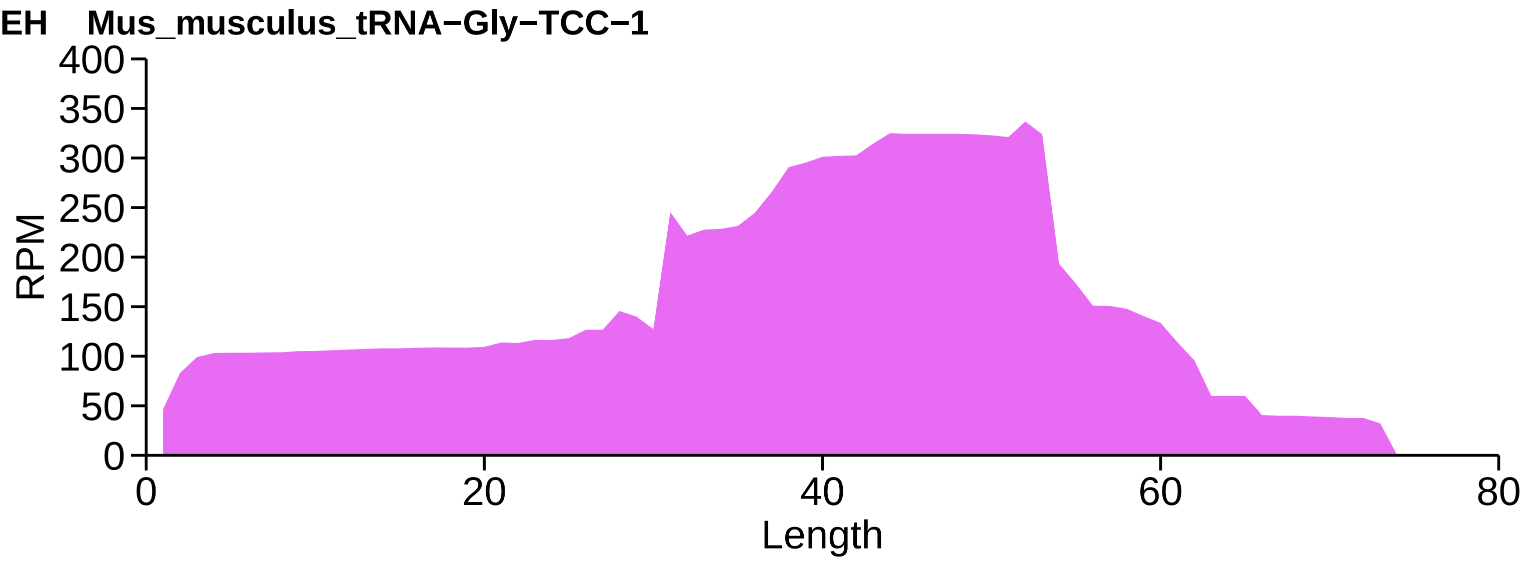
EH Mus_musculus_tRNA−Gly−TCC−1
RPM
0
50
100
150
200
250
300
350
400
0
20
40
60
80
Length

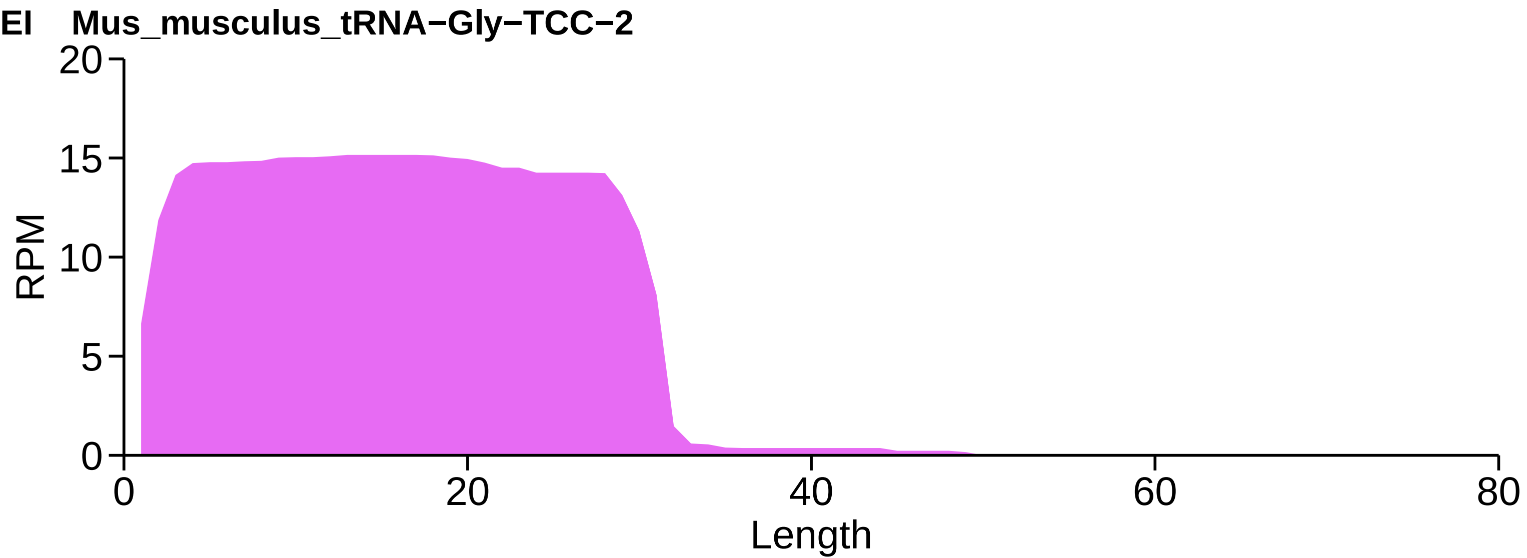
EI Mus_musculus_tRNA−Gly−TCC−2
20
15
10
5
0
RPM
0
20
40
60
80
Length

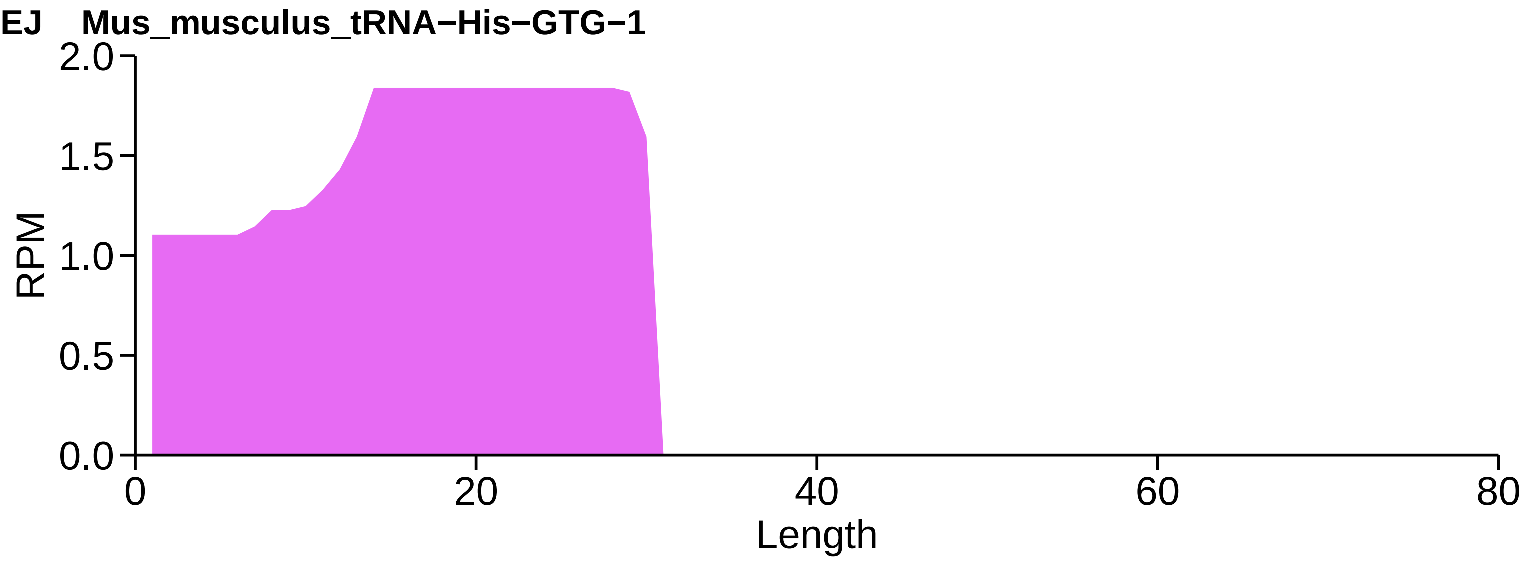

EJ Mus_musculus_tRNA−His−GTG−1
RPM
0.0
0.5
1.0
1.5
2.0
0
20
40
60
80
Length

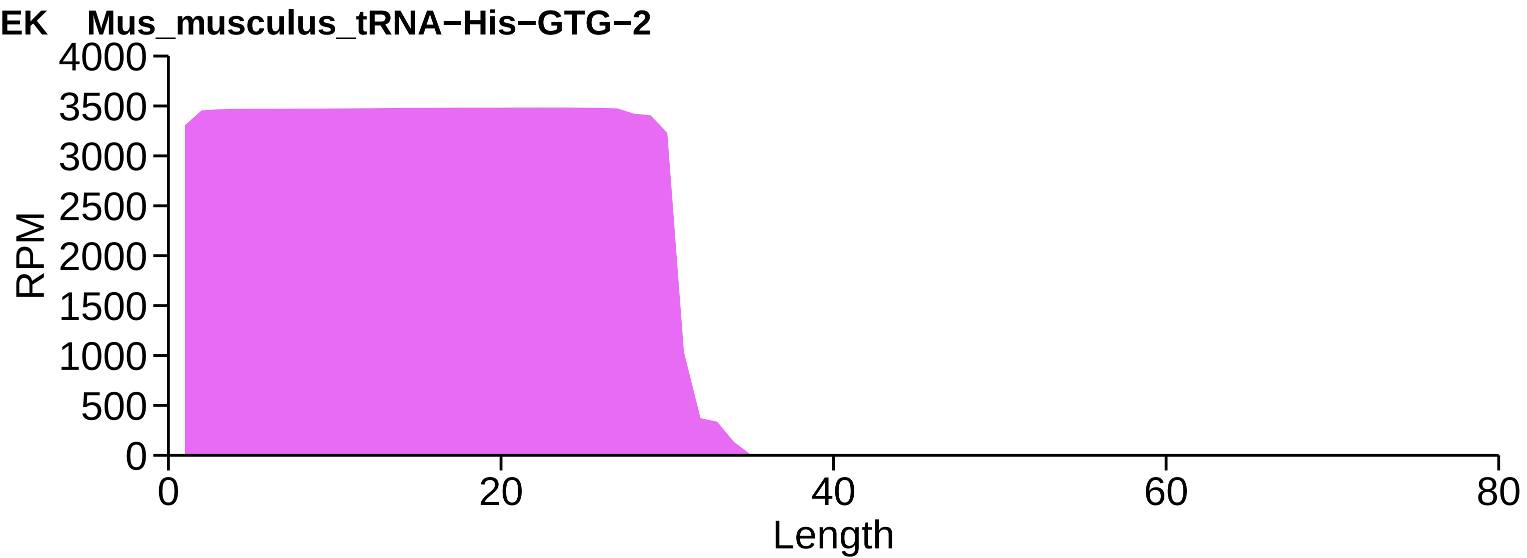
EK Mus_musculus_tRNA−His−GTG−2
4000
3500
3000
2500
2000
1500
1000
500
0
RPM
0
20
40
60
80
Length

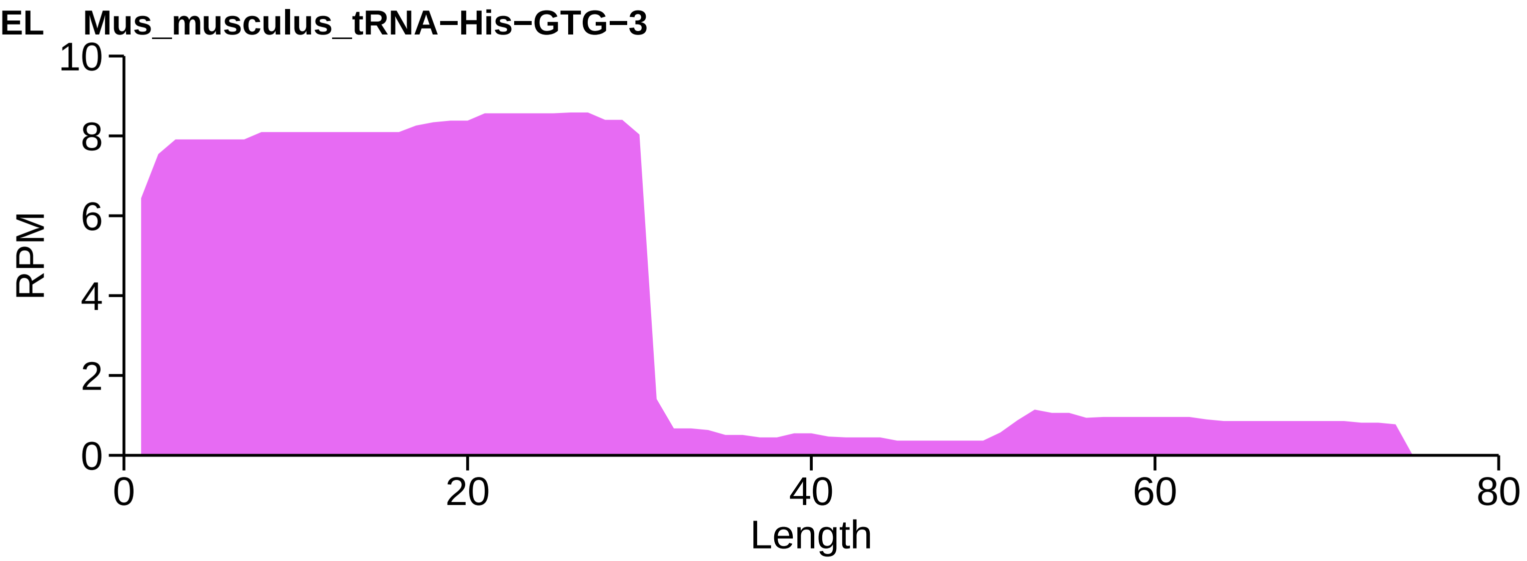

EL Mus_musculus_tRNA−His−GTG−3
RPM
Length
0
2
4
6
8
10
0
20
40
60
80

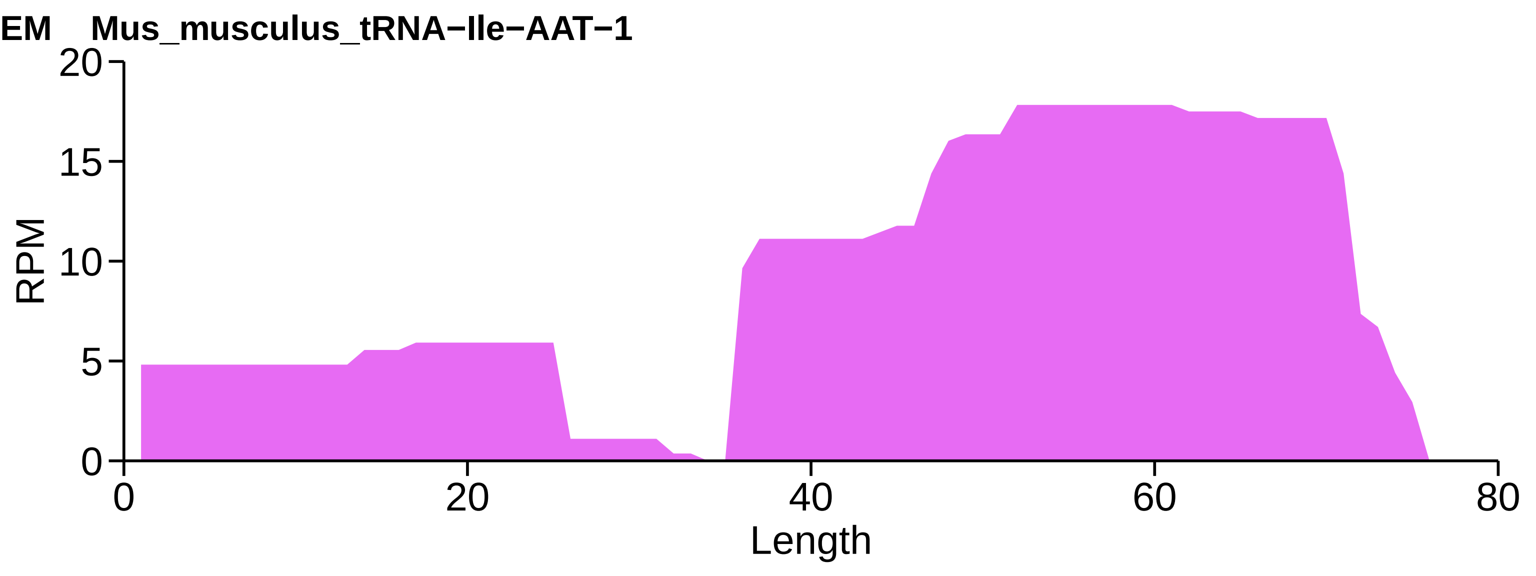
EM Mus_musculus_tRNA−Ile−AAT−1
RPM
20
15
10
5
0
0
20
40
60
80
Length

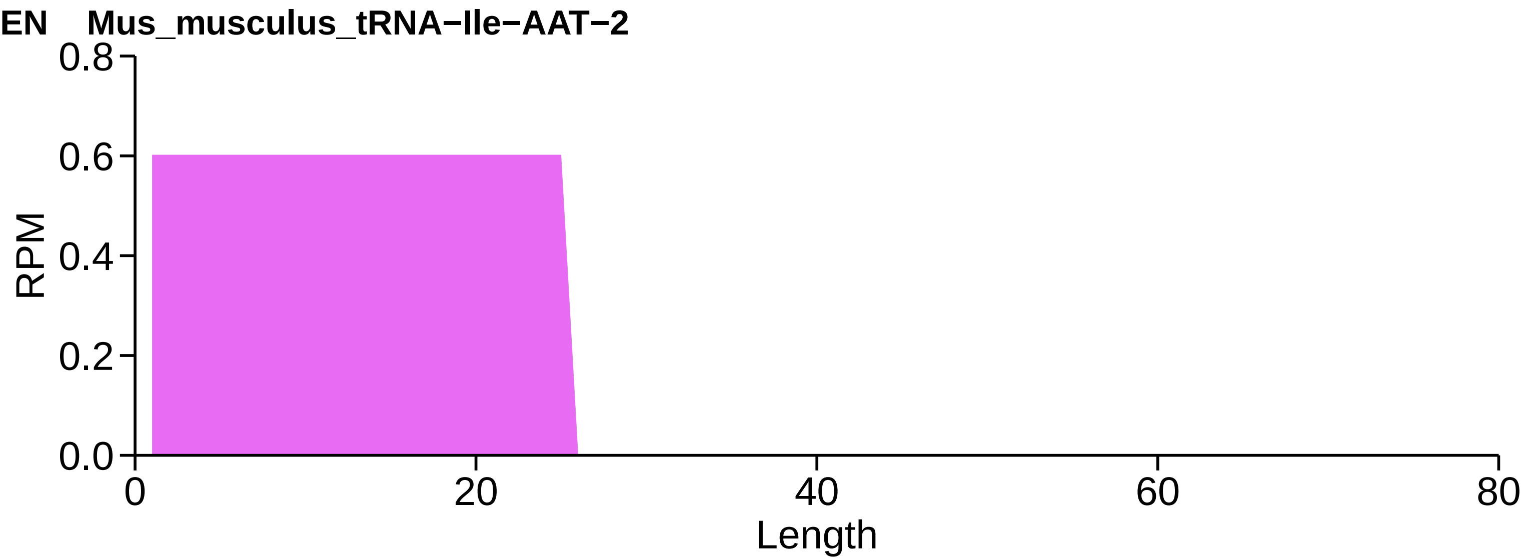

EN Mus_musculus_tRNA−Ile−AAT−2
0.8
0.6
0.4
0.2
0.0
RPM
0
20
40
60
80
Length

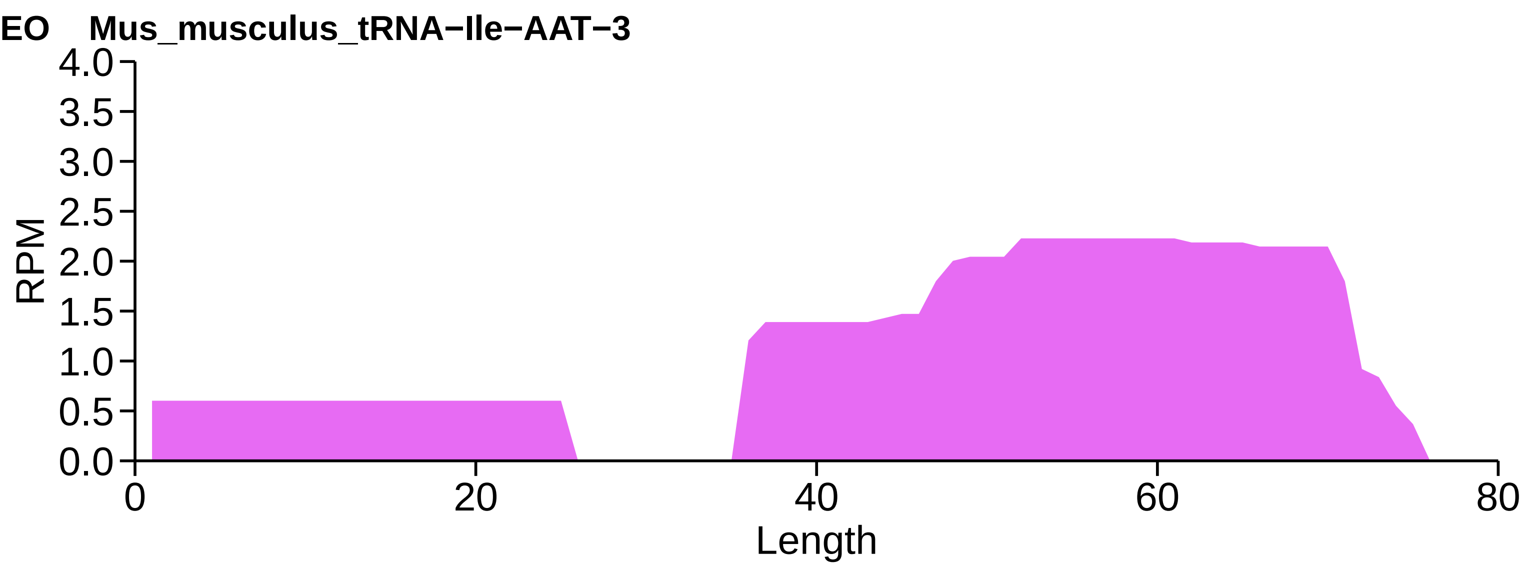
EO Mus_musculus_tRNA−Ile−AAT−3
RPM
4.0
3.5
3.0
2.5
2.0
1.5
1.0
0.5
0.0
0
20
40
60
80
Length

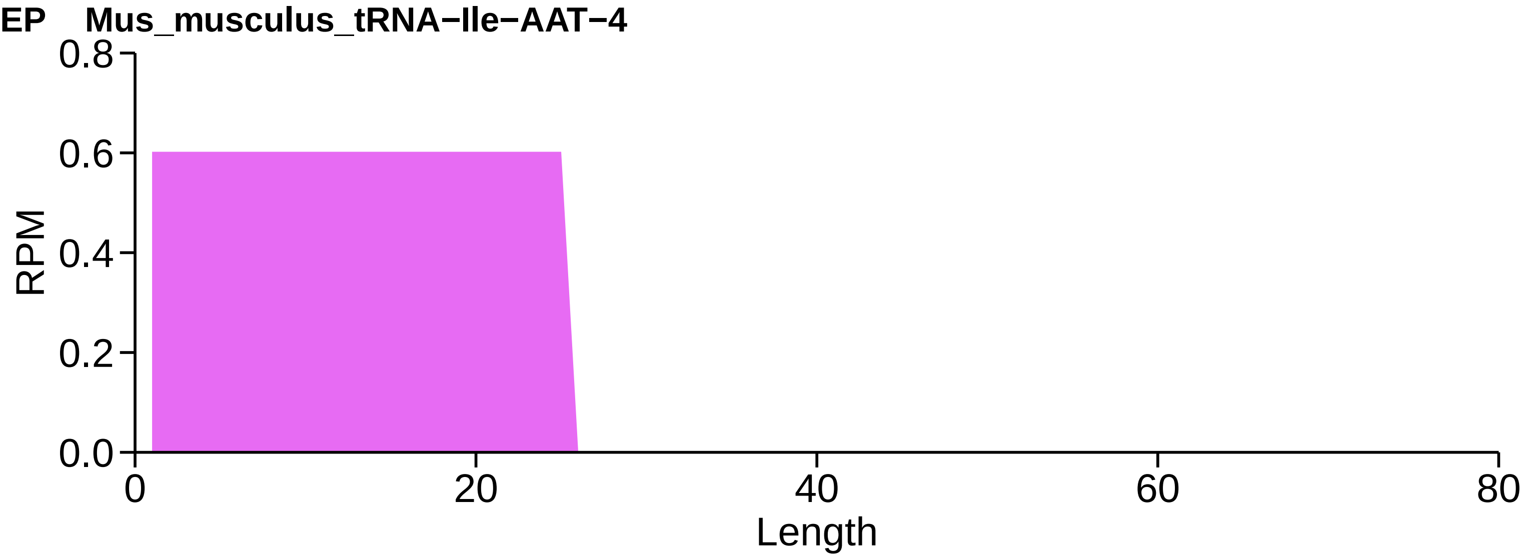

EP Mus_musculus_tRNA−Ile−AAT−4
0.8
0.6
0.4
0.2
0.0
RPM
0
20
40
60
80
Length

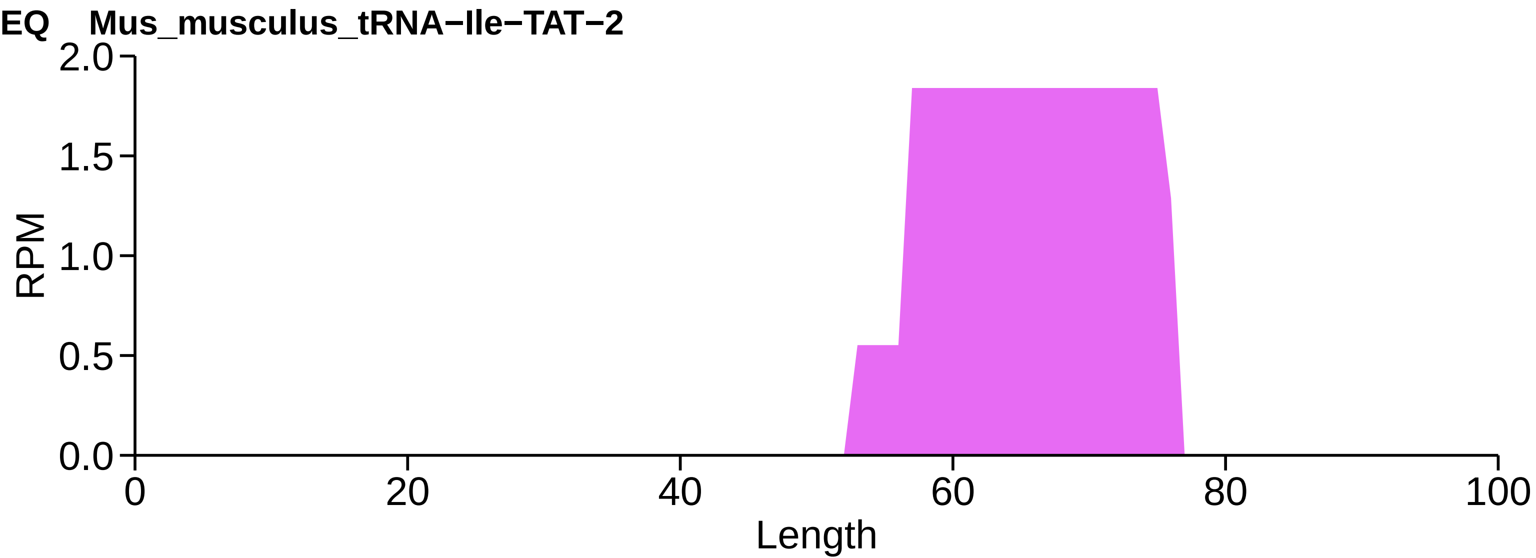
EQ Mus_musculus_tRNA−Ile−TAT−2
RPM
2.0
1.5
1.0
0.5
0.0
0
20
40
60
80
100
Length

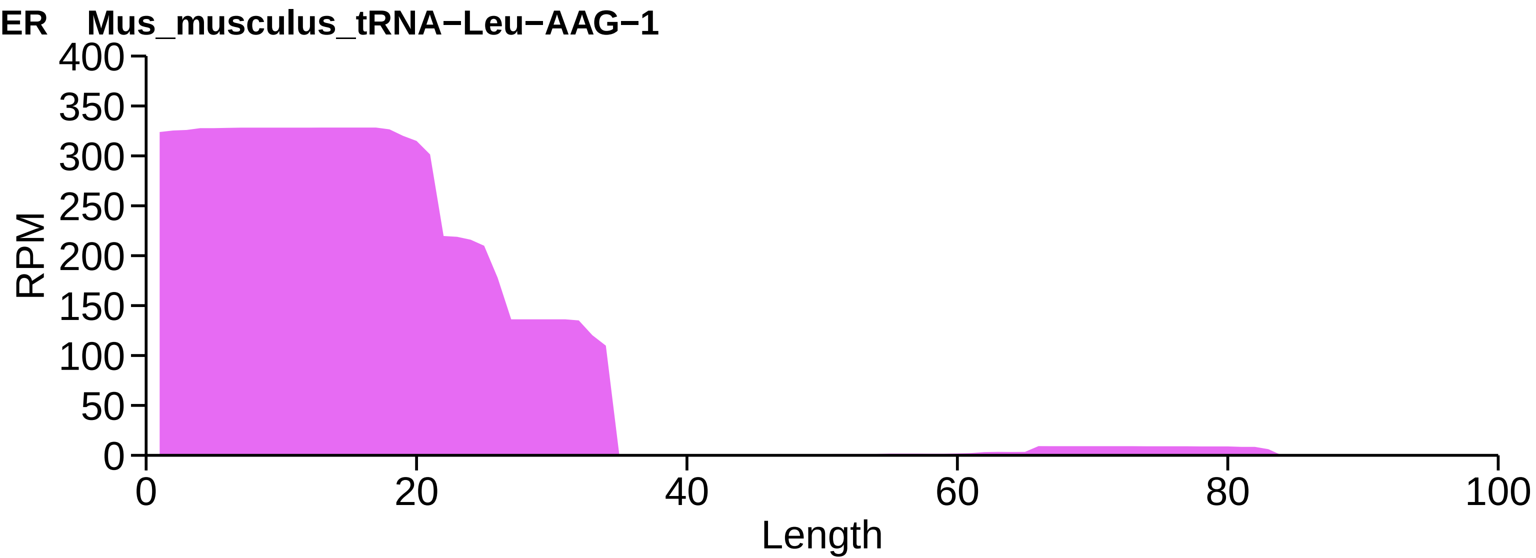

ER Mus_musculus_tRNA−Leu−AAG−1
RPM
400
350
300
250
200
150
100
50
0
0
20
40
60
80
100
Length

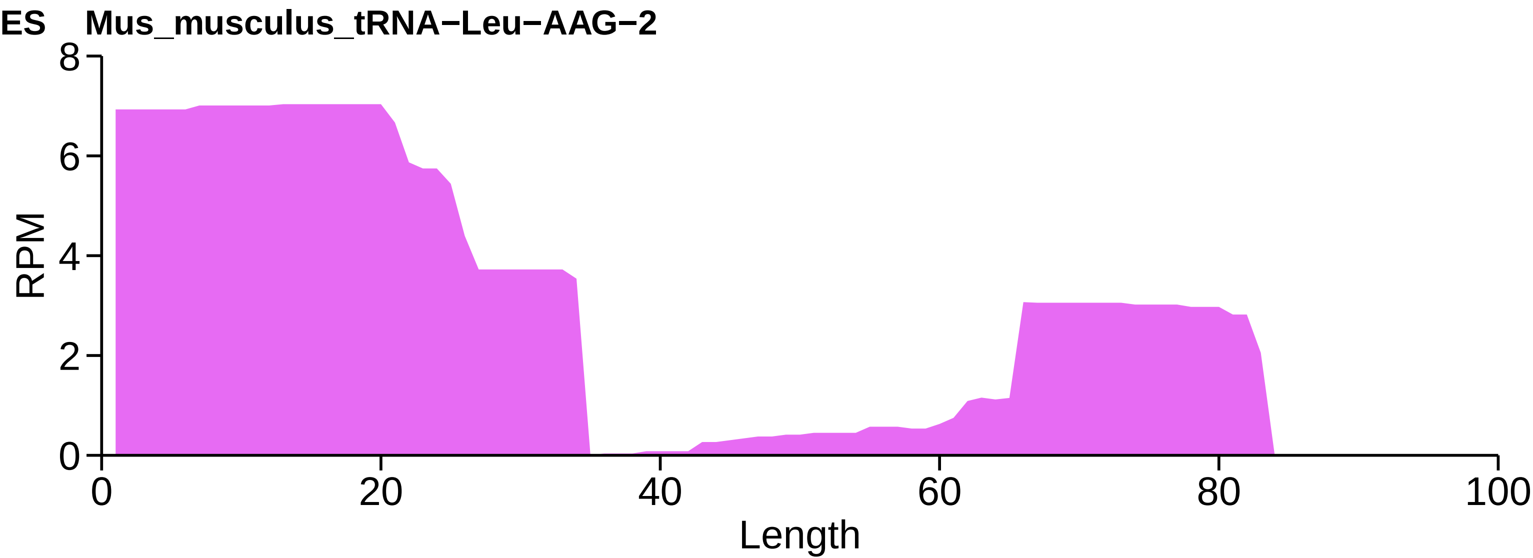
ES Mus_musculus_tRNA−Leu−AAG−2
RPM
8
6
4
2
0
0
20
40
60
80
100
Length

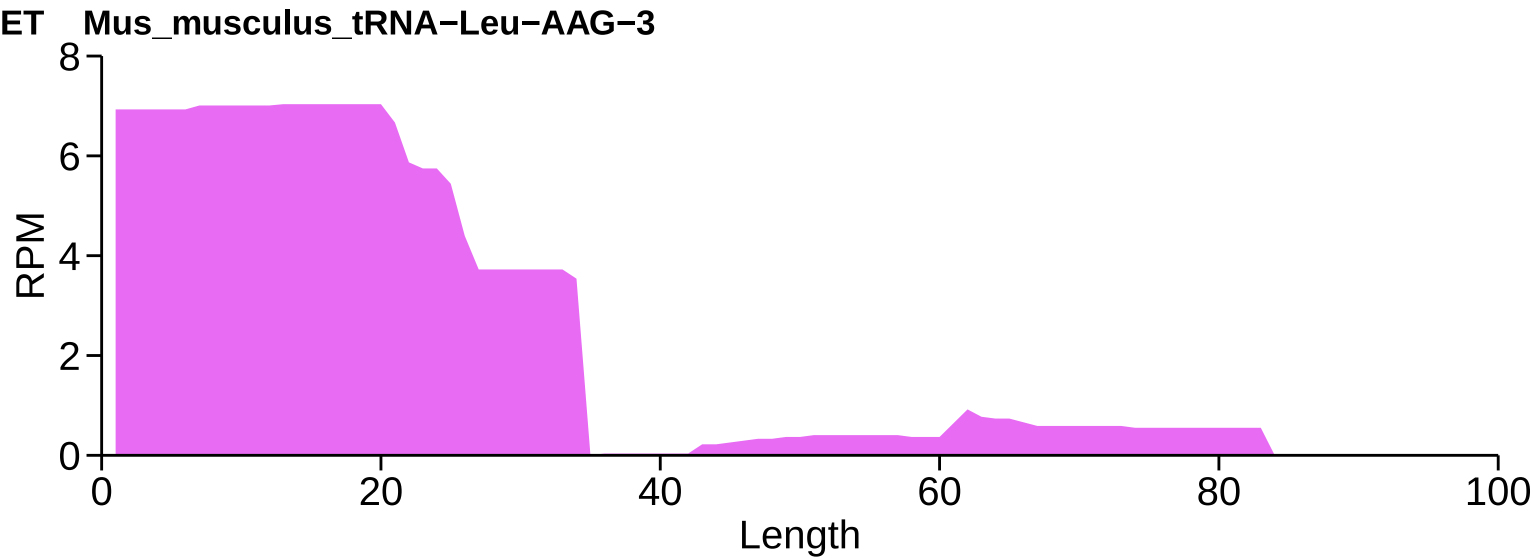
ET Mus_musculus_tRNA−Leu−AAG−3
RPM
0
2
4
6
8
0
20
40
60
80
100
Length

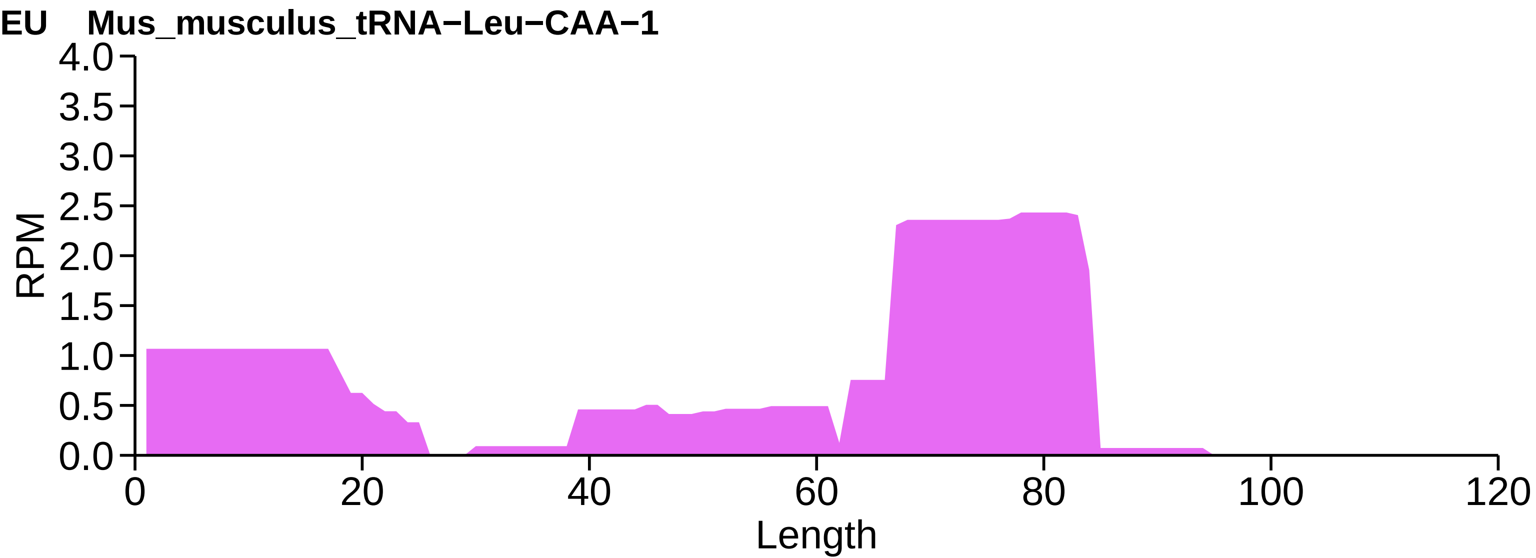

EU Mus_musculus_tRNA−Leu−CAA−1
RPM
4.0
3.5
3.0
2.5
2.0
1.5
1.0
0.5
0.0
0
20
40
60
80
100
120
Length

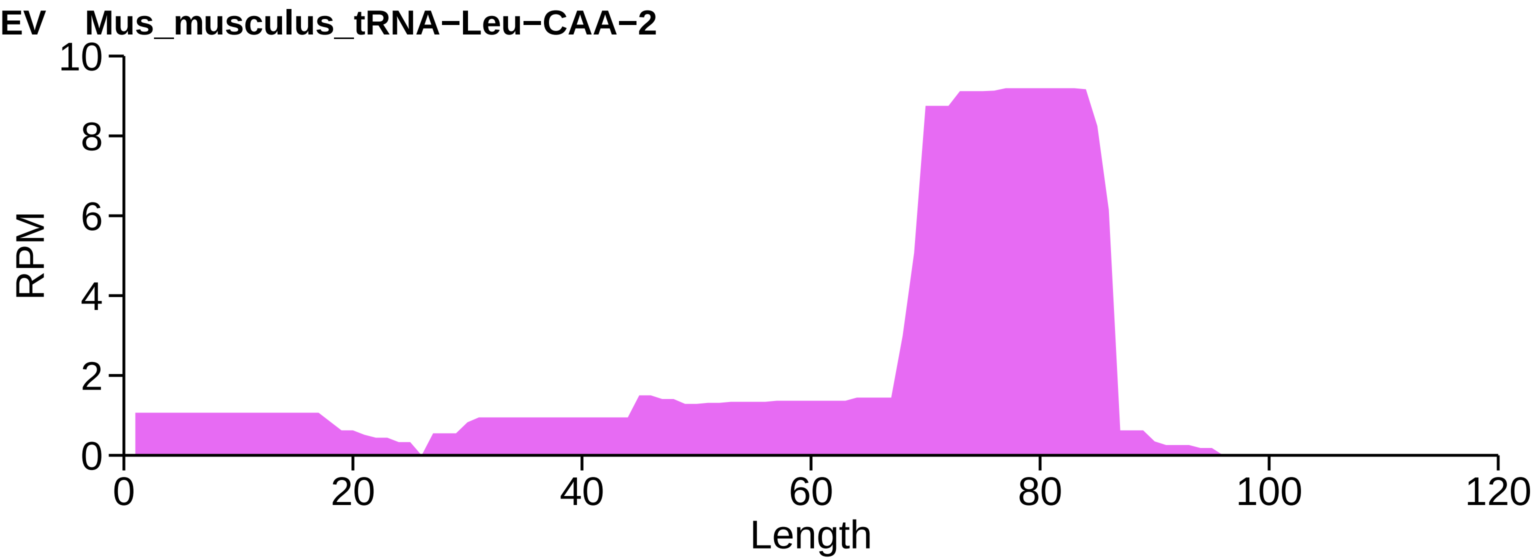
EV Mus_musculus_tRNA−Leu−CAA−2
RPM
Length
0
2
4
6
8
10
0
20
40
60
80
100
120

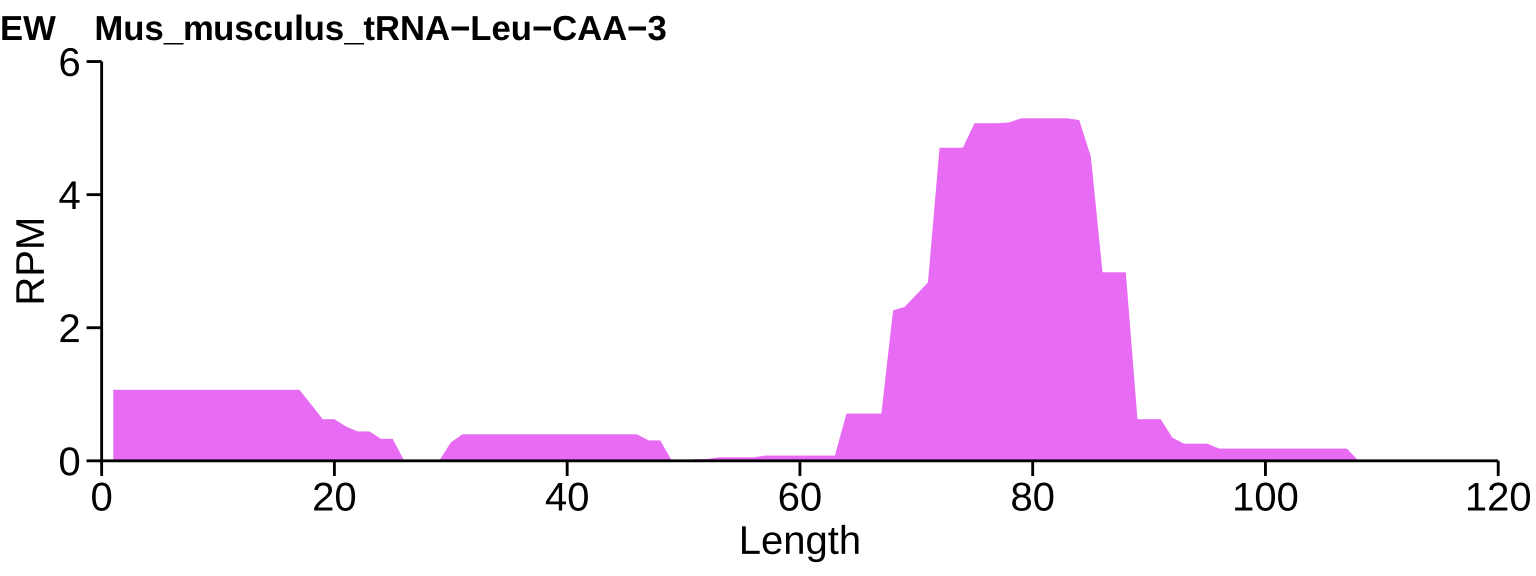

EW Mus_musculus_tRNA−Leu−CAA−3
RPM
6
4
2
0
0
20
40
60
80
100
120
Length

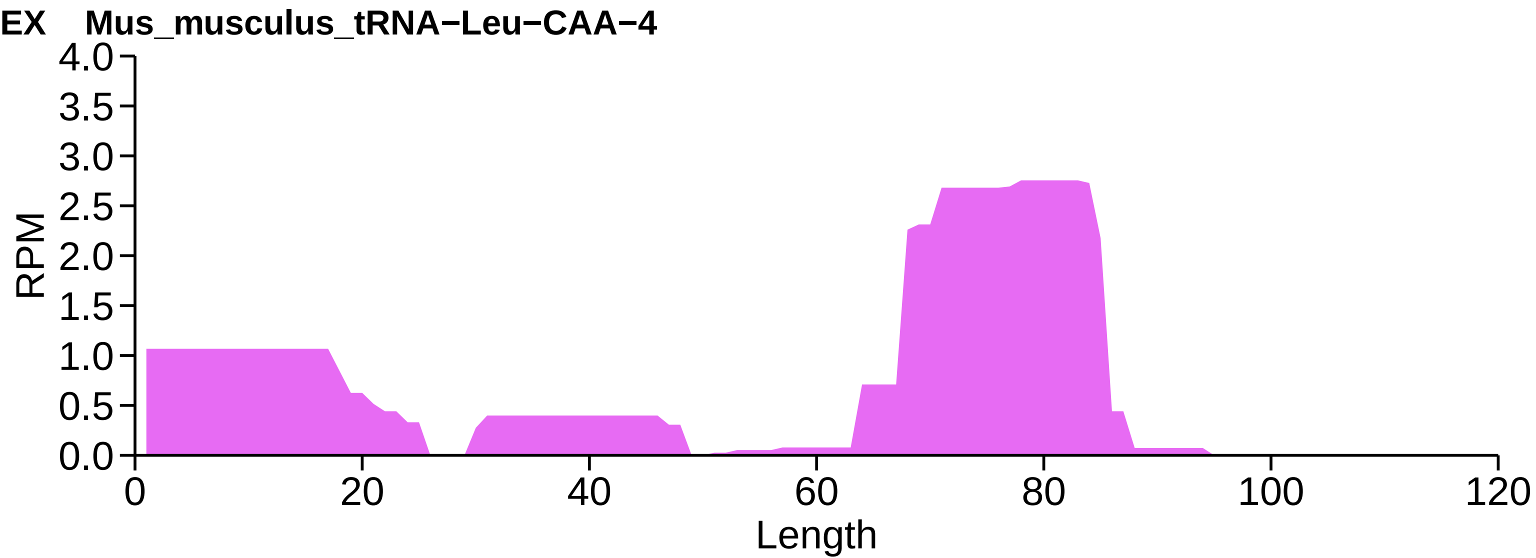
EX Mus_musculus_tRNA−Leu−CAA−4
RPM
4.0
3.5
3.0
2.5
2.0
1.5
1.0
0.5
0.0
0
20
40
60
80
100
120
Length

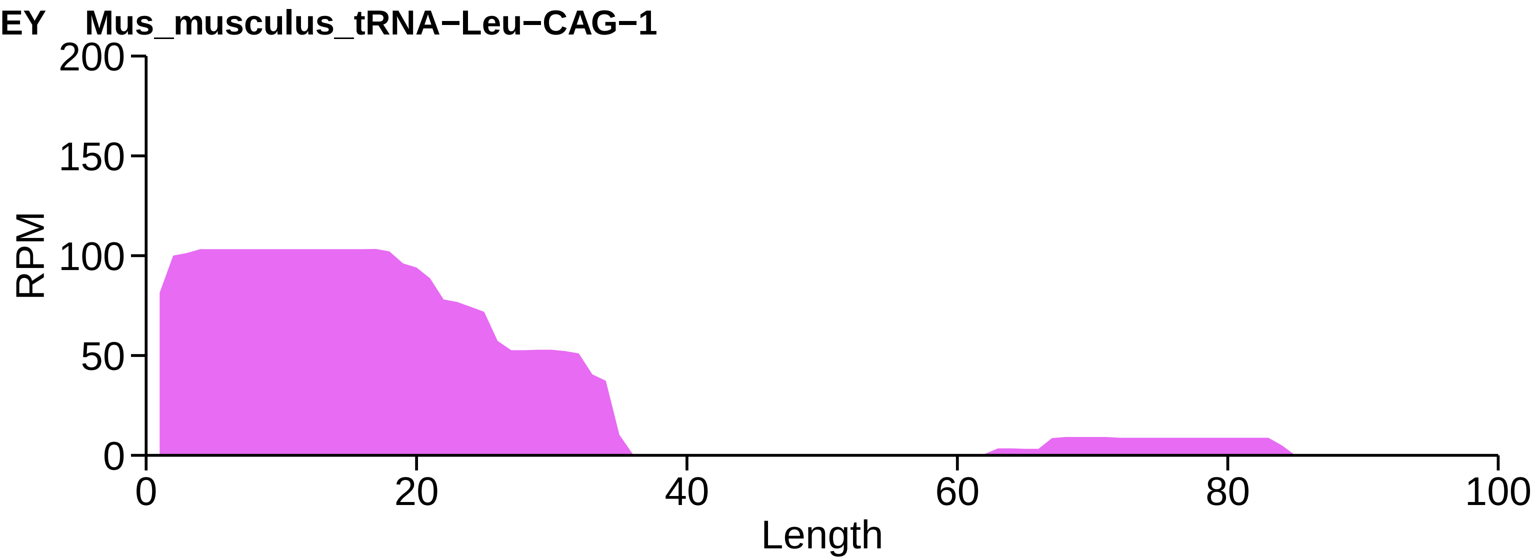
EY Mus_musculus_tRNA−Leu−CAG−1
200
150
100
50
0
RPM
0
20
40
60
80
100
Length

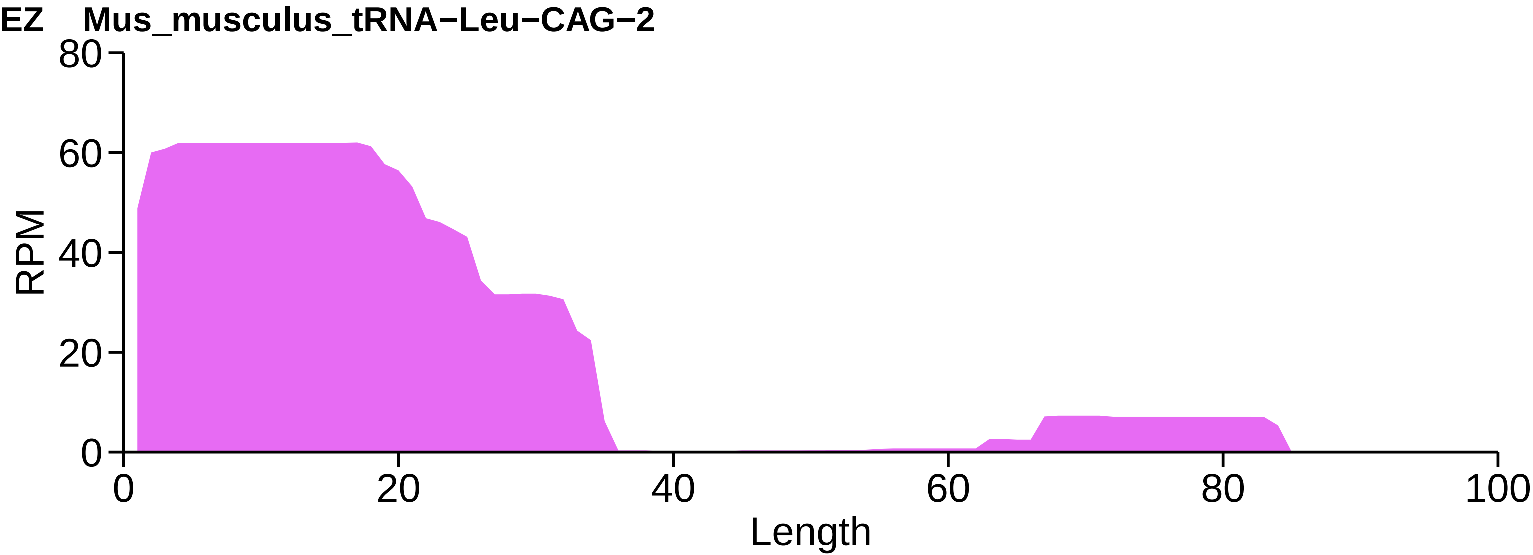
EZ Mus_musculus_tRNA−Leu−CAG−2
RPM
80
60
40
20
0
0
20
40
60
80
100
Length

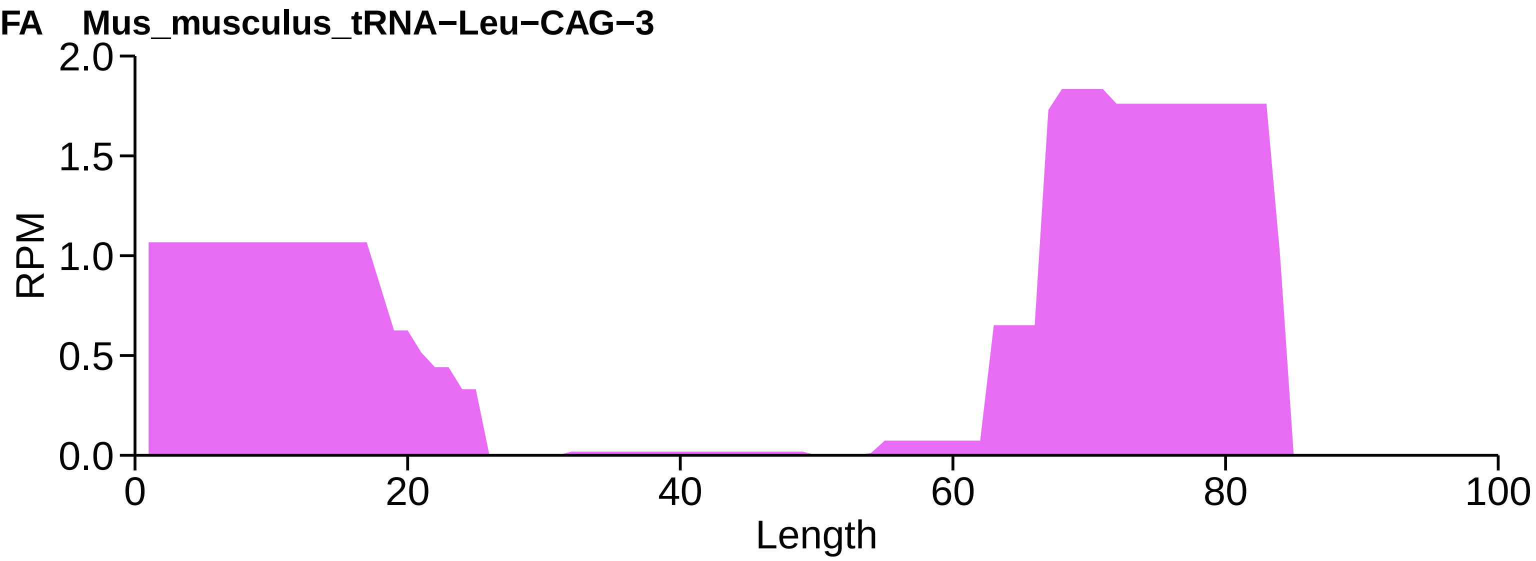
FA Mus_musculus_tRNA−Leu−CAG−3
RPM
2.0
1.5
1.0
0.5
0.0
0
20
40
60
80
100
Length

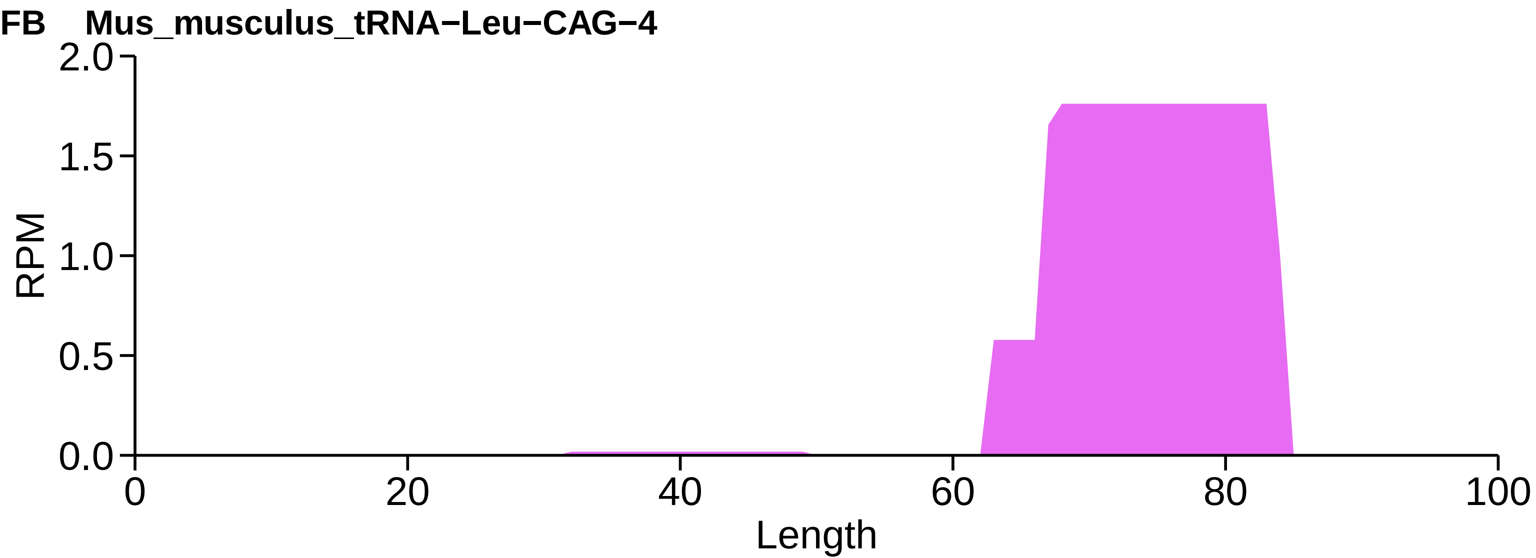
FB Mus_musculus_tRNA−Leu−CAG−4
RPM
2.0
1.5
1.0
0.5
0.0
0
20
40
60
80
100
Length

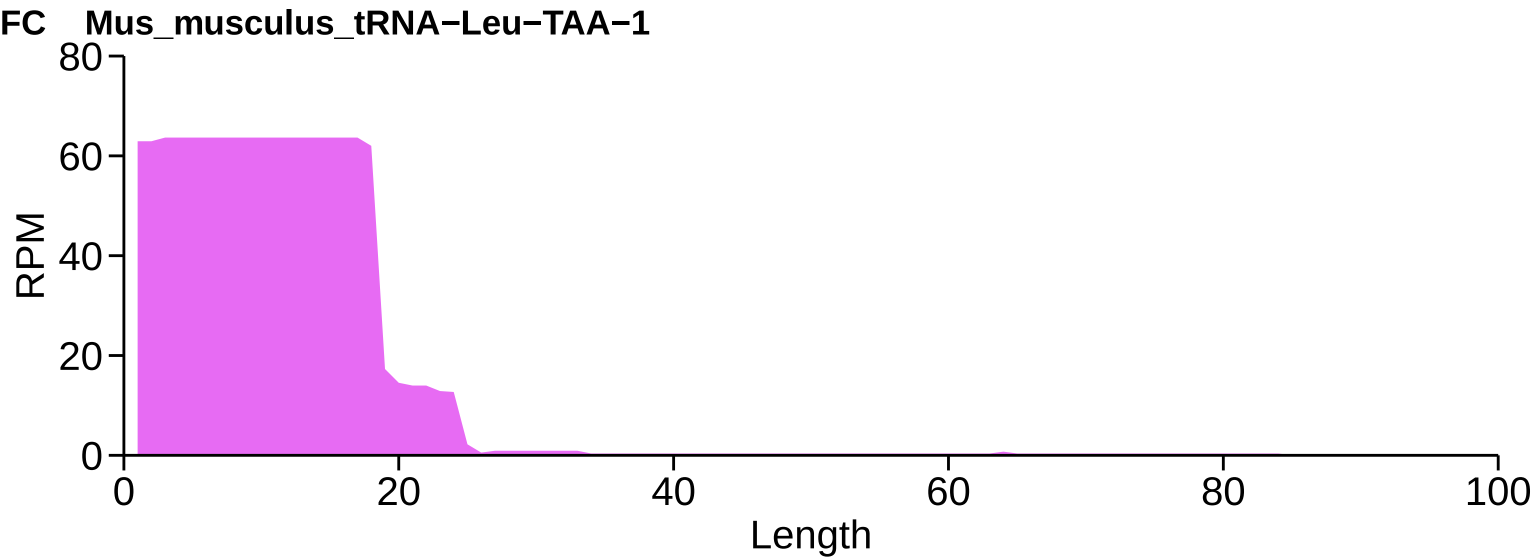
FC Mus_musculus_tRNA−Leu−TAA−1
RPM
Length
0
20
40
60
80
0
20
40
60
80
100

FD Mus_musculus_tRNA−Leu−TAA−2

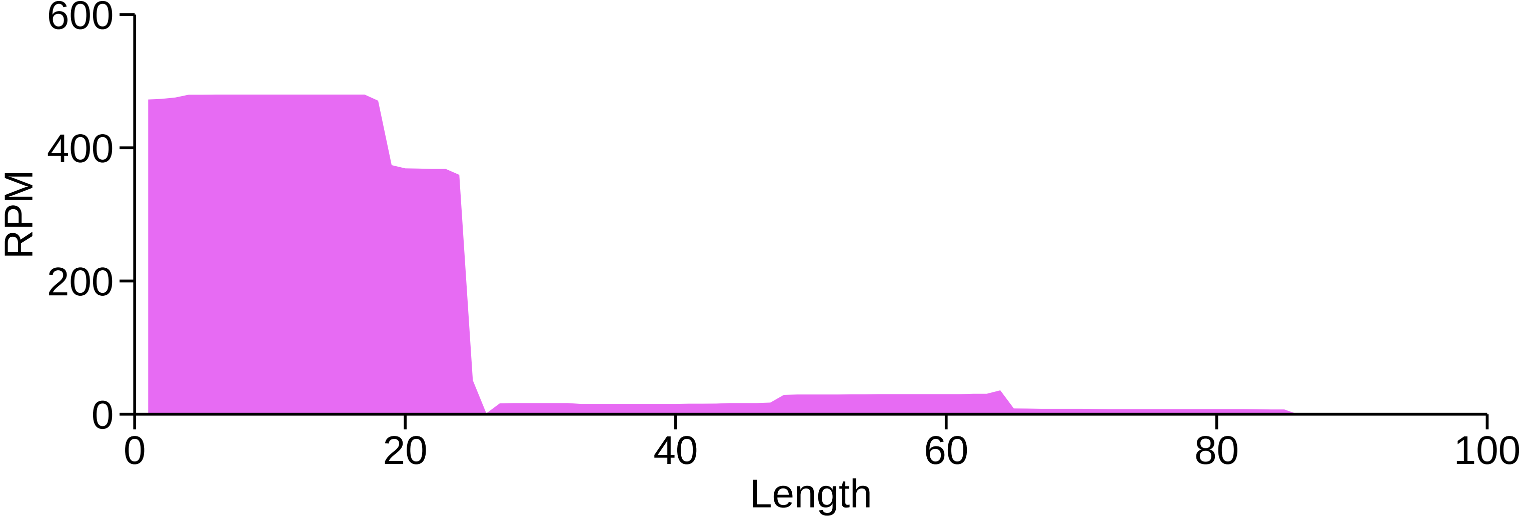

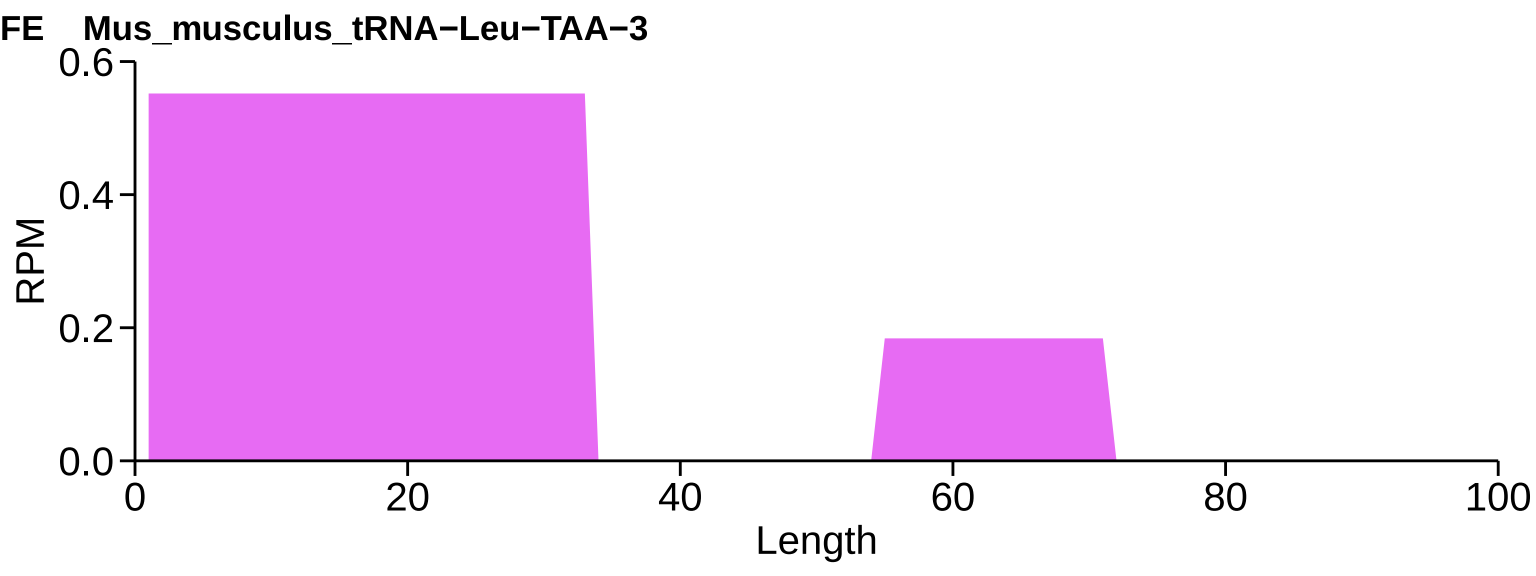
FE Mus_musculus_tRNA−Leu−TAA−3
RPM
0.0
0.2
0.4
0.6
0
20
40
60
80
100
Length

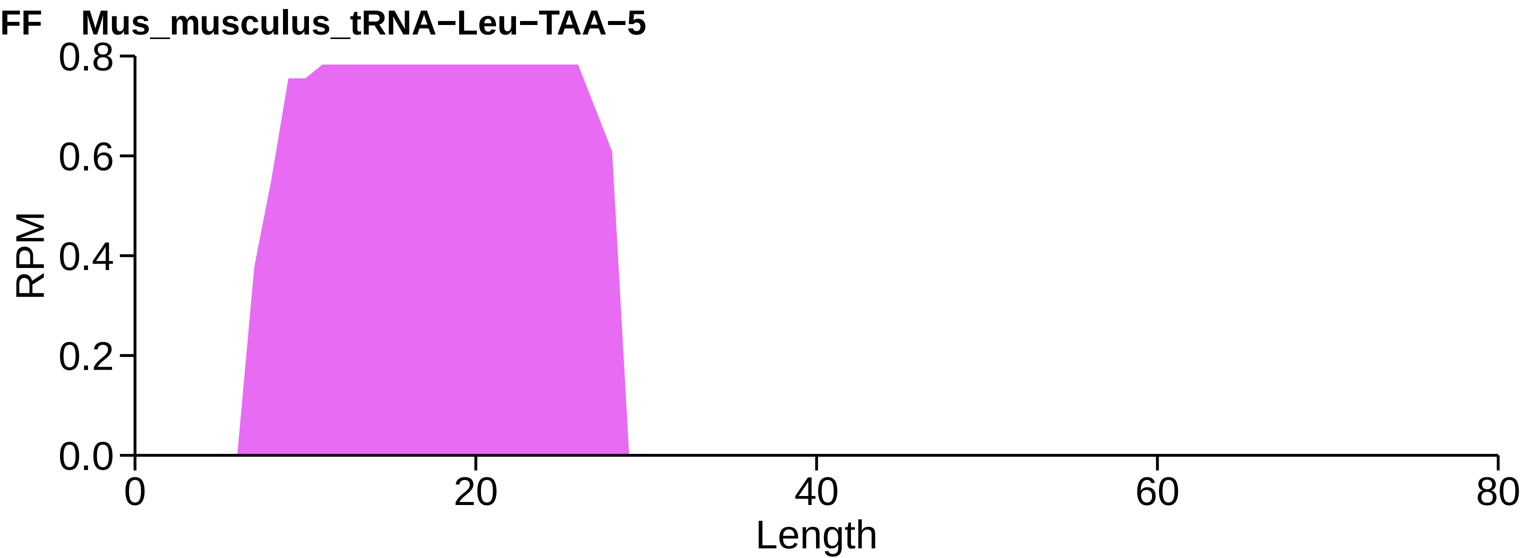

FF Mus_musculus_tRNA−Leu−TAA−5
RPM
0.0
0.2
0.4
0.6
0.8
0
20
40
60
80
Length

FG Mus_musculus_tRNA−Leu−TAG−1

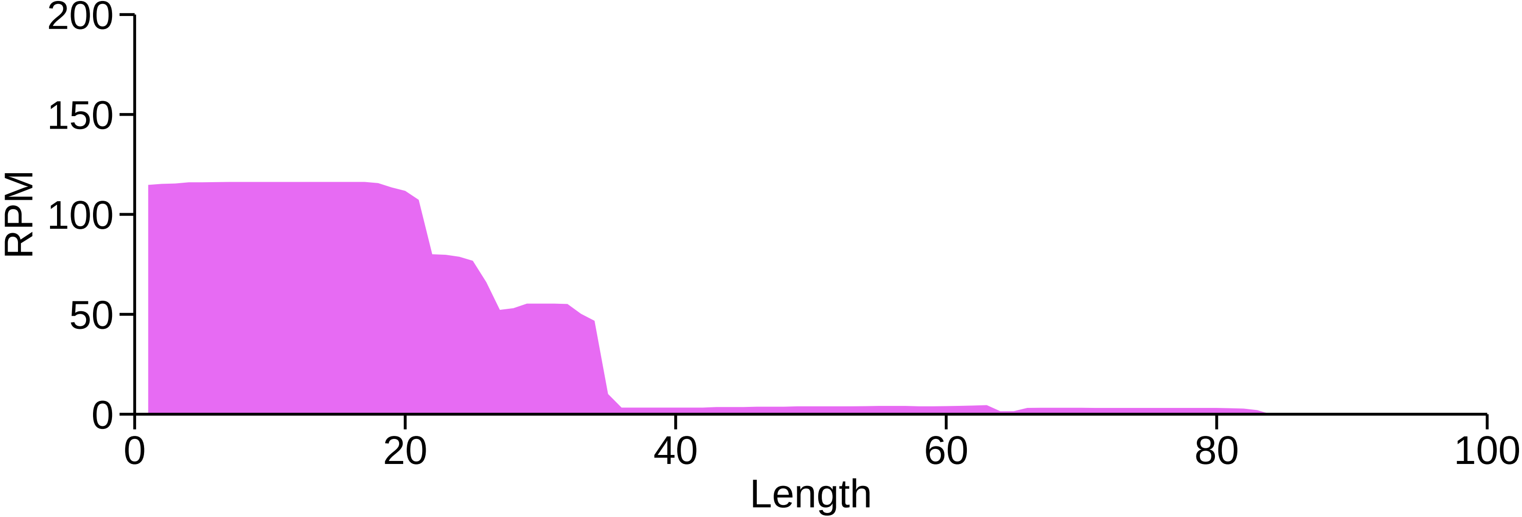

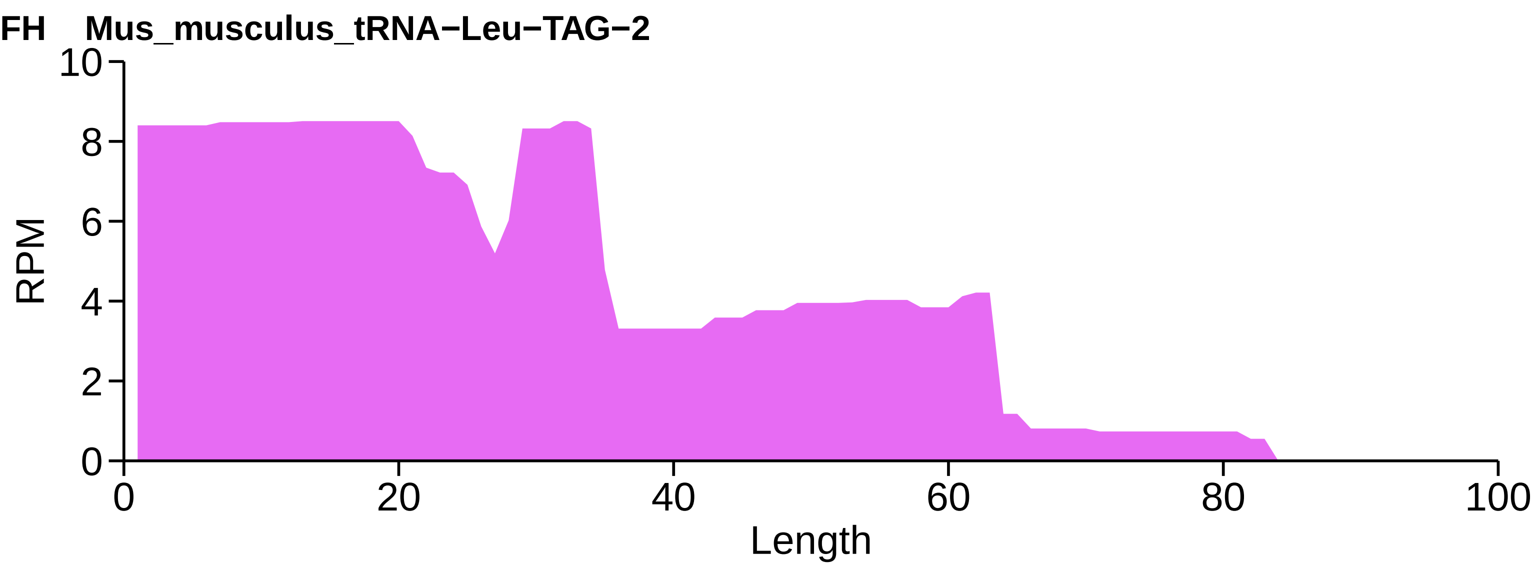

FH Mus_musculus_tRNA−Leu−TAG−2
RPM
Length
0
2
4
6
8
10
0
20
40
60
80
100

FI Mus_musculus_tRNA−Leu−TAG−3

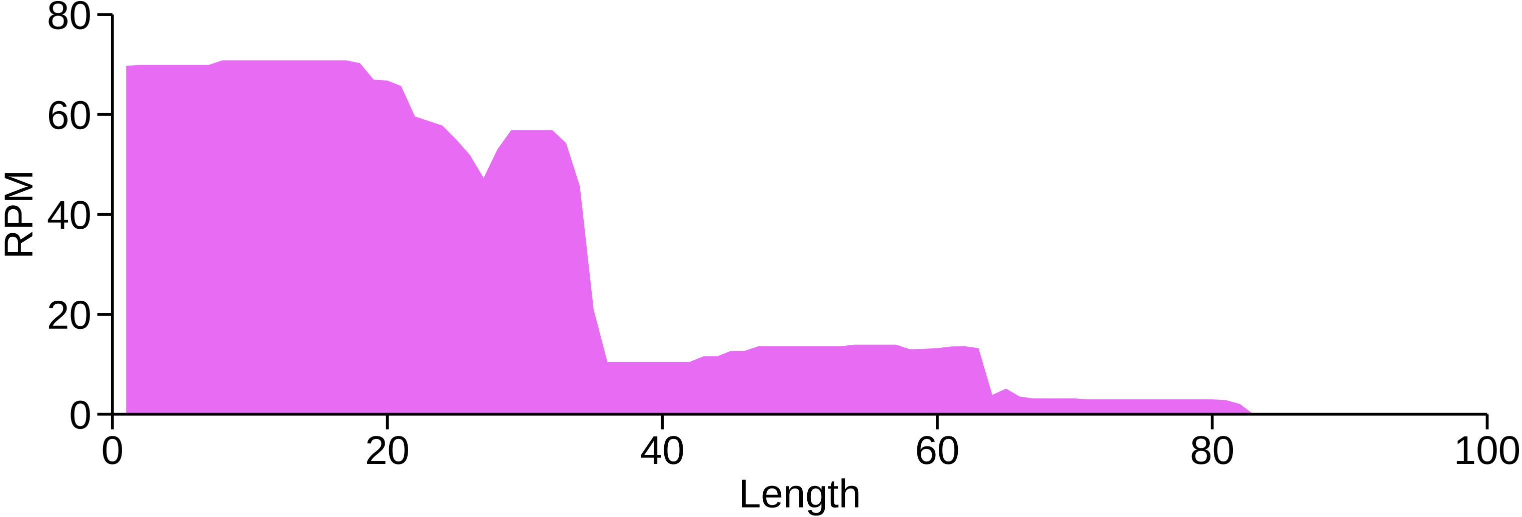

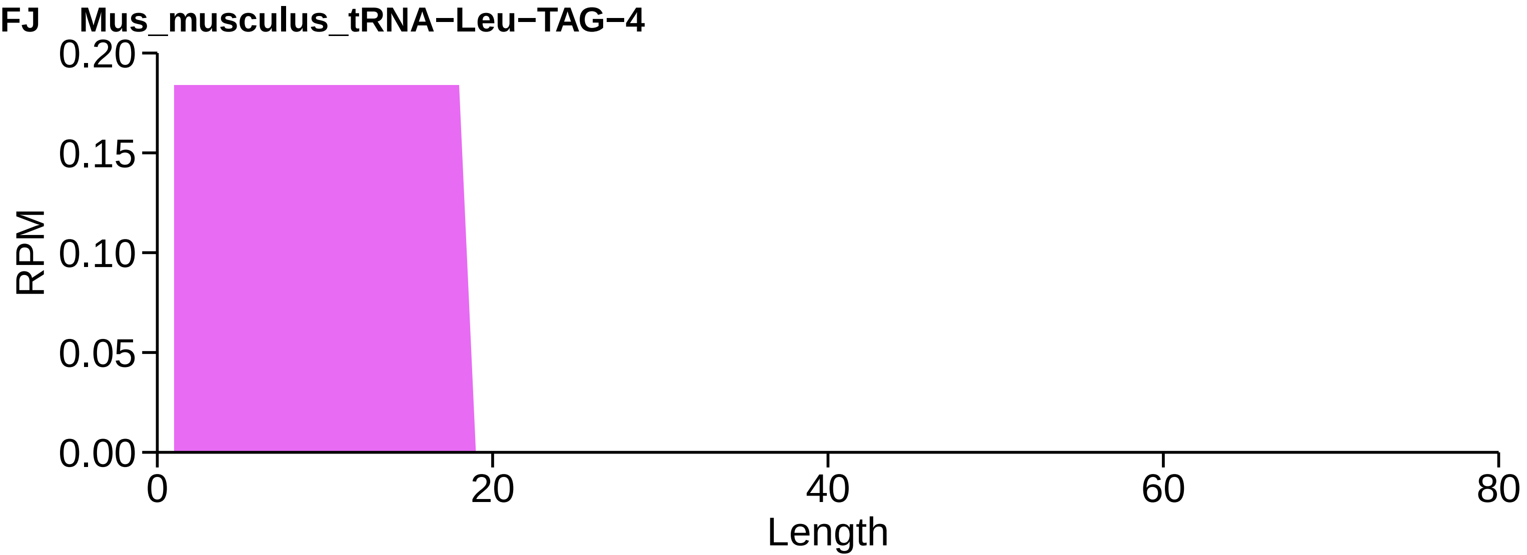
FJ Mus_musculus_tRNA−Leu−TAG−4
0.20
0.15
0.10
0.05
0.00
RPM
0
20
40
60
80
Length

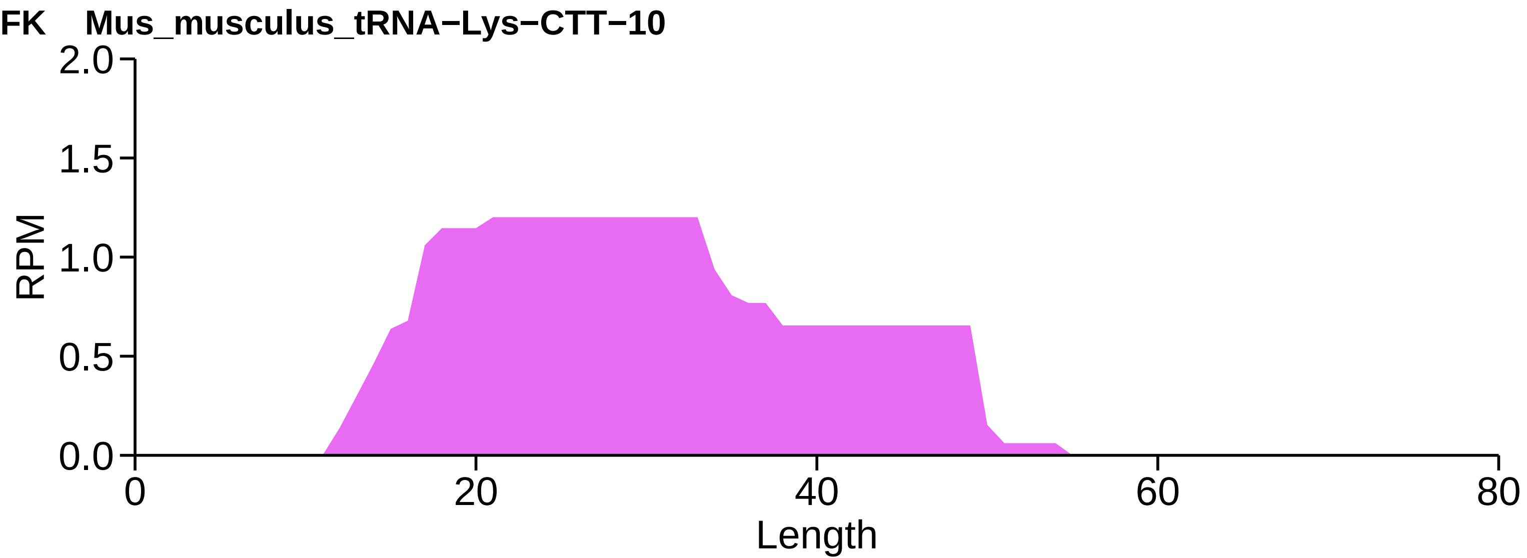
FK Mus_musculus_tRNA−Lys−CTT−10
2.0
1.5
1.0
0.5
0.0
RPM
0
20
40
60
80
Length

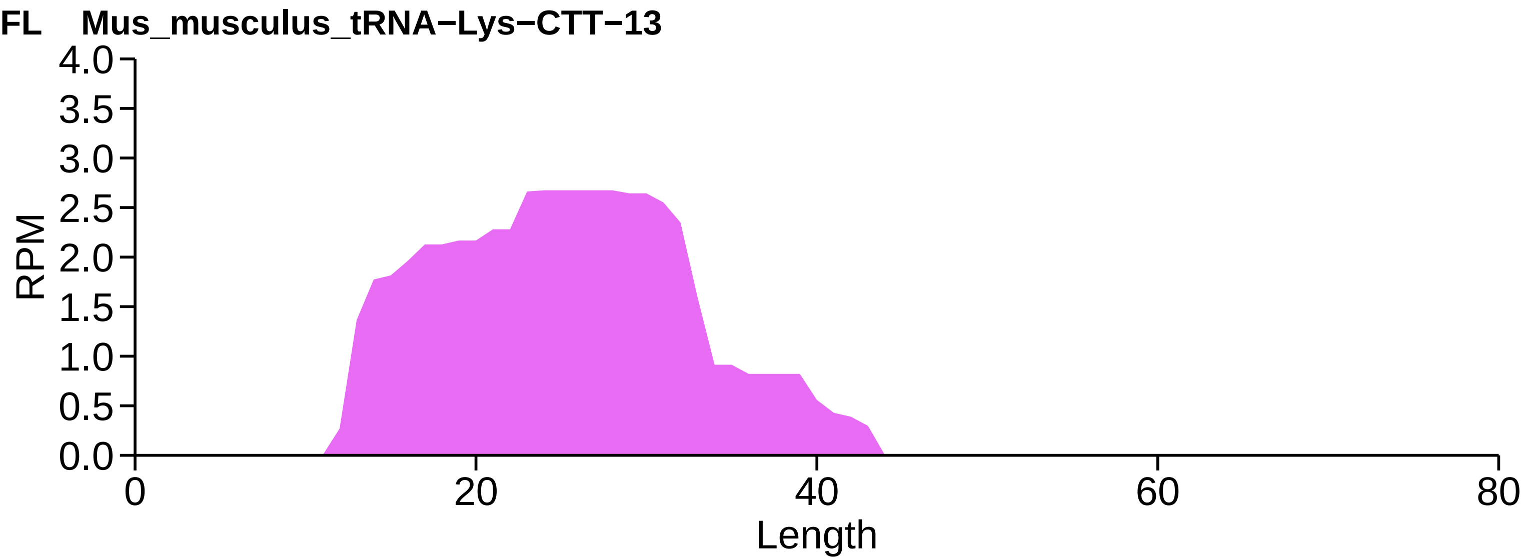

FL Mus_musculus_tRNA−Lys−CTT−13
RPM
4.0
3.5
3.0
2.5
2.0
1.5
1.0
0.5
0.0
0
20
40
60
80
Length

FM Mus_musculus_tRNA−Lys−CTT−15

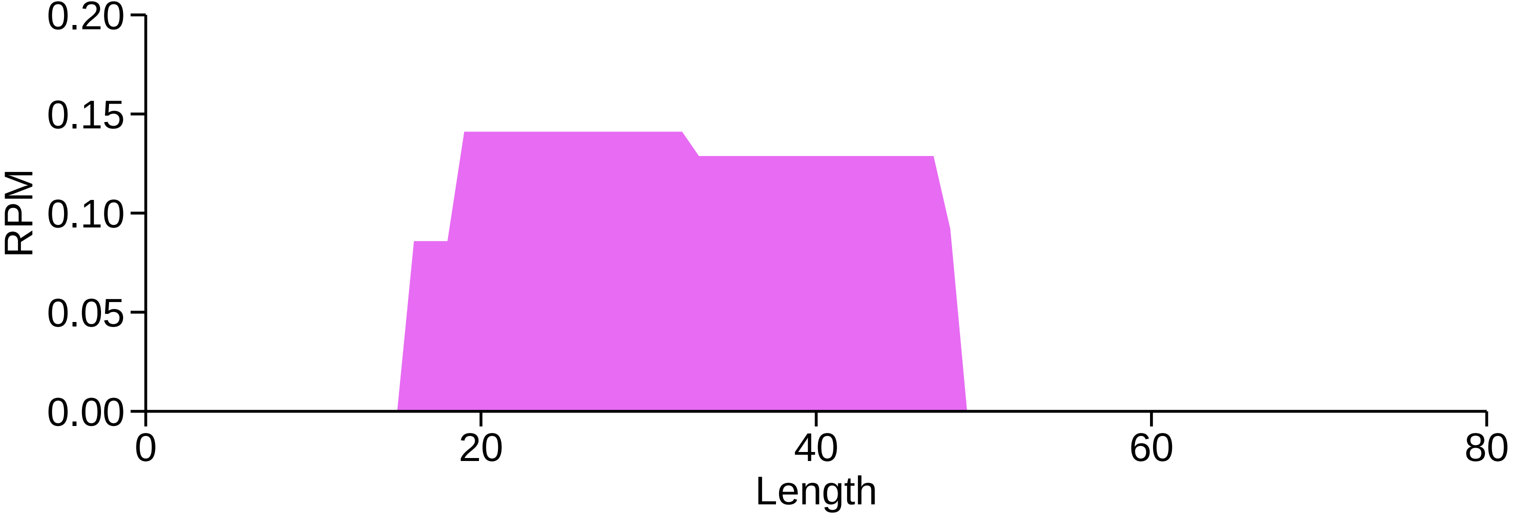

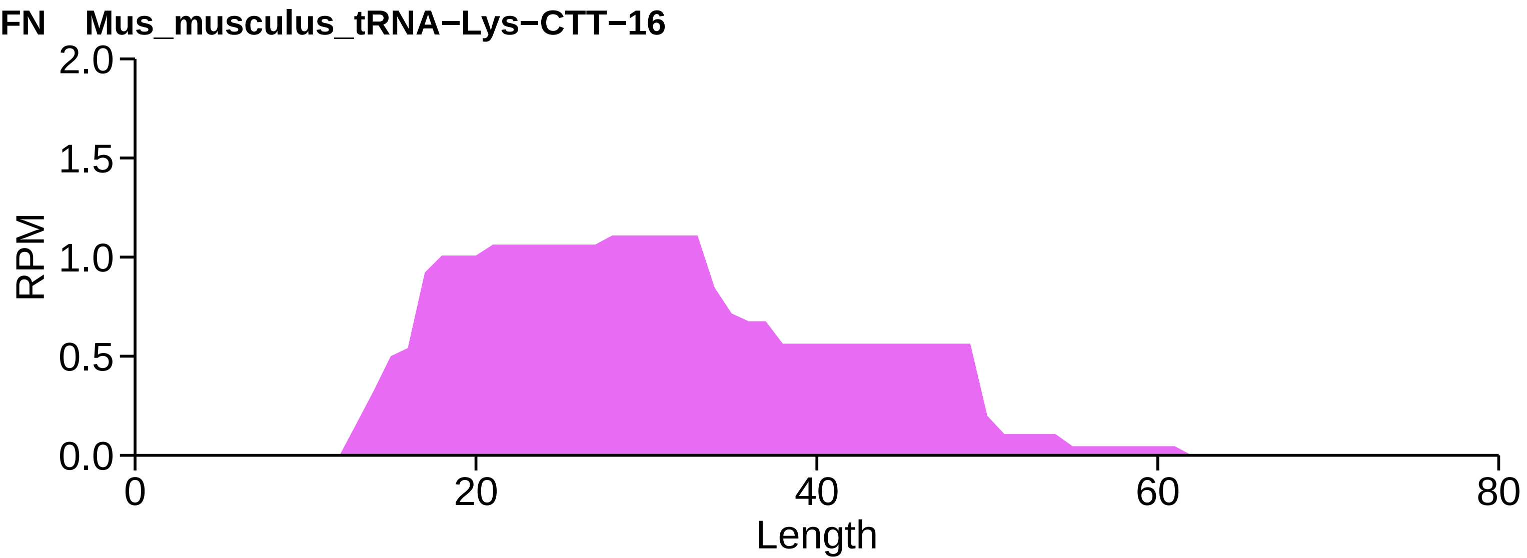

FN Mus_musculus_tRNA−Lys−CTT−16
RPM
2.0
1.5
1.0
0.5
0.0
0
20
40
60
80
Length

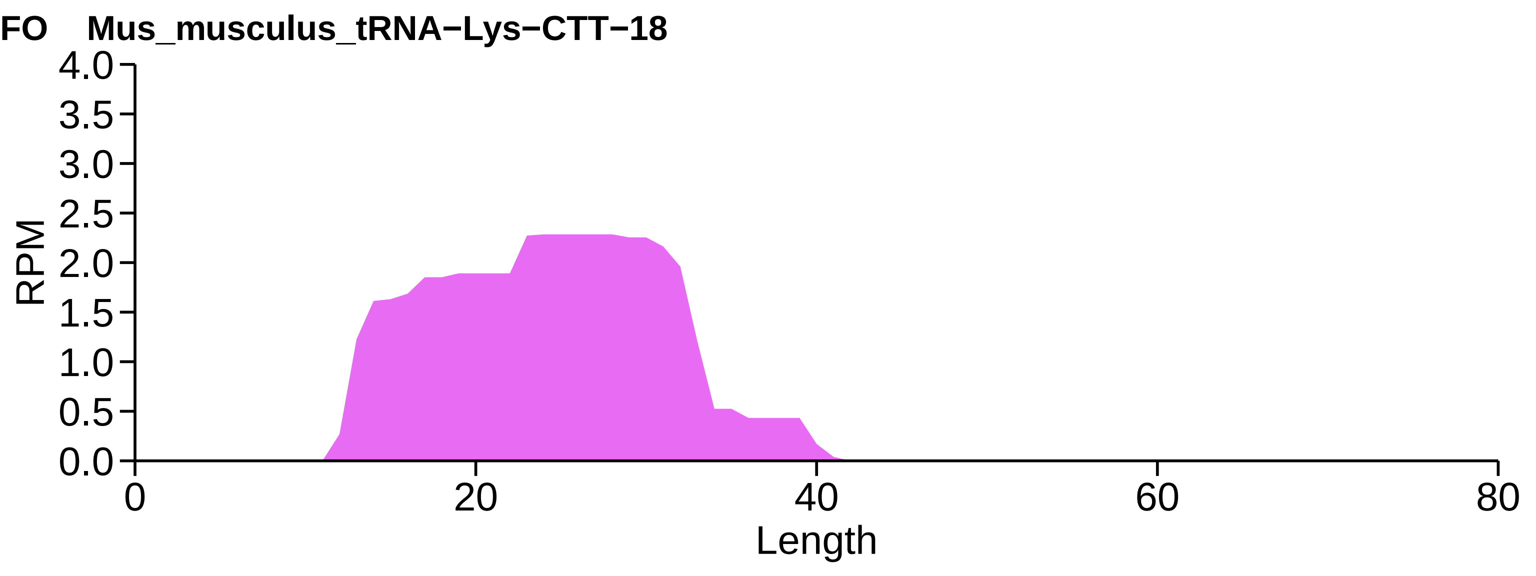
FO Mus_musculus_tRNA−Lys−CTT−18
RPM
4.0
3.5
3.0
2.5
2.0
1.5
1.0
0.5
0.0
0
20
40
60
80
Length

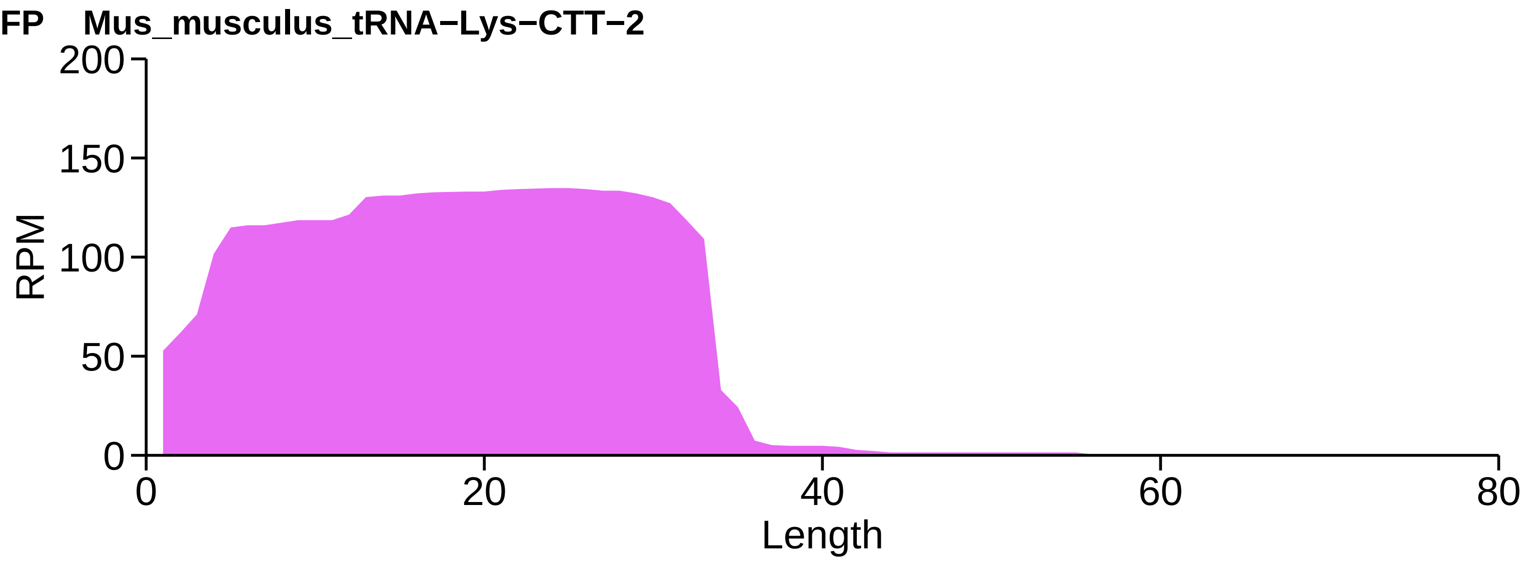
FP Mus_musculus_tRNA−Lys−CTT−2
RPM
200
150
100
50
0
0
20
40
60
80
Length

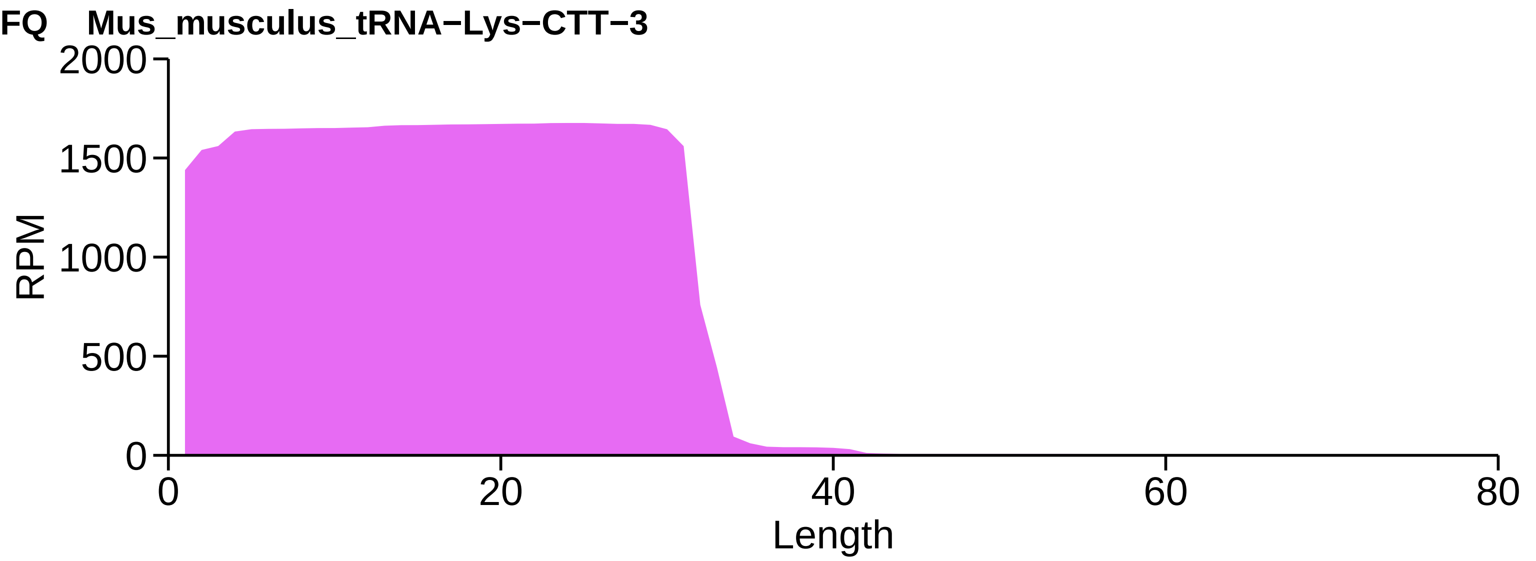

FQ Mus_musculus_tRNA−Lys−CTT−3
2000
1500
1000
500
0
RPM
0
20
40
60
80
Length

FR Mus_musculus_tRNA−Lys−CTT−4

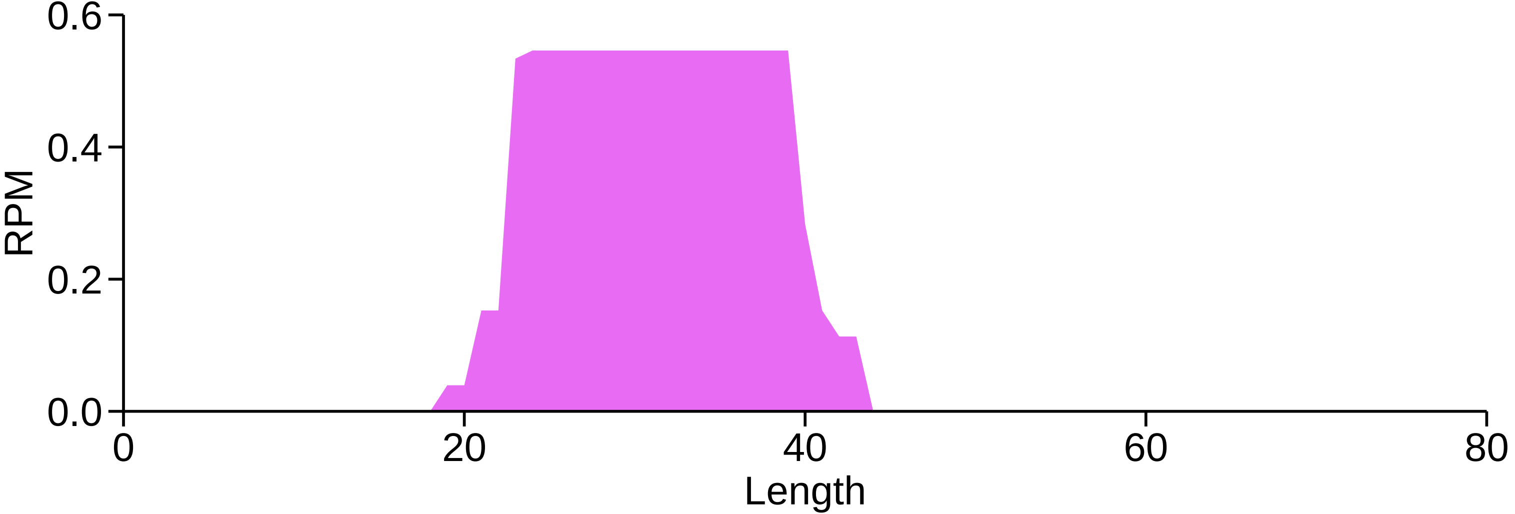

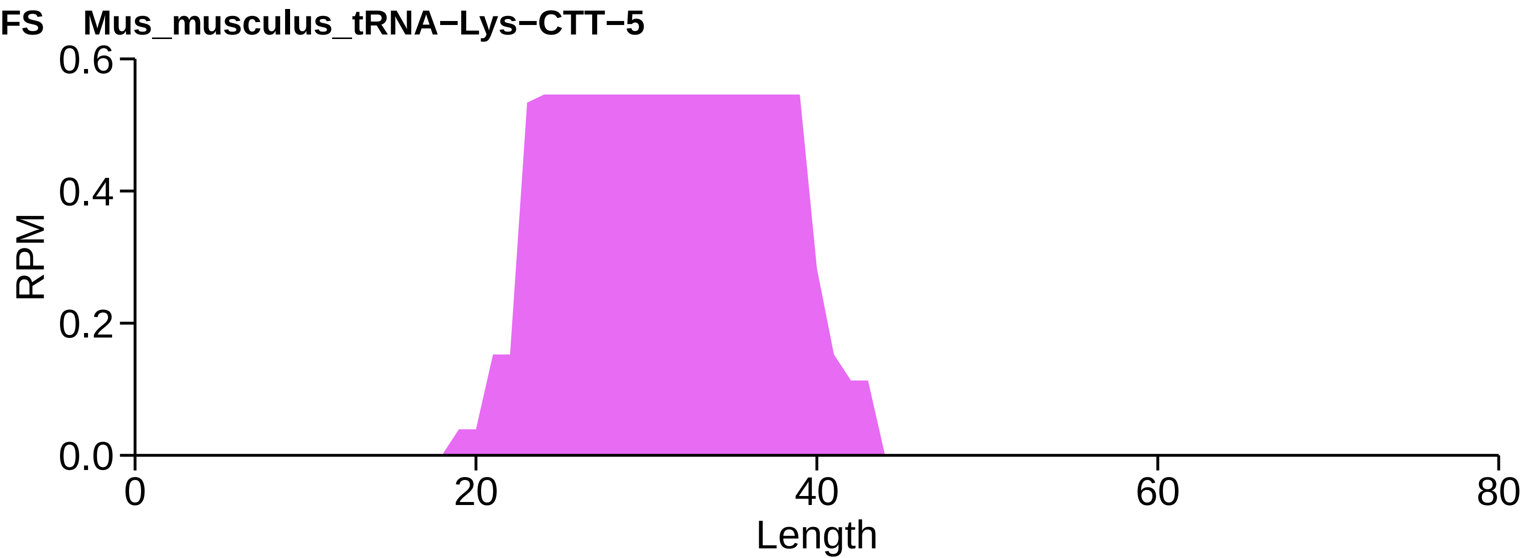

FS Mus_musculus_tRNA−Lys−CTT−5
0.6
0.4
0.2
0.0
RPM
0
20
40
60
80
Length

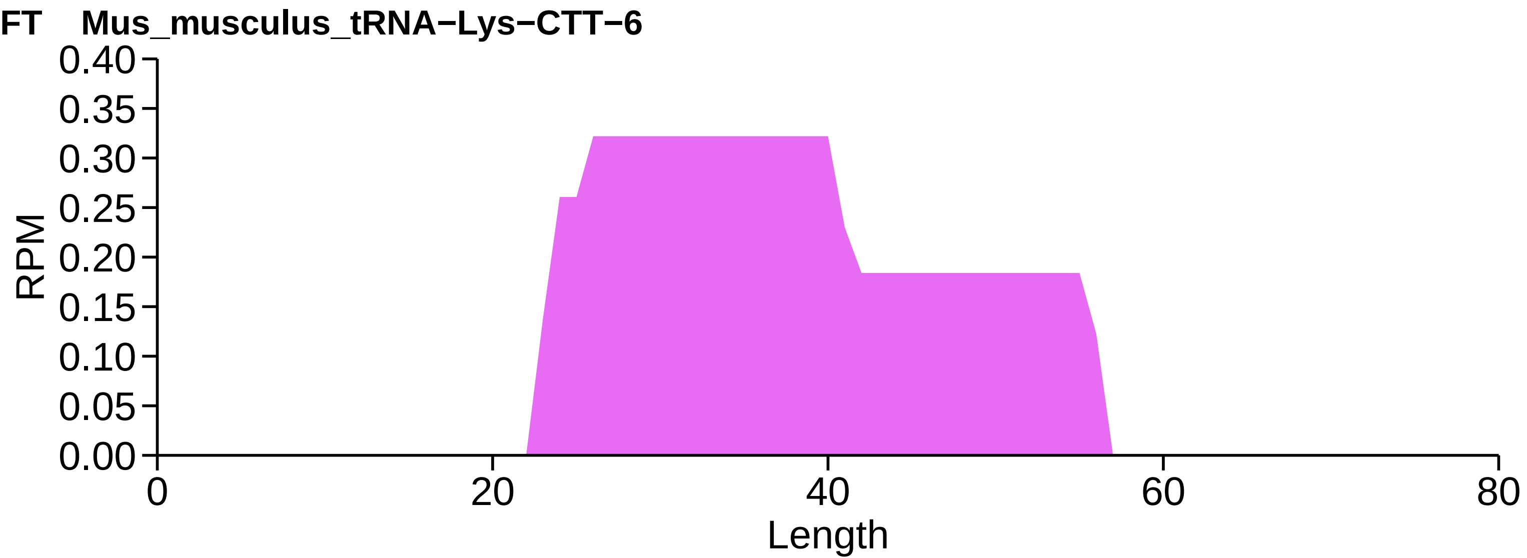
FT Mus_musculus_tRNA−Lys−CTT−6
RPM
0.00
0.05
0.10
0.15
0.20
0.25
0.30
0.35
0.40
0
20
40
60
80
Length

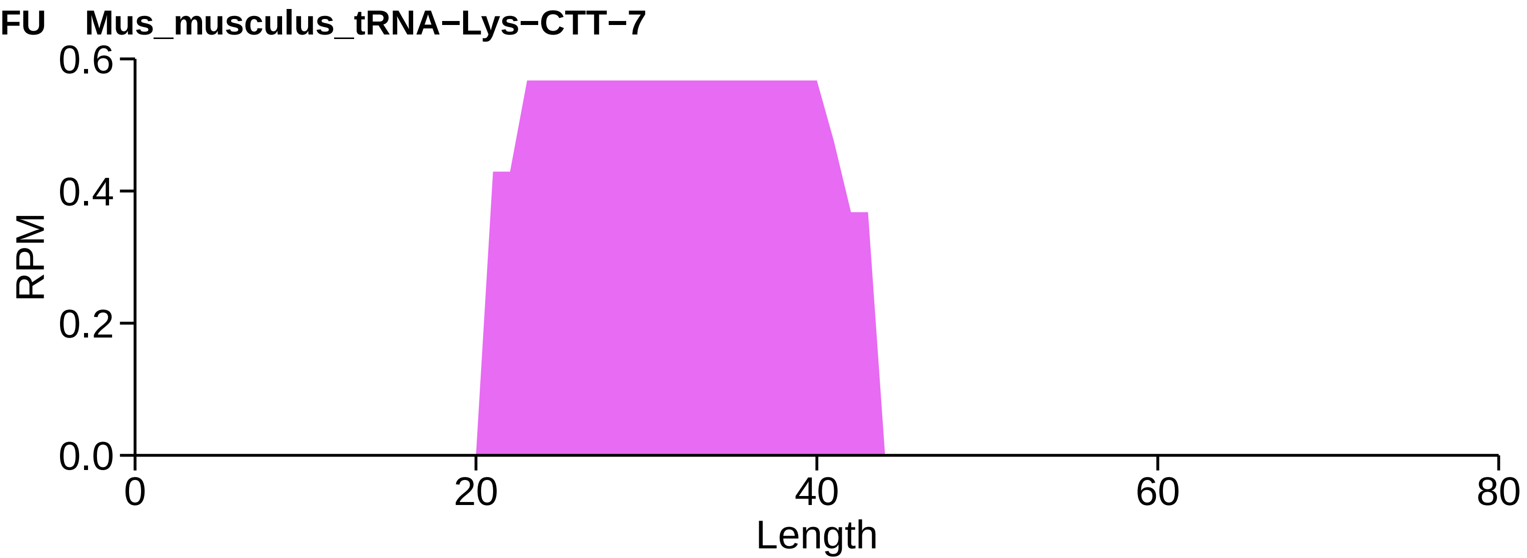
FU Mus_musculus_tRNA−Lys−CTT−7
RPM
0.0
0.2
0.4
0.6
0
20
40
60
80
Length

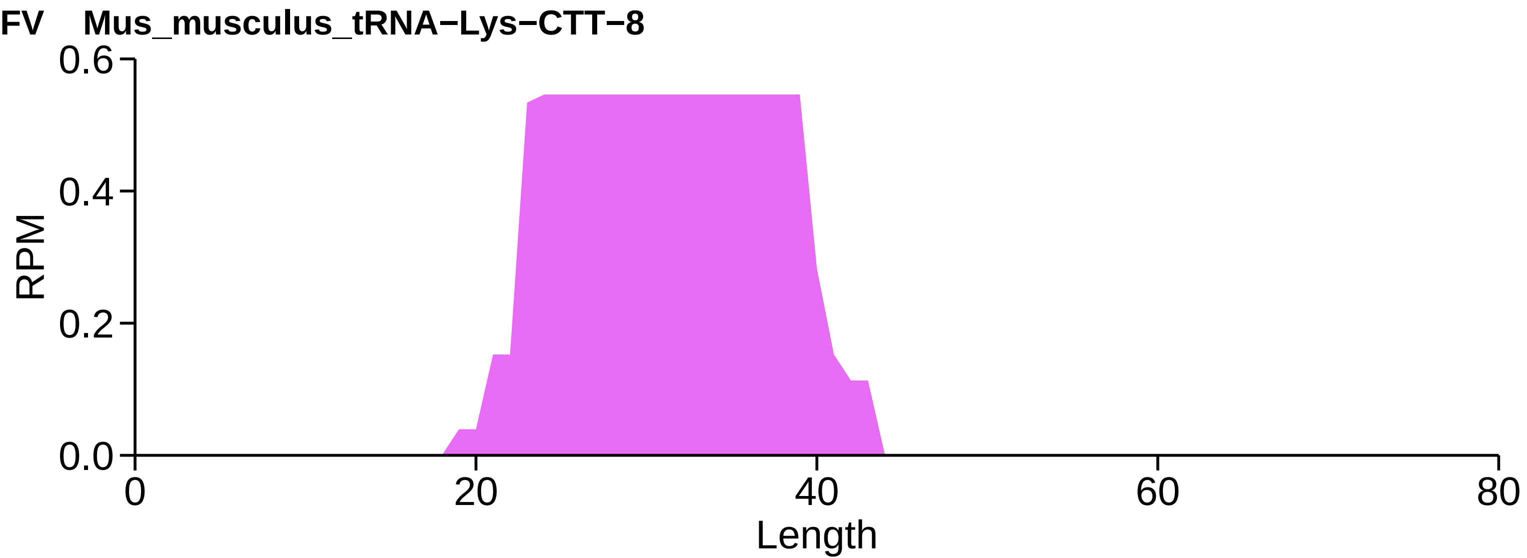
FV Mus_musculus_tRNA−Lys−CTT−8
0.6
0.4
0.2
0.0
RPM
0
20
40
60
80
Length

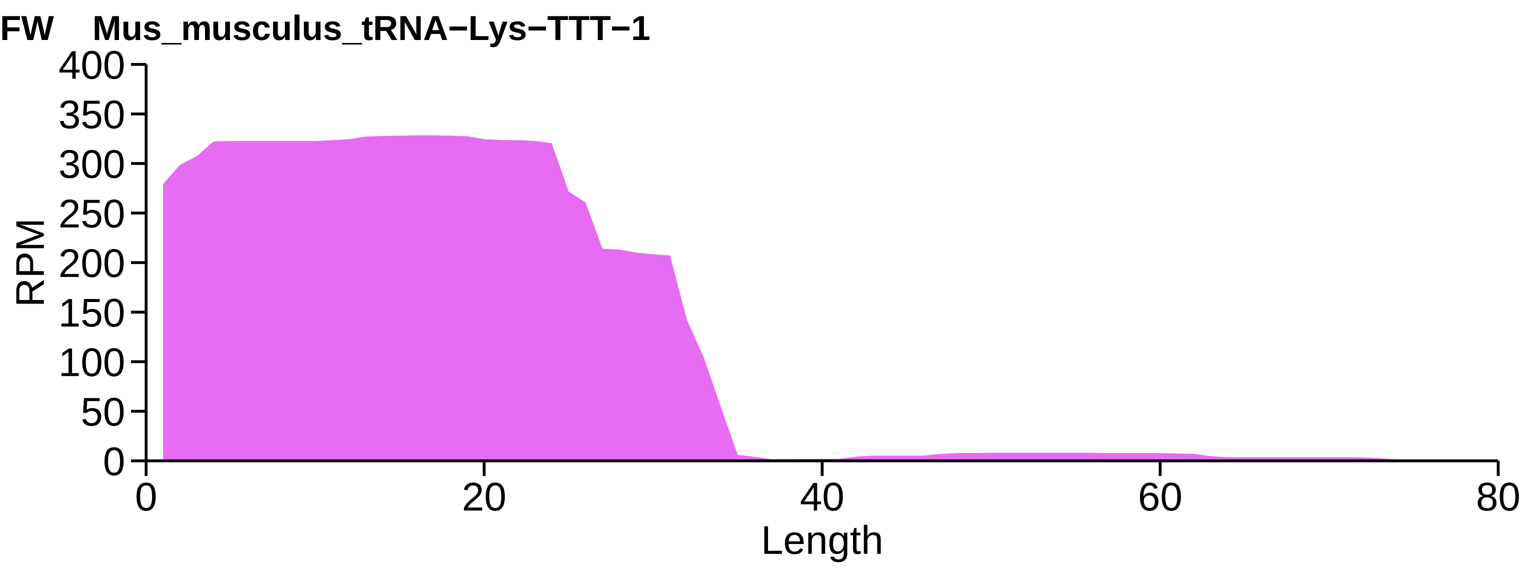

FW Mus_musculus_tRNA−Lys−TTT−1
RPM
400
350
300
250
200
150
100
50
0
0
20
40
60
80
Length

FX Mus_musculus_tRNA−Lys−TTT−2

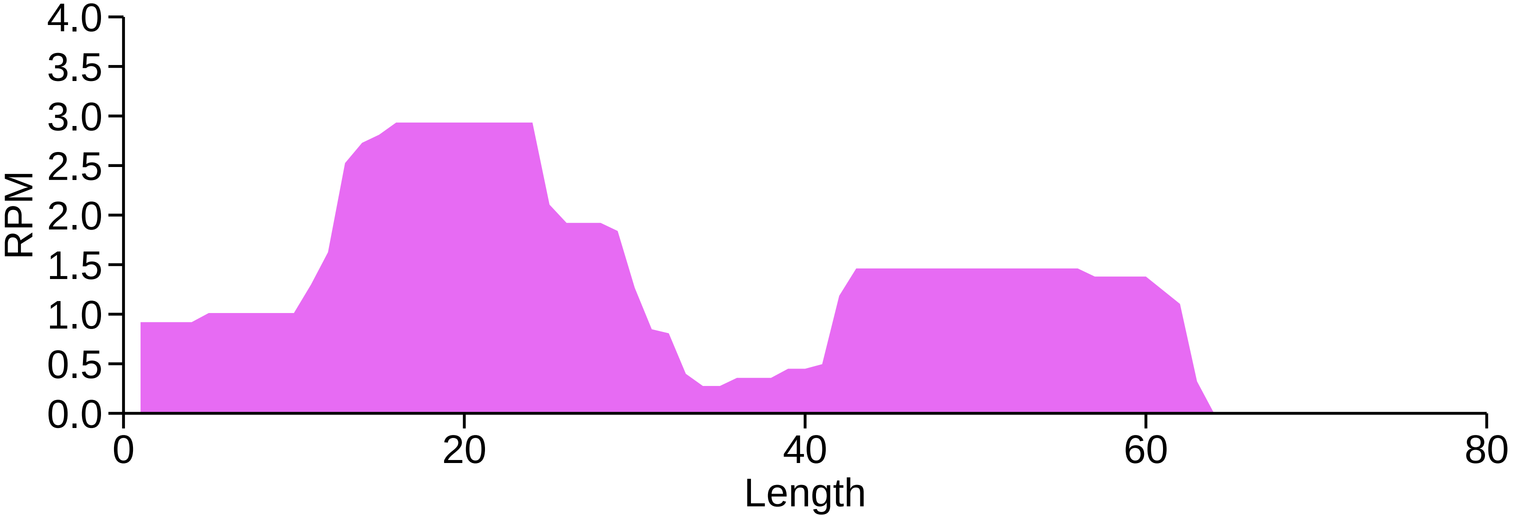

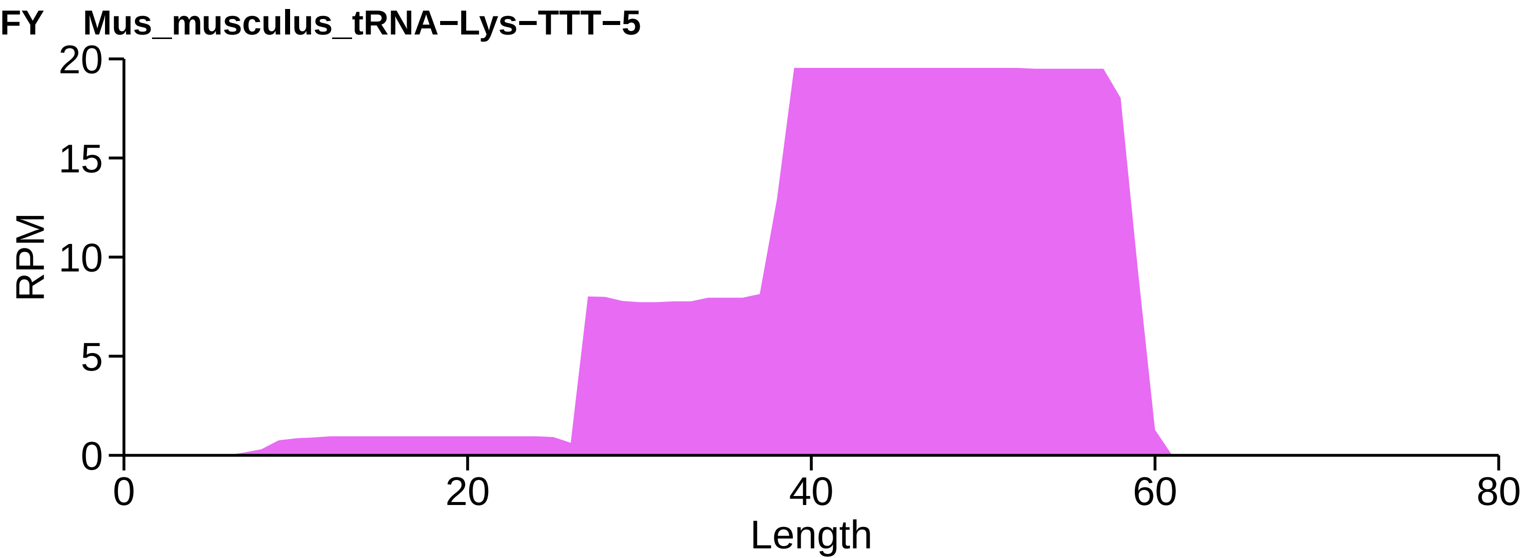

FY Mus_musculus_tRNA−Lys−TTT−5
RPM
20
15
10
5
0
0
20
40
60
80
Length

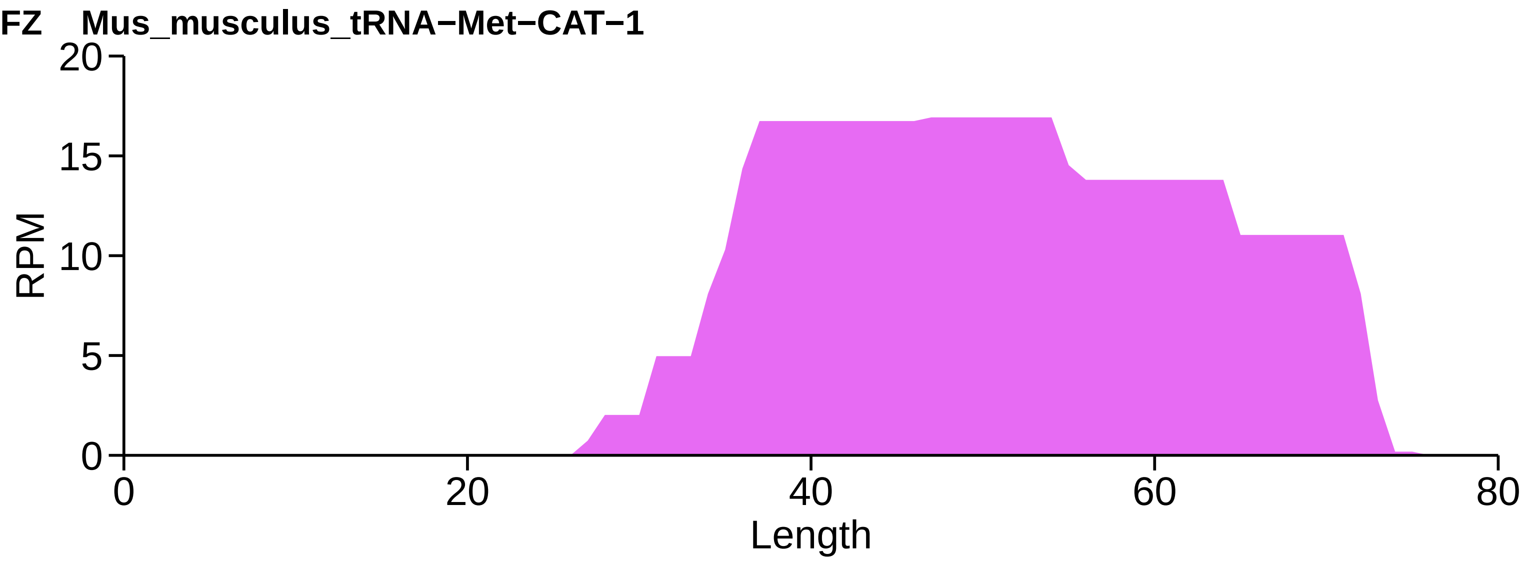

FZ Mus_musculus_tRNA−Met−CAT−1
RPM
20
15
10
5
0
0
20
40
60
80
Length

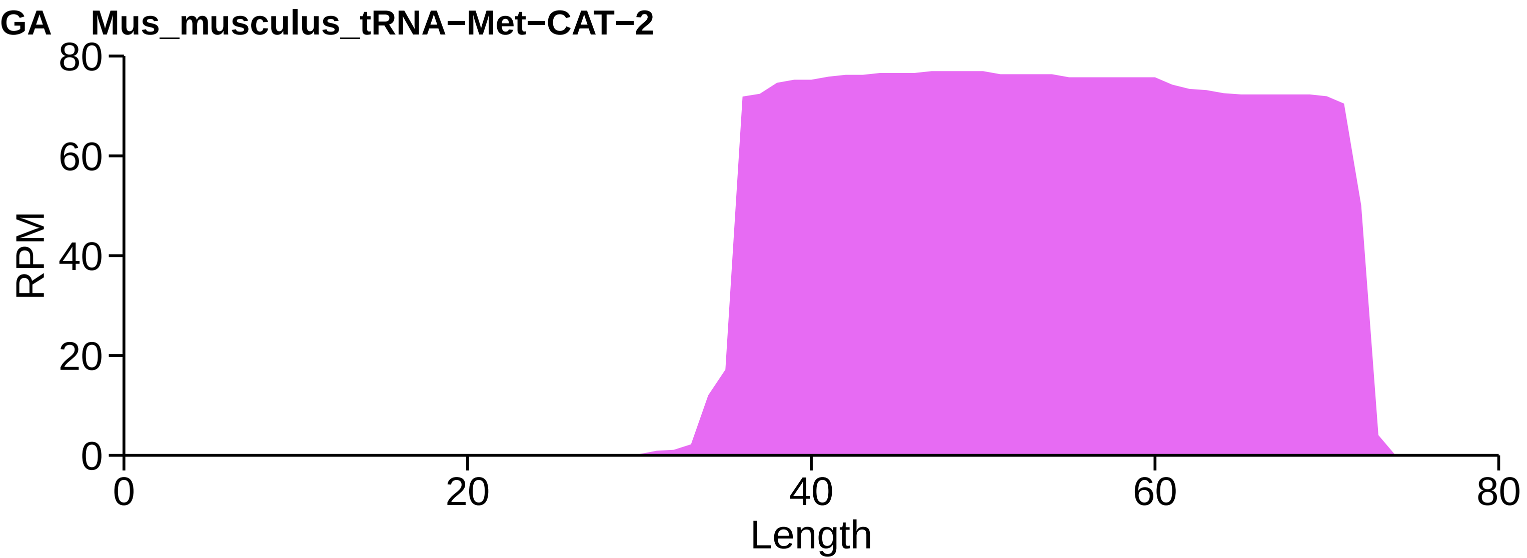
GA Mus_musculus_tRNA−Met−CAT−2
RPM
80
60
40
20
0
0
20
40
60
80
Length

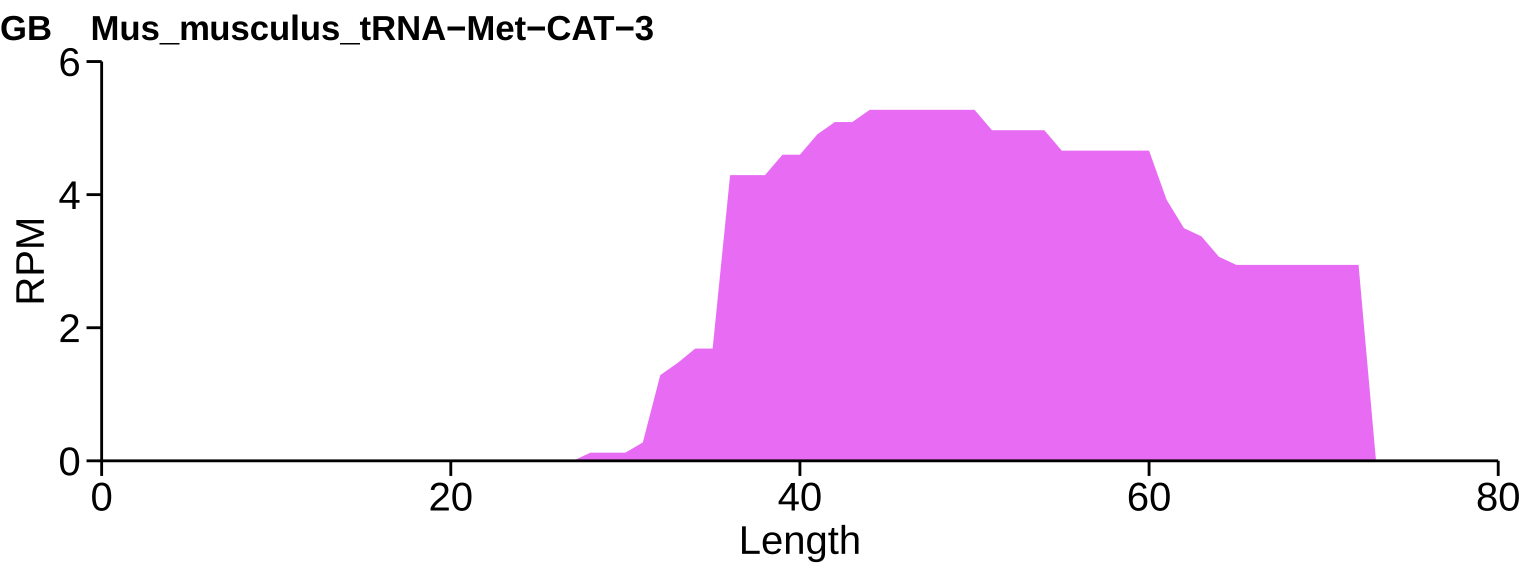
GB Mus_musculus_tRNA−Met−CAT−3
RPM
0
2
4
6
0
20
40
60
80
Length

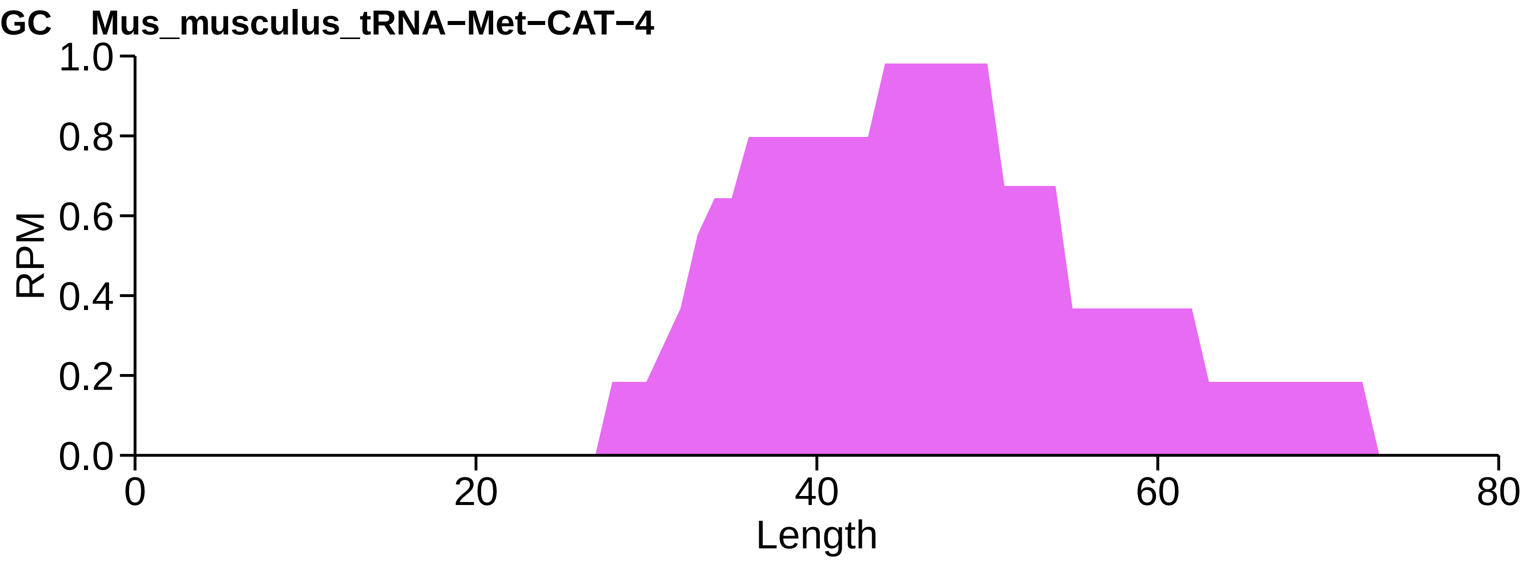

GC Mus_musculus_tRNA−Met−CAT−4
RPM
1.0
0.8
0.6
0.4
0.2
0.0
0
20
40
60
80
Length

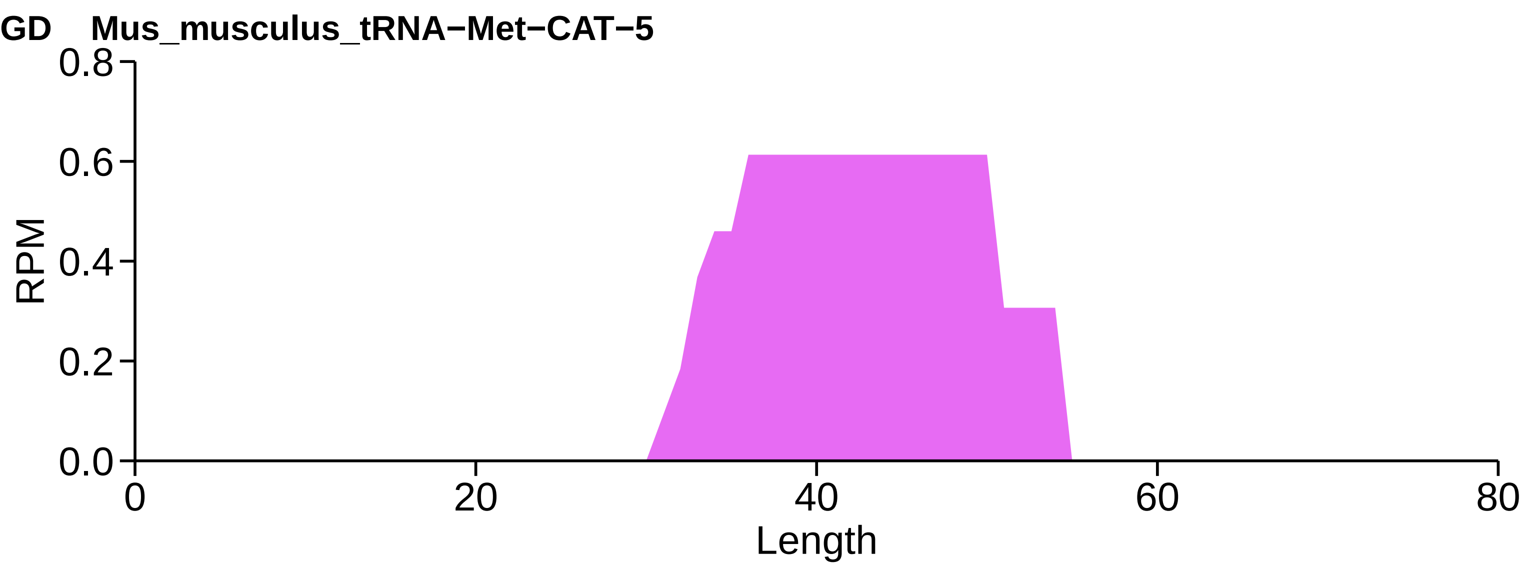
GD Mus_musculus_tRNA−Met−CAT−5
RPM
0.0
0.2
0.4
0.6
0.8
0
20
40
60
80
Length

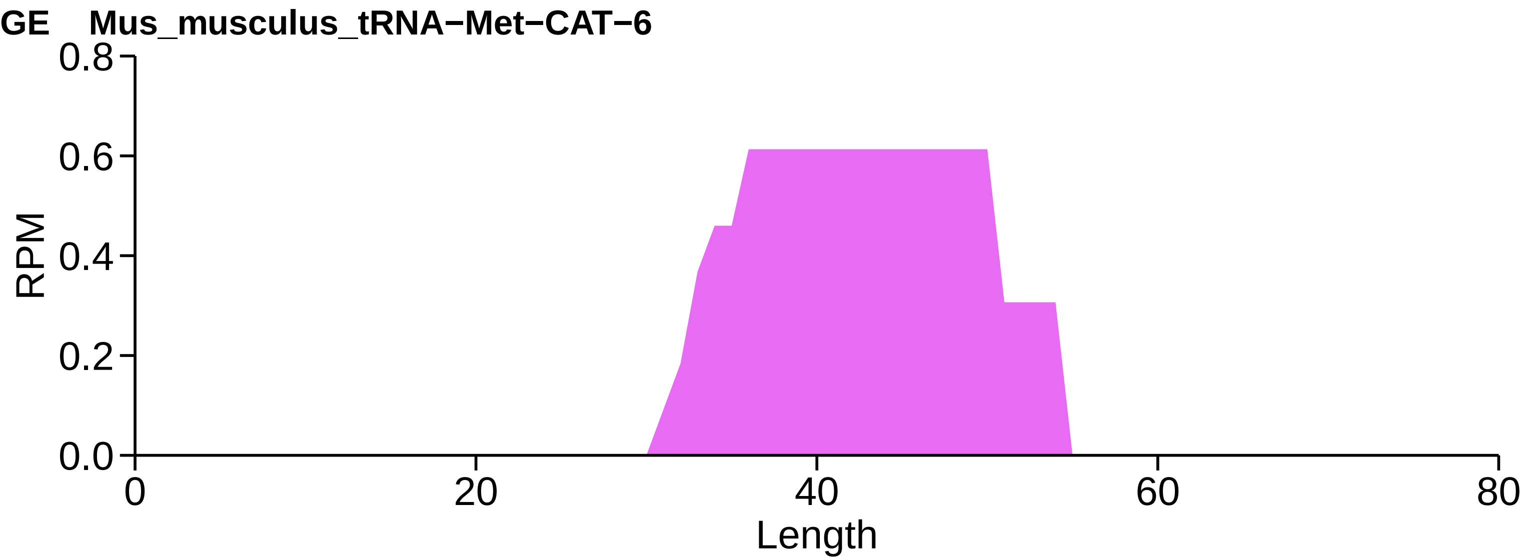
GE Mus_musculus_tRNA−Met−CAT−6
0.8
0.6
0.4
0.2
0.0
RPM
0
20
40
60
80
Length

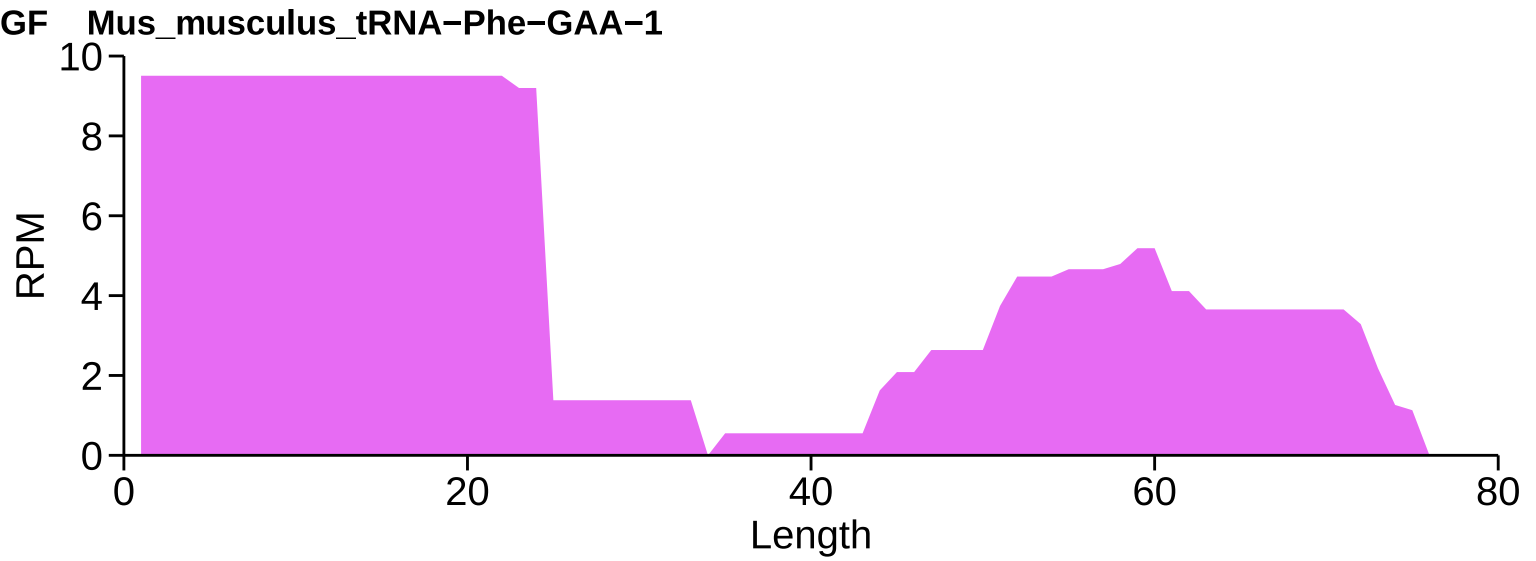

GF Mus_musculus_tRNA−Phe−GAA−1
RPM
Length
0
2
4
6
8
10
0
20
40
60
80

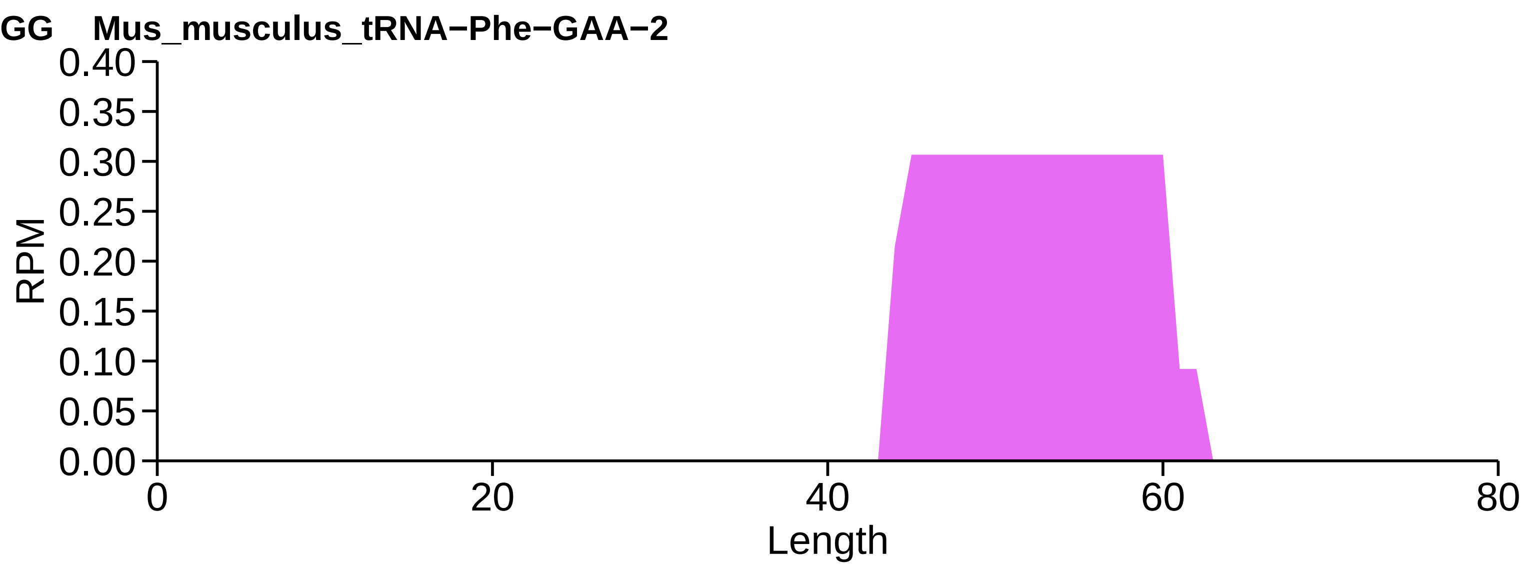
GG Mus_musculus_tRNA−Phe−GAA−2
RPM
0.40
0.35
0.30
0.25
0.20
0.15
0.10
0.05
0.00
0
20
40
60
80
Length

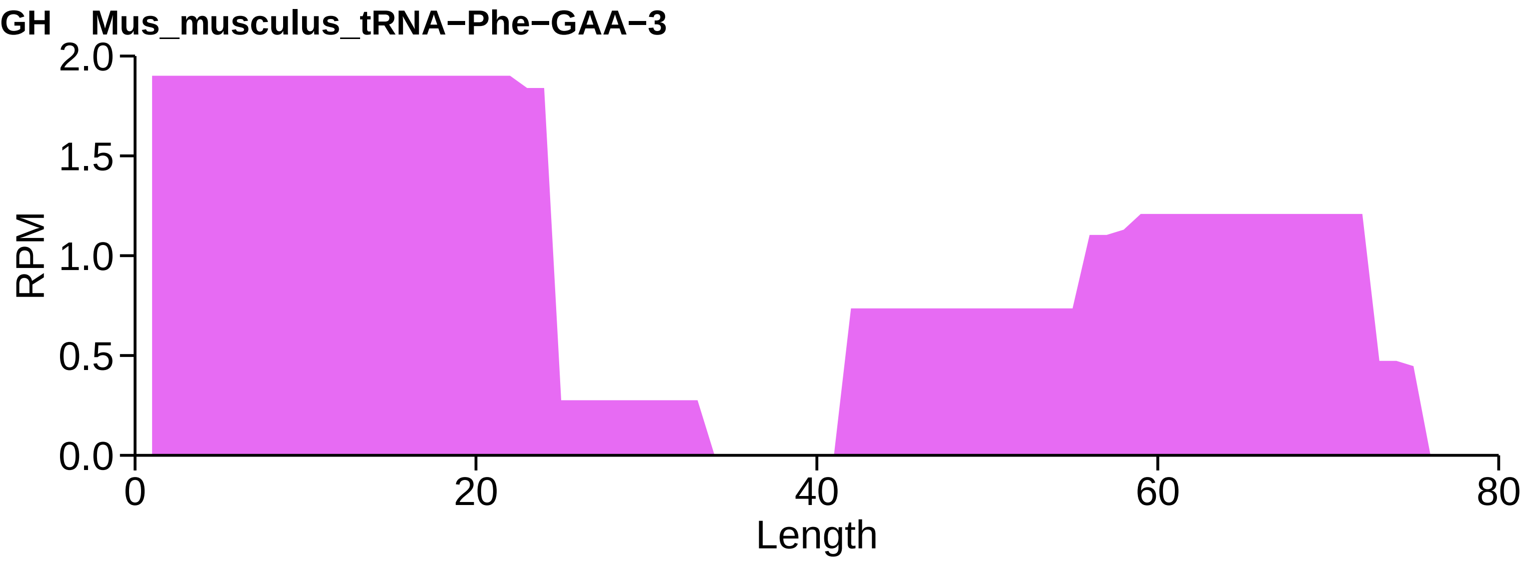

GH Mus_musculus_tRNA−Phe−GAA−3
2.0
1.5
1.0
0.5
0.0
RPM
0
20
40
60
80
Length

GI Mus_musculus_tRNA−Pro−AGG−1

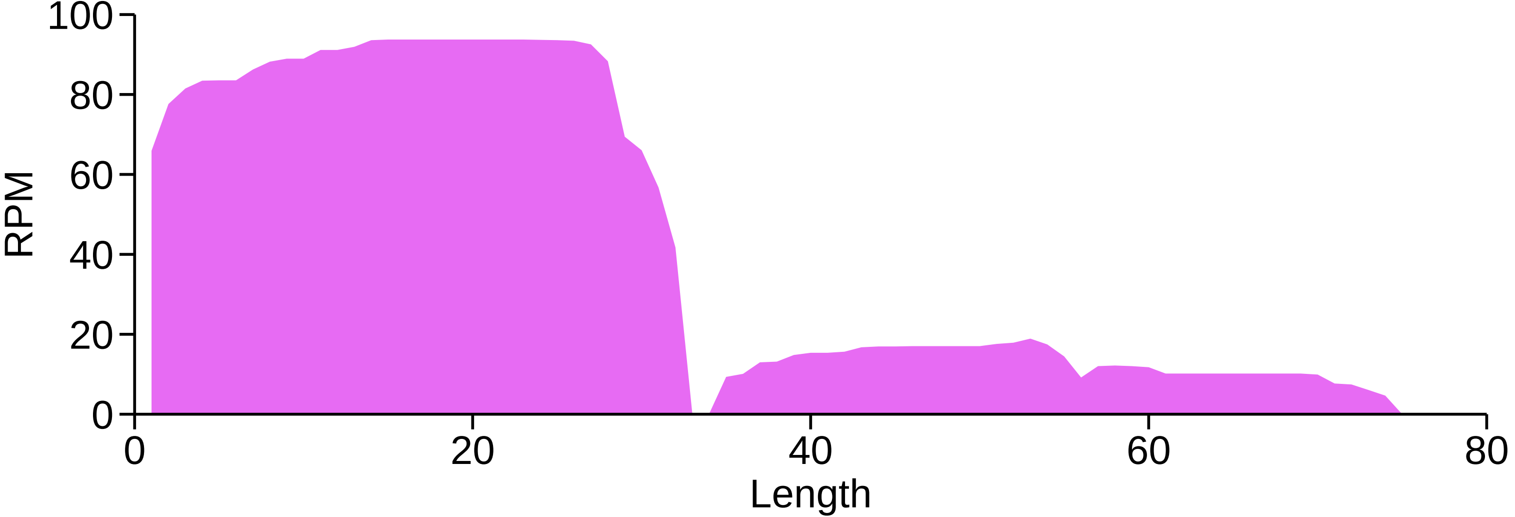

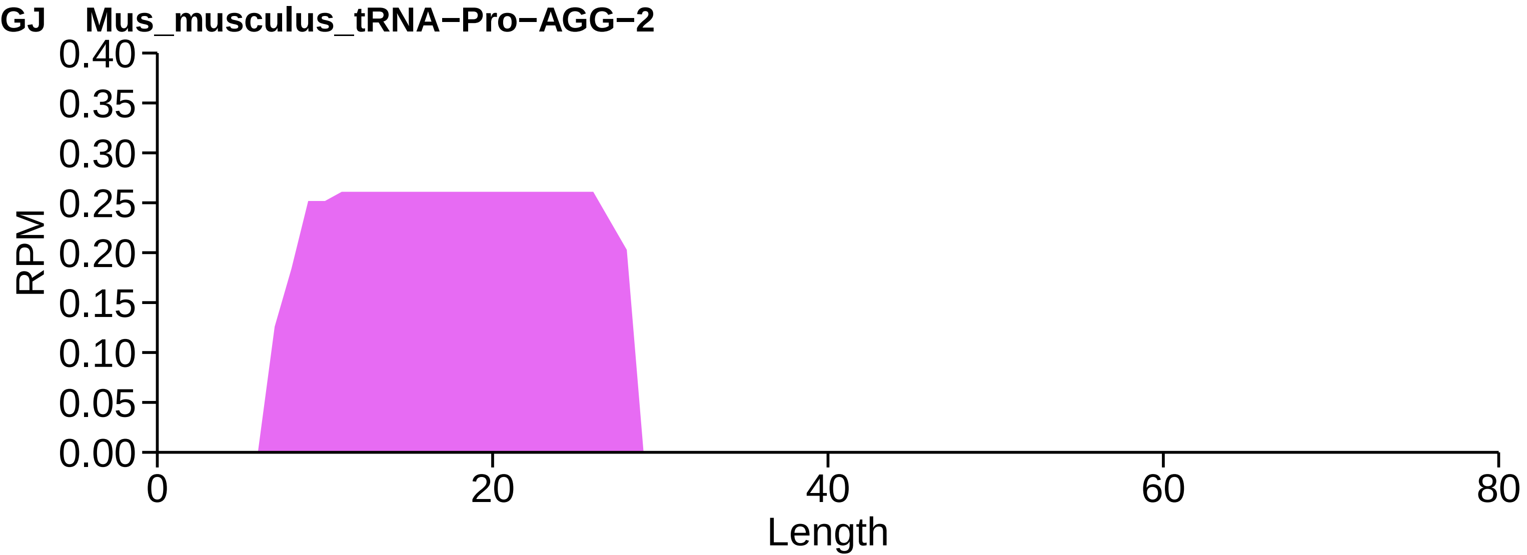

GJ Mus_musculus_tRNA−Pro−AGG−2
RPM
0.40
0.35
0.30
0.25
0.20
0.15
0.10
0.05
0.00
0
20
40
60
80
Length

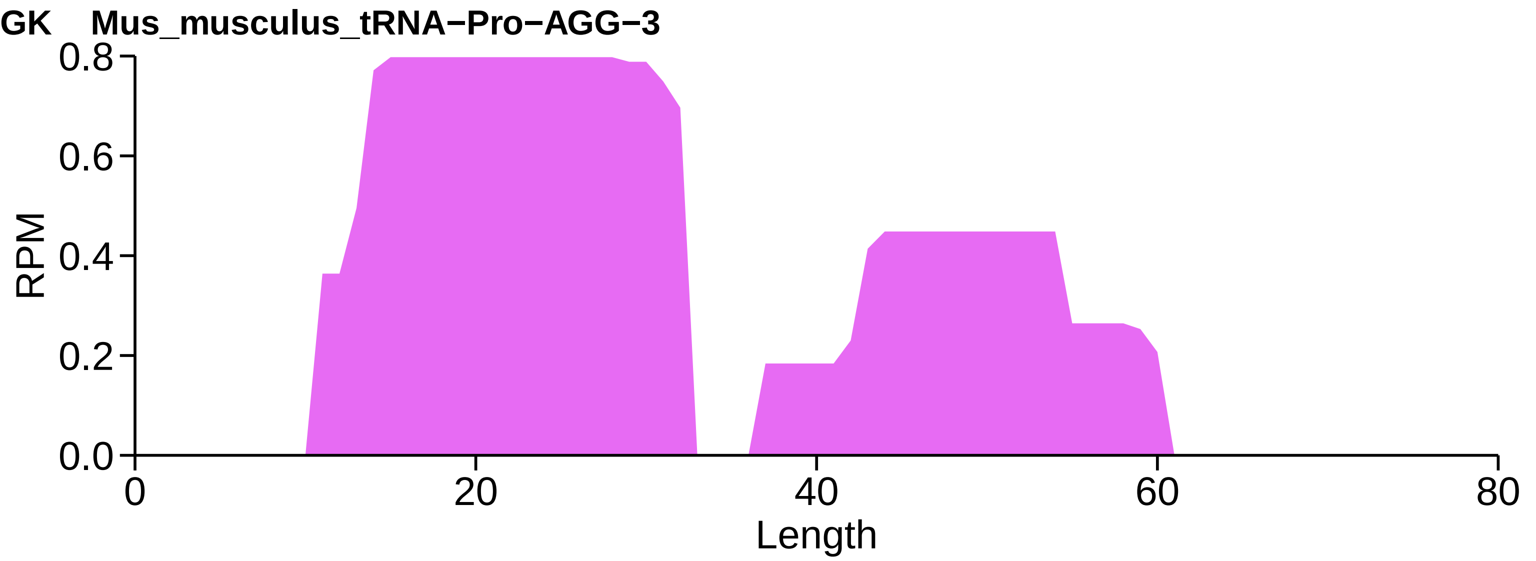

GK Mus_musculus_tRNA−Pro−AGG−3
0.8
0.6
0.4
0.2
0.0
RPM
0
20
40
60
80
Length

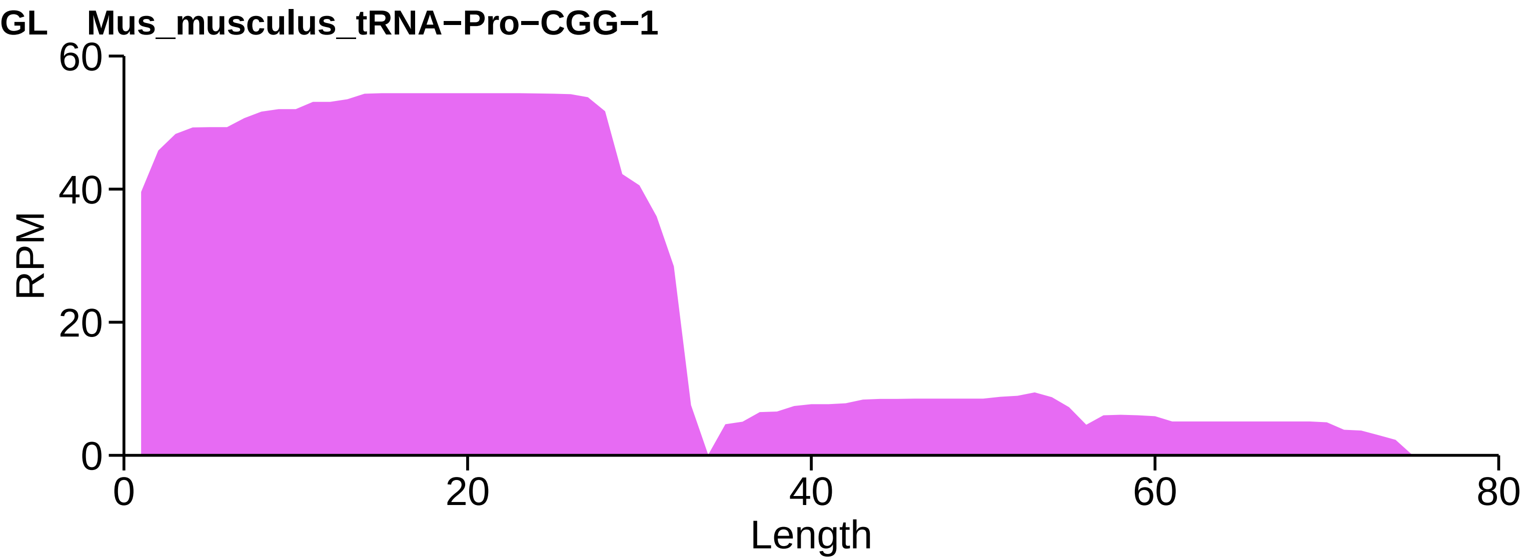

GL Mus_musculus_tRNA−Pro−CGG−1
RPM
60
40
20
0
0
20
40
60
80
Length

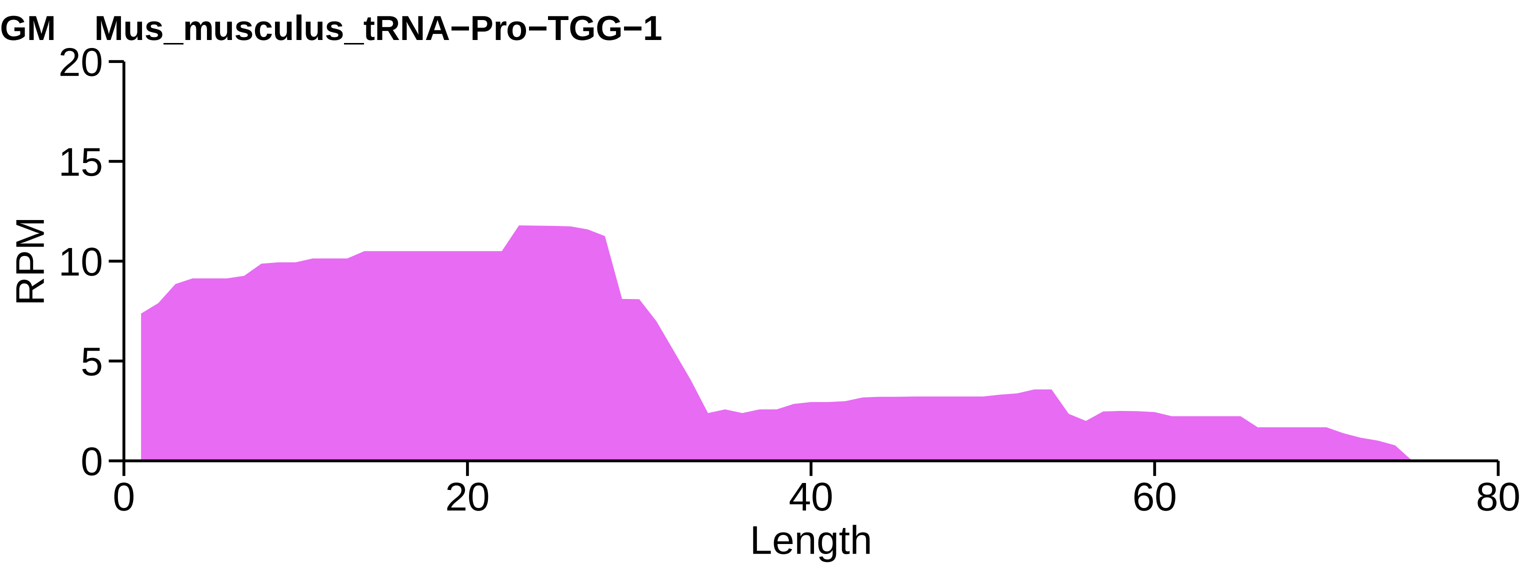
GM Mus_musculus_tRNA−Pro−TGG−1
RPM
Length
0
5
10
15
20
0
20
40
60
80

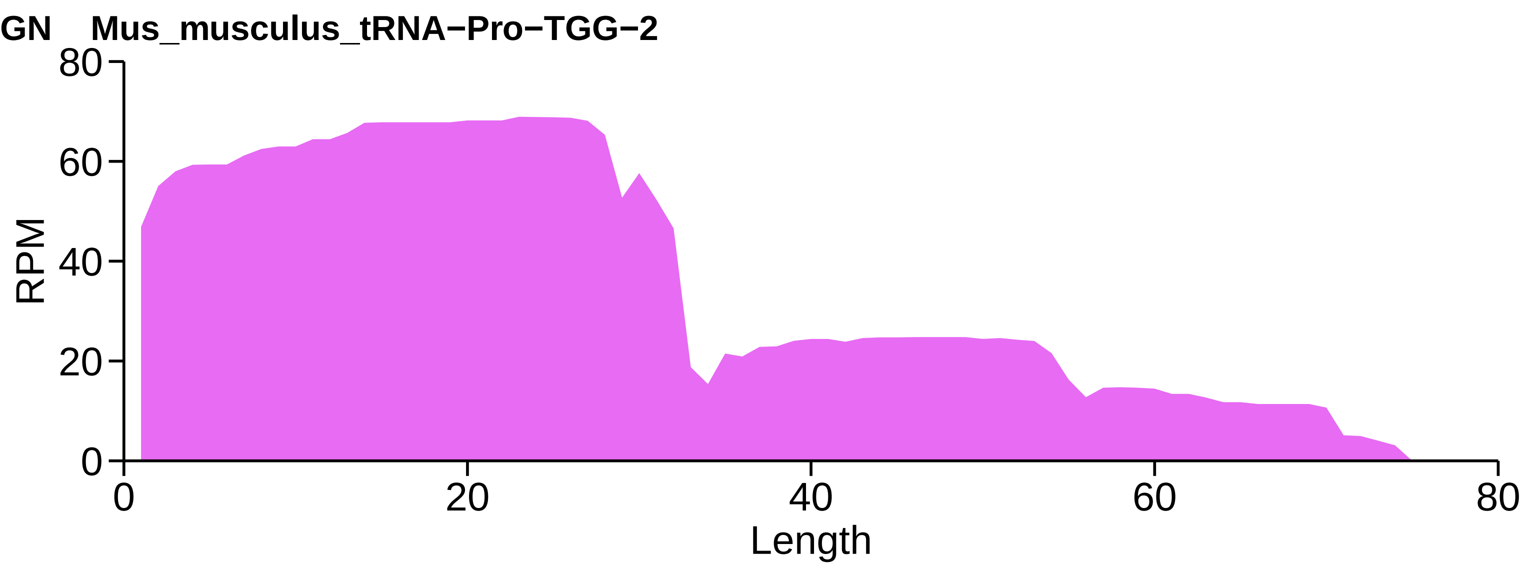

GN Mus_musculus_tRNA−Pro−TGG−2
RPM
80
60
40
20
0
0
20
40
60
80
Length

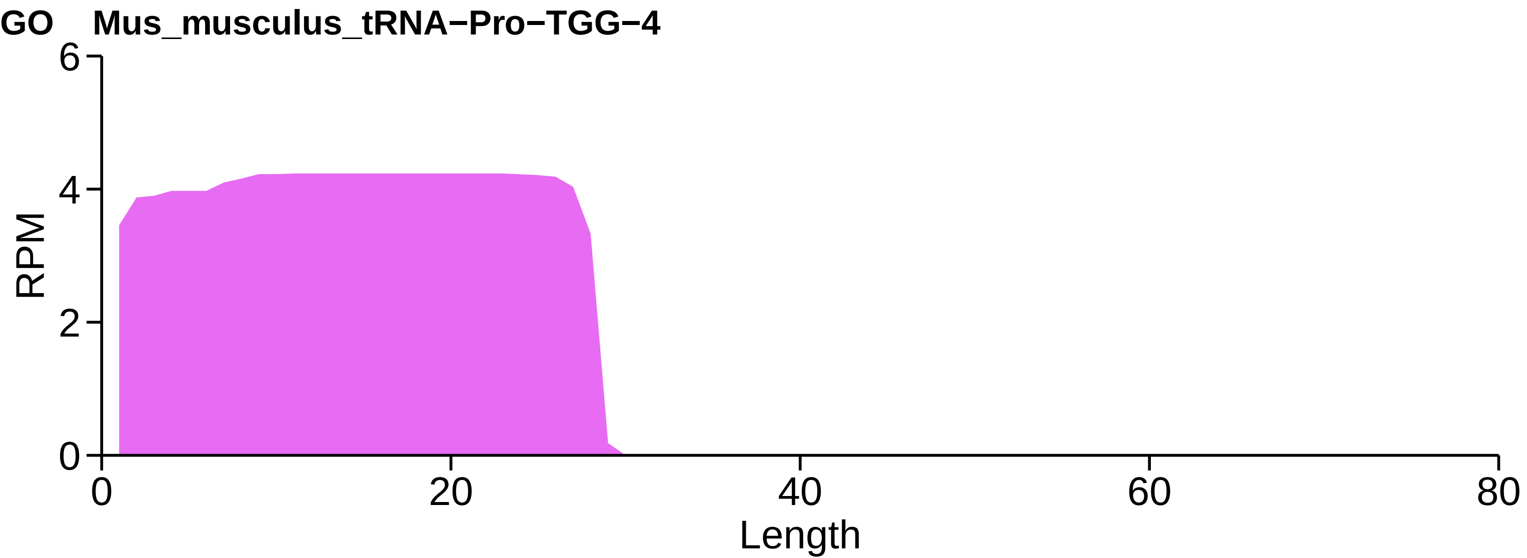
GO Mus_musculus_tRNA−Pro−TGG−4
RPM
0
2
4
6
0
20
40
60
80
Length

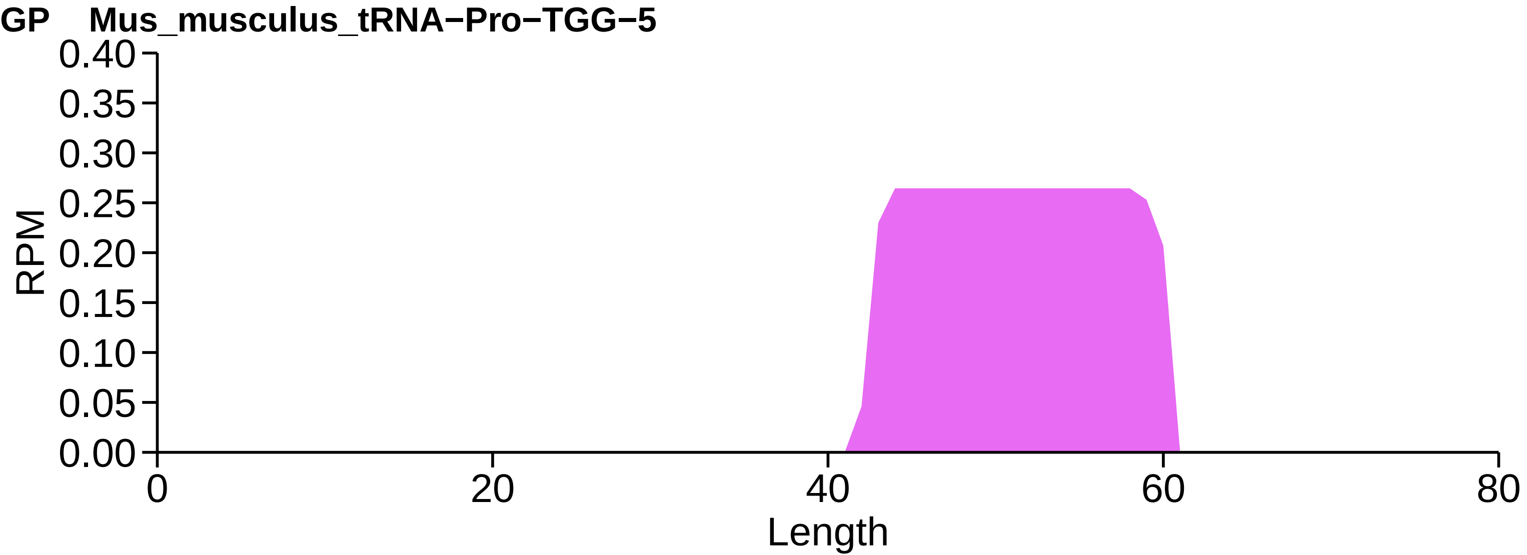

GP Mus_musculus_tRNA−Pro−TGG−5
RPM
0.40
0.35
0.30
0.25
0.20
0.15
0.10
0.05
0.00
0
20
40
60
80
Length

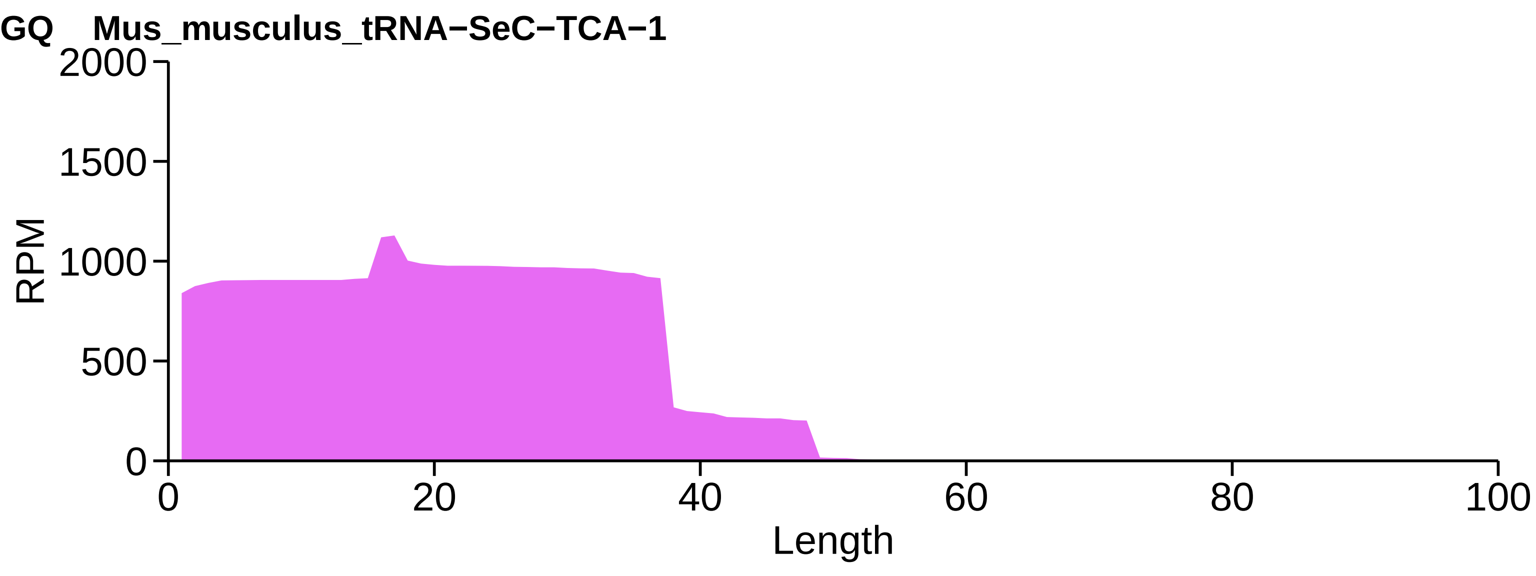

GQ Mus_musculus_tRNA−SeC−TCA−1
RPM
2000
1500
1000
500
0
0
20
40
60
80
100
Length

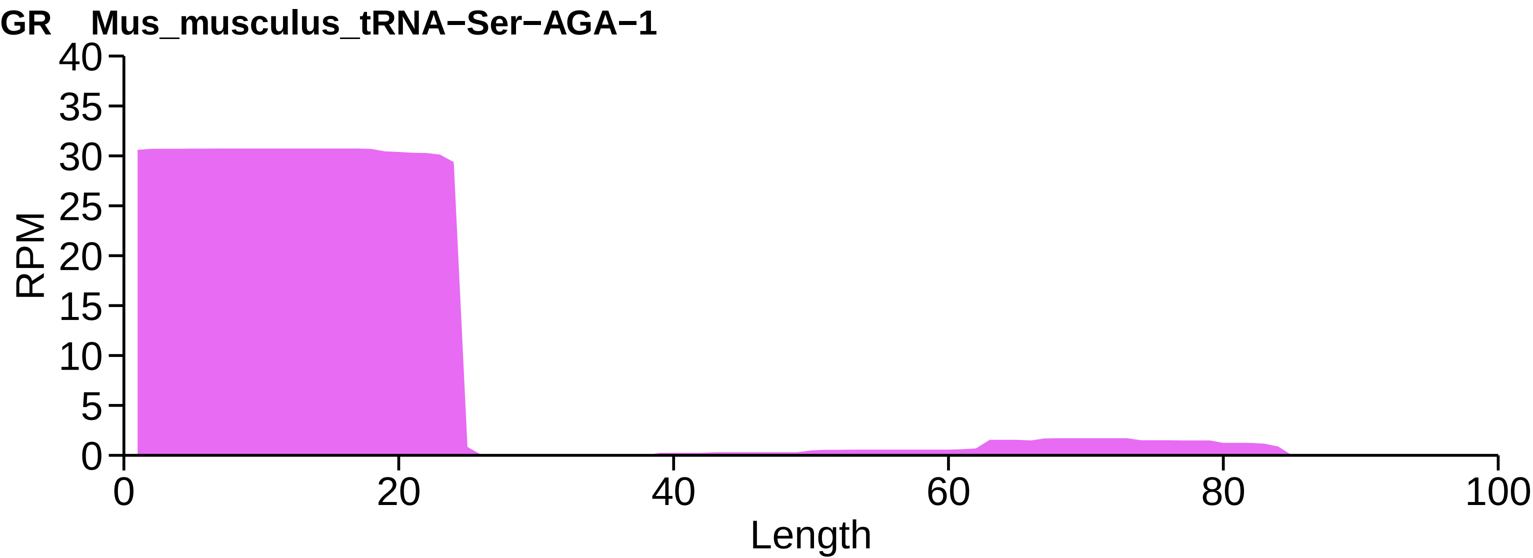
GR Mus_musculus_tRNA−Ser−AGA−1
RPM
0
5
10
15
20
25
30
35
40
0
20
40
60
80
100
Length

GS   Mus_musculus_tRNA−Ser−AGA−2

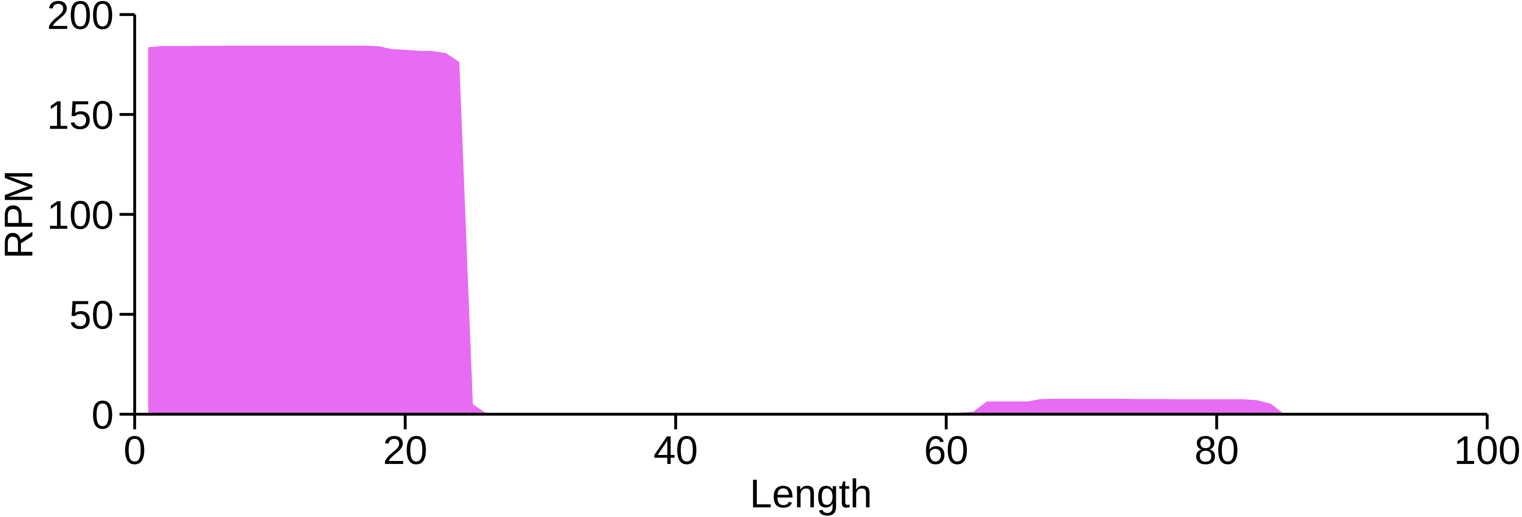

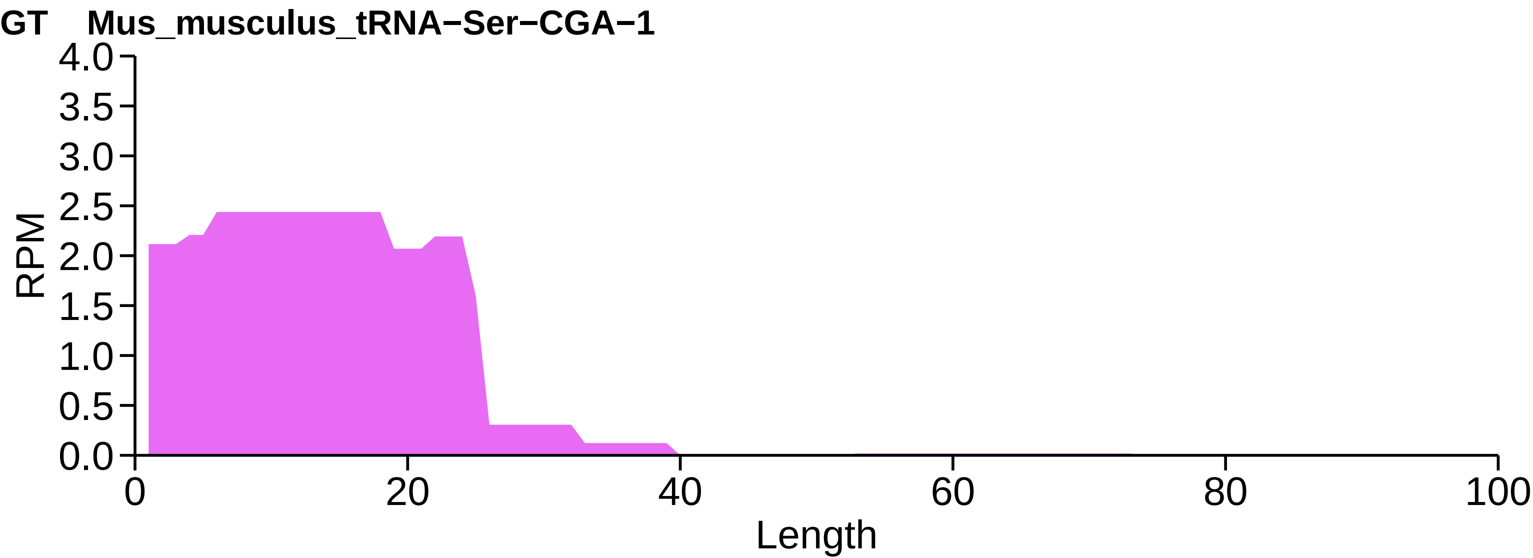

GT Mus_musculus_tRNA−Ser−CGA−1
RPM
4.0
3.5
3.0
2.5
2.0
1.5
1.0
0.5
0.0
0
20
40
60
80
100
Length

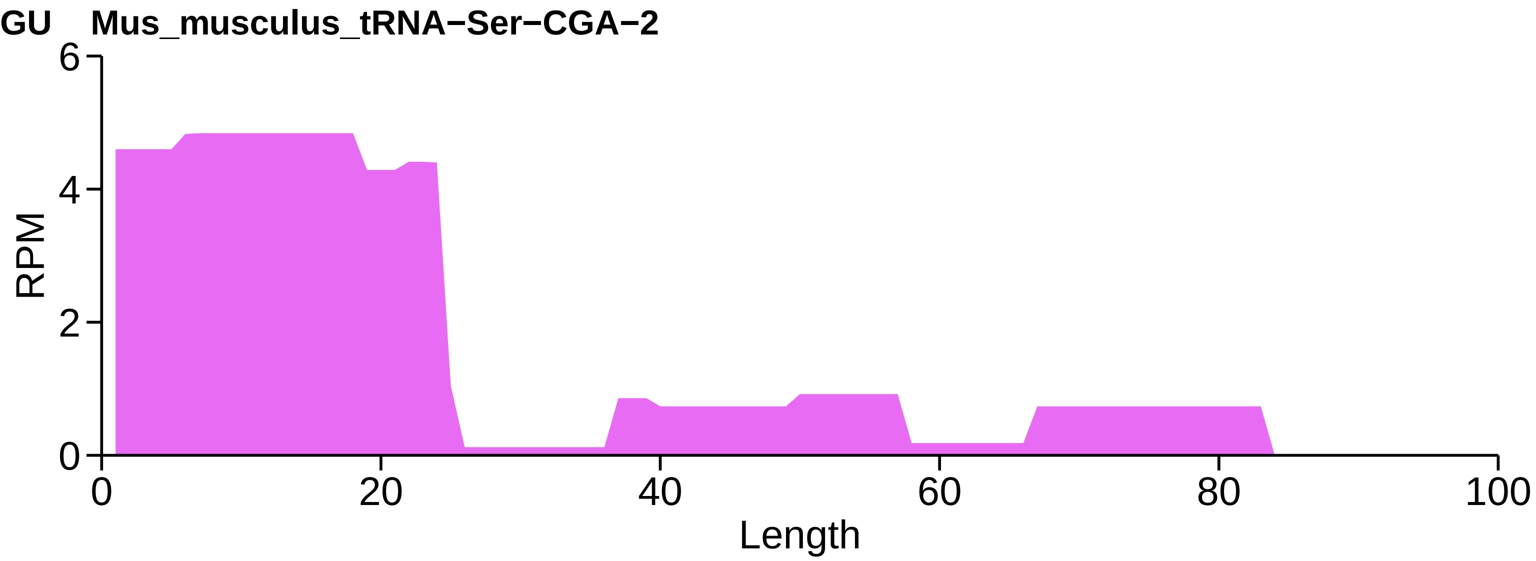
GU Mus_musculus_tRNA−Ser−CGA−2
RPM
0
2
4
6
0
20
40
60
80
100
Length

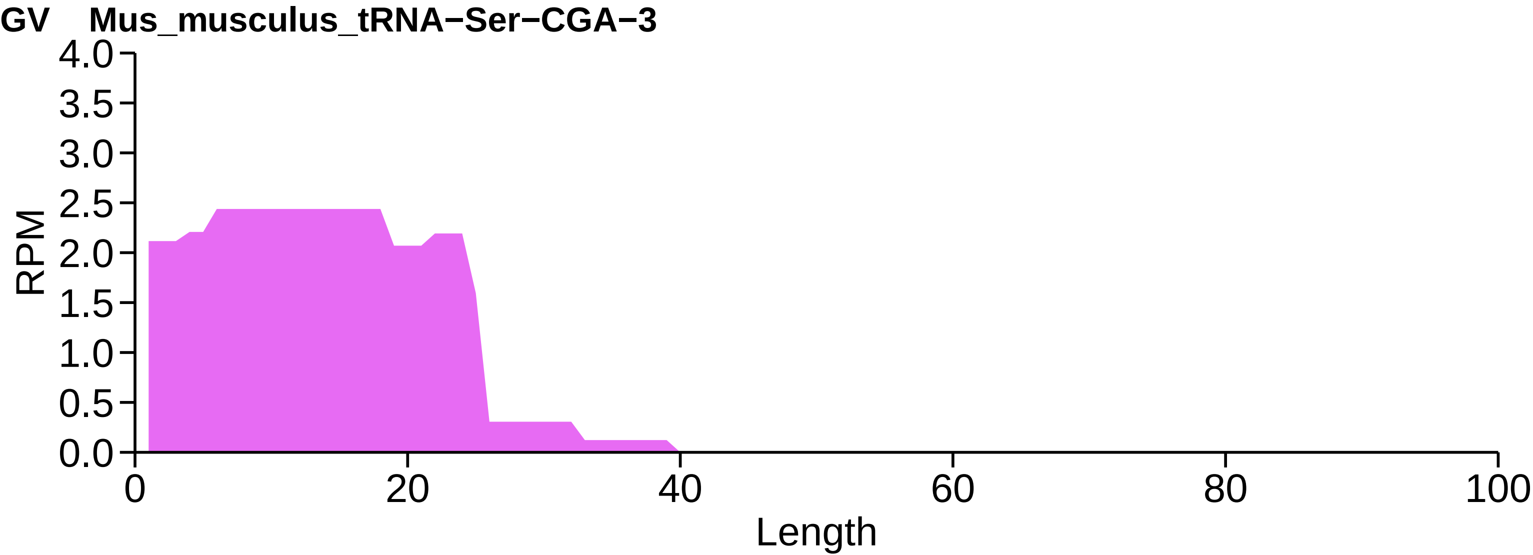
GV Mus_musculus_tRNA−Ser−CGA−3
RPM
4.0
3.5
3.0
2.5
2.0
1.5
1.0
0.5
0.0
0
20
40
60
80
100
Length

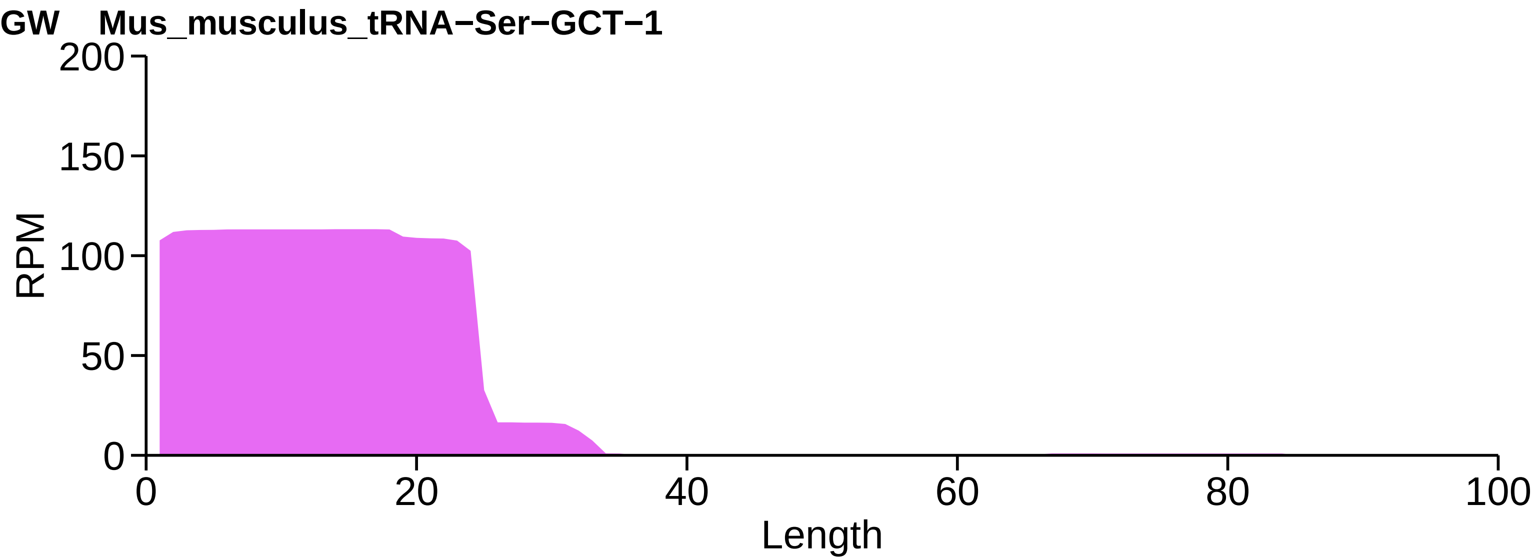

GW Mus_musculus_tRNA−Ser−GCT−1
RPM
200
150
100
50
0
0
20
40
60
80
100
Length

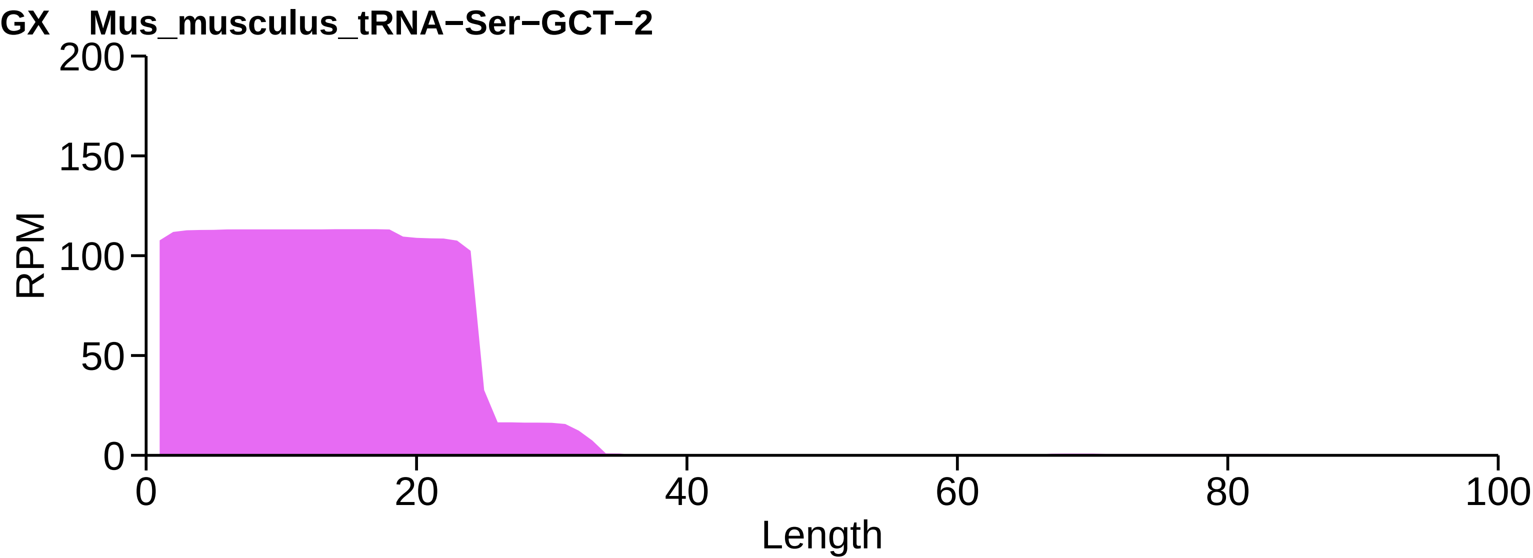
GX Mus_musculus_tRNA−Ser−GCT−2
RPM
Length
0
50
100
150
200
0
20
40
60
80
100

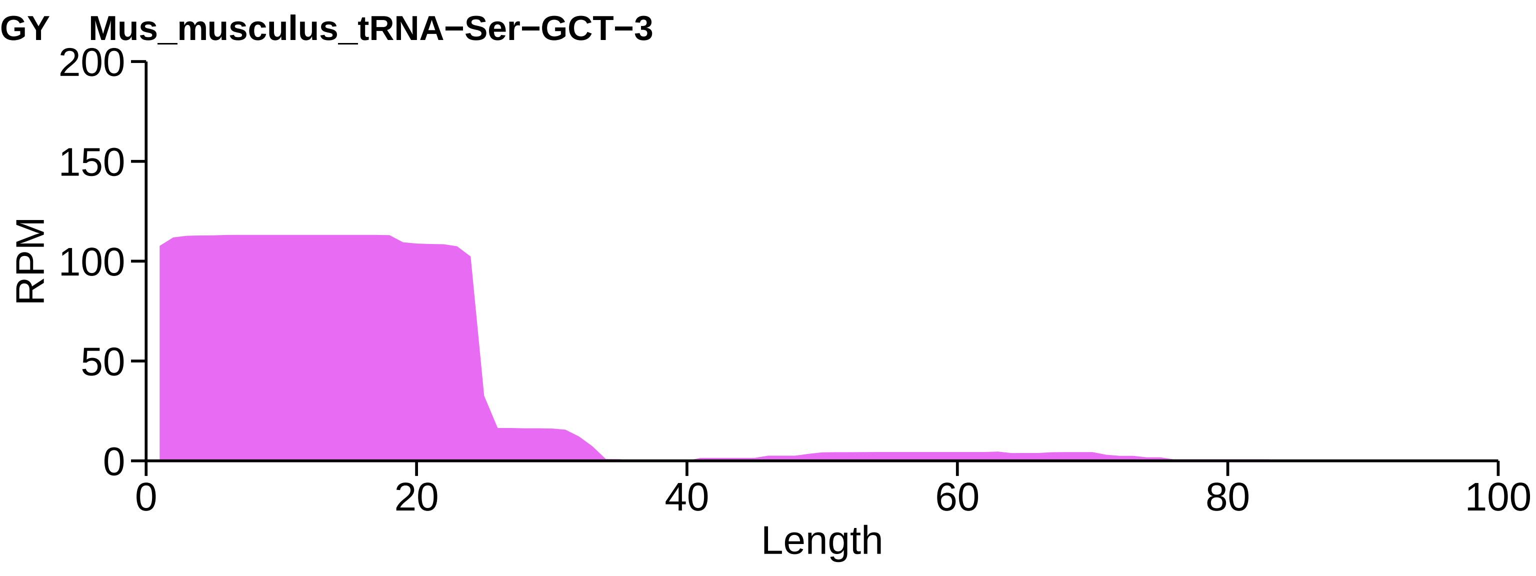
GY Mus_musculus_tRNA−Ser−GCT−3
RPM
200
150
100
50
0
0
20
40
60
80
100
Length

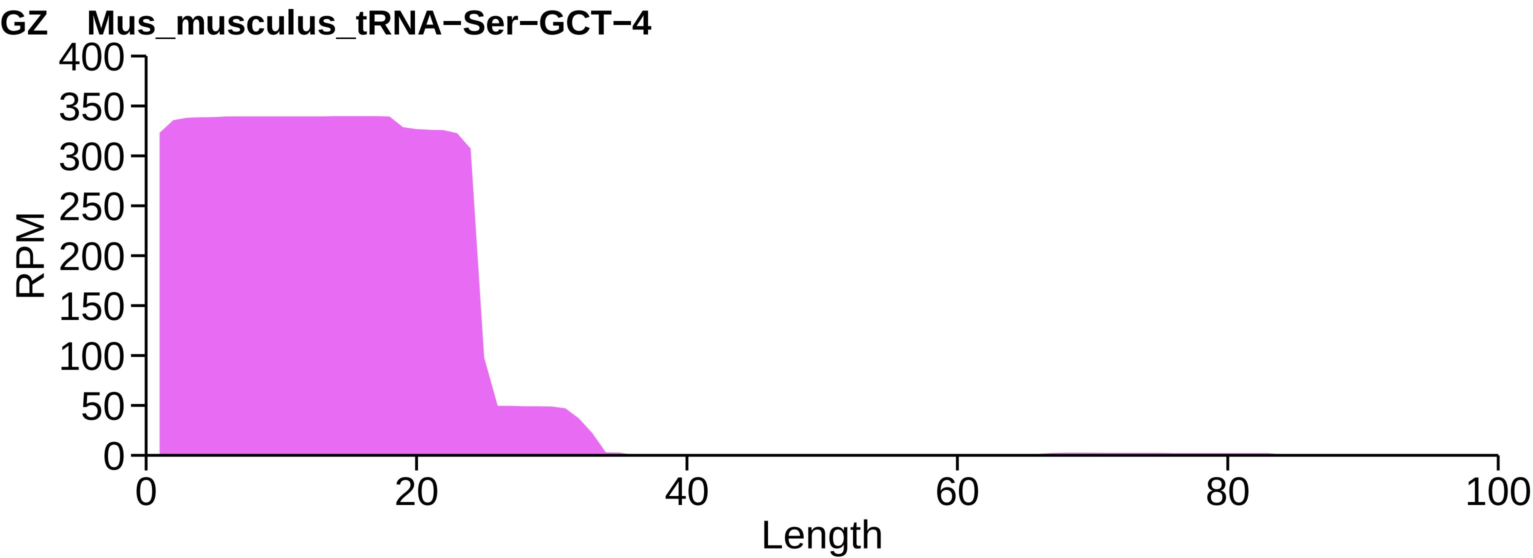

GZ Mus_musculus_tRNA−Ser−GCT−4
RPM
400
350
300
250
200
150
100
50
0
0
20
40
60
80
100
Length

HA Mus_musculus_tRNA−Ser−GCT−5

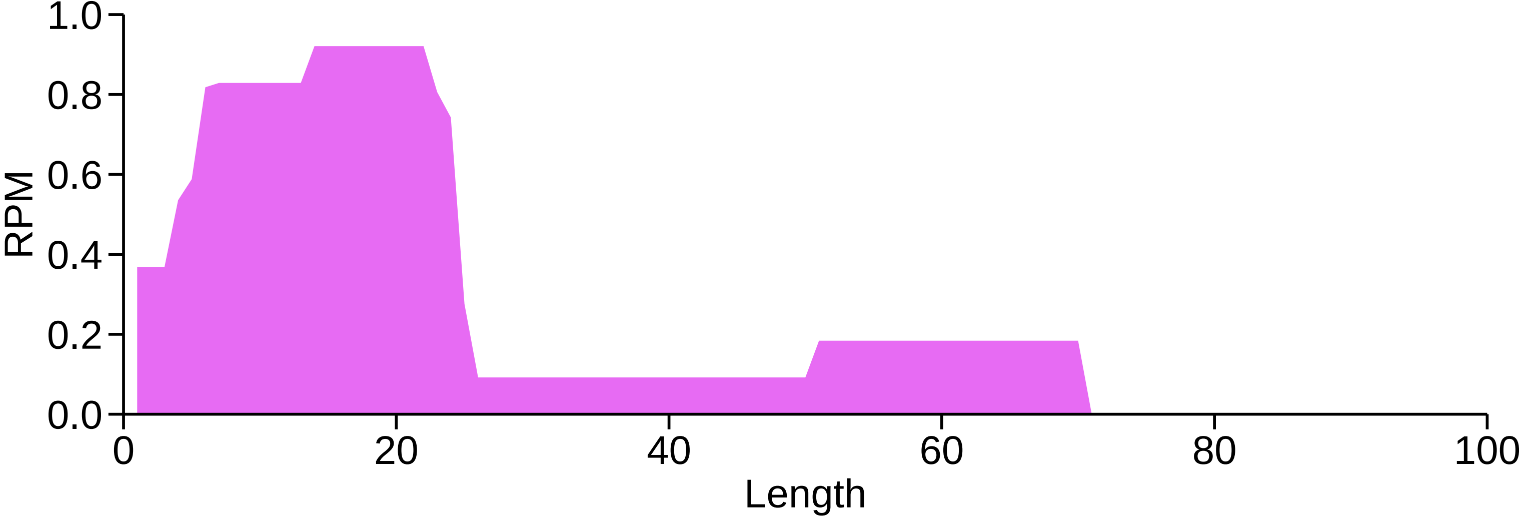

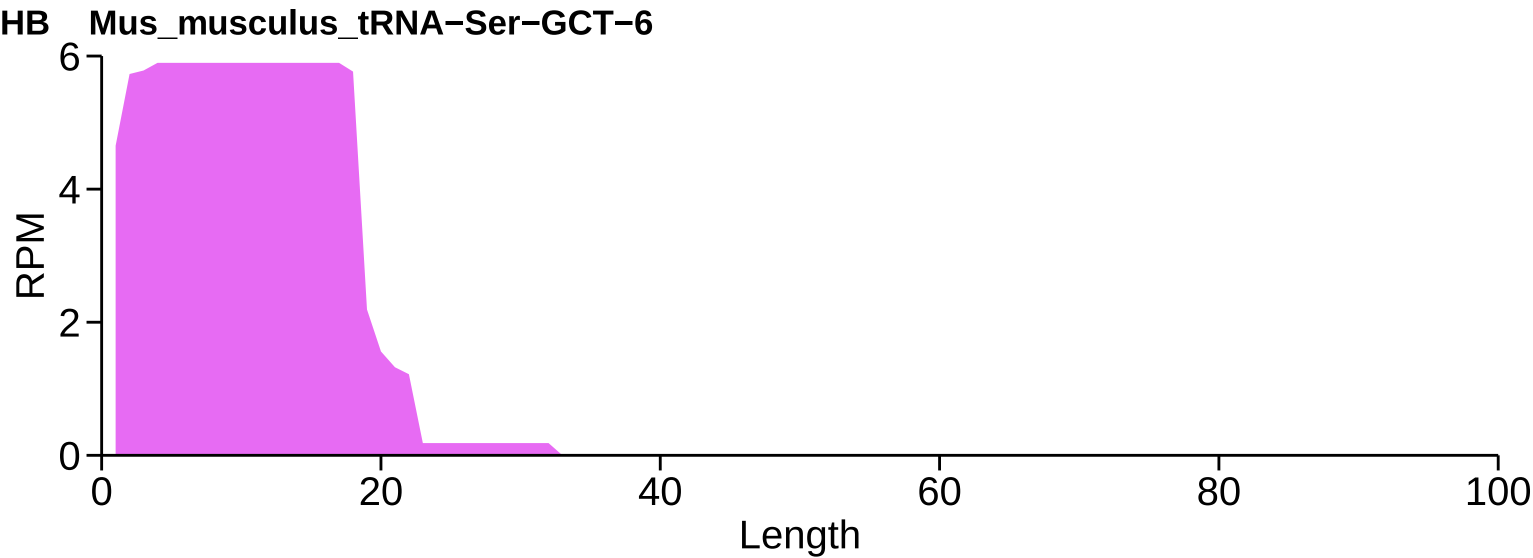
HB
Mus_musculus_tRNA−Ser−GCT−6
RPM
Length
0
2
4
6
0
20
40
60
80
100

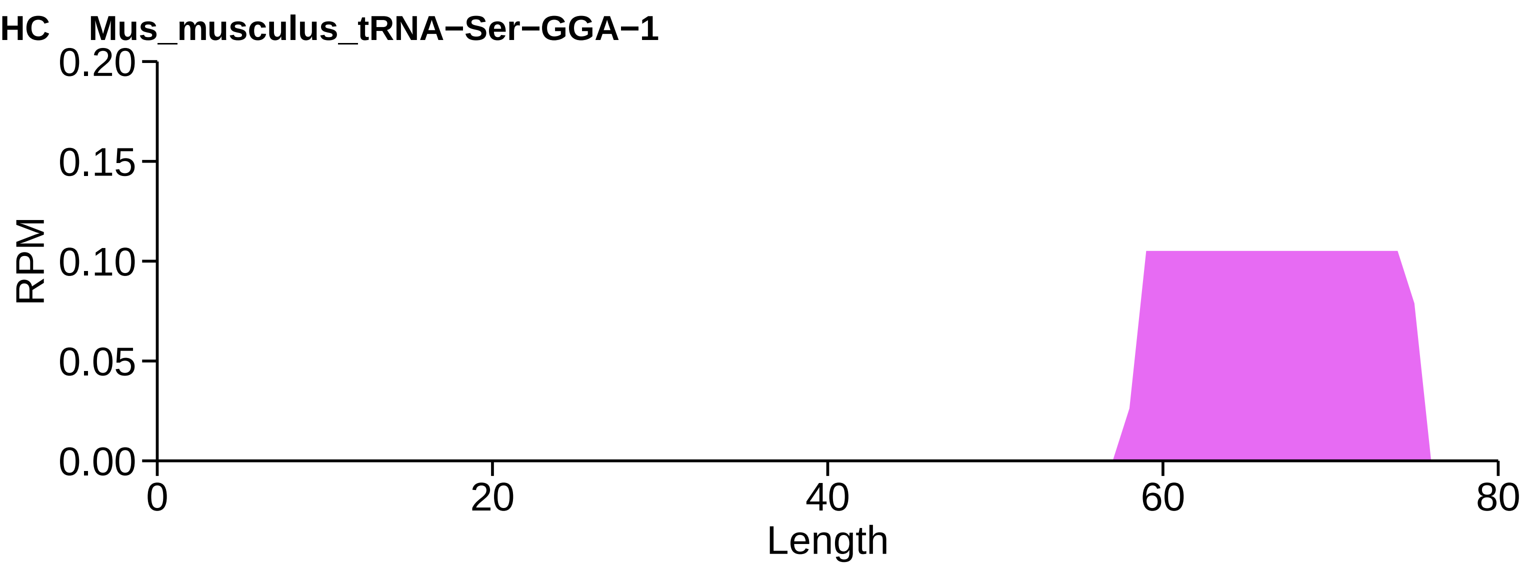
HC Mus_musculus_tRNA−Ser−GGA−1
RPM
0.20
0.15
0.10
0.05
0.00
0
20
40
60
80
Length

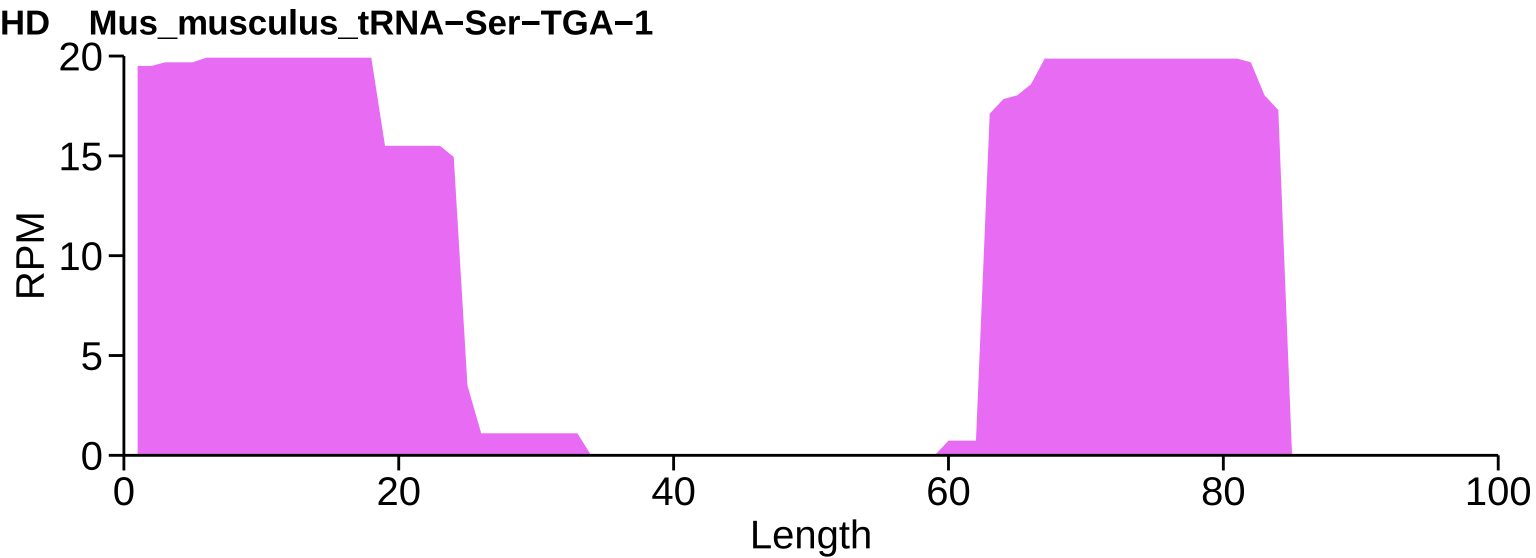

HD Mus_musculus_tRNA−Ser−TGA−1
RPM
0
5
10
15
20
0
20
40
60
80
100
Length

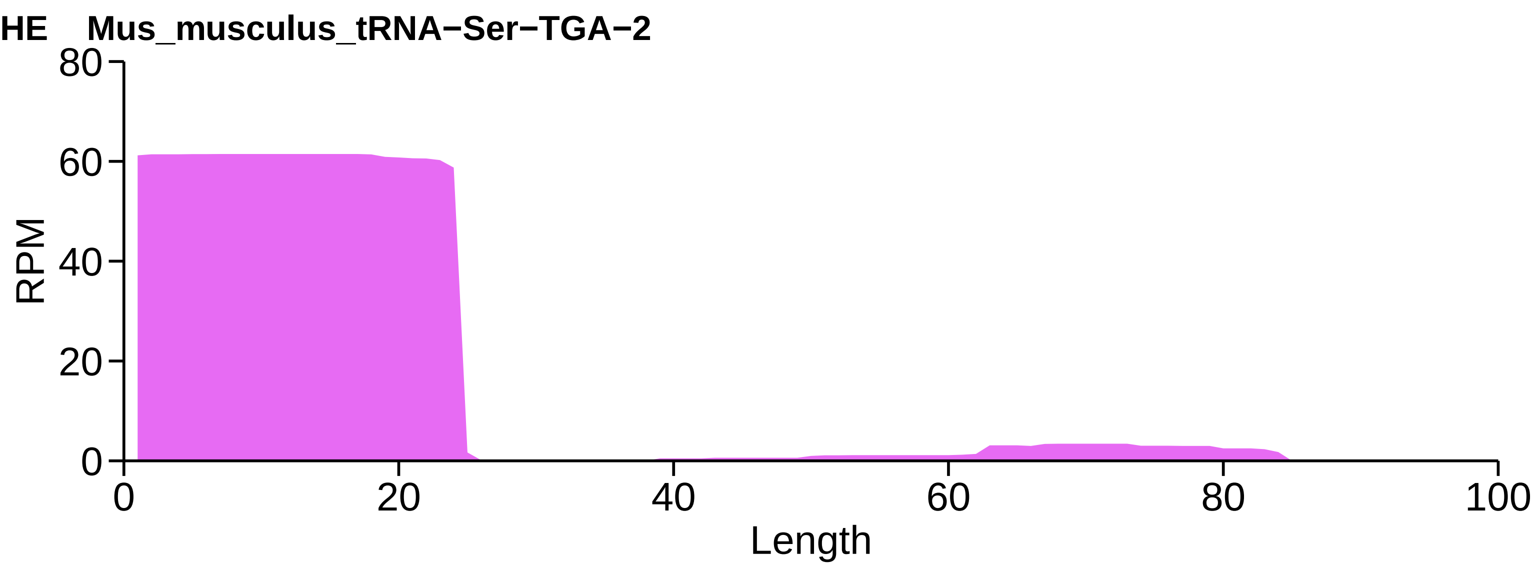
HE Mus_musculus_tRNA−Ser−TGA−2
RPM
Length
0
20
40
60
80
100

HF Mus_musculus_tRNA−Thr−AGT−1

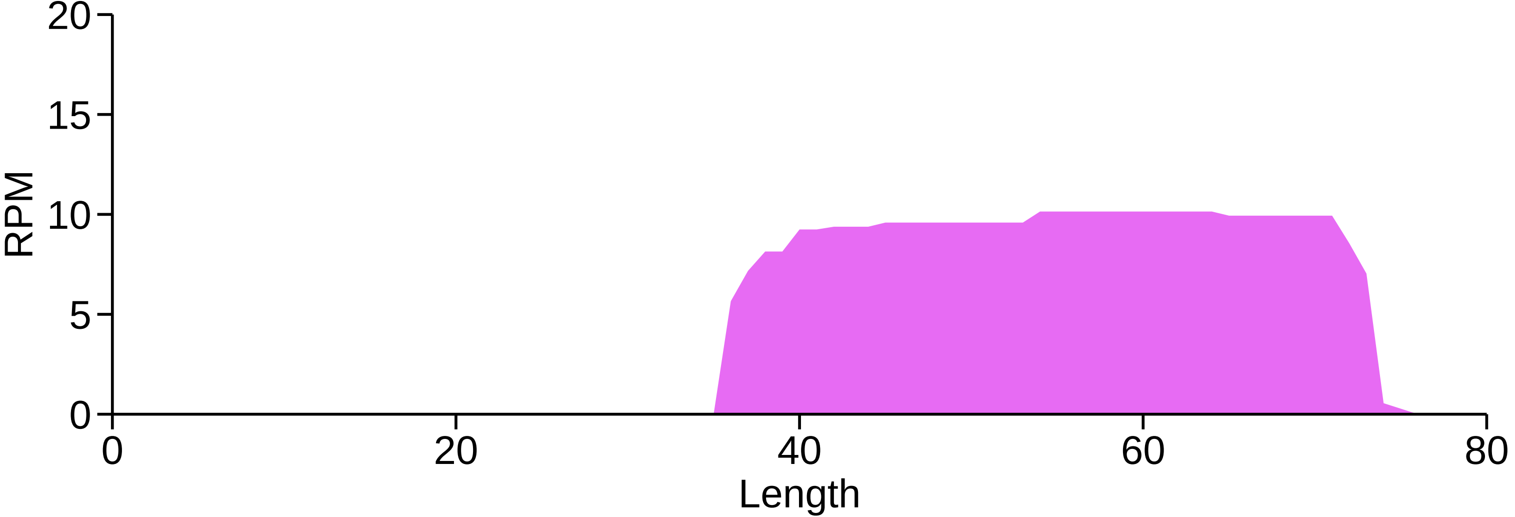

HG Mus_musculus_tRNA−Thr−AGT−2

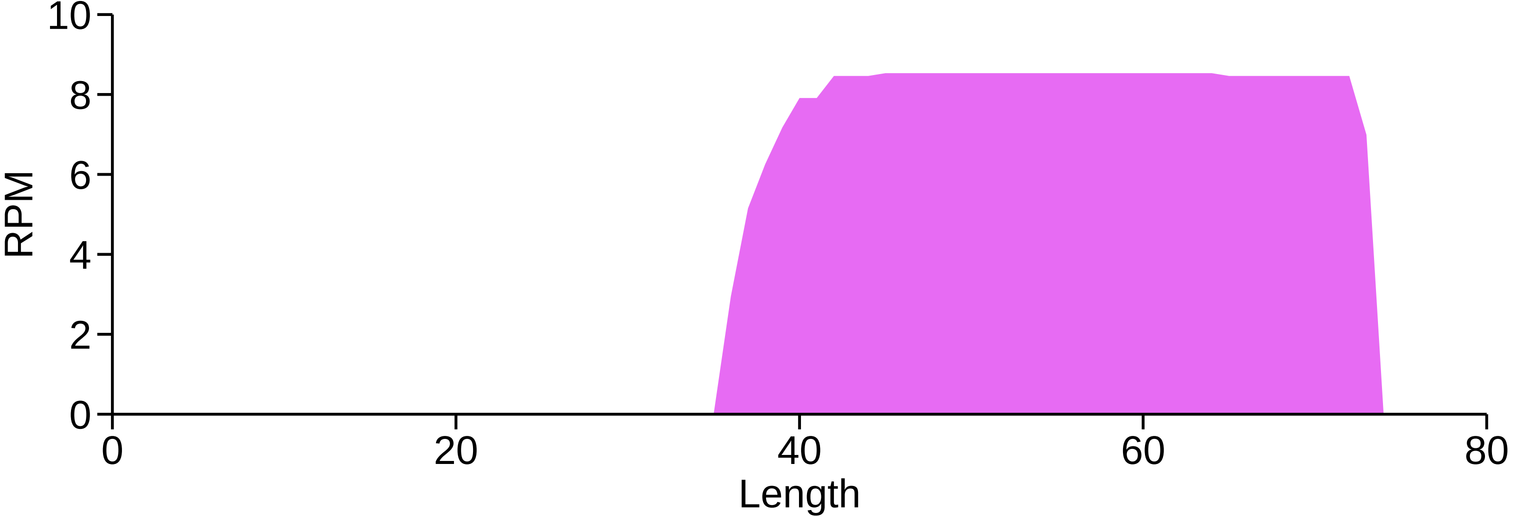

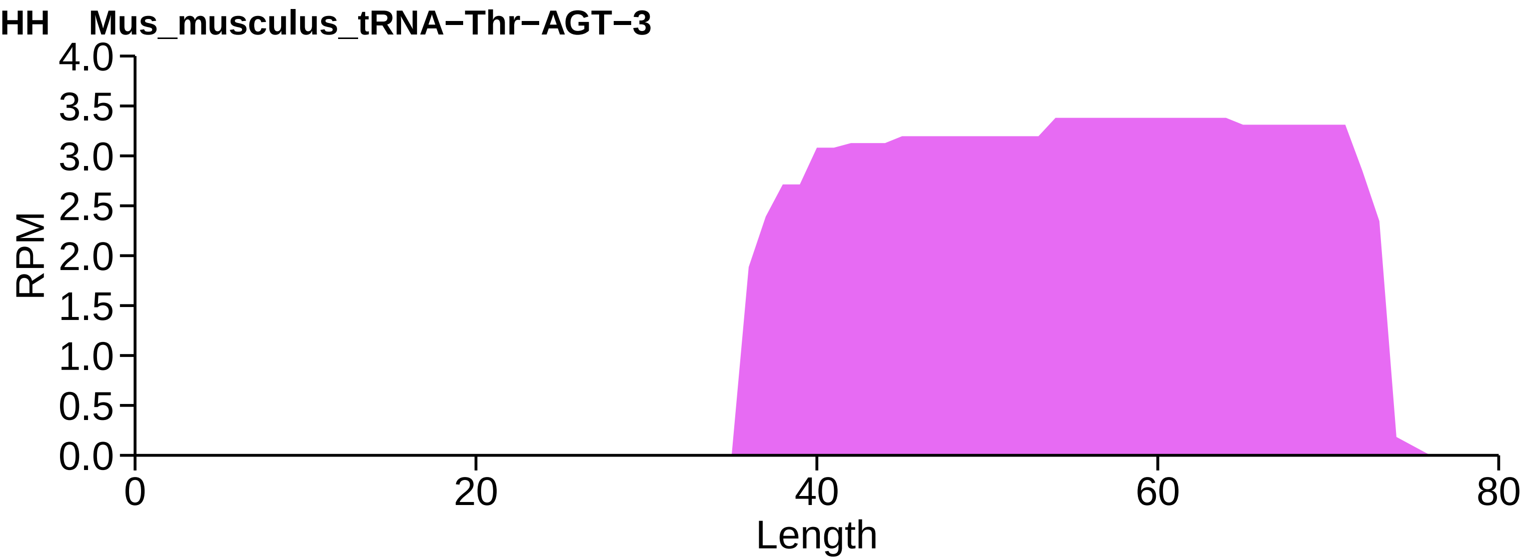
HH Mus_musculus_tRNA−Thr−AGT−3
RPM
4.0
3.5
3.0
2.5
2.0
1.5
1.0
0.5
0.0
0
20
40
60
80
Length

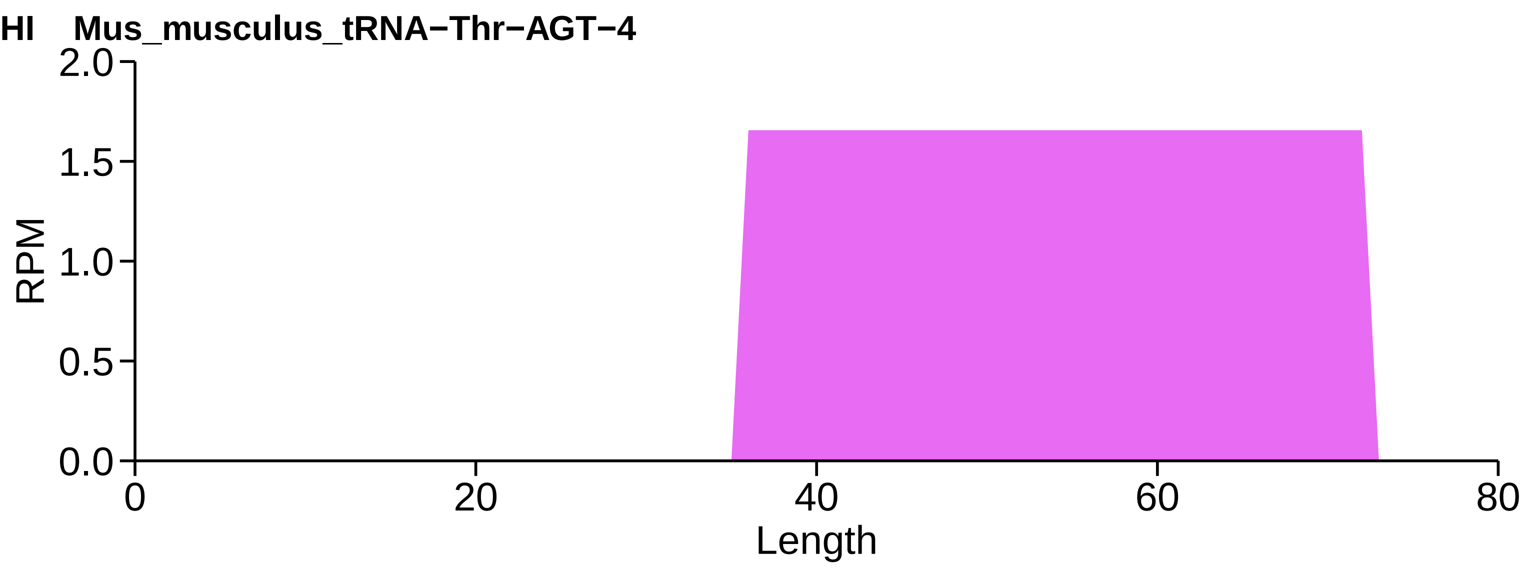
HI Mus_musculus_tRNA−Thr−AGT−4
2.0
1.5
1.0
0.5
0.0
RPM
0
20
40
60
80
Length

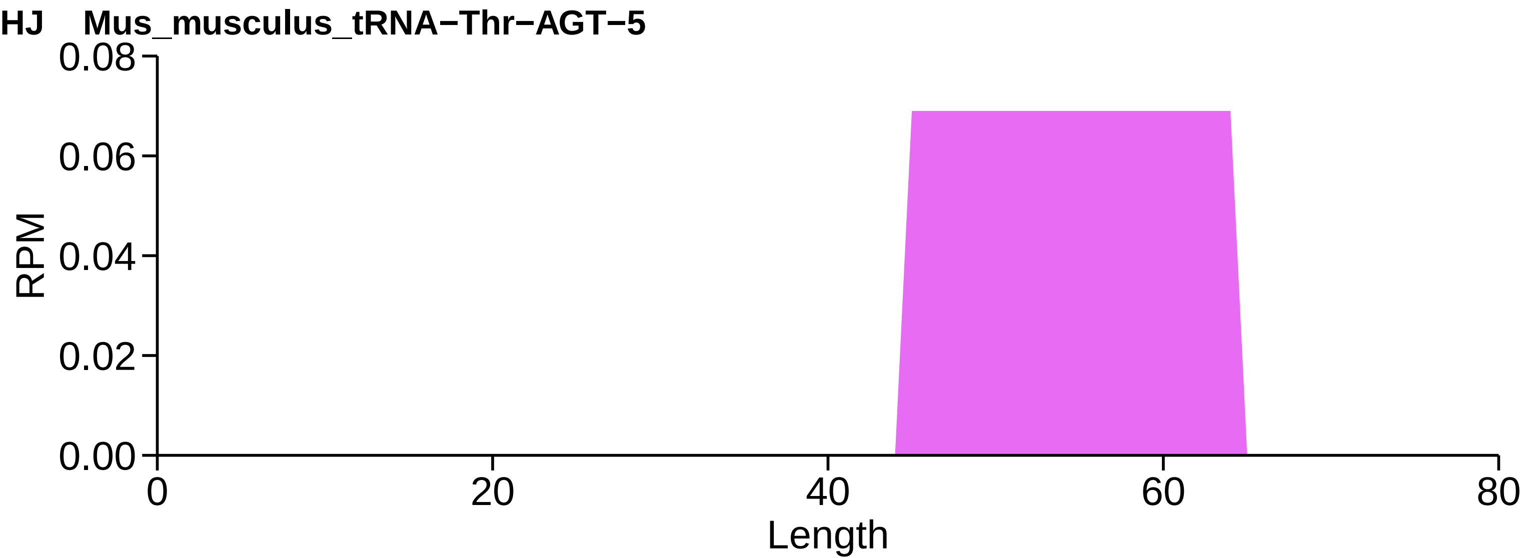

HJ Mus_musculus_tRNA−Thr−AGT−5
0.08
0.06
0.04
0.02
0.00
RPM
0
20
40
60
80
Length

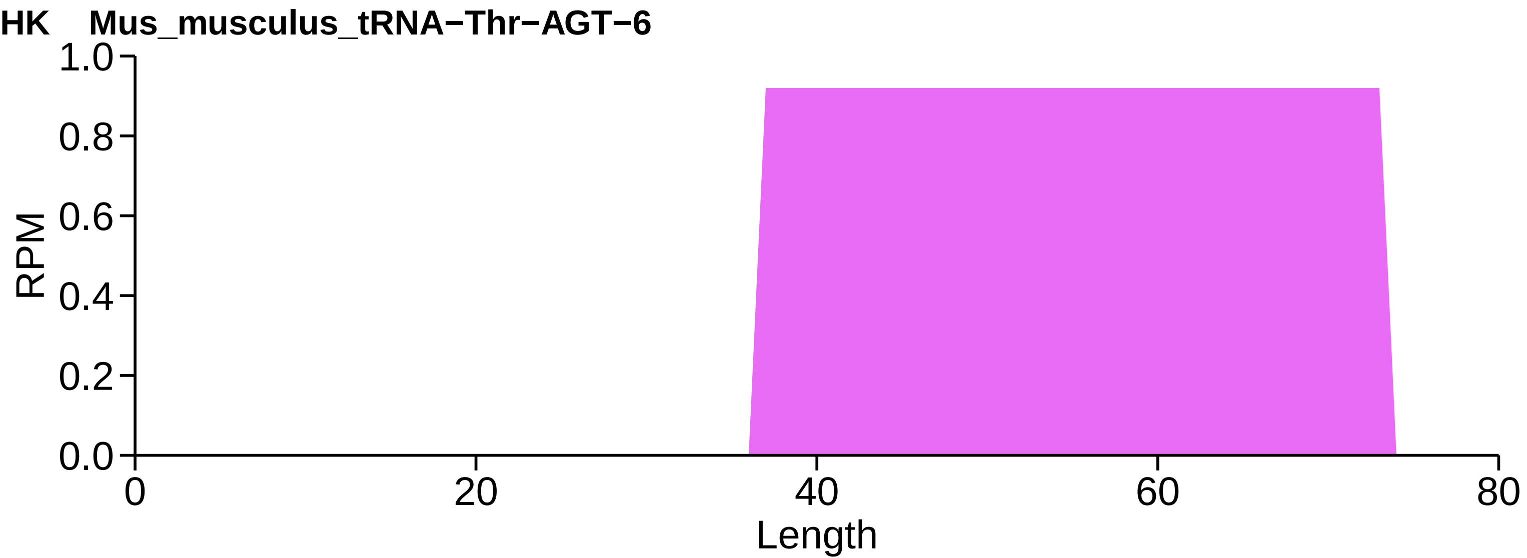
HK Mus_musculus_tRNA−Thr−AGT−6
1.0
0.8
0.6
0.4
0.2
0.0
RPM
0
20
40
60
80
Length

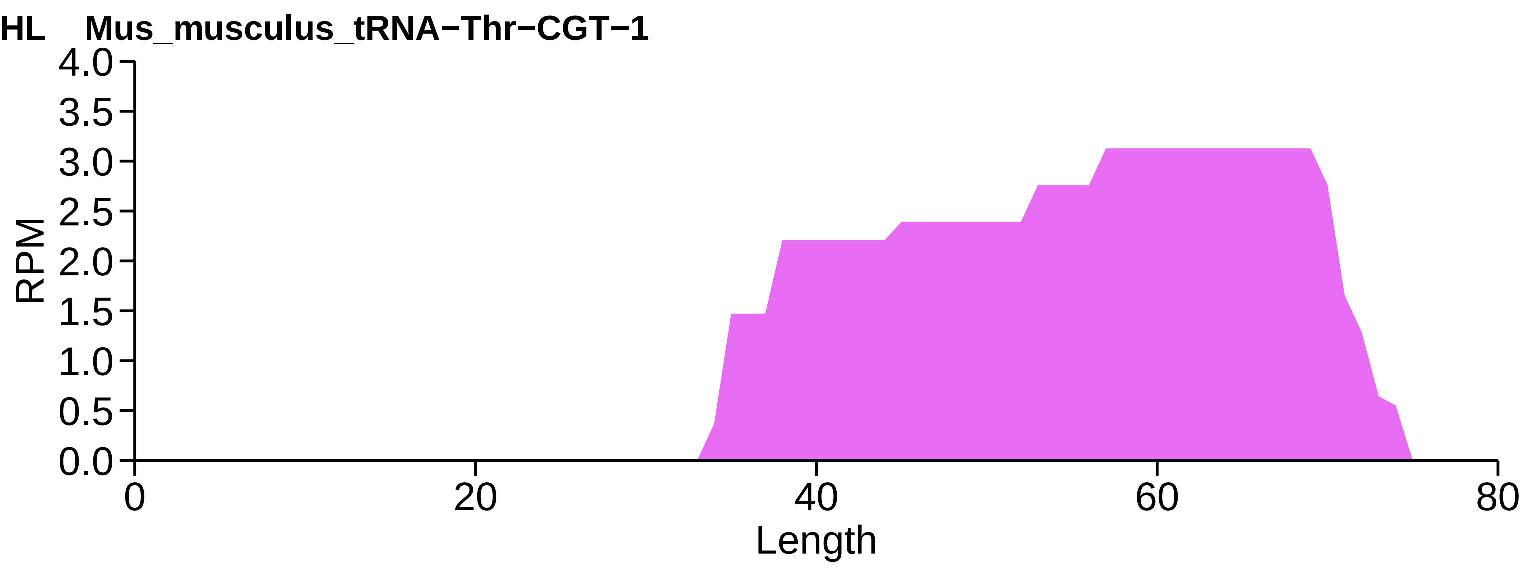
HL Mus_musculus_tRNA−Thr−CGT−1
RPM
4.0
3.5
3.0
2.5
2.0
1.5
1.0
0.5
0.0
0
20
40
60
80
Length

HM Mus_musculus_tRNA−Thr−CGT−2

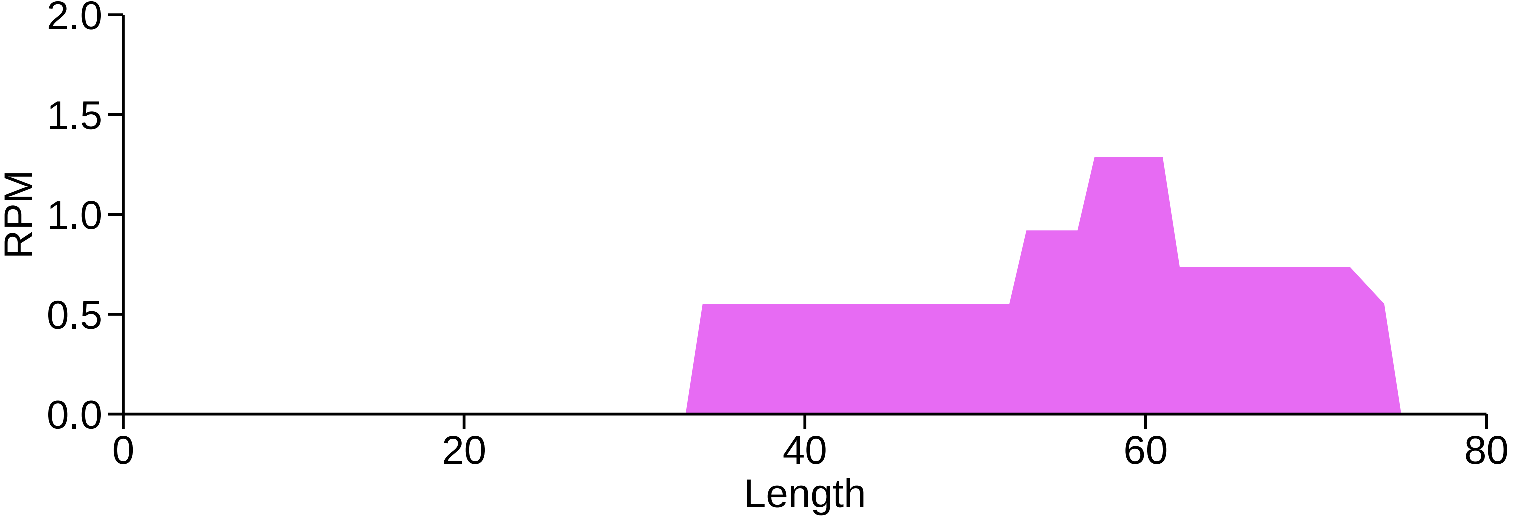

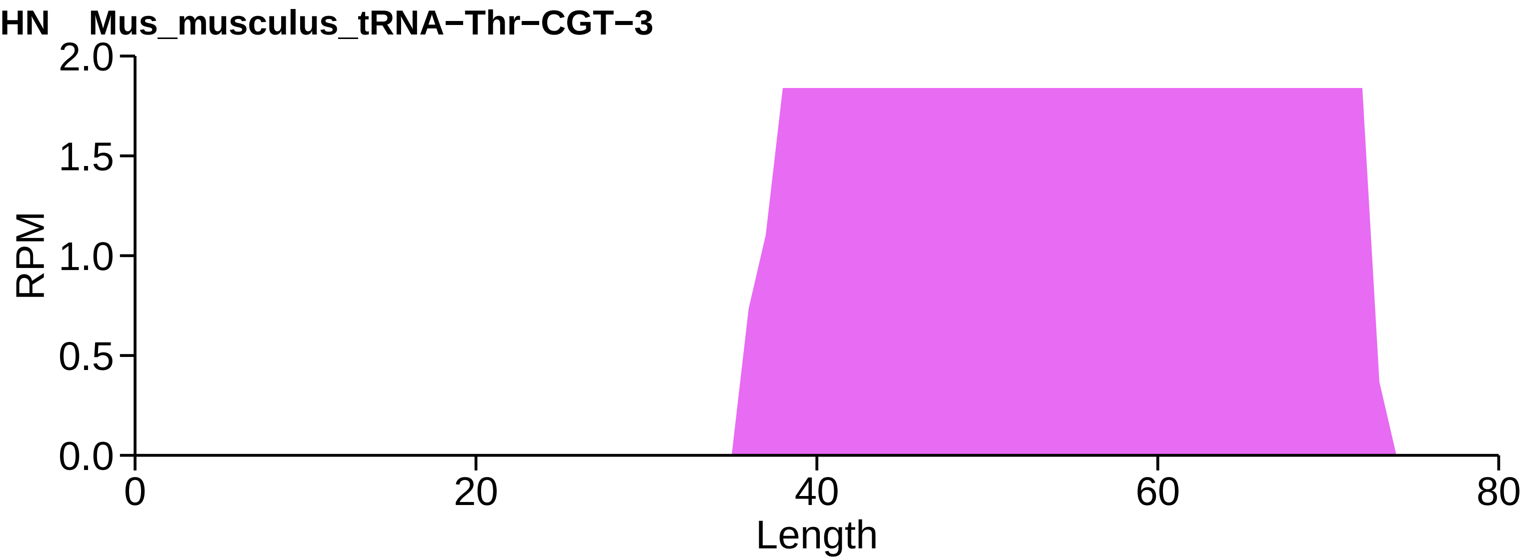
HN Mus_musculus_tRNA−Thr−CGT−3
RPM
2.0
1.5
1.0
0.5
0.0
0
20
40
60
80
Length

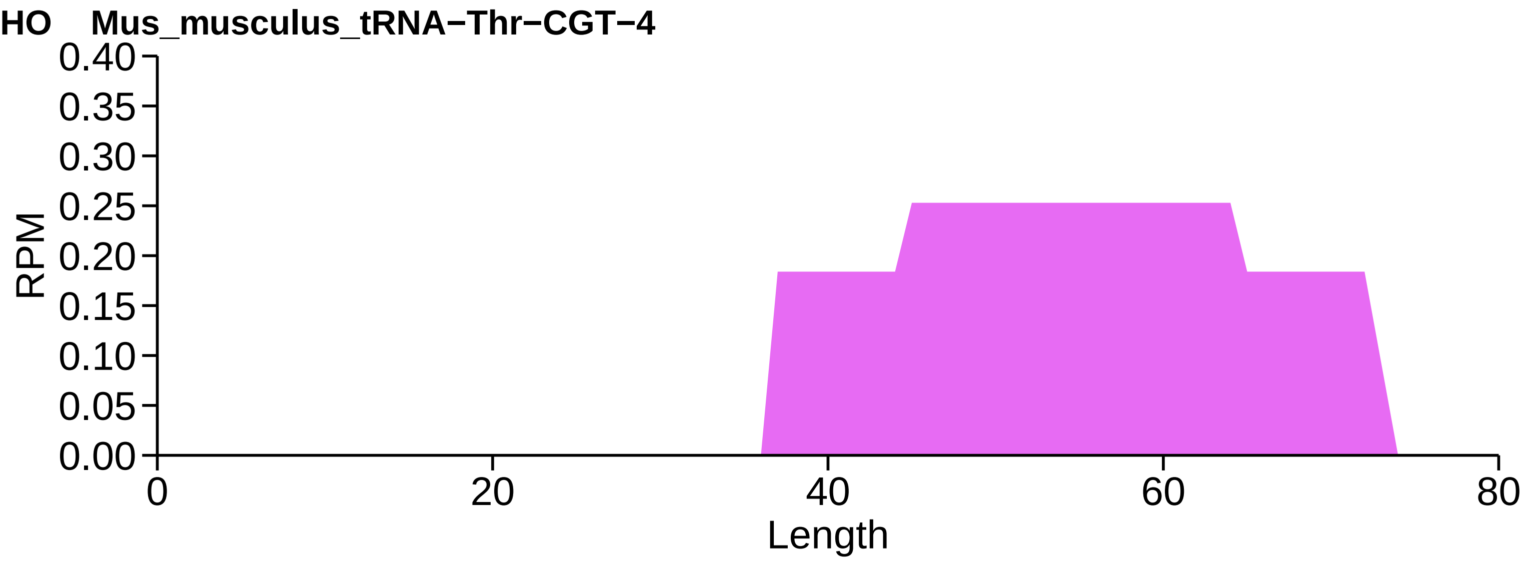
HO Mus_musculus_tRNA−Thr−CGT−4
0.40
0.35
0.30
0.25
0.20
0.15
0.10
0.05
0.00
RPM
0
20
40
60
80
Length

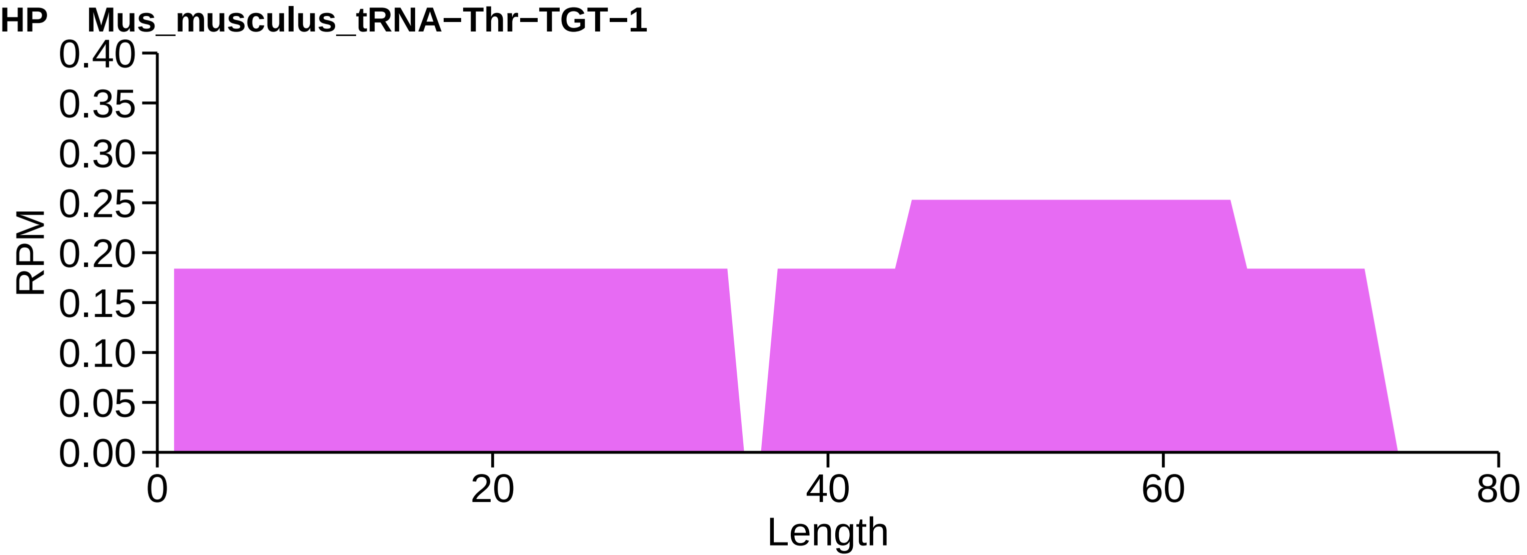

HP Mus_musculus_tRNA−Thr−TGT−1
RPM
0.40
0.35
0.30
0.25
0.20
0.15
0.10
0.05
0.00
0
20
40
60
80
Length

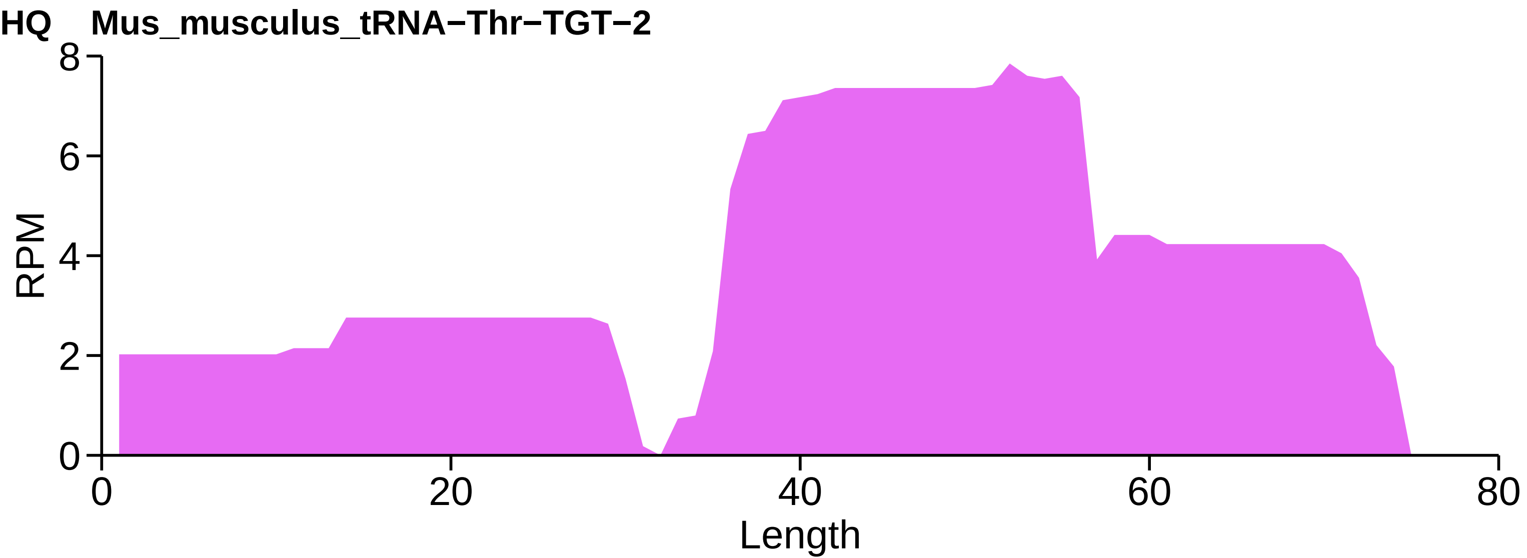
HQ Mus_musculus_tRNA−Thr−TGT−2
RPM
Length
0
2
4
6
8
0
20
40
60
80

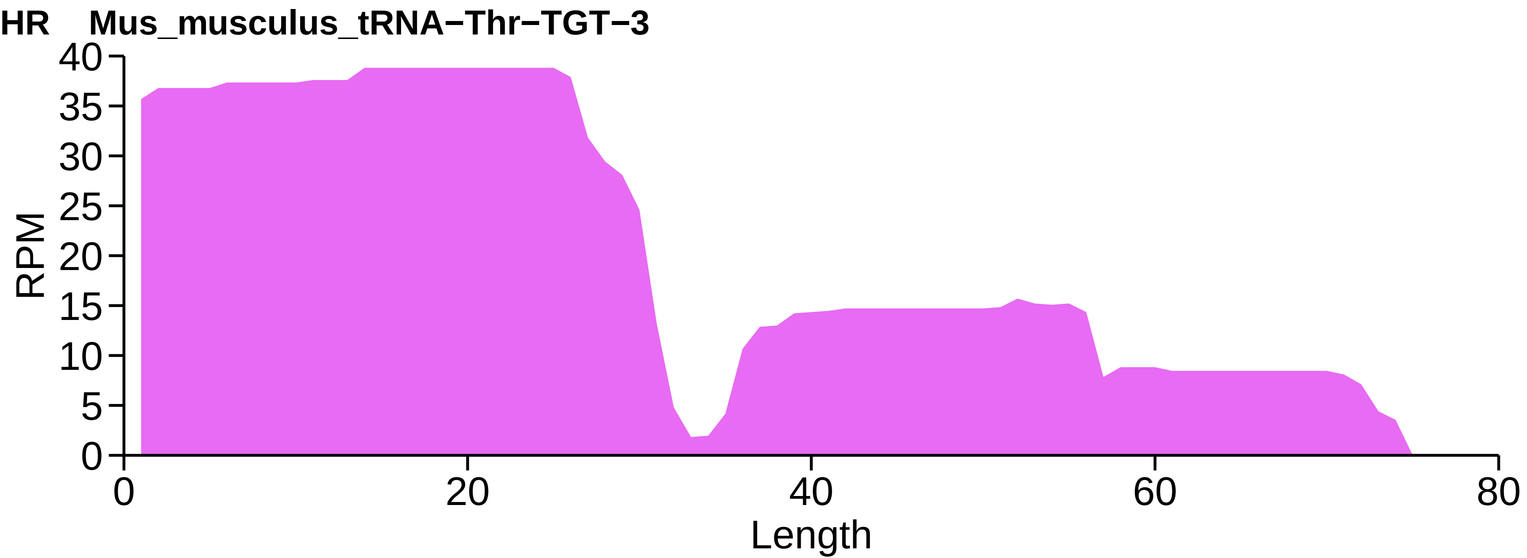
HR Mus_musculus_tRNA−Thr−TGT−3
RPM
Length
0
5
10
15
20
25
30
35
40
0
20
40
60
80

HS Mus_musculus_tRNA−Trp−CCA−1

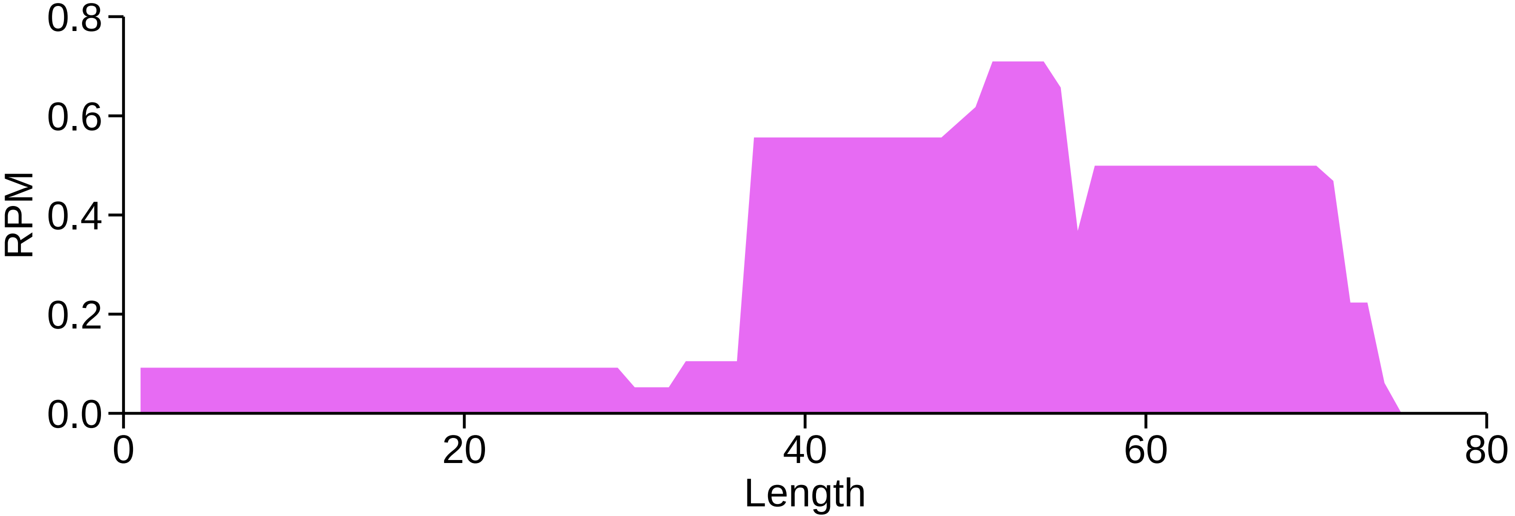

HT   Mus_musculus_tRNA−Trp−CCA−2

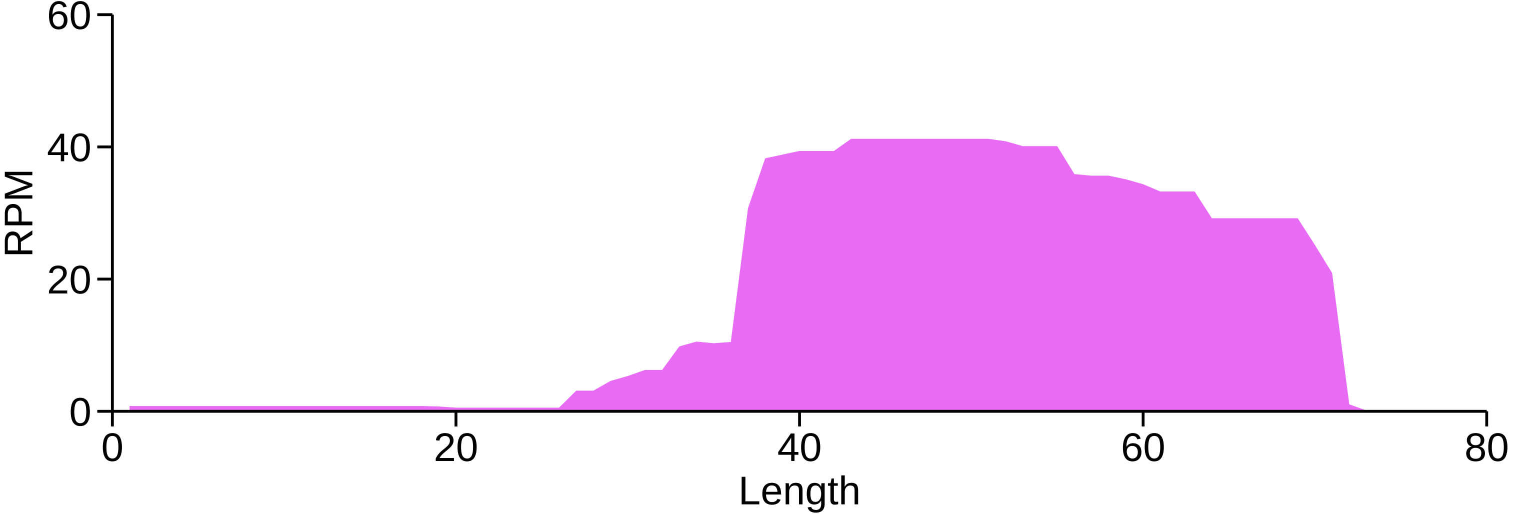

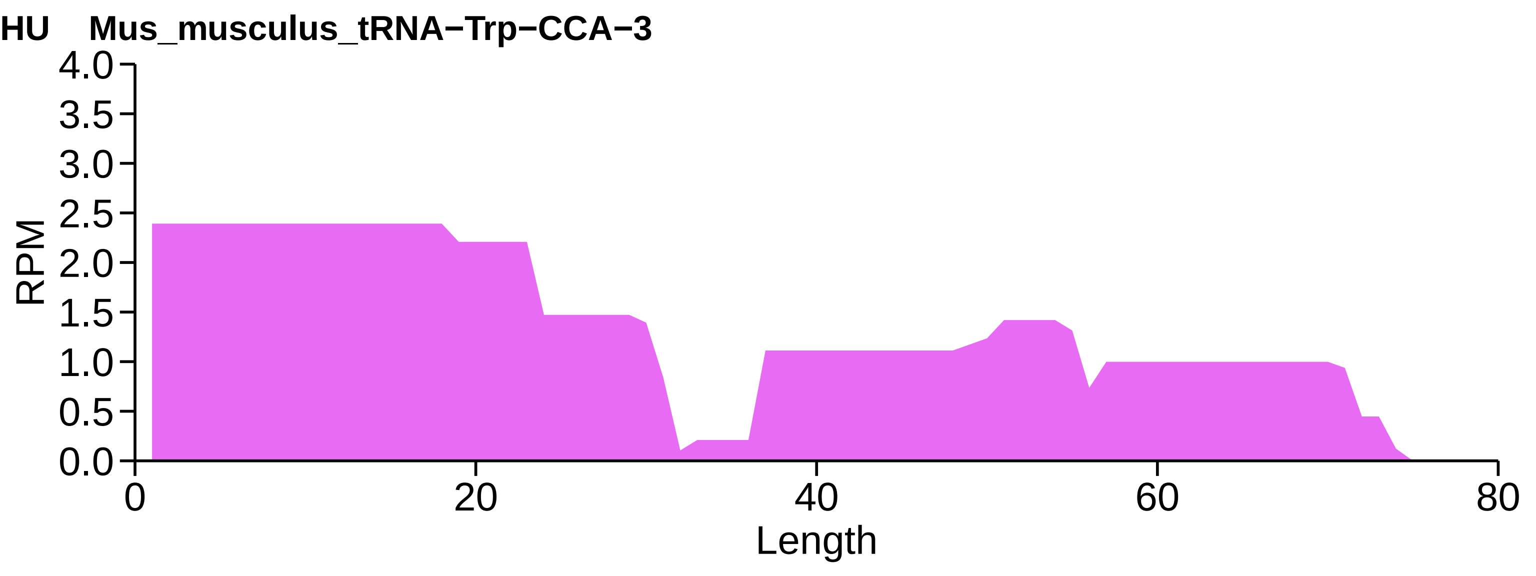
HU Mus_musculus_tRNA−Trp−CCA−3
RPM
0.0
0.5
1.0
1.5
2.0
2.5
3.0
3.5
4.0
0
20
40
60
80
Length

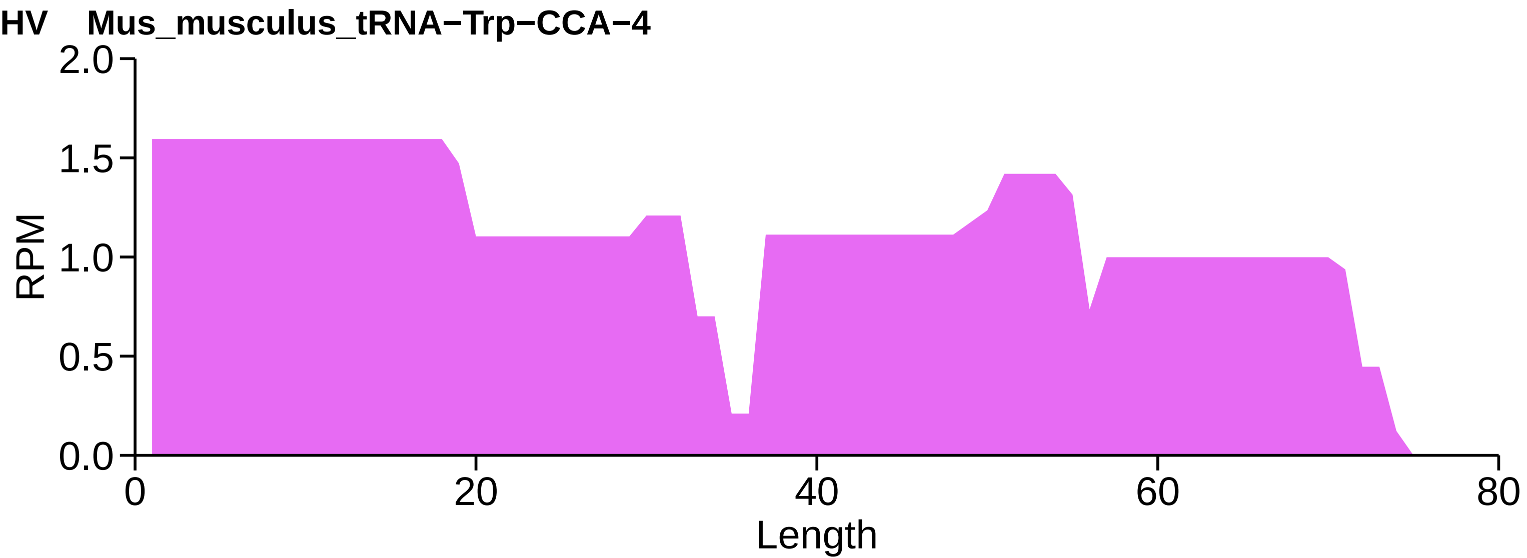

HV Mus_musculus_tRNA−Trp−CCA−4
2.0
1.5
1.0
0.5
0.0
RPM
0
20
40
60
80
Length

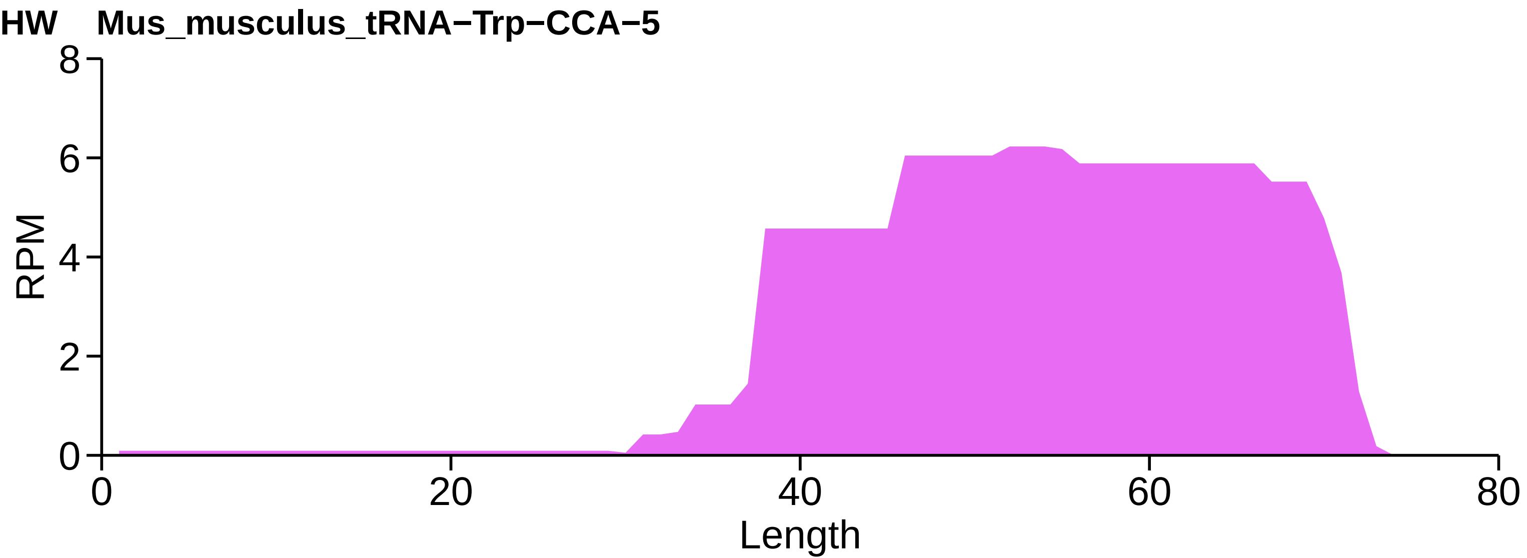

HW Mus_musculus_tRNA−Trp−CCA−5
RPM
8
6
4
2
0
0
20
40
60
80
Length

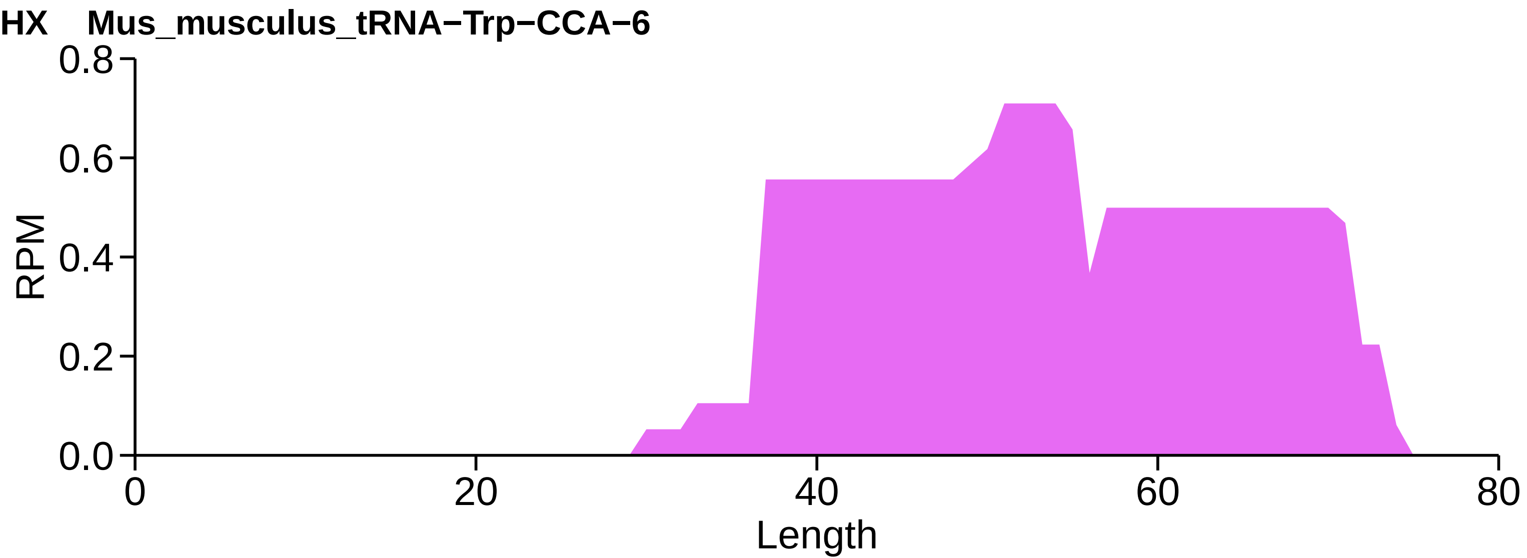
HX Mus_musculus_tRNA−Trp−CCA−6
RPM
0.0
0.2
0.4
0.6
0.8
0
20
40
60
80
Length

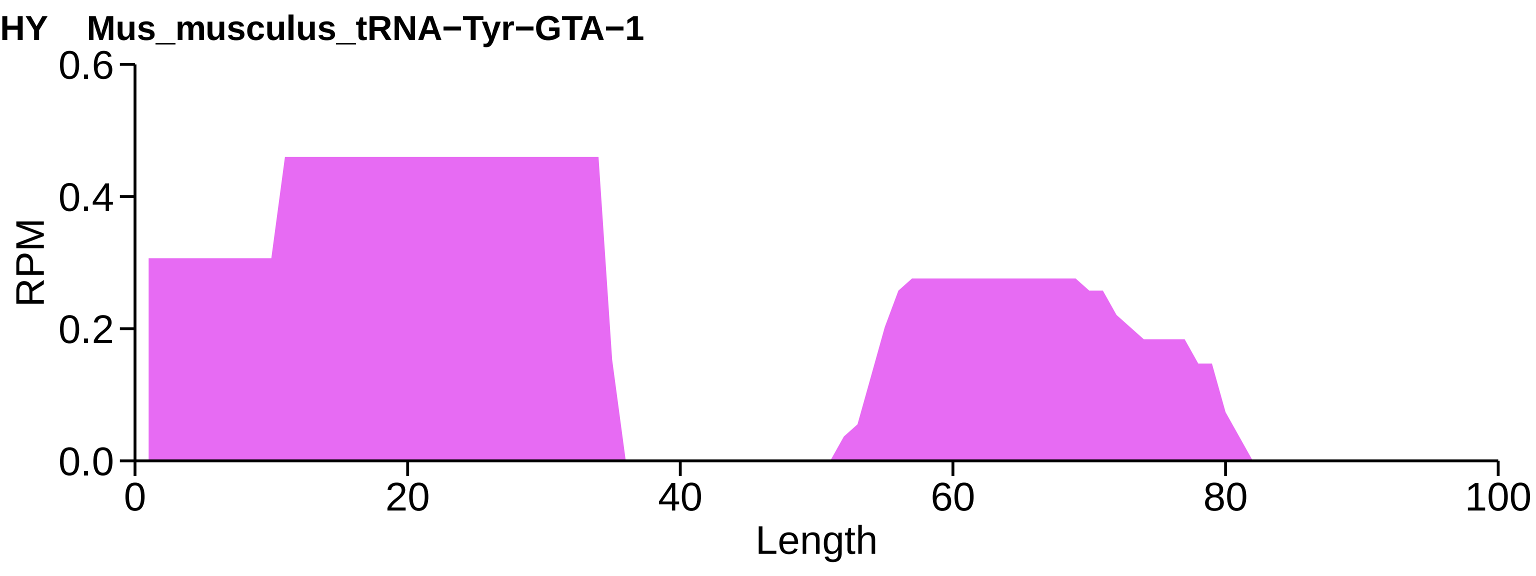
HY Mus_musculus_tRNA−Tyr−GTA−1
RPM
0.0
0.2
0.4
0.6
0
20
40
60
80
100
Length

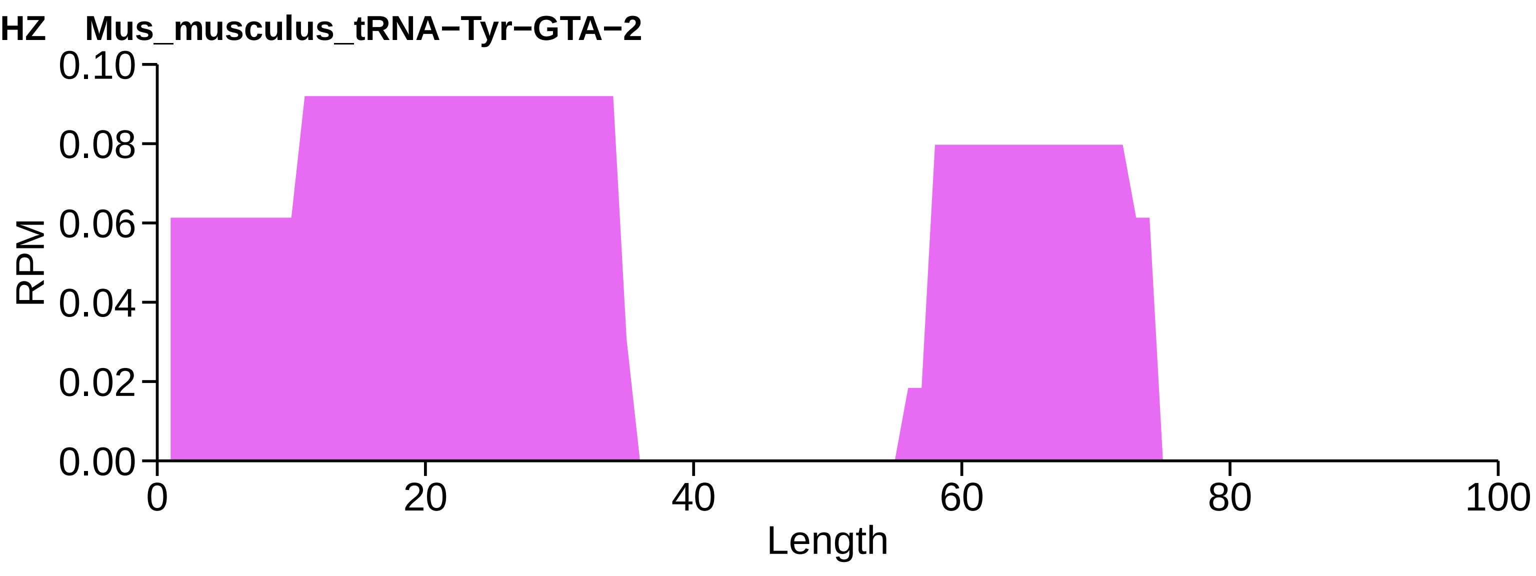
HZ Mus_musculus_tRNA−Tyr−GTA−2
RPM
0.00
0.02
0.04
0.06
0.08
0.10
0
20
40
60
80
100
Length

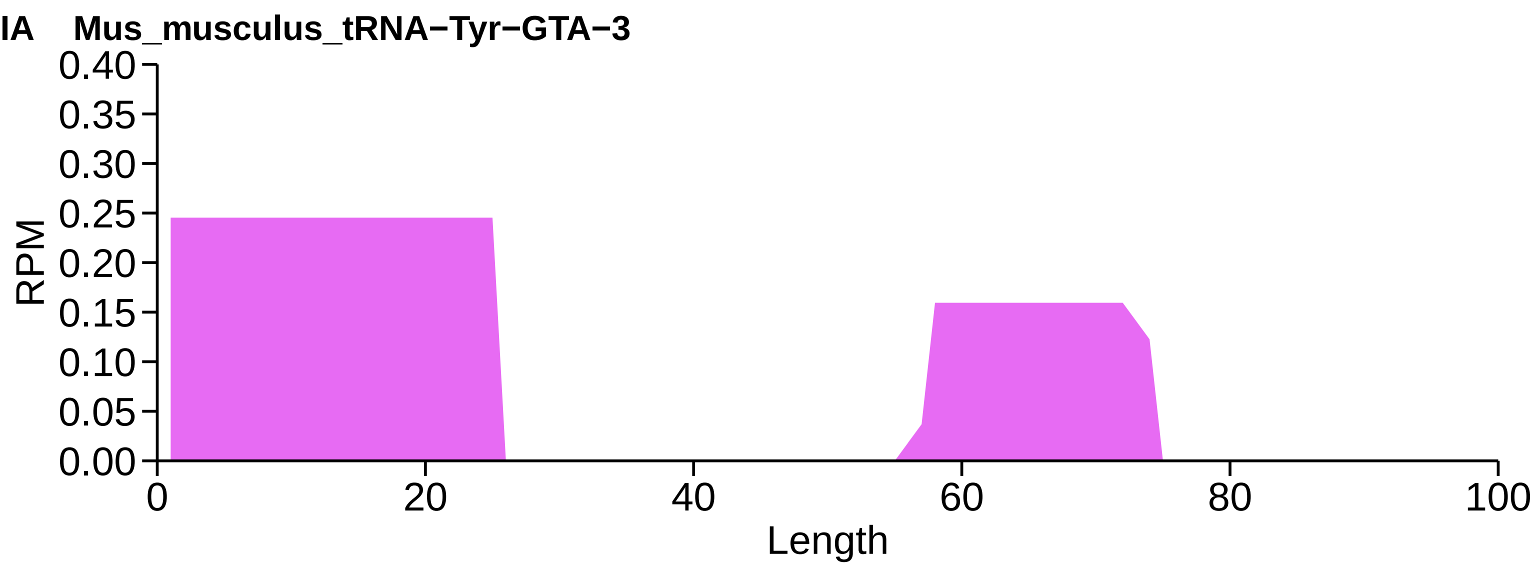

IA Mus_musculus_tRNA−Tyr−GTA−3
0.40
0.35
0.30
0.25
0.20
0.15
0.10
0.05
0.00
RPM
0
20
40
60
80
100
Length

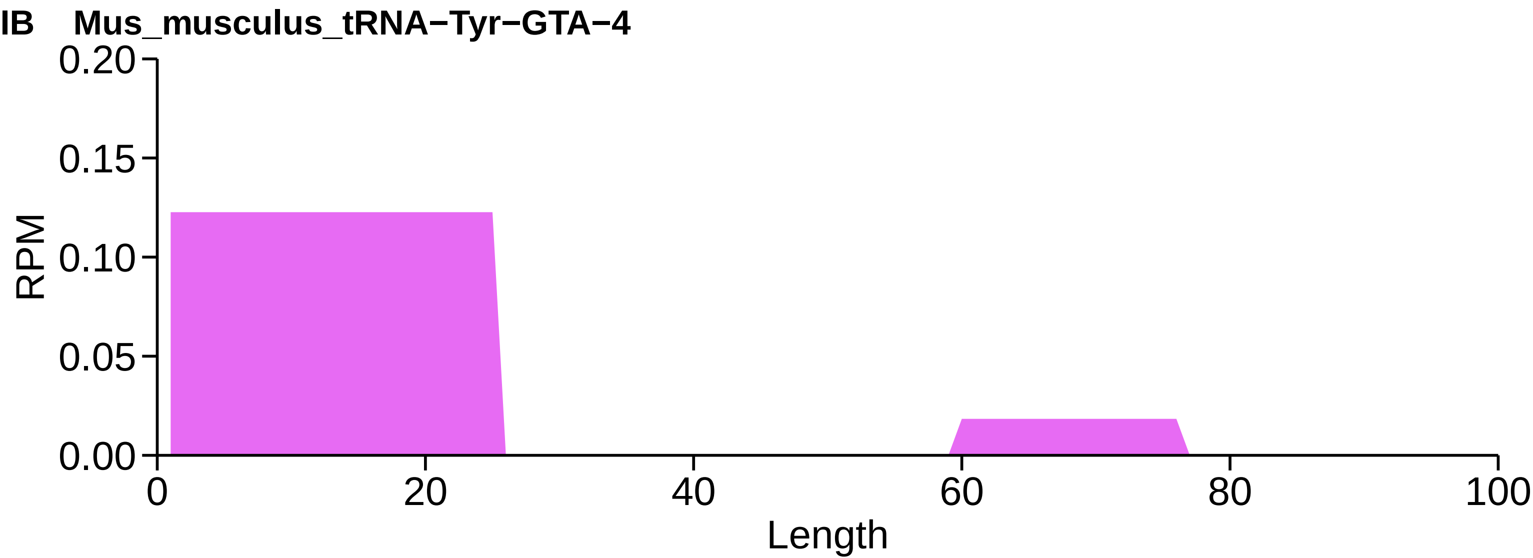

IB Mus_musculus_tRNA−Tyr−GTA−4
RPM
0.00
0.05
0.10
0.15
0.20
0
20
40
60
80
100
Length

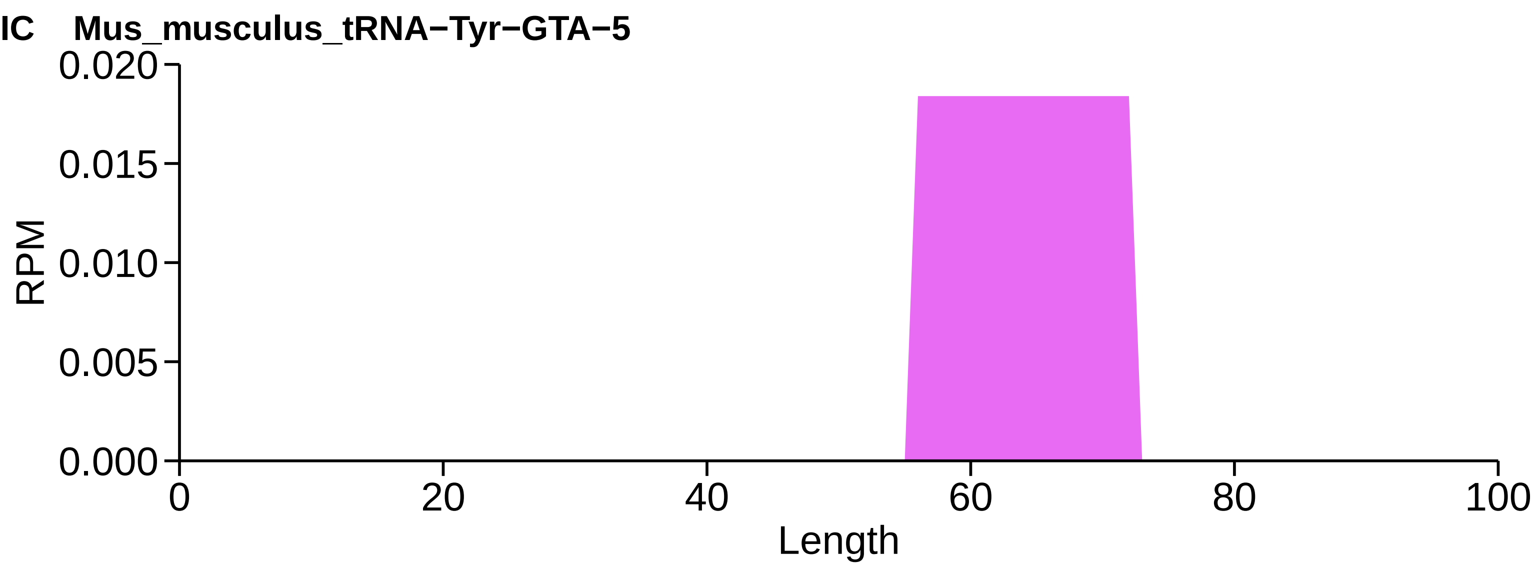
IC Mus_musculus_tRNA−Tyr−GTA−5
0.020
0.015
0.010
0.005
0.000
RPM
0
20
40
60
80
100
Length

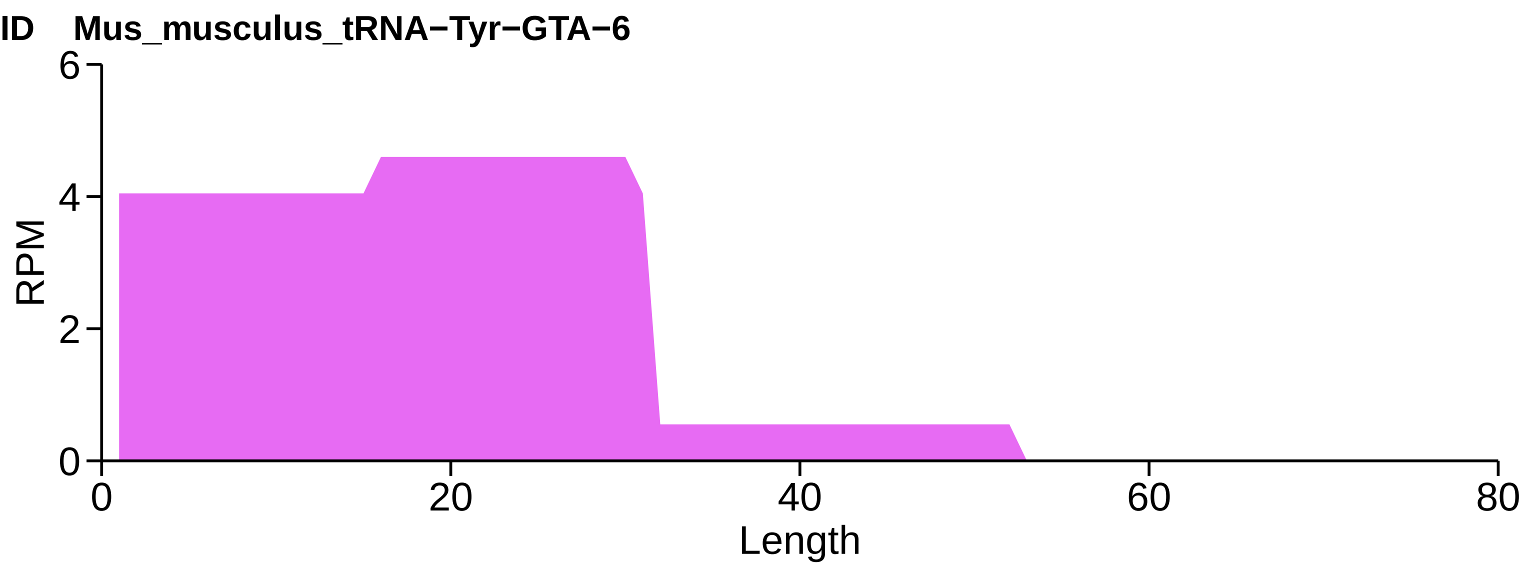

ID Mus_musculus_tRNA−Tyr−GTA−6
6
4
2
0
RPM
0
20
40
60
80
Length

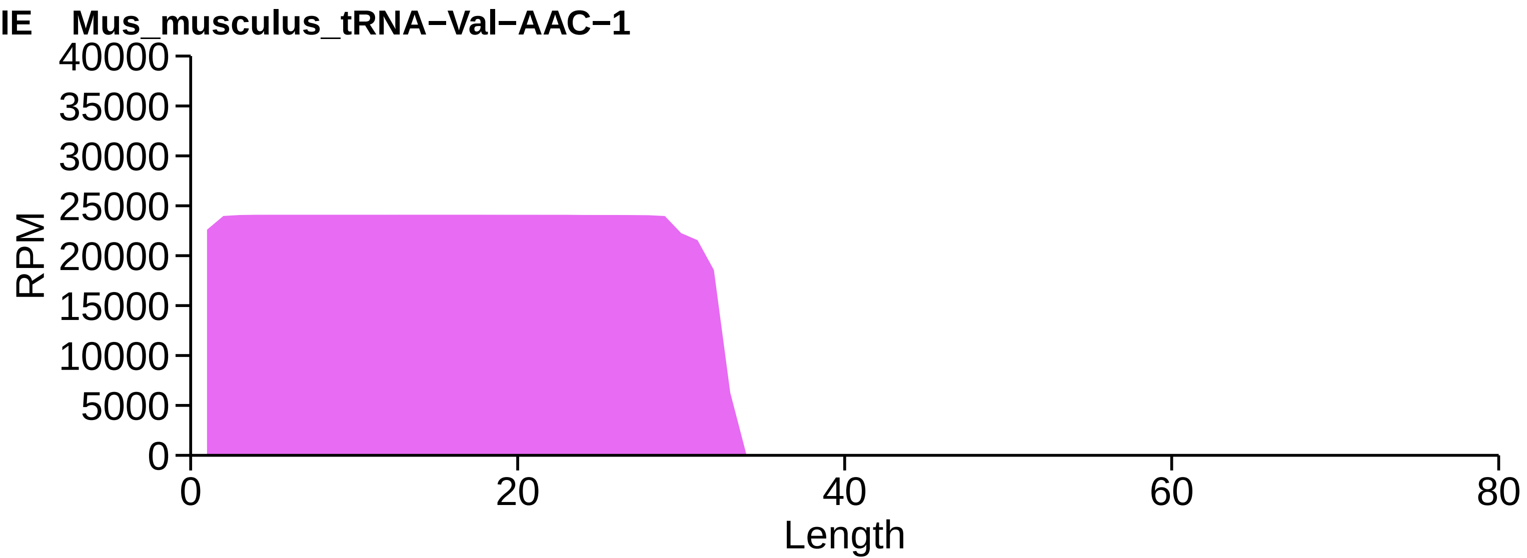

IE Mus_musculus_tRNA−Val−AAC−1
40000
35000
30000
25000
20000
15000
10000
5000
0
RPM
0
20
40
60
80
Length

IF Mus_musculus_tRNA−Val−AAC−2

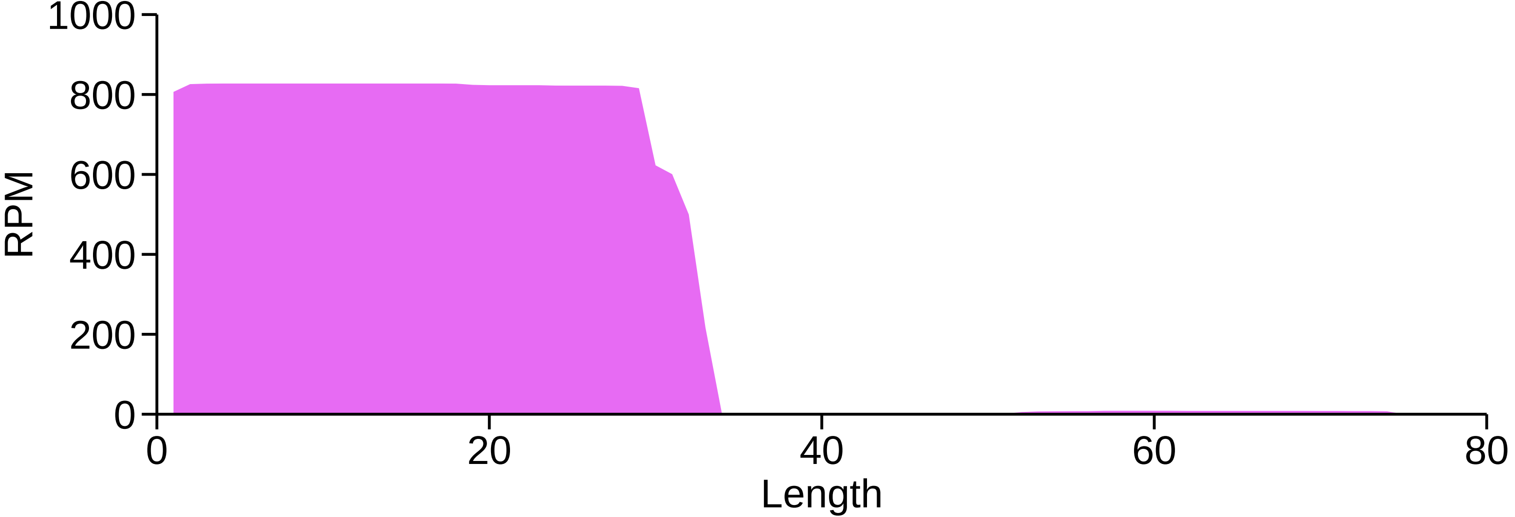

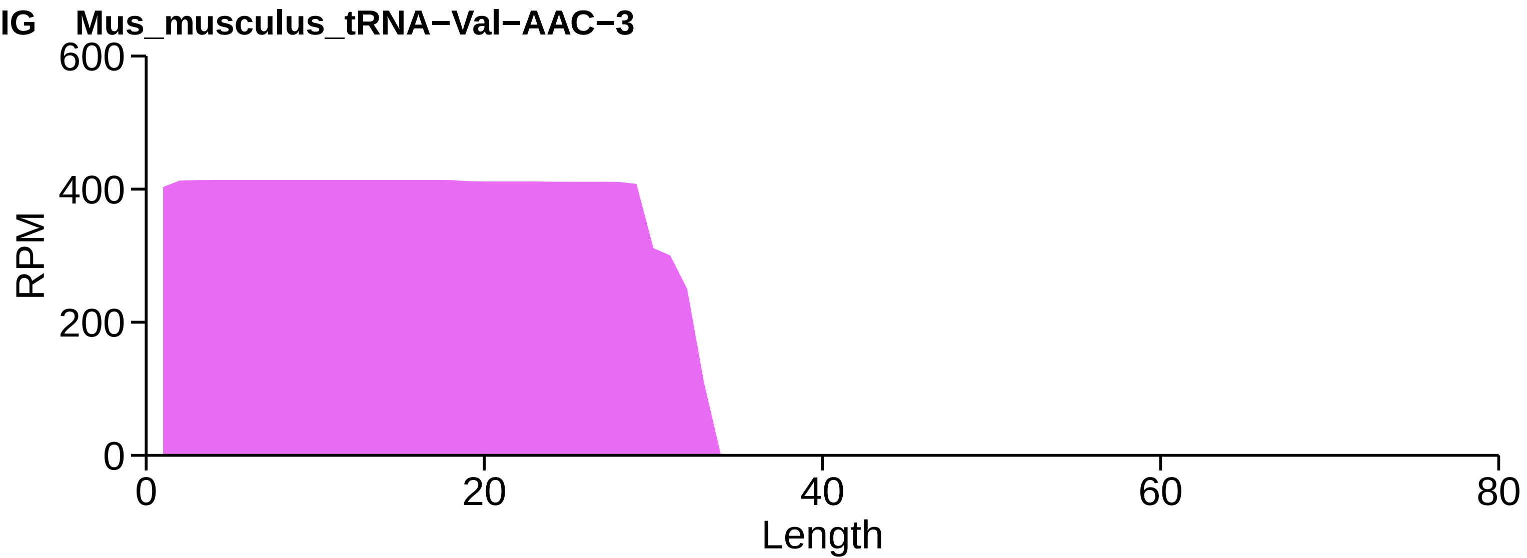
IG Mus_musculus_tRNA−Val−AAC−3
RPM
0
200
400
600
0
20
40
60
80
Length

IH **Mus_musculus_tRNA−Val−AAC−4**

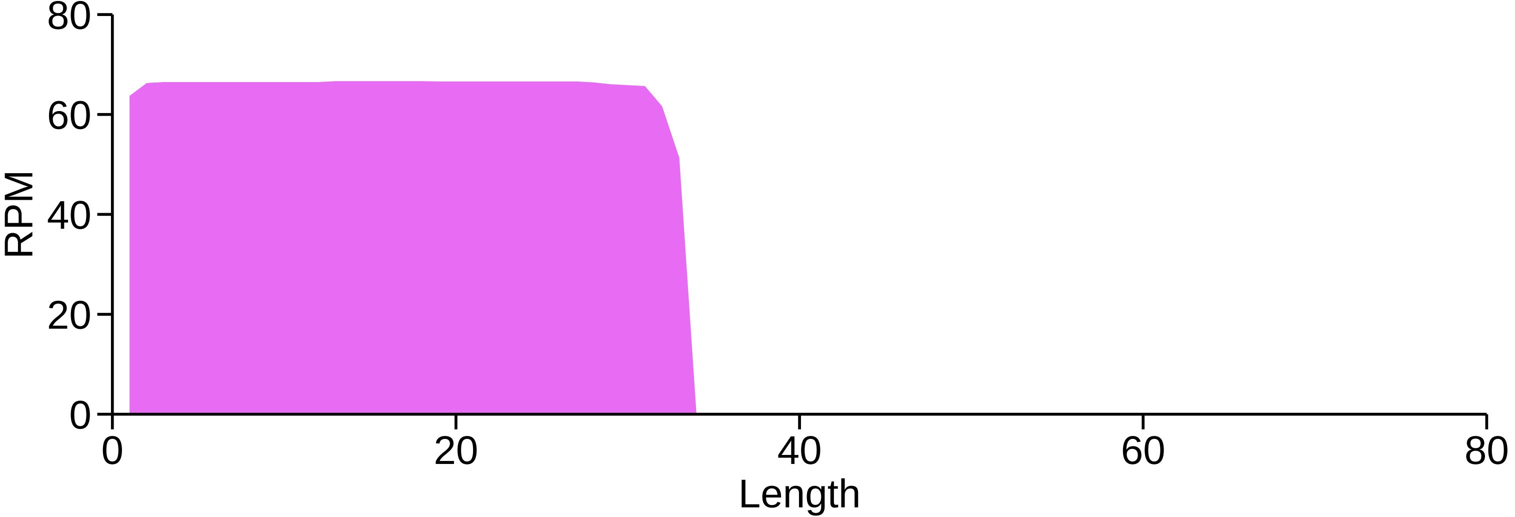

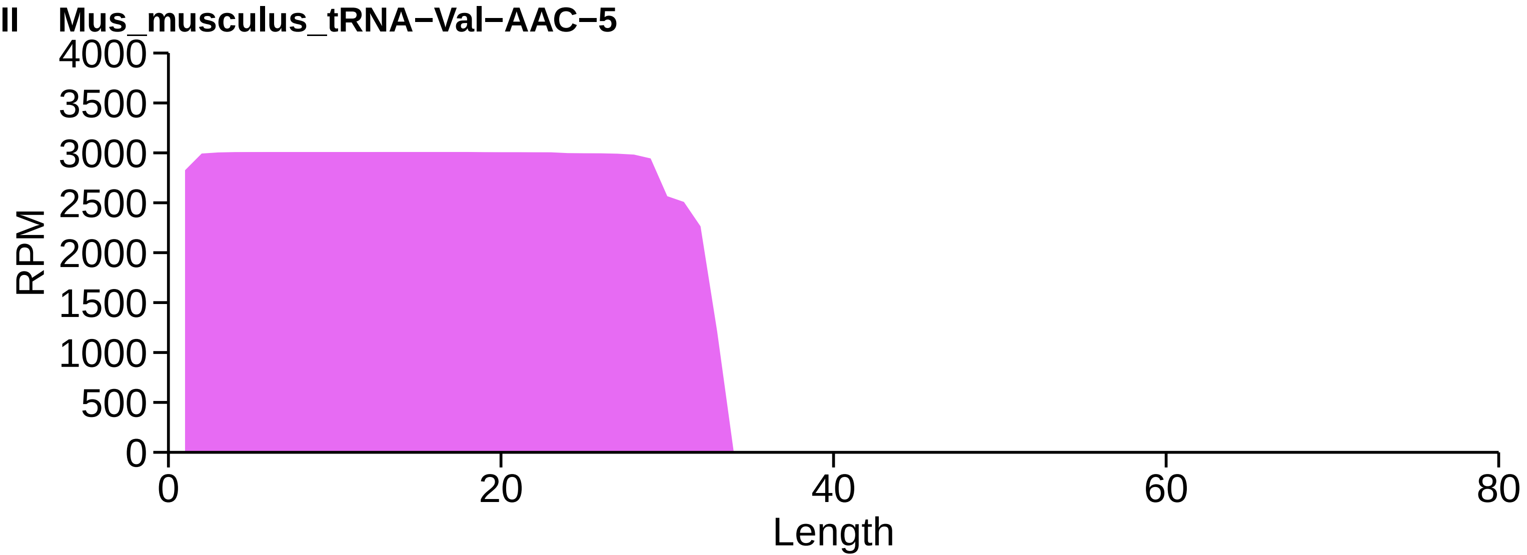

ll Mus_musculus_tRNA−Val−AAC−5
4000
3500
3000
2500
2000
1500
1000
500
0
RPM
0
20
40
60
80
Length

IJ Mus_musculus_tRNA−Val−CAC−1

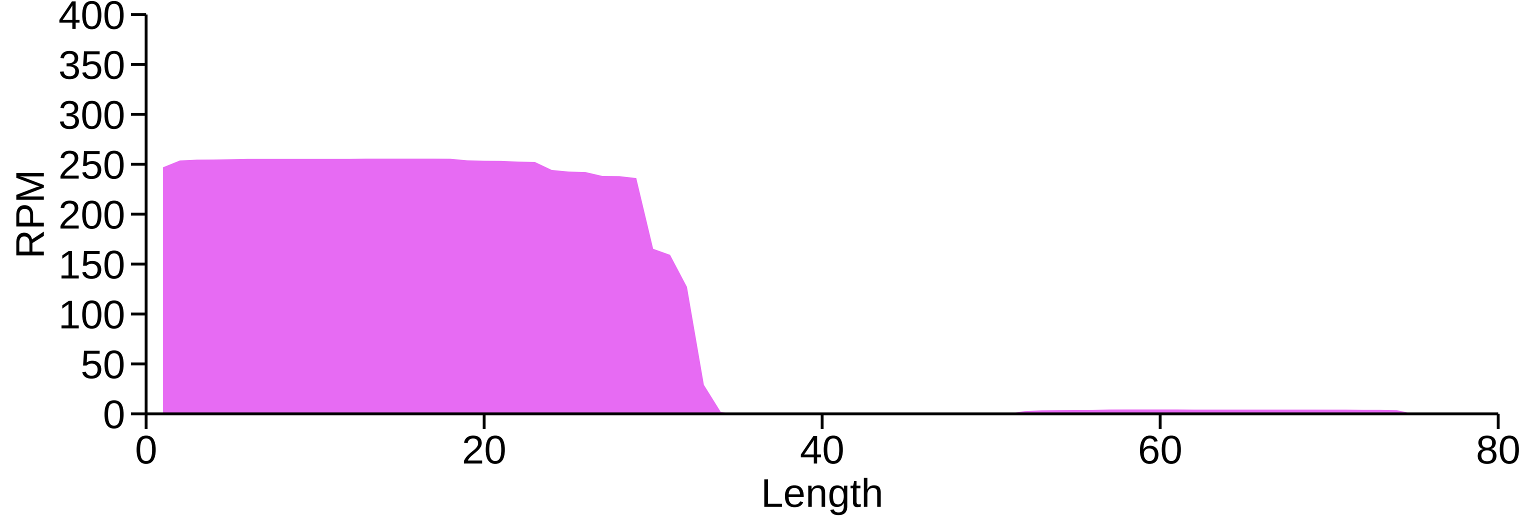

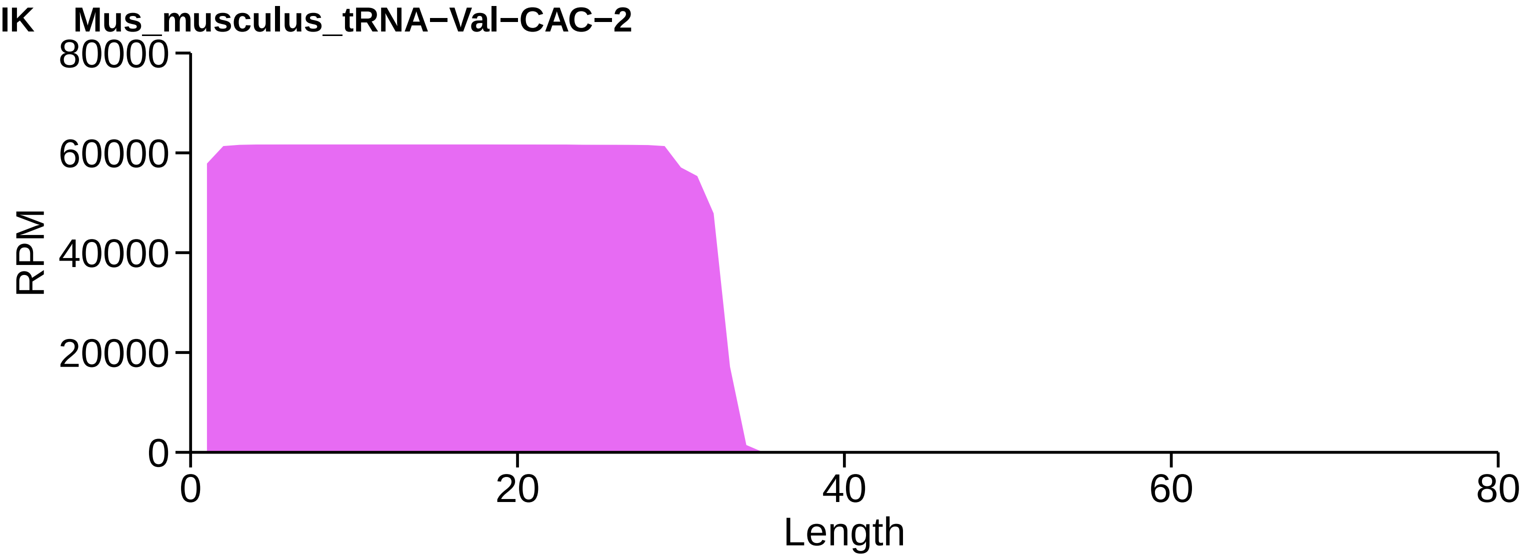
IK Mus_musculus_tRNA−Val−CAC−2
80000
60000
40000
20000
0
RPM
0
20
40
60
80
Length

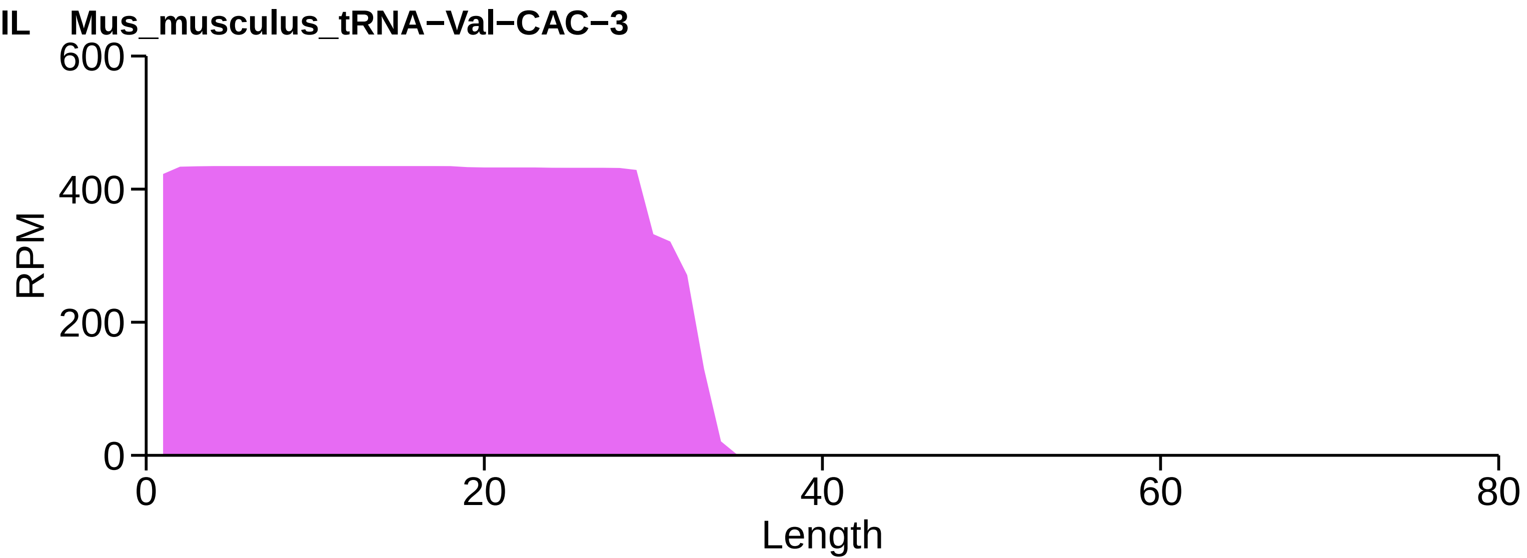

IL Mus_musculus_tRNA−Val−CAC−3
600
400
200
0
RPM
0
20
40
60
80
Length

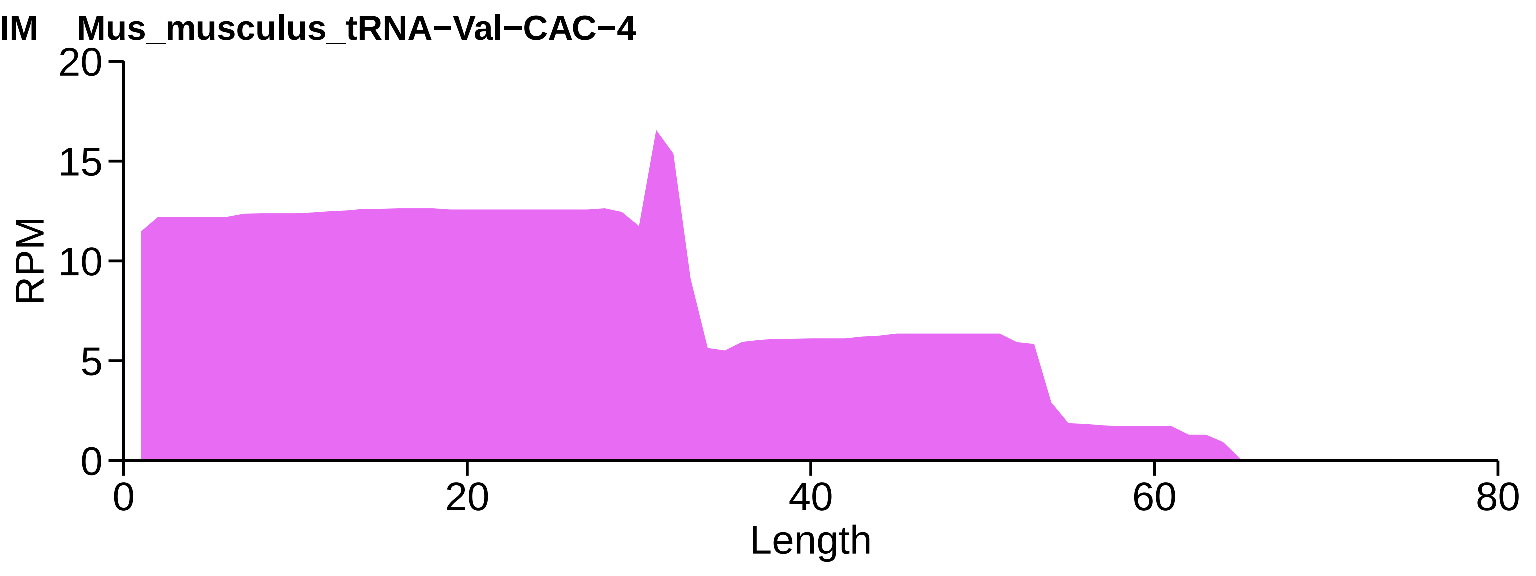
IM Mus_musculus_tRNA−Val−CAC−4
RPM
0
5
10
15
20
0
20
40
60
80
Length

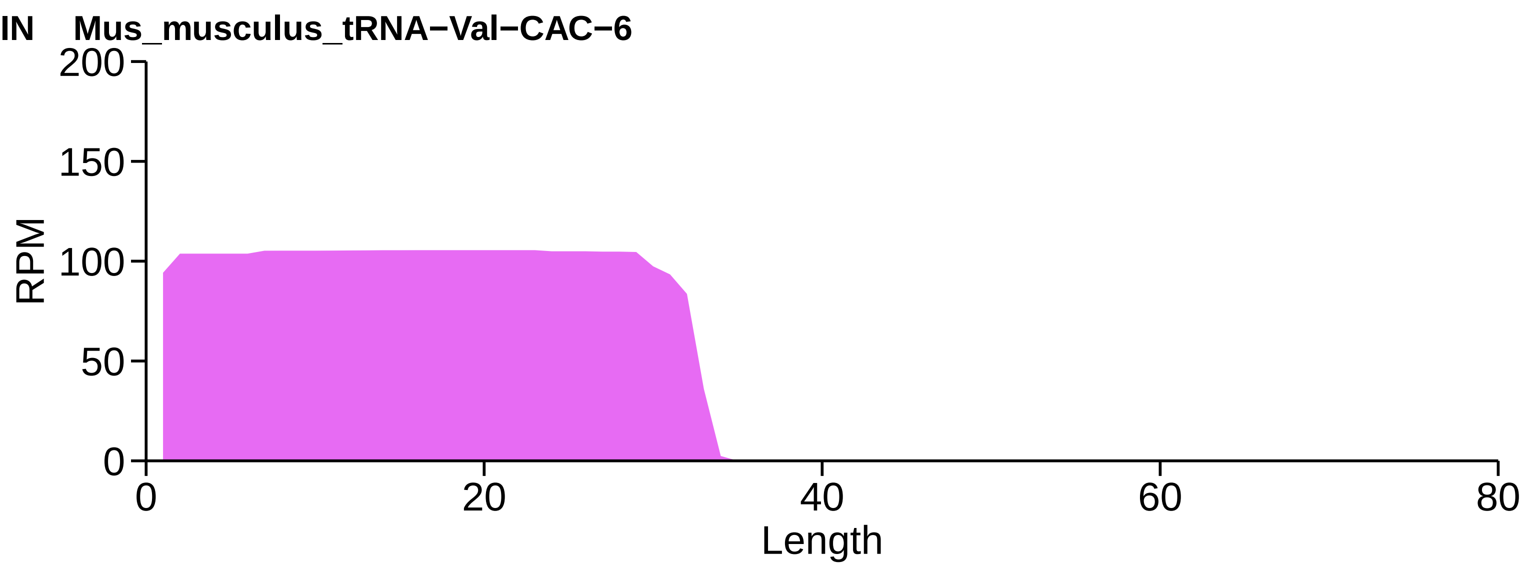
IN Mus_musculus_tRNA−Val−CAC−6
RPM
200
150
100
50
0
0
20
40
60
80
Length

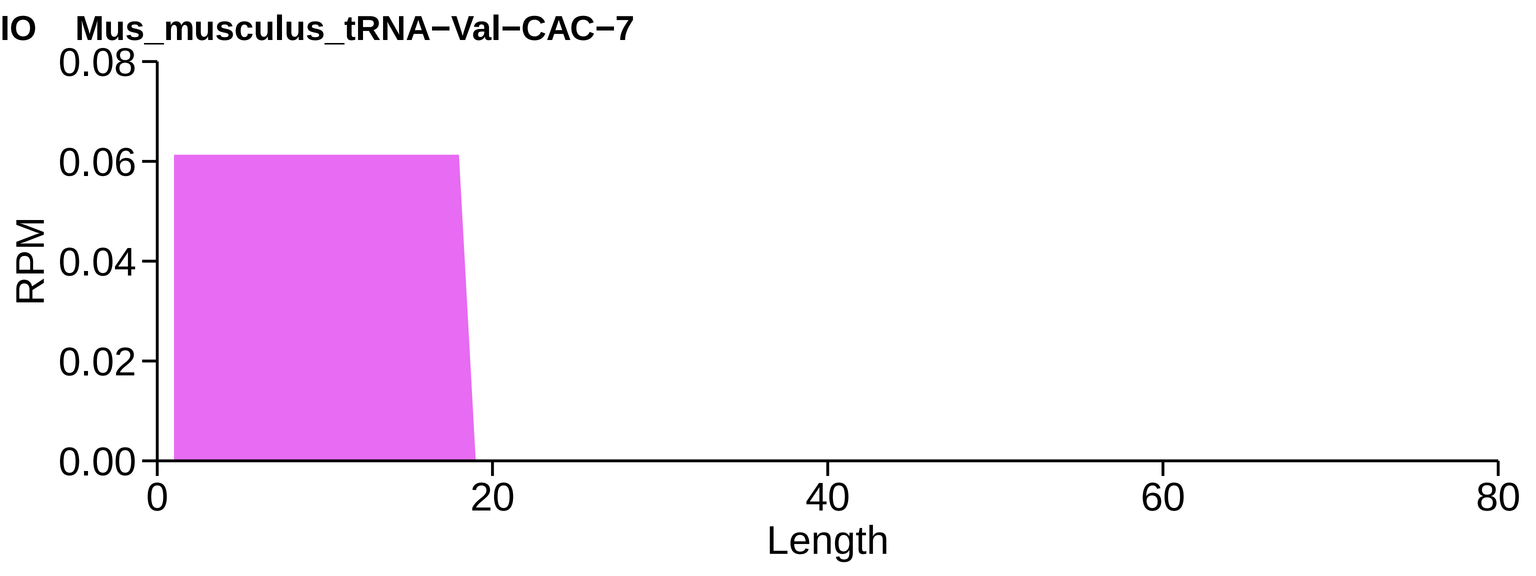
IO Mus_musculus_tRNA−Val−CAC−7
0.08
0.06
0.04
0.02
0.00
RPM
0
20
40
60
80
Length

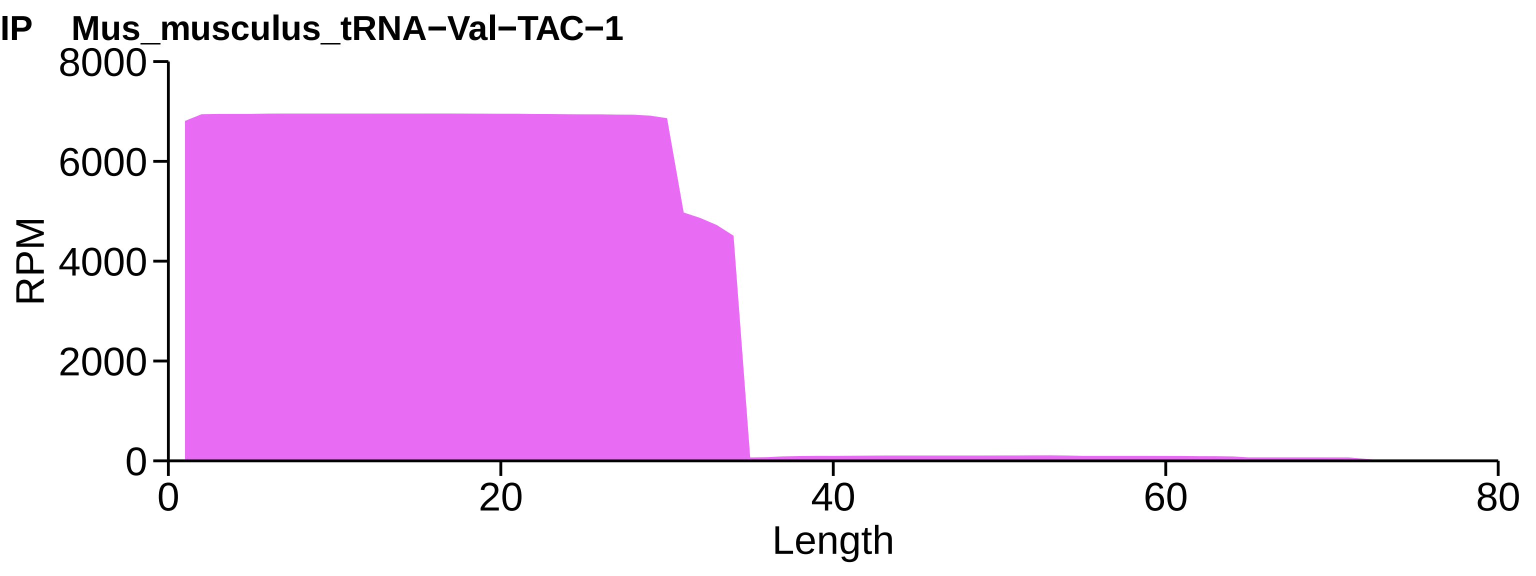
IP Mus_musculus_tRNA−Val−TAC−1
RPM
8000
6000
4000
2000
0
0
20
40
60
80
Length

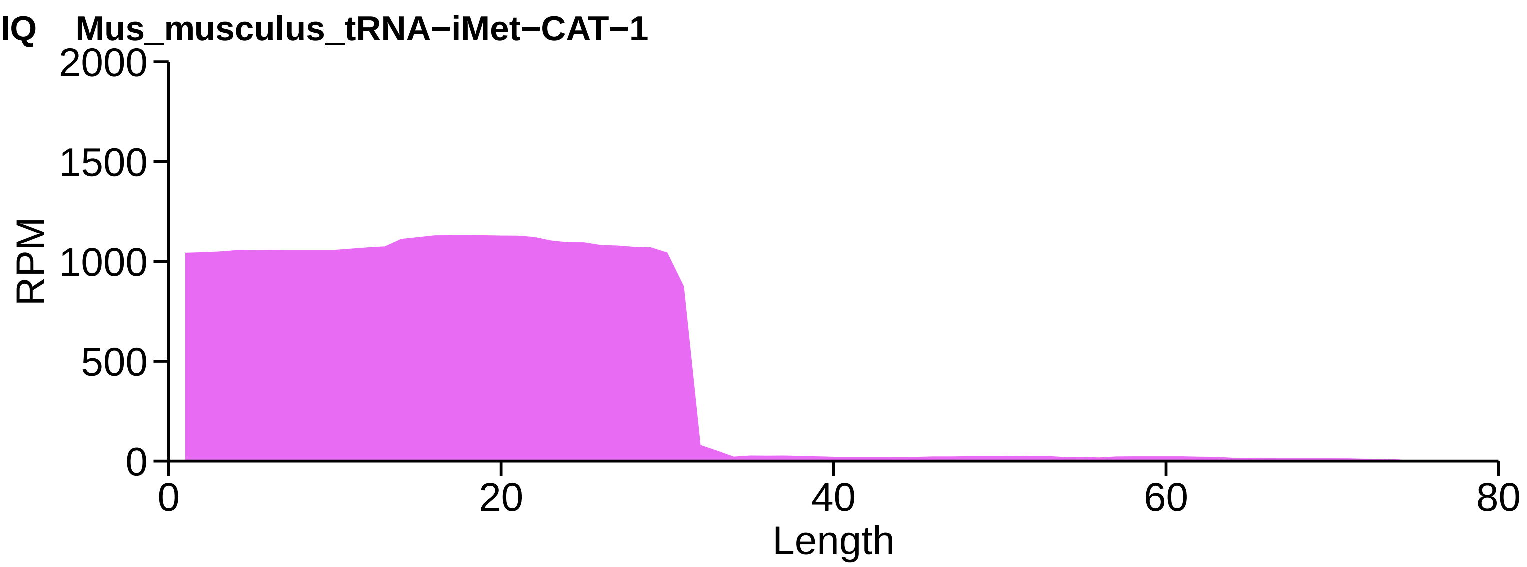

IQ Mus_musculus_tRNA−iMet−CAT−1
2000
1500
1000
500
0
RPM
0
20
40
60
80
Length

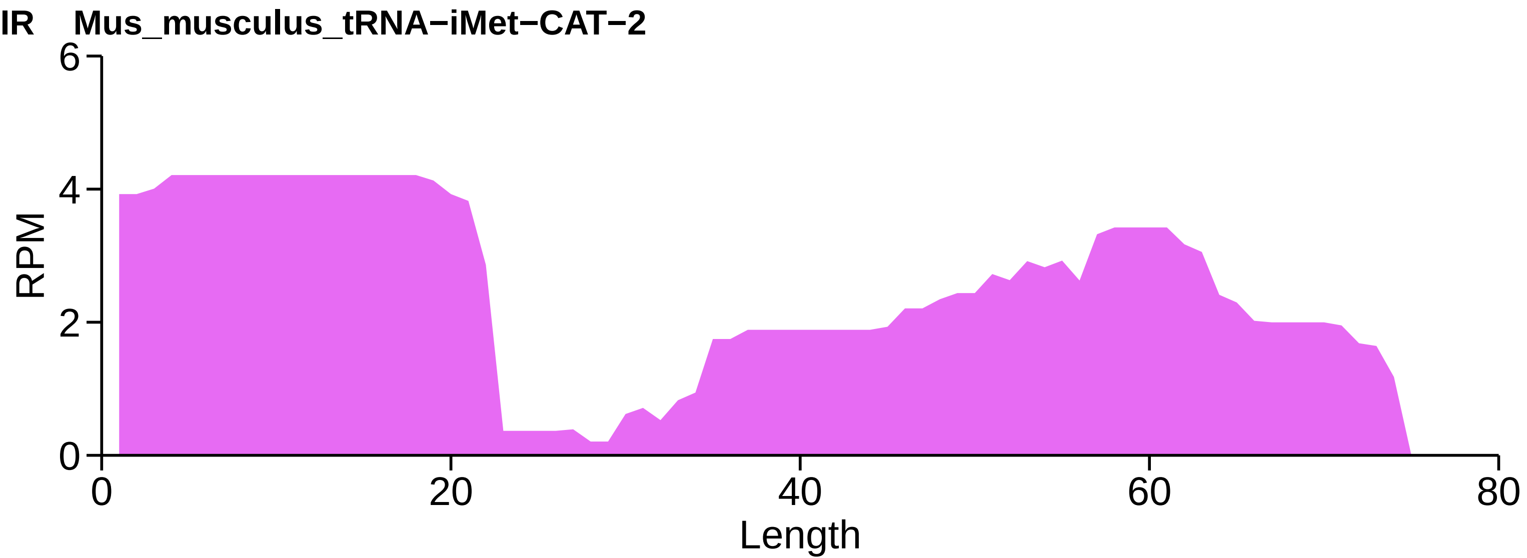
IR Mus_musculus_tRNA−iMet−CAT−2
RPM
0
2
4
6
0
20
40
60
80
Length

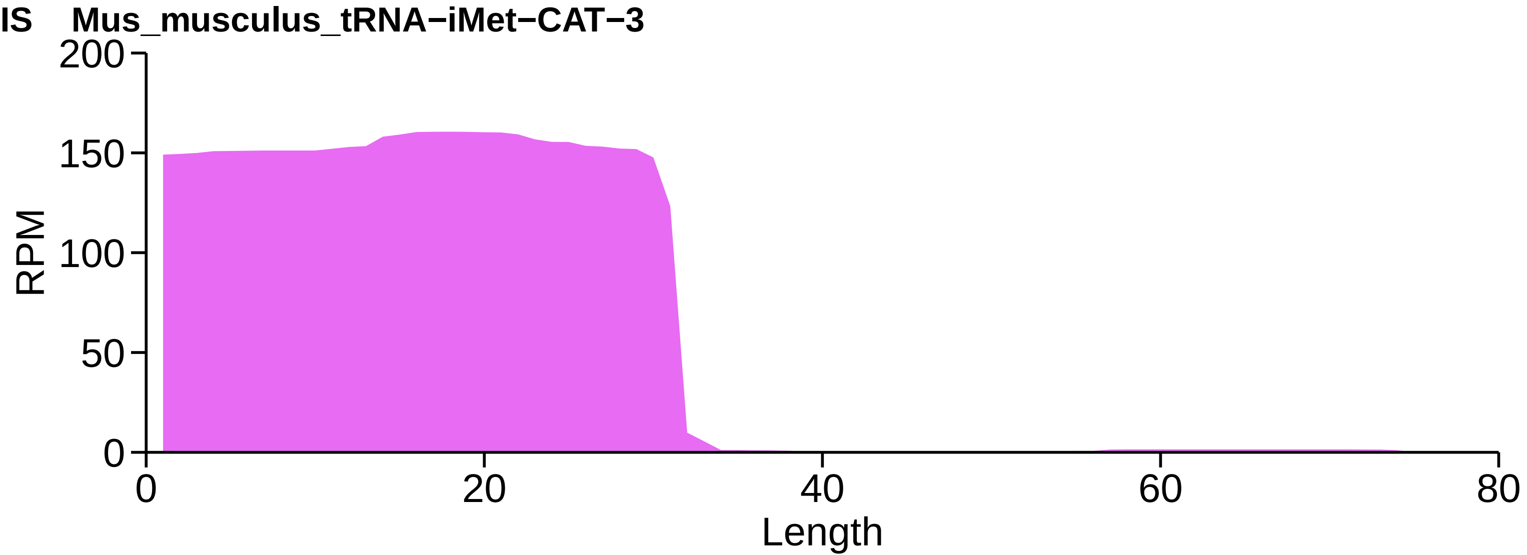

IS Mus_musculus_tRNA−iMet−CAT−3
200
150
100
50
0
RPM
0
20
40
60
80
Length
