# Supplementary material for: SPORTS1.0: A Tool for Annotating and Profiling Non-coding RNAs Optimized for rRNA- and tRNA-derived Small RNAs
Source: Genomics Proteomics Bioinformatics. 2018 May 3;16(2):144–51. doi: 10.1016/j.gpb.2018.04.004 (PMC6112344; doi:10.1016/j.gpb.2018.04.004)
Supplement: Supplementary Figure S2 — The mouse bone marrow cell tsRNA mapping results against tRNA loci revealed by SPORTS1.0Mapping result for each annotated tsRNA was provided. [file mmc2.pdf]

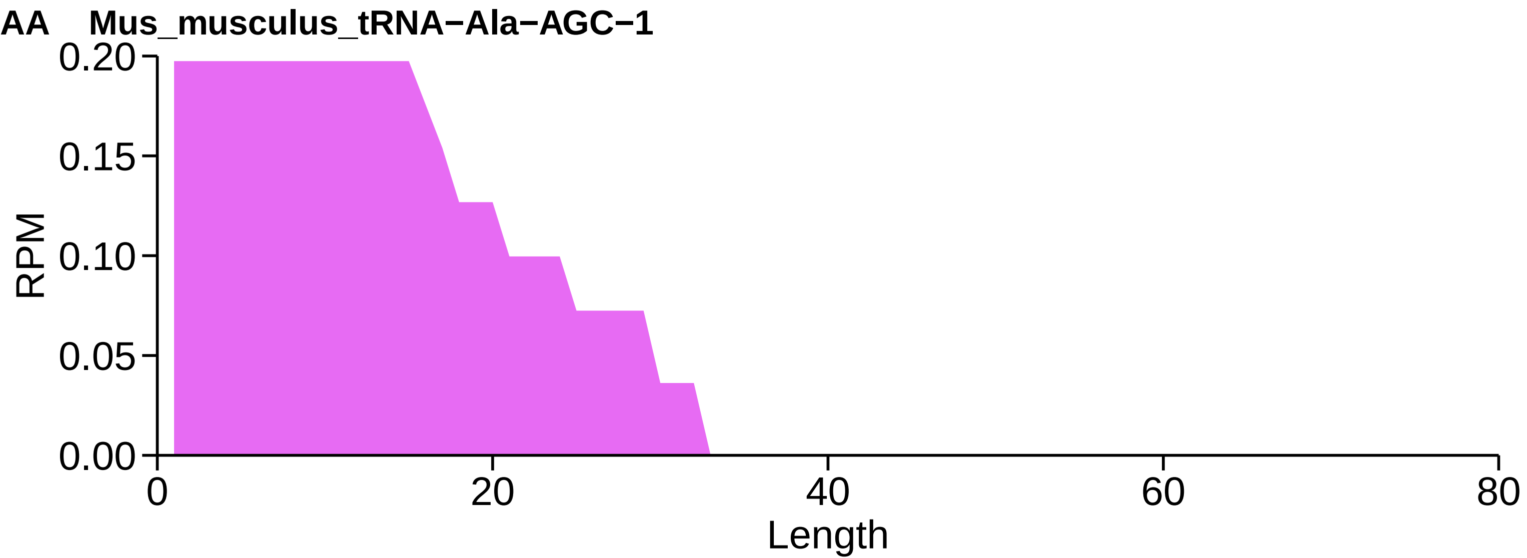
AA Mus_musculus_tRNA−Ala−AGC−1
RPM
0.20
0.15
0.10
0.05
0.00
0
20
40
60
80
Length

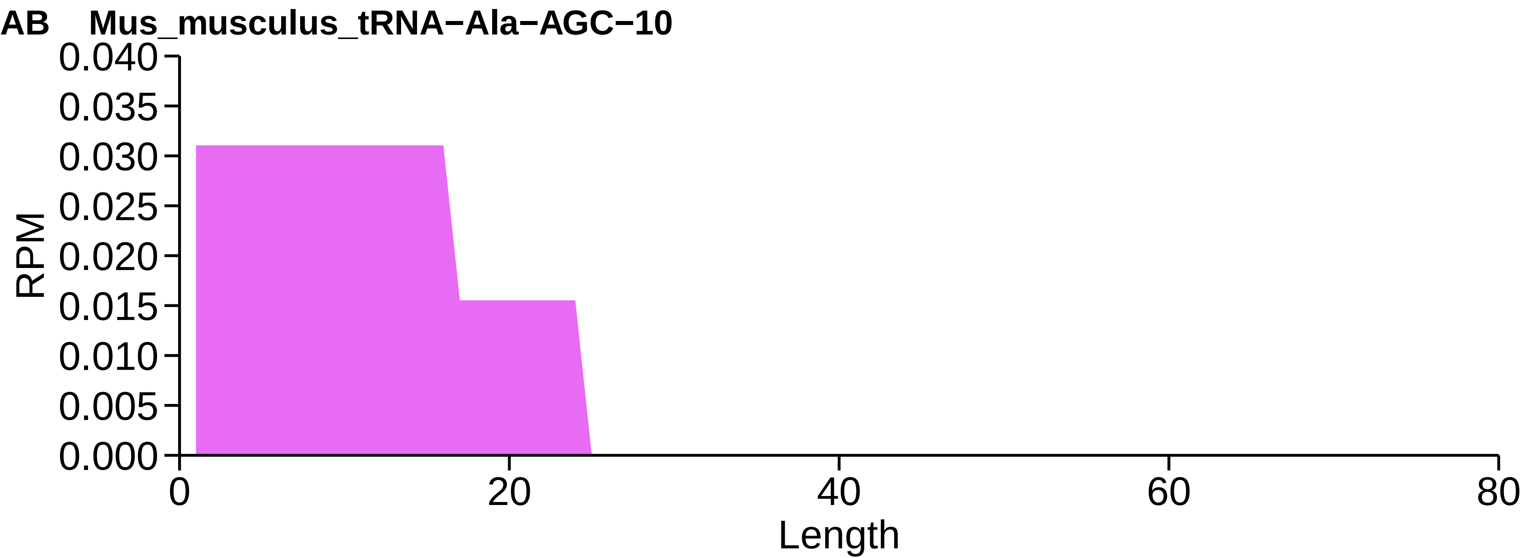
AB Mus_musculus_tRNA−Ala−AGC−10
0.040
0.035
0.030
0.025
0.020
0.015
0.010
0.005
0.000
RPM
0
20
40
60
80
Length

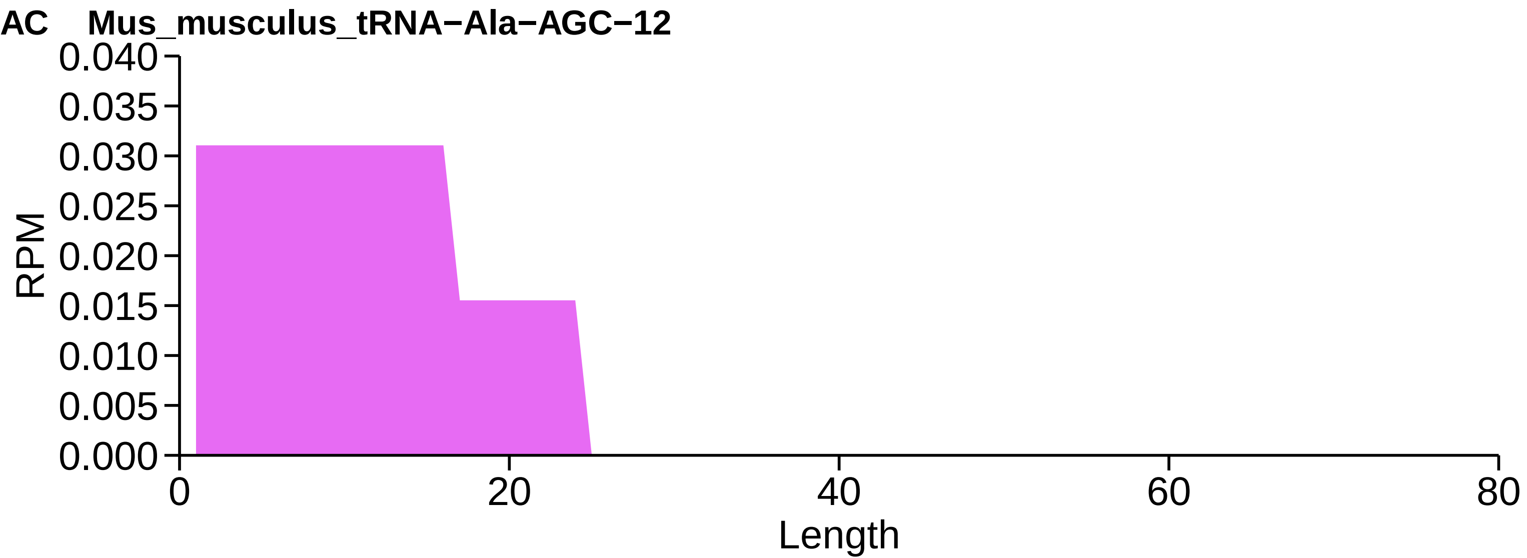

AC Mus_musculus_tRNA−Ala−AGC−12
0.040
0.035
0.030
0.025
0.020
0.015
0.010
0.005
0.000
RPM
0
20
40
60
80
Length

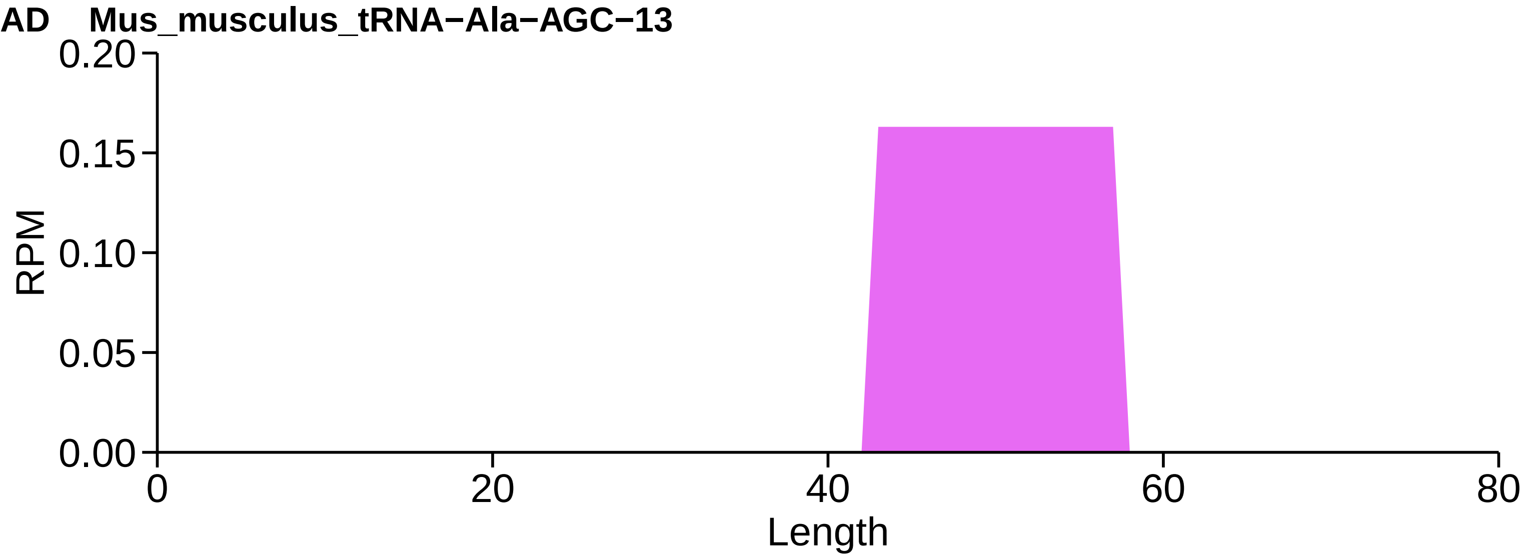
AD Mus_musculus_tRNA−Ala−AGC−13
RPM
0.20
0.15
0.10
0.05
0.00
0
20
40
60
80
Length

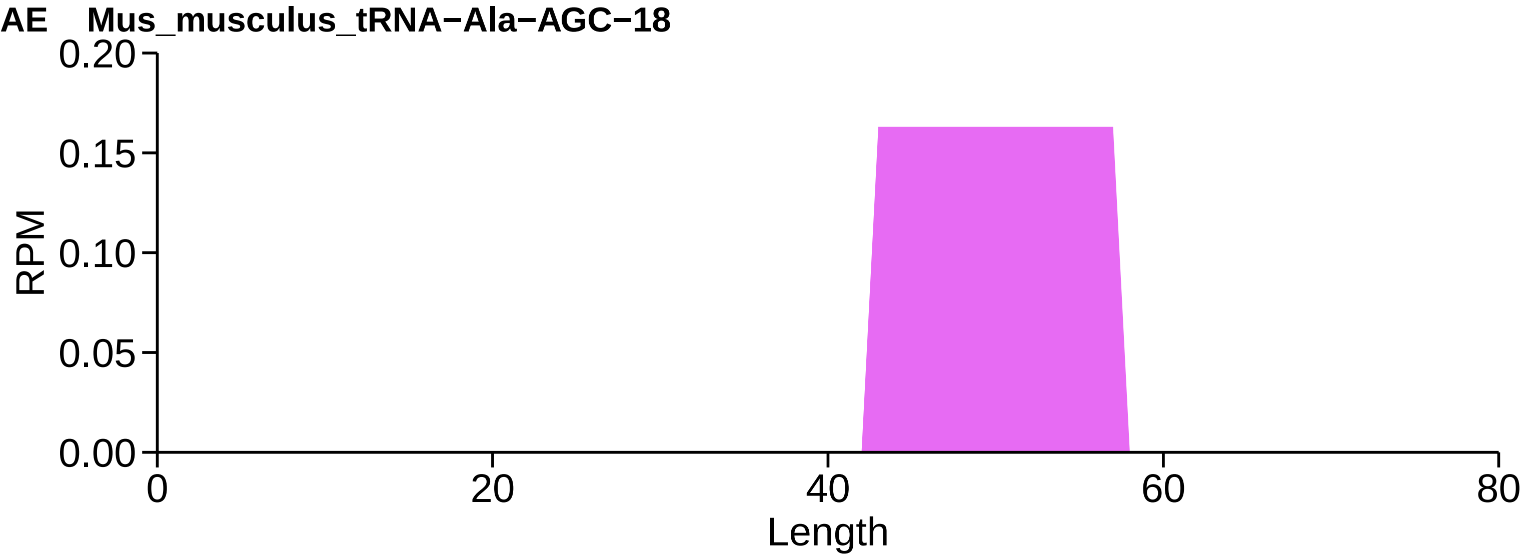
AE Mus_musculus_tRNA−Ala−AGC−18
0.20
0.15
0.10
0.05
0.00
RPM
0
20
40
60
80
Length

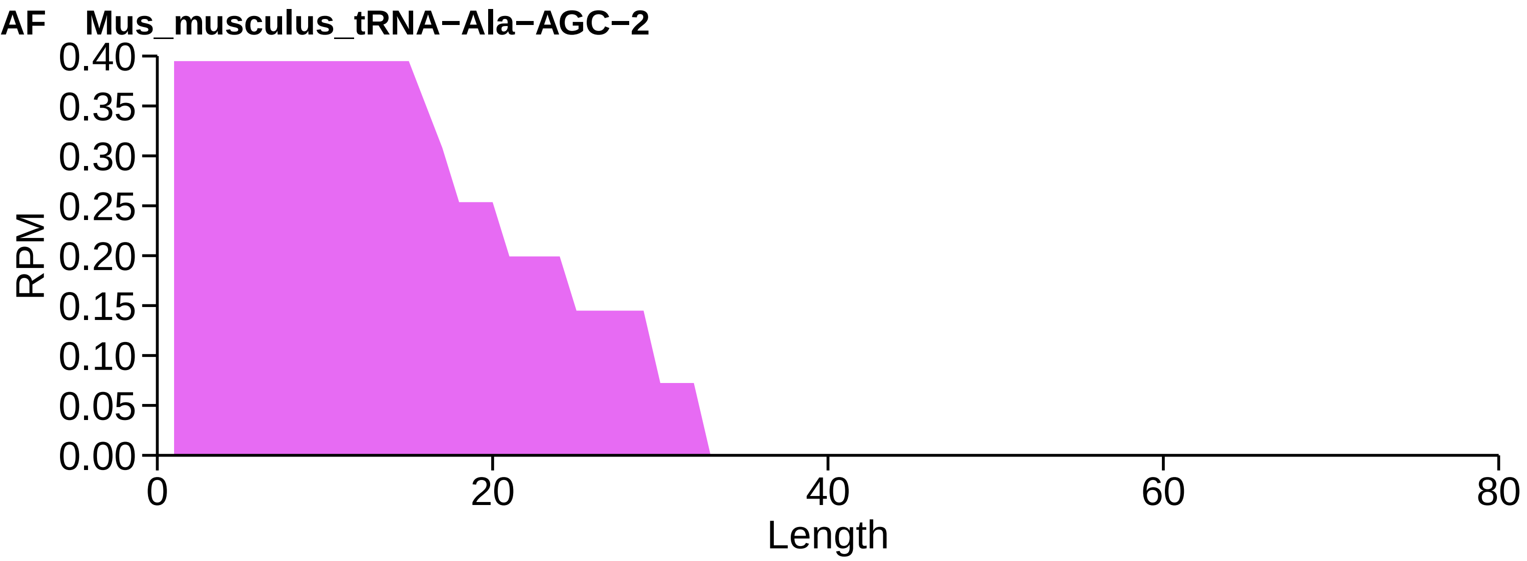
AF Mus_musculus_tRNA−Ala−AGC−2
RPM
0.00
0.05
0.10
0.15
0.20
0.25
0.30
0.35
0.40
0
20
40
60
80
Length

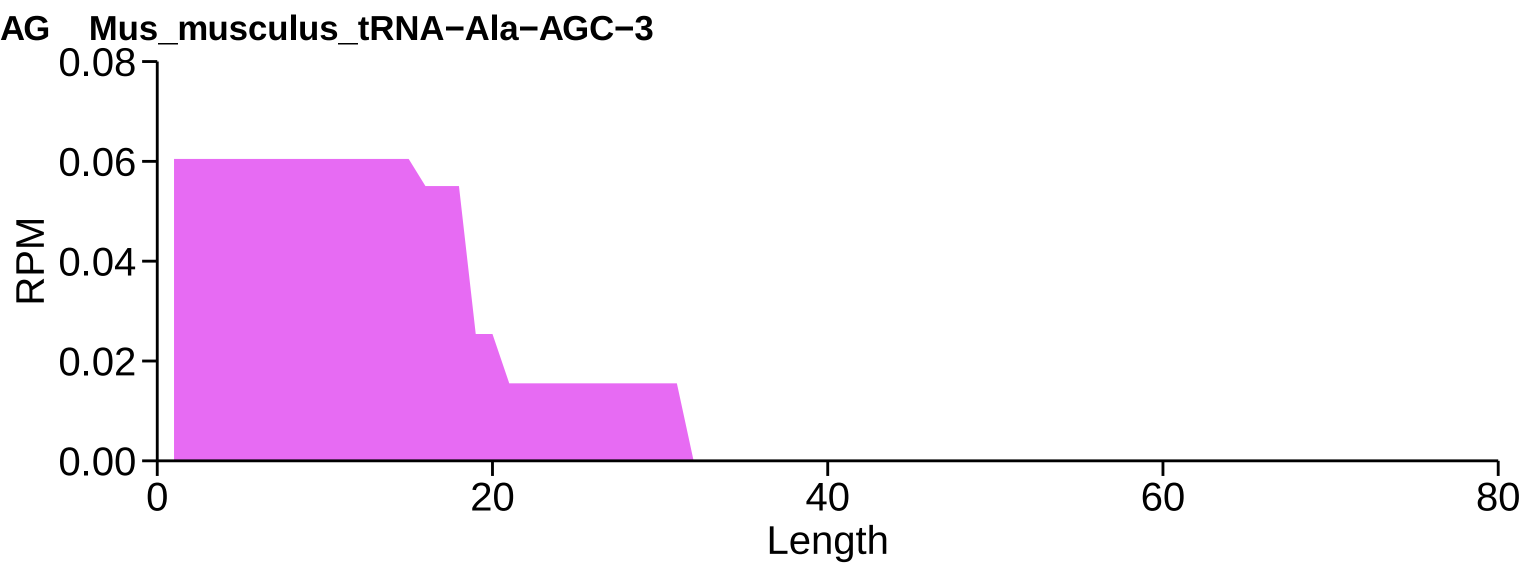
AG Mus_musculus_tRNA−Ala−AGC−3
0.08
0.06
0.04
0.02
0.00
RPM
0
20
40
60
80
Length

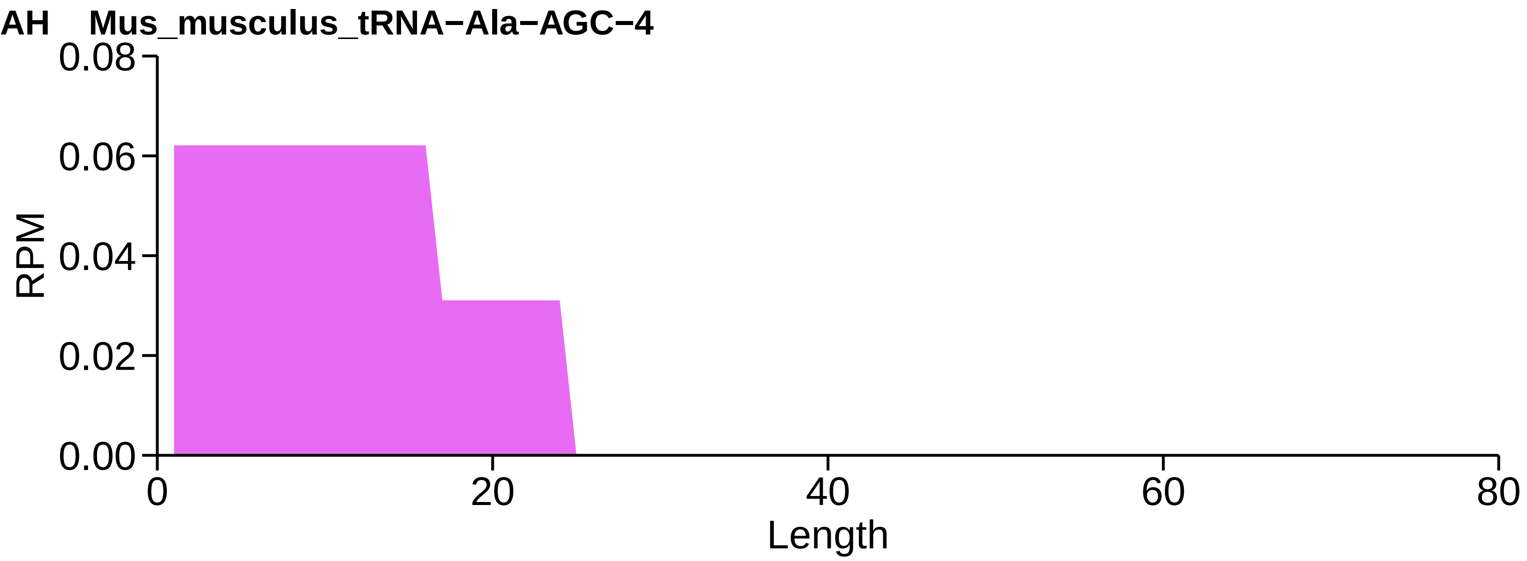
AH Mus_musculus_tRNA−Ala−AGC−4
0.08
0.06
0.04
0.02
0.00
RPM
0
20
40
60
80
Length

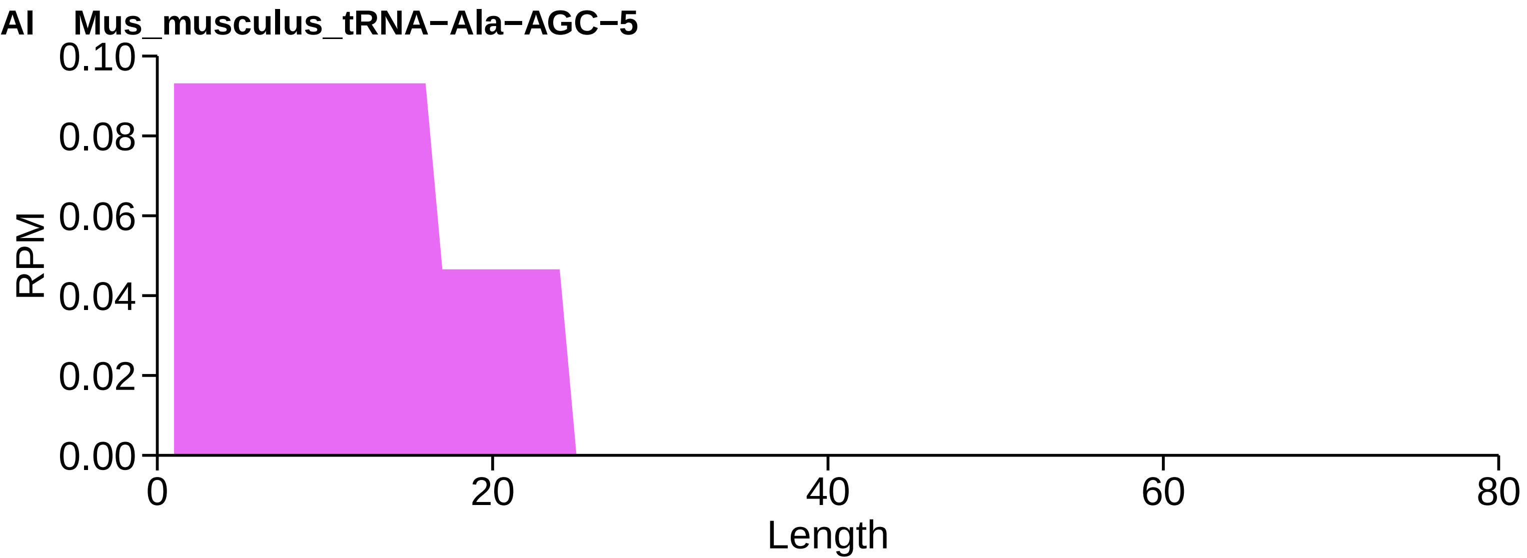
AI
Mus_musculus_tRNA−Ala−AGC−5
0.10
0.08
0.06
0.04
0.02
0.00
RPM
0
20
40
60
80
Length

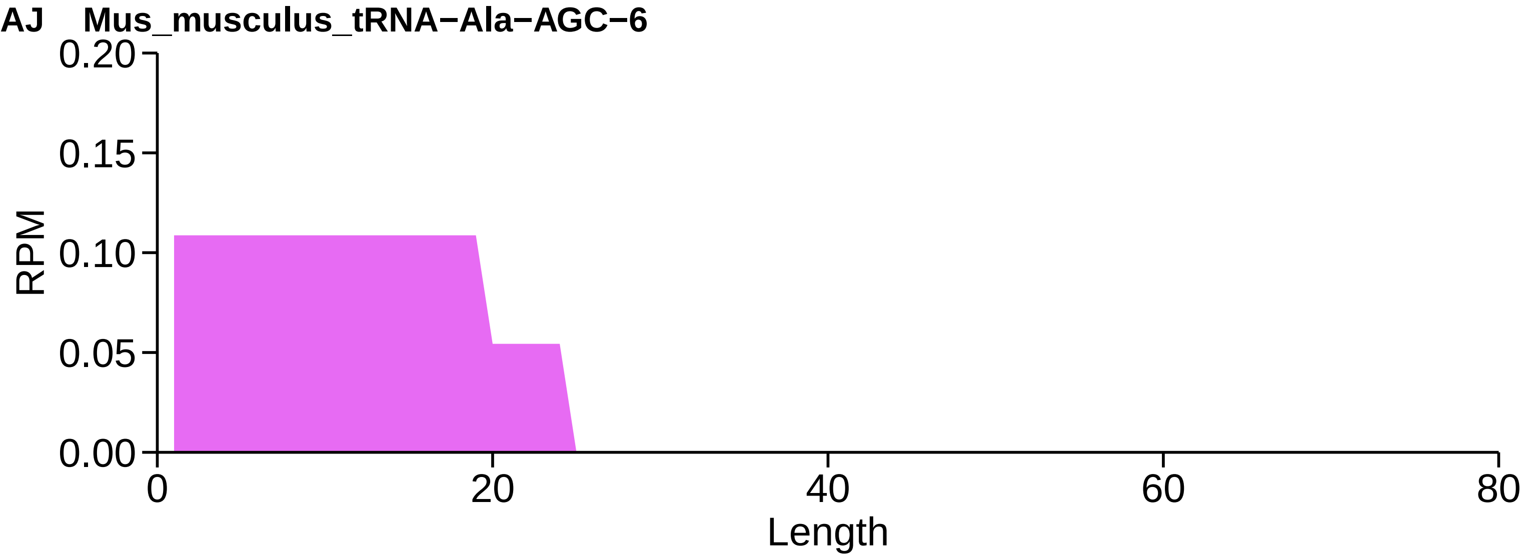

AJ Mus_musculus_tRNA−Ala−AGC−6
0.20
0.15
0.10
0.05
0.00
RPM
0
20
40
60
80
Length

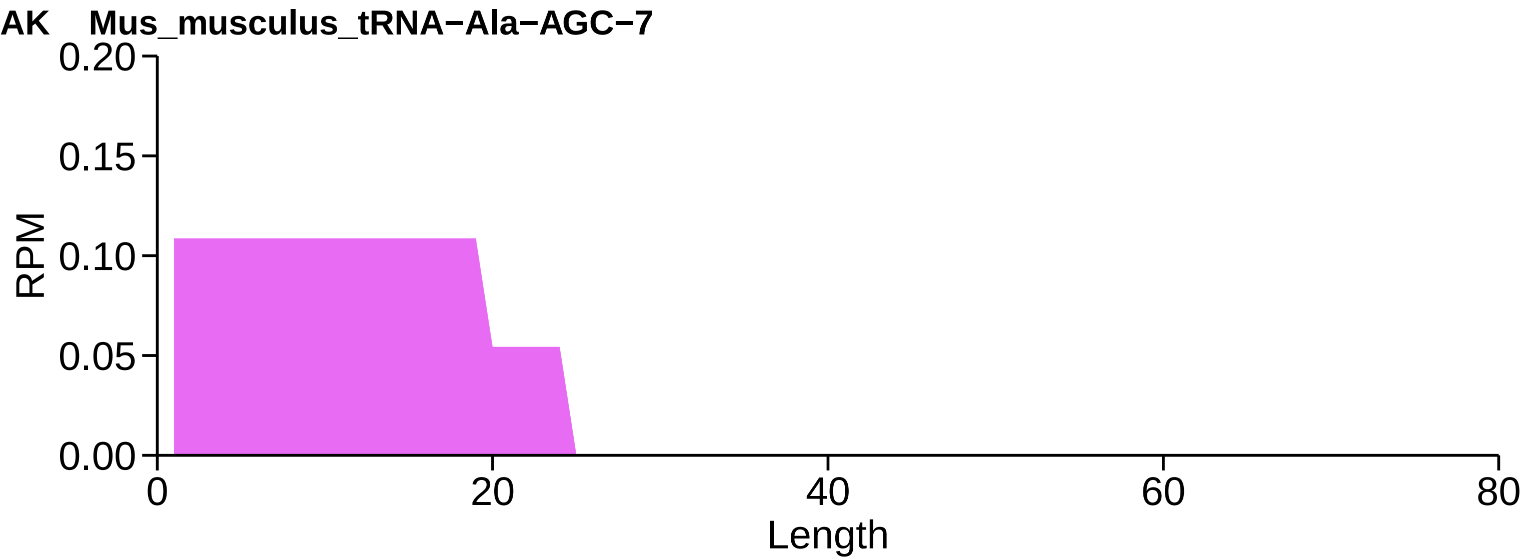
AK
Mus_musculus_tRNA−Ala−AGC−7
0.20
0.15
0.10
0.05
0.00
RPM
0
20
40
60
80
Length

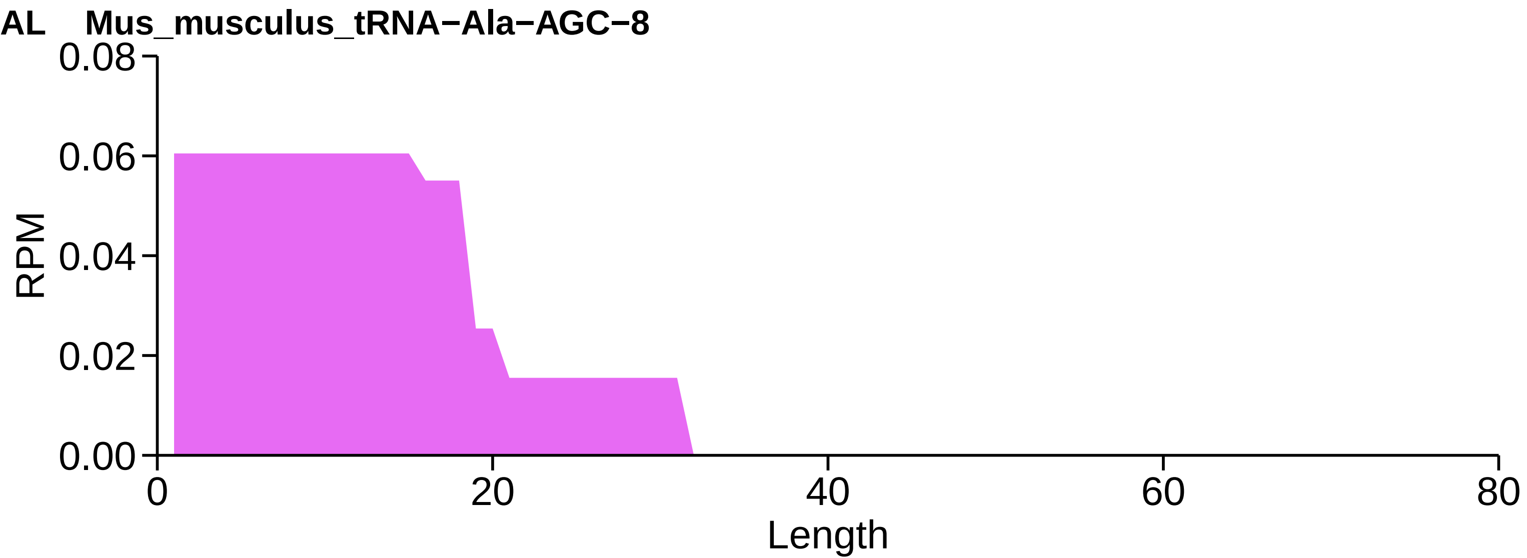
AL Mus_musculus_tRNA−Ala−AGC−8
0.08
0.06
0.04
0.02
0.00
RPM
0
20
40
60
80
Length

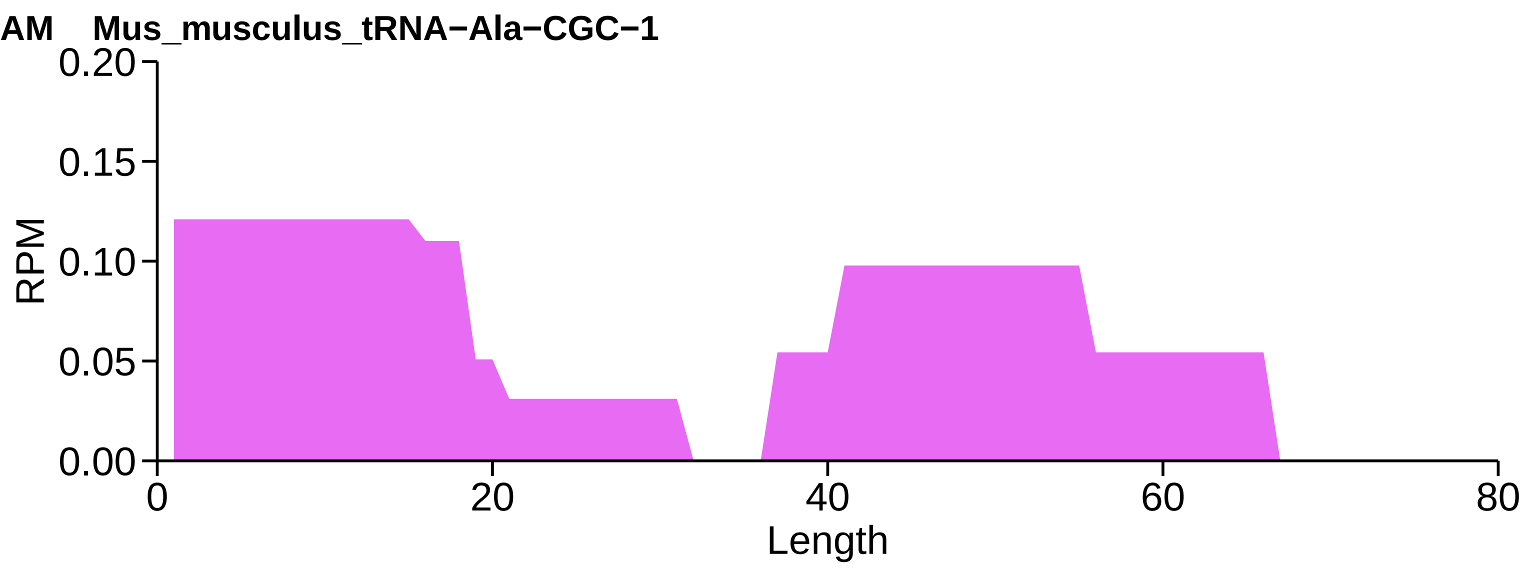
AM Mus_musculus_tRNA−Ala−CGC−1
RPM
0.20
0.15
0.10
0.05
0.00
0
20
40
60
80
Length

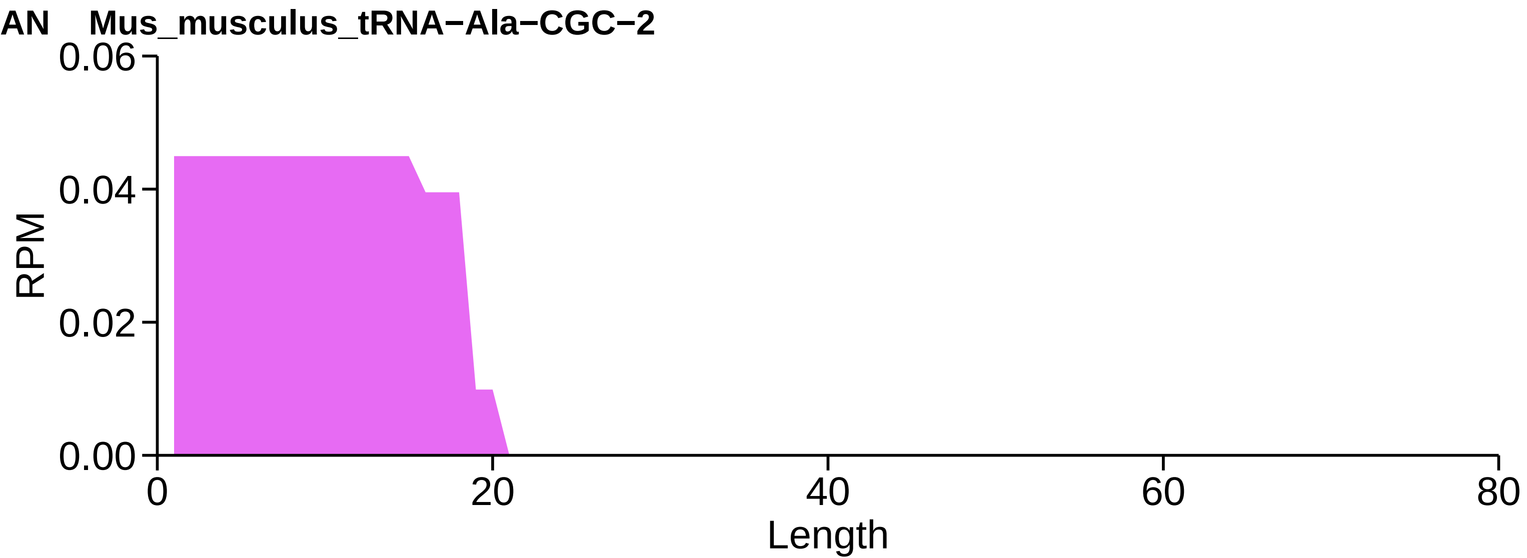
AN Mus_musculus_tRNA−Ala−CGC−2
0.06
0.04
0.02
0.00
RPM
0
20
40
60
80
Length

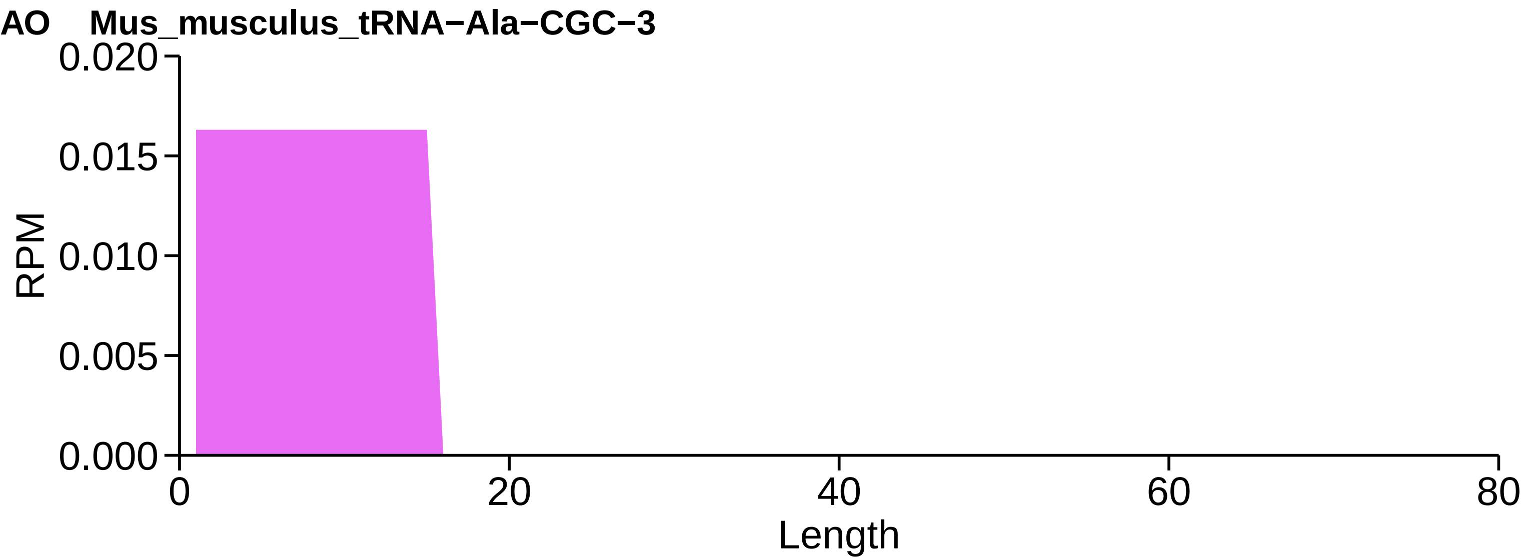

AO Mus_musculus_tRNA−Ala−CGC−3
RPM
0.020
0.015
0.010
0.005
0.000
0
20
40
60
80
Length

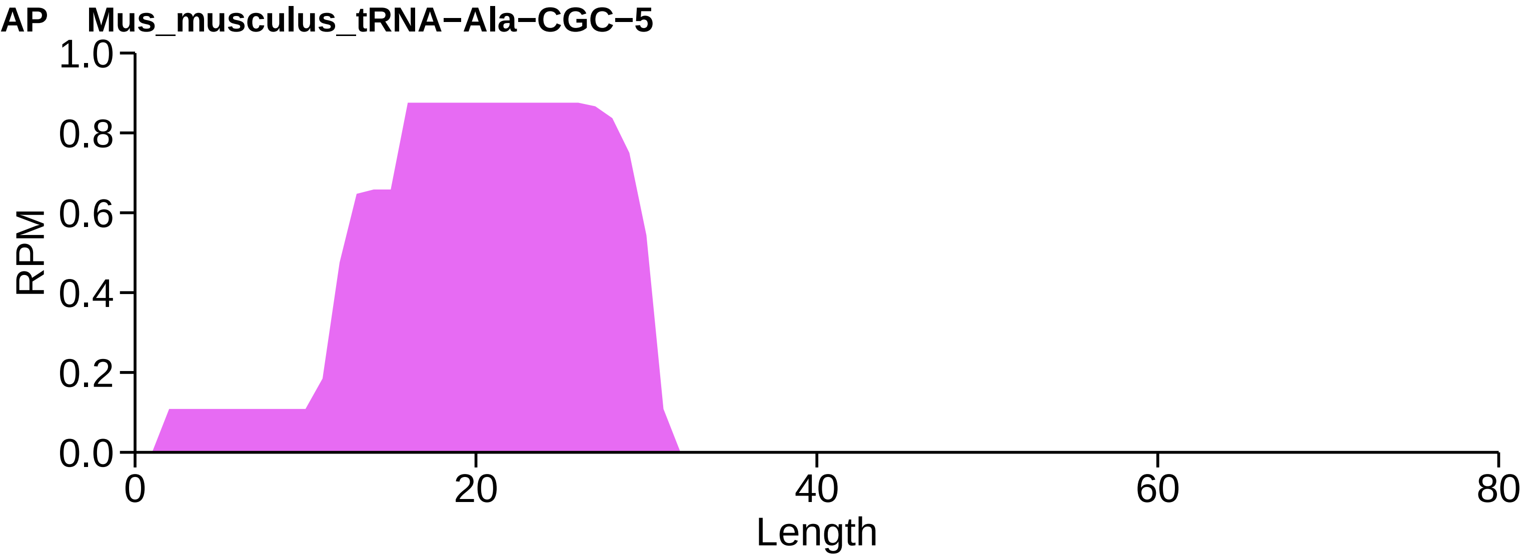
AP Mus_musculus_tRNA−Ala−CGC−5
RPM
1.0
0.8
0.6
0.4
0.2
0.0
0
20
40
60
80
Length

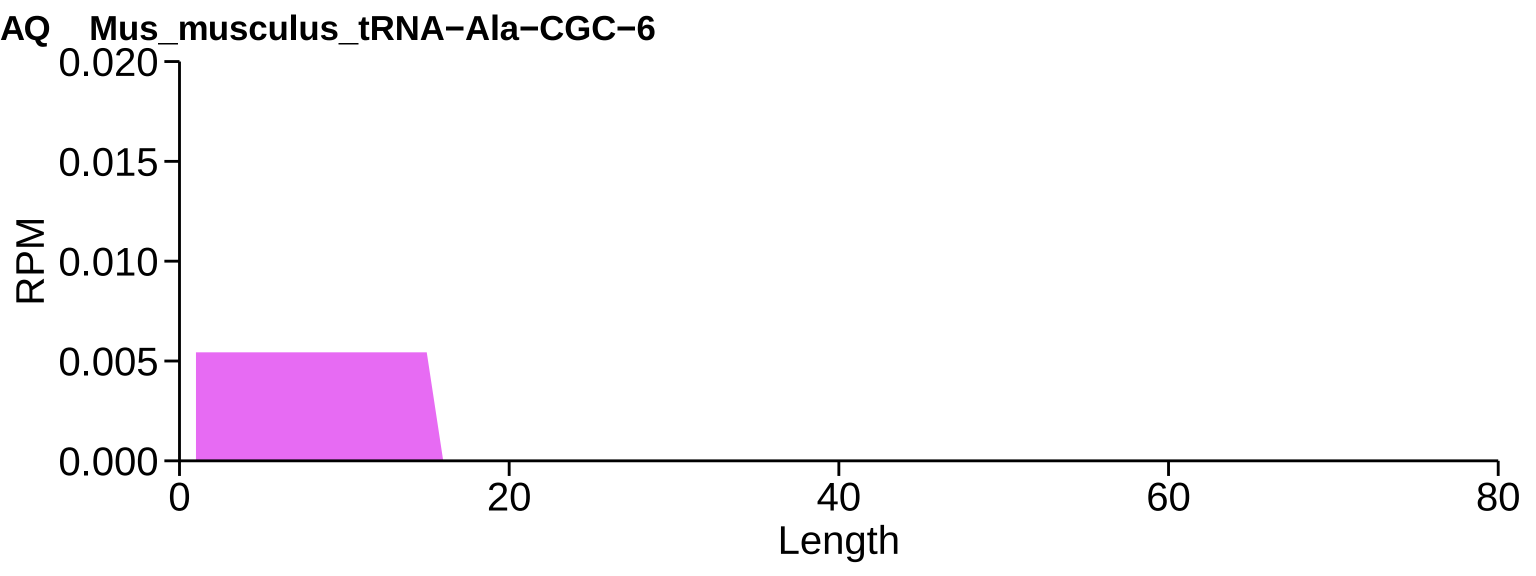
AQ Mus_musculus_tRNA−Ala−CGC−6
RPM
0.020
0.015
0.010
0.005
0.000
0
20
40
60
80
Length

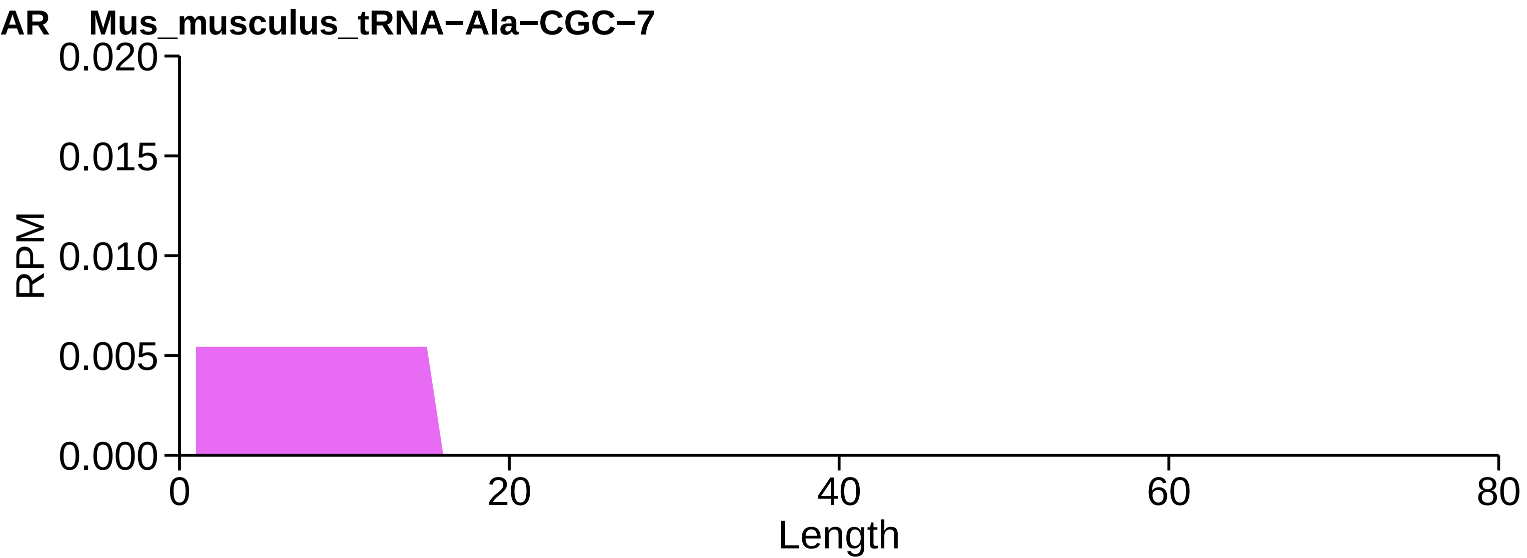
AR Mus_musculus_tRNA−Ala−CGC−7
RPM
0.020
0.015
0.010
0.005
0.000
0
20
40
60
80
Length

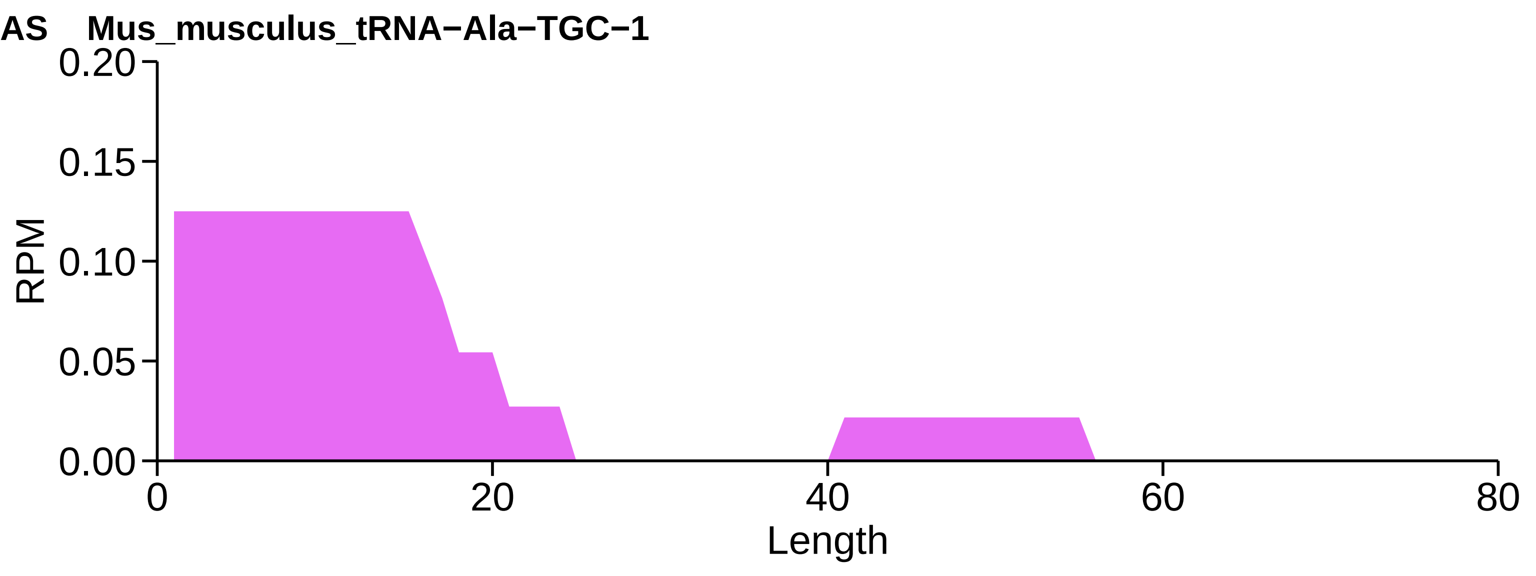
AS Mus_musculus_tRNA−Ala−TGC−1
RPM
0.20
0.15
0.10
0.05
0.00
0
20
40
60
80
Length

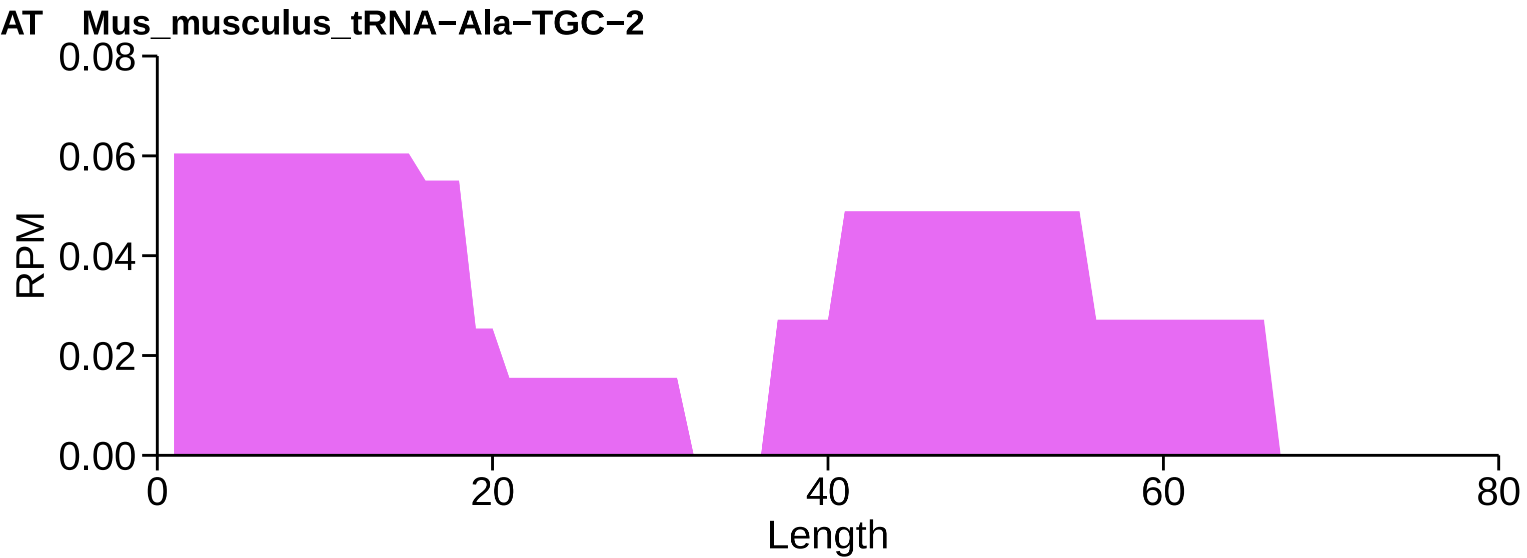

AT Mus_musculus_tRNA−Ala−TGC−2
RPM
0.00
0.02
0.04
0.06
0.08
0
20
40
60
80
Length

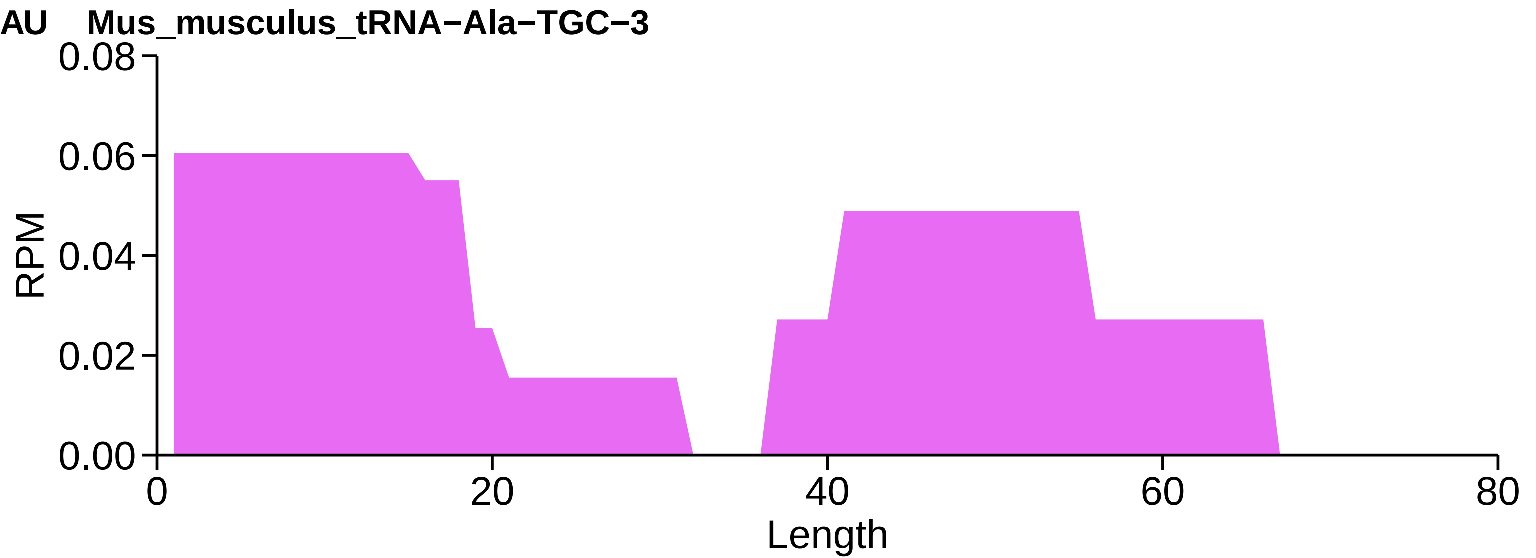
AU Mus_musculus_tRNA−Ala−TGC−3
RPM
0.08
0.06
0.04
0.02
0.00
0
20
40
60
80
Length

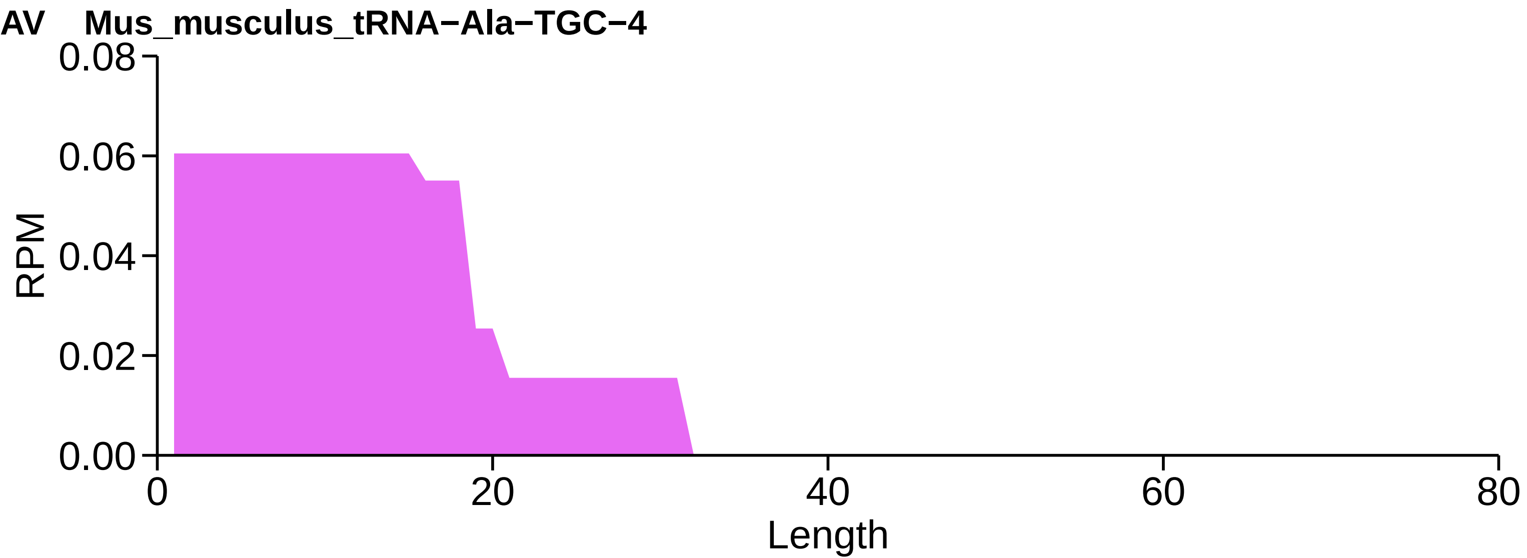
AV Mus_musculus_tRNA−Ala−TGC−4
RPM
0.00
0.02
0.04
0.06
0.08
0
20
40
60
80
Length

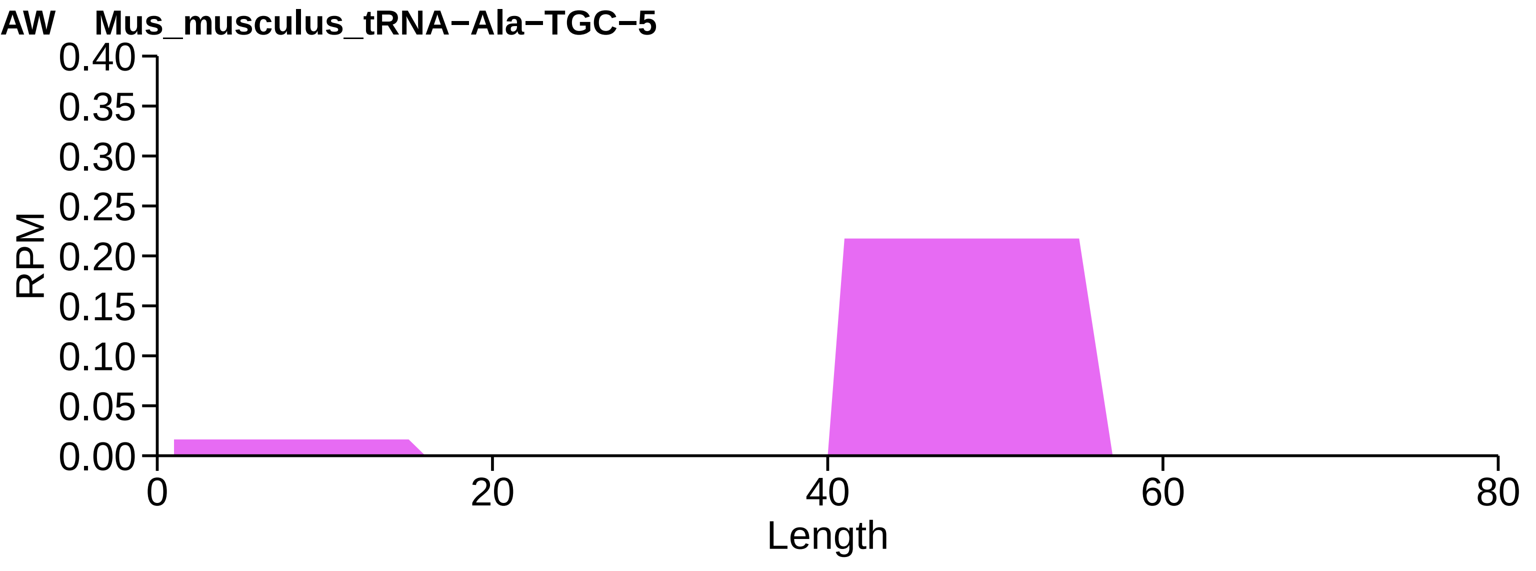
AW Mus_musculus_tRNA−Ala−TGC−5
RPM
0.40
0.35
0.30
0.25
0.20
0.15
0.10
0.05
0.00
0
20
40
60
80
Length

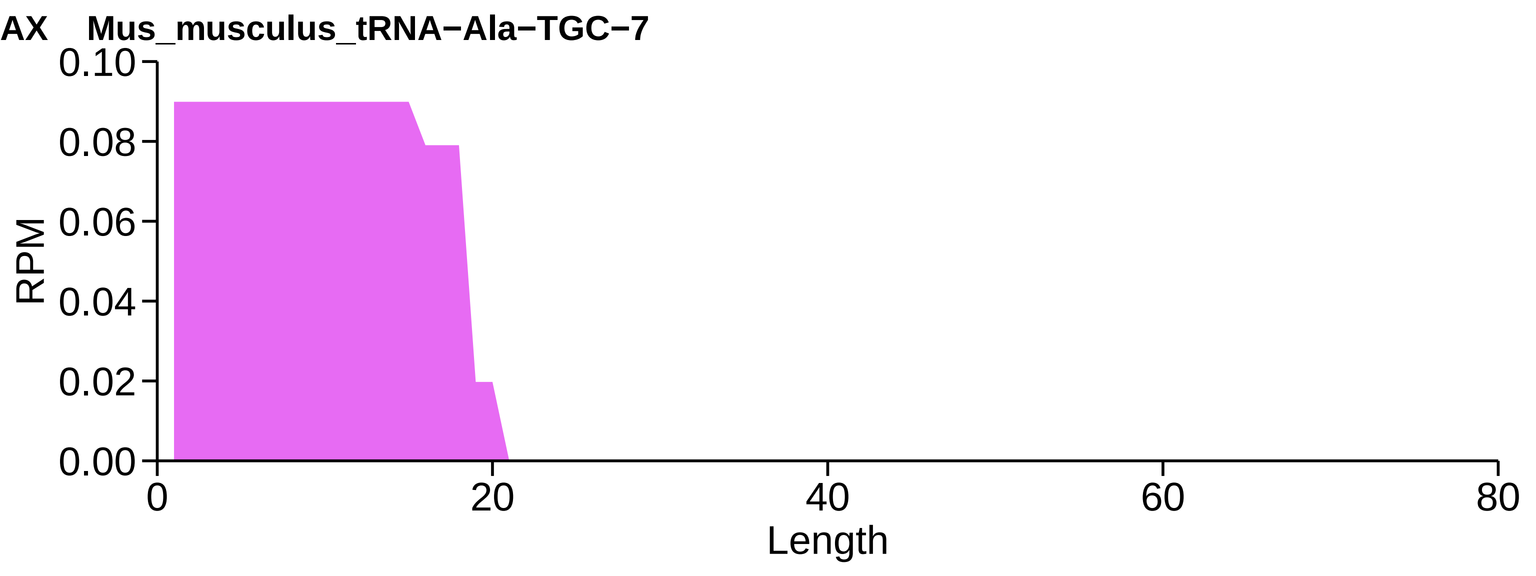
AX Mus_musculus_tRNA−Ala−TGC−7
RPM
0.00
0.02
0.04
0.06
0.08
0.10
0
20
40
60
80
Length

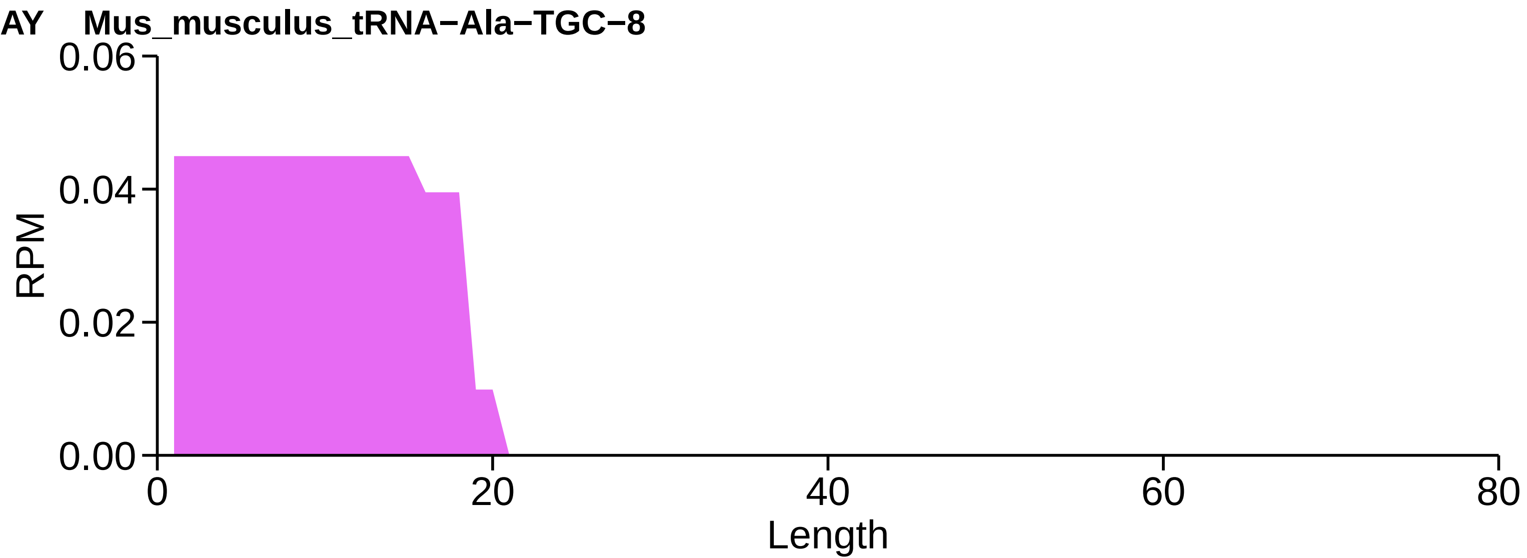

AY Mus_musculus_tRNA−Ala−TGC−8
0.06
0.04
0.02
0.00
RPM
0
20
40
60
80
Length

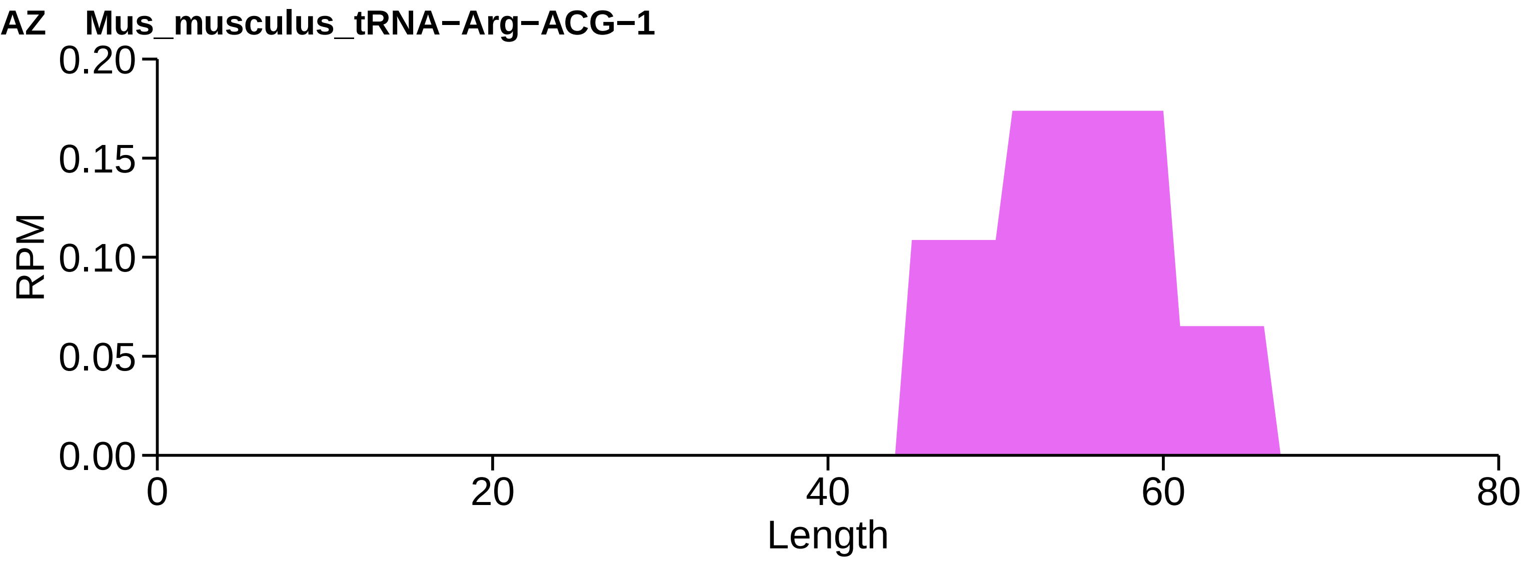
AZ Mus_musculus_tRNA−Arg−ACG−1
RPM
0.20
0.15
0.10
0.05
0.00
0
20
40
60
80
Length

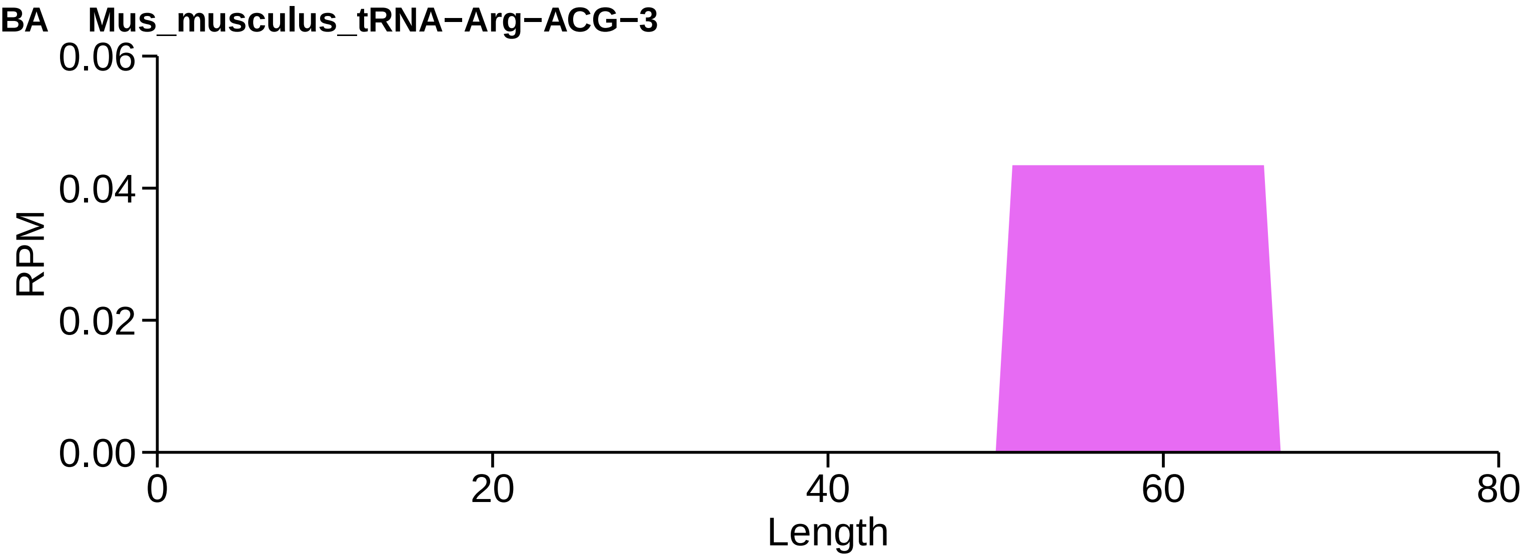
BA Mus_musculus_tRNA−Arg−ACG−3
0.06
0.04
0.02
0.00
RPM
0
20
40
60
80
Length

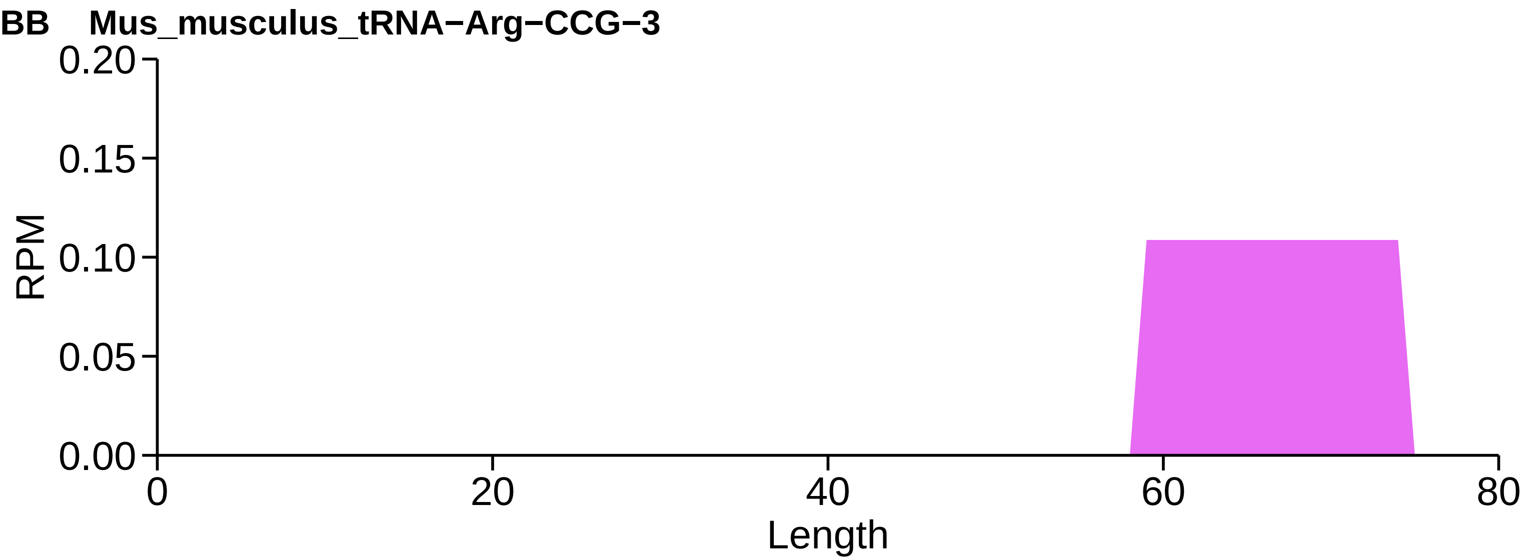
BB Mus_musculus_tRNA−Arg−CCG−3
RPM
0.00
0.05
0.10
0.15
0.20
0
20
40
60
80
Length

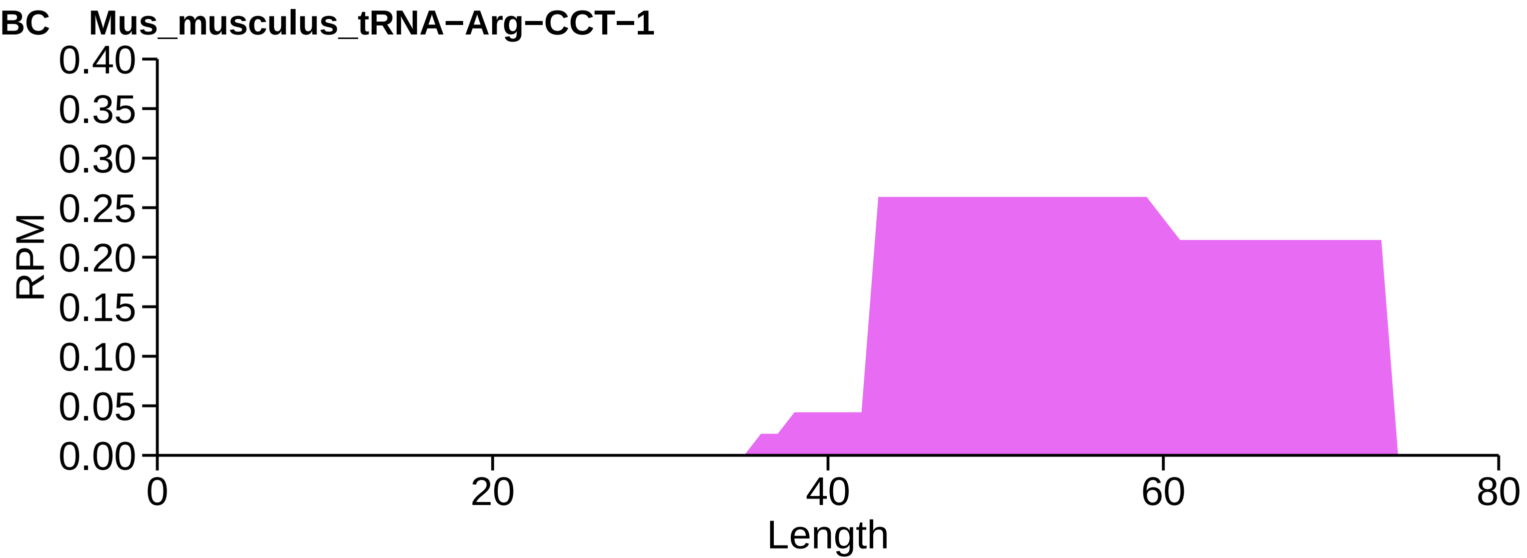
BC Mus_musculus_tRNA−Arg−CCT−1
RPM
0.00
0.05
0.10
0.15
0.20
0.25
0.30
0.35
0.40
0
20
40
60
80
Length

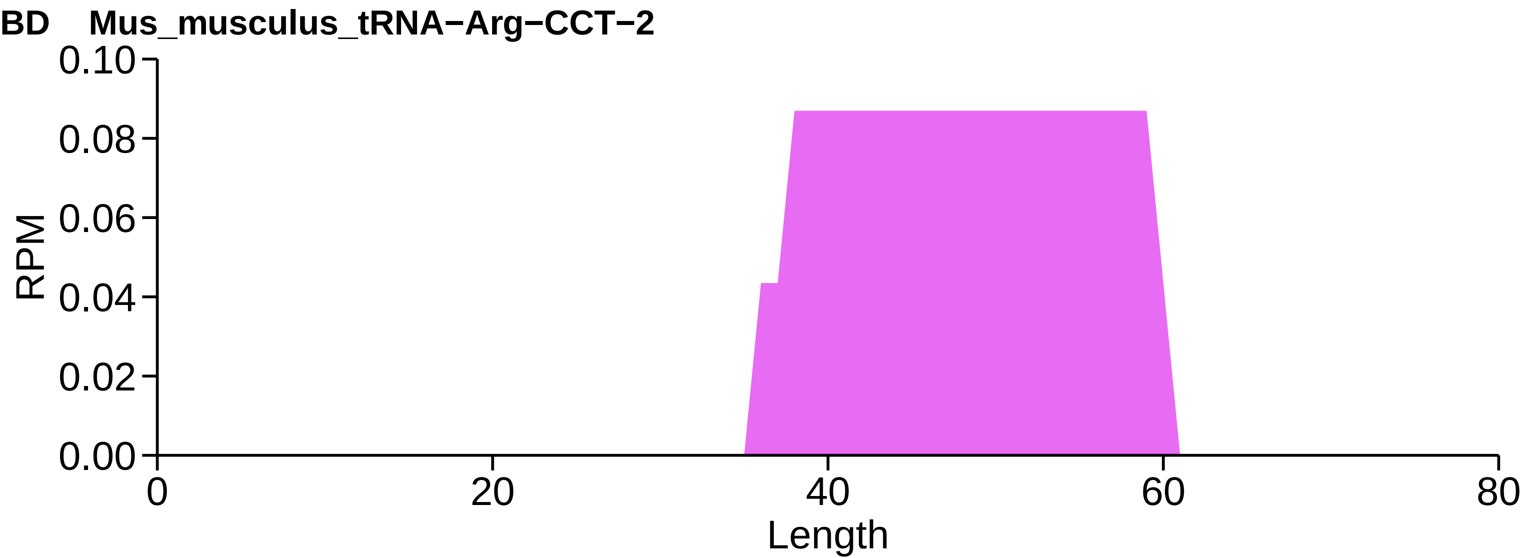
BD Mus_musculus_tRNA−Arg−CCT−2
0.10
0.08
0.06
0.04
0.02
0.00
RPM
0
20
40
60
80
Length

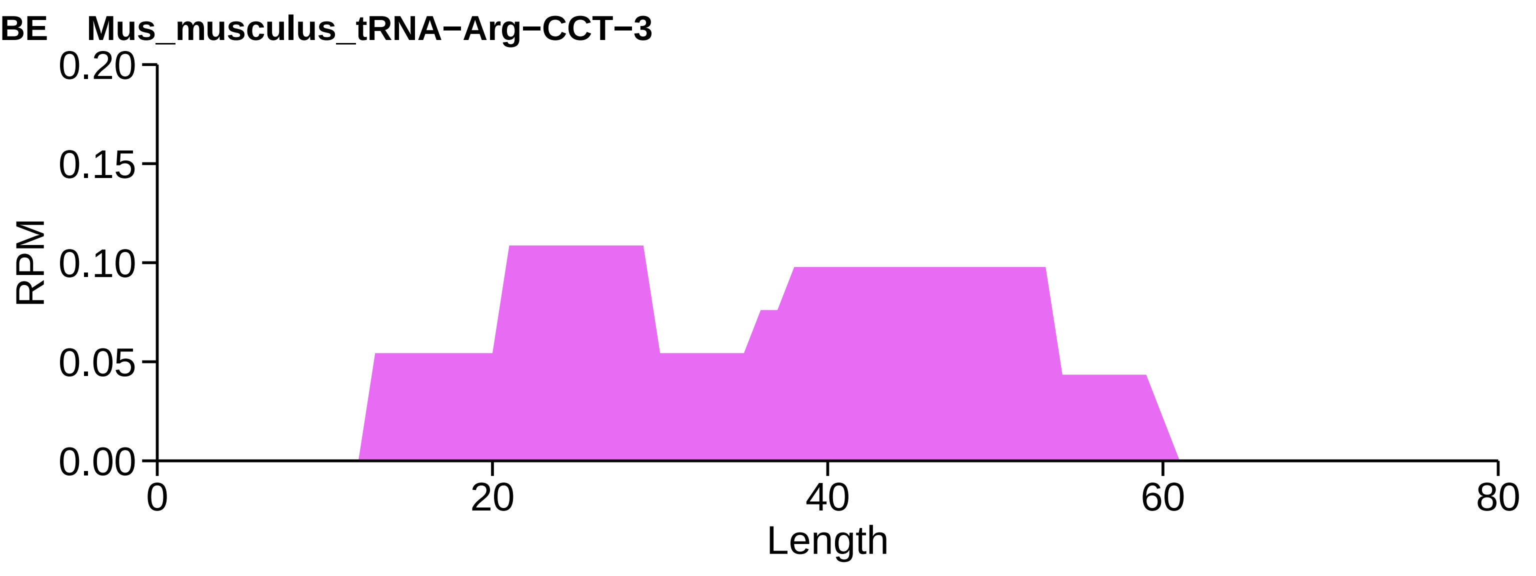
BE Mus_musculus_tRNA−Arg−CCT−3
0.20
0.15
0.10
0.05
0.00
RPM
0
20
40
60
80
Length

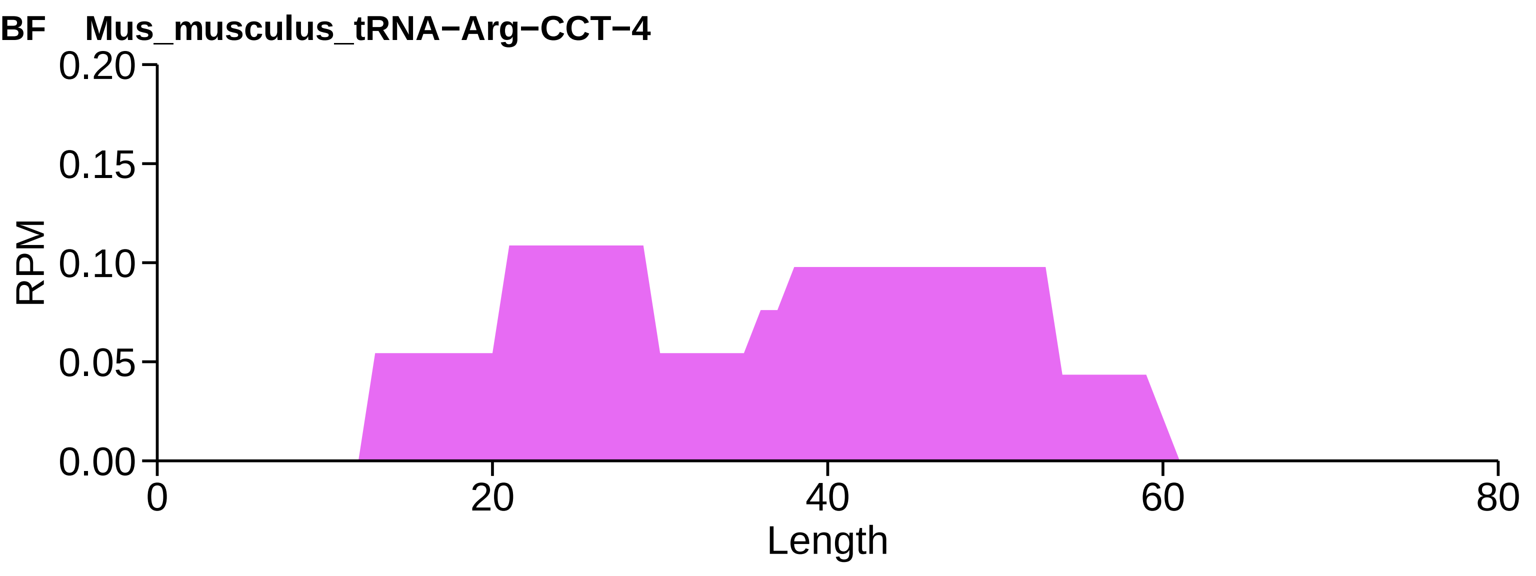
BF Mus_musculus_tRNA−Arg−CCT−4
RPM
0.00
0.05
0.10
0.15
0.20
0
20
40
60
80
Length

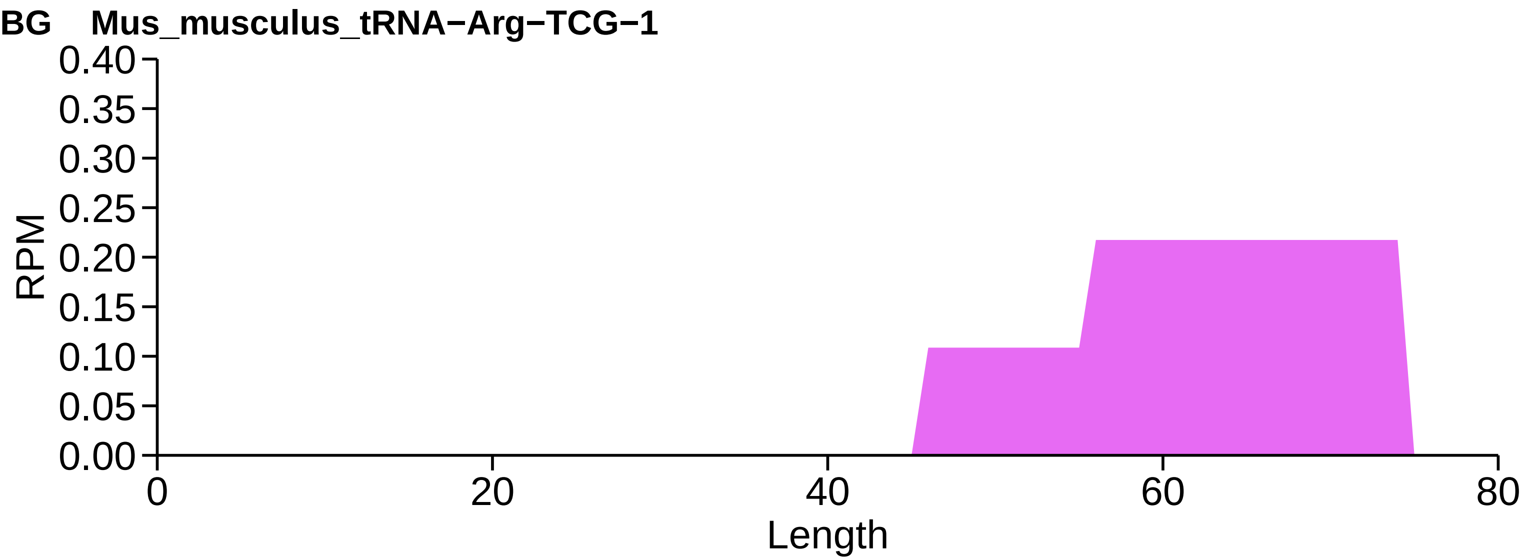
BG Mus_musculus_tRNA−Arg−TCG−1
RPM
0.00
0.05
0.10
0.15
0.20
0.25
0.30
0.35
0.40
0
20
40
60
80
Length

BH Mus_musculus_tRNA−Arg−TCT−1

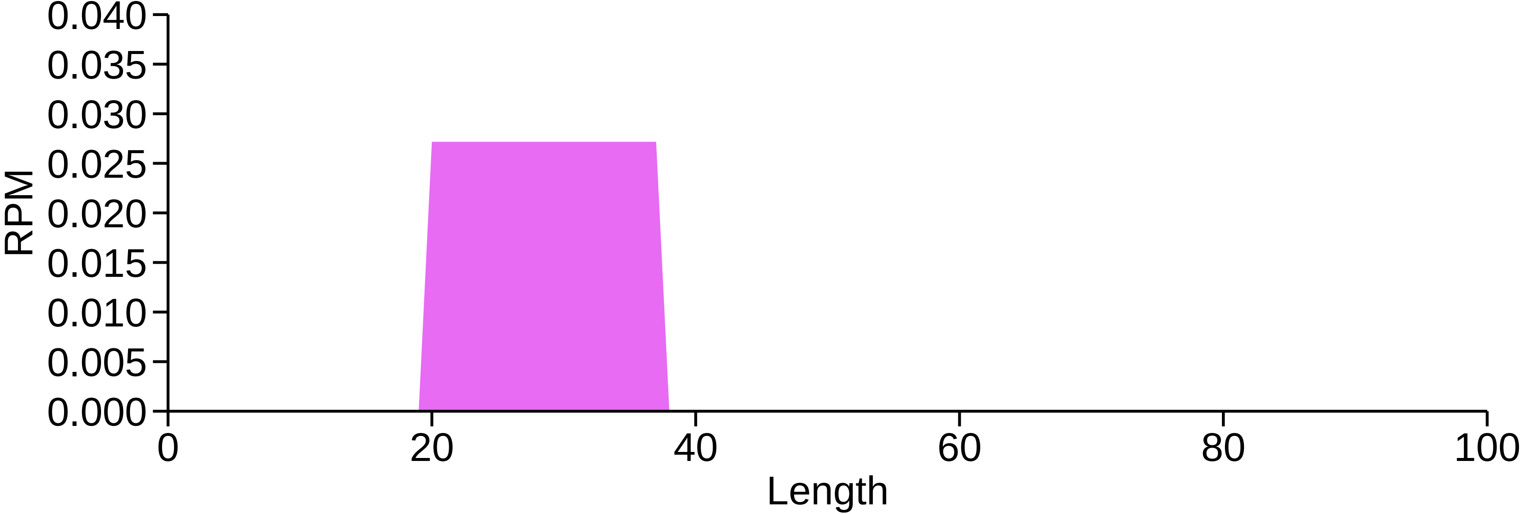

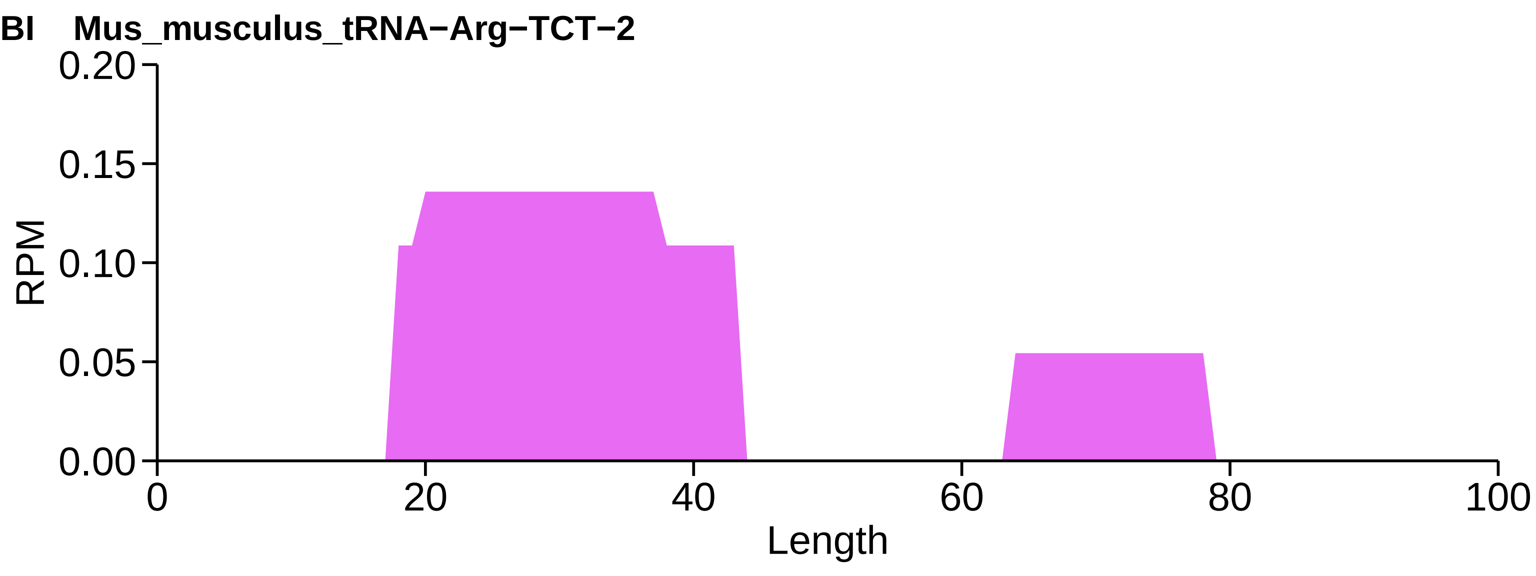
BI Mus_musculus_tRNA−Arg−TCT−2
RPM
0.20
0.15
0.10
0.05
0.00
0
20
40
60
80
100
Length

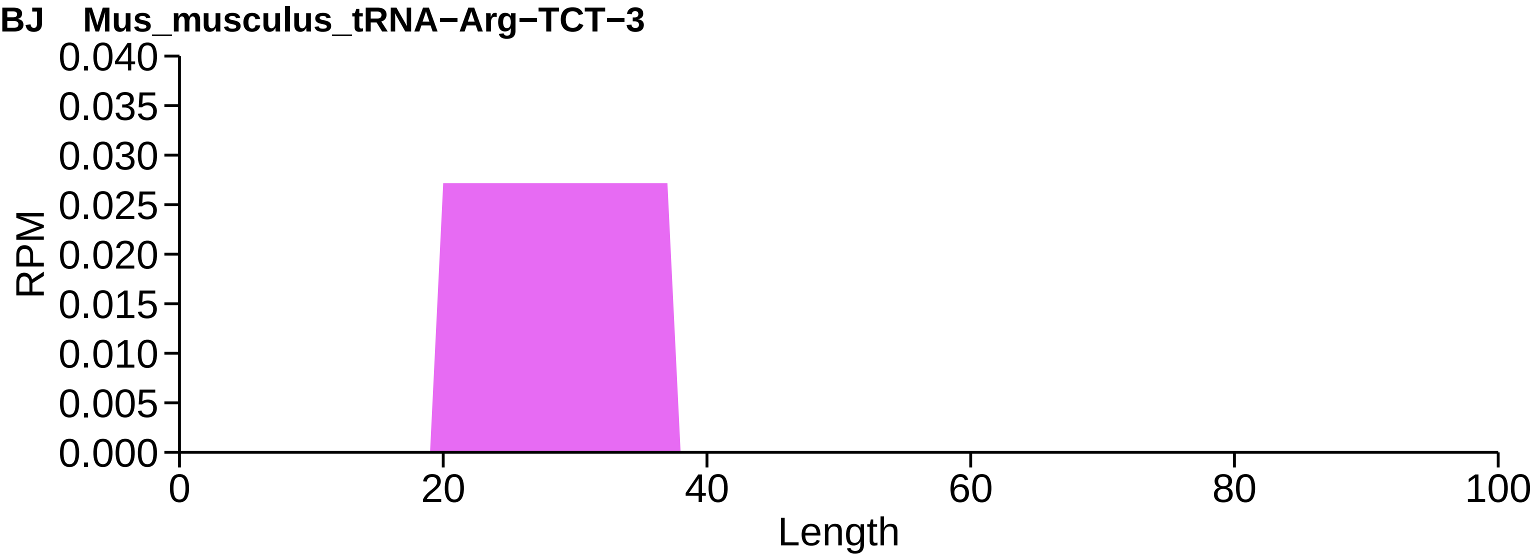
BJ Mus_musculus_tRNA−Arg−TCT−3
RPM
0.000
0.005
0.010
0.015
0.020
0.025
0.030
0.035
0.040
0
20
40
60
80
100
Length

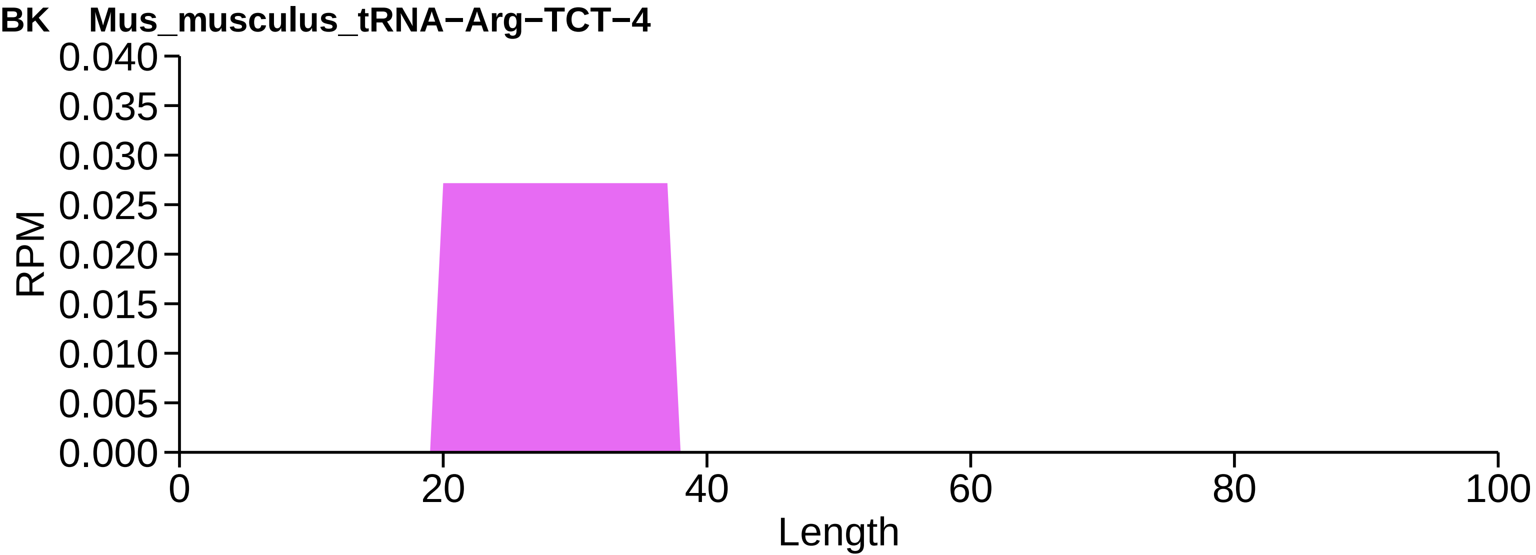
BK Mus_musculus_tRNA−Arg−TCT−4
RPM
0.040
0.035
0.030
0.025
0.020
0.015
0.010
0.005
0.000
0
20
40
60
80
100
Length

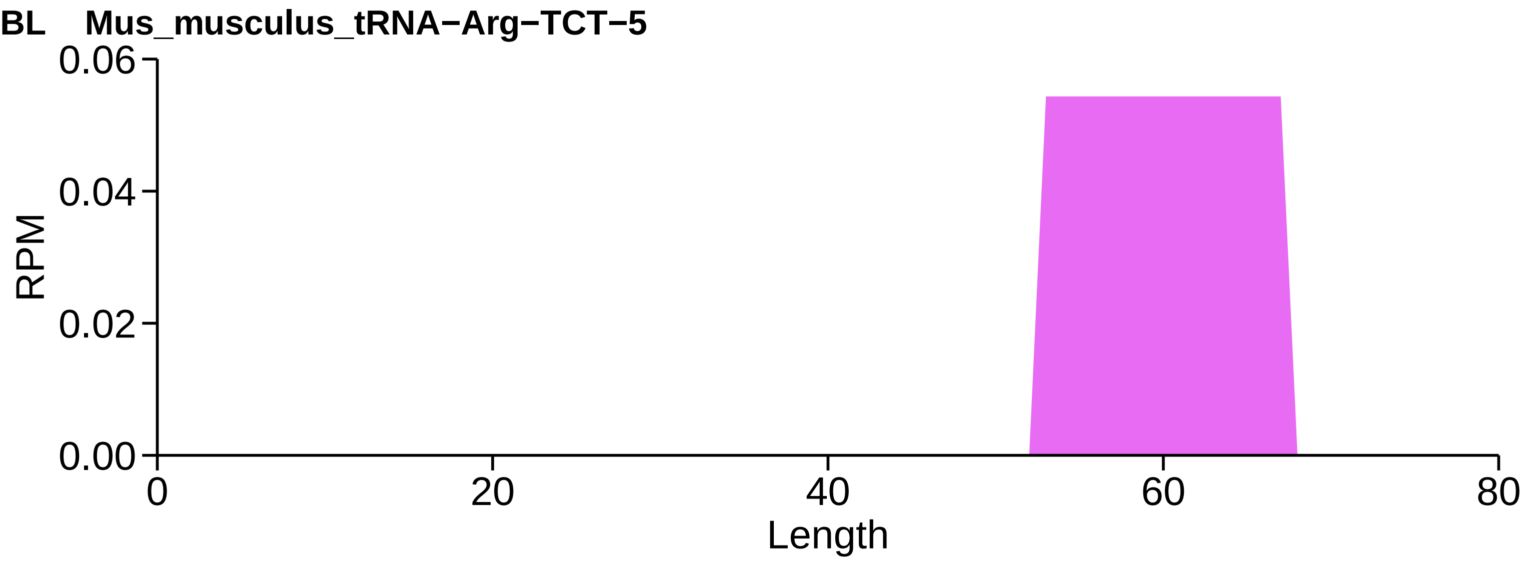
BL Mus_musculus_tRNA−Arg−TCT−5
0.06
0.04
0.02
0.00
RPM
0
20
40
60
80
Length

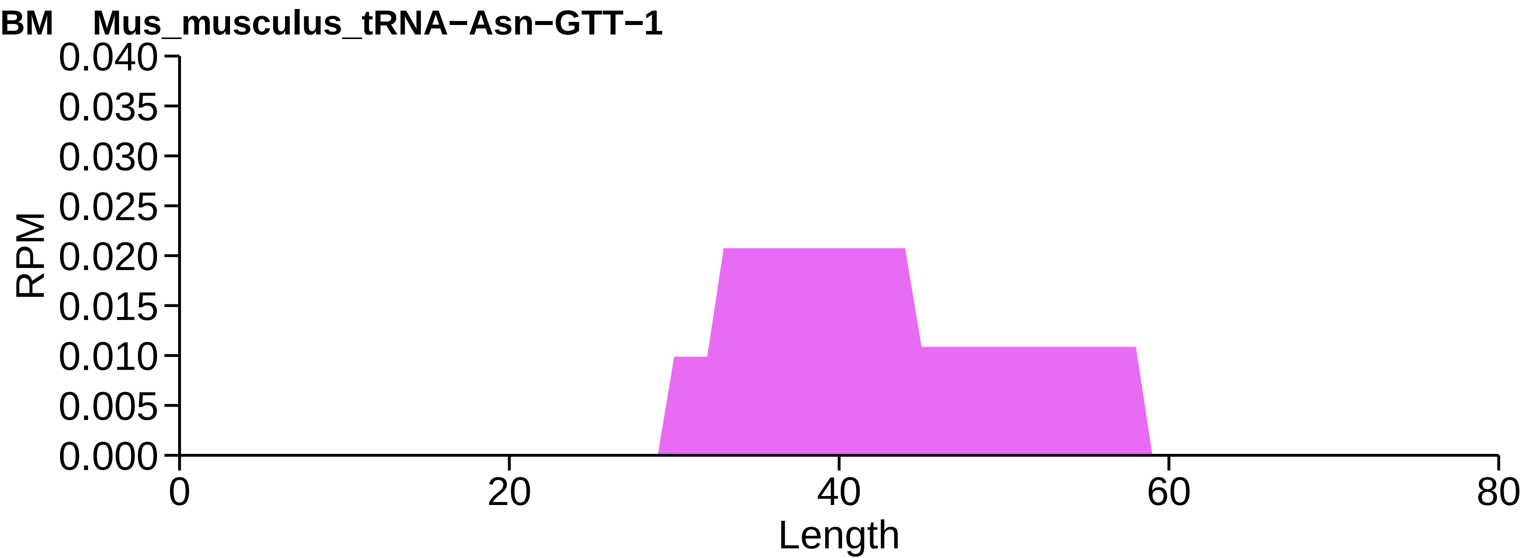
BM Mus_musculus_tRNA−Asn−GTT−1
RPM
0.040
0.035
0.030
0.025
0.020
0.015
0.010
0.005
0.000
0
20
40
60
80
Length

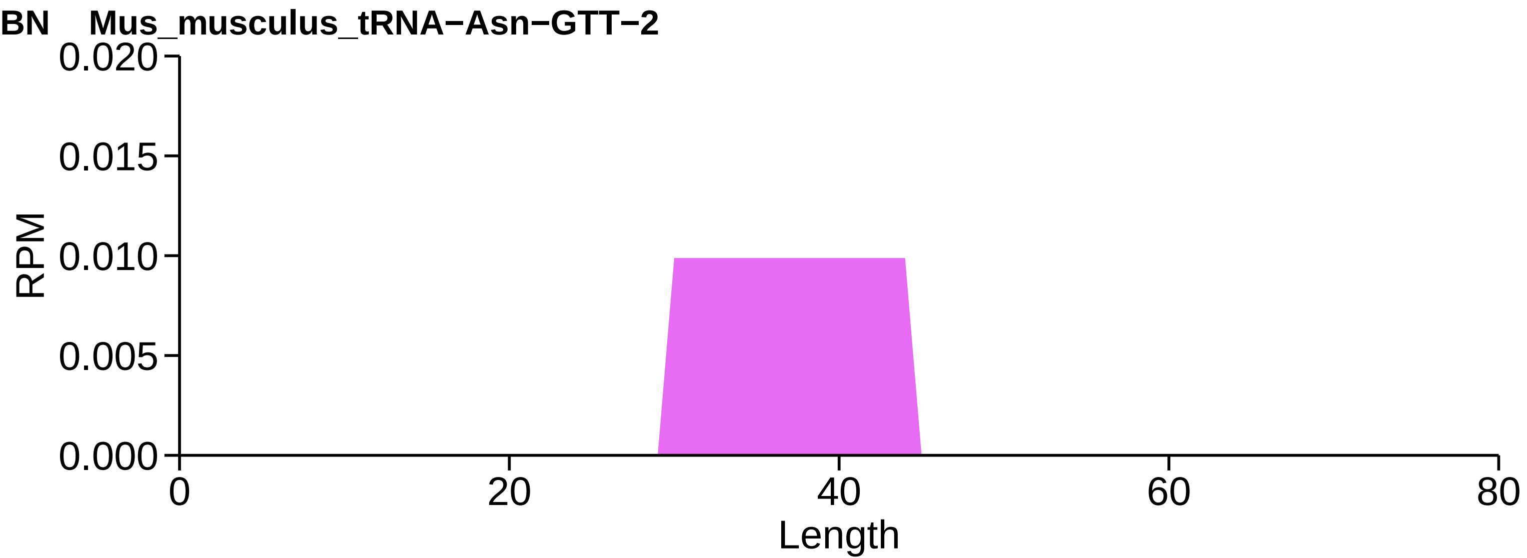
BN Mus_musculus_tRNA−Asn−GTT−2
0.020
0.015
0.010
0.005
0.000
RPM
0
20
40
60
80
Length

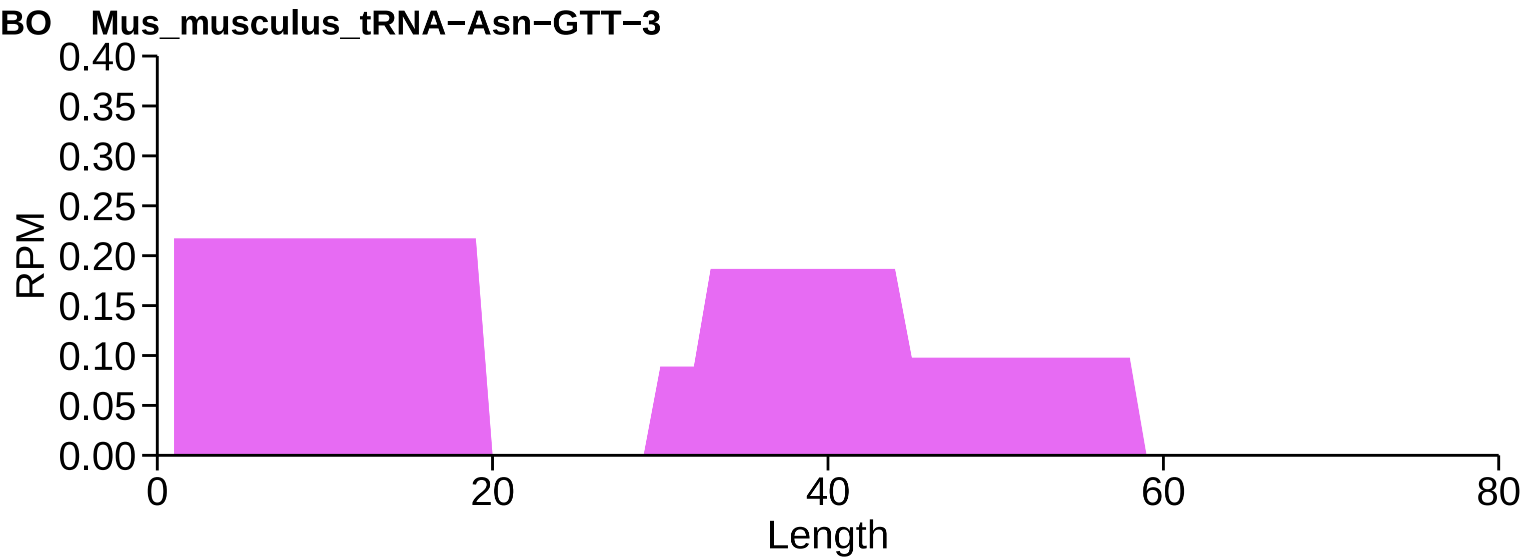
BO Mus_musculus_tRNA−Asn−GTT−3
0.40
0.35
0.30
0.25
0.20
0.15
0.10
0.05
0.00
RPM
0
20
40
60
80
Length

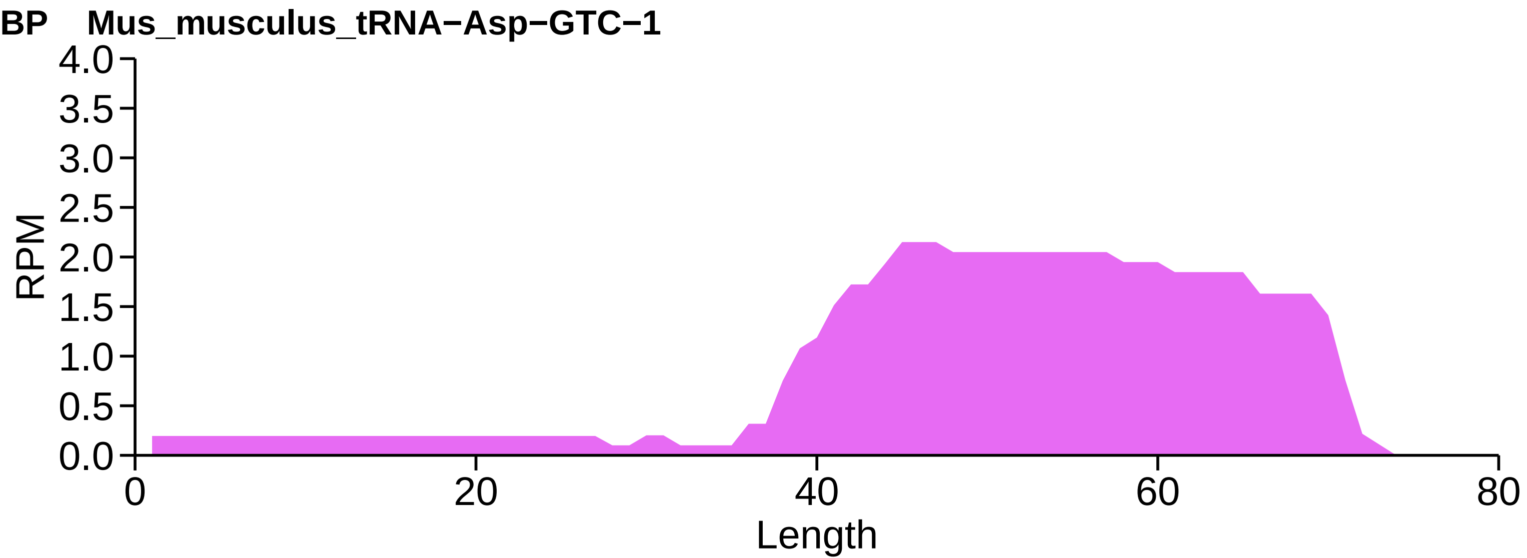
BP Mus_musculus_tRNA−Asp−GTC−1
RPM
Length
0.0
0.5
1.0
1.5
2.0
2.5
3.0
3.5
4.0
0
20
40
60
80

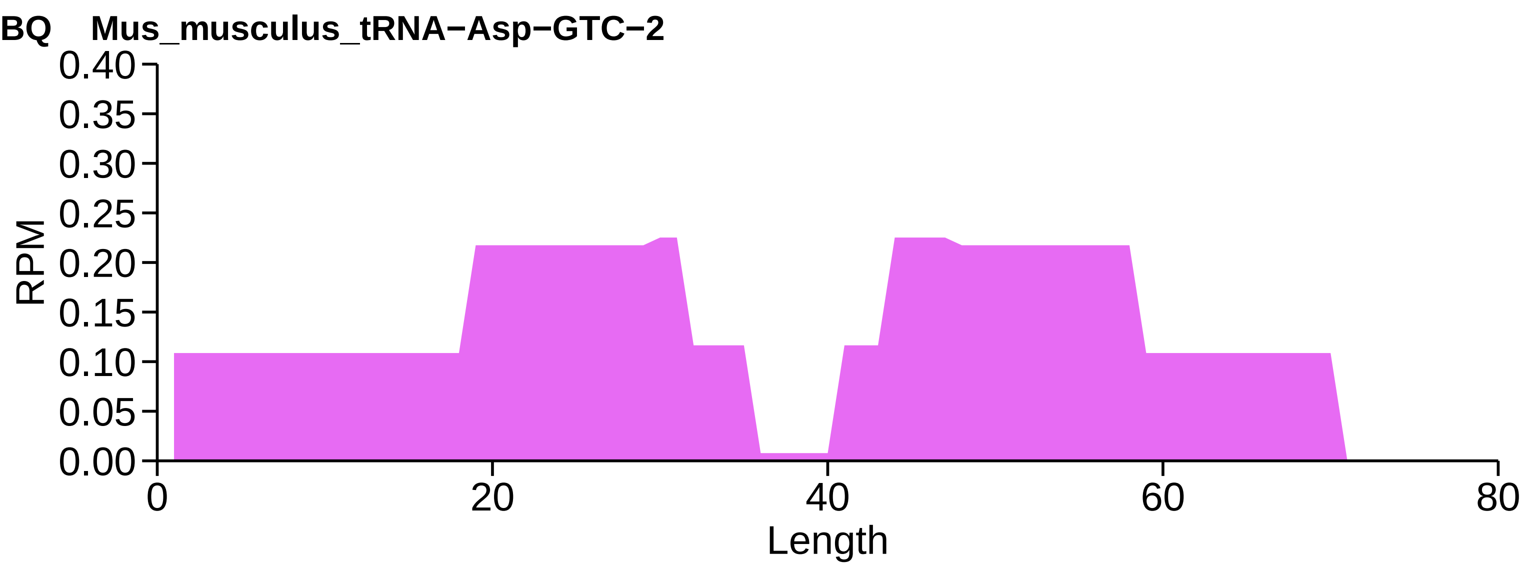
BQ Mus_musculus_tRNA−Asp−GTC−2
RPM
0.40
0.35
0.30
0.25
0.20
0.15
0.10
0.05
0.00
0
20
40
60
80
Length

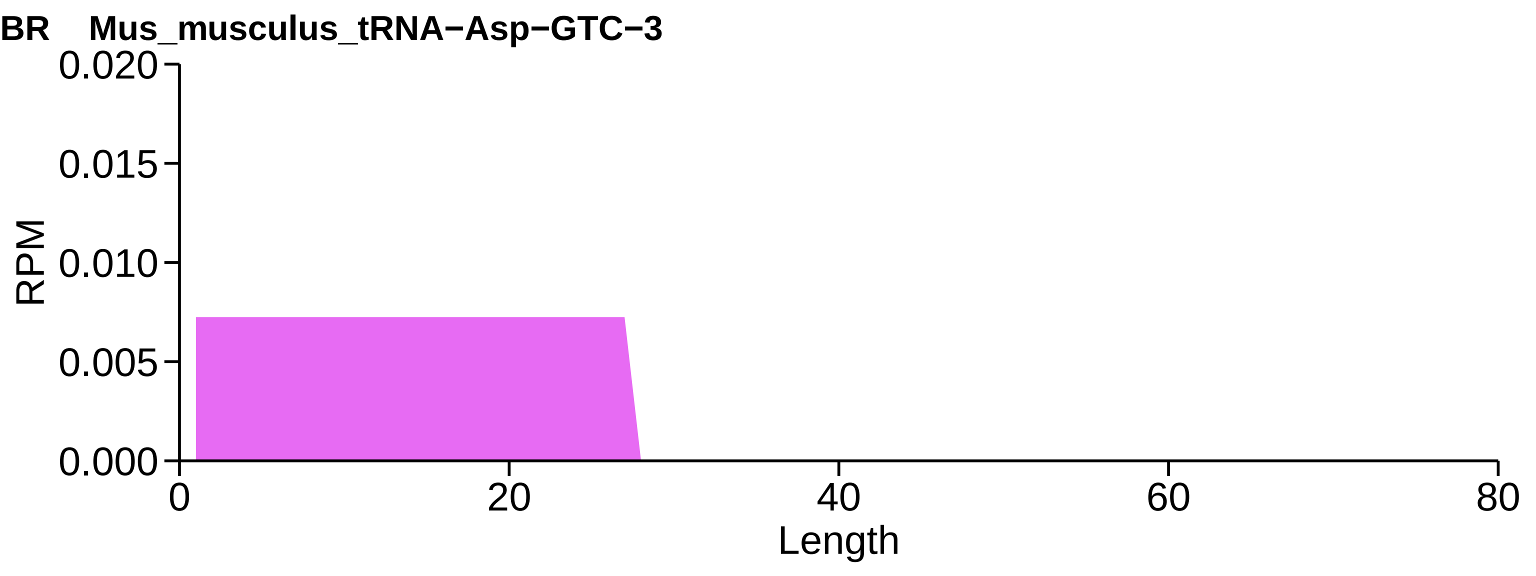
BR Mus_musculus_tRNA−Asp−GTC−3
RPM
0.020
0.015
0.010
0.005
0.000
0
20
40
60
80
Length

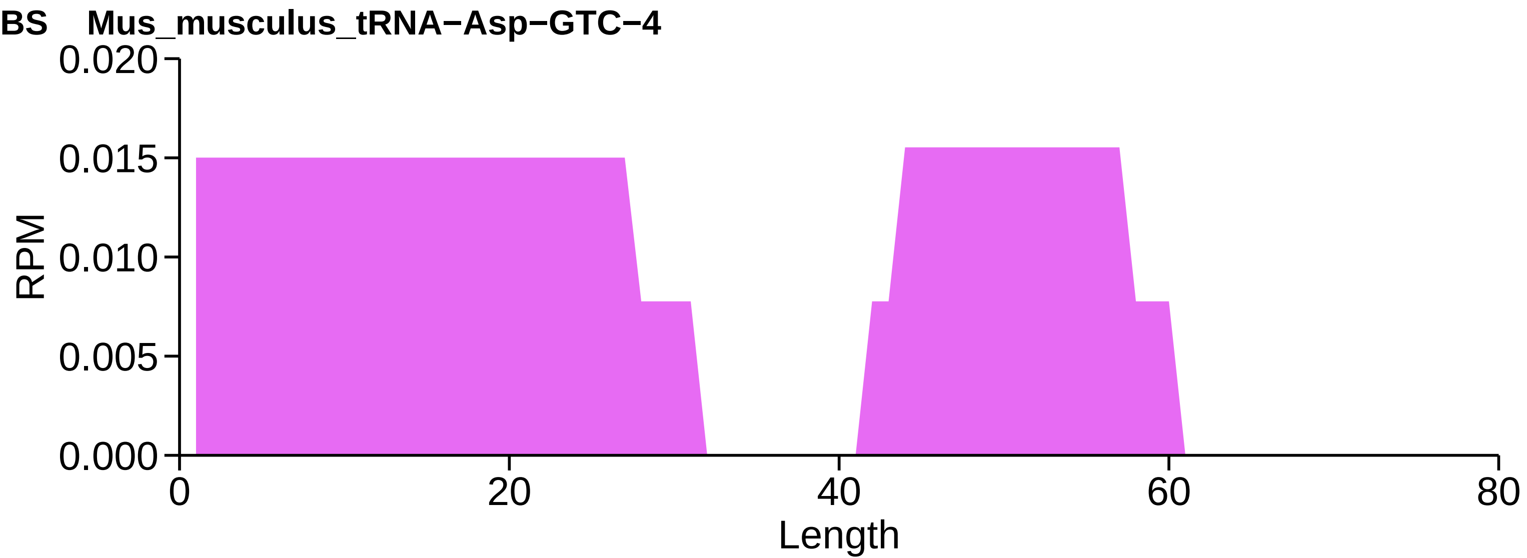
BS
Mus_musculus_tRNA−Asp−GTC−4
0.020
0.015
0.010
0.005
0.000
RPM
0
20
40
60
80
Length

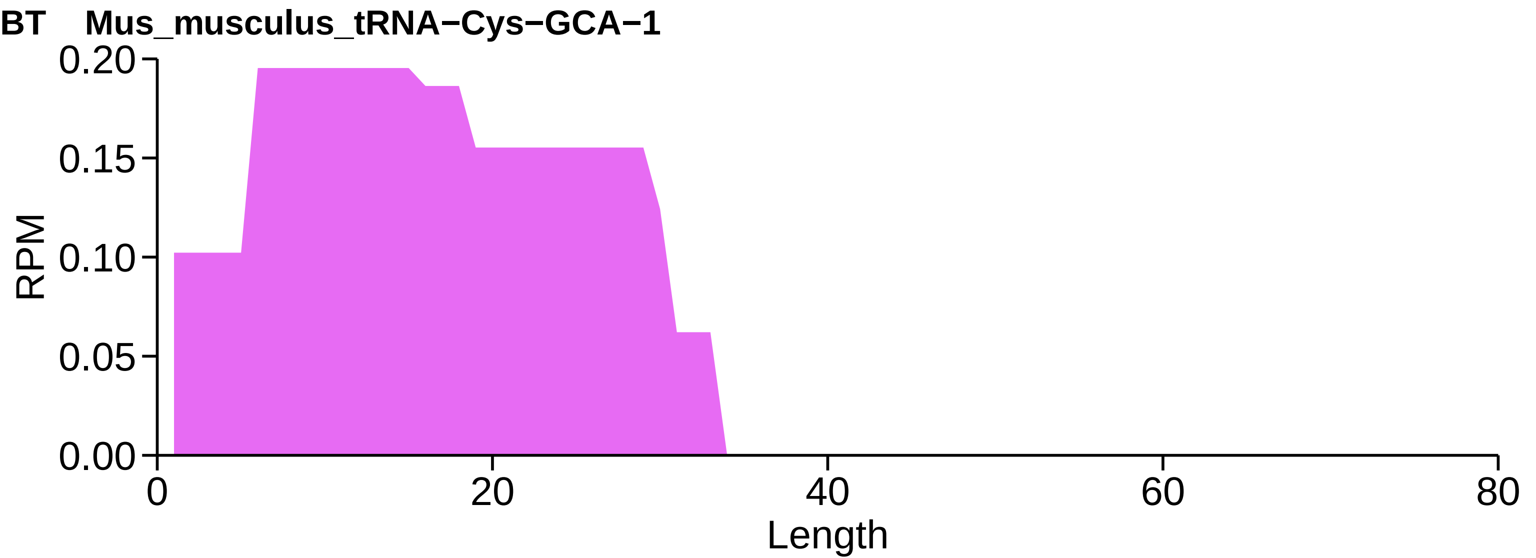

BT Mus_musculus_tRNA−Cys−GCA−1
0.20
0.15
0.10
0.05
0.00
RPM
0
20
40
60
80
Length

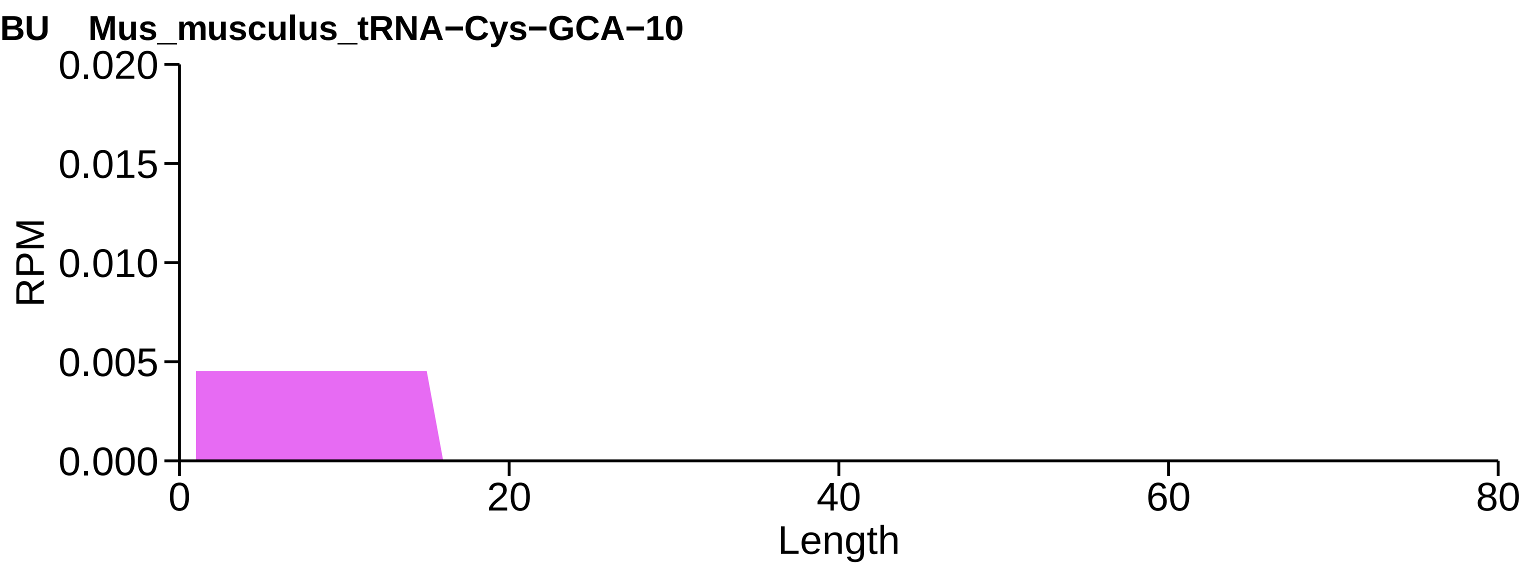

BU Mus_musculus_tRNA−Cys−GCA−10
0.020
0.015
0.010
0.005
0.000
RPM
0
20
40
60
80
Length

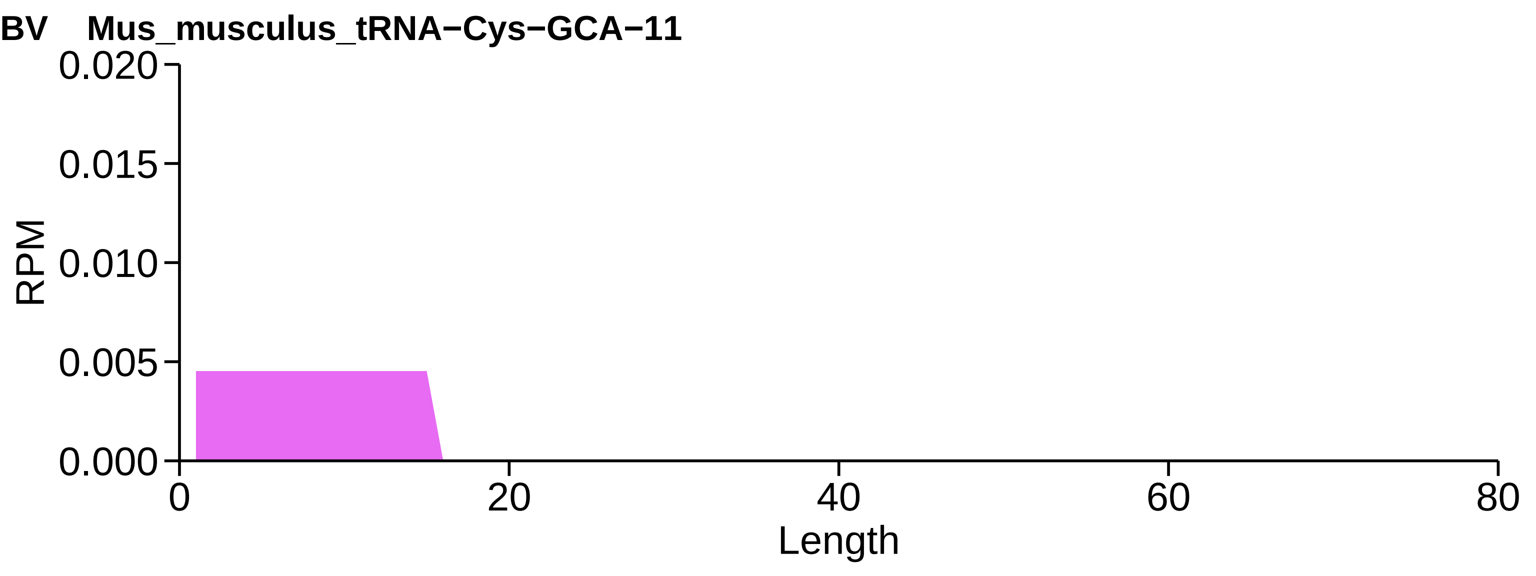

BV Mus_musculus_tRNA−Cys−GCA−11
0.020
0.015
0.010
0.005
0.000
RPM
0
20
40
60
80
Length

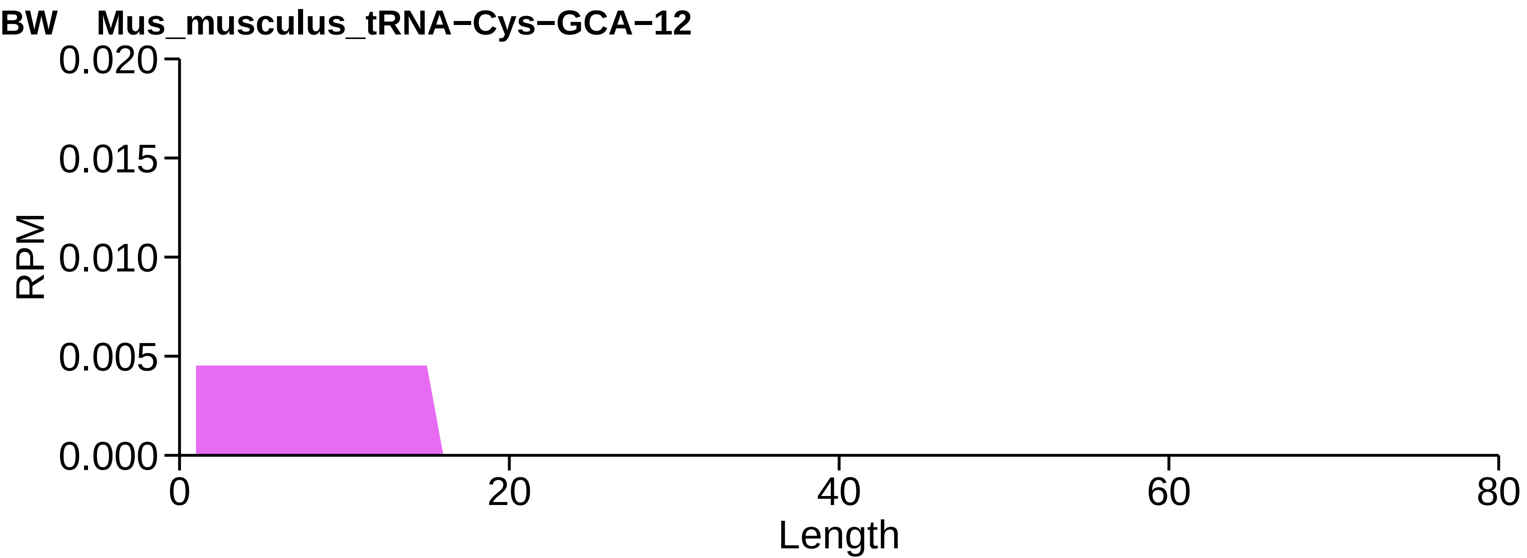

BW Mus_musculus_tRNA−Cys−GCA−12
RPM
0.020
0.015
0.010
0.005
0.000
0
20
40
60
80
Length

BX Mus_musculus_tRNA−Cys−GCA−13

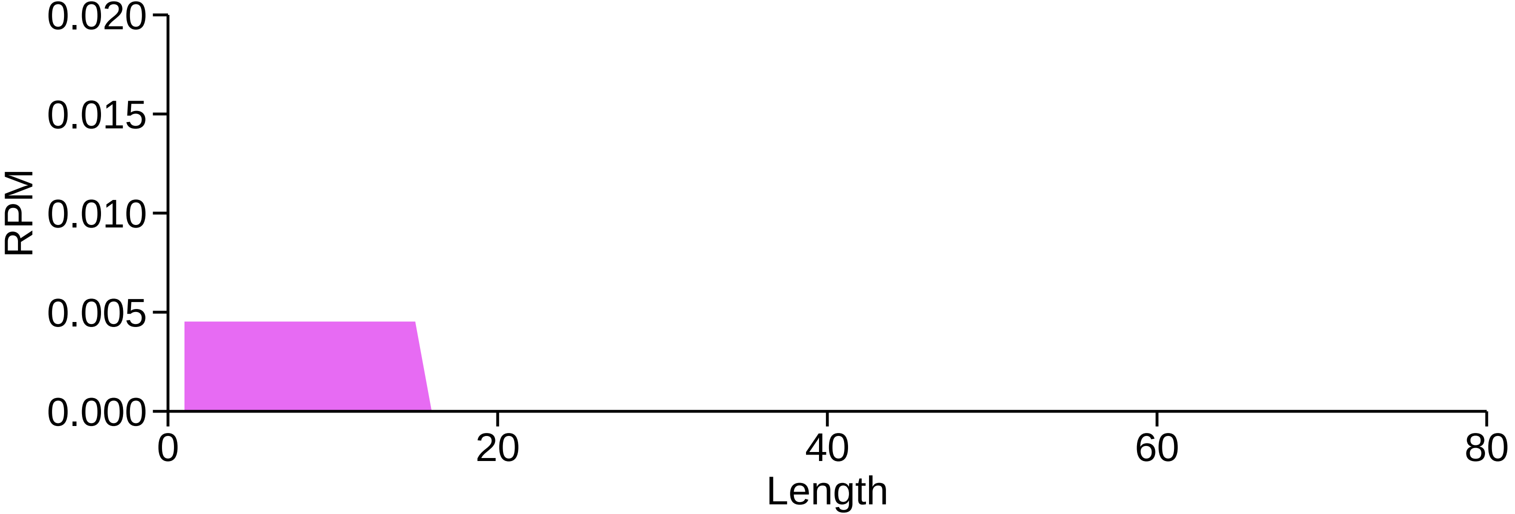

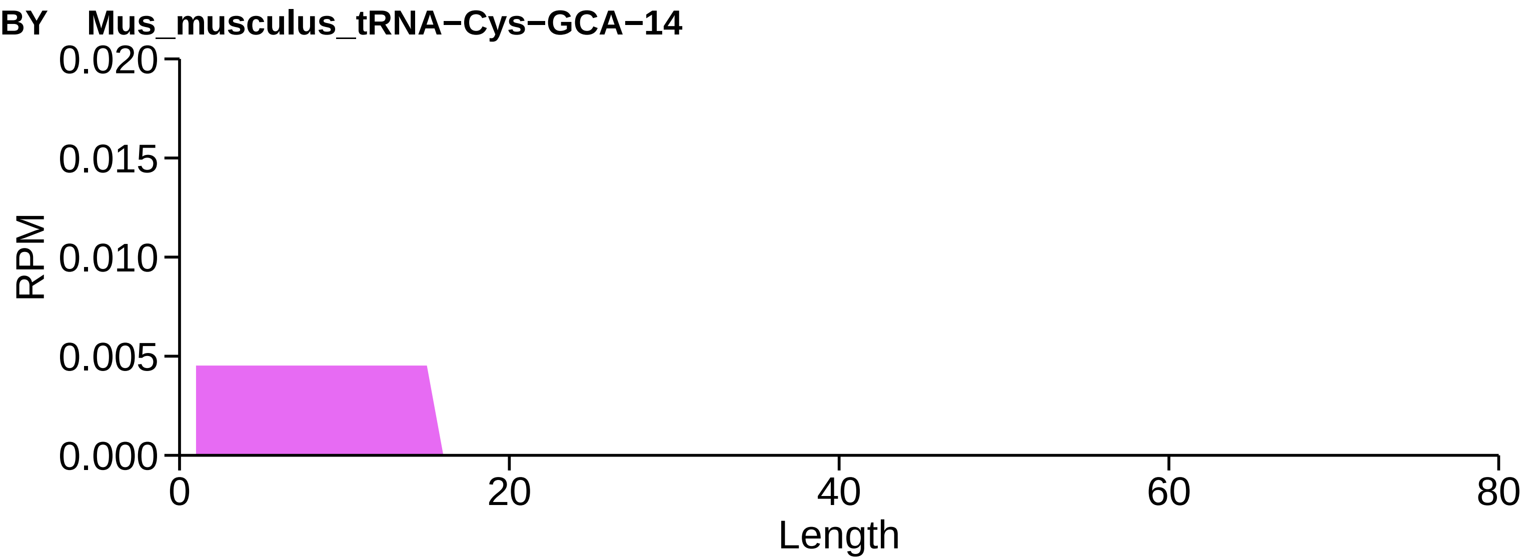
BY Mus_musculus_tRNA−Cys−GCA−14
0.020
0.015
0.010
0.005
0.000
RPM
0
20
40
60
80
Length

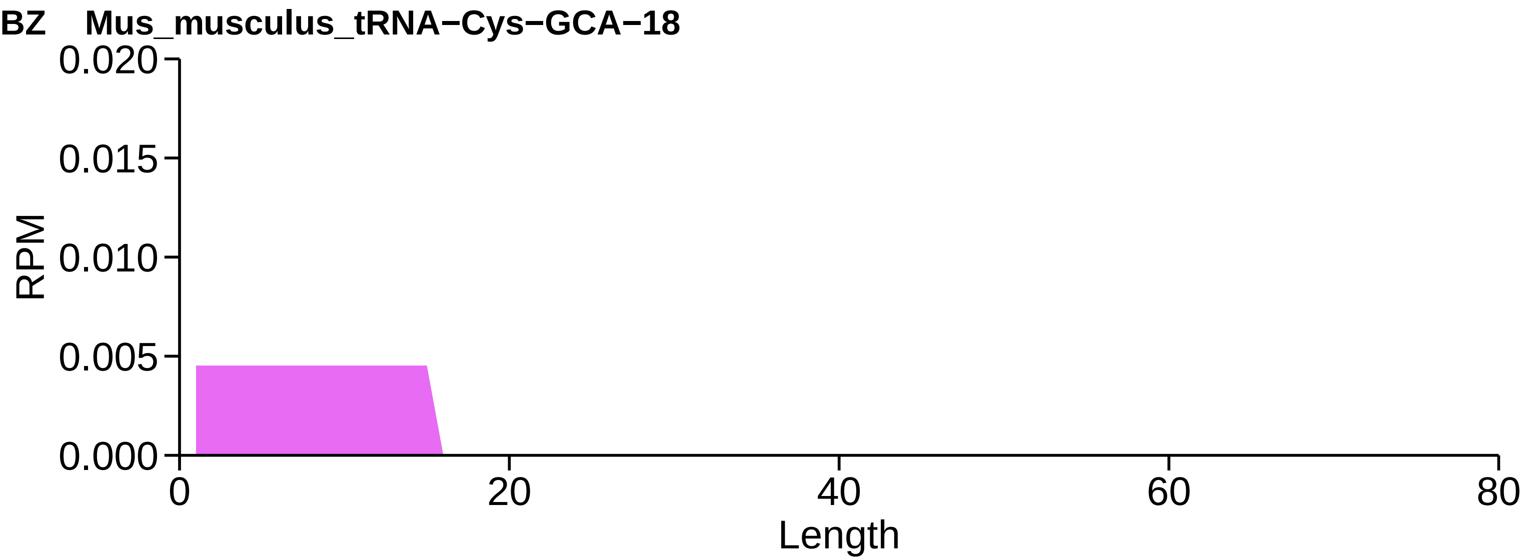
BZ Mus_musculus_tRNA−Cys−GCA−18
RPM
0.020
0.015
0.010
0.005
0.000
0
20
40
60
80
Length

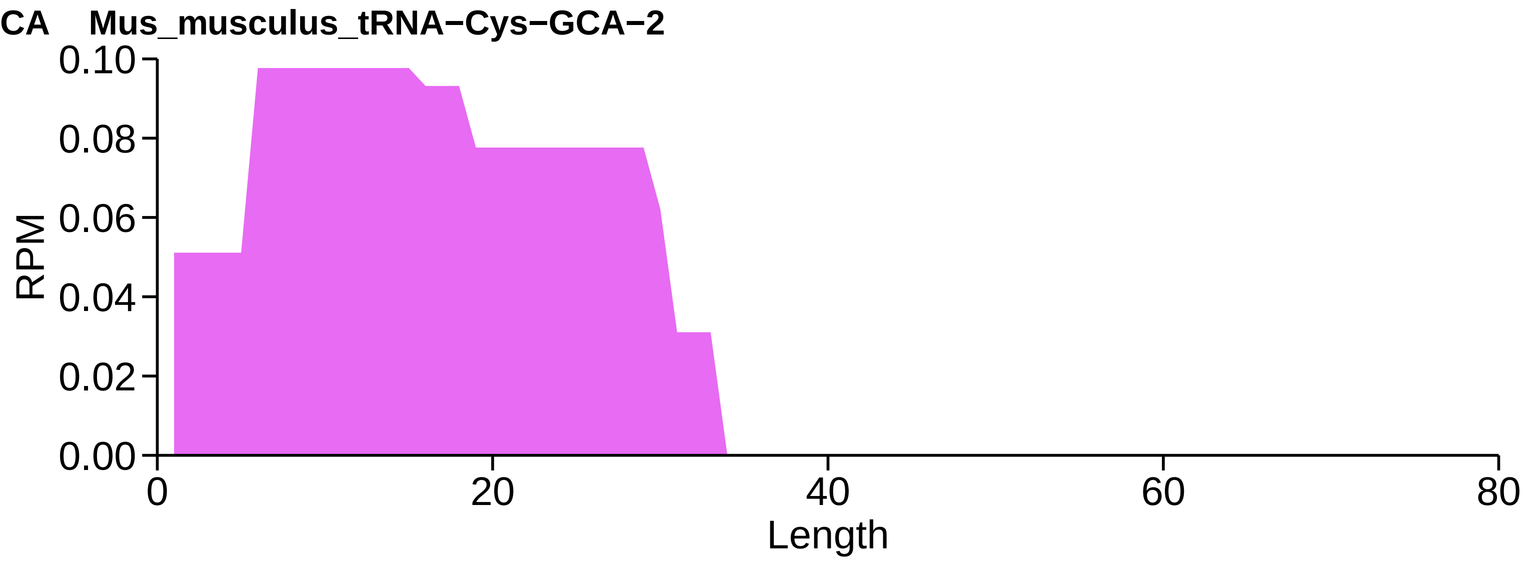
CA Mus_musculus_tRNA−Cys−GCA−2
RPM
0.00
0.02
0.04
0.06
0.08
0.10
0
20
40
60
80
Length

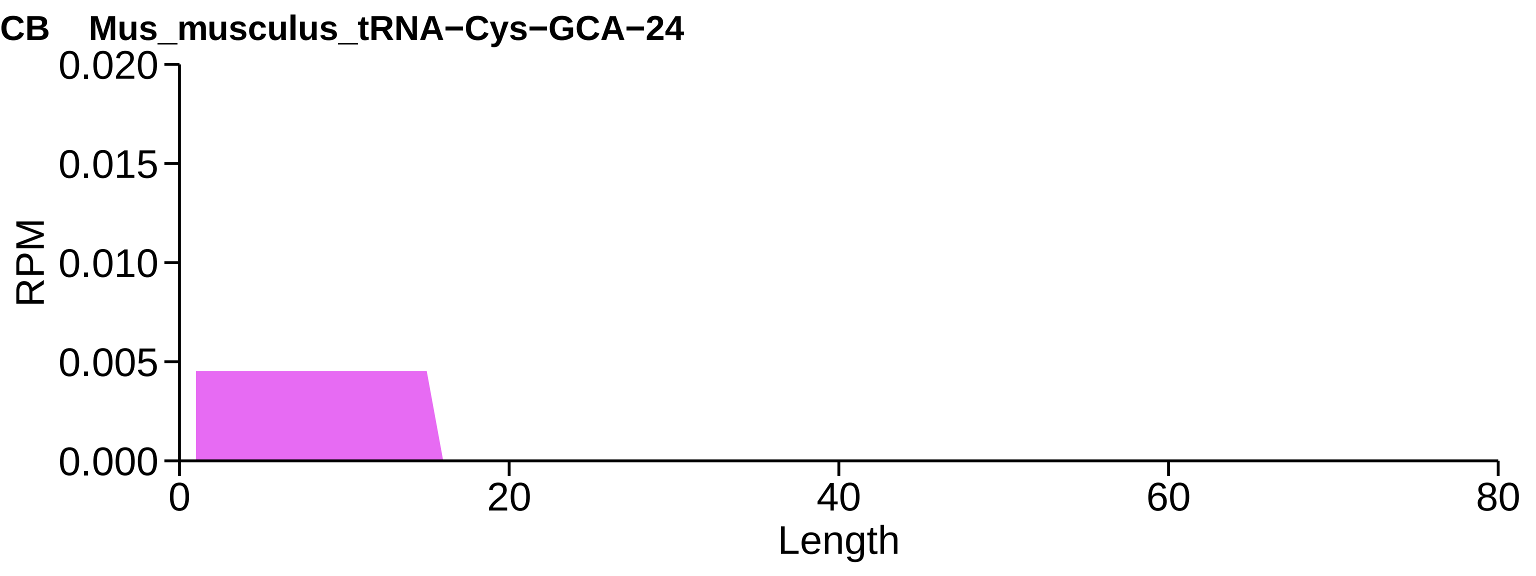

CB Mus_musculus_tRNA−Cys−GCA−24
RPM
0.020
0.015
0.010
0.005
0.000
0
20
40
60
80
Length

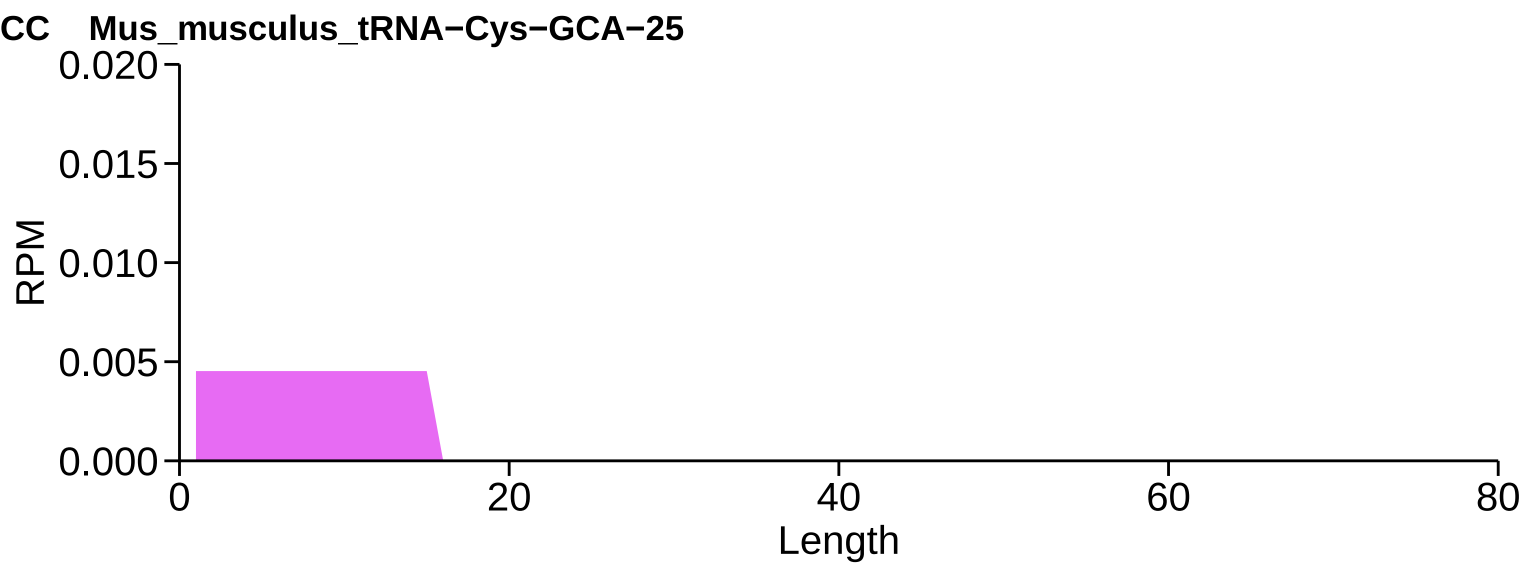

CC Mus_musculus_tRNA−Cys−GCA−25
0.020
0.015
0.010
0.005
0.000
RPM
0
20
40
60
80
Length

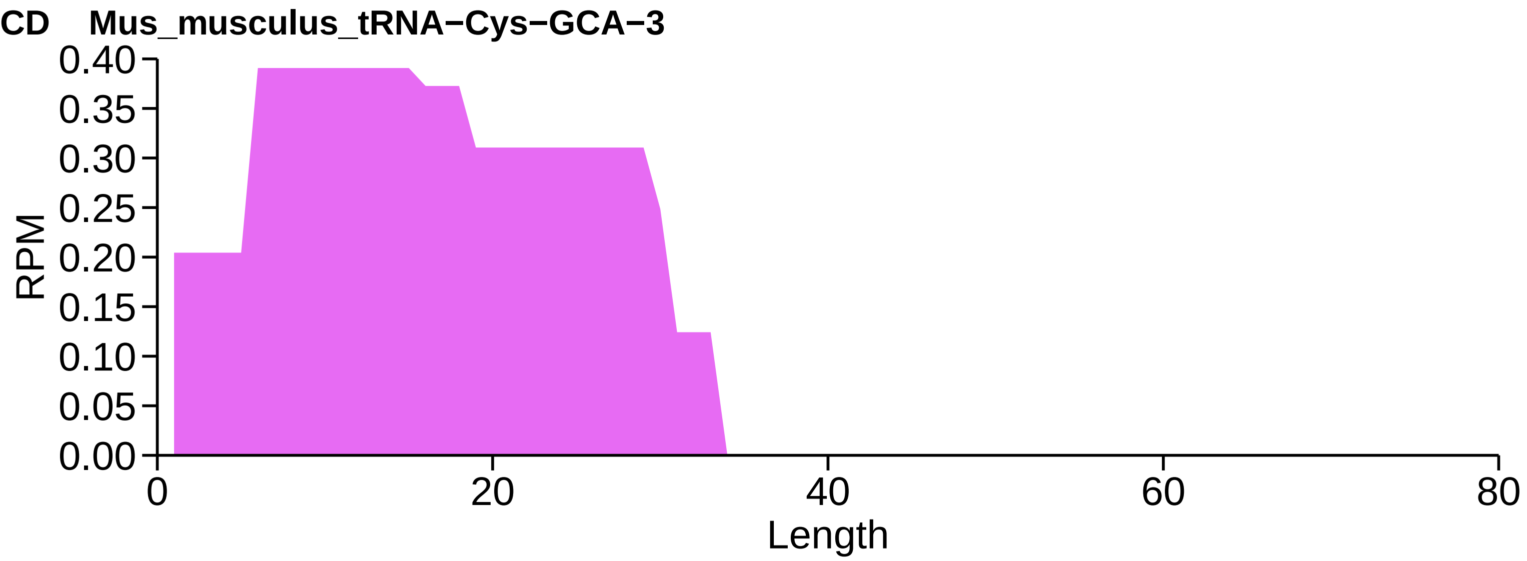
CD Mus_musculus_tRNA−Cys−GCA−3
RPM
0.40
0.35
0.30
0.25
0.20
0.15
0.10
0.05
0.00
0
20
40
60
80
Length

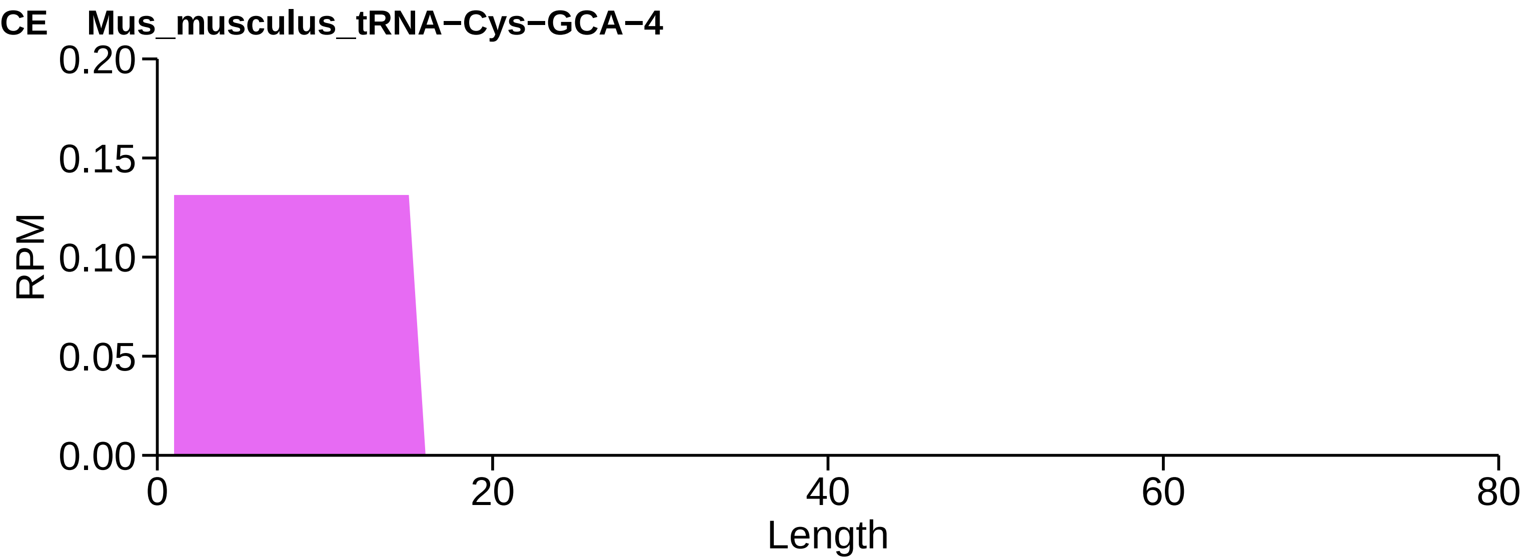
CE Mus_musculus_tRNA−Cys−GCA−4
0.20
0.15
0.10
0.05
0.00
RPM
0
20
40
60
80
Length

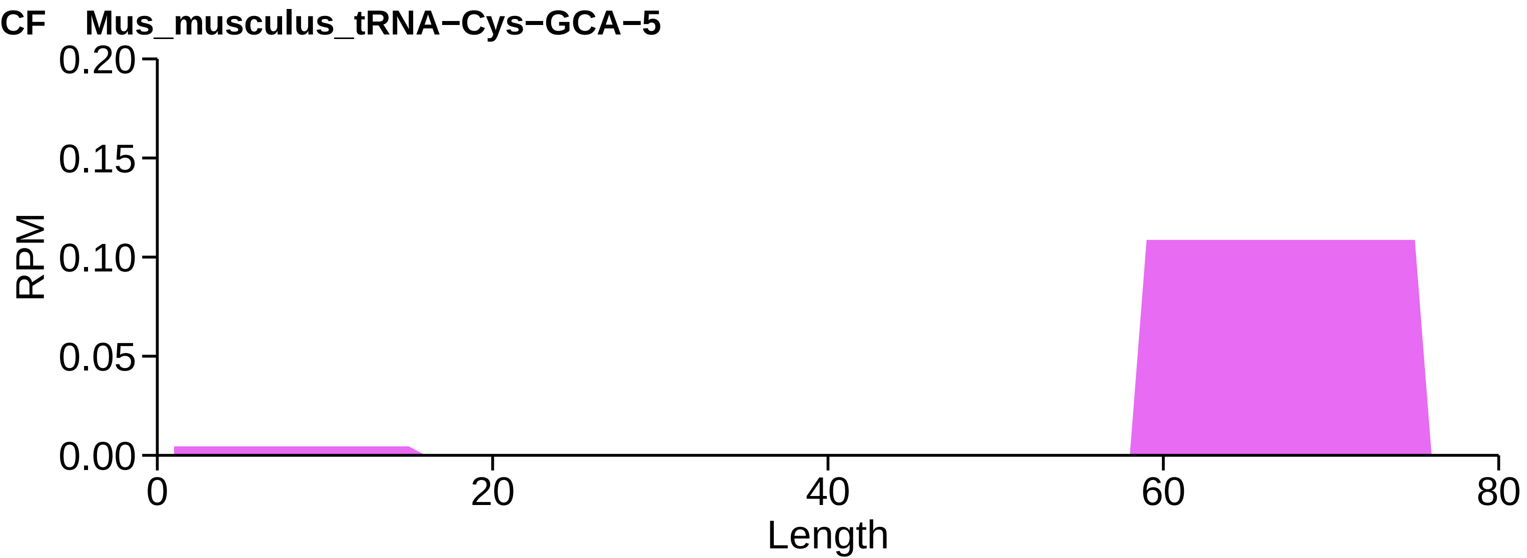

CF Mus_musculus_tRNA−Cys−GCA−5
RPM
0.20
0.15
0.10
0.05
0.00
0
20
40
60
80
Length

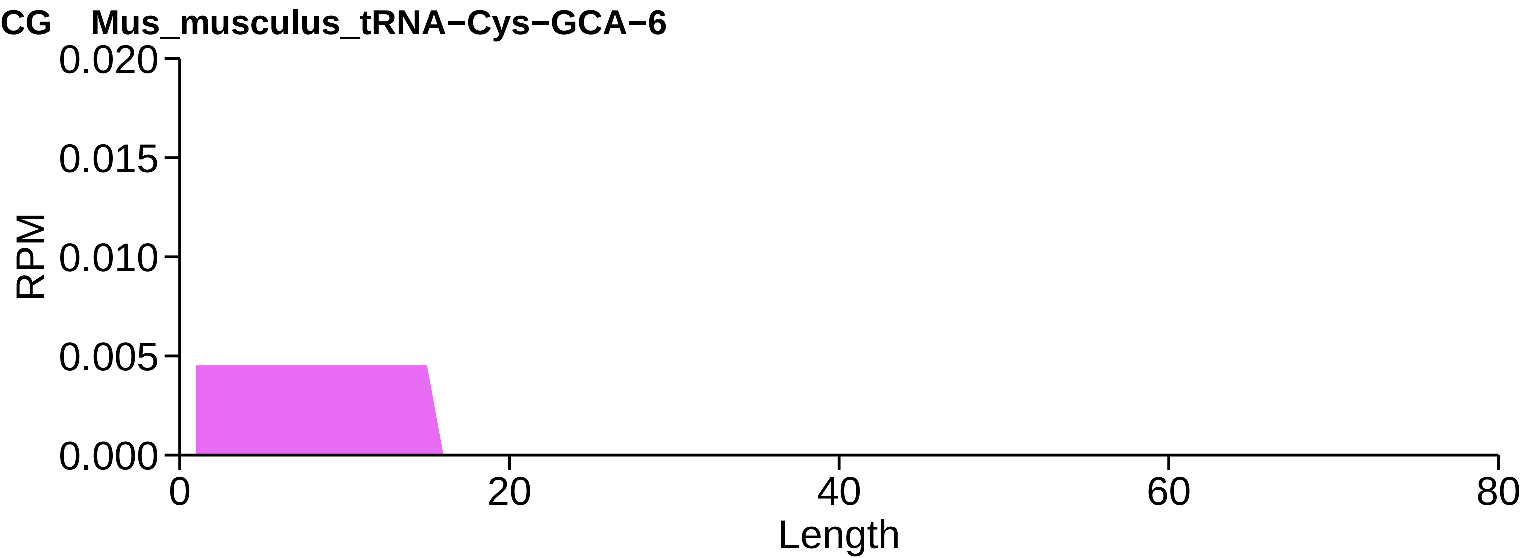
CG Mus_musculus_tRNA−Cys−GCA−6
RPM
0.020
0.015
0.010
0.005
0.000
0
20
40
60
80
Length

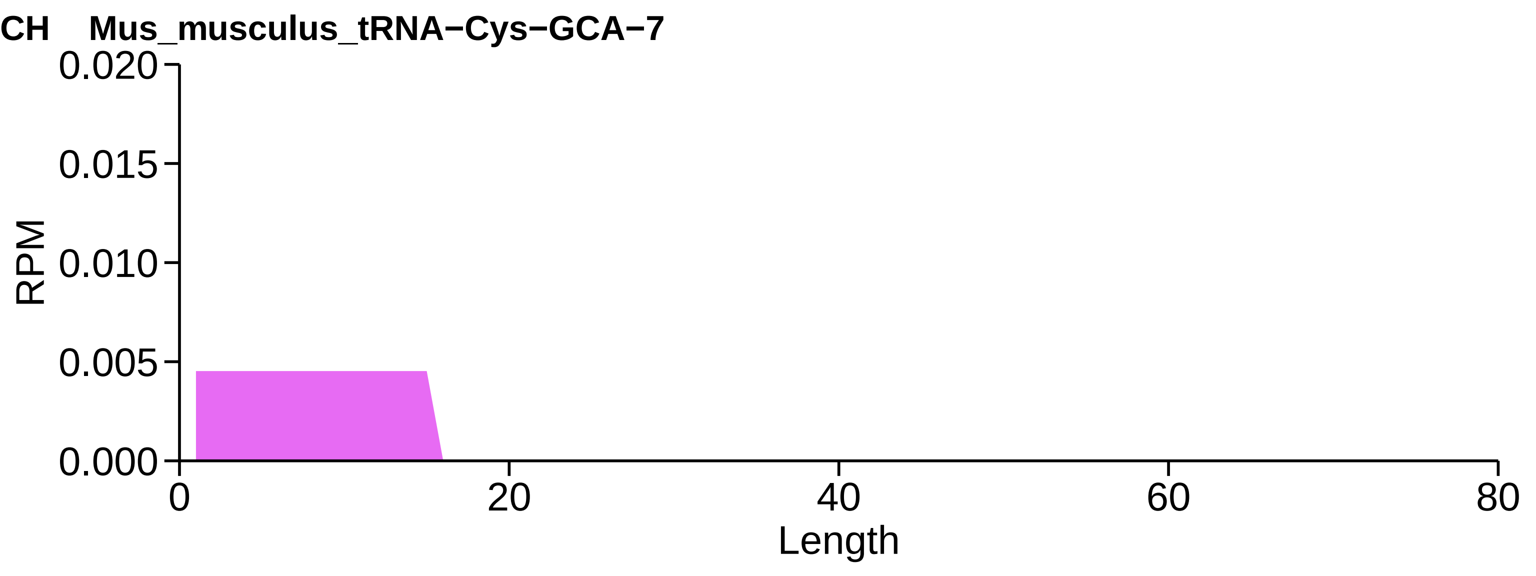
CH Mus_musculus_tRNA−Cys−GCA−7
RPM
0.000
0.005
0.010
0.015
0.020
0
20
40
60
80
Length

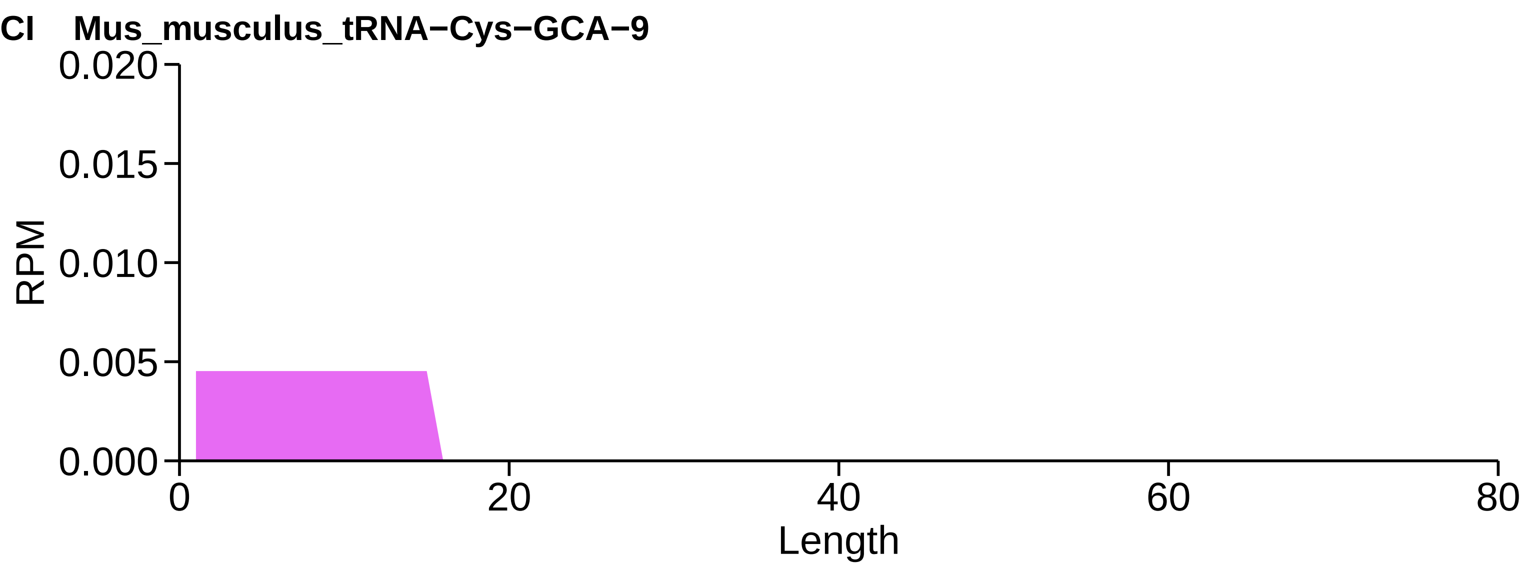
CI Mus_musculus_tRNA−Cys−GCA−9
0.020
0.015
0.010
0.005
0.000
RPM
0
20
40
60
80
Length

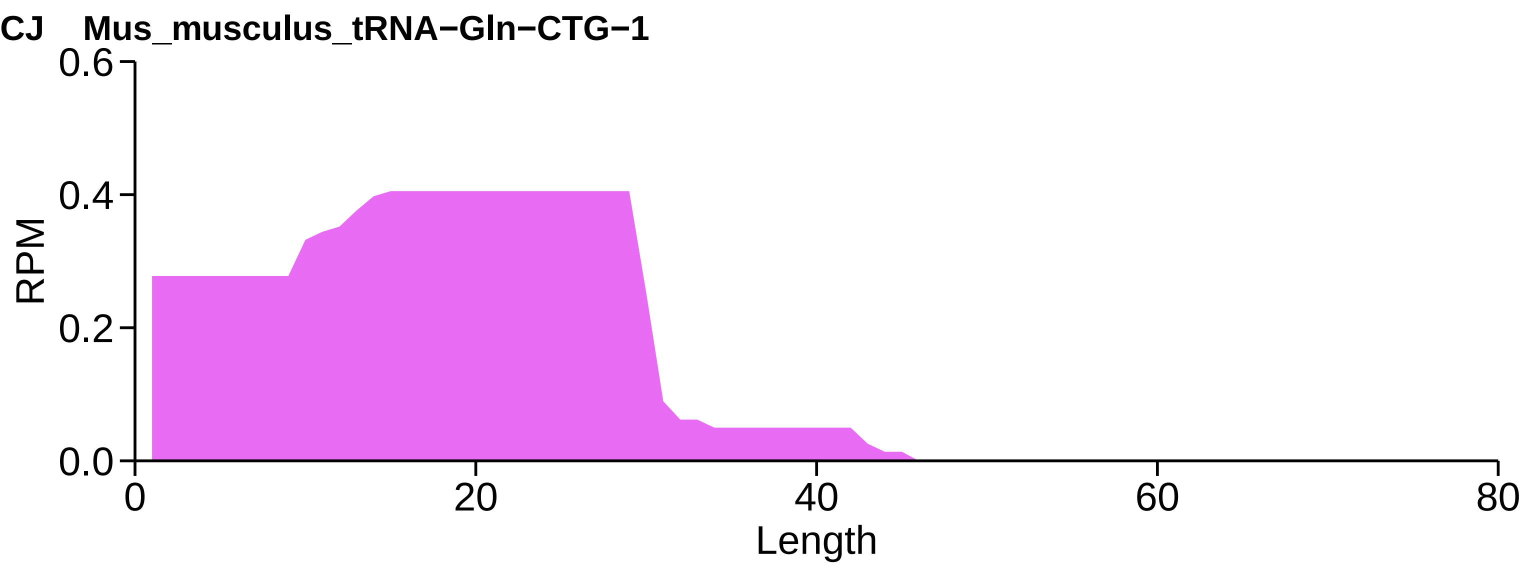

CJ Mus_musculus_tRNA−Gln−CTG−1
RPM
0.0
0.2
0.4
0.6
0
20
40
60
80
Length

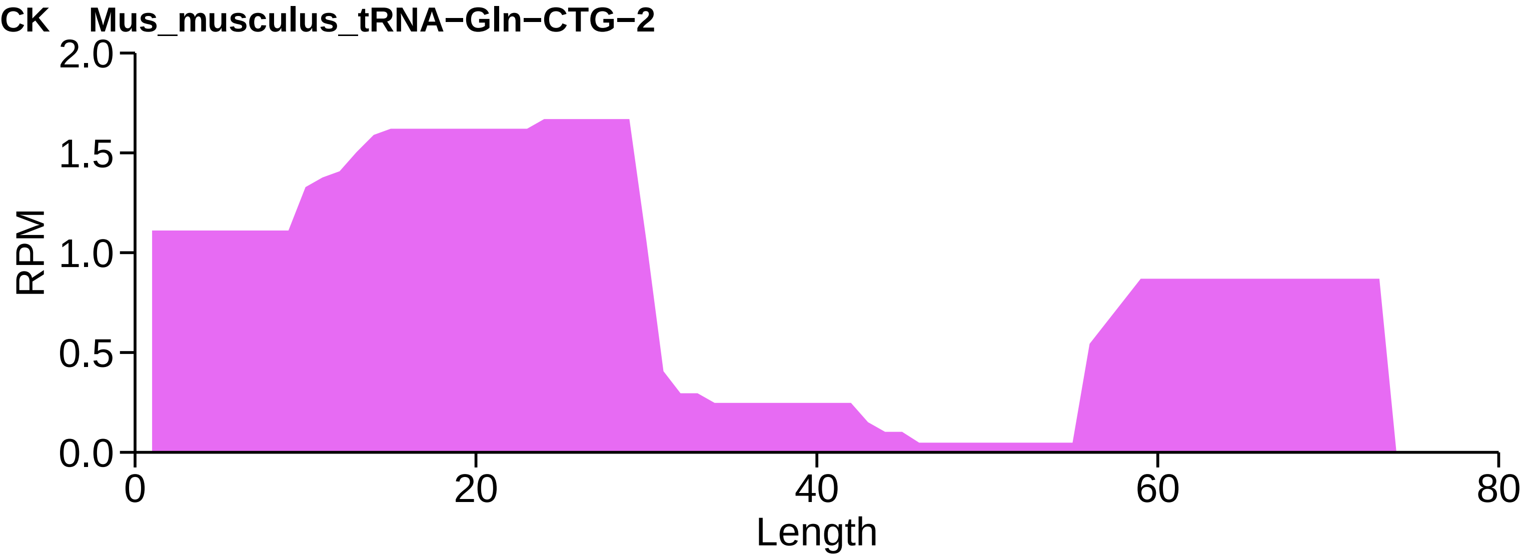
CK Mus_musculus_tRNA−Gln−CTG−2
RPM
2.0
1.5
1.0
0.5
0.0
0
20
40
60
80
Length

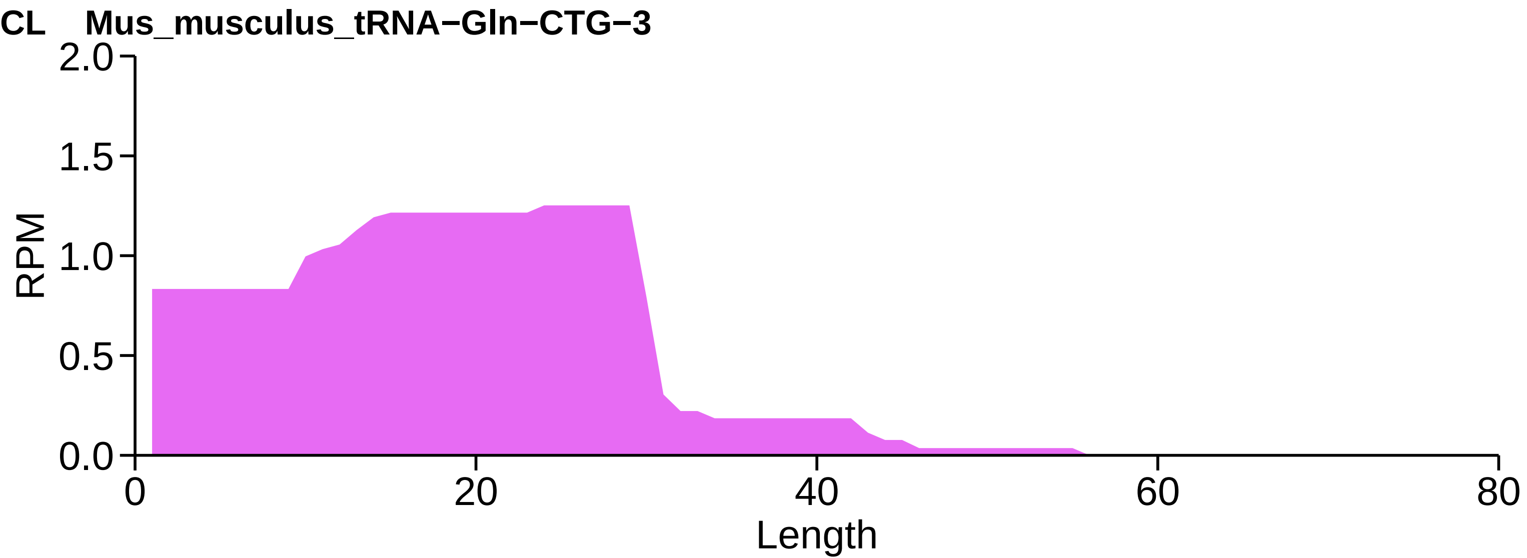

CL Mus_musculus_tRNA−Gln−CTG−3
2.0
1.5
1.0
0.5
0.0
RPM
0
20
40
60
80
Length

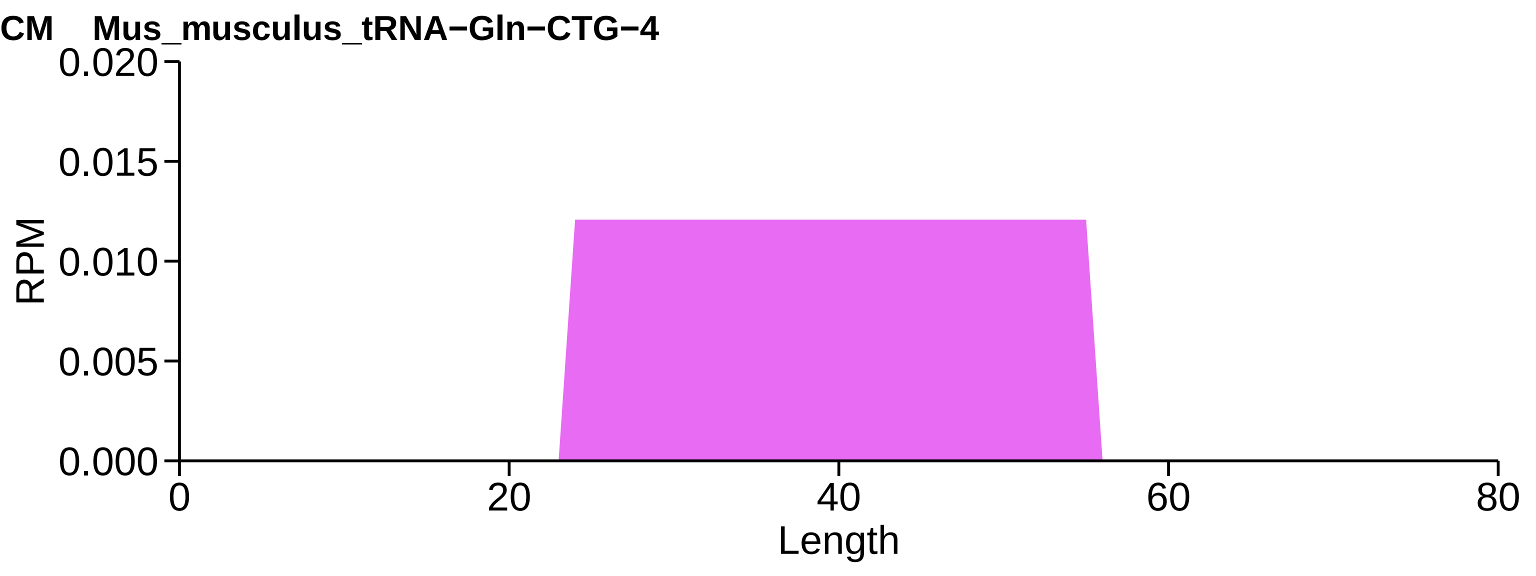
CM Mus_musculus_tRNA−Gln−CTG−4
0.020
0.015
0.010
0.005
0.000
RPM
0
20
40
60
80
Length

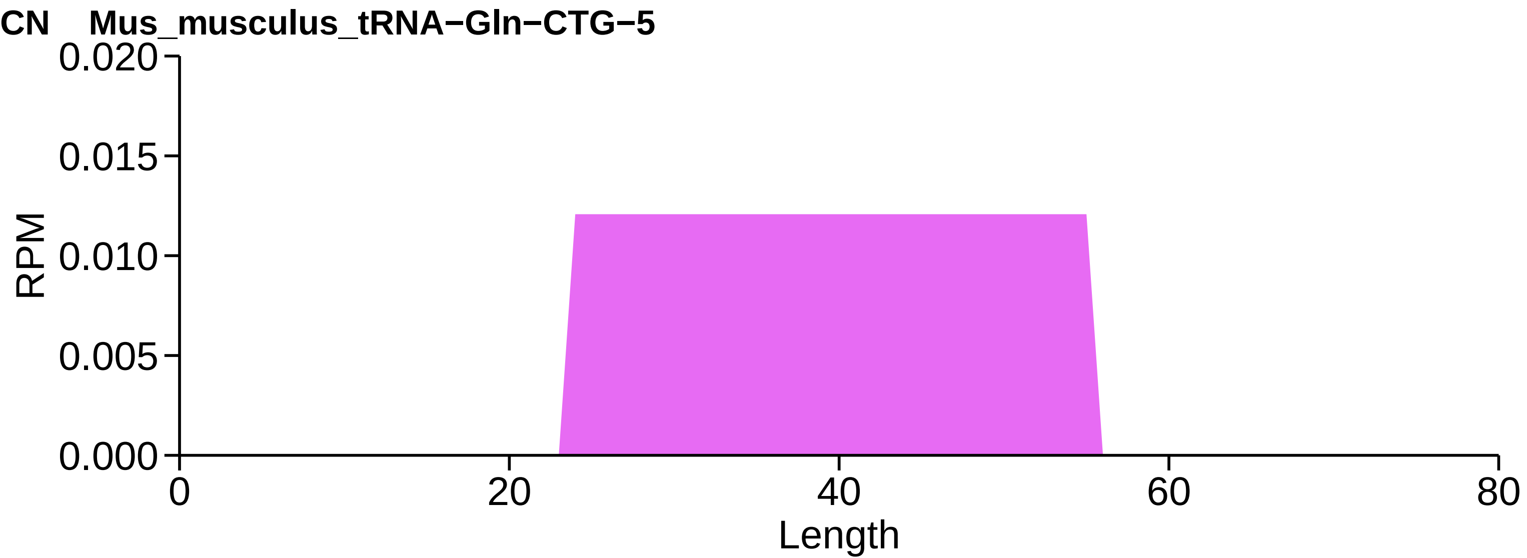
CN Mus_musculus_tRNA−Gln−CTG−5
0.020
0.015
0.010
0.005
0.000
RPM
0
20
40
60
80
Length

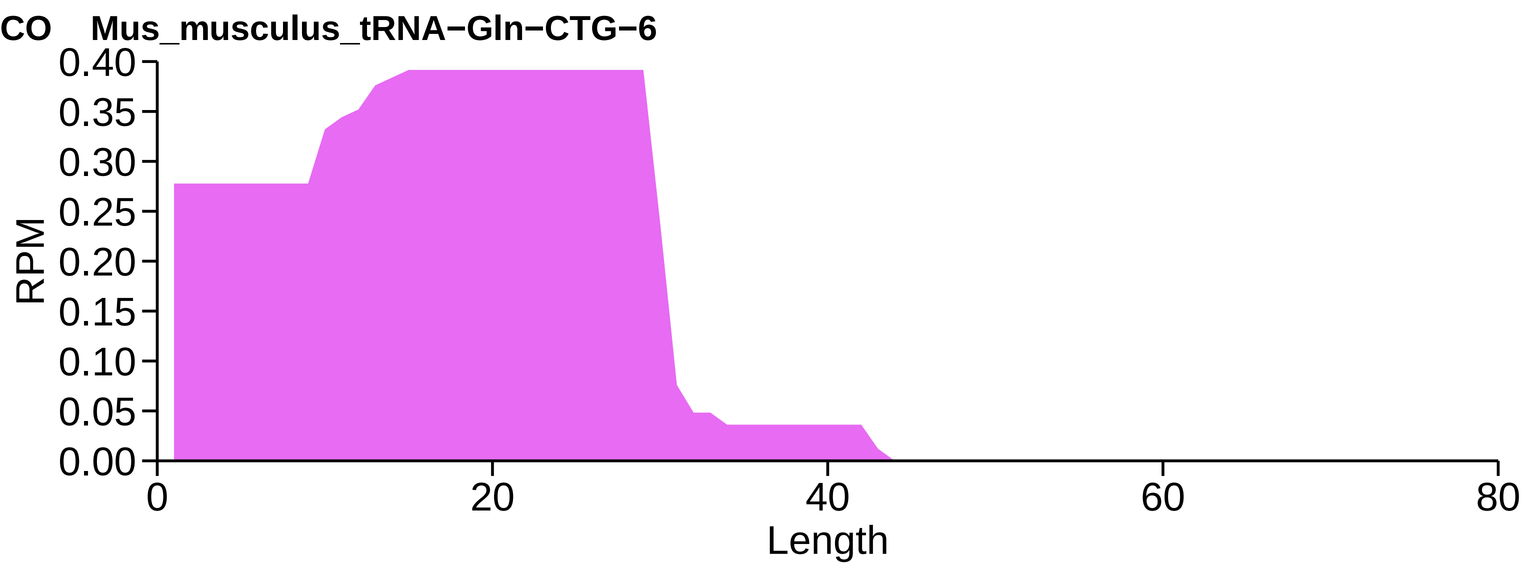

CO Mus_musculus_tRNA−Gln−CTG−6
RPM
0.00
0.05
0.10
0.15
0.20
0.25
0.30
0.35
0.40
0
20
40
60
80
Length

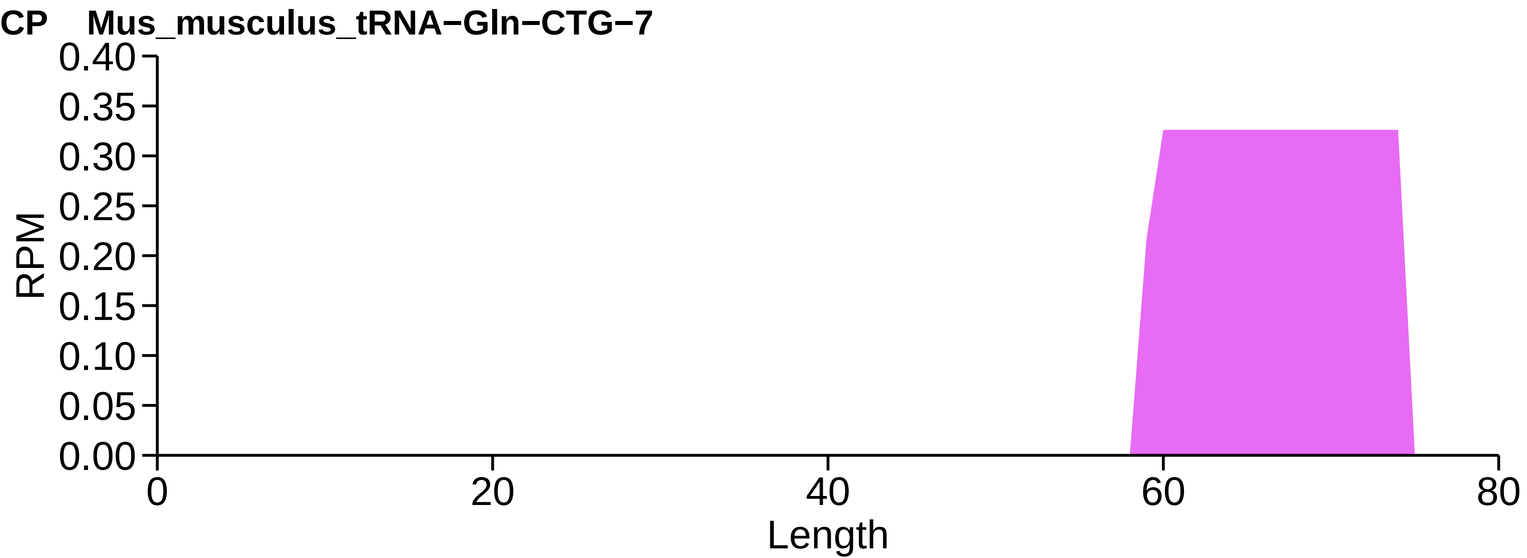

CP Mus_musculus_tRNA−Gln−CTG−7
0.40
0.35
0.30
0.25
0.20
0.15
0.10
0.05
0.00
RPM
0
20
40
60
80
Length

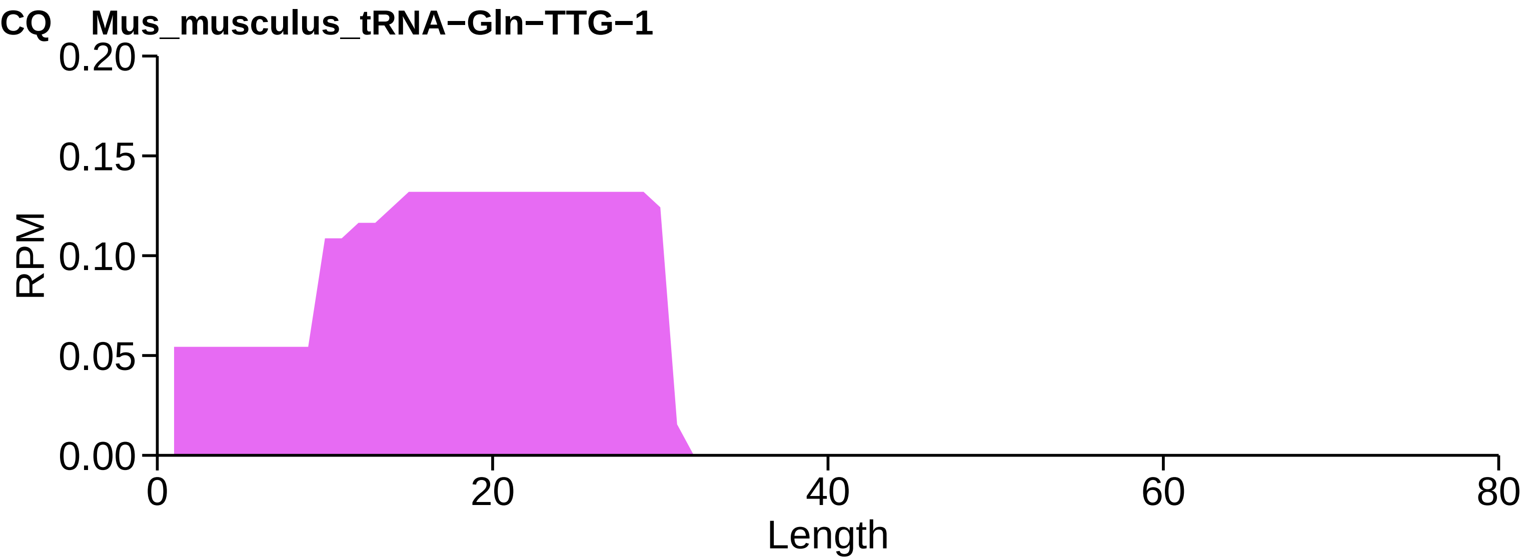
CQ Mus_musculus_tRNA−Gln−TTG−1
0.20
0.15
0.10
0.05
0.00
RPM
0
20
40
60
80
Length

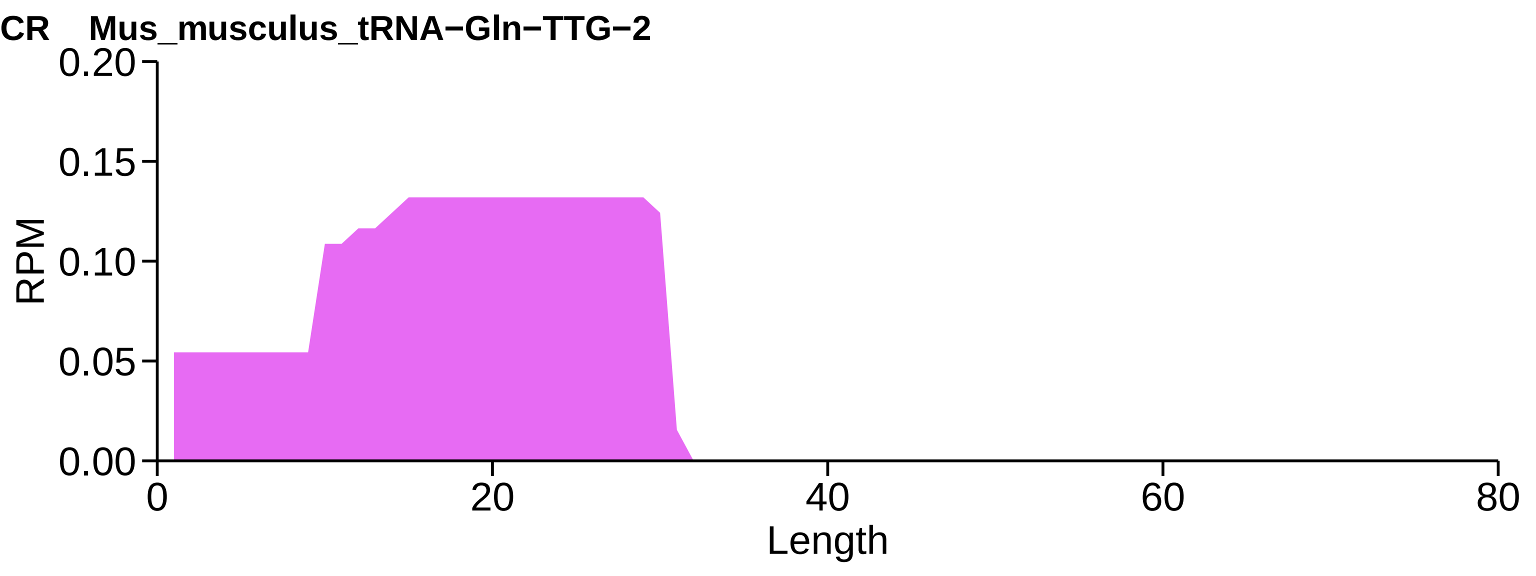
CR Mus_musculus_tRNA−Gln−TTG−2
0.20
0.15
0.10
0.05
0.00
RPM
0
20
40
60
80
Length

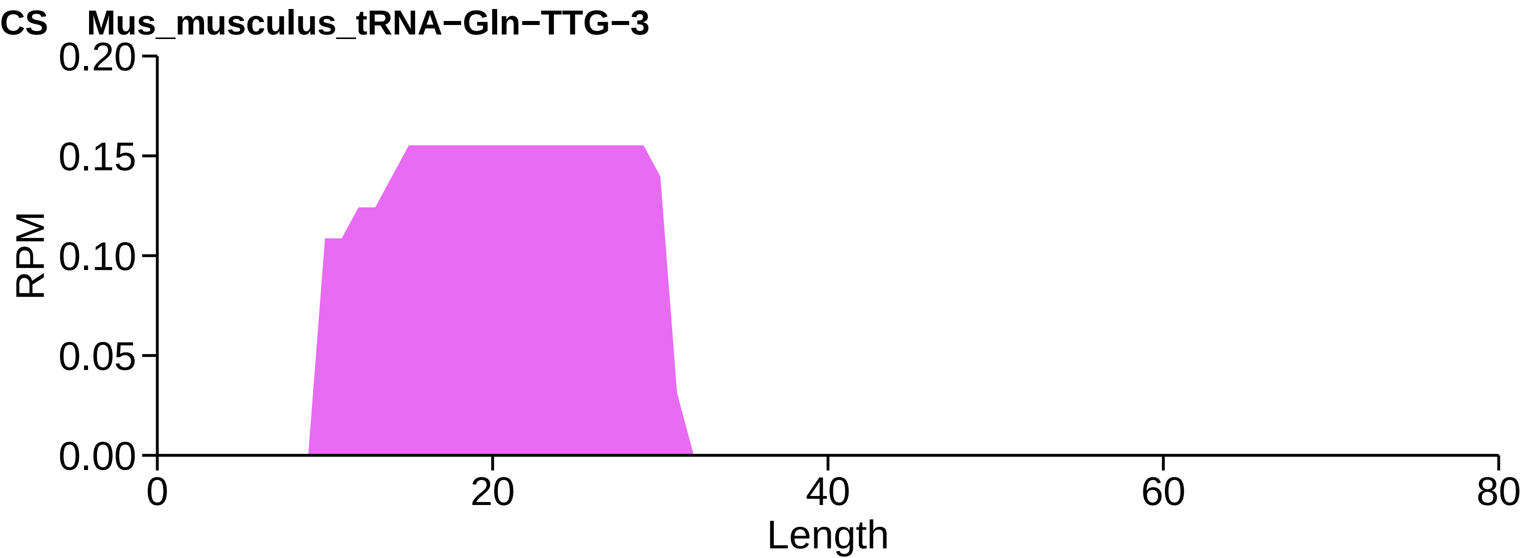
CS Mus_musculus_tRNA−Gln−TTG−3
RPM
0.20
0.15
0.10
0.05
0.00
0
20
40
60
80
Length

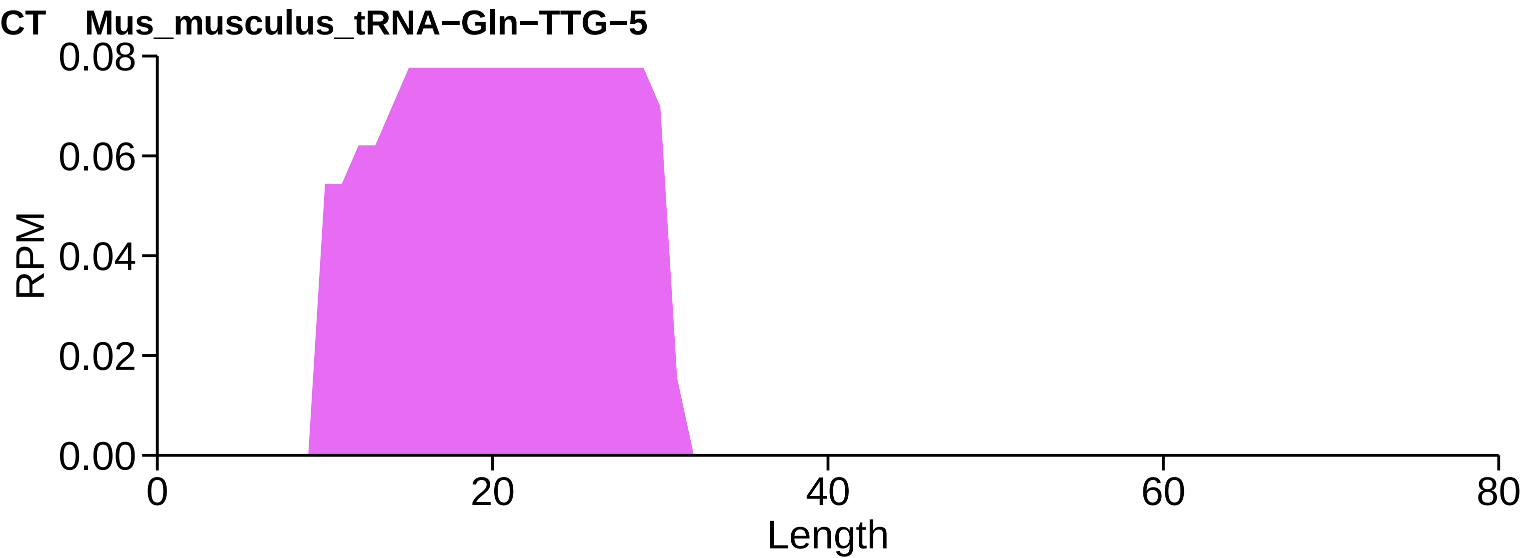
CT Mus_musculus_tRNA−Gln−TTG−5
0.08
0.06
0.04
0.02
0.00
RPM
0
20
40
60
80
Length

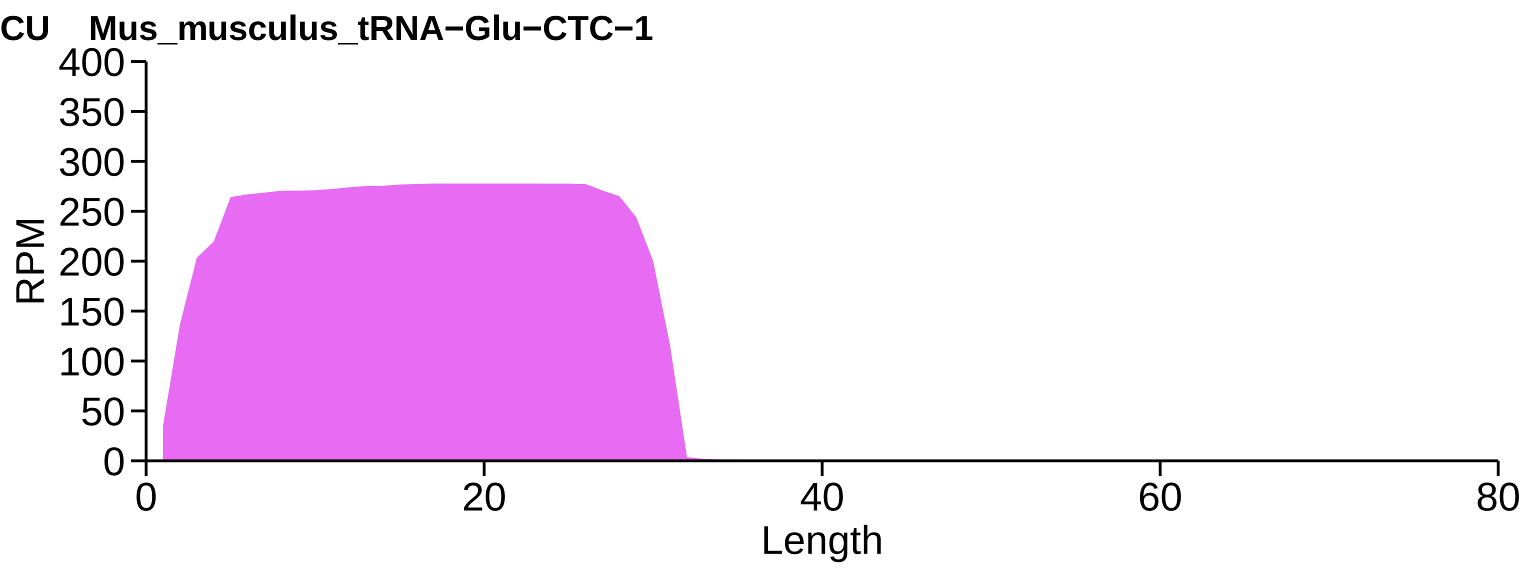
CU Mus_musculus_tRNA−Glu−CTC−1
RPM
400
350
300
250
200
150
100
50
0
0
20
40
60
80
Length

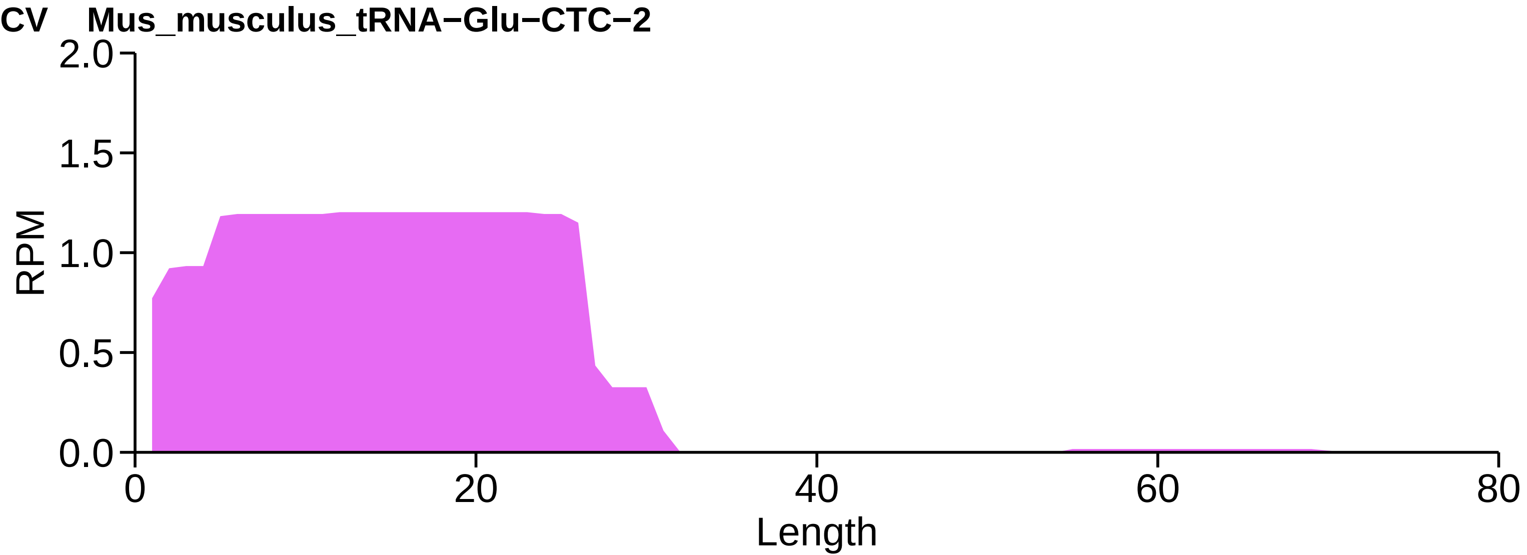
CV
Mus_musculus_tRNA−Glu−CTC−2
RPM
2.0
1.5
1.0
0.5
0.0
0
20
40
60
80
Length

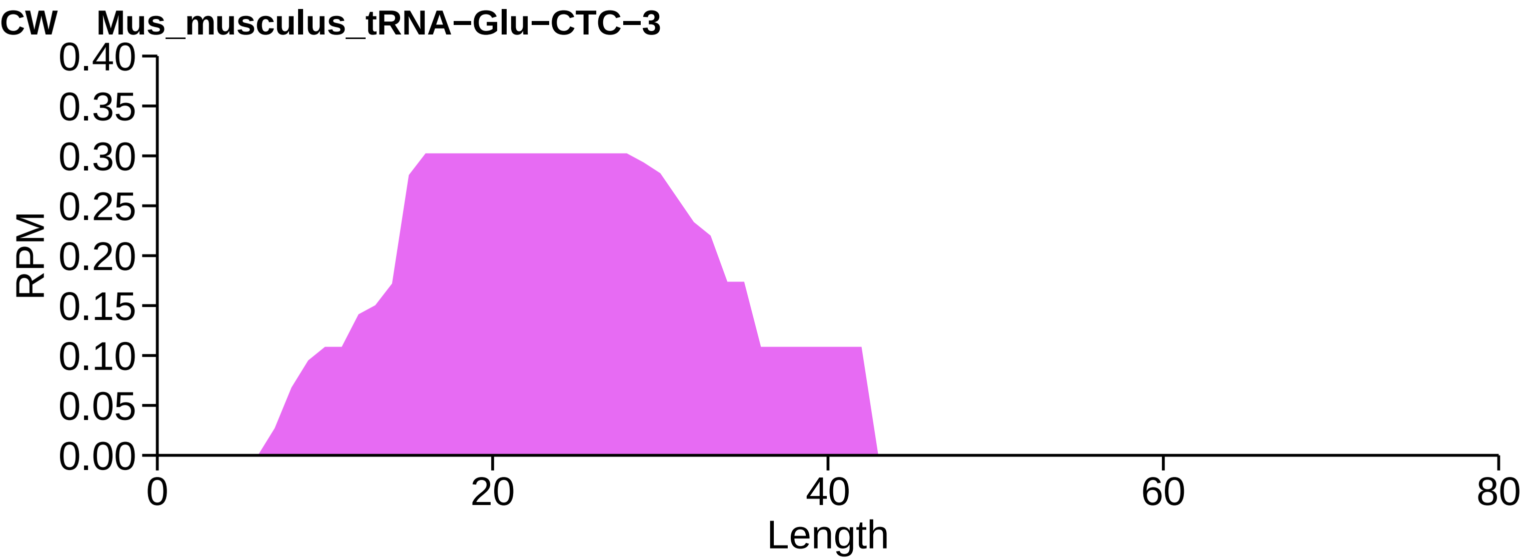

CW Mus_musculus_tRNA−Glu−CTC−3
RPM
0.00
0.05
0.10
0.15
0.20
0.25
0.30
0.35
0.40
0
20
40
60
80
Length

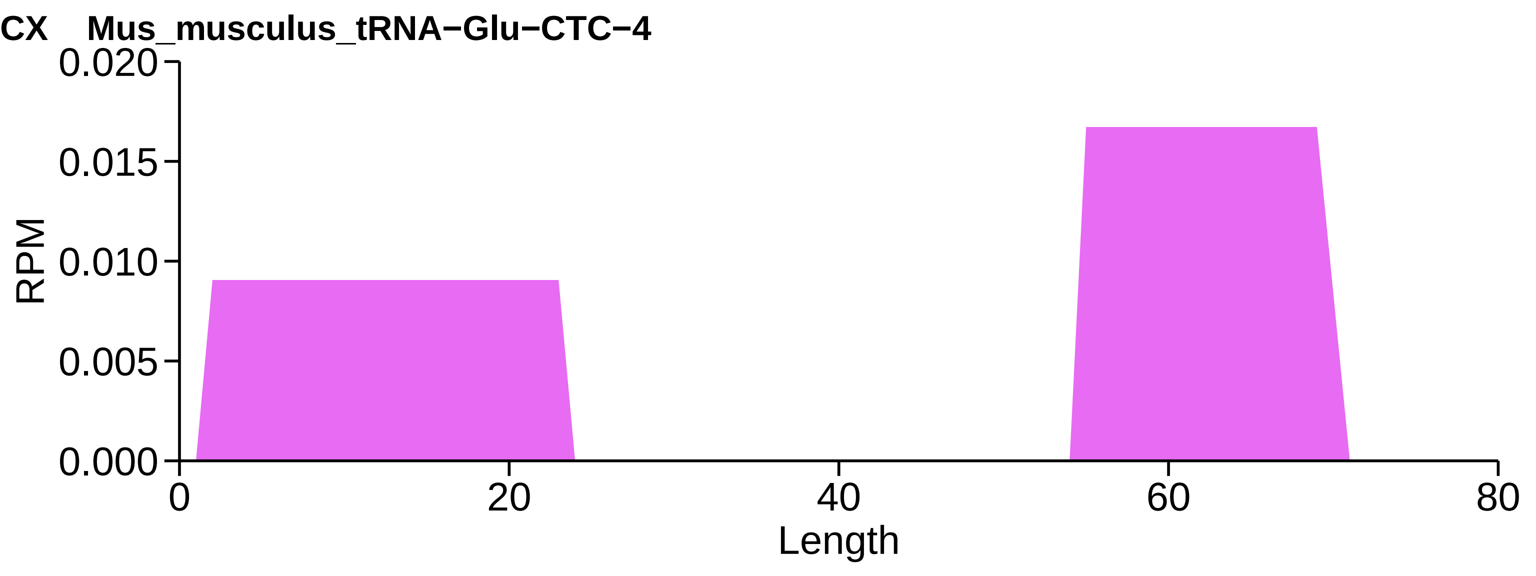

CX Mus_musculus_tRNA−Glu−CTC−4
0.020
0.015
0.010
0.005
0.000
RPM
0
20
40
60
80
Length

CY Mus_musculus_tRNA−Glu−CTC−5

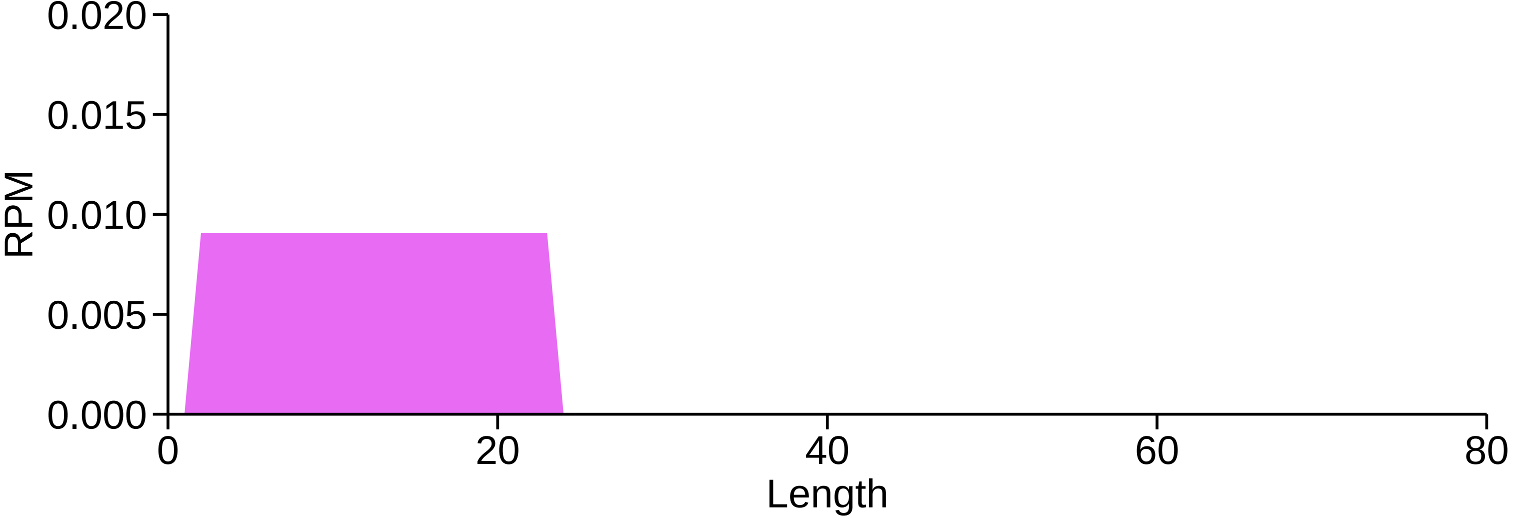

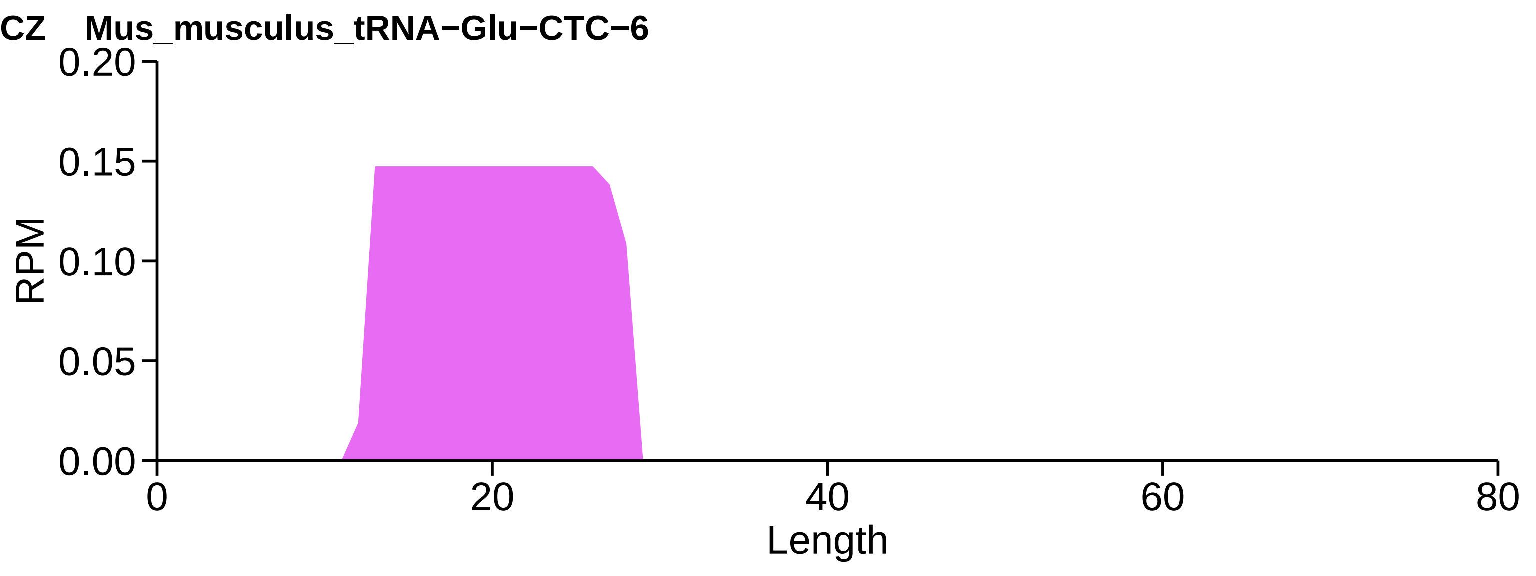
CZ Mus_musculus_tRNA−Glu−CTC−6
RPM
0.20
0.15
0.10
0.05
0.00
0
20
40
60
80
Length

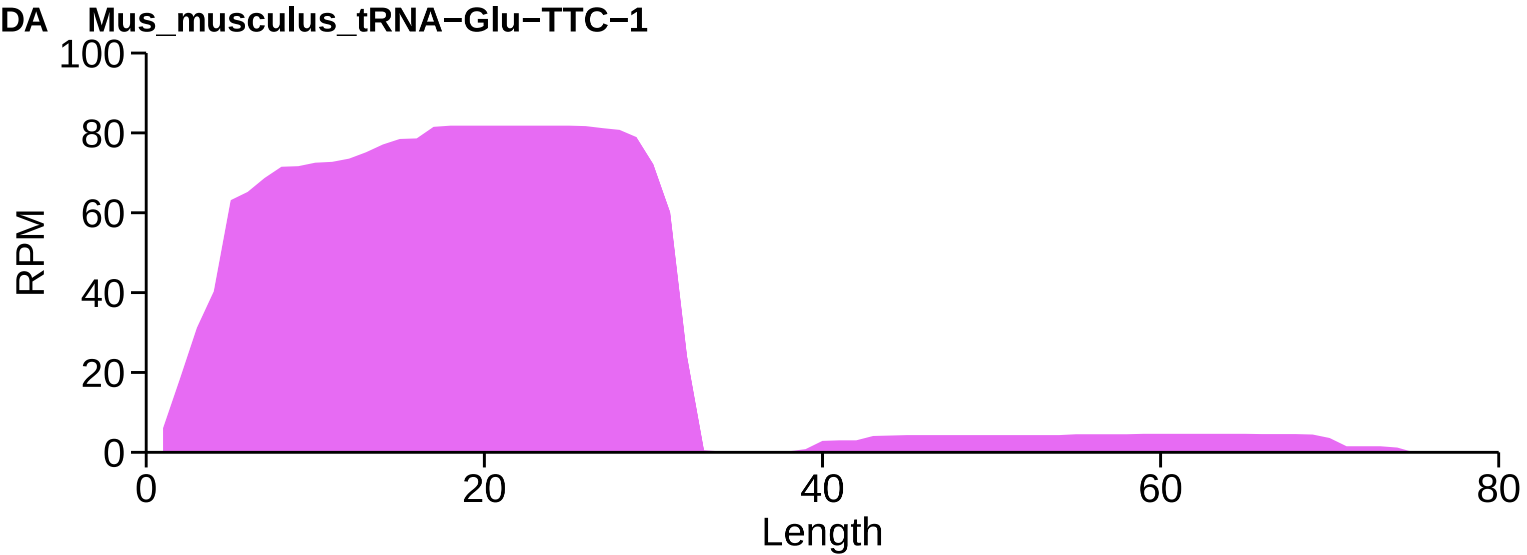

DA Mus_musculus_tRNA−Glu−TTC−1
RPM
Length
0
20
40
60
80
100

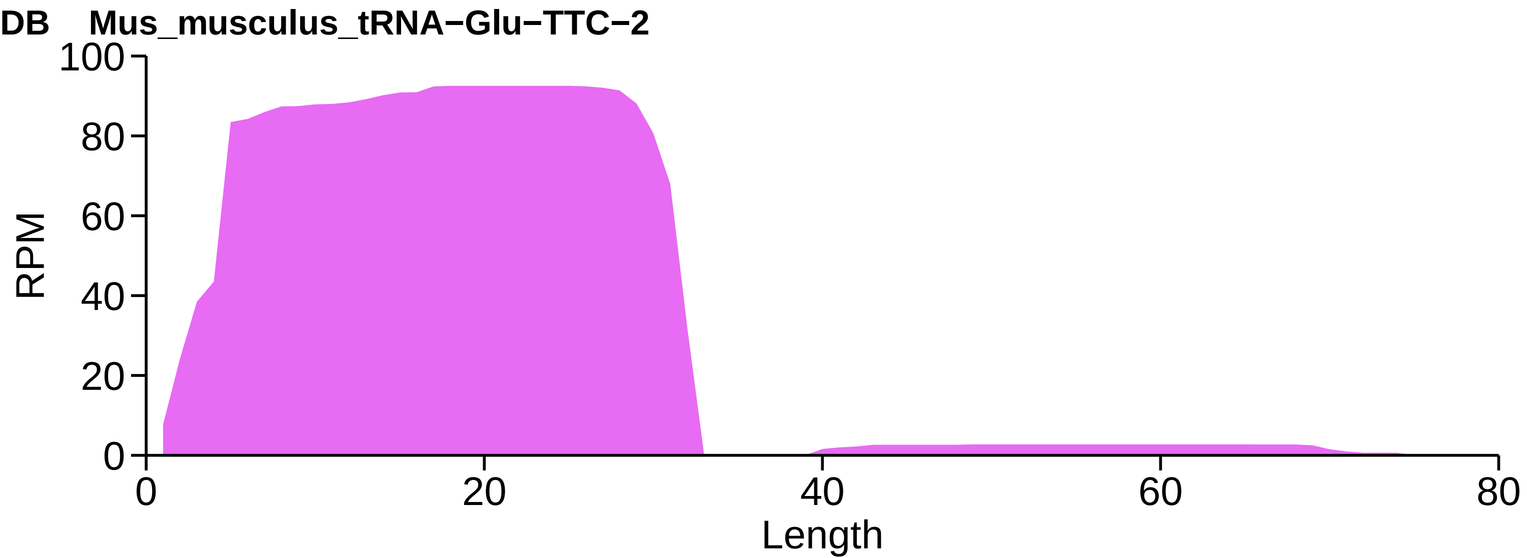
DB Mus_musculus_tRNA−Glu−TTC−2
RPM
100
80
60
40
20
0
0
20
40
60
80
Length

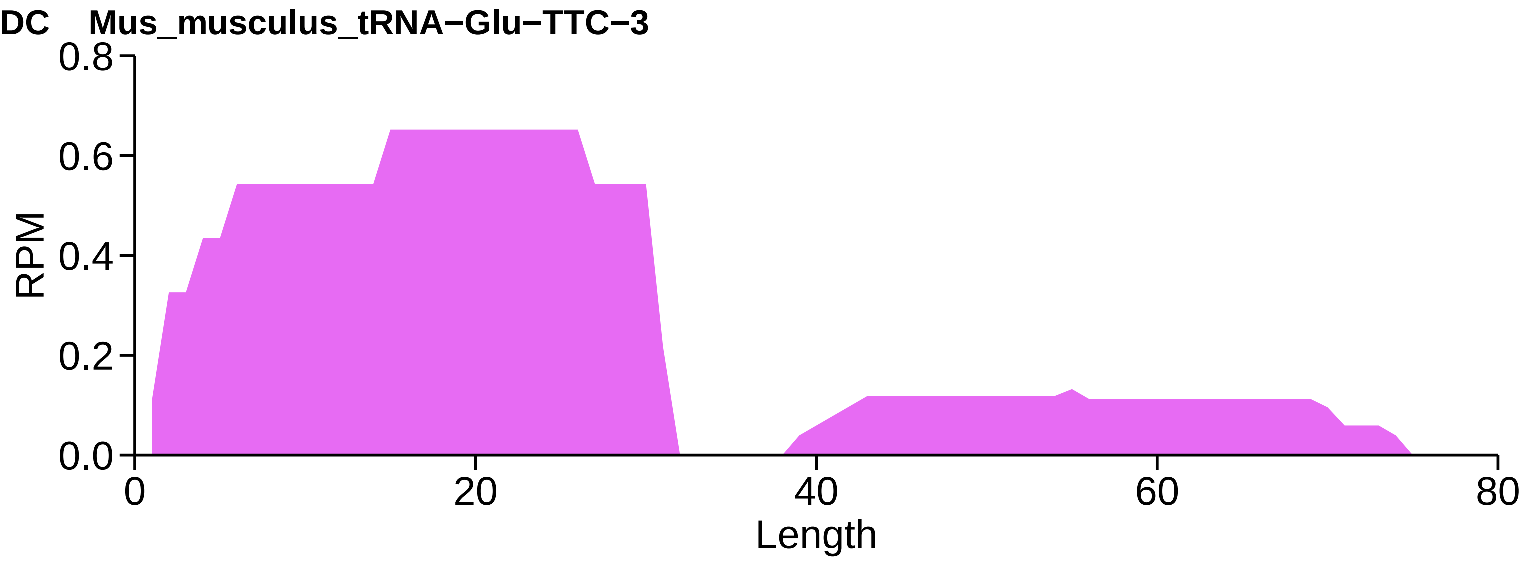
DC
Mus_musculus_tRNA−Glu−TTC−3
0.8
0.6
0.4
0.2
0.0
RPM
0
20
40
60
80
Length

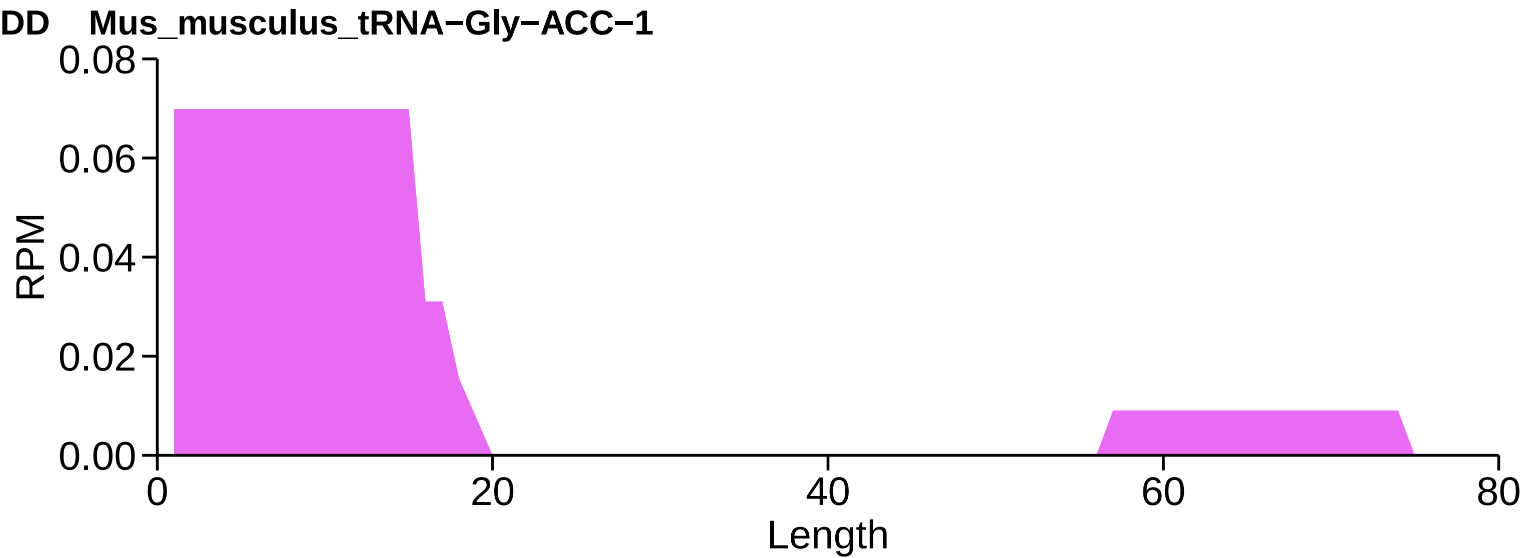

DD Mus_musculus_tRNA−Gly−ACC−1
0.08
0.06
0.04
0.02
0.00
RPM
0
20
40
60
80
Length

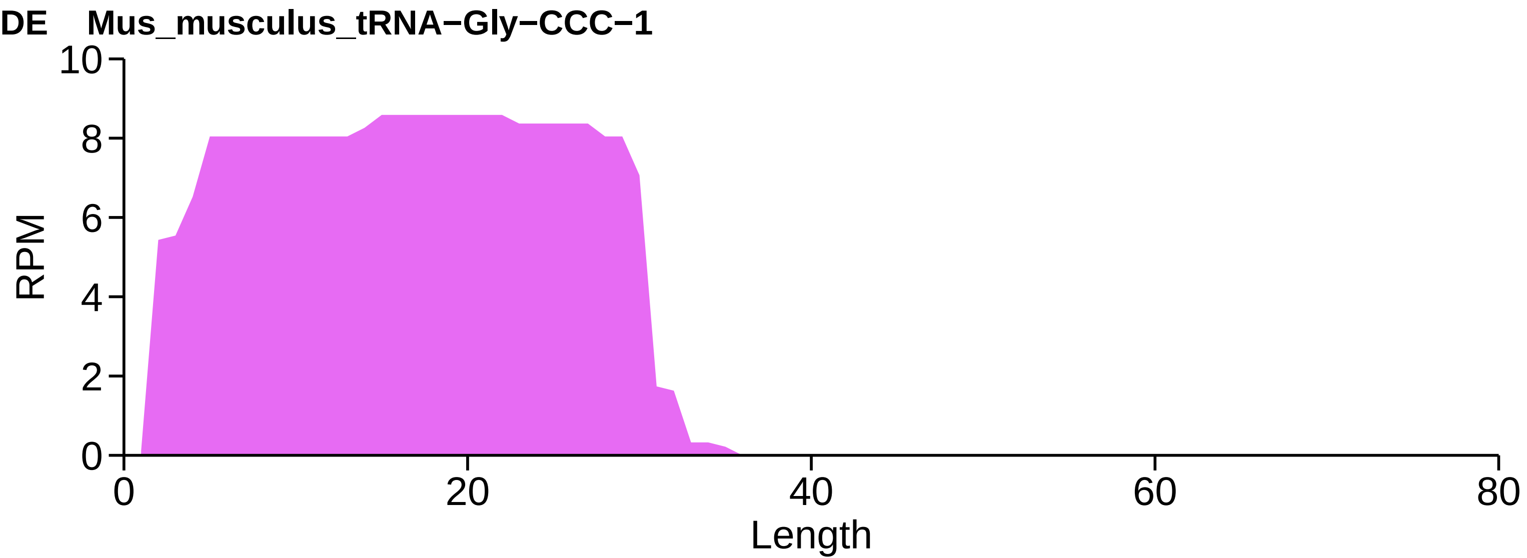
DE Mus_musculus_tRNA−Gly−CCC−1
10
8
6
4
2
0
RPM
0
20
40
60
80
Length

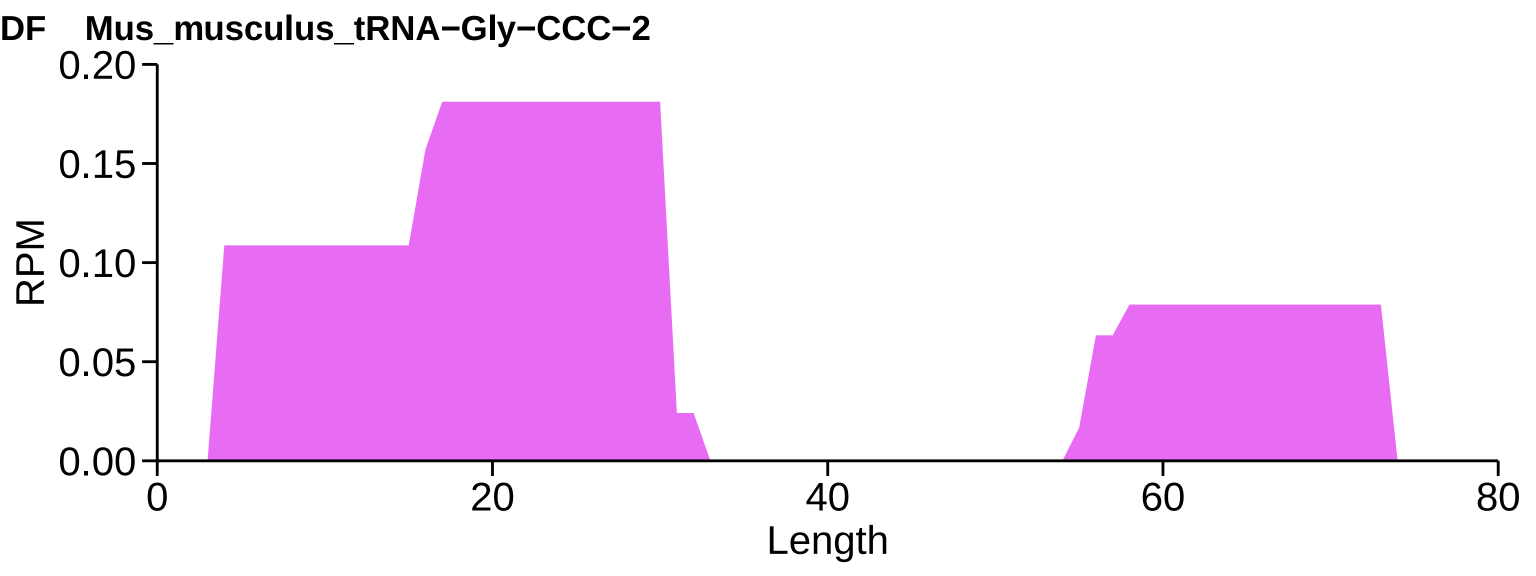
DF Mus_musculus_tRNA−Gly−CCC−2
0.20
0.15
0.10
0.05
0.00
RPM
0
20
40
60
80
Length

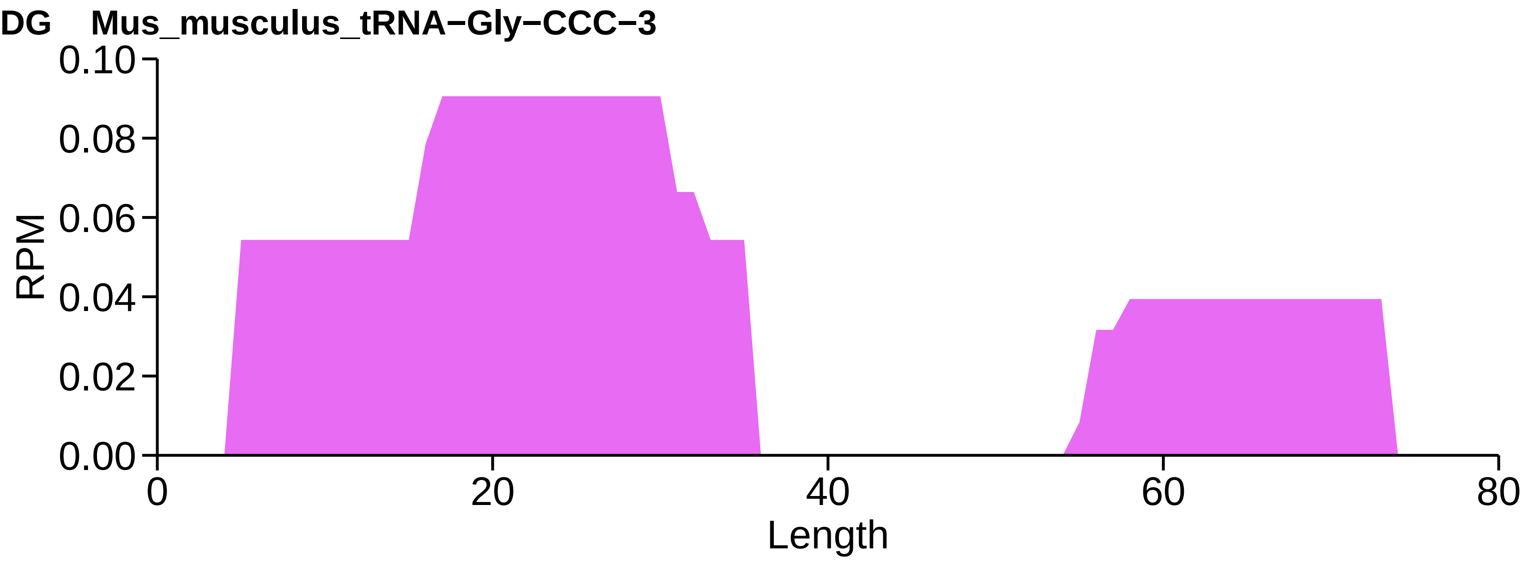
DG Mus_musculus_tRNA−Gly−CCC−3
RPM
0.10
0.08
0.06
0.04
0.02
0.00
0
20
40
60
80
Length

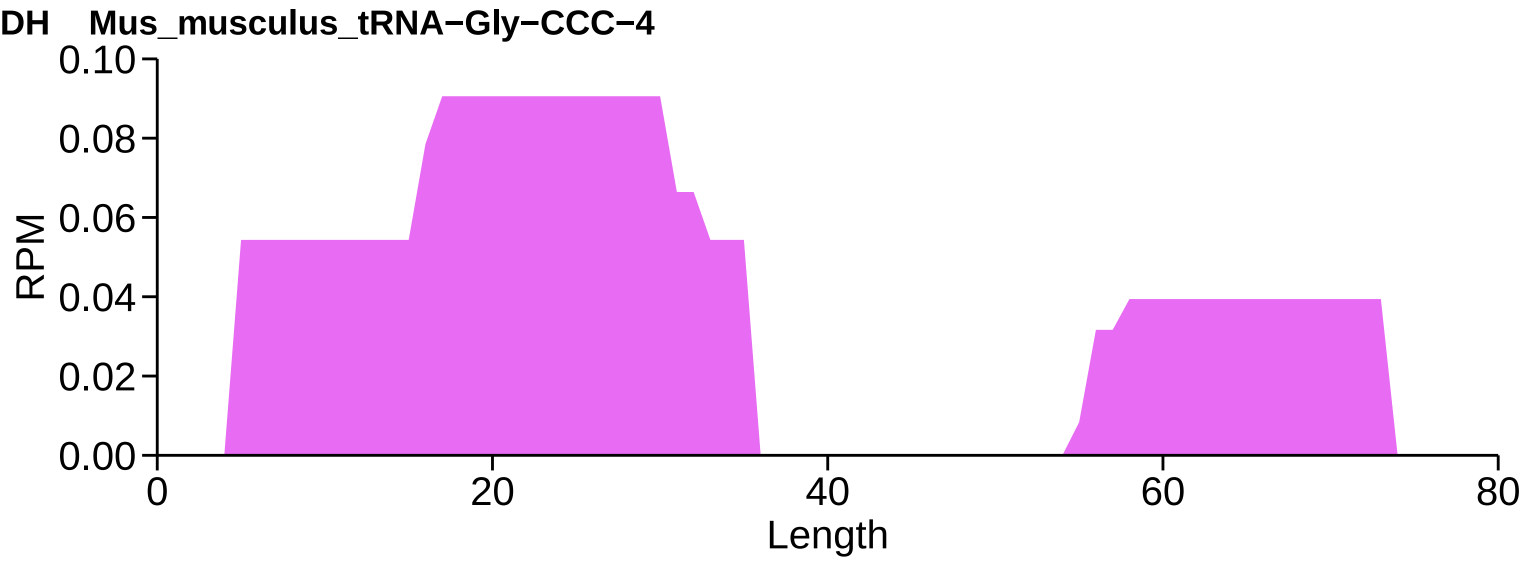
DH Mus_musculus_tRNA−Gly−CCC−4
RPM
0.10
0.08
0.06
0.04
0.02
0.00
0
20
40
60
80
Length

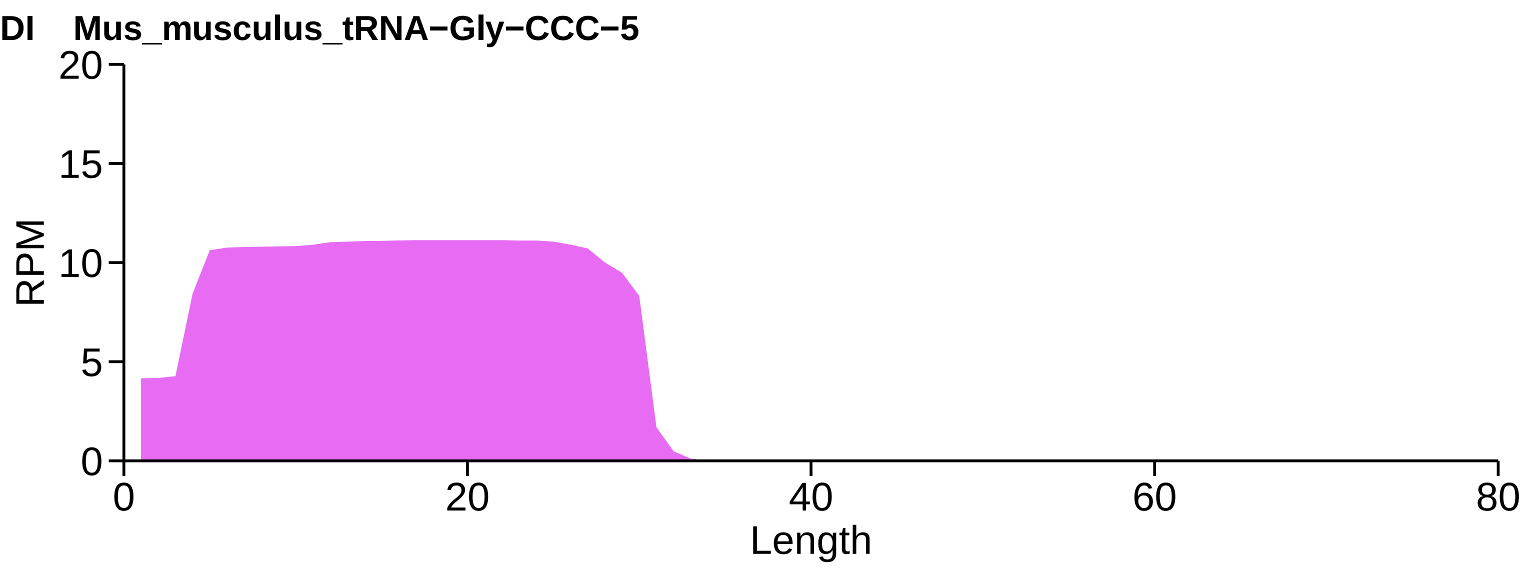
DI Mus_musculus_tRNA−Gly−CCC−5
RPM
Length
0
5
10
15
20
0
20
40
60
80

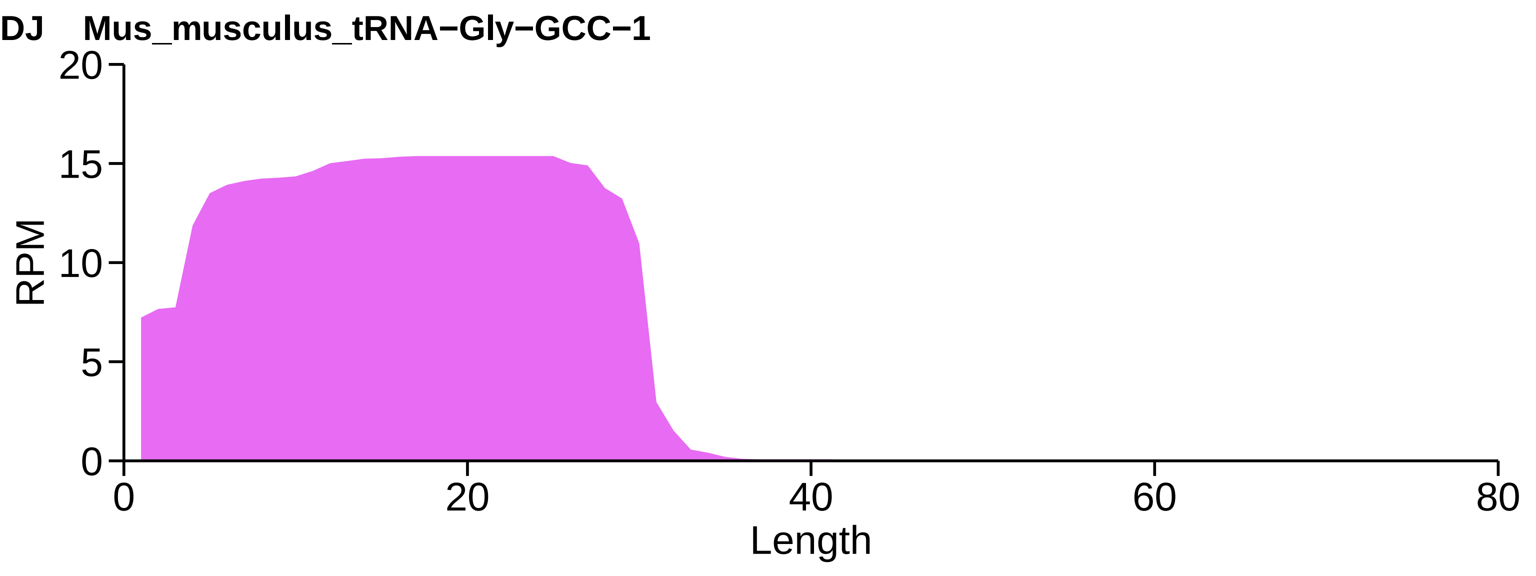
DJ Mus_musculus_tRNA−Gly−GCC−1
RPM
20
15
10
5
0
0
20
40
60
80
Length

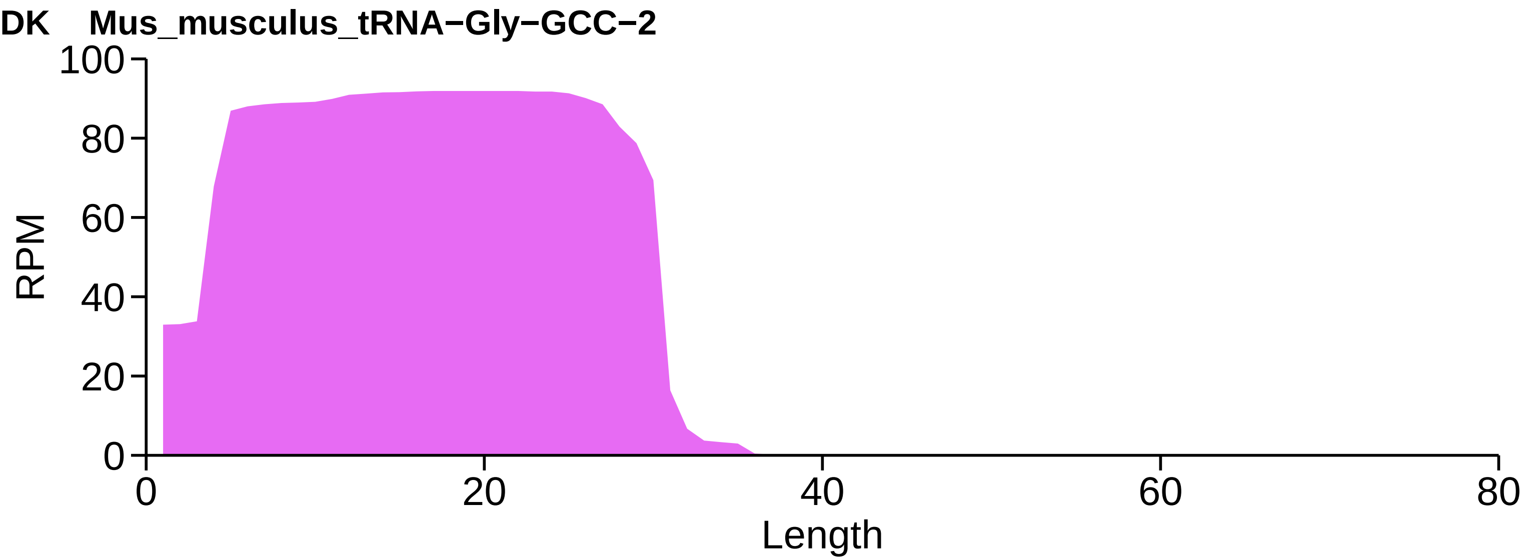

DK Mus_musculus_tRNA−Gly−GCC−2
RPM
0
20
40
60
80
100
Length
0
20
40
60
80

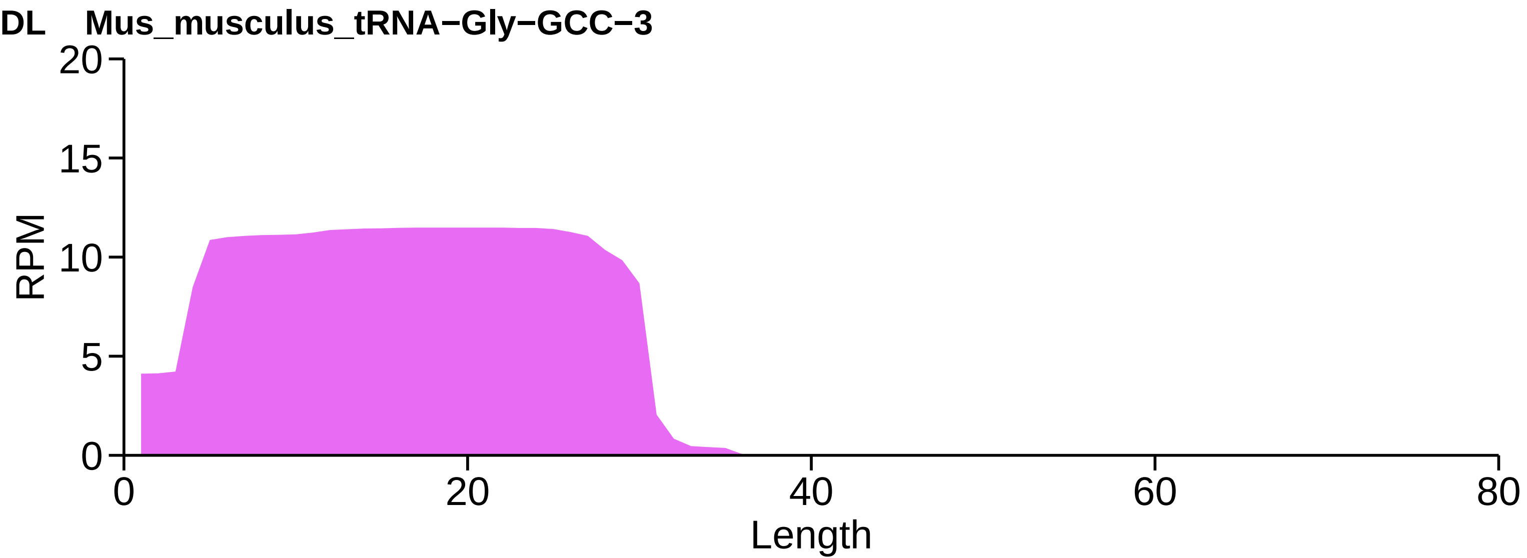

DL Mus_musculus_tRNA−Gly−GCC−3
RPM
0
5
10
15
20
0
20
40
60
80
Length

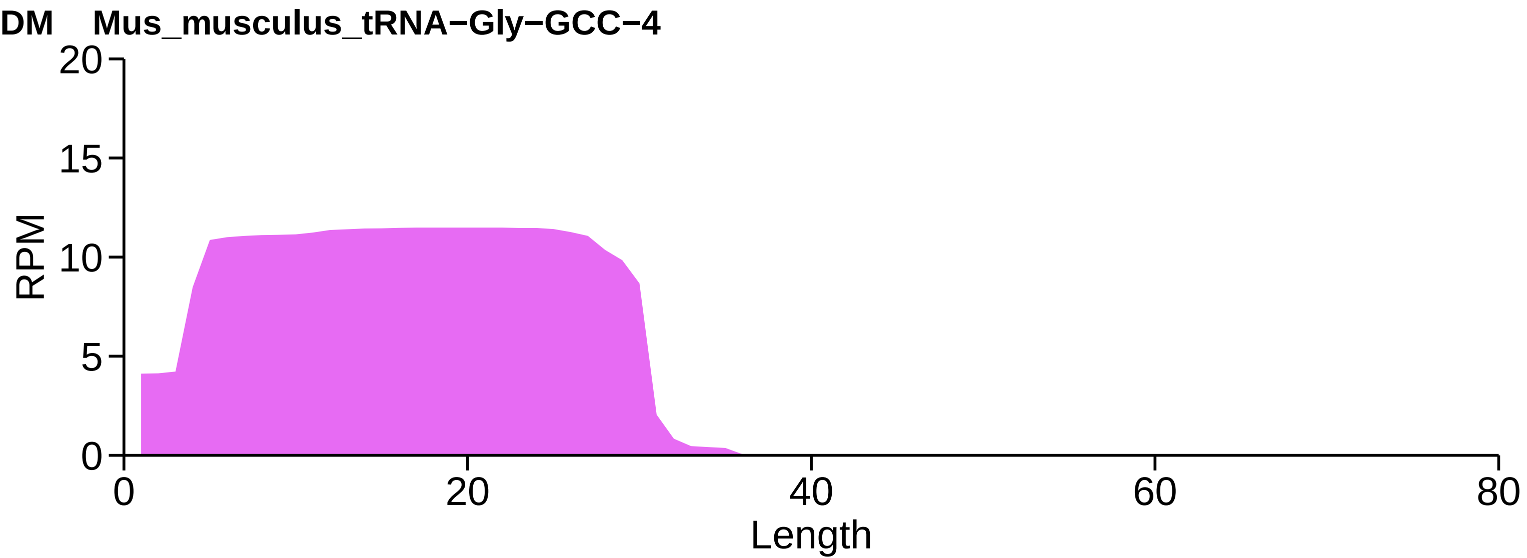
DM Mus_musculus_tRNA−Gly−GCC−4
RPM
Length
0
5
10
15
20
0
20
40
60
80

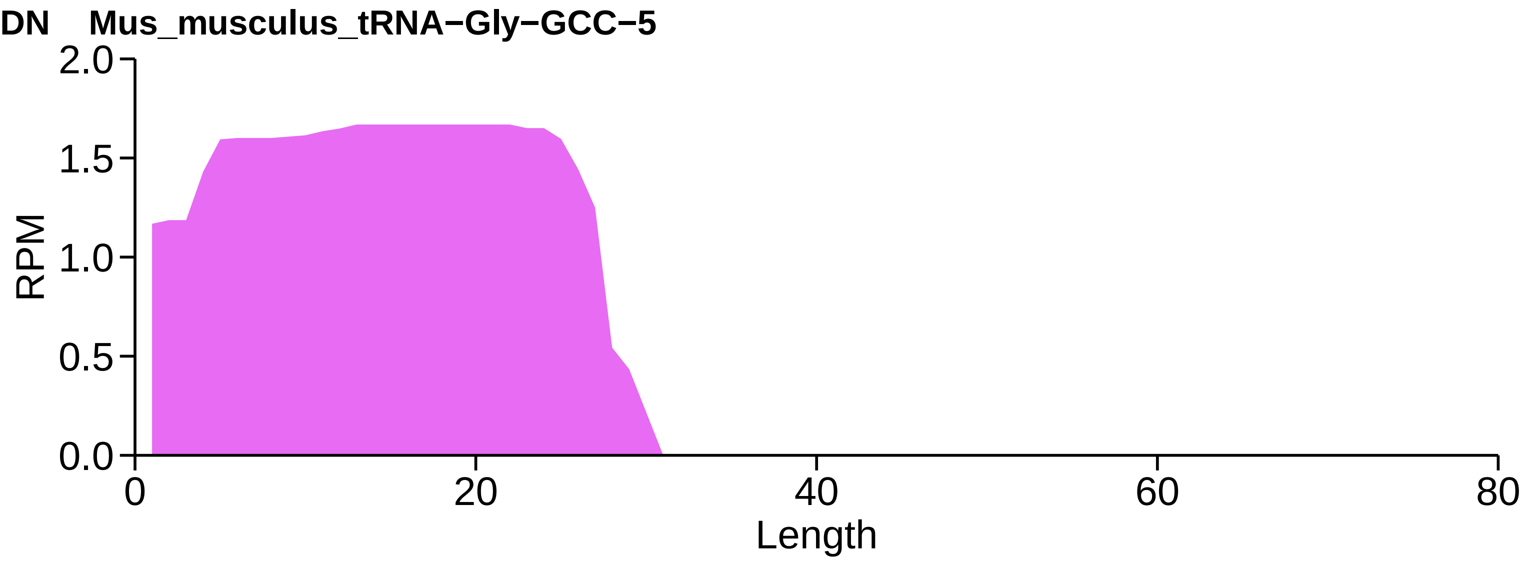
DN Mus_musculus_tRNA−Gly−GCC−5
RPM
2.0
1.5
1.0
0.5
0.0
0
20
40
60
80
Length

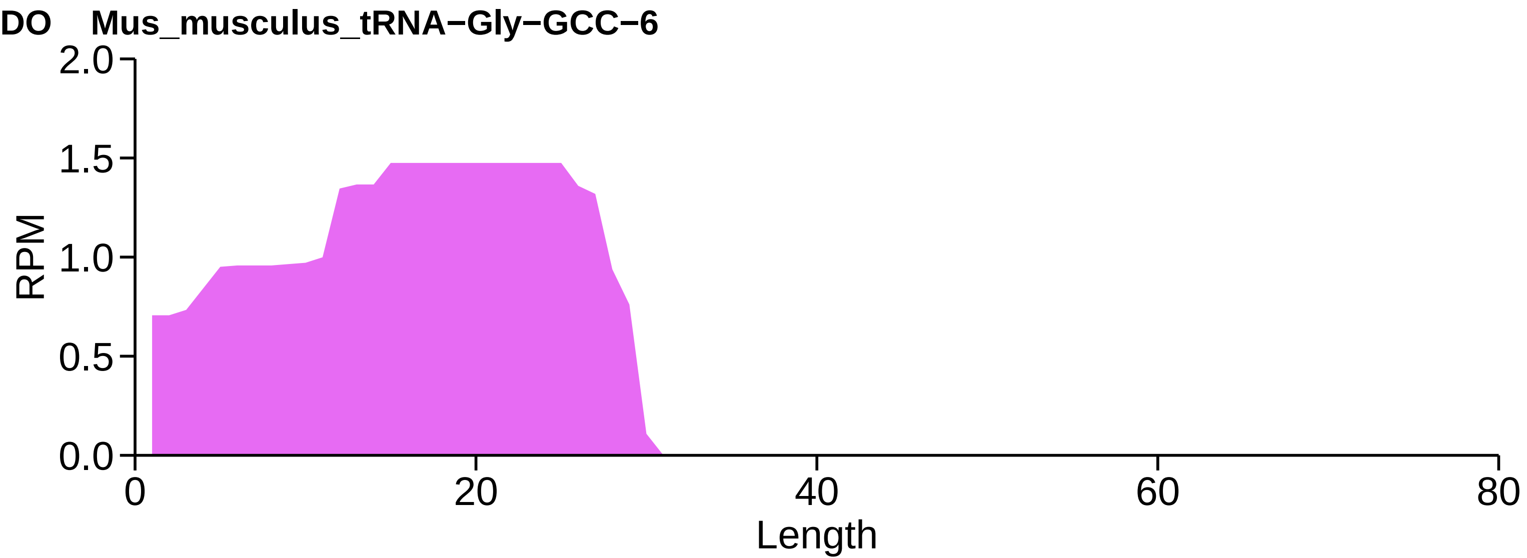
DO Mus_musculus_tRNA−Gly−GCC−6
RPM
2.0
1.5
1.0
0.5
0.0
0
20
40
60
80
Length

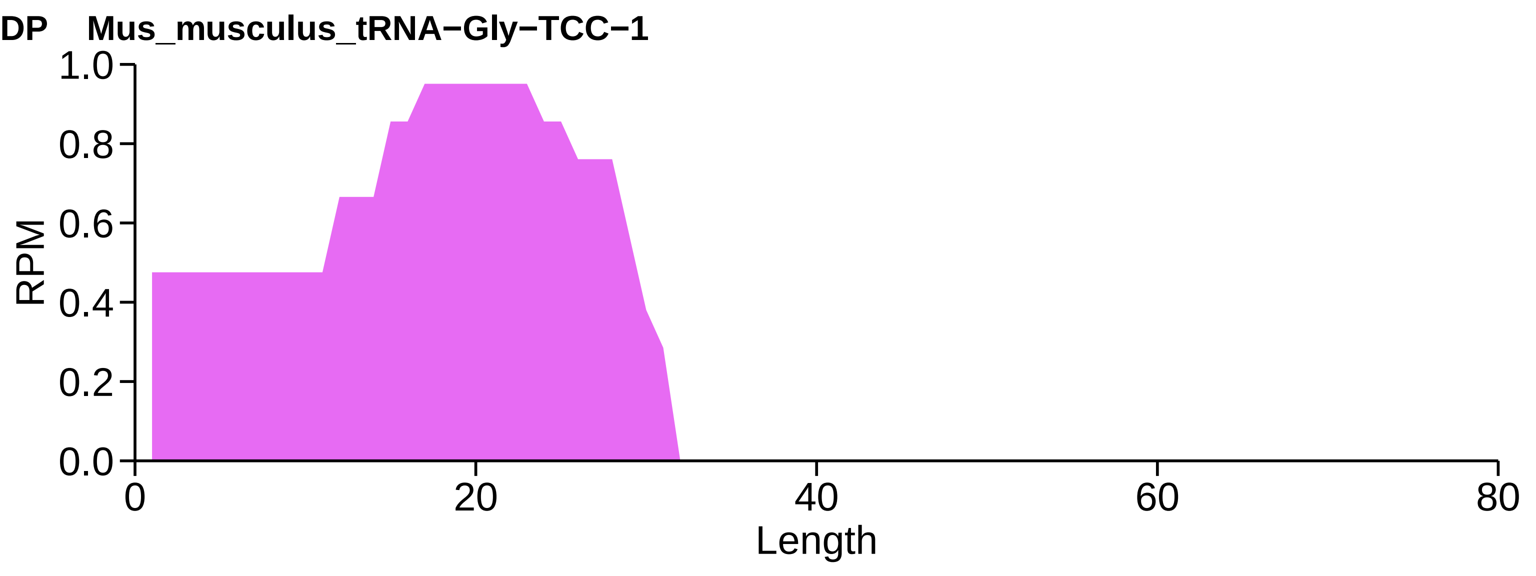
DP Mus_musculus_tRNA−Gly−TCC−1
RPM
1.0
0.8
0.6
0.4
0.2
0.0
0
20
40
60
80
Length

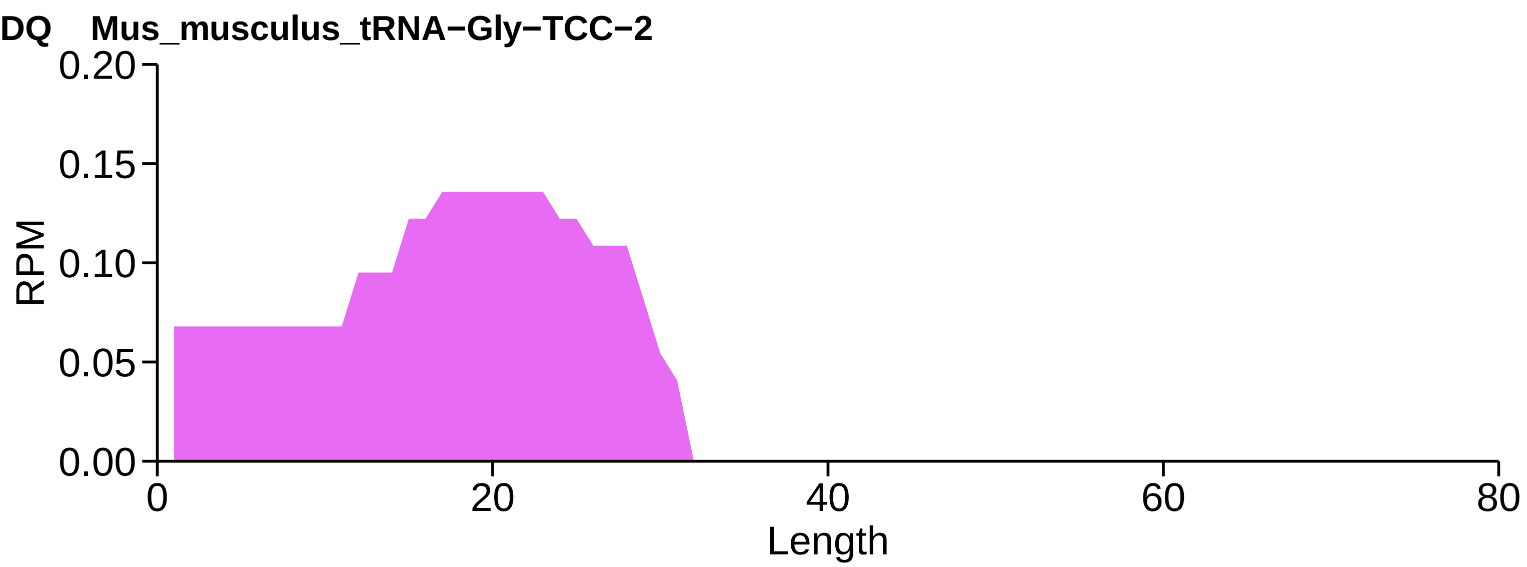
DQ Mus_musculus_tRNA−Gly−TCC−2
RPM
0.20
0.15
0.10
0.05
0.00
0
20
40
60
80
Length

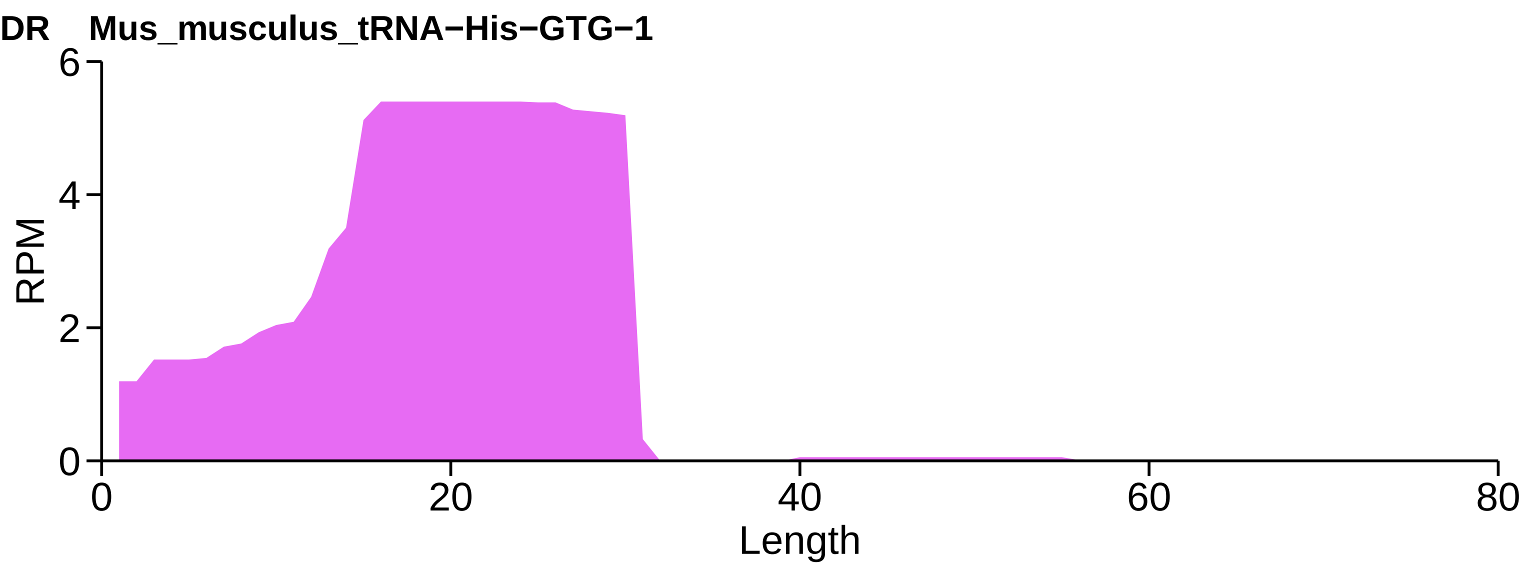
DR Mus_musculus_tRNA−His−GTG−1
RPM
0
2
4
6
0
20
40
60
80
Length

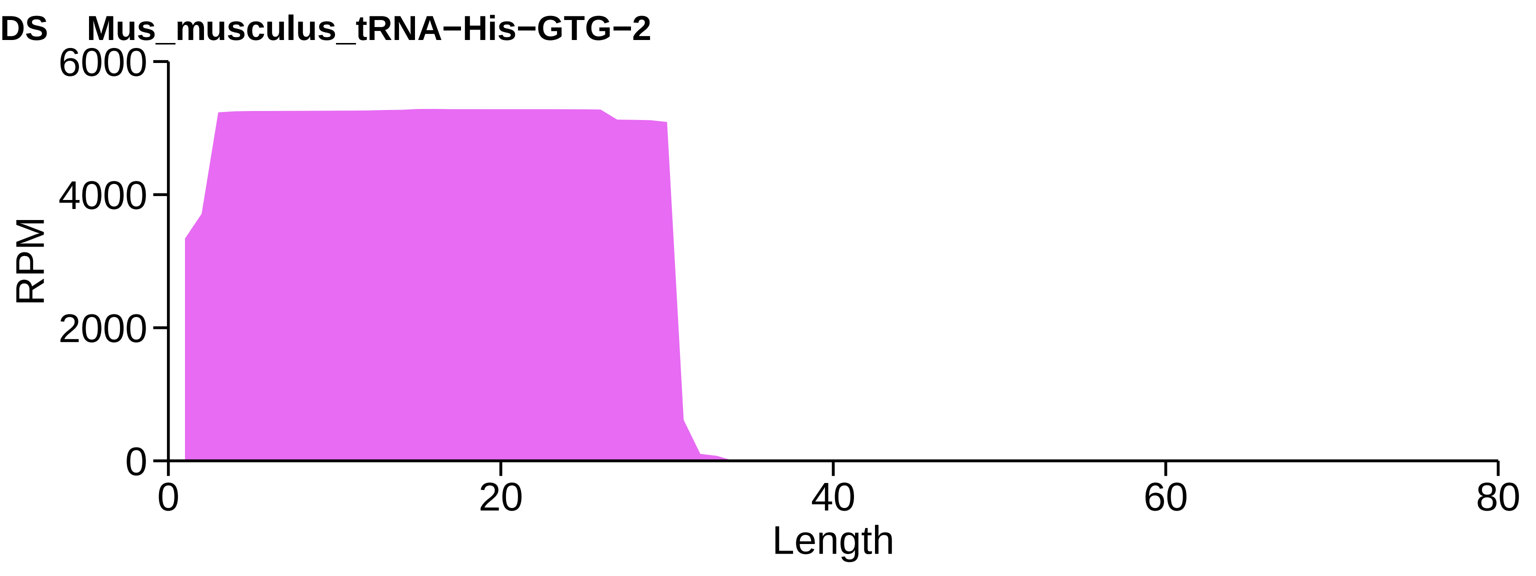
DS Mus_musculus_tRNA−His−GTG−2
6000
4000
2000
0
RPM
0
20
40
60
80
Length

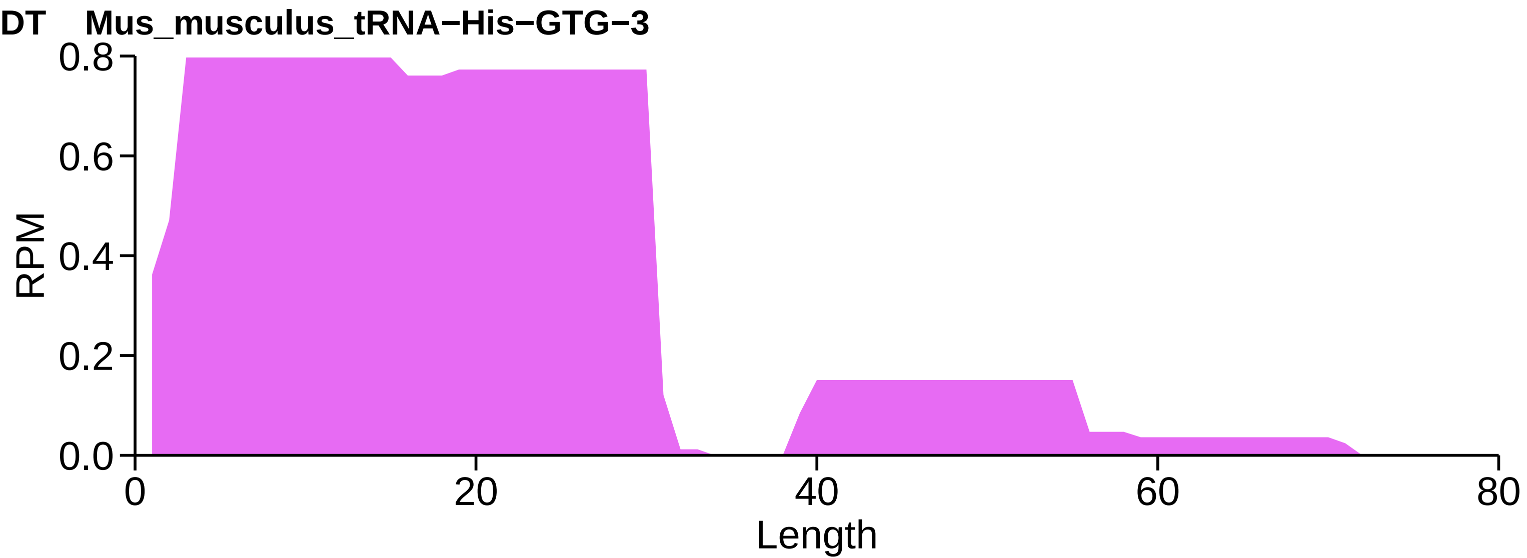
DT Mus_musculus_tRNA−His−GTG−3
RPM
0.0
0.2
0.4
0.6
0.8
0
20
40
60
80
Length

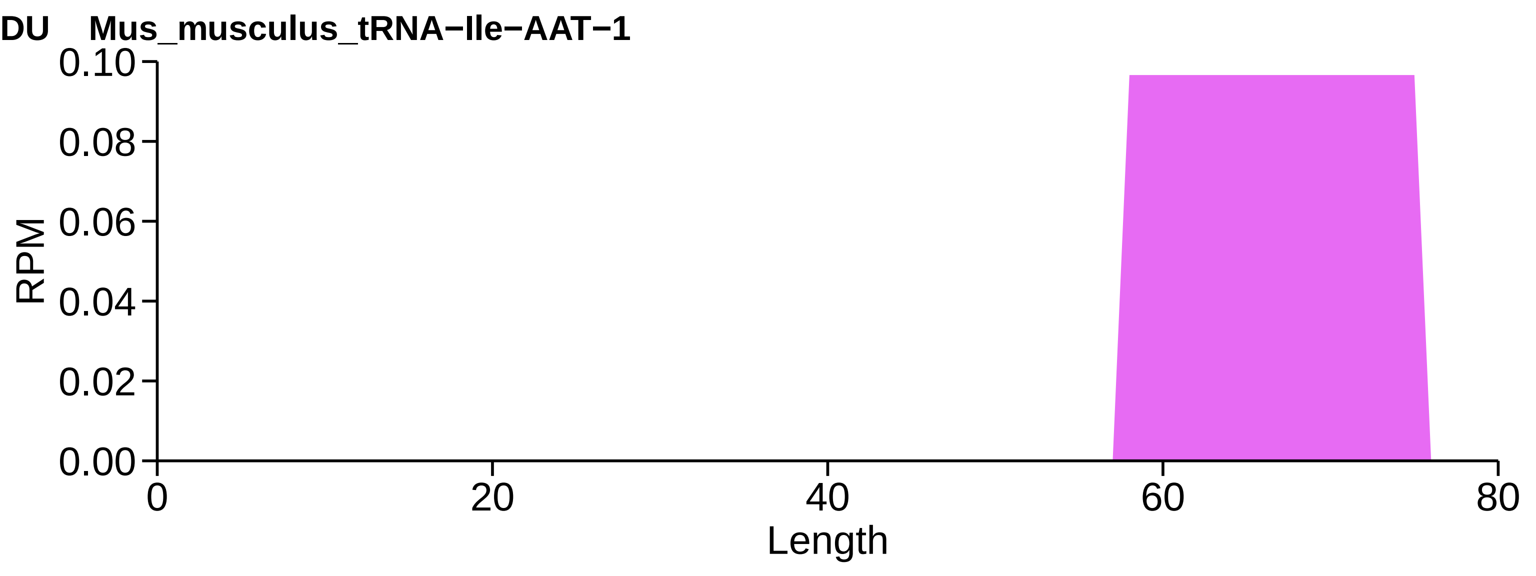
DU Mus_musculus_tRNA−Ile−AAT−1
RPM
0.10
0.08
0.06
0.04
0.02
0.00
0
20
40
60
80
Length

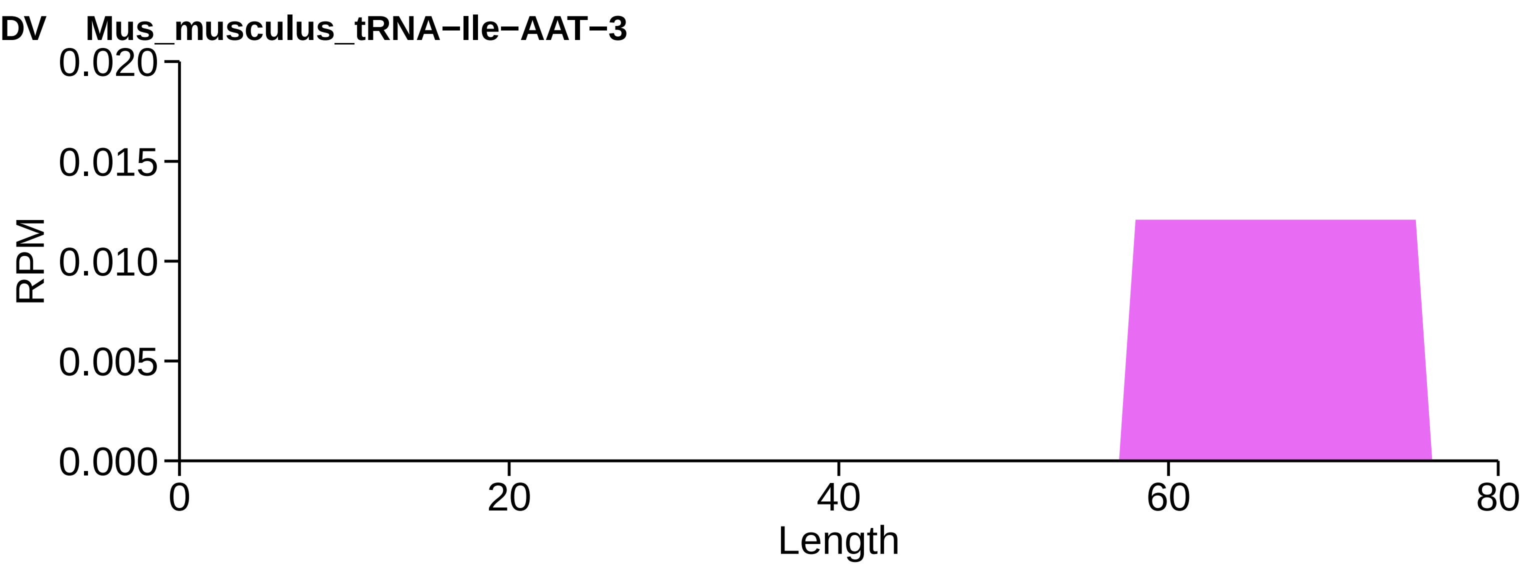
DV
Mus_musculus_tRNA−Ile−AAT−3
RPM
0.020
0.015
0.010
0.005
0.000
0
20
40
60
80
Length

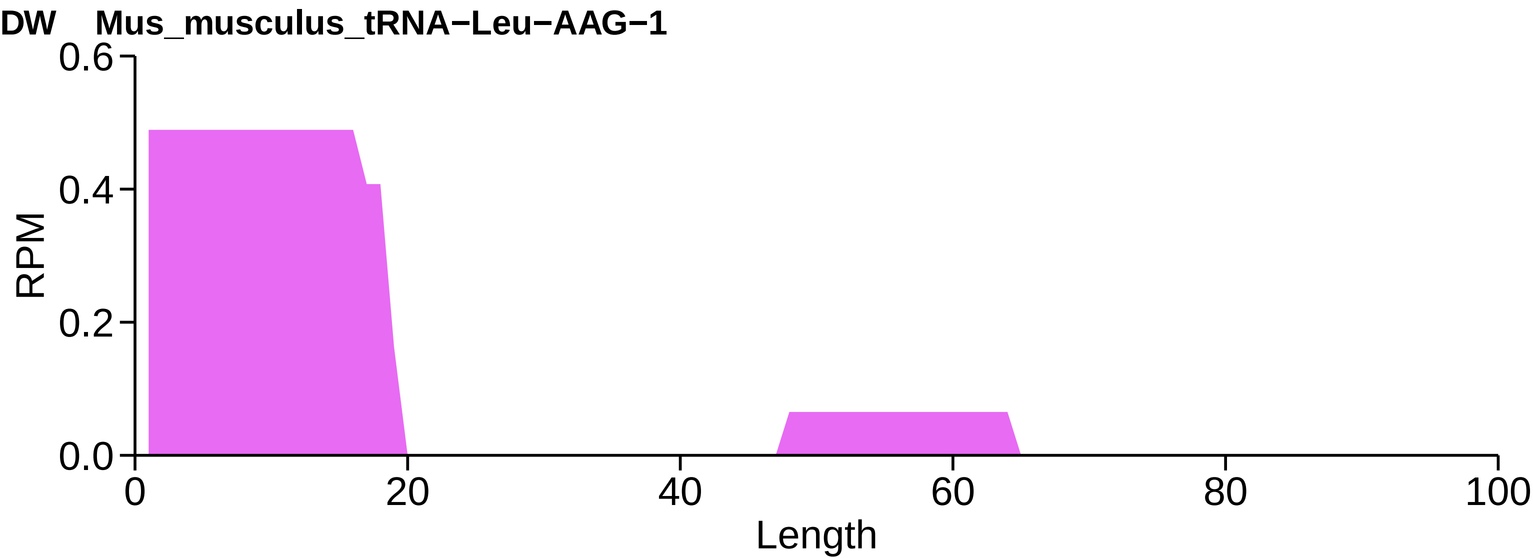
DW Mus_musculus_tRNA−Leu−AAG−1
0.6
0.4
0.2
0.0
RPM
0
20
40
60
80
100
Length

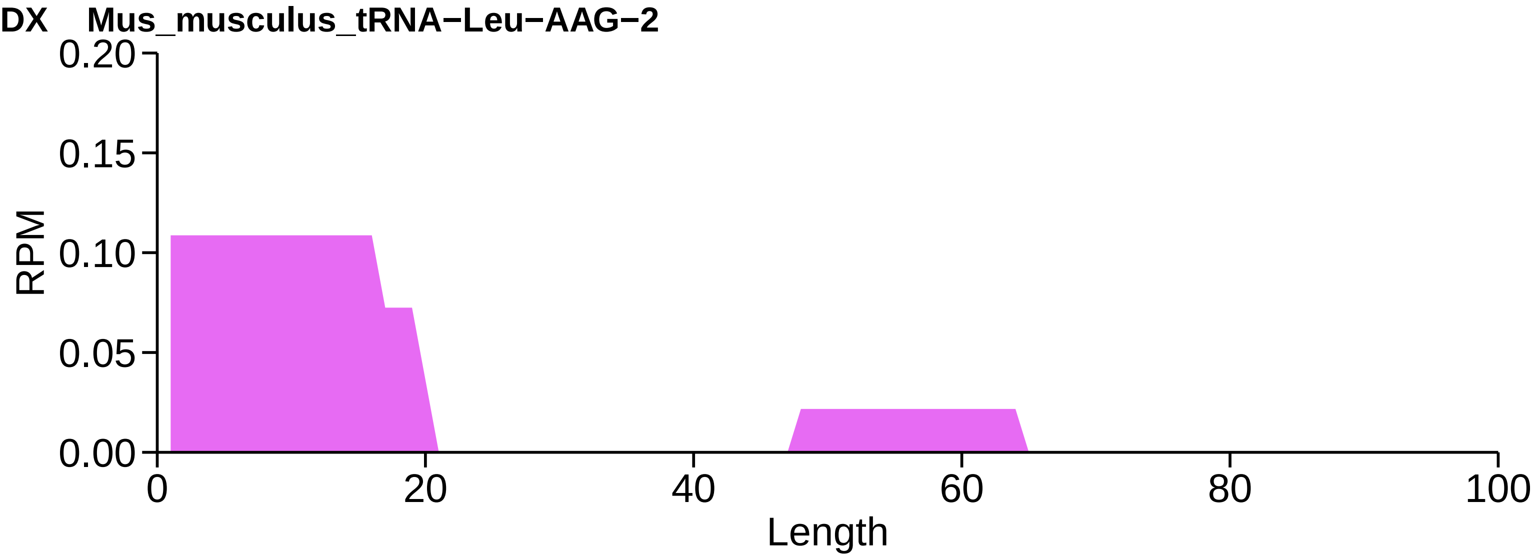
DX Mus_musculus_tRNA−Leu−AAG−2
RPM
0.20
0.15
0.10
0.05
0.00
0
20
40
60
80
100
Length

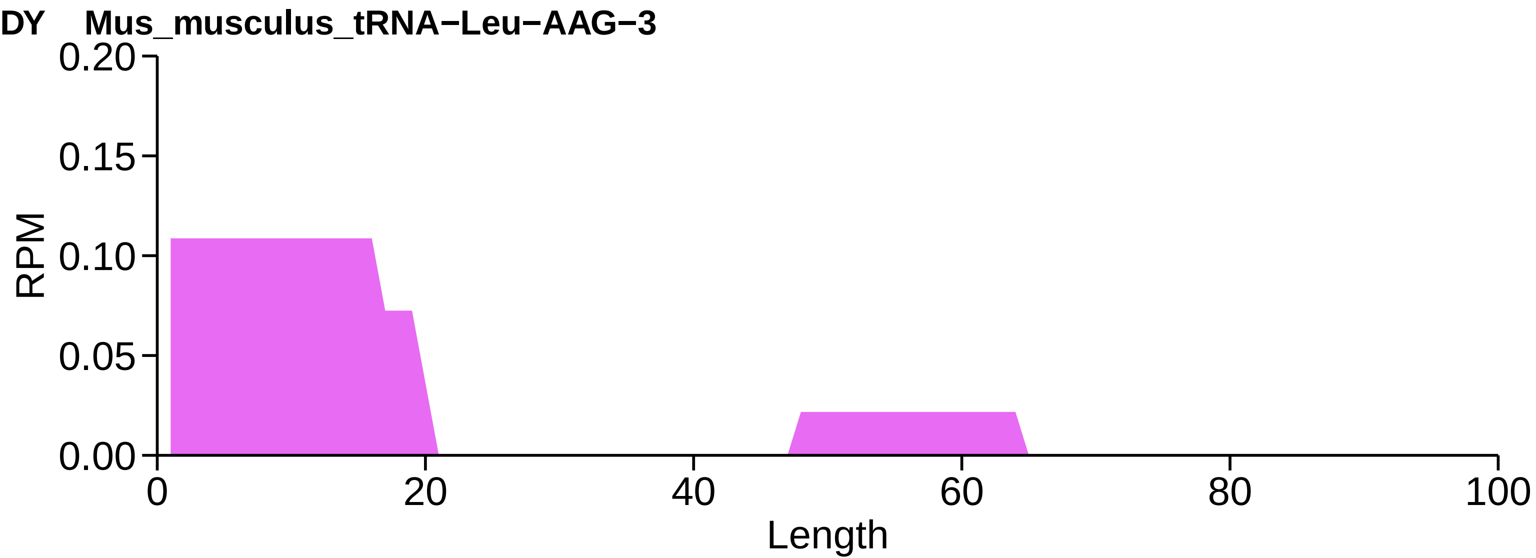
DY Mus_musculus_tRNA−Leu−AAG−3
RPM
0.20
0.15
0.10
0.05
0.00
0
20
40
60
80
100
Length

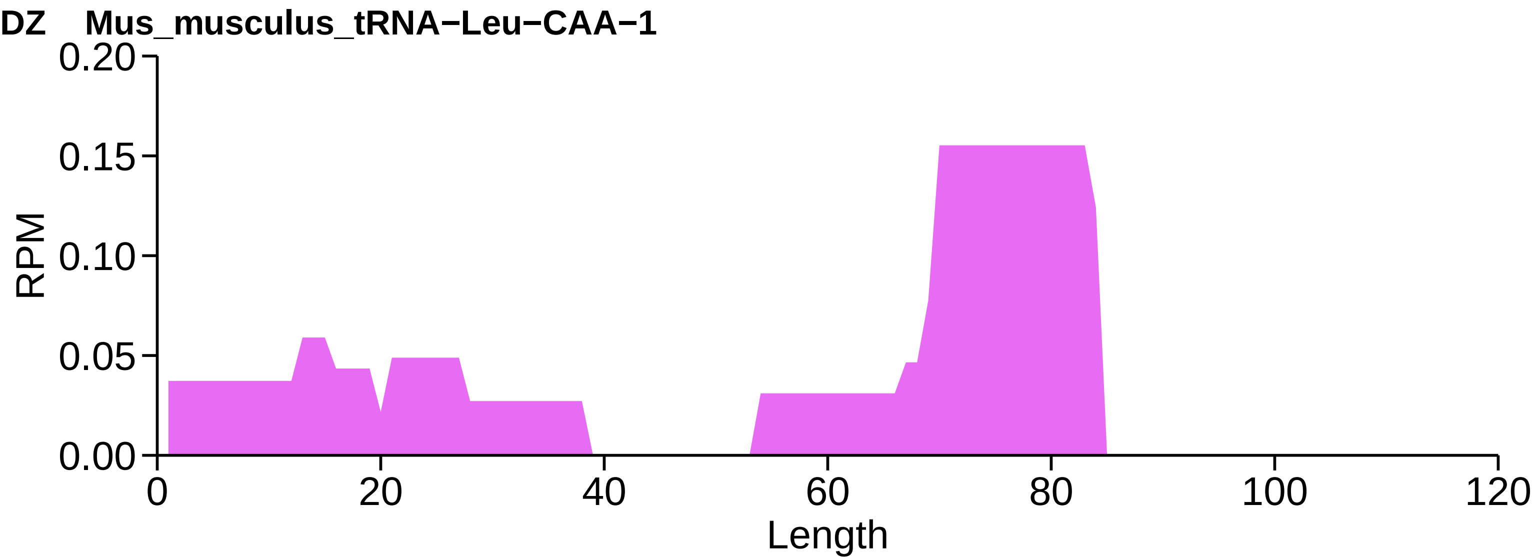
DZ Mus_musculus_tRNA−Leu−CAA−1
RPM
0.20
0.15
0.10
0.05
0.00
0
20
40
60
80
100
120
Length

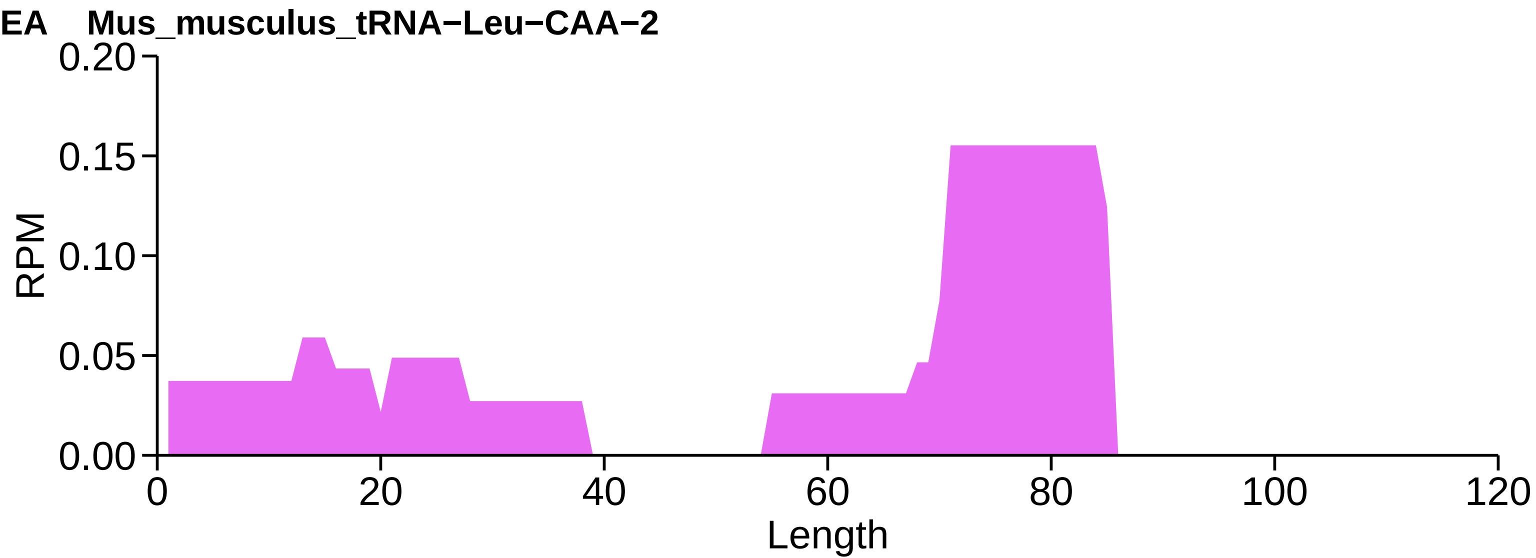
EA Mus_musculus_tRNA−Leu−CAA−2
0.20
0.15
0.10
0.05
0.00
RPM
0
20
40
60
80
100
120
Length

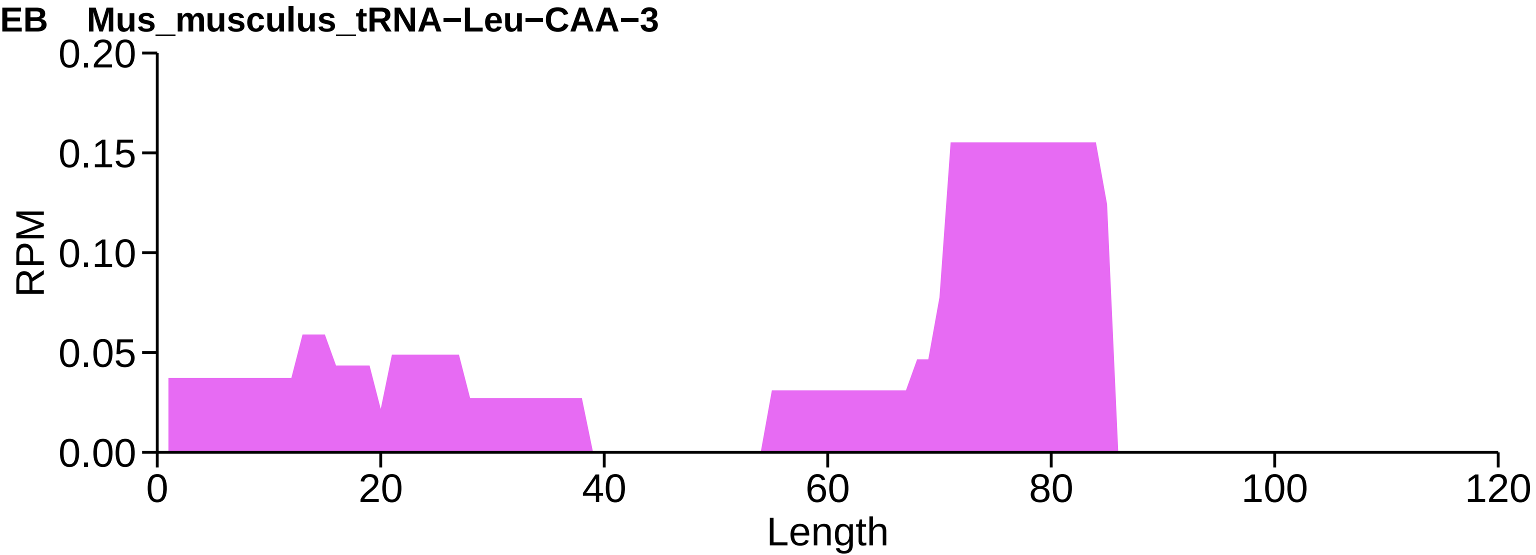

EB Mus_musculus_tRNA−Leu−CAA−3
RPM
0.00
0.05
0.10
0.15
0.20
0
20
40
60
80
100
120
Length

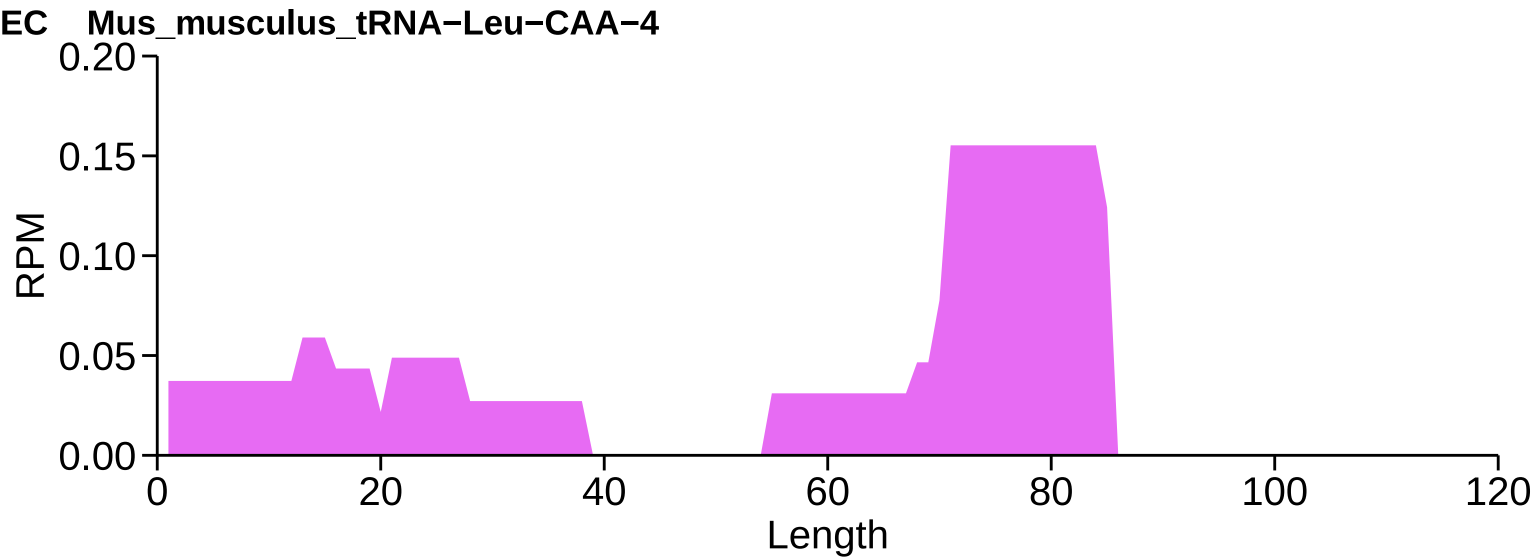
EC Mus_musculus_tRNA−Leu−CAA−4
0.20
0.15
0.10
0.05
0.00
RPM
0
20
40
60
80
100
120
Length

ED Mus_musculus_tRNA−Leu−CAG−1

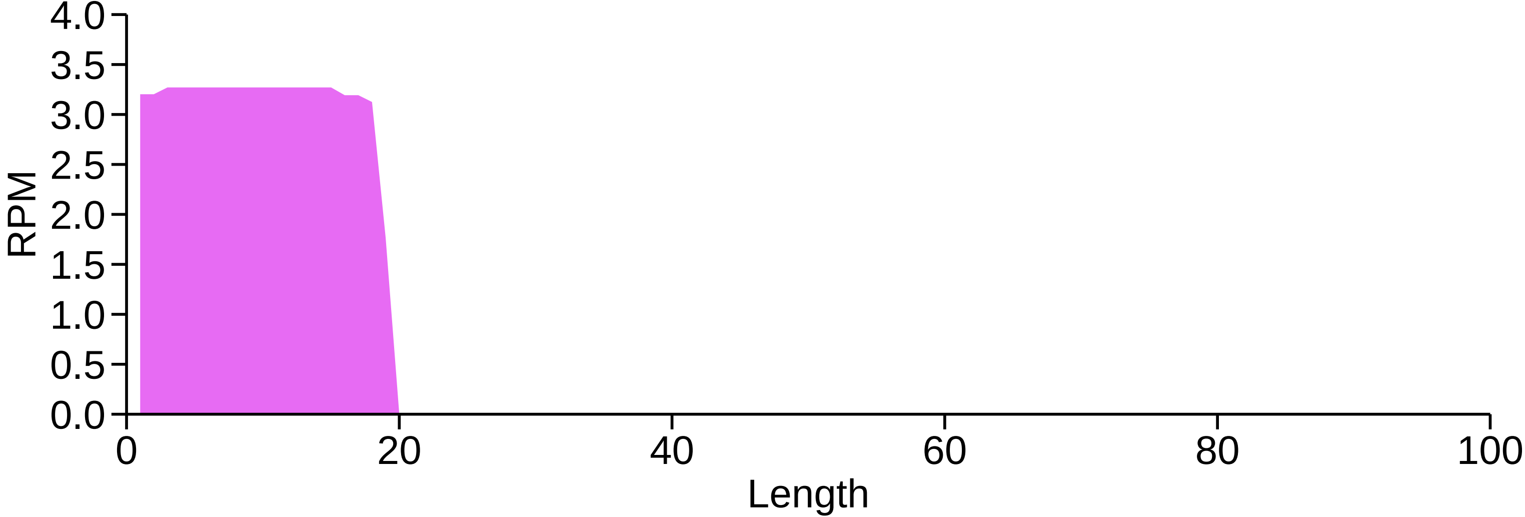

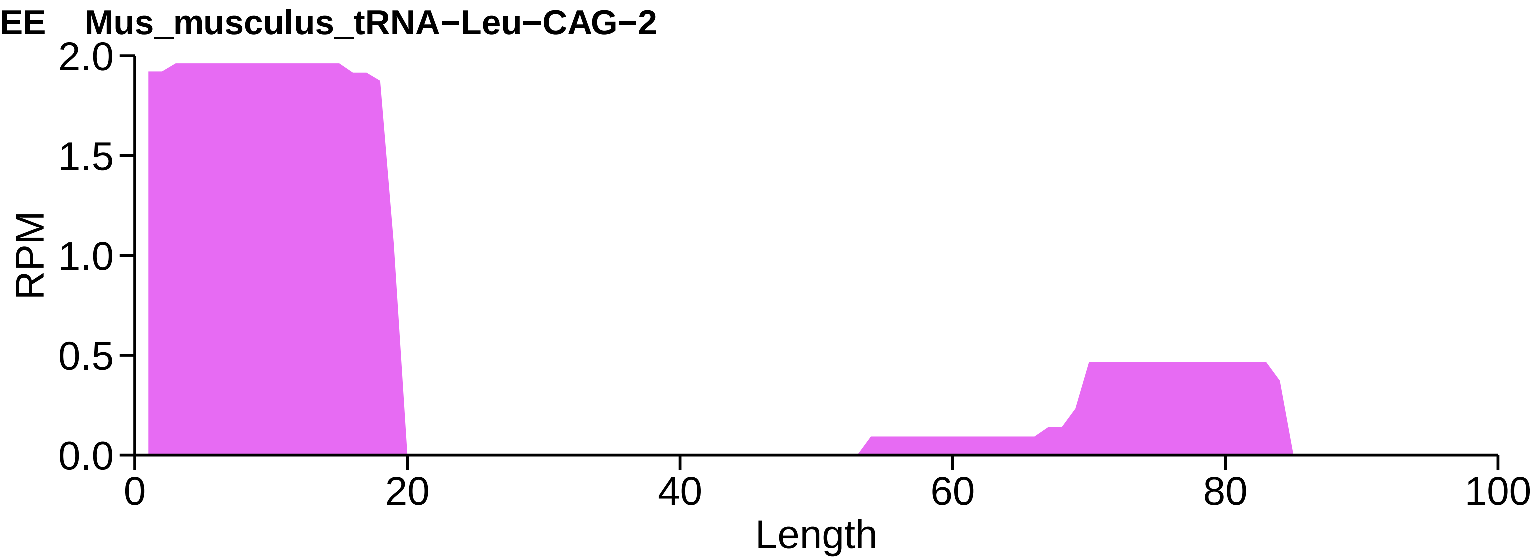
EE Mus_musculus_tRNA−Leu−CAG−2
RPM
2.0
1.5
1.0
0.5
0.0
0
20
40
60
80
100
Length

EF Mus_musculus_tRNA−Leu−CAG−3

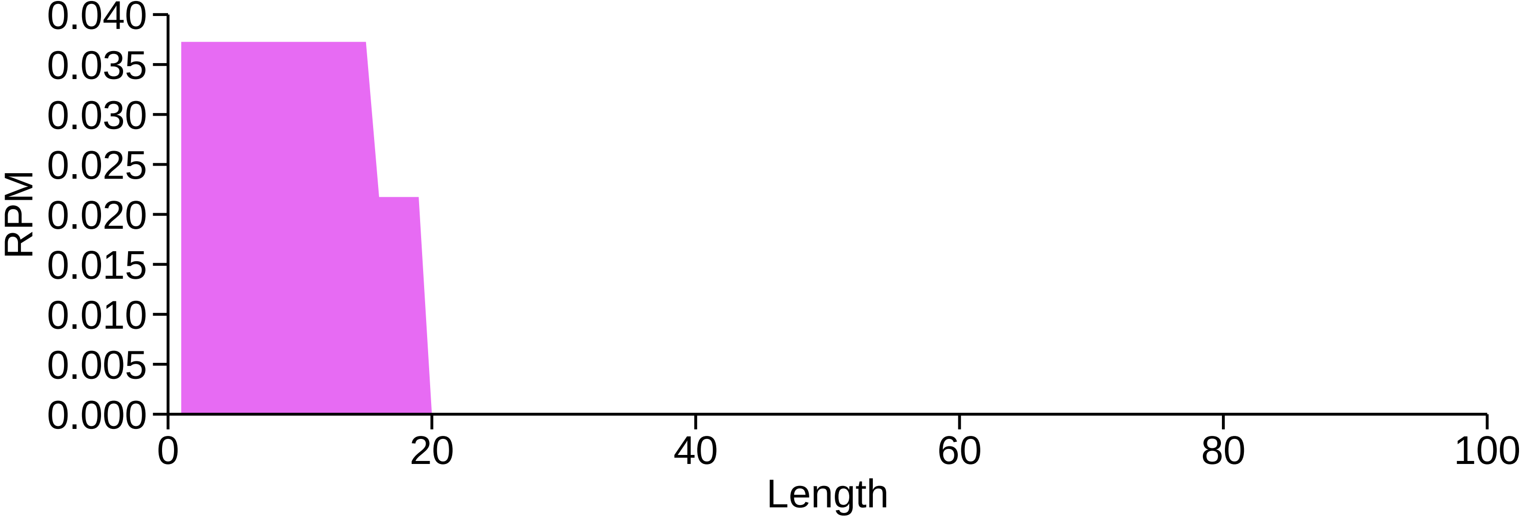

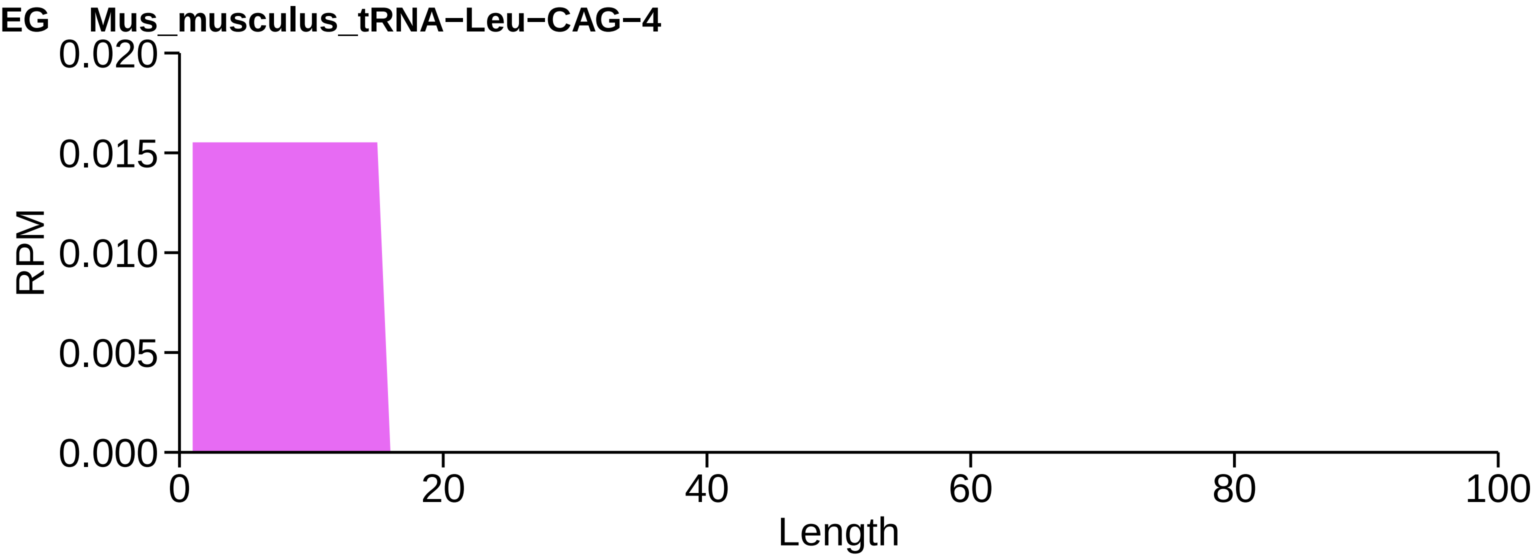
EG Mus_musculus_tRNA−Leu−CAG−4
RPM
0.020
0.015
0.010
0.005
0.000
0
20
40
60
80
100
Length

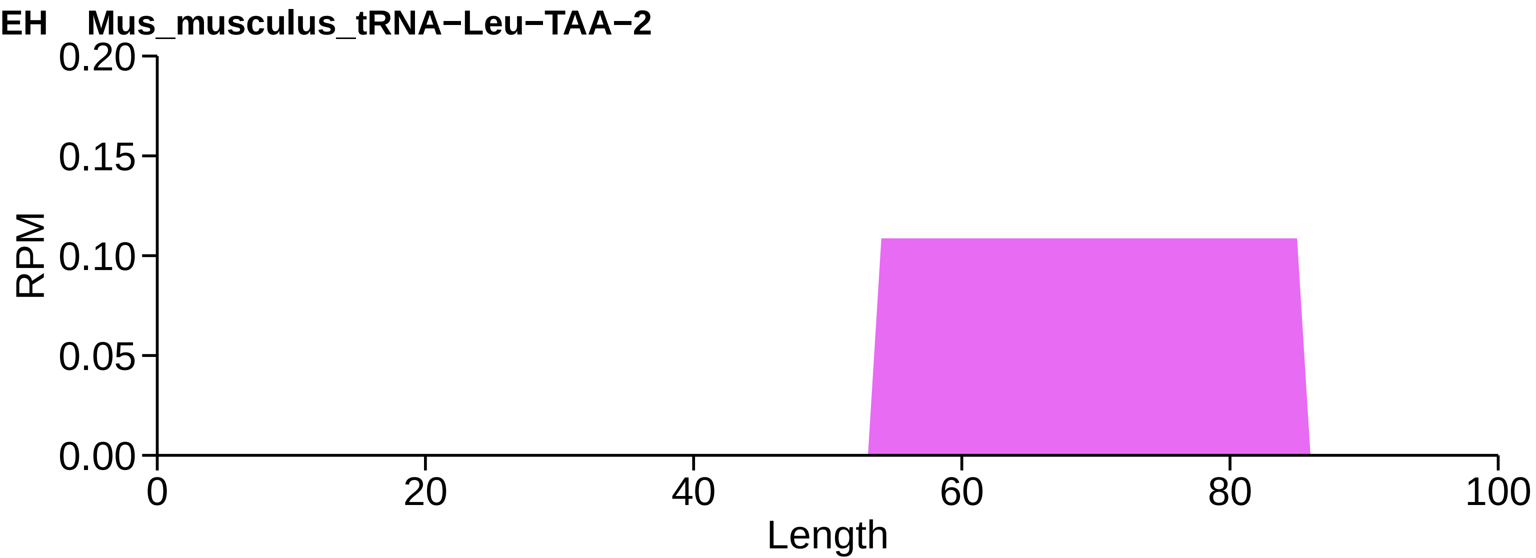
EH Mus_musculus_tRNA−Leu−TAA−2
RPM
0.20
0.15
0.10
0.05
0.00
0
20
40
60
80
100
Length

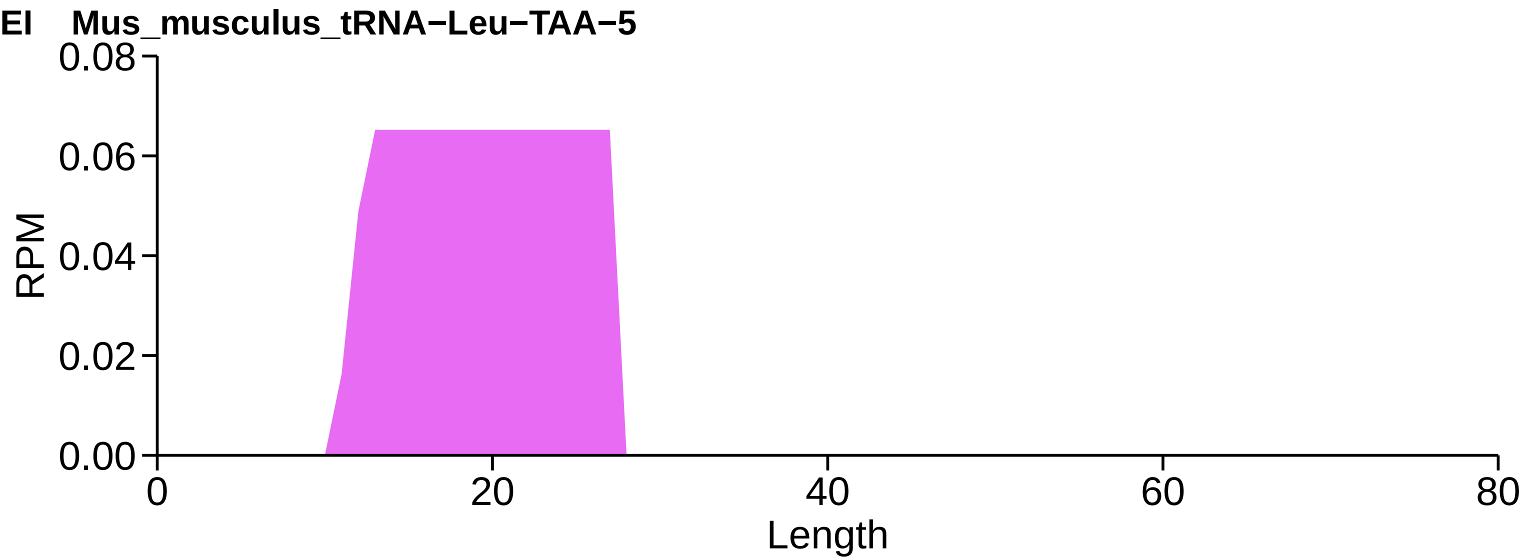

EI Mus_musculus_tRNA−Leu−TAA−5
0.08
0.06
0.04
0.02
0.00
RPM
0
20
40
60
80
Length

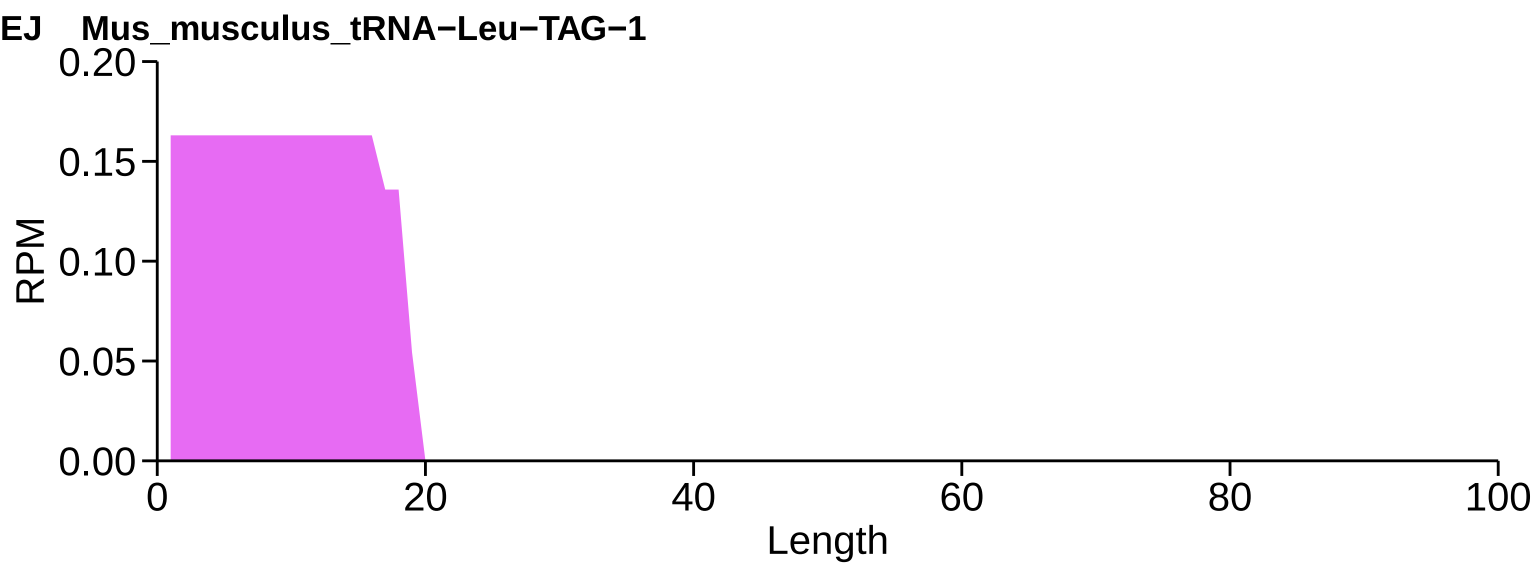
EJ Mus_musculus_tRNA−Leu−TAG−1
0.20
0.15
0.10
0.05
0.00
RPM
0
20
40
60
80
100
Length

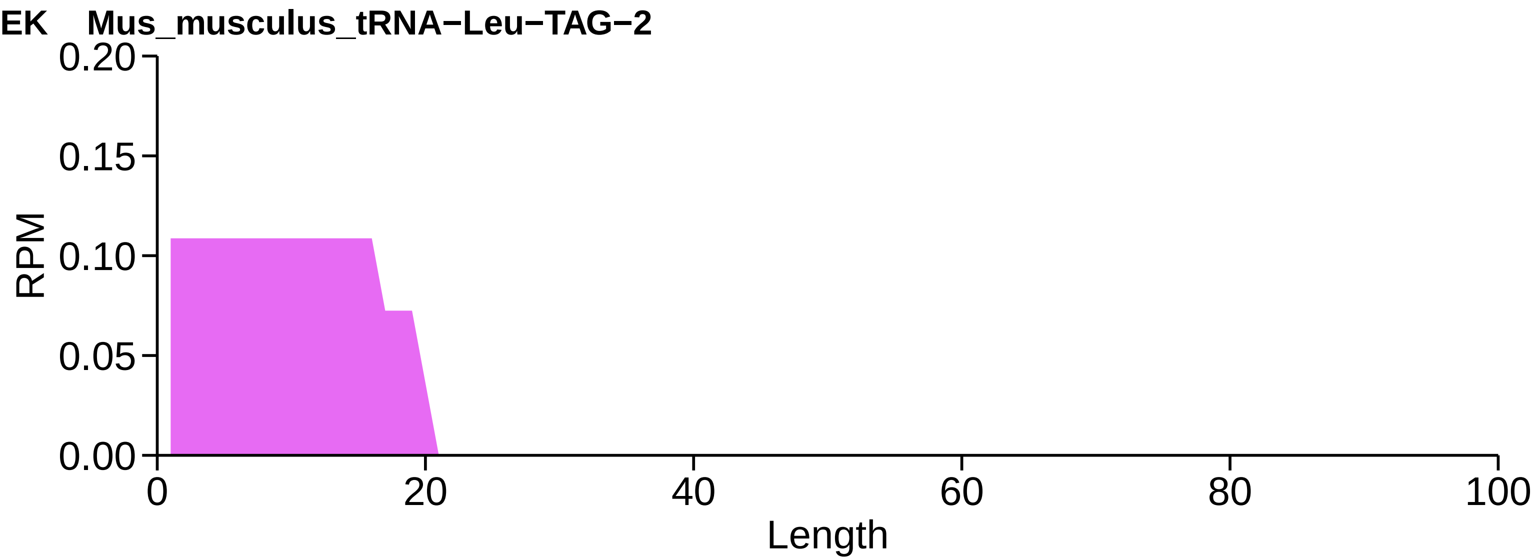
EK Mus_musculus_tRNA−Leu−TAG−2
RPM
0.20
0.15
0.10
0.05
0.00
0
20
40
60
80
100
Length

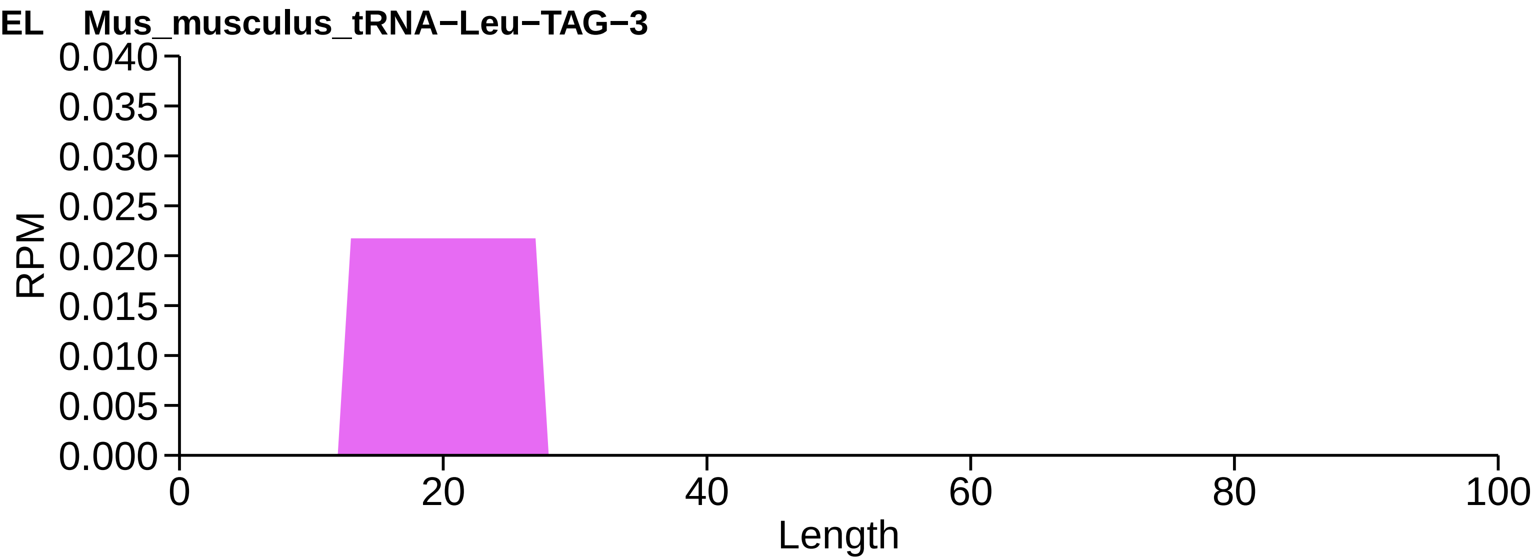
EL Mus_musculus_tRNA−Leu−TAG−3
RPM
0.040
0.035
0.030
0.025
0.020
0.015
0.010
0.005
0.000
0
20
40
60
80
100
Length

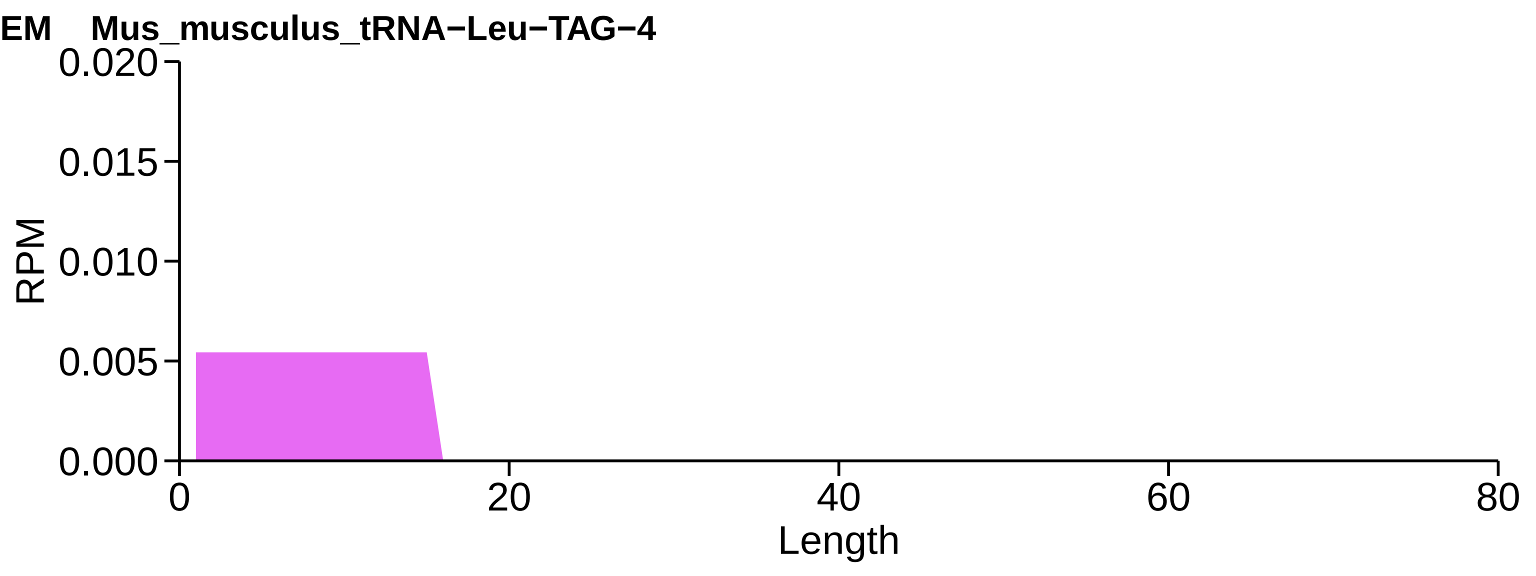
EM Mus_musculus_tRNA−Leu−TAG−4
RPM
0.020
0.015
0.010
0.005
0.000
0
20
40
60
80
Length

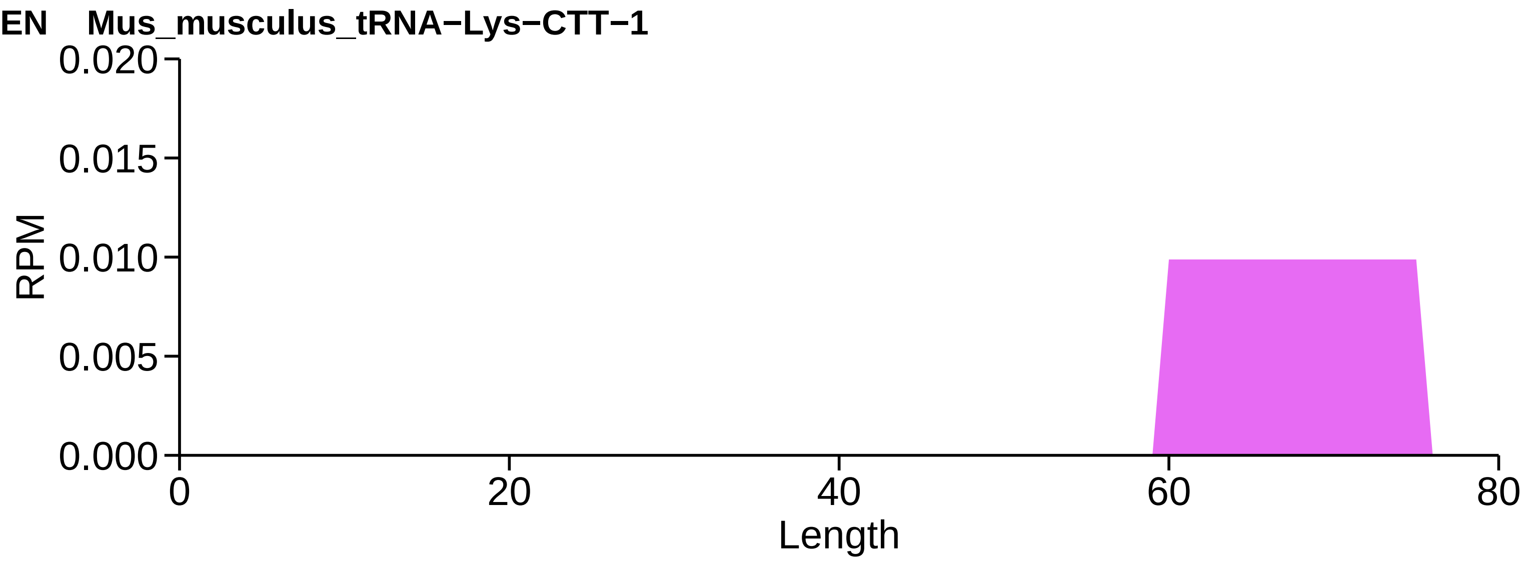
EN Mus_musculus_tRNA−Lys−CTT−1
0.020
0.015
0.010
0.005
0.000
RPM
0
20
40
60
80
Length

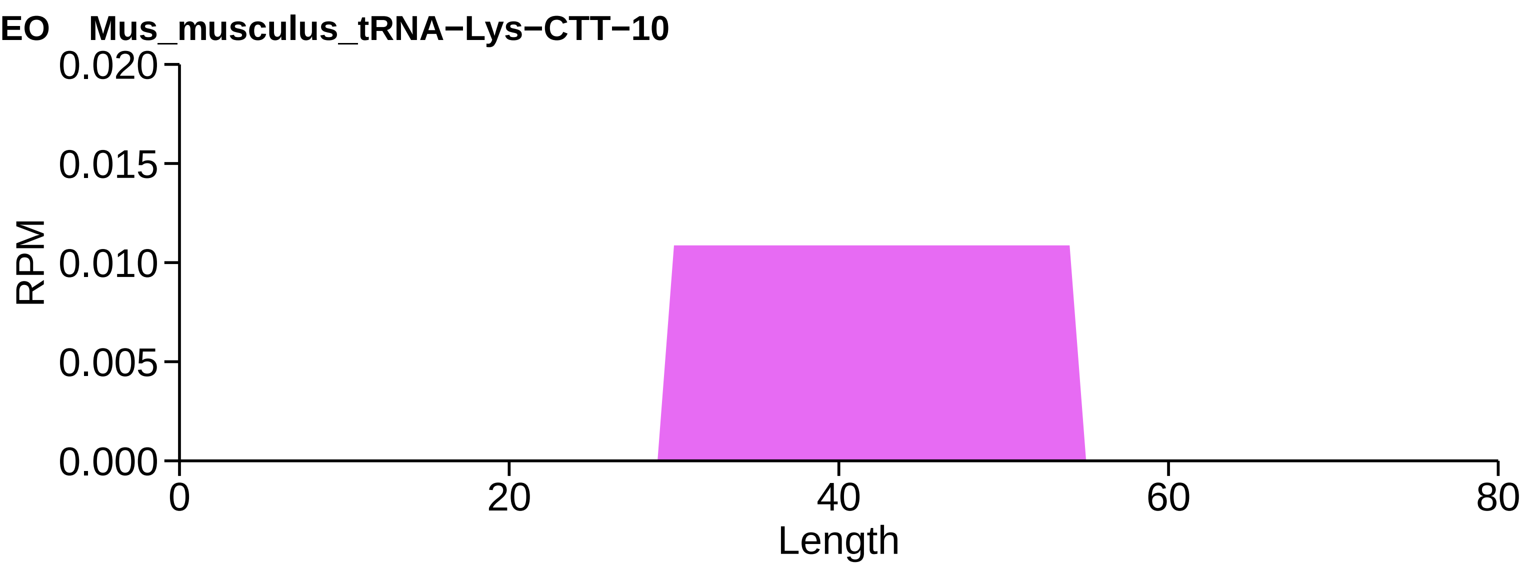
EO Mus_musculus_tRNA−Lys−CTT−10
0.020
0.015
0.010
0.005
0.000
RPM
0
20
40
60
80
Length

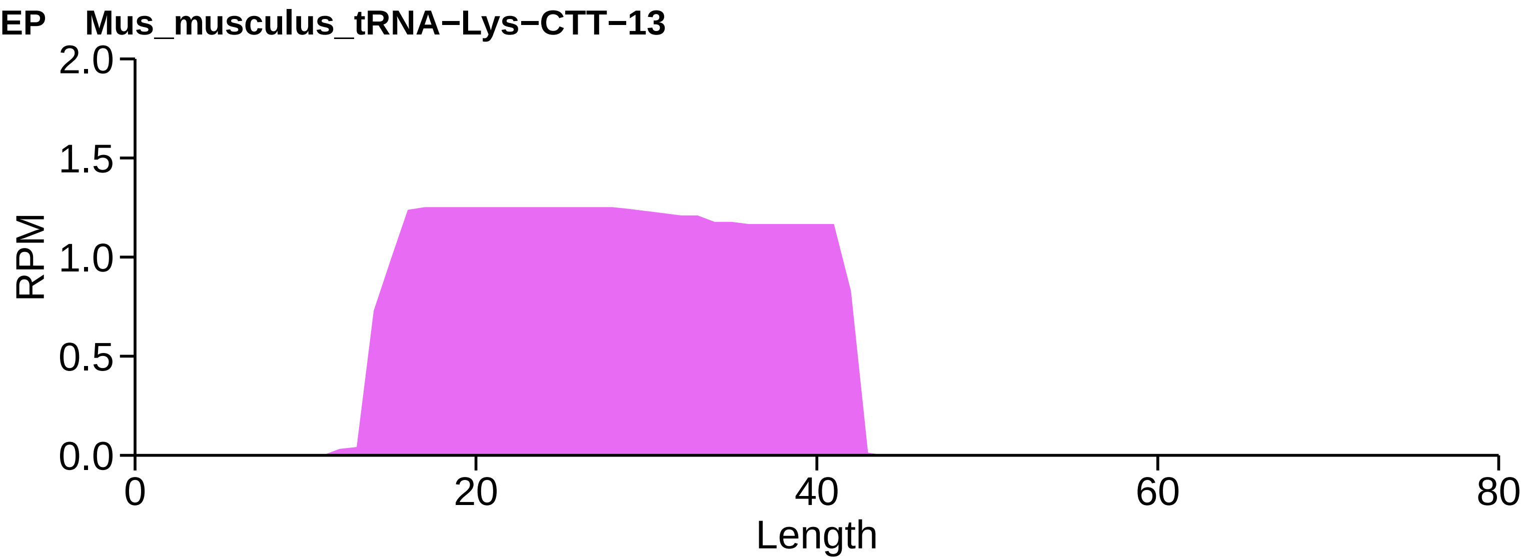
EP Mus_musculus_tRNA−Lys−CTT−13
2.0
1.5
1.0
0.5
0.0
RPM
0
20
40
60
80
Length

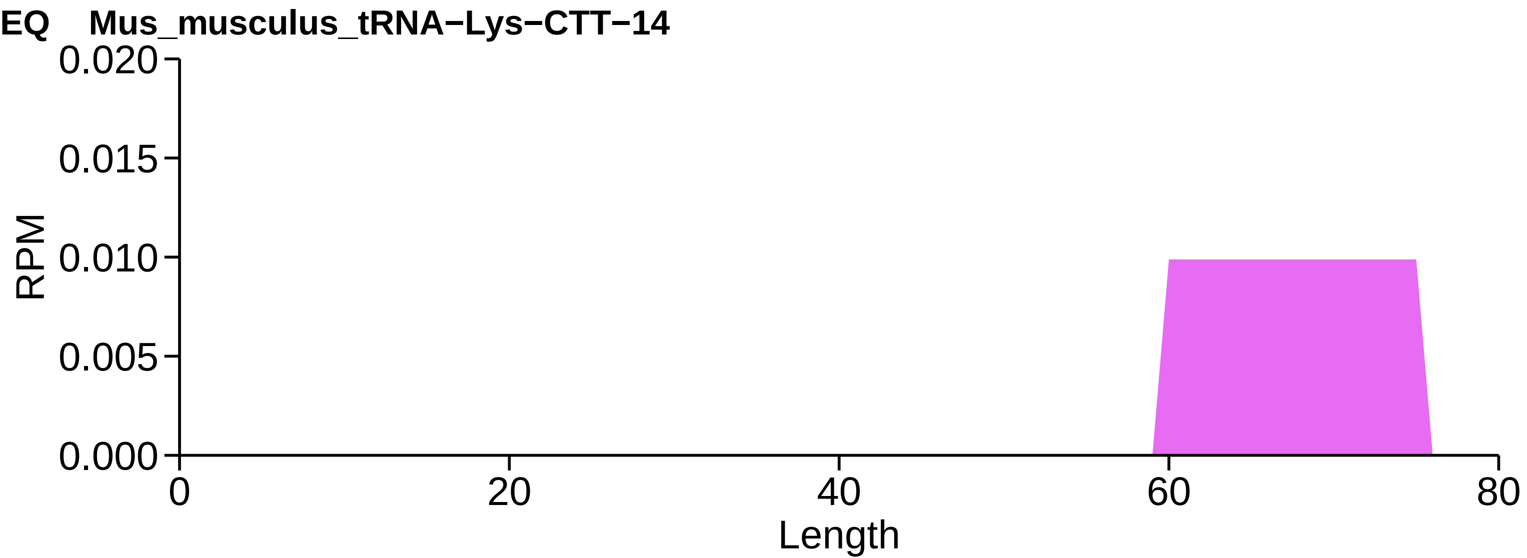

EQ Mus_musculus_tRNA−Lys−CTT−14
0.020
0.015
0.010
0.005
0.000
RPM
0
20
40
60
80
Length

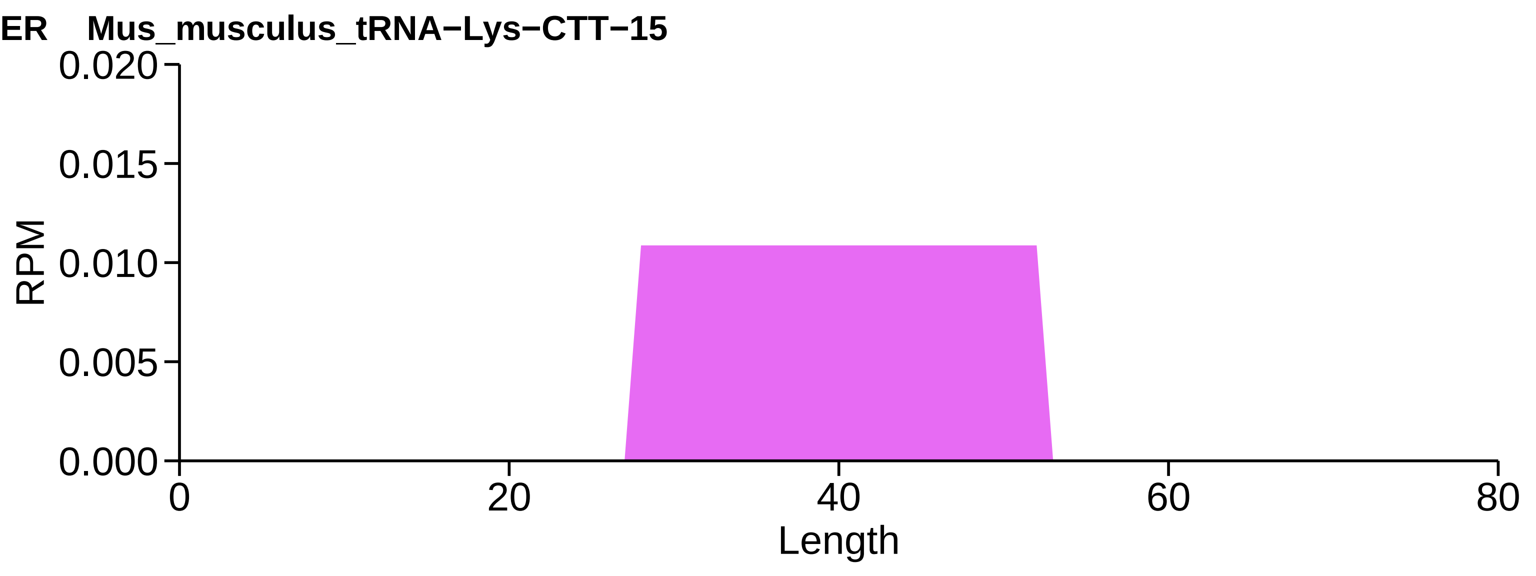

ER Mus_musculus_tRNA−Lys−CTT−15
RPM
0.020
0.015
0.010
0.005
0.000
0
20
40
60
80
Length

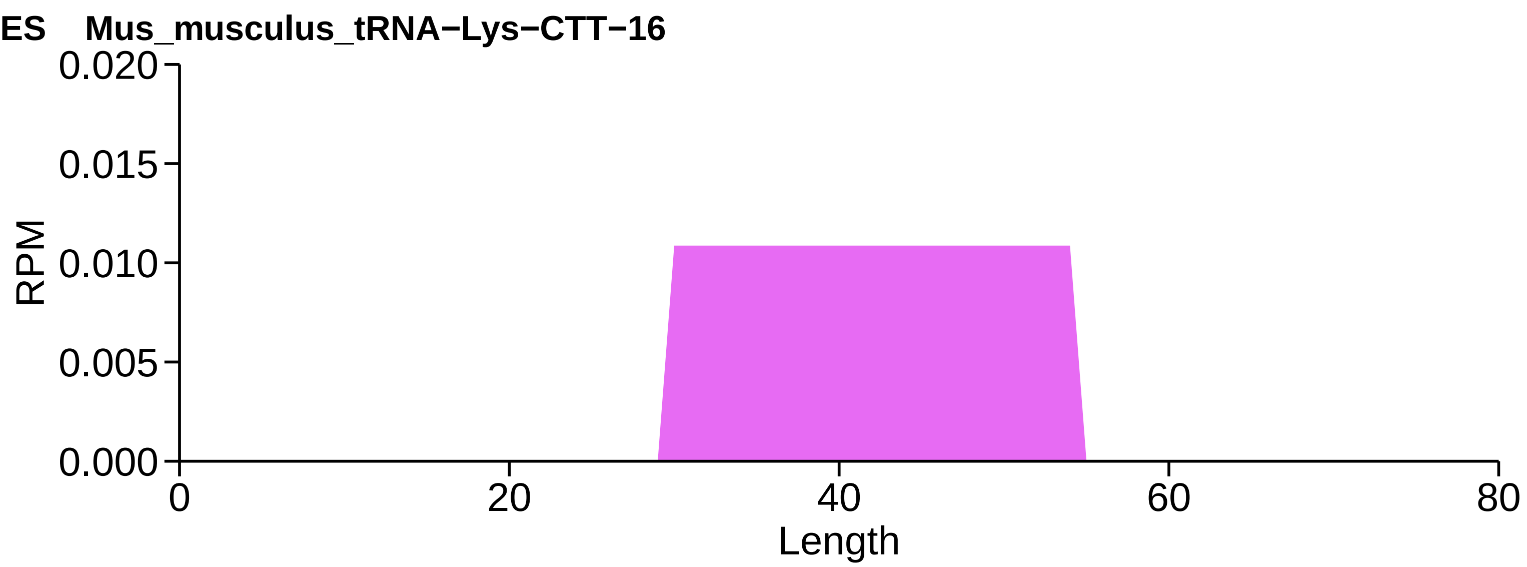
ES Mus_musculus_tRNA−Lys−CTT−16
RPM
0.020
0.015
0.010
0.005
0.000
0
20
40
60
80
Length

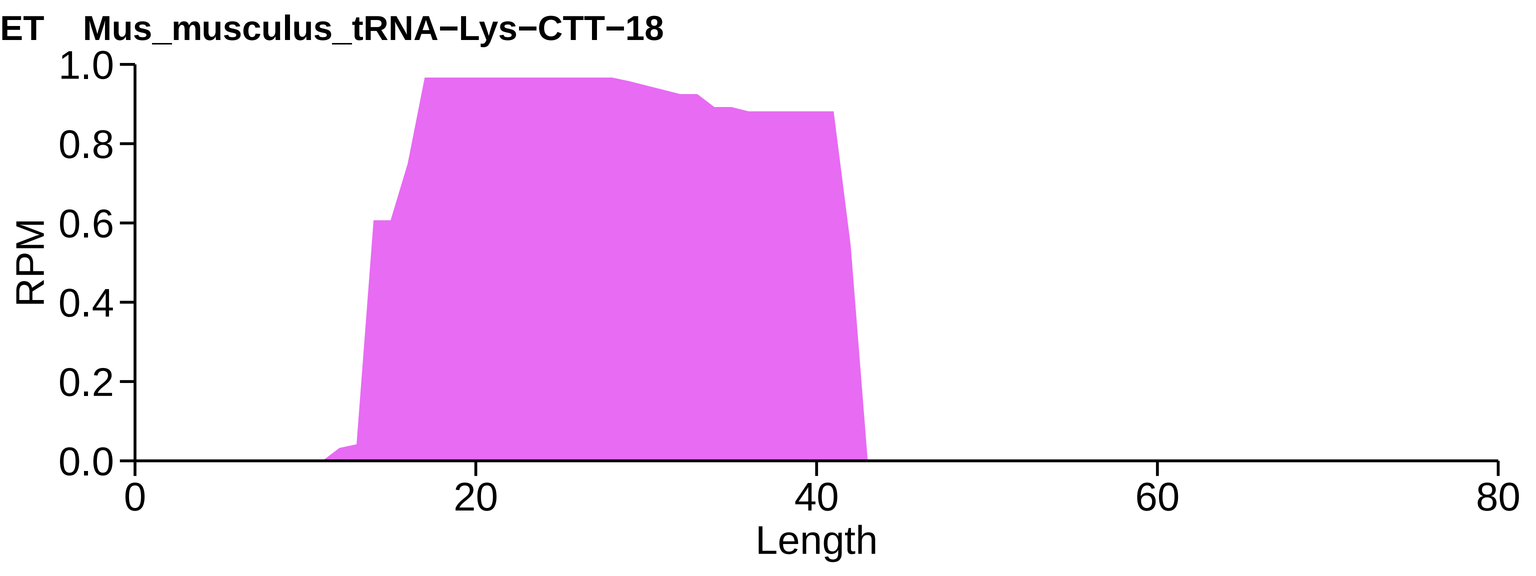
ET Mus_musculus_tRNA−Lys−CTT−18
RPM
1.0
0.8
0.6
0.4
0.2
0.0
0
20
40
60
80
Length

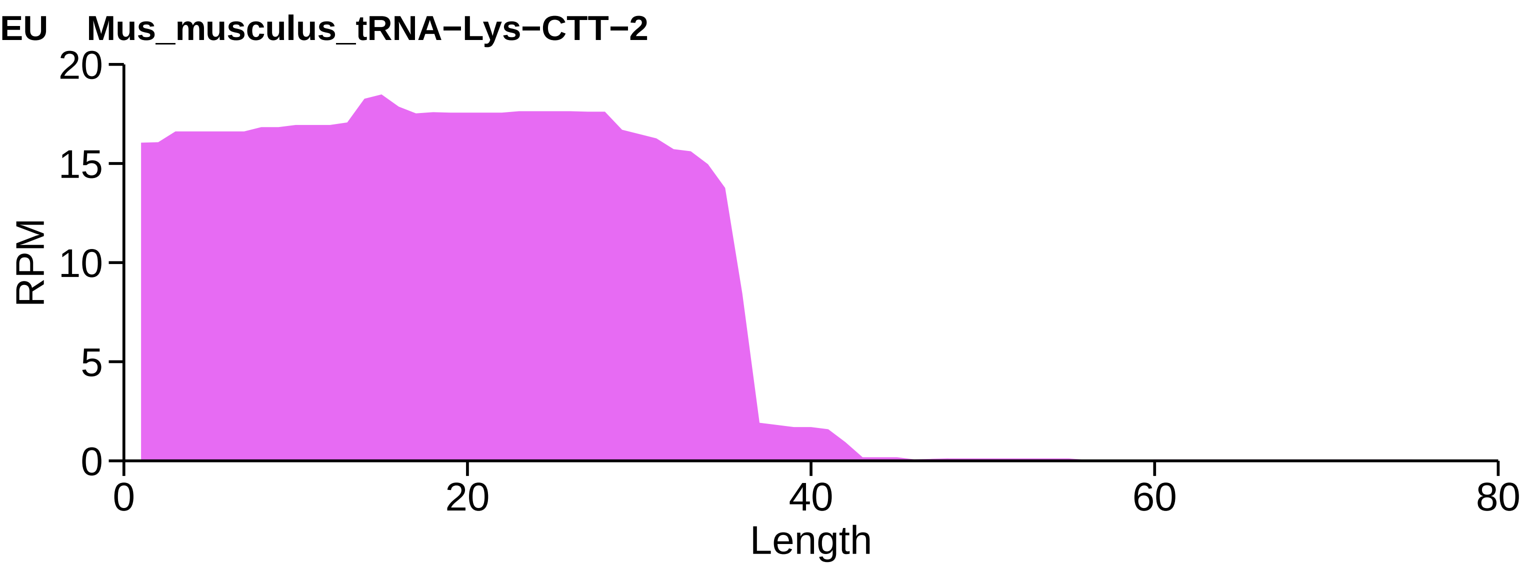
EU Mus_musculus_tRNA−Lys−CTT−2
RPM
20
15
10
5
0
0
20
40
60
80
Length

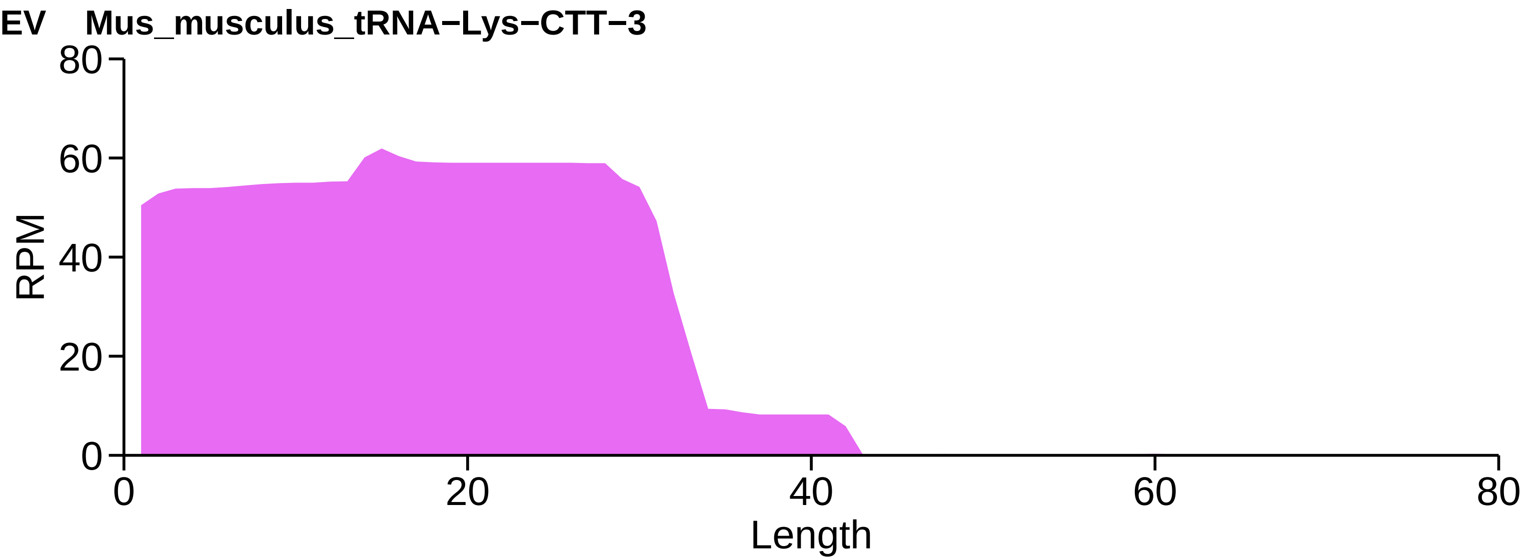
EV Mus_musculus_tRNA−Lys−CTT−3
RPM
0
20
40
60
80
0
20
40
60
80
Length

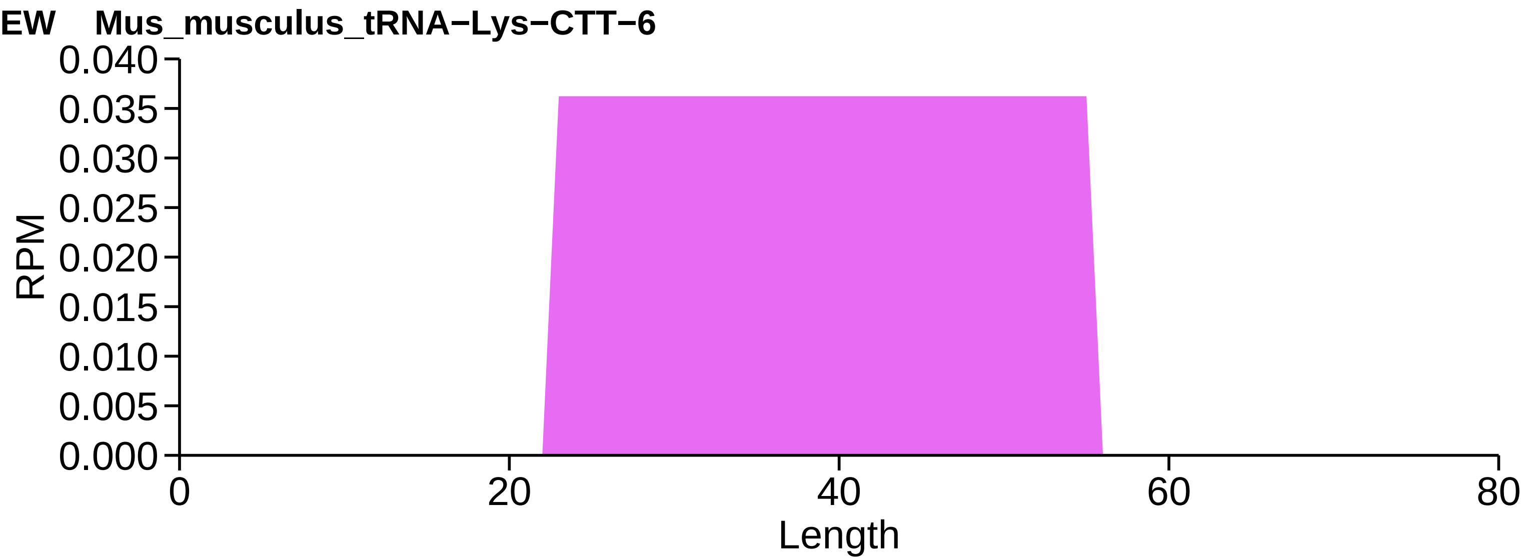

EW Mus_musculus_tRNA−Lys−CTT−6
RPM
0.040
0.035
0.030
0.025
0.020
0.015
0.010
0.005
0.000
0
20
40
60
80
Length

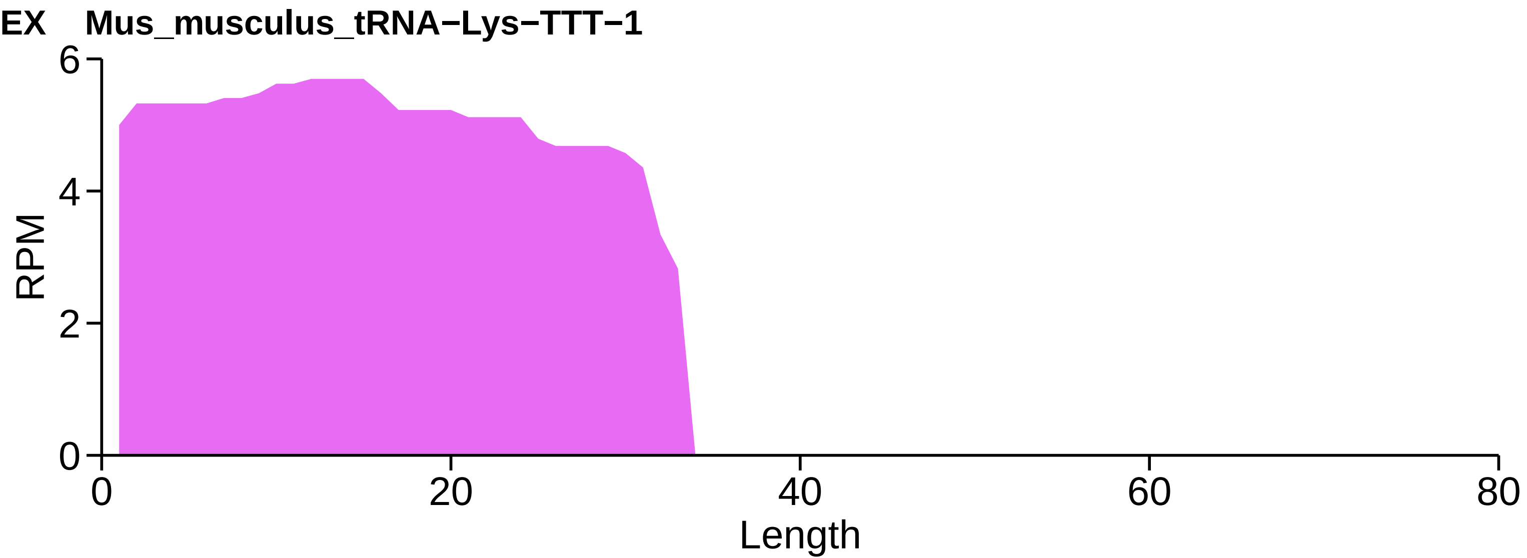

EX Mus_musculus_tRNA−Lys−TTT−1
RPM
Length
0
2
4
6
0
20
40
60
80

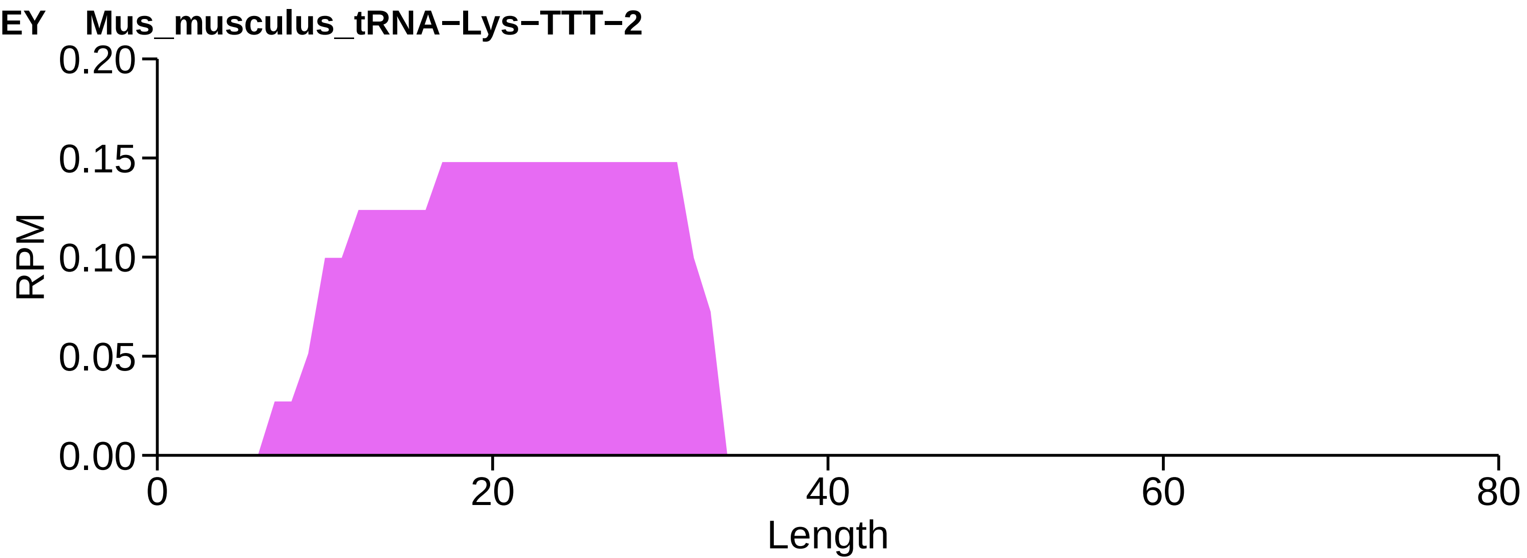
EY Mus_musculus_tRNA−Lys−TTT−2
RPM
0.20
0.15
0.10
0.05
0.00
0
20
40
60
80
Length

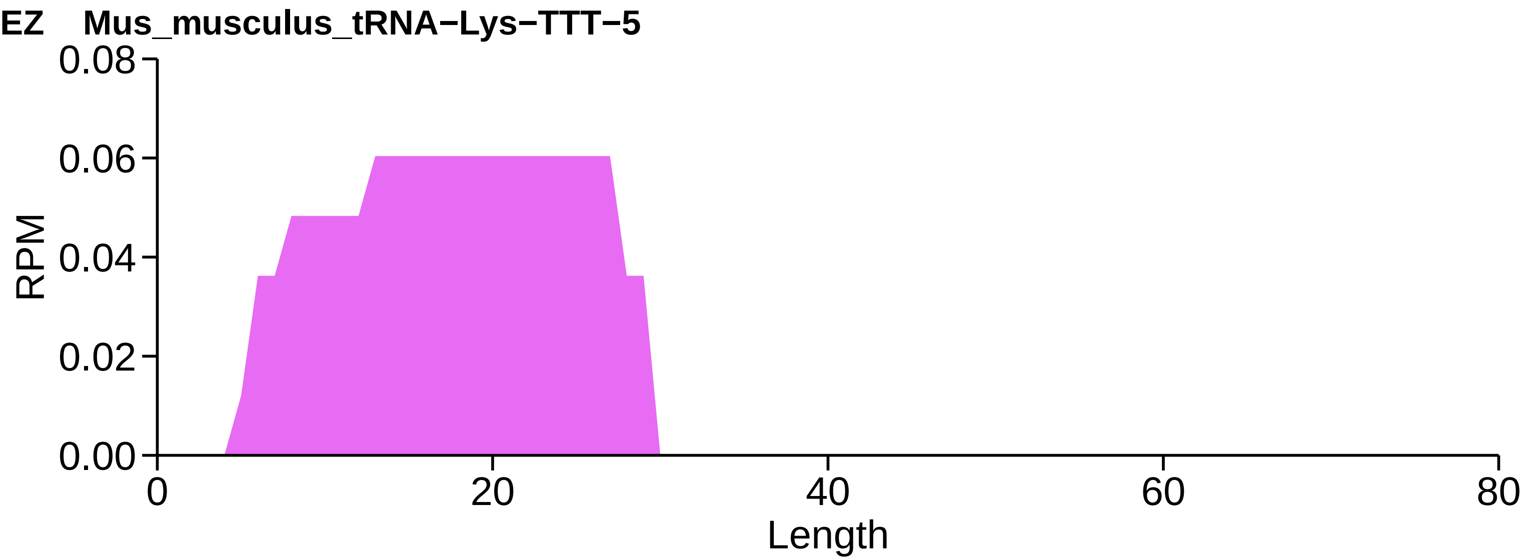
EZ Mus_musculus_tRNA−Lys−TTT−5
RPM
0.08
0.06
0.04
0.02
0.00
0
20
40
60
80
Length

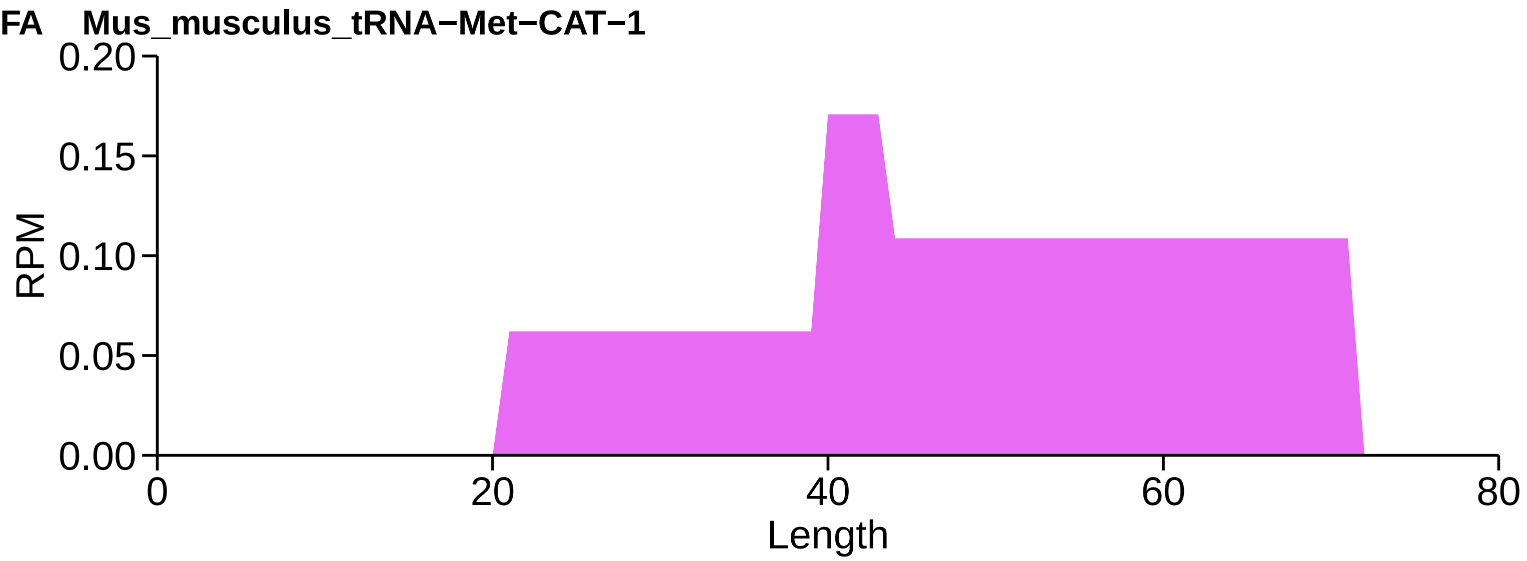
FA Mus_musculus_tRNA−Met−CAT−1
RPM
0.20
0.15
0.10
0.05
0.00
0
20
40
60
80
Length

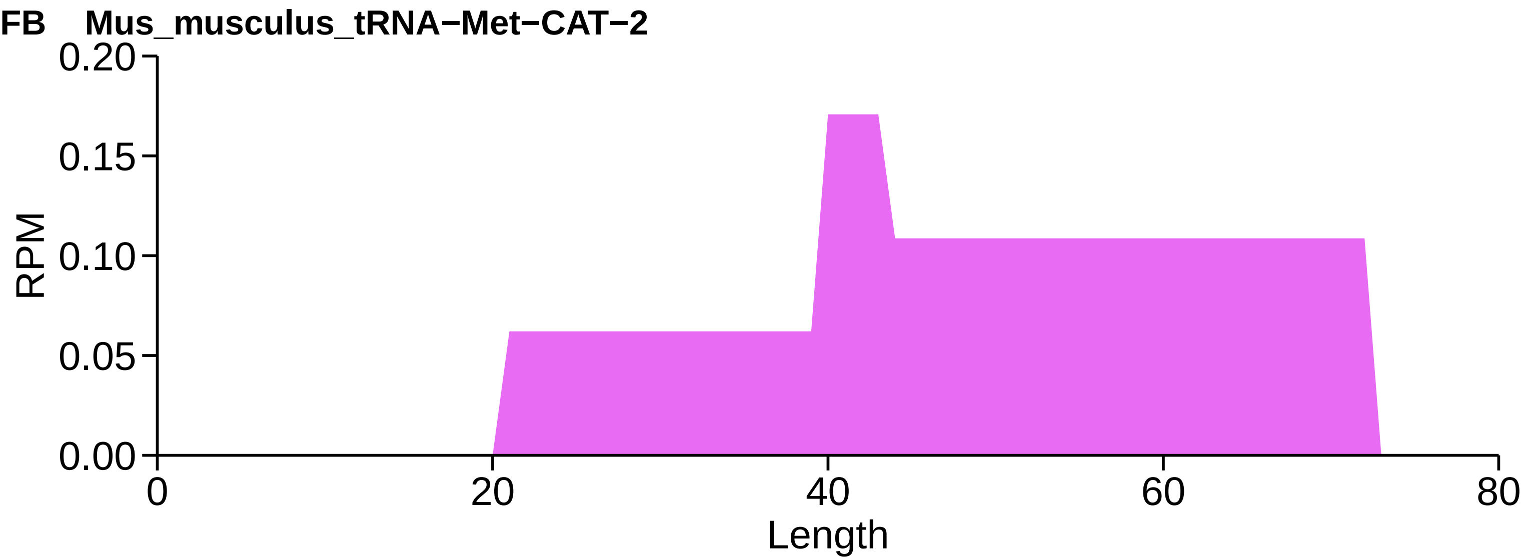
FB Mus_musculus_tRNA−Met−CAT−2
RPM
0.20
0.15
0.10
0.05
0.00
0
20
40
60
80
Length

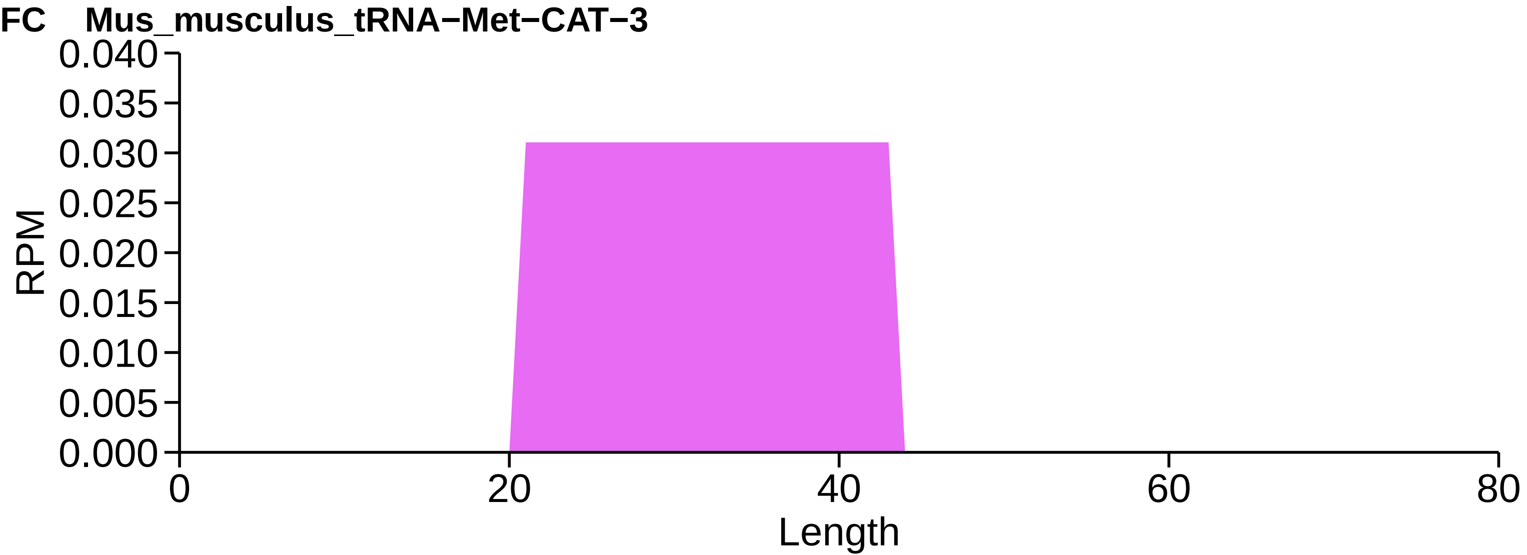

FC Mus_musculus_tRNA−Met−CAT−3
RPM
0.040
0.035
0.030
0.025
0.020
0.015
0.010
0.005
0.000
0
20
40
60
80
Length

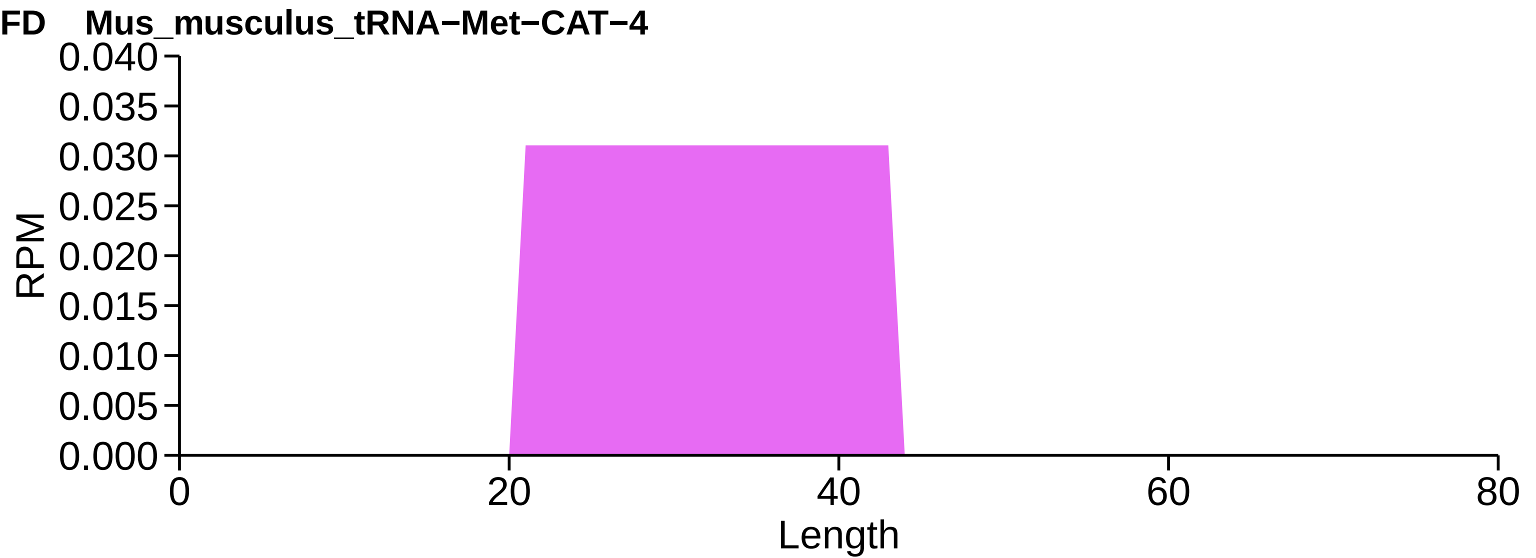
FD
Mus_musculus_tRNA−Met−CAT−4
0.040
0.035
0.030
0.025
0.020
0.015
0.010
0.005
0.000
RPM
0
20
40
60
80
Length

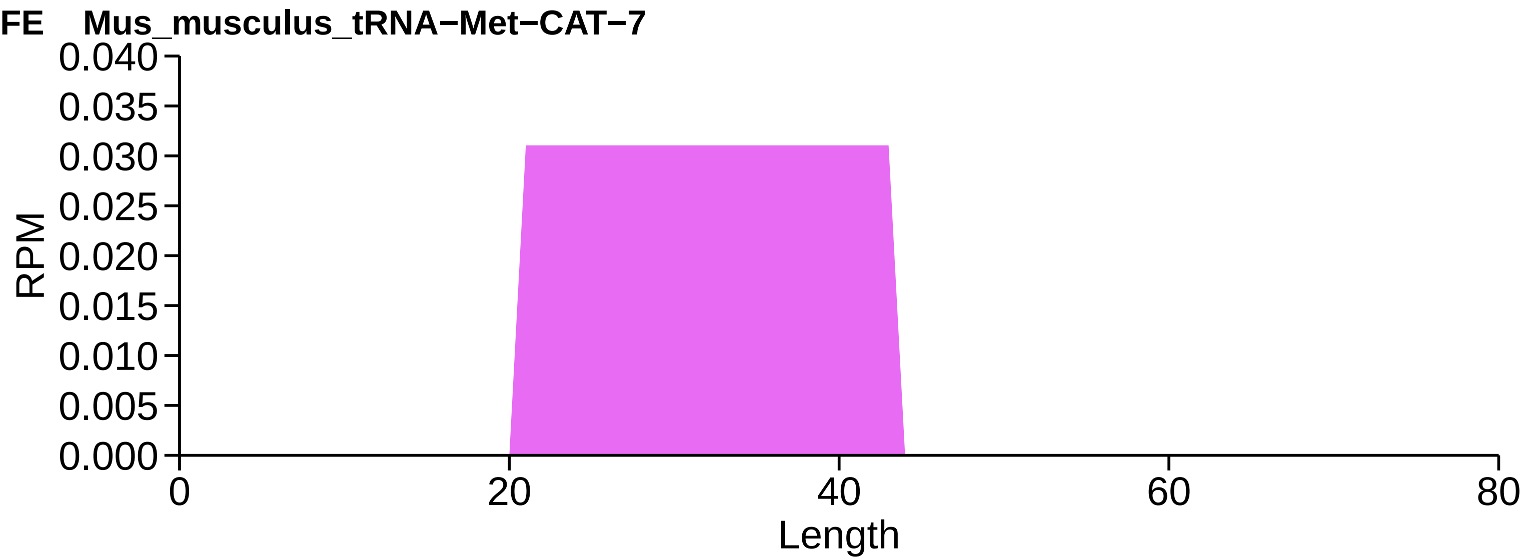
FE Mus_musculus_tRNA−Met−CAT−7
RPM
0.040
0.035
0.030
0.025
0.020
0.015
0.010
0.005
0.000
0
20
40
60
80
Length

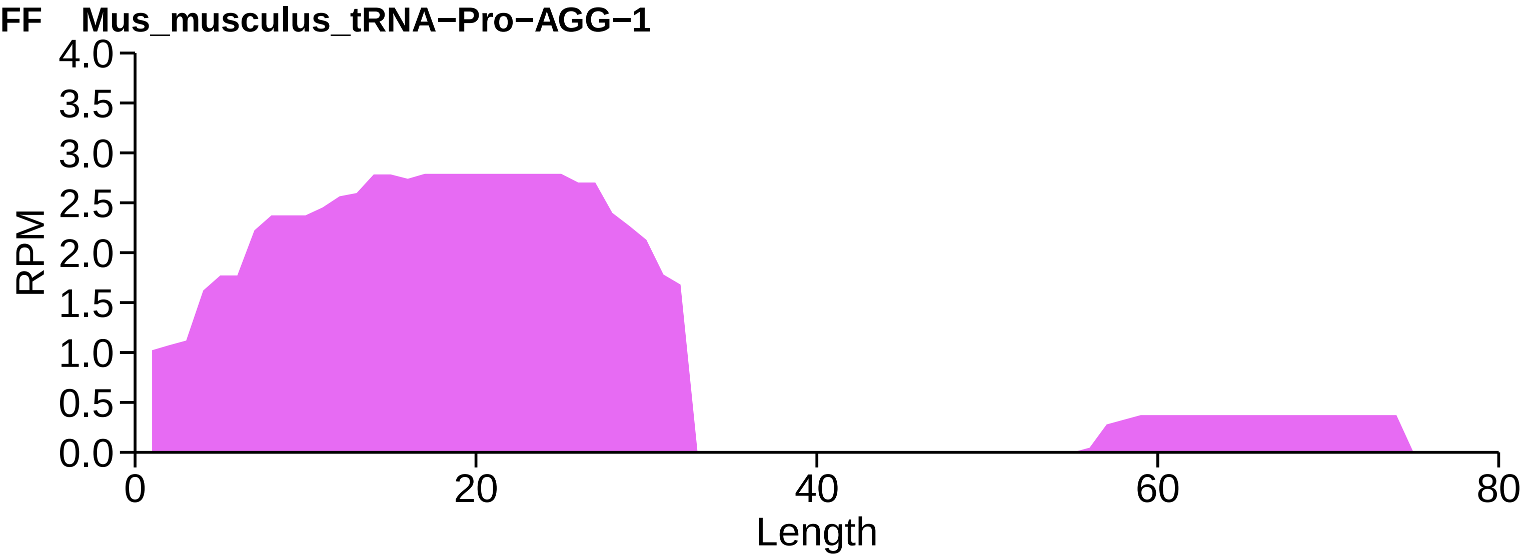

FF Mus_musculus_tRNA−Pro−AGG−1
RPM
4.0
3.5
3.0
2.5
2.0
1.5
1.0
0.5
0.0
0
20
40
60
80
Length

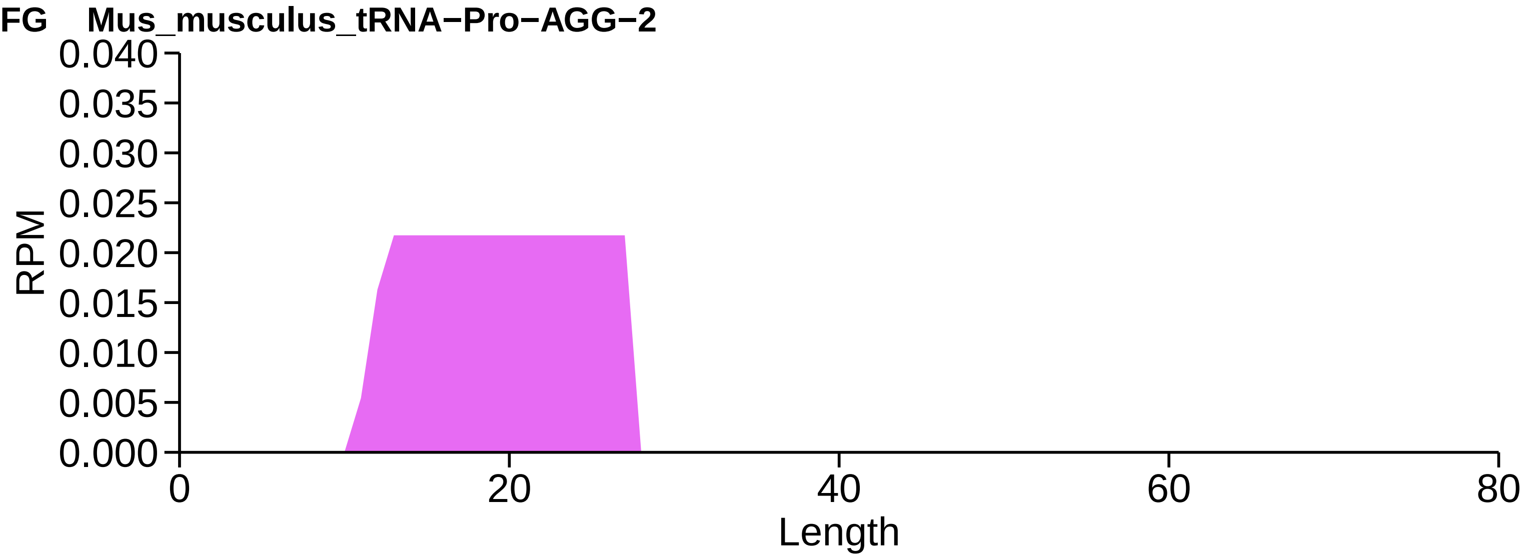
FG Mus_musculus_tRNA−Pro−AGG−2
RPM
0.040
0.035
0.030
0.025
0.020
0.015
0.010
0.005
0.000
0
20
40
60
80
Length

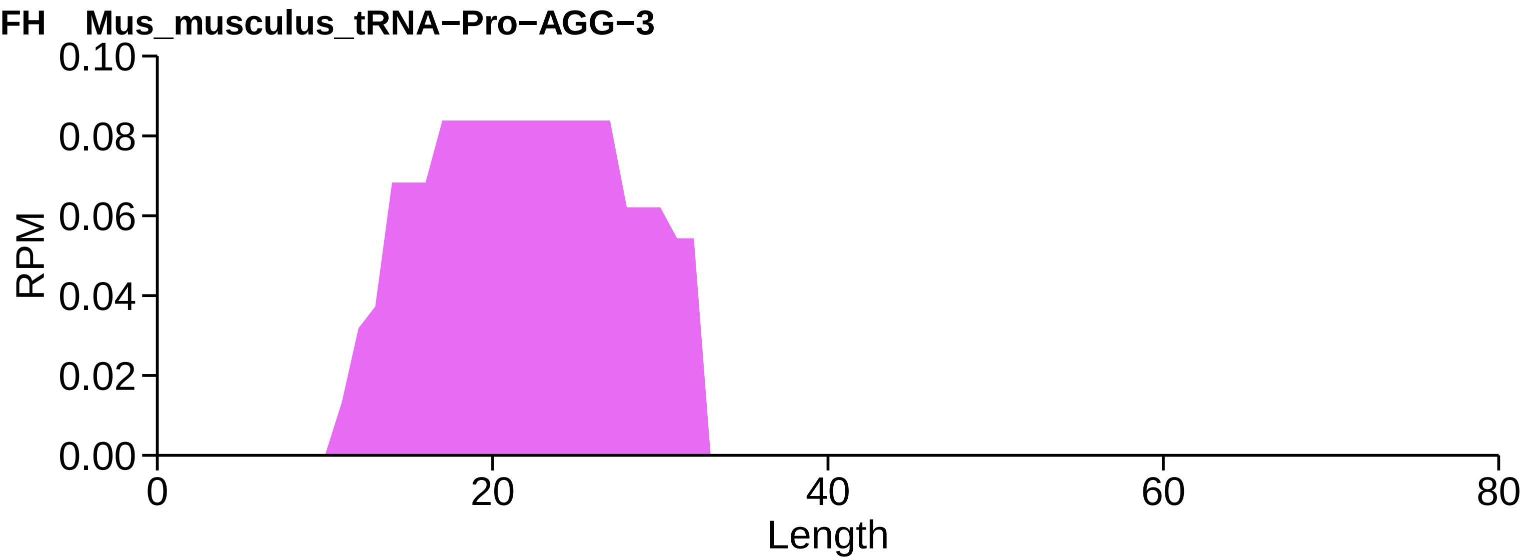
FH Mus_musculus_tRNA−Pro−AGG−3
RPM
0.10
0.08
0.06
0.04
0.02
0.00
0
20
40
60
80
Length

FI Mus_musculus_tRNA−Pro−CGG−1

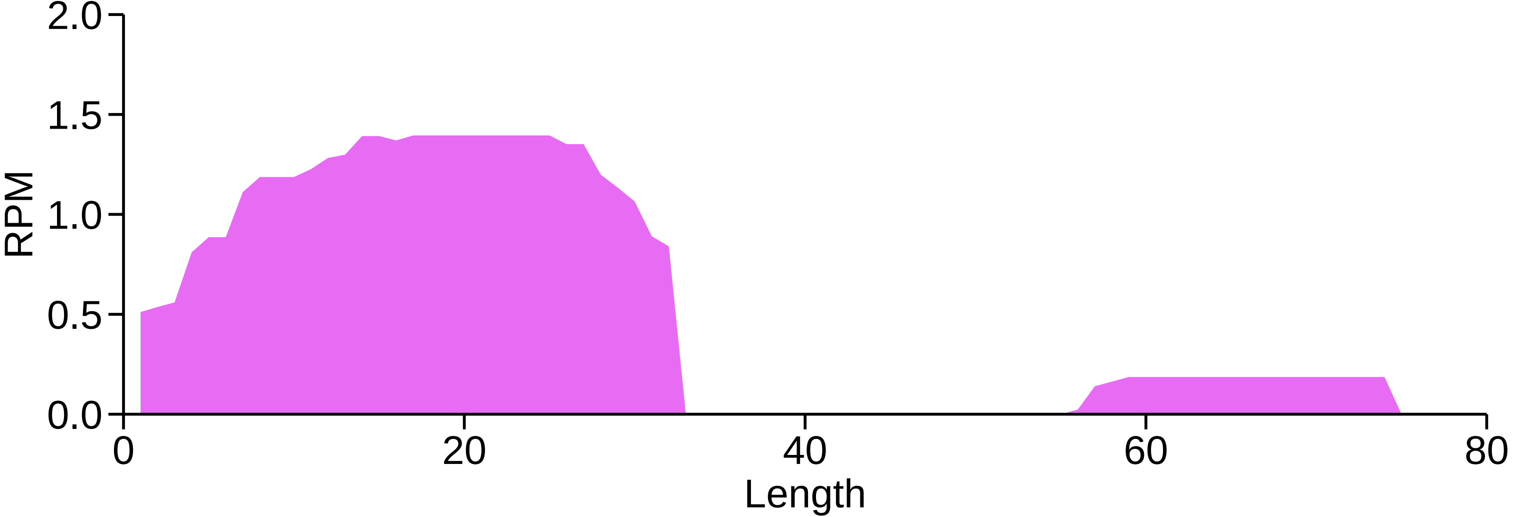

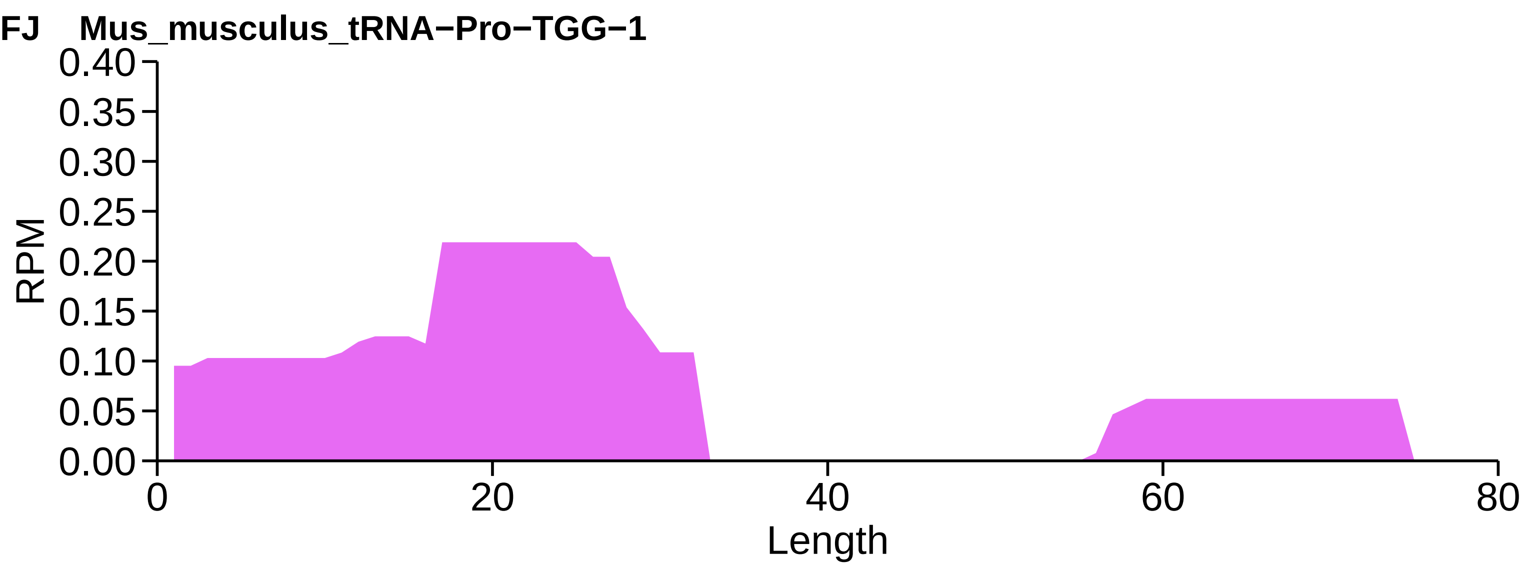
FJ Mus_musculus_tRNA−Pro−TGG−1
RPM
0.40
0.35
0.30
0.25
0.20
0.15
0.10
0.05
0.00
0
20
40
60
80
Length

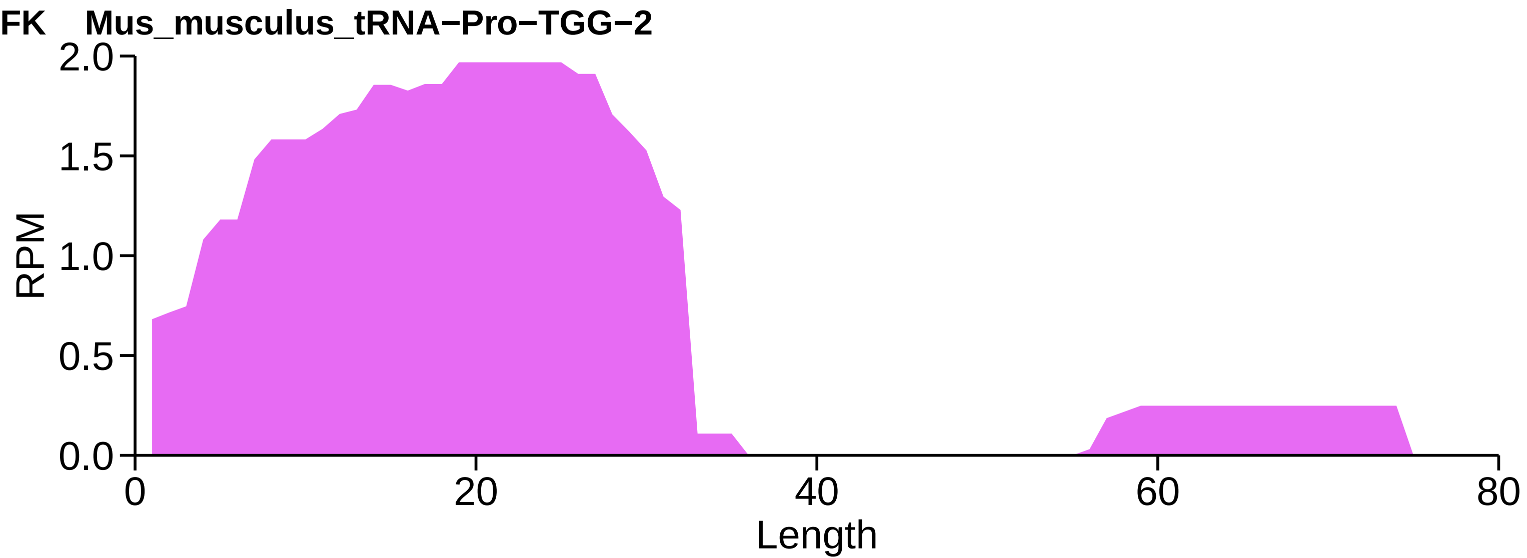
FK Mus_musculus_tRNA−Pro−TGG−2
RPM
2.0
1.5
1.0
0.5
0.0
0
20
40
60
80
Length

FL Mus_musculus_tRNA−Pro−TGG−3

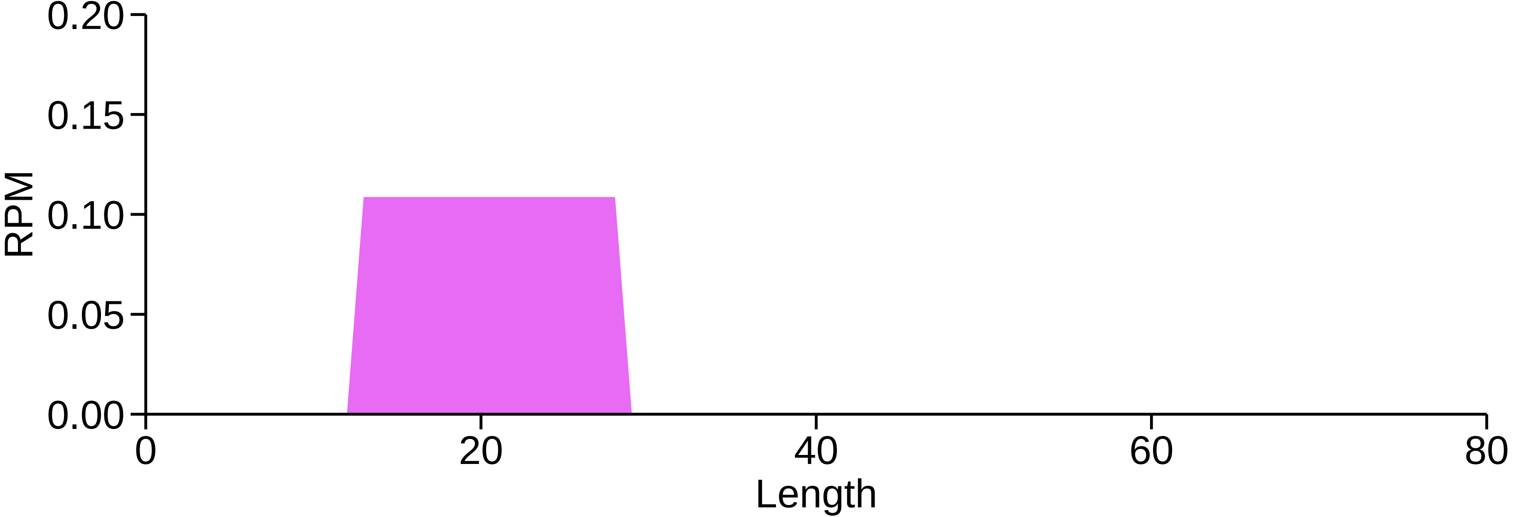

FM Mus_musculus_tRNA−Pro−TGG−4

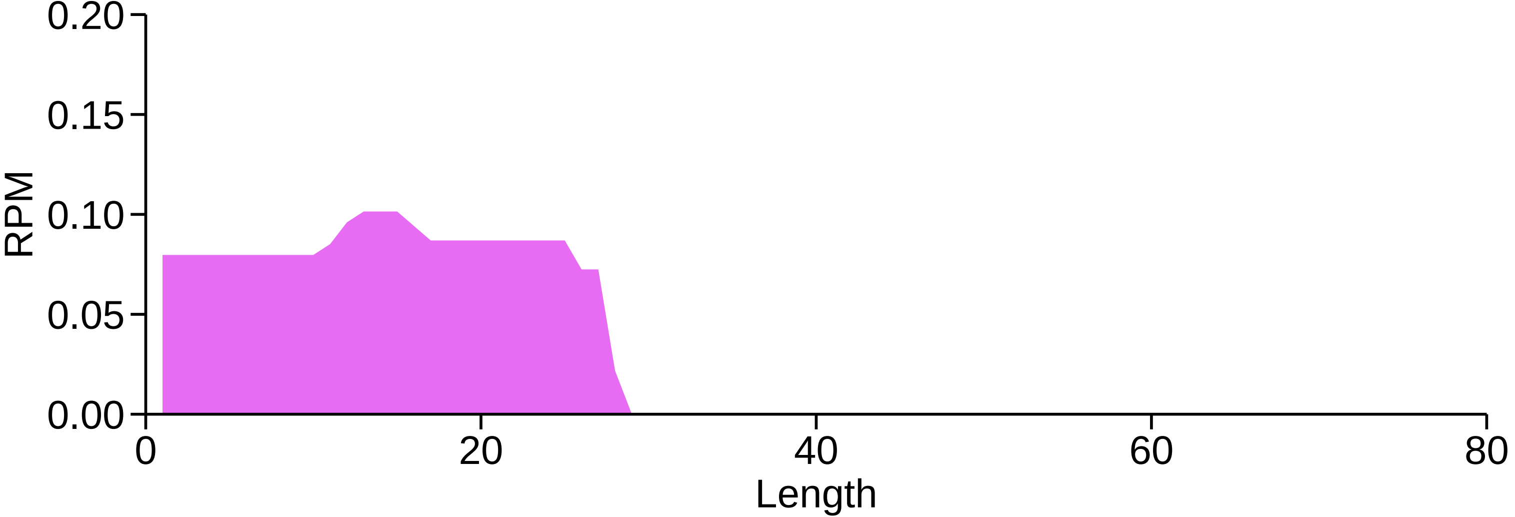

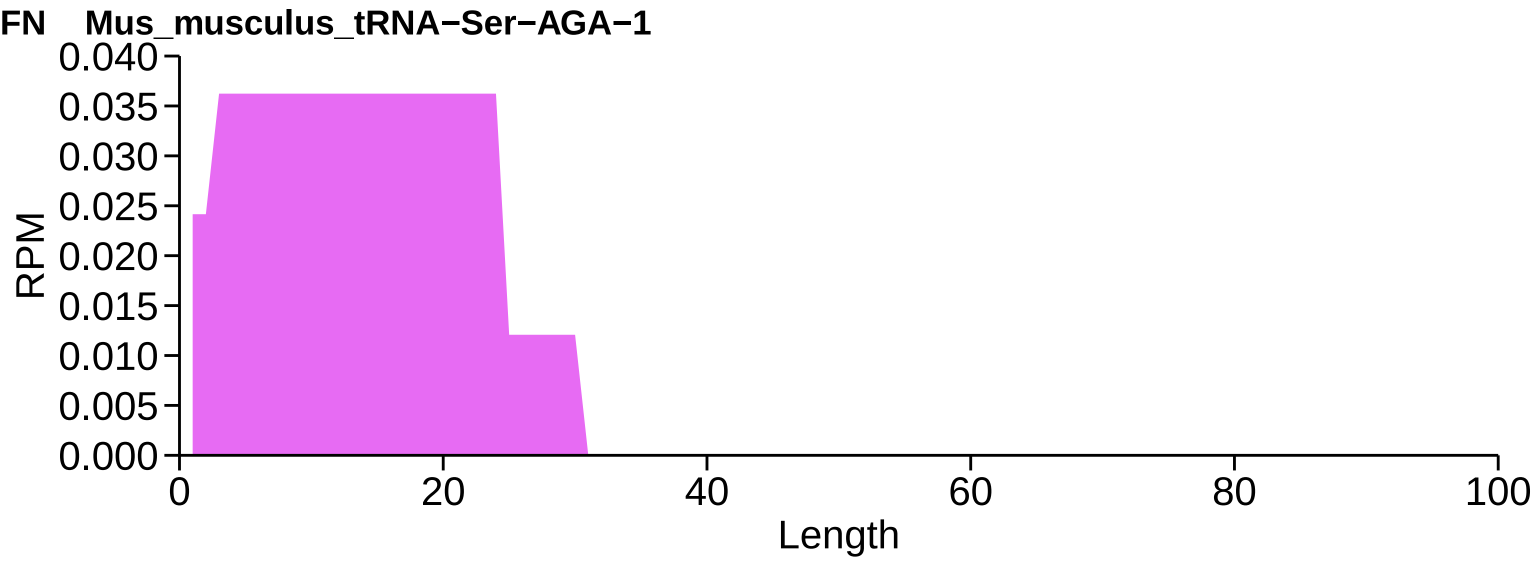
FN Mus_musculus_tRNA−Ser−AGA−1
0.040
0.035
0.030
0.025
0.020
0.015
0.010
0.005
0.000
RPM
0
20
40
60
80
100
Length

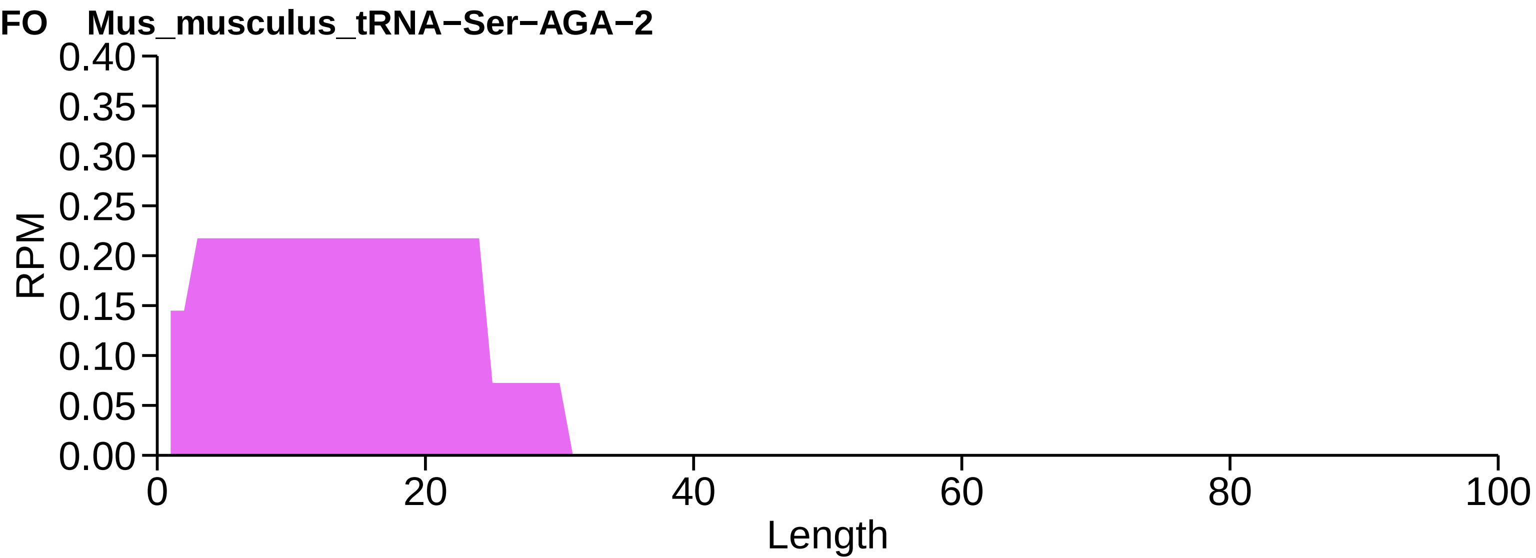
FO Mus_musculus_tRNA−Ser−AGA−2
RPM
0.40
0.35
0.30
0.25
0.20
0.15
0.10
0.05
0.00
0
20
40
60
80
100
Length

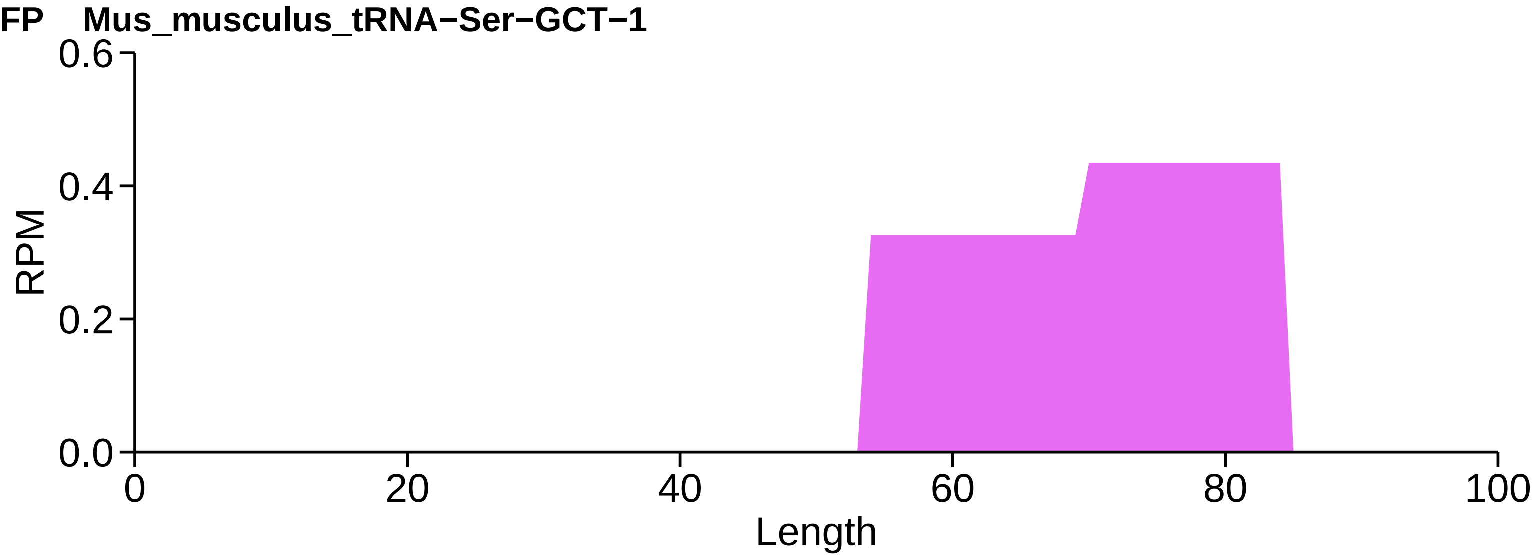

FP Mus_musculus_tRNA−Ser−GCT−1
0.6
0.4
0.2
0.0
RPM
0
20
40
60
80
100
Length

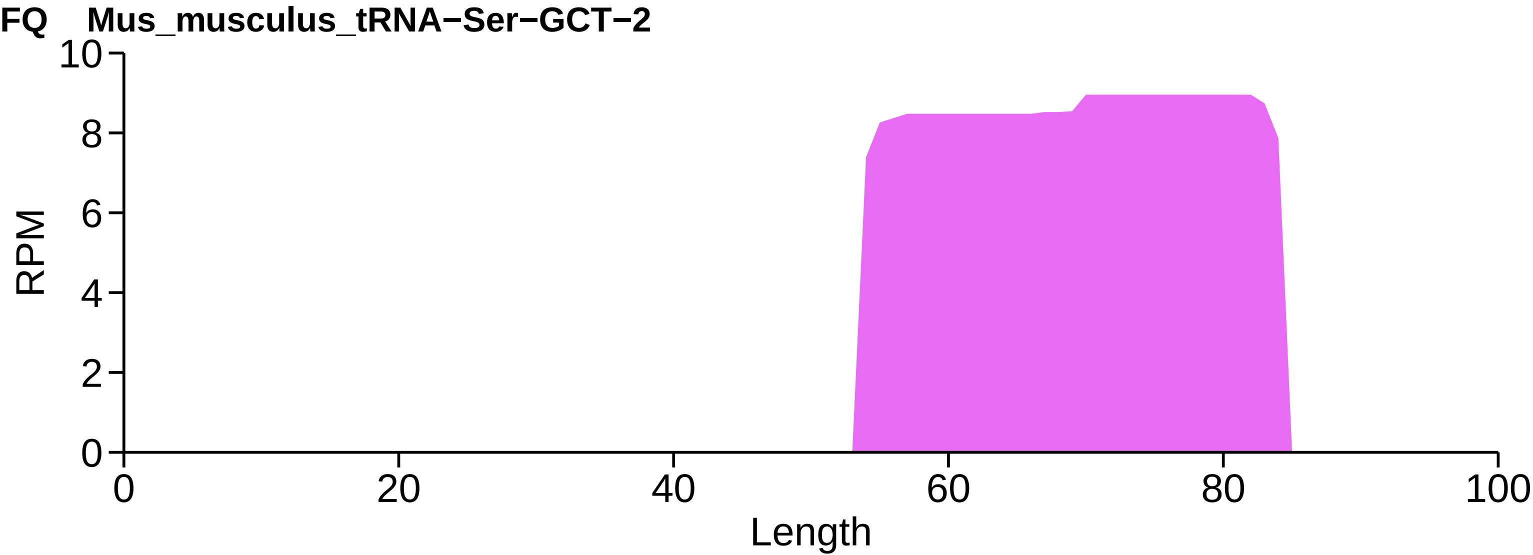

FQ Mus_musculus_tRNA−Ser−GCT−2
RPM
10
8
6
4
2
0
0
20
40
60
80
100
Length

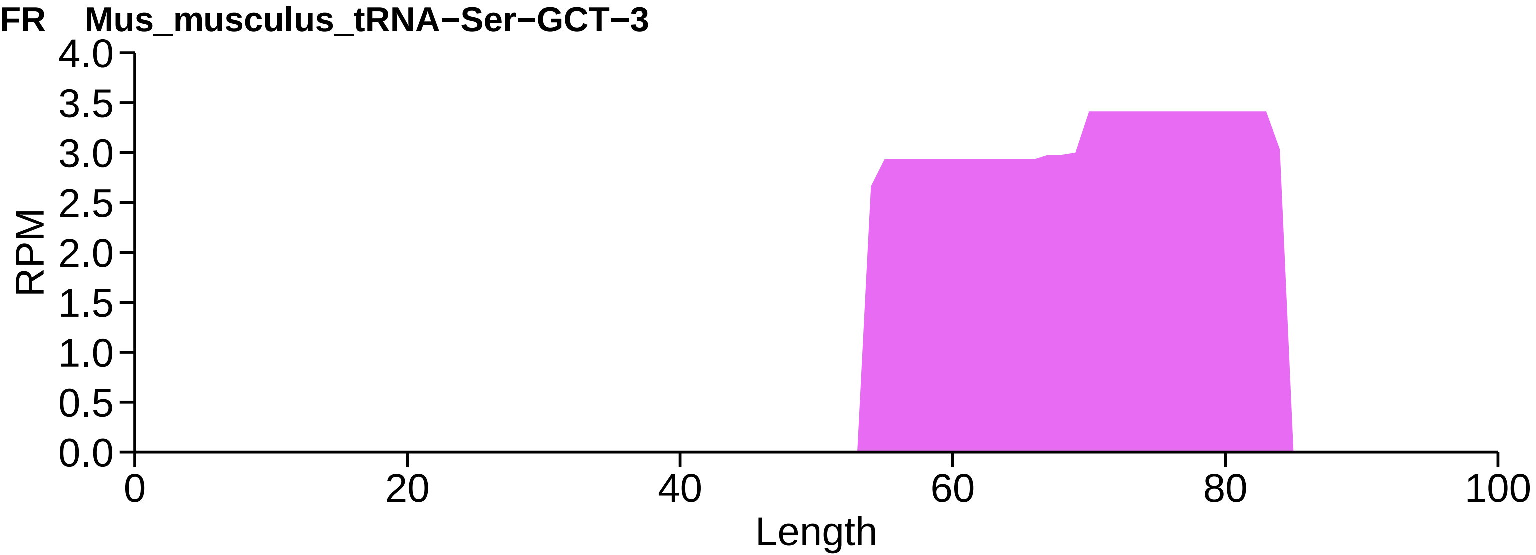
FR Mus_musculus_tRNA−Ser−GCT−3
RPM
4.0
3.5
3.0
2.5
2.0
1.5
1.0
0.5
0.0
0
20
40
60
80
100
Length

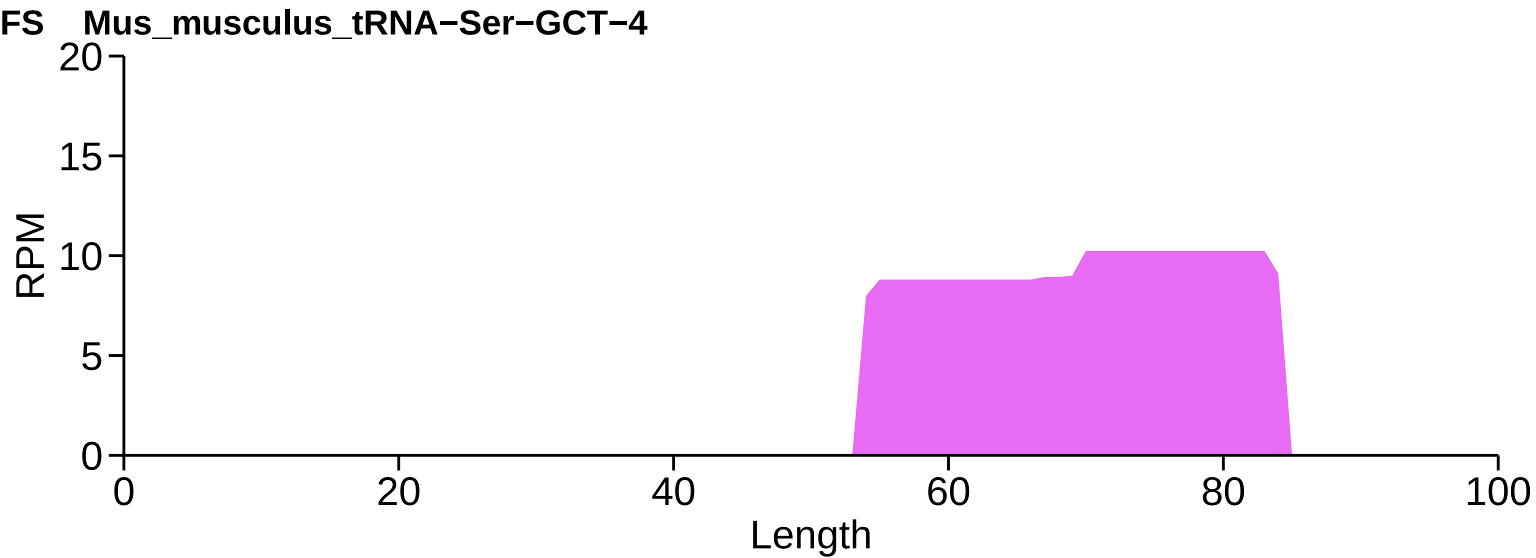
FS Mus_musculus_tRNA−Ser−GCT−4
RPM
0
5
10
15
20
0
20
40
60
80
100
Length

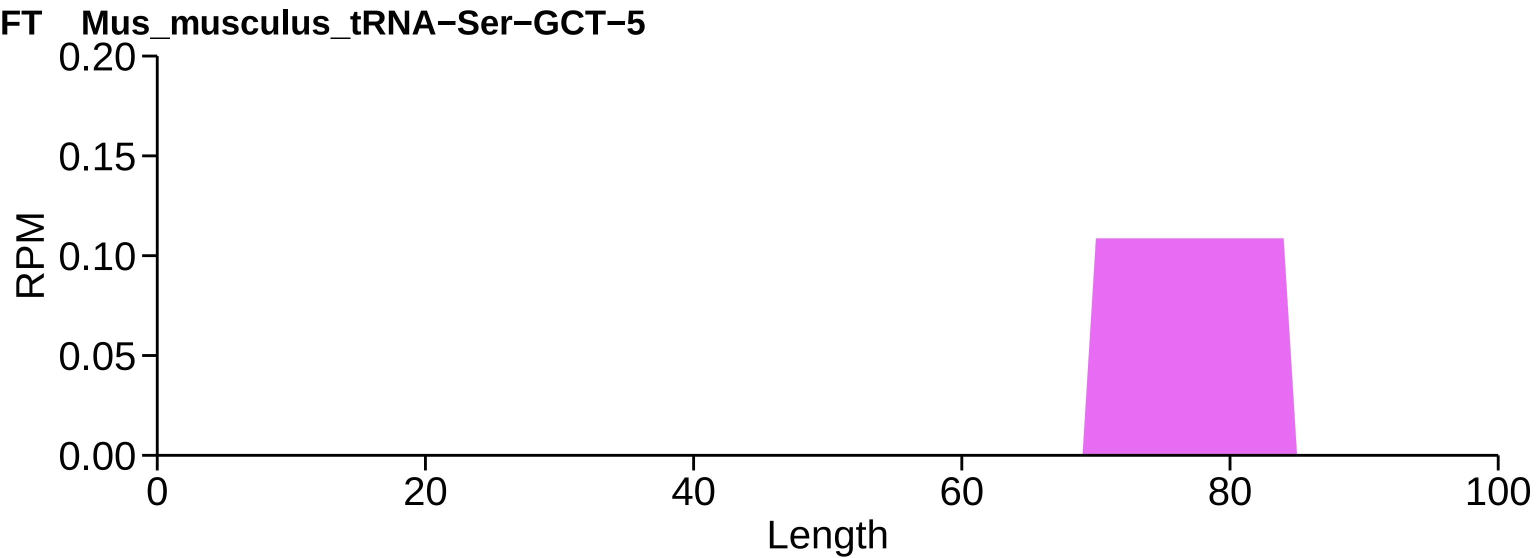
FT Mus_musculus_tRNA−Ser−GCT−5
RPM
0.20
0.15
0.10
0.05
0.00
0
20
40
60
80
100
Length

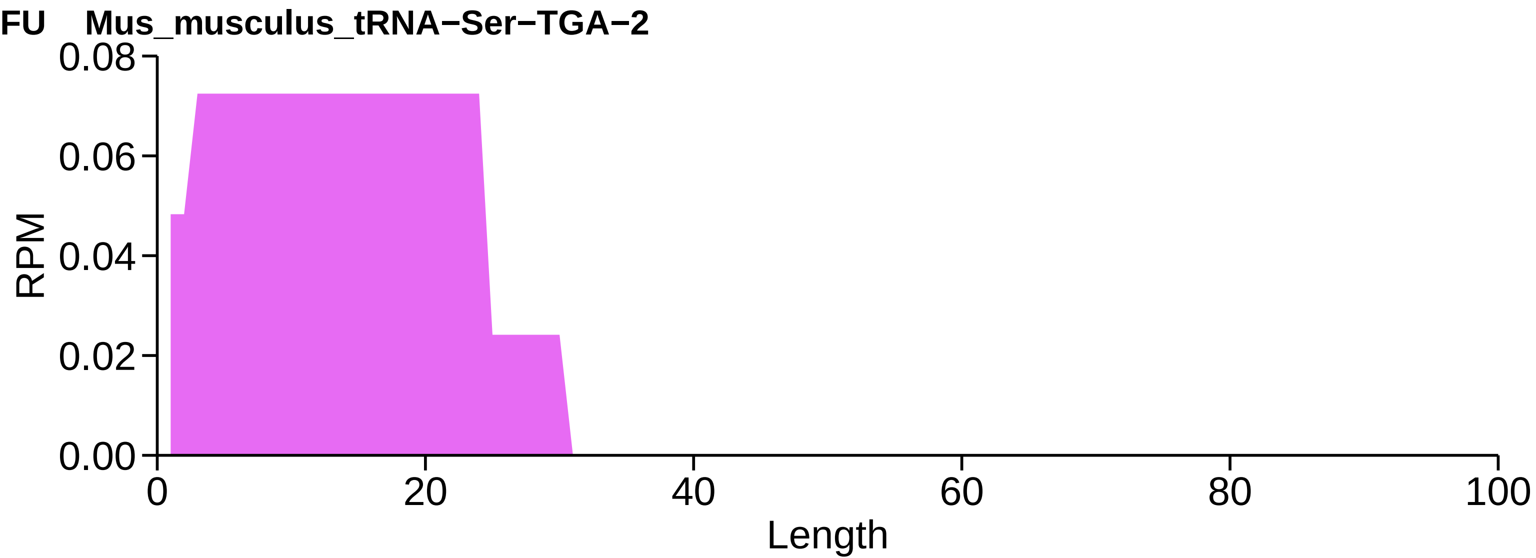
FU Mus_musculus_tRNA−Ser−TGA−2
RPM
0.08
0.06
0.04
0.02
0.00
0
20
40
60
80
100
Length

FV Mus_musculus_tRNA−Thr−AGT−1

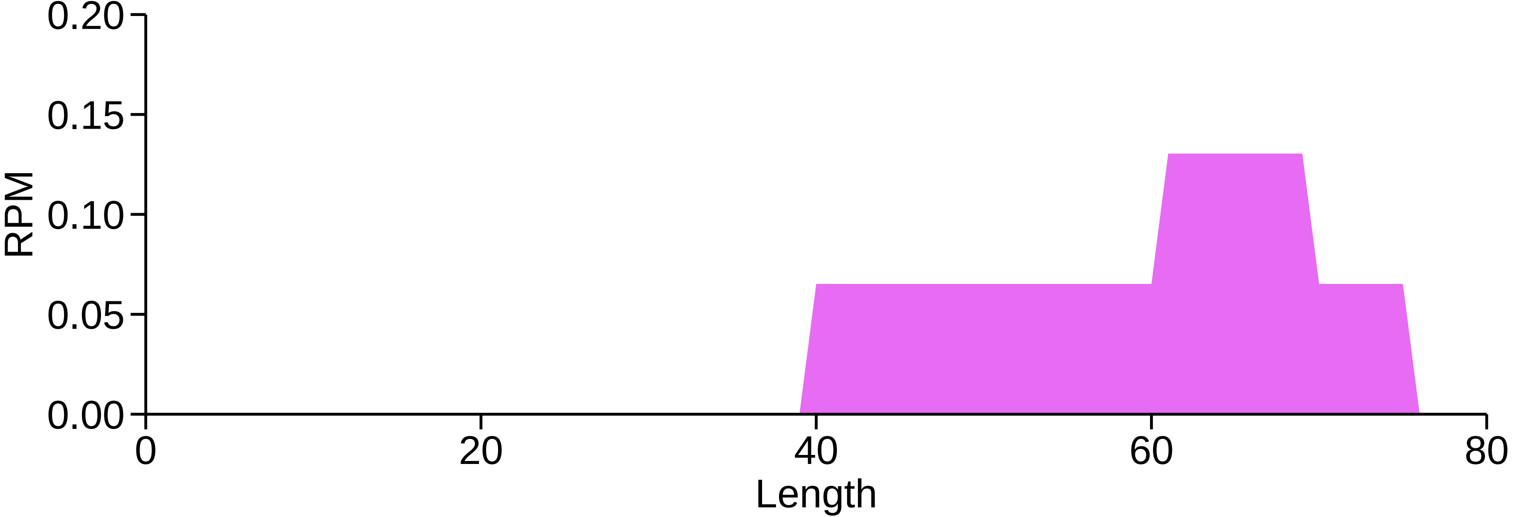

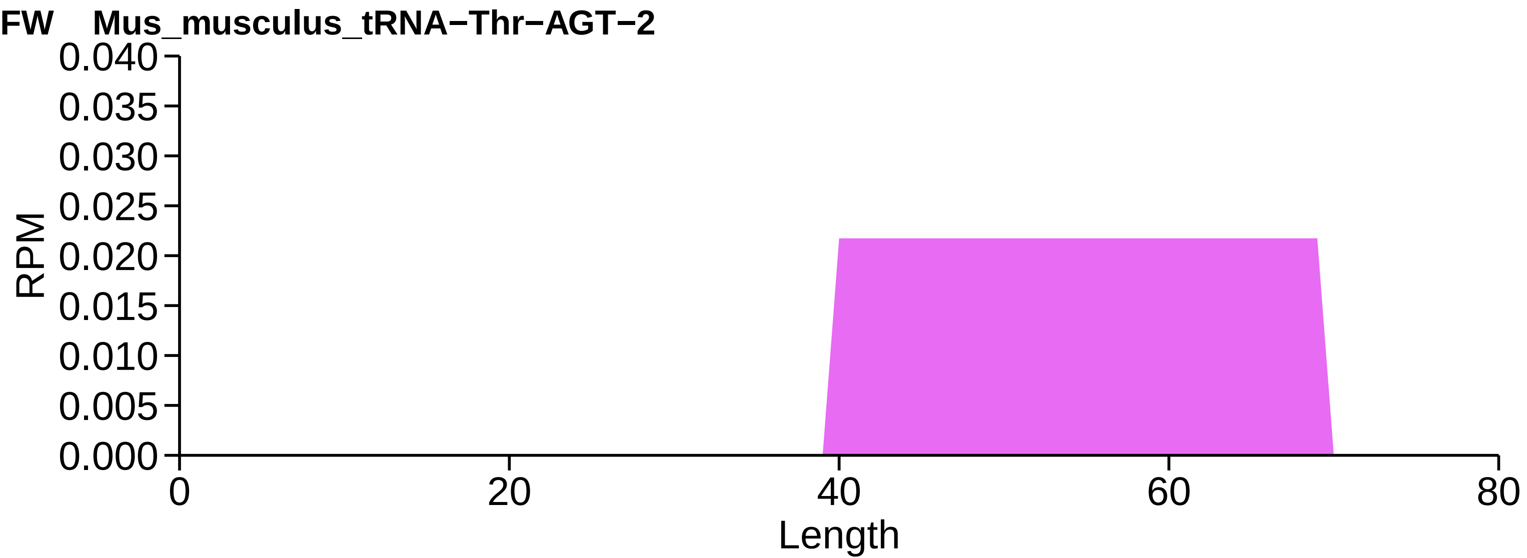

FW Mus_musculus_tRNA−Thr−AGT−2
0.040
0.035
0.030
0.025
0.020
0.015
0.010
0.005
0.000
RPM
0
20
40
60
80
Length

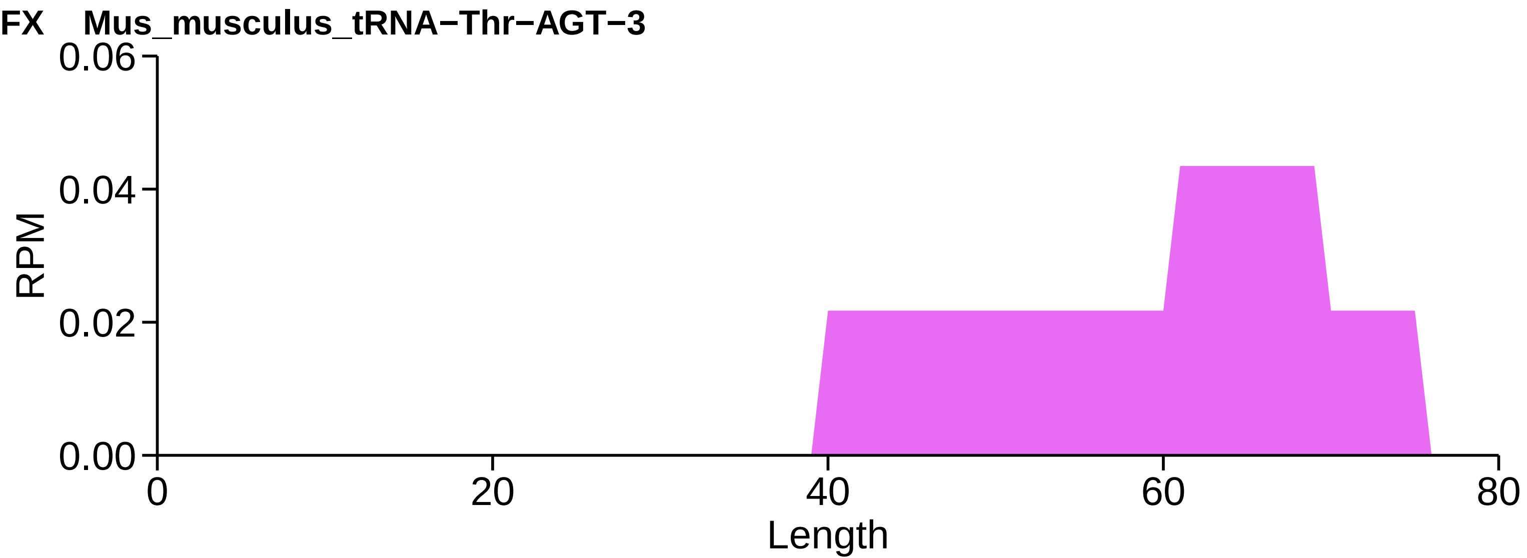
FX Mus_musculus_tRNA−Thr−AGT−3
0.06
0.04
0.02
0.00
RPM
0
20
40
60
80
Length

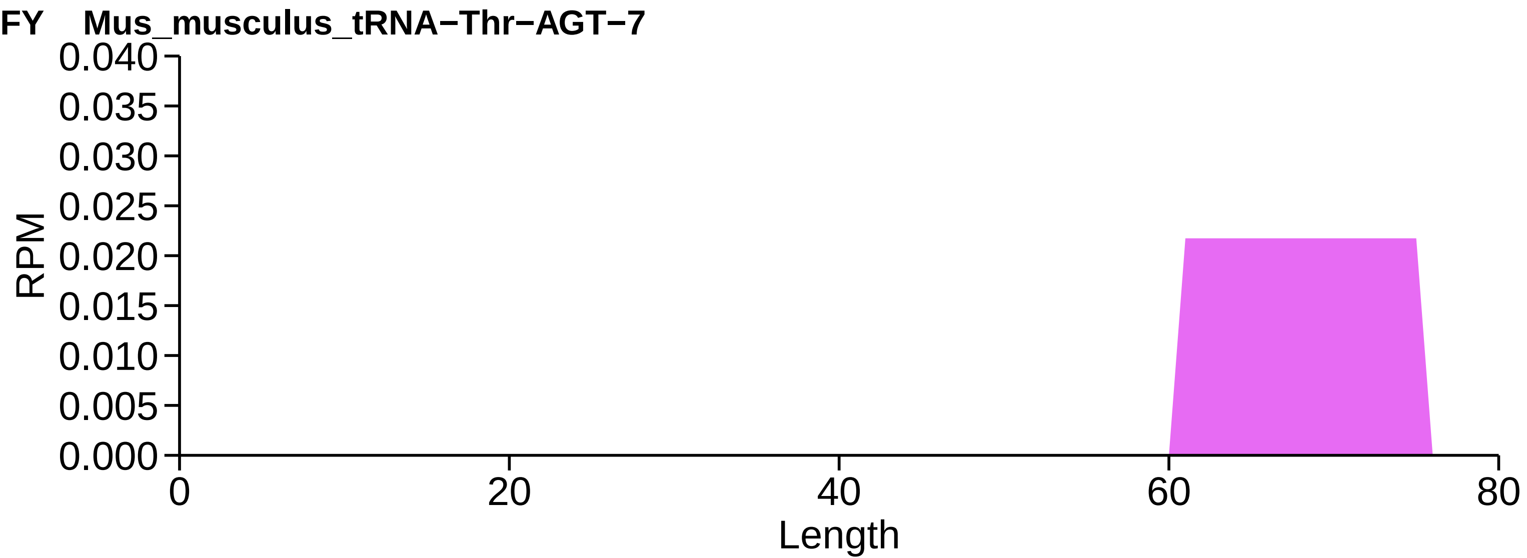
FY Mus_musculus_tRNA−Thr−AGT−7
0.040
0.035
0.030
0.025
0.020
0.015
0.010
0.005
0.000
RPM
0
20
40
60
80
Length

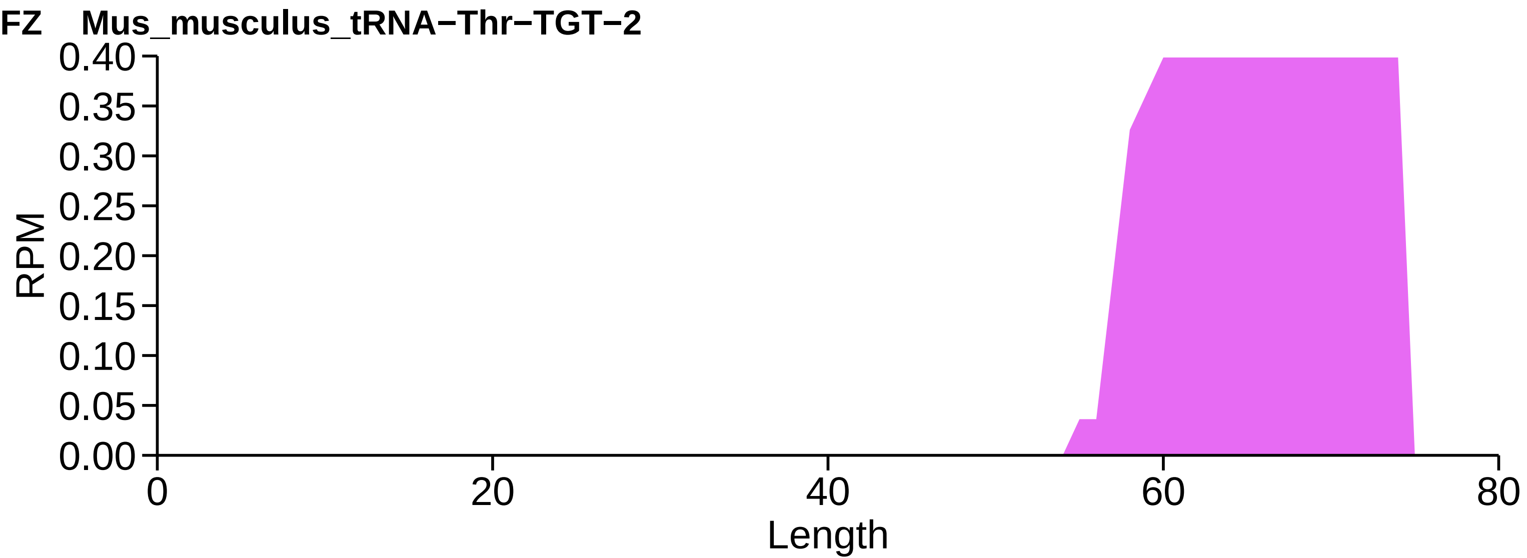
FZ Mus_musculus_tRNA−Thr−TGT−2
RPM
0.40
0.35
0.30
0.25
0.20
0.15
0.10
0.05
0.00
0
20
40
60
80
Length

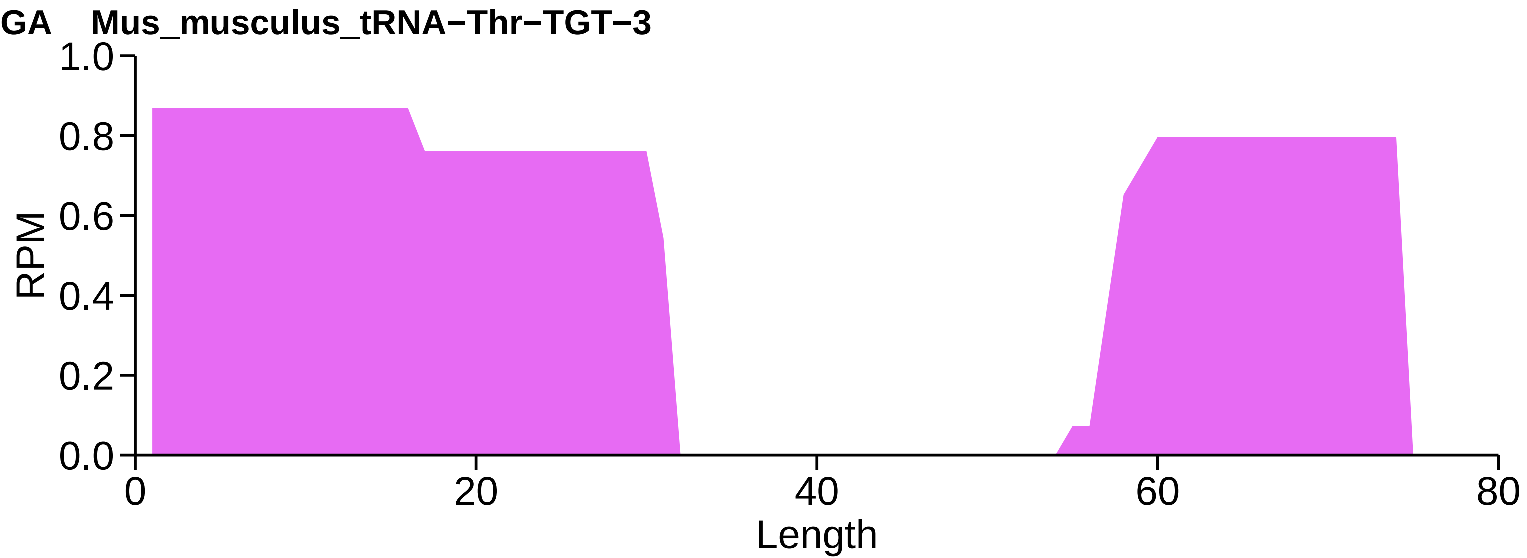

GA Mus_musculus_tRNA−Thr−TGT−3
1.0
0.8
0.6
0.4
0.2
0.0
RPM
0
20
40
60
80
Length

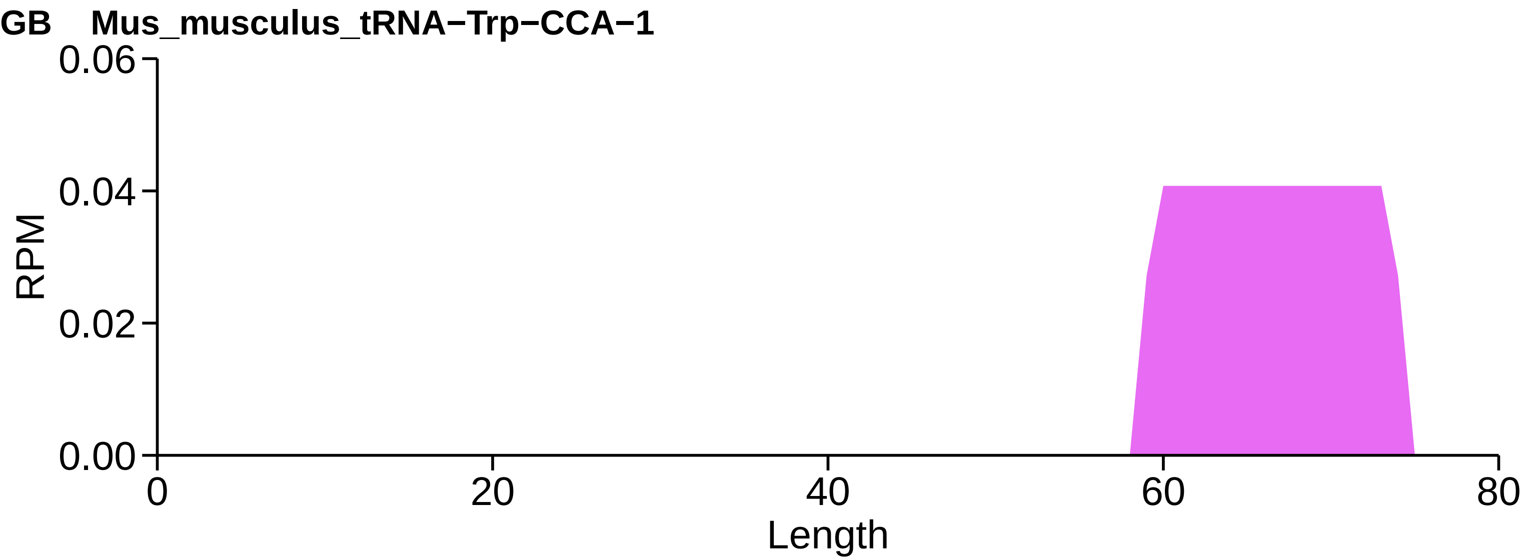
GB Mus_musculus_tRNA−Trp−CCA−1
0.06
0.04
0.02
0.00
RPM
0
20
40
60
80
Length

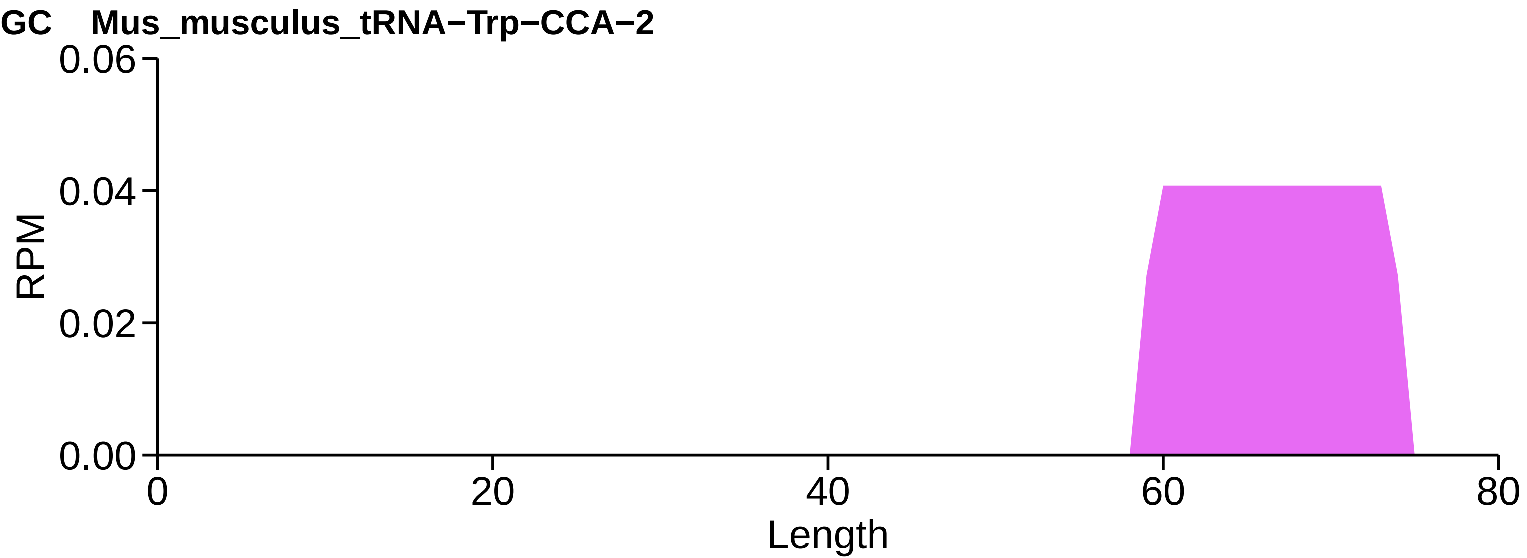

GC Mus_musculus_tRNA−Trp−CCA−2
0.06
0.04
0.02
0.00
RPM
0
20
40
60
80
Length

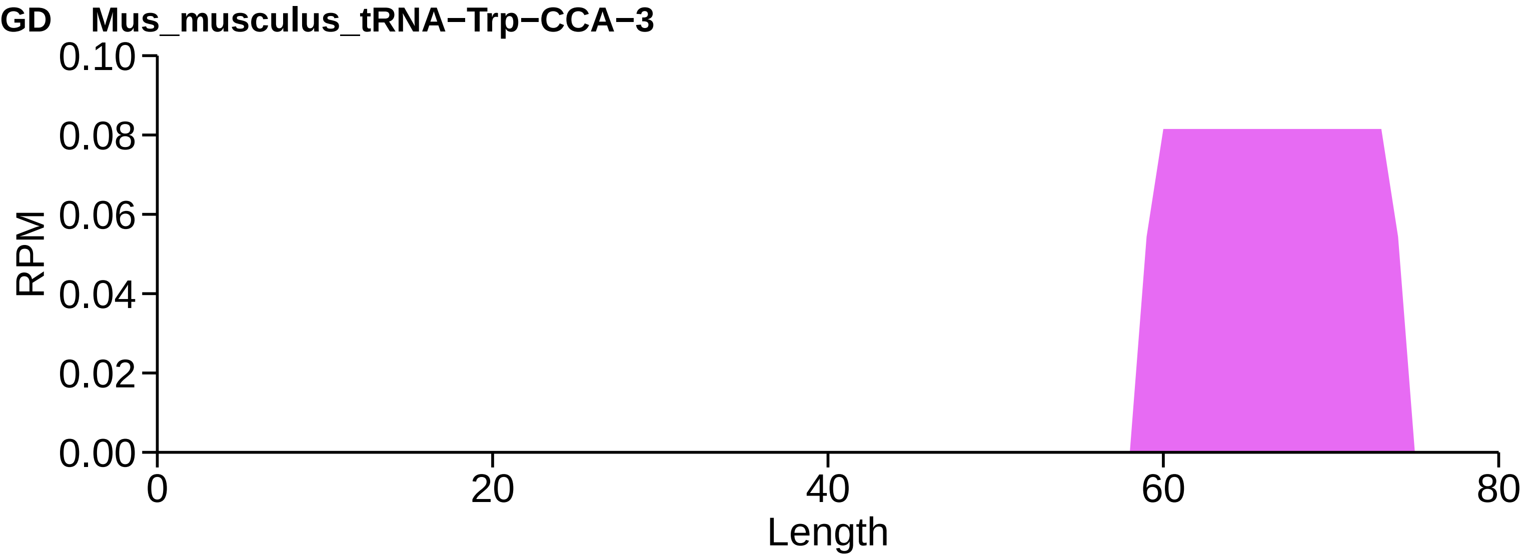
GD Mus_musculus_tRNA−Trp−CCA−3
0.10
0.08
0.06
0.04
0.02
0.00
RPM
0
20
40
60
80
Length

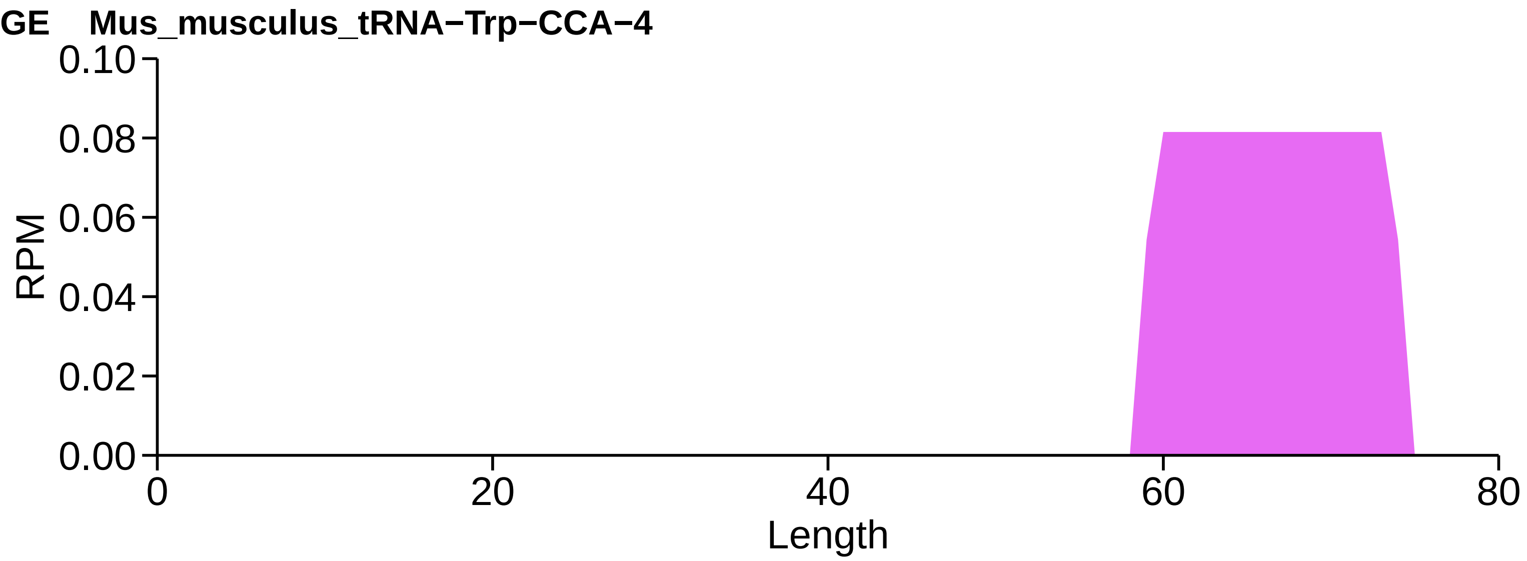
GE Mus_musculus_tRNA−Trp−CCA−4
0.10
0.08
0.06
0.04
0.02
0.00
RPM
0
20
40
60
80
Length

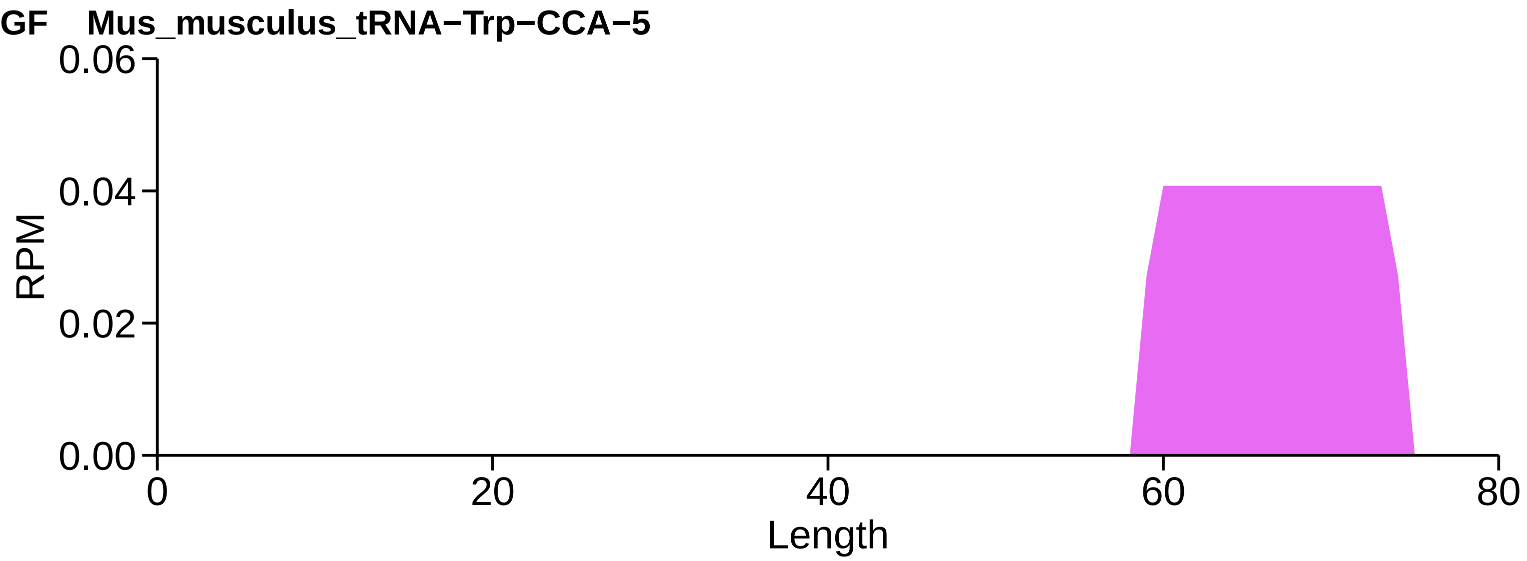
GF Mus_musculus_tRNA−Trp−CCA−5
RPM
0.06
0.04
0.02
0.00
0
20
40
60
80
Length

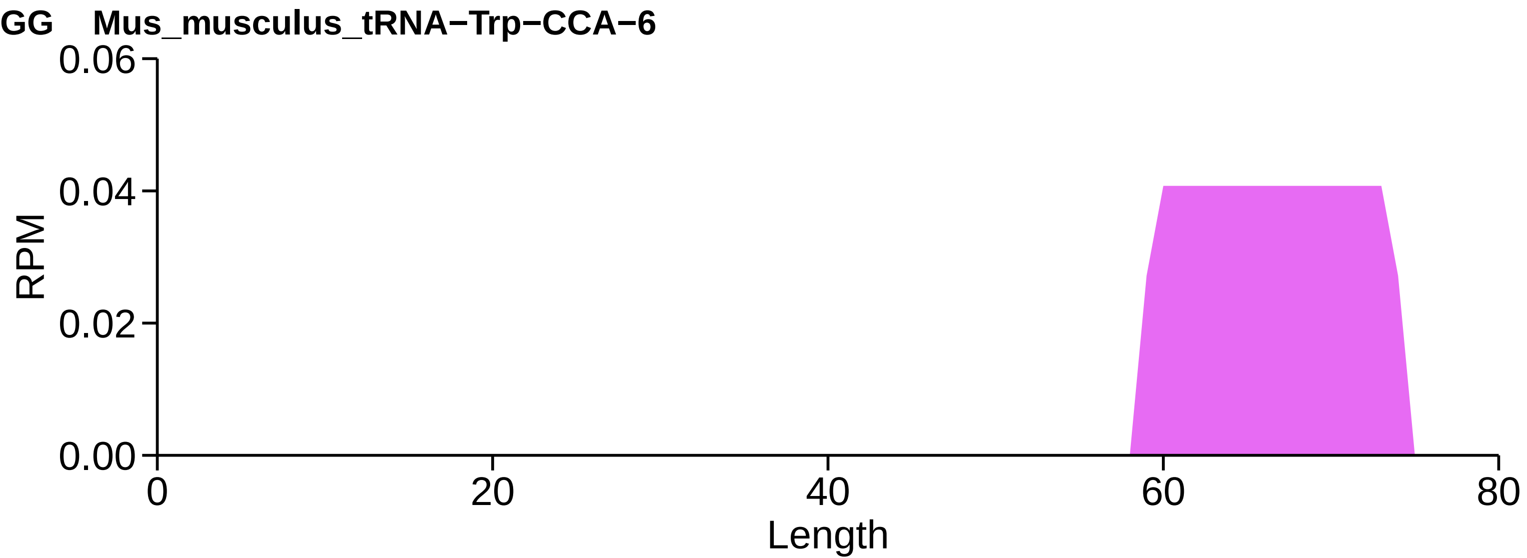
GG Mus_musculus_tRNA−Trp−CCA−6
0.06
0.04
0.02
0.00
RPM
0
20
40
60
80
Length

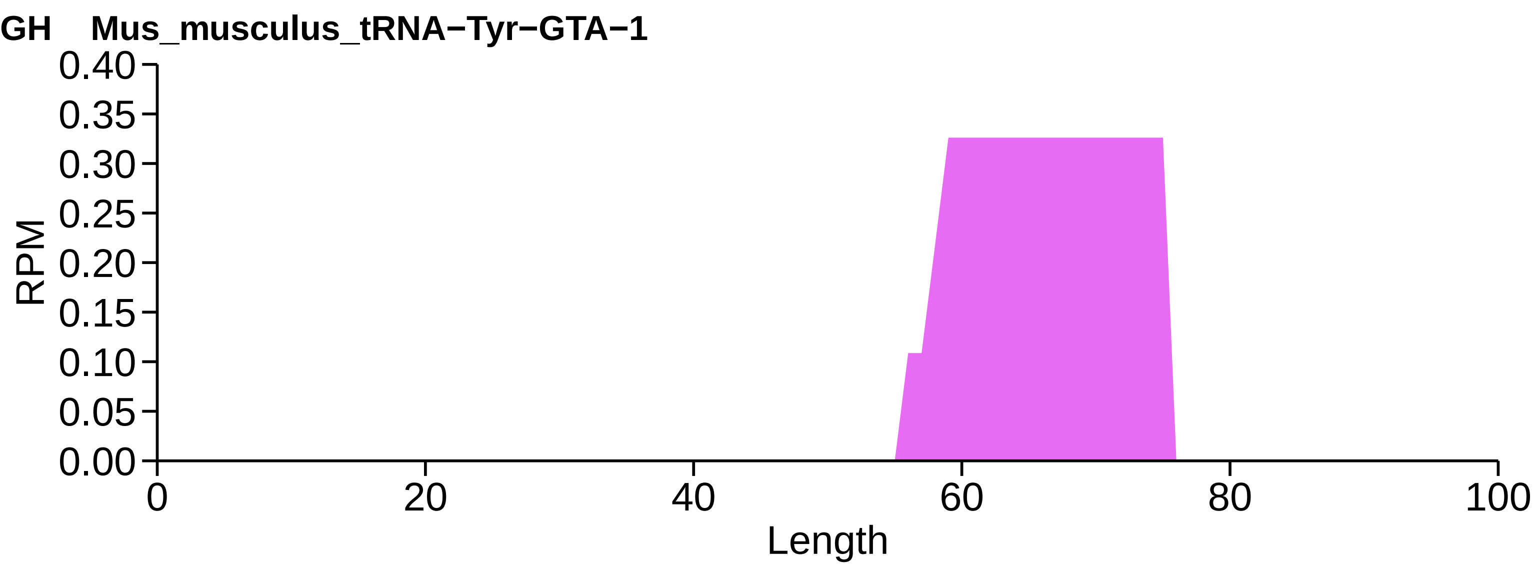
GH Mus_musculus_tRNA−Tyr−GTA−1
RPM
0.40
0.35
0.30
0.25
0.20
0.15
0.10
0.05
0.00
0
20
40
60
80
100
Length

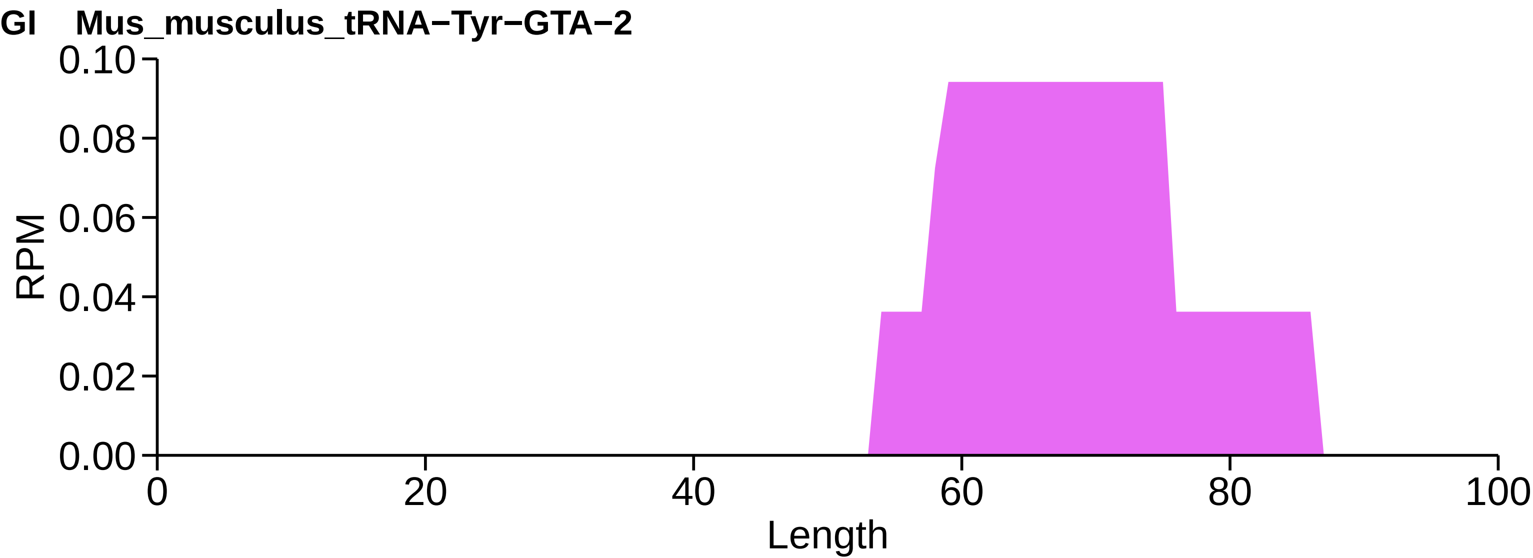

GI Mus_musculus_tRNA−Tyr−GTA−2
0.10
0.08
0.06
0.04
0.02
0.00
RPM
0
20
40
60
80
100
Length

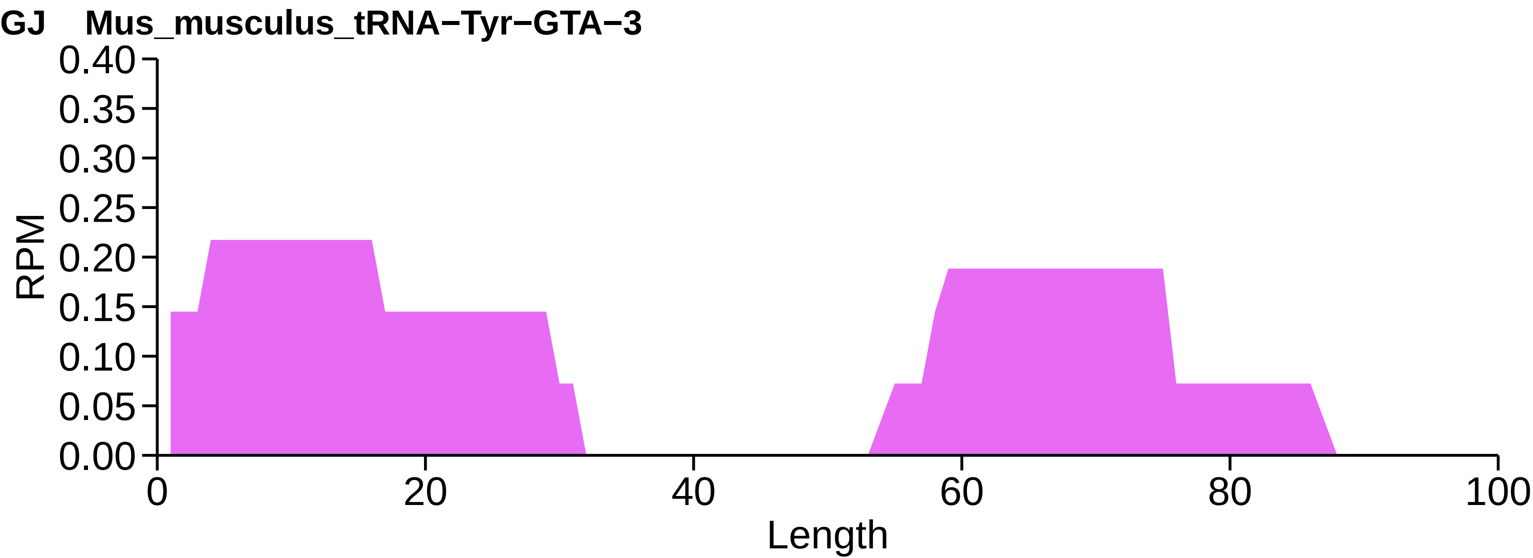
GJ Mus_musculus_tRNA−Tyr−GTA−3
RPM
0.40
0.35
0.30
0.25
0.20
0.15
0.10
0.05
0.00
0
20
40
60
80
100
Length

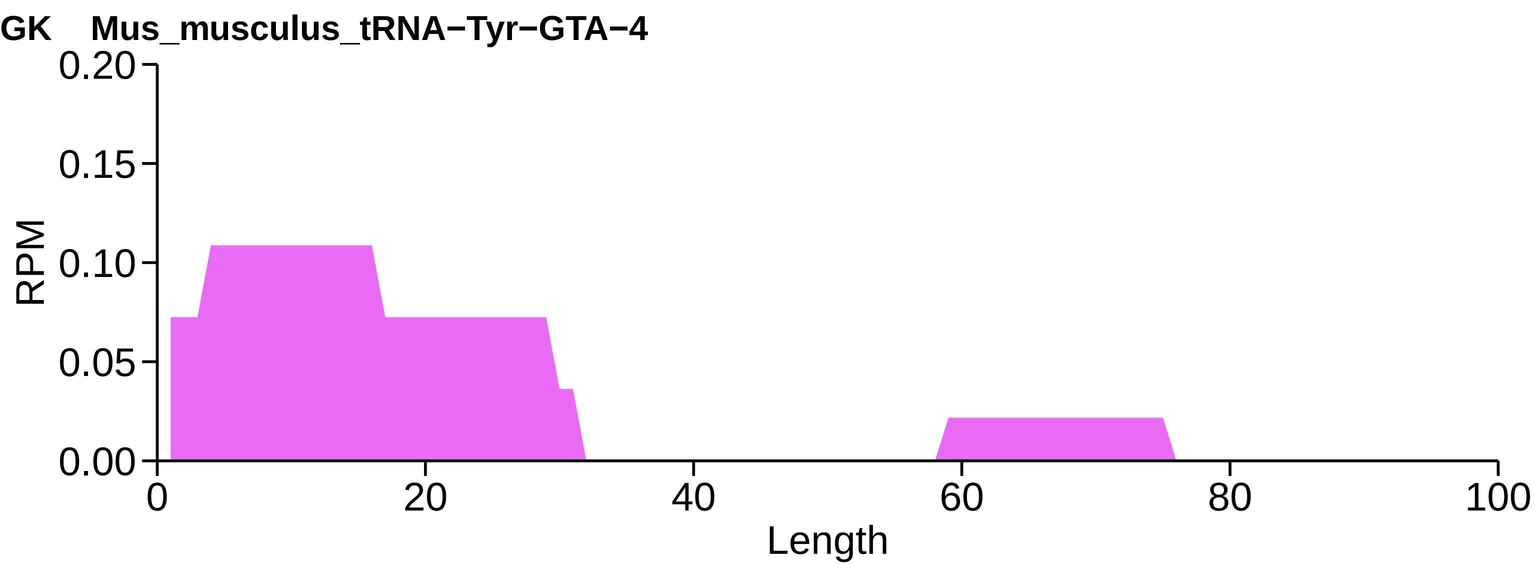

GK Mus_musculus_tRNA−Tyr−GTA−4
RPM
0.00
0.05
0.10
0.15
0.20
0
20
40
60
80
100
Length

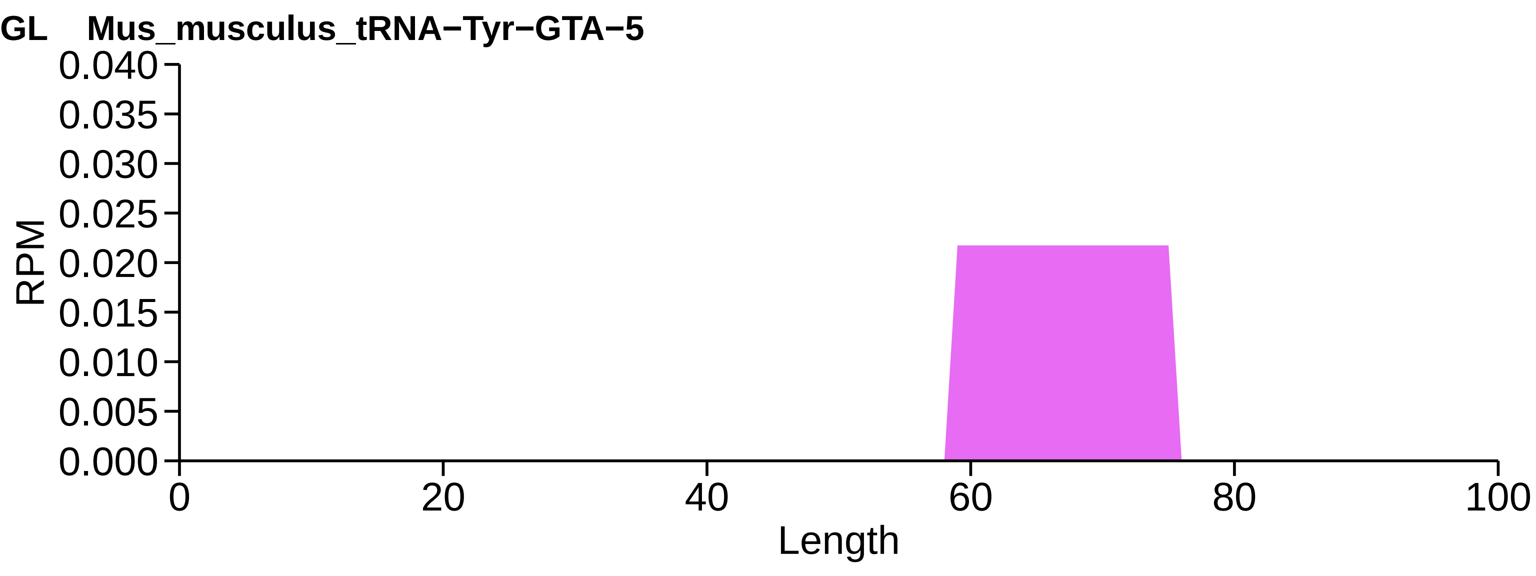
GL Mus_musculus_tRNA−Tyr−GTA−5
RPM
0.040
0.035
0.030
0.025
0.020
0.015
0.010
0.005
0.000
0
20
40
60
80
100
Length

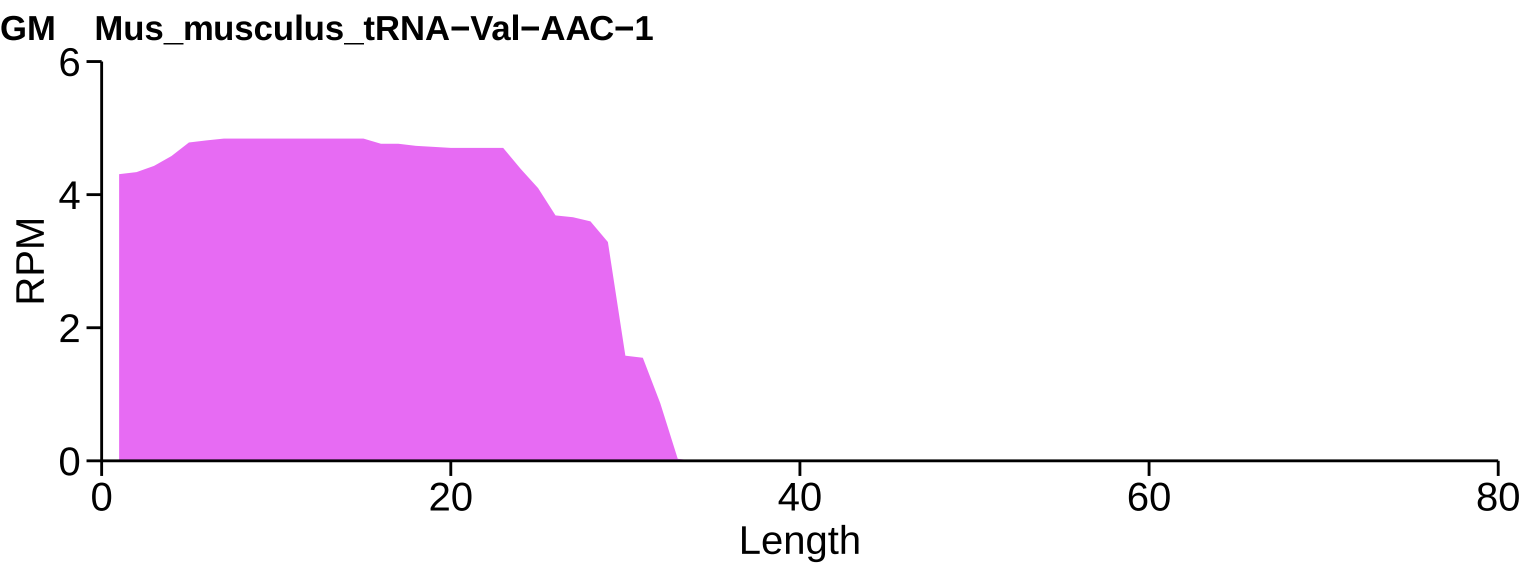
GM Mus_musculus_tRNA−Val−AAC−1
RPM
0
2
4
6
0
20
40
60
80
Length

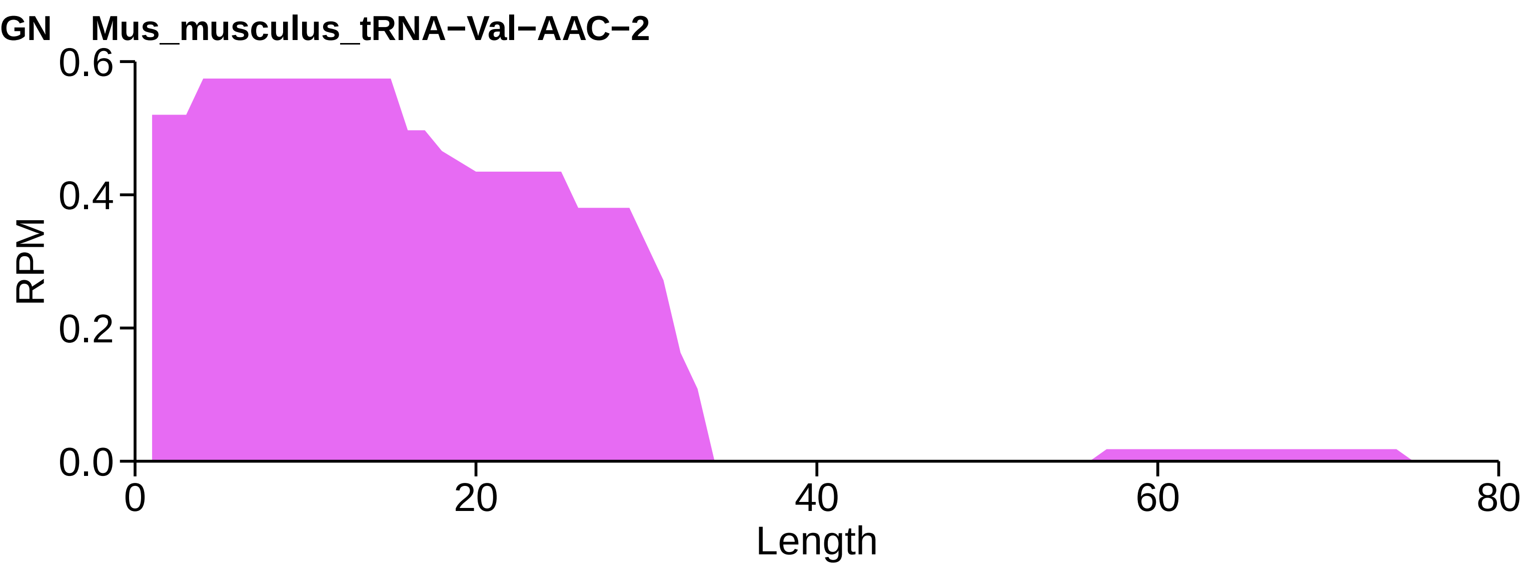

GN Mus_musculus_tRNA−Val−AAC−2
RPM
0.0
0.2
0.4
0.6
0
20
40
60
80
Length

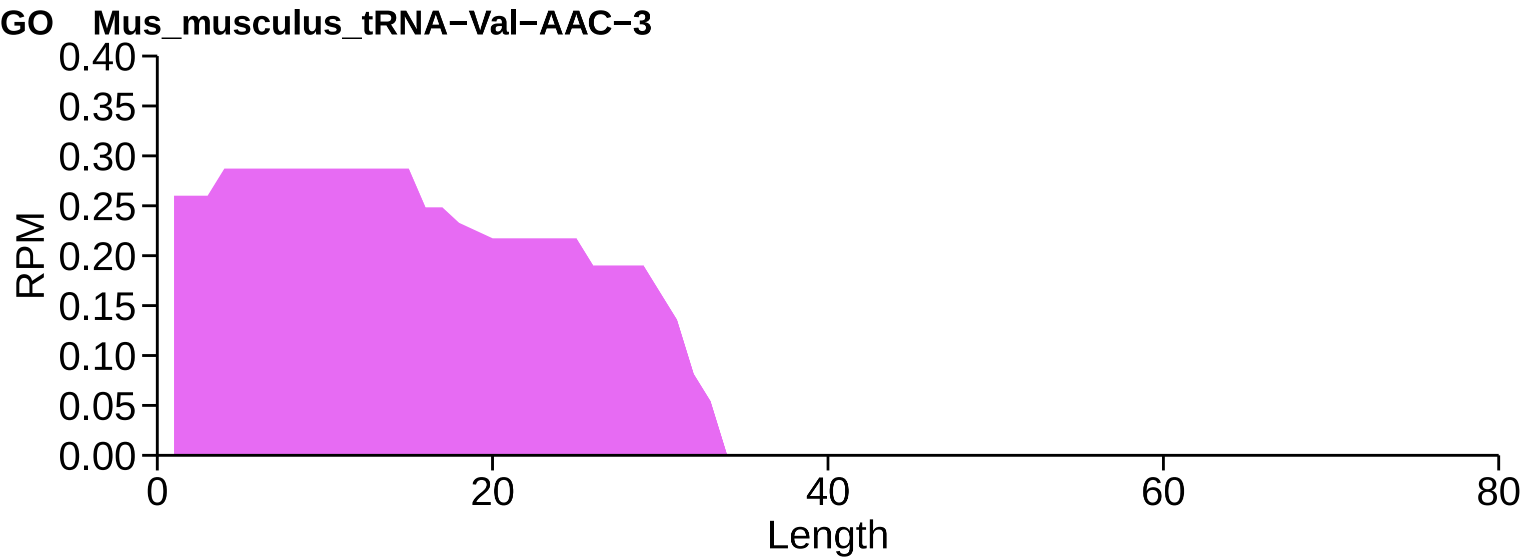
GO Mus_musculus_tRNA−Val−AAC−3
0.40
0.35
0.30
0.25
0.20
0.15
0.10
0.05
0.00
RPM
0
20
40
60
80
Length

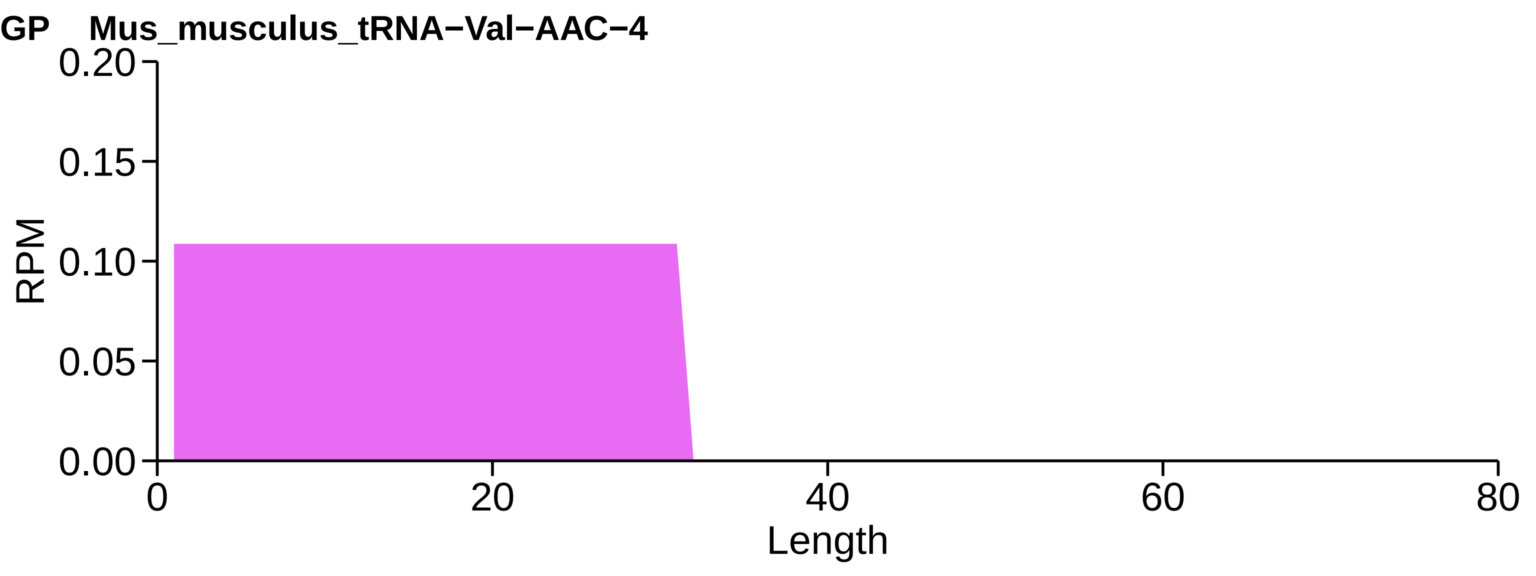
GP Mus_musculus_tRNA−Val−AAC−4
0.20
0.15
0.10
0.05
0.00
RPM
0
20
40
60
80
Length

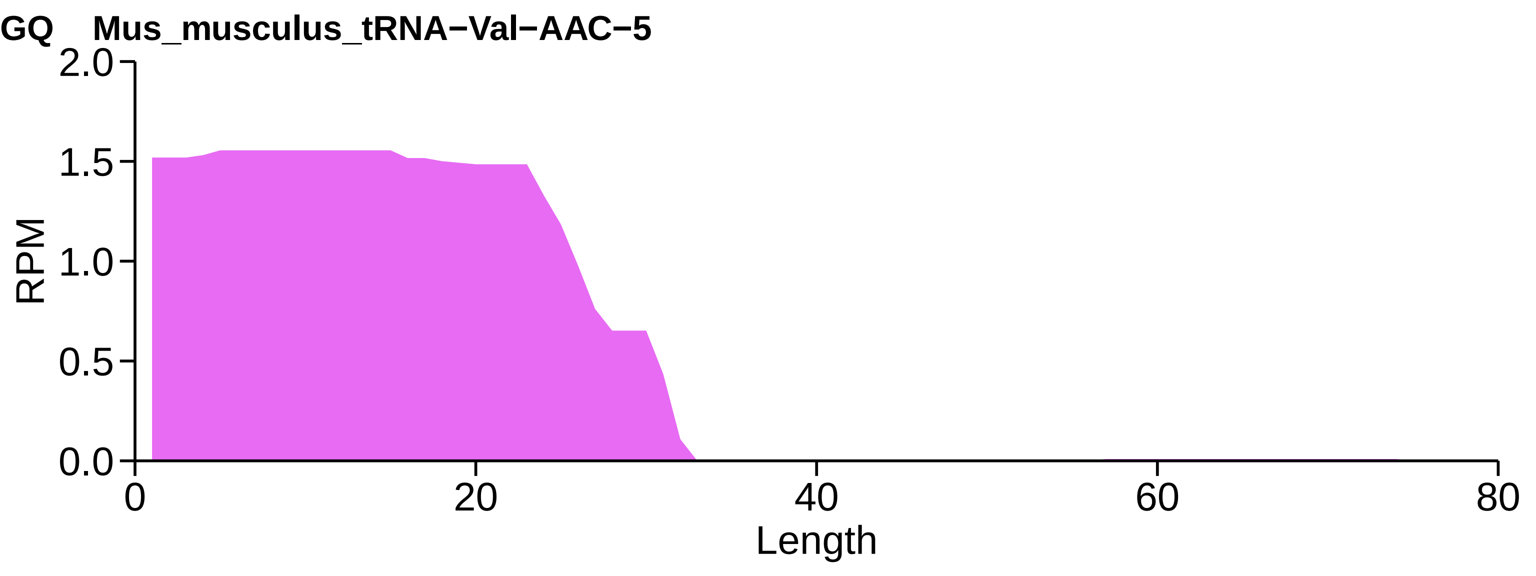
GQ Mus_musculus_tRNA−Val−AAC−5
2.0
1.5
1.0
0.5
0.0
RPM
0
20
40
60
80
Length

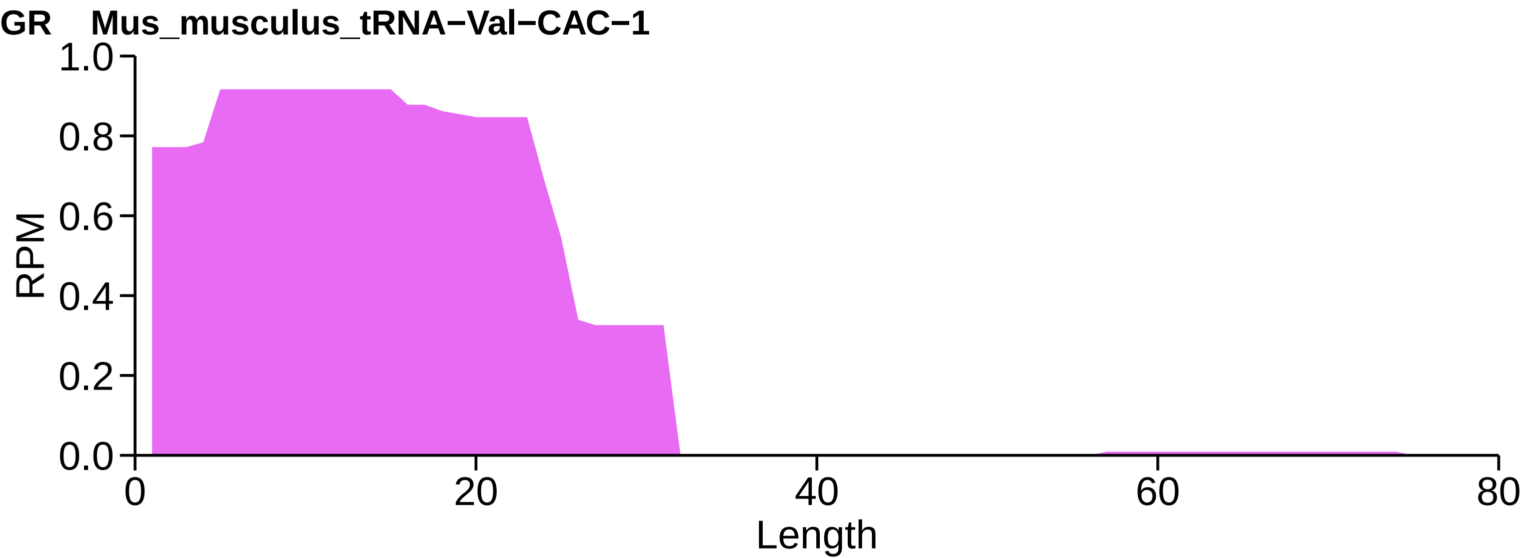
GR Mus_musculus_tRNA−Val−CAC−1
RPM
1.0
0.8
0.6
0.4
0.2
0.0
0
20
40
60
80
Length

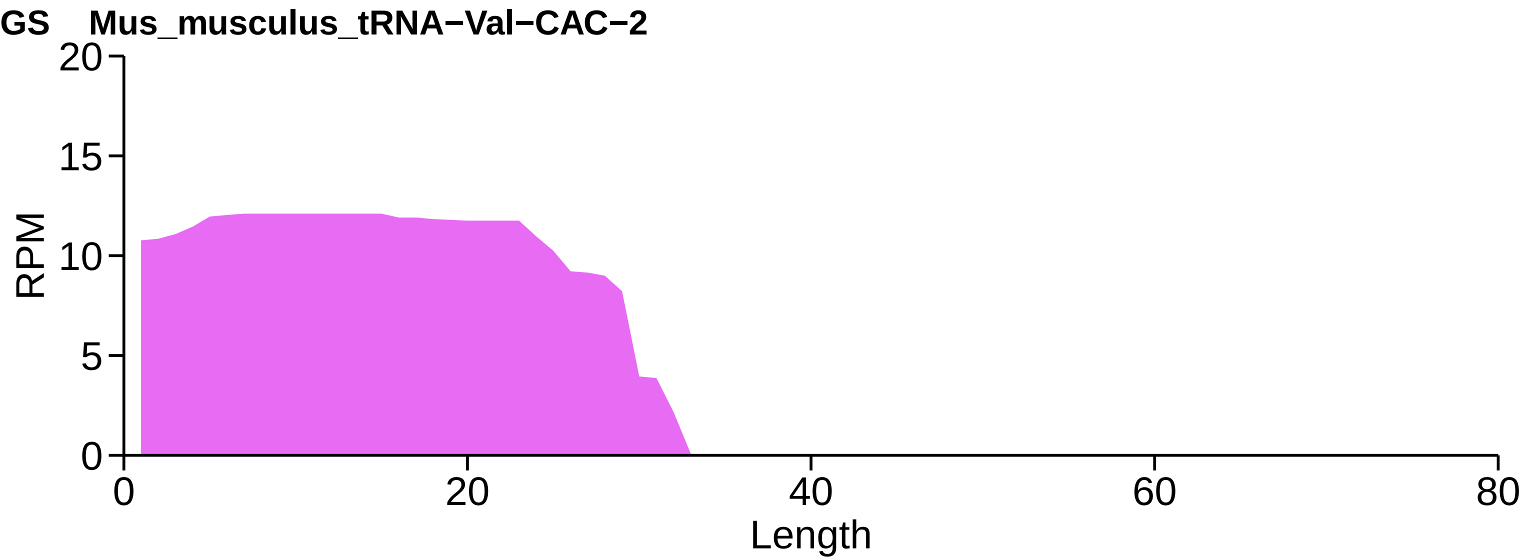
GS Mus_musculus_tRNA−Val−CAC−2
RPM
0
5
10
15
20
Length
0
20
40
60
80

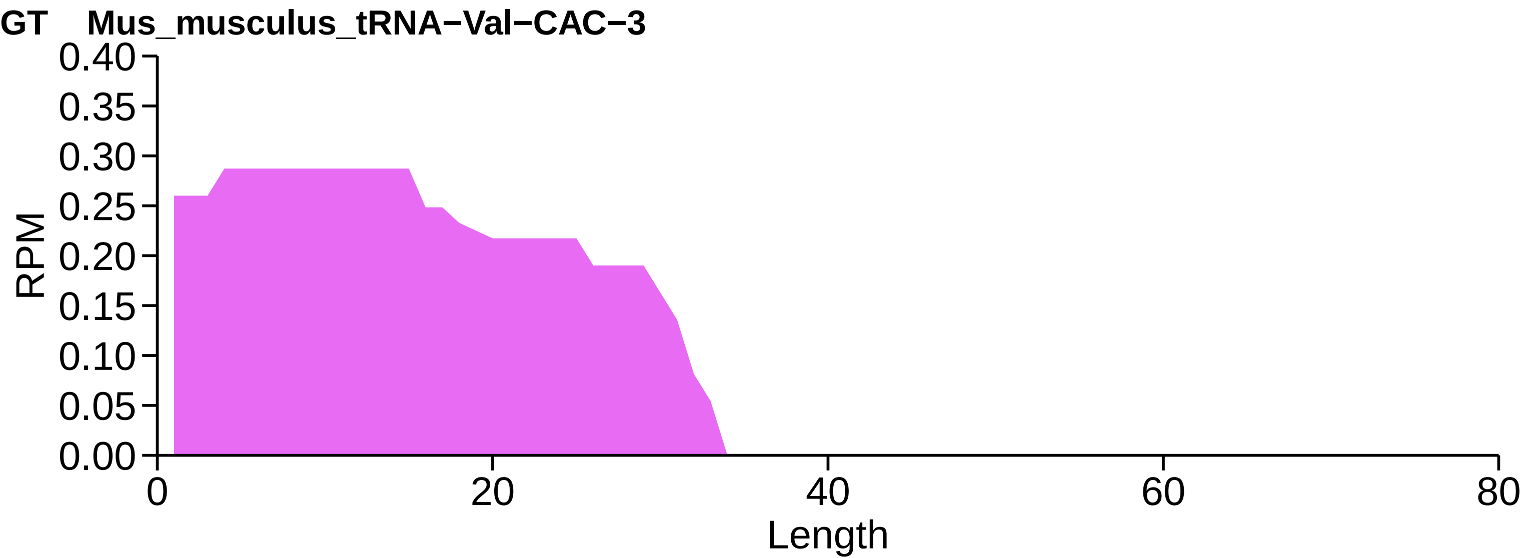
GT Mus_musculus_tRNA−Val−CAC−3
RPM
0.00
0.05
0.10
0.15
0.20
0.25
0.30
0.35
0.40
0
20
40
60
80
Length

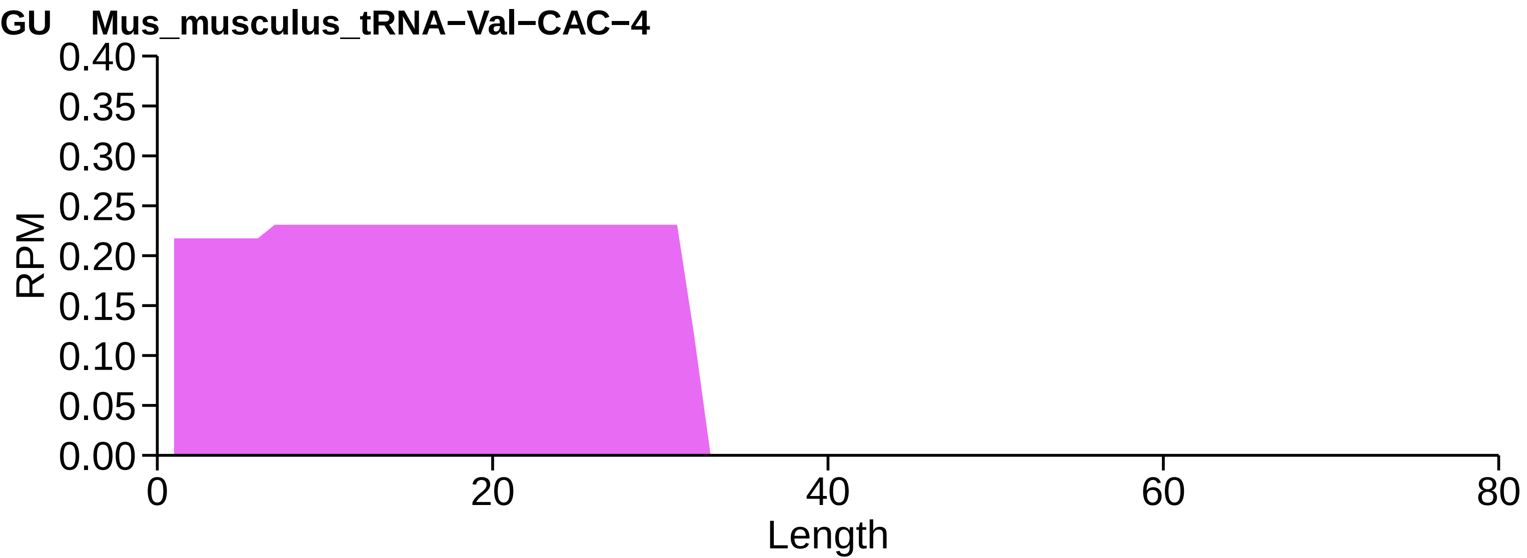
GU Mus_musculus_tRNA−Val−CAC−4
RPM
0.40
0.35
0.30
0.25
0.20
0.15
0.10
0.05
0.00
0
20
40
60
80
Length

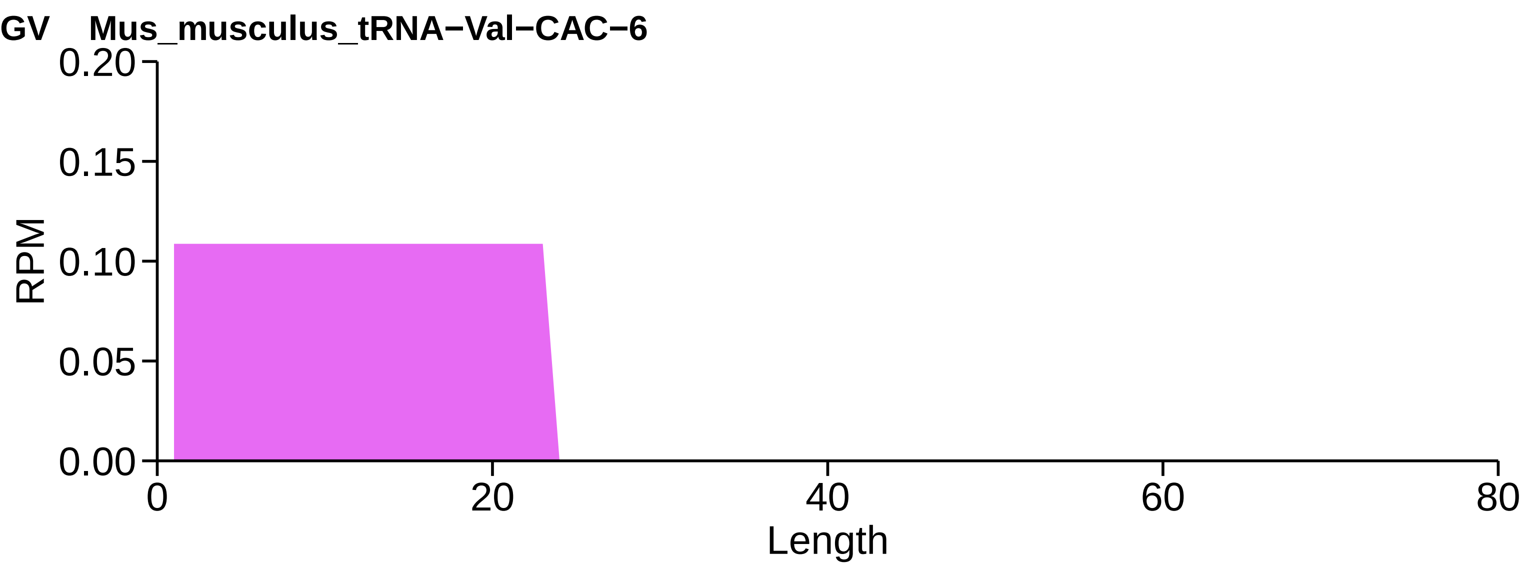
GV Mus_musculus_tRNA−Val−CAC−6
0.20
0.15
0.10
0.05
0.00
RPM
0
20
40
60
80
Length

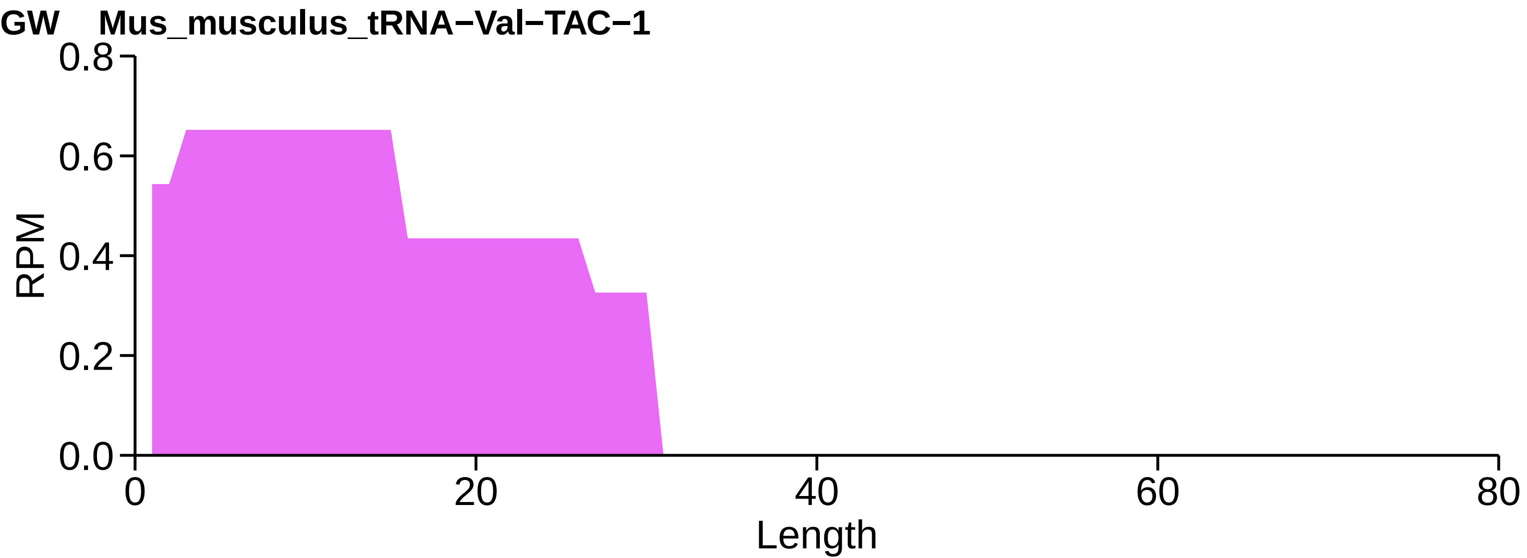

GW Mus_musculus_tRNA−Val−TAC−1
RPM
0.0
0.2
0.4
0.6
0.8
0
20
40
60
80
Length

GX Mus_musculus_tRNA−iMet−CAT−1

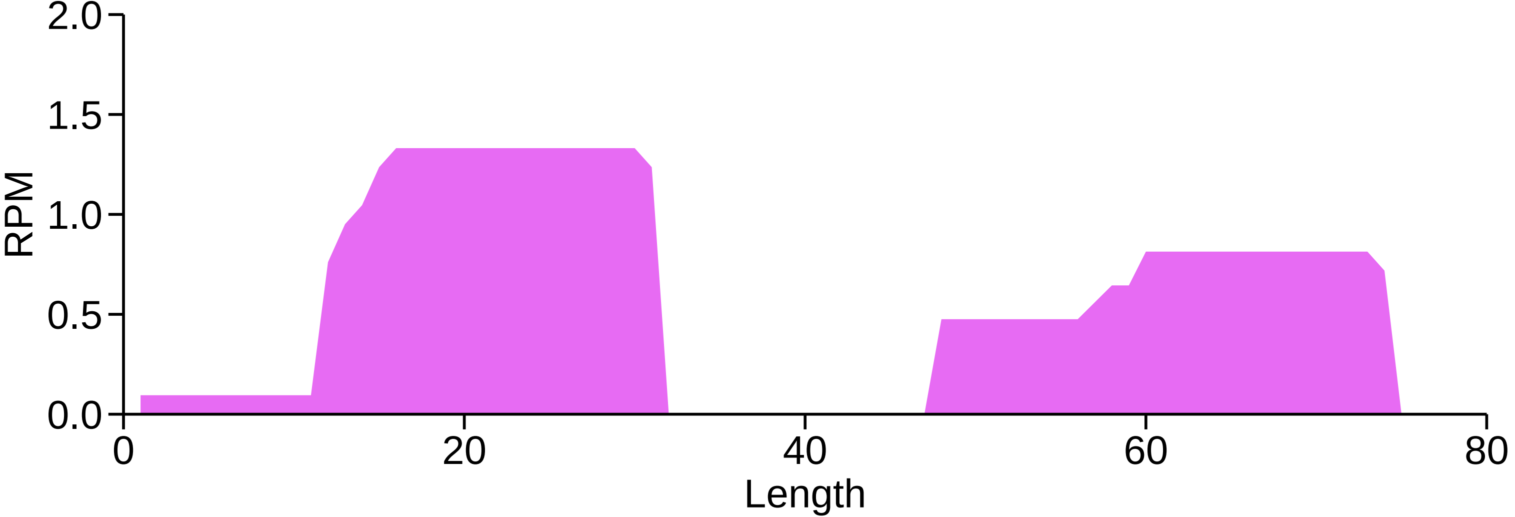

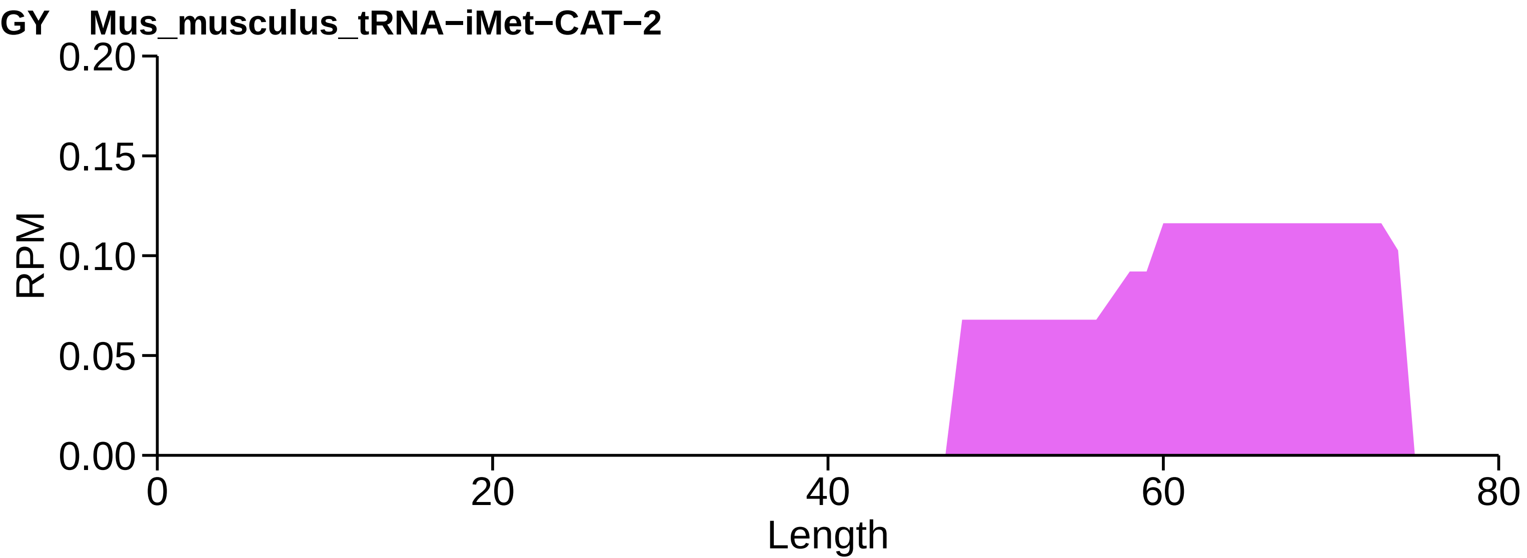
GY Mus_musculus_tRNA−iMet−CAT−2
RPM
0.20
0.15
0.10
0.05
0.00
0
20
40
60
80
Length

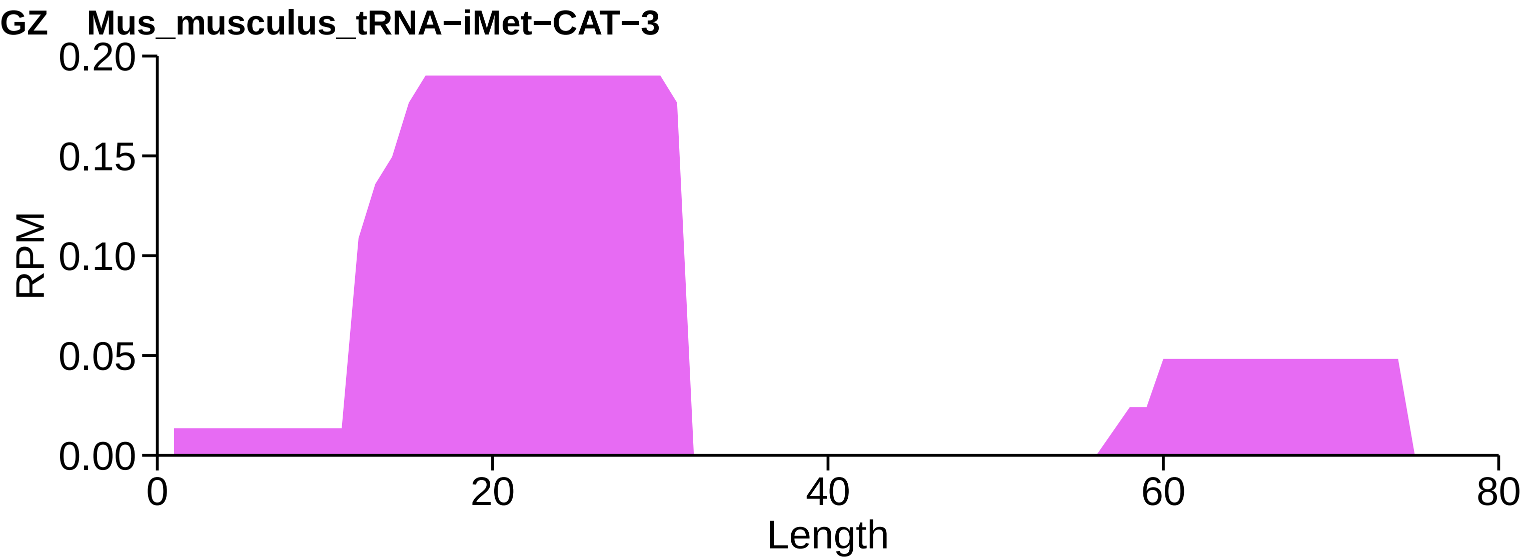
GZ Mus_musculus_tRNA−iMet−CAT−3
RPM
0.20
0.15
0.10
0.05
0.00
0
20
40
60
80
Length
