# Supplementary material for: SPORTS1.0: A Tool for Annotating and Profiling Non-coding RNAs Optimized for rRNA- and tRNA-derived Small RNAs
Source: Genomics Proteomics Bioinformatics. 2018 May 3;16(2):144–51. doi: 10.1016/j.gpb.2018.04.004 (PMC6112344; doi:10.1016/j.gpb.2018.04.004)
Supplement: Supplementary Figure S3 — The mouse intestinal epithelial cell tsRNA mapping results against tRNA loci revealed by SPORTS1.0Mapping result for each annotated tsRNA was provided. [file mmc3.pdf]

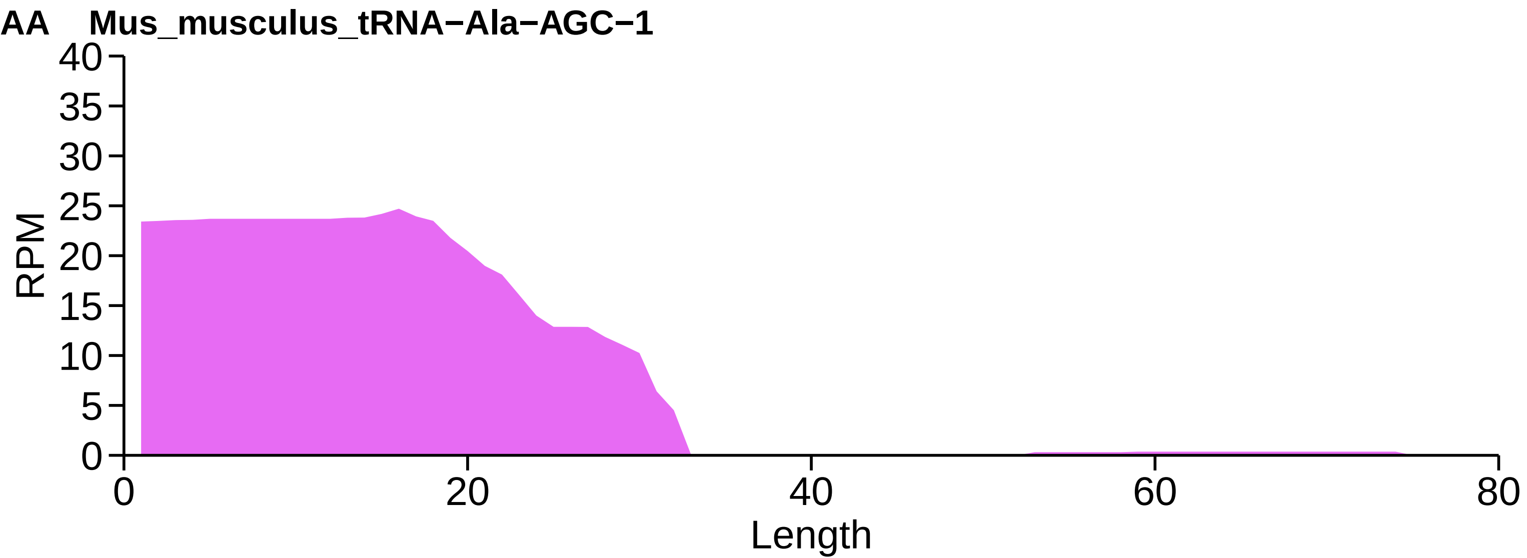
AA Mus_musculus_tRNA−Ala−AGC−1
RPM
Length
0
5
10
15
20
25
30
35
40
0
20
40
60
80

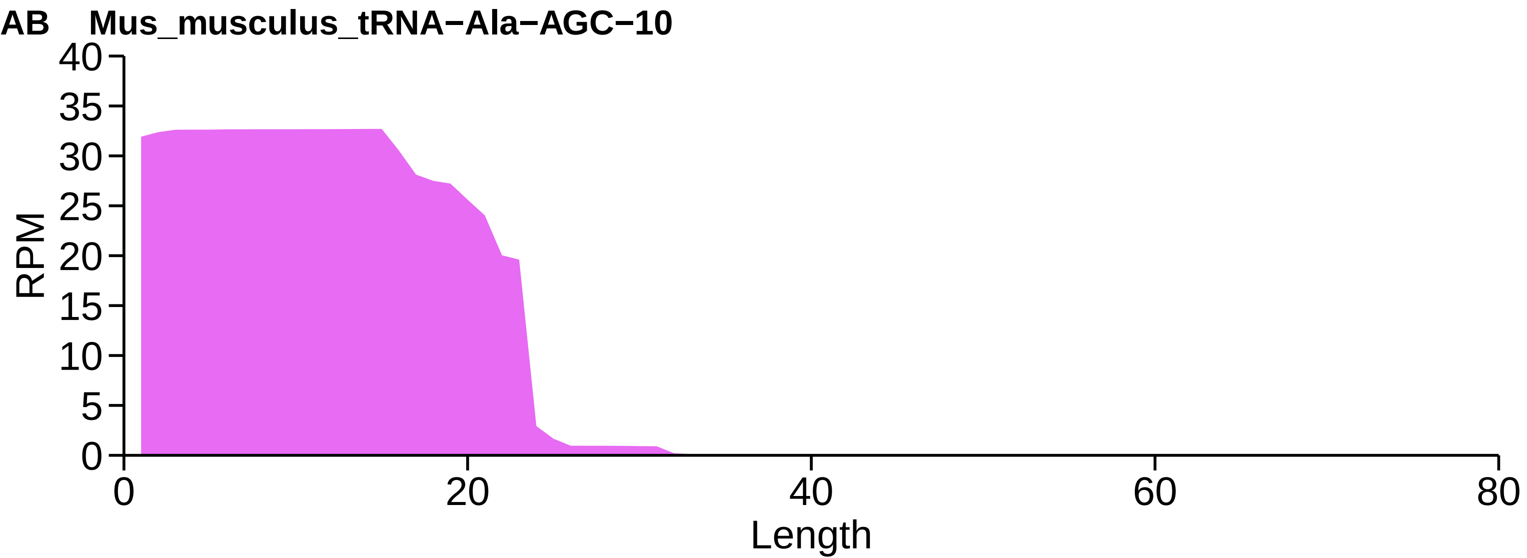
AB Mus_musculus_tRNA−Ala−AGC−10
RPM
40
35
30
25
20
15
10
5
0
0
20
40
60
80
Length

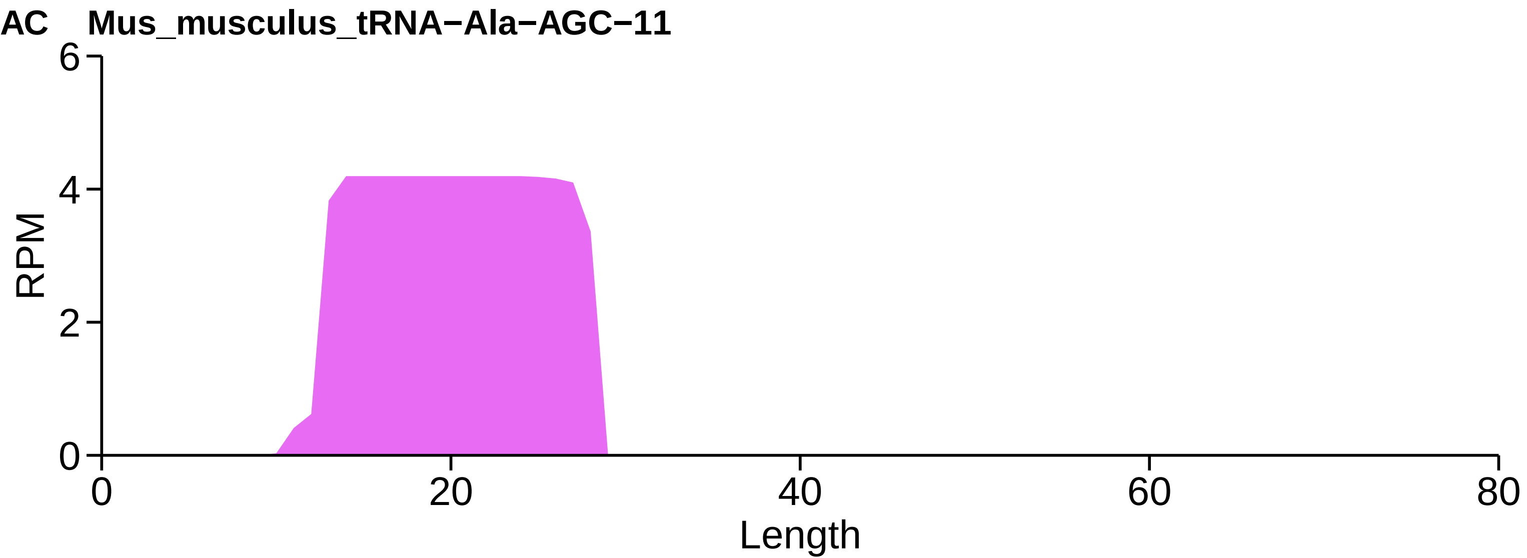

AC Mus_musculus_tRNA−Ala−AGC−11
RPM
Length
0
2
4
6
0
20
40
60
80

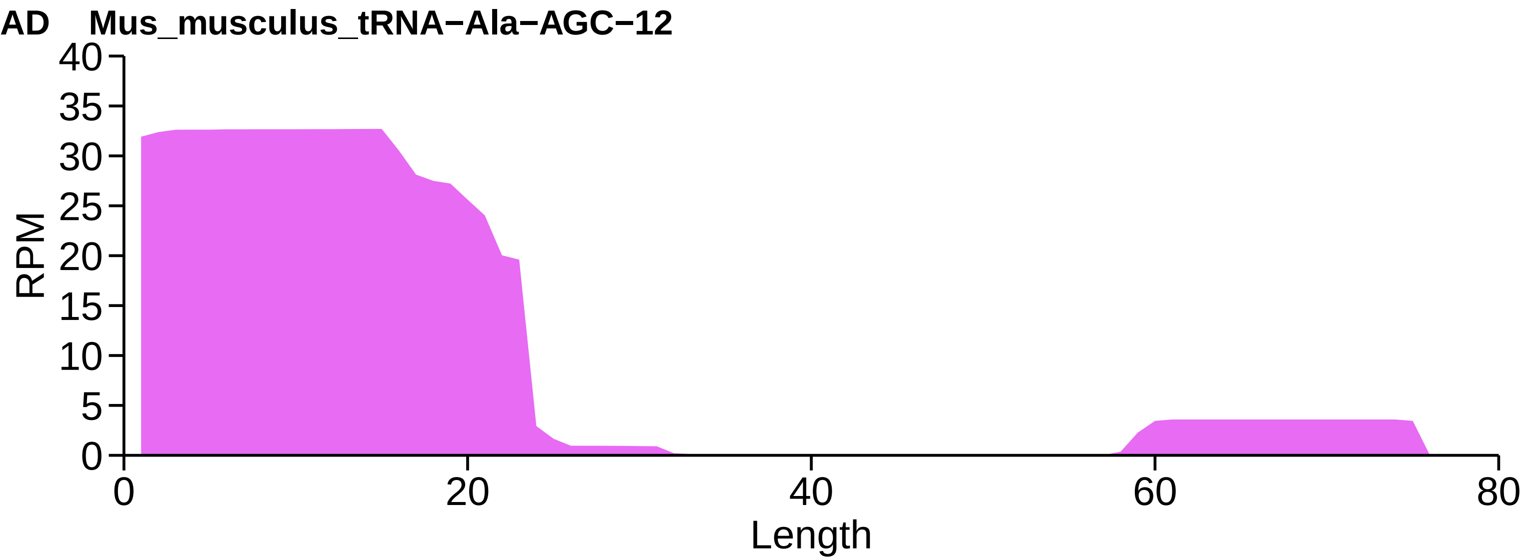
AD Mus_musculus_tRNA−Ala−AGC−12
RPM
40
35
30
25
20
15
10
5
0
0
20
40
60
80
Length

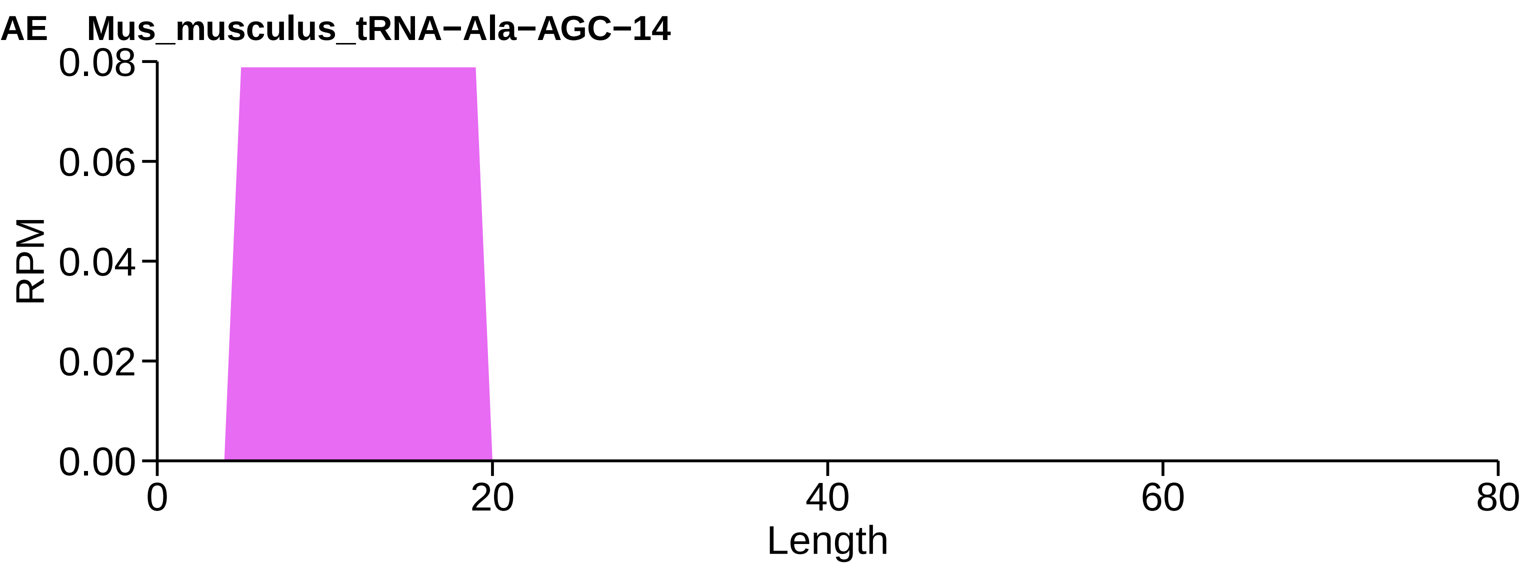
AE Mus_musculus_tRNA−Ala−AGC−14
0.08
0.06
0.04
0.02
0.00
RPM
0
20
40
60
80
Length

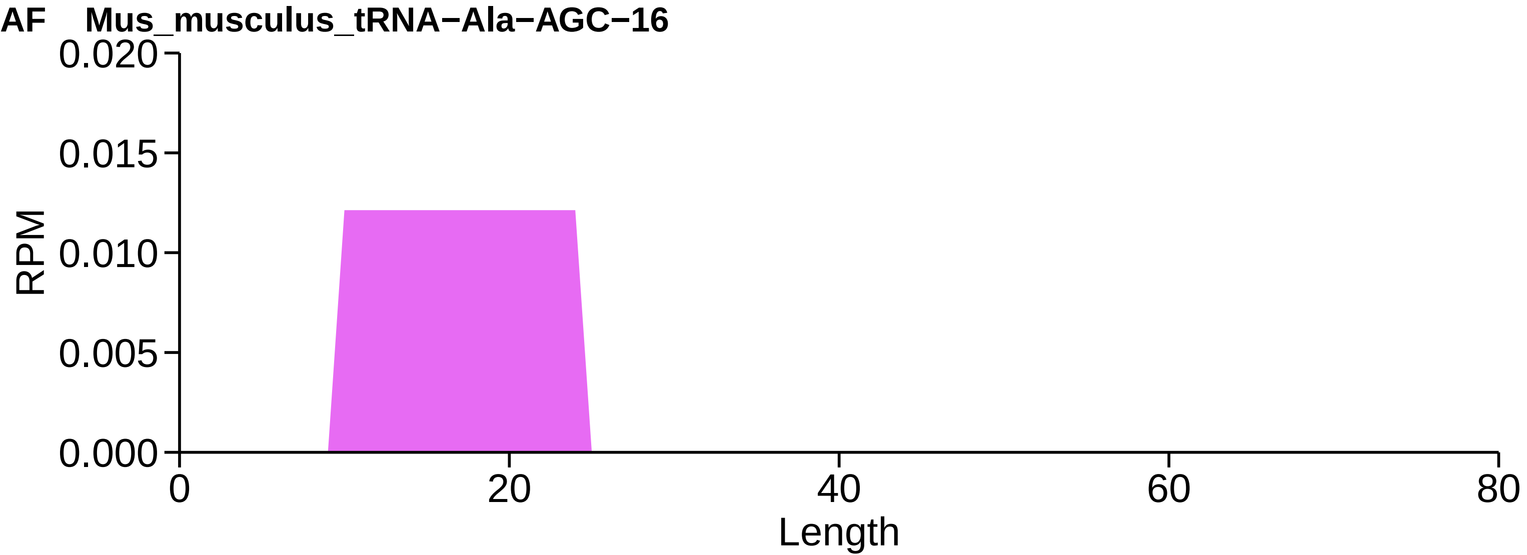

AF Mus_musculus_tRNA−Ala−AGC−16
0.020
0.015
0.010
0.005
0.000
RPM
0
20
40
60
80
Length

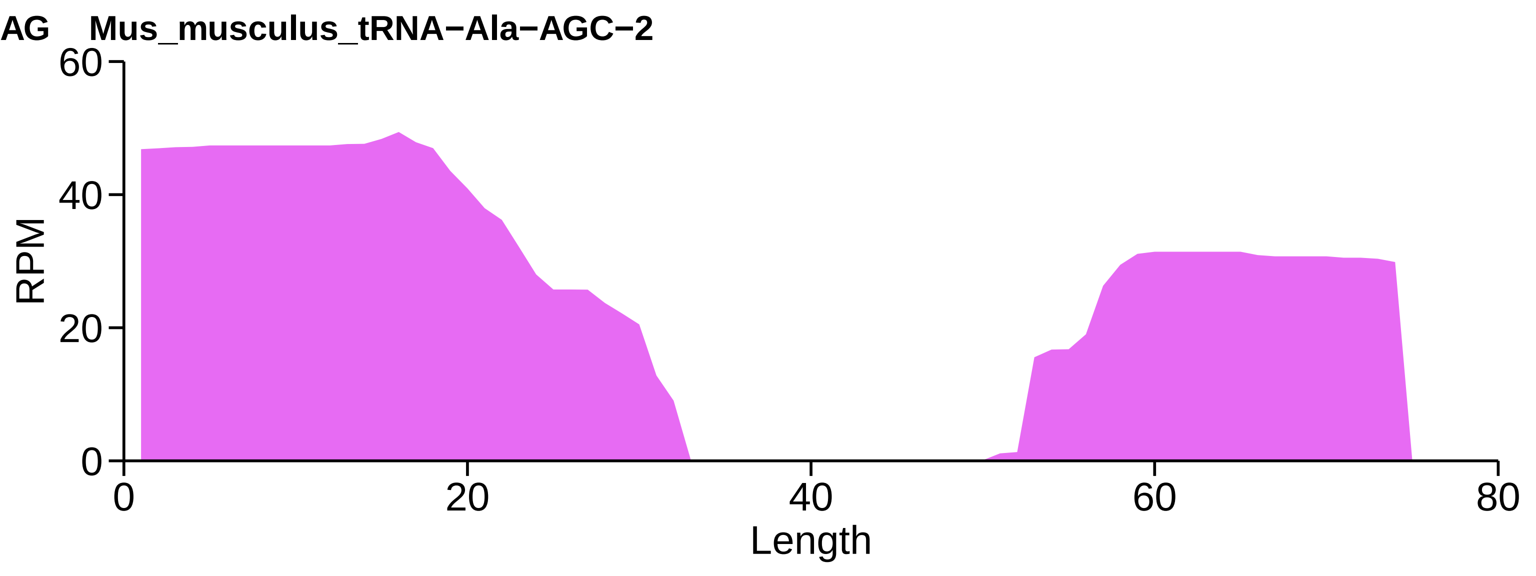
AG Mus_musculus_tRNA−Ala−AGC−2
RPM
0
20
40
60
0
20
40
60
80
Length

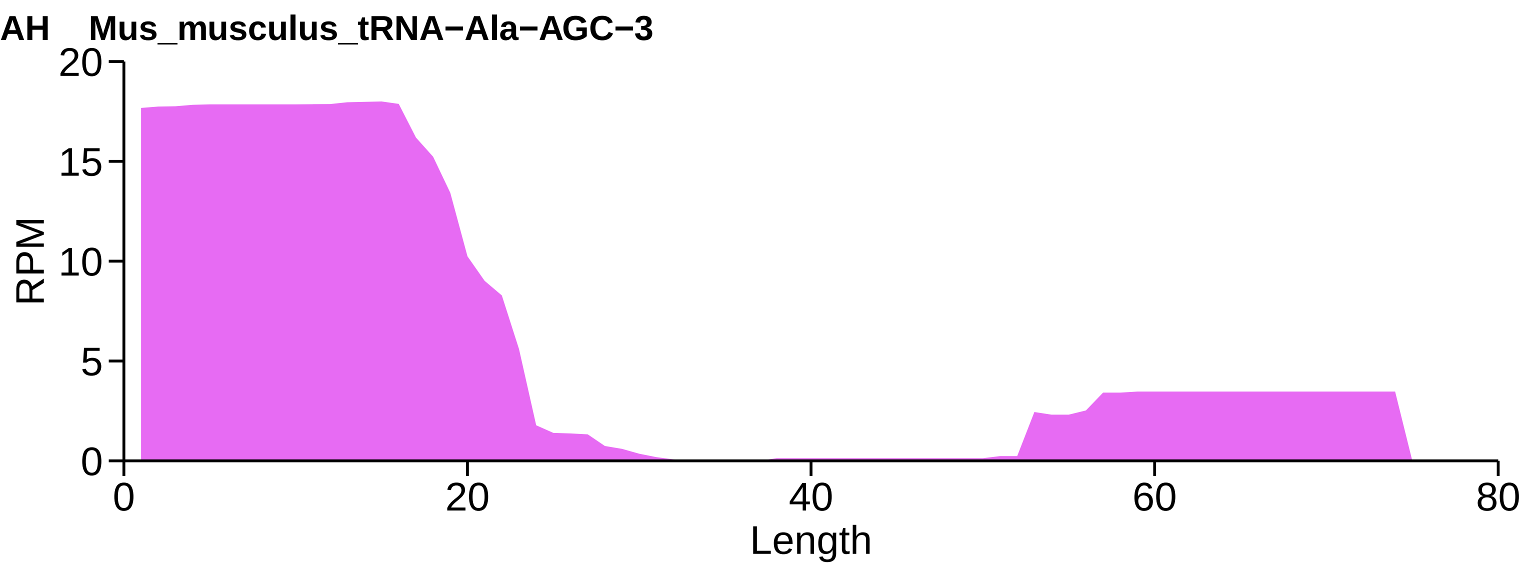

AH Mus_musculus_tRNA−Ala−AGC−3
RPM
0
5
10
15
20
0
20
40
60
80
Length

AI Mus_musculus_tRNA−Ala−AGC−4

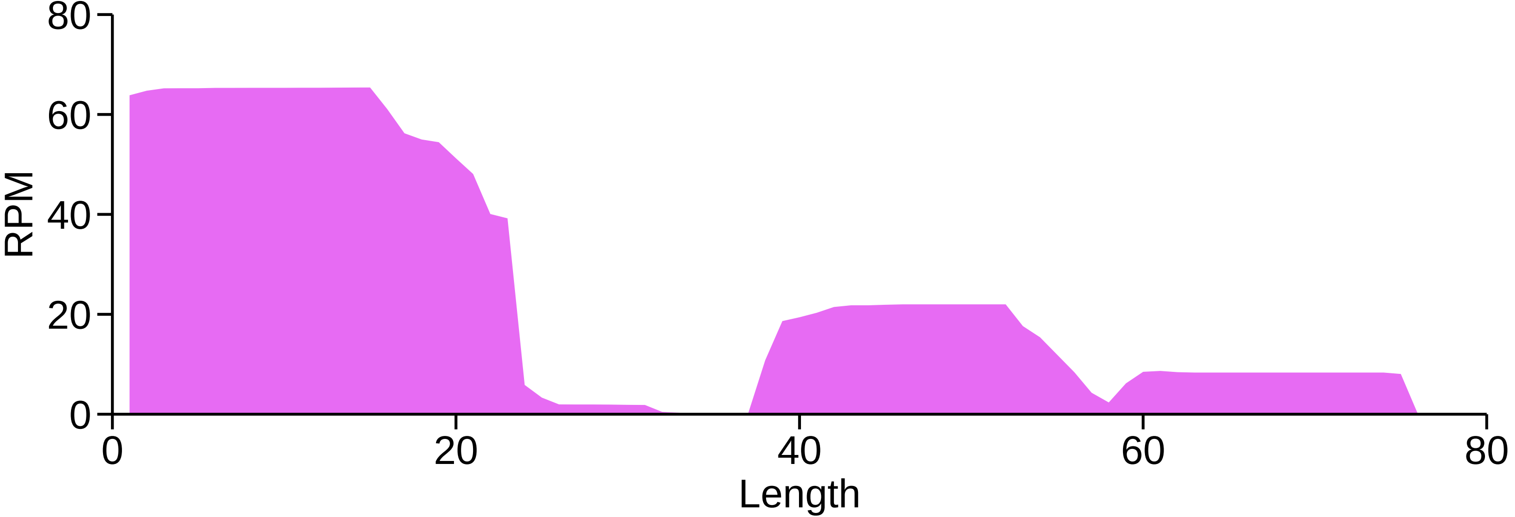

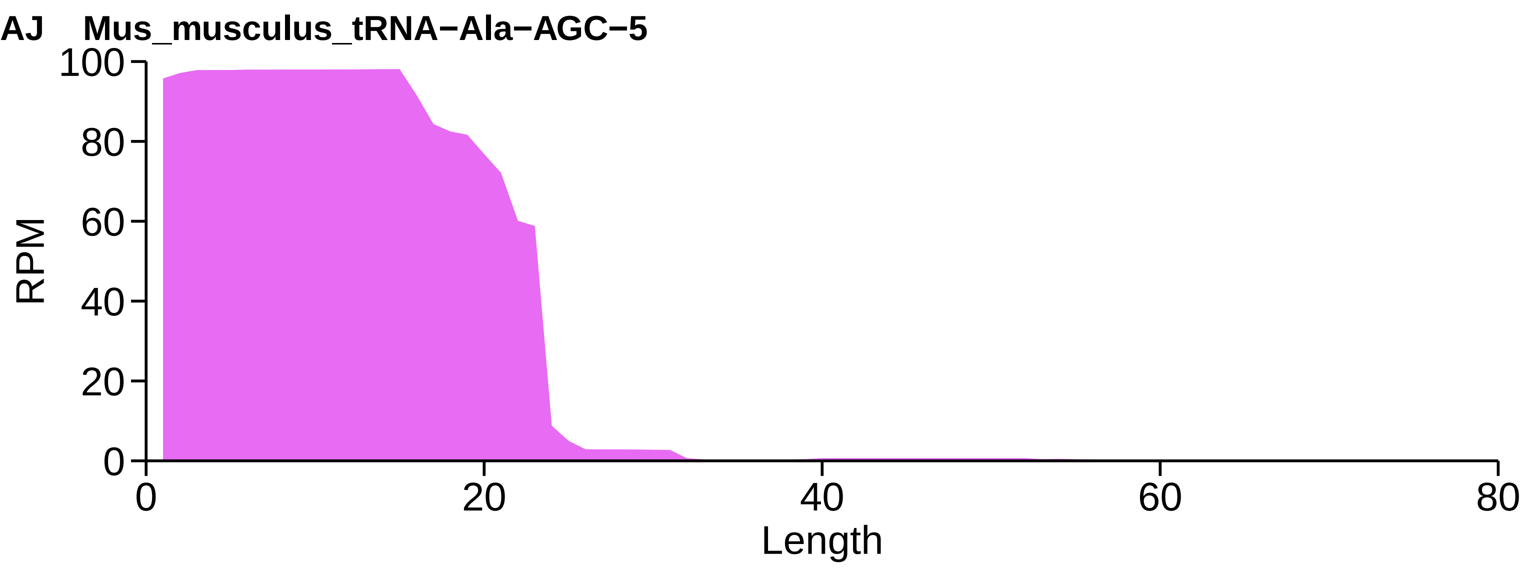
AJ Mus_musculus_tRNA−Ala−AGC−5
RPM
100
80
60
40
20
0
0
20
40
60
80
Length

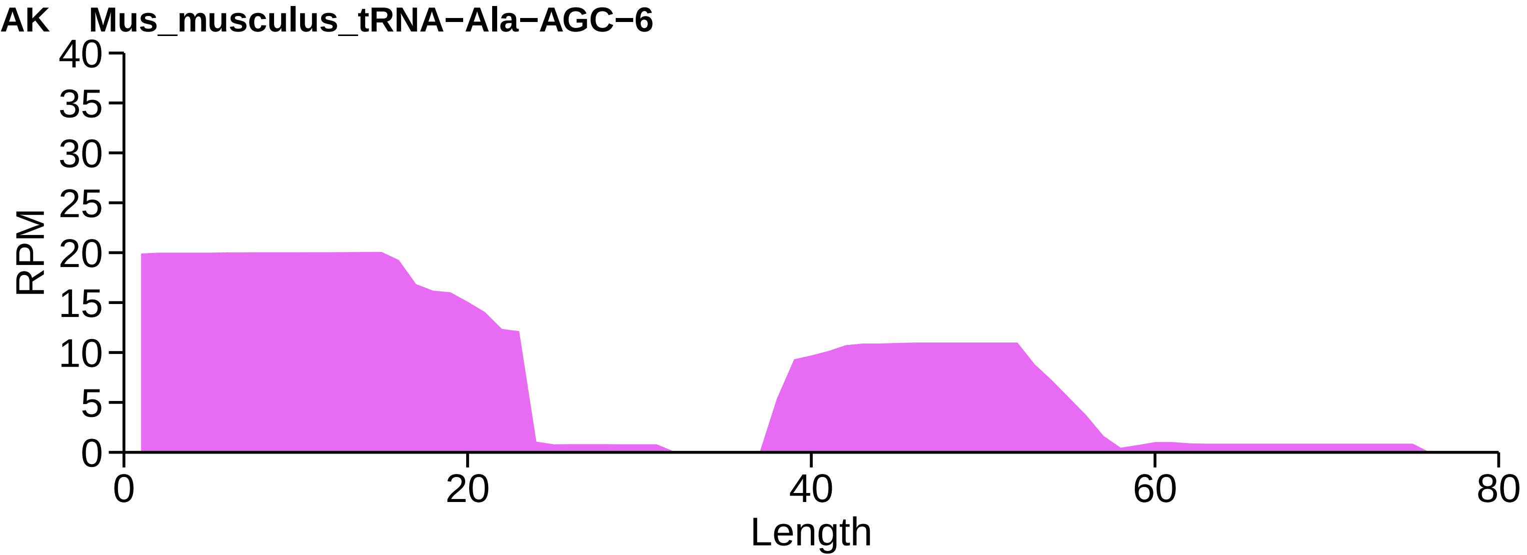

AK Mus_musculus_tRNA−Ala−AGC−6
RPM
Length
0
5
10
15
20
25
30
35
40
0
20
40
60
80

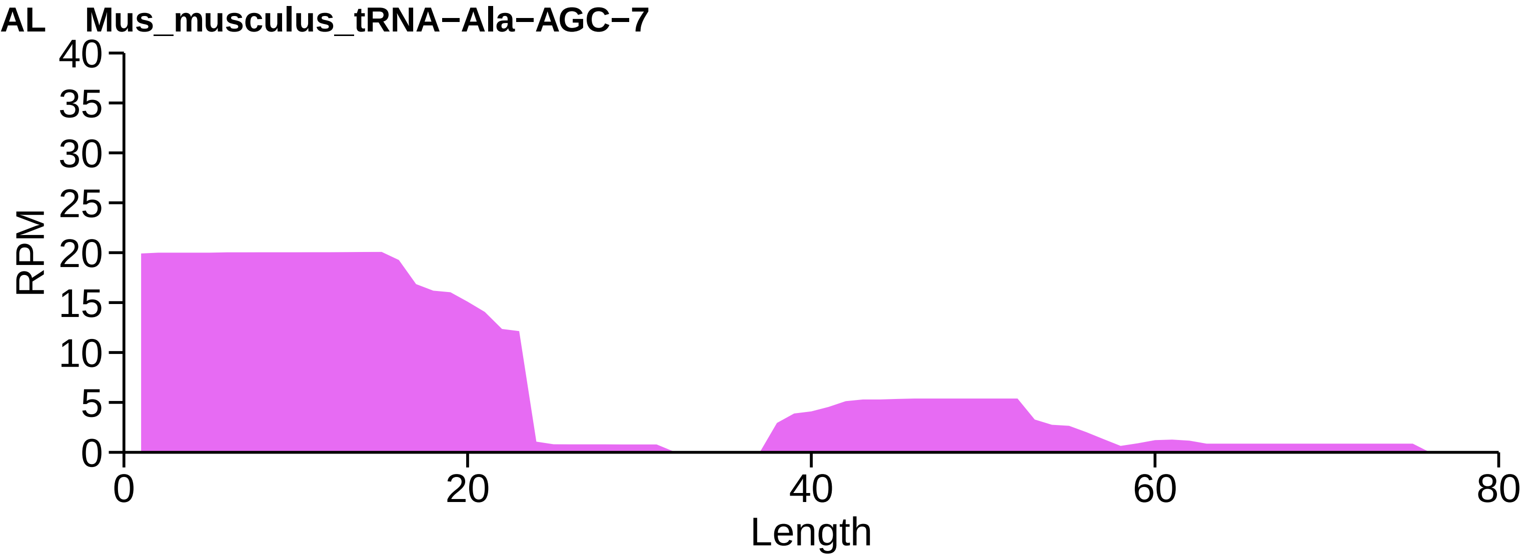
AL Mus_musculus_tRNA−Ala−AGC−7
RPM
40
35
30
25
20
15
10
5
0
0
20
40
60
80
Length

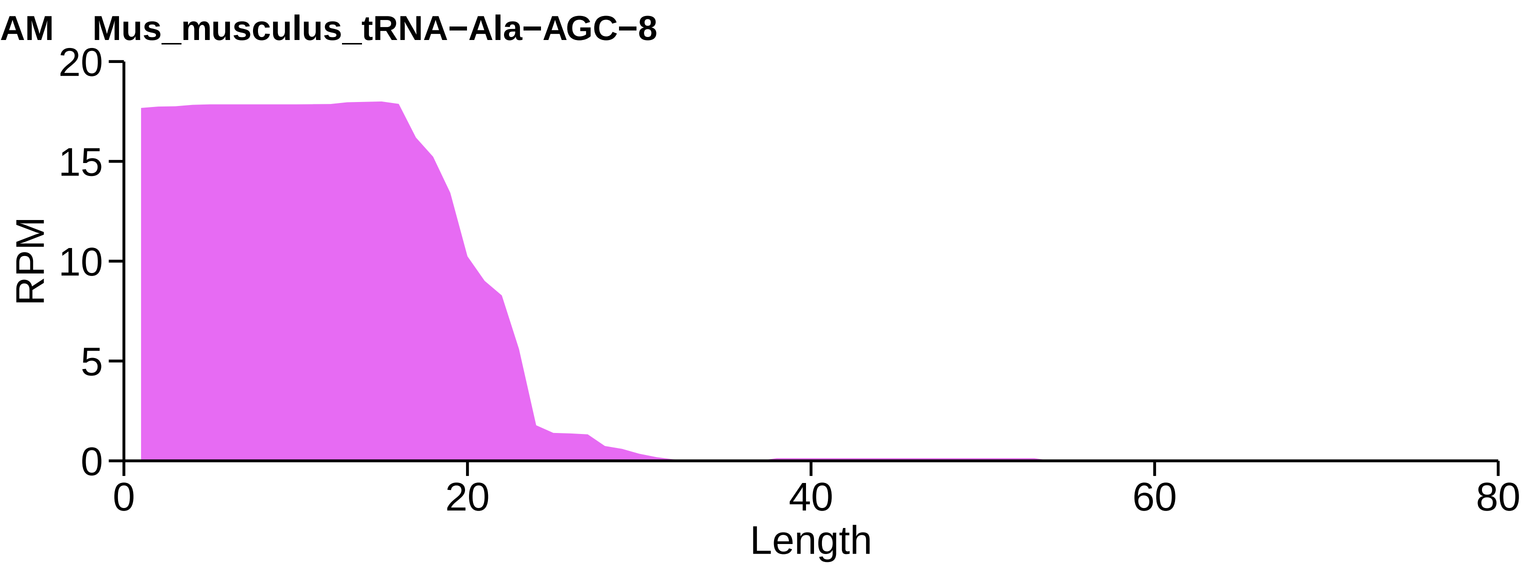

AM Mus_musculus_tRNA−Ala−AGC−8
RPM
20
15
10
5
0
0
20
40
60
80
Length

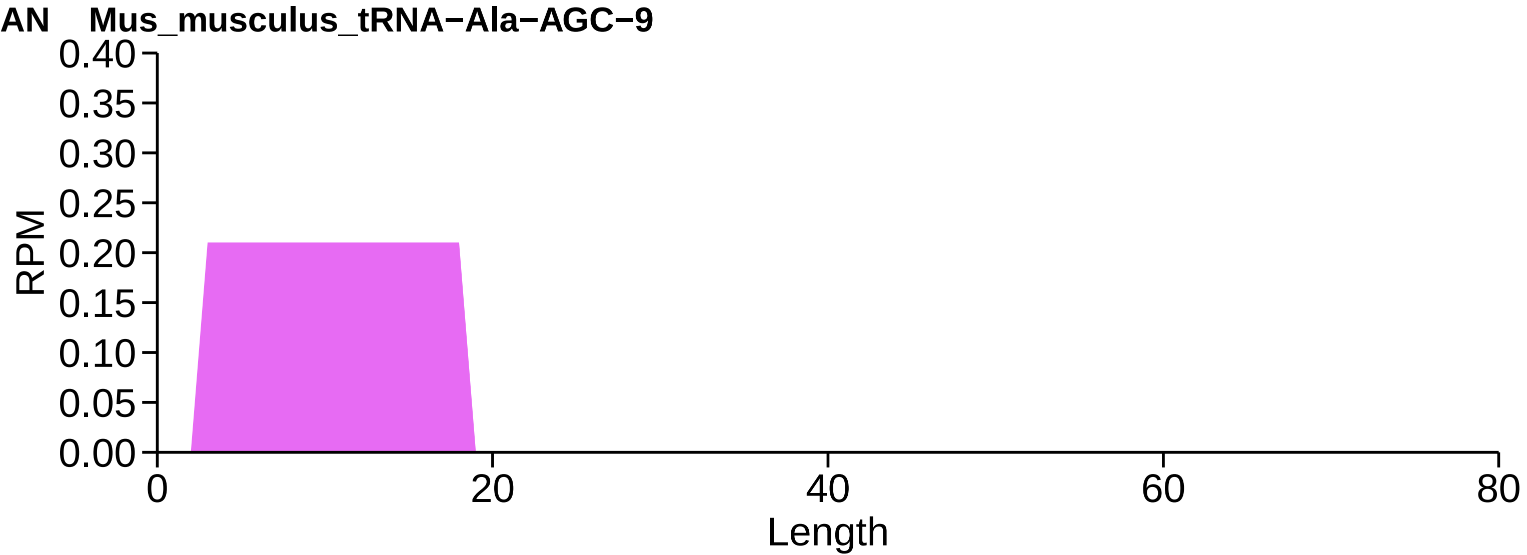

AN Mus_musculus_tRNA−Ala−AGC−9
0.40
0.35
0.30
0.25
0.20
0.15
0.10
0.05
0.00
RPM
0
20
40
60
80
Length

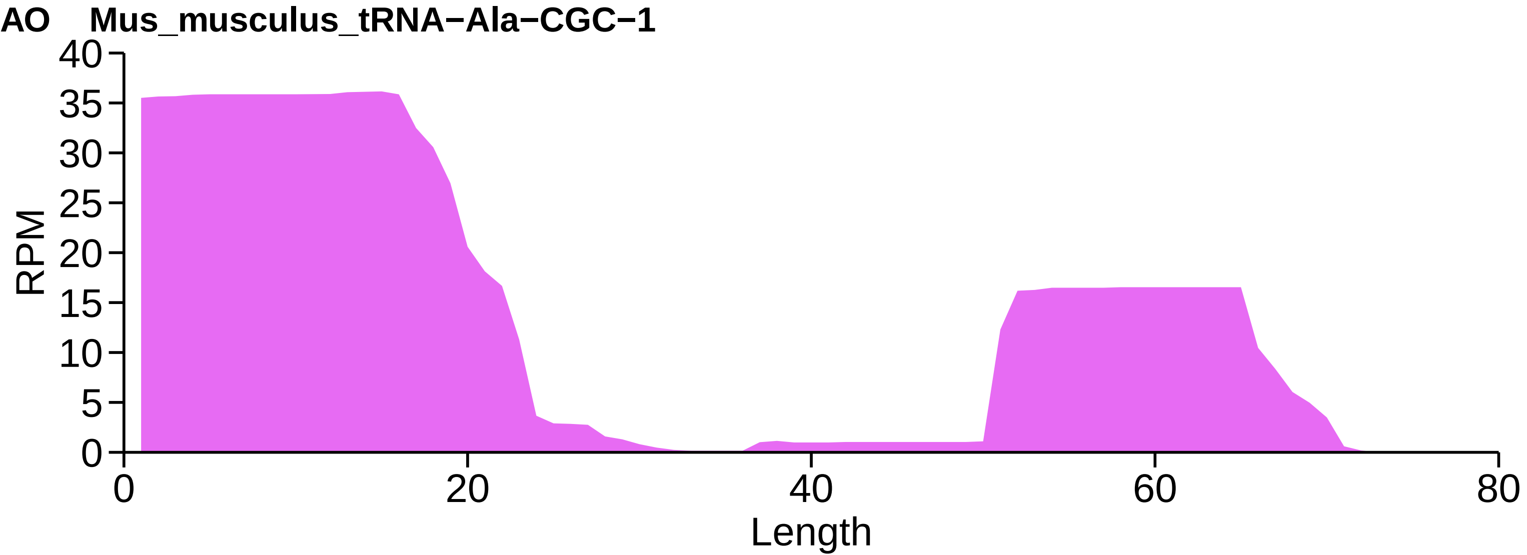
AO Mus_musculus_tRNA−Ala−CGC−1
RPM
Length
0
5
10
15
20
25
30
35
40
0
20
40
60
80

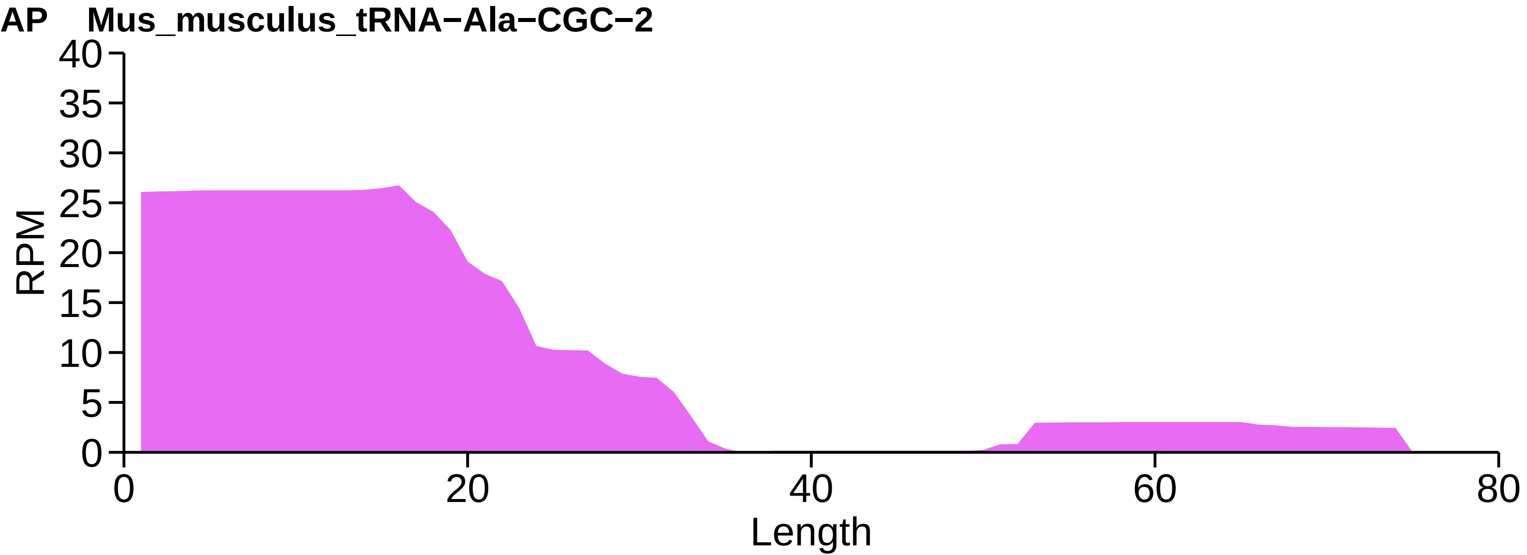

AP Mus_musculus_tRNA−Ala−CGC−2
RPM
40
35
30
25
20
15
10
5
0
0
20
40
60
80
Length

**AQ Mus_musculus_tRNA−Ala−CGC−3**

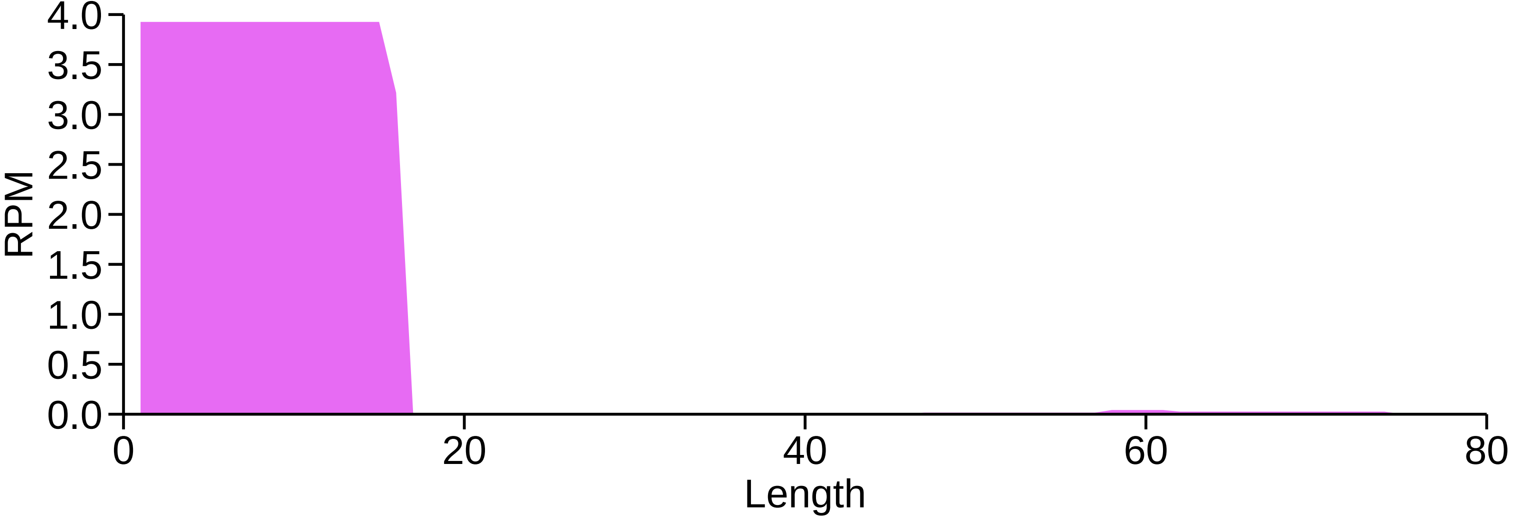

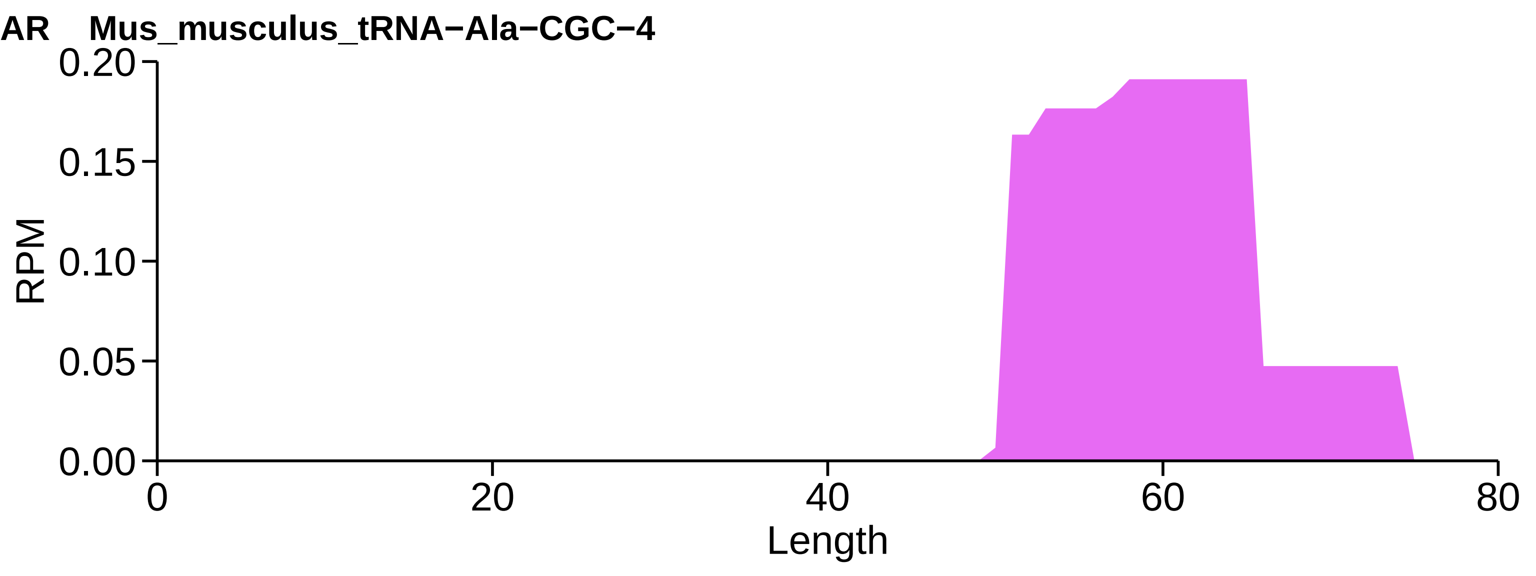
AR Mus_musculus_tRNA−Ala−CGC−4
RPM
0.00
0.05
0.10
0.15
0.20
0
20
40
60
80
Length

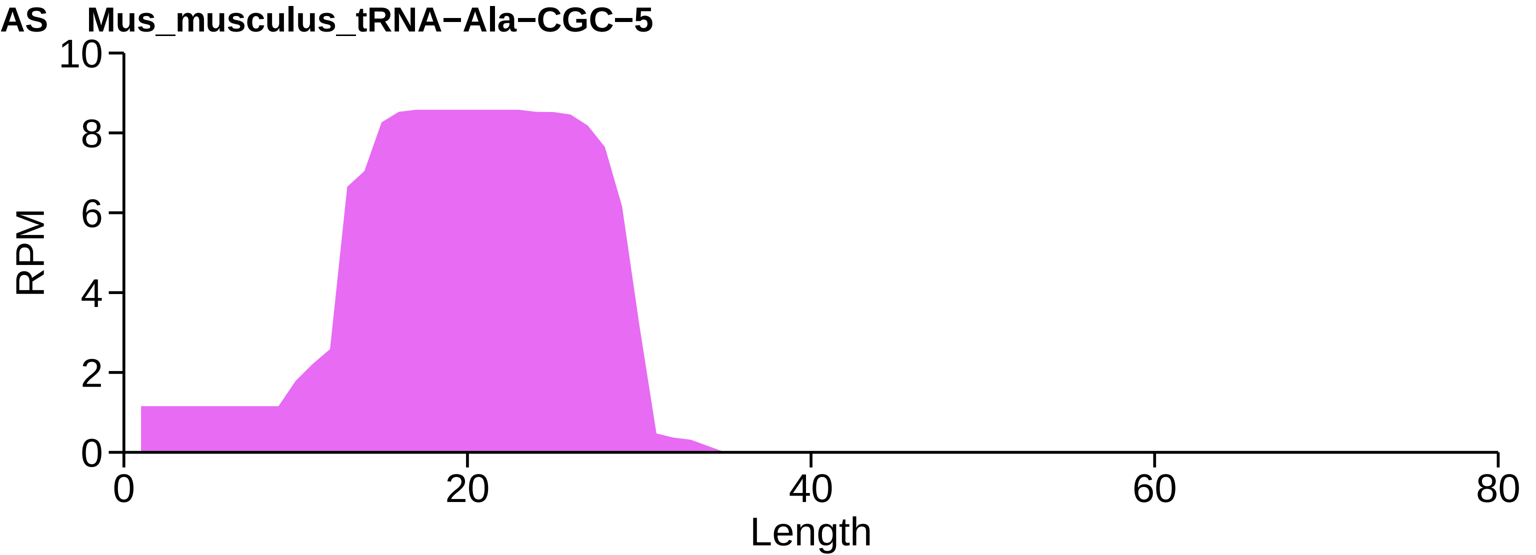

AS Mus_musculus_tRNA−Ala−CGC−5
10
8
6
4
2
0
RPM
0
20
40
60
80
Length

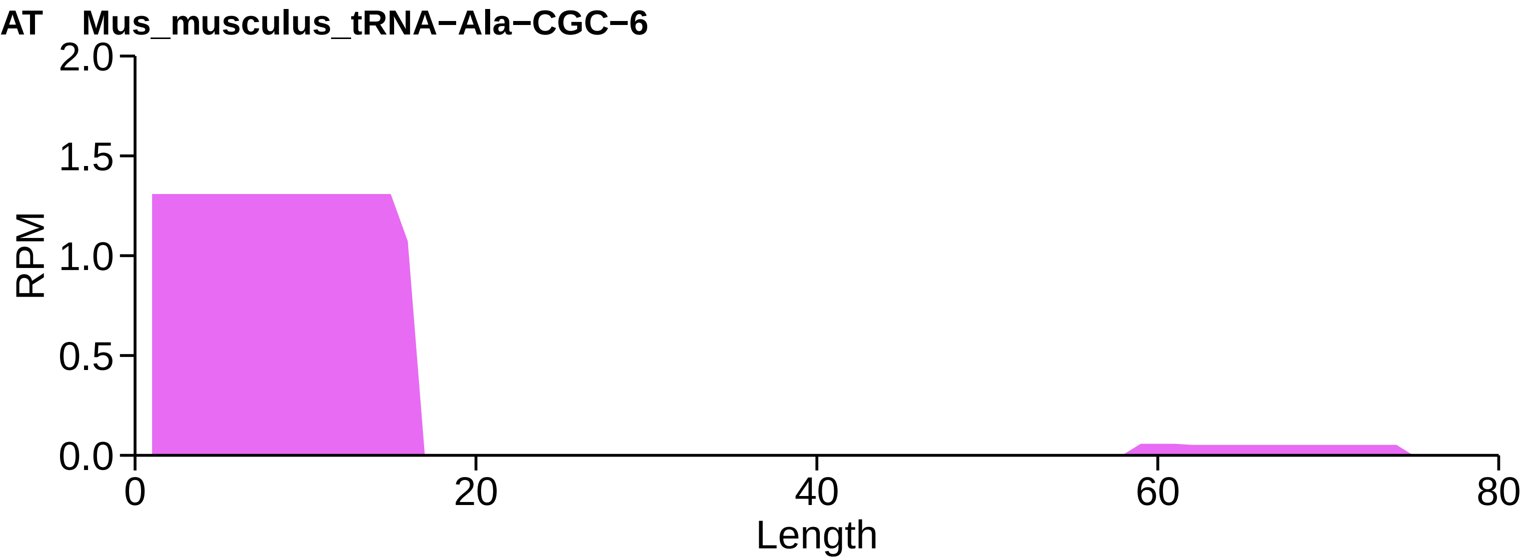
AT Mus_musculus_tRNA−Ala−CGC−6
RPM
2.0
1.5
1.0
0.5
0.0
0
20
40
60
80
Length

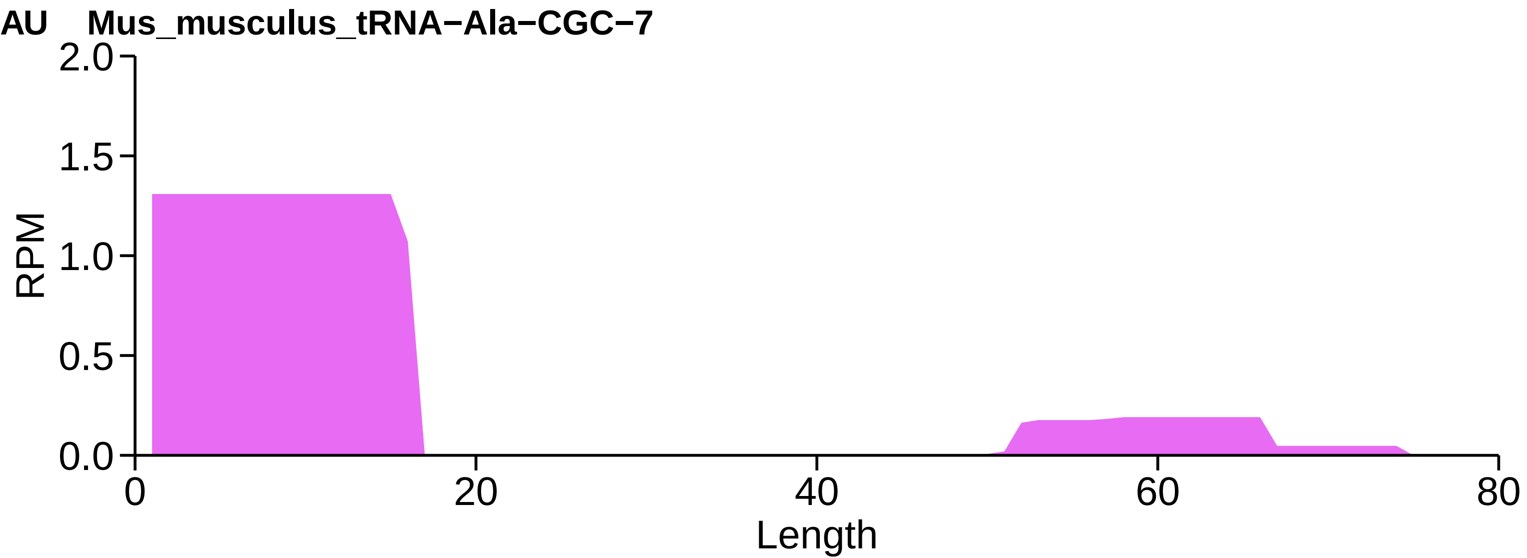

AU Mus_musculus_tRNA−Ala−CGC−7
2.0
1.5
1.0
0.5
0.0
RPM
0
20
40
60
80
Length

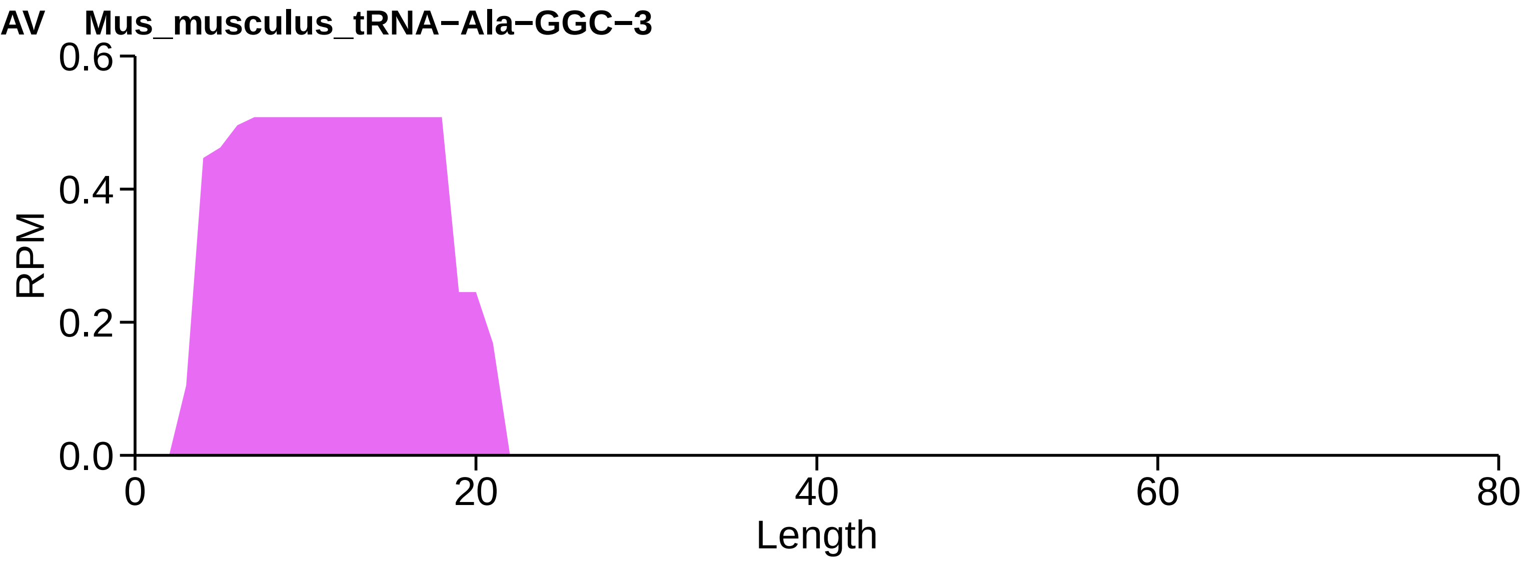
AV Mus_musculus_tRNA−Ala−GGC−3
0.6
0.4
0.2
0.0
RPM
0
20
40
60
80
Length

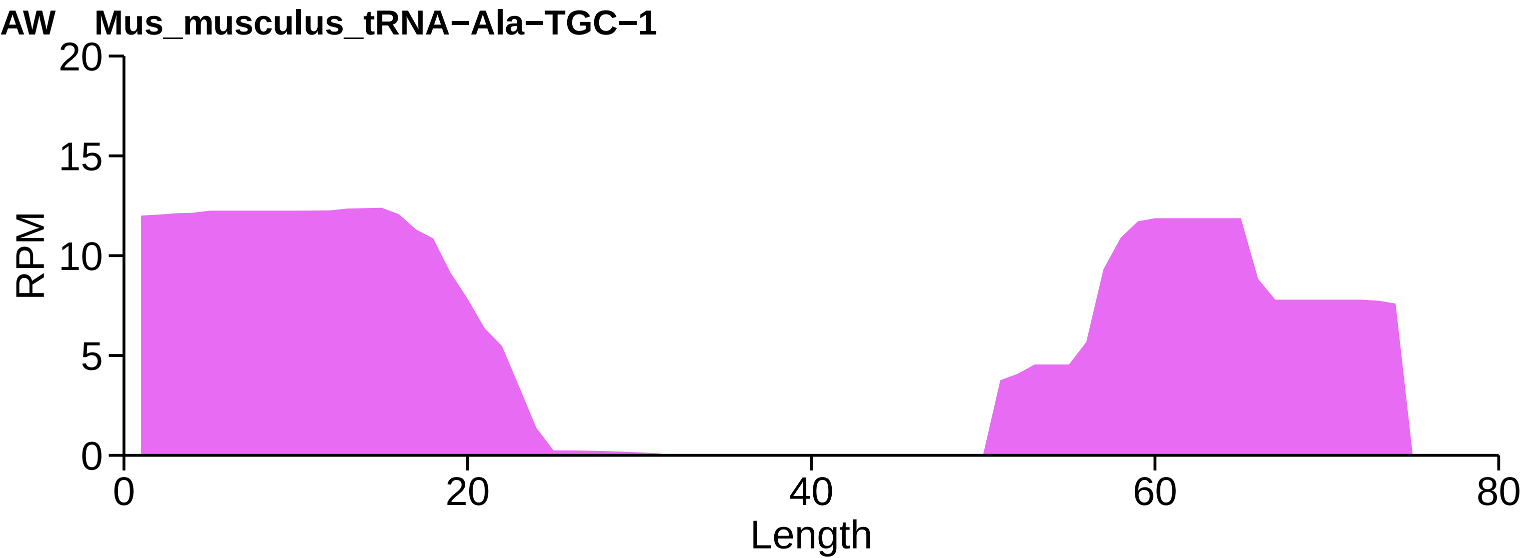
AW Mus_musculus_tRNA−Ala−TGC−1
20
15
10
5
0
RPM
0
20
40
60
80
Length

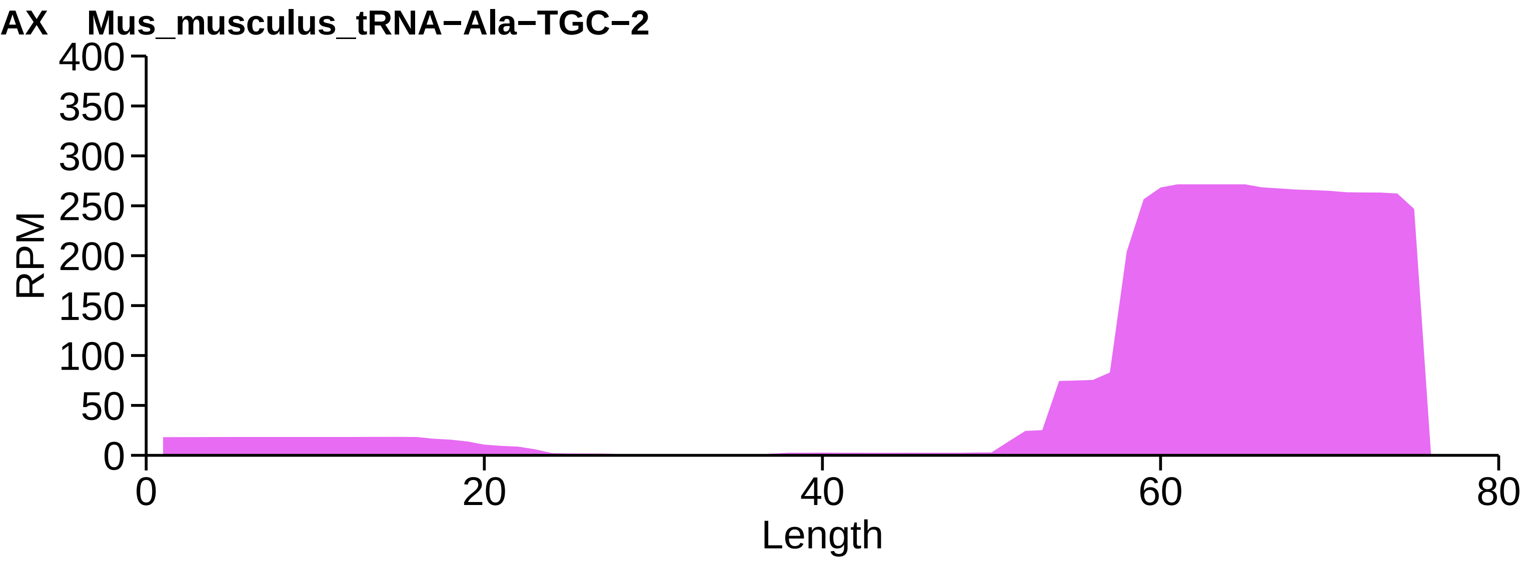

AX Mus_musculus_tRNA−Ala−TGC−2
RPM
0
50
100
150
200
250
300
350
400
0
20
40
60
80
Length

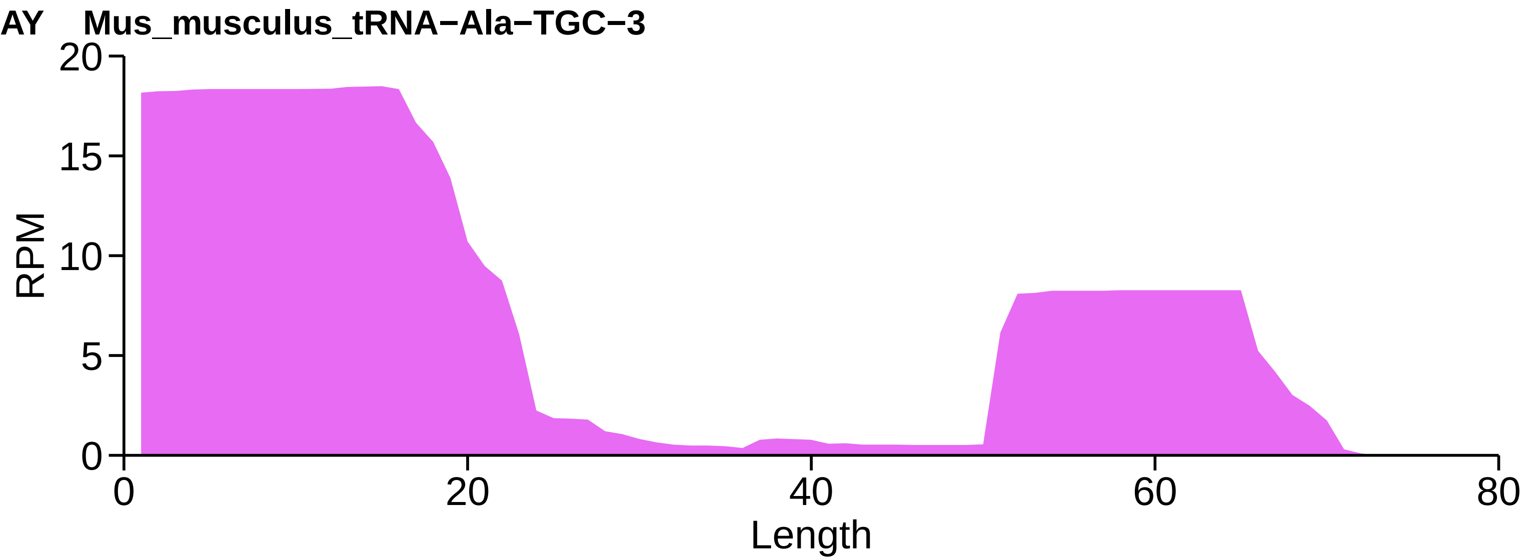
AY
Mus_musculus_tRNA−Ala−TGC−3
RPM
Length
0
5
10
15
20
0
20
40
60
80

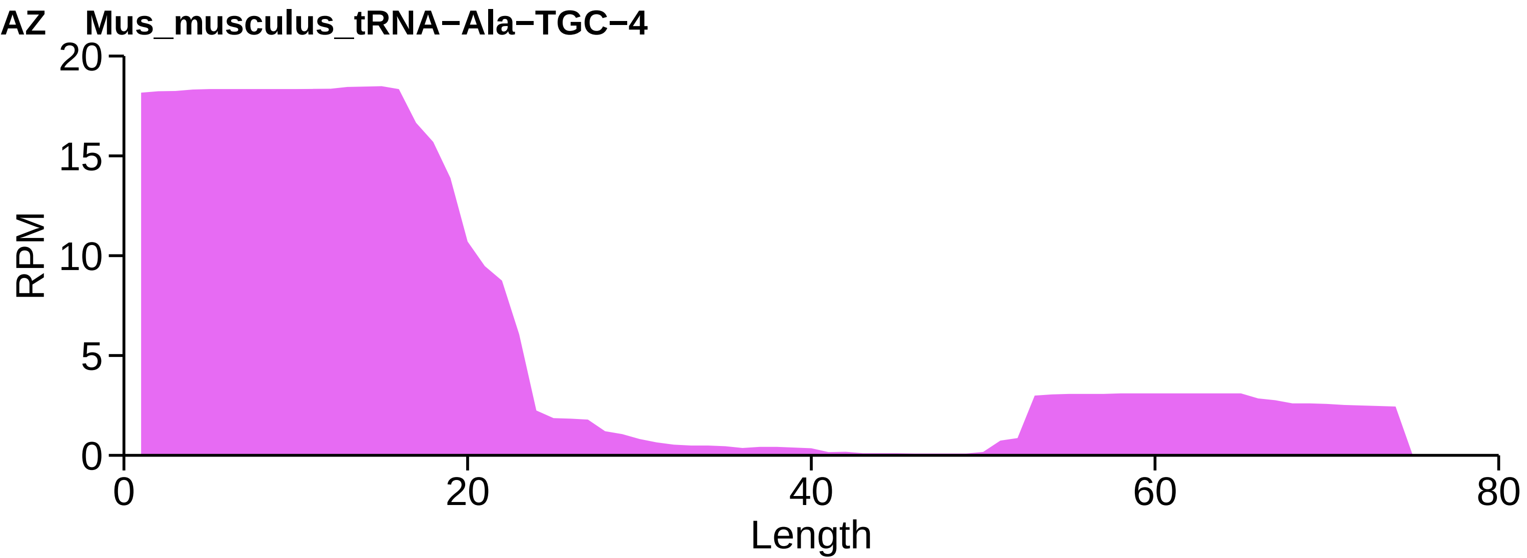

AZ Mus_musculus_tRNA−Ala−TGC−4
RPM
20
15
10
5
0
0
20
40
60
80
Length

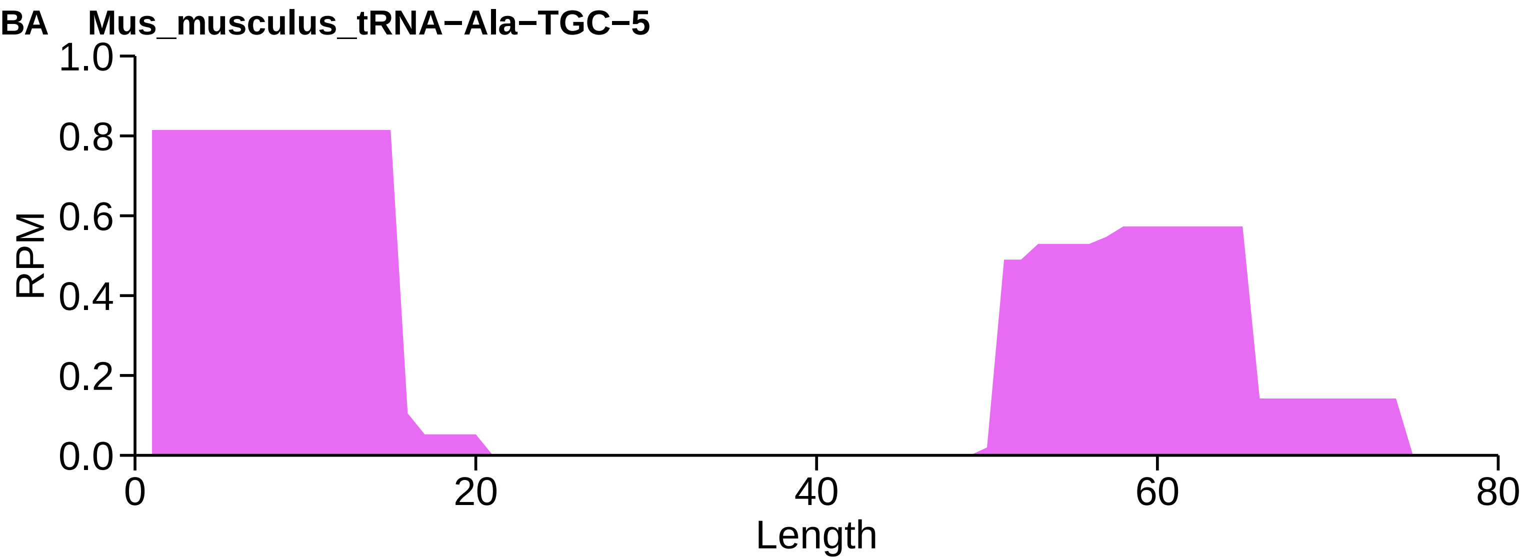
BA Mus_musculus_tRNA−Ala−TGC−5
1.0
0.8
0.6
0.4
0.2
0.0
RPM
0
20
40
60
80
Length

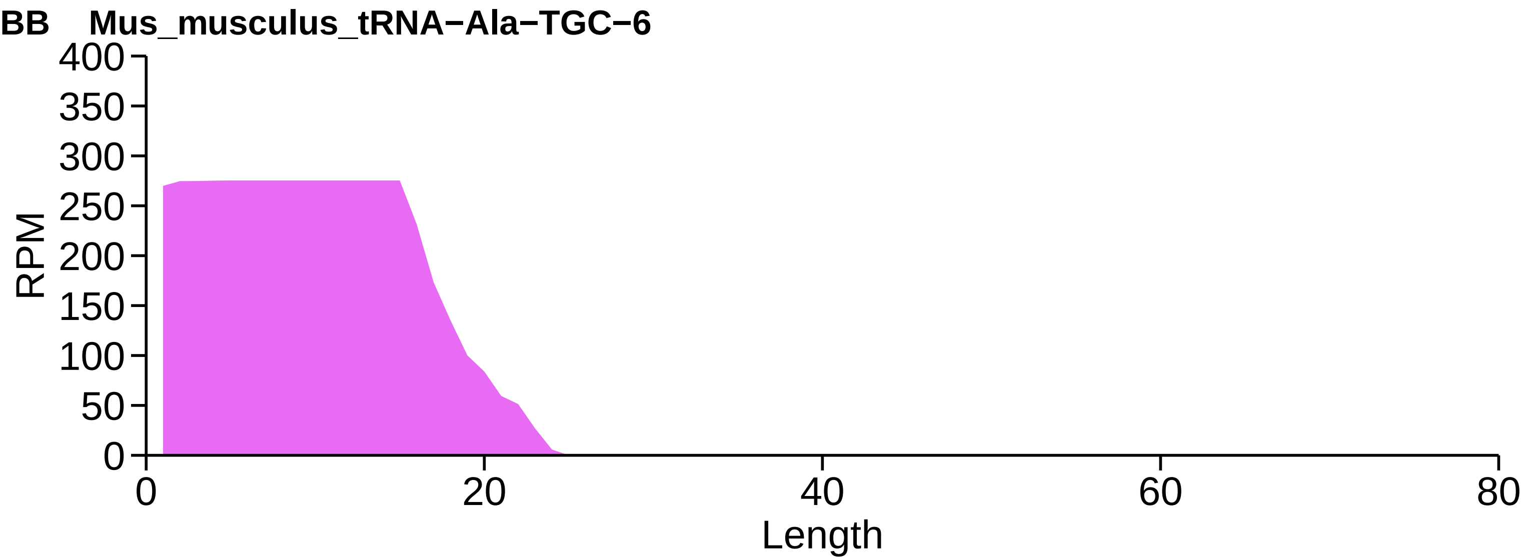
BB Mus_musculus_tRNA−Ala−TGC−6
RPM
400
350
300
250
200
150
100
50
0
0
20
40
60
80
Length

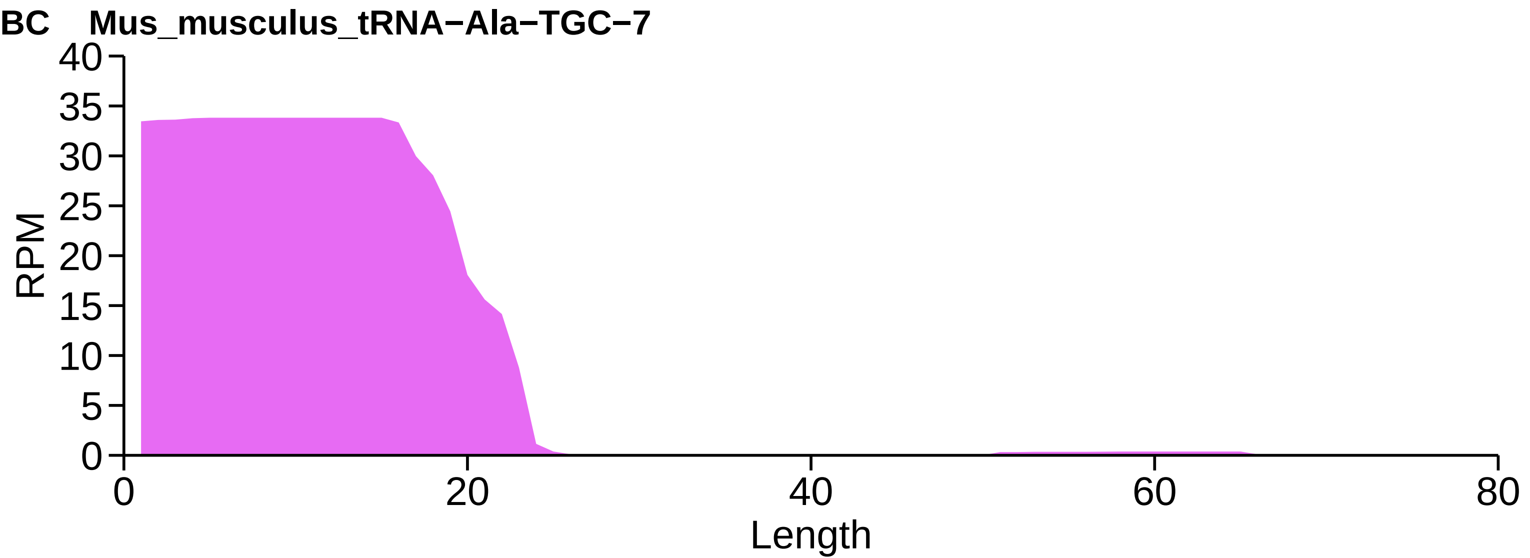
BC Mus_musculus_tRNA−Ala−TGC−7
RPM
Length
0
5
10
15
20
25
30
35
40
0
20
40
60
80

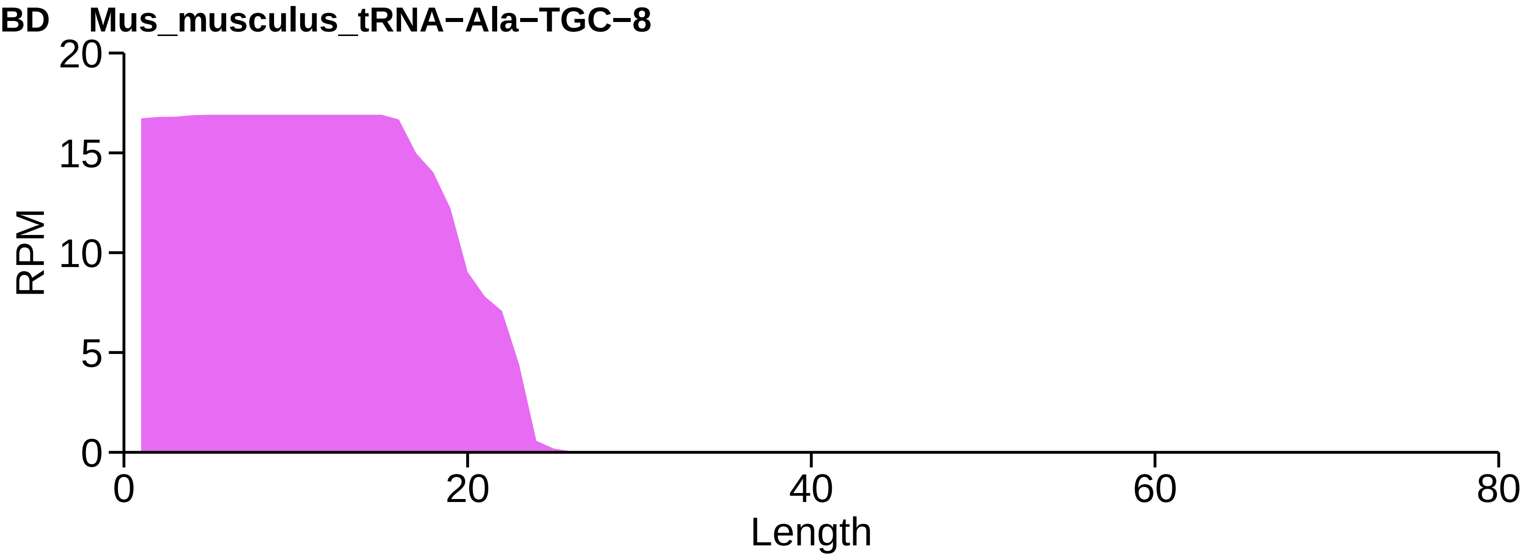
BD Mus_musculus_tRNA−Ala−TGC−8
RPM
20
15
10
5
0
0
20
40
60
80
Length

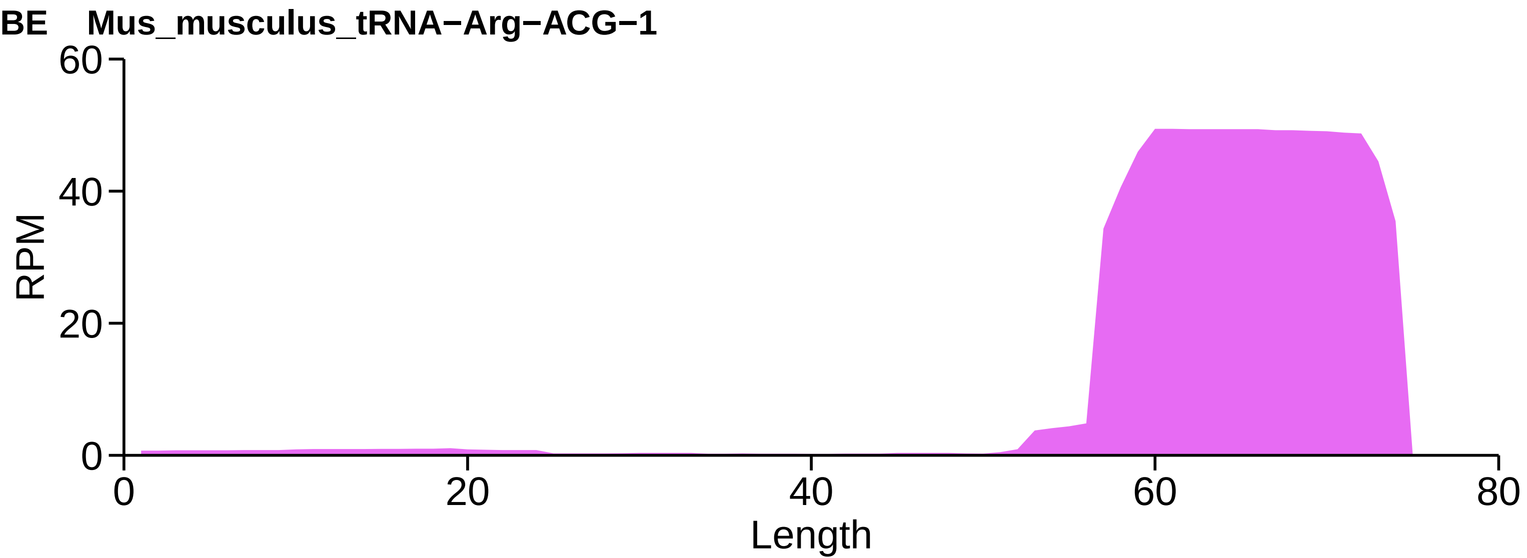

BE Mus_musculus_tRNA−Arg−ACG−1
RPM
60
40
20
0
0
20
40
60
80
Length

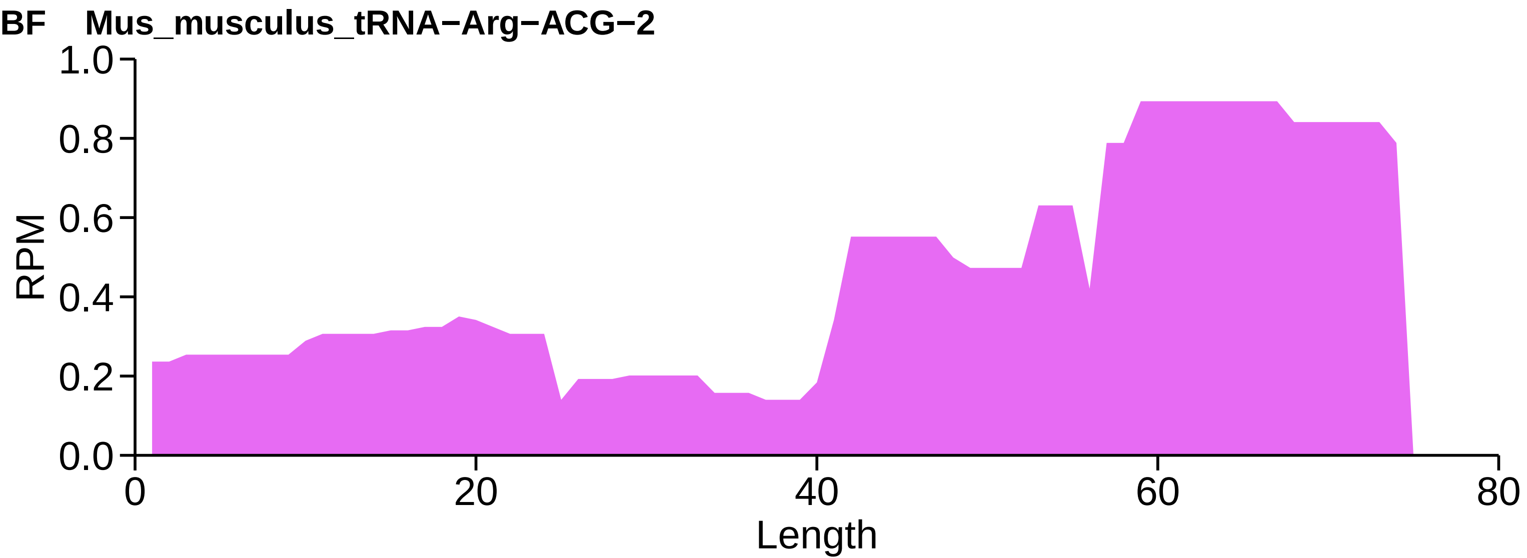

BF Mus_musculus_tRNA−Arg−ACG−2
1.0
0.8
0.6
0.4
0.2
0.0
RPM
0
20
40
60
80
Length

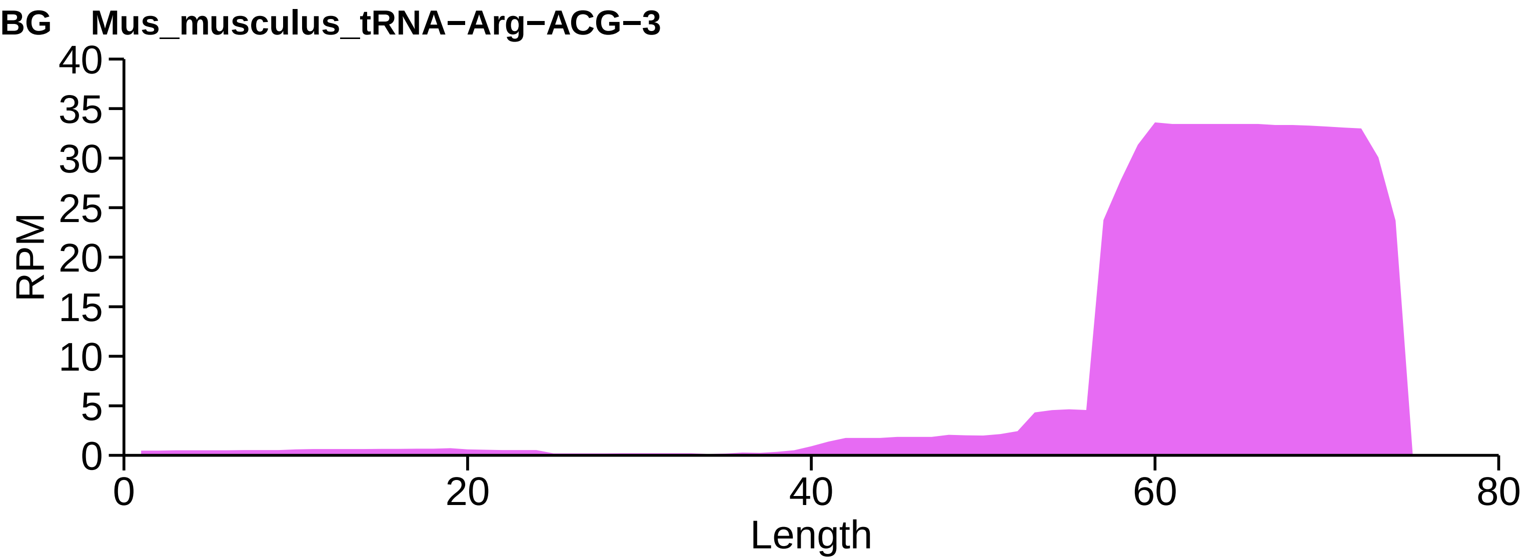
BG Mus_musculus_tRNA−Arg−ACG−3
RPM
Length
0
5
10
15
20
25
30
35
40
0
20
40
60
80

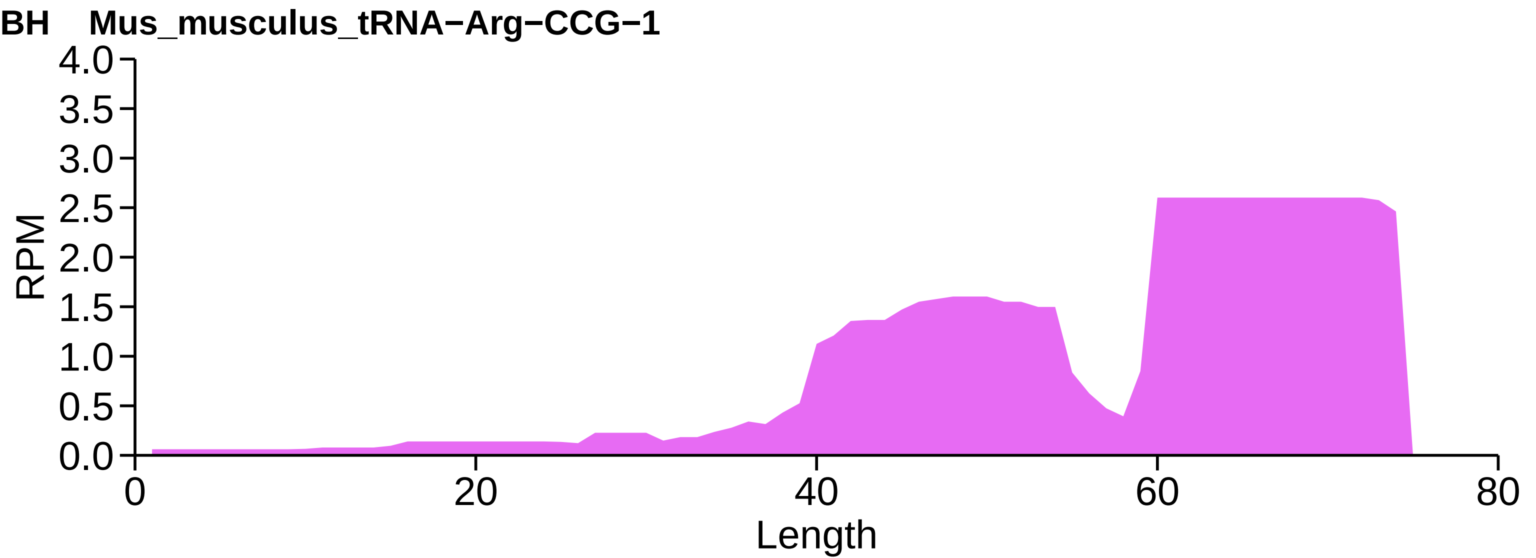
BH Mus_musculus_tRNA−Arg−CCG−1
RPM
Length
0.0
0.5
1.0
1.5
2.0
2.5
3.0
3.5
4.0
0
20
40
60
80

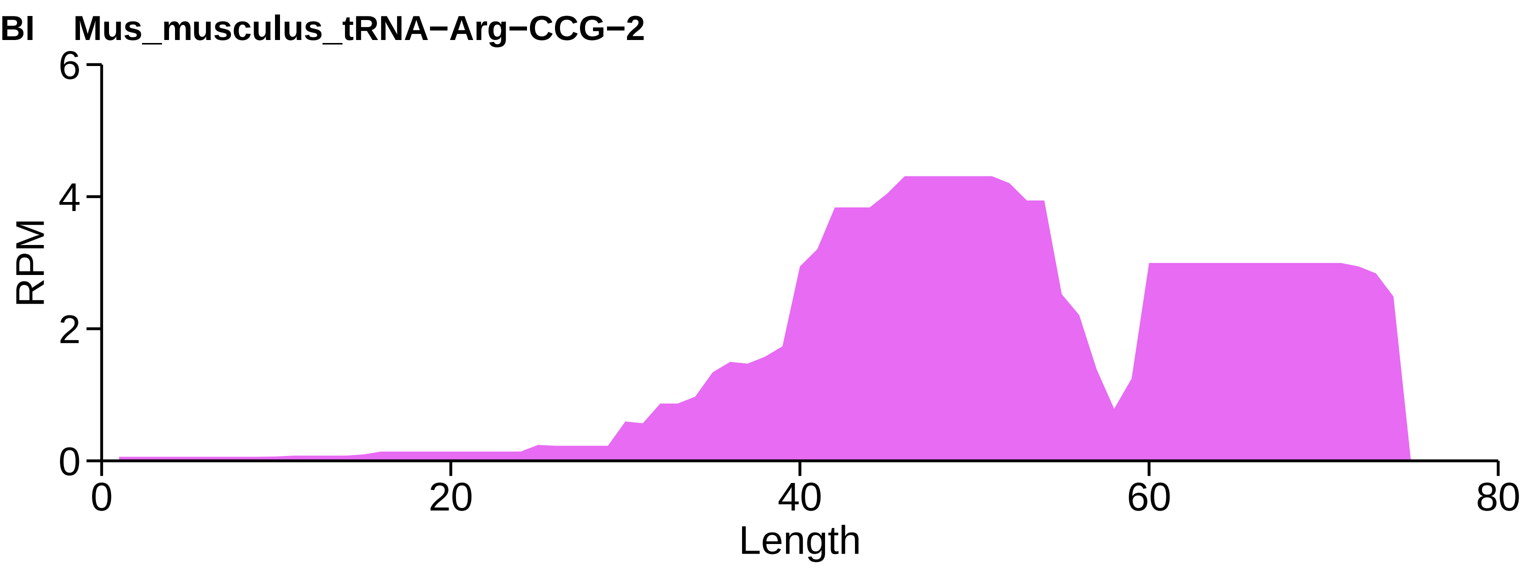
BI Mus_musculus_tRNA−Arg−CCG−2
RPM
0
2
4
6
0
20
40
60
80
Length

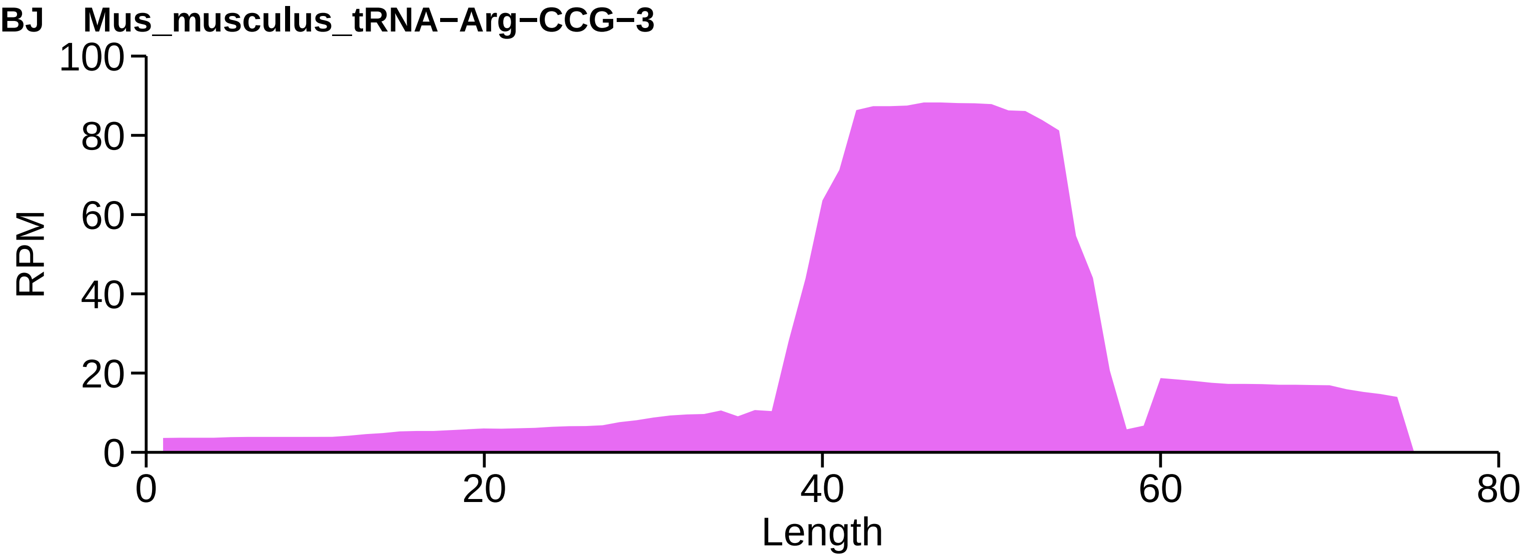
BJ Mus_musculus_tRNA−Arg−CCG−3
RPM
Length
0
20
40
60
80
100
0
20
40
60
80

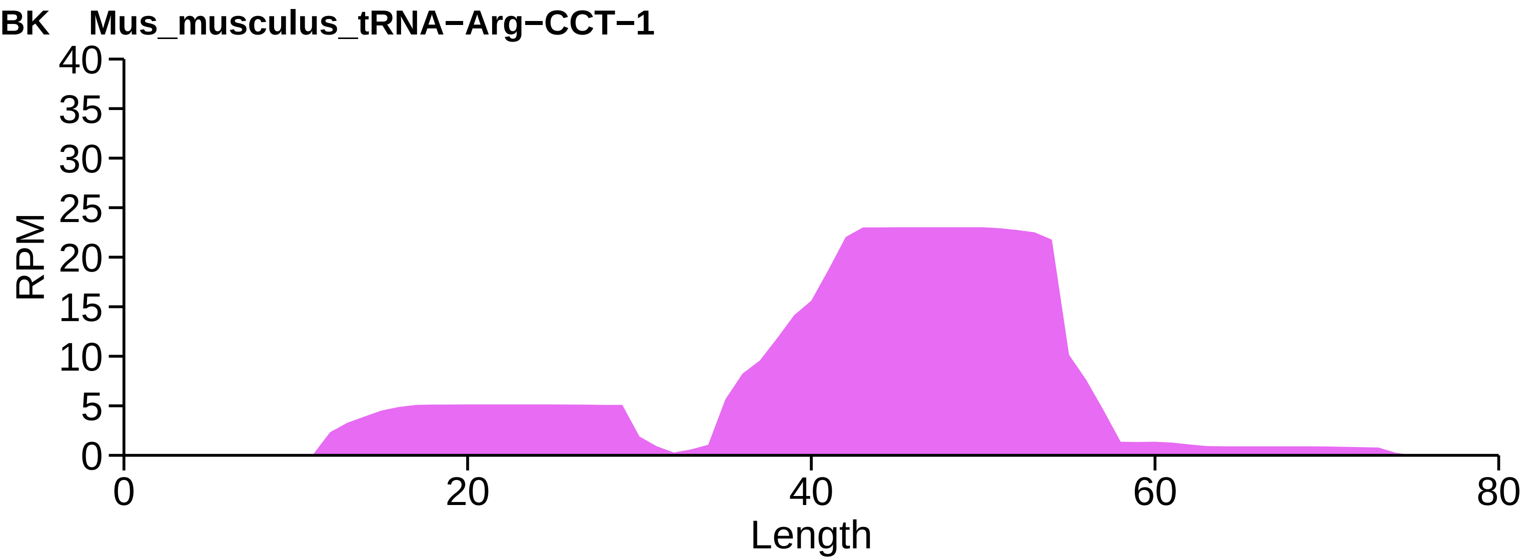

BK Mus_musculus_tRNA−Arg−CCT−1
RPM
0
5
10
15
20
25
30
35
40
0
20
40
60
80
Length

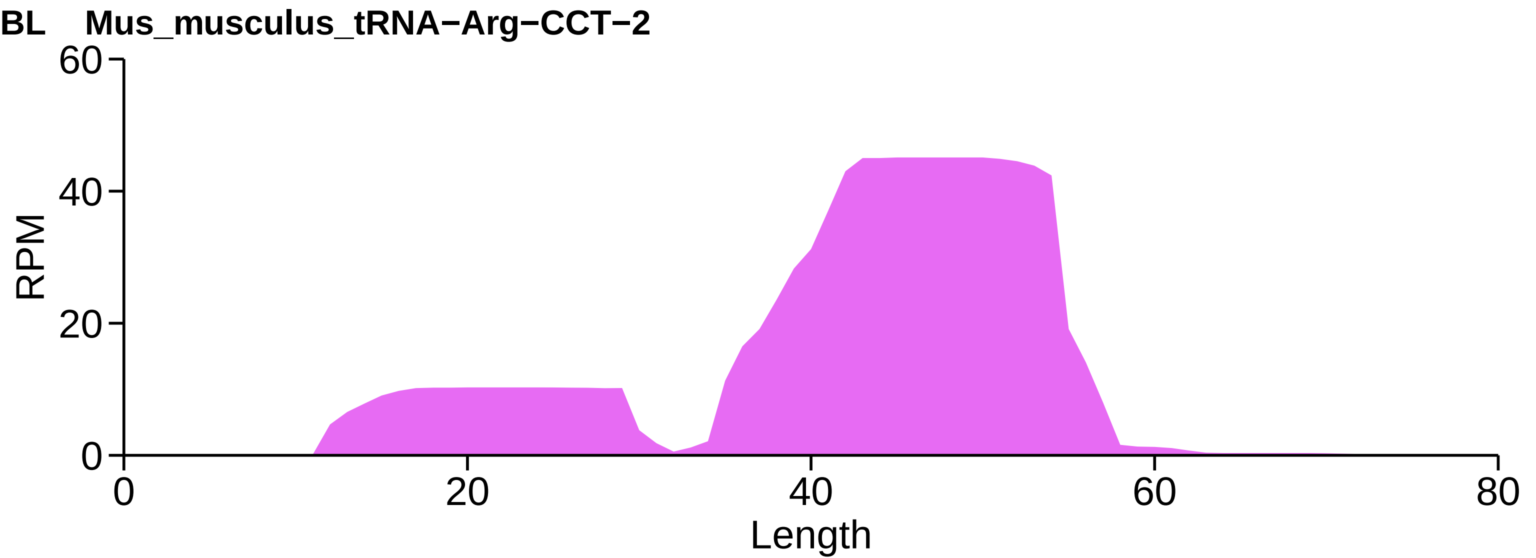

BL Mus_musculus_tRNA−Arg−CCT−2
RPM
60
40
20
0
0
20
40
60
80
Length

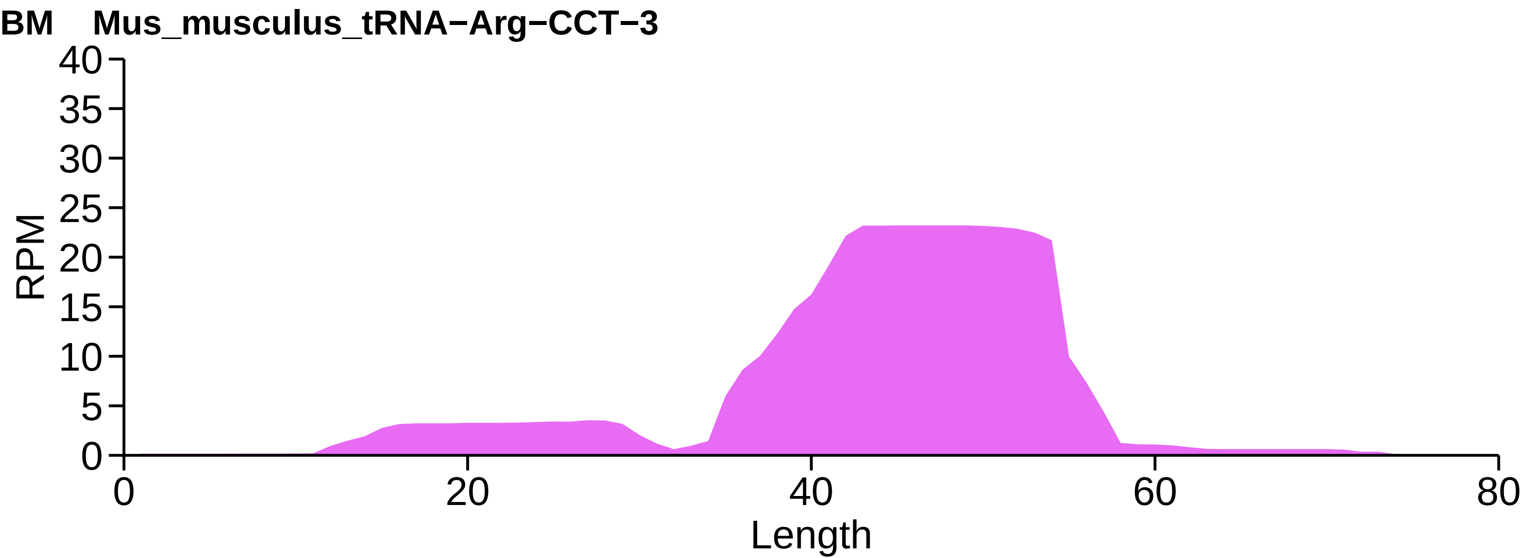
BM Mus_musculus_tRNA−Arg−CCT−3
RPM
Length
0
5
10
15
20
25
30
35
40
0
20
40
60
80

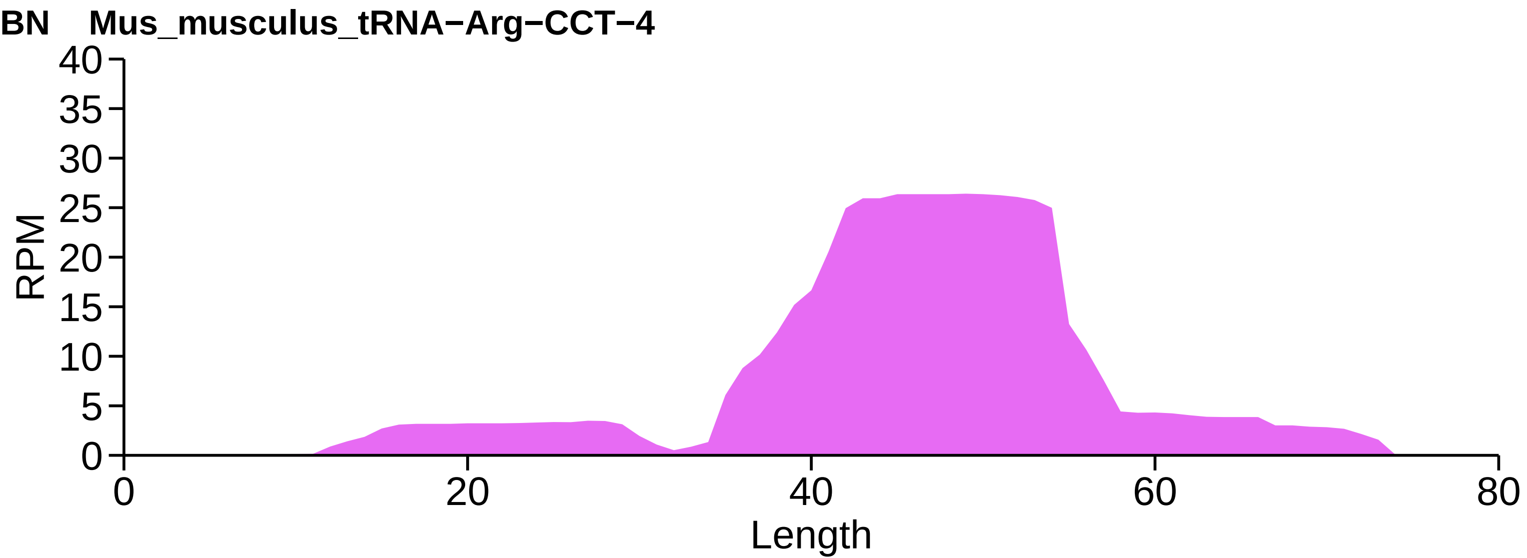

BN Mus_musculus_tRNA−Arg−CCT−4
RPM
0
5
10
15
20
25
30
35
40
0
20
40
60
80
Length

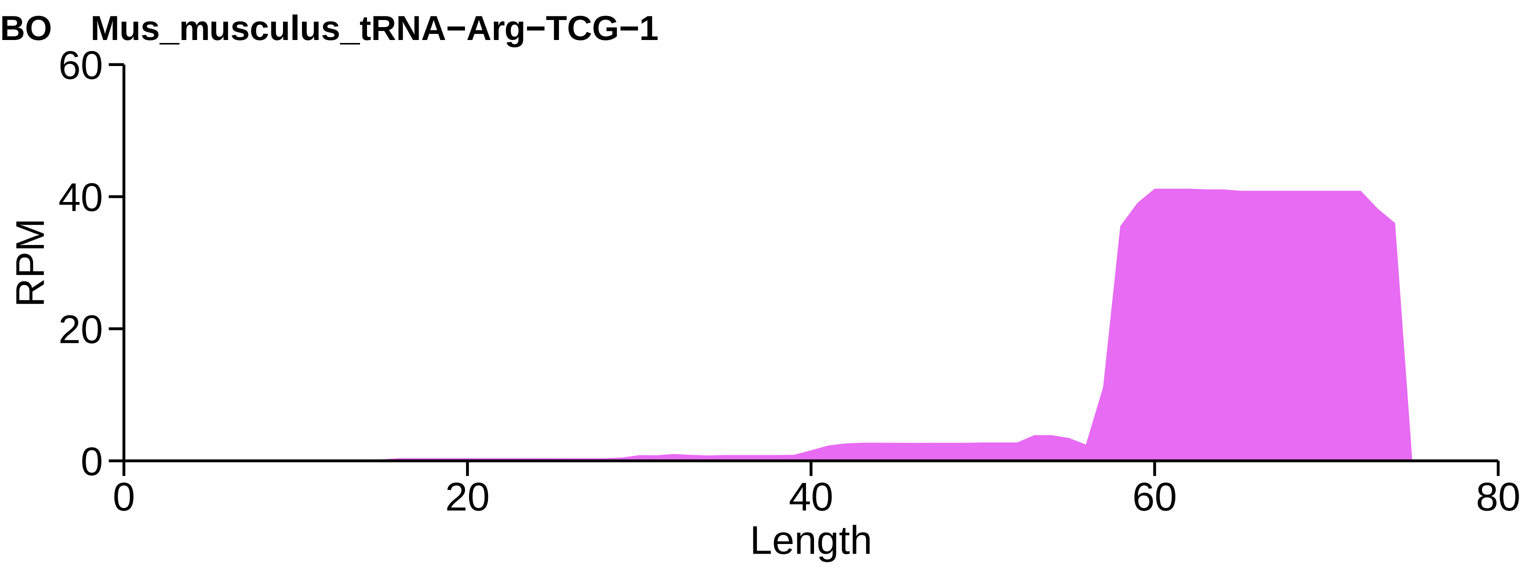
BO Mus_musculus_tRNA−Arg−TCG−1
RPM
Length
0
20
40
60
0
20
40
60
80

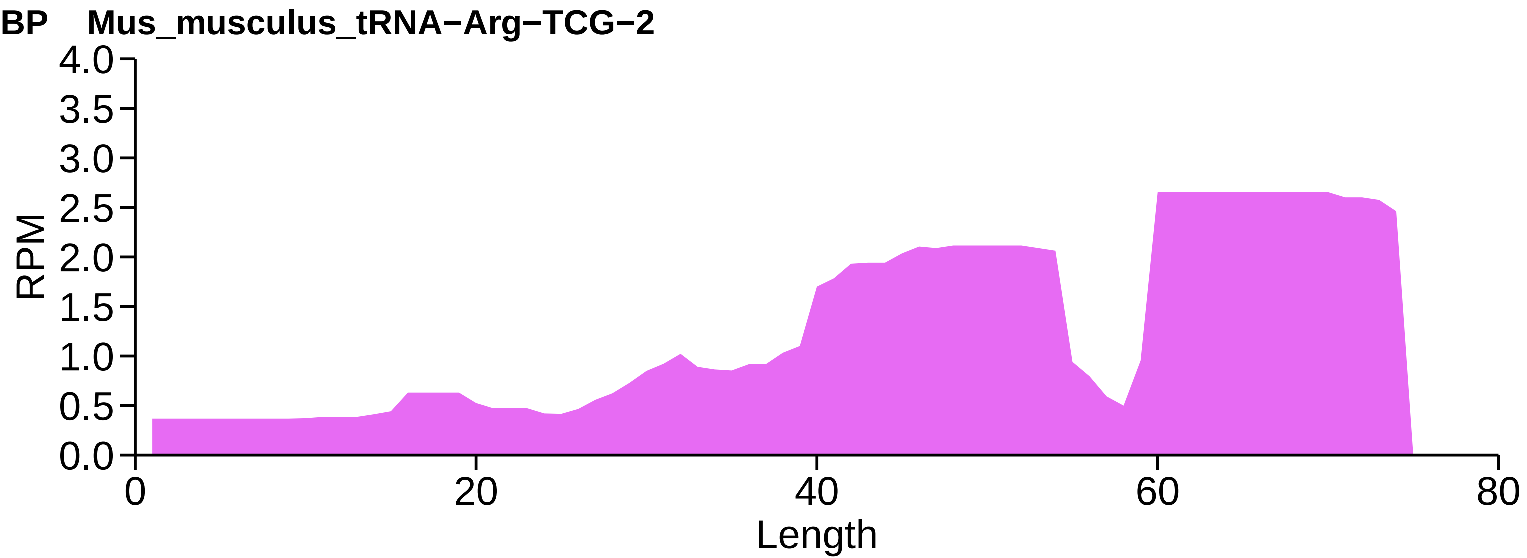
BP Mus_musculus_tRNA−Arg−TCG−2
RPM
4.0
3.5
3.0
2.5
2.0
1.5
1.0
0.5
0.0
0
20
40
60
80
Length

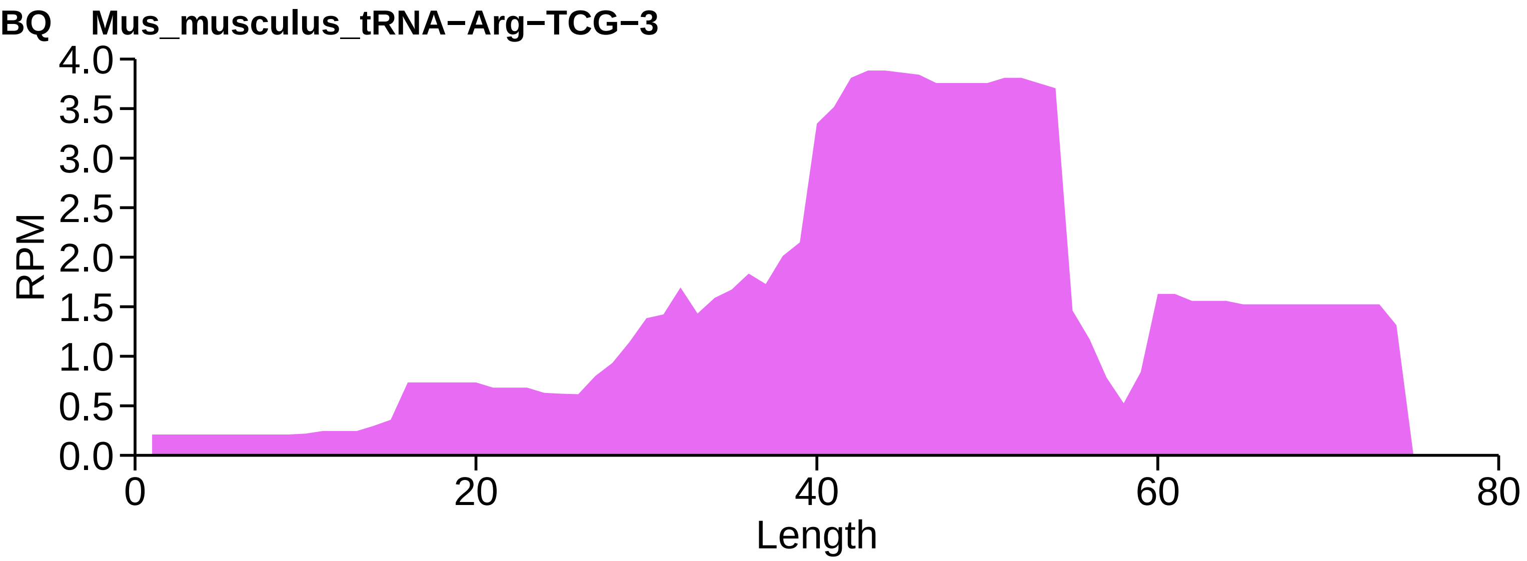
BQ Mus_musculus_tRNA−Arg−TCG−3
RPM
Length
4.0
3.5
3.0
2.5
2.0
1.5
1.0
0.5
0.0
0
20
40
60
80

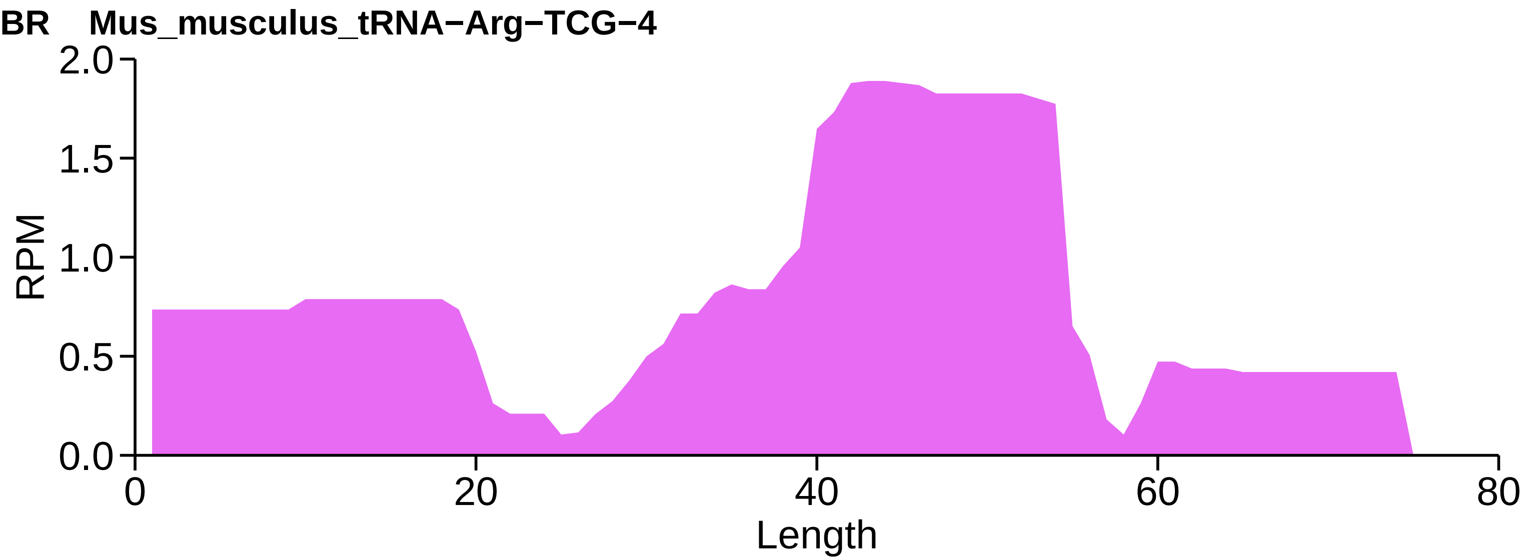

BR Mus_musculus_tRNA−Arg−TCG−4
2.0
1.5
1.0
0.5
0.0
RPM
0
20
40
60
80
Length

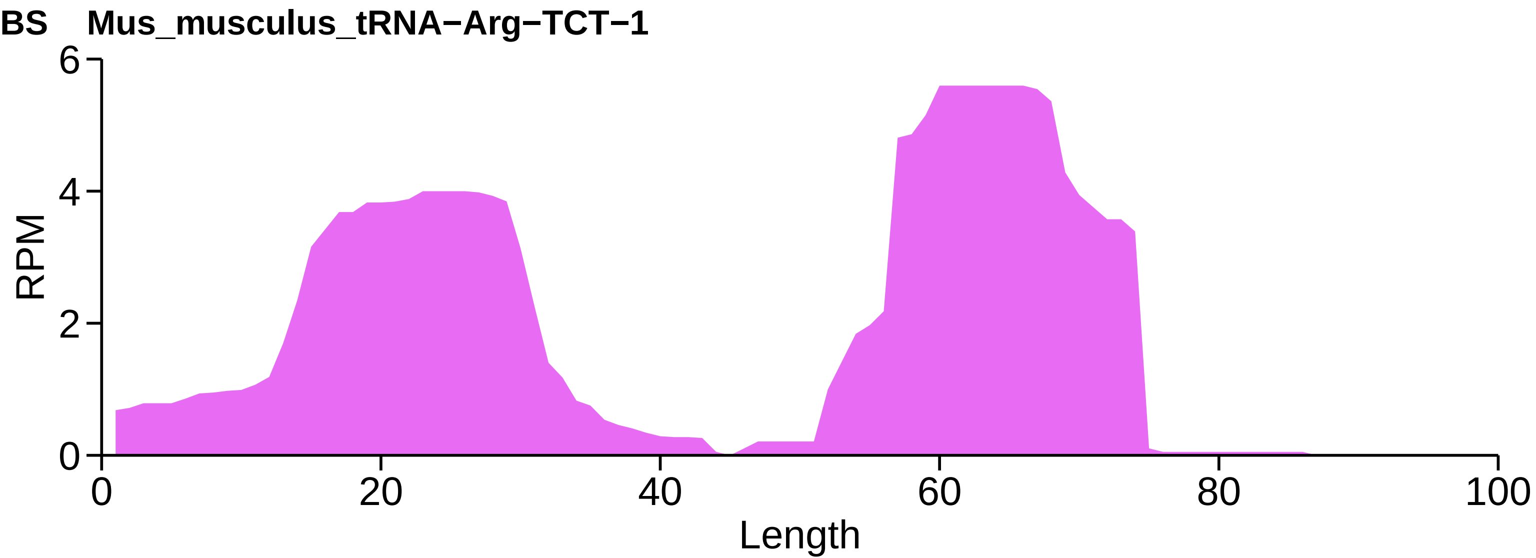

BS Mus_musculus_tRNA−Arg−TCT−1
RPM
0
2
4
6
0
20
40
60
80
100
Length

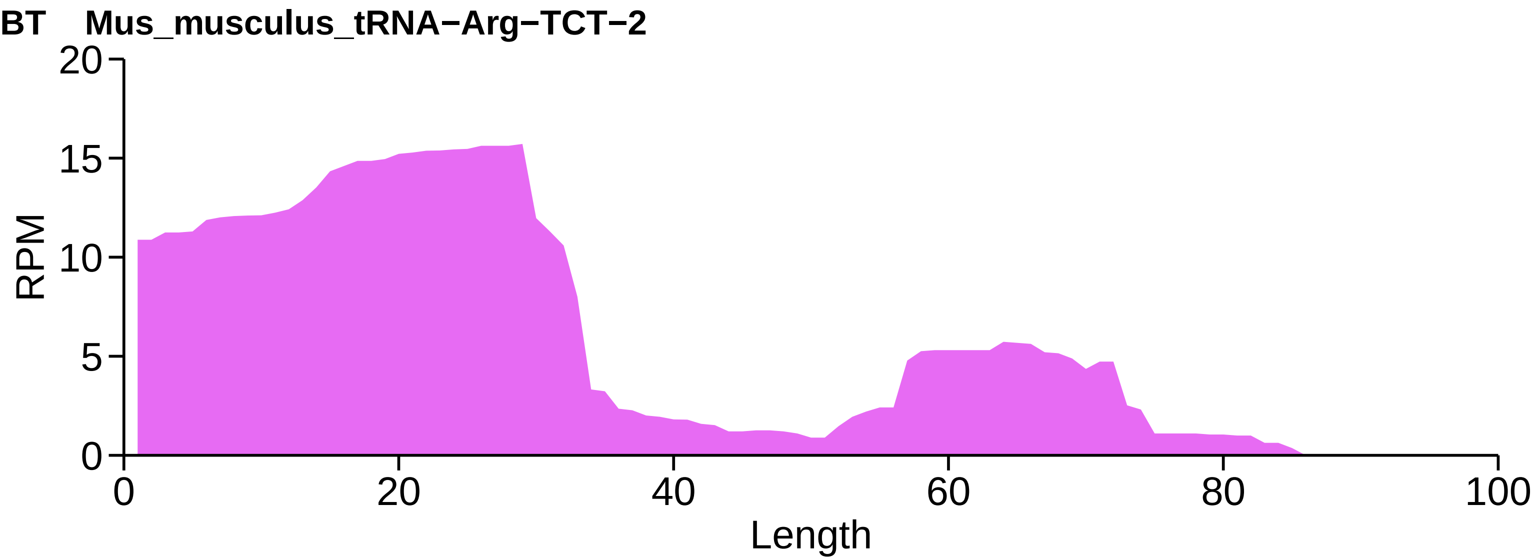
BT Mus_musculus_tRNA−Arg−TCT−2
RPM
20
15
10
5
0
0
20
40
60
80
100
Length

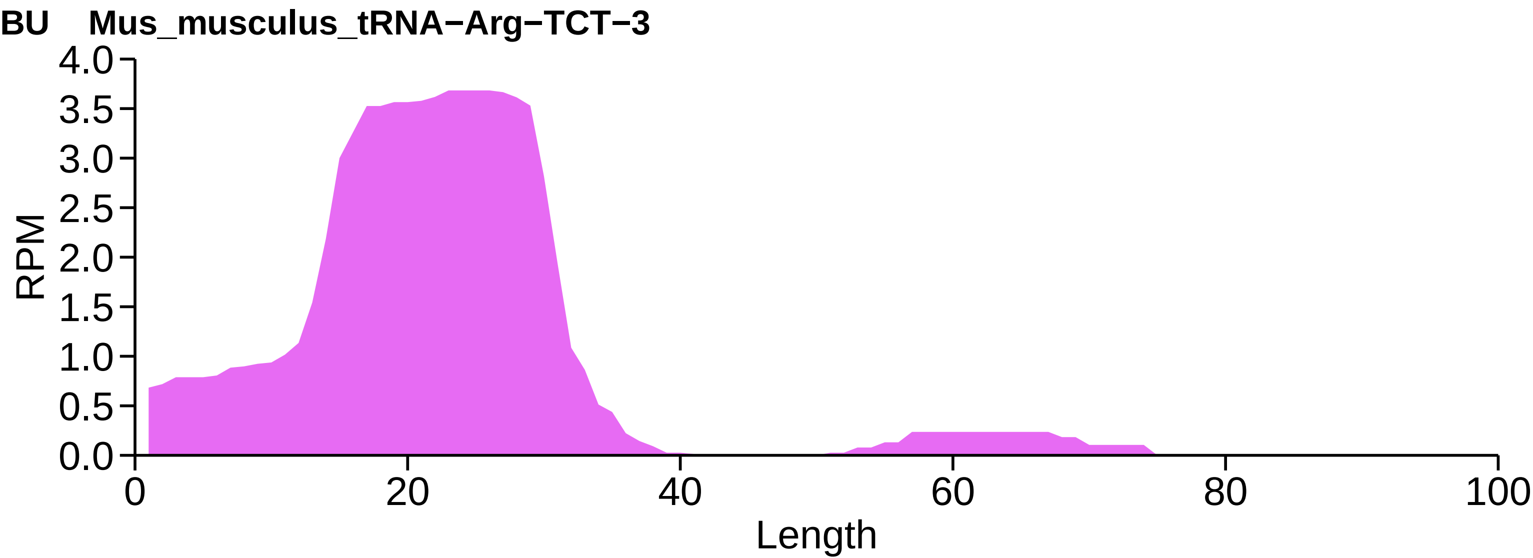
BU Mus_musculus_tRNA−Arg−TCT−3
RPM
4.0
3.5
3.0
2.5
2.0
1.5
1.0
0.5
0.0
0
20
40
60
80
100
Length

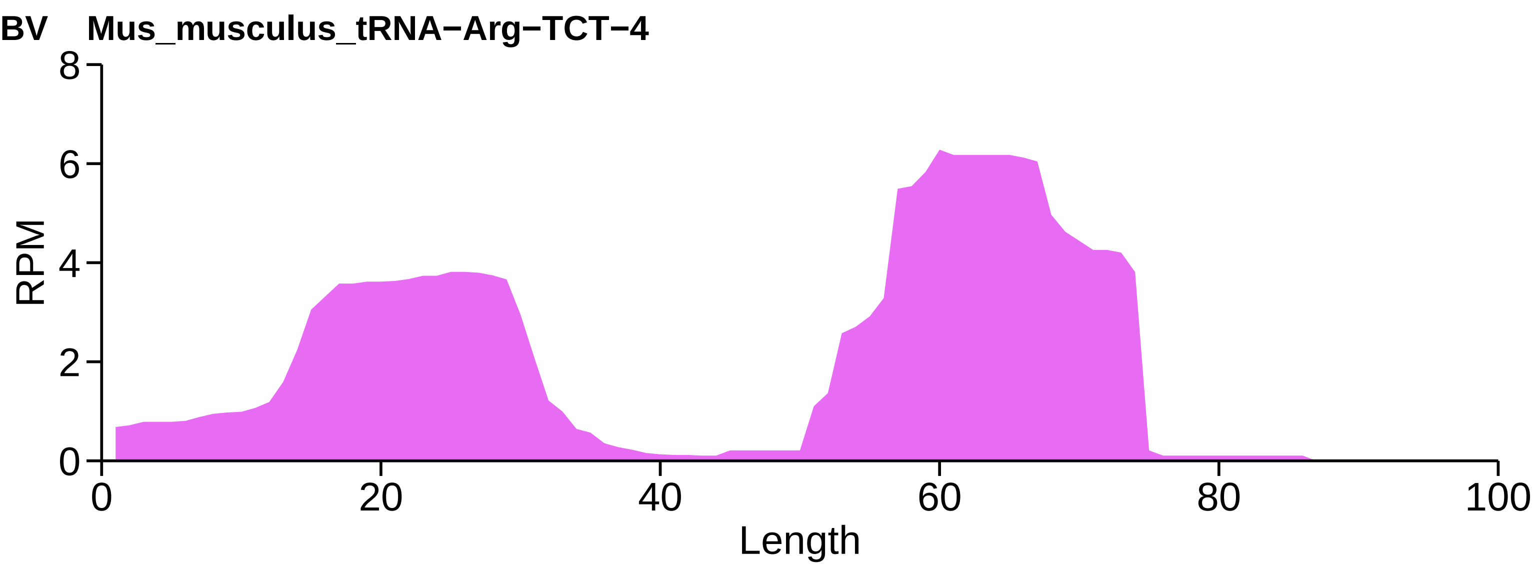
BV Mus_musculus_tRNA−Arg−TCT−4
RPM
Length
0
2
4
6
8
0
20
40
60
80
100

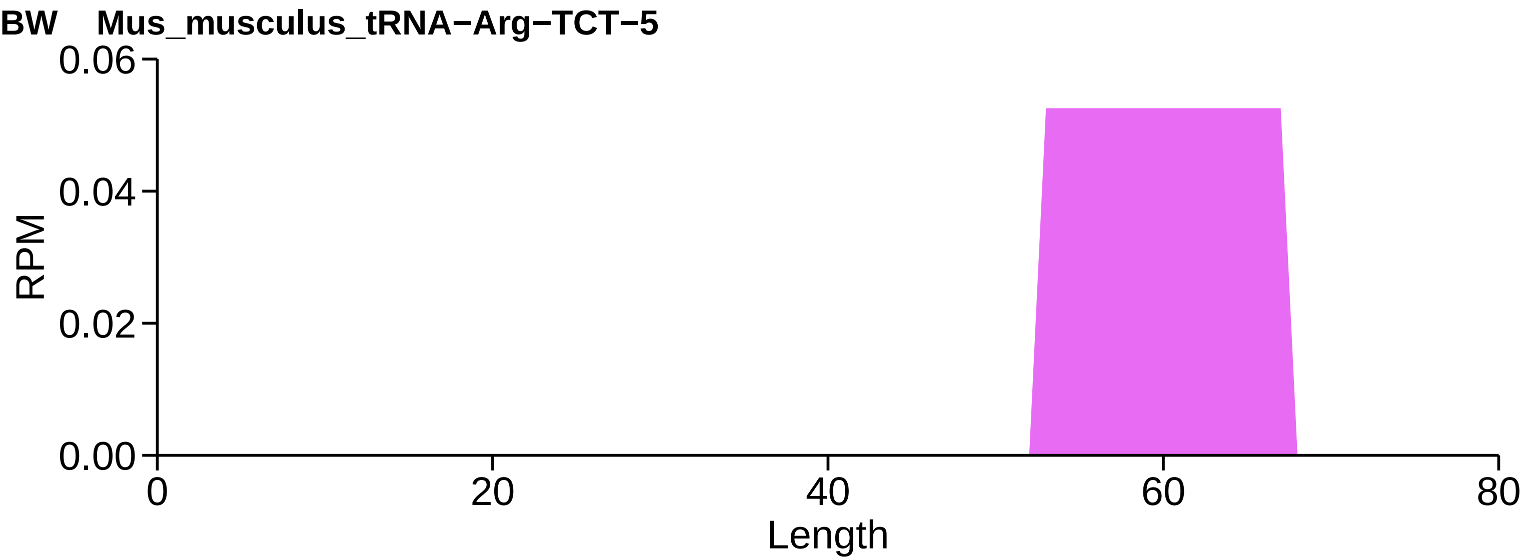
BW Mus_musculus_tRNA−Arg−TCT−5
0.06
0.04
0.02
0.00
RPM
0
20
40
60
80
Length

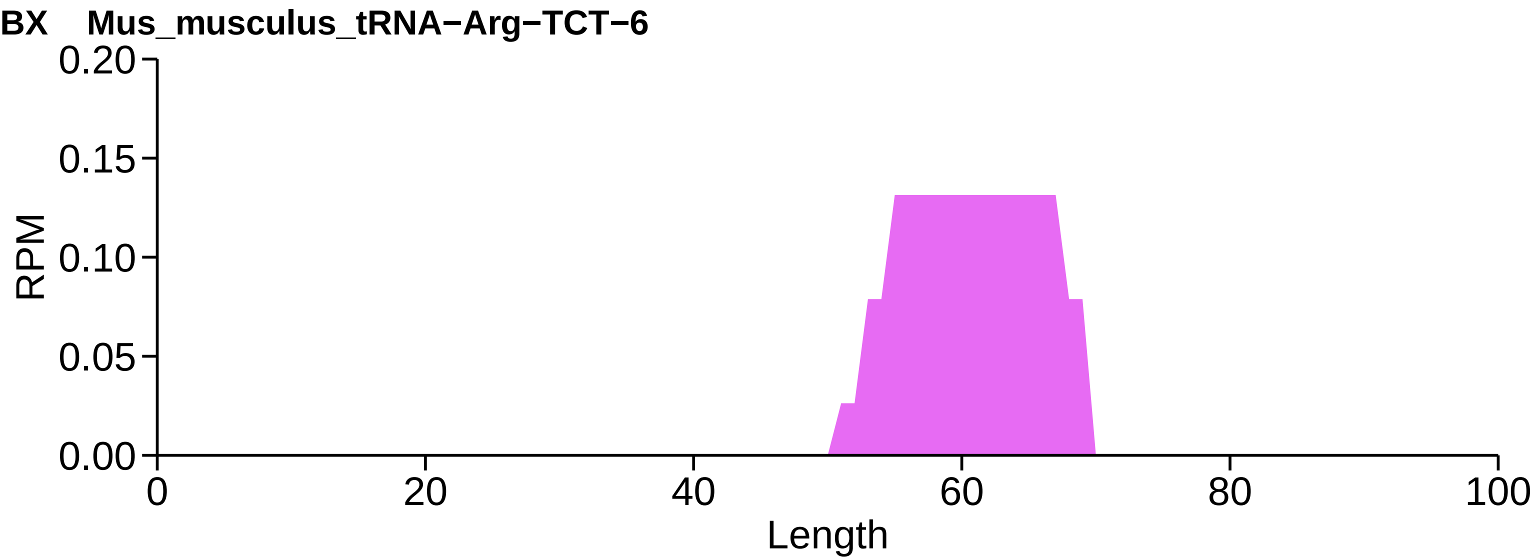
BX Mus_musculus_tRNA−Arg−TCT−6
RPM
0.00
0.05
0.10
0.15
0.20
0
20
40
60
80
100
Length

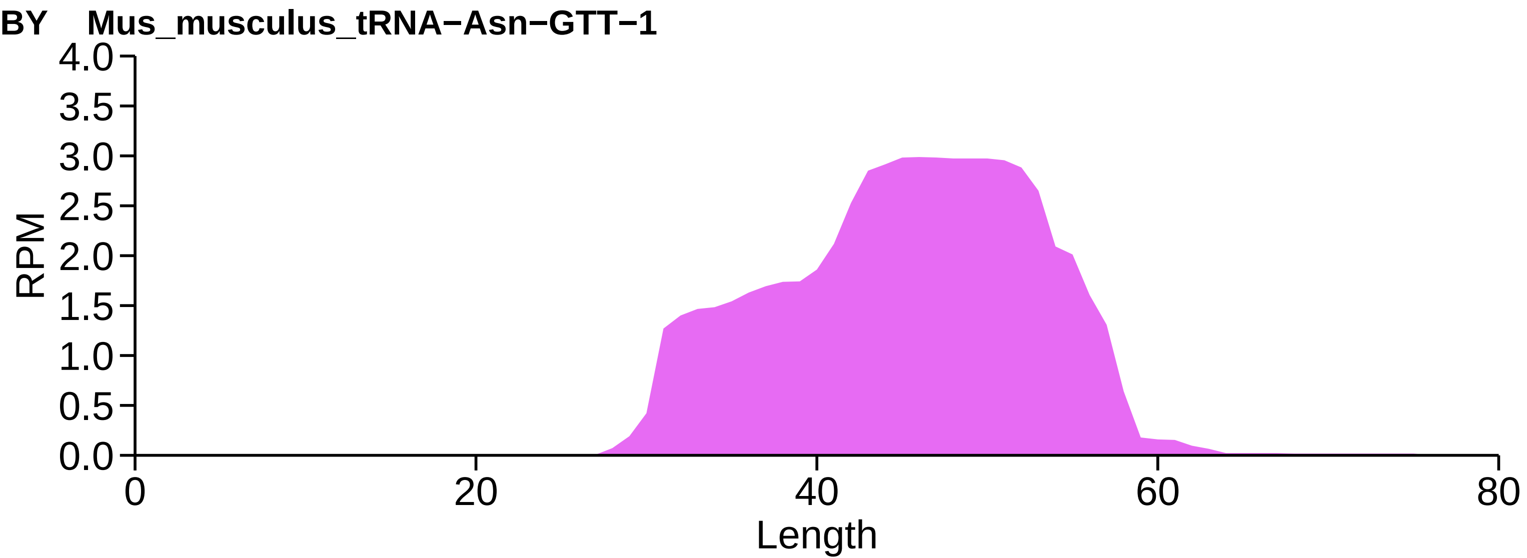

BY Mus_musculus_tRNA−Asn−GTT−1
RPM
4.0
3.5
3.0
2.5
2.0
1.5
1.0
0.5
0.0
0
20
40
60
80
Length

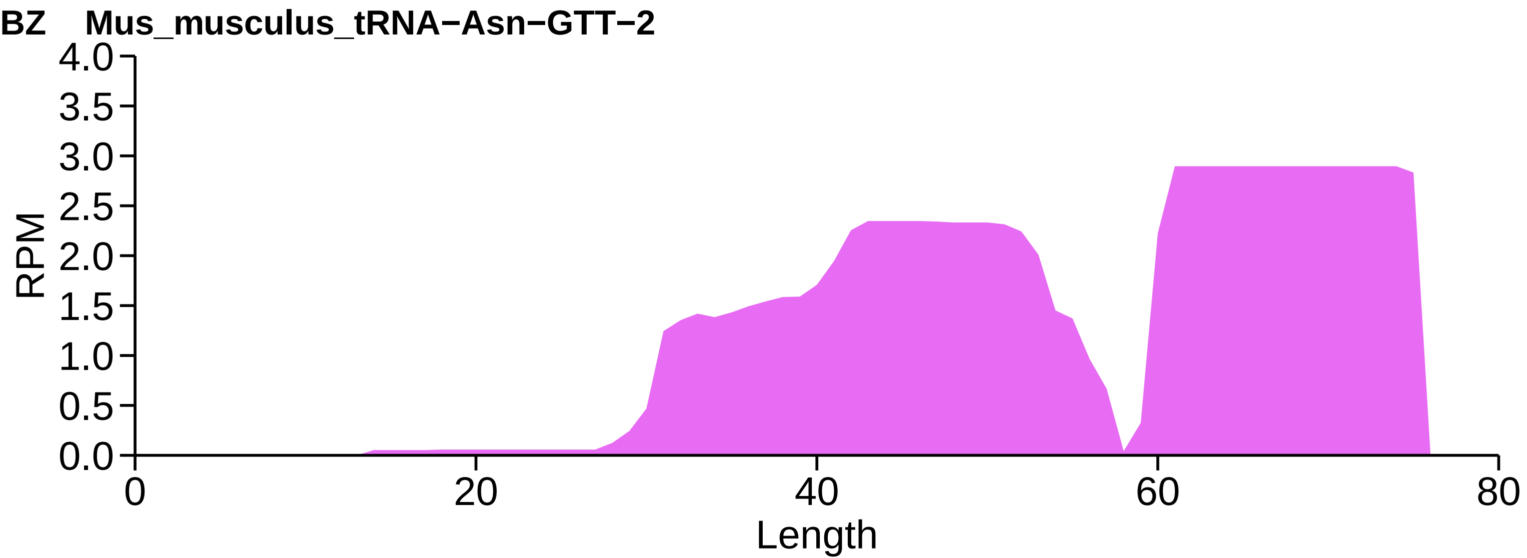
BZ Mus_musculus_tRNA−Asn−GTT−2
RPM
4.0
3.5
3.0
2.5
2.0
1.5
1.0
0.5
0.0
0
20
40
60
80
Length

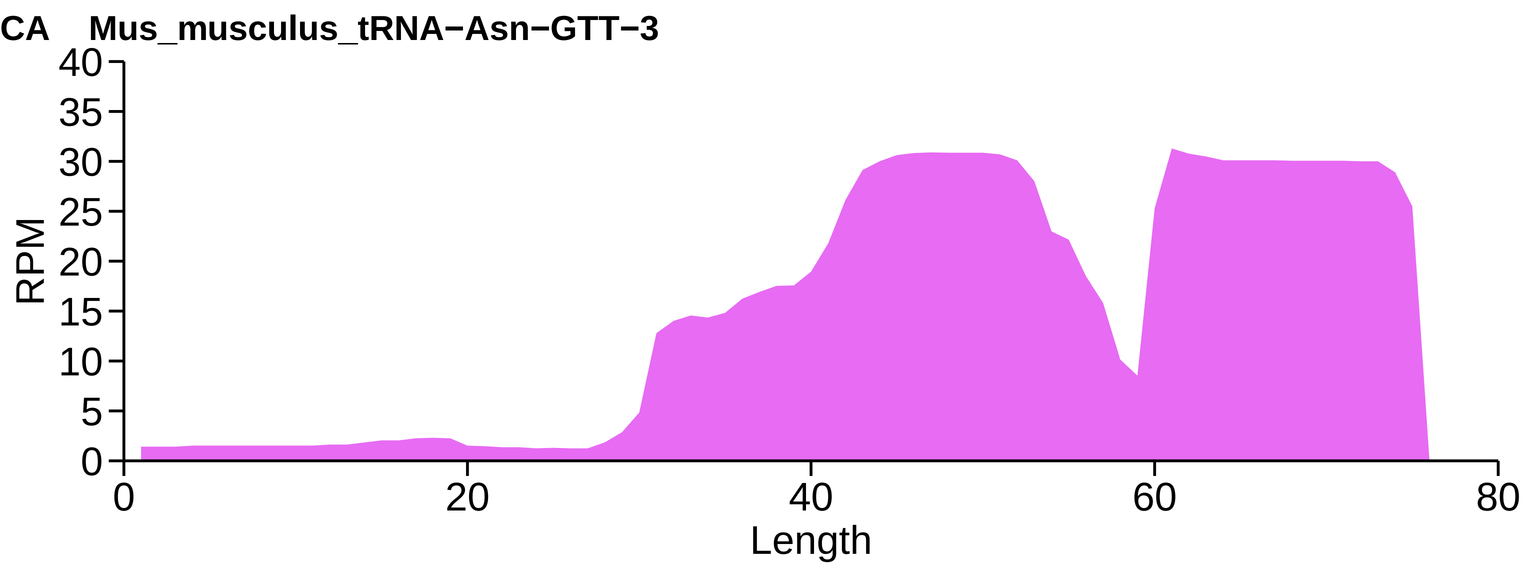

CA Mus_musculus_tRNA−Asn−GTT−3
RPM
40
35
30
25
20
15
10
5
0
0
20
40
60
80
Length

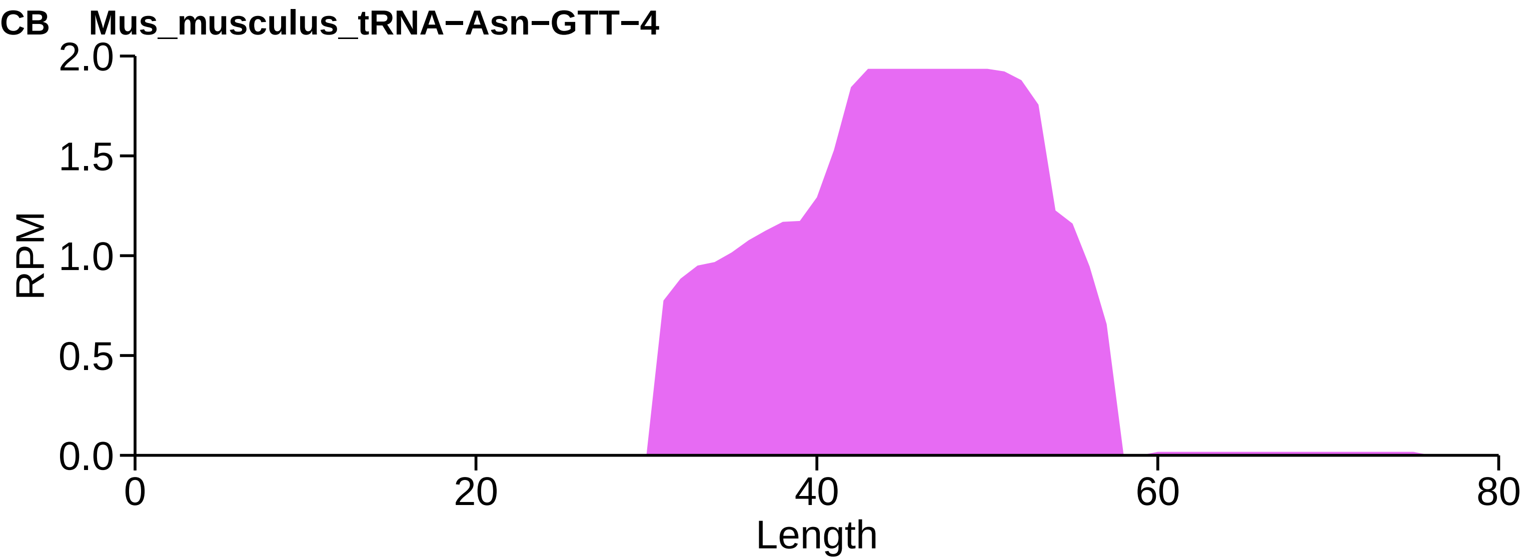

CB Mus_musculus_tRNA−Asn−GTT−4
2.0
1.5
1.0
0.5
0.0
RPM
0
20
40
60
80
Length

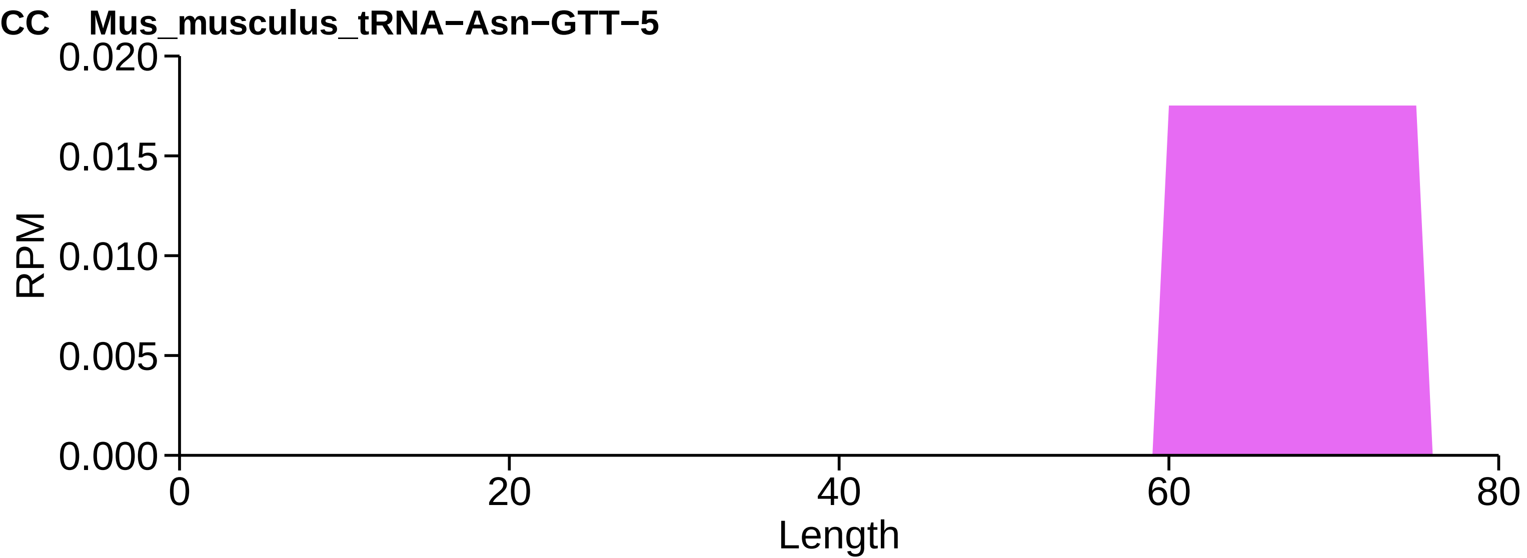
CC Mus_musculus_tRNA−Asn−GTT−5
0.020
0.015
0.010
0.005
0.000
RPM
0
20
40
60
80
Length

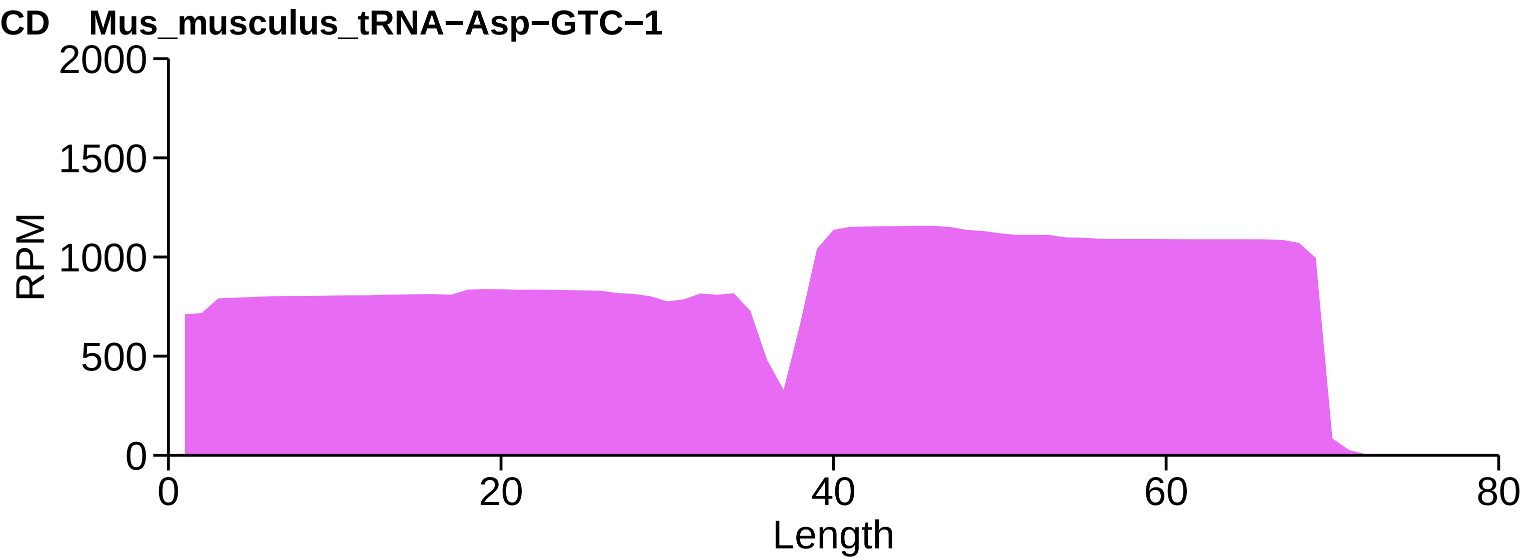

CD Mus_musculus_tRNA−Asp−GTC−1
2000
1500
1000
500
0
RPM
0
20
40
60
80
Length

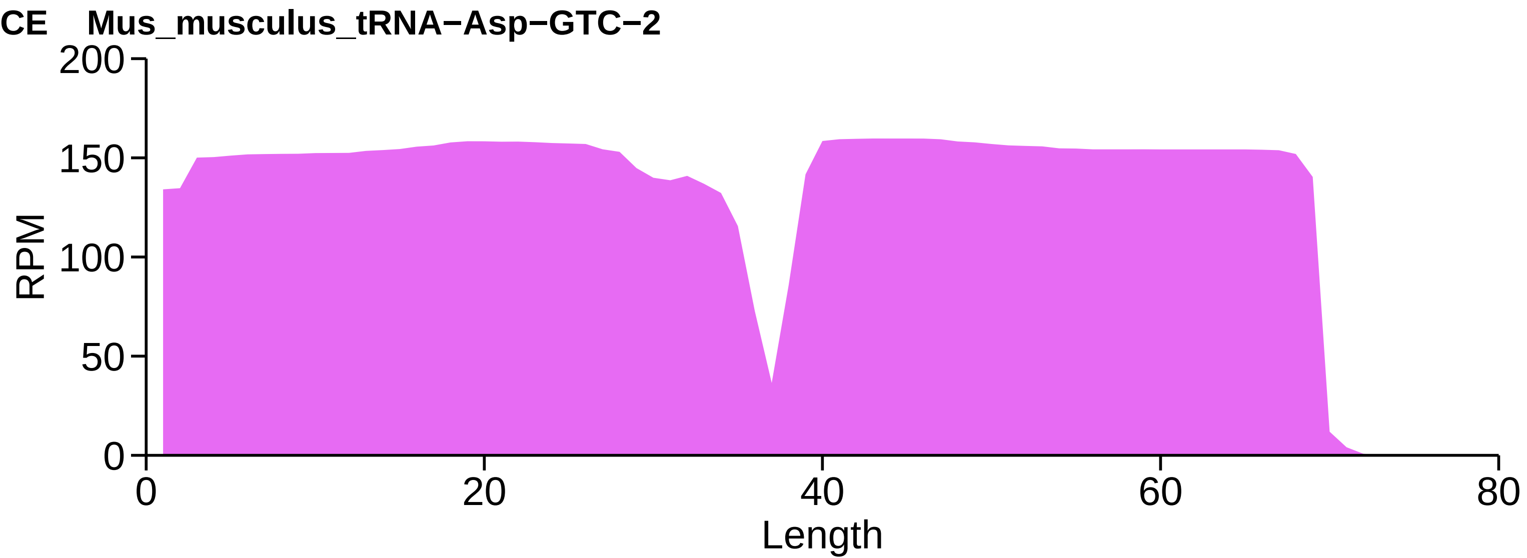

CE Mus_musculus_tRNA−Asp−GTC−2
200
150
100
50
0
RPM
0
20
40
60
80
Length

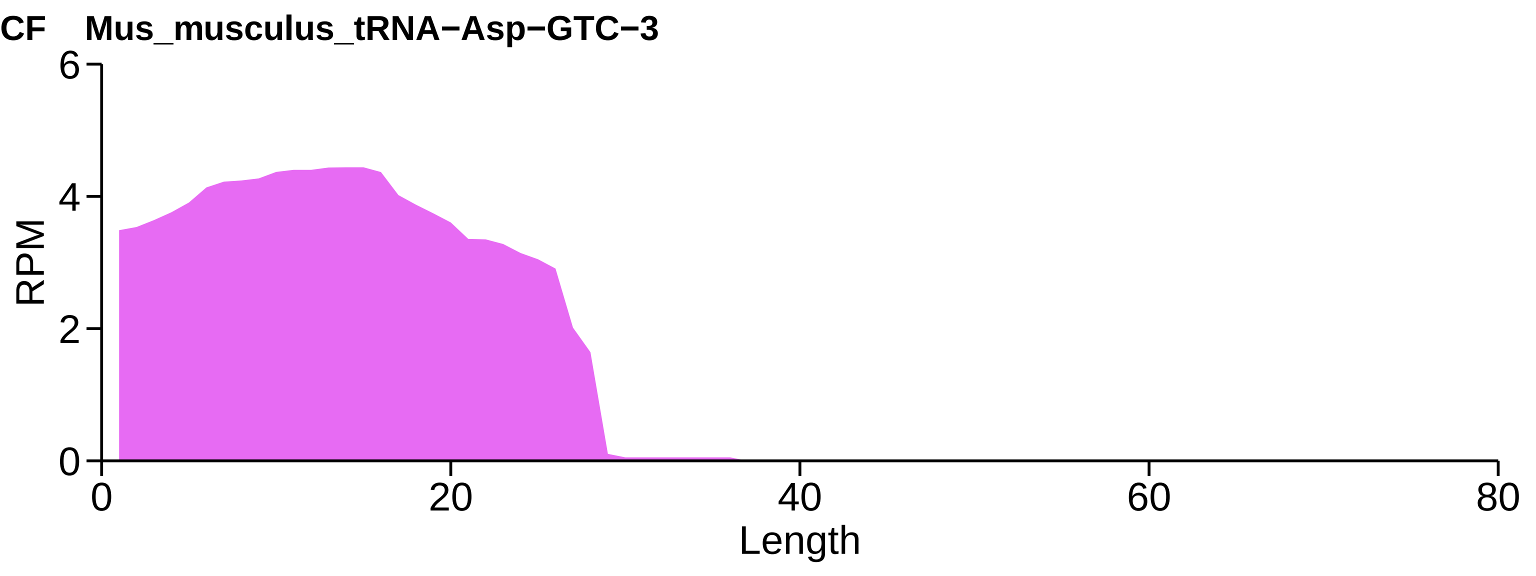
CF Mus_musculus_tRNA−Asp−GTC−3
RPM
Length
0
2
4
6
0
20
40
60
80

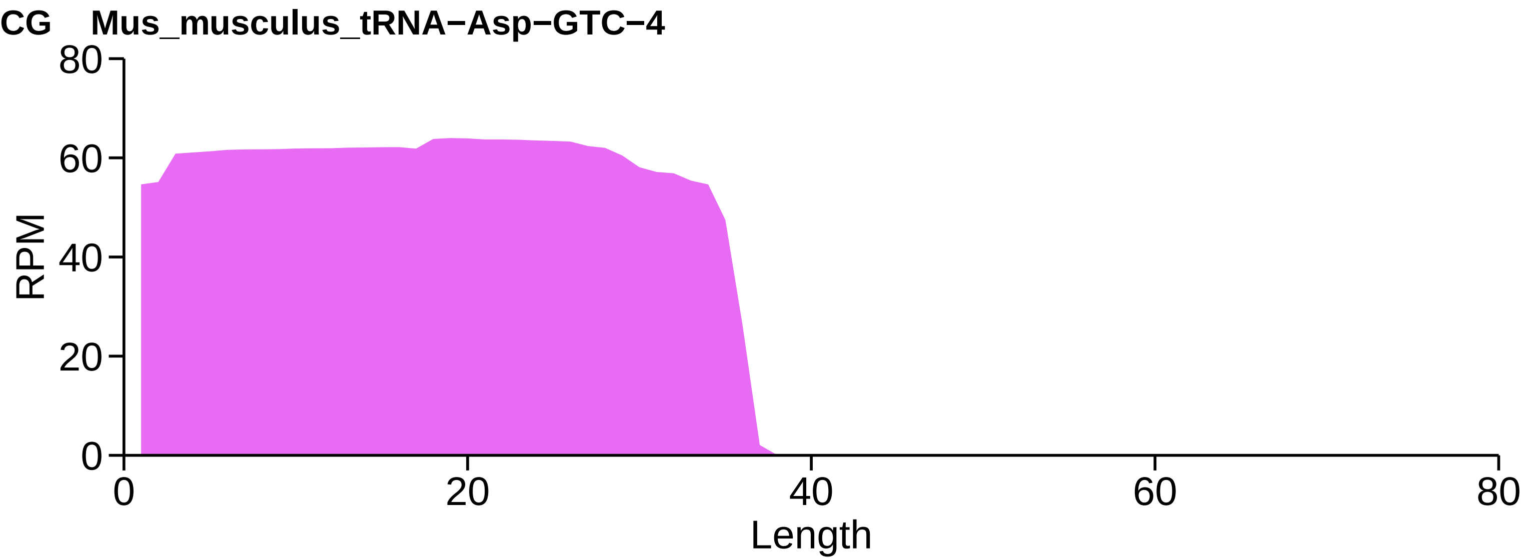

CG Mus_musculus_tRNA−Asp−GTC−4
RPM
80
60
40
20
0
0
20
40
60
80
Length

CH Mus_musculus_tRNA−Cys−ACA−1

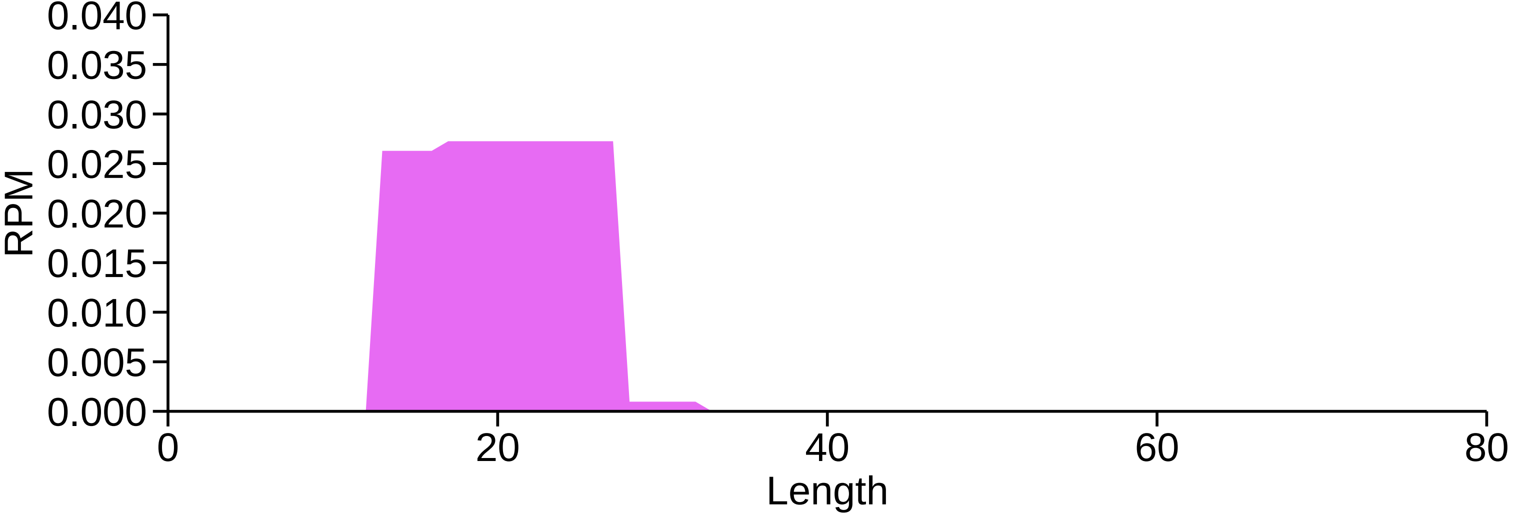

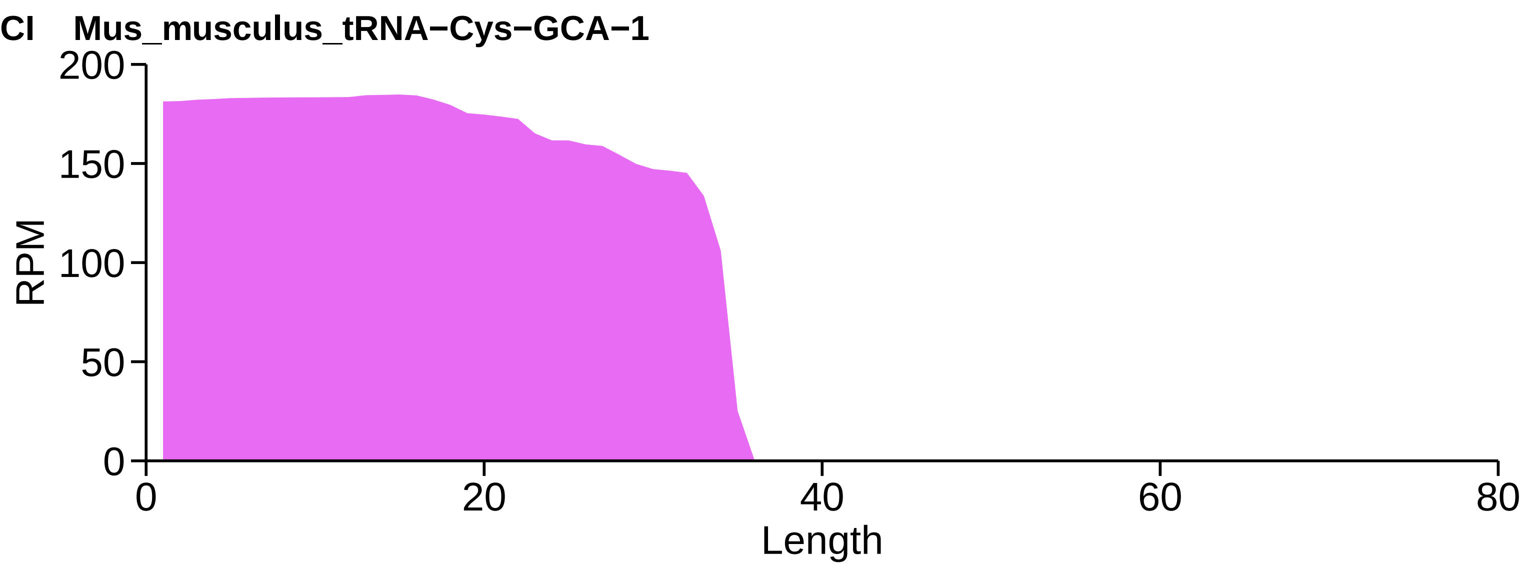
CI Mus_musculus_tRNA−Cys−GCA−1
RPM
200
150
100
50
0
0
20
40
60
80
Length

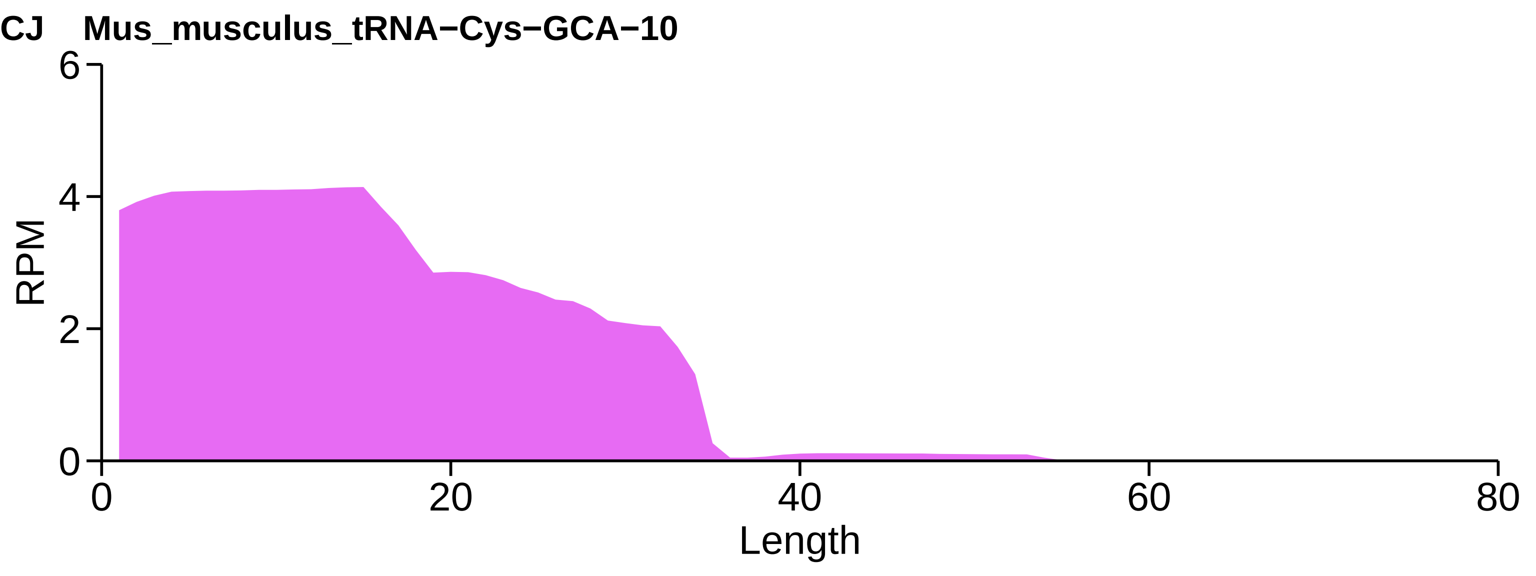
CJ Mus_musculus_tRNA−Cys−GCA−10
RPM
6
4
2
0
0
20
40
60
80
Length

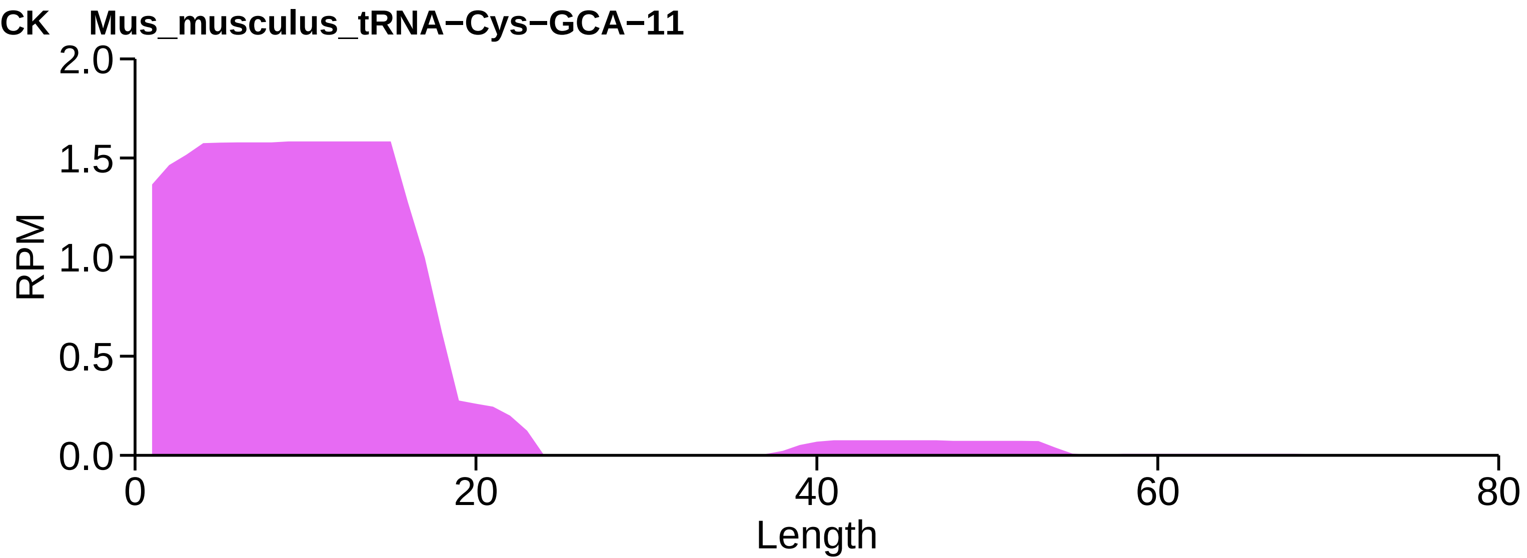
CK Mus_musculus_tRNA−Cys−GCA−11
2.0
1.5
1.0
0.5
0.0
RPM
0
20
40
60
80
Length

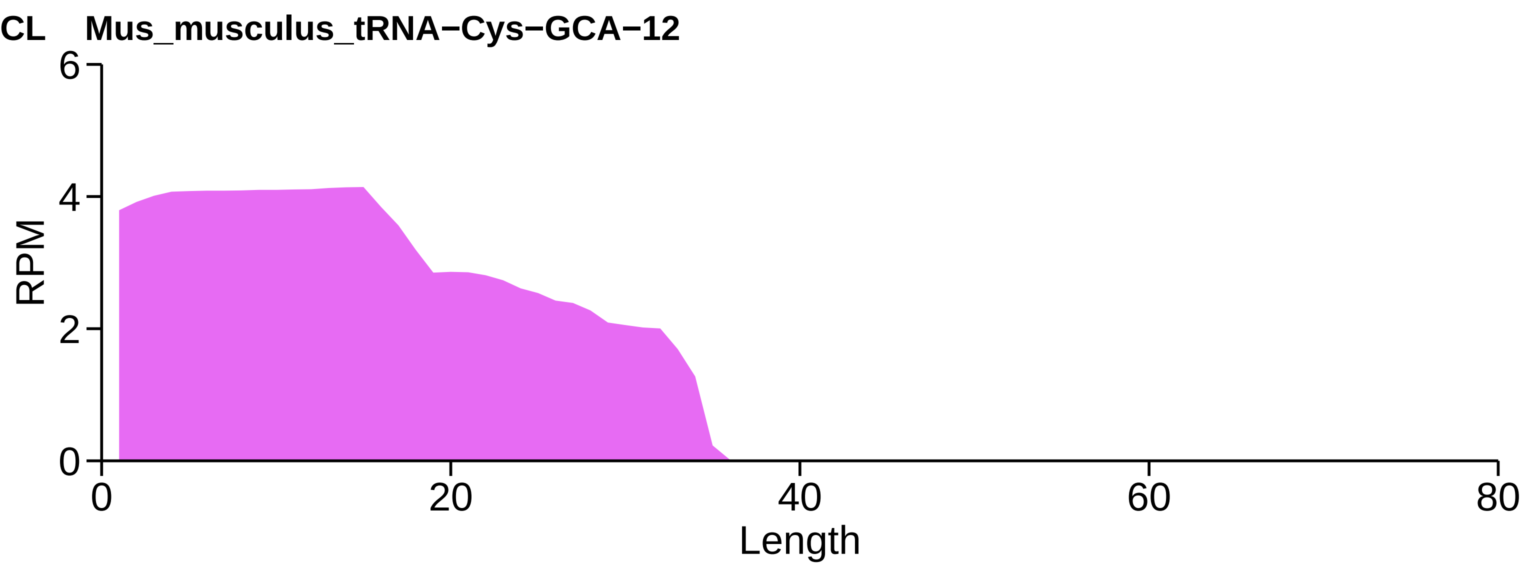

CL Mus_musculus_tRNA−Cys−GCA−12
6
4
2
0
RPM
0
20
40
60
80
Length

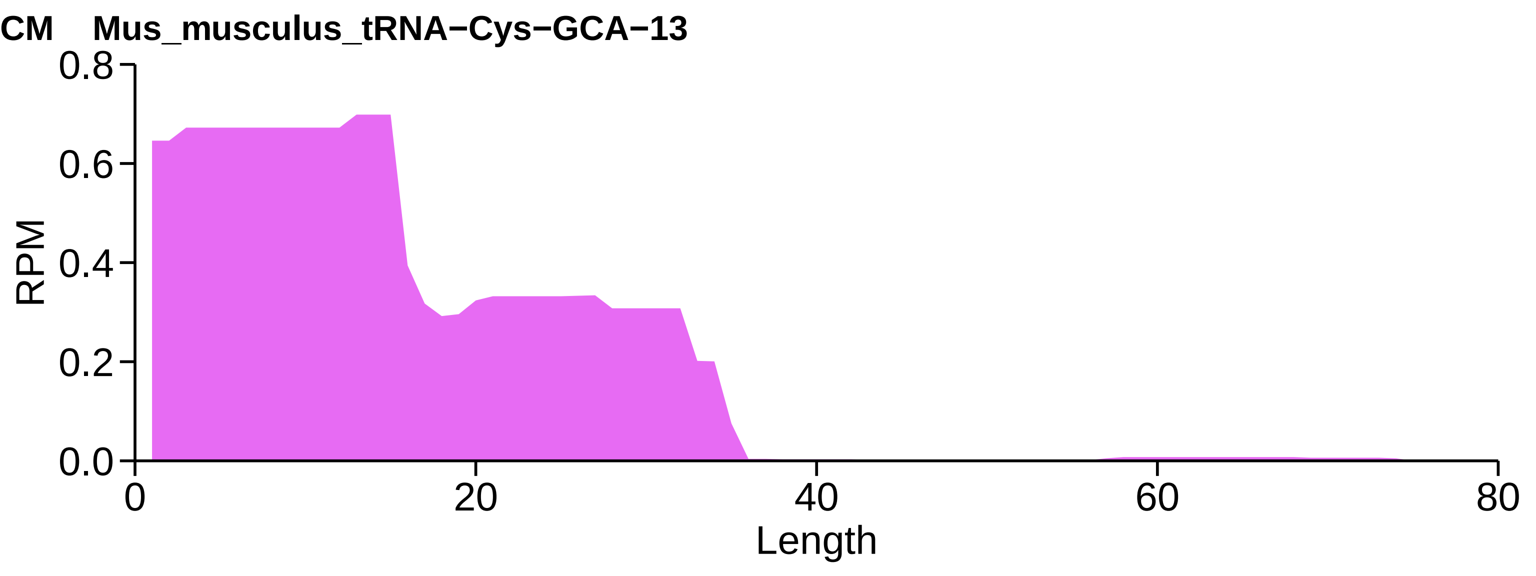
CM Mus_musculus_tRNA−Cys−GCA−13
0.8
0.6
0.4
0.2
0.0
RPM
0
20
40
60
80
Length

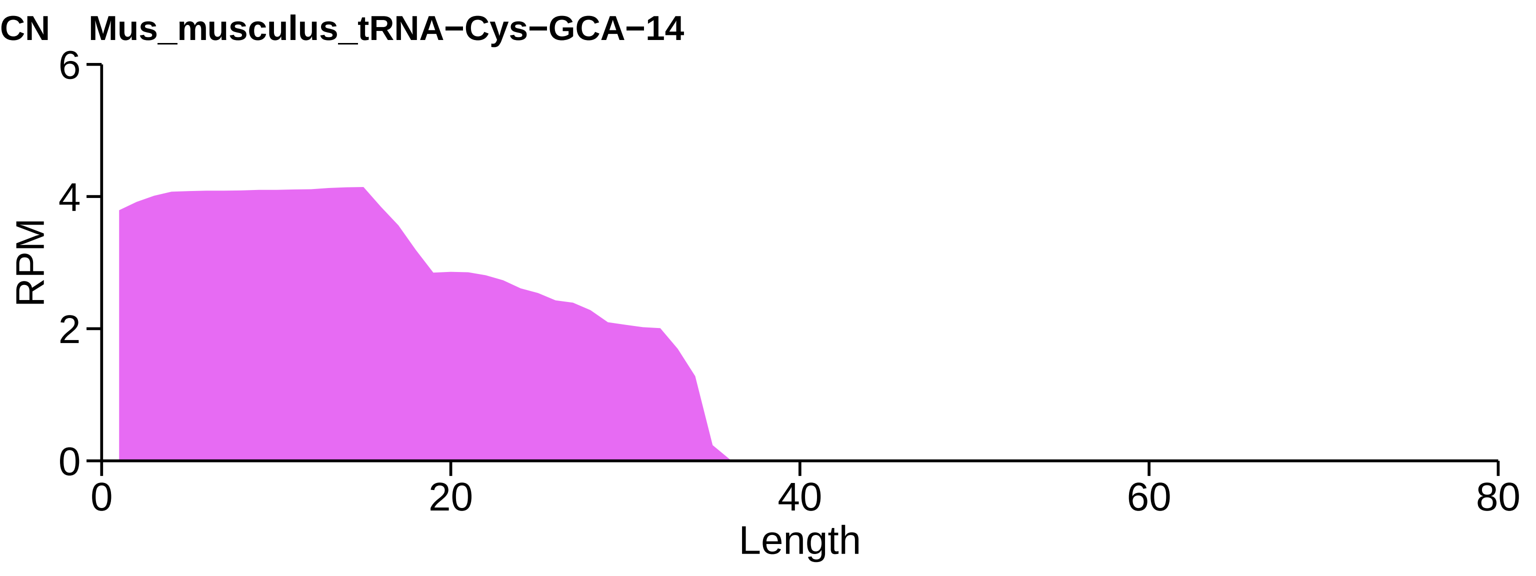

CN Mus_musculus_tRNA−Cys−GCA−14
RPM
Length
0
2
4
6
0
20
40
60
80

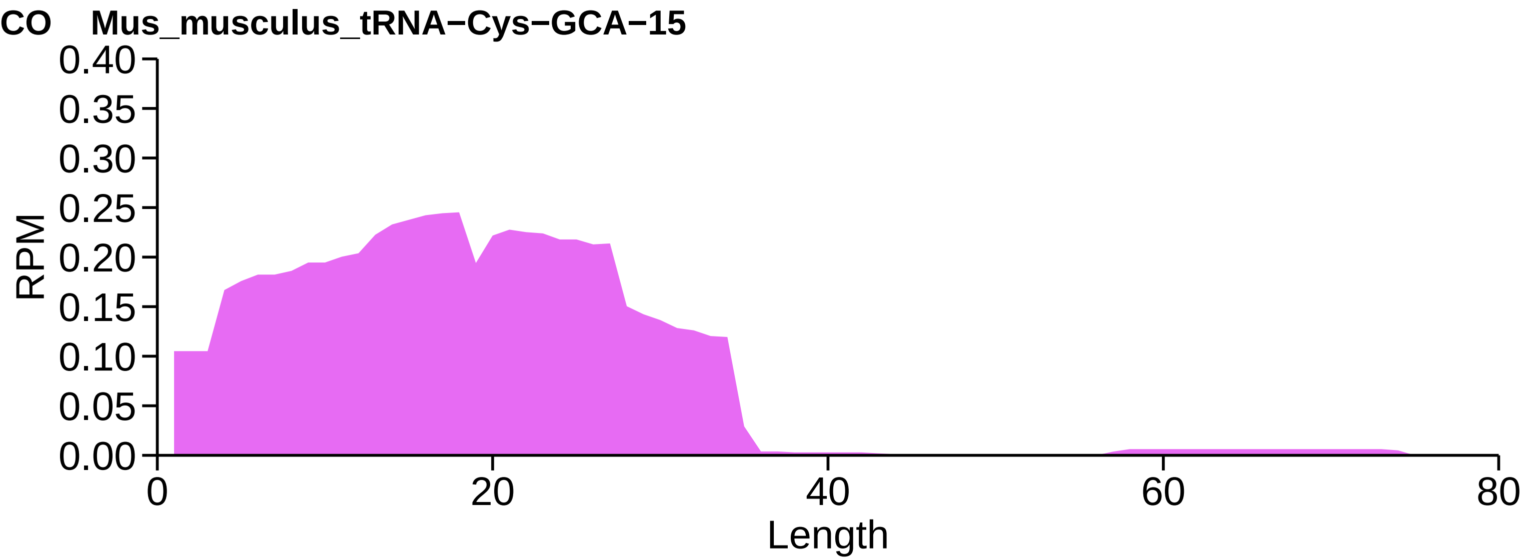
CO Mus_musculus_tRNA−Cys−GCA−15
RPM
0.00
0.05
0.10
0.15
0.20
0.25
0.30
0.35
0.40
0
20
40
60
80
Length

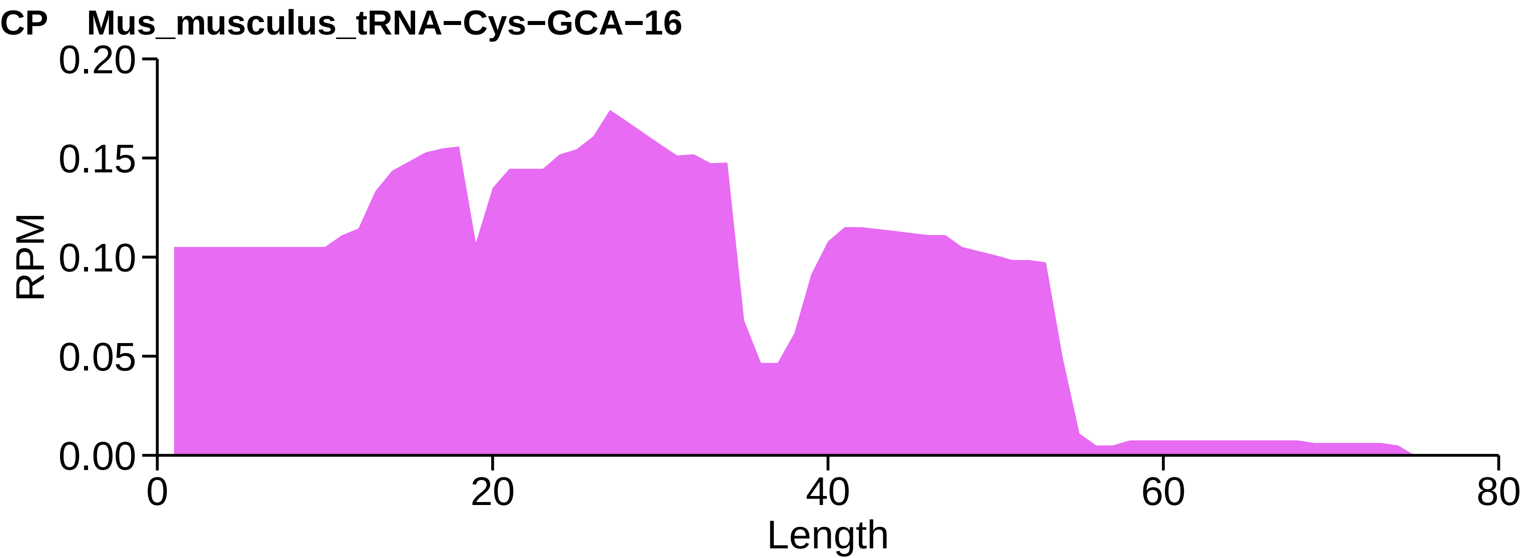

CP Mus_musculus_tRNA−Cys−GCA−16
0.20
0.15
0.10
0.05
0.00
RPM
0
20
40
60
80
Length

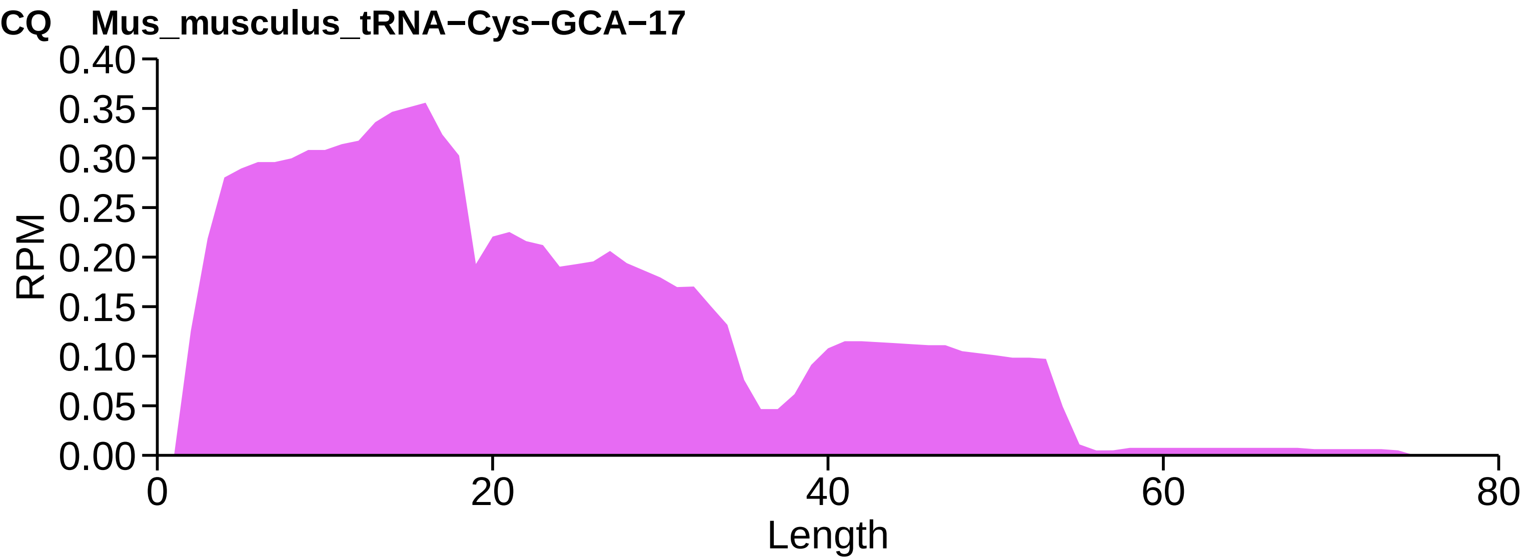
CQ Mus_musculus_tRNA−Cys−GCA−17
RPM
0.40
0.35
0.30
0.25
0.20
0.15
0.10
0.05
0.00
0
20
40
60
80
Length

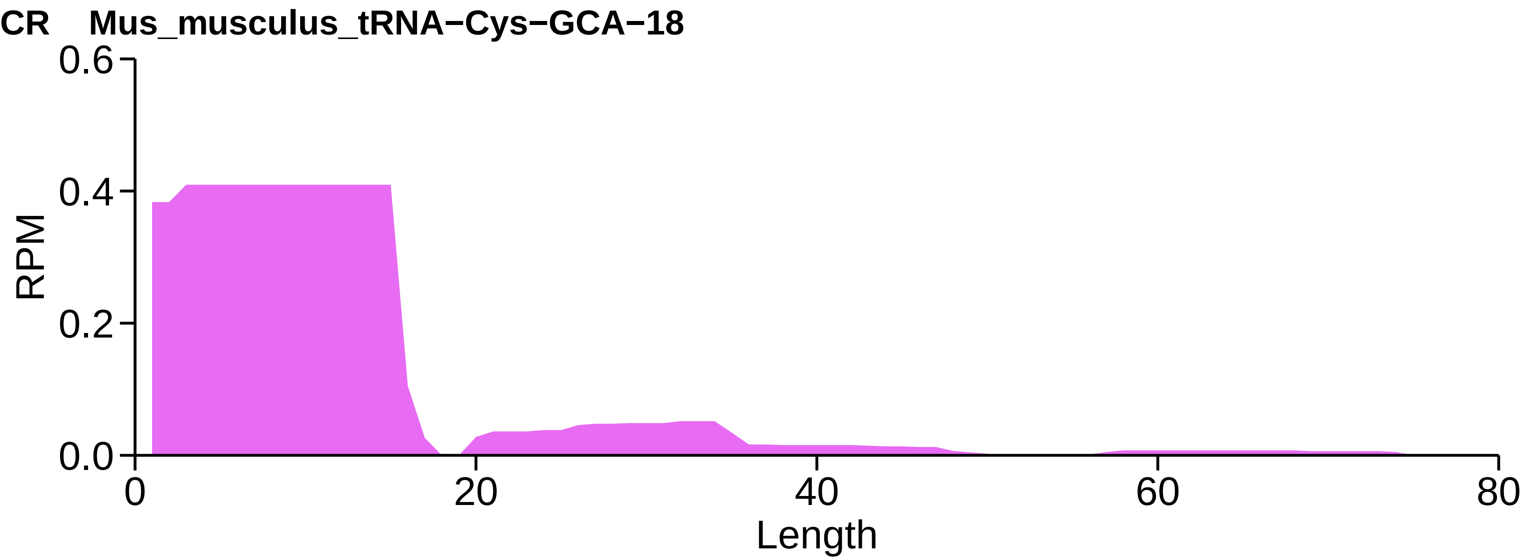
CR Mus_musculus_tRNA−Cys−GCA−18
RPM
0.6
0.4
0.2
0.0
0
20
40
60
80
Length

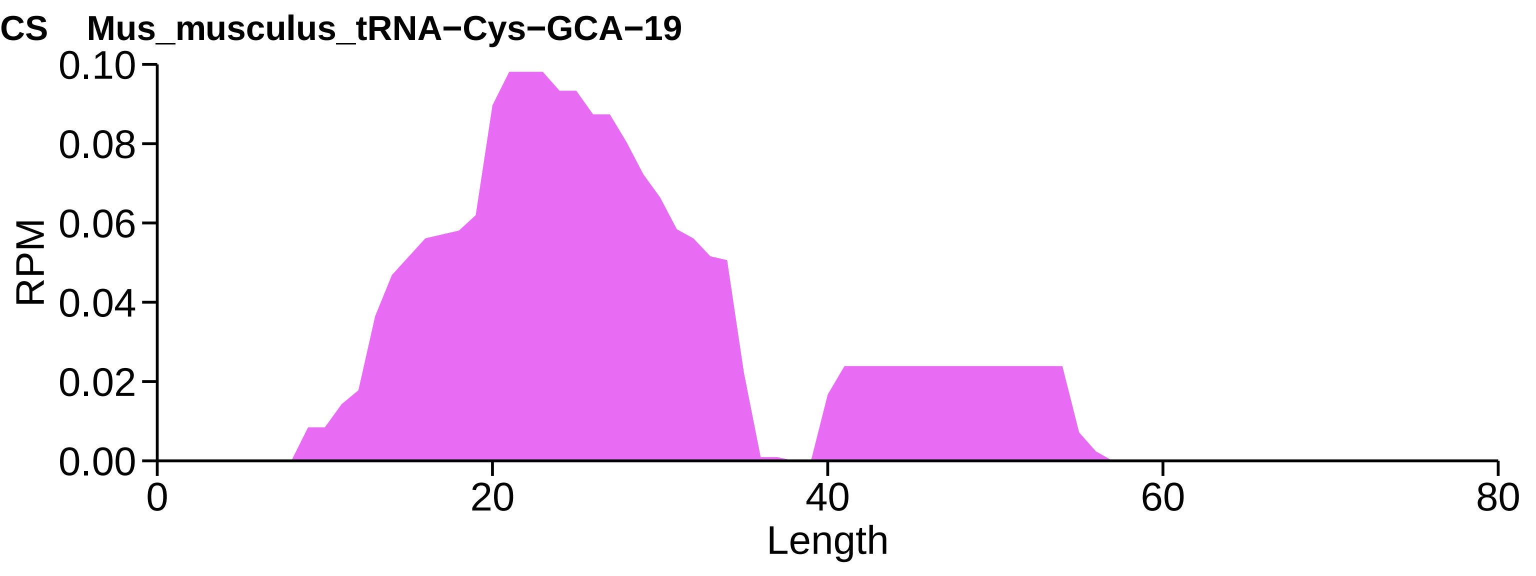
CS Mus_musculus_tRNA−Cys−GCA−19
0.10
0.08
0.06
0.04
0.02
0.00
RPM
0
20
40
60
80
Length

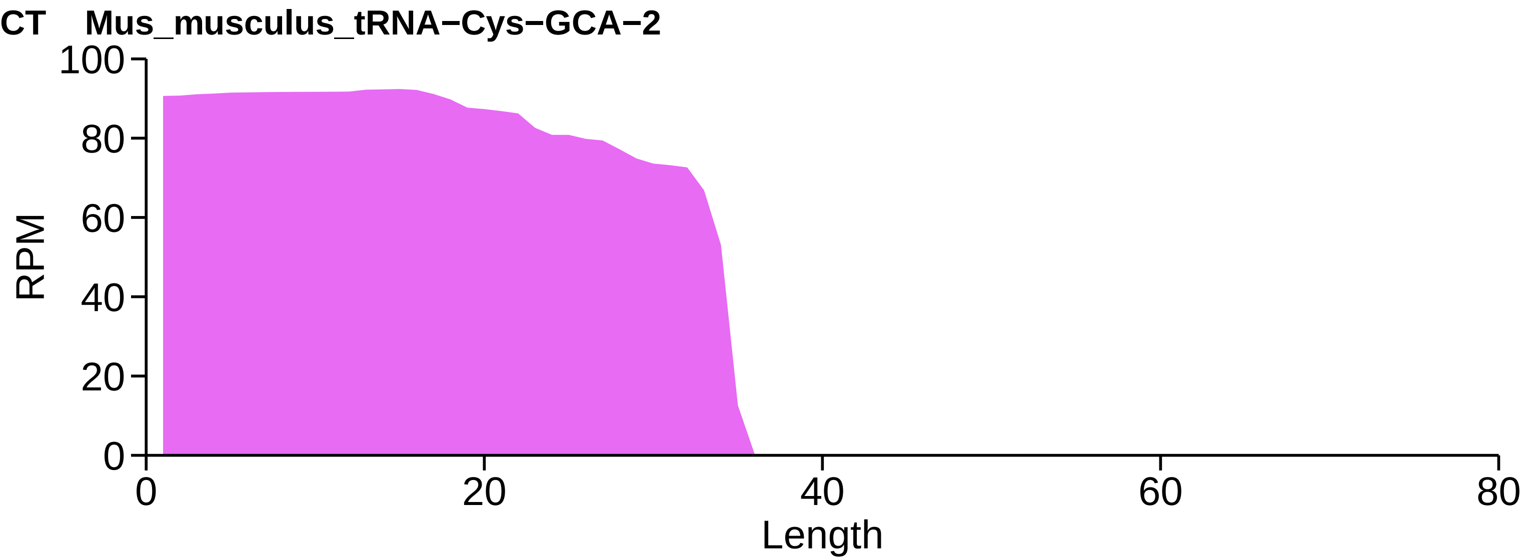

CT Mus_musculus_tRNA−Cys−GCA−2
RPM
100
80
60
40
20
0
0
20
40
60
80
Length

CU Mus_musculus_tRNA−Cys−GCA−20

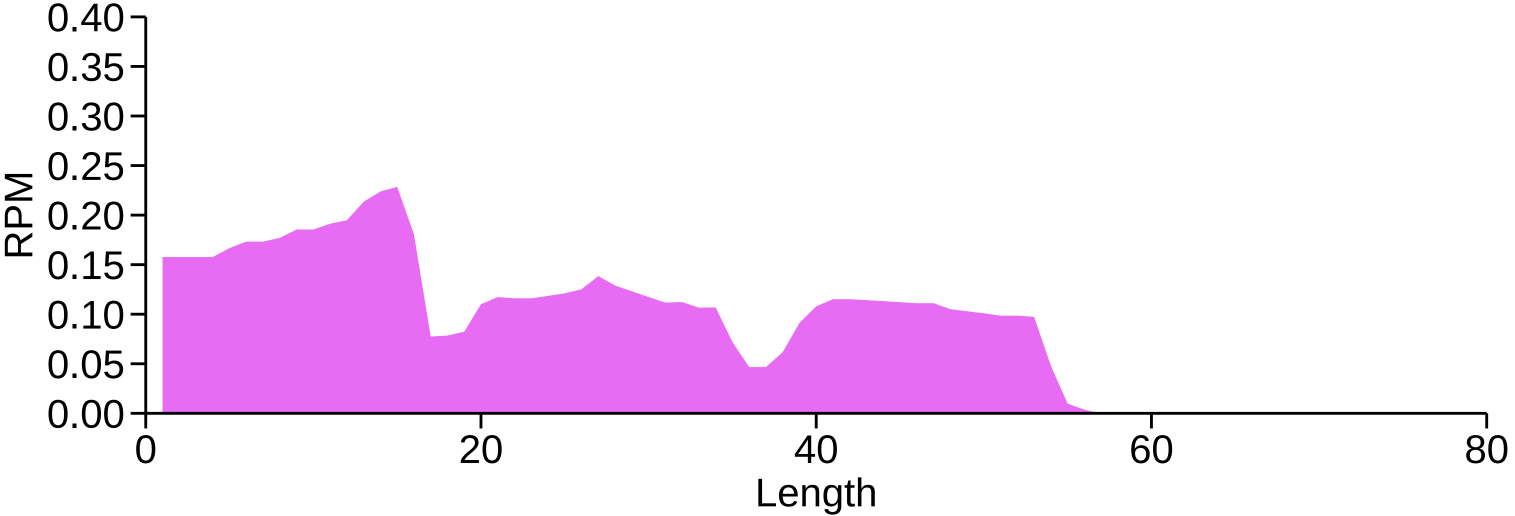

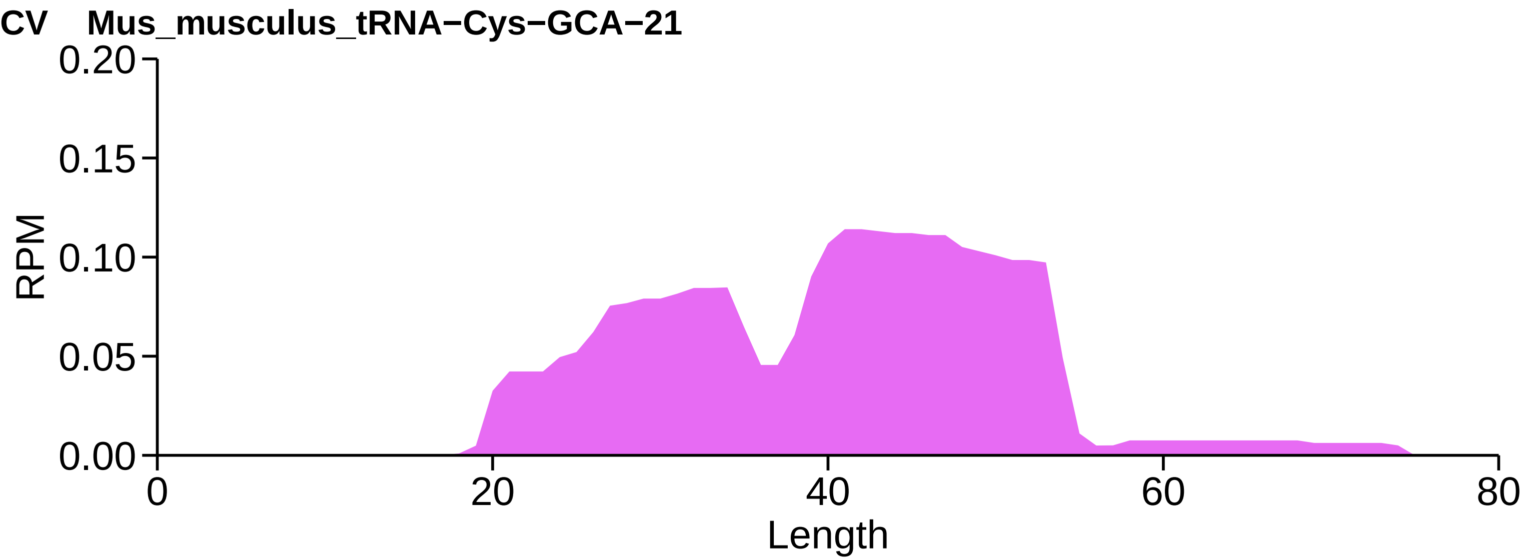
CV Mus_musculus_tRNA−Cys−GCA−21
0.20
0.15
0.10
0.05
0.00
RPM
0
20
40
60
80
Length

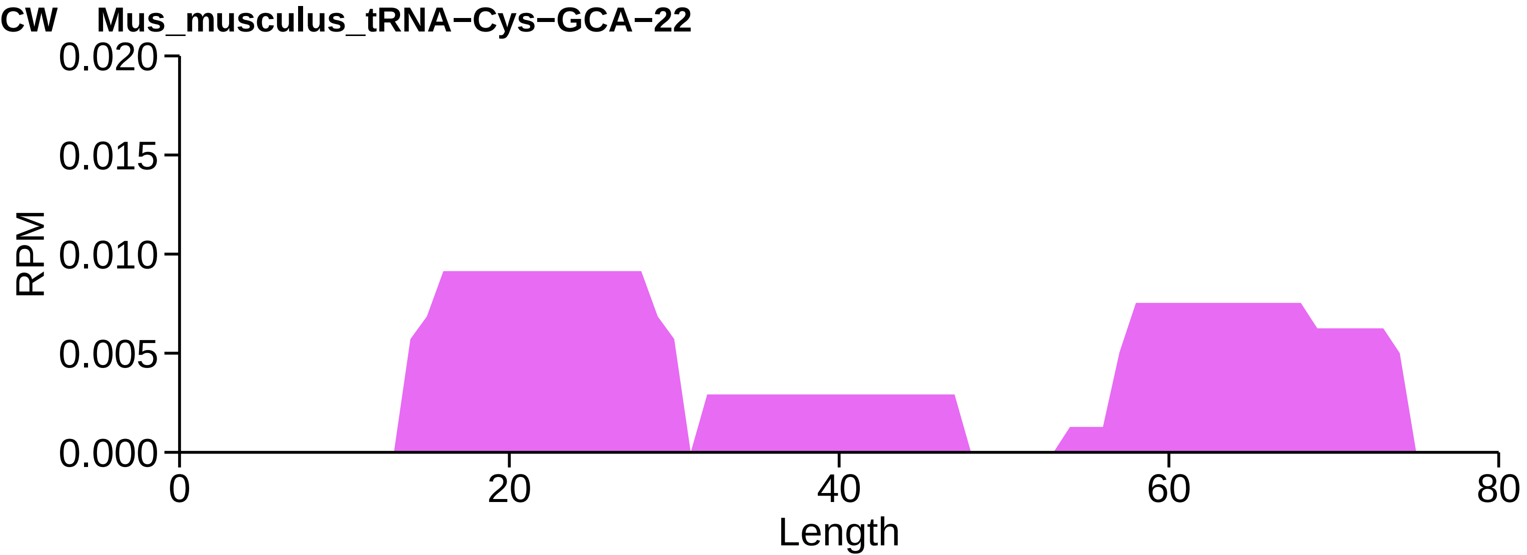
CW Mus_musculus_tRNA−Cys−GCA−22
RPM
0.020
0.015
0.010
0.005
0.000
0
20
40
60
80
Length

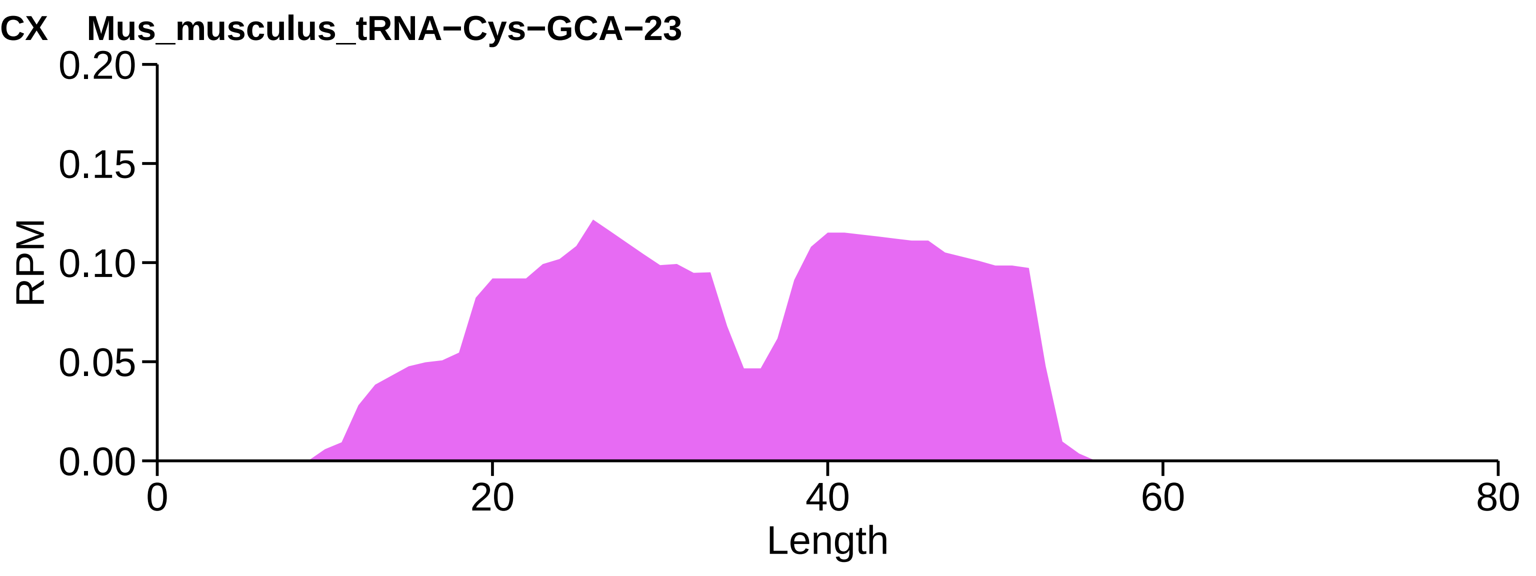

CX Mus_musculus_tRNA−Cys−GCA−23
RPM
0.00
0.05
0.10
0.15
0.20
0
20
40
60
80
Length

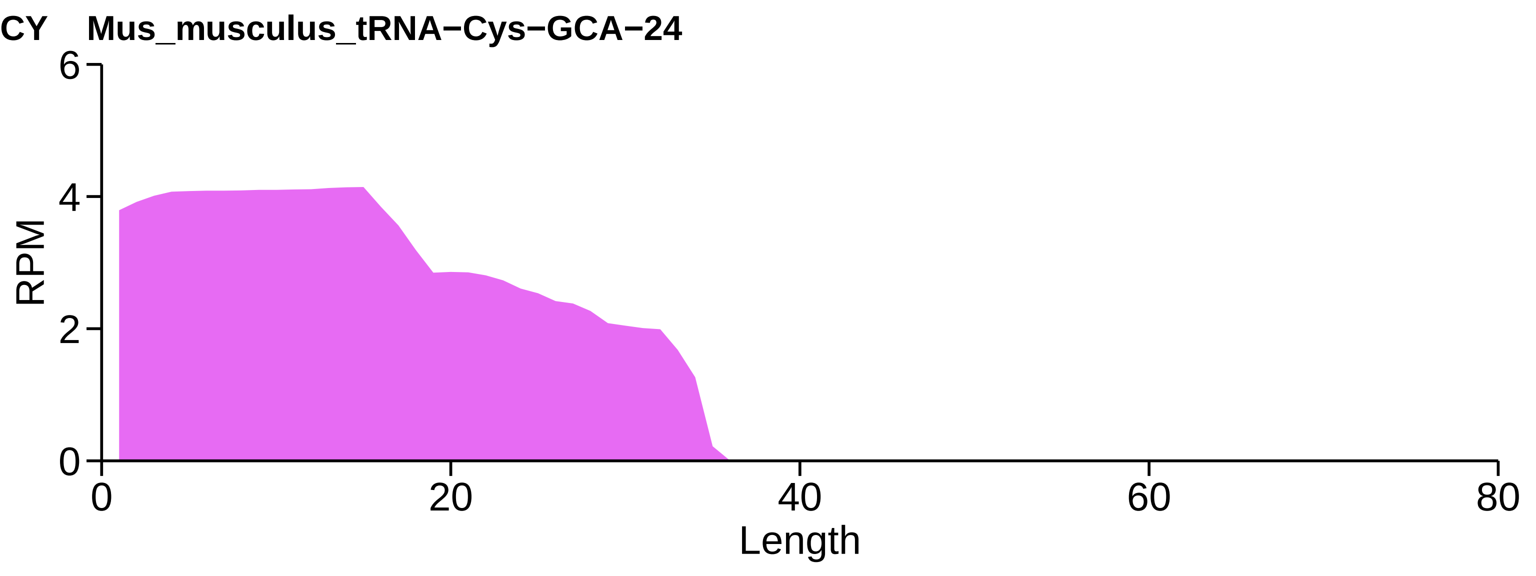

CY Mus_musculus_tRNA−Cys−GCA−24
6
4
2
0
RPM
0
20
40
60
80
Length

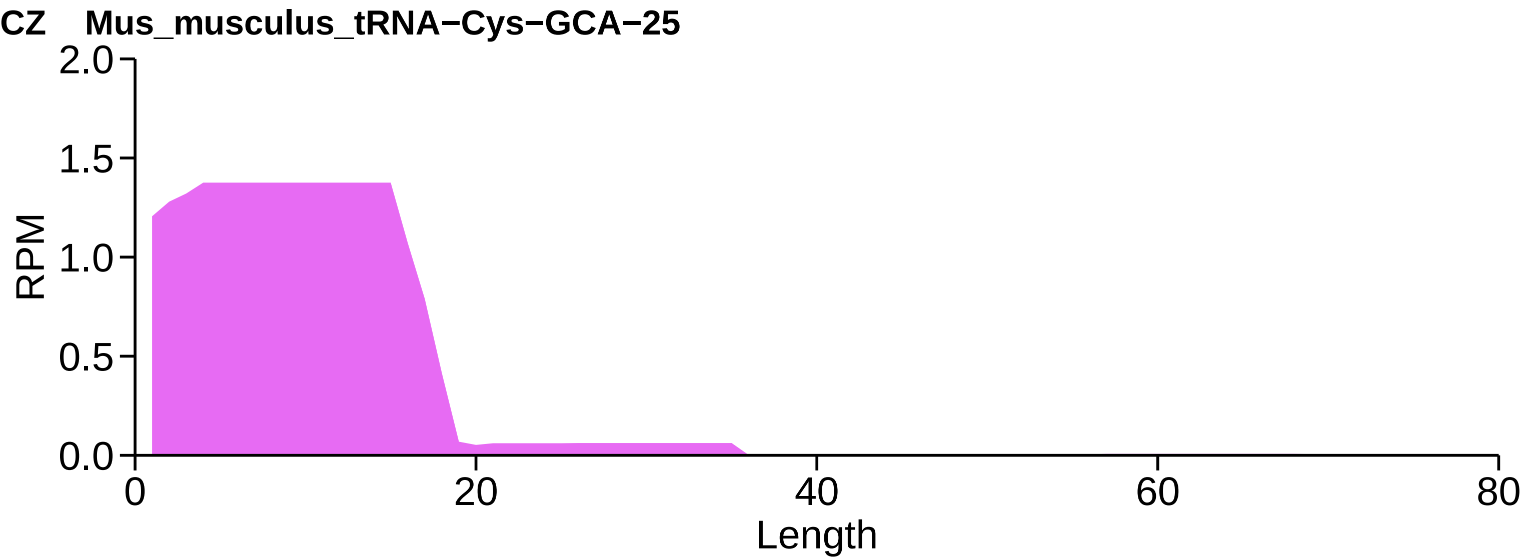
CZ Mus_musculus_tRNA−Cys−GCA−25
RPM
2.0
1.5
1.0
0.5
0.0
0
20
40
60
80
Length

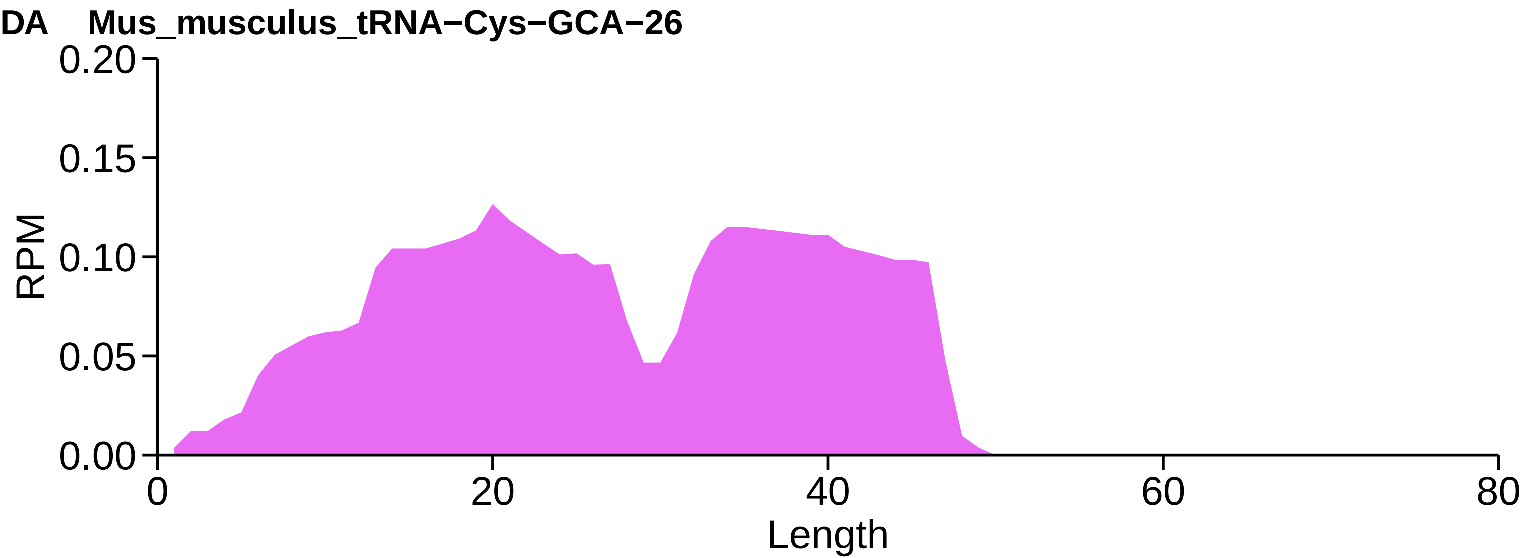
DA Mus_musculus_tRNA−Cys−GCA−26
0.20
0.15
0.10
0.05
0.00
RPM
0
20
40
60
80
Length

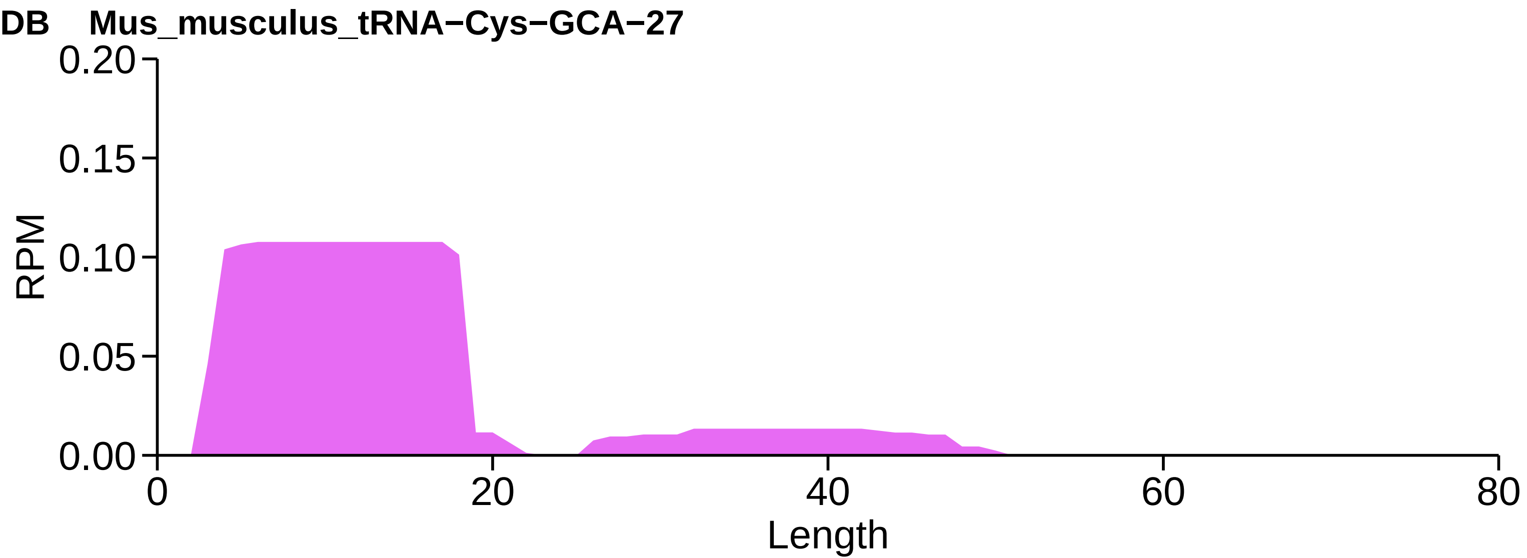
DB Mus_musculus_tRNA−Cys−GCA−27
RPM
0.00
0.05
0.10
0.15
0.20
0
20
40
60
80
Length

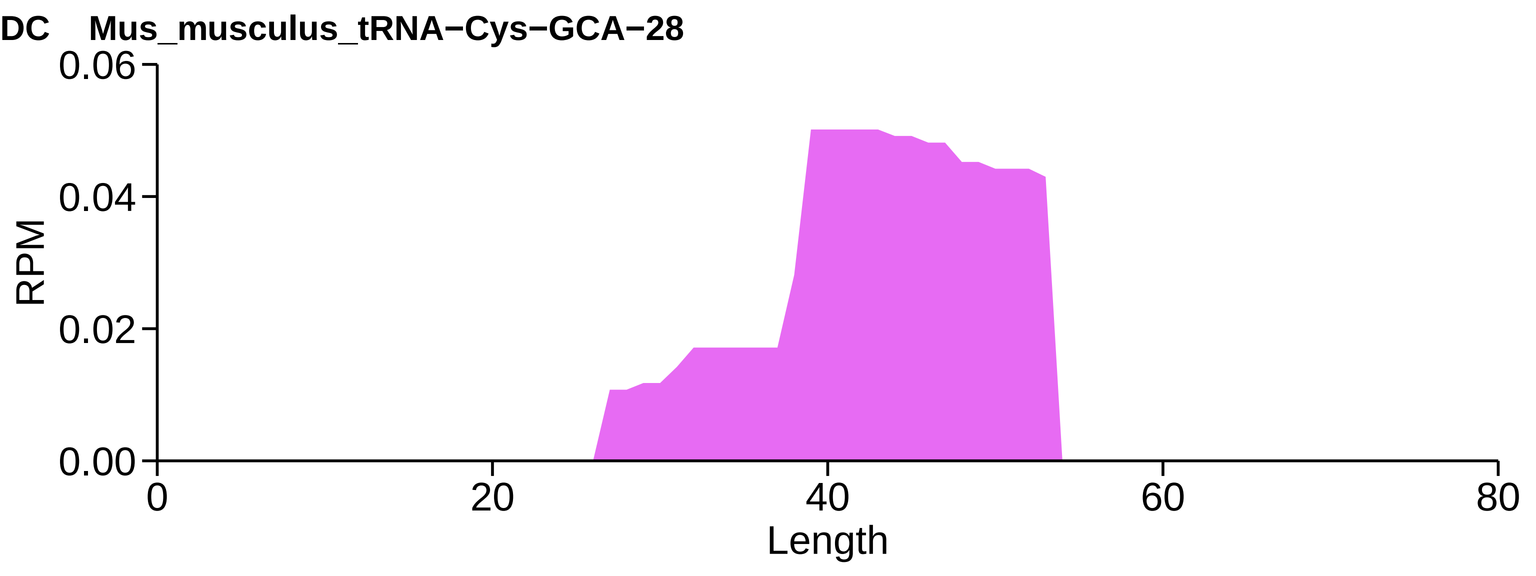
DC Mus_musculus_tRNA−Cys−GCA−28
RPM
0.06
0.04
0.02
0.00
0
20
40
60
80
Length

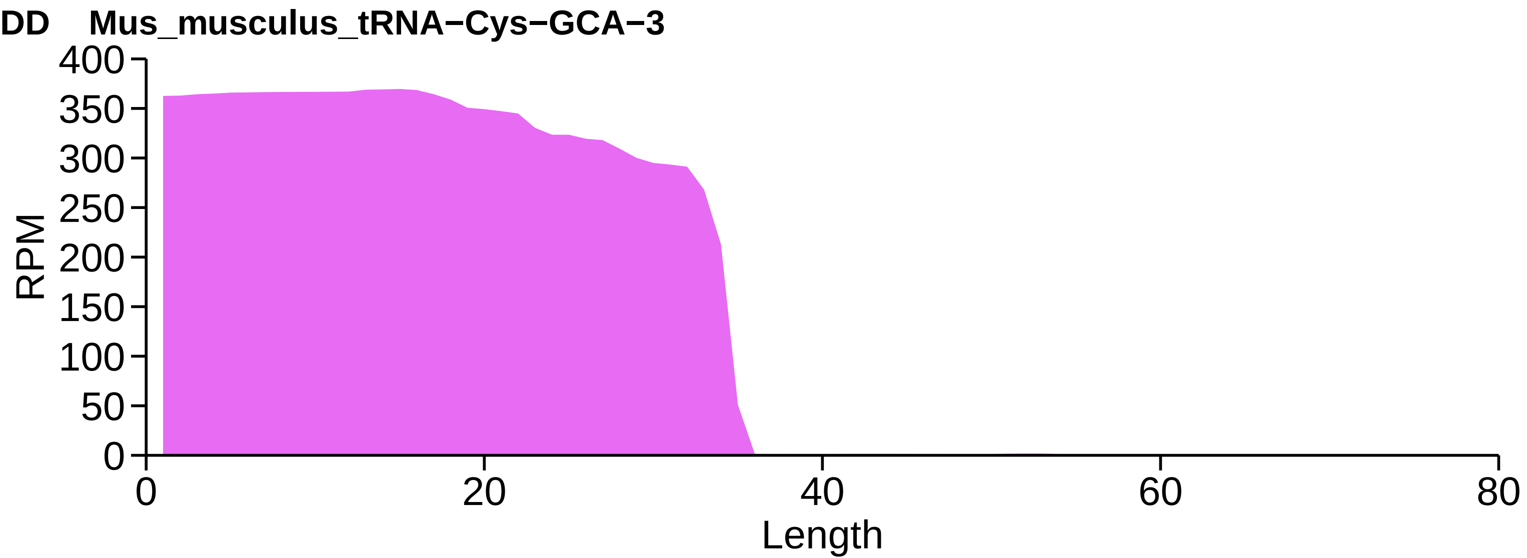

DD Mus_musculus_tRNA−Cys−GCA−3
400
350
300
250
200
150
100
50
0
RPM
0
20
40
60
80
Length

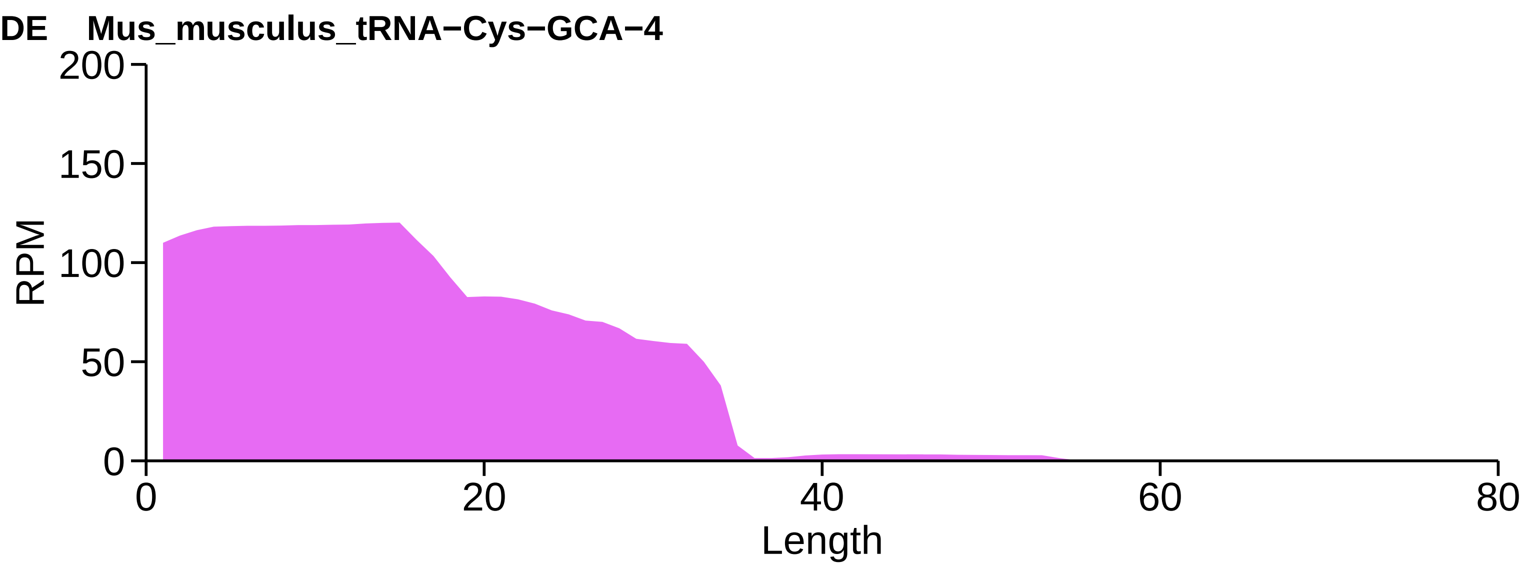
DE Mus_musculus_tRNA−Cys−GCA−4
200
150
100
50
0
RPM
0
20
40
60
80
Length

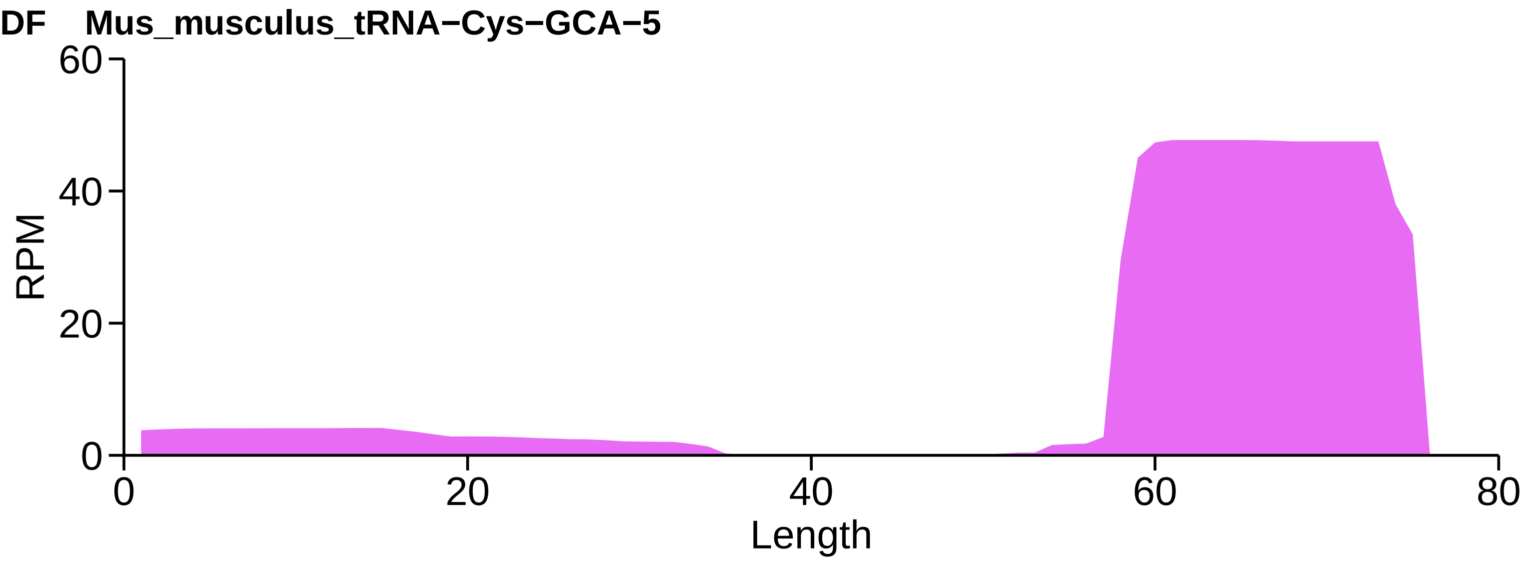
DF Mus_musculus_tRNA−Cys−GCA−5
RPM
0
20
40
60
0
20
40
60
80
Length

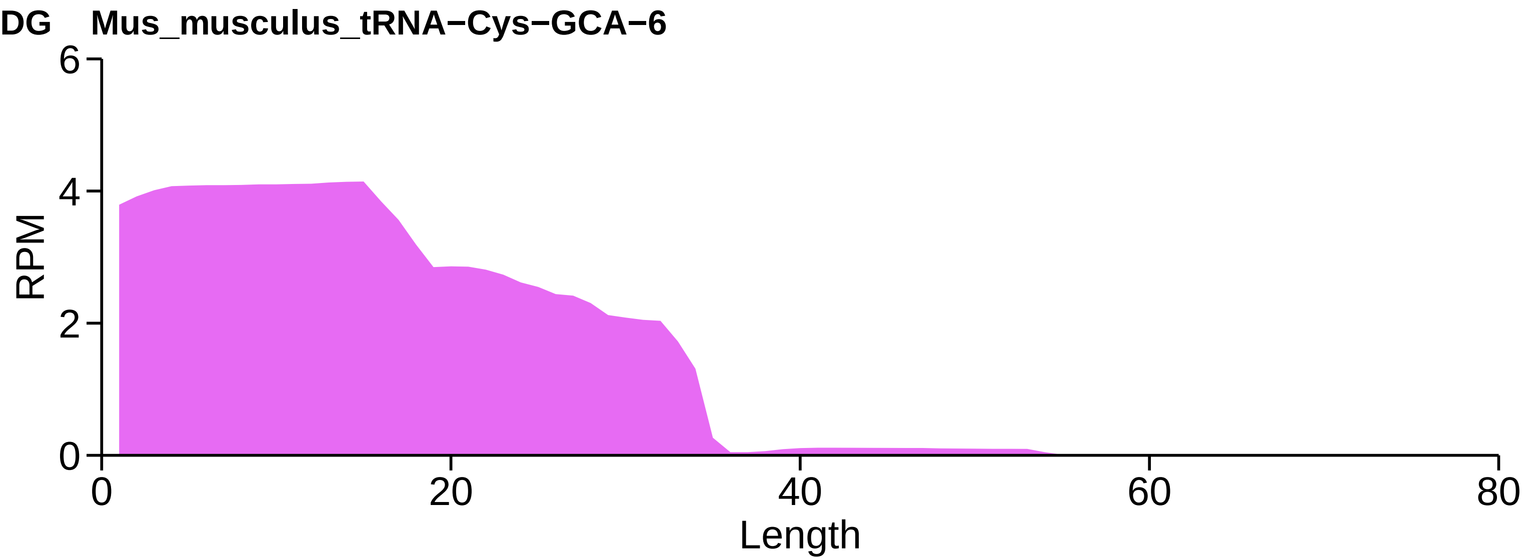
DG Mus_musculus_tRNA−Cys−GCA−6
RPM
0
2
4
6
0
20
40
60
80
Length

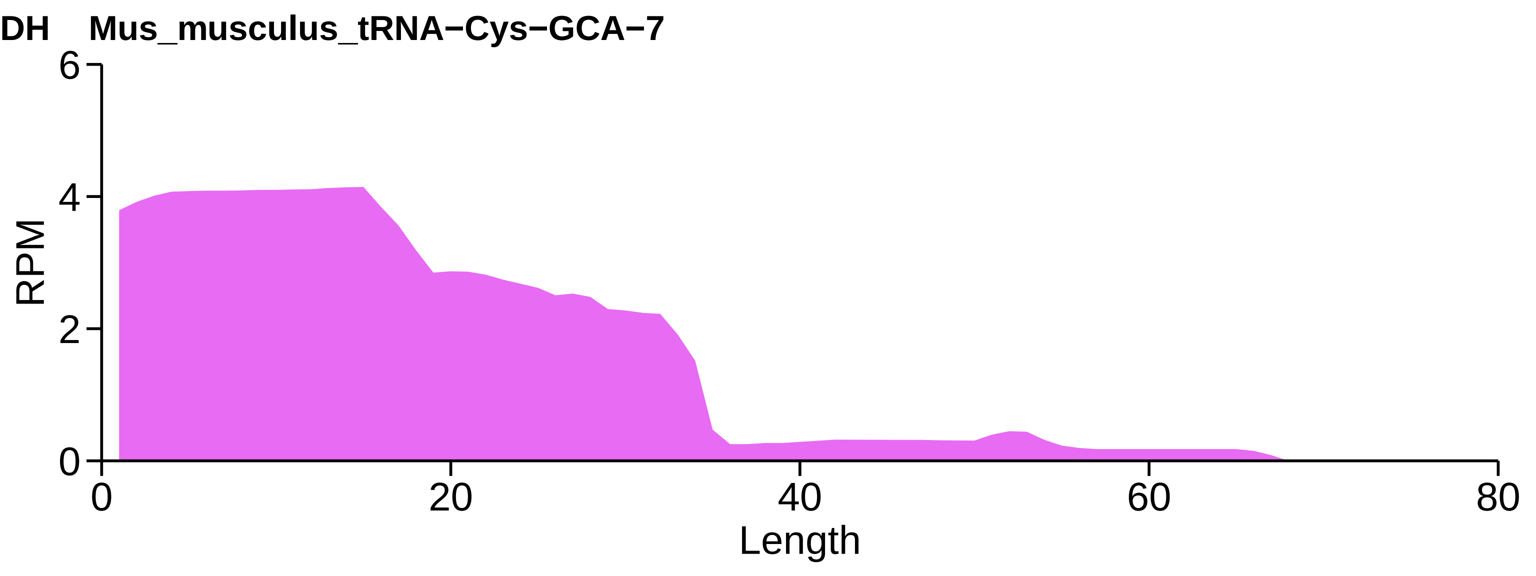
DH Mus_musculus_tRNA−Cys−GCA−7
RPM
0
2
4
6
Length
0
20
40
60
80

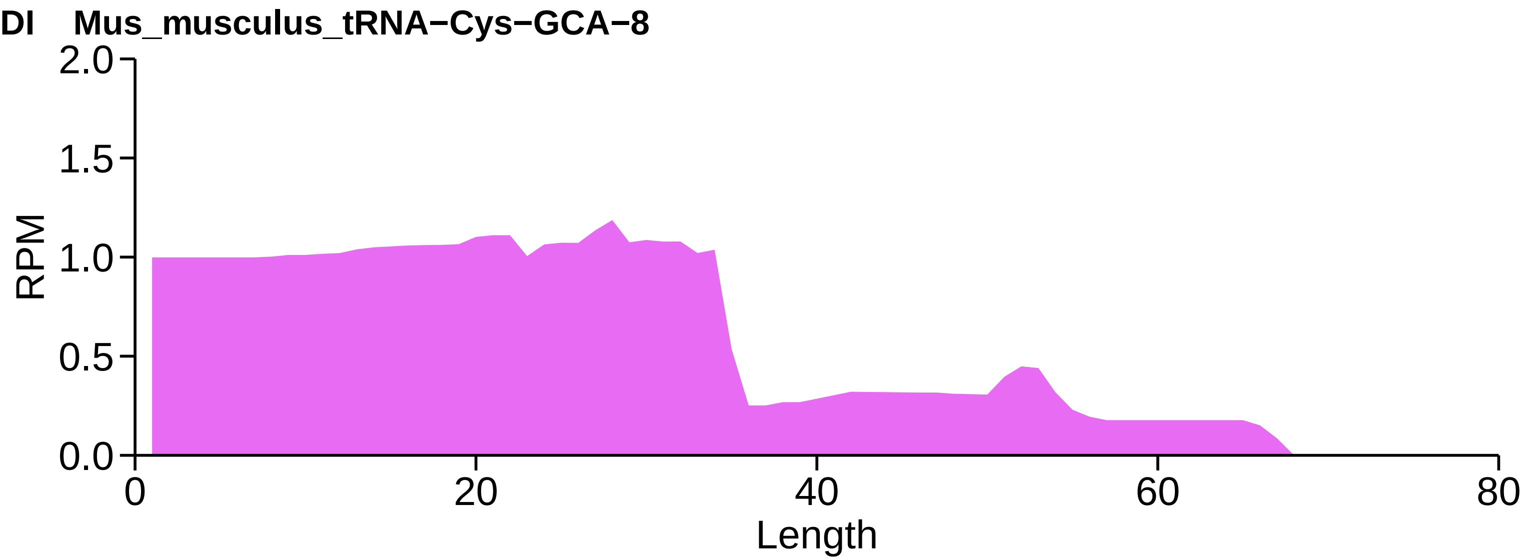
DI Mus_musculus_tRNA−Cys−GCA−8
RPM
2.0
1.5
1.0
0.5
0.0
0
20
40
60
80
Length

**DJ Mus_musculus_tRNA−Cys−GCA−9**

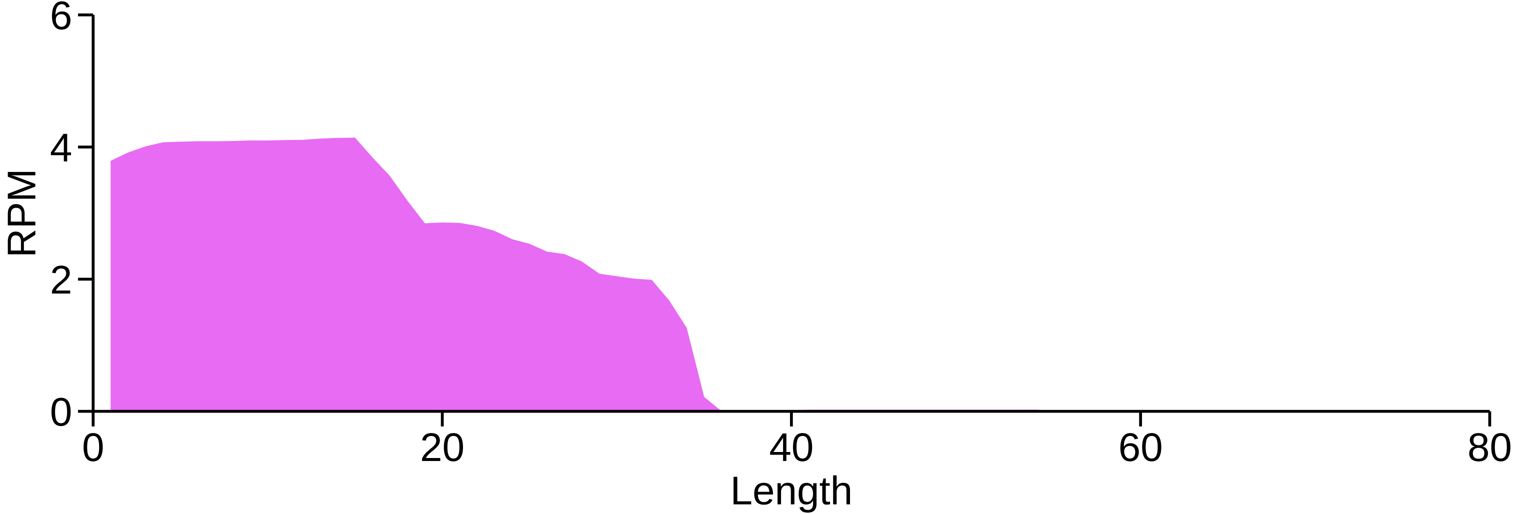

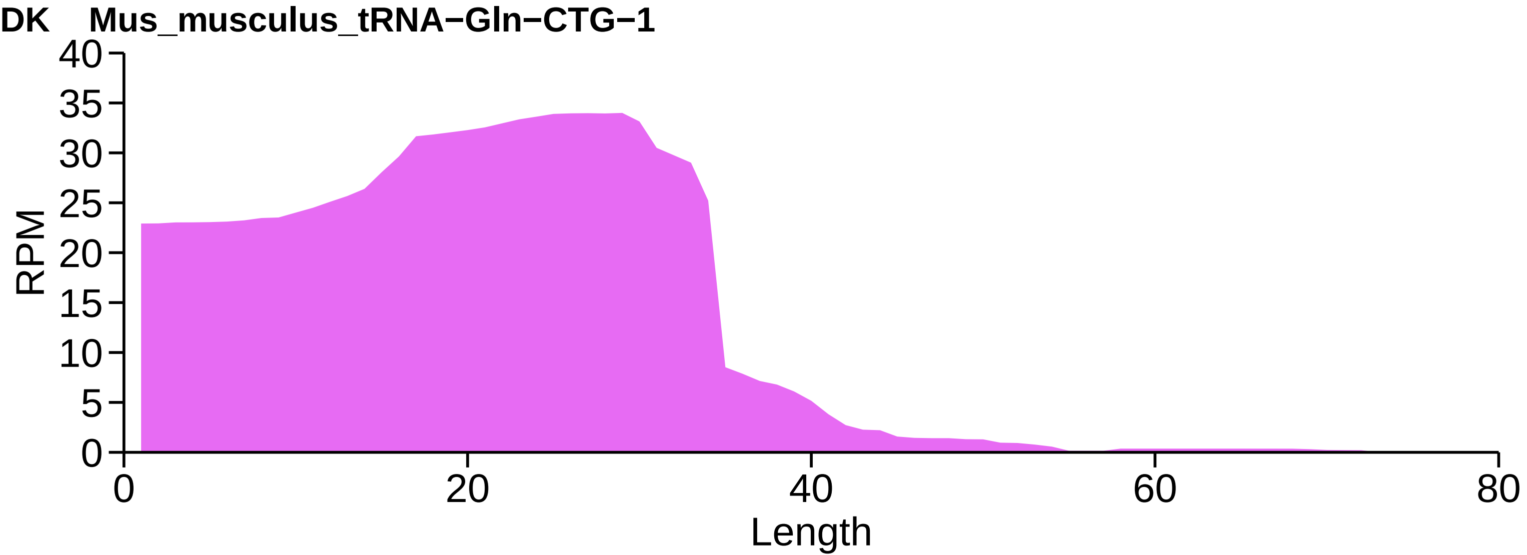
DK Mus_musculus_tRNA−Gln−CTG−1
RPM
Length
0
5
10
15
20
25
30
35
40
0
20
40
60
80

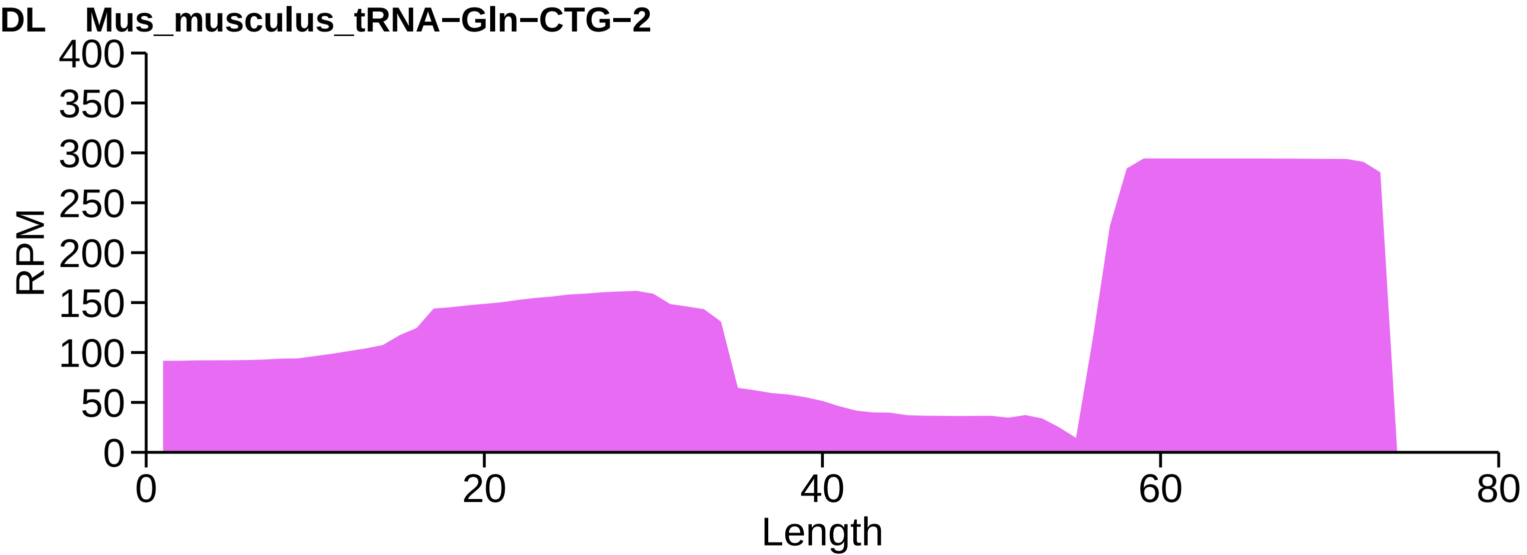
DL Mus_musculus_tRNA−Gln−CTG−2
RPM
0
50
100
150
200
250
300
350
400
0
20
40
60
80
Length

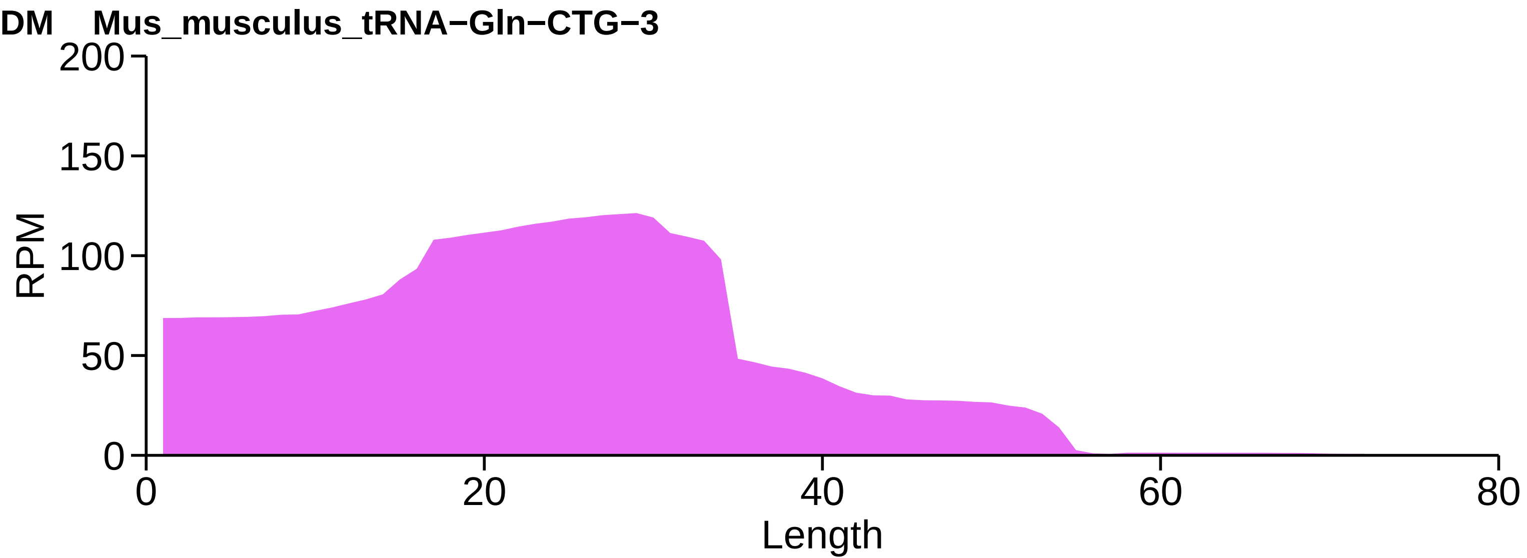
DM Mus_musculus_tRNA−Gln−CTG−3
RPM
200
150
100
50
0
0
20
40
60
80
Length

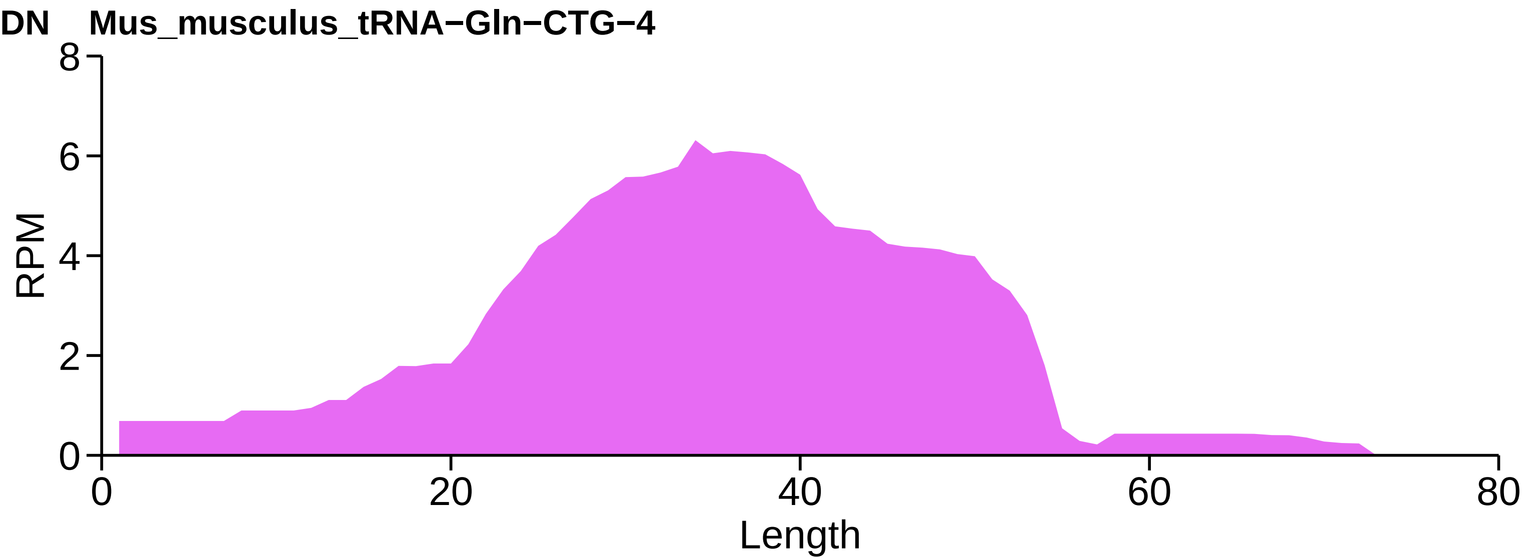
DN Mus_musculus_tRNA−Gln−CTG−4
RPM
Length
0
2
4
6
8
0
20
40
60
80

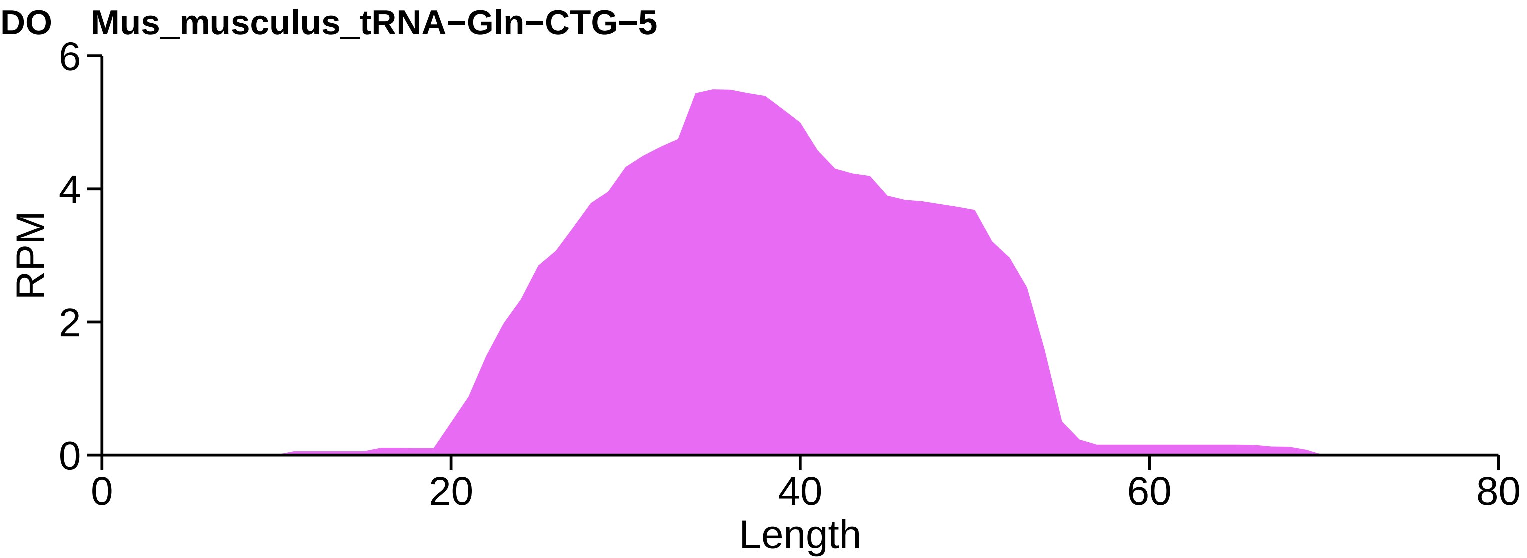
DO Mus_musculus_tRNA−Gln−CTG−5
RPM
Length
0
2
4
6
0
20
40
60
80

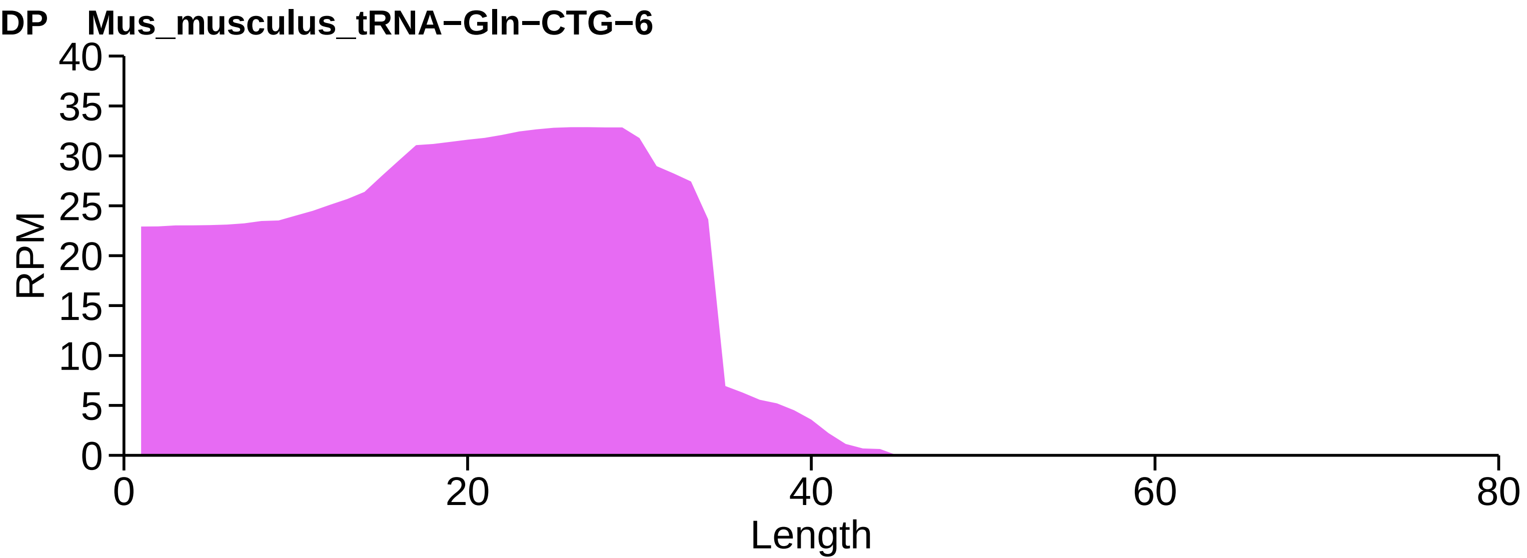

DP Mus_musculus_tRNA−Gln−CTG−6
RPM
0
5
10
15
20
25
30
35
40
0
20
40
60
80
Length

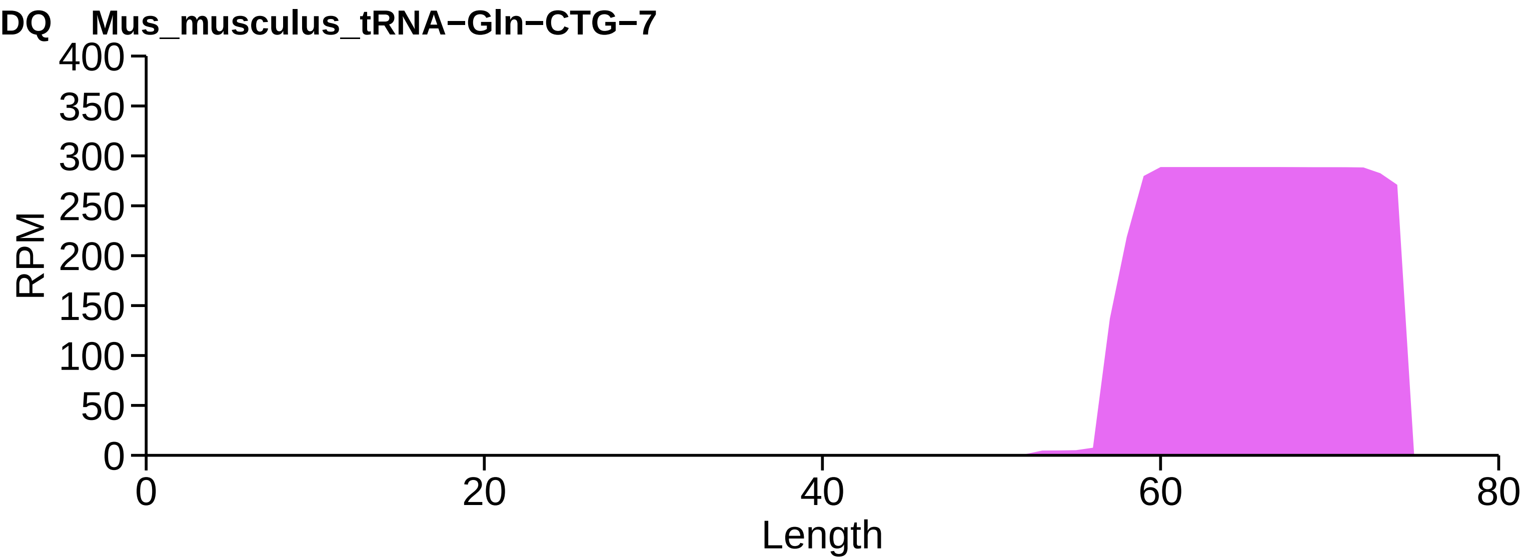
DQ Mus_musculus_tRNA−Gln−CTG−7
RPM
Length
0
50
100
150
200
250
300
350
400
0
20
40
60
80

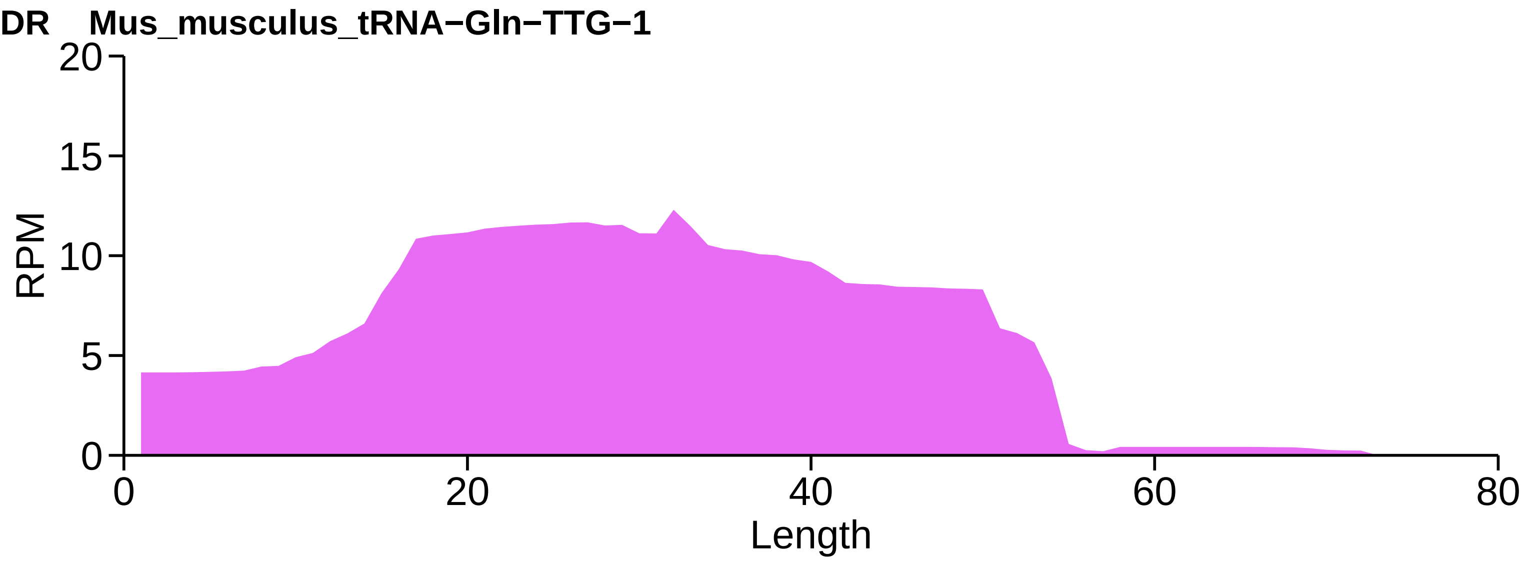
DR Mus_musculus_tRNA−Gln−TTG−1
RPM
Length
0
5
10
15
20
0
20
40
60
80

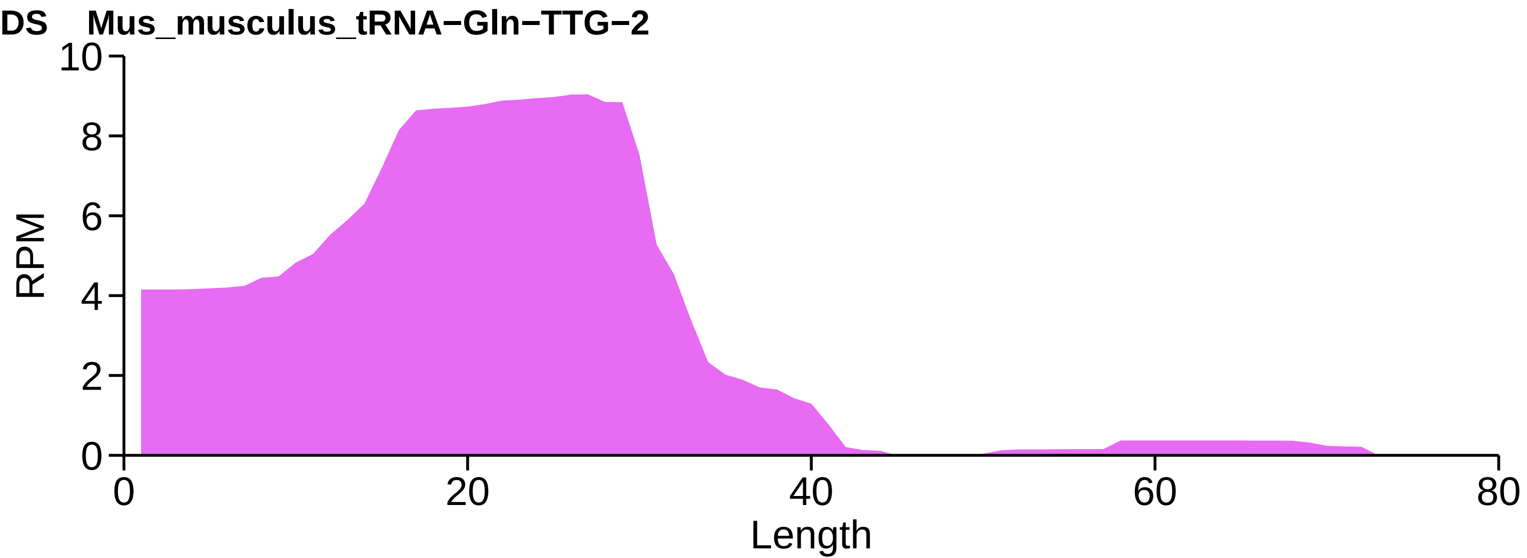
DS Mus_musculus_tRNA−Gln−TTG−2
RPM
Length
0
2
4
6
8
10
0
20
40
60
80

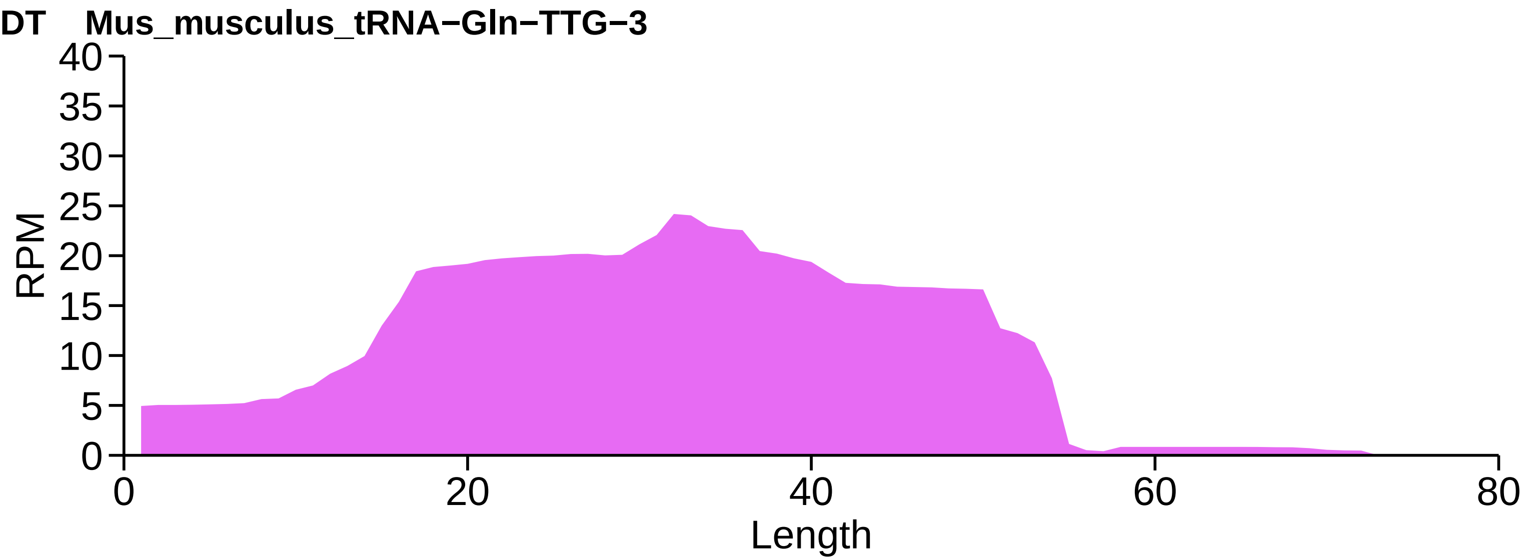
DT Mus_musculus_tRNA−Gln−TTG−3
RPM
Length
0
5
10
15
20
25
30
35
40
0
20
40
60
80

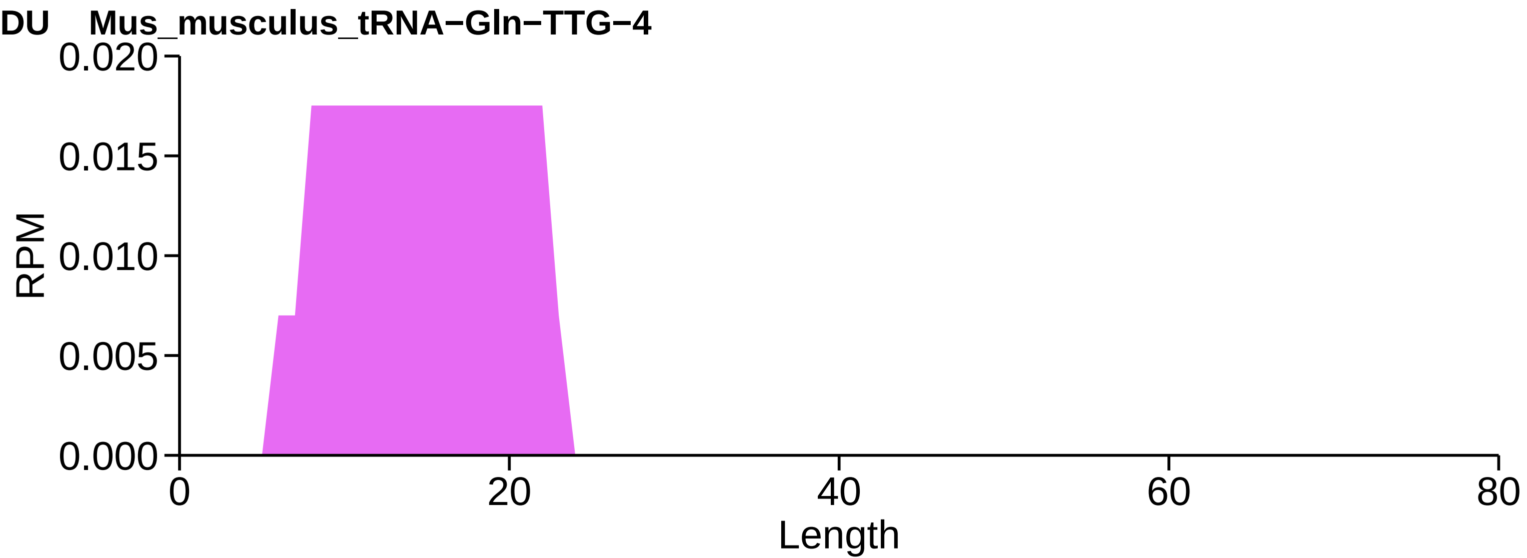

DU Mus_musculus_tRNA−Gln−TTG−4
0.020
0.015
0.010
0.005
0.000
RPM
0
20
40
60
80
Length

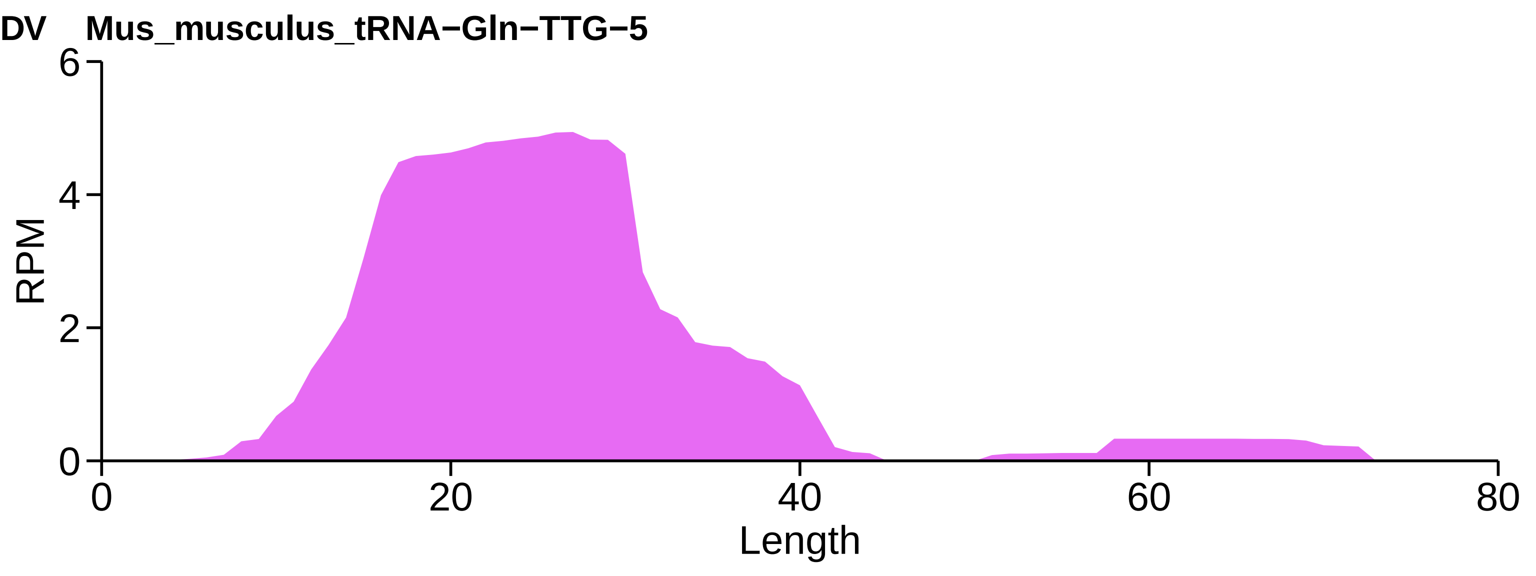
DV Mus_musculus_tRNA−Gln−TTG−5
RPM
0
2
4
6
0
20
40
60
80
Length

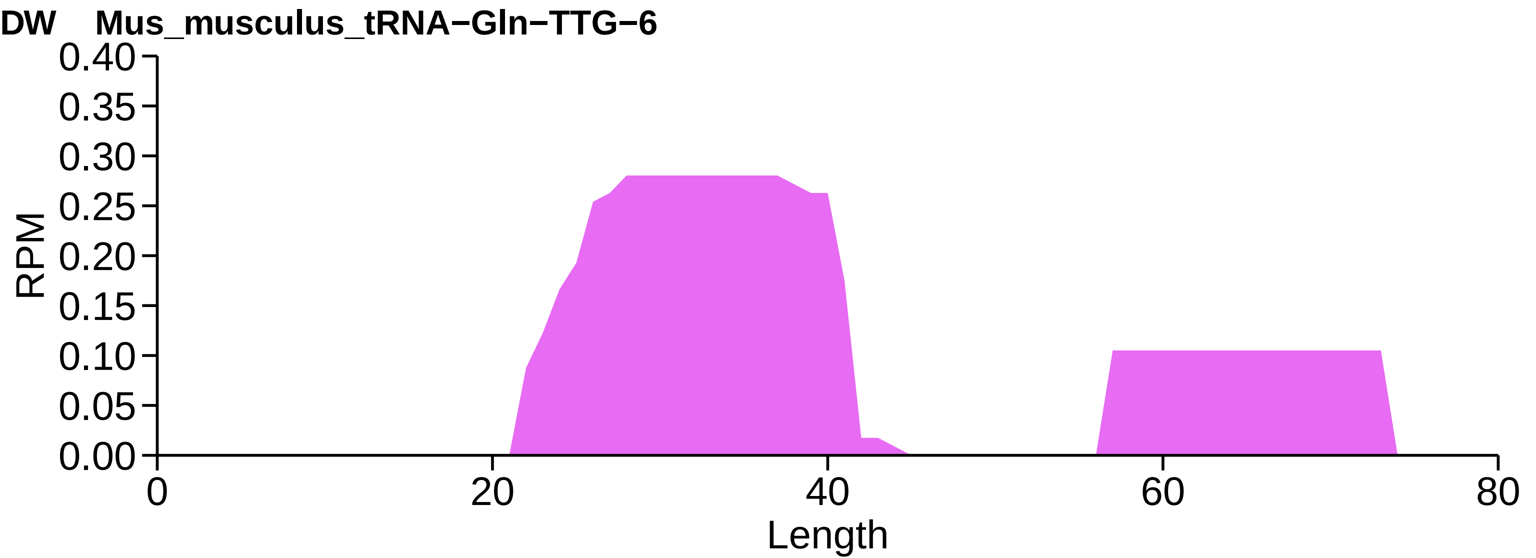
DW Mus_musculus_tRNA−Gln−TTG−6
RPM
0.40
0.35
0.30
0.25
0.20
0.15
0.10
0.05
0.00
0
20
40
60
80
Length

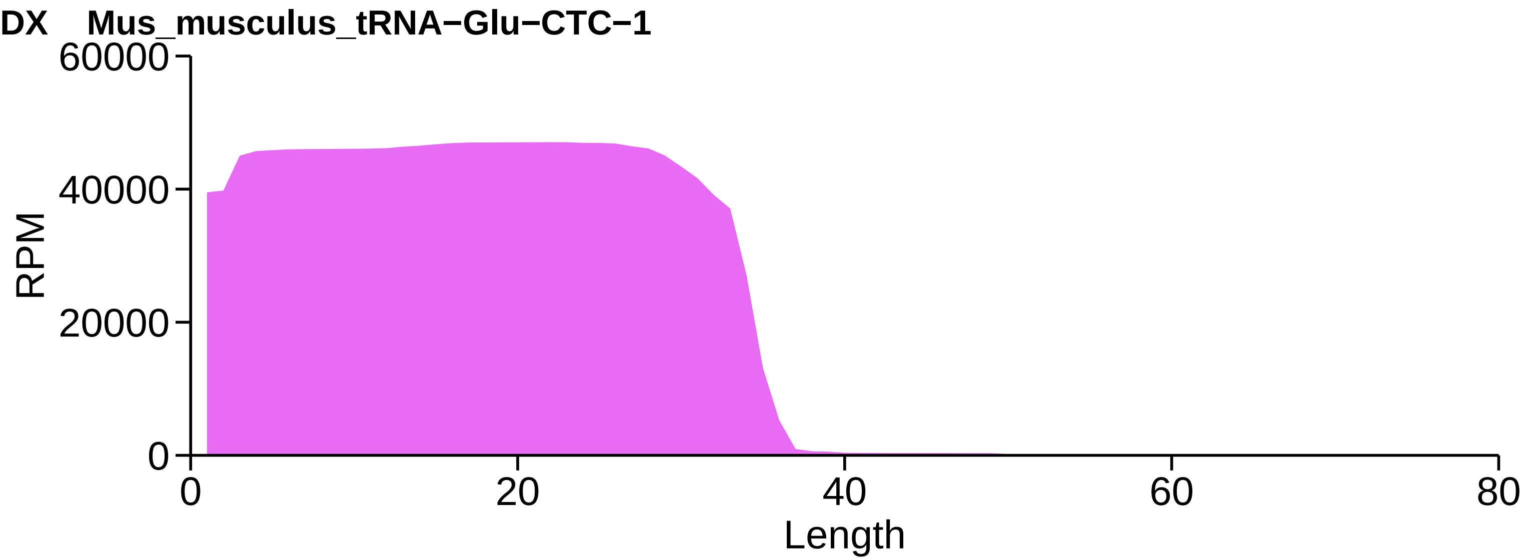
DX Mus_musculus_tRNA−Glu−CTC−1
RPM
60000
40000
20000
0
0
20
40
60
80
Length

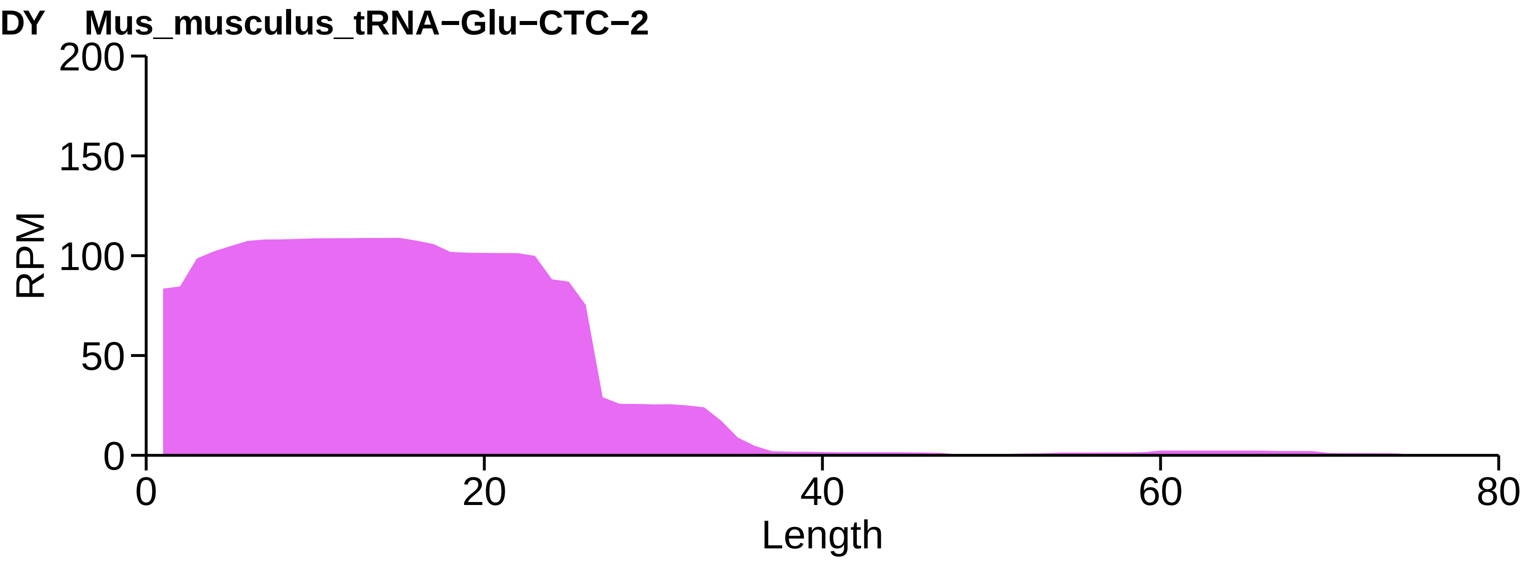

DY Mus_musculus_tRNA−Glu−CTC−2
RPM
200
150
100
50
0
0
20
40
60
80
Length

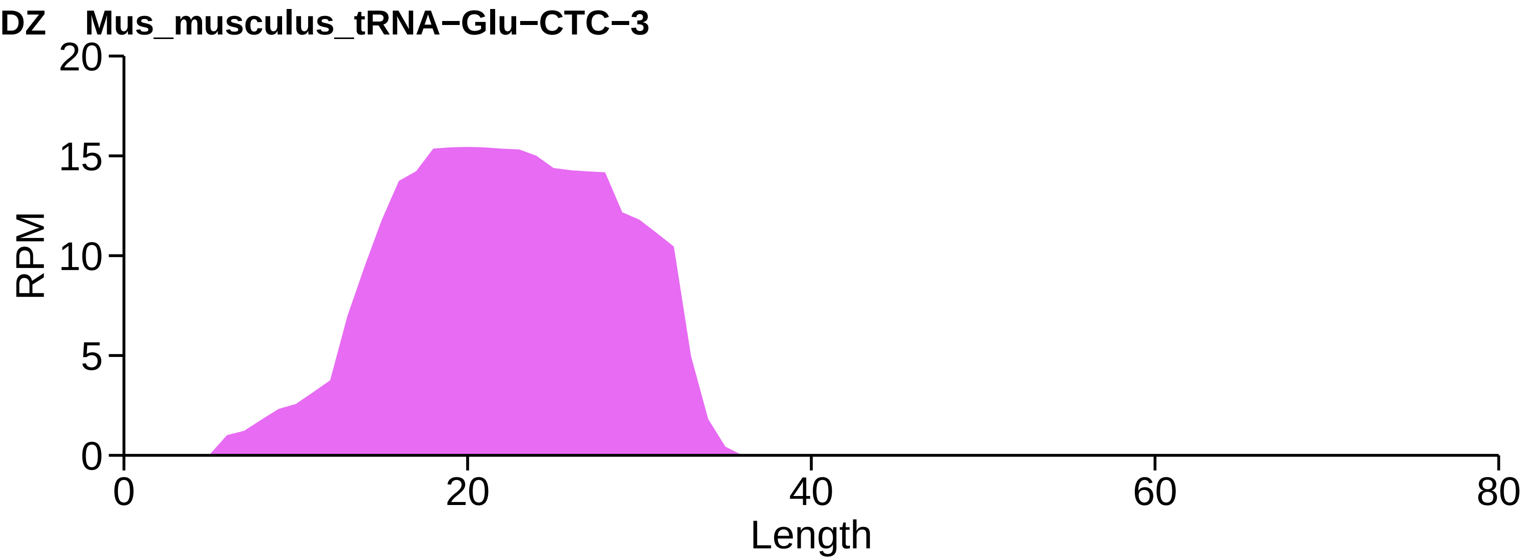
DZ Mus_musculus_tRNA−Glu−CTC−3
RPM
0
5
10
15
20
0
20
40
60
80
Length

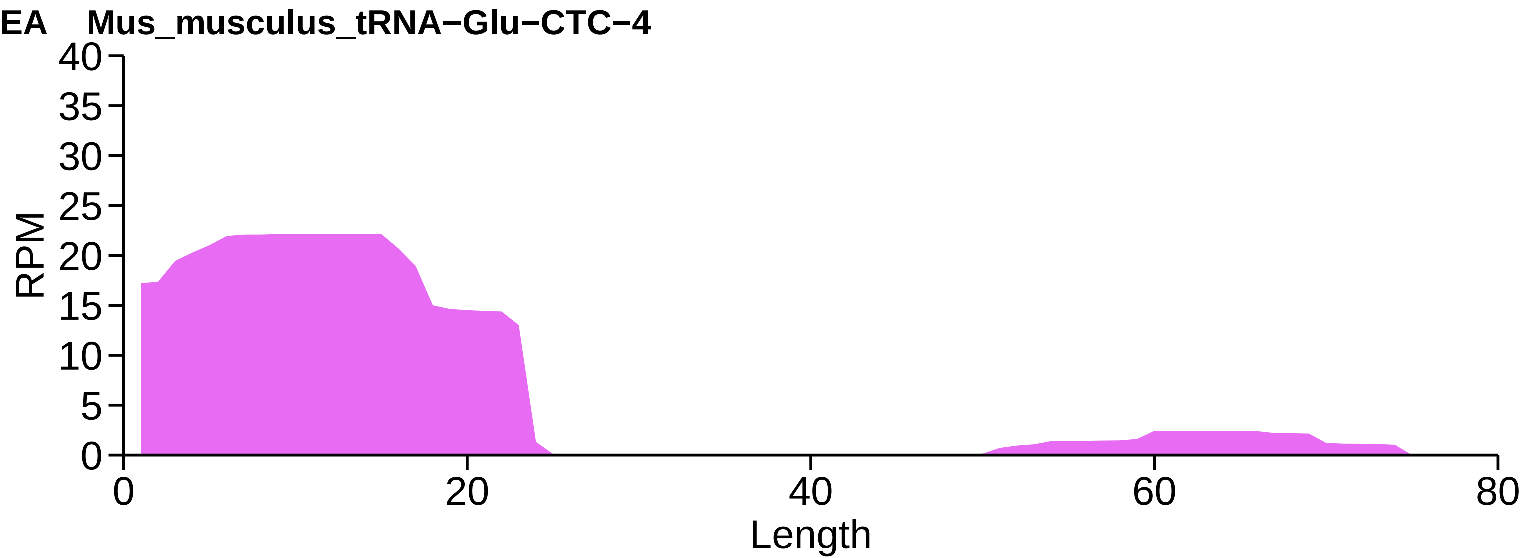
EA Mus_musculus_tRNA−Glu−CTC−4
RPM
Length
0
5
10
15
20
25
30
35
40
0
20
40
60
80

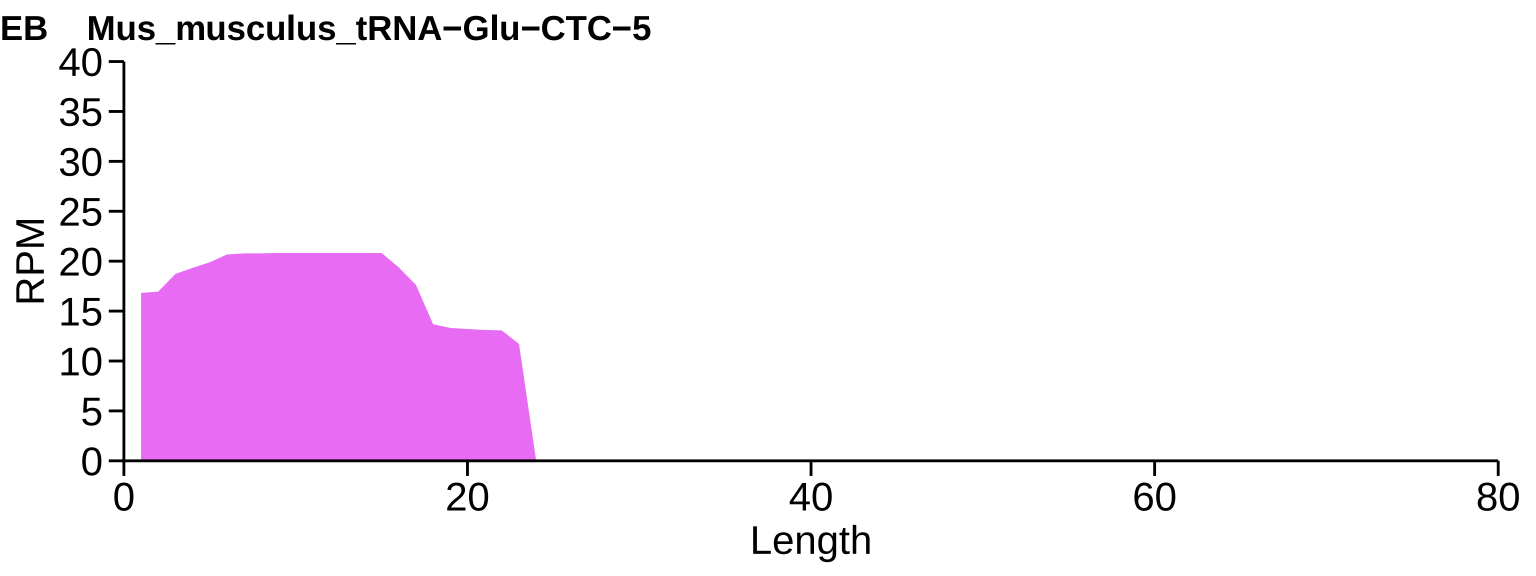

EB Mus_musculus_tRNA−Glu−CTC−5
RPM
Length
0
5
10
15
20
25
30
35
40
0
20
40
60
80

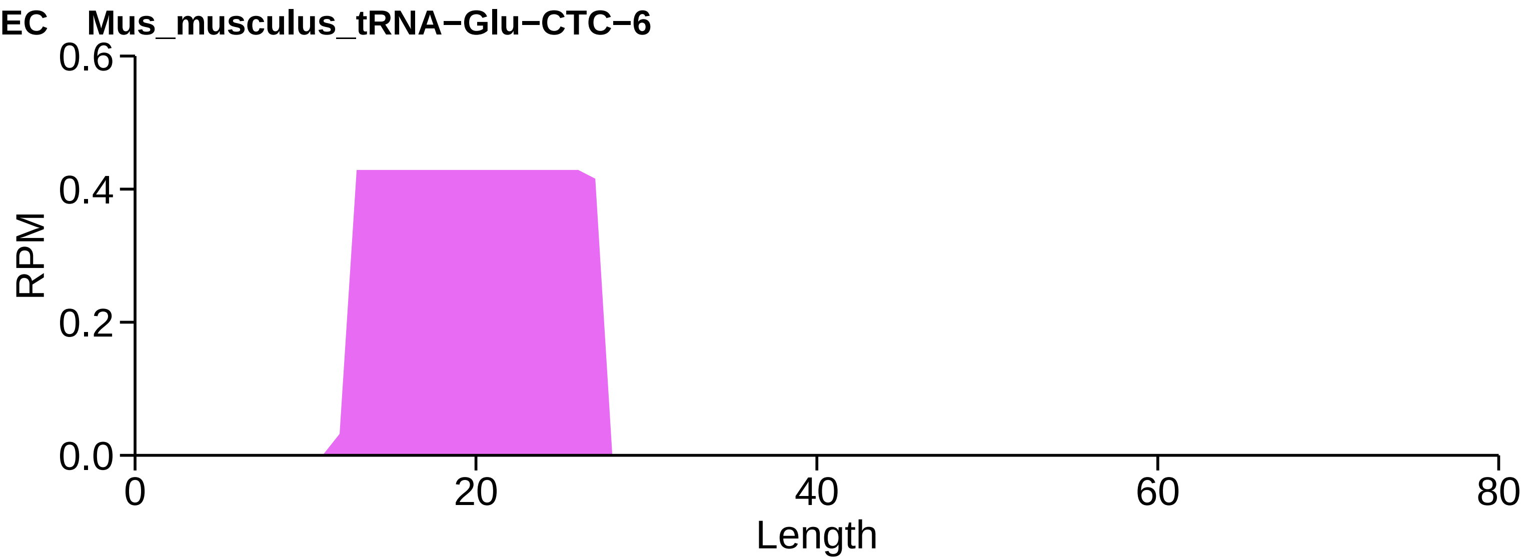
EC Mus_musculus_tRNA−Glu−CTC−6
0.6
0.4
0.2
0.0
RPM
0
20
40
60
80
Length

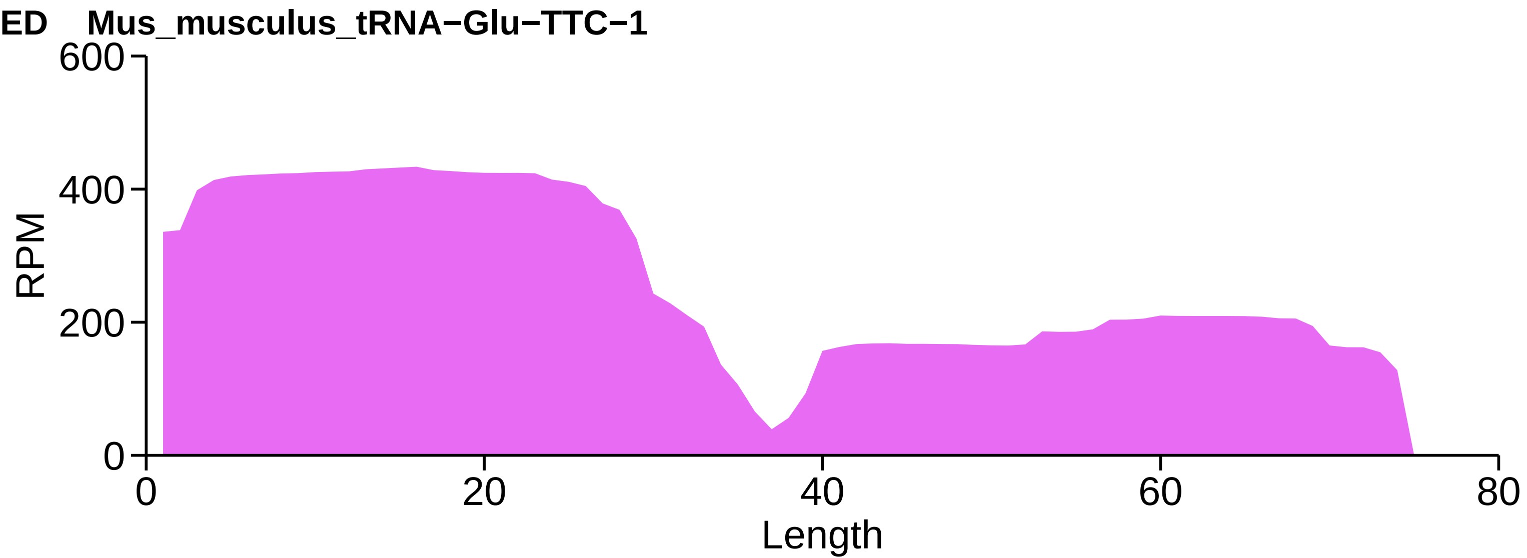
ED Mus_musculus_tRNA−Glu−TTC−1
600
400
200
0
RPM
0
20
40
60
80
Length

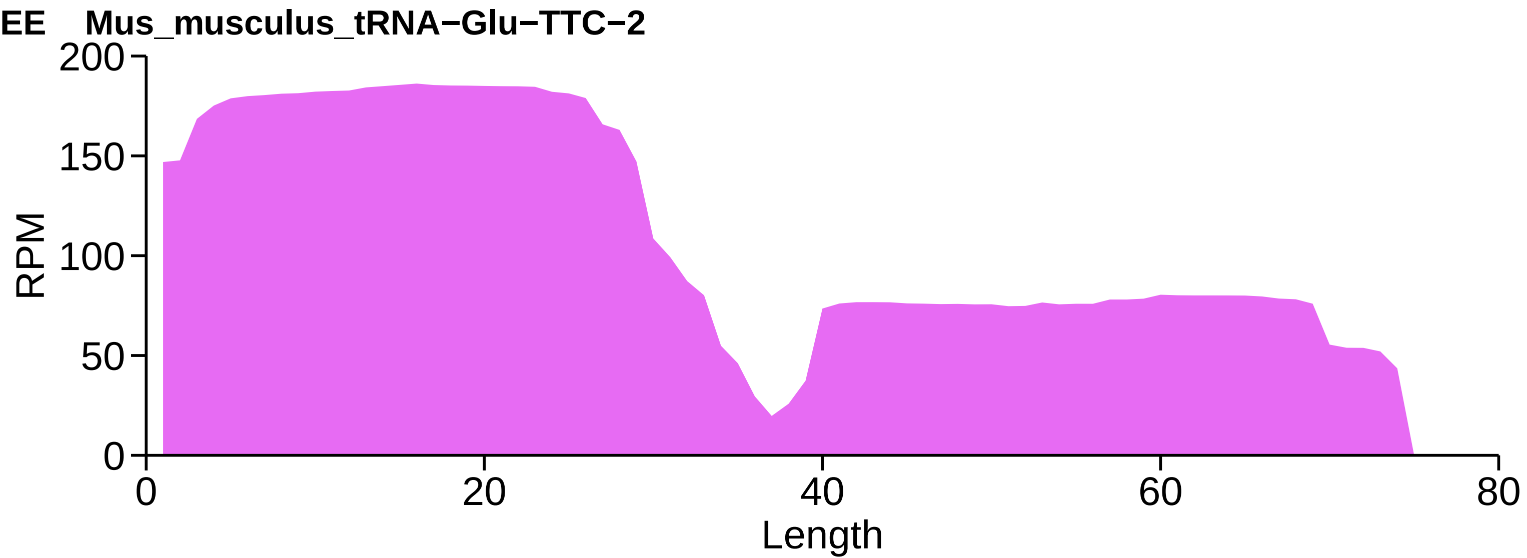
EE Mus_musculus_tRNA−Glu−TTC−2
RPM
200
150
100
50
0
0
20
40
60
80
Length

EF Mus_musculus_tRNA−Glu−TTC−3

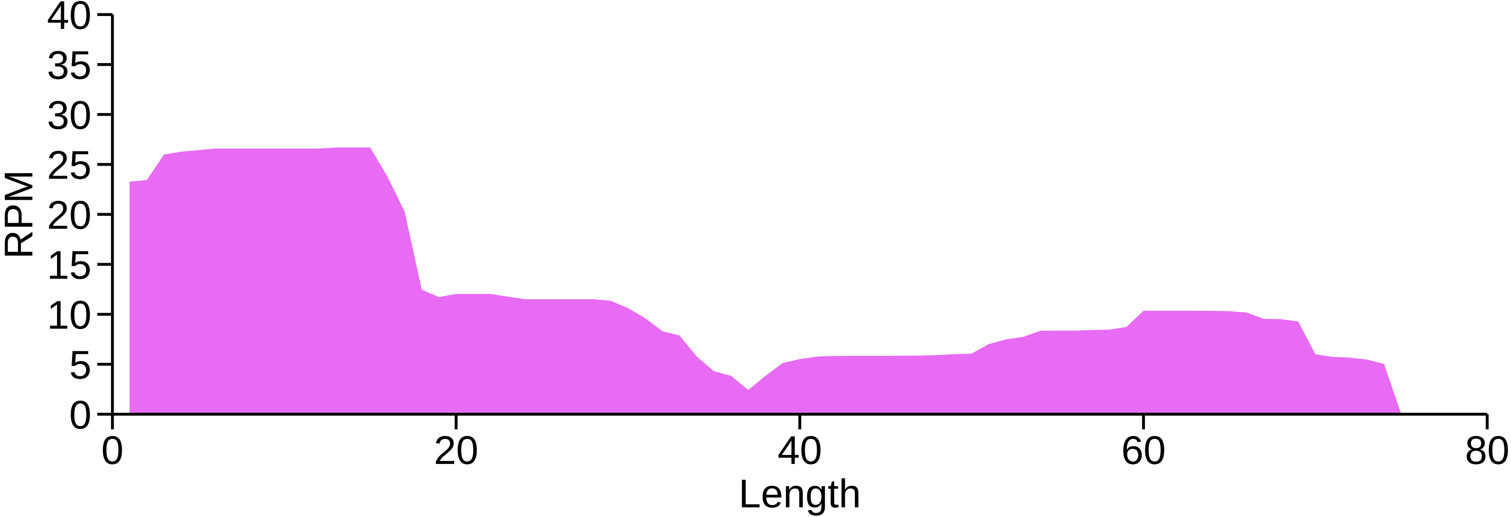

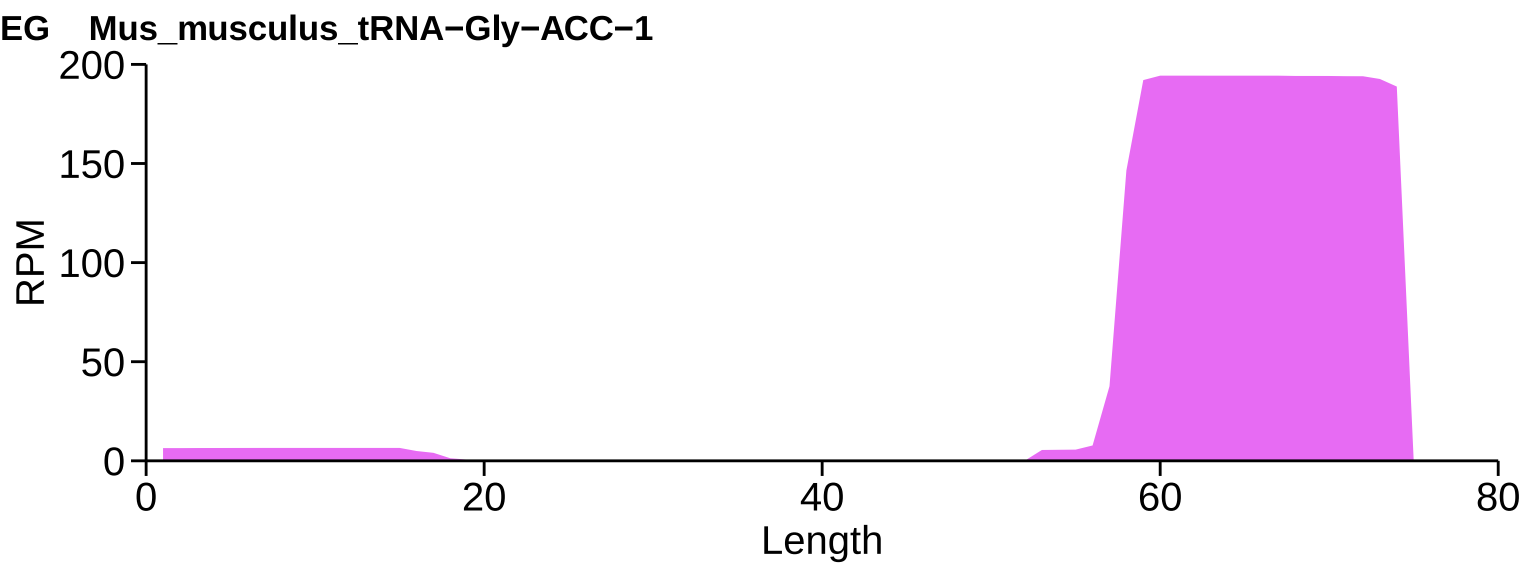
EG Mus_musculus_tRNA−Gly−ACC−1
200
150
100
50
0
RPM
0
20
40
60
80
Length

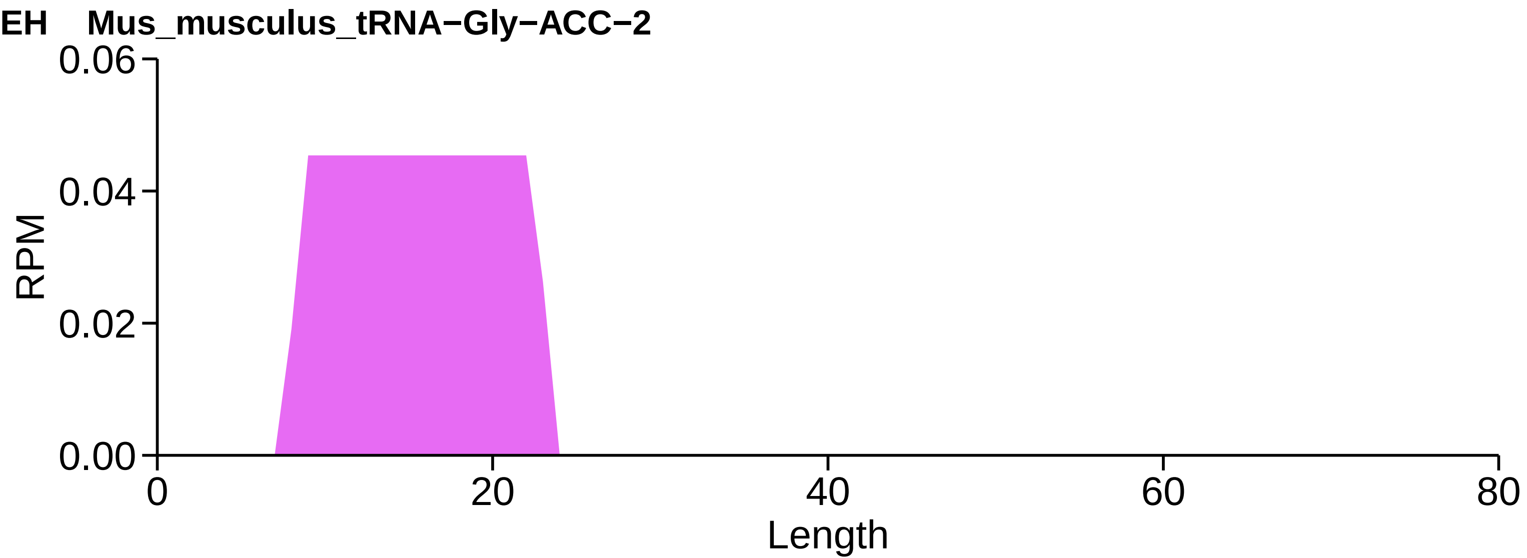

EH Mus_musculus_tRNA−Gly−ACC−2
0.06
0.04
0.02
0.00
RPM
0
20
40
60
80
Length

**EI** **Mus_musculus_tRNA−Gly−CCC−1**

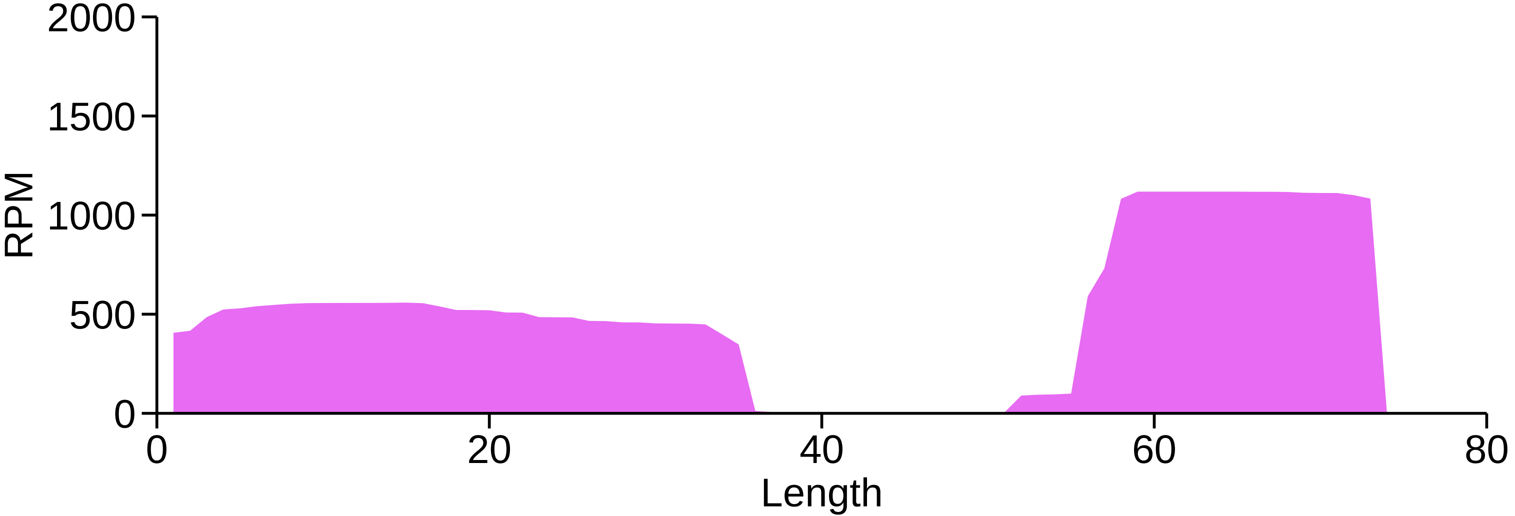

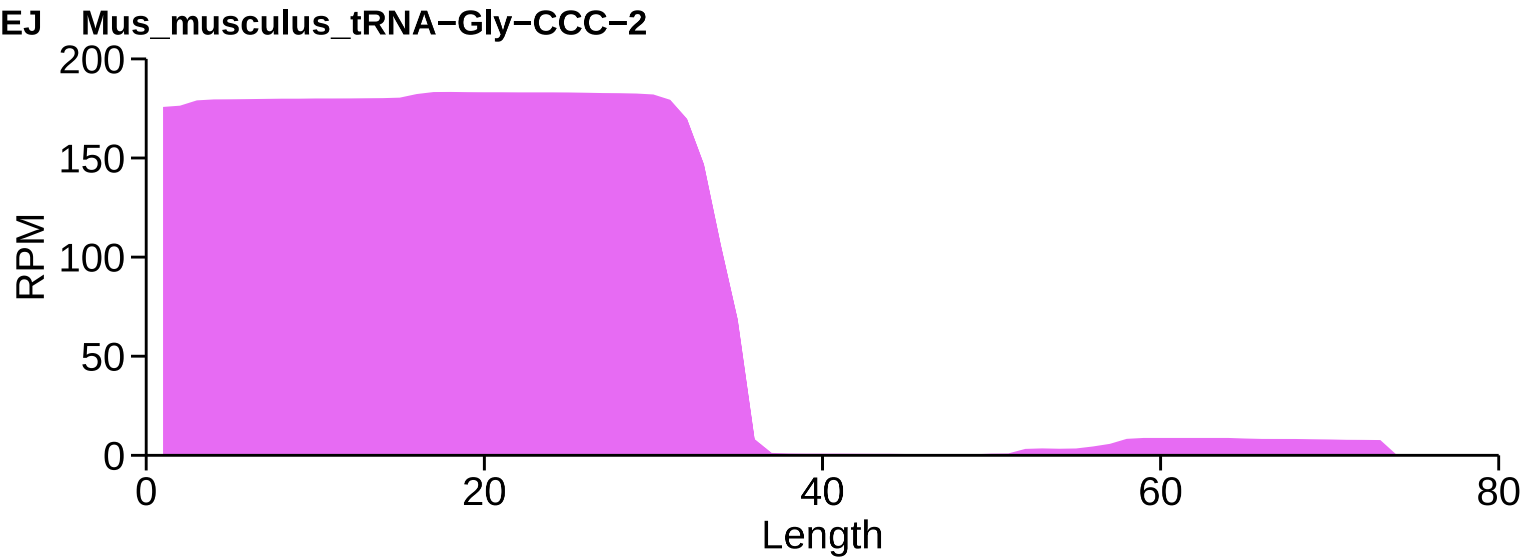
EJ Mus_musculus_tRNA−Gly−CCC−2
200
150
100
50
0
RPM
0
20
40
60
80
Length

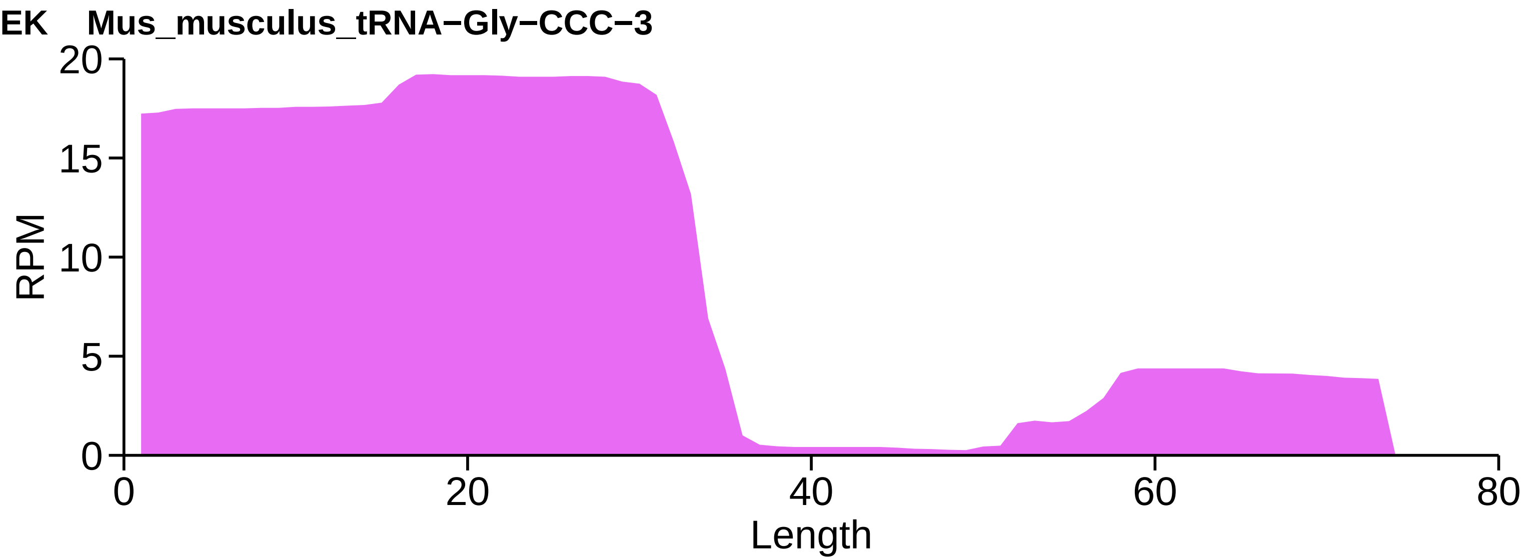

EK Mus_musculus_tRNA−Gly−CCC−3
RPM
20
15
10
5
0
0
20
40
60
80
Length

EL Mus_musculus_tRNA−Gly−CCC−4

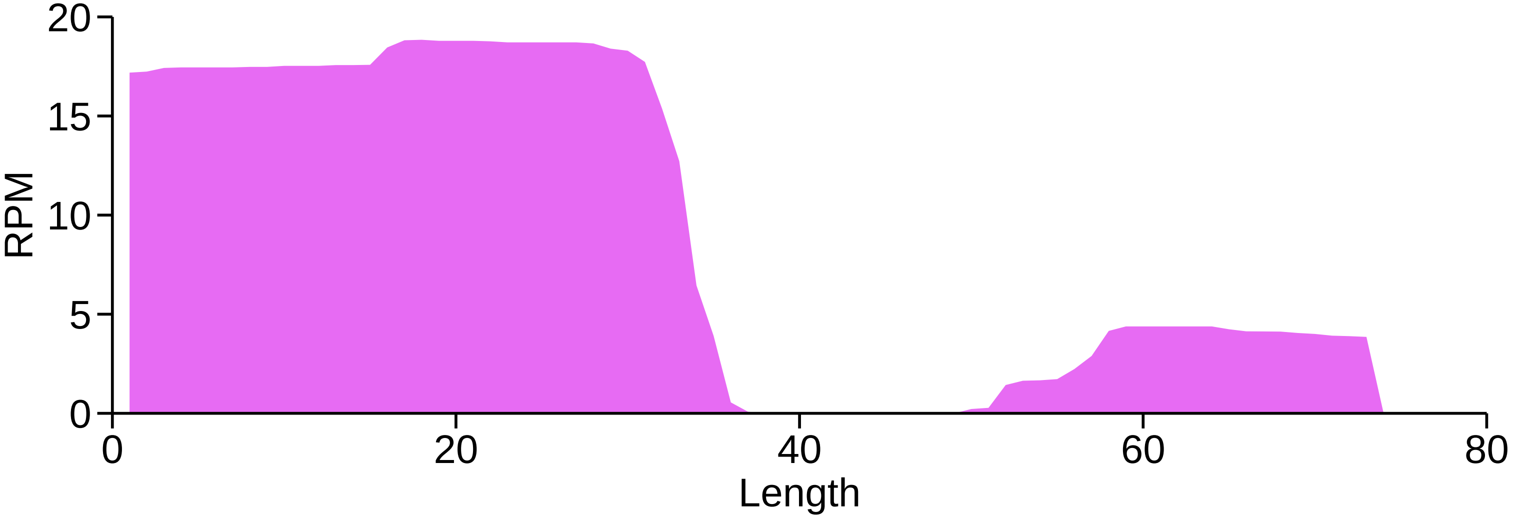

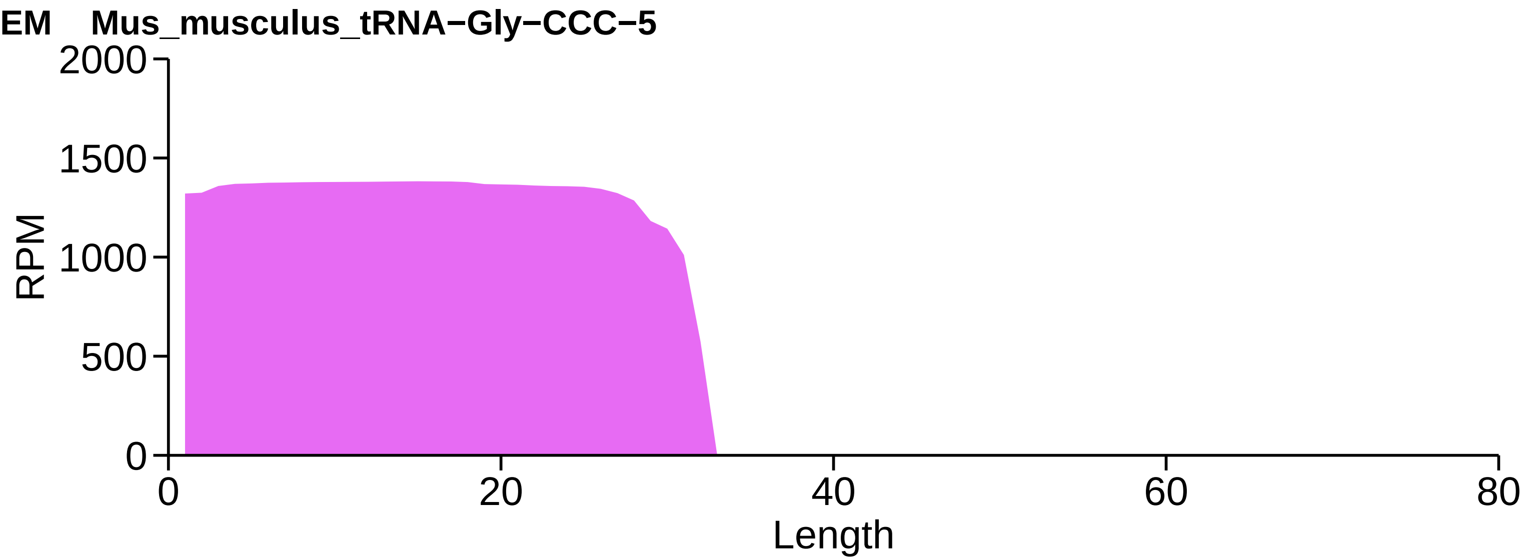
EM Mus_musculus_tRNA−Gly−CCC−5
2000
1500
1000
500
0
RPM
0
20
40
60
80
Length

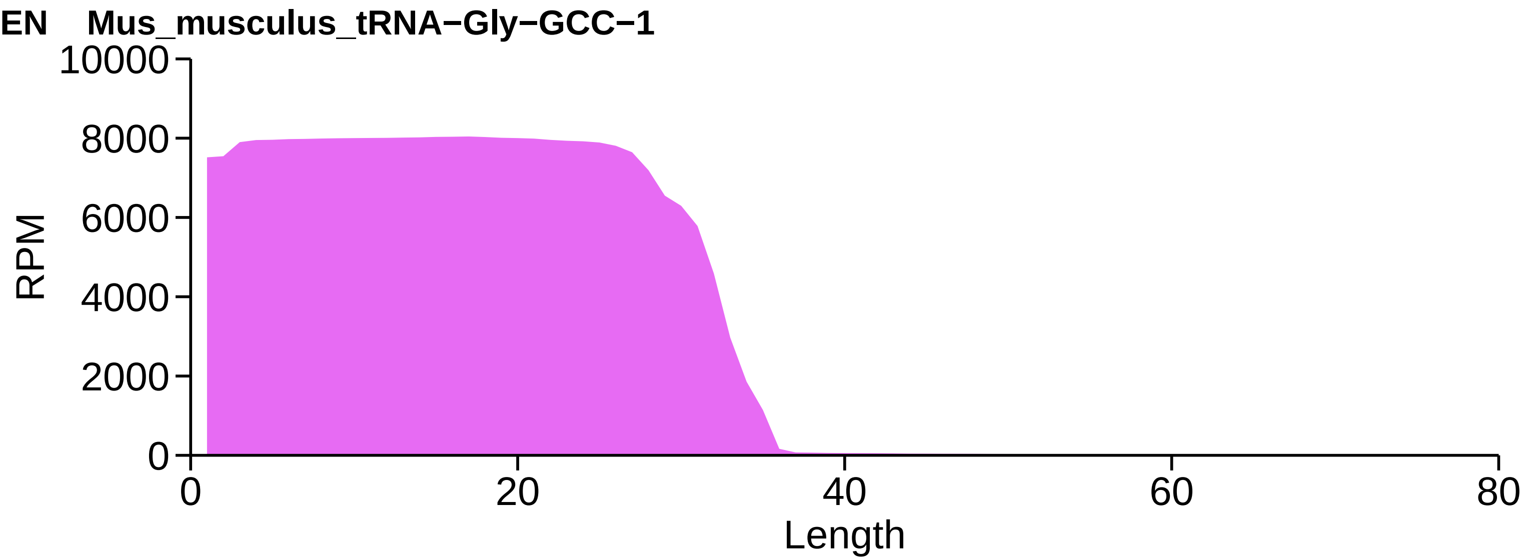

EN Mus_musculus_tRNA−Gly−GCC−1
RPM
10000
8000
6000
4000
2000
0
0
20
40
60
80
Length

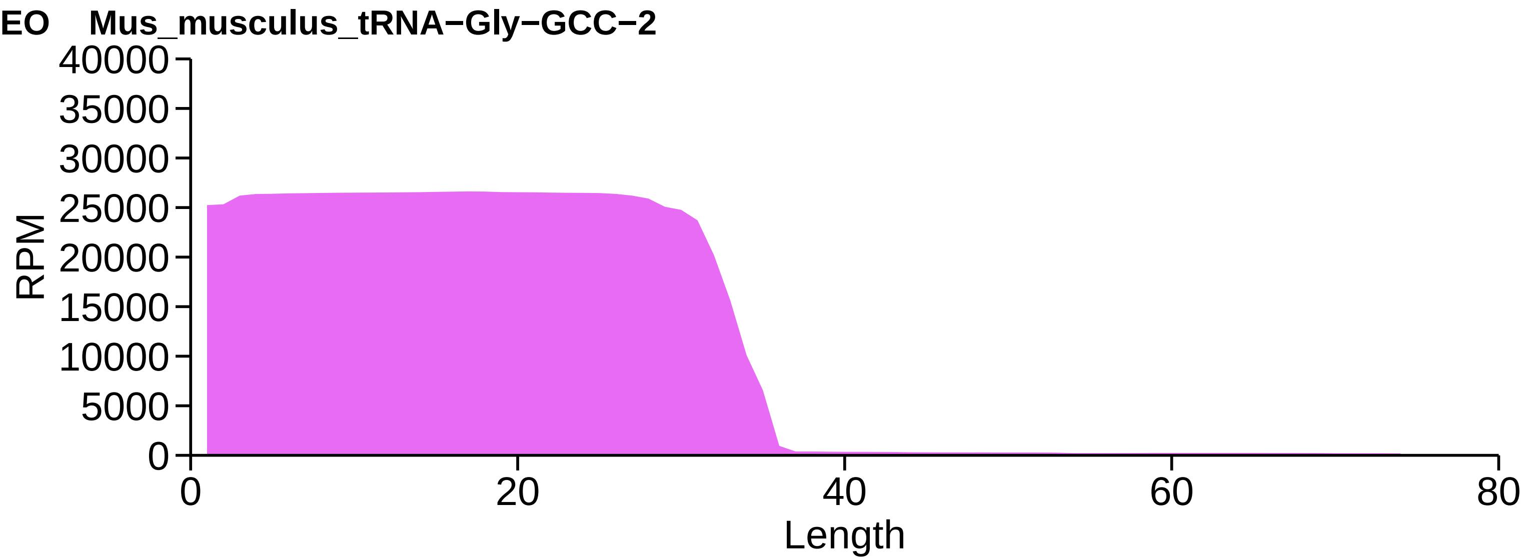

EO Mus_musculus_tRNA−Gly−GCC−2
40000
35000
30000
25000
20000
15000
10000
5000
0
RPM
0
20
40
60
80
Length

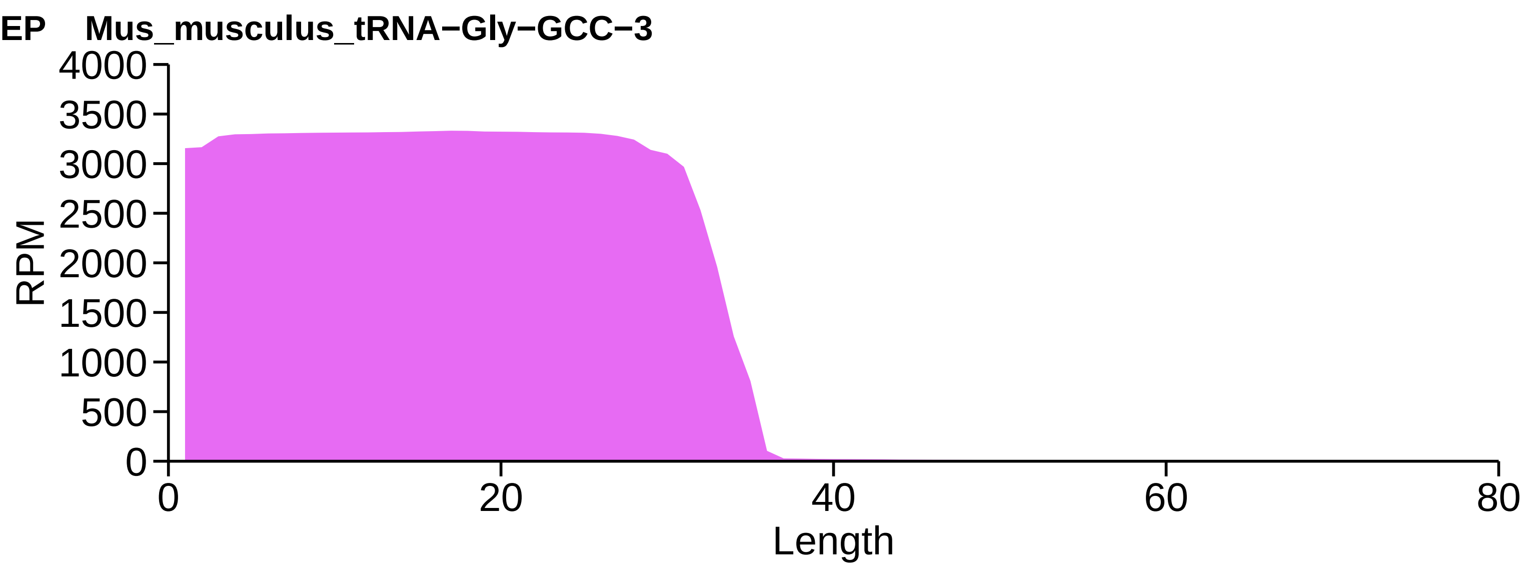
EP Mus_musculus_tRNA−Gly−GCC−3
RPM
4000
3500
3000
2500
2000
1500
1000
500
0
0
20
40
60
80
Length

EQ    Mus_musculus_tRNA−Gly−GCC−4

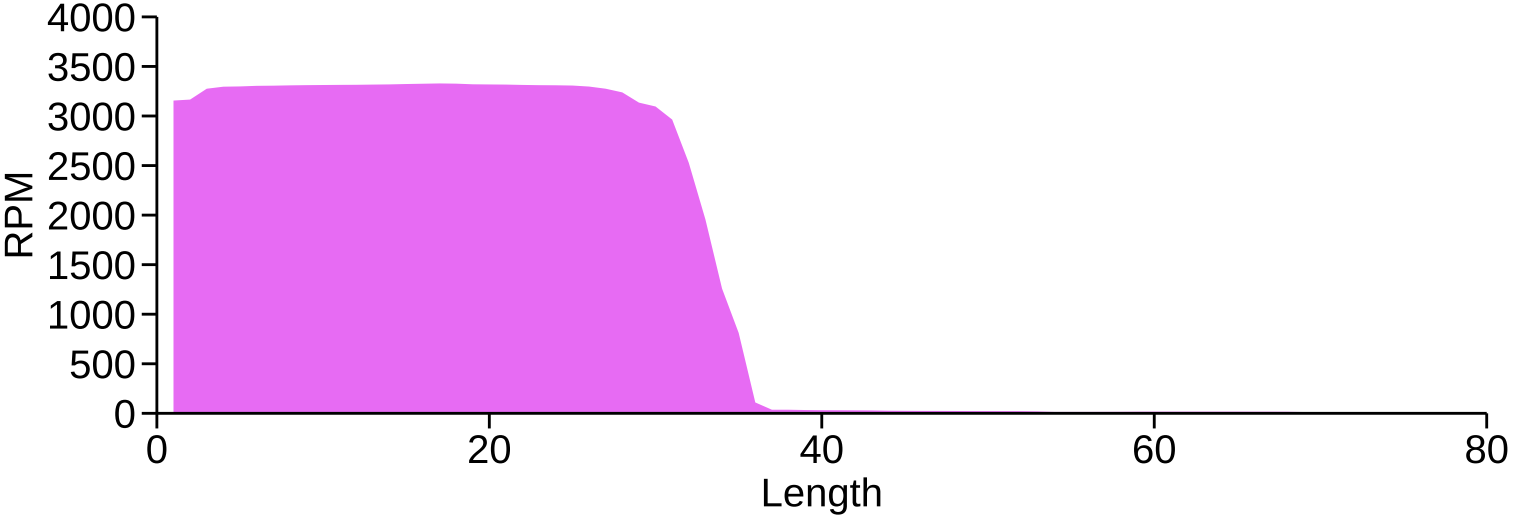

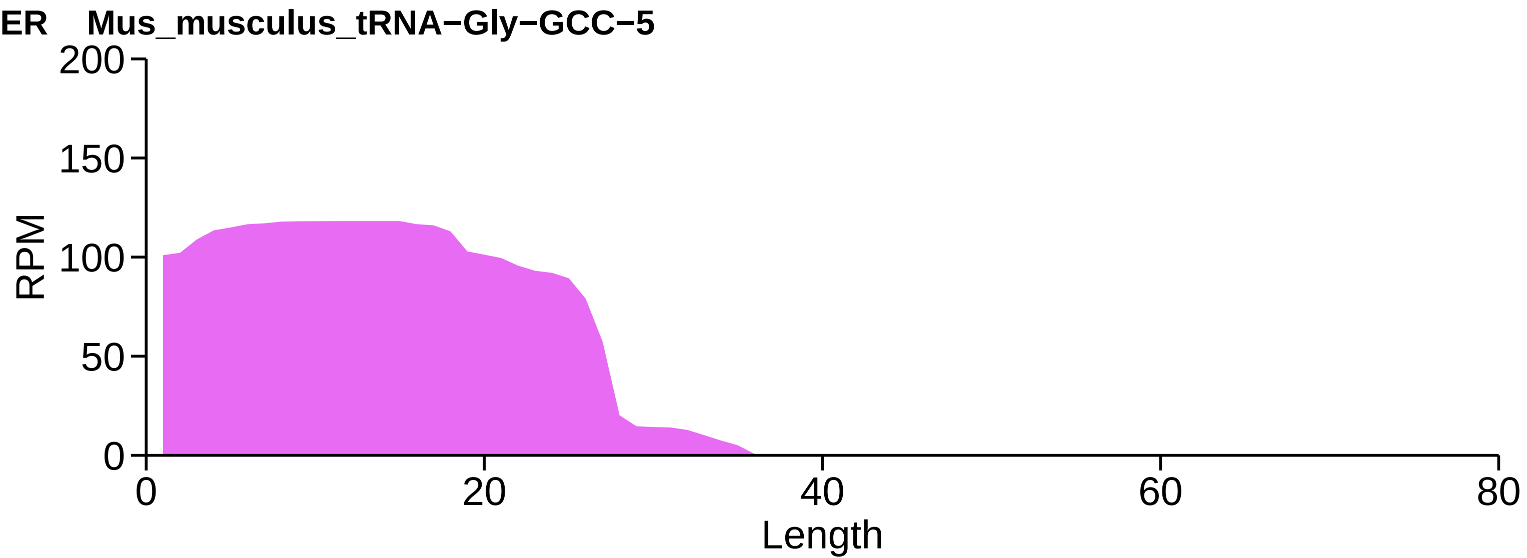

ER Mus_musculus_tRNA−Gly−GCC−5
200
150
100
50
0
RPM
0
20
40
60
80
Length

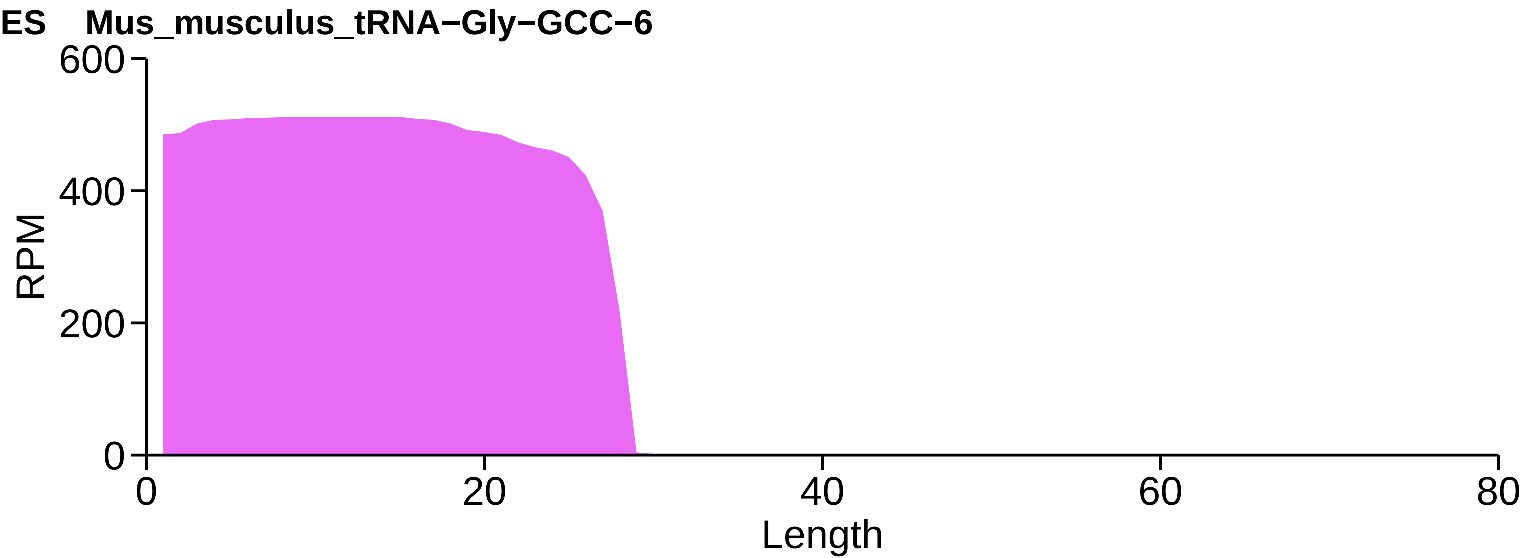

ES Mus_musculus_tRNA−Gly−GCC−6
600
400
200
0
RPM
0
20
40
60
80
Length

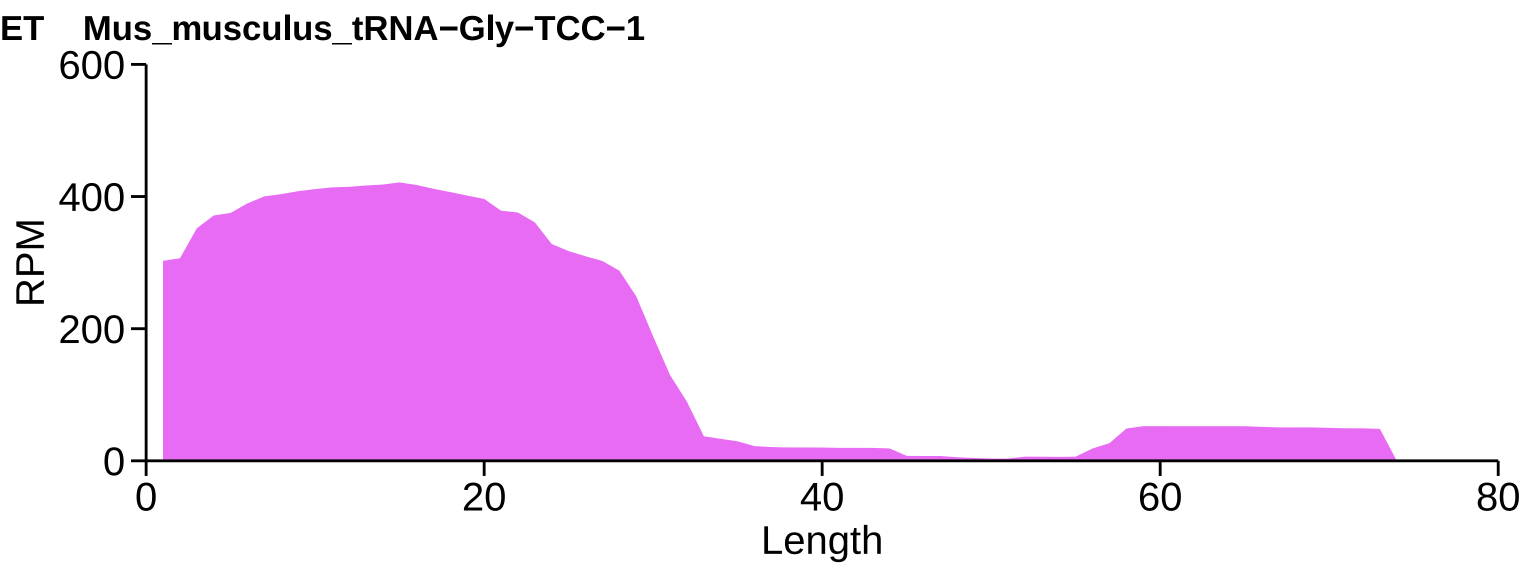
ET Mus_musculus_tRNA−Gly−TCC−1
600
400
200
0
RPM
0
20
40
60
80
Length

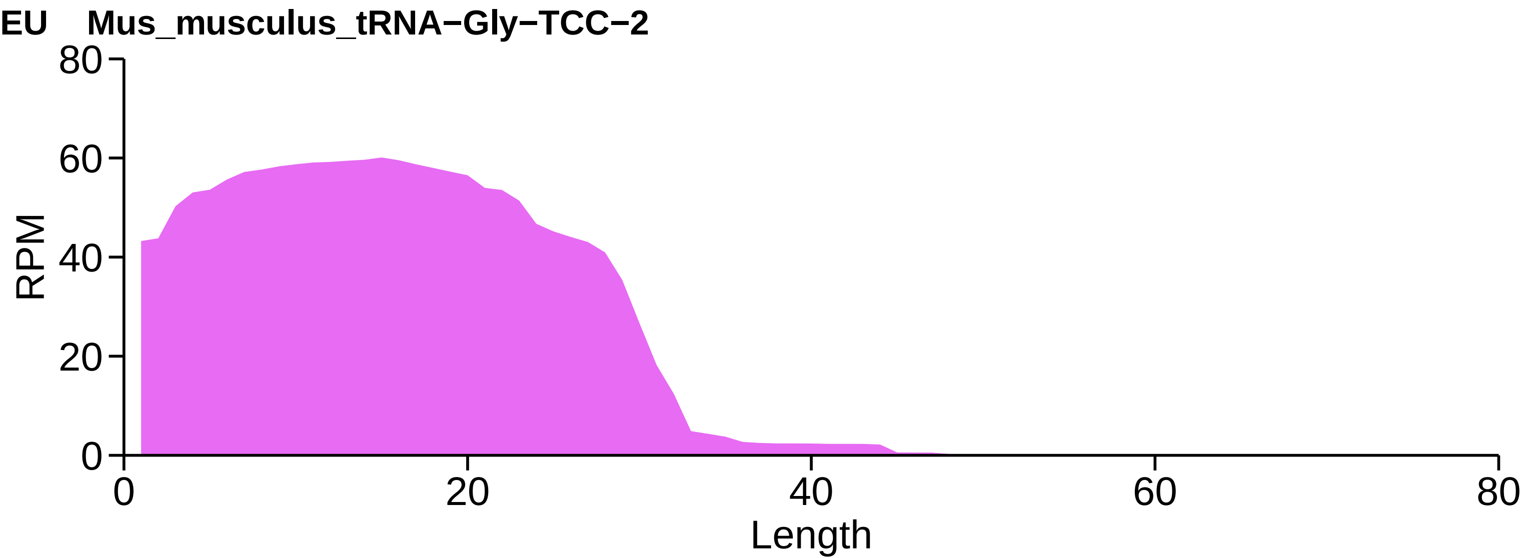
EU Mus_musculus_tRNA−Gly−TCC−2
RPM
0
20
40
60
80
Length

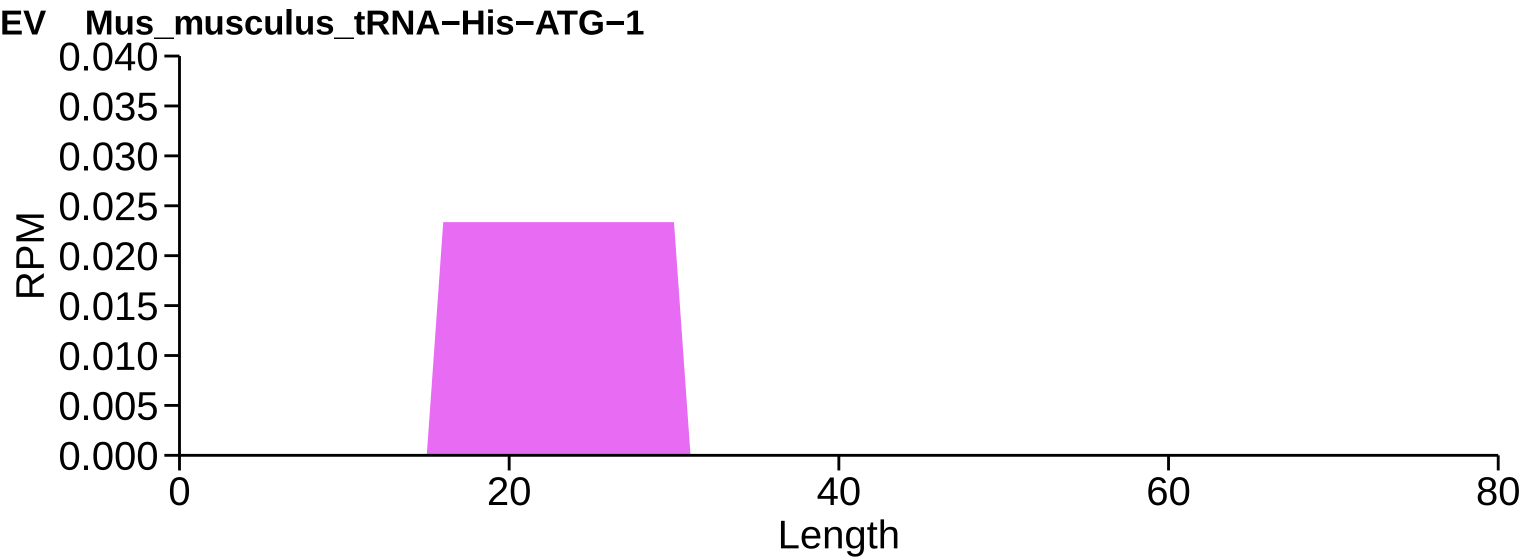
EV Mus_musculus_tRNA−His−ATG−1
RPM
0.040
0.035
0.030
0.025
0.020
0.015
0.010
0.005
0.000
0
20
40
60
80
Length

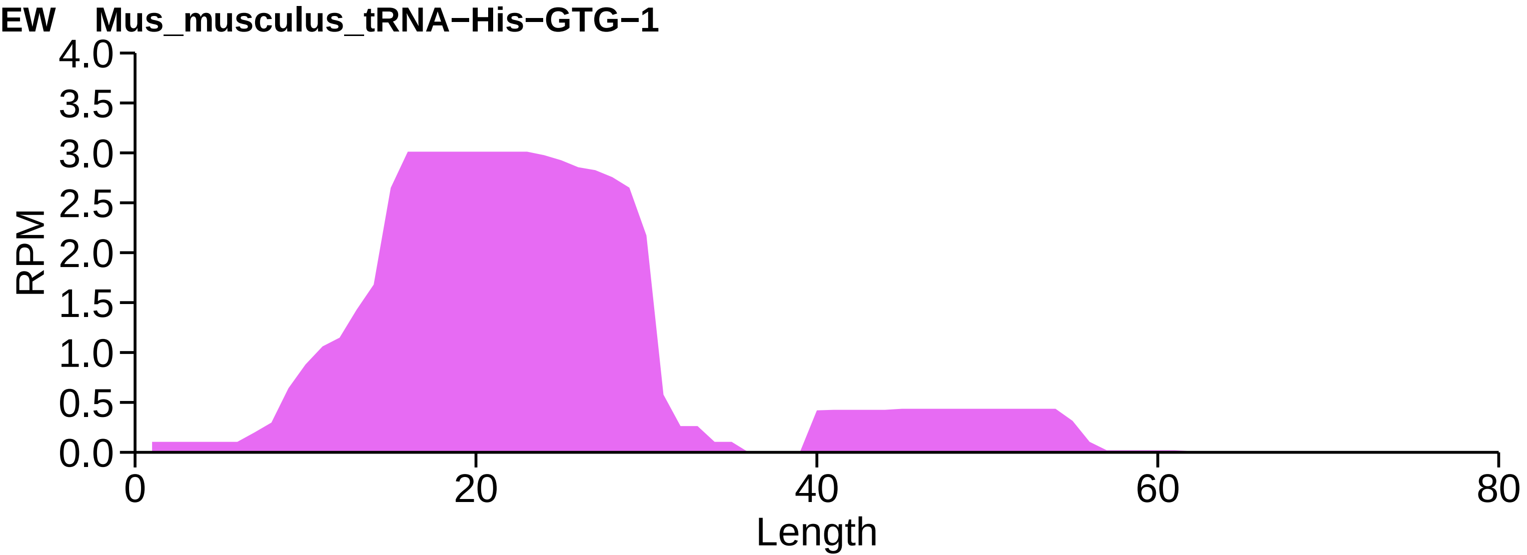

EW Mus_musculus_tRNA−His−GTG−1
RPM
4.0
3.5
3.0
2.5
2.0
1.5
1.0
0.5
0.0
0
20
40
60
80
Length

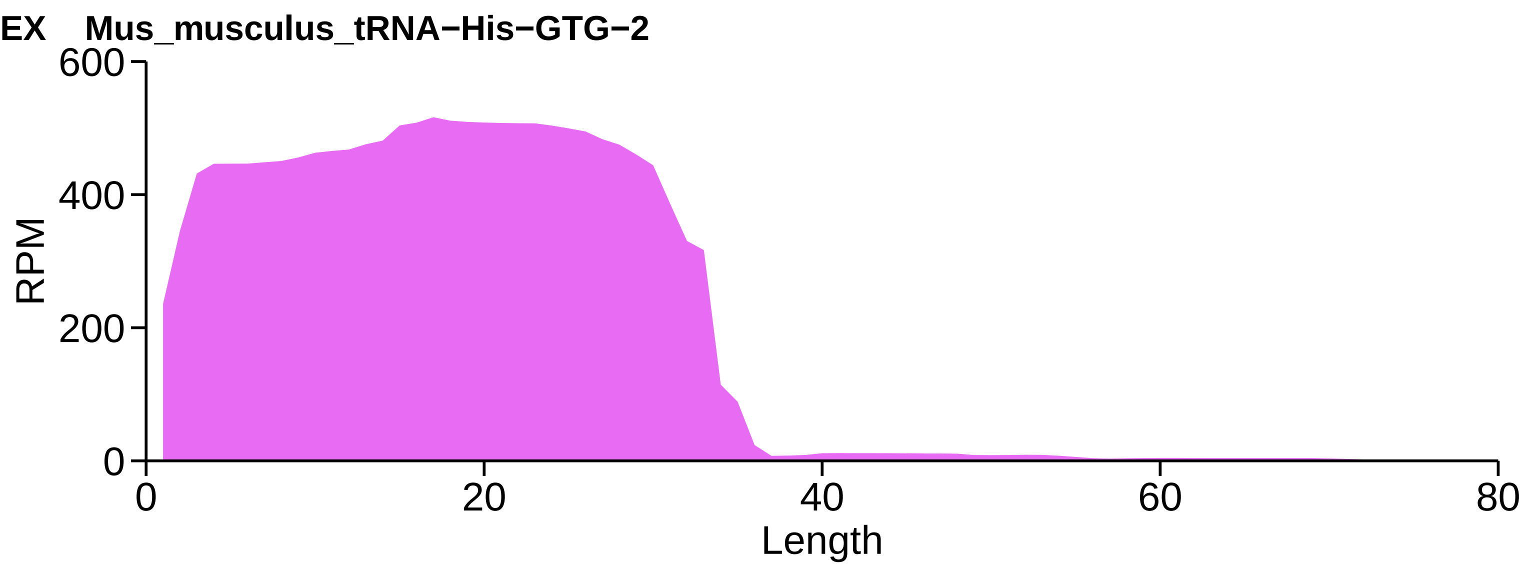

EX Mus_musculus_tRNA−His−GTG−2
600
400
200
0
RPM
0
20
40
60
80
Length

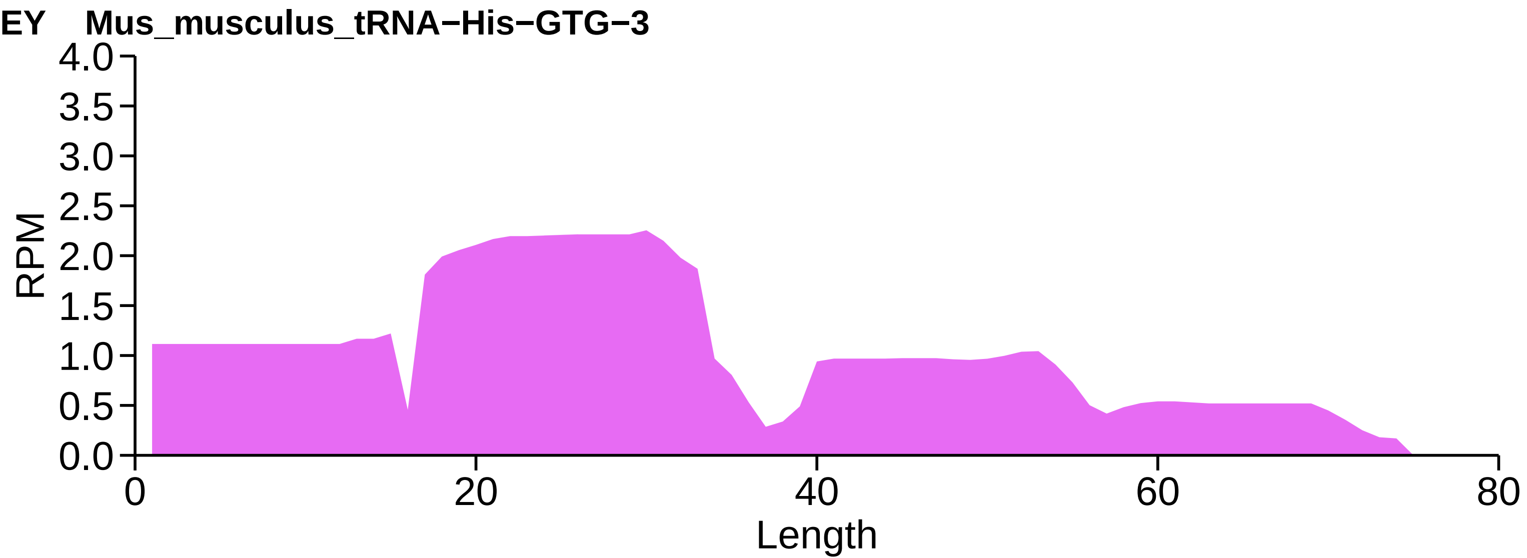
EY Mus_musculus_tRNA−His−GTG−3
RPM
Length
0.0
0.5
1.0
1.5
2.0
2.5
3.0
3.5
4.0
0
20
40
60
80

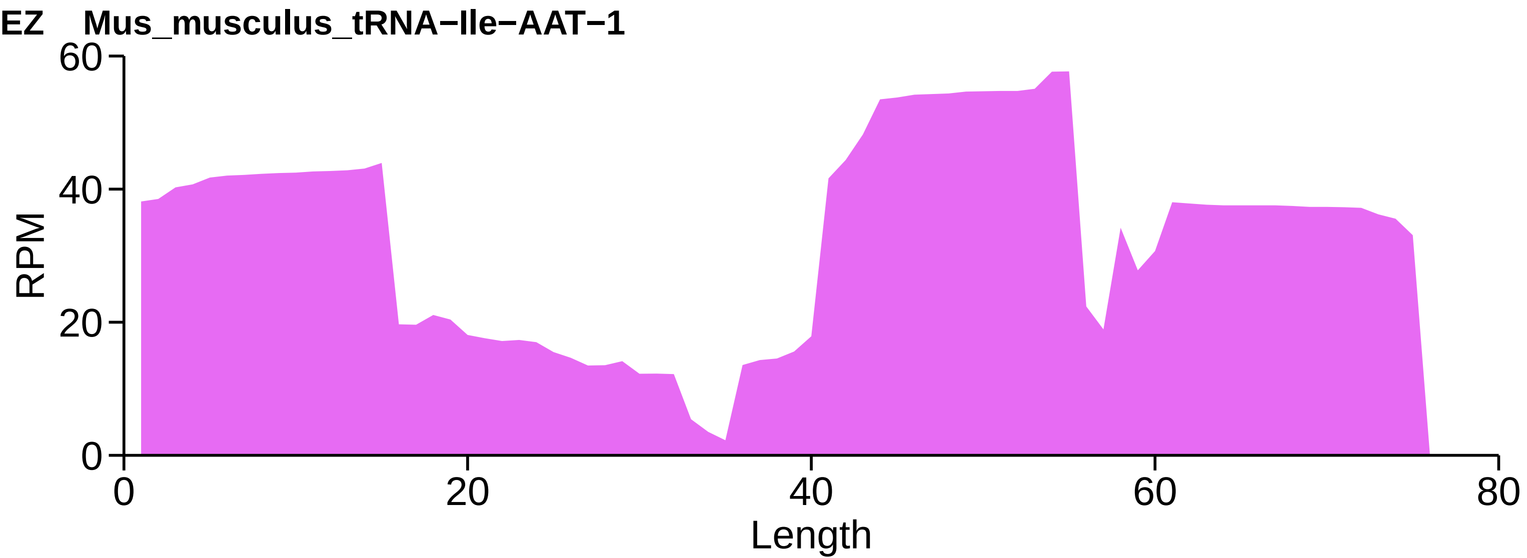
EZ Mus_musculus_tRNA−Ile−AAT−1
RPM
60
40
20
0
0
20
40
60
80
Length

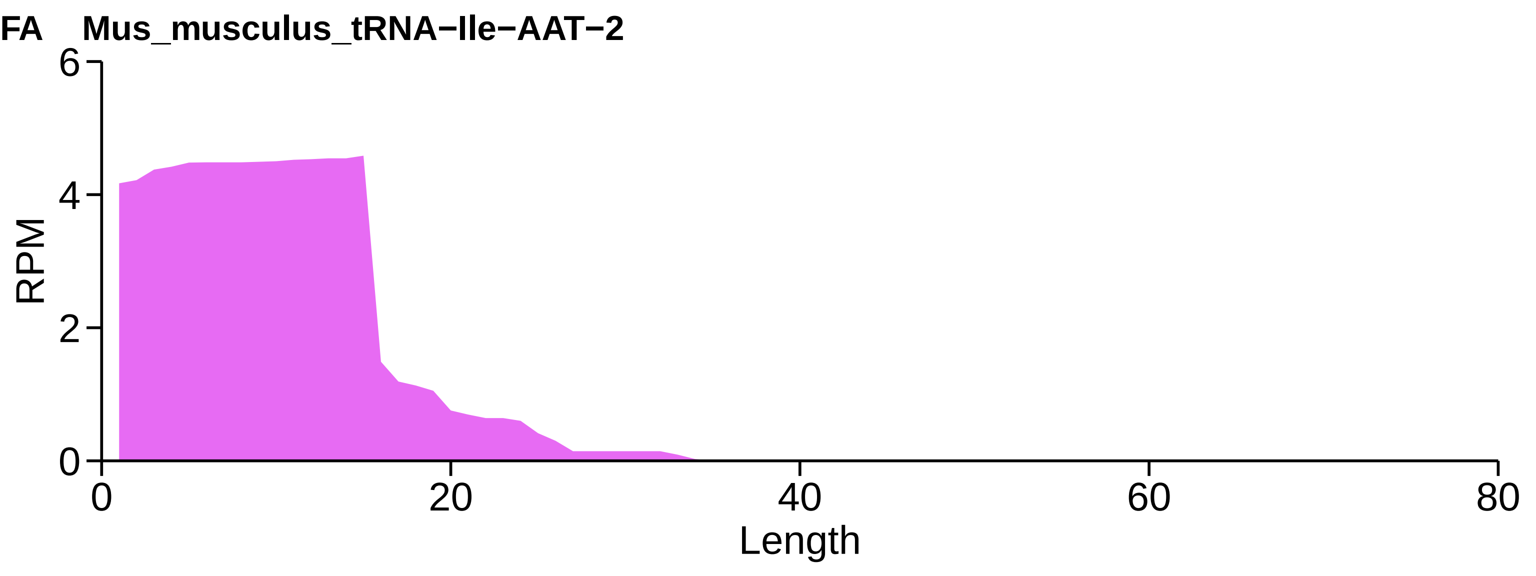

FA Mus_musculus_tRNA−Ile−AAT−2
RPM
6
4
2
0
0
20
40
60
80
Length

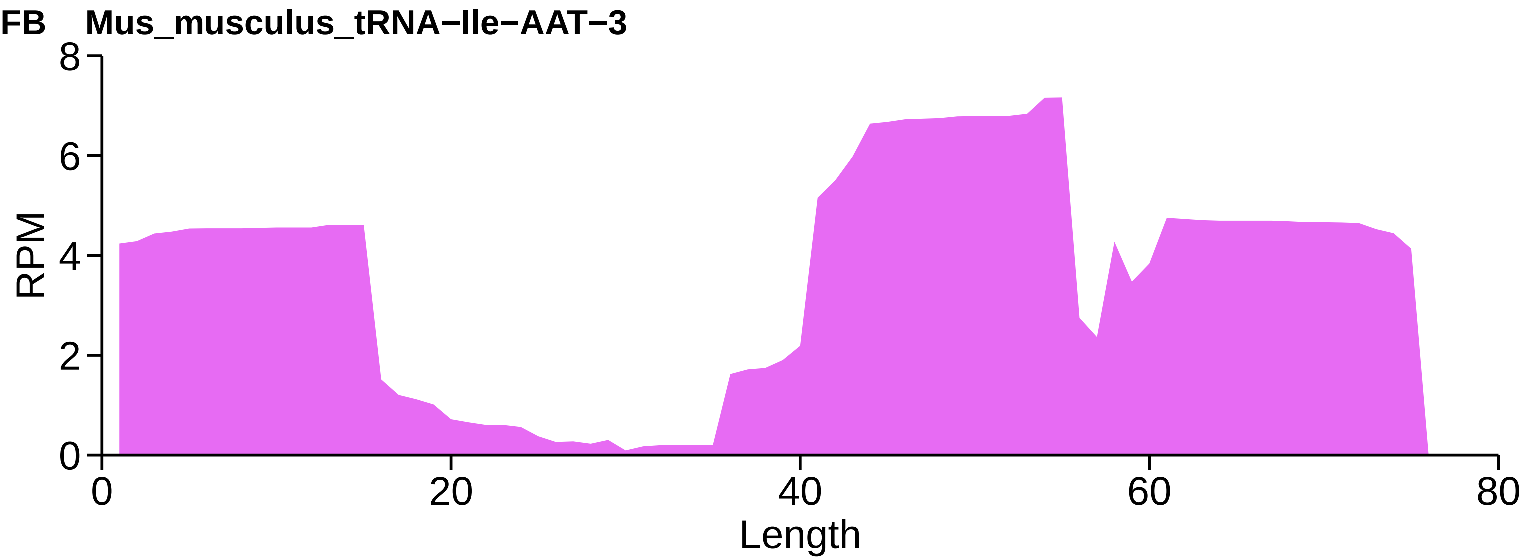
FB Mus_musculus_tRNA−Ile−AAT−3
RPM
Length
0
2
4
6
8
0
20
40
60
80

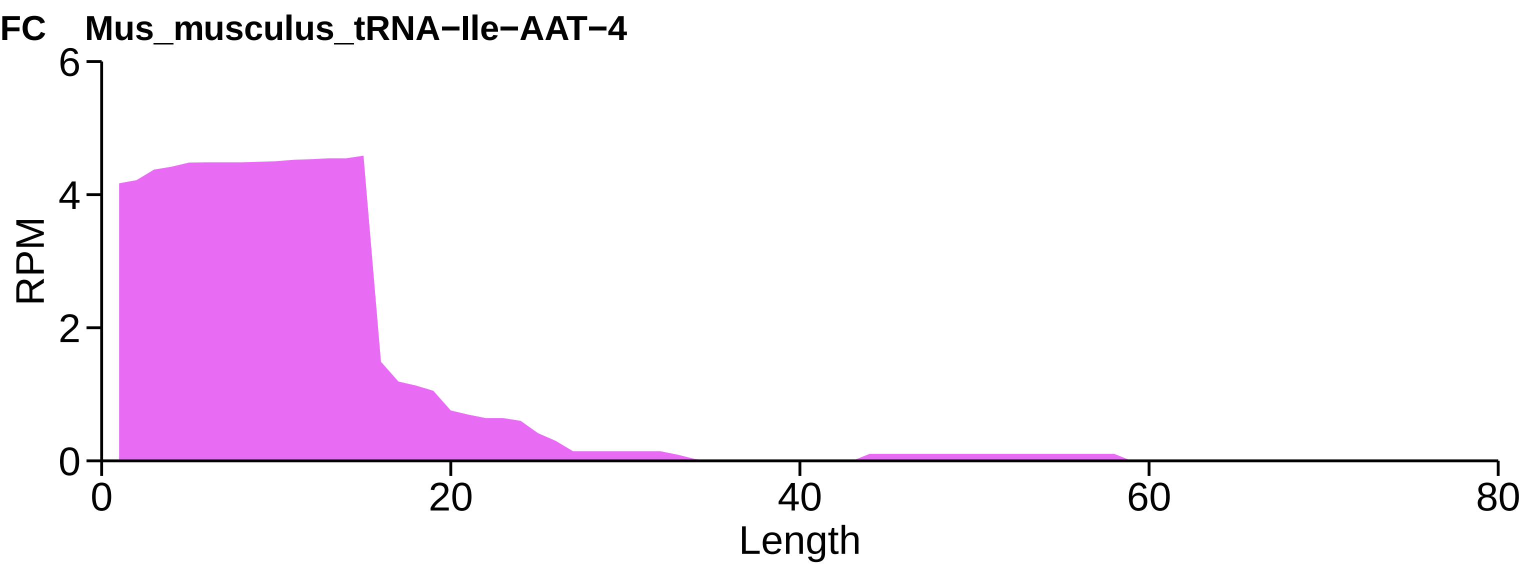
FC Mus_musculus_tRNA−Ile−AAT−4
RPM
Length
0
2
4
6
0
20
40
60
80

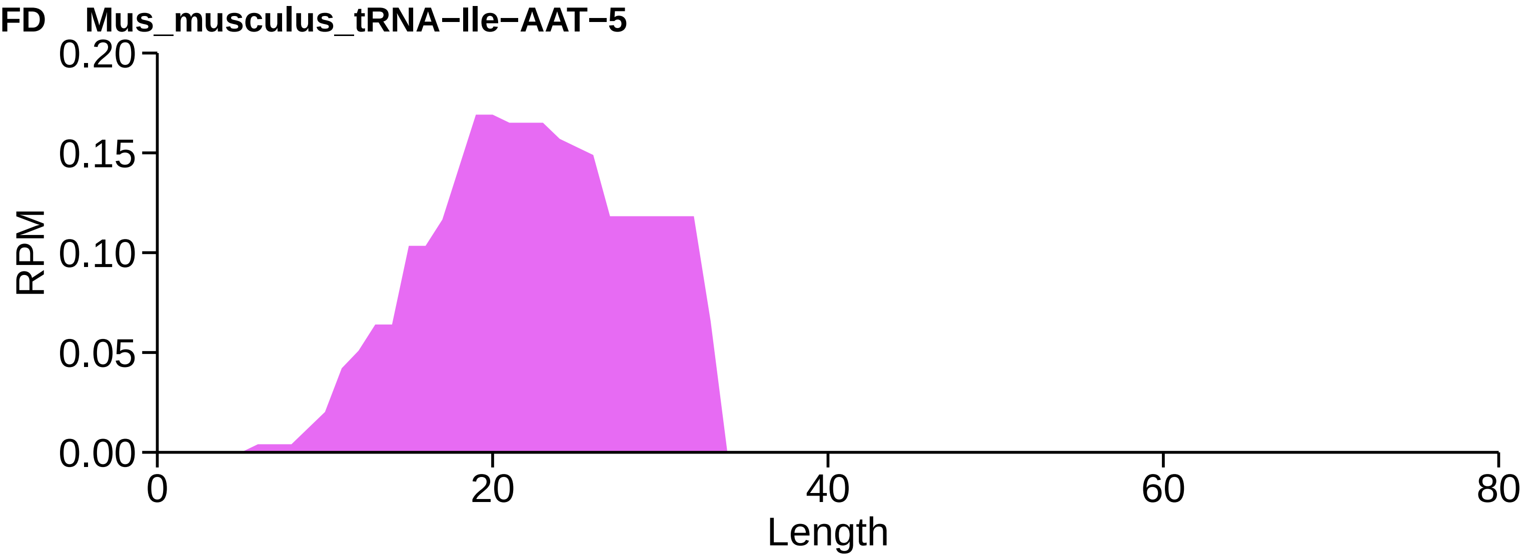

FD Mus_musculus_tRNA−Ile−AAT−5
RPM
0.00
0.05
0.10
0.15
0.20
0
20
40
60
80
Length

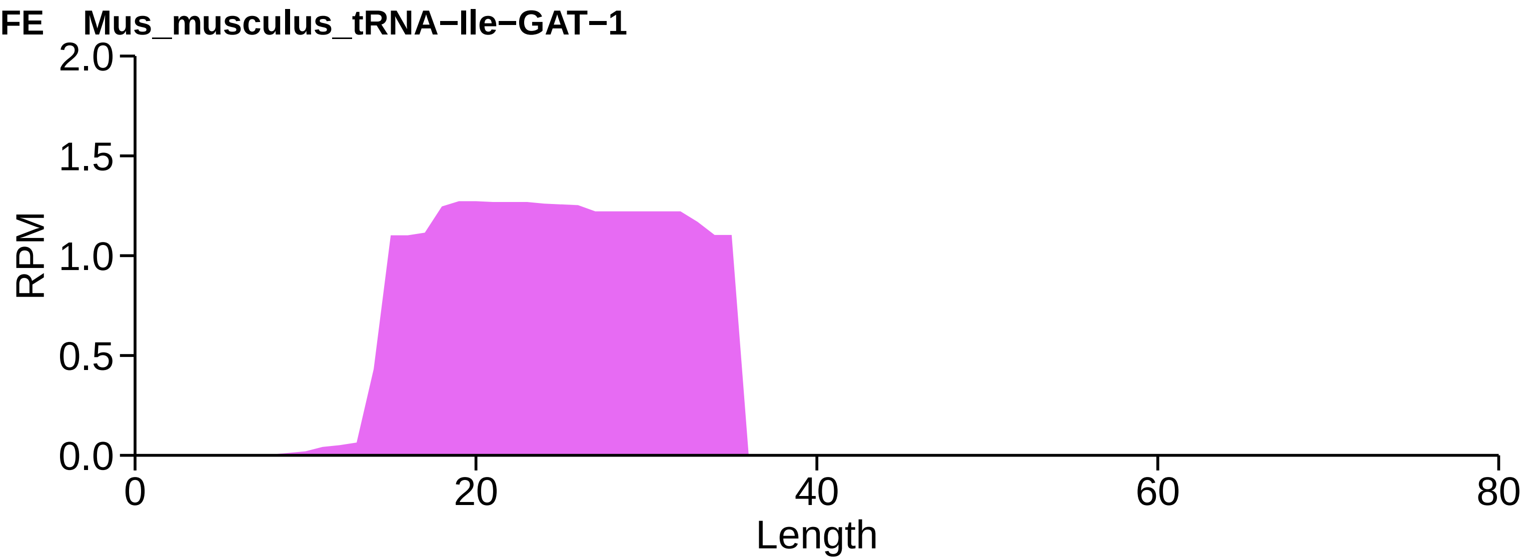

FE Mus_musculus_tRNA−Ile−GAT−1
RPM
2.0
1.5
1.0
0.5
0.0
0
20
40
60
80
Length

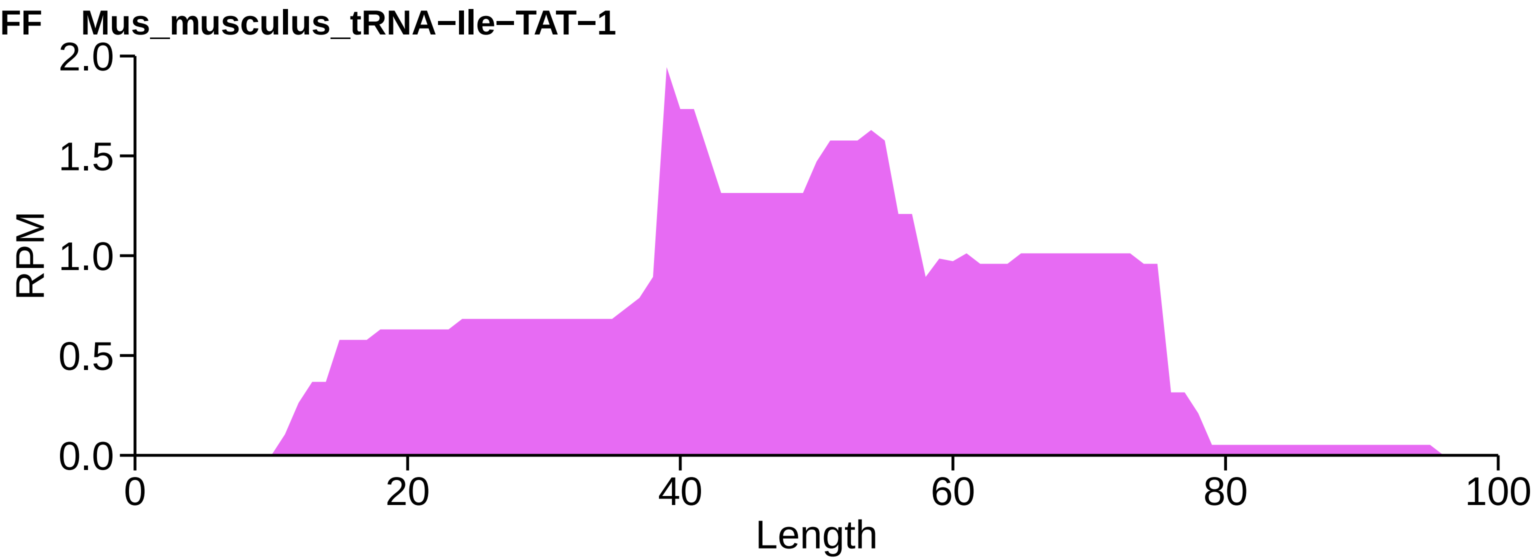
FF Mus_musculus_tRNA−Ile−TAT−1
RPM
0.0
0.5
1.0
1.5
2.0
0
20
40
60
80
100
Length

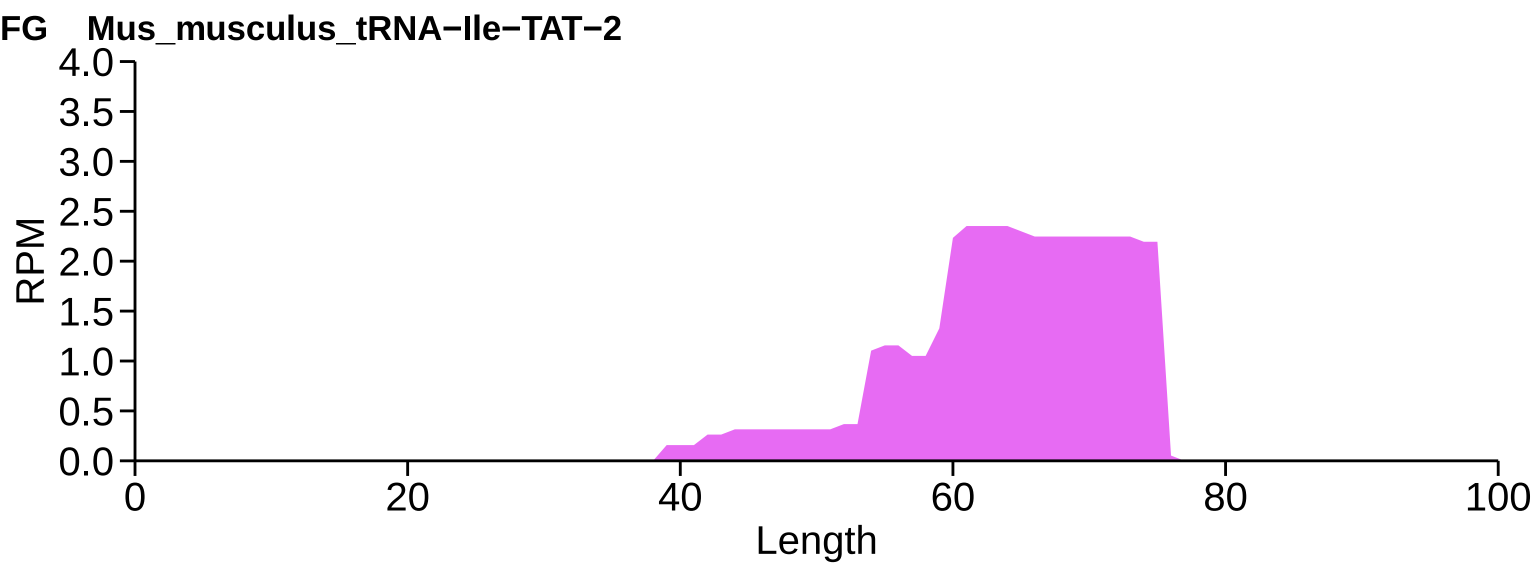
FG Mus_musculus_tRNA−Ile−TAT−2
RPM
4.0
3.5
3.0
2.5
2.0
1.5
1.0
0.5
0.0
0
20
40
60
80
100
Length

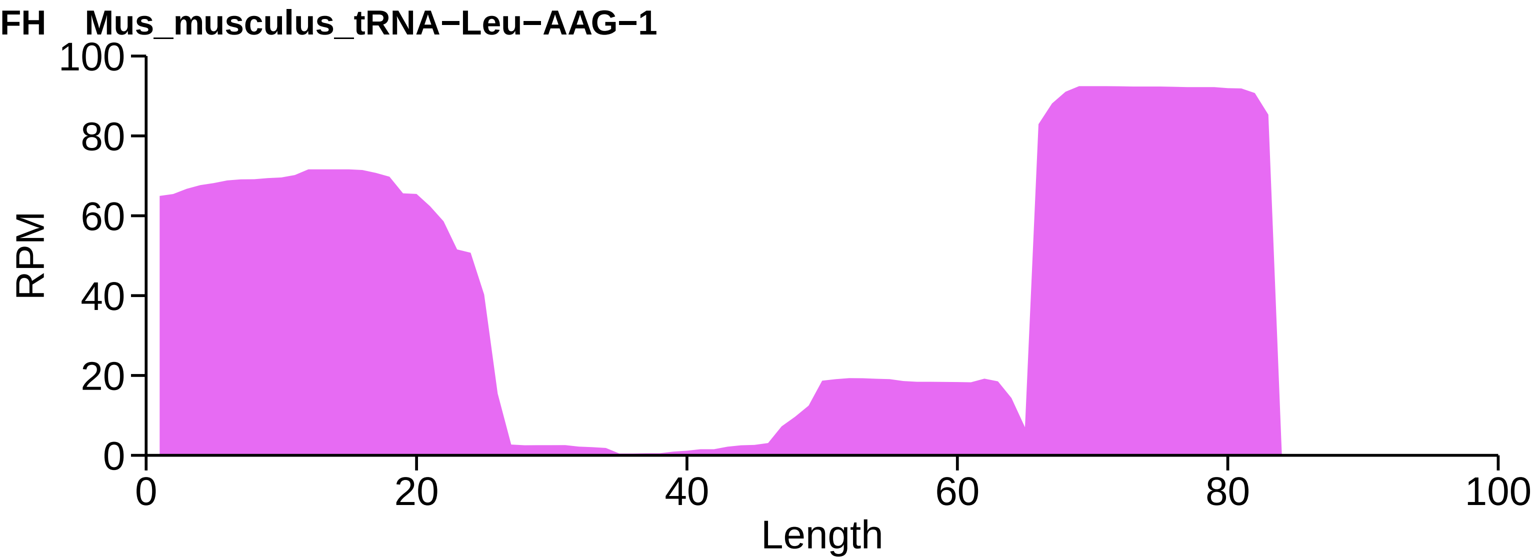
FH Mus_musculus_tRNA−Leu−AAG−1
RPM
Length
0
20
40
60
80
100

FI Mus_musculus_tRNA−Leu−AAG−2

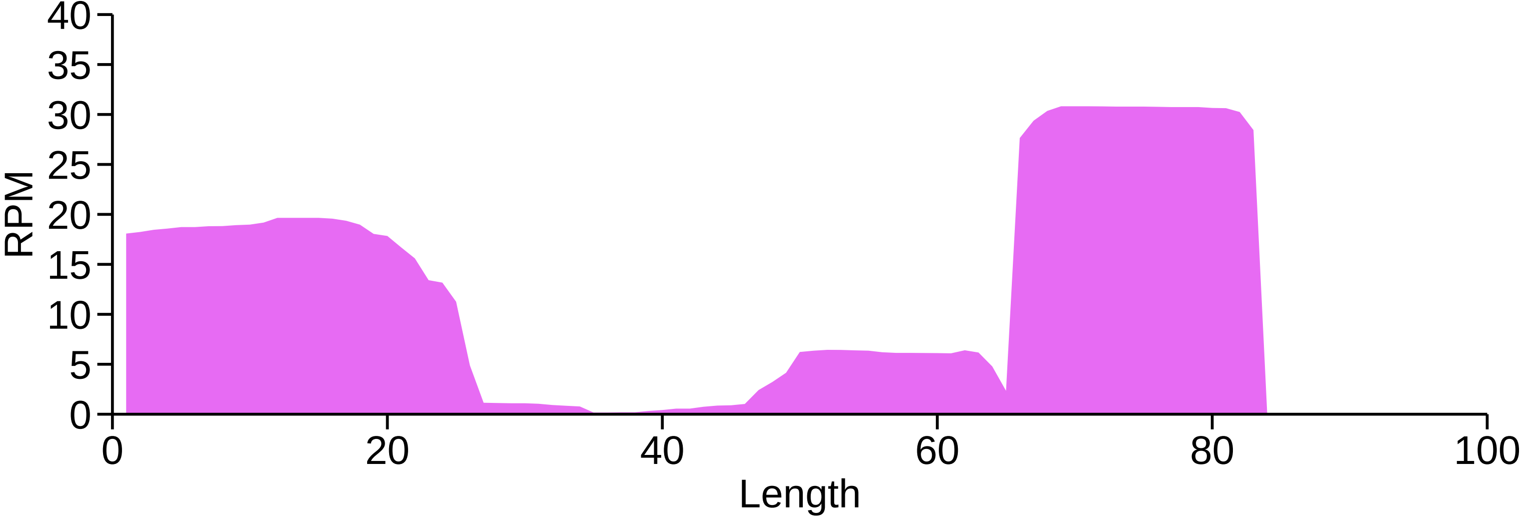

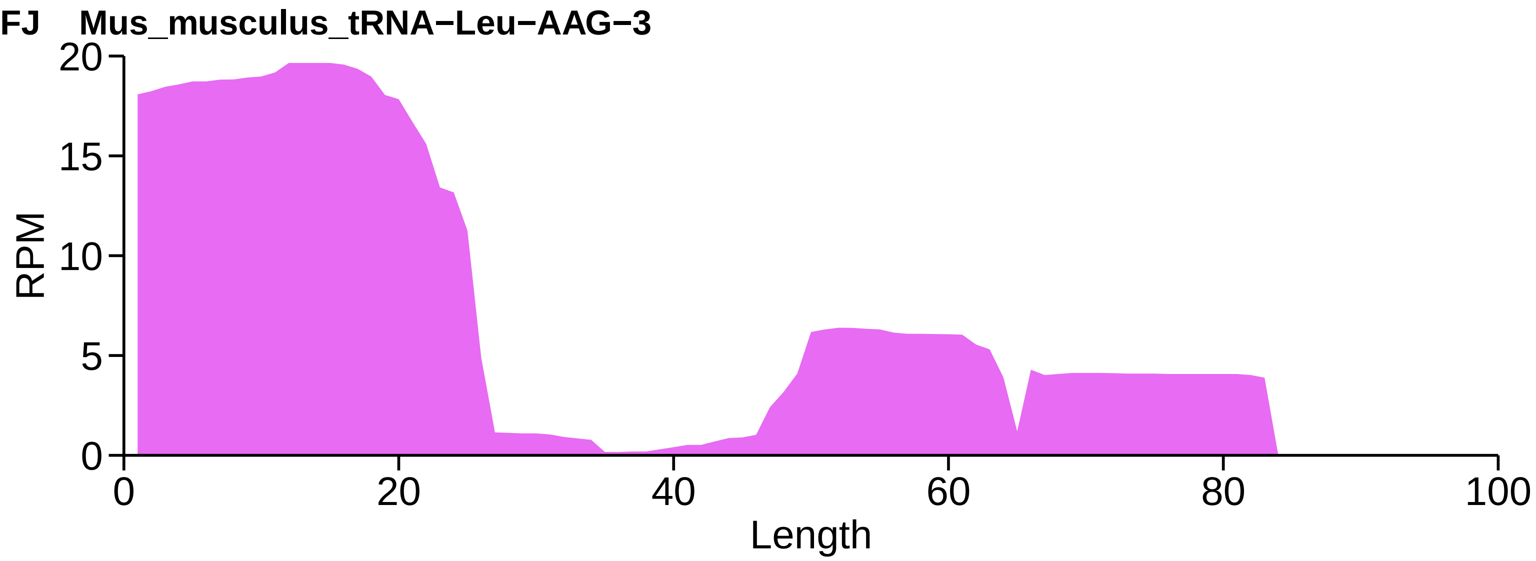

FJ Mus_musculus_tRNA−Leu−AAG−3
RPM
0
5
10
15
20
0
20
40
60
80
100
Length

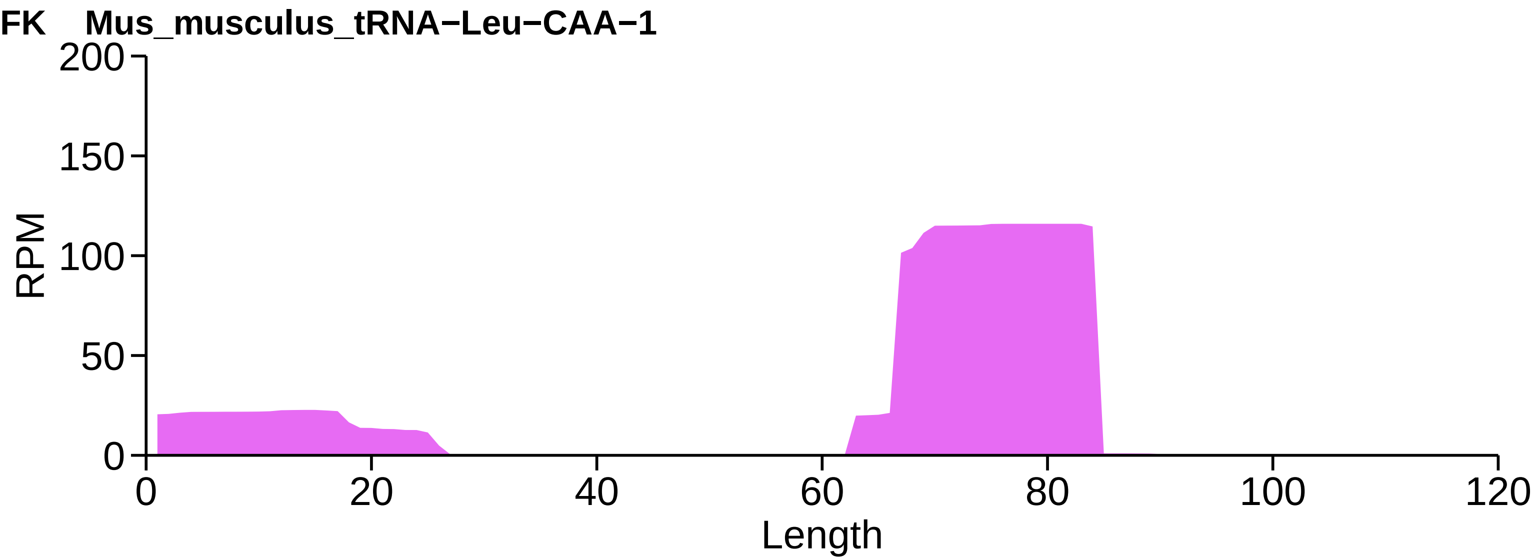
FK Mus_musculus_tRNA−Leu−CAA−1
RPM
200
150
100
50
0
0
20
40
60
80
100
120
Length

FL Mus_musculus_tRNA−Leu−CAA−2

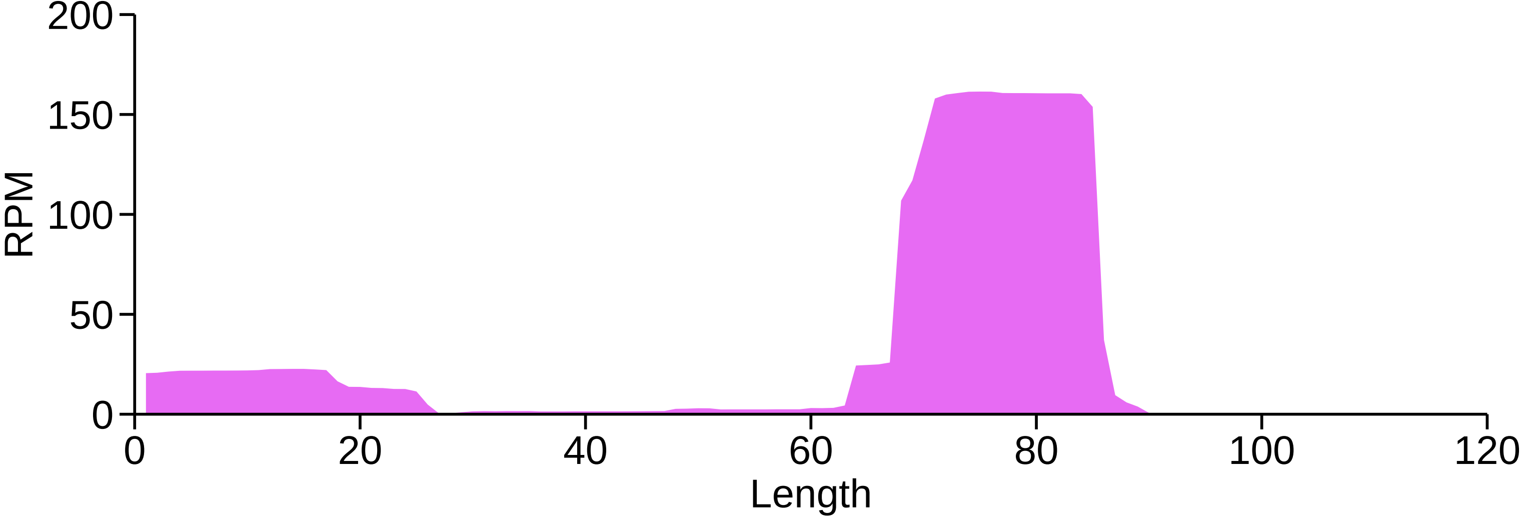

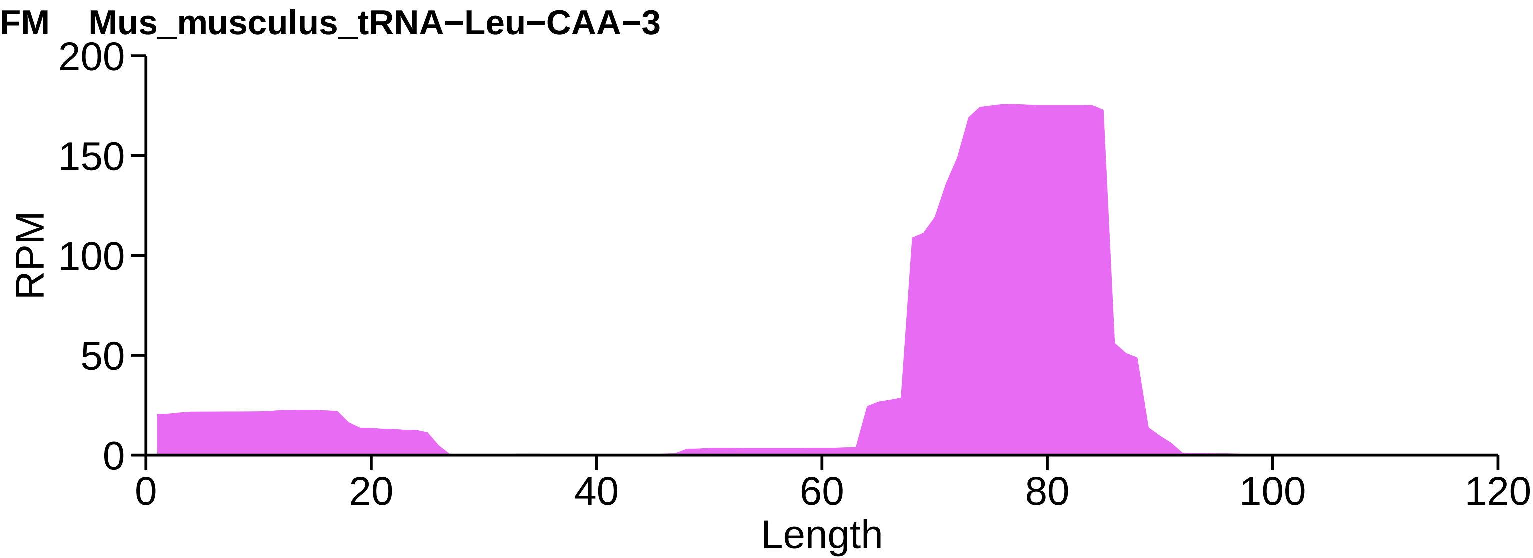
FM Mus_musculus_tRNA−Leu−CAA−3
RPM
200
150
100
50
0
0
20
40
60
80
100
120
Length

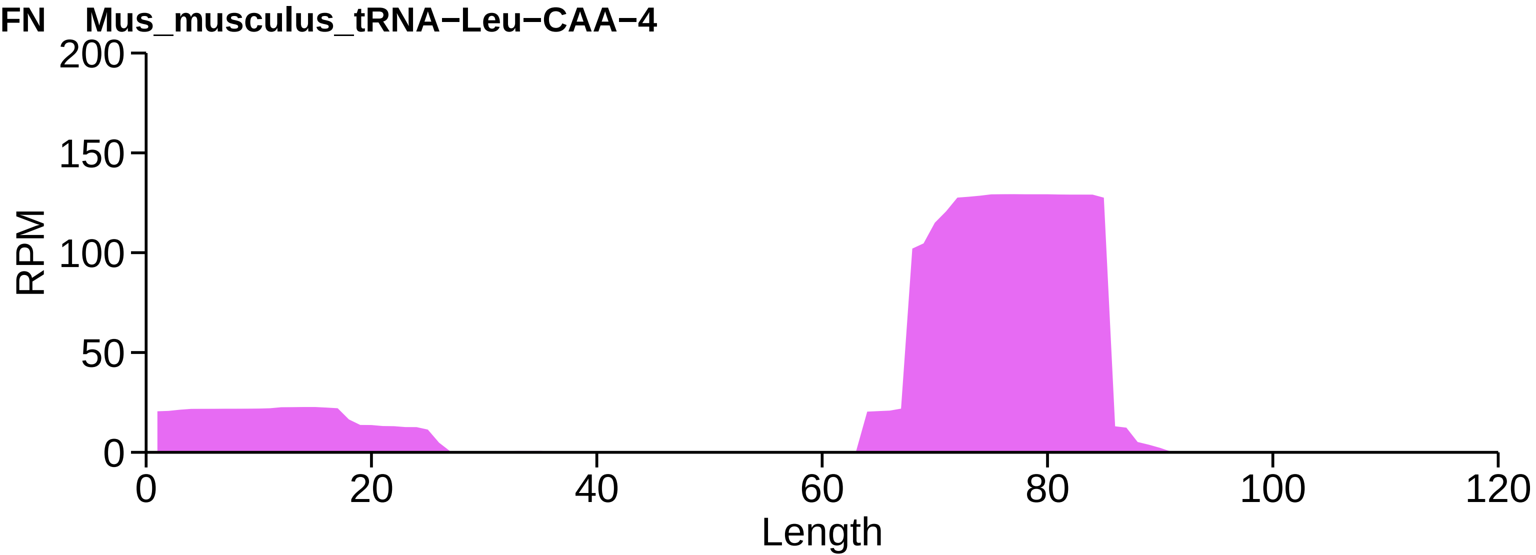

FN Mus_musculus_tRNA−Leu−CAA−4
RPM
200
150
100
50
0
0
20
40
60
80
100
120
Length

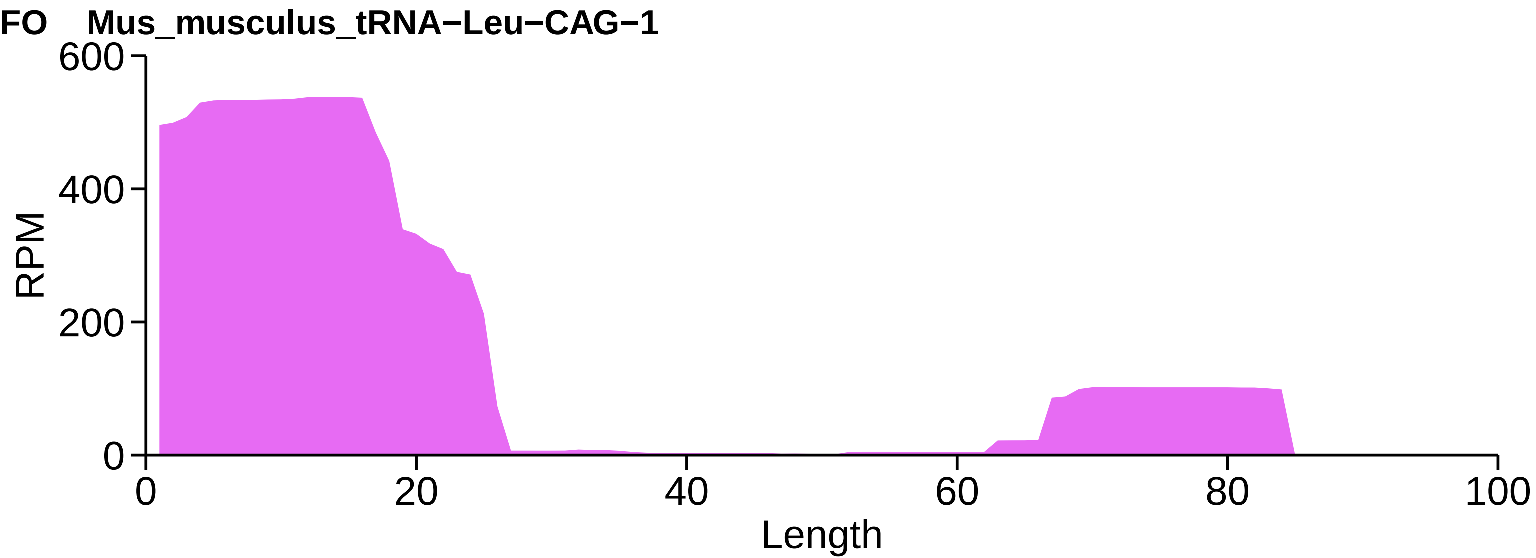
FO Mus_musculus_tRNA−Leu−CAG−1
600
400
200
0
RPM
0
20
40
60
80
100
Length

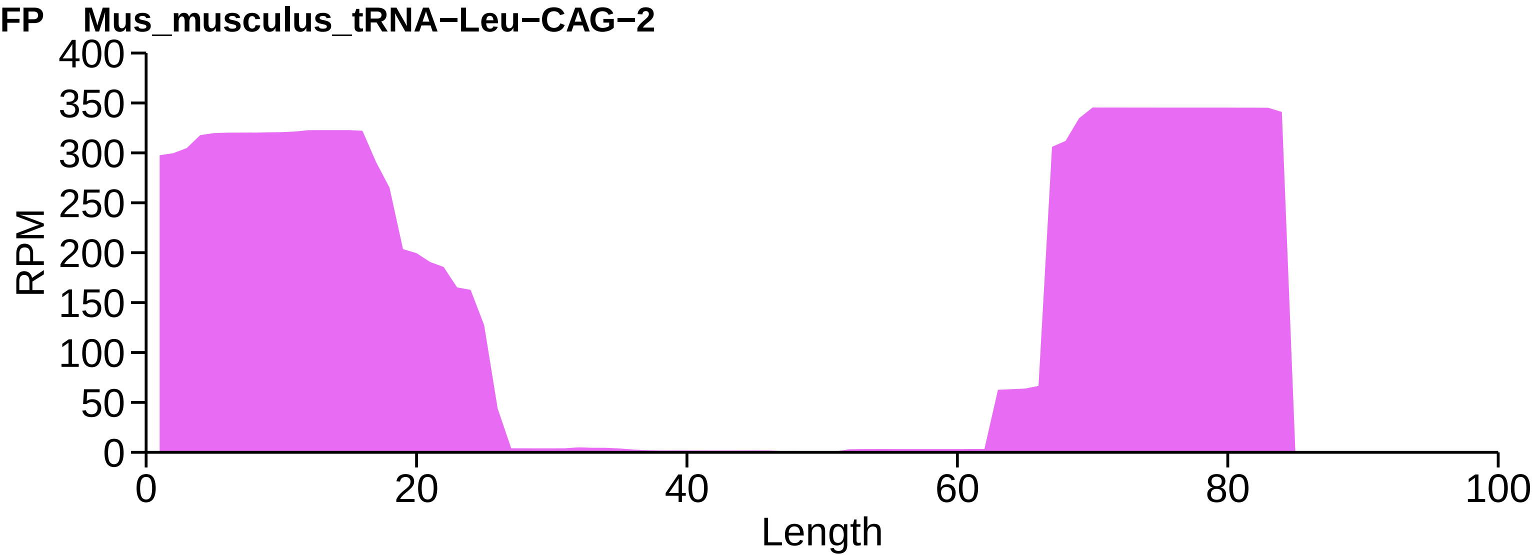
FP Mus_musculus_tRNA−Leu−CAG−2
RPM
400
350
300
250
200
150
100
50
0
0
20
40
60
80
100
Length

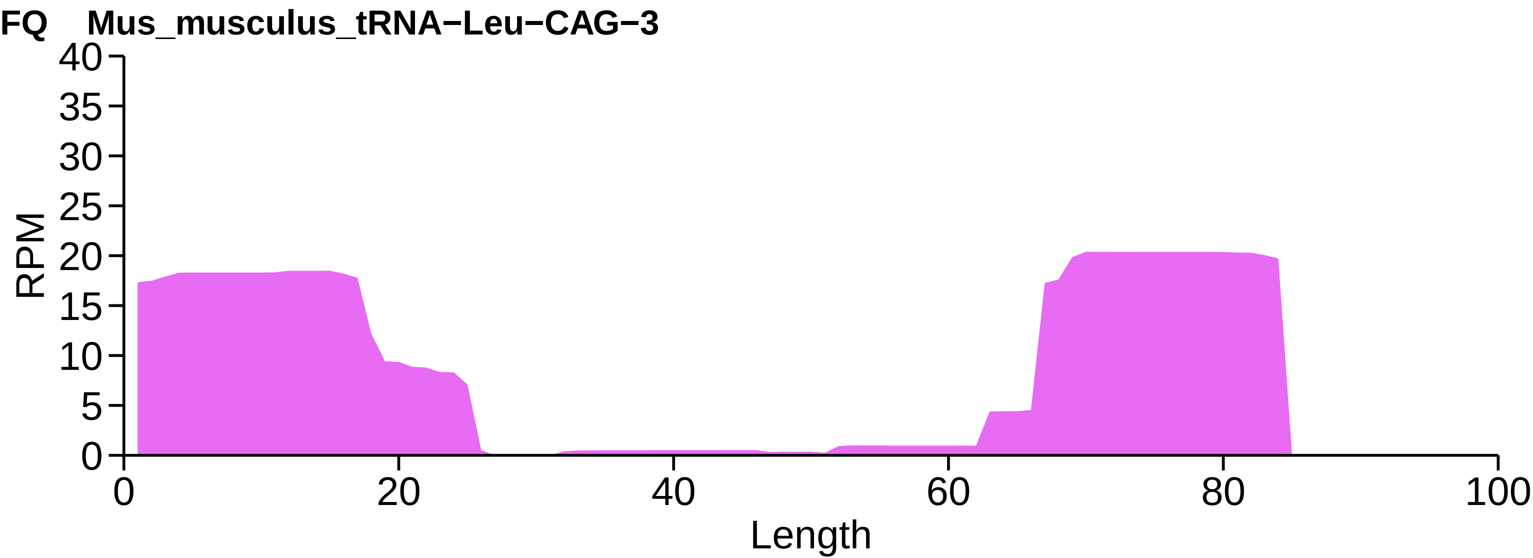
FQ Mus_musculus_tRNA−Leu−CAG−3
RPM
Length
0
5
10
15
20
25
30
35
40
0
20
40
60
80
100

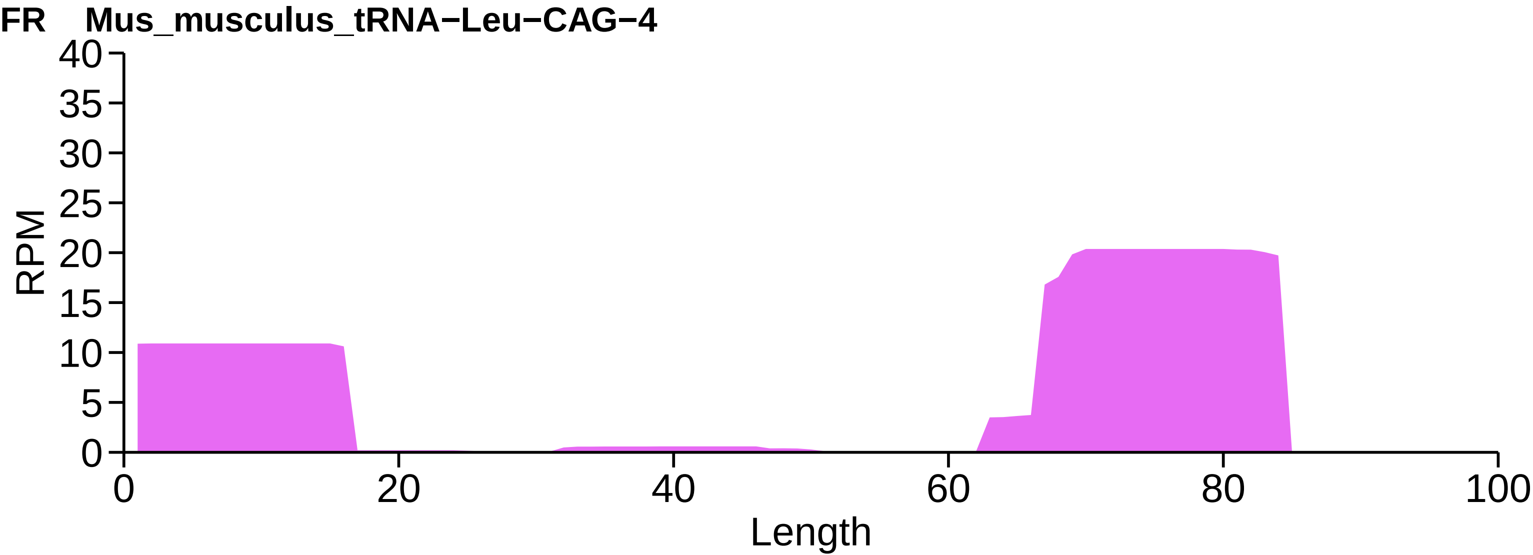

FR Mus_musculus_tRNA−Leu−CAG−4
RPM
40
35
30
25
20
15
10
5
0
0
20
40
60
80
100
Length

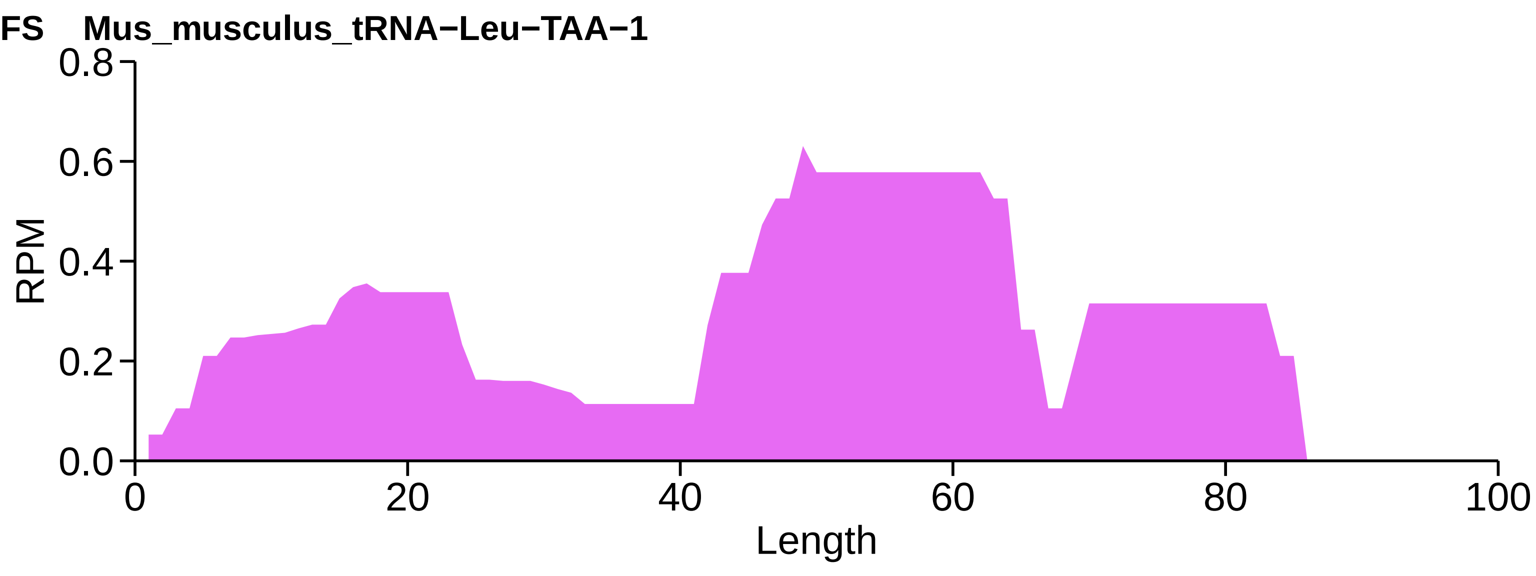

FS Mus_musculus_tRNA−Leu−TAA−1
0.8
0.6
0.4
0.2
0.0
RPM
0
20
40
60
80
100
Length

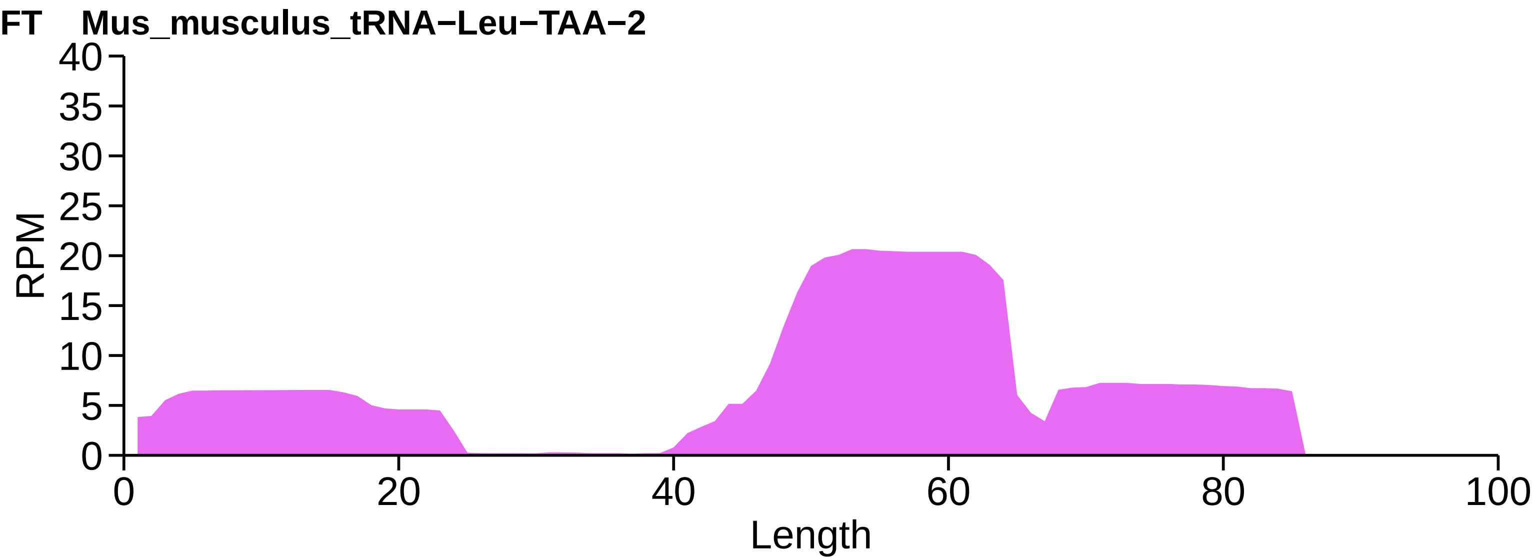
FT Mus_musculus_tRNA−Leu−TAA−2
RPM
Length
0
5
10
15
20
25
30
35
40
0
20
40
60
80
100

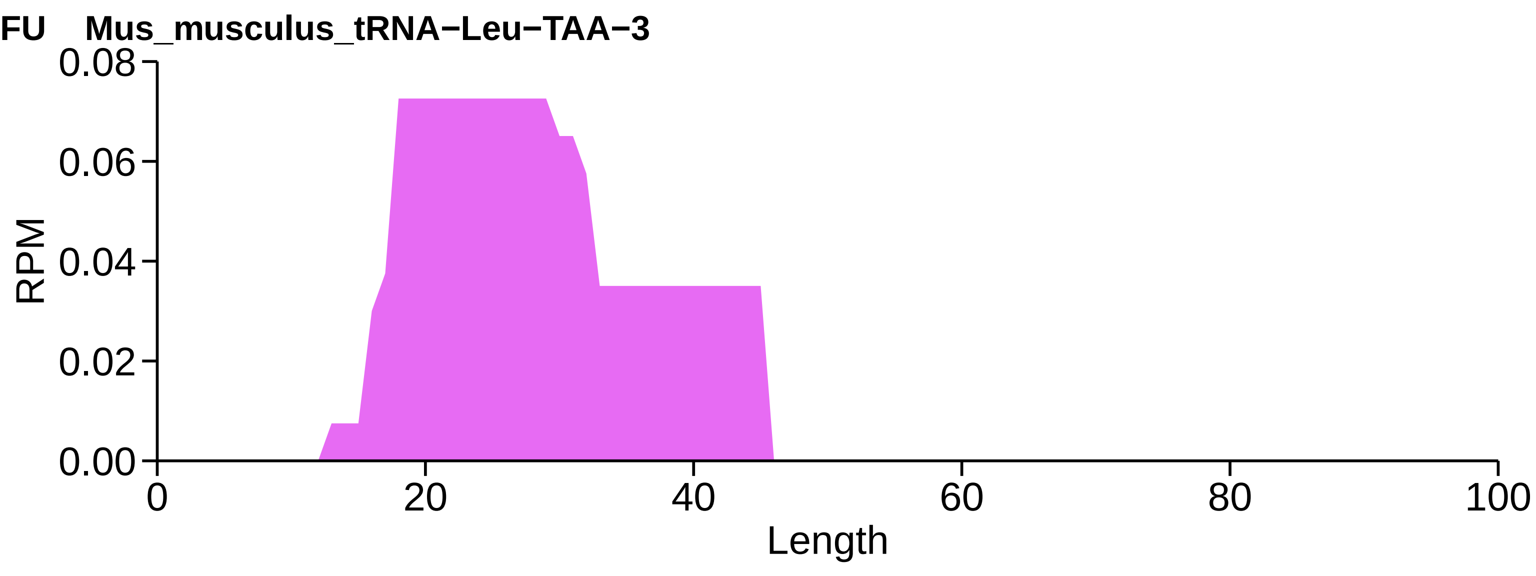
FU Mus_musculus_tRNA−Leu−TAA−3
0.08
0.06
0.04
0.02
0.00
RPM
0
20
40
60
80
100
Length

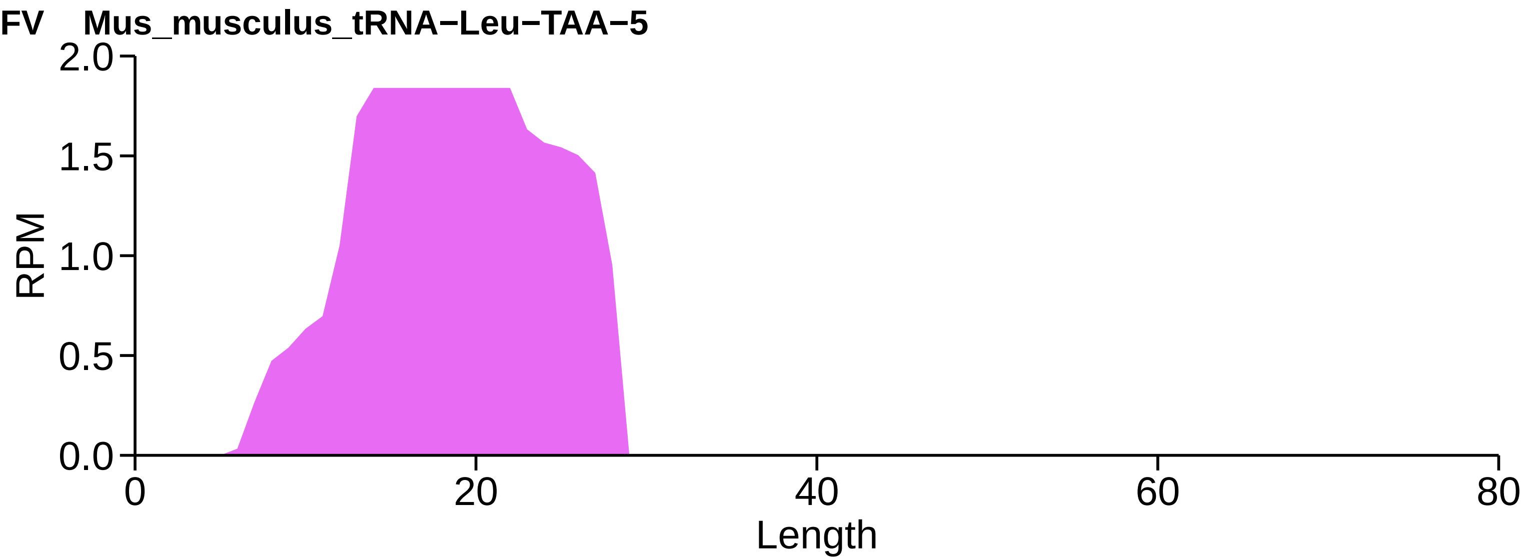
FV Mus_musculus_tRNA−Leu−TAA−5
RPM
2.0
1.5
1.0
0.5
0.0
0
20
40
60
80
Length

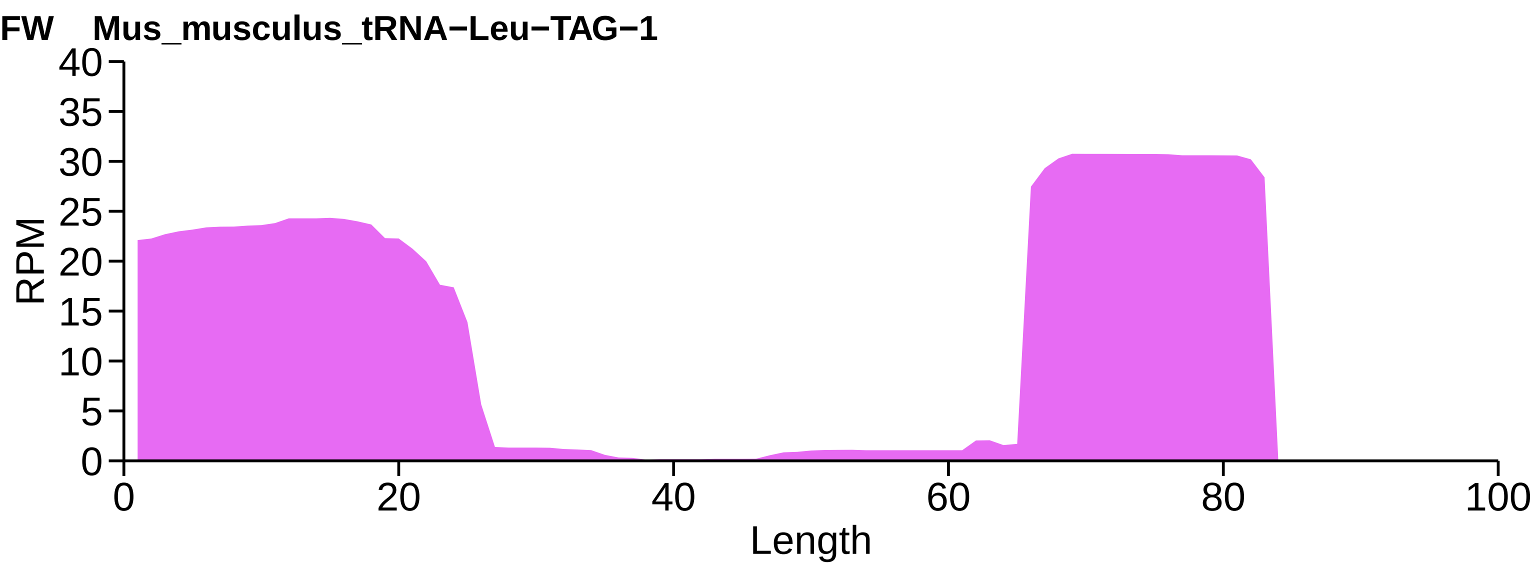

FW Mus_musculus_tRNA−Leu−TAG−1
RPM
Length
0
5
10
15
20
25
30
35
40
0
20
40
60
80
100

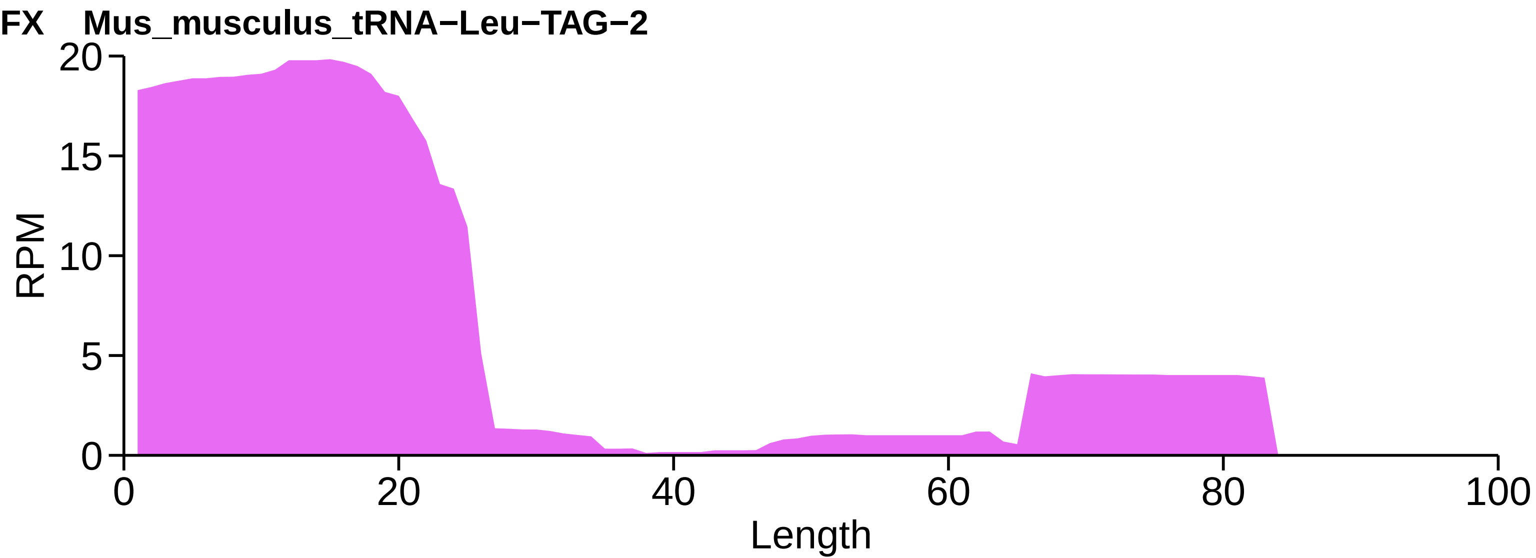
FX Mus_musculus_tRNA−Leu−TAG−2
RPM
0
5
10
15
20
0
20
40
60
80
100
Length

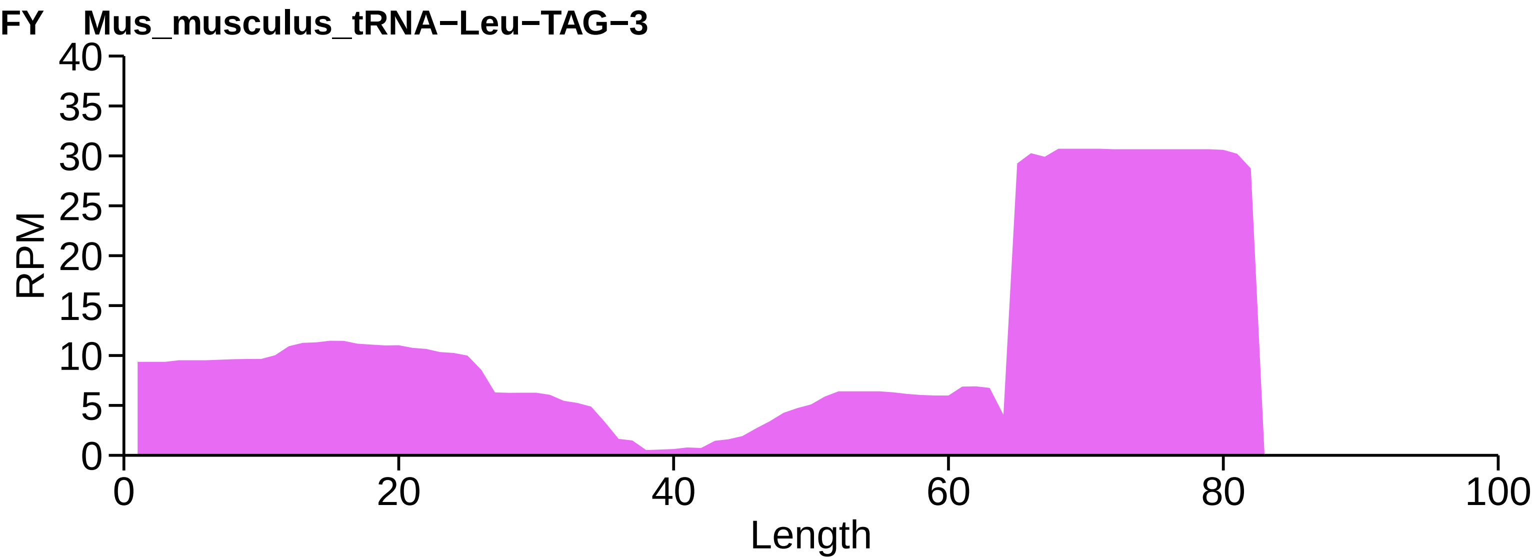

FY Mus_musculus_tRNA−Leu−TAG−3
RPM
Length
0
5
10
15
20
25
30
35
40
0
20
40
60
80
100

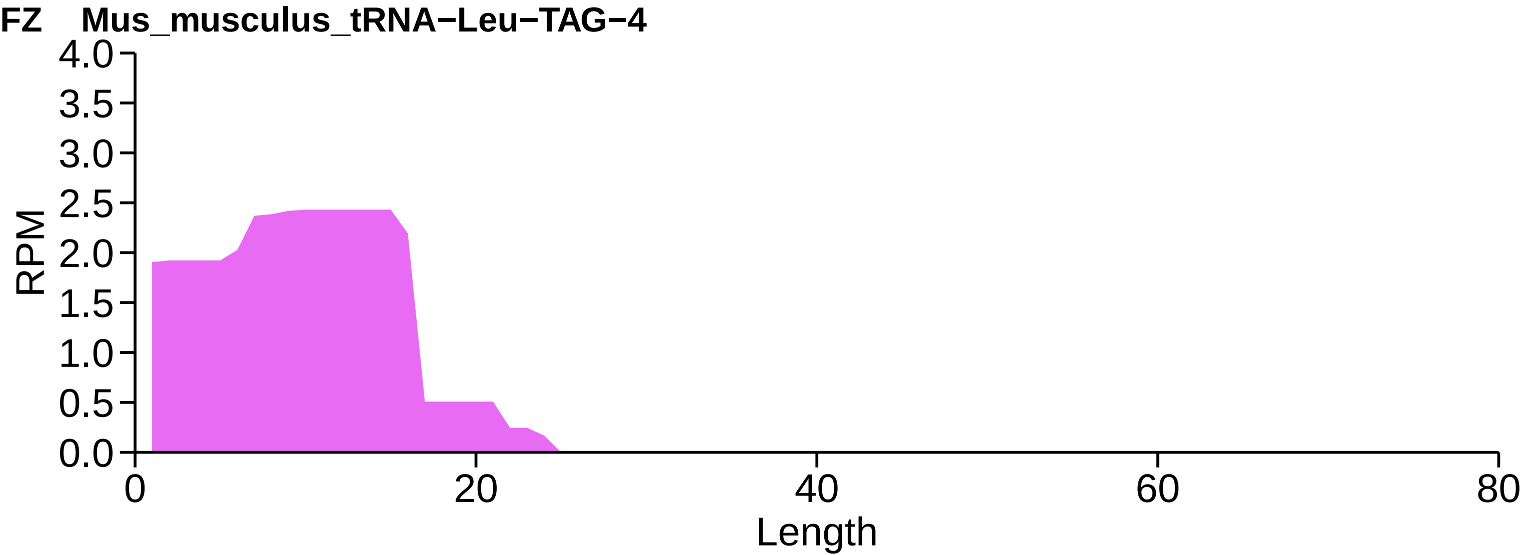
FZ Mus_musculus_tRNA−Leu−TAG−4
4.0
3.5
3.0
2.5
2.0
1.5
1.0
0.5
0.0
RPM
0
20
40
60
80
Length

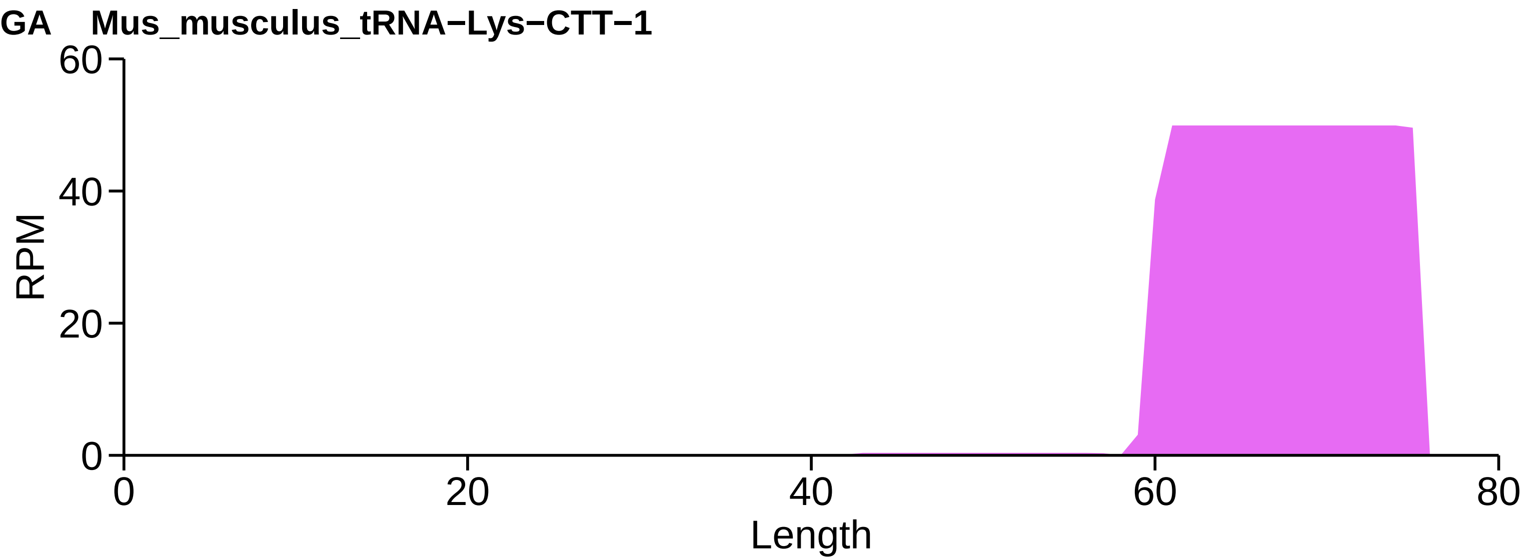
GA Mus_musculus_tRNA−Lys−CTT−1
60
40
20
0
RPM
0
20
40
60
80
Length

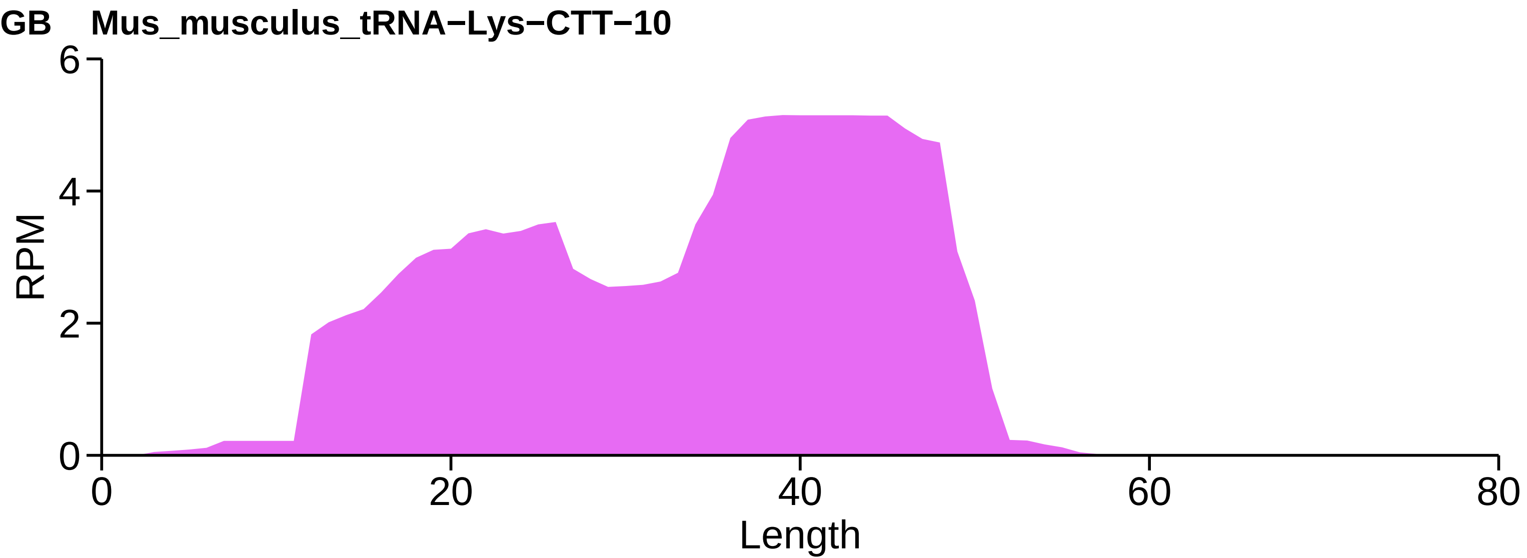

GB Mus_musculus_tRNA−Lys−CTT−10
RPM
6
4
2
0
0
20
40
60
80
Length

GC **Mus_musculus_tRNA−Lys−CTT−11**

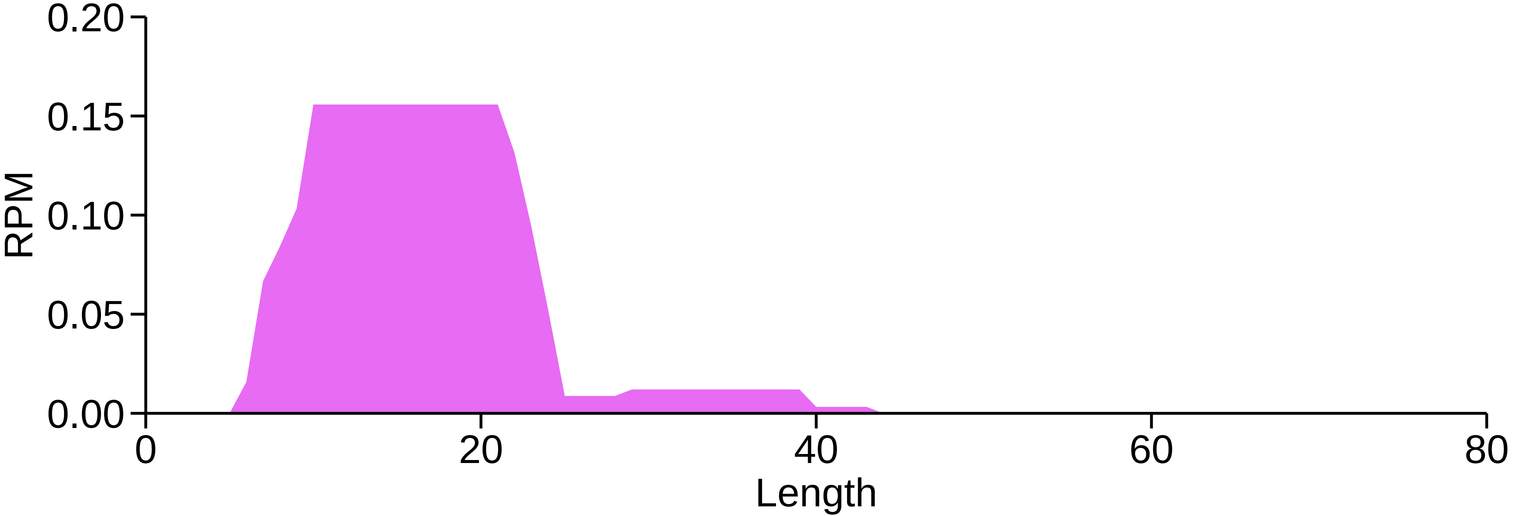

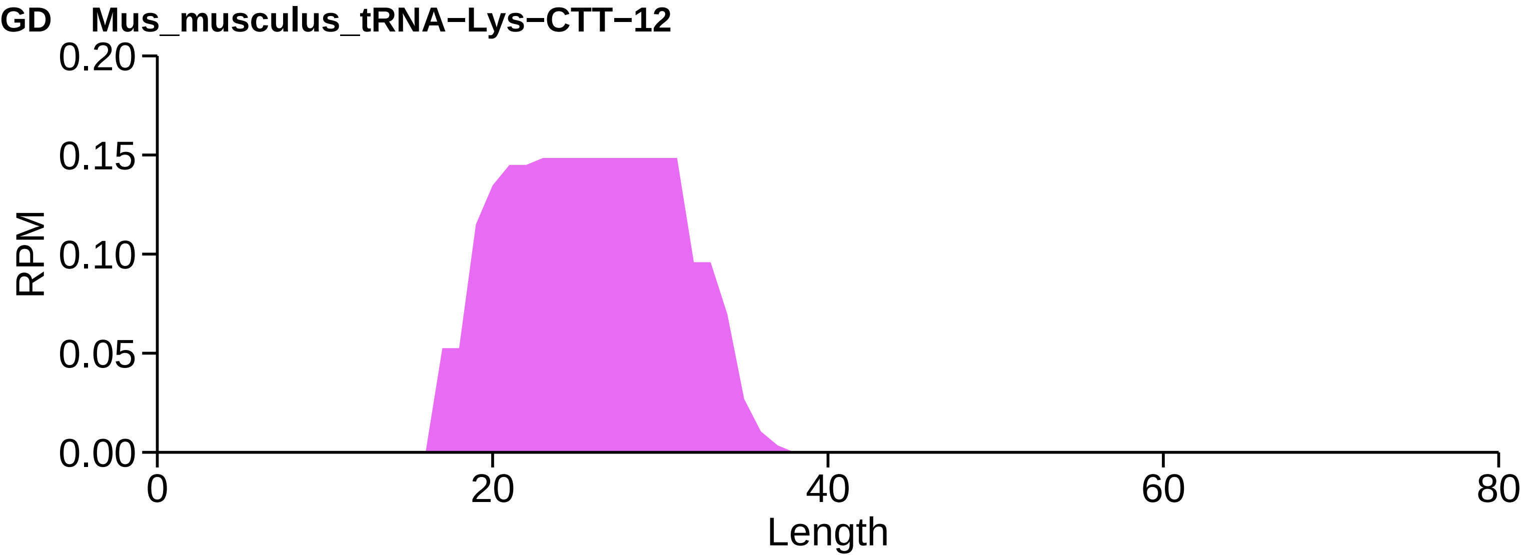
GD Mus_musculus_tRNA−Lys−CTT−12
0.20
0.15
0.10
0.05
0.00
RPM
0
20
40
60
80
Length

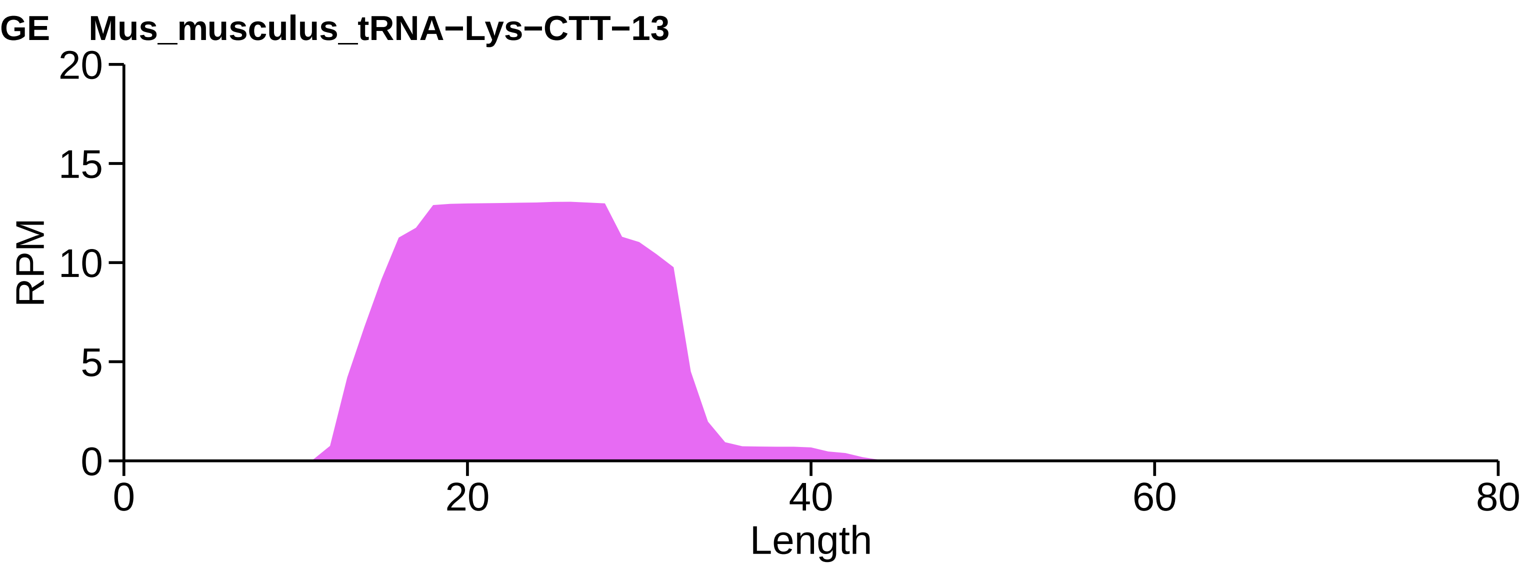

GE Mus_musculus_tRNA−Lys−CTT−13
RPM
Length
0
5
10
15
20
0
20
40
60
80

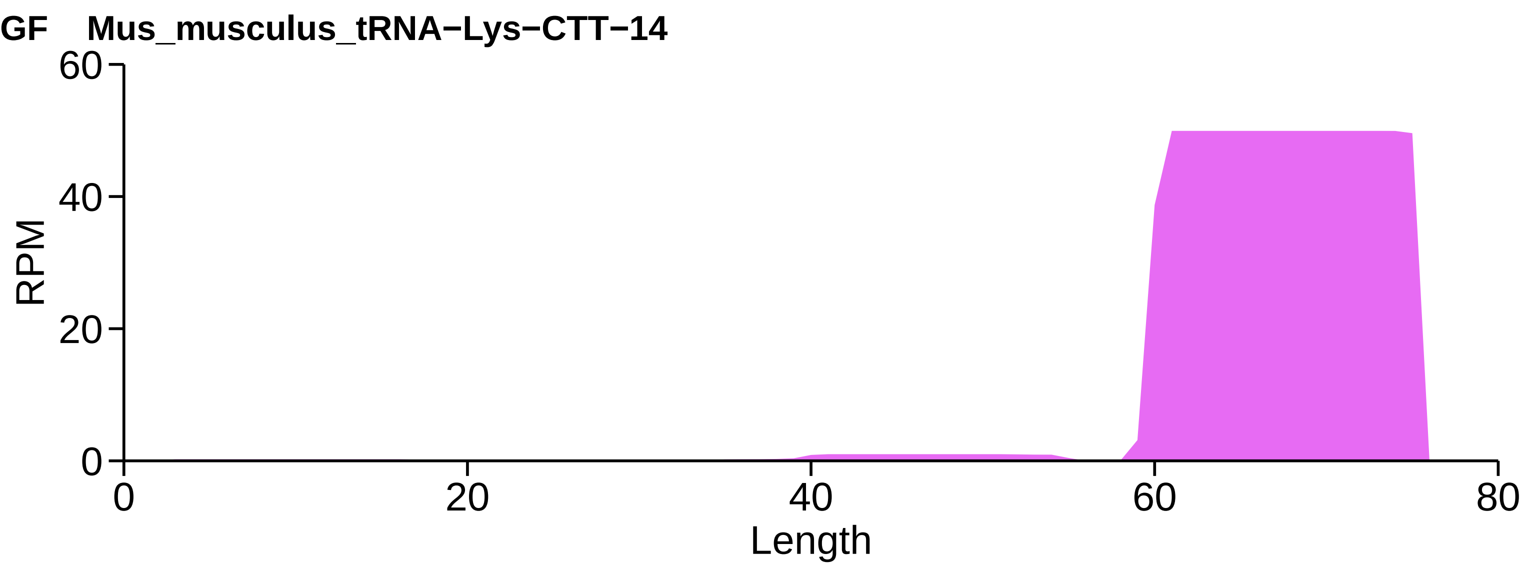
GF Mus_musculus_tRNA−Lys−CTT−14
60
40
20
0
RPM
0
20
40
60
80
Length

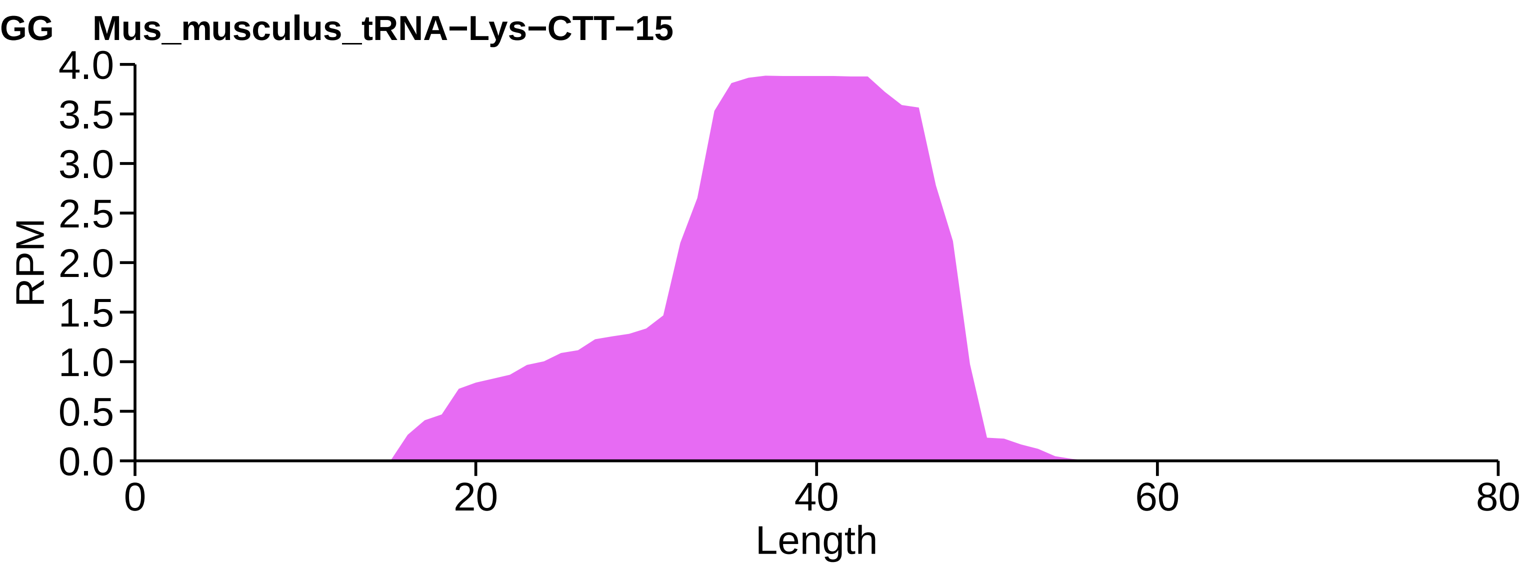
GG Mus_musculus_tRNA−Lys−CTT−15
RPM
Length
0.0
0.5
1.0
1.5
2.0
2.5
3.0
3.5
4.0
0
20
40
60
80

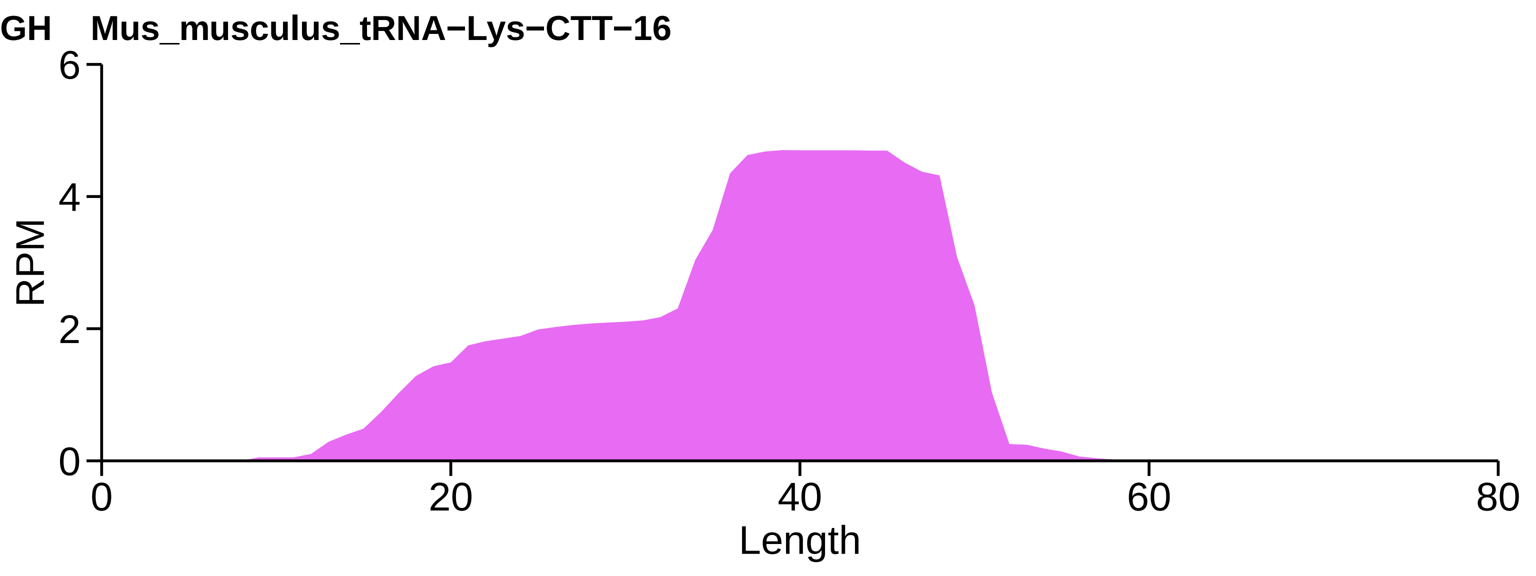

GH Mus_musculus_tRNA−Lys−CTT−16
RPM
0
2
4
6
0
20
40
60
80
Length

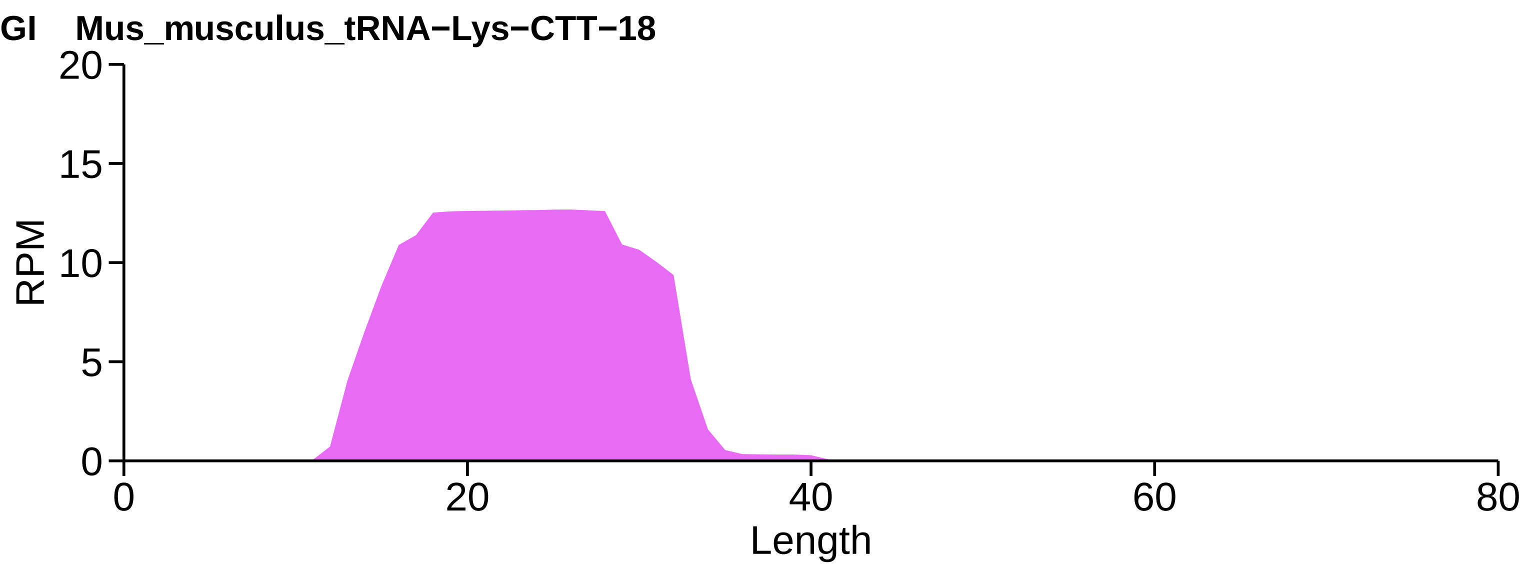
GI Mus_musculus_tRNA−Lys−CTT−18
RPM
Length
0
5
10
15
20
0
20
40
60
80

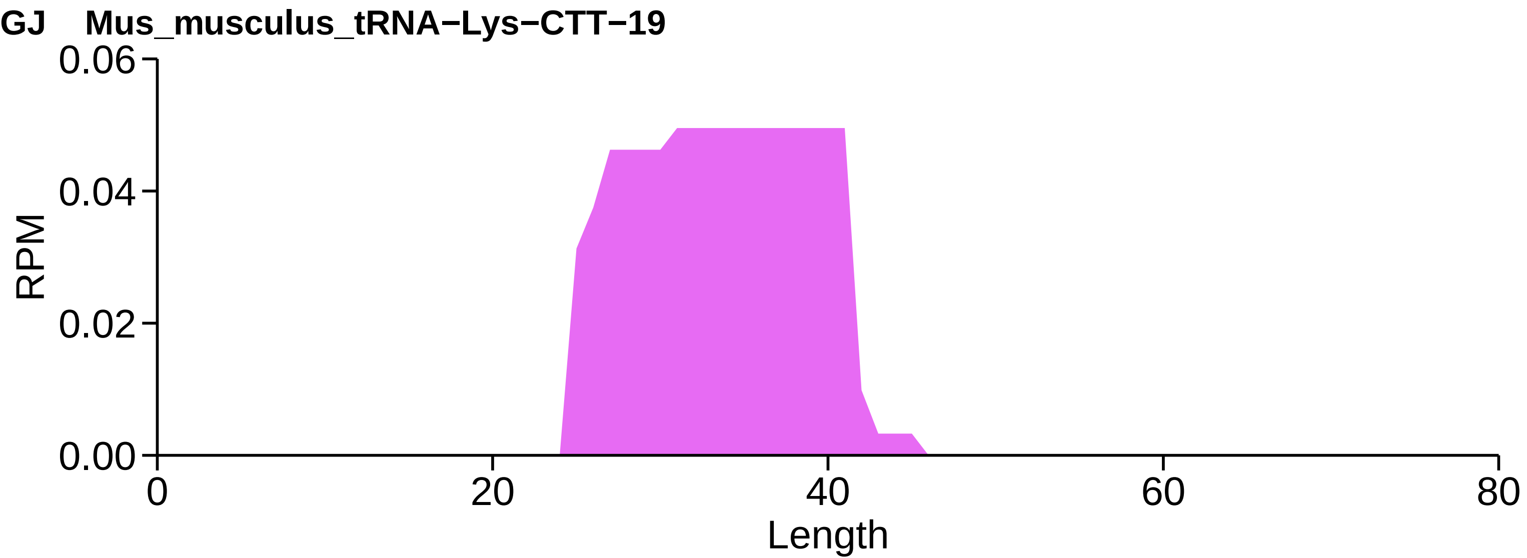
GJ Mus_musculus_tRNA−Lys−CTT−19
0.06
0.04
0.02
0.00
RPM
0
20
40
60
80
Length

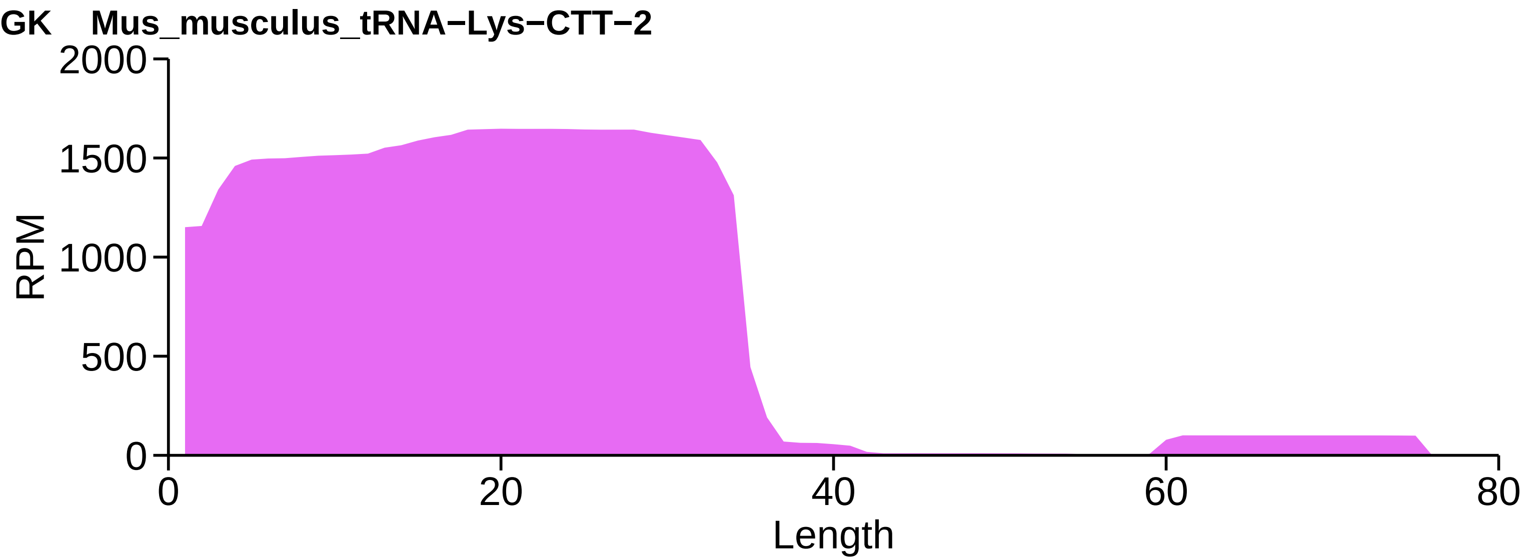

GK Mus_musculus_tRNA−Lys−CTT−2
RPM
2000
1500
1000
500
0
0
20
40
60
80
Length

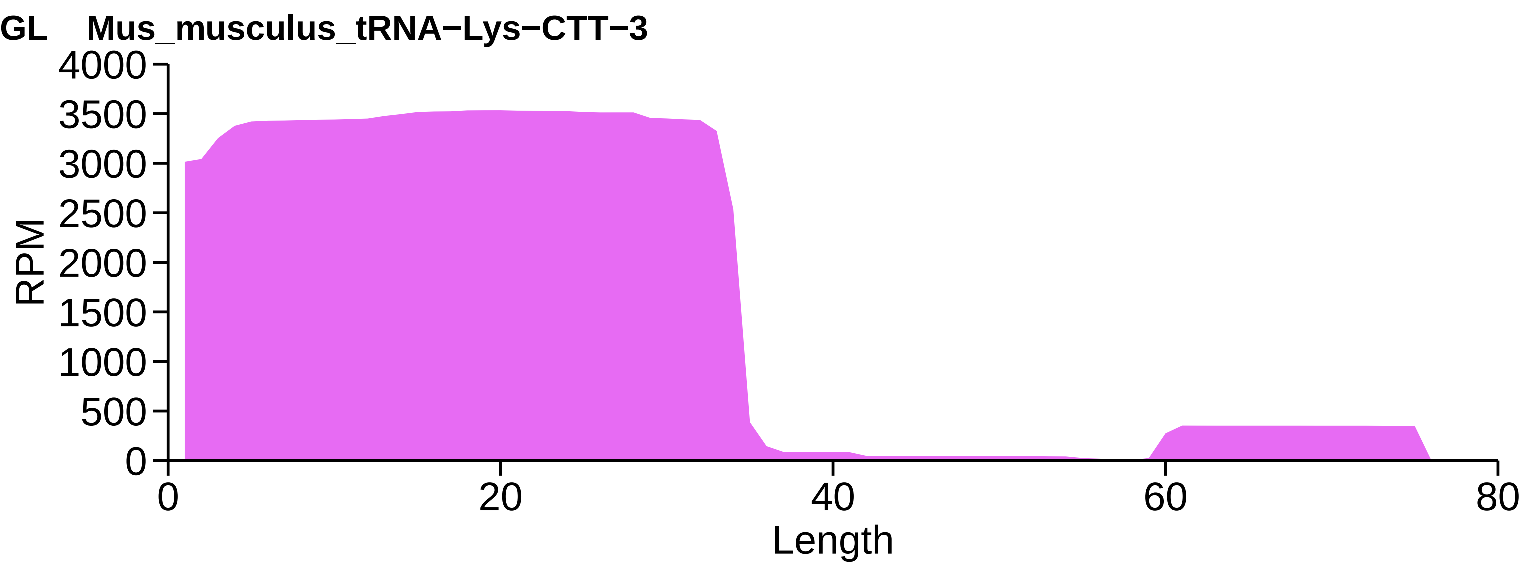
GL Mus_musculus_tRNA−Lys−CTT−3
RPM
4000
3500
3000
2500
2000
1500
1000
500
0
0
20
40
60
80
Length

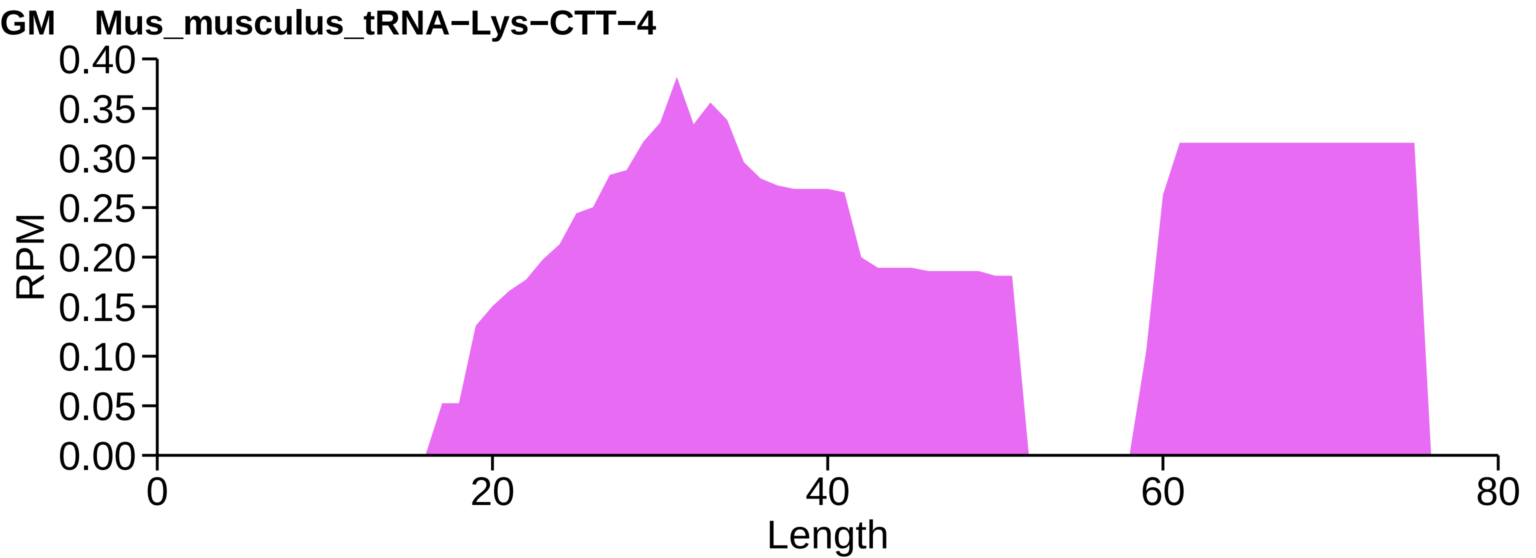

GM Mus_musculus_tRNA−Lys−CTT−4
RPM
0.00
0.05
0.10
0.15
0.20
0.25
0.30
0.35
0.40
0
20
40
60
80
Length

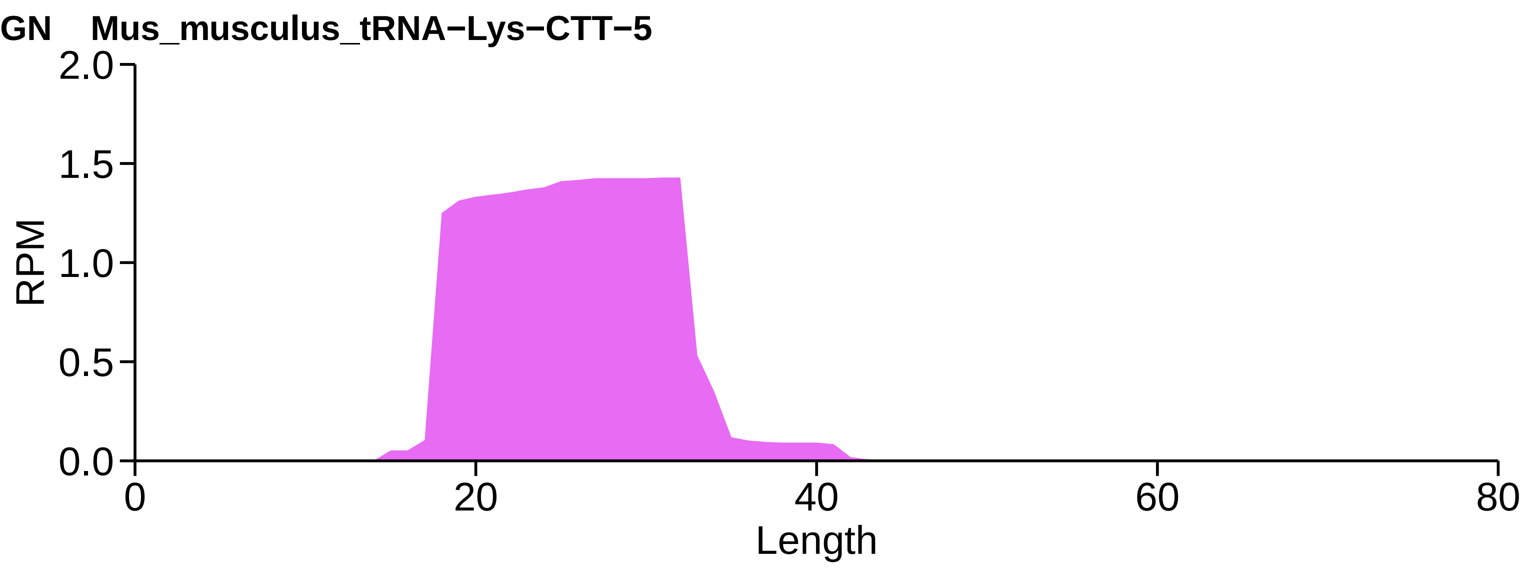

GN Mus_musculus_tRNA−Lys−CTT−5
2.0
1.5
1.0
0.5
0.0
RPM
0
20
40
60
80
Length

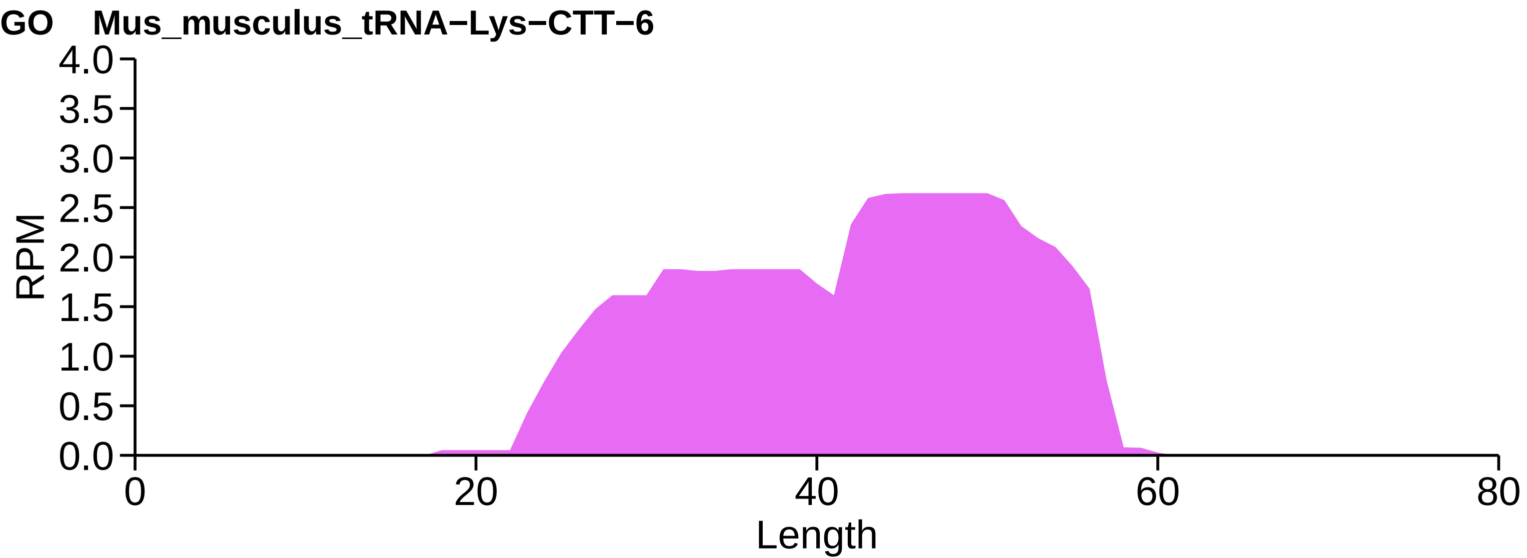
GO Mus_musculus_tRNA−Lys−CTT−6
RPM
4.0
3.5
3.0
2.5
2.0
1.5
1.0
0.5
0.0
0
20
40
60
80
Length

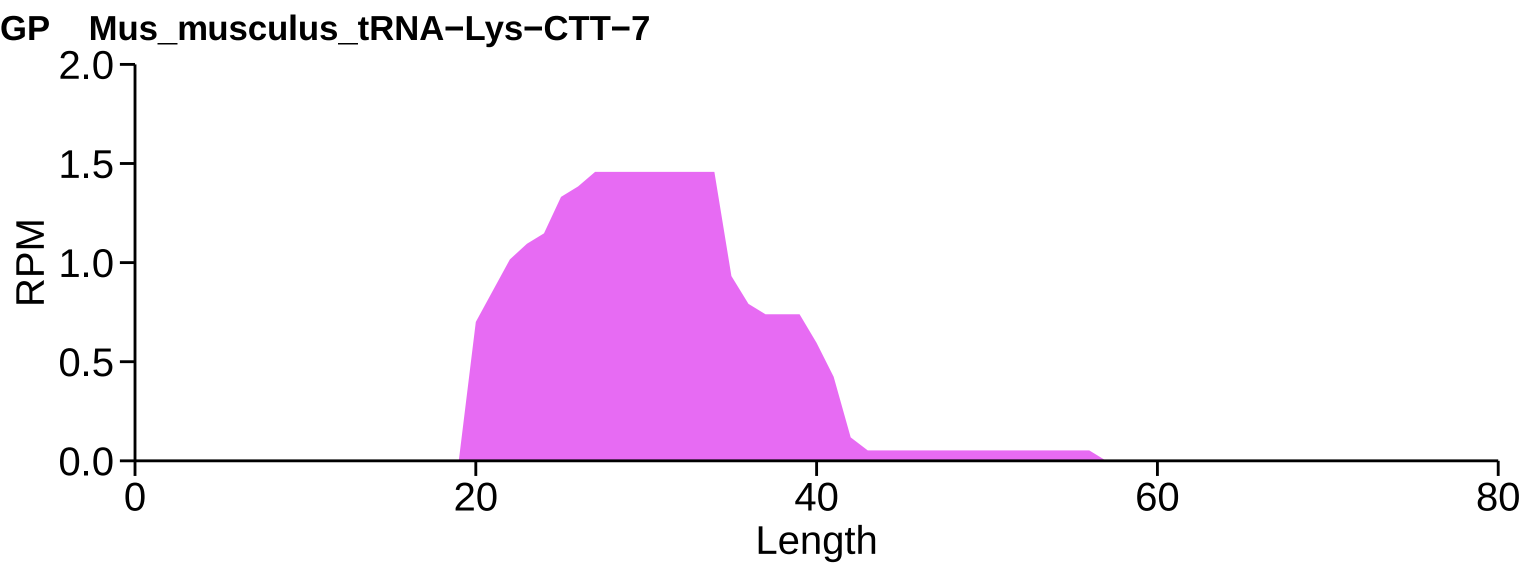
GP Mus_musculus_tRNA−Lys−CTT−7
RPM
Length
2.0
1.5
1.0
0.5
0.0
0
20
40
60
80

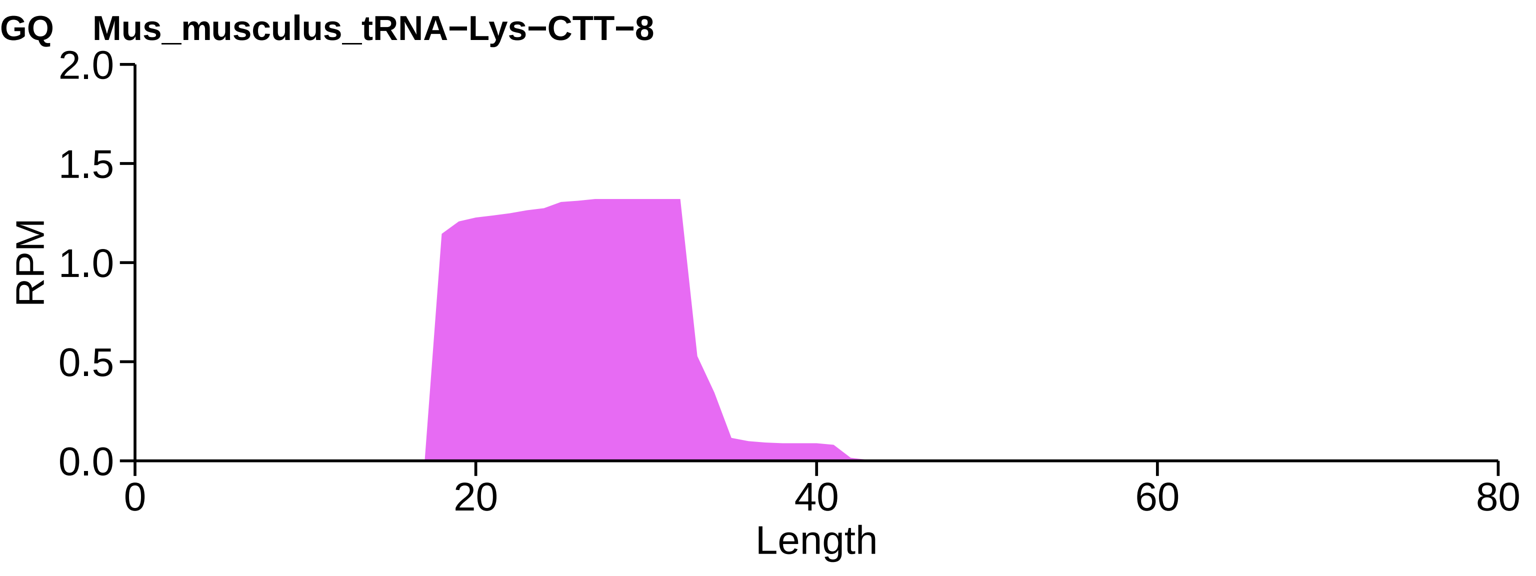
GQ Mus_musculus_tRNA−Lys−CTT−8
RPM
2.0
1.5
1.0
0.5
0.0
0
20
40
60
80
Length

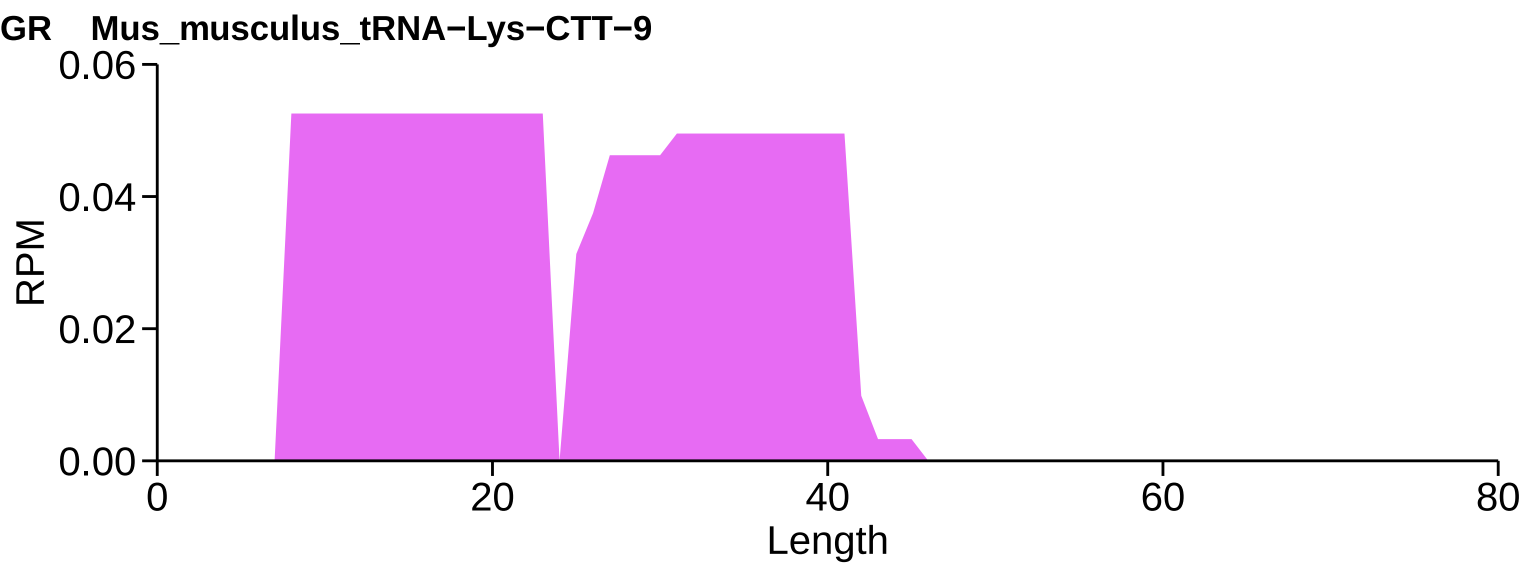
GR Mus_musculus_tRNA−Lys−CTT−9
0.06
0.04
0.02
0.00
RPM
0
20
40
60
80
Length

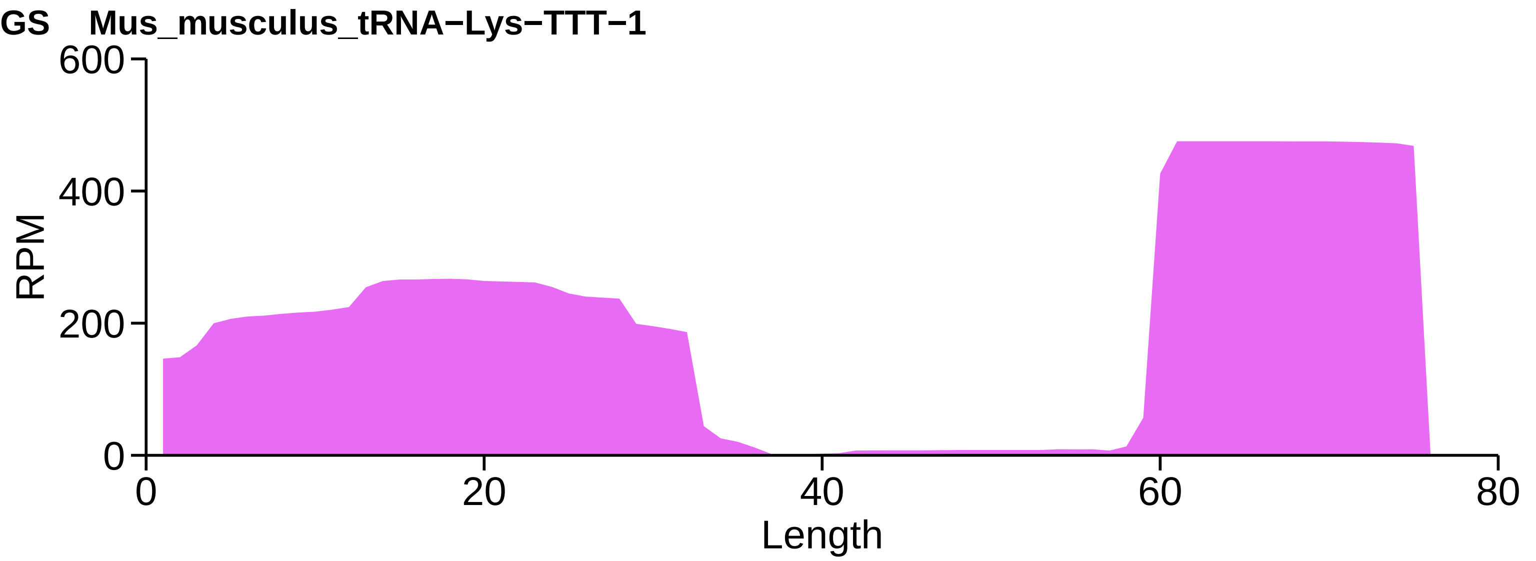
GS Mus_musculus_tRNA−Lys−TTT−1
600
400
200
0
RPM
0
20
40
60
80
Length

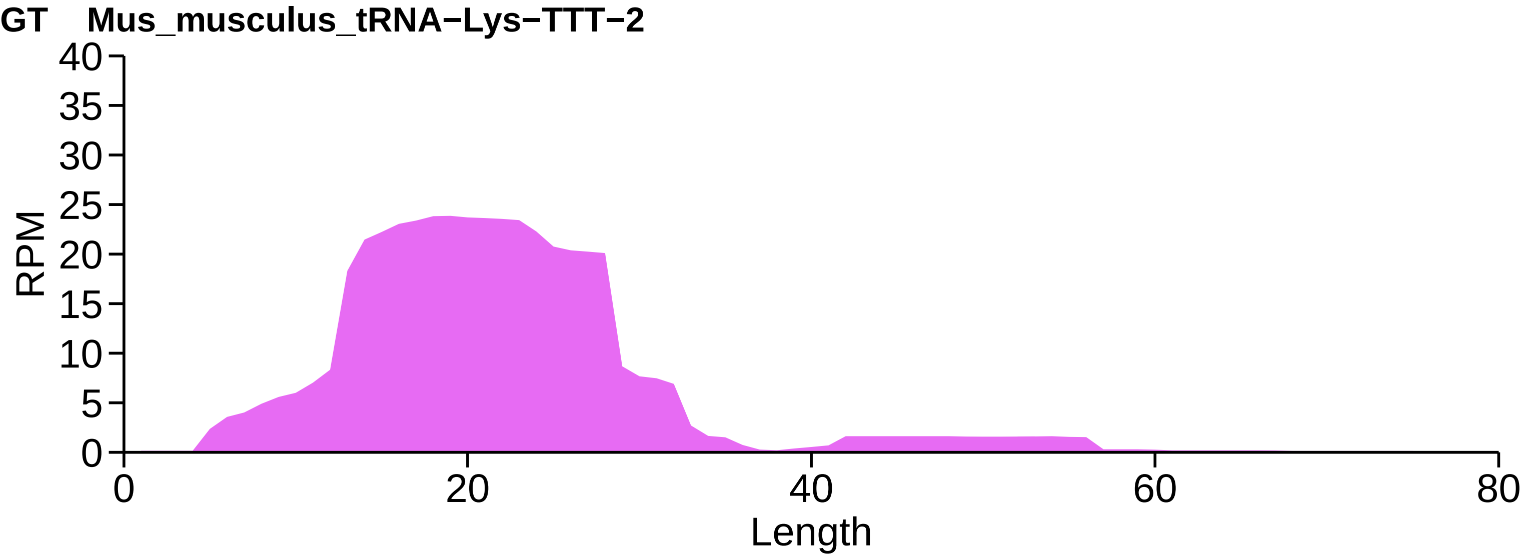

GT Mus_musculus_tRNA−Lys−TTT−2
RPM
40
35
30
25
20
15
10
5
0
0
20
40
60
80
Length

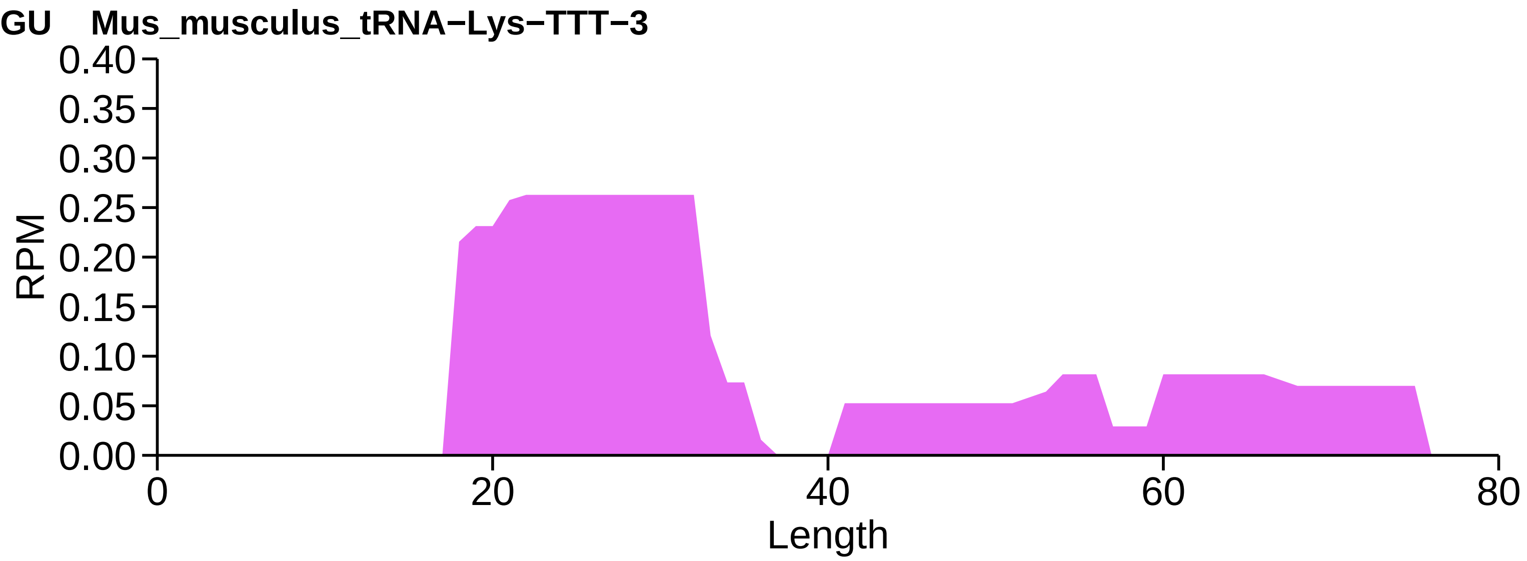

GU Mus_musculus_tRNA−Lys−TTT−3
RPM
0.40
0.35
0.30
0.25
0.20
0.15
0.10
0.05
0.00
0
20
40
60
80
Length

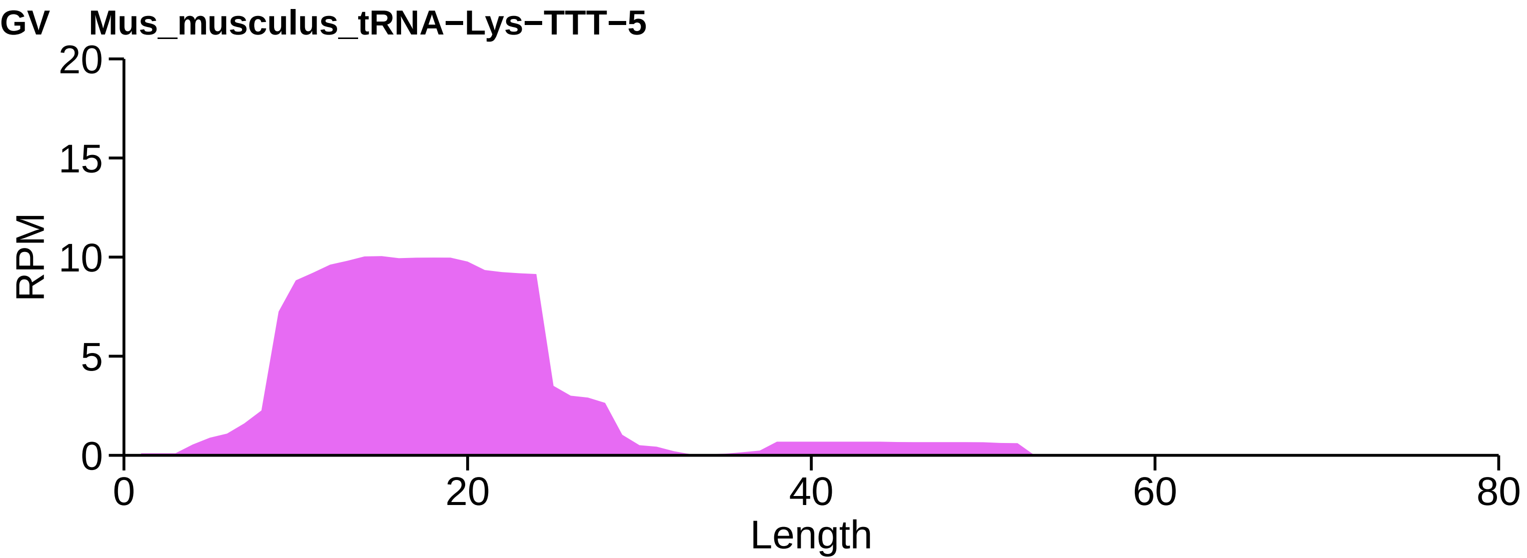
GV Mus_musculus_tRNA−Lys−TTT−5
RPM
Length
0
5
10
15
20
0
20
40
60
80

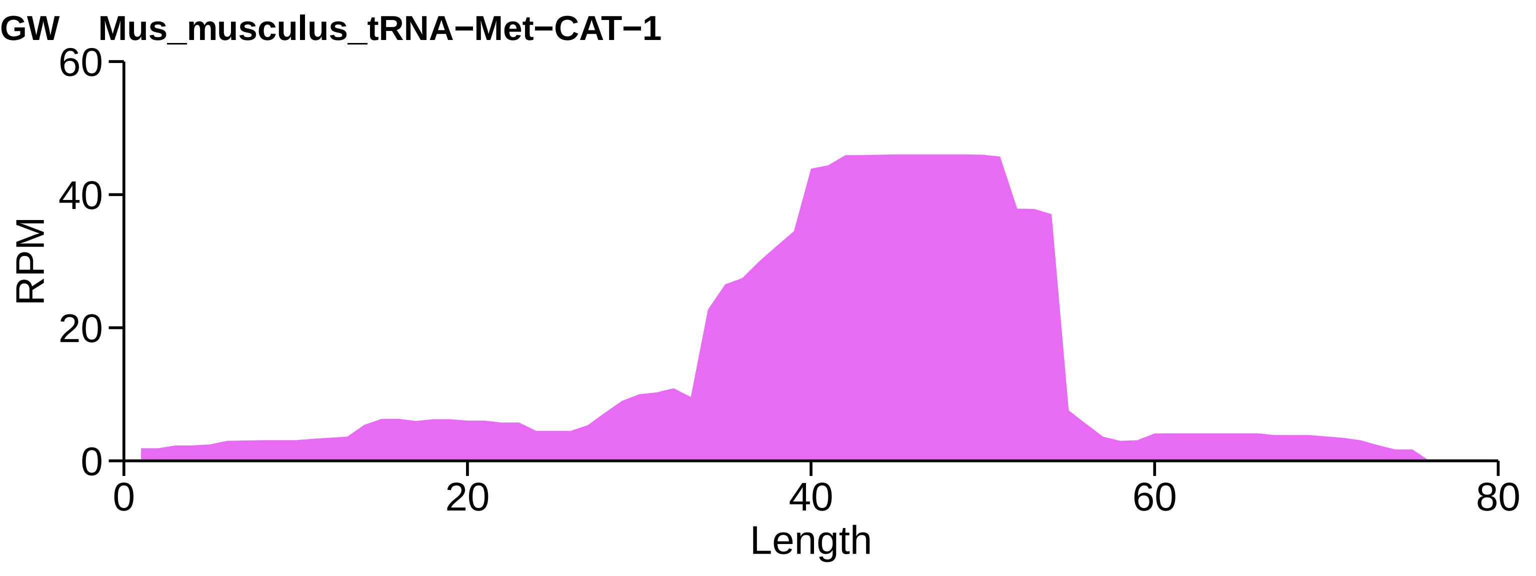
GW Mus_musculus_tRNA−Met−CAT−1
RPM
0
20
40
60
Length
0
20
40
60
80

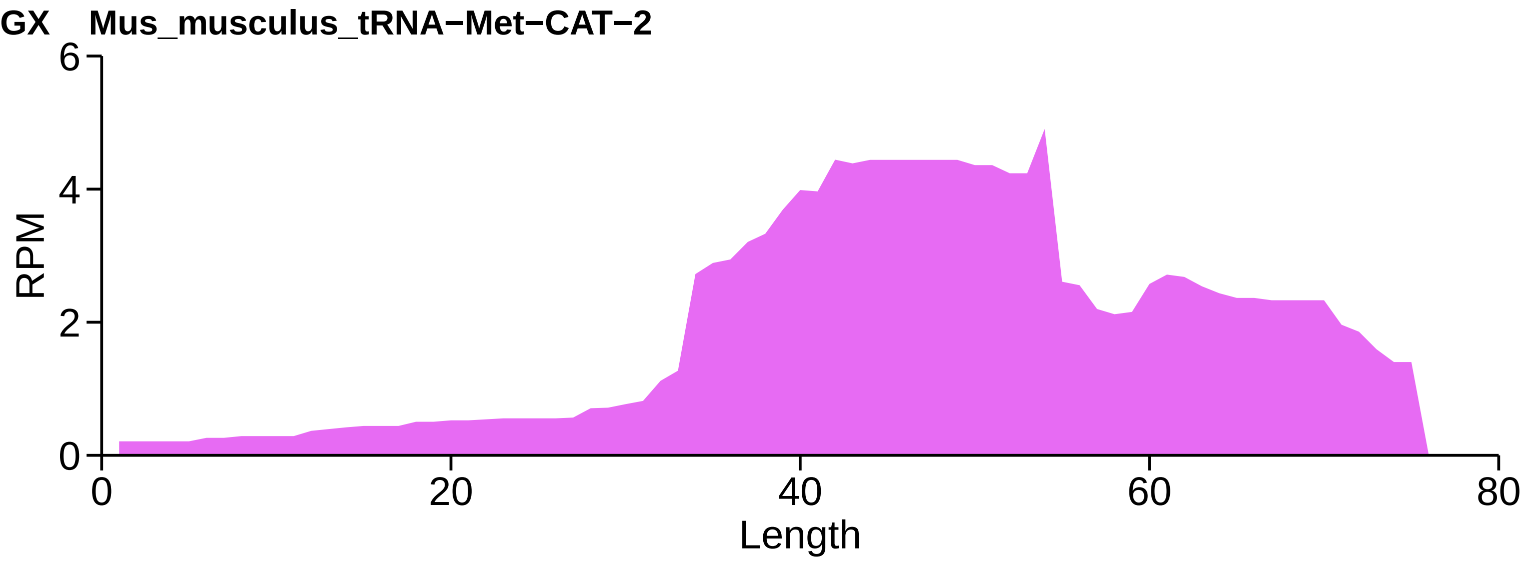
GX Mus_musculus_tRNA−Met−CAT−2
RPM
0
2
4
6
0
20
40
60
80
Length

GY Mus_musculus_tRNA−Met−CAT−3

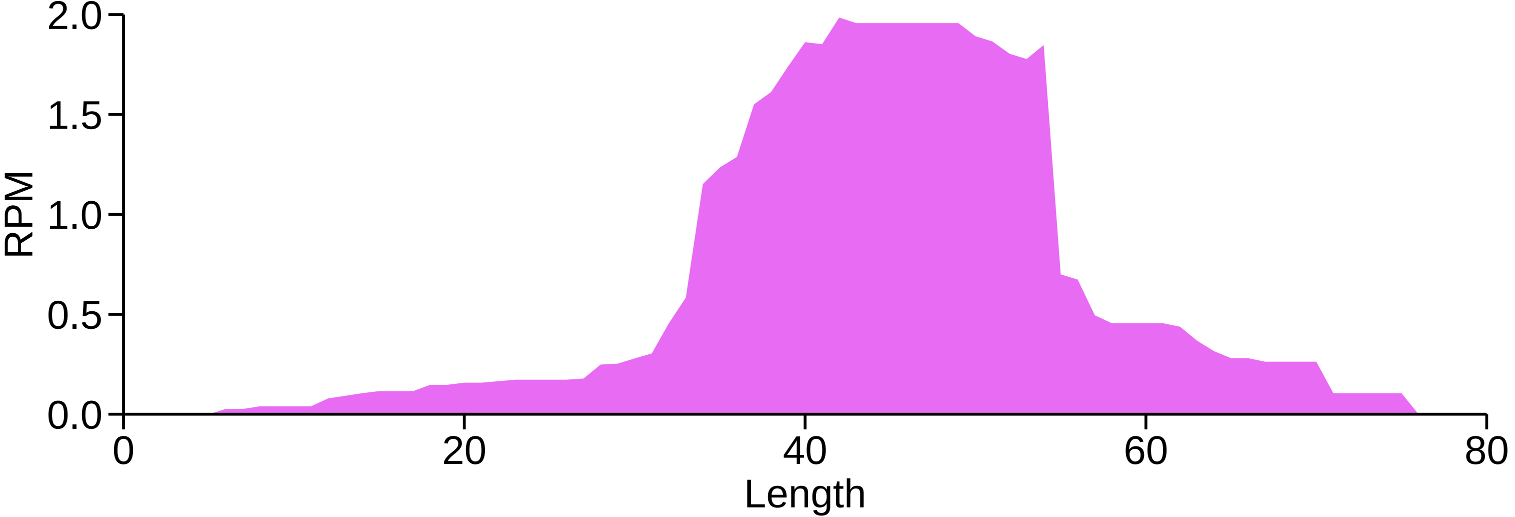

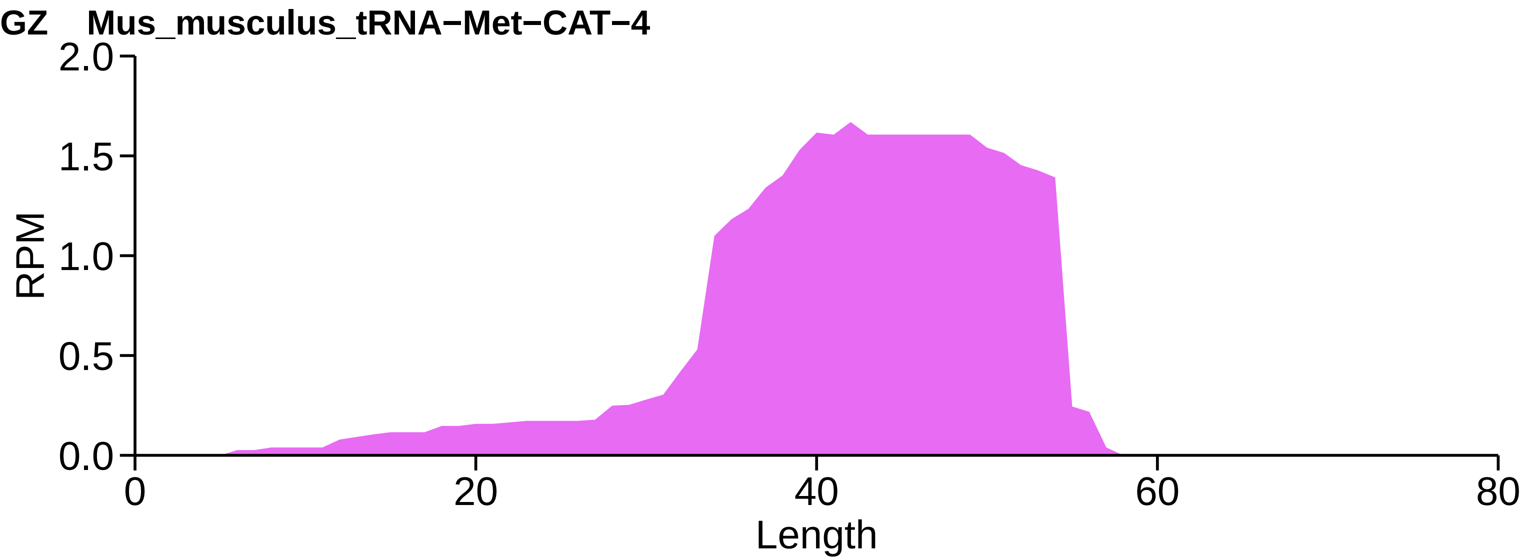
GZ Mus_musculus_tRNA−Met−CAT−4
RPM
2.0
1.5
1.0
0.5
0.0
0
20
40
60
80
Length

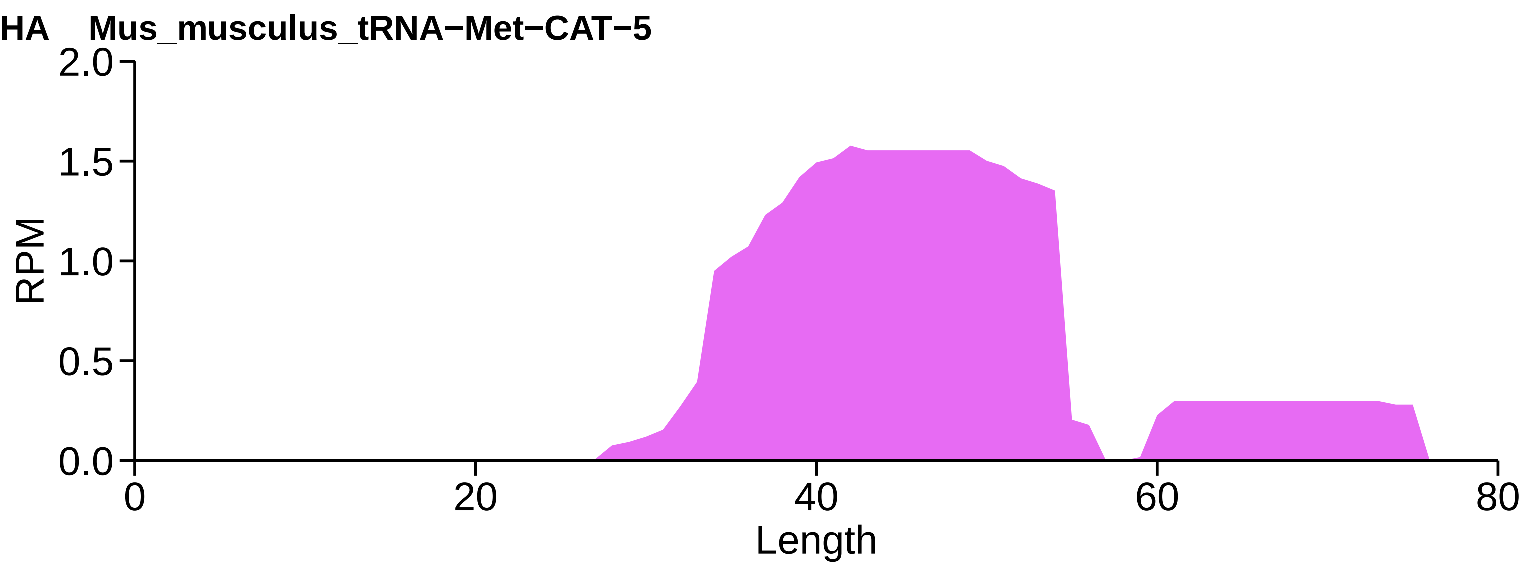

HA Mus_musculus_tRNA−Met−CAT−5
RPM
2.0
1.5
1.0
0.5
0.0
0
20
40
60
80
Length

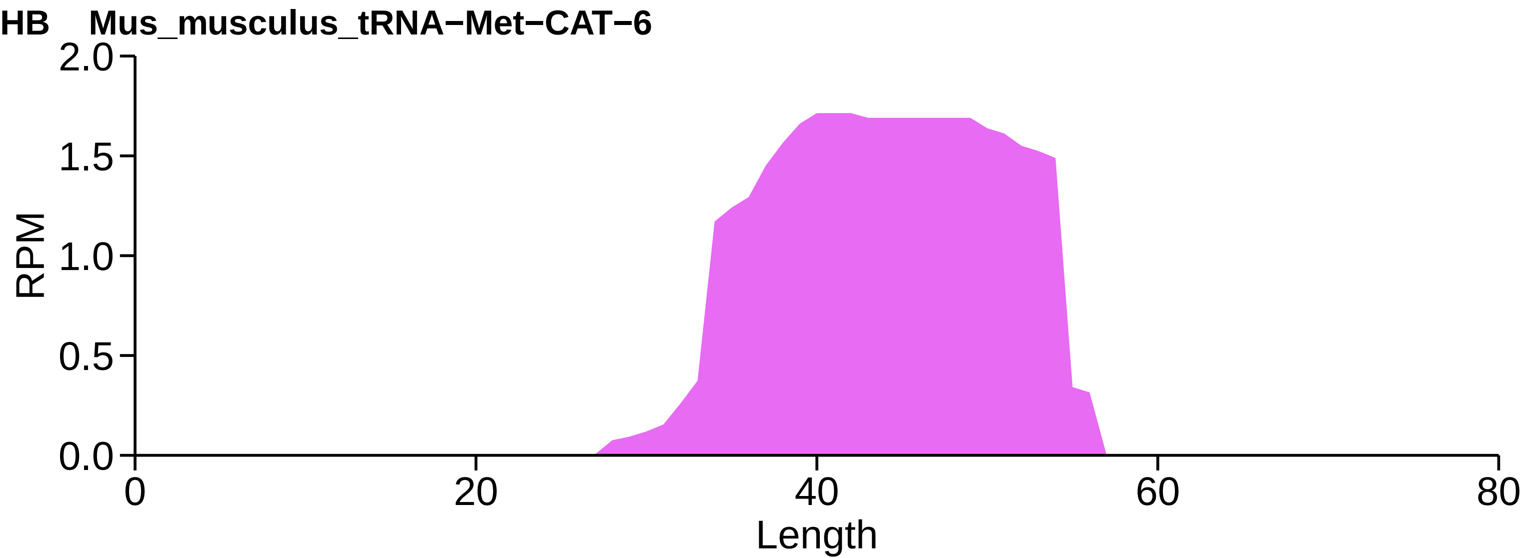

HB Mus_musculus_tRNA−Met−CAT−6
RPM
2.0
1.5
1.0
0.5
0.0
0
20
40
60
80
Length

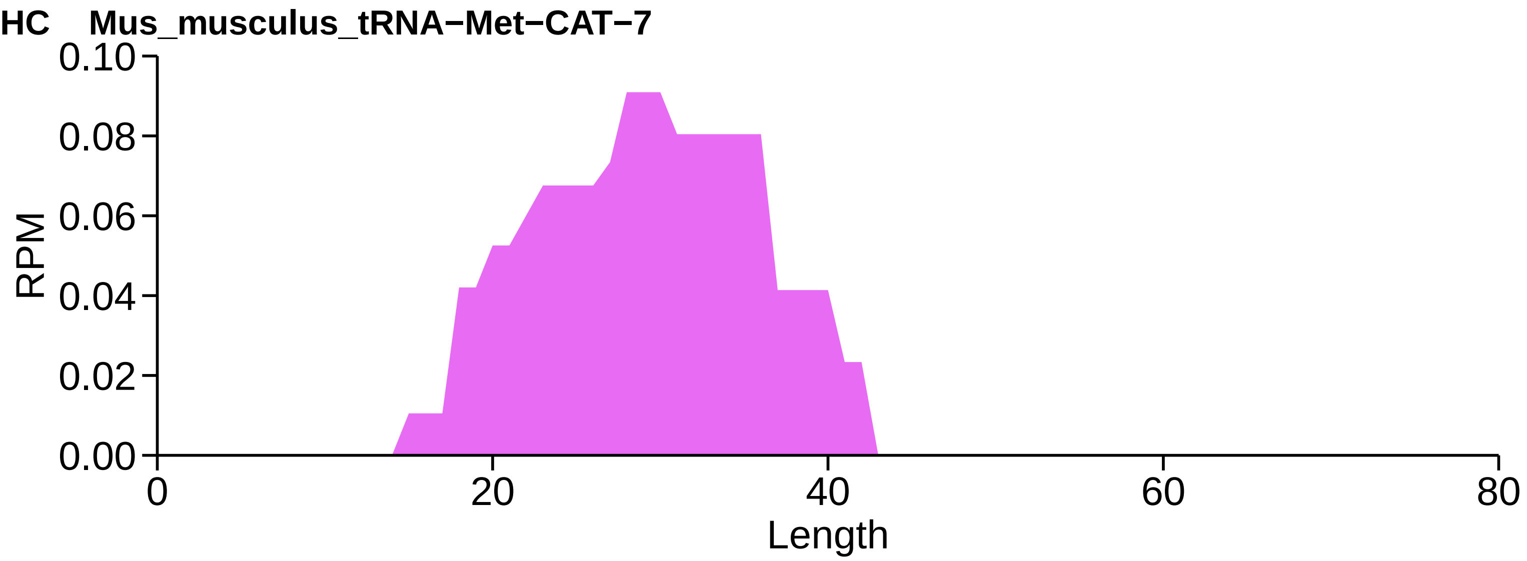
HC Mus_musculus_tRNA−Met−CAT−7
RPM
0.10
0.08
0.06
0.04
0.02
0.00
0
20
40
60
80
Length

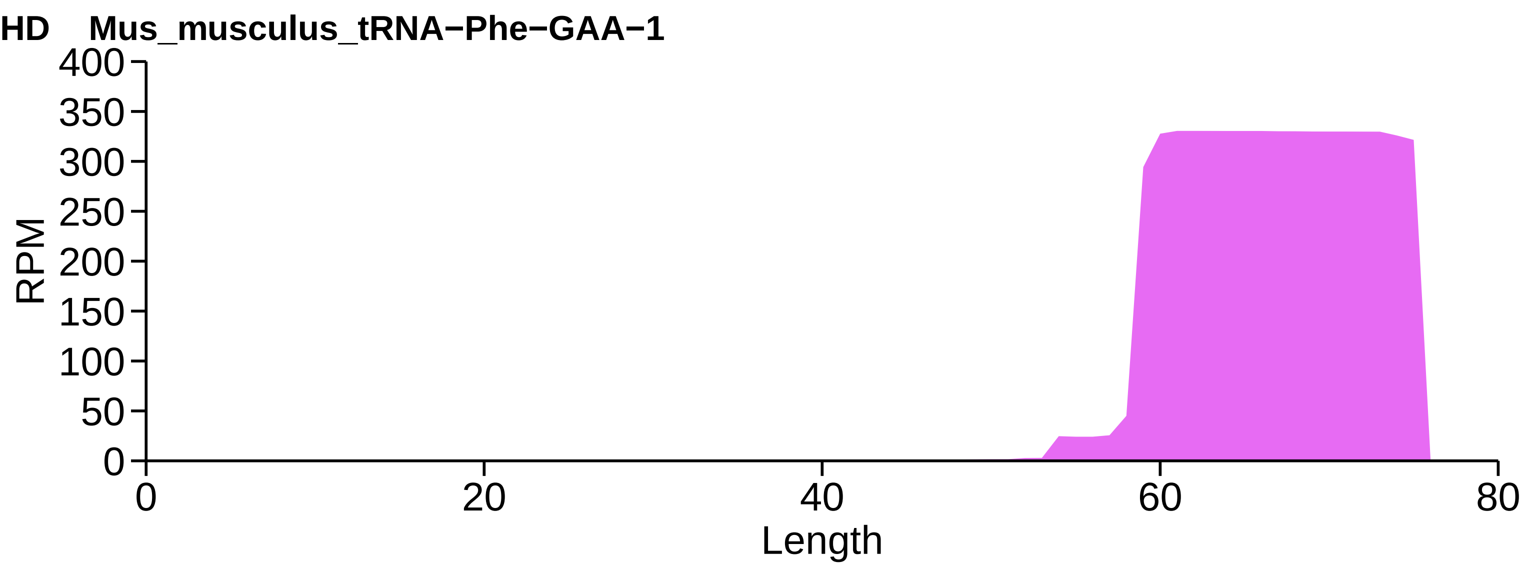
HD Mus_musculus_tRNA−Phe−GAA−1
RPM
400
350
300
250
200
150
100
50
0
0
20
40
60
80
Length

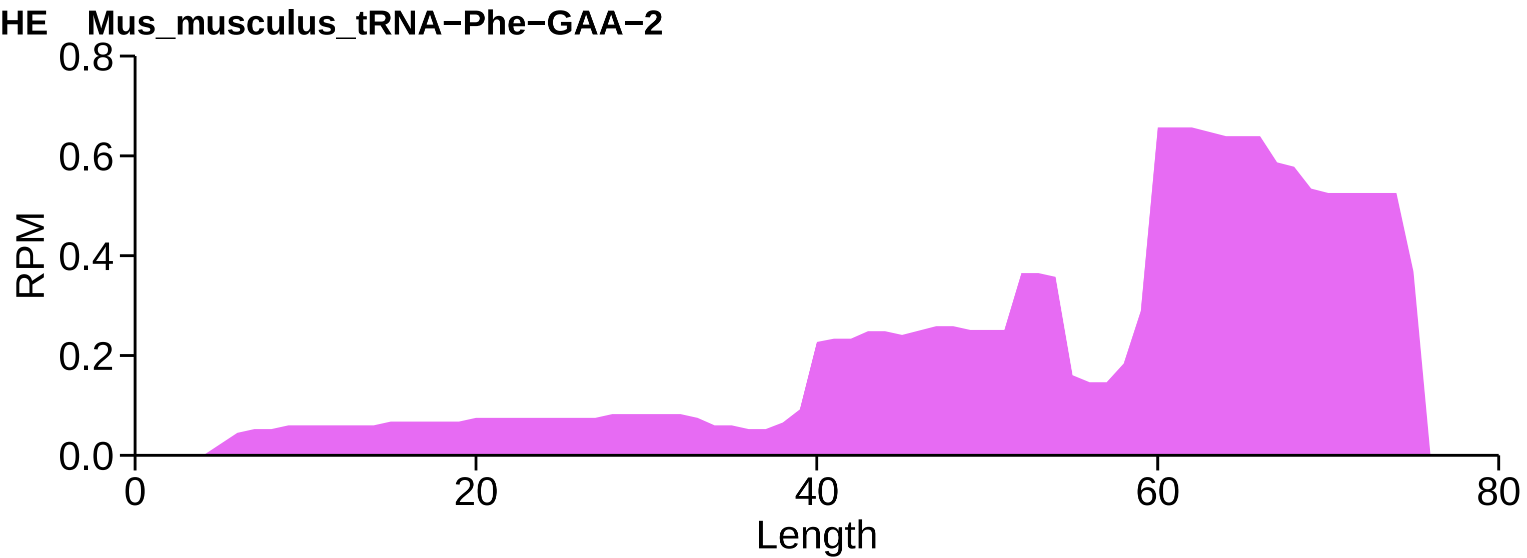

HE Mus_musculus_tRNA−Phe−GAA−2
0.8
0.6
0.4
0.2
0.0
RPM
0
20
40
60
80
Length

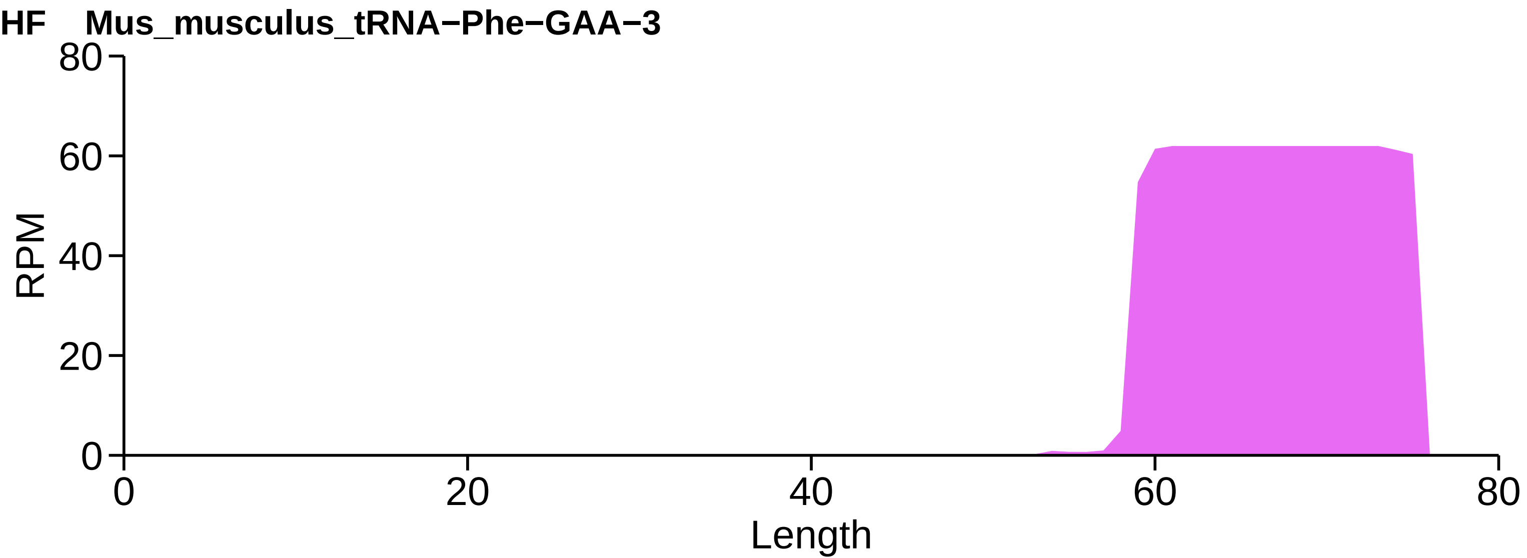
HF Mus_musculus_tRNA−Phe−GAA−3
RPM
0
20
40
60
80
Length
0
20
40
60
80

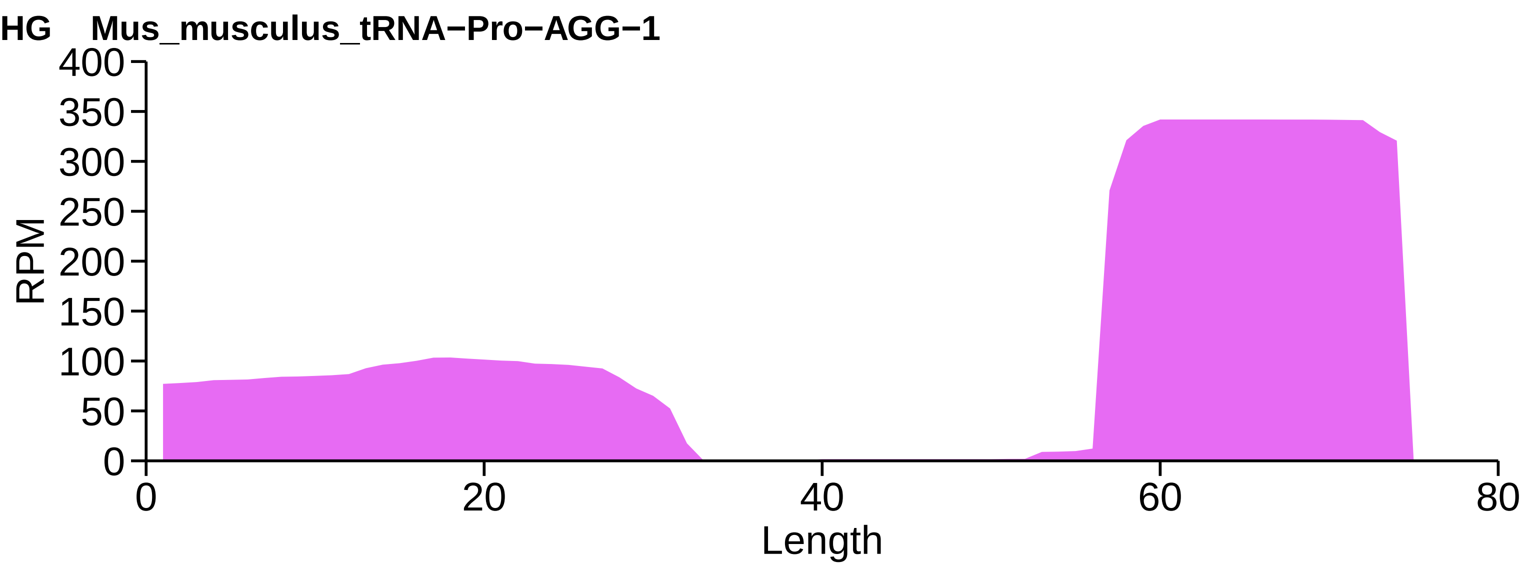

HG Mus_musculus_tRNA−Pro−AGG−1
RPM
400
350
300
250
200
150
100
50
0
0
20
40
60
80
Length

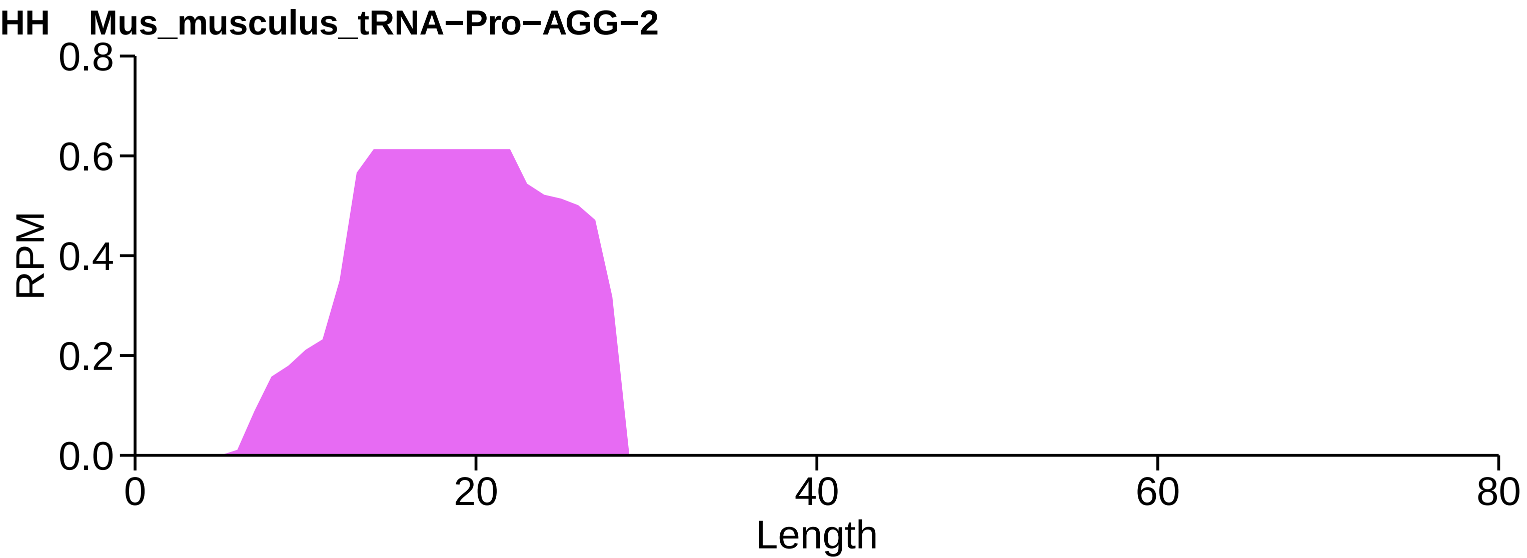
HH Mus_musculus_tRNA−Pro−AGG−2
RPM
0.0
0.2
0.4
0.6
0.8
0
20
40
60
80
Length

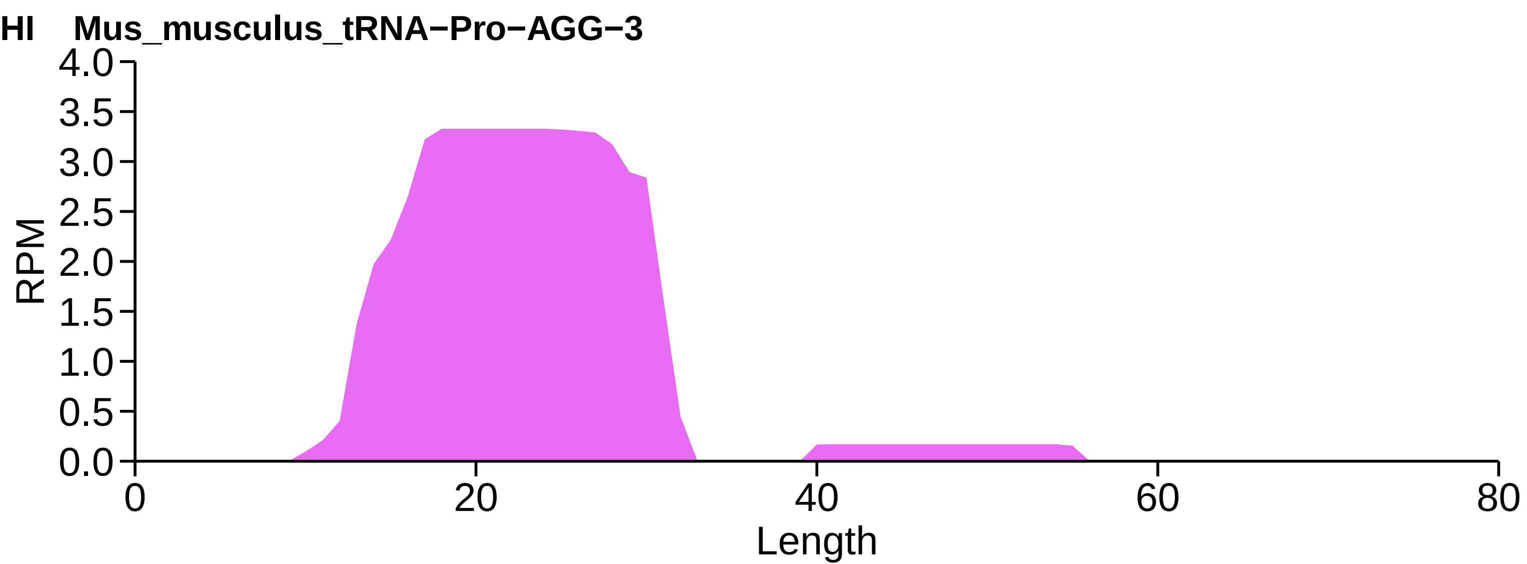

HI Mus_musculus_tRNA−Pro−AGG−3
RPM
4.0
3.5
3.0
2.5
2.0
1.5
1.0
0.5
0.0
0
20
40
60
80
Length

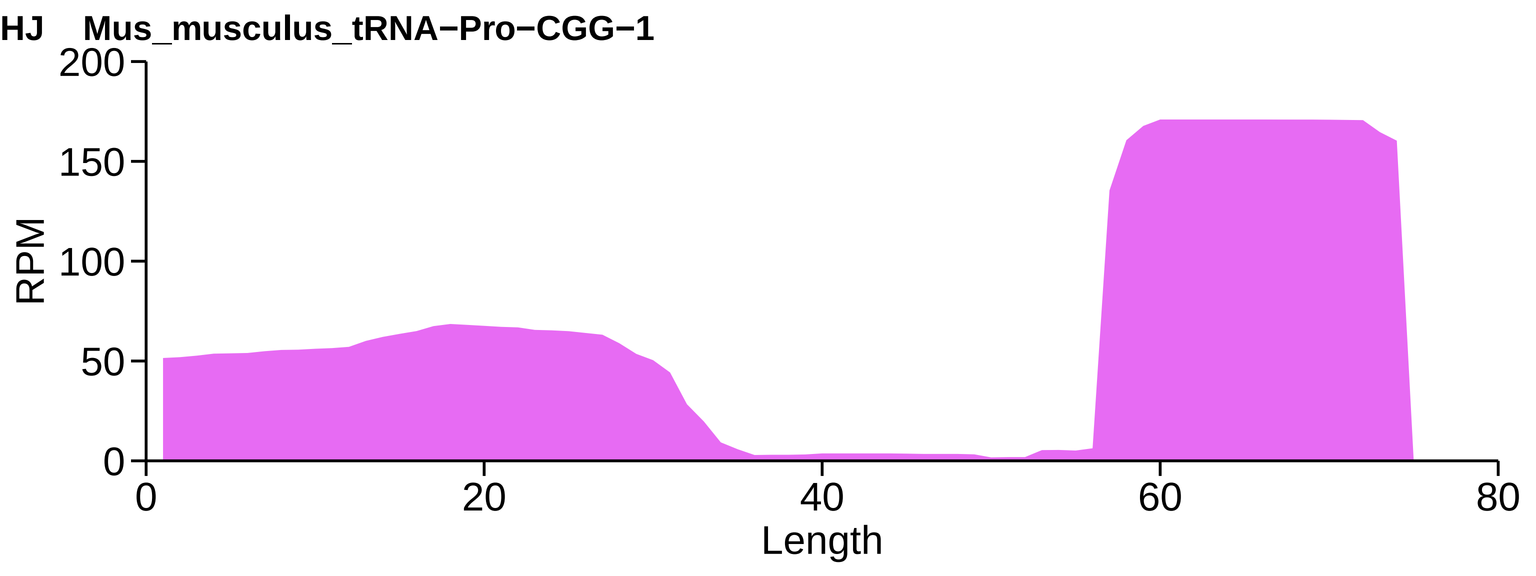
HJ Mus_musculus_tRNA−Pro−CGG−1
RPM
200
150
100
50
0
0
20
40
60
80
Length

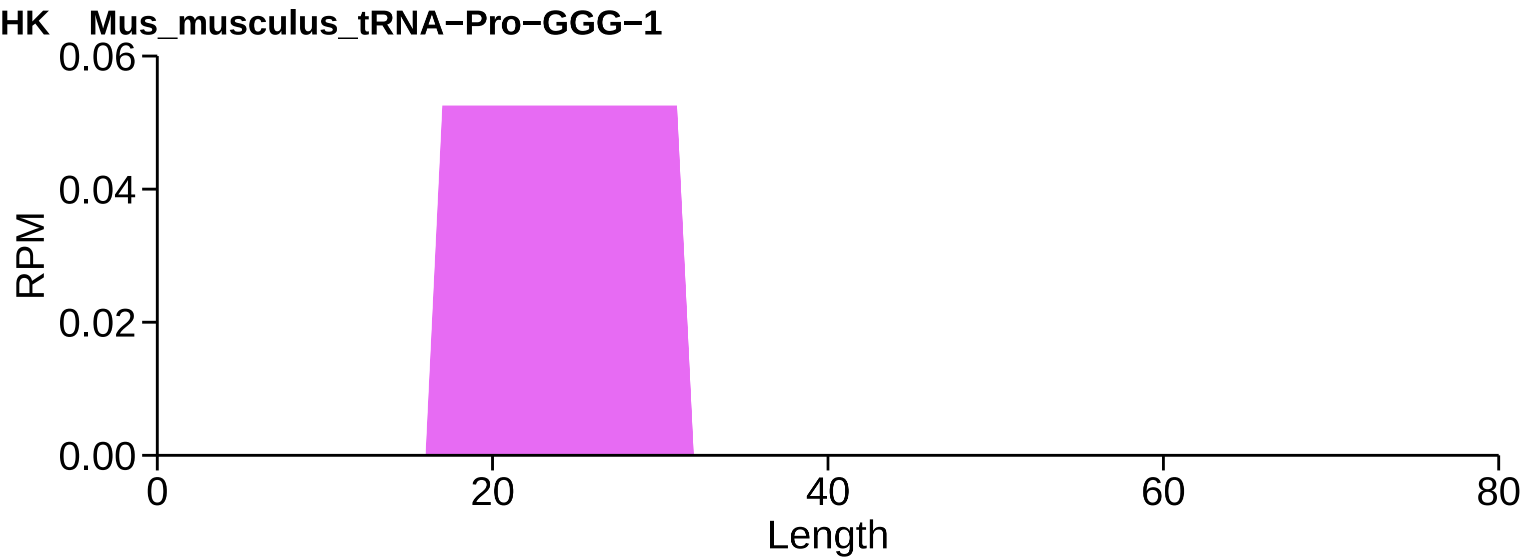
HK Mus_musculus_tRNA−Pro−GGG−1
0.06
0.04
0.02
0.00
RPM
0
20
40
60
80
Length

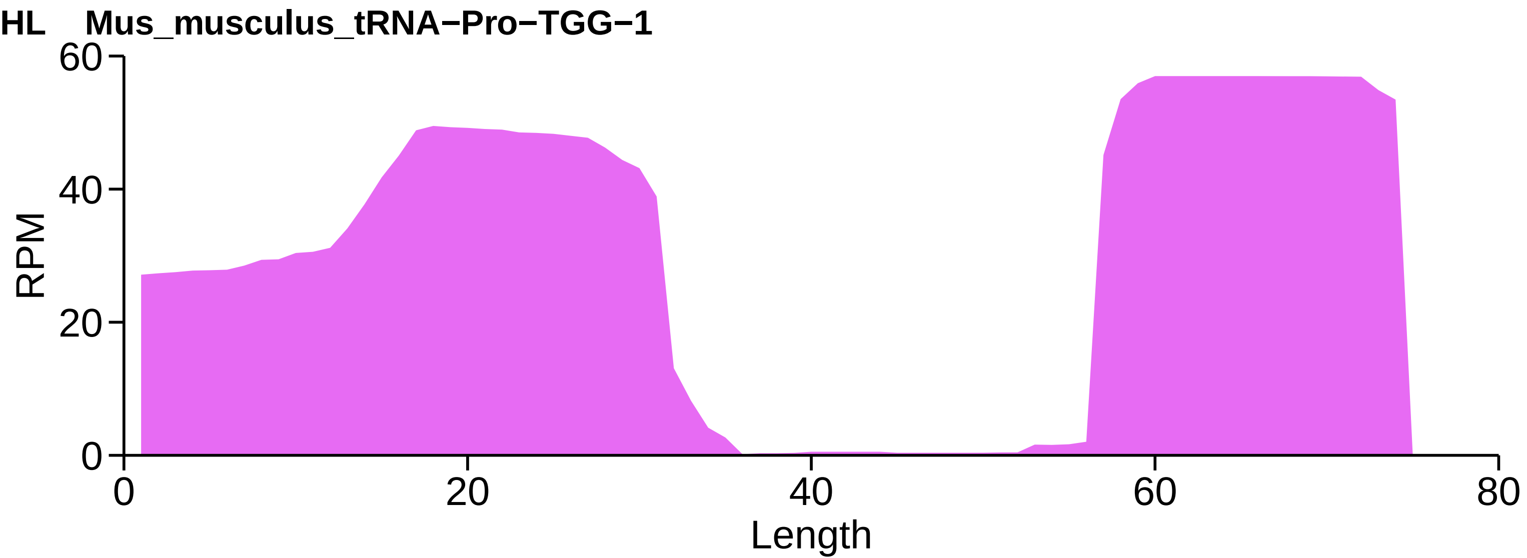
HL Mus_musculus_tRNA−Pro−TGG−1
60
40
20
0
RPM
0
20
40
60
80
Length

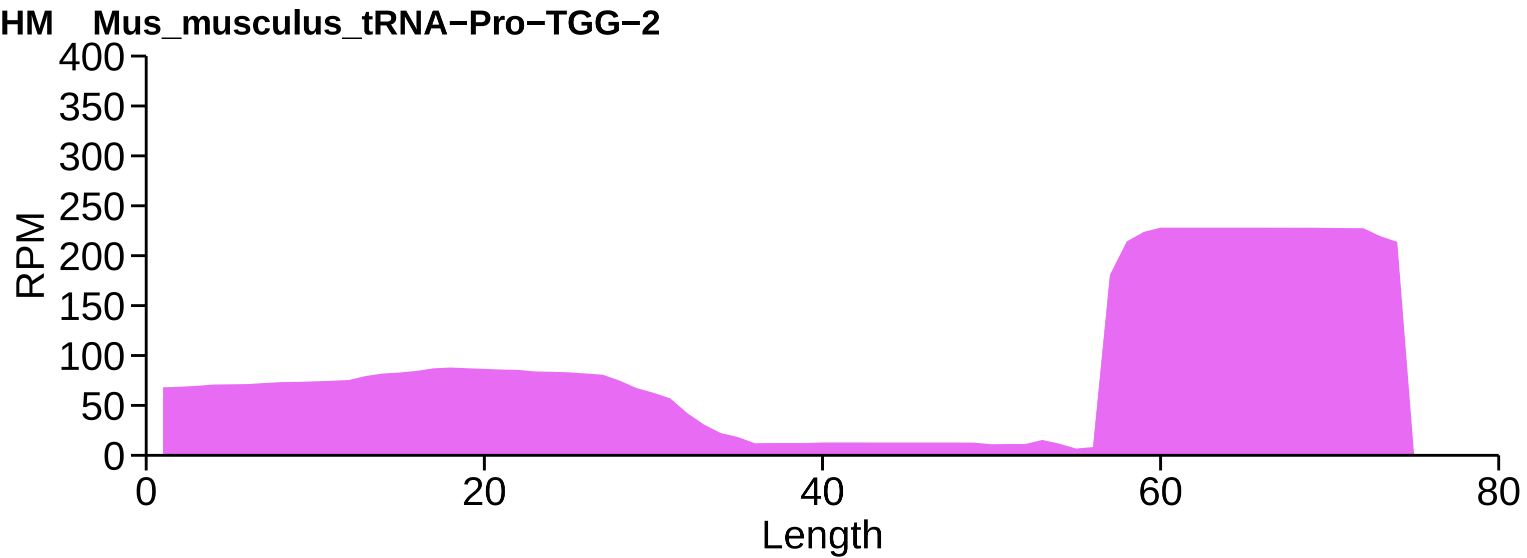

HM Mus_musculus_tRNA−Pro−TGG−2
RPM
400
350
300
250
200
150
100
50
0
0
20
40
60
80
Length

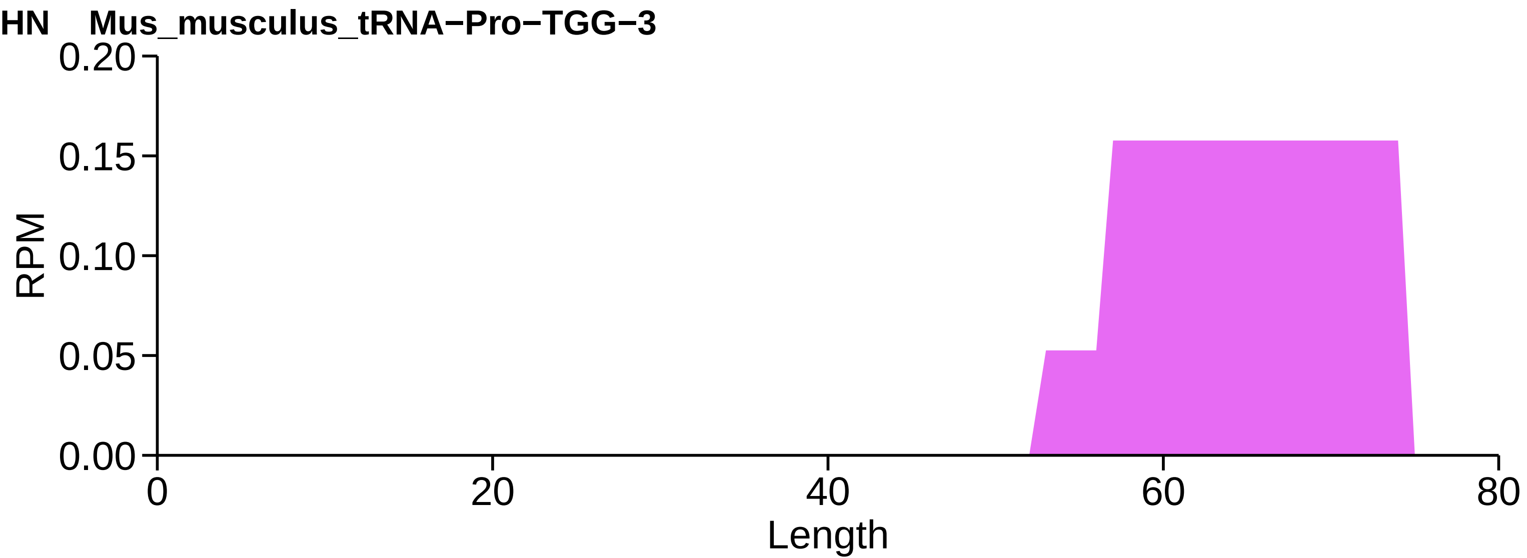
HN Mus_musculus_tRNA−Pro−TGG−3
RPM
0.20
0.15
0.10
0.05
0.00
0
20
40
60
80
Length

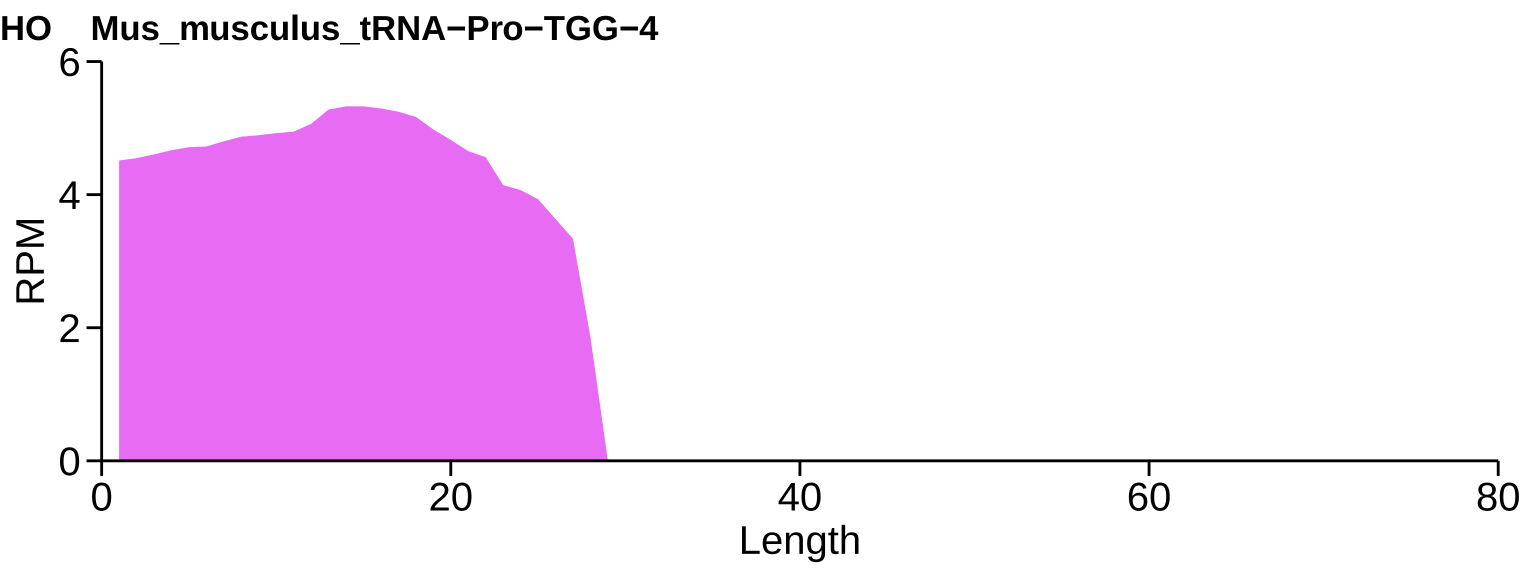
HO Mus_musculus_tRNA−Pro−TGG−4
RPM
0
2
4
6
0
20
40
60
80
Length

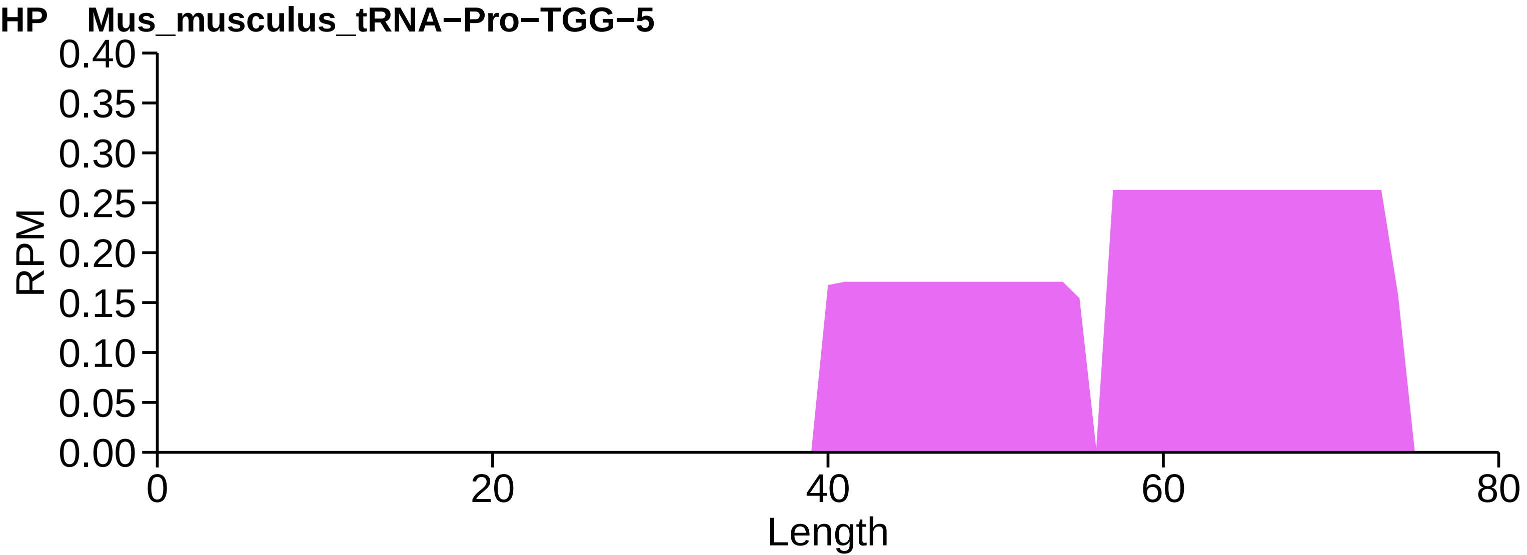
HP Mus_musculus_tRNA−Pro−TGG−5
RPM
0.40
0.35
0.30
0.25
0.20
0.15
0.10
0.05
0.00
0
20
40
60
80
Length

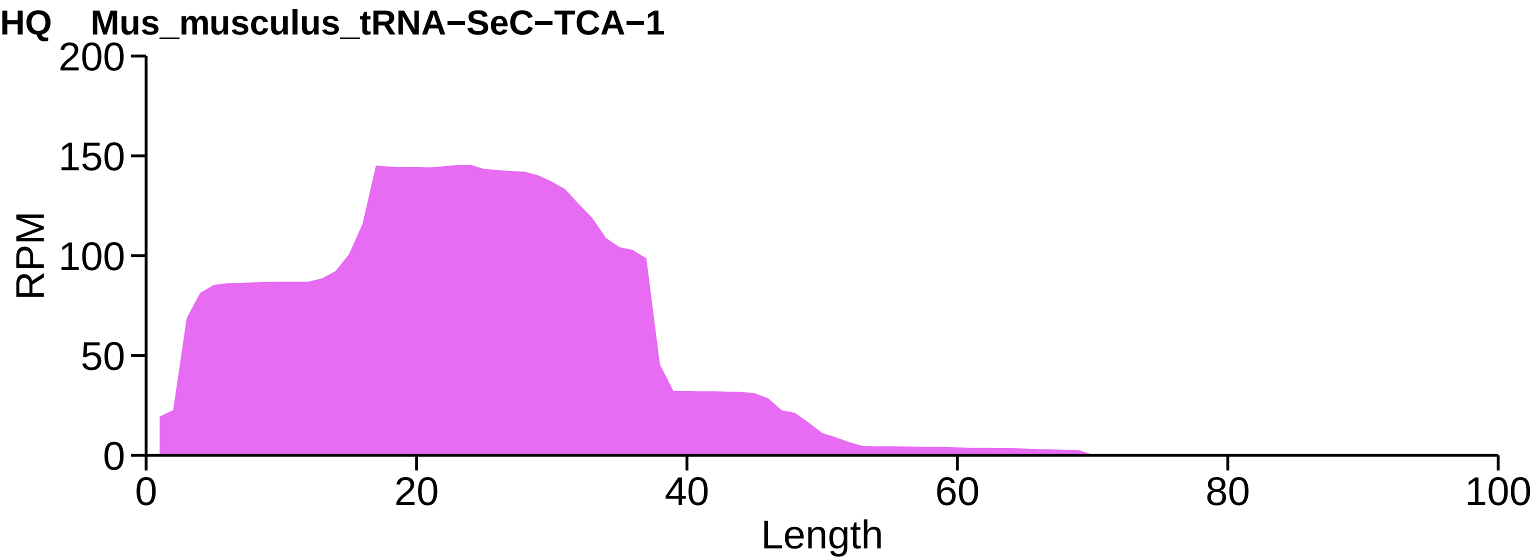

HQ Mus_musculus_tRNA−SeC−TCA−1
RPM
200
150
100
50
0
0
20
40
60
80
100
Length

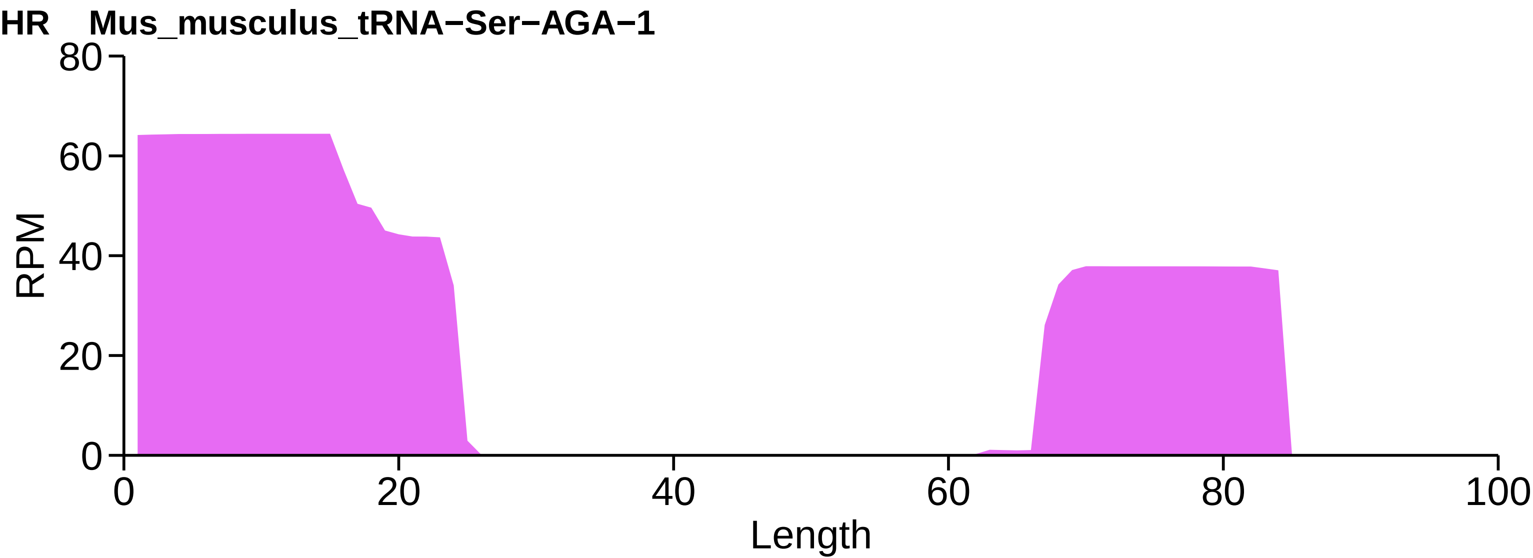
HR Mus_musculus_tRNA−Ser−AGA−1
RPM
0
20
40
60
80
0
20
40
60
80
100
Length

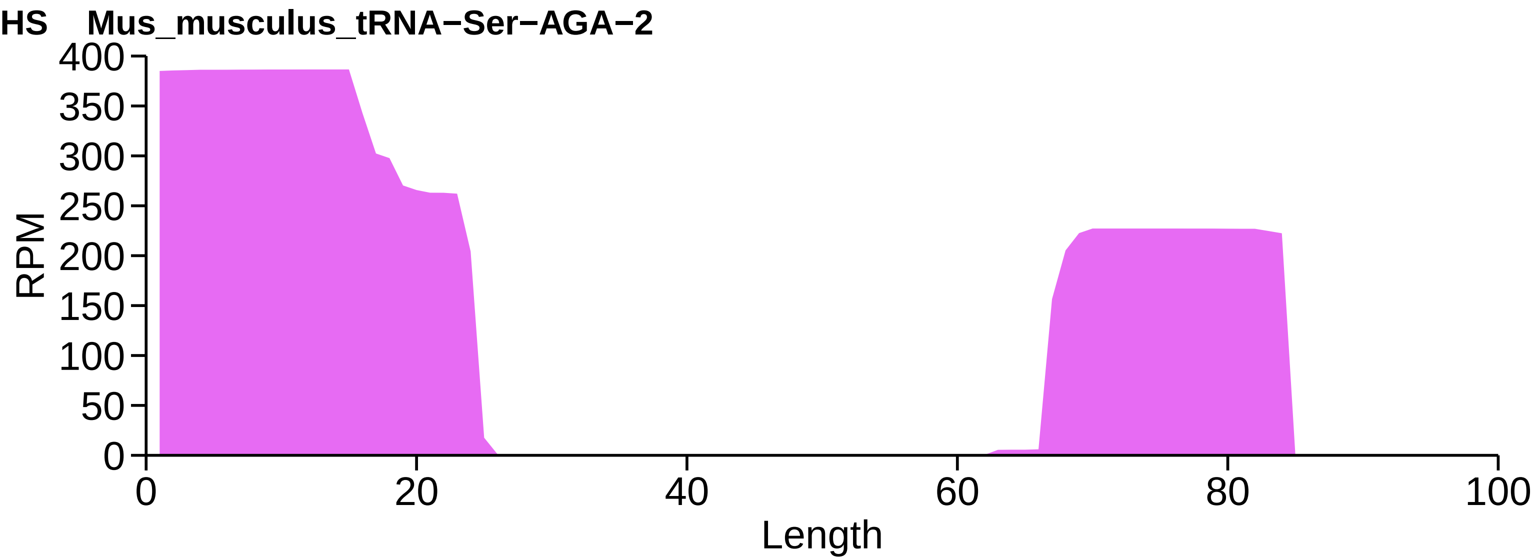
HS Mus_musculus_tRNA−Ser−AGA−2
RPM
400
350
300
250
200
150
100
50
0
0
20
40
60
80
100
Length

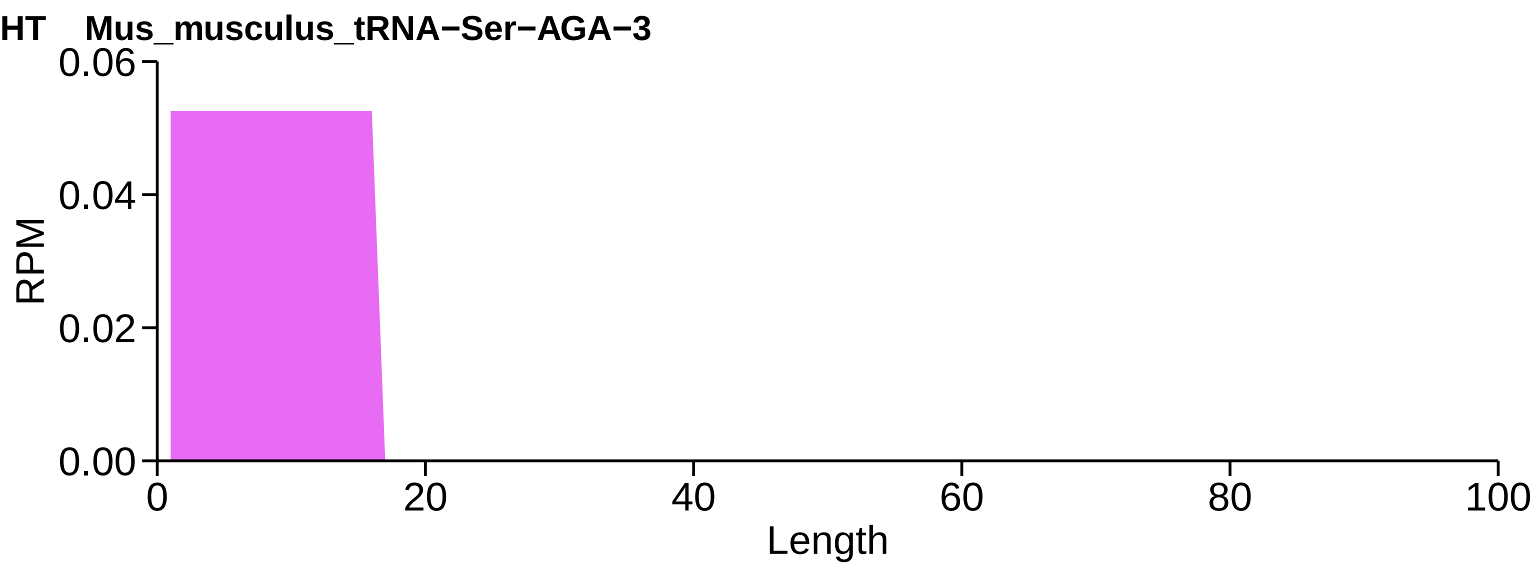

HT Mus_musculus_tRNA−Ser−AGA−3
0.06
0.04
0.02
0.00
RPM
0
20
40
60
80
100
Length

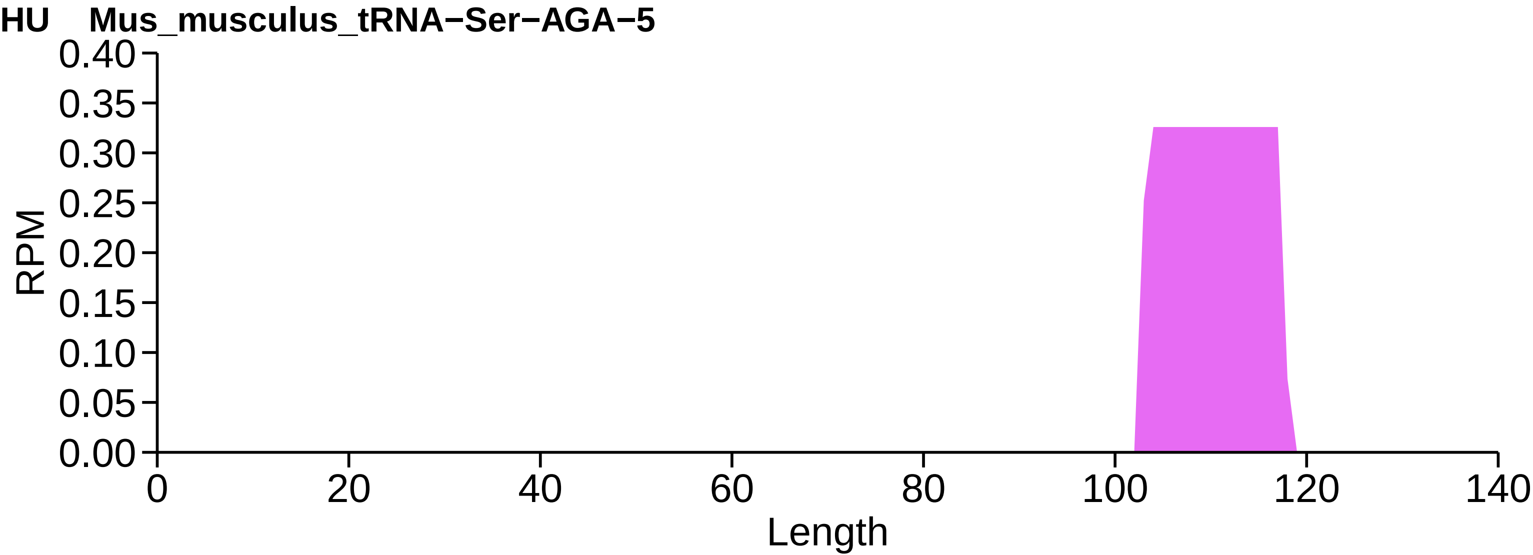

HU Mus_musculus_tRNA−Ser−AGA−5
RPM
0.40
0.35
0.30
0.25
0.20
0.15
0.10
0.05
0.00
0
20
40
60
80
100
120
140
Length

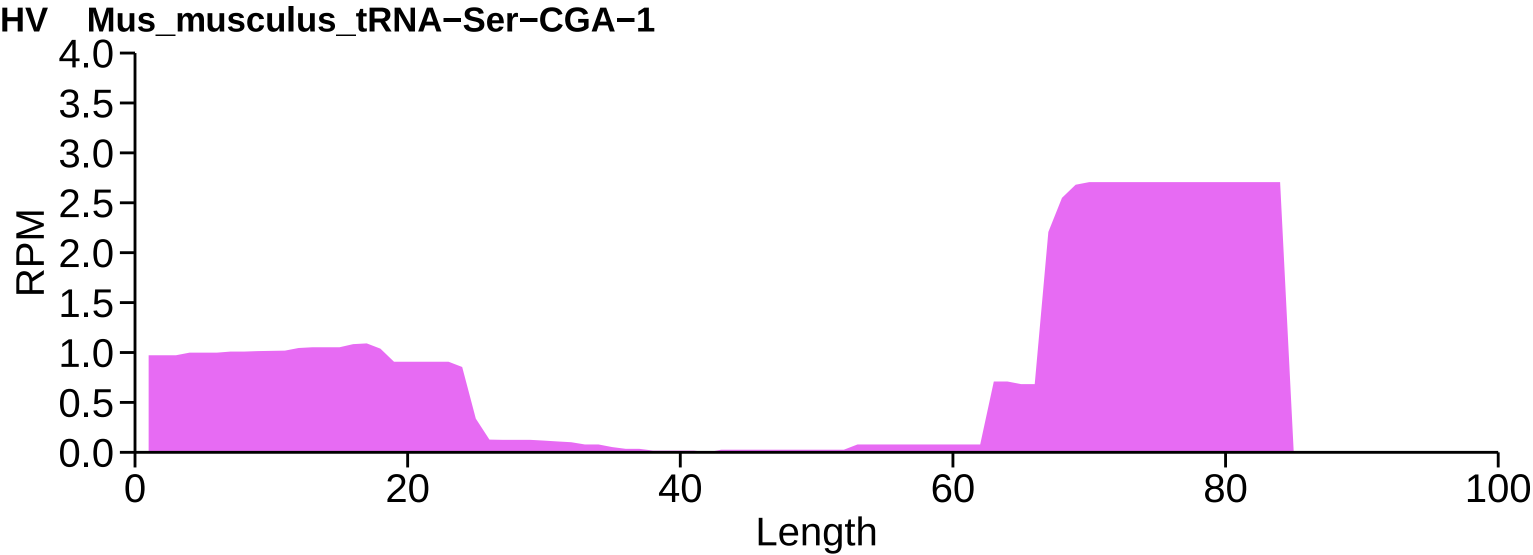
HV Mus_musculus_tRNA−Ser−CGA−1
RPM
4.0
3.5
3.0
2.5
2.0
1.5
1.0
0.5
0.0
0
20
40
60
80
100
Length

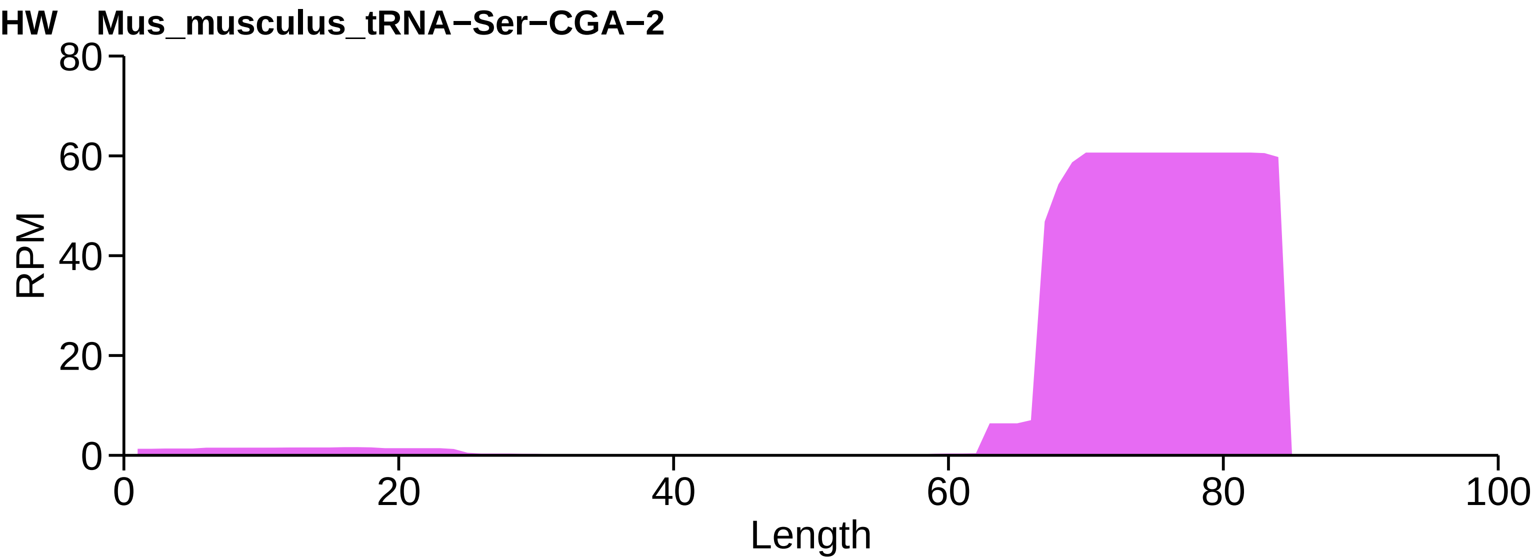
HW Mus_musculus_tRNA−Ser−CGA−2
RPM
Length
0
20
40
60
80
0
20
40
60
80
100

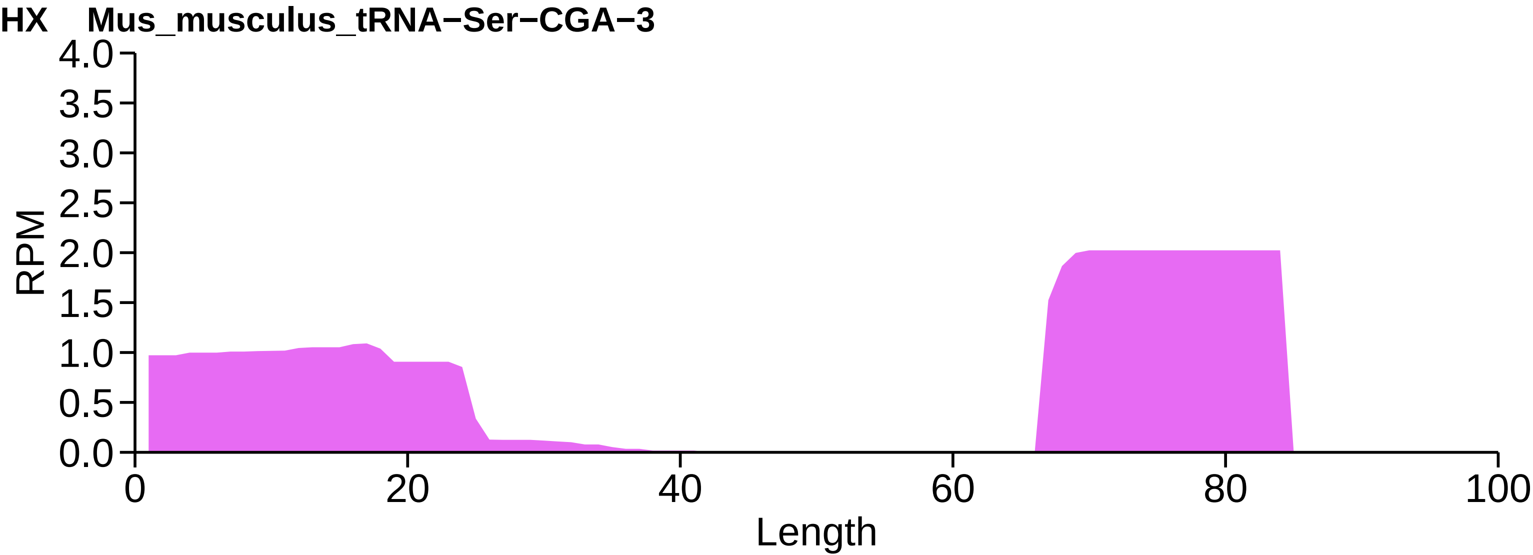

HX Mus_musculus_tRNA−Ser−CGA−3
RPM
4.0
3.5
3.0
2.5
2.0
1.5
1.0
0.5
0.0
0
20
40
60
80
100
Length

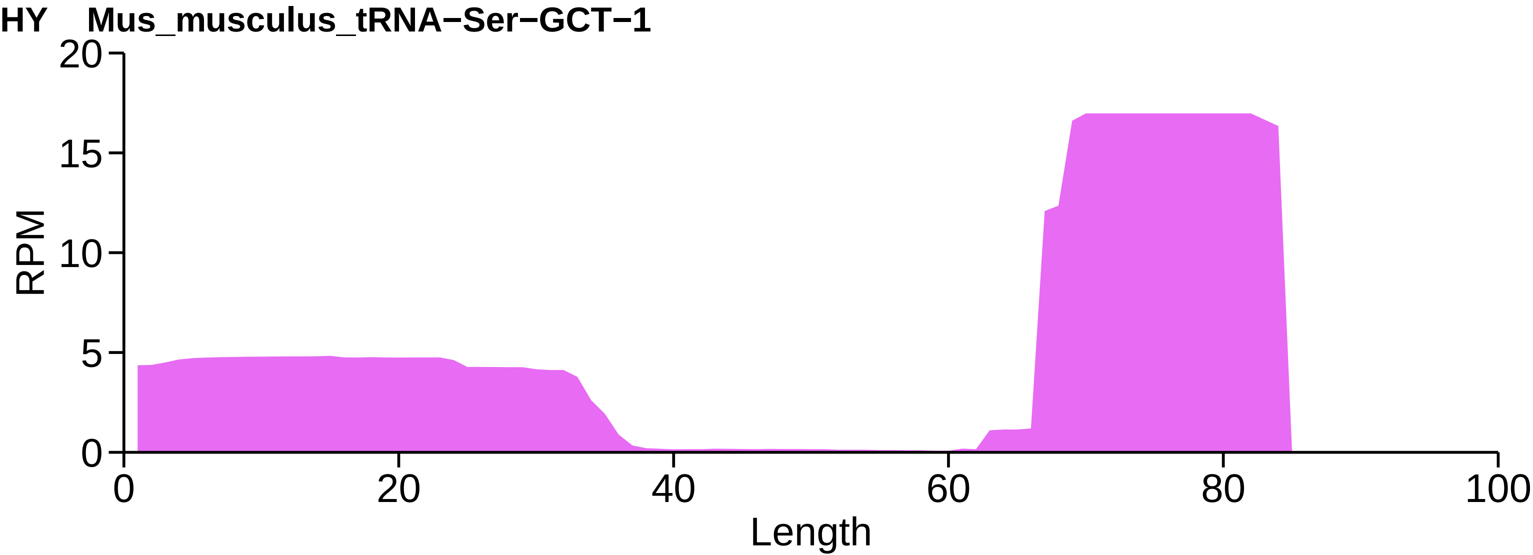

HY Mus_musculus_tRNA−Ser−GCT−1
RPM
20
15
10
5
0
0
20
40
60
80
100
Length

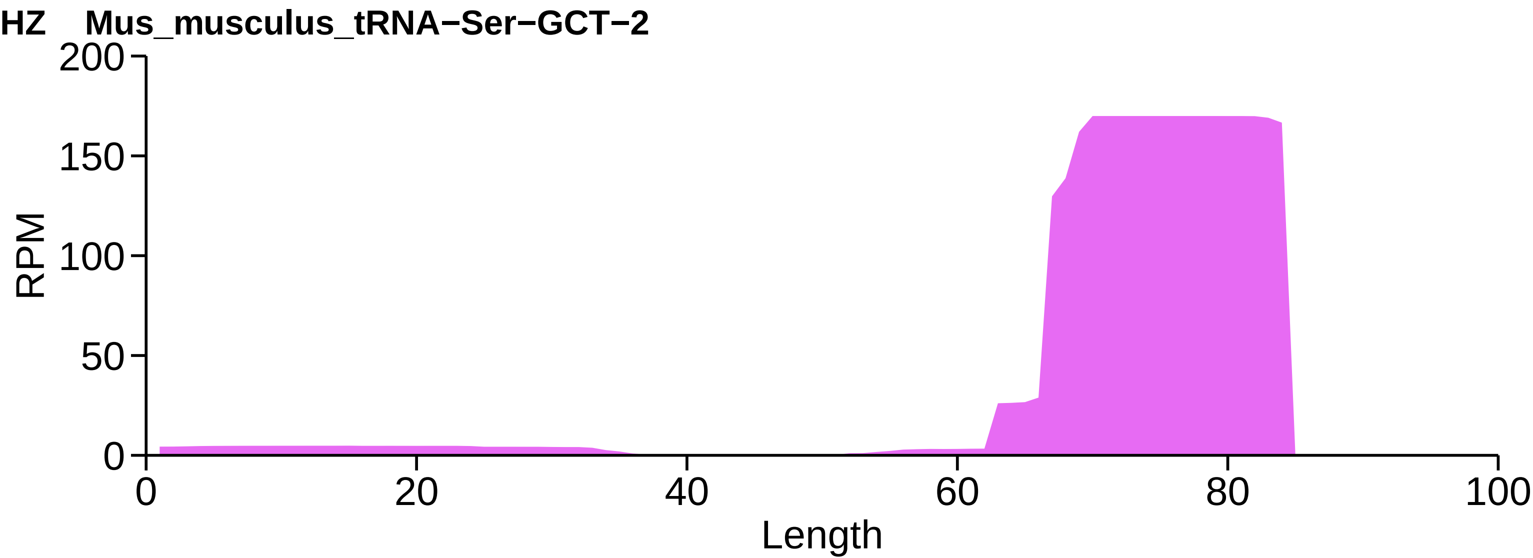
HZ Mus_musculus_tRNA−Ser−GCT−2
RPM
200
150
100
50
0
0
20
40
60
80
100
Length

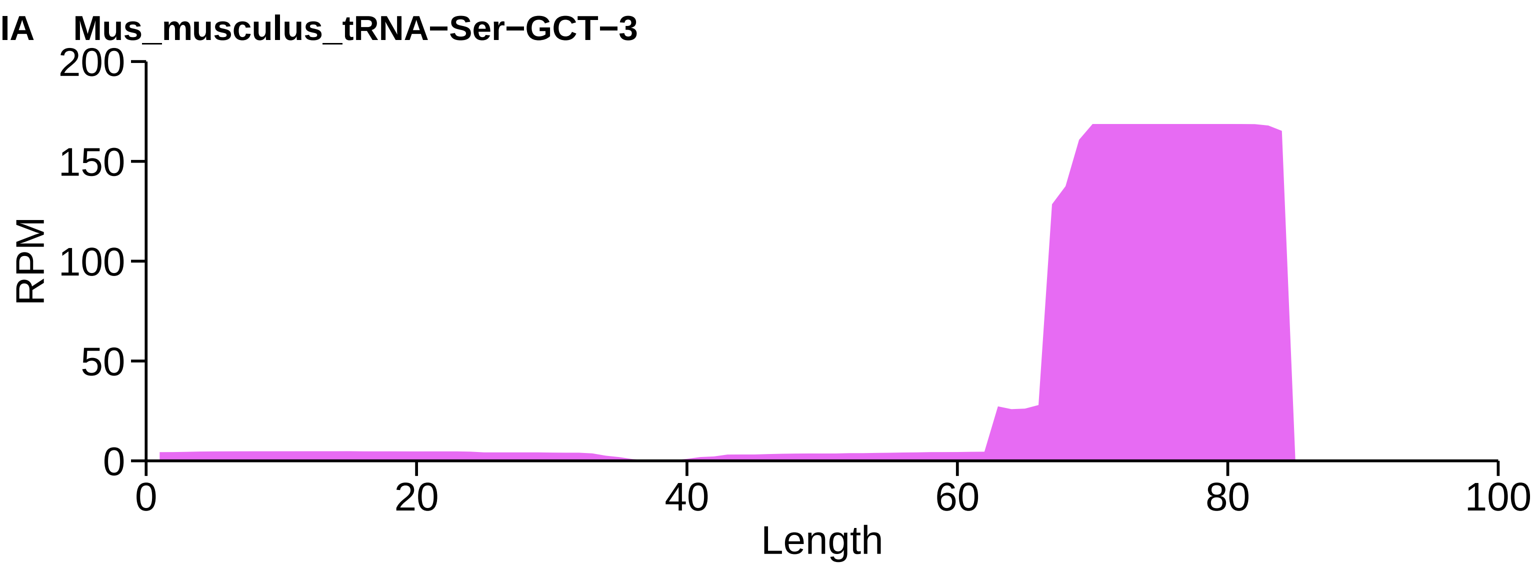

IA Mus_musculus_tRNA−Ser−GCT−3
200
150
100
50
0
RPM
0
20
40
60
80
100
Length

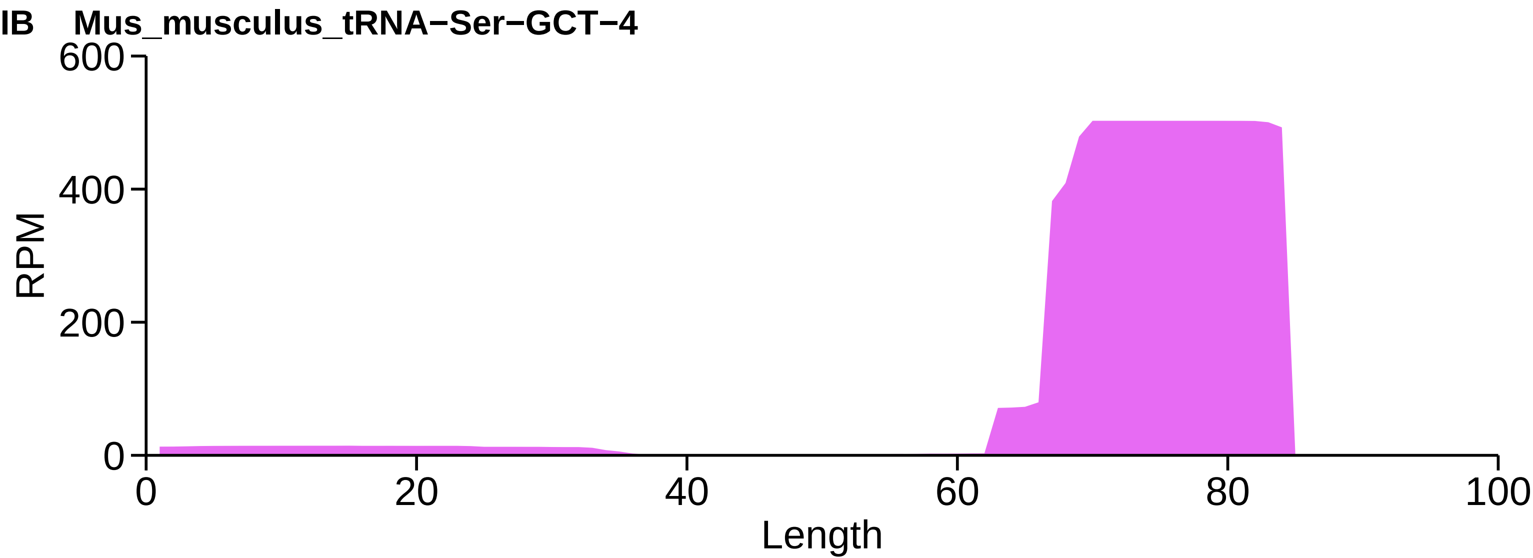
IB Mus_musculus_tRNA−Ser−GCT−4
600
400
200
0
RPM
0
20
40
60
80
100
Length

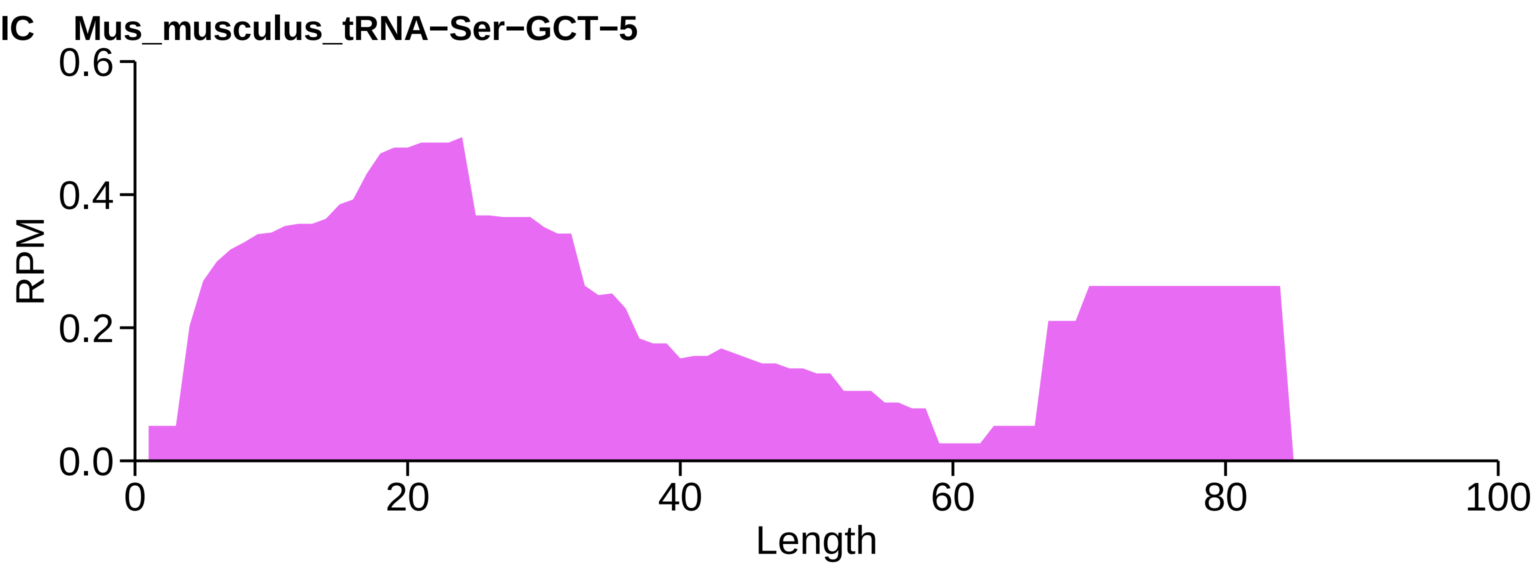
IC Mus_musculus_tRNA−Ser−GCT−5
RPM
0.6
0.4
0.2
0.0
0
20
40
60
80
100
Length

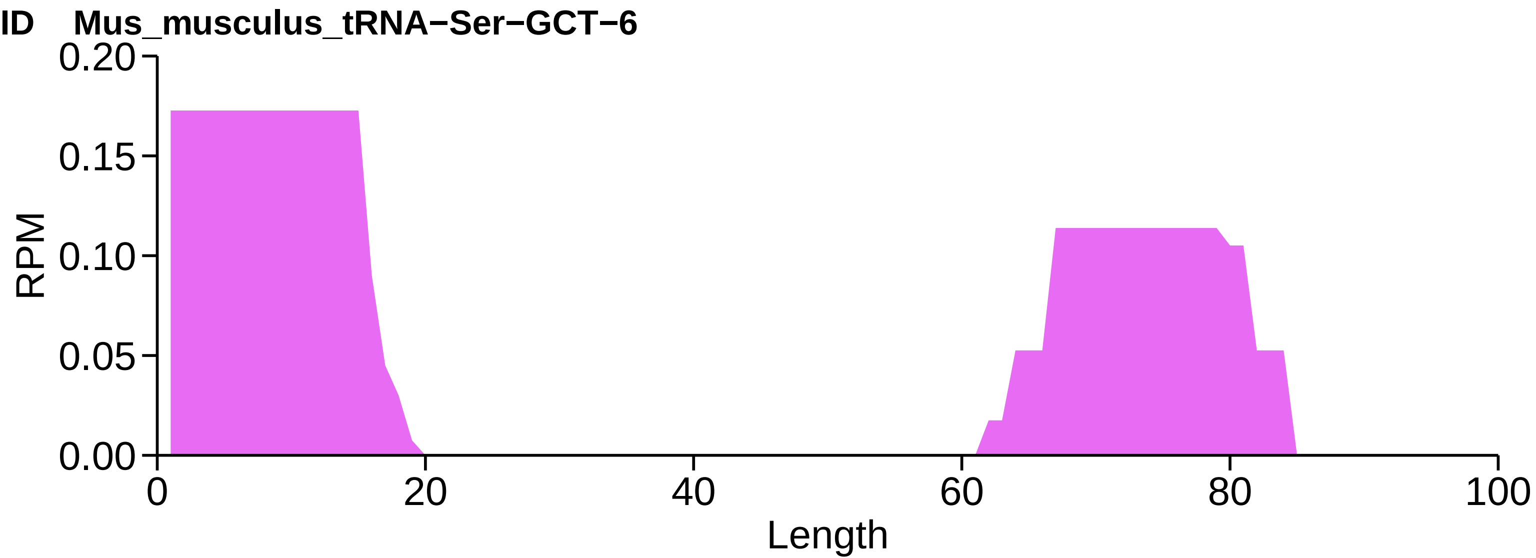
ID Mus_musculus_tRNA−Ser−GCT−6
RPM
0.20
0.15
0.10
0.05
0.00
0
20
40
60
80
100
Length

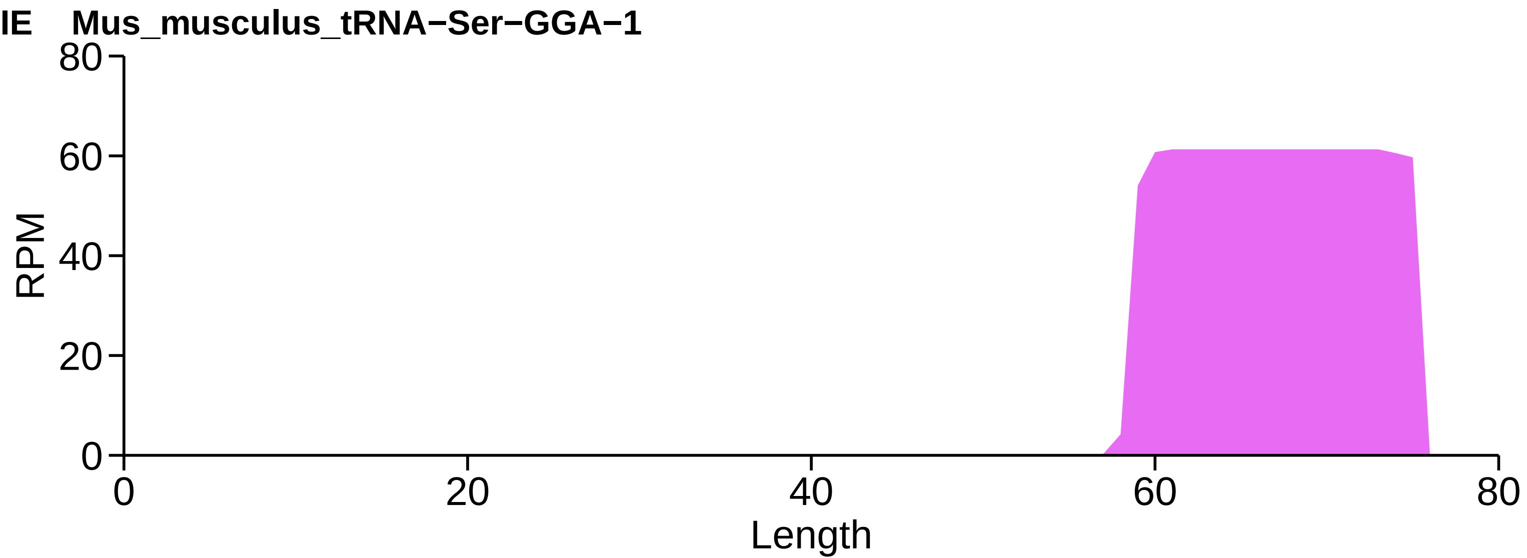
IE Mus_musculus_tRNA−Ser−GGA−1
RPM
0
20
40
60
80
Length
0
20
40
60
80

**IF Mus_musculus_tRNA−Ser−TGA−1**

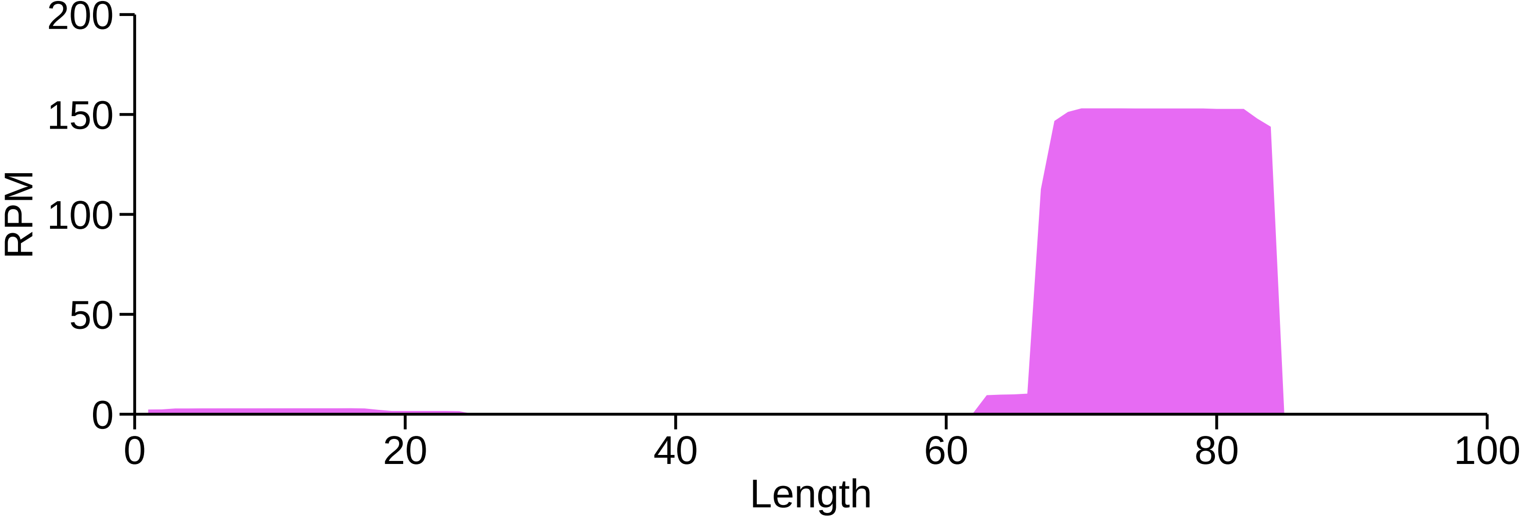

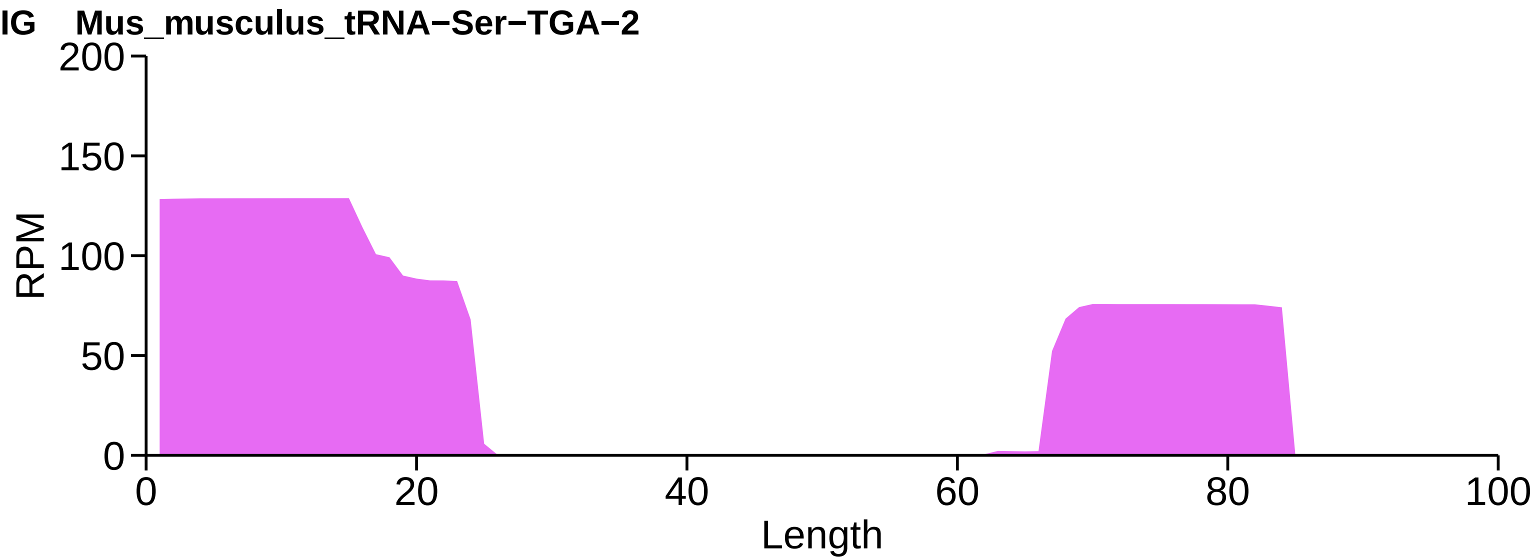
IG Mus_musculus_tRNA−Ser−TGA−2
200
150
100
50
0
RPM
0
20
40
60
80
100
Length

IH Mus_musculus_tRNA−Thr−AGT−1

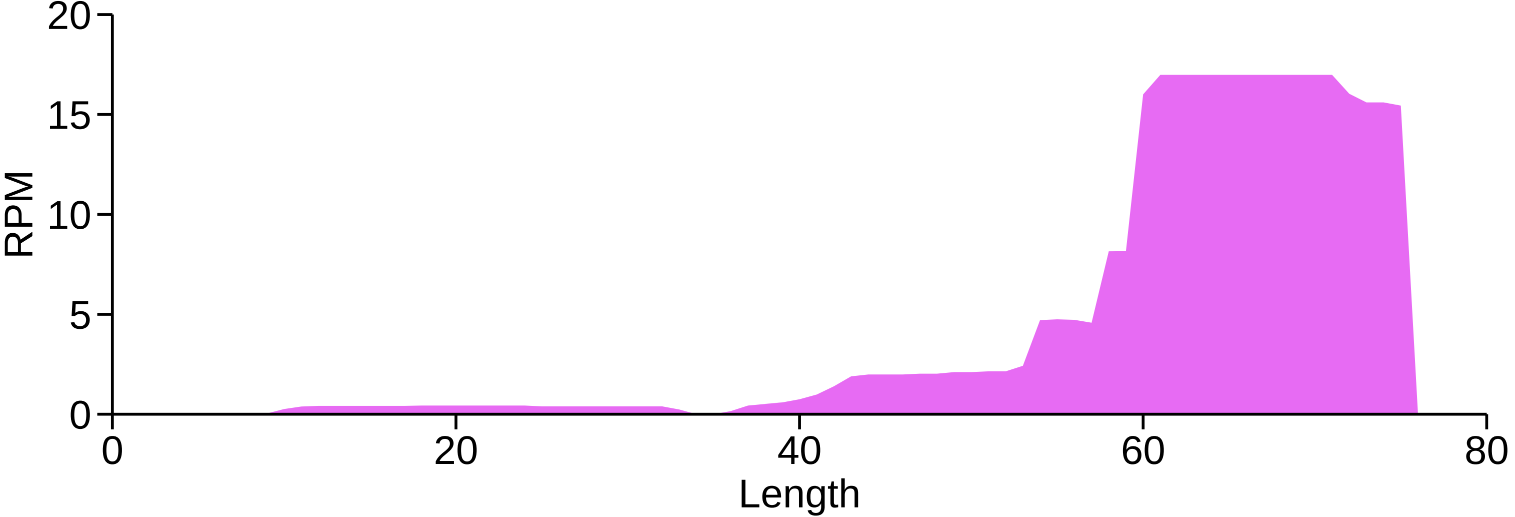

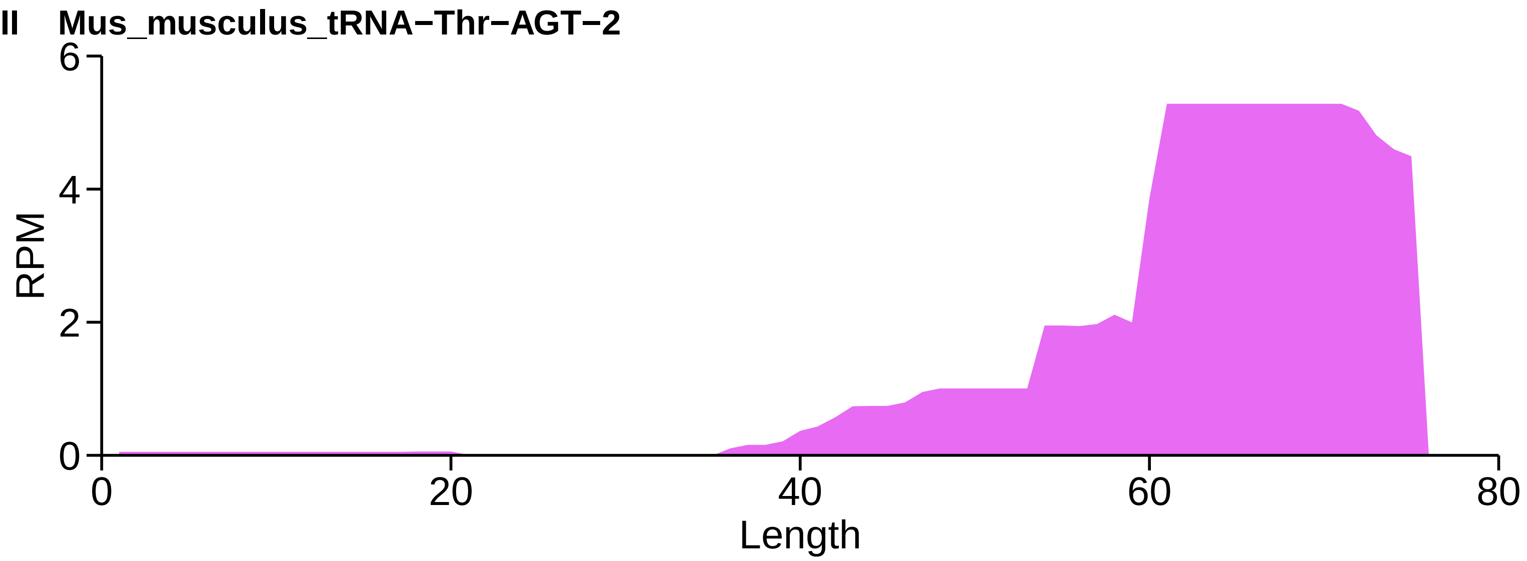

II Mus_musculus_tRNA−Thr−AGT−2
RPM
0
2
4
6
0
20
40
60
80
Length

IJ Mus_musculus_tRNA−Thr−AGT−3

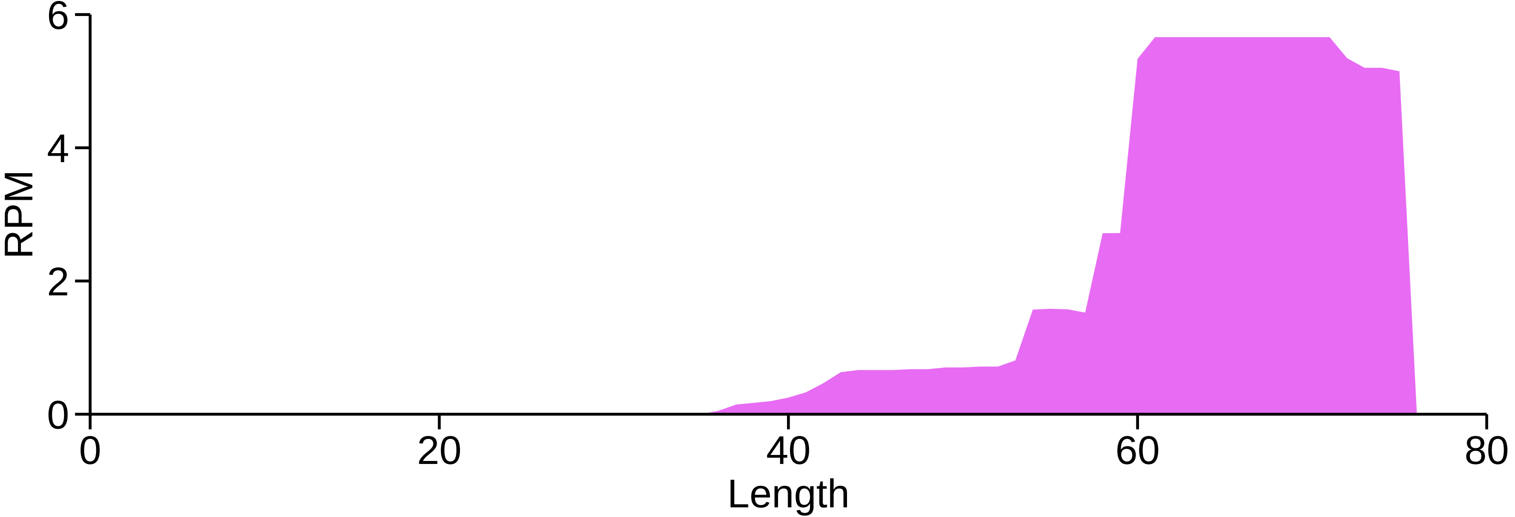

IK Mus_musculus_tRNA−Thr−AGT−4

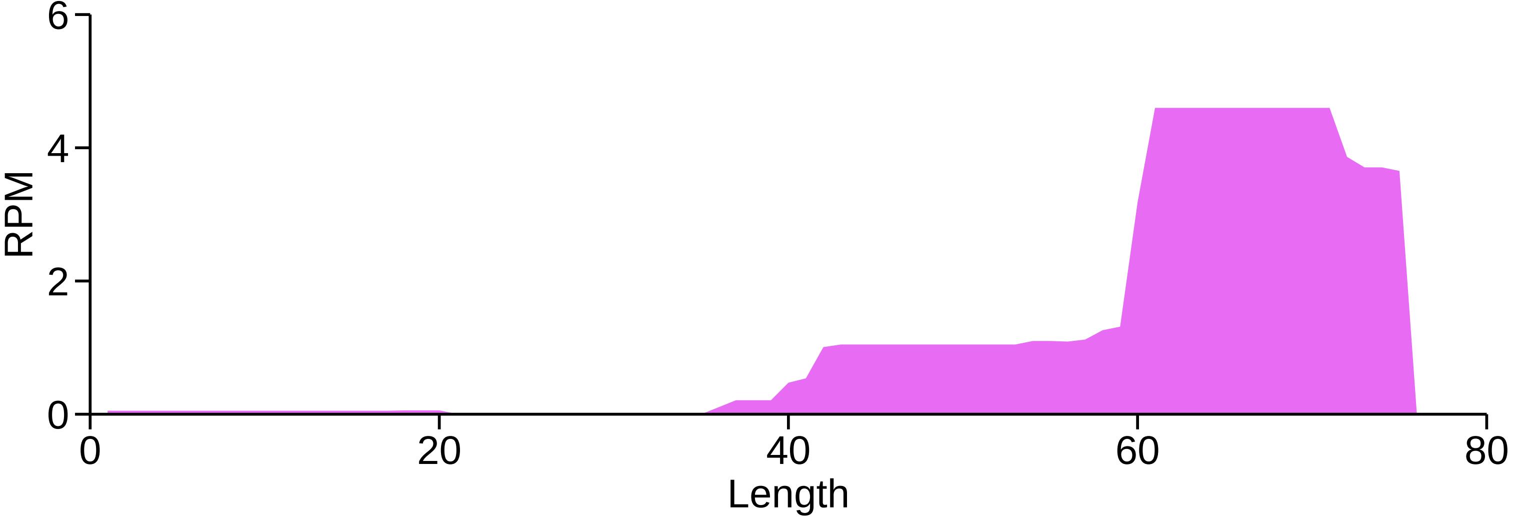

IL Mus_musculus_tRNA−Thr−AGT−5

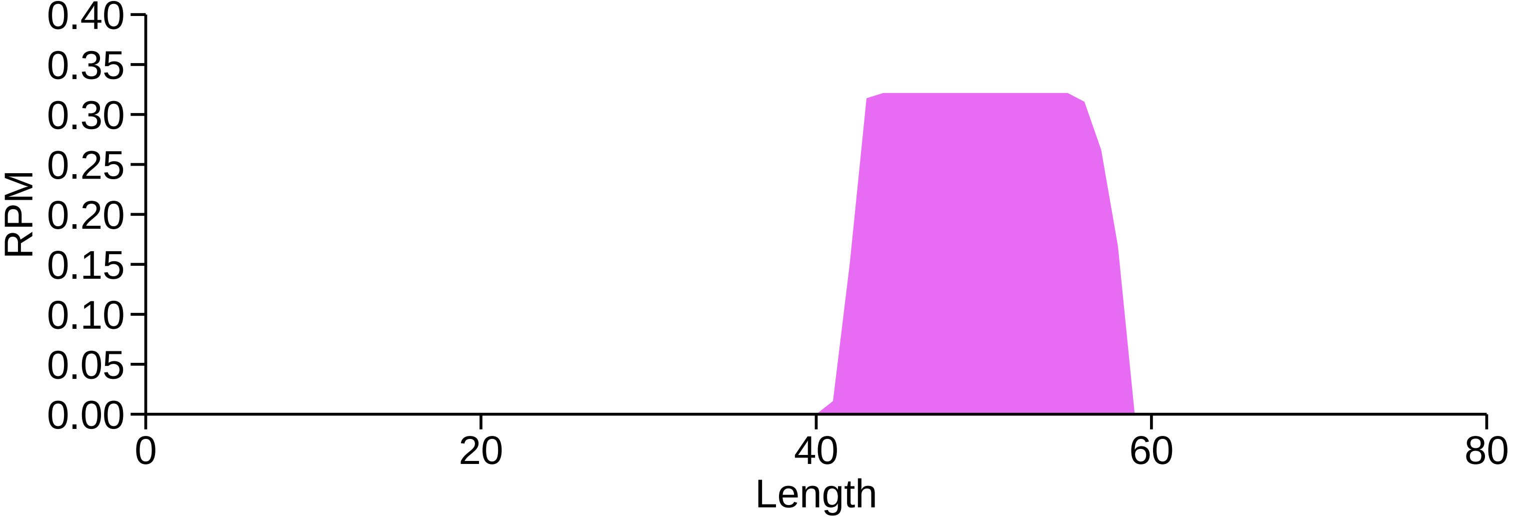

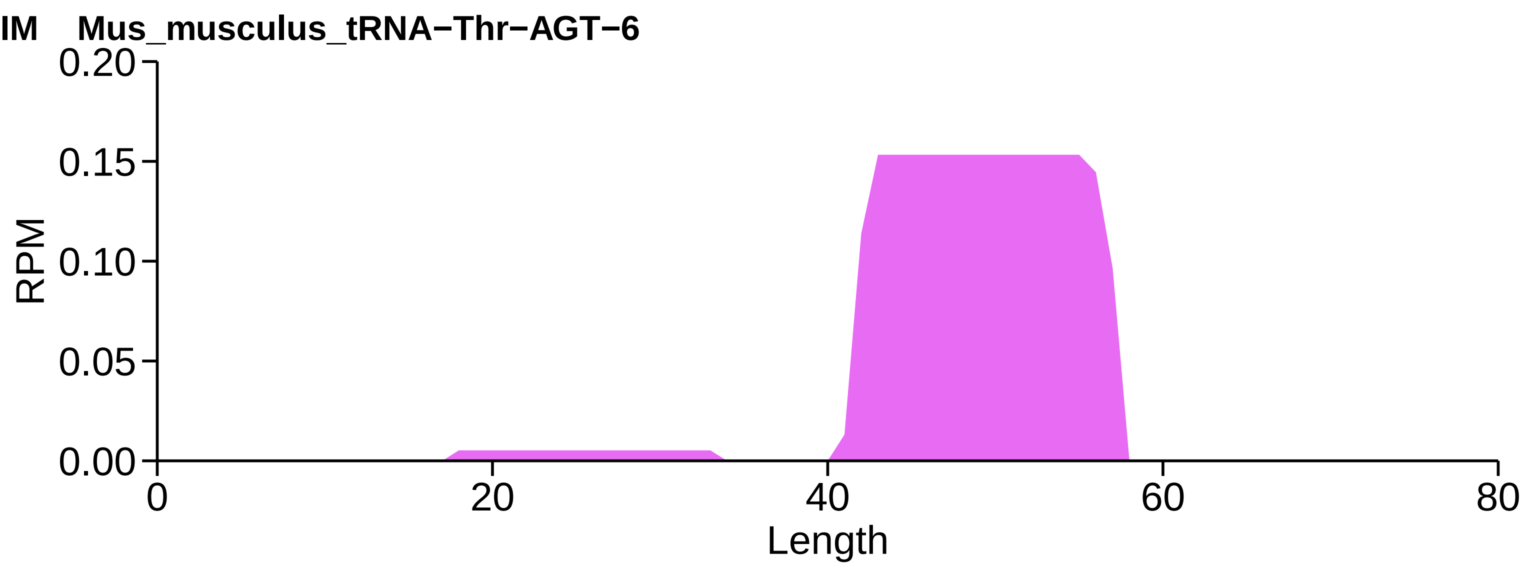
IM Mus_musculus_tRNA−Thr−AGT−6
0.20
0.15
0.10
0.05
0.00
RPM
0
20
40
60
80
Length

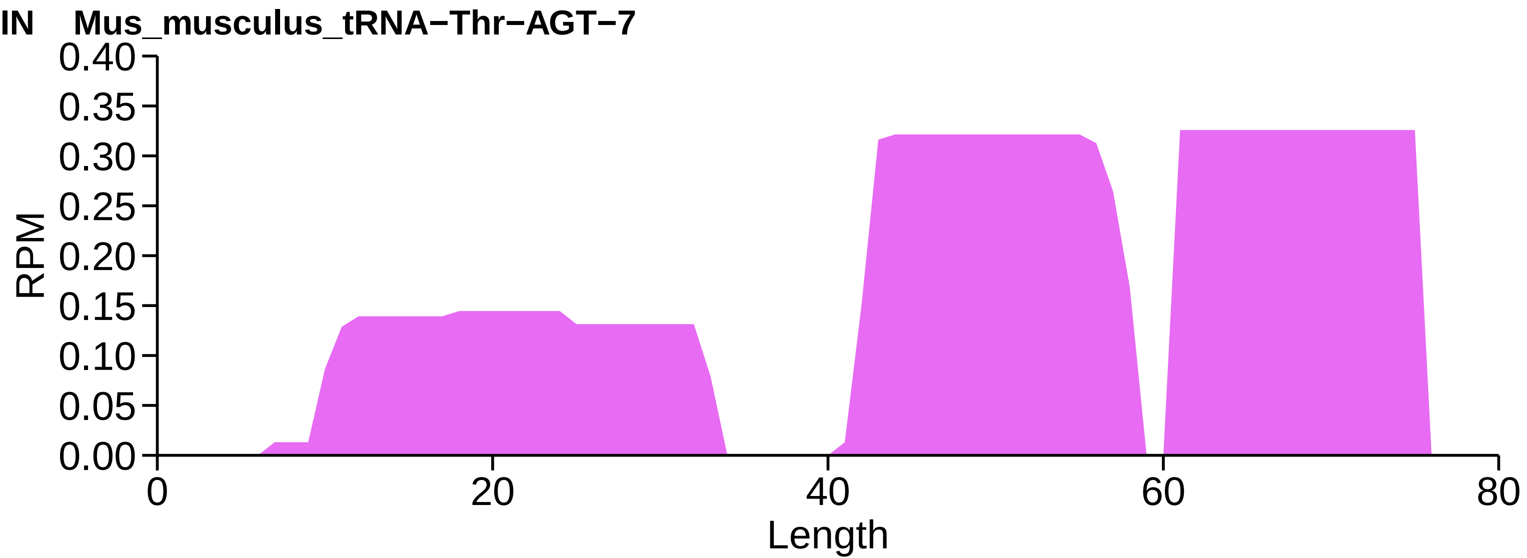
IN Mus_musculus_tRNA−Thr−AGT−7
RPM
0.00
0.05
0.10
0.15
0.20
0.25
0.30
0.35
0.40
0
20
40
60
80
Length

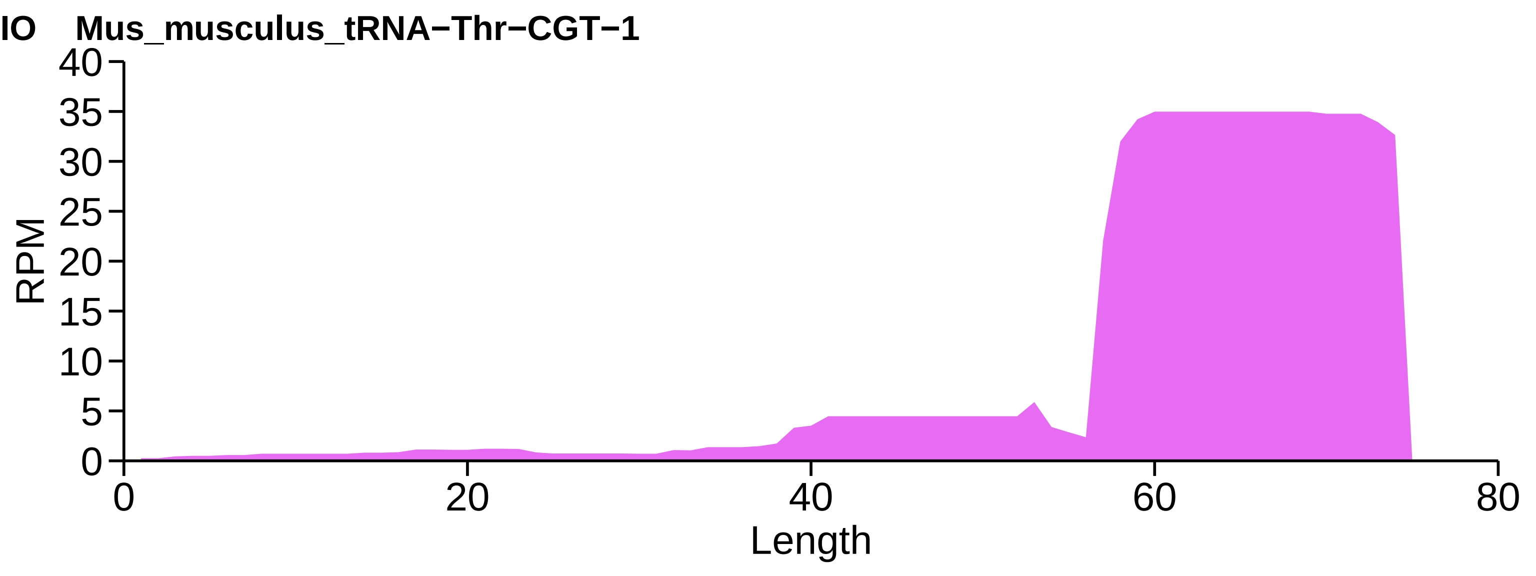
IO Mus_musculus_tRNA−Thr−CGT−1
RPM
0
5
10
15
20
25
30
35
40
0
20
40
60
80
Length

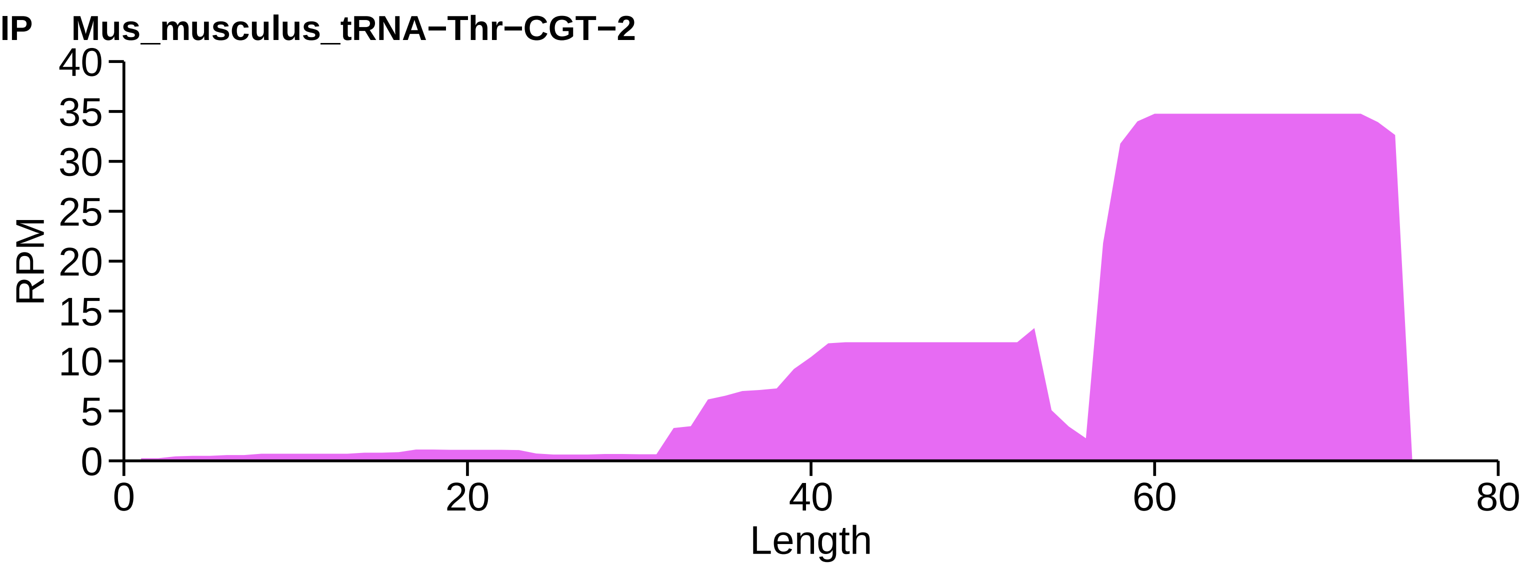
IP Mus_musculus_tRNA−Thr−CGT−2
RPM
0
5
10
15
20
25
30
35
40
0
20
40
60
80
Length

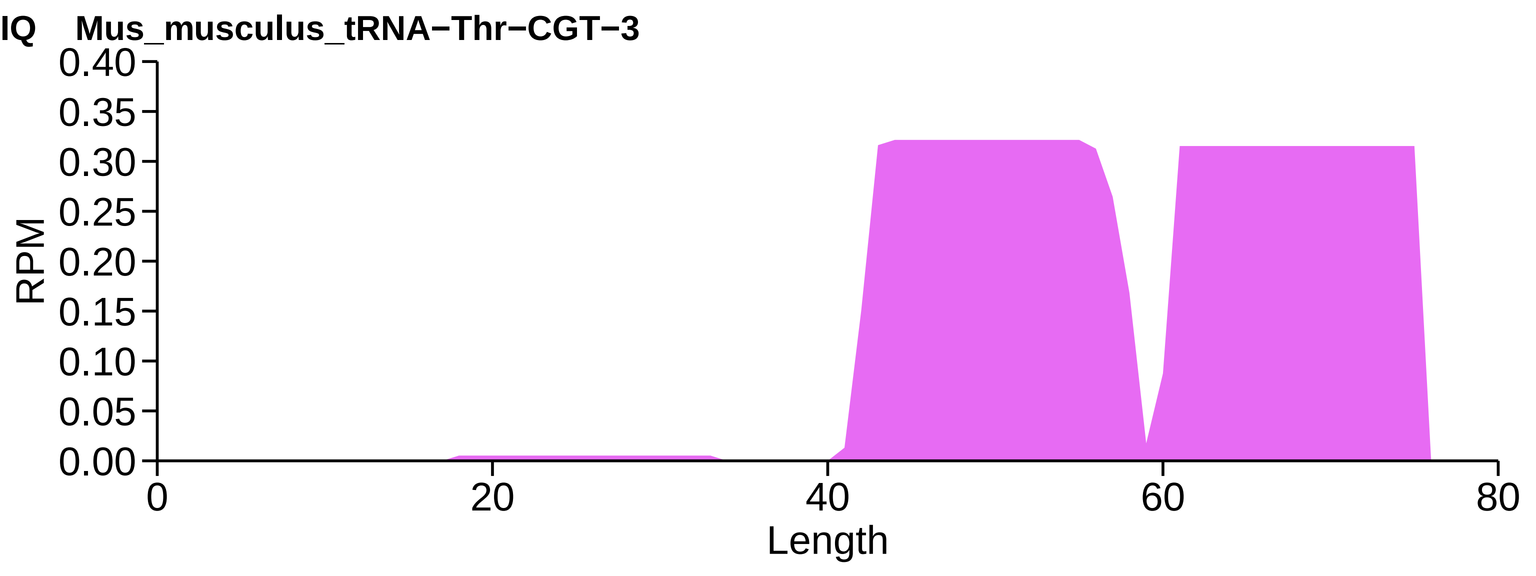

IQ Mus_musculus_tRNA−Thr−CGT−3
RPM
0.40
0.35
0.30
0.25
0.20
0.15
0.10
0.05
0.00
0
20
40
60
80
Length

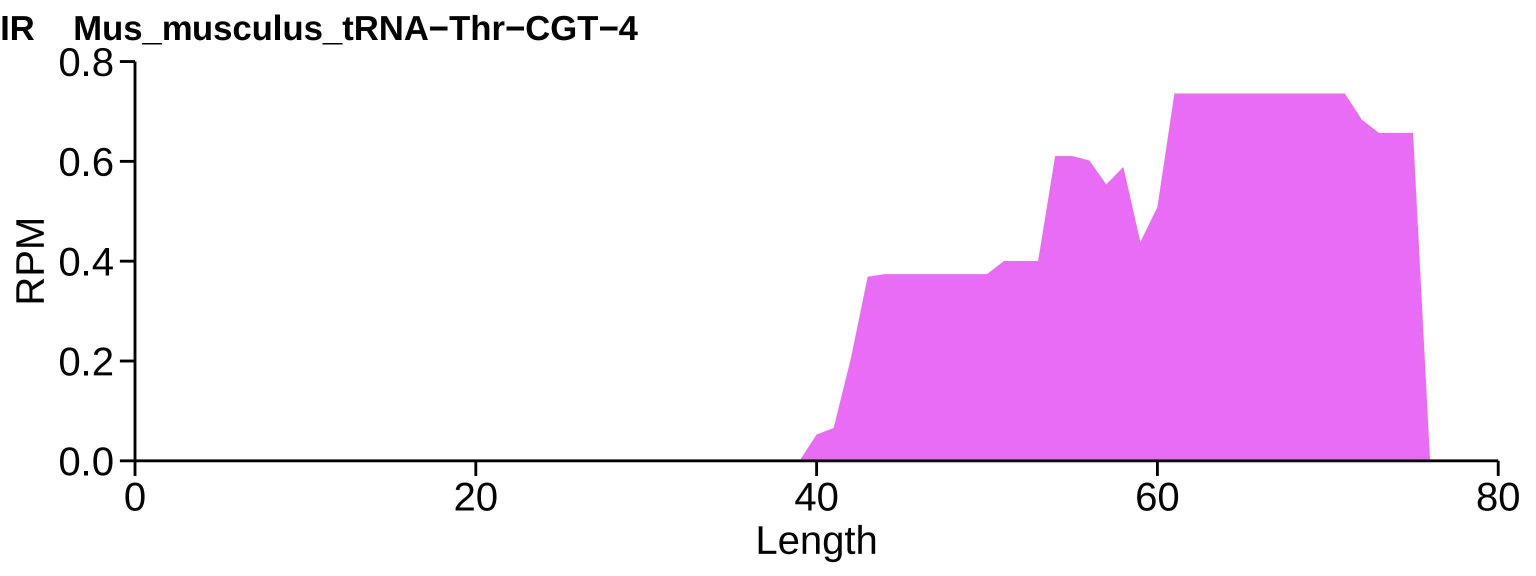

IR Mus_musculus_tRNA−Thr−CGT−4
0.8
0.6
0.4
0.2
0.0
RPM
0
20
40
60
80
Length

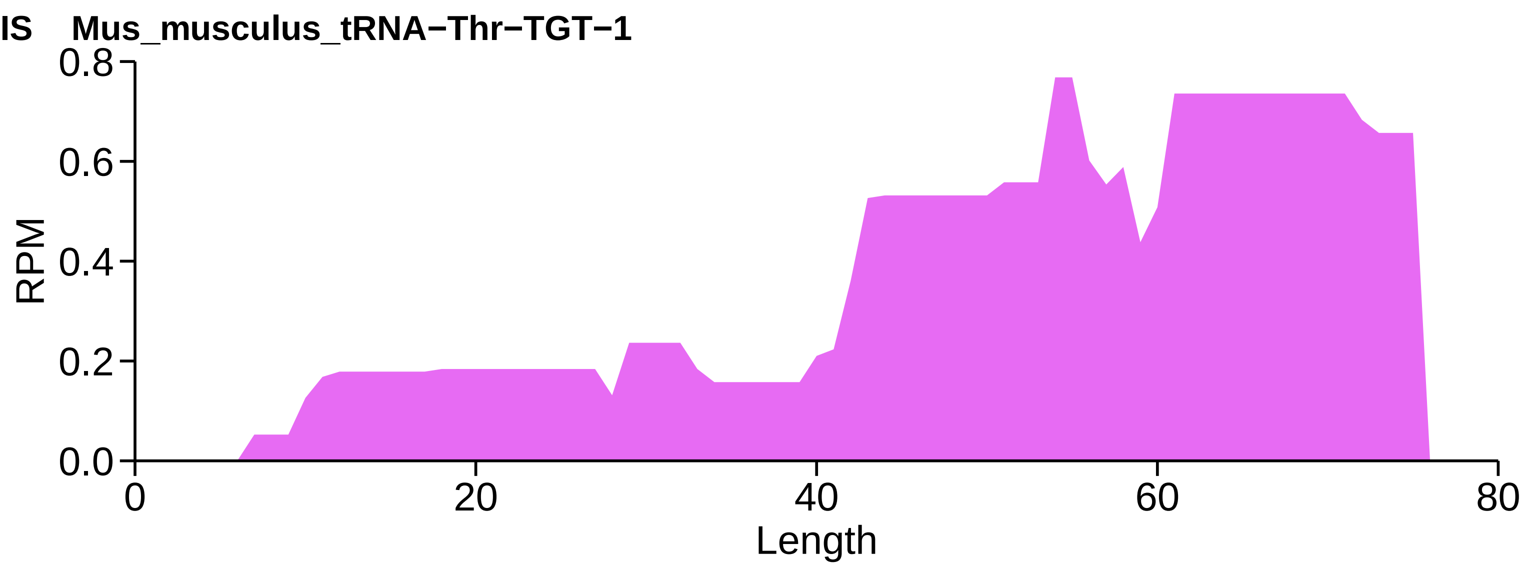
IS Mus_musculus_tRNA−Thr−TGT−1
RPM
0.0
0.2
0.4
0.6
0.8
0
20
40
60
80
Length

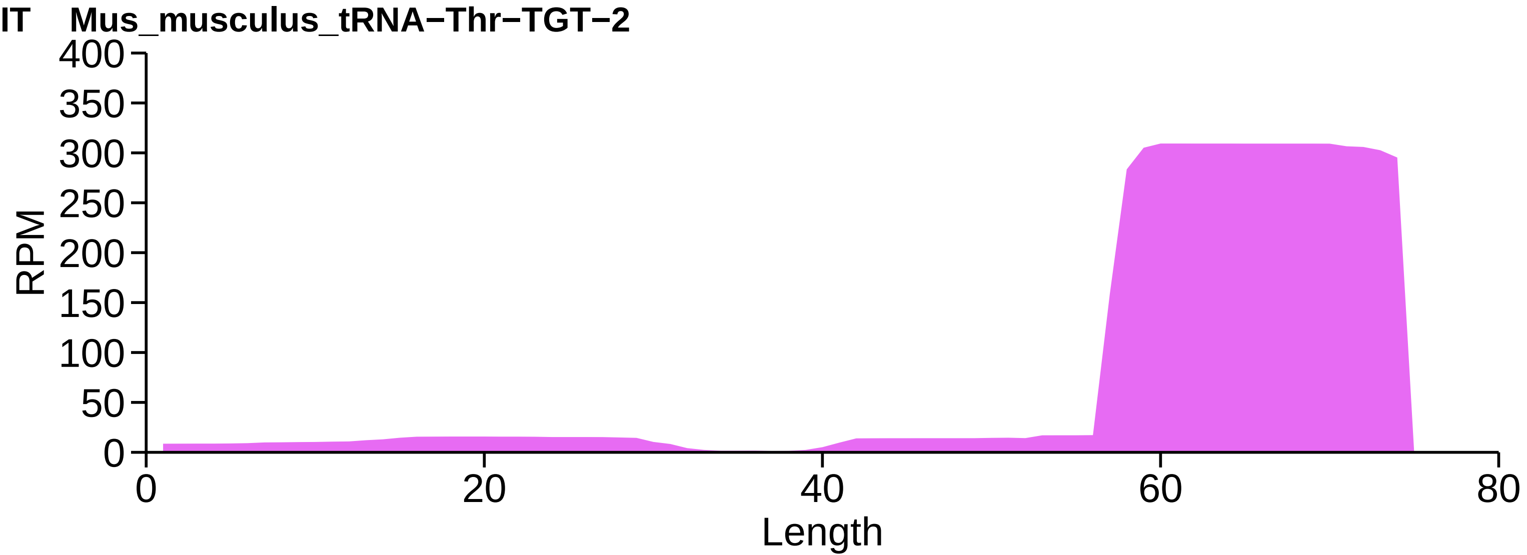

IT Mus_musculus_tRNA−Thr−TGT−2
400
350
300
250
200
150
100
50
0
RPM
0
20
40
60
80
Length

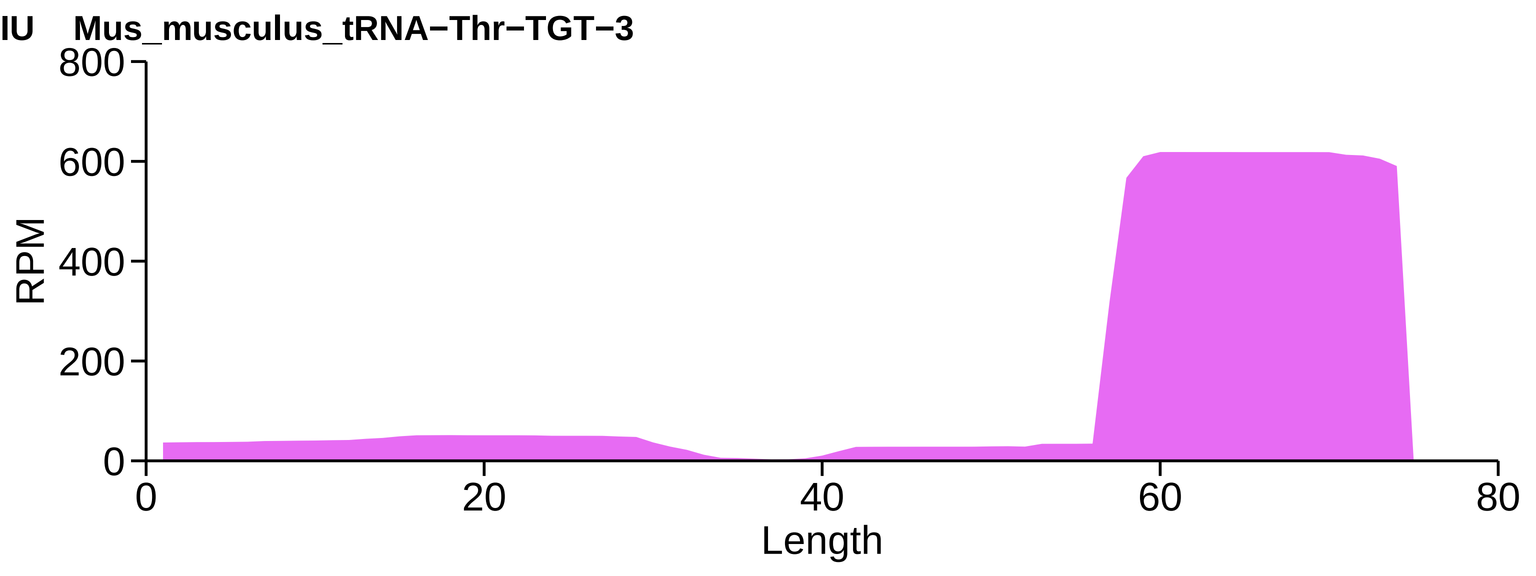
IU Mus_musculus_tRNA−Thr−TGT−3
800
600
400
200
0
RPM
0
20
40
60
80
Length

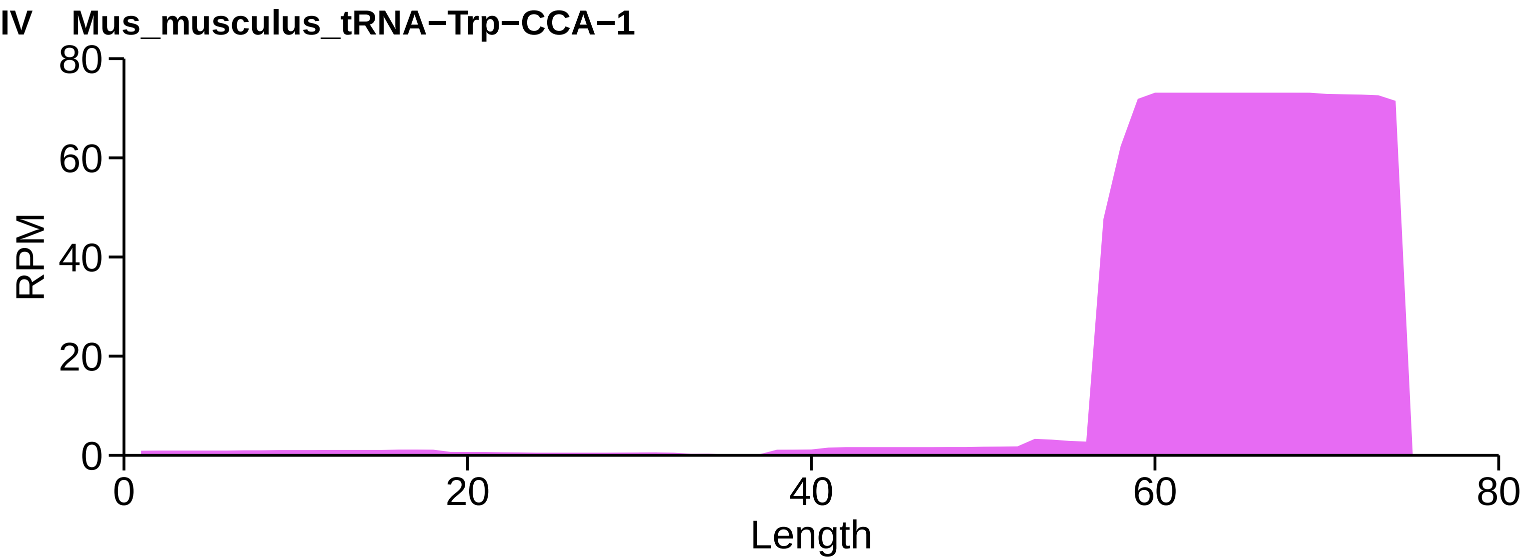
IV Mus_musculus_tRNA−Trp−CCA−1
80
60
40
20
0
RPM
0
20
40
60
80
Length

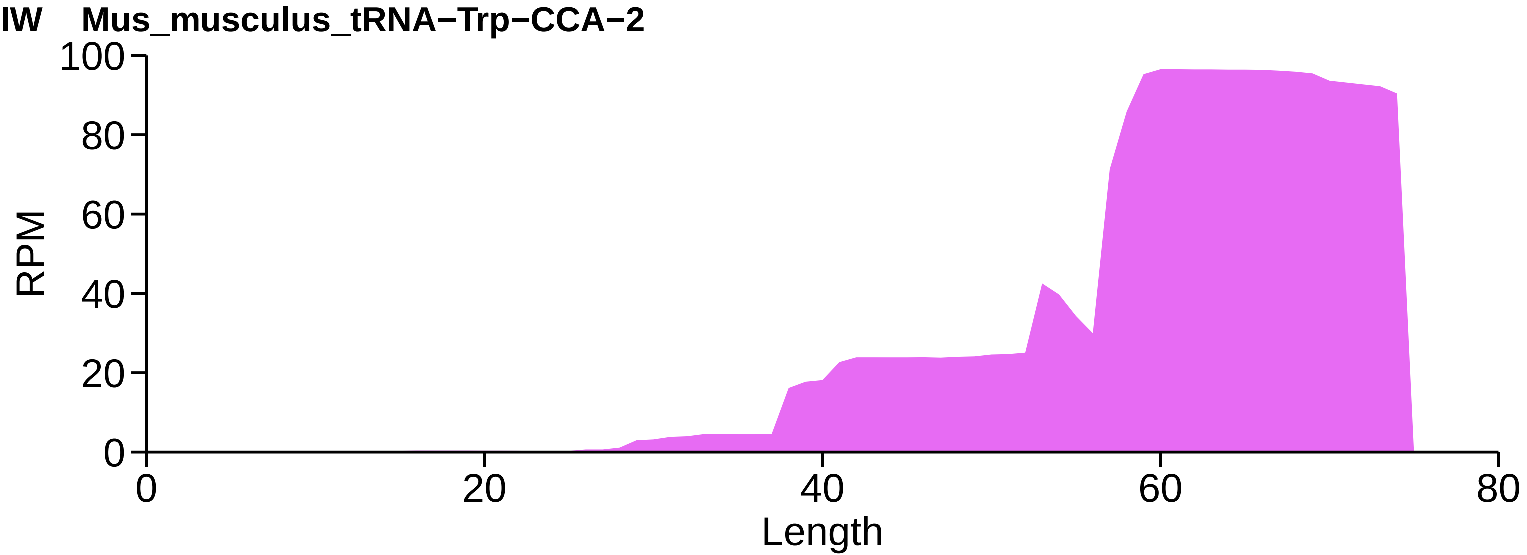

IW Mus_musculus_tRNA−Trp−CCA−2
RPM
100
80
60
40
20
0
0
20
40
60
80
Length

IX **Mus_musculus_tRNA−Trp−CCA−3**

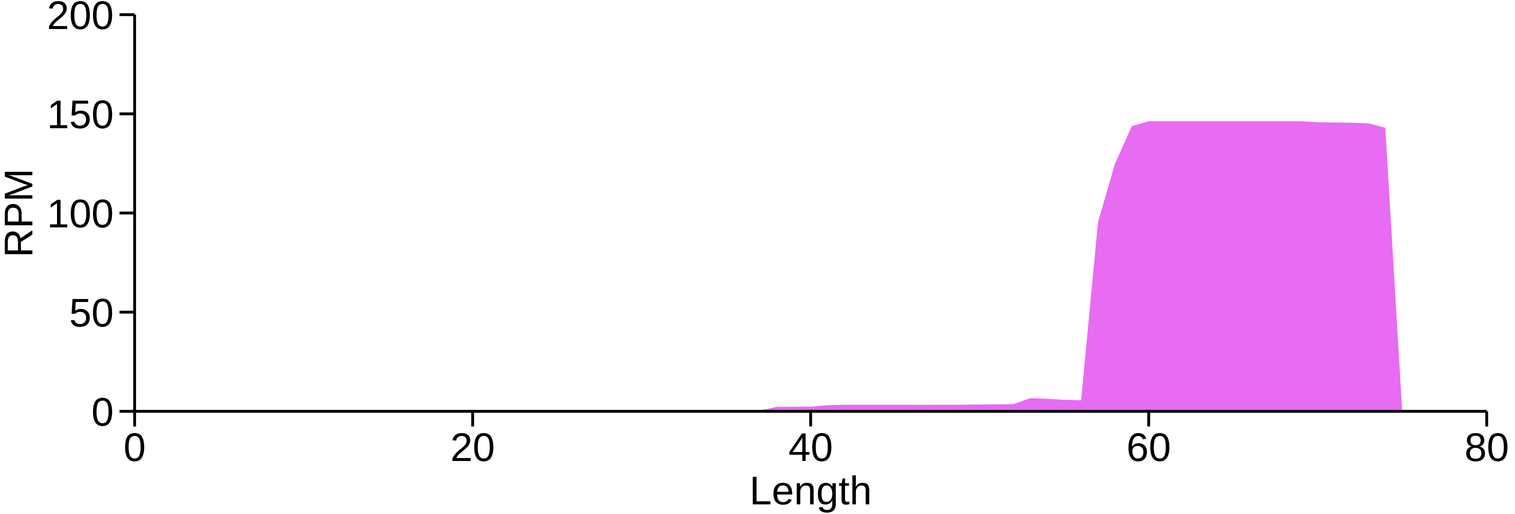

IY Mus_musculus_tRNA−Trp−CCA−4

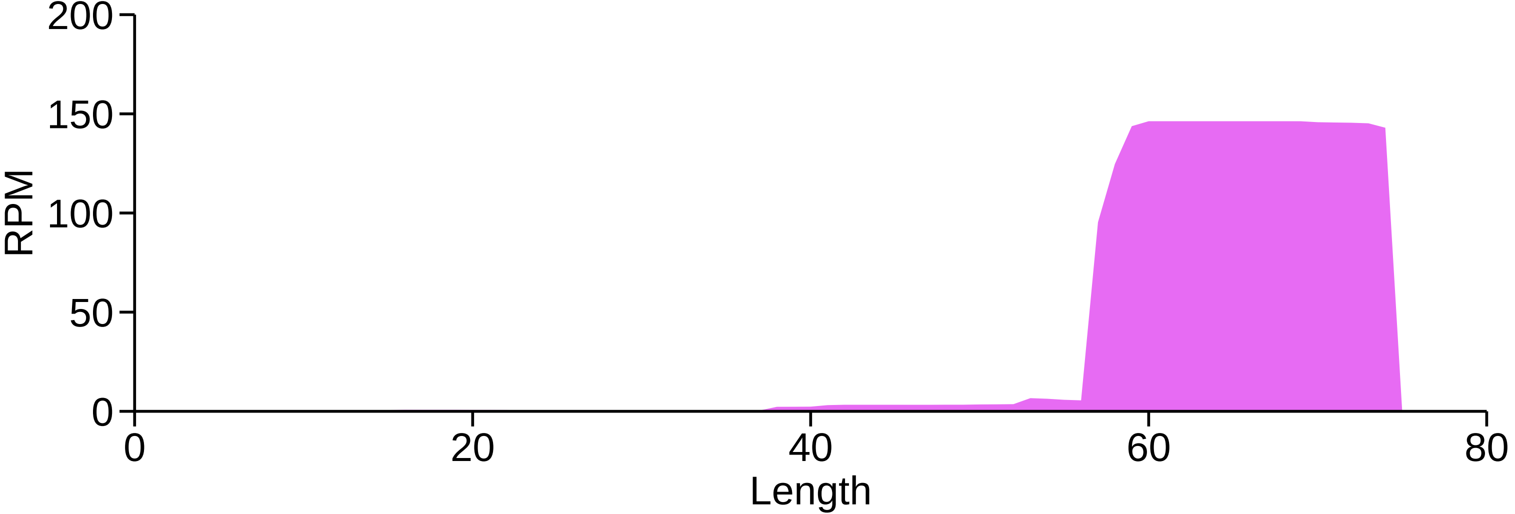

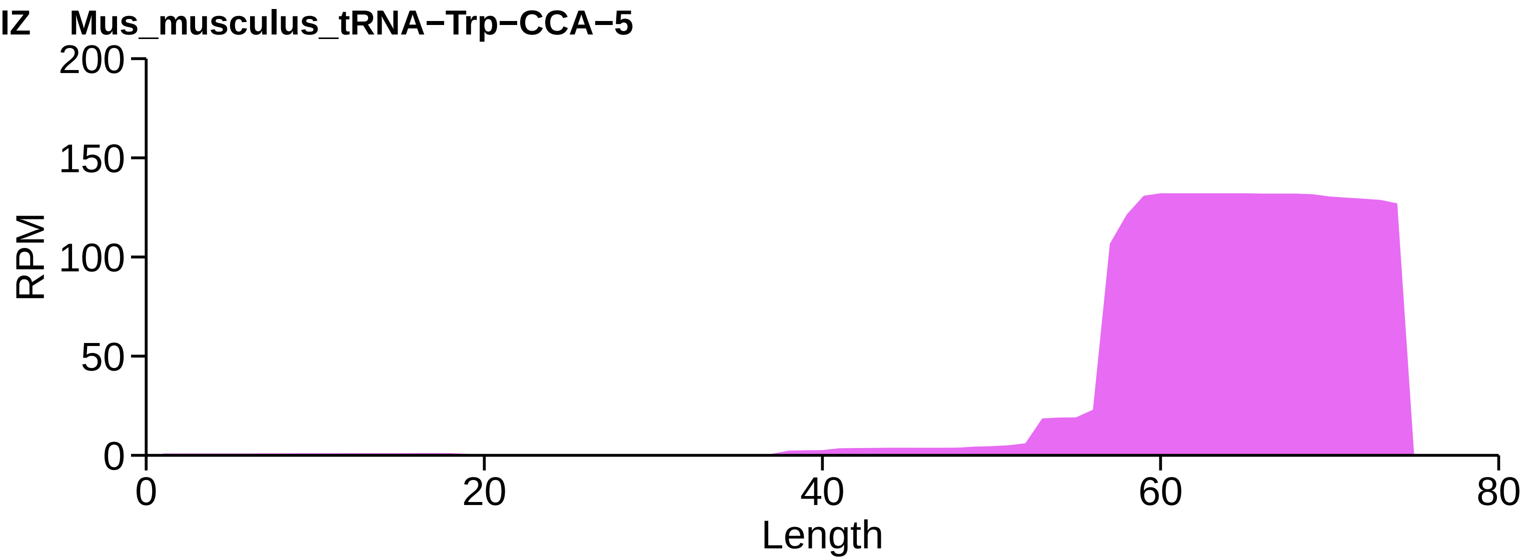
IZ Mus_musculus_tRNA−Trp−CCA−5
RPM
200
150
100
50
0
0
20
40
60
80
Length

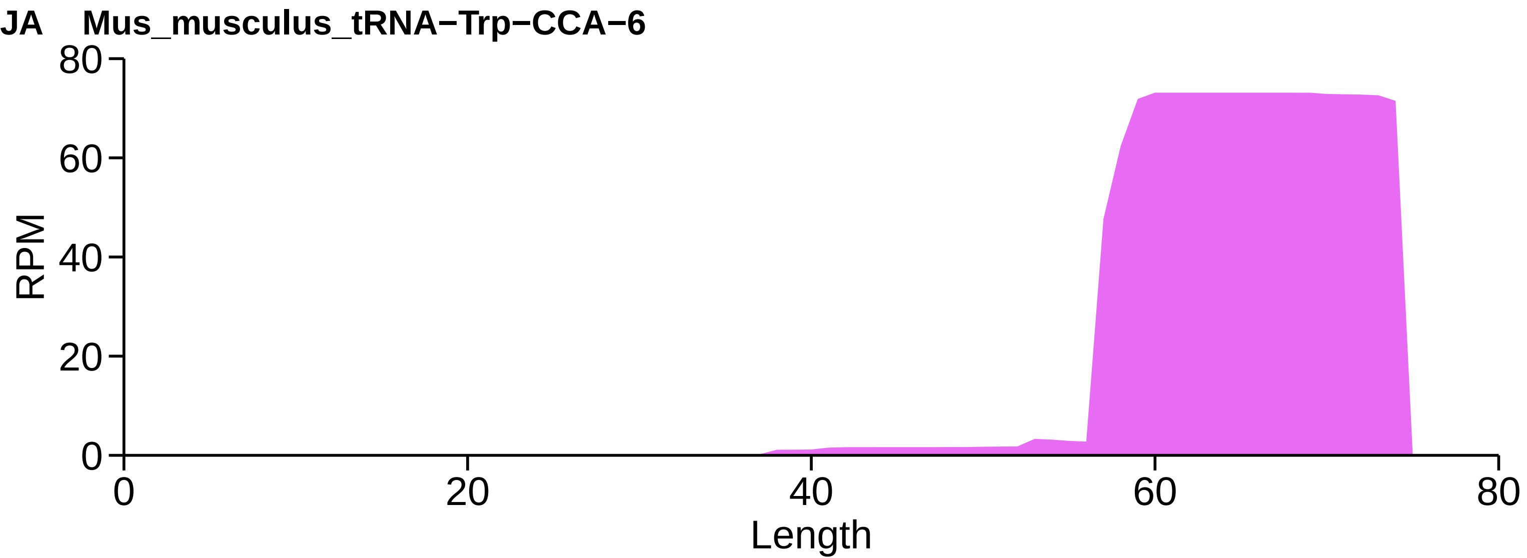
JA Mus_musculus_tRNA−Trp−CCA−6
RPM
Length
0
20
40
60
80
0
20
40
60
80

JB Mus_musculus_tRNA−Tyr−GTA−1

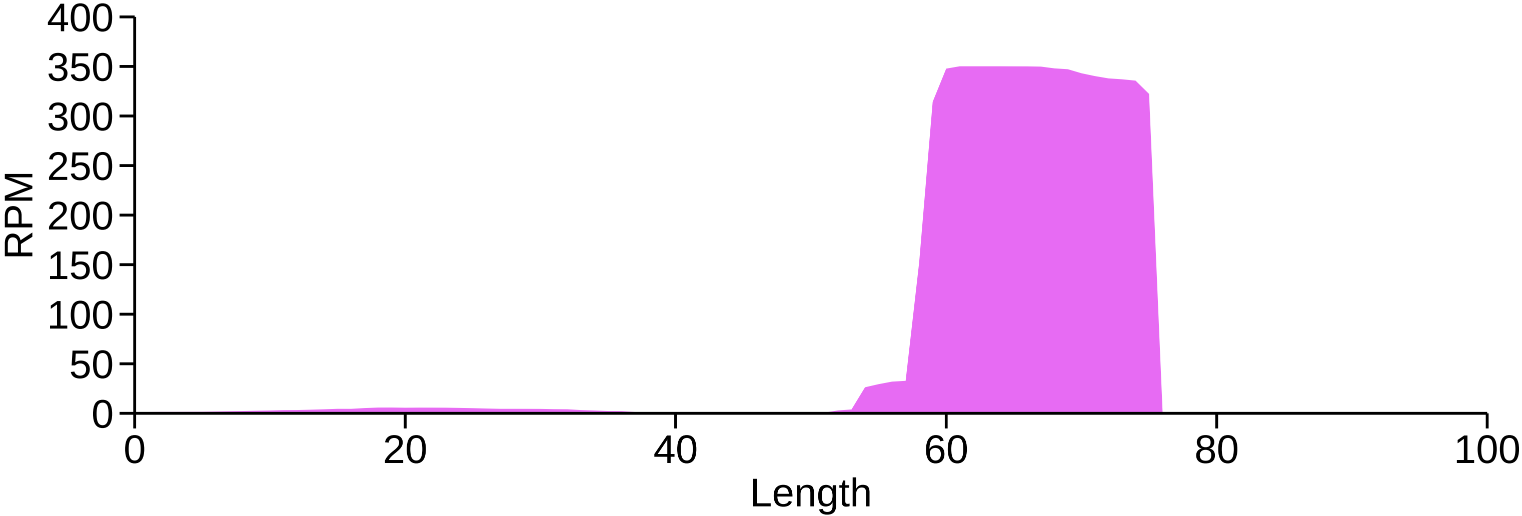

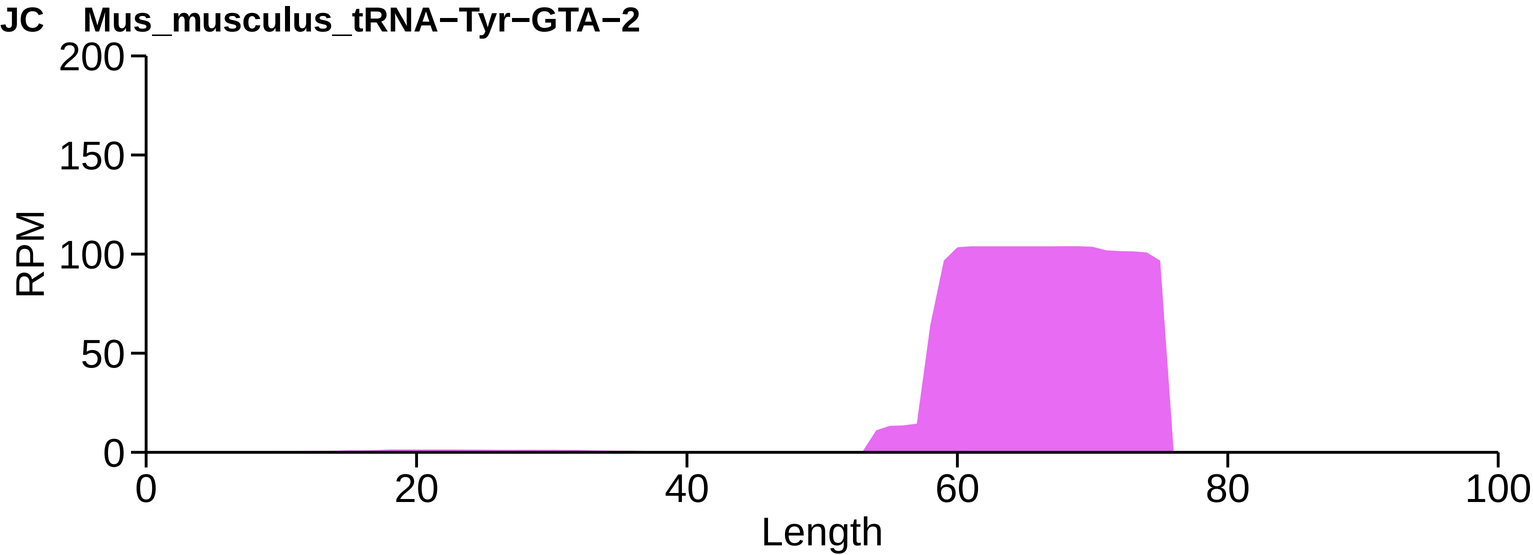
JC Mus_musculus_tRNA−Tyr−GTA−2
RPM
200
150
100
50
0
0
20
40
60
80
100
Length

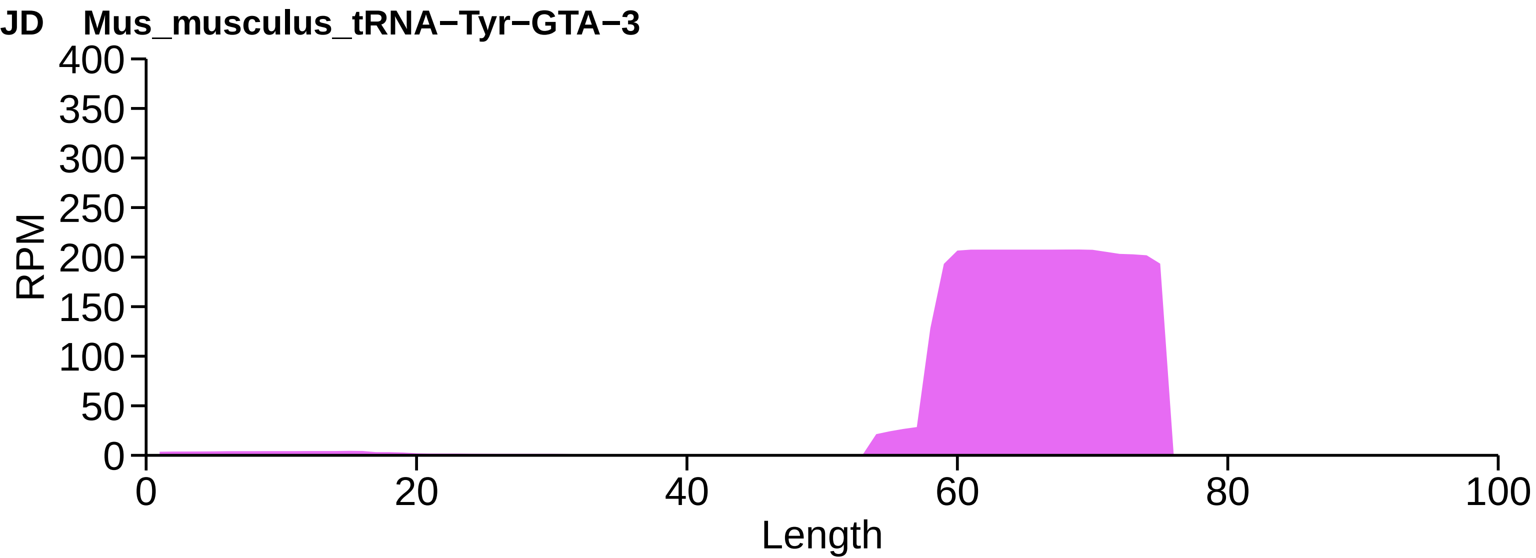
JD Mus_musculus_tRNA−Tyr−GTA−3
RPM
400
350
300
250
200
150
100
50
0
0
20
40
60
80
100
Length

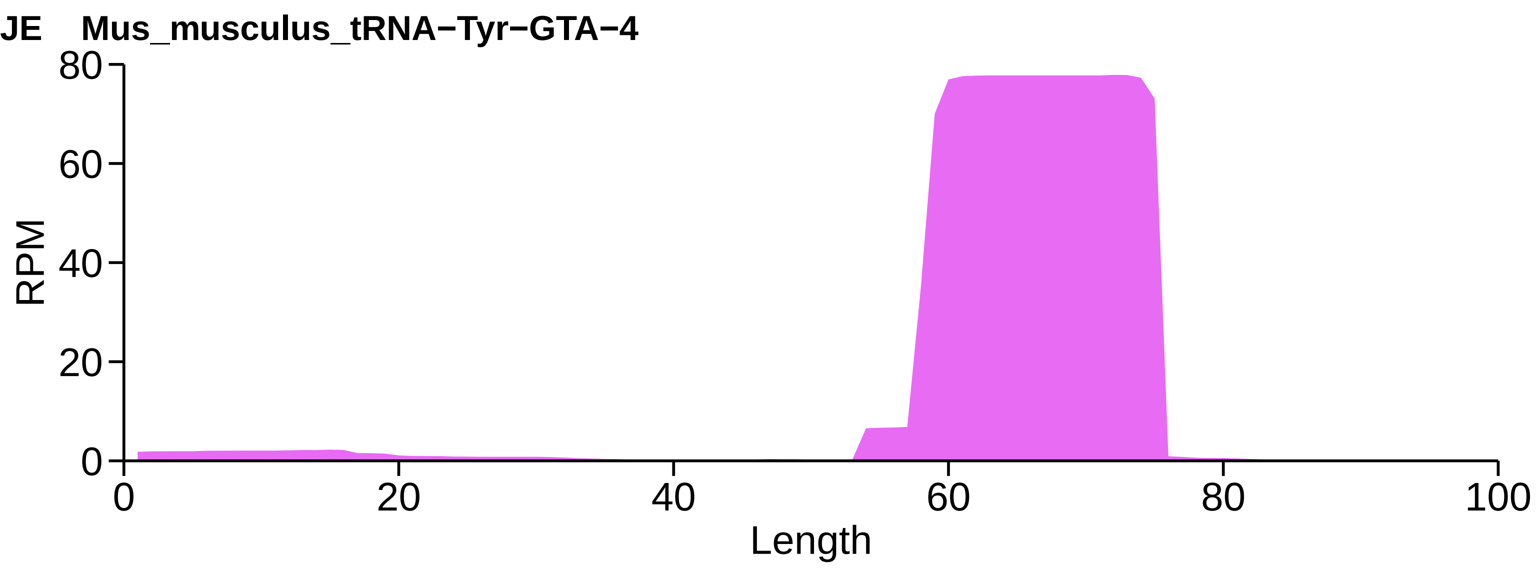
JE Mus_musculus_tRNA−Tyr−GTA−4
RPM
80
60
40
20
0
0
20
40
60
80
100
Length

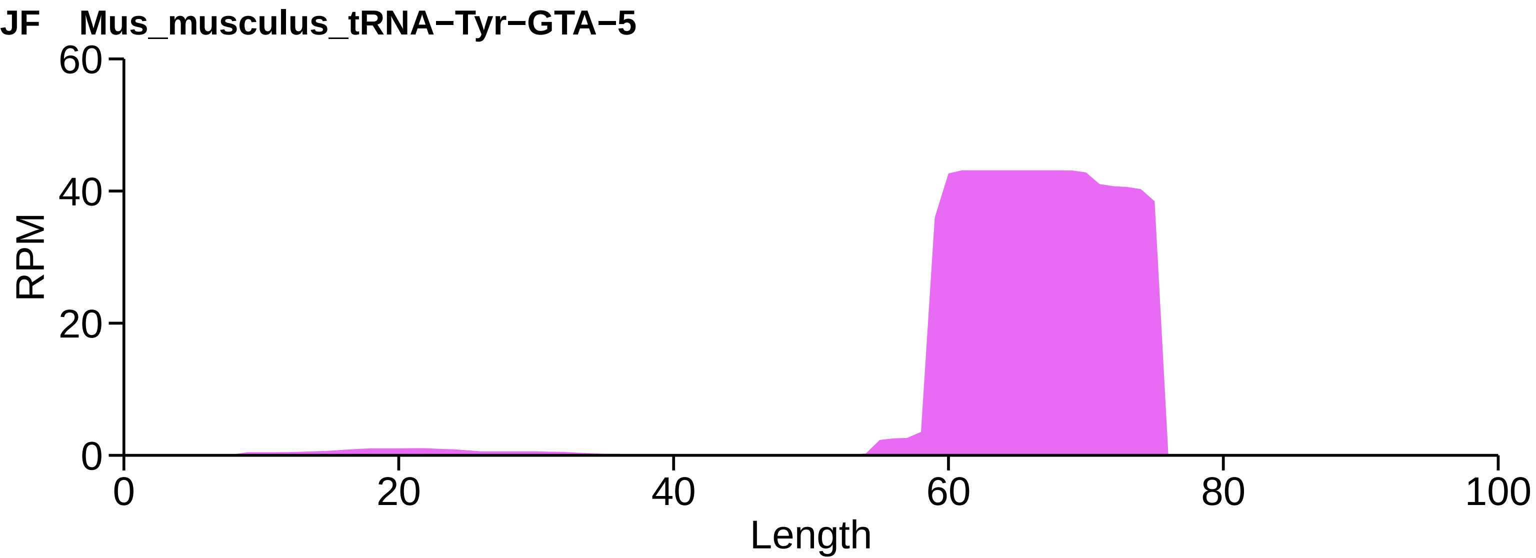
JF Mus_musculus_tRNA−Tyr−GTA−5
60
40
20
0
RPM
0
20
40
60
80
100
Length

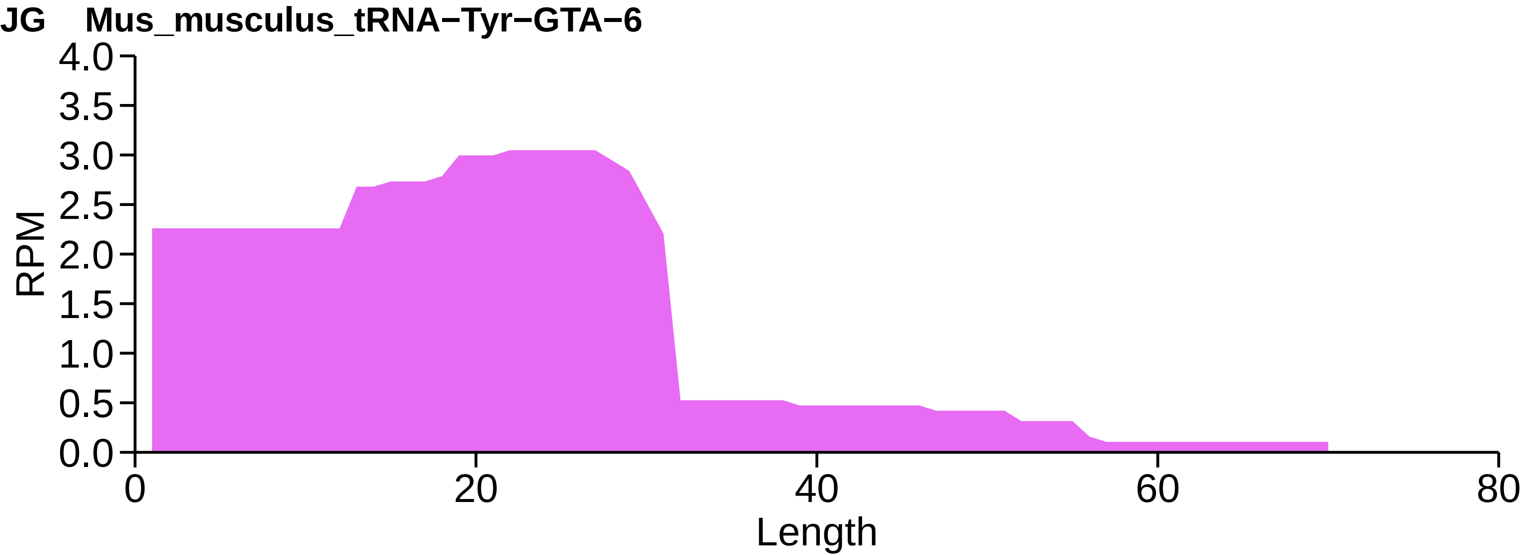

JG Mus_musculus_tRNA−Tyr−GTA−6
RPM
4.0
3.5
3.0
2.5
2.0
1.5
1.0
0.5
0.0
0
20
40
60
80
Length

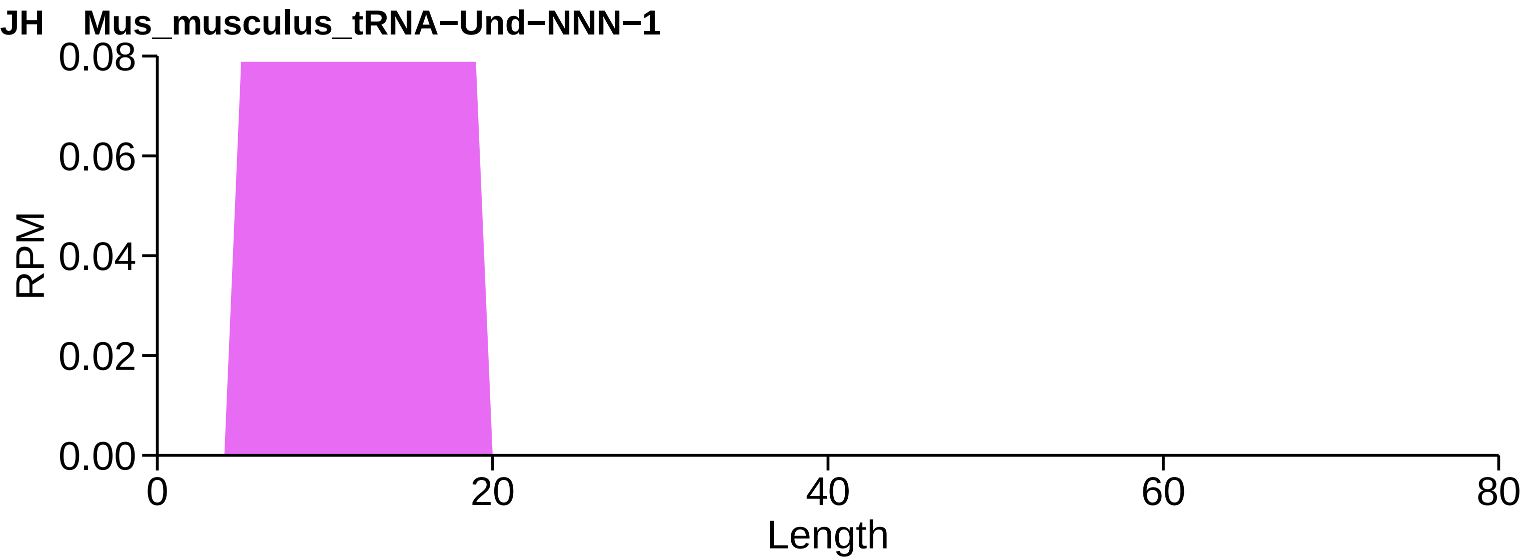
JH Mus_musculus_tRNA−Und−NNN−1
0.08
0.06
0.04
0.02
0.00
RPM
0
20
40
60
80
Length

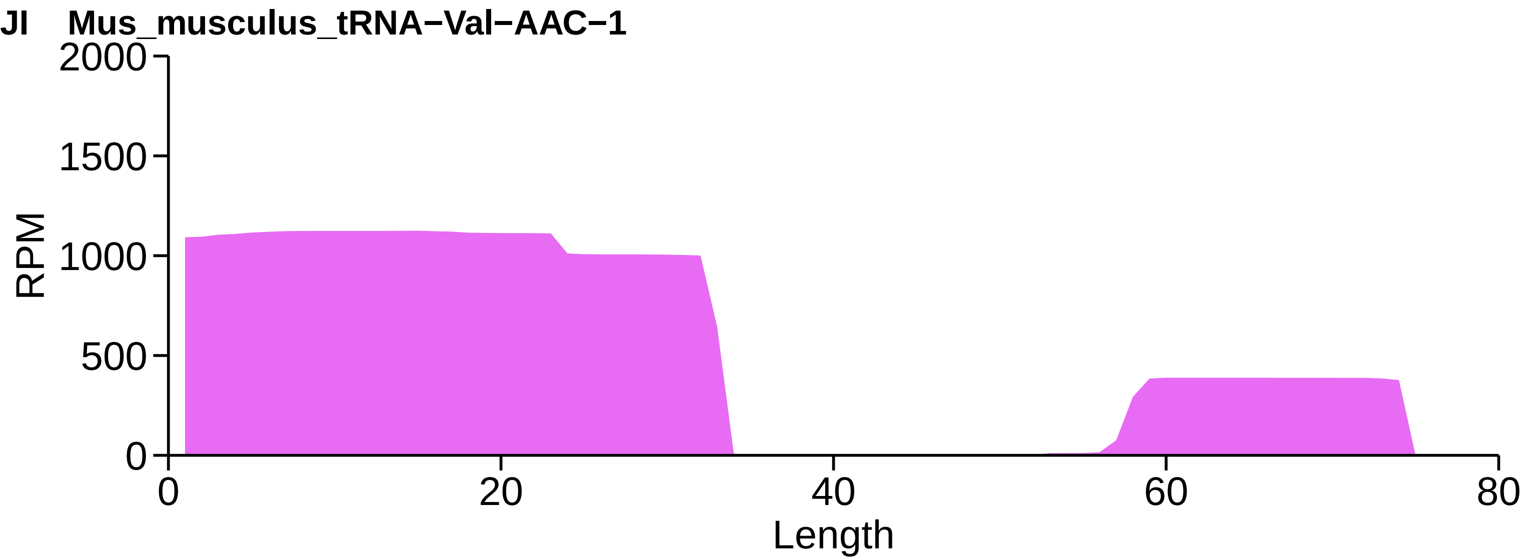

JI Mus_musculus_tRNA−Val−AAC−1
2000
1500
1000
500
0
RPM
0
20
40
60
80
Length

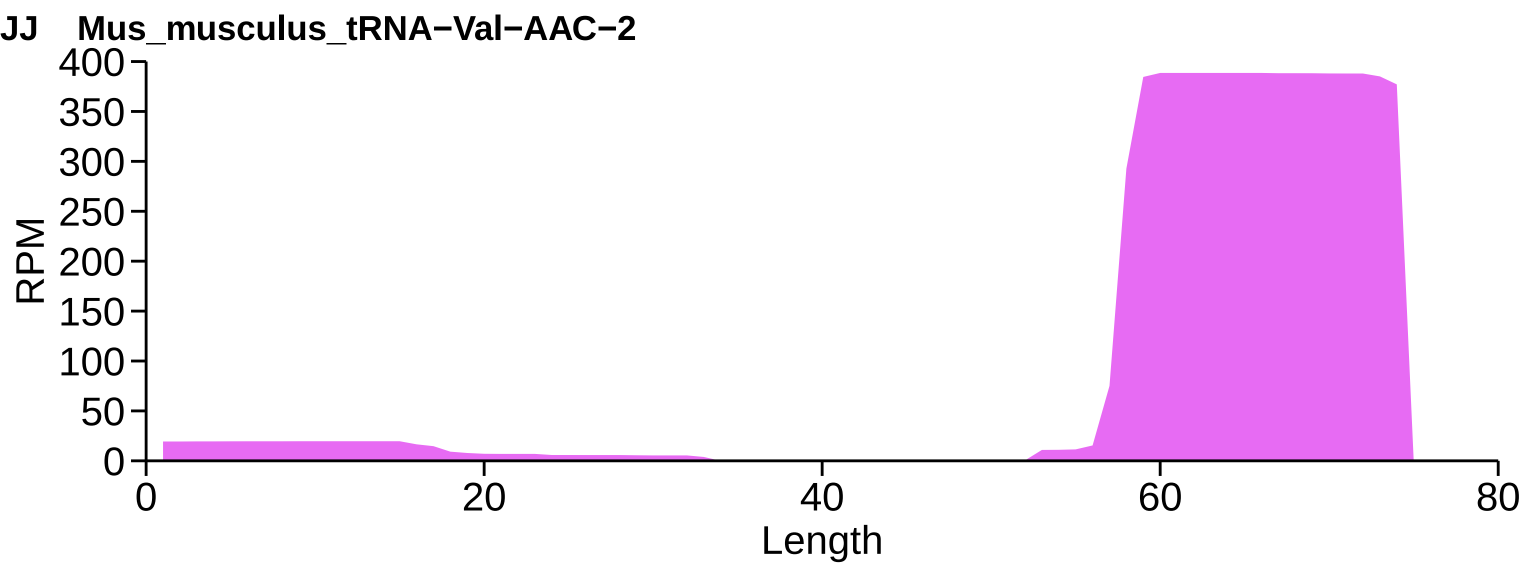
JJ Mus_musculus_tRNA−Val−AAC−2
RPM
400
350
300
250
200
150
100
50
0
0
20
40
60
80
Length

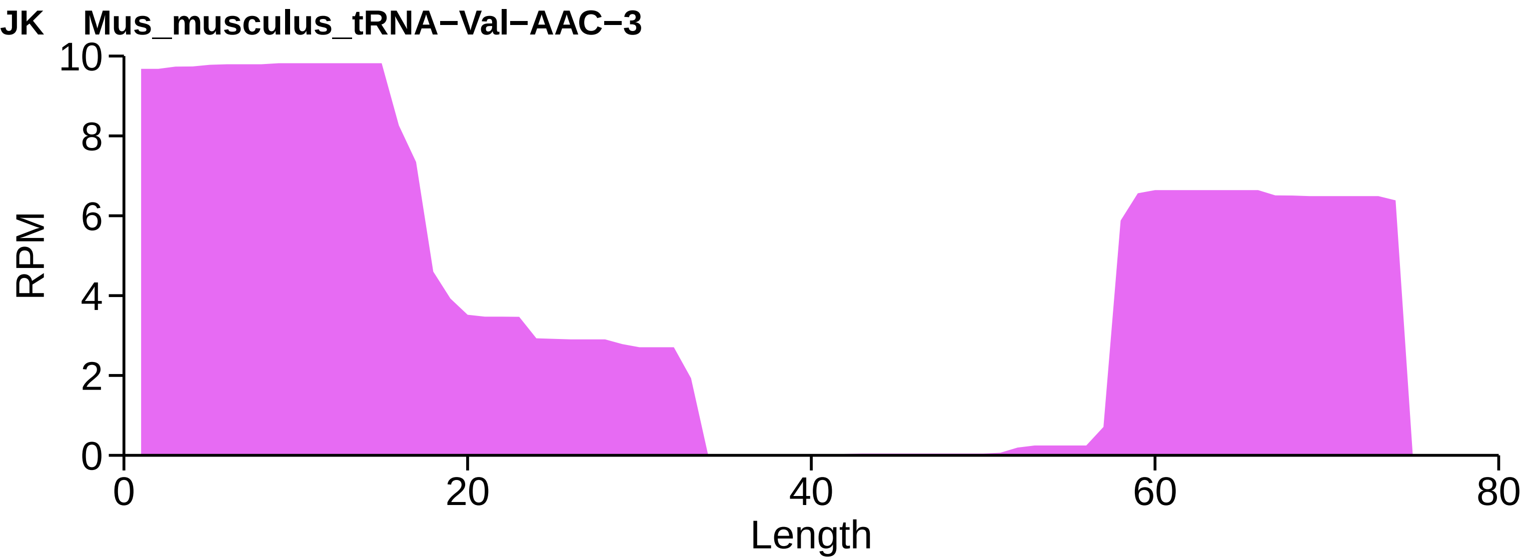

JK Mus_musculus_tRNA−Val−AAC−3
RPM
Length
0
2
4
6
8
10
0
20
40
60
80

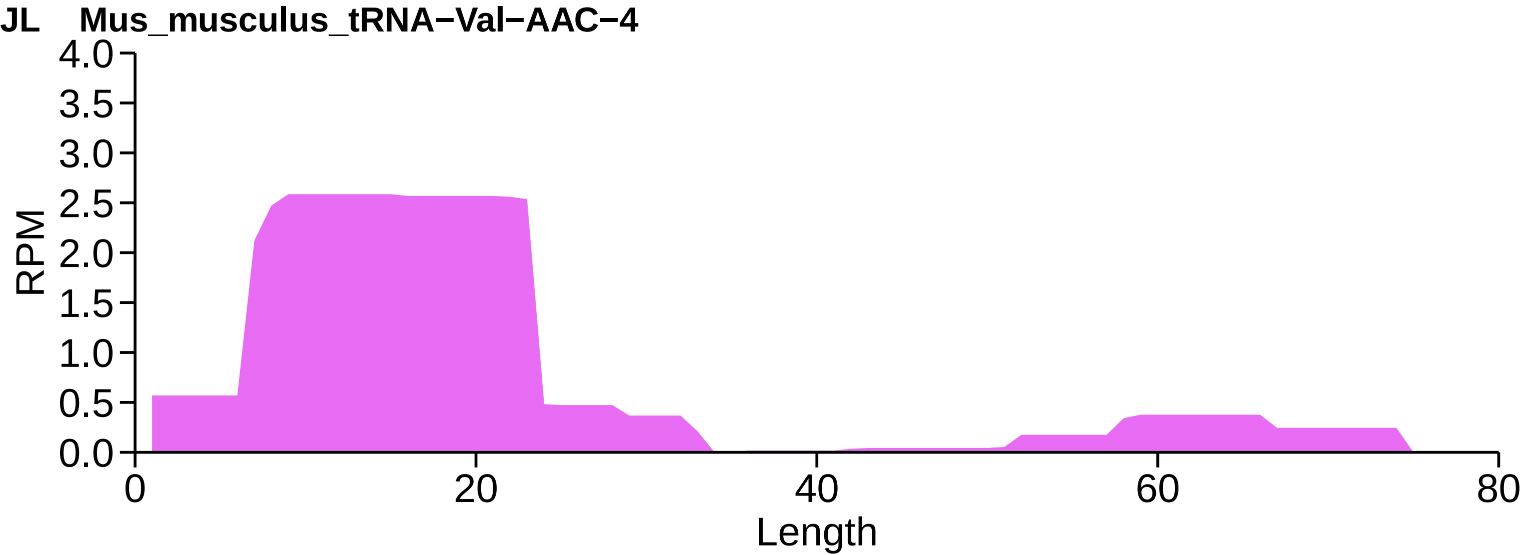

JL Mus_musculus_tRNA−Val−AAC−4
RPM
4.0
3.5
3.0
2.5
2.0
1.5
1.0
0.5
0.0
0
20
40
60
80
Length

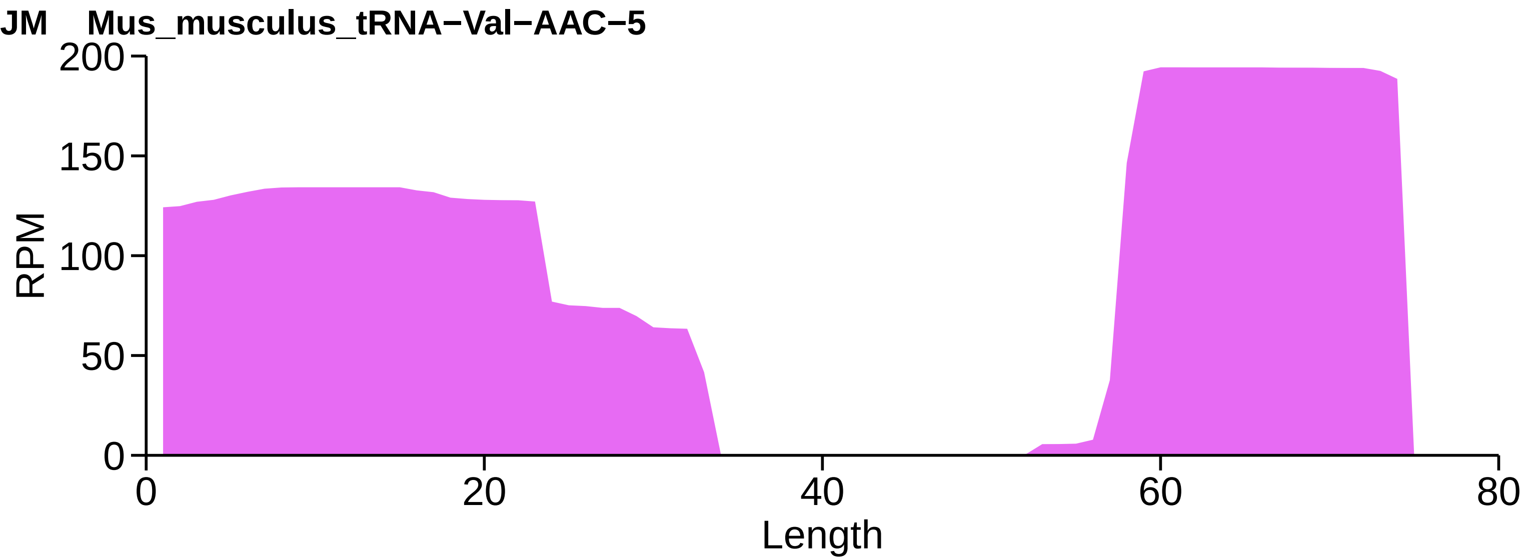
JM Mus_musculus_tRNA−Val−AAC−5
RPM
200
150
100
50
0
0
20
40
60
80
Length

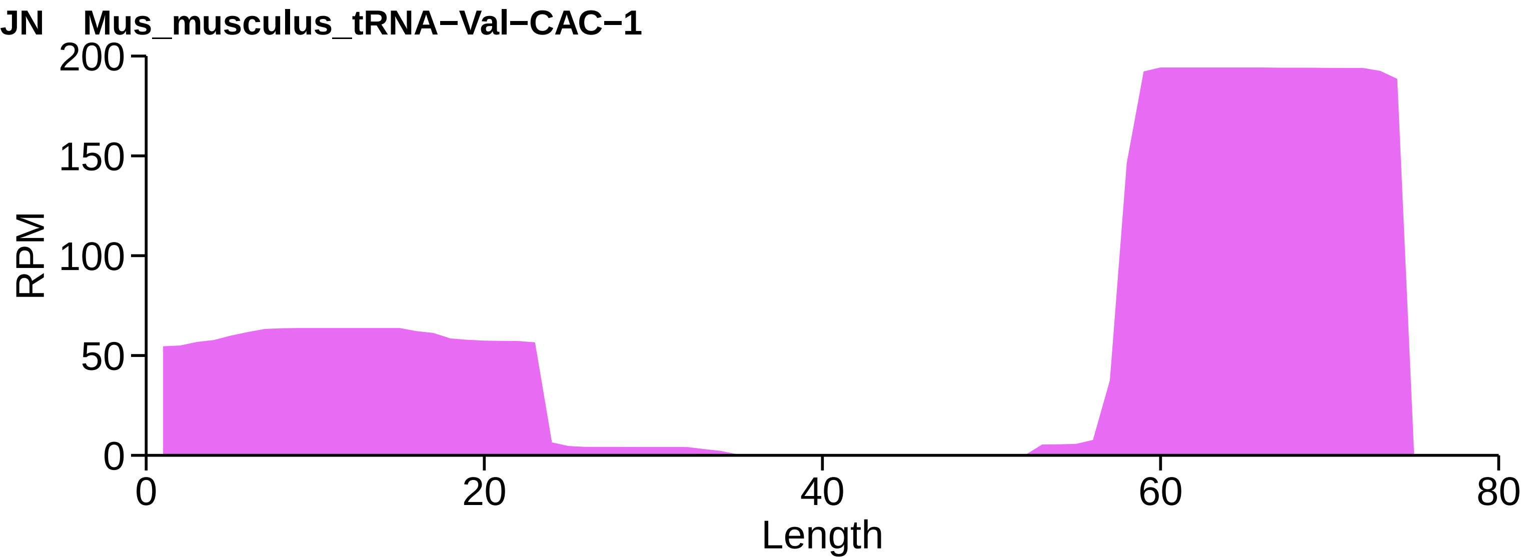

JN Mus_musculus_tRNA−Val−CAC−1
RPM
200
150
100
50
0
0
20
40
60
80
Length

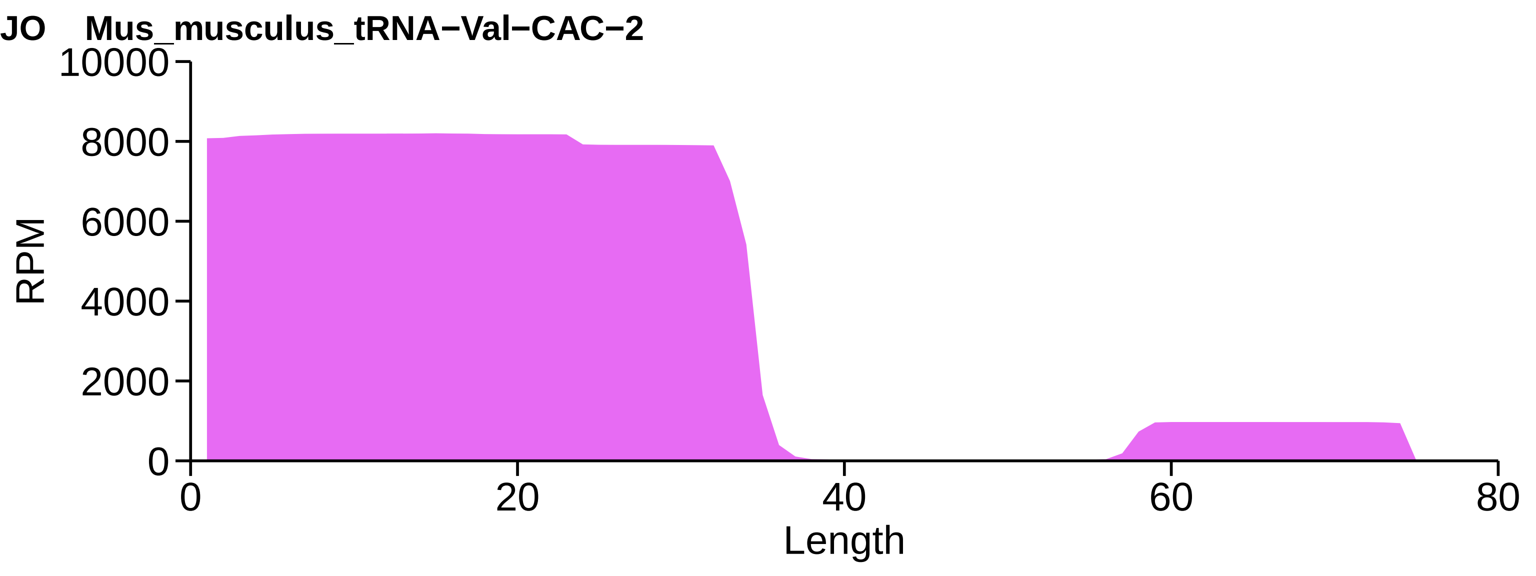

JO Mus_musculus_tRNA−Val−CAC−2
10000
8000
6000
4000
2000
0
RPM
0
20
40
60
80
Length

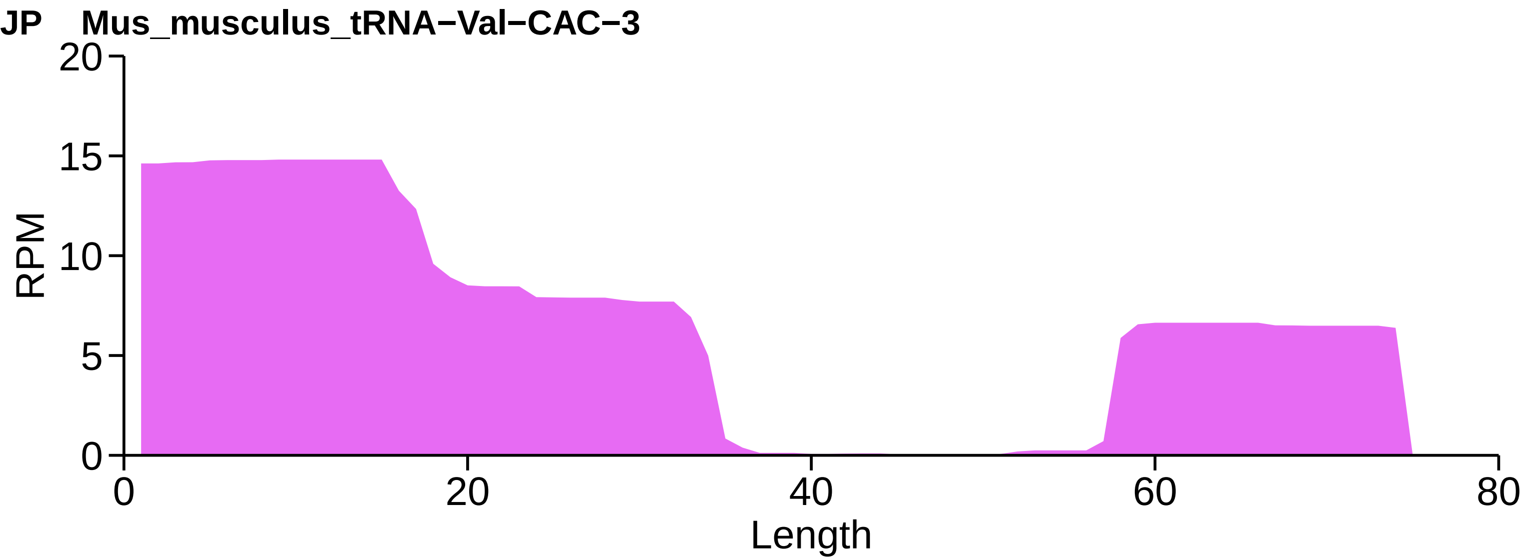
JP Mus_musculus_tRNA−Val−CAC−3
RPM
20
15
10
5
0
0
20
40
60
80
Length

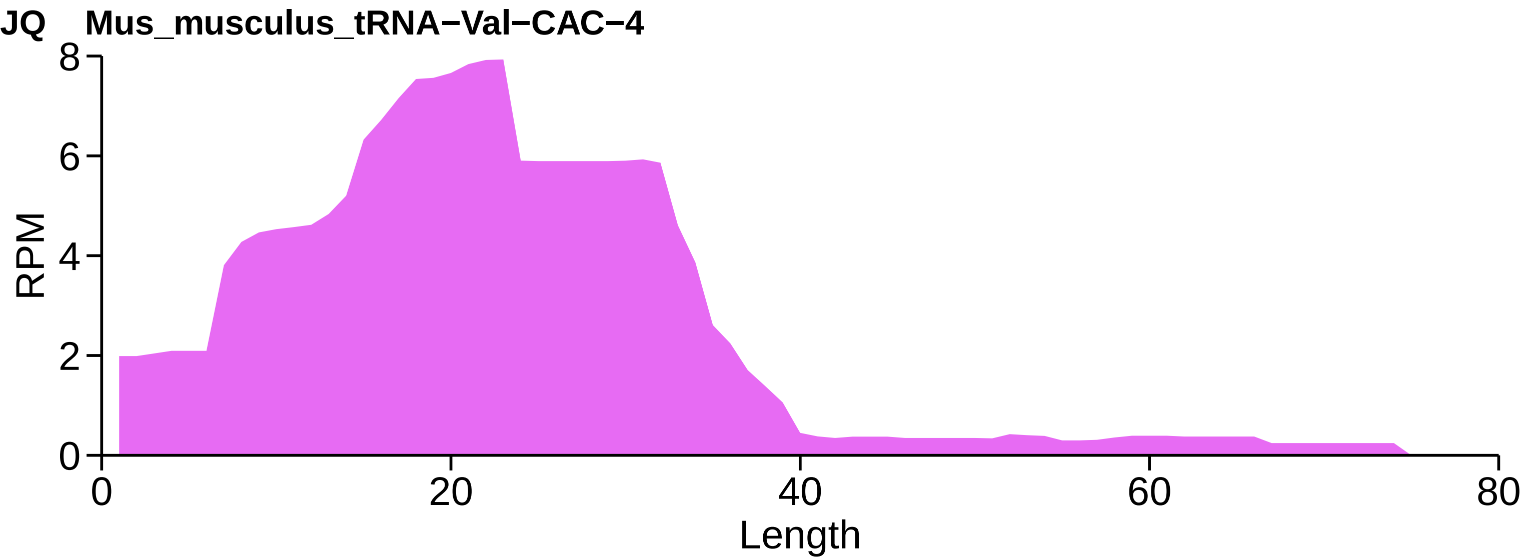

JQ Mus_musculus_tRNA−Val−CAC−4
RPM
Length
0
2
4
6
8
0
20
40
60
80

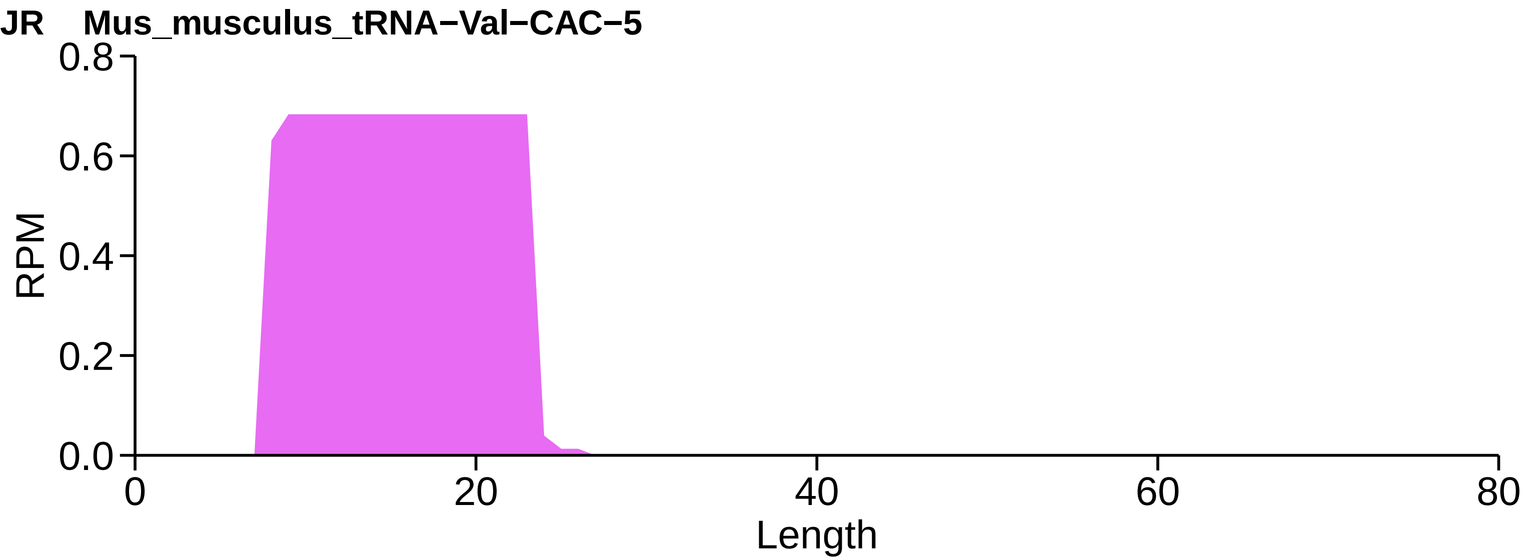
JR Mus_musculus_tRNA−Val−CAC−5
0.8
0.6
0.4
0.2
0.0
RPM
0
20
40
60
80
Length

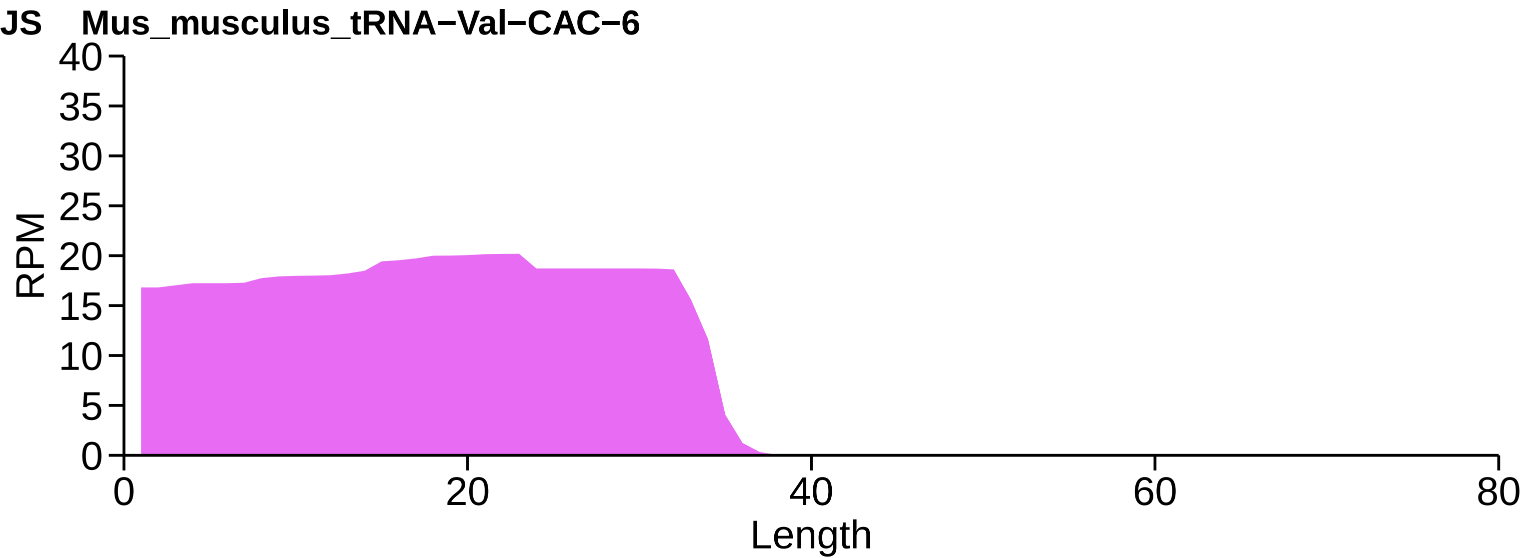

JS Mus_musculus_tRNA−Val−CAC−6
RPM
0
5
10
15
20
25
30
35
40
0
20
40
60
80
Length

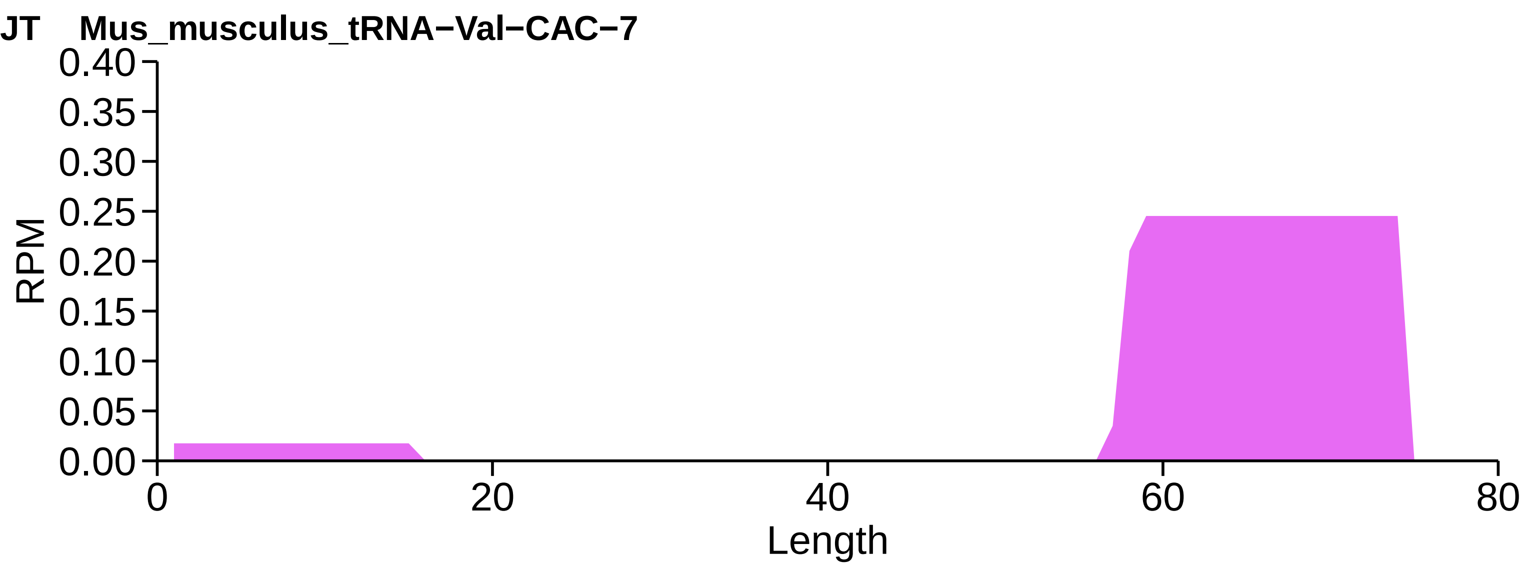
JT Mus_musculus_tRNA−Val−CAC−7
RPM
0.40
0.35
0.30
0.25
0.20
0.15
0.10
0.05
0.00
0
20
40
60
80
Length

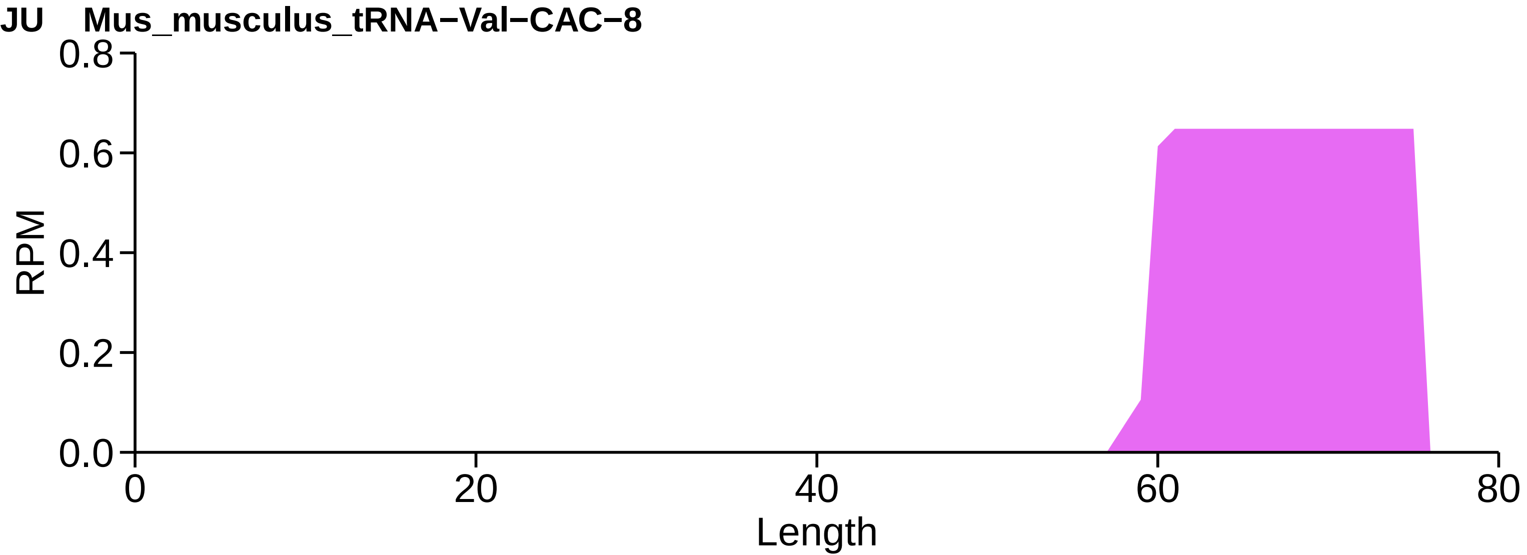

JU Mus_musculus_tRNA−Val−CAC−8
0.8
0.6
0.4
0.2
0.0
RPM
0
20
40
60
80
Length

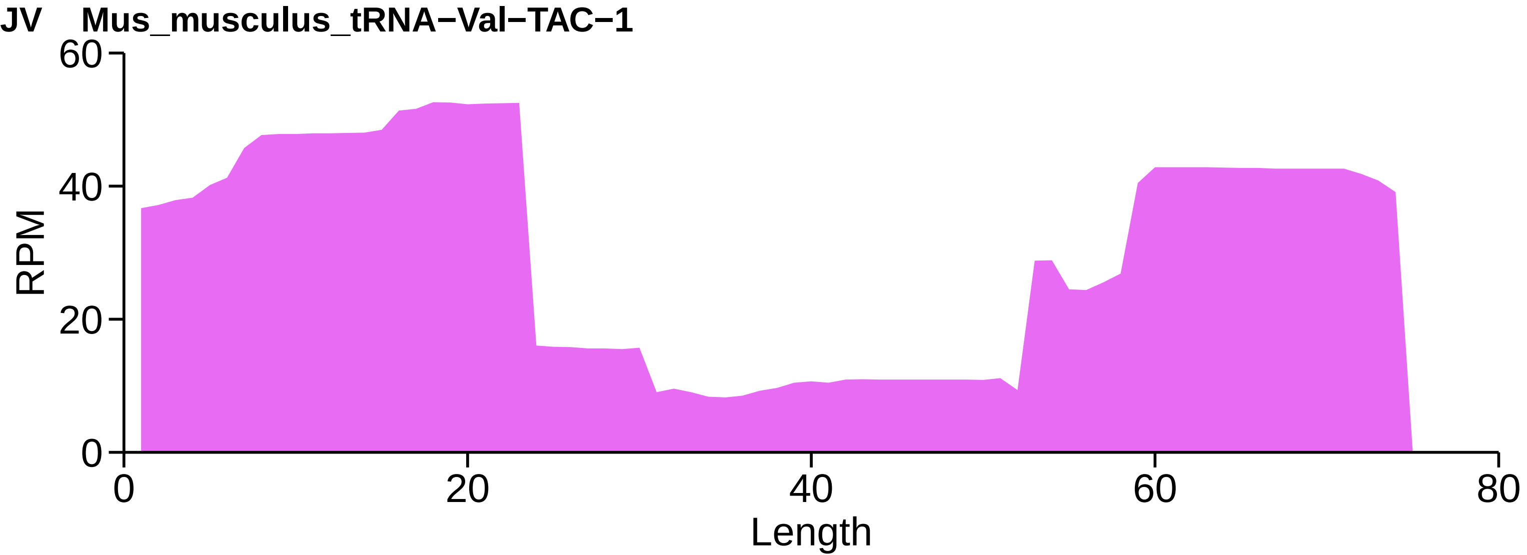

JV Mus_musculus_tRNA−Val−TAC−1
RPM
0
20
40
60
0
20
40
60
80
Length

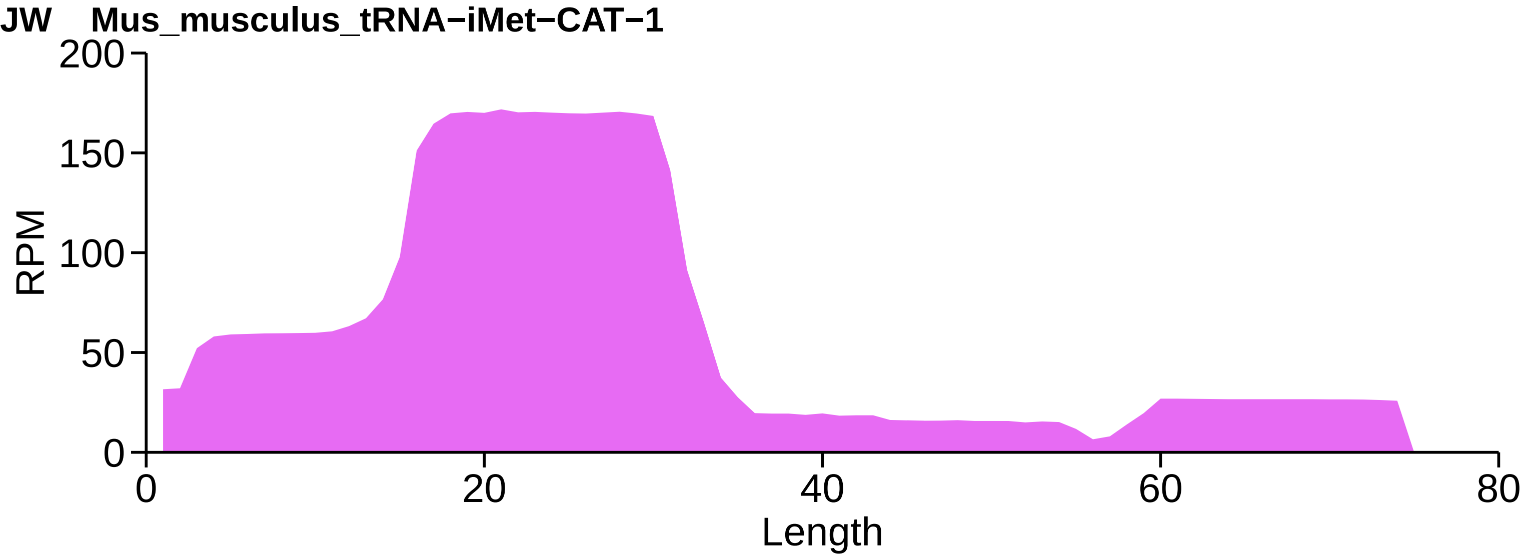
JW Mus_musculus_tRNA−iMet−CAT−1
200
150
100
50
0
RPM
0
20
40
60
80
Length

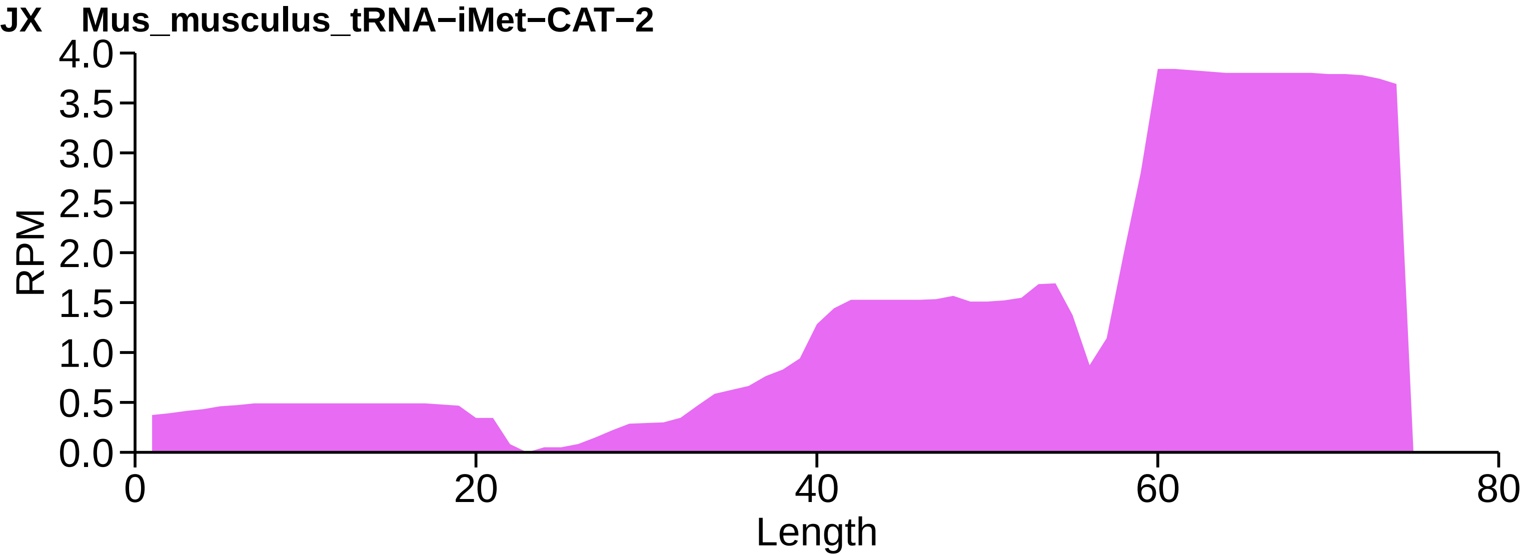
JX Mus_musculus_tRNA−iMet−CAT−2
RPM
4.0
3.5
3.0
2.5
2.0
1.5
1.0
0.5
0.0
0
20
40
60
80
Length

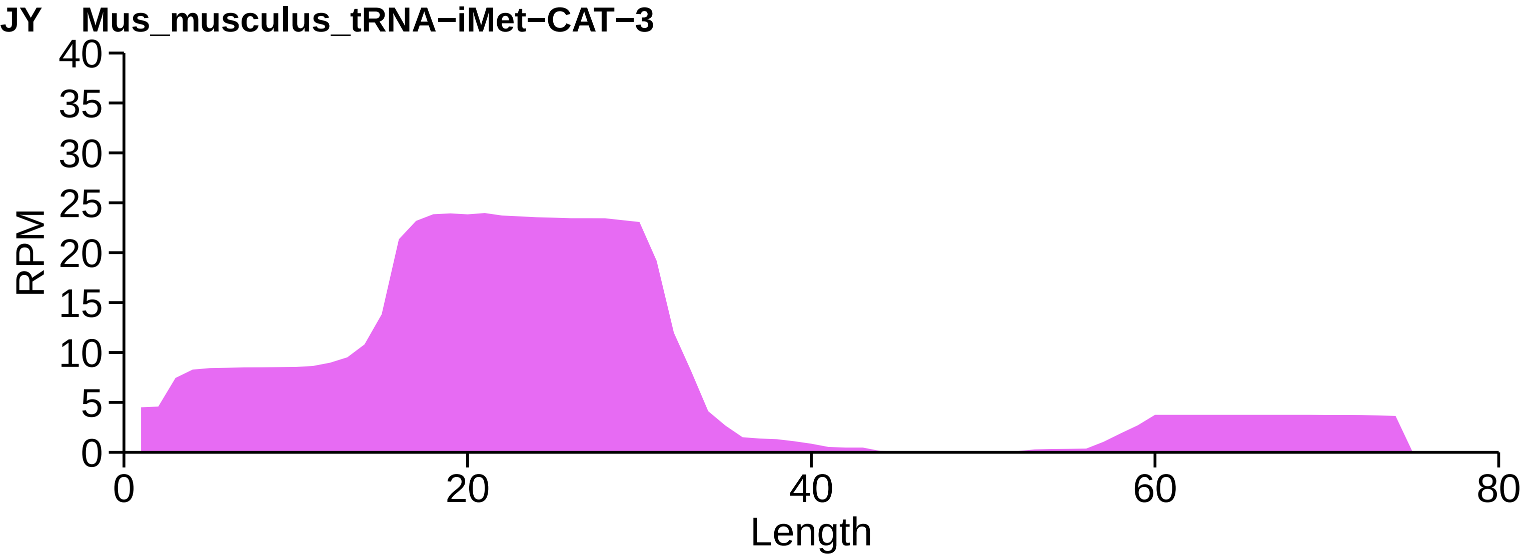

JY Mus_musculus_tRNA−iMet−CAT−3
RPM
40
35
30
25
20
15
10
5
0
0
20
40
60
80
Length
